# Oral Fungal Alterations in Patients with COVID‐19 and Recovered Patients

**DOI:** 10.1002/advs.202205058

**Published:** 2023-04-29

**Authors:** Xiaobo Hu, Haiyu Wang, Bo Yu, Jia Yu, Haifeng Lu, Junyi Sun, Ying Sun, Yawen Zou, Hong Luo, Zhaohai Zeng, Shanshuo Liu, Yan Jiang, Zhongwen Wu, Zhigang Ren

**Affiliations:** ^1^ Department of Infectious Diseases the First Affiliated Hospital of Zhengzhou University #1 Jianshe East Road Zhengzhou 450052 P. R. China; ^2^ Gene Hospital of Henan Province Precision Medicine Center the First Affiliated Hospital of Zhengzhou University Zhengzhou 450052 P. R. China; ^3^ Jinan Microecological Biomedicine Shandong Laboratory Jinan 250000 P. R. China; ^4^ Henan Key Laboratory of Ion‐beam Bioengineering School of Agricultural Sciences Zhengzhou University Zhengzhou 455004 P. R. China; ^5^ State Key Laboratory for Diagnosis and Treatment of Infectious Disease National Clinical Research Center for Infectious Diseases Department of Infectious Diseases the First Affiliated Hospital School of Medicine Zhejiang University Hangzhou 310003 P. R. China; ^6^ Department of Infectious Diseases Guangshan County People's Hospital Guangshan County Xinyang Henan 465450 P. R. China; ^7^ Department of Neurology the First Affiliated Hospital of Zhengzhou University Zhengzhou 450052 P. R. China

**Keywords:** coronavirus disease 2019, mycobiome, non‐invasive biomarkers, oral fungi, severe acute respiratory syndrome coronavirus 2

## Abstract

The oral bacteriome, gut bacteriome, and gut mycobiome are associated with coronavirus disease 2019 (COVID‐19). However, the oral fungal microbiota in COVID‐19 remains unclear. This article aims to characterize the oral mycobiome in COVID‐19 and recovered patients. Tongue coating specimens of 71 COVID‐19 patients, 36 suspected cases (SCs), 22 recovered COVID‐19 patients, 36 SCs who recovered, and 132 controls from Henan are collected and analyzed using internal transcribed spacer sequencing. The richness of oral fungi is increased in COVID‐19 versus controls, and beta diversity analysis reveals separate fungal communities for COVID‐19 and control. The ratio of *Ascomycota* and *Basidiomycota* is higher in COVID‐19, and the opportunistic pathogens, including the genera *Candida*, *Saccharomyces*, and *Simplicillium*, are increased in COVID‐19. The classifier based on two fungal biomarkers is constructed and can distinguish COVID‐19 patients from controls in the training, testing, and independent cohorts. Importantly, the classifier successfully diagnoses SCs with positive specific severe acute respiratory syndrome coronavirus 2 immunoglobulin G antibodies as COVID‐19 patients. The correlation between distinct fungi and bacteria in COVID‐19 and control groups is depicted. These data suggest that the oral mycobiome may play a role in COVID‐19.

## Graphical abstract

This research characterizes oral mycobiome in coronavirus disease 2019 (COVID‐19) patients and recovered COVID‐19 patients, constructs and validates the diagnostic model for COVID‐19, successfully diagnoses suspected cases with positive immunoglobulin G antibody as confirmed patients, and depicts the correlations between the oral microbiome and mycobiome.

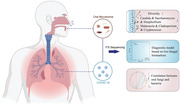

## 1 Introduction

The coronavirus disease 2019 (COVID‐19) pandemic caused by severe acute respiratory syndrome coronavirus 2 (SARS‐CoV‐2) continues to pose a public health threat. To date, COVID‐19 has infected 700 million people and killed 6 million.^[^ ^1^ ^]^ The main route of transmission of SARS‐CoV‐2 is through respiratory droplets and close contact.^[^ ^2^ ^]^ SARS‐CoV‐2 enters human cells by binding to angiotensin‐converting enzyme 2 (ACE2) and transmembrane protease serine 2 (TMPRSS2) receptors,^[^ ^3^ ^]^ which are highly expressed in intestinal epithelial cells, salivary gland cells, and lungs.^[^ ^4^ ^]^ This suggests that the virus may affect disease progression by disrupting the resident bacterial community of the oral cavity and gut, as recently reported with altered oral and gut microbiomes in patients with COVID‐19.^[^ ^5^ ^]^

The oral cavity has the second largest microbial community in the human body after the gut, including bacteria, fungi, and viruses, and is closely related to human health. Much research has focused on the human bacteriome in health and diseases, with little attention given to the human mycobiome. This may be due to the small proportion of fungi (less than 1%) in the human microbiome.^[^ ^6^ ^]^ However, the mycobiota plays a key role in human health and disease states. It maintains the structure and metabolism of the microbial community, interacts with the bacteriome, and participates in the host immune response, thereby regulating the degree of the inflammatory response and affecting human health and disease states, such as human immunodeficiency virus,^[^ ^7^ ^]^ inflammatory bowel disease, and asthma.^[^ ^8^ ^]^ Existing research elucidates the oral bacterial profile of patients with COVID‐19 and links oral bacteria to the severity of COVID‐19. However, oral fungal alterations in COVID‐19 patients and those who have recovered from COVID‐19 remain unclear.

Analysis of the microbiome as a diagnostic tool for diseases has become a hot research topic including in colon cancer,^[^ ^9^ ^]^ liver cirrhosis,^[^ ^10^ ^]^ and type 2 diabetes.^[^ ^11^ ^]^ At present, the gold standard for the diagnosis of COVID‐19 is reverse transcription‐polymerase chain reaction (RT‐PCR), but due to irregular sampling, improper collection sites, experimental operation error, low virus volume, and other reasons, there are false‐negative cases, which lead to the spread of COVID‐19. At the same time, a small number of people have typical clinical manifestations of COVID‐19 and positive serum antibodies, but the nucleic acid test continues to be negative, which is undoubtedly a challenge to epidemic prevention and control. Therefore, there is an urgent need for a novel auxiliary diagnostic tool that can compensate for the deficiencies of RT‐PCR. Recently, it has been found that oral,^[^ ^5a^ ^]^ pharyngeal,^[^ ^12^ ^]^ and intestinal^[^ ^13^ ^]^ microbial markers can effectively distinguish COVID‐19 from healthy people, with good diagnostic efficacy. However, the possibility of using oral fungal markers to diagnose COVID‐19 has not yet been explored.

Given the emerging association between the human oral microbiome and COVID‐19 and the possibility of false‐negative cases by RT‐PCR, we sought to explore whether oral fungal dysregulation is associated with the progression of COVID‐19 and the possibility of oral fungal markers for the diagnosis of COVID‐19. Therefore, we recruited a cohort of COVID‐19 patients (who tested positive for SARS‐CoV‐2 by PCR) and suspected cases (SCs) from Henan Province, China, and collected tongue coating swabs during early admission and at discharge. The composition of oral fungi was analyzed by internal transcribed spacer (ITS) sequencing. Our findings uniquely describe how oral fungal dysbiosis is involved in the disease progression of COVID‐19 while establishing a diagnostic model that enables cross‐regional validation.

## 2 Results

### 2.1 Study Design and Subject Characteristics

We prospectively collected 484 tongue‐coating samples from Central and East China. After rigorous screening, a total of 372 samples were sequenced including 71 COVID‐19 patients (COVID‐19) from Henan, 36 SCs with positive specific SARS‐CoV‐2 antibodies from Henan, 22 matched recovered COVID‐19 (Post‐COVID‐19), 36 matched recovered SCs (SCRs), 132 healthy individuals (controls) from Henan, and 75 COVID‐19 patients from Hangzhou (COVID‐19‐HZ) (Figure S1a, Supporting Information). An average of 21 999 high‐quality reads per sample were obtained for analysis, with the rarefaction curves approaching saturation (Figure S2a–d, Supporting Information), showing that the number of samples is reasonable.

COVID‐19 patients and controls from Henan (discovery cohort) were randomly divided into two groups (train phase: 48 COVID‐19 and 88 controls; test phase: 23 COVID‐19 and 44 controls). We characterized the oral mycobiome and construct COVID‐19 diagnostic model in the train phase and verified the efficacy of the model in the test phase and independent cohort (75 COVID‐19‐HZ and 36 SCs).

The clinical characterization of the subjects in the train and test phase is described in **Table** **1**. Age and sex were matched between the COVID‐19 and control groups (*p* > 0.05). The comorbidities in the COVID‐19 group were mainly hypertension (*n* = 6), diabetes mellitus (*n* = 5), and coronary artery disease (*n* = 4) (Table S1, Supporting Information). The most common symptoms were fever and cough. Compared with controls, white blood cells and lymphocytes were decreased in the COVID‐19 groups (*p* < 0.01), while alanine aminotransferase, aspartate aminotransferase, total bilirubin, and the neutrophil‐to‐lymphocyte ratio were increased in the COVID‐19 patients in both groups (*p* <0.05).

**Table 1:** Demographics and clinical characteristics of subjects in discovery cohort

Clinical indicators	Train phase (*n* = 136)		*p*‐value	Test phase (*n* = 67)		*p*‐value^a)^
	Healthy controls (*n* = 88)	COVID‐19 patients (*n* = 48)		Healthy controls (*n* = 44)	COVID‐19 patients (*n* = 23)	
Age (years)	47.50 ± 6.95	47.98 ± 15.94	0.422	45.52 ± 6.38	48.22 ± 10.17	0.193
Sex (female/male)	49/39	28/20	0.766	26/18	14/9	0.888
Comorbidities^b)^	NA	9 (18.75%)		NA	6 (26.09%)	
Confirmed cases or exposure to Wuhan	NA	39 (81.25%)		NA	20 (86.96%)	
Symptoms at admission						
Fever	NA	29 (60.42%)		NA	16 (69.57%)	
Cough	NA	22 (45.83%)		NA	9 (39.13%)	
Sputum	NA	4 (8.33%)		NA	4 (17.39%)	
Headache	NA	3 (6.25%)		NA	3 (13.04%)	
Fatigue	NA	8 (16.67%)		NA	4 (17.39%)	
Diarrhea	NA	1 (2.08%)		NA	0 (0%)	
Dyspnea	NA	2 (4.17%)		NA	1 (4.35%)	
Laboratory results						
Red blood cells (10^12/L)	4.68 ± 0.50	4.58 ± 0.59	0.383	4.60 ± 0.26	4.61 ± 0.65	0.979
White blood cells (10^9/L)	6.46 ± 2.04	5.47 ± 2.23	<0.001	6.57 ± 1.73	5.24 ± 1.63	0.008
Neutrophils (10^9/L)	3.97 ± 1.79	4.31 ± 2.25	0.726	3.97 ± 1.31	4.39 ± 1.78	0.322
Lymphocytes (10^9/L)	1.94 ± 0.58	1.43 ± 0.56	<0.0001	2.04 ± 0.57	2.25 ± 2.98	0.005
N/L	2.18 ± 1.24	3.48 ± 2.38	<0.0001	2.01 ± 0.66	3.24 ± 2.50	0.042
Blood Platelet (10^9/L)	223.85 ± 76.54	189.48 ± 66.16	0.004	221.82 ± 57.87	206.96 ± 86.54	0.309
Hemoglobin (g L^−1^)	142.22 ± 17.59	137.00 ± 17.94	0.098	138.50 ± 18.03	144.35 ± 46.56	0.383
Alanine aminotransferase (U L^−1^)	19.00 ± 8.87	25.12 ± 12.99	0.002	18.57 ± 11.82	30.45 ± 24.45	0.047
Aspartate aminotransferase (U L^−1^)	19.78 ± 5.01	26.80 ± 14.01	<0.0001	18.68 ± 6.37	25.63 ± 7.76	<0.0001
Albumin (g L^−1^)	43.53 ± 3.80	41.93 ± 6.41	0.02	43.79 ± 4.16	42.10 ± 4.12	0.20
Total bilirubin (µmol L^−1^)	6.97 ± 4.72	14.78 ± 11.41	<0.0001	7.14 ± 3.98	11.16 ± 6.66	<0.0001
Serum creatinine (µmol L^−1^)	73.63 ± 12.11	68.01 ± 18.12	0.009	72.29 ± 15.96	77.21 ± 34.62	0.417

Abbreviations: NA, not available; N/L, neutrophil‐to‐lymphocyte ratio;

^a)^ Statistical significance was defined by *p* < 0.05 (two‐tailed);

^b)^ Comorbidities included high blood pressure, chronic obstructive pulmonary disease, diabetes, malignant tumor, cardiovascular disease, and chronic liver disease. Details could be found in Table S1, Supporting Information.

### 2.2 Fungal Dysbiosis in the Oral Cavity of COVID‐19 Patients

**Figure 1:**
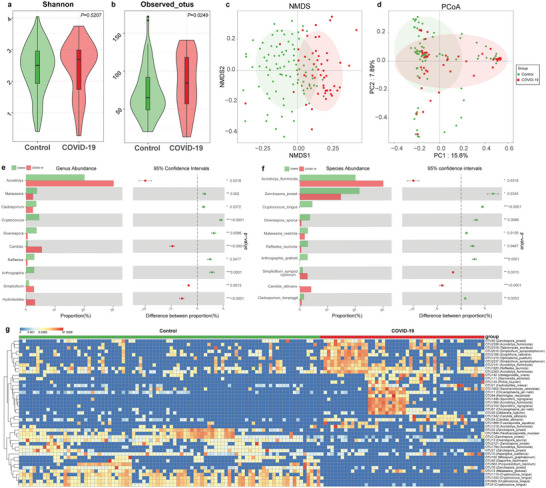
Fungal dysbiosis in the oral cavity of COVID‐19 patients. a) Shannon index and b) Observed OTUs diversity indices between COVID‐19 (*n* = 48) and control (*n* = 88) groups. The NMDS (c) and PCoA (d) analysis showed the oral fungal taxonomic composition was conspicuously different between the two groups. The ten most abundant fungi among the differential fungi between COVID‐19 and controls at the e) genus and f) species levels. g) Heatmap showed the relative abundances of differential OTUs for each sample in both groups. COVID‐19, COVID‐19 patients; OTUs, operational taxonomy units; PCoA, principal coordinate analysis; NMDS, nonmetric multidimensional scaling. * *p* < 0.05, ** *p* < 0.01, *** *p* < 0.001.

The train phase comprised 48 patients with COVID‐19 and 88 controls. Through ITS sequencing, 2341 operational taxonomy units (OTUs) were identified including 303 OTUs unique to COVID‐19 patients, and 839 OTUs unique to controls (Figure S3a, Supporting Information). The oral fungal diversity of COVID‐19 and controls was evaluated through the Shannon index and Observed OTUs for alpha diversity, principal coordinates analysis (PCoA), and nonmetric multidimensional scaling (NMDS) for beta diversity. Shannon index, reflecting species richness and evenness, was not significantly different between COVID‐19 and controls (**Figure** **1**a) (Table S2, Supporting Information), while Observed OTUs, representing the species richness, was significantly increased in COVD‐19 versus controls (Figure 1b). Both PCoA and NMDS plots showed that oral mycobiome separates COVID‐19 and controls into two strikingly distinct groups (Figure 1c,d), indicating that oral mycobiome dysbiosis occurred in COVID‐19.

Further analysis of COVID‐19 and control groups' composition and alterations found that phylum *Ascomycota*, *Basidiomycota*, and *Mucoromycota* accounted for 95% of sequences on average and were the most abundant phylum, and the genera *Acrodictys*, *Zanclospora*, *Aspergillus*, *Blumeria*, and *Malassezia* were the five leading genera in both groups (Figure S3c,d, Supporting Information) (Table S3, Supporting Information). The composition of fungi at species levels is shown in Figure S3e, Supporting Information. At the phylum level, the phylum *Ascomycota* and *Zoopagomycota* were increased, and the phylum *Basidiomycota*, *Mucoromycota*, and *Rozellomycota* were diminished in the COVID‐19 compared with controls (Figure S3b, Supporting Information) (Table S4, Supporting Information). At the genus level, 37 fungal features were enriched in COVID‐19, including *Acrodictys*, *Candida*, and *Simplicillium*, while 52 fungal features were depleted in COVID‐19 including *Malassezia*, *Cladosporium*, and Cryptococcus (Figure 1e). Furthermore, at the species level, 71 fungal species were reduced in COVID‐19, including *Zanclospora jonesii*, *Cryptococcus longus*, and *Diversispora spurca*, while 52 fungal species were decreased in controls, including *Acrodictys fluminicola*, *Simplicillium sympodiophorum*, and *Candida albicans* (Figure 1f). The heatmap based on the abundance of differential OTUs showed that 27 OTUs were enriched in COVID‐19, and 16 OTUs were depleted in COVID‐19 compared with controls (Figure 1g) (Table S5, Supporting Information).

Considering the possibility that comorbidities may have an impact on oral fungi, we performed a subgroup analysis. The 71 COVID‐19 patients from Henan were divided into COVID‐19 patients without comorbidities (COVID‐19‐N, *n* = 56) and COVID‐19 patients with comorbidities (COVID‐19‐C, *n* = 15). Then we analyzed the oral fungal characteristics of COVID‐19‐N, COVID‐19‐C, and control (*n* = 132) groups. The Shannon index was not significantly different between COVID‐19‐N and controls (Figure S4a, Supporting Information) (Table S6, Supporting Information) but was significantly increased in COVID‐19‐C compared with COVID‐19‐N and controls. The Observed OTUs, were significantly increased in COVID‐19‐N and COVID‐19‐C versus control (Figure S4b, Supporting Information) but were not significantly different between COVID‐19‐N and COVID‐19‐C. Both PCoA and NMDS plots showed that the oral mycobiome separated COVID‐19 patients and controls into two strikingly distinct groups (Figure S4c,d, Supporting Information), while the oral fungal communities of COVID‐19‐N were similar to those of COVID‐19‐C. At the genus level, a total of 143 genera differed between the three groups, most of which did not differ between COVID‐19‐N and COVID‐C groups but differed significantly from controls. Only 13 genera differed between COVID‐19‐N and COVID‐C groups (Figure S4e and Table S7, Supporting Information). The results were similar at the species level (Figure S4f, Supporting Information). The heatmap showed that the fungal characterization of COVID‐19‐N was similar to that of COVID‐19‐C but different from that of the controls (Figure S4g, Supporting Information) (Table S8, Supporting Information). In conclusion, these results suggest that comorbidity has a much lesser effect on oral fungi than COVID‐19.

### 2.3 Identification of an Oral Fungal Classifier for COVID‐19

To identify oral fungi with potential value for COVID‐19 diagnosis, we constructed the random forest classifier model in the train phase (Figure S1b, Supporting Information). Through fivefold cross‐validation, a total of 2 OTU markers, including OTU4 (*C. longus*) and OTU925 (*C. longus*), were identified as the optimal marker set (Figure S5a,b, Supporting Information). In the test phase, the remaining one‐third of the samples from the discovery cohort were used to validate the diagnostic efficacy. Moreover, 75 COVID‐19 patients from Hangzhou and 36 SCs were used as an independent external validation cohort.

**Figure 2:**
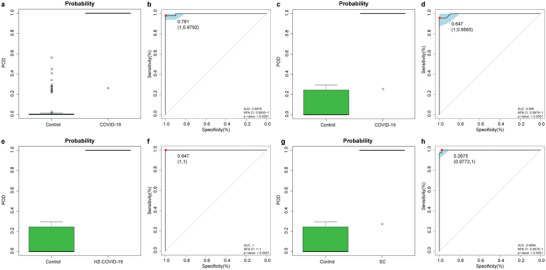
Identification of an oral fungal classifier for COVID‐19. The POD value was significantly increased in COVID‐19 compared with controls, and achieved good diagnostic efficacy in the train phase (a,b) (containing 88 controls and 48 COVID‐19, *p* < 0.0001), the test phase (c,d) (containing 44 controls and 23 COVID‐19, *p* < 0.0001), the HZ‐independent cohort (e,f) (containing 44 controls and 75 COVID‐19 from Hangzhou, *p* < 0.0001). Compared with controls, the POD value was significantly increased in SCs (g), achieving an AUC value of 0.9994 (h) (containing 44 controls and 36 SCs, *p* < 0.0001). COVID‐19, COVID‐19 patients; COVID‐19‐HZ, COVID‐19 patients from Hangzhou; SCs, suspected cases; POD, probability of disease; AUC, area under the curve. Centerline, median; box limits, upper and lower quartiles; circle or square symbol, mean; error bars, 95% CI.

We observed that these fungal markers could accurately differentiate COVID‐19 patients and controls, and the probability of disease (POD) index was markedly higher in the COVID‐19 patients than in the controls, with an area under the curve (AUC) of 99.79% (95% CI 99.35% to 100%, *p* < 0.0001) in the train phase (**Figure** **2**a,b) (Table S9, Supporting Information). The performance of the oral fungal markers identified in the train phase was demonstrated in the test phase (44 COVID‐19 and 23 controls). Compared with that in controls, the POD index was markedly increased in COVID‐19 patients, with an AUC of 99.6% (95% CI 98.76% to 100%, *p* < 0.0001) (Figure 2c,d) (Table S10, Supporting Information). Furthermore, our oral fungal classifier could distinctly stratify COVID‐19 patients from controls with an AUC of 100% (95% CI 100% to 100%, *p* < 0.0001) in the independent cohort (Hangzhou Group) (Figure 2e,f) (Table S11, Supporting Information), suggesting that our diagnostic model successfully achieves cross‐regional validation.

To further expand the application of fungal markers and reduce the false‐negative rate of RT‐PCR, we collected tongue coating and serum samples from 36 SCs and matched SCRs, with positive specific SARS‐CoV‐2 serum immunoglobulin G antibodies (IgG) but negative nucleic acid for SARS‐CoV‐2 (Figure S5c, Supporting Information) (Table S12, Supporting Information). We observed that our fungal markers could diagnose SCs as COVID‐19, with an AUC of 99.94% (95% CI 99.76% to 100%, *p* < 0.0001) between SCs and controls (Figure 2g,h) (Table S13, Supporting Information), indicating that the oral fungal classifier could be used as an adjunct noninvasive diagnostic tool for RT‐PCR.

### 2.4 Oral Mycobiome Profile Alterations among COVID‐19 Patients, SCs, and Controls

**Figure 3:**
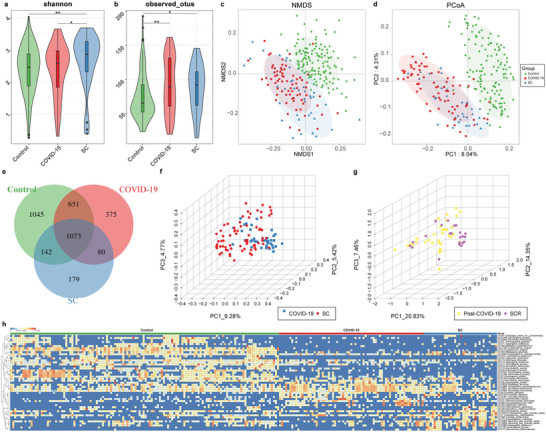
Oral mycobiome profile alterations among COVID‐19, SC, and control groups. a) Shannon index and b) Observed OTUs diversity indices among COVID‐19 (*n* = 71), SC (*n* = 36), and control (*n* = 132) groups. The NMDS (c) and PCoA (d) analysis exhibited that the oral fungal communities in the COVID‐19 and SCs were similar but significantly different from those in the controls. e) A Venn diagram revealed that 1073 of 3545 OTUs were shared in the COVID‐19, SC, and control groups, while 1045 OTUs were unique to controls. The PCoA showed that there was no significant difference in the oral mycobiome distribution between COVID‐19 (*n* = 71) and SCs (*n* = 36) (f) or between Post‐COVID‐19 (*n* = 22) and SCRs (*n* = 36) (g). h) Heatmap of the relative abundances of differential OTUs for each sample in three groups. OTUs, operational taxonomic units; COVID‐19, COVID‐19 patients; SCs, suspected cases; Post‐COVID‐19, recovered COVID‐19 patients; SCRs suspected cases who recovered; NMDS, nonmetric multidimensional scaling; PCoA, principal coordinate analysis. * *p* < 0.05, ** *p* < 0.01, *** *p* < 0.001.

To elucidate the feasibility of using this fungal classifier to diagnose SCs as COVID‐19, we analyzed the oral fungal characteristics among 71 COVID‐19, 36 SCs, and 132 controls from the microbial perspective. We observed that the Shannon index of SCs was significantly higher than that of COVID‐19 and controls (*p* < 0.05 and *p* < 0.01, respectively, **Figure** **3**a), and the Observed OTUs of COVID‐19 and SCs were remarkedly higher than that of controls (*p* < 0.01 and *p* < 0.05, respectively, Figure 3b) (Table S14, Supporting Information). The beta diversity of PCoA and NMDS analysis revealed that the fungal communities of SCs were similar to COVID‐19, but both of them were significantly different from controls (Figure 3c,d). Importantly, the OTUs identified between the SC and COVID‐19 groups were basically the same, with only 375 OTUs exclusive to COVID‐19, 179 OTUs exclusive to SC, and 1045 OTUs exclusive to the control group (Figure 3e). Moreover, the abundance of differential oral fungi in the SC group was basically consistent with that of the COVID‐19 group, but significantly different from the control group (Figure 3h) (Table S15, Supporting Information).

We further analyzed the oral fungal characteristics of COVID‐19, SC, Post‐COVID‐19, and SCR in order to further prove that both suspected and confirmed cases are COVID‐19 from the microbiological point of view. The fungal community distribution between COVID‐19 and SCs, as well as Post‐COVID‐19 and SCRs, was observed with no significant difference (Figure 3f,g) (Table S16, Supporting Information). Additionally, the fungal microbial variation between COVID‐19 and Post‐COVID‐19 was similar to the variation between SC and SCR (Figure S5d, Supporting Information).

Taken together, the oral fungal profile of SCs was similar to COVID‐19. Thus, we could speculate that SCs could be judged as COVID‐19 based on the assumption that the same disease has unique microbial characteristics. This also confirms the feasibility of our use of microbial classifiers to diagnose SP as COVID‐19.

### 2.5 Alterations in the Oral Mycobiome Along with COVID‐19 Recovery

**Figure 4:**
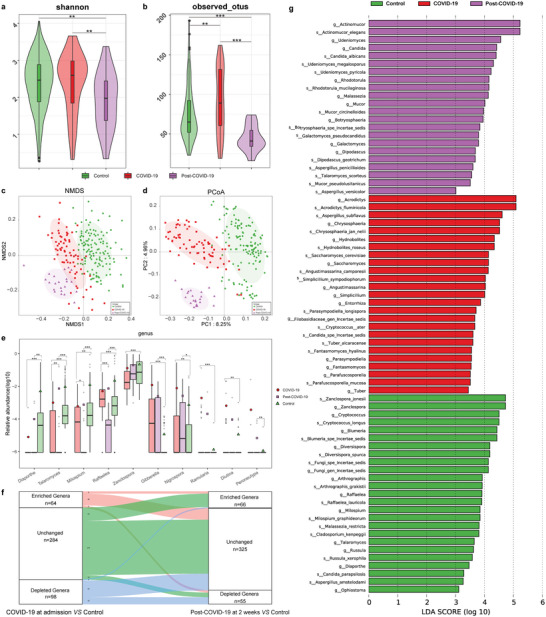
Alterations in oral mycobiome along with COVID‐19 recovery. a) Shannon index and b) Observed OTUs diversity indices among COVID‐19 (*n* = 71), Post‐COVID‐19 (*n* = 22), and control (*n* = 132) groups. The PCoA (c) and NMDS (d) showed that the oral fungal microbiota in the Post‐COVID‐19 was different from those in the COVID‐19 and controls. e) Along with the recovery of COVID‐19, the relative abundances of five genera gradually enriched, while the abundances of five genera gradually reduced, all of them were remarkedly different among the three groups. f) Changes in oral fungal composition in COVID‐19 patients from admission to 2 weeks after discharge. g) Linear discriminant analysis (LDA) effect size in oral mycobiome among three groups at genus (g) and species (s) levels (*p* < 0.05, LDA > 3.0). COVID‐19, COVID‐19 patients; Post‐COVID‐19, recovered COVID‐19 patients; PCoA, principal coordinate analysis; NMDS, nonmetric multidimensional scaling. * *p* < 0.05, ** *p* < 0.01, *** *p* < 0.001.

We explored and compared the oral mycobiome characteristics in 71 COVID‐19, 22 Post‐COVID‐19, and 132 controls. The Shannon index and the Observed OTUs in Post‐COVID‐19 were significantly reduced in Post‐COVID‐19 compared with COVID‐19 and controls (*p* < 0.01, **Figure** **4**a,b) (Table S17, Supporting Information), reflecting the decreased evenness and richness of oral fungi. The NMDS and PCoA analysis showed that the fungal microbiota distribution characteristics of Post‐COVID‐19 were significantly different from those of COVID‐19 and controls (Figure 4c,d). A total of 123 OTUs were unique to Post‐COVID‐19, 416 OTUs were shared between COVID‐19 and Post‐COVID‐19, and 431 OTUs were shared between controls and Post‐COVID‐19 (Figure S6a, Supporting Information).

The average oral fungal composition and relative abundance in the COVID‐19, Post‐COVID‐19, control groups at the phylum, genus, and species level are presented in Figure S6d–f, Supporting Information (Table S18, Supporting Information). The most dominant fungi in COVID‐19 and controls were *Acrodictys* but were *Zanclospora* in Post‐COVID‐19, and the differential fungi among three groups displayed the fungi characterization of Post‐COVID‐19 were significantly different from controls and COVID‐19 (Figure S6g, Supporting Information) (Table S19, Supporting Information). We further performed differential analysis at the phylum, genus, and species levels (Figure 4e and Figure S6b, Supporting Information) (Table S20, Supporting Information). The results showed that there was a total of 144 different genera in the three groups, of which the abundance of 5 genera gradually decreased with the recovery of COVID‐19, including *Gibberella*, *Nigrospora*, *Ramularia*, *Diutina*, and *Peroneutypa*,16 genera gradually increased including *Zanclospora*, *Raffaelea*, *Milospium*, *Talaromyces*, and *Diaporthe* (Figure 4e). A total of 201 species were different among three groups, of which the abundance of 15 species gradually raised with the recovery of COVID‐19, including *Z. jonesii*, *Malassezia restricta*, and *Raffaelea lauricola*, 7 species gradually reduced including *Gibberella fujikuroi*, *Nigrospora oryzae*, and *Penicillium hirsutum* (Figure S6c, Supporting Information). Among the detected oral fungal genera in the three groups (Figure 4f,g) (Table S21, Supporting Information), 64 genera were enriched in the COVID‐19, of which 46 were normalized in recovered patients, 98 genera were diminished in the COVID‐19, of which 66 were recovered in recovered patients returned to normal.

The enriched pathways in which the fungi may affect the progression of COVID‐19 were identified in the MetaCyc database. The results showed that many metabolic pathways were enriched in the COVID‐19 group, such as adenine and adenosine salvage III, galactose degradation I Leloir pathway, and valine biosynthesis, while some pathways were enriched in the Post‐COVID‐19 group such as fatty acid elongation saturated, sulfate reduction I assimilatory, and pantothenate and coenzyme A biosynthesis I (Figure S7, Supporting Information) (Table S22, Supporting Information).

### 2.6 Associations between the Oral Mycobiome and Clinical Indicators

**Figure 5:**
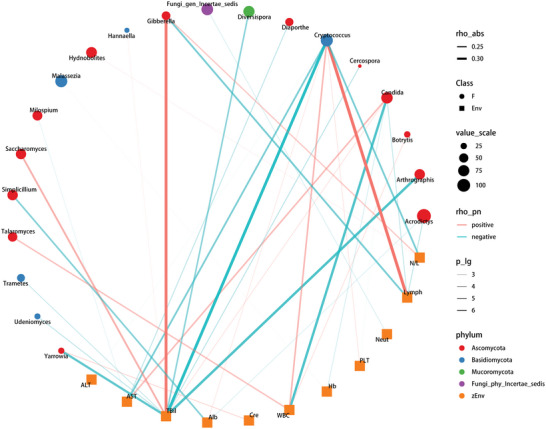
Oral mycobiome markers correlated with clinical indicators. The relationship between the 20 discriminative oral fungal genera and 11 discriminative clinical indicators in COVID‐19 (*n* = 71) and controls (*n* = 132) (*p* < 0.05 and absolute rho > 0.2). The colors of the circle points show the different phyla of the genera. The size of the circle points of each genus shows the mean relative abundance. The circle points represent the oral fungal genera, square points represent the clinical indicators. The transparency of the lines represents the negative logarithm (base 10) of the *p*‐value of correlation (Spearman's), red lines represent positive correlations, blue lines represent negative correlations, and the width of the lines represents the size of the correlation (Spearman's). RBC, red blood cell; WBC, white blood cell; NEUT, neutrophils; LYMPH, lymphocytes; Hb, hemoglobin; PLT, platelets; AST, aspartate aminotransferase; Alb, albumin; TBIL, total bilirubin; CREA, creatinine; N/L, the ratio neutrophils and lymphocytes.

The correlation between oral fungi and clinical indicators was identified in COVID‐19 patients and controls through Spearman correlation analysis (*p* < 0.05 and absolute rho > 0.2) (**Figure** **5** and Table S23, Supporting Information). We found that ten clinical indicators (AST, ALB, WBC, RBC, HB, PLT, CREA, NEUT, N/L, and LYMPH) were correlated with no more than five genera, while one clinical indicator (TBIL) was related to more than five genera. Among them, AST was negatively correlated with three genera (*Cryptococcus*, *Milospium*, and *Diaporthe*) and positively correlated with one genus (*Candida*). LYMPH was negatively correlated with three genera (*Acrodictys*, *Candida*, and *Gibberella*) and positively correlated with one genus (*Cryptococcus*). N/L was negatively related to one genus (*Cryptococcus*) and positively correlated with two genera (*Gibberella and Hydnobolites*). This explains the interaction of oral fungi, liver and kidney function, and routine blood tests, pathways that may be involved in affecting disease progression.

### 2.7 The Oral Mycobiome Correlated with Oral Bacteriome and Serum Lipidomics

**Figure 6:**
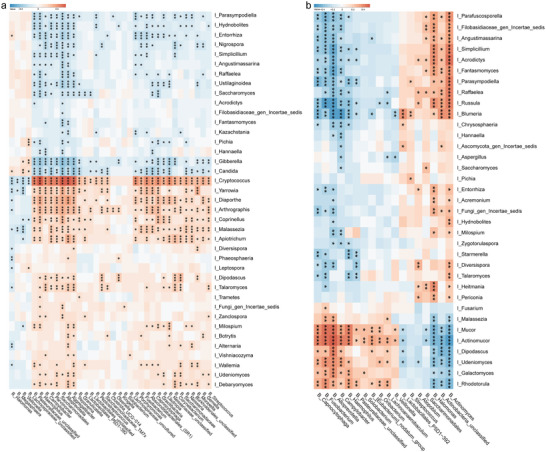
Associations between the oral mycobiome and the oral bacteriome. Spearman correlations of significantly altered oral mycobiome with significantly altered oral microbiota in a) COVID‐19 (*n* = 71) and controls (*n* = 79) or b) COVID‐19 (*n* = 71) and Post‐COVID‐19 (*n* = 21). Red color represents the positive correlation, and blue color represents the negative correlation. I, fungi; B, bacteria. * *p* < 0.05, ** *p* < 0.01, *** *p* < 0.001.

We performed additional oral bacterial differential analysis (Table S24, Supporting Information) and bacteria‐fungal correlation analysis at the genus level in order to further study the potential interplay between differential fungi and bacteria. For example, among distinct bacteria and fungi in COVID‐19 and controls, *Cryptococcus* was negatively related with 4 bacteria, including *Halomonas*, *Megasphaera*, and *Veillonella*, and positively related with 28 bacteria including *Streptococcus*, *Neisseria*, and *Fusobacterium*. *Candida* was negatively correlated with 25 bacteria, including *Campylobacter*, *Haemophilus*, and *Lautropia*, and positively correlated with 3 bacteria including *Halomonas*, *Veillonella*, and *Lachnospiraceae unclassified* (**Figure** **6**a) (Table S25, Supporting Information). Among distinct bacteria and fungi in COVID‐19 and Post‐COVID‐19, *Mucor* was negatively related with five bacteria, such as *Actinomyces*, *Halomonas*, and *Saccharimonadales*, and positively related with ten bacteria such as *Oribacterium*, *Campylobacter*, and *Alloprevotella* (Figure 6b) (Table S26, Supporting Information).

Moreover, the correlation between oral fungi and serum lipidomics was investigated through Spearman correlation analysis in the COVID‐19 and Post‐COVID‐19 groups (Figure S8, Supporting Information) (Table S27, Supporting Information). The results showed that two serum lipid molecules, including ChE (22:1) and SiE (18:1), were positively correlated with seven distinct fungi, namely *Simplicillium*, *Acrodictys*, *Raffaelea*, and *Russula*, and were negatively correlated with five distinct fungi such as *Actinomucor*, *Rhodotorula*, and *Galactomyces*.

## 3 Discussion

In this study, we identified the oral mycobiomes of COVID‐19 and recovered patients. Our previous study on the oral microbiome of COVID‐19 found that the Shannon index and richness index, reflecting oral bacterial diversity, were significantly decreased in COVID‐19 patients; however, in this study, we found that compared with controls, the richness index of oral fungi in COVID‐19 group was significantly increased, and the Shannon index was slightly increased, indicating increased fungal colonization. A study of enteric fungal characterization in COVID‐19 patients found alterations in the gut fungal diversity consistent with our results.^[^ ^14^ ^]^ We speculate that one of the possible reasons is that under normal conditions, bacteria in the oral cavity occupy the main living space and can limit the colonization and invasion of fungi and viruses. The colonization of SARS‐CoV‐2 restricted the growth of bacteria. After the reduction in bacteria, the inhibition of fungi was lost, resulting in fungal invasions and an increase in fungal diversity. After the first specimen was collected at the time of admission, some Post‐COVID‐19 patients received antibiotic therapy. The results of the analysis showed that the oral fungal diversity of the recovered patients was significantly lower than that of the control group, and the oral bacterial diversity gradually returned to normal, indicating that fungi require a longer recovery time than bacteria. This is similar to the results of a study where the authors found that bacterial communities recovered after 30 days of antibiotic treatment, while fungal communities did not, and that fungal communities shifted from a symbiotic to a competitive relationship.^[^ ^15^ ^]^ Moreover, from baseline to 90 days post‐treatment, bacterial diversity shifted from decreased to increased, and fungal diversity shifted from increased to decreased. We speculate that the possible reason for the decreased *α*‐diversity in Post‐COVID‐19 than that of HC is that bacteria recovered significantly faster than fungi, leading to a resumption of bacterial inhibition of fungi in the oral cavity and limiting fungal colonization. However, the causal relationship cannot be determined, and further research is needed.

We report for the first time the oral fungal characteristics of COVID‐19 and recovered patients, confirming that the fungal composition and abundance of COVID‐19 and recovered patients are significantly different from those of healthy individuals. The phyla *Ascomycota* and *Basidiomycota* are predominant for both COVID‐19 and healthy individuals, but the ratio of *Ascomycota* to *Basidiomycota* is higher in COVID‐19 patients. We identified an increase in opportunistic pathogens in patients with COVID‐19, such as *Candida*, *Saccharomyces*, and *Simplicillium*, whose pathogenicity has been reported. *Candida* is part of normal skin and mucosal microbial communities. The overgrowth of *Candida* can lead to thrush or life‐threatening invasive candidiasis in immunocompromised patients^[^ ^16^ ^]^ and is even involved in promoting colon cancer^[^ ^17^ ^]^ and oral cancer.^[^ ^18^ ^]^ *C. albicans* promotes the secretion of the toxin candidalysin by expressing fungal transcription factors, thereby destroying immune cells and inducing intestinal inflammation.^[^ ^19^ ^]^ In vitro and in a rat caries model, colonization by *C. albicans* was found to interact with *Streptococcus spp*. and alter the composition of the oral biofilm, increasing the acid production and cariesogenicity of the oral biofilm, thereby worsening the severity of caries lesions and dental demineralization.^[^ ^20^ ^]^ The biological roles of *Saccharomyces* in health and disease are inconsistent across studies. Researchers have found that *Saccharomyces* may be able to enhance host purine metabolism and uric acid, increase the permeability of the intestinal barrier, and ultimately aggravate colitis in mice.^[^ ^21^ ^]^ The presence of *Saccharomyces cerevisiae* in the intestinal microbiota is associated with impaired attention and executive function.^[^ ^22^ ^]^ *Simplicillium spp*. is pathogenic in humans under certain conditions and can cause respiratory and skin infections.^[^ ^23^ ^]^ The enrichment in the abundance of these opportunistic pathogens suggests their contribution to COVID‐19 progression and warrants further study.

The correlations between oral fungi and clinical indicators are reported in this article. For example, *Candida* was negatively correlated with white blood cells (rho = −0.310) and lymphocytes (rho = −0.233), suggesting that the interaction between *Candida* and human immune cells may be involved in the progression of COVID‐19. CD4+ T cells and innate lymphoid cells play a protective role in invasive candidiasis,^[^ ^24^ ^]^ and the reduction in lymphocytes in patients with COVID‐19 may induce *Candida* invasion and colonization, while *Candida* can destroy immune cells,^[^ ^19^ ^]^ induce inflammatory responses, and aggravate COVID‐19 progression. However, the results of the correlation analysis between clinical indicators and fungi can only suggest that there may be a relationship between the two and provide potential possible directions for subsequent studies. Whether there is a causal relationship and its specific mechanism, needs to be verified experimentally.

Dysbiosis of the human mycobiome has been associated with a variety of diseases, and fecal fungi, plasma fungi, intratumoral fungi, vaginal fungi, gastric mucosal fungi, and other fungi from several parts of the body have been found to be useful in the diagnosis of various diseases such as gastric cancer,^[^ ^25^ ^]^ schizophrenia,^[^ ^26^ ^]^ colorectal cancer,^[^ ^27^ ^]^ Crohn's disease,^[^ ^28^ ^]^ Type I diabetes,^[^ ^29^ ^]^ and Clostridium difficile infection.^[^ ^30^ ^]^ For example, Jun Yu et al. found that fungal fecal markers have the potential for CRC diagnosis with an AUC of 0.93 and successful cross‐national validation.^[^ ^31^ ^]^ Ningning Liu established CRC cohorts from eight countries/regions and found that intestinal fungi were used for CRC diagnosis and were best combined with bacteria and archaea.^[^ ^32^ ^]^ However, there has never been a study using oral fungi to diagnose COVID‐19 or even other diseases. Herein, we are the first to find that oral fungi can effectively distinguish COVID‐19 patients from healthy people, and the diagnostic performance is better than that of oral, pharyngeal, and intestinal bacterial markers. Moreover, the classifier was validated in the test phase, that is, Hangzhou independent cohort, suggesting that fungal markers have the potential to detect COVID‐19. More importantly, we found that the fungal classifier could diagnose suspected patients with negative nucleic acid but positive IgG antibody as COVID‐19, suggesting that fungal markers may be used as an auxiliary diagnostic tool for RT‐PCR, and the combination of the two will be able to identify potential COVID‐19 patients in the population. In addition, we found that the oral fungal diversity and composition of suspected patients and COVID‐19 patients were similar, confirming that suspected patients were undiagnosed patients with COVID‐19, which again demonstrated the feasibility of using this model to diagnose suspected patients with COVID‐19.

Bacteria and fungi have complex interactions, such as competition and symbiosis, and biofilms provide a venue for these interactions. Bacteria and fungi secrete signaling molecules and chemicals to inhibit or promote the survival and growth of each other, thereby affecting the immune function and health of the host. For example, *Pseudomonas aeruginosa* can affect the respiration of *C. albicans* by secreting phenazine compounds, thereby inhibiting its morphological transition and biofilm formation.^[^ ^33^ ^]^ *Bacteroides polymorpha* can activate host immune effector molecules, such as hypoxia‐inducible factor 1*α* and antimicrobial peptide LL‐37, conferring resistance to *C. albicans* colonization in the intestine.^[^ ^34^ ^]^ Compounds derived from lactic acid bacteria can inhibit the toxicology of *Candida parapsilosis*; reduce the proliferation, viability, and metabolic activity of the fungus; and increase the resistance of epithelial cells to the fungus.^[^ ^35^ ^]^ The interaction between bacteria and fungi can influence the development and progression of infection. Peptidoglycan fragments of bacterial cell walls activate adenylyl cyclase by binding to leucine‐rich repeat domains to promote the growth of *C. albicans* and increase susceptibility to *Candida* infection.^[^ ^36^ ^]^ Our findings revealed that alterations in some key oral fungi in COVID‐19 patients and recovered patients were closely related to changes in oral bacteria, suggesting that oral fungi and bacteria may interact to influence the disease progression of COVID‐19. For example, gram‐negative bacilli that produce the proinflammatory factor lipopolysaccharide, such as *Veillonella* and *Halomonas*, are enriched in COVID‐19 patients and are positively associated with *Candida*. *Prevotella* and *Alloprevotella*, which helps breakdown protein and carbohydrate foods, were reduced in COVID‐19 patients and increased gradually in recovered patients and were inversely associated with the opportunistic pathogen *Simplicillium*. The above results provide potential clues for exploring the pathogenesis of COVID‐19 and can be verified by experiments such as coculture.

We tried our best to elucidate the changes and potential contributions of oral fungi in COVID‐19, but there are still some deficiencies in this work. First, the SARS‐CoV‐2 we studied was the original strain, which did not include Delta, Omicron, and other mutant strains. Follow‐up work will collect specimens from patients infected with mutant strains for further research. Second, our sample size is relatively small, and we still need to validate the model with a larger, cross‐regional multi‐center population cohort to enhance the generalizability of the model in the future. Third, ITS sequencing results focus on reflecting the composition and evolutionary relationship of community species, but the information on the functional composition and metabolic pathways of community species is not accurate enough. Metagenome sequencing can solve this problem. Fourth, due to insufficient data in the existing fungal database, some unclassified fungi were not annotated by ITS sequencing; finally, omics studies can only describe the correlation between fungi and COVID‐19, as well as fungi and bacteria. A causal relationship cannot be established, and the molecular mechanism cannot be elucidated. Microbial‐related experiments are needed to validate our findings.

Taken together, our study elucidates the alterations in the oral mycobiome in COVID‐19 and recovered patients and demonstrates the potential usefulness of oral fungal markers in diagnosing COVID‐19 patients. Moreover, the classifiers could diagnose SCs as COVID‐19, making up for the deficiency of RT‐PCR, and may be used as an auxiliary diagnostic tool for RT‐PCR. Importantly, we reveal the ecological network in COVID‐19 and recovered patients, indicating that the synergistic and antagonistic interaction between bacteria and fungi may contribute to the progression of COVID‐19. These findings provide a new perspective on the pathology, diagnosis, and treatment of COVID‐19. Of course, more research and trials are needed in the future before they can be applied to the clinic.

## 4 Experimental Section

### Study Subjects and Design

This prospective study enrolled 71 COVID‐19 patients, 36 SCs in Henan Province, and 75 COVID‐19 patients in Zhejiang Province from February 2020 to March 2020. COVID‐19 patients and SCs were diagnosed and treated in accordance with the “COVID‐19 diagnosis and treatment plan” (trial version 5 or version 6) issued by the National Health Commission of the People's Republic of China.^[^ ^37^ ^]^ After admission, blood samples were collected from all patients for liver and kidney function and routine blood tests, and tongue coating samples were collected for subsequent ITS sequencing. Among them, 22 COVID‐19 patients and 36 SCs were followed up 2 weeks after discharge, and tongue‐coating samples were collected (Figure S1, Supporting Information).

A total of 132 age‐ and sex‐matched healthy individuals (controls) who were negative for SARS‐CoV‐2 by RT‐PCR were recruited from the First Affiliated Hospital of Zhengzhou University from January 2021 to March 2021. Controls had not taken antibiotics or probiotics in the past 8 weeks and had no underlying disease.

The detailed inclusion and exclusion criteria of participants are described in the Experimental Section, Supporting Information. Patient data, such as demographic data, epidemiological characteristics, clinical symptoms and signs, and laboratory test results, were obtained from electronic medical records (Table S1, Supporting Information). All participants provided informed consent to be involved in this research. This study was approved by the Ethics Committee of the First Affiliated Hospital of Zhengzhou University (2020‐KY‐055). The study was carried out in accordance with the Declaration of Helsinki.

### Sample Collection and IgG Antibody Detection

Each subject provided a sample of tongue coating. Participants first rinsed their mouths with saline twice, and then a professional scraped the tongue coating from the posterior‐medial to the anterior‐medial area with a throat swab. The swab head was placed into a freezing tube containing virus preservation solution and then transferred to a −80 °C freezer for storage.

Serum samples from recovered COVID‐19 patients (Post‐COVID‐19) and recovered SCs (SCR) were used to detect specific SARS‐CoV‐2 IgG antibody levels through chemiluminescence immunoassays (kits from Shenzhen Mairui Biomedical Electronics Co., Ltd., Guangdong). The positive cutoff value was defined as 10 U mL^−1^.

All samples were inactivated at 56 °C for at least 30 min. The collection, transportation, storage, and testing of all COVID‐19‐related samples were strictly implemented in accordance with the “COVID‐19 Prevention and Control Plan (Fifth Edition).”^[^ ^38^ ^]^

### DNA Extraction

Each sample was placed in a 2‐mL centrifuge tube, and 790 µL of lysis buffer (4 m guanidine thiocyanate; 10% *N*‐lauroyl sarcosine; 5% *N*‐lauroyl sarcosine‐0.1 m phosphate buffer (pH 8.0)) was added, followed by vigorous vortexing and incubation at 70 °C for 1 h. After incubation, 750 µL of glass beads (0.1 mm) was added to mix and beat for 10 min (25 HZ/S). Subsequent extraction was performed according to the instructions of the extraction kit (E.Z.N.A. Stool DNA Kit) (Experimental Section, Supporting Information).

### PCR Amplification

The universal primers ITS3F (5′‐GCATCGATGAAGAACGCAGC‐3′) and ITS4R (5′‐ TCCTCCGCTTATTGATATGC‐3′) were used for amplification of the ITS2 region of the fungal ITS gene. The PCR system included 12.5 µL of 2× Phanta Max Buffer, 0.5 µL of 10 mm dNTPs, 0.5 µL of each primer (10 µm), 0.5 µL of Phanta Max Super‐Fidelity DNA Polymerase, 5 µL of template DNA, and 5.5 µL of ddH_2_O. Samples were subjected to four reactions using a PCR machine (ABI GeneAmp 9700): 95 °C for 3 min; 35 cycles of 94 °C for 30 s, 55 °C for 30 s, and 72 °C for 30 s; and finally 72 °C for 5 min. Agarose gel (Axygen Biosciences, Union City, CA) was used to separate, extract, and purify the PCR products, and a fluorescence assay kit (Quant‐iT PicoGreen, Invitrogen) to quantify the products.

### Library Construction and Sequencing

The amplified products were purified by the magnetic bead method, and the purified products were used to construct DNA libraries according to the official operating instructions. The library products of different samples were mixed in an equimolar ratio, and then single‐end sequencing analysis was performed using the Illumina MiSeq platform (Shanghai Mobio Biomedical Technology, China). Raw Illumina read data of all specimens were deposited in the European Bioinformatics Institute European Nucleotide Archive database (PRJNA850097).

### OTU Clustering and Taxonomy Annotation

Clean single‐read data were extracted from raw data using USEARCH (version 11.0.667), and reads with >1 expected error per base were discarded. Quality‐filtered sequences were clustered into unique sequences and sorted in order of decreasing abundance to identify representative sequences using UPARSE according to the UPARSE OTU analysis pipeline, and singletons were omitted in this step. OTUs were classified based on 98.5% similarity after chimeric sequences were removed using UPARSE (version 7.1 http://drive5.com/uparse/) and annotated using unite v8.3 (https://unite.ut.ee/repository.php). Those annotated as unclassified fungi were further subjected to BLAST against ITS_RefSeq_Fungi version 1.1 (ftp://ftp.ncbi.nlm.nih.gov/blast/db/ITS_RefSeq_Fungi.tar.gz).

### Bioinformatics and Statistical Analysis

Alpha diversity metrics (ACE estimator, Chao 1 estimator, Shannon‒Wiener diversity index, and Simpson diversity index) were assessed by using Mothur v1.42.1. Both Bray–Curtis and weighted and unweighted UniFrac dissimilarity were calculated in QIIME. PCoA, NMDS plots, and PERMANOVA, which were used to test for statistical significance between the groups using 10 000 permutations, were generated in the R (version 3.6.0) package vegan 2.5‐7. The linear discriminant analysis (LDA) effect size (LEfSe) was used to detect taxa with differential abundance among groups (lefse 1.1, https://github.com/SegataLab/lefse). PICRUSt2 v2.4.1 (https://github.com/picrust/picrust2/wiki) was used to predict functional abundances based on ITS rRNA gene sequences.

Data are presented as the mean values ± standard deviations (SDs) for continuous variables and the number (percentage) for categorical variables. The nonparametric Mann–Whitney U test was used to compare significant differences between two groups for nonnormally distributed continuous variables. Student's *t*‐test was used to compare differences between two groups for normally distributed continuous variables. The 𝜒 2‐test or Fisher's exact test was used for categorical variables. A comparison of nonnormally distributed continuous variables in multiple groups was performed using a nonparametric Kruskal–Wallis test. Values of *p* < 0.05 were defined as statistically significant. SPSS V.17.0 (SPSS, Chicago, Illinois, USA) was used to complete the statistical analysis.

### Ethics Approval and Consent to Participate

This study was approved by the Institutional Review Board from the First Affiliated Hospital of Zhengzhou University (2020‐KY‐055). The study was performed in accordance with the Helsinki Declaration and Rules of Good Clinical Practice. All participants signed written informed consent after the study protocol was fully explained.

### Patient and Public Involvement

Patients or the public were not involved in the design, conduct, reporting, or dissemination plans of our research.

## Conflict of Interest

The authors declare no conflict of interest.

## Author Contributions

X.H., H.W., B.Y., and J.Y. contributed equally to this work. Z.R., Y.J. and Z.W. designed the study. Z.R., Y.J., X.H., H.L., and Z.Z. treated the patients. H.W., B.Y., X.H., J.Y., J.S., Y.S., Y.Z., H.L., Z.Z., and S.L. collected clinical samples and clinical data. Z.W., Y.J., and HF.L. extracted bacterial DNA and completed MiSeq sequencing. H.W., B.Y., and J.Y. analyzed the data. H.W. and Z.R. wrote the manuscript. All authors reviewed and approved the manuscript.

## Supporting information

Supporting Information

## Data Availability

The raw Illumina read data for all samples were deposited in the European Bioinformatics Institute European Nucleotide Archive database (accession number: PRJNA850097). Correspondence and requests for materials should be addressed to Z.R.
