# Supplementary material for: Oral Fungal Alterations in Patients with COVID‐19 and Recovered Patients
Source: Adv Sci (Weinh). 2023 Apr 29;10(19):2205058. doi: 10.1002/advs.202205058 (PMC10323652; doi:10.1002/advs.202205058)
Supplement: Supplementary file 1 — Supporting Information [file ADVS-10-2205058-s001.pdf]

ADVANCED SCIENCE
Open Access

Supporting Information

for *Adv. Sci.*, DOI 10.1002/advs.202205058

Oral Fungal Alterations in Patients with COVID-19 and Recovered Patients

Xiaobo Hu, Haiyu Wang, Bo Yu, Jia Yu, Haifeng Lu, Junyi Sun, Ying Sun, Yawen Zou, Hong Luo, Zhaohai Zeng, Shanshuo Liu, Yan Jiang, Zhongwen Wu* and Zhigang Ren**

Supplementary Method

Enrollment process

The isolation ward is divided into two areas in hospital, one for suspected cases ward and the other for confirmed cases ward. Patients with fever, cough, or other related symptoms, or clinical confirmed COVID-19 were admitted to suspected cases ward. After hospitalization, the throat swab specimens were collected for nucleic acid test by PT-PCR the next day. Patients were transferred to confirmed cases ward if the nucleic acid test was positive. Then our researcher evaluated the patient whether meeting our criteria and enrolled eligible patients after signing written informed consent. Patients with exposure to confirmed cases or Wuhan, but without symptoms, were isolated outside the hospital and collected throat swab specimens. Patients were admitted to hospital and transferred to confirmed cases ward if the result of nucleic acid test was positive. Then researchers screened and enrolled eligible patients.

Convalescent patients were transferred to another independent isolated region under observation for 15 days. If the nucleic acid test was still negative during this period, convalescent patients could departure from the independent isolated region. Samples from recovery patients were collected two days before discharge. The samples collection methods were consistent with our previous description.

Diagnostic, inclusion, and exclusion criteria

Diagnostic criteria are based on the "Diagnosis and treatment program trial version 5 guidelines" issued by the National Health Commission of the People's Republic of China.

1. Suspected cases.

Combined with the following comprehensive analysis of epidemiological history and clinical manifestations: any one of the epidemiological history and any two of the clinical manifestations, or no clear epidemiological history and three of the clinical manifestations can be diagnosed as a suspected case.

(1) History of Epidemiology

1) Travel history or residence history of Wuhan city and surrounding areas, or other communities with case reports within 14 days before onset.
2) A history of contact with a infected person (positive nucleic acid test) within 14 days before onset.
3) Have contacted patients with fever or respiratory symptoms from Wuhan city and surrounding areas, or from communities with case reports within 14 days before onset.
4) Aggregated onset (2 or more cases of fever and / or respiratory symptoms occurred within 2 weeks in a small area such as home, office, school class, etc.,).
(2) Clinical manifestations
1) Fever and / or respiratory symptoms.
2) With the characteristic image of COVID-19.
3) The total number of white blood cells in the early stage of the disease is normal or decreased, and the lymphocyte count is normal or decreased.

2. Confirmed cases (COVID-19 patients).
Suspected cases with one of the following etiological or serological evidence:
(1) Fluorescence RT-PCR detects the positive of the COVID-19 virus nucleic acid.
(2) Viral gene sequencing finds highly homologous to COVID-19 virus.

3. Cured patients (Convalescent patients).
Need to meet the following conditions at the same time:
(1) Body temperature returned to normal for more than 3 days.
(2) Respiratory symptoms improved significantly.
(3) Pulmonary imaging showed a significant improvement in acute exudative lesions.
(4) Two consecutive sputum, nasopharyngeal swabs and other respiratory tract specimens with negative nucleic acid test (sampling time needs to be at least 24 hours apart).

4. Healthy volunteers
1) Inclusion criteria.

(1) Aged ≥18 years old.

(2) Competent to provide informed consent (no mental illness or dementia).

(3) Have lived in the same geographic area for the preceding 6 months.

(4) All enrolled individuals had healthy oral tissues and gingiva (determined by a professional dentist).

2) exclusion criteria

(1) Individuals who received antibiotics and/or probiotics within 8 weeks before enrolment were also excluded.

(2) Current pregnancy.

(3) The use of laxatives or anti-diarrheal drugs in the last 3 months.

(4) Have no underlying infectious, acute disease, or severe organ failure.

The inclusion criteria for healthy volunteers referred to our previous study. In all healthy controls, exclusion criteria included hypertension, diabetes, obesity, metabolic syndrome, irritable bowel syndrome (IBD), nonalcoholic fatty liver disease, coeliac disease and liver cirrhosis.

All enrolled individuals had healthy oral tissues and gingiva (determined by a professional dentist). Individuals who received antibiotics and/or probiotics within 8 weeks before enrolment were also excluded.

DNA extraction

Centrifuge at 14,000 rpm for 5 min, take the supernatant and place it in a new 2 mL centrifuge tube, add 250 μL SP2 Buffer, and vortex for 5 min in ice bath; 200 μL of cHTR Reagent, mixed and placed at room temperature for 2 min; 14,000 rpm, 2 min, take 500 μL of supernatant and put it in a new 2 ml centrifuge tube, add 20 μL proteinase K (20 mg/ml), 500 μL BL buffer, vortex and mix, Incubate at 70°C for 10 min; after incubation, add 500 μL of absolute ethanol, insert the HiBind® DNA spin column into a 2 mL collection tube, centrifuge (12,000 rpm, 1 min), and discard the waste solution; 500ul HB Buffer once, 750ul Wash Buffer twice time, discard the waste solution; vacate

the collection tube and spin column for 1 min and leave at room temperature for 2 min; transfer the spin column to a new 1.5 ml collection tube and elute with 100 μL of Elution Buffer.

Construction of POD

We used the abundance profile of the optimal OTUs markers in the discovery set to perform fivefold cross-validation on a random forest model (R 3.4.1, randomForest 4.6-12 package). Then we acquired the cross-validation error curve through five trials of the fivefold cross-validation. We defined the point with the minimum cross-validation error as the cut-off point through the minimum error plus the standard deviation (SD). The sets of OTU markers with the error less than the cut-off value were listed and the set with the smallest number of OTUs were defined as the optimal set. Then we used the optimal set of OTUs to calculate the probability of disease (POD) index in discovery cohort and validation cohort. The constructed models was evaluated using the receiver operating characteristic (ROC) curve (R 3.3.0, pROC package), and the ROC effect was showed using AUC.

Supplementary Figures Legend

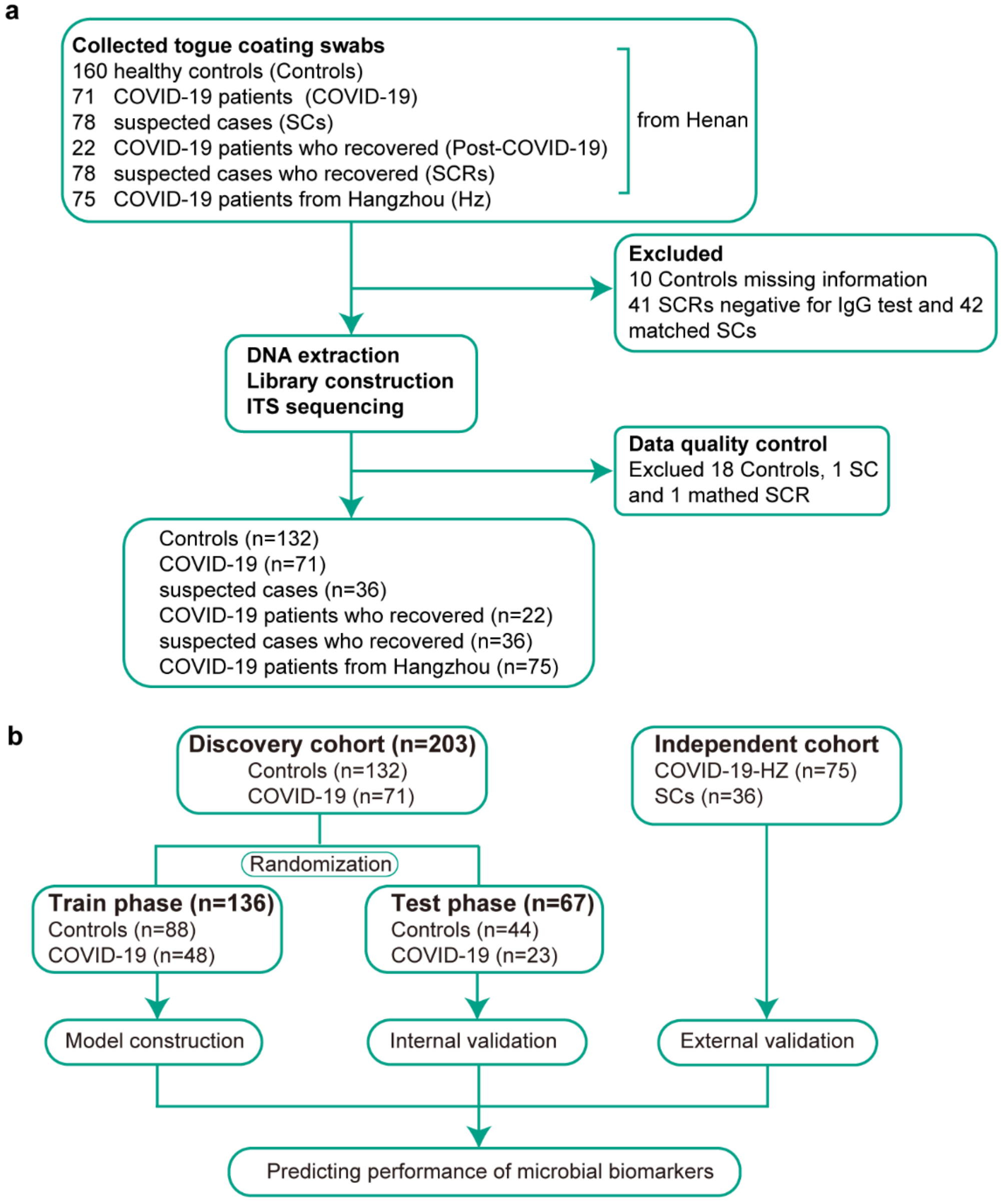

Fig.S1 Study design and flow diagram.

(a) A total of 484 tongue coating specimens from Central China and East China were collected. After screening, 372 samples were used for further sequencing using ITS sequencing, including 71 COVID-19 patients from Henan, 36 SCs from Henan, 20 matched Post-COVID-19, 36 matched SCRs, 132 controls from Henan, and 75 COVID-19 patients from Hangzhou. (b)To investigate the potential of oral fungi for diagnostic markers, random-forest classifiers were built to discriminate 71 COVID-19 from 132 controls in the discovery cohort. Five-fold cross-validated random-forest model in the training phase (two thirds of the samples in each group randomly selected

from the discovery cohort) was performed to select key discriminatory oral fungal markers. The remaining 30% samples from the discovery cohort were used to validate the diagnosis efficacy of classifiers. Moreover, 75 COVID-19 from Hangzhou, and 36 SCs served as an independent external validation phase to verify the potential of the classifiers. ITS, internal transcribed spacer; SCs, suspected cases; Post-COVID-19, recovered COVID-19 patients; SCRs, suspected cases who recovered; RFC, random forest classifier.

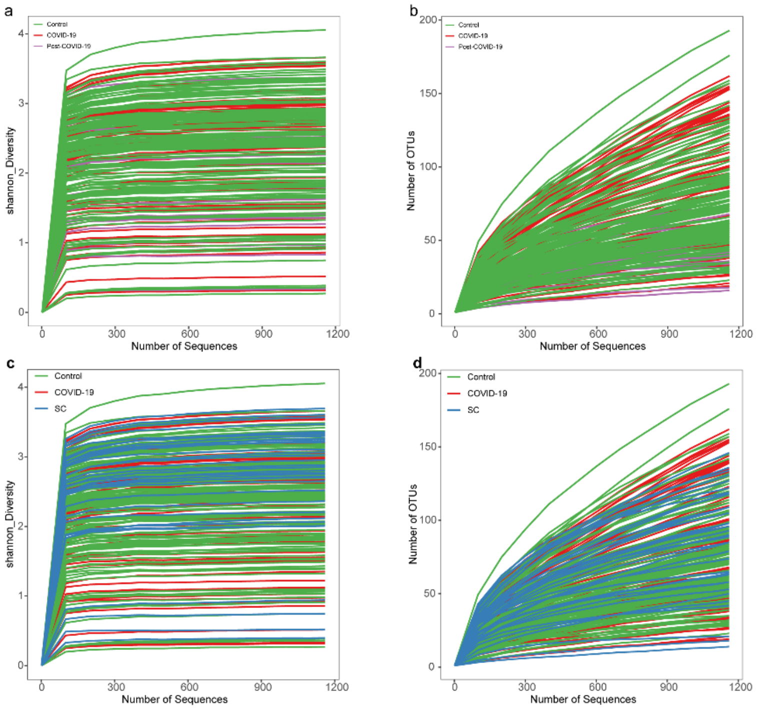

Fig.S2 The rarefaction analysis of oral fungi. Rarefaction analysis showed as the number of sequences raised, (a) shannon diversity and (b) the number of OTUs approached saturation in COVID-19 (n=71), Post-COVID-19 (n=22), and controls (n=132). As the number of sequences raised, (c) shannon diversity and (d) the number of OTUs approached saturation in COVID-19 (n=71), SCs (n=22), and controls (n=132). SCs, suspected cases; Post-COVID-19, recovered COVID-19 patients.

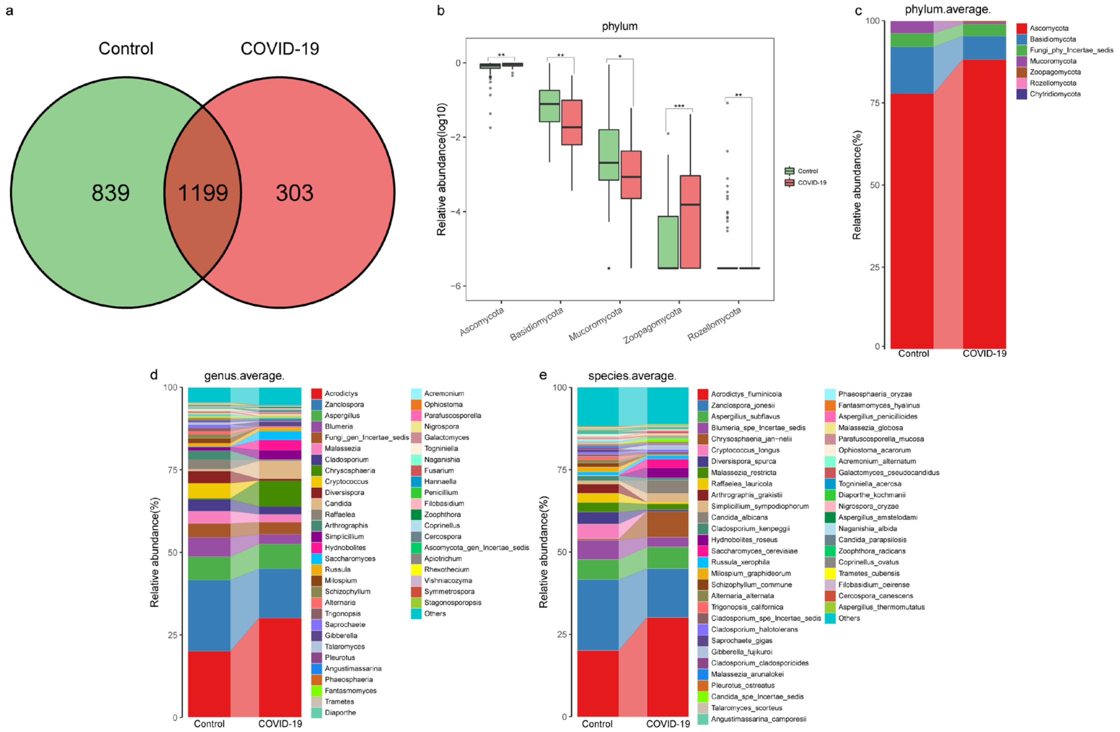

Fig.S3 The oral fungal alterations between COVID-19 patients and controls. (a) A Venn diagram revealed that 1199 of 2341 OTUs were shared in the COVID-19 and controls, while 303 OTUs were unique to COVID-19. (b) Two phyla were remarkedly increased, while 3 phyla were remarkedly reduced in COVID-19 versus controls. Average compositions and relative abundance of the fungal community in both groups at the (c) phylum, (d) genus, (e) species levels. * $p < 0.05$, ** $p < 0.01$, *** $p < 0.001$.

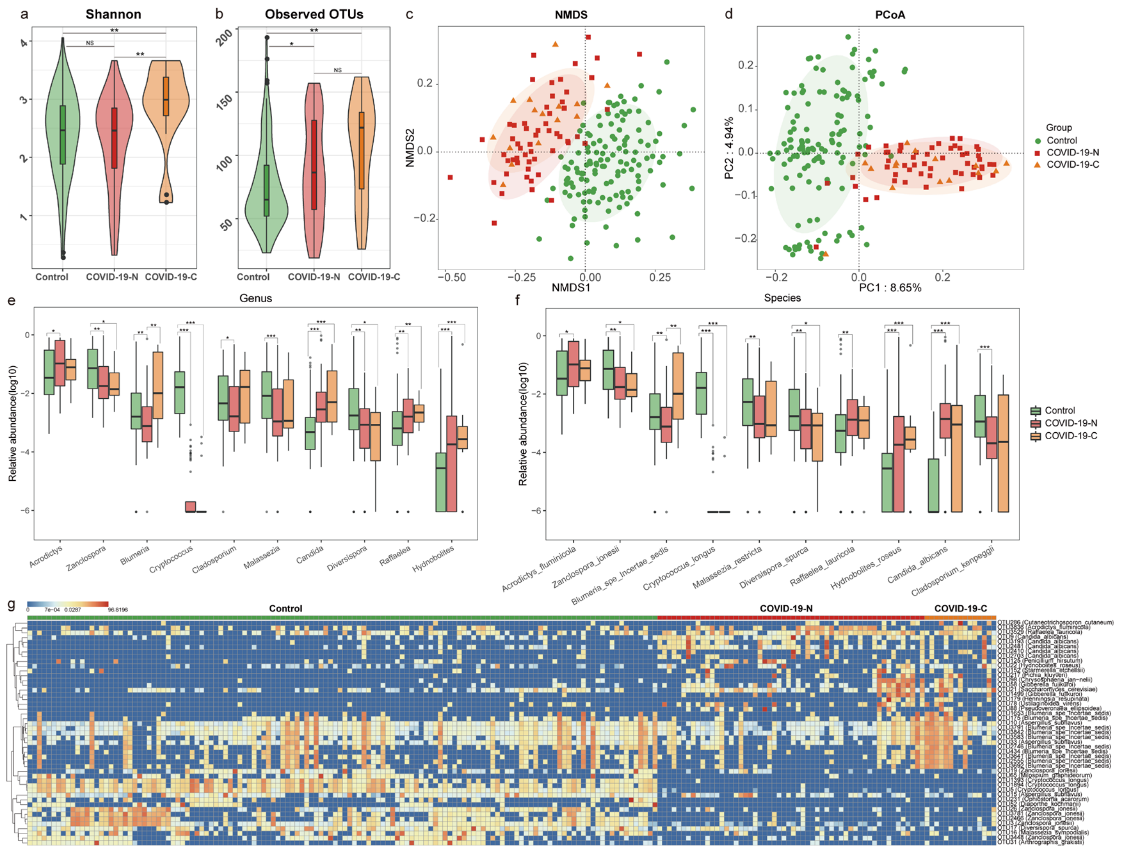

Fig.S4 The characterization of oral fungi among COVID-19-N, COVID-19-C, and control groups. (a) Shannon index and (b) Observed OTUs diversity indices among COVID-19-N (n=56), COVID-19-C (n=15), and control (n=132) groups. The NMDS (c) and PCoA (d) analysis showed the oral fungal taxonomic composition among three groups. The 10 most abundant fungi among the differential fungi among three groups at the (e) genus and (f) species levels. (g) Heatmap showed the relative abundances of differential OTUs for each sample in both groups. COVID-19-N, COVID-19 patients without comorbidities; COVID-19-C, COVID-19 patients with comorbidities; OTUs, operational taxonomy units; PCoA, principal coordinate analysis; NMDS, nonmetric multidimensional scaling. NS, no significance, * $p < 0.05$, ** $p < 0.01$, *** $p < 0.001$.

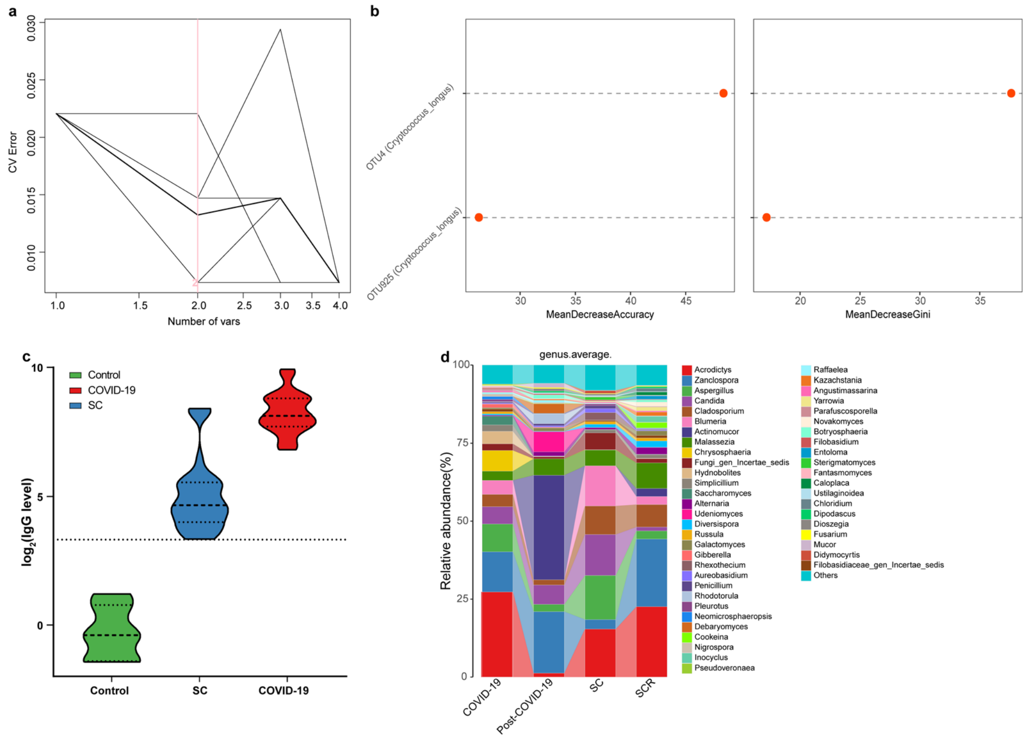

Fig.S5. The COVID-19 diagnostic model and the characterization of oral fungi among COVID-19, Post-COVID-19, SCs, and SCRs. (a) Importance distribution map of the selected microbial markers in the model. (b) Two fungal markers were selected as the best markers set by random forest model. (c) Levels of antibodies against SARS-CoV-2 in COVID-19 (n=22), SCs (n=36), and controls (n=6) after recovery. The positive judgment value was 10 U/ml in the kit (> 10 U/ml was positive, and < 10 U/ml was negative). The antibody levels in the figure were computed as log2(value). (d) Average compositions and relative abundance of the fungal community in COVID-19, Post-COVID-19, SCs, and SCRs at the genus level. SCs, suspected cases; Post-COVID-19, recovered COVID-19 patients; SCRs, suspected cases who recovered.

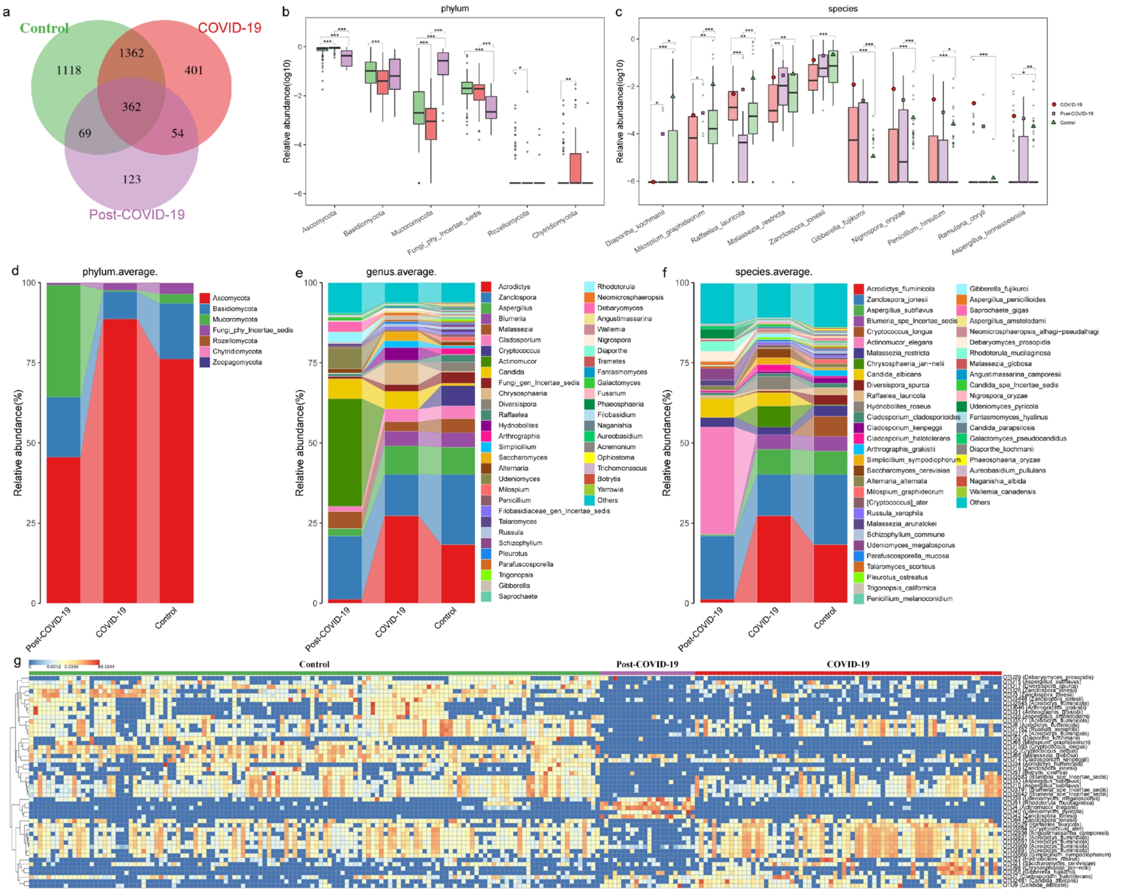

Fig.S6 The characterization of oral mycobiome in COVID-19, Post-COVID-19, and control groups. (a) A Venn diagram revealed that 362 of 3489 OTUs were shared in three groups, while 123 OTUs were unique to Post-COVID-19. The differential fungi among three groups at the (b) phyla and (c) species levels. Average compositions and relative abundance of the fungal community in three groups at the (d) phylum, (e) genus, (f) species levels. (g) Heatmap of the relative abundances of differential OTUs for each sample in the three groups. * $p < 0.05$, ** $p < 0.01$, *** $p < 0.001$. COVID-19, COVID-19 patients; Post-COVID-19, recovered COVID-19 patients.

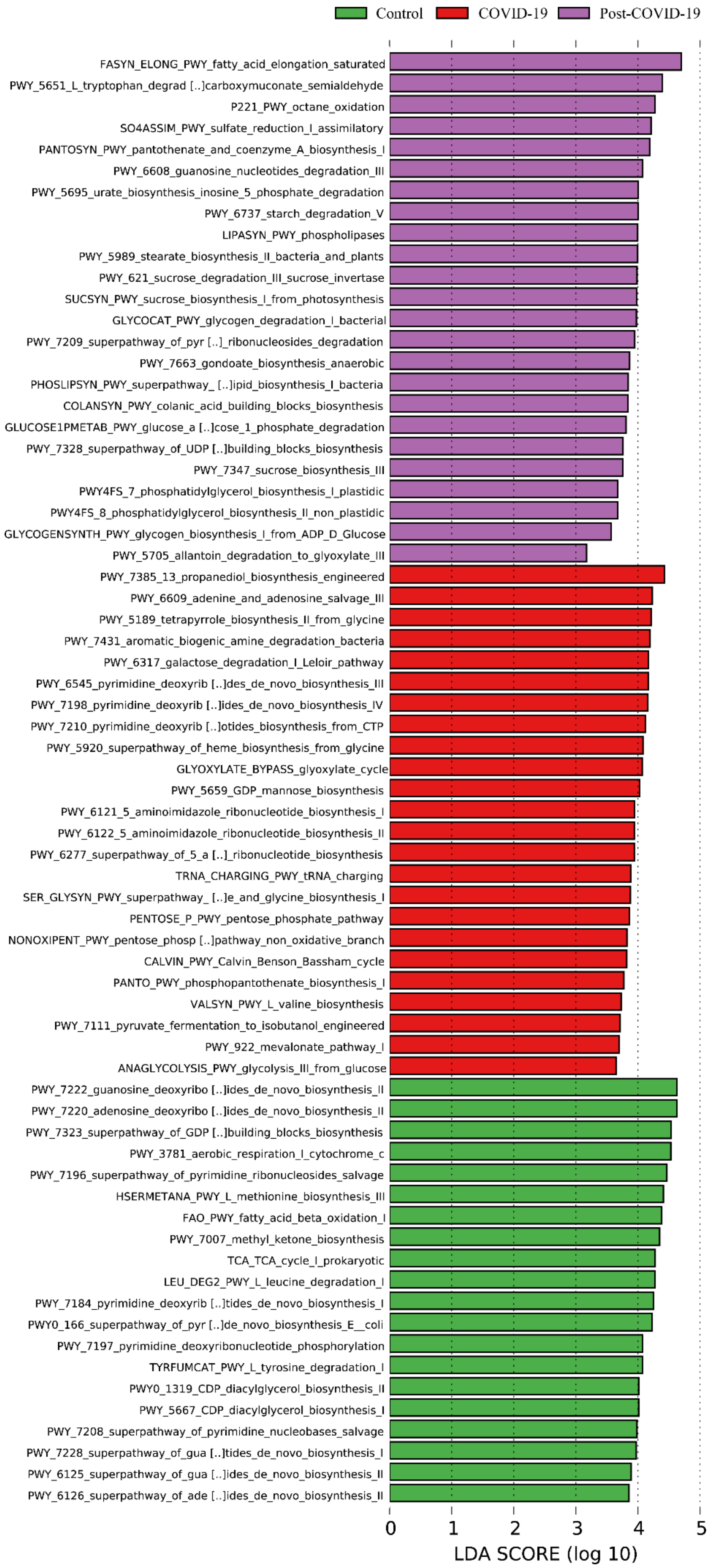

Control
COVID-19
Post-COVID-19
FASYN_ELONG_PWY_fatty_acid_elongation_saturated
PWY_5651_L_tryptophan_degrad [..]carboxymuconate_semialdehyde
P221_PWY_octane_oxidation
SO4ASSIM_PWY_sulfate_reduction_I_assimilatory
PANTOSYN_PWY_pantothenate_and_coenzyme_A_biosynthesis_I
PWY_6608_guanosine_nucleotides_degradation_III
PWY_5695_urate_biosynthesis_inosine_5_phosphate_degradation
PWY_6737_starch_degradation_V
LIPASYN_PWY_phospholipases
PWY_5989_stearate_biosynthesis_II_bacteria_and_plants
PWY_621_sucrose_degradation_III_sucrose_invertase
SUCSYN_PWY_sucrose_biosynthesis_I_from_photosynthesis
GLYCOCAT_PWY_glycogen_degradation_I_bacterial
PWY_7209_superpathway_of_pyr [..]_ribonucleosides_degradation
PWY_7663_gondoate_biosynthesis_anaerobic
PHOSLIPSYN_PWY_superpathway_ [..]ipid_biosynthesis_I_bacteria
COLANSYN_PWY_colanic_acid_building_blocks_biosynthesis
GLUCOSE1PMETAB_PWY_glucose_a [..]cose_1_phosphate_degradation
PWY_7328_superpathway_of_UDP [..]building_blocks_biosynthesis
PWY_7347_sucrose_biosynthesis_III
PWY4FS_7_phosphatidylglycerol_biosynthesis_I_plastidic
PWY4FS_8_phosphatidylglycerol_biosynthesis_II_non_plastidic
GLYCOGENSYNTH_PWY_glycogen_biosynthesis_I_from_ADP_D_Glucose
PWY_5705_allantoin_degradation_to_glyoxylate_III
PWY_7385_13_propanediol_biosynthesis_engineered
PWY_6609_adenine_and_adenosine_salvage_III
PWY_5189_tetrapyrrole_biosynthesis_II_from_glycine
PWY_7431_aromatic_biogenic_amine_degradation_bacteria
PWY_6317_galactose_degradation_I_Leloir_pathway
PWY_6545_pyrimidine_deoxyrib [..]des_de_novo_biosynthesis_III
PWY_7198_pyrimidine_deoxyrib [..]ides_de_novo_biosynthesis_IV
PWY_7210_pyrimidine_deoxyrib [..]otides_biosynthesis_from_CTP
PWY_5920_superpathway_of_heme_biosynthesis_from_glycine
GLYOXYLATE_BYPASS_glyoxylate_cycle
PWY_5659_GDP_mannose_biosynthesis
PWY_6121_5_aminoimidazole_ribonucleotide_biosynthesis_I
PWY_6122_5_aminoimidazole_ribonucleotide_biosynthesis_II
PWY_6277_superpathway_of_5_a [..]_ribonucleotide_biosynthesis
TRNA_CHARGING_PWY_tRNA_charging
SER_GLYSYN_PWY_superpathway_ [..]e_and_glycine_biosynthesis_I
PENTOSE_P_PWY_pentose_phosphate_pathway
NONOXIPENT_PWY_pentose_phosp [..]pathway_non_oxidative_branch
CALVIN_PWY_Calvin_Benson_Bassham_cycle
PANTO_PWY_phosphopantothenate_biosynthesis_I
VALSYN_PWY_L_valine_biosynthesis
PWY_7111_pyruvate_fermentation_to_isobutanol_engineered
PWY_922_mevalonate_pathway_I
ANAGLYCOLYSIS_PWY_glycolysis_III_from_glucose
PWY_7222_guanosine_deoxyribo [..]ides_de_novo_biosynthesis_II
PWY_7220_adenosine_deoxyribo [..]ides_de_novo_biosynthesis_II
PWY_7323_superpathway_of_GDP [..]building_blocks_biosynthesis
PWY_3781_aerobic_respiration_I_cytochrome_c
PWY_7196_superpathway_of_pyrimidine_ribonucleosides_salvage
HSERMETANA_PWY_L_methionine_biosynthesis_III
FAO_PWY_fatty_acid_beta_oxidation_I
PWY_7007_methyl_ketone_biosynthesis
TCA_TCA_cycle_I_prokaryotic
LEU_DEG2_PWY_L_leucine_degradation_I
PWY_7184_pyrimidine_deoxyrib [..]tides_de_novo_biosynthesis_I
PWY0_166_superpathway_of_pyr [..]de_novo_biosynthesis_E__coli
PWY_7197_pyrimidine_deoxyribonucleotide_phosphorylation
TYRFUMCAT_PWY_L_tyrosine_degradation_I
PWY0_1319_CDP_diacylglycerol_biosynthesis_II
PWY_5667_CDP_diacylglycerol_biosynthesis_I
PWY_7208_superpathway_of_pyrimidine_nucleobases_salvage
PWY_7228_superpathway_of_gua [..]tides_de_novo_biosynthesis_I
PWY_6125_superpathway_of_gua [..]ides_de_novo_biosynthesis_II
PWY_6126_superpathway_of_ade [..]ides_de_novo_biosynthesis_II
0
1
2
3
4
5
LDA SCORE (log 10)

Fig.S7 Linear discriminant analysis (LDA) among COVID-19, COVID-19-C, and control groups. (a) Based on the LDA selection, 24 gene functions were remarkedly raised in Post-COVID-19, 24 gene functions were significantly increased in COVID-19, while 20 gene functions were notably increased in controls. (p<0.05, LDA>3.0).

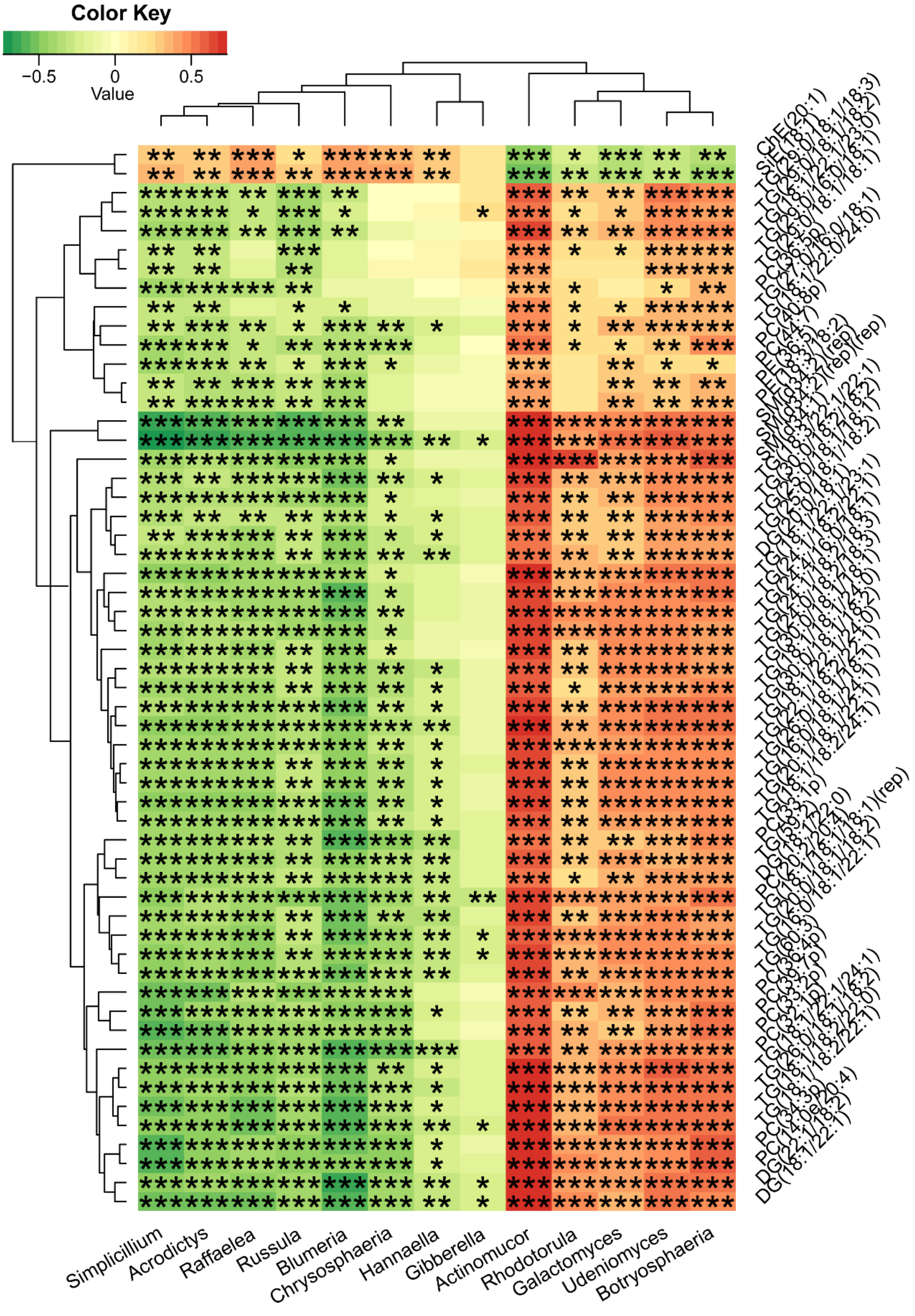

Fig.S8 Association between oral mycobiome and serum lipidomics in COVID-19 and Post-COVID-19. Spearman correlations of significantly altered oral mycobiome with significantly altered serum lipid in COVID-19 (n=71) and Post-COVID-19 (n=22). Red color represents the positive correlation, and blue color represents the negative correlation. * $p < 0.05$, ** $p < 0.01$, *** $p < 0.001$.

Supplementary table legends

Supplementary table S1. Clinical data of COVID-19 patients (n=71) and controls (n=132).

Supplementary table S2. The detailed values of oral fungal α-diversity index in the control (n=132) and COVID-19 (n=71).

Supplementary table S3. The abundance and composition of mycobiome at the phylum, genus, and species levels of each sample in the control (n=132) and COVID-19 (n=71) groups.

Supplementary table S4. The different degree of mycobiome at the phylum, genus, and species levels (p value) in the control (n=132) and COVID-19 (n=71) groups.

Supplementary table S5. The relative abundance and distribution of the key 43 OTUs in the control (n=132) and COVID-19 (n=71) groups.

Supplementary table S6. The detailed values of oral fungal α-diversity index in the control (n=132), COVID-19-N (n=56), and COVID-19-C (n=15) groups.

Supplementary table S7. The different degree of mycobiome at the phylum, genus, and species levels (p value) in the control (n=132), COVID-19-N (n=56), and COVID-19-C (n=15) groups.

Supplementary table S8. The relative abundance and distribution of the key 47 OTUs in the control (n=132), COVID-19-N (n=56), and COVID-19-C (n=15) groups.

Supplementary table S9. By random forest classifier model, the corresponding abundance of each optimal fungal marker and POD value in each sample in the train phase (88 controls and 48 COVID-19).

Supplementary table S10. By random forest classifier model, the corresponding abundance of each optimal fungal marker and POD value in each sample in the test phase (44 controls and 23 COVID-19).

Supplementary table S11. By random forest classifier model, the corresponding abundance of each optimal fungal marker and POD value in each sample in the Hangzhou independent cohort (44 controls and 75 COVID-19 from Hangzhou).

Supplementary table S12. The levels of Ig-G antibody in COVID-19 (n=20), SC (n=36), and controls (n=6).

Supplementary table S13. By random forest classifier model, the corresponding abundance of each optimal fungal marker and POD value in each sample in the SC (n=36) and control (n=44) groups.

Supplementary table S14. The detailed values of oral fungal diversity index and observed OTUs among COVID-19 (n=71), SC (n=36), and control (n=132) groups.

Supplementary table S15. The relative abundance and distribution of the key 47 OTUs among COVID-19 (n=71), SC (n=36), and control (n=132) groups.

Supplementary table S16. The average abundance and composition at the genus level among COVID-19 (n=71), SC (n=36), Post-COVID-19 (n=22), and SCR (n=36) groups.

Supplementary table S17. The detailed values of oral fungal diversity index and observed OTUs among COVID-19 (n=71), Post-COVID-19 (n=22), and controls (n=132) groups.

Supplementary table S18. The abundance and composition at the phylum, genus, and species levels of each sample among COVID-19 (n=71), Post-COVID-19 (n=22), and controls (n=132) groups.

Supplementary table S19. The relative abundance and distribution of the key 49 OTUs among the COVID-19 (n=71), Post-COVID-19 (n=22), and controls (n=132) groups.

Supplementary table S20. The different degree of mycobiome at the phylum, genus, and species levels (p value) among the COVID-19 (n=71), Post-COVID-19 (n=22), and controls (n=132) groups.

Supplementary table S21. The cladogram of oral fungal structure and their predominant fungal in the COVID-19 (n=71), Post-COVID-19 (n=22), and controls (n=132) groups.

Supplementary table S22. The corresponding LDA value and p value of fungal community gene function for samples in the COVID-19 (n=71), Post-COVID-19 (n=22), and controls (n=132) groups.

Supplementary table S23. The p value and correlation coefficient between 20 oral fungal OTUs and 11 clinical indicators of COVID-19 (n=71) and controls (n=132) obtained by spearman correlation analysis.

Supplementary table S24. The different bacteria of genus level (p value) between the control (n=79) and COVID-19 (n=71) groups.

Supplementary table S25. The p value and correlation coefficients between fungi and bacteria in control (n=79) and COVID-19 (n=71) groups.

Supplementary table S26. The p value and correlation coefficients between fungi and bacteria in COVID-19 patients (n=71) and Post-COVID-19 (n=21).

Supplementary table S27. The p value and correlation coefficients between fungi and lipid in COVID-19 patients (n=71) and Post-COVID-19 (n=22).

Supplementary Table S1. Clinical data of all enrolled subjects

Number	Group	Age	Sex	ALT	AST	TBIL	ALB	CREA	WBC	RBC	Hb	PLT	Neut#	Lymph #	Neut#/ Lymph #
3	controls	52	1	10	14	9.8	38.7	86.8	6.44	5.02	164	302.00	4.190	1.76	2.3807
17	controls	38	1	26	25	4.4	45.1	79.6	6.16	4.66	145	202.00	4.270	1.52	2.8092
18	controls	46	1	54	33	6	45.1	78.7	6.39	5.42	163	222.00	3.410	2.55	1.3373
23	controls	58	2	11	19	5.5	44.5	71.8	6.25	4.06	130	212.00	3.410	2.38	1.4328
24	controls	46	2	13	21	6.3	41.4	64.4	4.18	4.64	145	154.00	2.320	1.59	1.4591
91	controls	36	1	37	30	3.6	50.1	98.9	5.87	5.30	162	213.00	3.200	2.28	1.4035
93	controls	52	1	16	16	4.9	47.4	72	12.35	4.97	158	374.00	7.140	3.70	1.9297
94	controls	56	1	18	23	4.8	40.3	82.5	5.98	4.37	142	198.00	3.550	1.95	1.8205
96	controls	43	1	52	30	5.6	55.2	80.4	8.07	5.17	152	204.00	4.850	2.74	1.7701
105	controls	47	2	14	15	5.9	36.8	67.3	7.24	4.51	133	129.00	4.170	2.56	1.6289
109	controls	51	2	12	19	3.5	35.9	59.1	3.22	3.81	123	222.00	1.710	1.30	1.3154
113	controls	46	2	10	13	2.1	36.3	61.8	6.68	4.33	134	273.00	3.830	2.39	1.6025
118	controls	58	2	14	18	13.9	47.3	70.8	5.94	4.35	126	259.00	3.680	1.90	1.9368
119	controls	54	1	17	19	3.4	38.8	81.5	8.16	4.92	143	205.00	5.650	2.06	2.7427
120	controls	42	2	28	21	6.1	44.2	68.6	8.37	5.39	96	287.00	5.200	2.66	1.9549
121	controls	56	2	11	12	7.7	41.1	64.9	5.45	4.35	130	150.00	3.040	2.06	1.4757
123	controls	55	1	46	29	3.5	41.6	78.7	6.60	5.00	164	151.00	4.340	1.86	2.3333
151	controls	39	1	30	28	8.5	43.8	93	6.48	4.84	155	217.00	4.300	1.69	2.5444
152	controls	43	1	25	23	2.9	40.3	92	5.19	4.33	147	180.00	3.640	1.11	3.2793
162	controls	38	1	18	19	4.7	44.5	76.2	5.80	5.24	145	317.00	3.710	1.73	2.1445
163	controls	38	1	25	19	7.1	43.6	81	6.48	5.12	159	262.00	3.890	2.13	1.8263
164	controls	58	1	15	19	4.7	41.4	77.2	4.35	4.33	143	202.00	2.130	1.87	1.139
170	controls	48	1	15	13	8	42	85.9	5.89	4.22	137	216.00	4.330	1.07	4.0467
173	controls	43	1	14	20	5.9	45.8	68.3	5.47	4.37	112	77.00	3.330	1.83	1.8197
174	controls	40	1	13	18	10.3	47.3	70.6	5.36	4.83	152	105.00	3.760	1.32	2.8485
175	controls	43	2	11	15	4.5	40.1	60.3	6.18	3.97	124	129.00	4.310	1.57	2.7452
179	controls	47	1	22	15	4.4	40.7	100.6	8.00	4.94	156	118.00	4.530	2.73	1.6593
186	controls	44	2	9	12	8.9	43.2	59.5	9.37	3.99	120	287.00	6.620	2.31	2.8658
187	controls	58	2	24	28	5.3	36.5	60.8	6.38	4.10	127	237.00	4.190	1.86	2.2527
188	controls	50	1	19	27	5.7	43.5	71	7.39	5.80	179	245.00	4.080	2.88	1.4167
189	controls	43	1	28	24	6.5	45.5	71.8	5.24	4.87	162	207.00	3.220	1.61	2
195	controls	51	2	10	18	3.3	38.5	70.7	7.93	5.01	125	248.00	4.090	3.46	1.1821
197	controls	43	1	19	20	3.4	41.9	73.4	6.57	4.63	143	206.00	3.260	2.61	1.249
198	controls	51	2	17	17	3.9	42.5	54.9	8.84	5.02	153	226.00	6.160	1.98	3.1111
199	controls	58	2	10	19	5.3	41.4	62.8	7.03	3.52	107	263.00	4.000	2.58	1.5504
201	controls	54	2	12	19	5	45.7	72.4	5.94	3.65	116	189.00	3.590	2.11	1.7014
202	controls	55	2	2	19	1.5	40	68.1	9.56	4.78	142	238.00	6.100	2.89	2.1107
204	controls	40	1	11	14	5.8	38.1	73.1	5.46	4.84	141	158.00	3.670	1.52	2.4145
205	controls	59	1	17	17	4.5	41.6	65.7	5.37	3.86	155	231.00	4.18	1.76	2.375
206	controls	53	1	12	19	5.3	33.8	81.2	4.87	4.64	134	216.00	2.920	1.61	1.8137
207	controls	53	1	33	25	4.4	39.3	86.8	8.57	4.79	146	278.00	5.410	2.22	2.4369
209	controls	47	2	14	17	3.8	41.9	56.9	4.49	4.50	137	294.00	2.570	1.67	1.5389
212	controls	38	1	24	17	2.5	41.5	73.9	14.69	5.21	158	144.00	10.830	3.23	3.3529
215	controls	54	2	9	16	0.5	35.8	80.4	5.67	4.03	123	153.00	2.870	2.48	1.1573
216	controls	50	2	6	10	6.4	33.9	62.7	4.17	3.21	71	228.00	2.850	1.18	2.4153
220	controls	53	1	29	25	10.1	41.3	85.4	5.12	5.44	162	210.00	2.880	1.72	1.6744
225	controls	39	1	17	14	15	42.1	84.5	6.77	4.90	152	335.00	4.900	1.39	3.5252
226	controls	41	1	15	19	12	36.4	76.1	3.36	4.86	144	118.00	1.950	1.08	1.8056
230	controls	40	1	28	20	5.3	41.1	82.4	7.26	4.86	147	51.00	3.480	3.29	1.0578
231	controls	53	1	10	17	14.6	43	90.4	5.64	5.11	149	44.00	3.830	1.34	2.8582
233	controls	54	1	17	15	8.1	41.4	75.3	6.54	5.20	160	285.00	4.430	1.64	2.7012
238	controls	58	1	17	19	7.5	39.1	76.3	5.78	4.57	153	202.00	3.300	2.09	1.5789
245	controls	57	1	38	29	22	43.8	98.5	6.50	4.90	169	169.00	3.810	2.17	1.7558
246	controls	50	2	22	21	5.5	37.9	51.2	8.70	4.63	139	282.00	6.170	1.94	3.1804
253	controls	45	2	11	17	6.3	37.7	56.6	6.37	4.02	126	119.00	4.500	1.46	3.0822

258	controls	54	2	15	22	1.7	40.5	70.6	4.28	3.50	93	125.00	3.250	0.91	3.5714
264	controls	50	1	16	12	6.8	39.8	66	7.91	4.76	140	132.00	5.650	1.89	2.9894
270	controls	35	1	9	12	7.1	45.9	82.8	4.77	4.76	142	220.00	2.890	1.43	2.021
271	controls	38	1	36	25	-12.7	57.2	88.8	11.16	5.06	177	363.00	8.360	2.25	3.7156
276	controls	36	1	33	21	3.7	41.3	74.3	7.15	5.47	153	211.00	4.860	1.75	2.7771
278	controls	54	1	27	27	8.2	41.3	76.2	7.50	4.34	135	199.00	4.660	2.24	2.0804
279	controls	54	1	11	15	7.4	42.9	65.8	4.09	4.96	140	200.00	2.310	1.44	1.6042
280	controls	50	1	13	19	6.1	36.2	80.4	6.51	5.18	150	176.00	4.210	2.09	2.0144
281	controls	49	1	15	12	5.9	40.5	80.2	10.32	4.64	150	256.00	7.670	2.03	3.7783
291	controls	36	1	34	21	6.4	47.8	99.3	10.42	5.53	171	257.00	7.680	2.20	3.4909
292	controls	45	1	27	15	5.3	38.5	95.7	4.67	4.77	140	136.00	3.270	1.11	2.9459
293	controls	44	2	12	14	4.3	42.5	52.3	10.13	4.45	135	533.00	7.320	2.10	3.4857
294	controls	40	1	9	17	3.5	49	76.7	9.70	5.58	165	312.00	5.440	3.83	1.4204
298	controls	35	1	8	14	7.3	41.3	72.4	8.05	5.26	164	252.00	5.630	1.70	3.3118
300	controls	58	1	21	17	10.3	42	65.8	3.99	4.87	150	199.00	2.570	1.13	2.2743
305	controls	48	1	21	18	3.9	41.7	84.7	5.93	5.36	165	114.00	3.930	1.59	2.4717
317	controls	46	2	11	14	4.2	44.9	56.9	6.50	3.97	125	135.00	3.860	2.29	1.6856
319	controls	47	1	11	14	3.2	39.7	88.9	5.90	5.19	146	224.00	3.240	2.17	1.4931
324	controls	43	2	15	16	8.9	41.6	53.2	5.90	4.19	127	291.00	3.700	1.82	2.033
326	controls	55	1	19	22	4.4	47	60.8	6.68	4.86	143	285.00	4.420	1.66	2.6627
327	controls	53	1	29	31	17.6	44	76.7	7.29	5.08	159	177.00	4.550	2.09	2.177
328	controls	50	1	24	19	7.8	44.5	61.2	5.18	5.35	158	231.00	3.370	1.39	2.4245
329	controls	47	1	10	15	6.2	40.7	76.3	5.00	4.83	145	147.00	2.690	1.79	1.5028
331	controls	46	1	12	18	9.2	43.7	72.9	4.99	4.64	141	212.00	3.220	1.11	2.9009
343	controls	52	1	31	21	6.6	43.3	72.2	7.71	5.03	156	252.00	5.280	1.74	3.0345
344	controls	50	2	12	13	7.4	42.3	61.4	7.75	4.55	142	293.00	5.680	1.41	4.0284
345	controls	53	1	35	35	7.6	44.6	76.6	6.86	4.64	135	188.00	4.57	1.59	2.8742
356	controls	41	1	15	17	7.9	42.3	89.9	6.77	4.89	162	205.00	3.900	2.21	1.7647
361	controls	58	1	18	16	1.9	44.8	94.7	6.60	4.54	149	191.00	3.53	1.11	3.1802
367	controls	55	2	10	22	6.1	41.7	52.7	4.78	4.27	129	267.00	2.610	1.67	1.5629
368	controls	56	2	16	17	7.1	46.1	58.3	4.18	4.76	147	251.00	2.440	1.36	1.7941
372	controls	49	1	44	32	4.9	43.8	92.9	8.05	4.90	158	249.00	4.470	2.78	1.6079
655	controls	39	1	9	21	13.2	48.8	66.9	5.36	5.02	152	126	3.2	1.67	1.9162
733	controls	39	1	13	6	5	47.3	82.9	7.44	4.51	142	384	4.7	2.07	2.2705
755	controls	35	1	13	21	28.4	50.9	91.7	7	4.68	152	210	4.4	1.92	2.2917
801	controls	55	2	15	20	4.1	47.7	66.4	2.21	3.44	91	221	0.73	0.97	0.7526
802	controls	46	2	22	21	13.9	44.6	60.7	5.73	4.05	120	205	3.15	2.07	1.5217
805	controls	42	2	11	11	7.7	45.1	63.8	8.70	4.75	147	246	6.19	1.92	3.224
806	controls	46	2	11	19	4.4	45.9	66.4	5.09	4.48	119	363	2.62	1.90	1.3789
807	controls	41	2	34	23	10.0	47.8	72.0	7.86	4.73	138	313	5.40	1.86	2.9032
809	controls	52	2	13	20	4.7	47.9	73.7	7.15	4.14	98	378	4.27	2.16	1.9769
810	controls	58	2	17	22	6.5	46.1	75.5	3.96	4.30	128	202	1.68	1.67	1.006
811	controls	46	1	24	25	6	46.8	92.3	6.35	5.16	162	210	3.76	1.97	1.9086
812	controls	42	2	12	19	7.7	46.1	54.2	7.34	4.66	134	247	4.67	1.91	2.445
813	controls	57	2	27	34	7.7	47.7	65.5	5.54	4.50	138	281	2.58	2.31	1.1169
814	controls	39	1	11	15	6.7	45.1	98.9	6.33	5.22	151	154	3.09	2.39	1.2929
819	controls	52	2	25	24	7.2	46.4	64.6	3.76	4.90	140	136	1.89	1.23	1.5366
820	controls	35	1	9	14	13.4	47.5	98.1	6.30	5.67	161	210	2.98	2.62	1.1374
823	controls	49	2	16	20	5.1	44.9	71.9	7.40	4.52	137	300	3.59	3.07	1.1694
825	controls	41	2	12	21	7.1	48.0	52.3	4.26	4.17	124	188	1.89	1.78	1.0618
827	controls	52	2	19	24	8.0	45.3	70.2	3.97	4.41	112	202	2.34	1.01	2.3168
832	controls	46	2	28	16	5.1	48.8	86.7	14.86	4.58	132	367	12.94	1.17	11.06
833	controls	48	2	18	18	4.5	45	83.3	6.44	4.44	133	225	3.92	1.87	2.0963
838	controls	51	2	11	18	9.2	45.0	73.5	5.03	3.82	131	326	2.57	1.84	1.3967
843	controls	45	2	11	11	19.0	50.9	62.5	6.27	4.53	140	222	3.84	1.69	2.2722
847	controls	41	2	18	23	9.1	43.1	58	5.55	3.54	122	156	3.30	1.65	2
851	controls	53	1	27	28	9.5	47.0	76.1	4.77	4.95	148	181	2.53	1.54	1.6429
854	controls	47	2	12	13	7.8	48.3	66.7	5.62	4.30	129	227	3.58	1.57	2.2803

855	controls	45	2	10	17	5.7	44.7	72.3	6.52	4.00	127	176	3.81	1.91	1.9948
859	controls	41	2	8	12	5.5	44.1	65.5	5.45	4.40	137	246	2.84	1.98	1.4343
862	controls	41	2	19	18	4.2	44.2	56.1	6.15	3.95	122	200	3.45	2.16	1.5972
867	controls	43	2	17	17	19.2	47.2	66.2	6.24	4.19	136	230	3.82	1.92	1.9896
868	controls	52	2	16	21	5.9	45.9	55.1	7.55	4.61	138	266	3.91	2.64	1.4811
872	controls	50	1	21	22	11.7	43.1	82.2	5.40	5.12	158	210	2.30	2.05	1.122
878	controls	42	2	13	14	5.5	44.0	59.2	6.36	3.86	112	254	3.15	2.50	1.26
880	controls	42	2	16	23	5.7	45.2	61.1	7.43	4.18	134	209	4.28	2.11	2.0284
886	controls	43	1	37	28	6.5	45.1	80.7	7.00	4.98	149	238	3.50	2.67	1.3109
887	controls	43	2	21	18	7.7	46.5	79.0	7.92	4.56	131	324	3.92	3.37	1.1632
893	controls	40	1	15	12	7.5	46.0	85.9	6.64	5.20	153	301	3.07	2.67	1.1498
895	controls	36	1	21	19	19.4	48.1	67.5	3.69	4.79	151	178	1.27	1.52	0.8355
899	controls	36	1	18	29	8	47.9	72.5	5.44	4.59	143	184	2.35	2.41	0.9751
900	controls	48	2	11	12	5.1	46.4	83.4	6.24	4.51	140	255	3.86	1.68	2.2976
901	controls	43	2	19	21	7.3	45.2	1.5	5.77	4.55	130	230	2.77	1.96	1.4133
902	controls	35	1	18	20	10.2	49.8	79.7	6.95	5.65	168	225	2.95	3.22	0.9161
6001	controls	36	1	23	17	12.3	44.3	83.9	5.36	4.43	134	252	3.03	1.83	1.6557
6007	controls	43	1	14	20	5.9	45.8	68.3	7.99	5.33	160	250	4.71	2.32	2.0302
6008	controls	36	1	58	33	10.8	44.6	72.2	4.54	4.57	133	225	2.62	1.53	1.7124

number	Group	Subgroup	nucleic tesing	ICU or not	age (year)	sex(1,1; 2,2)	nationality	height(cm)	weight (kg)	BMI (kg/m2)	Confirmed patient or Wuhan exposure	Smoking or not	comorbidities	diabetes	high blood pressure
GY-301	COVID-19	COVID-19-N	positive	n	50	2	Han	168	64	22.676	y	n	n	n	n
GY-302	COVID-19	COVID-19-N	positive	n	45	2	Han	160	73	28.516	y	n	n	n	n
GY-303	COVID-19	COVID-19-N	positive	n	56	2	Han	156	62	25.48	y	n	n	n	n
GY-304	COVID-19	COVID-19-N	positive	n	40	1	Han	168	89	26.48	n	n	n	n	n
GY-305	COVID-19	COVID-19-N	positive	n	39	1	Han	165	75	27.548	y	y	n	n	n
GY-308	COVID-19	COVID-19-N	positive	n	47	1	Han	176	72	23.244	y	n	n	n	n
GY-309	COVID-19	COVID-19-N	positive	n	39	1	Han	179	80	24.968	y	y	n	n	n
GY-310	COVID-19	COVID-19-N	positive	n	50	1	Han	173	80	26.73	y	n	n	n	n
GY-311	COVID-19	COVID-19-N	positive	n	51	2	Han	160	60	23.438	y	n	n	n	n
GY-313	COVID-19	COVID-19-N	positive	n	40	1	Han	170	61	21.107	y	n	n	n	n
GY-314	COVID-19	COVID-19-N	positive	n	41	2	Han	158	58	23.233	y	n	n	n	n
GY-315	COVID-19	COVID-19-N	positive	n	44	2	Han	162	62	23.624	y	n	n	n	n
GY-316	COVID-19	COVID-19-N	positive	n	49	2	Han	159	56	22.151	y	n	n	n	n
GY-317	COVID-19	COVID-19-N	positive	n	51	1	Han	172	85	28.732	y	n	n	n	n
GY-323	COVID-19	COVID-19-N	positive	n	38	1	Han	155	50	20.812	y	n	n	n	n
GY-326	COVID-19	COVID-19-N	positive	n	56	1	Han	170	60	20.761	y	n	n	n	n
GY-327	COVID-19	COVID-19-N	positive	n	62	1	Han	175	60	19.592	y	n	n	n	n
GY-330	COVID-19	COVID-19-N	positive	n	46	2	Han	162	56	21.338	y	n	n	n	n
GY-331	COVID-19	COVID-19-N	positive	n	55	2	Han	157	60	24.342	y	n	n	n	n
GY-332	COVID-19	COVID-19-N	positive	n	46	2	Han	155	60	24.974	y	n	n	n	n
GY-333	COVID-19	COVID-19-N	positive	n	53	1	Han	170	60	20.761	y	n	n	n	n
GY-335	COVID-19	COVID-19-N	positive	n	35	1	Han	170	74	25.606	y	y	n	n	n
GY-337	COVID-19	COVID-19-N	positive	n	48	2	Han	175	68	22.204	y	n	n	n	n
GY-339	COVID-19	COVID-19-N	positive	n	56	1	Han	170	85	29.412	y	y	n	n	n
GY-343	COVID-19	COVID-19-N	positive	n	25	1	Han	176	68	21.952	y	n	n	n	n
GY-344	COVID-19	COVID-19-N	positive	n	36	2	Han	160	53	20.703	n	n	n	n	n
GY-345	COVID-19	COVID-19-N	positive	n	71	1	Han	168	69	24.447	y	n	n	n	n
GY-346	COVID-19	COVID-19-N	positive	n	68	2	Han	155	56	23.309	n	n	n	n	n
GY-347	COVID-19	COVID-19-N	positive	n	48	1	Han	173	90	30.071	y	n	n	n	n
GY-348	COVID-19	COVID-19-N	positive	n	12	1	Han	165	52	19.1	n	n	n	n	n
GY-349	COVID-19	COVID-19-N	positive	n	30	1	Han	178	75	23.671	y	n	n	n	n
GY-350	COVID-19	COVID-19-N	positive	n	46	2	Han	161	54	20.833	y	n	n	n	n
GY-351	COVID-19	COVID-19-N	positive	n	58	2	Han	153	55	23.495	y	n	n	n	n
GY-352	COVID-19	COVID-19-N	positive	n	25	1	Han	170	65	22.491	n	n	n	n	n
GY-353	COVID-19	COVID-19-N	positive	n	46	1	Han	175	80	26.122	y	n	n	n	n
GY-354	COVID-19	COVID-19-N	positive	n	79	2	Han	150	65	28.889	n	n	n	n	n
GY-355	COVID-19	COVID-19-N	positive	n	22	1	Han	180	100	30.864	y	n	n	n	n
GY-357	COVID-19	COVID-19-N	positive	n	49	2	Han	165	50	18.365	y	n	n	n	n
GY-358	COVID-19	COVID-19-N	positive	n	56	2	Han	157	60	24.342	y	n	n	n	n
GY-359	COVID-19	COVID-19-N	positive	n	35	1	Han	176	74	23.889	y	n	n	n	n
GY-359-2	COVID-19	COVID-19-N	positive	n	55	1	Han	172	70	23.661	y	n	n	n	n
GY-360	COVID-19	COVID-19-N	positive	n	30	1	Han	172	85	28.732	y	n	n	n	n
GY-361	COVID-19	COVID-19-N	positive	n	57	1	Han	175	72	23.51	y	n	n	n	n
GY-362	COVID-19	COVID-19-N	positive	n	65	1	Han	170	68	23.529	n	n	n	n	n
GY-364	COVID-19	COVID-19-N	positive	n	53	2	Han	158	60	24.035	y	n	n	n	n
GY-365	COVID-19	COVID-19-N	positive	n	48	1	Han	168	67	23.739	y	y	n	n	n
GY-366	COVID-19	COVID-19-N	positive	n	17	1	Han	165	70	25.712	y	y	n	n	n
GY-367	COVID-19	COVID-19-N	positive	n	76	2	Han	154	60	25.299	y	n	n	n	n
GY-368	COVID-19	COVID-19-N	positive	n	75	1	Han	165	65	23.875	y	n	n	n	n
GY-369	COVID-19	COVID-19-N	positive	n	43	2	Han	153	54	23.068	n	n	n	n	n
GY-370	COVID-19	COVID-19-N	positive	n	24	2	Han	154	50	21.083	y	n	n	n	n
GY-371	COVID-19	COVID-19-N	positive	n	57	1	Han	172	80	27.04	y	n	n	n	n
GY-372	COVID-19	COVID-19-N	positive	n	68	1	Han	175	69	28.04	y	n	n	n	n
GY-373	COVID-19	COVID-19-N	positive	n	29	1	Han	178	75	29.04	y	y	n	n	n

GY-374	COVID-19	COVID-19-N	positive	n	14	1	Han	163	52	30.04	n	n	n	n	n
GY-375	COVID-19	COVID-19-N	positive	n	39	1	Han	160	80	31.04	y	n	n	n	n
GY-306	COVID-19	COVID-19-C	positive	n	46	1	Han	176	70	22.598	y	n	y	n	n
GY-307	COVID-19	COVID-19-C	positive	n	56	2	Han	160	60	23.438	y	n	y	y	n
GY-312	COVID-19	COVID-19-C	positive	n	55	2	Han	169	65	22.758	y	n	y	y	n
GY-320	COVID-19	COVID-19-C	positive	n	65	2	Han	155	68	28.304	N	n	y	n	y
GY-322	COVID-19	COVID-19-C	positive	n	47	1	Han	175	72	23.51	y	n	y	n	y
GY-324	COVID-19	COVID-19-C	positive	n	50	2	Han	159	59	23.338	n	n	y	y	n
GY-328	COVID-19	COVID-19-C	positive	n	61	1	Han	175	60	19.592	y	n	y	n	y
GY-329	COVID-19	COVID-19-C	positive	n	64	2	Han	155	70	29.136	y	n	y	n	n
GY-334	COVID-19	COVID-19-C	positive	n	62	1	Han	170	82	28.374	y	n	y	n	y
GY-336	COVID-19	COVID-19-C	positive	n	54	2	Han	148	44	20.088	y	n	y	n	y
GY-338	COVID-19	COVID-19-C	positive	n	54	1	Han	175	62	20.245	y	n	y	y	n
GY-340	COVID-19	COVID-19-C	positive	n	58	2	Han	165	55	20.202	y	n	y	n	n
GY-356	COVID-19	COVID-19-C	positive	n	46	1	Han	168	76	26.927	y	y	y	n	y
GY-363	COVID-19	COVID-19-C	positive	n	61	1	Han	164	57	21.193	n	y	y	y	n
GY-376	COVID-19	COVID-19-C	positive	n	40	1	Han	178	78	32.04	y	y	y	n	n

cardiovascular diseases	COPD	malignant tumor	chronic liver diseases	signs and symptoms	The highest temperature （°）	cough	muscle pain	fatigue	cough phlegm	headache	diarrhea	dyspnea	Time from illness to dyspnea (day)	time from illness to admission (day)	Total hospital stay (day)	systolic pressure (mmHg)	diastolic pressure(mmHg)	heart rate(Times/min)
n	n	n	n		37.5	n	n	n	n	n	n	n	n	7	16	130	84	94
n	n	n	n		38.2	n	n	n	n	n	n	n	n	9	17	120	86	81
n	n	n	n		36.8	y	y	y	n	y	n	n	n	7	25	124	74	82
n	n	n	n		39.2	y	n	y	y	n	n	y	15	14	30	120	87	93
n	n	n	n		38.8	n	n	n	n	n	n	n	n	5	17	125	78	86
n	n	n	n		37.9	n	n	n	n	n	n	n	n	5	18	109	76	78
n	n	n	n		36.8	n	n	n	n	n	n	n	n	6	14	106	86	74
n	n	n	n		37.7	n	n	n	n	n	n	n	n	7	21	109	87	79
n	n	n	n		38.1	y	n	y	n	y	n	n	n	4	24	124	82	100
n	n	n	n		38.8	n	n	n	n	n	n	n	n	15	21	98	65	69
n	n	n	n		37.5	n	n	n	n	n	n	n	n	4	17	97	69	77
n	n	n	n		37.6	n	n	n	n	n	n	n	n	15	27	100	78	75
n	n	n	n		38.1	n	n	n	n	n	n	n	n	9	15	98	67	78
n	n	n	n		38.5	n	n	n	n	n	n	n	n	4	19	119	78	96
n	n	n	n		38.7	n	n	n	n	n	n	n	n	8	14	118	89	87
n	n	n	n		38.5	n	n	n	n	n	n	n	n	11	16	161	102	90
n	n	n	n		38	n	n	n	n	y	n	n	n	3	13	129	80	80
n	n	n	n		37.2	y	n	n	n	n	n	n	n	3	27	150	89	90
n	n	n	n		37	y	n	n	n	n	n	n	n	20	15	107	70	80
n	n	n	n		36.5	n	n	n	n	n	n	n	n	1	16	118	65	80
n	n	n	n		38	y	n	n	n	n	n	n	n	1	22	148	95	90
n	n	n	n		37	y	n	n	n	n	n	n	n	1	11	120	80	84
n	n	n	n		37.2	y	n	n	n	y	n	n	n	1	11	119	87	80
n	n	n	n		38.3	n	n	y	n	n	n	y	15	13	22	142	91	113
n	n	n	n		38.0	y	n	n	n	n	n	n	n	3	14	105	62	102
n	n	n	n		37.2	n	n	n	n	n	n	n	n	3	16	110	68	79
n	n	n	n		38.2	y	n	n	y	n	n	n	n	5	18	133	77	95
n	n	n	n		39.2	y	n	n	y	n	n	n	n	4	18	147	70	88
n	n	n	n		38.0	y	n	n	y	n	n	n	n	5	16	113	70	84
n	n	n	n		37.2	y	n	n	n	n	n	n	n	2	14	100	60	88
n	n	n	n		38.0	n	n	n	n	n	n	n	n	3	12	121	84	95
n	n	n	n		37.4	y	n	n	n	n	n	n	n	2	11	110	70	76
n	n	n	n		38.6	y	n	n	n	n	n	n	n	4	12	130	90	80
n	n	n	n		38.0	n	n	n	n	n	n	n	n	3	12	120	80	80
n	n	n	n		38.5	n	n	n	n	n	n	n	n	4	16	147	86	95
n	n	n	n		36.9	n	n	y	n	n	n	n	n	10	10	143	79	83
n	n	n	n		37.7	n	n	n	n	n	n	n	n	4	14	108	78	80
n	n	n	n		38.2	y	n	n	n	n	n	n	n	10	13	125	90	99
n	n	n	n		37.5	y	n	n	n	n	n	n	n	10	17	122	76	79
n	n	n	n		39	y	y	y	n	n	n	n	n	4	23	125	82	76
n	n	n	n		38	y	n	y	n	n	n	n	n	15	20	130	70	69
n	n	n	n		38.2	y	n	y	n	n	n	n	n	1	12	135	89	80
n	n	n	n		38.5	n	n	y	n	y	n	n	n	14	15	114	76	80
n	n	n	n		38.2	y	n	n	y	n	n	n	n	7	21	136	92	76
n	n	n	n		38	y	n	n	n	n	n	n	n	4	29	126	82	72
n	n	n	n		37.4	n	n	y	n	n	n	n	n	1	6	123	91	102
n	n	n	n		36.5	n	n	n	n	n	n	n	n	1	9	125	75	68
n	n	n	n		36.6	n	n	n	n	n	n	n	n	1	12	126	68	71
n	n	n	n		36.8	n	n	n	n	n	n	n	n	2	7	126	69	69
n	n	n	n		37.6	n	n	n	n	n	n	n	n	3	13	100	64	98
n	n	n	n		36.2	n	n	n	n	n	n	n	n	1	7	144	88	83
n	n	n	n		36.5	n	n	n	y	n	n	n	n	1	6	136	76	76
n	n	n	n		38.5	y	n	n	n	n	n	n	n	2	15	128	80	90
n	n	n	n		38.1	y	n	n	y	n	n	n	n	7	7	116	69	79

n	n	n	n		37.2	n	n	n	n	n	y	n	n	2	8	110	70	76
n	n	n	n		37.4	y	n	n	n	n	n	n	n	8	15	130	80	78
n	n	n	y		39.3	n	n	n	n	n	n	n	n	2	20	109	86	76
n	n	n	n		38.1	n	n	n	n	n	n	n	n	4	18	128	87	75
n	n	n	n		39.5	n	n	n	n	n	n	n	n	10	25	109	87	75
n	n	n	n		38.1	n	n	n	y	n	n	n	n	2	17	161	92	98
n	n	n	n		37.3	y	n	y	n	n	n	n	n	13	12	160	110	120
n	n	n	n		38.2	n	n	n	n	n	n	n	n	5	14	129	99	90
n	n	n	n		37.1	y	n	n	n	n	n	n	n	3	17	145	108	80
y	y	n	n		37.4	y	n	n	n	n	n	y	1	1	18	130	80	80
y	n	n	n		39.3	n	n	n	n	n	n	n	n	3	21	153	87	90
n	n	n	n		37.2	y	n	n	n	y	n	n	n	1	12	163	94	110
y	n	n	n		37	y	n	n	n	n	n	n	n	7	11	142	104	80
y	n	n	n		36.8	n	n	n	n	n	n	n	n	14	7	120	96	100
n	n	n	n		36.6	y	n	n	n	n	n	n	n	6	19	162	90	79
n	n	n	n		39	n	y	y	n	n	n	n	n	5	11	135	85	86
n	n	n	y		38.9	n	n	n	n	n	n	n	n	6	13	128	86	90

respiratory rate (Times/min)	laboratory testing results	ALT (U/L)	AST (U/L)	Total bilirubin (mmol/L)	Albumin (g/L)	Ccreatinine (μmol/L)	White blood cell （10^9/L）	Red blood cell （10^9/L）	Hemoglobin(g/L)	Platelet （10^9/L）	Neutrophil （10^9/L）	Lymphocyte （10^9/L）	Neutrophil /Lymphocyte	Prothrombin time (s)	Activated partial thromboplastin time (s)	D-dimer (mg/L)	Potassium ion,(mmol/L)	Sodion (mmol/L)
24		12.5	21.8	6.6	37.6	46.7	3.21	4.51	129	128	1.51	1.42	1.0634	10.8	25.2	0.11	4	144.3
21		43.9	34.2	12.6	43.5	67.4	4.51	5	150	124	2.25	1.9	1.1842	10.6	23.8	0.15	3.46	139.6
20		22.6	0.89	9.4	44.1	53.6	3.5	4.21	137	183	1.49	1.73	0.8613	9.8	24.9	0.26	4.82	142.2
24		28.2	23.5	9.1	42.5	68.3	8.13	4.87	146	213	6.24	1.35	4.6222	363	21.5	0.31	5.75	146.2
18		9.2	15.3	14.5	43.5	57	3.06	4.75	139	291	1.48	1.48	1	14.3	25.2	0.03	3.97	109.5
15		44.6	27.5	18.4	42.2	63.3	3.13	4.56	137	118	2.08	0.91	2.2857	14.2	31.4	0.30	3.88	140.8
17		30.2	21.5	34.2	46.3	135.2	3.53	5.32	153	136	2.53	0.95	2.6632	13.5	21.5	0.07	3.51	146.2
19		74.4	35	11.2	45.8	128.8	6.43	6.46	184	147	3.6	2.35	1.5319	21.6	28.2	0.07	5.25	140.7
20		11	10.3	14.5	40.3	60.5	2.94	3.93	116	122	1.73	0.93	1.8602	11.3	28.6	0.16	4.02	141.5
16		20.5	35.3	11.4	35.3	74.2	3.82	5.32	159	209	2.98	0.72	4.1389	13.5	27.5	0.15	4.25	135.3
18		21.8	25.7	6.5	41.9	54.3	4.83	3.88	118	165	2.21	2.27	0.9736	10.2	24.7	0.07	3.78	143.1
17		20.2	25.3	9.45	44.2	56	2.42	3.13	120	89	1.78	0.56	3.1786	9.2	21	0.04	4.01	139.2
14		19.6	16.1	9.24	39.5	47.9	10.82	4.47	136	319	8.92	1.51	5.9073	9.2	23.2	0.27	5.56	140.8
19		41.2	24.3	19.34	47.2	67.5	6.07	5.59	172	150	4.42	1.27	3.4803	12.6	21.5	0.39	4.01	139.0
15		17.7	22.8	8.5	46.5	43	5.39	4.54	126	211	3.30	1.78	1.8539	9.25	28.8	0.45	4.49	138.3
20		34	18	26.3	38.1	65.3	11.8	4.94	144	374	10.25	1.06	9.6698	9.2	21.2	2.6	3.33	142.2
20		14	10	46.3	62.8	86.6	12.3	4.21	124	240	10.95	1.03	10.631	11	23.2	0.4	3.99	139.8
20		39	29	20.0	41.0	52.8	4.3	4.14	91	230	3.00	0.96	3.125	10	29.4	0.06	3.20	139.0
20		12	15	13.1	41.9	57.8	7.1	4.22	125	287	4.51	2.06	2.1893	n	n	0.6	3.56	139.7
20		38	31	19.8	38.4	49.7	5.7	3.80	114	210	3.53	1.75	2.0171	11	23.4	0.2	3.77	138.9
20		18	24	28.1	39.8	79.5	4.9	5.22	150	179	3.17	1.23	2.5772	12.3	24.1	0.1	3.16	138.0
20		15	11	22.0	40.5	72.1	8.1	5.11	150	330	4.54	2.79	1.6272	11.6	23.1	0.1	3.64	141.8
20		8	16	37.2	46.3	68.0	5.3	4.26	113	269	3.54	1.44	2.4583	18.2	25.2	0.4	2.97	141.2
21		50	20	10.2	32.8	67.6	7	3.88	117	267	4.92	1.51	3.2583	11.2	25.1	0.4	2.9	135
18		23	23	7.7	39.2	103.2	8.59	4.25	161	319	3.18	1.21	2.6281	14.9	27.6	0.32	3.84	138.1
20		7	14	6	45.4	53.7	2.64	4.23	108	128	4.08	1.49	2.7383	14.4	37.2	0.27	3.67	141.6
20		18	42	18.3	40	94.2	6.45	5.15	126	93	6.77	0.69	9.8116	132	27.3	0.26	3.52	146.1
22		21	37	5.7	41.1	59.9	4.98	4	126	170	7.54	2.18	3.4587	14.5	29.8	0.29	3.64	139.2
20		40	26	8.4	46.9	202.2	5.22	3.57	156	196	8.21	0.75	10.947	14.9	33	0.3	3.51	138
16		8	20	2.8	52.6	52.2	4.75	5.21	137	177	5.62	2.28	2.4649	14	38.3	0.31	3.55	142.8
20		8	23	8.1	42.6	76.9	3.1	5.23	144	114	6.38	1.65	3.8667	15.8	35.1	0.27	3.8	138.9
19		23	48	11.6	39	88.5	3.73	4.75	102	132	5.37	1.28	4.1953	16.9	38.7	0.36	3.98	140.9
20		12	27	2.8	40.2	71.1	4.6	4.21	105	140	8.52	1.09	7.8165	15.5	45.8	0.29	3.08	141.2
19		18	27	34.3	48.5	92.7	6	5.21	152	199	4.09	1.74	2.3506	15	34	0.29	3.73	140.1
21		25	25	7.7	42	78.2	7.79	5.25	160	294	5.87	1.32	4.447	13.2	28.6	0.33	4.03	137.1
22		21	37	5.7	14.1	59.9	4.98	4	126	170	5.73	2.03	2.8227	14.5	29.8	0.29		
18		74.4	79.3	8.6	41.6	94.5	4.57	5.11	153	259	2.72	1.42	1.9155	9.7	25.8	1.37	5.77	142.4
20		20	24	4.8	40.4	57	2.36	5.02	151	113	1.36	0.83	1.6386	12.7	33.9	0.35	3.6	139.4
20		12	23	5.8	34.7	55	7.23	4.98	129	325	5.72	1.09	5.2477	12.1	25.4	0.49	3.8	140.1
22		47	45	8.8	39.5	57	4.55	4.23	138	163	2.87	1.15	2.4957	11	25.6	0.38	3.2	138.9
18		30	31	7.2	39.9	83	5.34	3.56	139	173	3.78	1.08	3.5	12.5	23.6	0.28	3.4	139
19		35	28	6.5	45.6	77	4.15	4.23	164	176	3.13	0.71	4.4085	13	27.9	0.32	4.2	142.2
19		33	35	5.9	35.3	71	3.61	4.63	137	285	2.44	0.91	2.6813	12.5	31	0.24	3.7	138.5
19		34	23	5.2	40.2	69	4.2	5.23	128	164	5.1	2.25	2.2667	n	n	0.21	3.8	139.2
18		21	25.2	8.2	39.2	46	3.61	5.23	132	193	4.16	6	0.6933	13.2	22.5	0.25	6.2	139.2
24		44.3	29.2	14.07	42.4	93	4.78	4.43	141	133	4.23	1.81	2.337	13.5	27.4	0.2	3.9	138
18		15	16	10.9	50	74.9	4.31	5.24	155	209	2.14	1.72	1.2442	14.6	31.1	0.27	4.35	139.9
21		30	40	6.4	43.2	46.8	5.37	4.25	115	210	3.28	1.65	1.9879	12.7	23.4	0.75	4.25	142.9
21		33	26	8.4	49	62.5	3.84	5.21	165	187	6.19	1.69	3.6627	13.8	26.5	0.31	4.32	140.9
16		15	17	7.7	45.8	47.3	5.18	4.13	138	219	3.37	1.87	1.8021	14.6	30.1	0.39	4.41	138.5
20		13	18	19.6	48.3	42.1	5.98	4.37	135	162	2.79	2.83	0.9859	14.6	33.3	0.26	3.58	137
22		38	27	8.5	43.1	79.4	5.54	4.8	342	159	3.69	1.5	2.46	12.8	27.3	0.03	4.31	136.4
23		15	45	18.1	42	94.8	6.04	4.9	120	94	8.43	15	0.562	15.2	26.7	0.21	5.02	140.6
19		18	17	14	47.5	64.2	7.53	5.12	145	227	4.84	2.06	2.3495	14.2	35.2	0.27	4.27	140.3

20		17	26	6.4	46	48.4	3.1	5.23	125	98	5.74	0.93	6.172	13.2	33.6	0.36	4.45	138.1
20		40	27	9.4	43.8	78	7.19	4.32	135	61	3.84	2.65	1.4491	13.6	28.4	0.28	4.38	140.1
21		31.5	36.3	17.4	37.4	85.3	5.13	4.56	138	241	3.5	1.35	2.5926	17.3	31.3	0.22	4.75	136.3
19		27.4	21.5	7.4	41.3	67.3	3.53	4.21	136	146	3.09	0.45	6.8667	17.5	30.2	0.51	5.32	135.3
15		18.2	20.3	7.8	38.3	70.2	3.87	4.16	132	142	2.37	0.99	2.3939	10.5	29.3	0.45	5.23	139.4
17		20.3	30	15.2	41.3	48.5	5.13	4.42	135	287	4.13	1.09	3.789	10.56	18.2	0.71	4.2	145.2
24		111.9	33.2	12.8	42.3	54.9	8.18	4.89	142	410	6.08	1.71	3.5556	9.6	27.8	0.16	4.12	140.6
18		28.2	29.2	7.2	40.1	60.2	3.09	3.58	123	180	1.98	1.04	1.9038	11.3	25.2	0.72	4.3	140.2
20		10	20	53.8	41.4	77.3	8.3	5.08	160	138	5.79	1.94	2.9845	11.5	24.2	0.1	3.66	140.4
20		16	25	41.5	41.6	84.2	5.6	3.33	117	150	3.70	1.53	2.4183	13.2	25.2	2.1	3.69	140.7
20		56	71	12.0	38.7	76.7	6.9	5.11	150	116	6.03	0.71	8.493	11.9	25.3	0.6	3.87	138.5
20		10	15	5.0	36.1	118.7	6.2	3.65	106	308	5.12	0.63	8.127	n	n	1.9	6.14	131.4
20		13	9	20.2	42.2	57.0	6.9	5.10	154	210	4.48	1.76	2.5455	n	n	0.1	3.61	140.3
20		36.8	36.6	7.6	40.5	46.4	4.9	4.16	123	247	2.49	2.13	1.169	17.8	28.9	0.52	3.1	139
21		29	15	10.3	49	56.2	4.98	4.23	139	223	4.23	0.73	5.7945	13.5	36.2	0.35	3.73	142.3
18		20.6	23.7	18.13	33.8	60.5	4.79	4.79	141	119	3.38	0.89	3.7978	12.5	25.4	0.15	4.28	139.7
21		23	26	8.5	46	80.3	6.23	5.04	160	205	5.39	1.52	3.5461	13.2	28.6	0.33	4.03	137.1

Creatine kinase (U/L)	Lactic dehydrogenase (U/L)	hs-cTnI, pg/mL3	PCT (ng/mL)	CRP(ng/mL)	HS-CRP(ng/mL)	ESR(mm/h)
33.2	179.8	0.53	0.14	13.4	》10	44
104.4	266.9	0.52	0.2	≤10	《0.499	64
76.2	183.6	0.38	0.21	<10	0.91	20
46.1	275.4	0.26	0.14	15	>10	81
101.3	153.6	0.53	《0.1	《10	2.05	26
65.9	303.9	0.60	《0.1	22	》10	14
85.3	204.2	0.4	0.14	35.2	》10	10
163.5	249.4	1.26	0.19	10.3	》10	34
36.2	180.8	0.72	<0.1	>10	13.5	60
180.3	370.4	0.01	0.13	17.5	》10	7
46.5	178.2	0.51	《0.1	《10	《0.499	44
102.4	198.2	0.02	0.13	53.4	》10	99
32	221.2	0.01	0.13	10.1	》10	5
28.5	180.1	0.01	0.19	《10	6.43	47
51.8	234.8	0.68	0.11	《10	2.06	12
148	343	0.02	0.13	61.4	》10	118
23	112	0.2	0.2	22.3	》10	40
25	129	0.06	0.02	18.01	》10	73
32	163	0.01	0.19	5.3		115
37	182	0.67	0.16	6.8		34
7	225	0.68	0.29	24.5	》10	50
25	133	0.57	0.34	0.3		15
44	191	0.47	0.19	4.90		110
5	304	0.05	0.17	<10	2.57	130
101	196	0.31	0.07	35.3	》10	46
46	123	0.42	0.04	26.3	》10	45
125	363	0.67	0.05	28.1	＞3	37
61	249	0.45	0.051	20.67	> 3	39
45	215	0.64	0.34	16.65	> 3	67
66	186	0.34	0.04	<3	2.27	23
43	215	n	0.04	<3	0.63	8
50	219	0.02	0.04	<3	<0.5	14
158	296	0.05	0.132	117.77	> 3	32
61	172	0.13	0.04	<3	1.14	10
68	165	0.05	0.04	8.8	>3	11
61	249		0.051	20.67	> 3	
539.6	561.6	0.80	0.11	136.3	》10	87
42	179	0.01	0.13	<10	<0.499	13
25	236	0.02	0	38.08	>10	127
56	212	0.34	0	10.63	>10	2
388	215	0.25	0.09	<10	<0.5	6
83	204	0.27	0	<10	<0.499	16
38	205	0.31	0	15.4	>10	43
42	198	0.07	0.14	1.3	1.3	19
45	153	0.23	0.21	20.4	20.4	29
135.9	164.8	0.028	0.05	10.31	>10	8
98	140	0.25	0.04	<3	<0.5	7
86	192	0.025	0.04	<3	<0.5	20
59	151	0.03	0.06	<3	<0.5	4
72	141	0.036	0.04	<3	<0.5	2
59	110	0.043	0.04	<3	<0.5	9
56	127	0.06	0.12	<3	<0.5	6
124	408	0.36	0.05	27.8	＞3	14
74	137	0.025	0.18	<3	<0.5	4

129	156	0.64	0.20	3.54	> 3	12
101	152	0.025	0.03	<3	<0.5	5
41	245.2	0.41	0.24	23.5	》10	21
63	274.3	0.53	0.21	53.2	》10	87
103	180.3	0.45	0.14	26.8	》10	46
30.1	213	0.67	《0.1	《10	《0.499	53
138.7	240.9	0.64	<0.1	《10	《0.499	30
58.3	210.3	0.56	0.2	《10	3.45	60
23	148	0.23	0.14	37.3	》10	52
58	190	0.04	0.19	55.5	》10	135
436	402	0.52	0.29	67.2	》10	48
22	196	0.43	6.85	110.6	》10	128
65	141	0.48	0.16	10.6	》10	25
55.4	249.1	0.38	N	<10	1.31	44
46	159.3	0.53	0.12	<10	<0.499	13
204	210.9	0.31	0.05	57.06	>10	11
68	165	0.05	0.04	8.8	>3	11

Table S2. The detailed values of oral fungal diversity index and observed OTUs								
sample	reads	label	ace	chao	shannon	simpson	coverage	observed_otus
Control001	1153	0.97	131.010013	79.5	1.735031	0.277142	0.980919	41
Control003	1153	0.97	184.753702	161	2.2165	0.243723	0.965308	83
Control004	1153	0.97	112.75471	75.111111	0.92401	0.712194	0.980052	47
Control005	1153	0.97	172.752368	104.833333	2.763361	0.122912	0.974848	71
Control006	1153	0.97	207.482764	125.176471	2.837108	0.1431	0.96791	86
Control007	1153	0.97	159.998583	170.5625	2.488082	0.194923	0.959237	103
Control008	1153	0.97	50.154614	47	1.044832	0.432513	0.98699	26
Control009	1153	0.97	190.564008	155.3	1.669714	0.362974	0.962706	65
Control010	1153	0.97	149.833028	107.076923	3.419509	0.044583	0.975716	78
Control012	1153	0.97	221.587321	182	2.221924	0.25628	0.9549	104
Control013	1153	0.97	204.885863	204	3.471806	0.070866	0.950564	128
Control017	1153	0.97	184.421482	124.2	1.263353	0.423861	0.974848	43
Control020	1153	0.97	103.633969	61.75	0.686175	0.779814	0.982654	38
Control026	1153	0.97	70.445191	65	2.860279	0.093336	0.986123	55
Control027	1153	0.97	321.17037	263.285714	4.043447	0.046669	0.937554	172
Control028	1153	0.97	217.786981	181.8	3.560369	0.057248	0.957502	123
Control030	1153	0.97	157.936039	107.333333	2.439059	0.139005	0.972246	66
Control031	1153	0.97	178.321311	140.588235	3.195949	0.080477	0.966175	97
Control033	1153	0.97	167.608199	141.4	3.2994	0.078134	0.96791	97
Control034	1153	0.97	163.207027	126	2.363147	0.160919	0.978317	51
Control035	1153	0.97	238.270353	228.235294	2.940839	0.168055	0.949696	131
Control036	1153	0.97	93.063696	129	2.563596	0.114723	0.978317	54
Control037	1153	0.97	64.400756	48.5	0.924028	0.666393	0.988725	29
Control038	1153	0.97	148.203307	107.857143	2.908114	0.109127	0.978317	65
Control039	1153	0.97	143.305377	134.6	2.523956	0.227821	0.972246	85
Control040	1153	0.97	99.85328	97.5	1.830397	0.365731	0.975716	66
Control041	1153	0.97	153.325241	111.25	2.792065	0.1277	0.973981	75
Control042	1153	0.97	202.85314	145.533333	3.106704	0.103826	0.961839	114
Control043	1153	0.97	86.57942	70.055556	0.831446	0.743411	0.97745	52
Control044	1153	0.97	220.910695	211.1	3.189662	0.075144	0.959237	103
Control045	1153	0.97	281.202331	206.125	3.624345	0.050797	0.954033	120
Control046	1153	0.97	113.555998	83.333333	2.951689	0.081922	0.981787	60
Control047	1153	0.97	127.744742	101.5	2.592855	0.155074	0.97745	69
Control048	1153	0.97	199.163627	147	2.160326	0.287988	0.96791	73
Control049	1153	0.97	121.37047	117.588235	2.973174	0.125778	0.973981	92
Control050	1153	0.97	325.187044	204.55	2.151399	0.282268	0.946227	110
Control051	1153	0.97	105.870903	85.3	2.060045	0.305619	0.980052	60
Control052	1153	0.97	160.63847	98.75	2.170208	0.189695	0.973114	60
Control053	1153	0.97	92.741955	91	1.768397	0.469646	0.979185	68
Control054	1153	0.97	85.866259	88.25	2.278507	0.198219	0.984389	50
Control056	1153	0.97	147.43011	139	3.527746	0.038479	0.97745	74
Control058	1153	0.97	85.343391	75.2	1.939597	0.314037	0.985256	48
Control059	1153	0.97	137.93894	119	3.213933	0.08486	0.976583	80
Control060	1153	0.97	221.04182	123	2.419348	0.153488	0.972246	61
Control061	1153	0.97	130.092006	130.1875	2.88899	0.11213	0.969644	93
Control062	1153	0.97	175.155162	145.75	2.980989	0.119673	0.965308	97
Control064	1153	0.97	123.911427	84	2.136697	0.258971	0.981787	49
Control065	1153	0.97	190.931218	133.333333	2.581803	0.142789	0.970512	71
Control067	1153	0.97	105.361012	74.142857	1.761948	0.376695	0.982654	47
Control068	1153	0.97	110.034808	64.666667	1.688407	0.420914	0.985256	42
Control069	1153	0.97	269.619815	140.230769	2.762817	0.092304	0.963573	74
Control071	1153	0.97	151.536073	124.6	2.664242	0.145826	0.972246	75
Control074	1153	0.97	39.78205	33.5	1.957661	0.282042	0.994796	31
Control076	1153	0.97	38.119141	27.25	1.318114	0.460371	0.993929	22
Control077	1153	0.97	119.524048	113.647059	3.274678	0.088621	0.976583	93
Control079	1153	0.97	128.302613	104	2.398865	0.2107	0.976583	65

Control081	1153	0.97	102.914348	72.428571	2.002879	0.296512	0.983521	48
Control082	1153	0.97	182.964949	178.954545	3.173852	0.113916	0.955768	121
Control084	1153	0.97	102.864198	67.5	0.956117	0.518287	0.987858	22
Control085	1153	0.97	176.386576	193.333333	3.470649	0.065414	0.957502	128
Control086	1153	0.97	77.816096	52	1.901969	0.231501	0.98699	37
Control087	1153	0.97	117.526794	74.375	2.561017	0.162848	0.983521	53
Control088	1153	0.97	60.279176	90	1.204584	0.524132	0.986123	30
Control090	1153	0.97	237.966344	173.954545	3.367588	0.063164	0.955768	116
Control091	1153	0.97	123.231053	93.545455	2.76814	0.102536	0.97745	64
Control092	1153	0.97	94.5197	77	2.371521	0.132196	0.985256	43
Control093	1153	0.97	46.56	39.5	0.4091	0.836605	0.988725	20
Control095	1153	0.97	103.504932	75.153846	2.959546	0.09384	0.983521	62
Control096	1153	0.97	176.263358	167.3	3.366473	0.060231	0.967042	97
Control097	1153	0.97	366.915363	273.5	2.619882	0.14362	0.973981	56
Control098	1153	0.97	134.517991	125.3	2.391263	0.194082	0.96791	92
Control101	1153	0.97	158.373511	109.2	2.690435	0.107923	0.979185	54
Control103	1153	0.97	106.377712	95.166667	1.818235	0.305778	0.980052	53
Control104	1153	0.97	684.727835	402.44	2.970242	0.142436	0.905464	167
Control106	1153	0.97	90.405266	86.769231	2.602314	0.133294	0.980919	69
Control108	1153	0.97	247.956677	278	3.238722	0.079554	0.937554	136
Control110	1153	0.97	158.699696	117.714286	2.987141	0.087536	0.971379	80
Control111	1153	0.97	101.923117	63.1	2.381641	0.134453	0.983521	46
Control112	1153	0.97	86.648673	92.5	2.810307	0.083404	0.980919	54
Control115	1153	0.97	41.799974	38.5	1.396628	0.484477	0.991327	31
Control118	1153	0.97	171.253141	118.125	2.179337	0.215017	0.973114	60
Control119	1153	0.97	117.071945	87.5	1.61884	0.428962	0.979185	53
Control121	1153	0.97	146.751953	75.5	0.420232	0.863544	0.98699	23
Control123	1153	0.97	121.690304	93.428571	1.025085	0.62948	0.97745	47
Control124	1153	0.97	172.78161	112.142857	2.509085	0.172646	0.976583	62
Control126	1153	0.97	123.5717	117.176471	2.688722	0.148913	0.972246	88
Control130	1153	0.97	167.975931	89.5	2.275881	0.214051	0.97745	57
Control131	1153	0.97	115.376255	79	2.426952	0.142733	0.986123	39
COVID-19-002	1153	0.97	210.579785	100.25	1.174602	0.509464	0.980052	37
COVID-19-003	1153	0.97	406.024229	267.956522	2.920419	0.144388	0.931483	134
COVID-19-005	1153	0.97	427.722493	286.588235	3.142647	0.109417	0.936687	132
COVID-19-006	1153	0.97	363.430139	198.157895	2.366962	0.242983	0.947962	105
COVID-19-008	1153	0.97	309.861821	199.588235	3.119197	0.088067	0.951431	109
COVID-19-010	1153	0.97	282.604799	221.526316	2.681193	0.191136	0.946227	122
COVID-19-011	1153	0.97	272.530146	207.391304	3.221056	0.121585	0.948829	133
COVID-19-013	1153	0.97	392.709318	263	2.793213	0.172491	0.940156	125
COVID-19-014	1153	0.97	268.988431	287.3	2.972398	0.146601	0.933218	141
COVID-19-015	1153	0.97	135.991504	135.214286	2.849925	0.096355	0.967042	85
COVID-19-016	1153	0.97	322.080399	243.24	2.877085	0.177159	0.937554	141
COVID-19-017	1153	0.97	217.932528	129.052632	2.866067	0.11359	0.965308	88
COVID-19-018	1153	0.97	337.730045	214.8	2.697125	0.194517	0.942758	129
COVID-19-019	1153	0.97	216.809895	168.176471	2.703743	0.145943	0.957502	99
COVID-19-023	1153	0.97	248.906575	181	2.598357	0.216082	0.955768	106
COVID-19-024	1153	0.97	120.844798	122.153846	2.901635	0.119715	0.972246	84
COVID-19-025	1153	0.97	267.659365	176.913043	3.112135	0.10744	0.954033	117
COVID-19-026	1153	0.97	84.813265	85.6	1.418813	0.497418	0.979185	58
COVID-19-027	1153	0.97	218.79619	198.5625	3.396765	0.07823	0.956635	122
COVID-19-028	1153	0.97	76.489899	71.25	1.798035	0.352812	0.983521	57
COVID-19-029	1153	0.97	120.640759	111.875	2.290937	0.251667	0.976583	68
COVID-19-030	1153	0.97	173.478573	176.6	2.443056	0.231939	0.971379	71
COVID-19-031	1153	0.97	204.595751	152.416667	3.539952	0.054337	0.961839	113
COVID-19-032	1153	0.97	106.138122	124.428571	3.028249	0.098975	0.97745	78
COVID-19-035	1153	0.97	188.939955	142.05	3.076585	0.091707	0.963573	99
COVID-19-037	1153	0.97	150.965067	151.05	3.412512	0.071971	0.966175	114

COVID-19-038	1153	0.97	381.201514	259.05	2.509661	0.209565	0.93582	124
COVID-19-040	1153	0.97	341.659637	146.333333	1.452952	0.381972	0.97745	38
COVID-19-041	1153	0.97	129.429249	126	2.757546	0.095061	0.979185	57
COVID-19-043	1153	0.97	190.894725	195.714286	3.745357	0.045423	0.955768	135
COVID-19-045	1153	0.97	131.849662	144	2.13436	0.192037	0.969644	59
COVID-19-046	1153	0.97	338.245526	156.25	1.350061	0.511776	0.960104	70
COVID-19-047	1153	0.97	59.317063	53.666667	1.482381	0.442123	0.98699	42
COVID-19-048	1153	0.97	398.760582	275.375	2.687132	0.174462	0.928881	137
COVID-19-049	1153	0.97	27.375	20	0.312119	0.886642	0.993929	13
COVID-19-050	1153	0.97	224.265405	163.625	2.512878	0.136936	0.966175	71
COVID-19-051	1153	0.97	111.698199	111.230769	3.39937	0.068368	0.979185	90
COVID-19-052	1153	0.97	137.237802	112.5	1.53627	0.477149	0.976583	54
COVID-19-054	1153	0.97	30.576505	18	0.279089	0.902736	0.994796	13
COVID-19-055	1153	0.97	43.934165	41	1.068172	0.623208	0.99046	30
COVID-19-057	1153	0.97	69.477419	58.75	0.771909	0.717563	0.982654	35
COVID-19-058	1153	0.97	109.997138	52	0.96995	0.448843	0.989592	19
COVID-19-061	1153	0.97	218.392762	214.6	2.930035	0.0901	0.967042	74
COVID-19-062	1153	0.97	105.568864	106.6	3.318555	0.062498	0.979185	79
COVID-19-063	1153	0.97	93.6305	80.428571	2.45701	0.183663	0.983521	56
COVID-19-064	1153	0.97	153.986734	143.434783	2.683671	0.179264	0.963573	106
COVID-19-065	1153	0.97	65.086056	40	1.570759	0.351271	0.991327	25
COVID-19-069	1153	0.97	87.37111	75.666667	2.650988	0.146252	0.985256	53

Table S3. The abundance and composition at the phylum level of each sample												
phylum	Control001	Control003	Control004	Control005	Control006	Control007	Control008	Control009	Control010	Control012	Control013	Control017
Ascomycota	774573	929480	42829	709862	863089	898554	531943	984265	734248	924193	813393	984473
Basidiomycota	173163	38765	5085	218481	116921	34157	5508	9940	233452	29195	151193	12588
Chytridiomycota	0	0	0	0	0	0	0	0	0	0	0	0
Fungi_phy_Incertae_sedis	11596	30935	47086	33272	16099	50209	461225	4525	30663	35241	31844	2572
Mucoromycota	40067	820	905000	38344	3813	16178	1324	1270	1637	11034	214	336
Rozellomycota	0	0	0	0	0	0	0	0	0	337	0	0
Zoopagomycota	601	0	0	41	78	902	0	0	0	0	3356	31

Table S3 .The abundance and composition at the genus level of each sample												
genus	Control001	Control003	Control004	Control005	Control006	Control007	Control008	Control009	Control010	Control012	Control013	Control017
Acremonium	0	0	0	0	0	0	0	80	0	0	0	0
Acrocalymma	0	46	0	0	0	43	49	0	54	0	65	0
Acrodictys	744214	812817	11466	559057	205691	480837	6191	275297	183709	533166	458328	205881
Acrodontium	0	0	0	0	0	0	0	0	0	0	0	0
Actinomucor	0	0	0	0	0	0	0	0	0	0	0	0
Agaricales_gen_Incertae_sedis	0	0	0	0	0	0	0	0	0	0	0	0
Agaricus	0	0	0	0	0	0	0	0	0	0	0	0
Ahmadea	0	0	0	0	0	0	0	0	0	0	0	202
Allocryptovalsa	0	0	0	0	0	0	0	0	0	0	0	0
Alternaria	205	6116	260	164	77	164	93	264	31354	1076	6280	671
Amphobotrys	0	0	0	0	0	0	0	122	0	0	3346	0
Amylostereum	0	0	0	0	0	0	0	0	0	0	0	0
Anastomitrabeculia	0	0	0	0	0	0	0	0	0	0	0	0
Angustimassarina	621	3940	0	84	144	2877	0	634	848	10696	3441	36
Anisomeridium	0	43	0	0	70	167	0	76	0	0	125	0
Antennariella	0	0	0	0	0	0	0	0	0	0	13314	35
Anthracina	0	2359	0	0	0	0	0	0	0	0	0	0
Apiotrichum	0	0	0	0	0	1355	0	80	77	0	72	144
Aquapteridospora	0	0	0	0	0	0	0	0	0	0	0	0
Archaeorhizomyces	0	0	0	0	0	0	0	0	0	0	0	0
Arthrographis	2138	1560	1863	13627	2563	3298	1103	672077	2421	9399	1064	747216
Ascochyta	0	0	54	0	0	0	2263	0	0	0	0	0
Ascomycota_gen_Incertae_sedis	0	0	0	0	0	337	0	0	63	0	0	0
Ascosphaera	0	0	0	0	0	0	0	0	0	0	0	0
Ascotricha	0	0	0	0	0	0	0	0	0	0	51	90
Aspergillus	2232	1063	1961	3826	12269	17817	1646	1369	122933	9440	5230	2254
Atractiella	0	0	0	0	0	0	0	0	0	0	0	0
Aureobasidium	0	0	0	0	0	0	0	0	0	0	0	205
Auricularia	0	0	0	0	0	0	0	64	0	0	179	185
Austropleospora	0	0	0	0	0	0	0	0	0	0	0	0
Avachytrium	0	0	0	0	0	0	0	0	0	0	0	0
Bandonia	0	0	0	0	0	0	0	0	0	0	0	0
Banksiophoma	0	0	0	0	0	0	0	0	0	0	0	0
Basidiomycota_gen_Incertae_sedis	0	0	0	0	0	0	0	0	0	0	0	0
Beauveria	0	0	0	0	0	0	0	160	0	0	0	0
Bergerella	0	0	0	0	0	0	0	0	0	0	175	0
Biatriosporaceae_gen_Incertae_sedis	0	0	0	0	0	0	0	0	0	0	0	0
Bjerkandera	151	0	290	256	322	1706	105	138	183	2124	0	37
Blastobotrys	0	0	0	0	0	0	0	0	0	0	0	0
Blumeria	1609	501	1109	1622	1319	2447	873	1447	1855	6484	290	190
Botryosphaeria	0	0	0	48	0	78	100	86	143	0	0	0
Botrytis	0	34	0	0	0	0	0	0	0	0	69	0
Bovista	0	0	0	0	0	0	0	0	0	0	0	0
Buckleyzyma	0	0	0	0	0	0	0	0	0	0	0	0
Bulleromyces	0	0	0	0	0	0	0	0	0	0	0	0
Byssochlamys	0	0	0	0	0	0	0	0	0	0	0	0
Calosphaeria	0	424	38	0	0	1830	0	131	253	722	304	34

Calycina	0	0	0	0	0	0	0	0	0	0	0	0
Calyptella	0	0	0	0	0	0	0	0	0	0	0	32
Candida	799	0	598	214	0	6091	1176	634	0	0	630	676
Capnodiales_gen_Incertae_sedis	641	0	0	0	0	0	0	0	0	0	0	0
Carlosrosaea	0	0	0	0	0	0	0	0	0	0	0	0
Ceraceosorus	0	0	0	0	0	0	0	0	0	0	0	0
Ceratocystis	0	0	0	0	0	0	0	0	0	0	0	0
Cercospora	0	79	49	0	0	45	0	70	74	0	0	0
Ceriporia	0	0	41	41	0	49	0	52	127	0	0	0
Ceriporiopsis	0	0	0	0	0	0	0	0	0	0	0	0
Cerrena	0	0	0	0	0	0	0	69	0	0	0	0
Chaenothecopsis	0	0	0	0	0	0	0	0	0	0	0	0
Chaetomium	0	0	0	0	0	0	0	0	0	0	0	0
Chalara	0	0	0	0	0	0	0	0	0	0	0	0
Choanephora	0	0	0	0	0	0	0	0	0	0	0	0
Chrysosphaeria	132	46	46	42	296	9099	0	0	139	367	0	127
Chytridiomycota_gen_Incertae_sedis	0	0	0	0	0	0	0	0	0	0	0	0
Ciliophora	0	0	0	10477	69	38	48	0	0	0	0	0
Cladophialophora	0	0	0	0	0	0	0	0	0	0	0	0
Cladosporium	1071	448	454	34486	48980	5151	2054	1718	453	4973	36942	3390
Clavispora	0	0	0	0	0	0	0	0	0	0	0	0
Clonostachys	0	0	0	0	0	0	0	0	0	0	0	0
Colletotrichum	0	0	50	0	0	937	0	0	0	0	0	0
Collybiopsis	0	0	0	0	0	0	0	0	0	0	0	0
Coniochaeta	0	0	0	0	0	0	0	0	0	0	0	0
Coniosporium	0	0	0	0	0	0	0	0	0	0	0	0
Coniothyrium	0	0	0	0	0	0	0	0	0	0	0	0
Cookeina	0	0	0	0	0	0	0	0	0	0	0	0
Coprinellus	77	69	58	0	165	174	0	130	52421	1058	198	186
Coprinopsis	78	47	44	170	0	0	94	77	136	346	0	0
Cordyceps	0	0	0	0	0	0	0	0	0	0	0	0
Coronicium	0	0	0	0	0	0	0	0	0	0	0	0
Crustoderma	0	0	0	0	0	0	0	0	0	0	0	0
Cryptococcus	1739	23993	1422	42617	1143	1569	971	1610	22608	7408	1781	3881
Cryptomarasmius	0	0	0	0	0	0	0	0	0	0	0	0
Curvularia	0	0	0	0	0	0	0	0	0	0	0	0
Cutaneotrichosporon	141	37	141	96	0	47	0	0	32393	1399	0	0
Cyphellaceae_gen_Incertae_sedis	0	0	0	0	0	0	0	0	0	0	0	0
Cyphellophora	0	0	0	0	0	0	0	0	0	0	0	0
Cystofilobasidium	0	0	0	0	0	0	0	0	63	354	118	382
Cytospora	0	0	0	0	0	0	0	0	0	0	11909	0
Dactylella	0	0	0	48	8548	0	0	0	0	0	0	0
Dactylodendron	0	0	0	0	0	0	0	0	0	0	0	0
Debaryomyces	0	0	0	0	0	0	0	0	0	0	119	97
Deconica	0	0	0	0	0	0	0	0	0	0	67	35
Delastria	0	0	0	0	0	0	0	0	0	0	0	0
Dendryphiella	0	0	0	0	0	0	0	0	0	0	0	0
Derxomyces	0	0	0	0	0	0	0	0	0	0	0	0
Diaporthe	0	0	2356	0	0	260	0	0	0	0	118	469
Didymella	66	68	230	0	0	0	0	56	27160	711	68	0
Didymocyrtis	0	0	0	0	0	0	0	0	0	0	0	0
Dimorphoma	0	0	0	0	0	0	0	0	0	0	0	0
Dioszegia	0	0	0	0	0	0	0	0	0	0	61	196
Dipodascus	0	0	0	0	0	0	0	0	0	0	0	0
Diutina	0	0	0	0	0	0	0	979	0	0	0	0
Diversispora	40067	820	905000	38344	3813	16178	1324	1270	1637	11034	171	299
Dothidea	0	0	0	0	11718	0	0	0	0	0	0	0
Dothiora	0	0	0	0	0	0	0	0	0	0	0	0

Echinoporia	0	0	0	0	0	0	0	135	0	0	0	0
Edenia	0	0	0	0	0	0	0	0	0	0	0	0
Effuseotrichosporon	0	0	0	0	0	0	0	0	0	0	0	0
Elaphomyces	0	0	0	0	0	0	0	0	0	0	2466	0
Elsinoe	0	0	0	0	0	0	0	0	0	0	0	0
Engyodontium	0	42	0	0	0	0	0	0	0	0	0	65
Entoloma	0	0	0	0	0	0	0	0	0	0	0	0
Entorrhiza	0	0	0	0	0	0	0	147	0	0	50	0
Entyloma	0	0	0	0	0	0	0	0	0	0	0	0
Epicoccum	69	44	117	84	94	115	518	62	28339	692	0	0
Eremothecium	0	0	0	0	0	0	0	0	0	0	0	0
Erythrobasidium	0	0	0	0	0	0	0	0	0	0	53	47
Eupenidiella	0	0	0	0	0	0	0	0	0	0	0	0
Eutypella	0	0	0	0	0	0	0	0	0	0	68	37
Extremus	0	0	0	0	0	0	0	0	0	0	0	0
Fantasmomyces	0	2433	43	37	159	2685	80	1275	830	727	753	310
Filobasidiaceae_gen_Incertae_sedis	0	2144	0	0	474	1002	0	0	450	0	846	0
Filobasidium	0	39	0	0	0	0	0	0	0	0	119	343
Flagelloscypha	0	0	0	0	0	0	0	0	0	0	0	0
Flammulina	0	0	0	0	0	0	0	0	0	0	68	95
Flavodon	0	0	0	0	0	0	0	0	0	0	0	0
Fomitopsis	0	0	0	0	0	0	0	0	0	0	0	0
Fungi_gen_Incertae_sedis	11596	30935	47086	33272	16099	50209	461225	4378	30663	35241	31794	2572
Fusarium	302	34	0	222	76	2692	243	343	180	716	0	33
Fuscosporella	161	39	0	0	0	0	0	0	0	0	119	0
Fusicolla	0	0	0	0	0	79	0	0	0	0	0	0
Galactomyces	0	0	0	0	0	0	0	0	0	0	0	0
Ganoderma	0	0	0	0	0	0	0	0	0	0	0	0
Genea	0	0	8482	0	0	125	0	0	72	0	0	0
Gibberella	0	0	46	0	0	0	0	0	0	0	119	0
Gibellula	0	265	0	0	62	556	753	0	0	0	1785	0
Gjaerumia	0	0	0	0	0	0	0	0	0	0	0	0
Glomus	0	0	0	0	0	0	0	0	0	0	43	37
Golovinomyces	0	0	0	0	0	0	0	0	0	0	0	0
Golubevia	0	0	0	0	0	0	0	0	0	0	0	0
Gonatophragmium	0	0	0	0	0	0	0	0	0	0	0	0
Gymnopus	0	0	0	0	0	0	0	0	0	0	0	0
Halosphaeriaceae_gen_Incertae_sedis	0	0	0	0	0	0	0	0	0	0	0	0
Hannaella	0	0	0	0	0	0	0	0	0	0	0	77
Hanseniaspora	0	0	0	0	0	0	0	0	0	0	0	0
Hazslinszkyomyces	0	0	0	0	0	0	0	0	0	0	0	0
Heitmania	0	249	0	0	694	2995	1692	2621	255	328	268	62
Helicoma	0	0	0	0	0	0	0	0	0	0	0	0
Heliocybe	0	0	0	43	0	0	41	0	0	0	0	0
Henningsia	0	0	0	0	0	0	0	0	0	0	0	0
Heydenia	0	0	0	0	0	0	0	0	0	0	0	0
Holtermanniella	0	0	0	0	0	0	0	0	0	0	0	0
Hormodochis	0	0	0	0	0	0	0	0	0	0	0	0
Hortaea	0	0	0	0	0	0	0	0	0	0	0	0
Houjia	0	0	0	0	0	0	0	0	0	0	0	0
Huntiella	0	0	0	0	0	0	0	0	0	0	0	0
Hydnobolites	64	67	96	45	77	137	0	221	0	681	49	0
Hyphopichia	0	0	0	0	0	0	0	0	0	0	0	0
Hypoxylon	0	44	135	0	73	84	45	157	69	725	0	0
Irpex	0	0	0	0	0	0	0	0	0	0	0	0
Issatchenkia	0	0	0	0	0	0	0	0	0	0	0	0
Itersonilia	0	0	0	0	0	0	0	0	0	0	0	0
Jaminaea	0	0	0	0	0	0	0	0	0	0	0	0

Kazachstania	131	155	91	88	154	129	182	362	57	1410	0	609
Keissleriella	74	47	93	85	80	41	43	214	63	364	0	0
Keratinophyton	0	0	0	0	0	0	0	0	0	0	0	0
Kneiffiella	0	0	0	0	0	0	0	0	0	0	0	0
Knufia	0	0	0	0	0	720	0	0	0	0	68	108
Kodamaea	0	0	0	0	0	0	0	0	0	0	0	0
Kurtzmaniella	0	0	0	0	0	0	0	0	0	0	0	0
Laburnicola	0	0	0	0	0	0	43	57	0	0	0	0
Laetisaria	0	0	0	0	0	28	0	0	0	0	0	0
Lecanicillium	0	0	0	0	0	0	0	0	0	0	0	0
Lentinula	0	4764	0	0	0	0	0	0	0	0	61	26
Lentinus	0	0	0	0	0	0	0	0	0	0	73	123
Lentithecium	0	0	0	0	0	0	0	0	0	0	0	0
Leptospora	0	0	0	0	0	0	0	0	0	0	0	0
Leucosporidium	0	0	0	0	0	44	0	0	0	0	0	0
Lipomyces	0	0	0	0	0	0	0	0	0	0	0	0
Lithohypha	0	0	0	0	0	0	0	0	0	0	0	0
Lophiostoma	0	0	0	0	0	0	0	0	0	0	64	0
Lyomyces	0	0	0	0	0	0	0	0	0	0	0	0
Macrohyporia	0	36	50	49	73	0	0	0	146	341	0	0
Malassezia	1416	6963	1820	79025	80527	8521	1936	2808	123749	9919	63306	1812
Marantokordyana	0	0	0	0	0	0	0	0	0	0	0	0
Meira	0	0	0	0	0	0	0	0	0	0	0	0
Meyerozyma	0	0	0	0	0	0	0	0	0	0	0	0
Microascus	0	0	0	0	0	0	0	0	0	0	0	0
Milospium	114	0	102	104	45889	175	49	75	39675	0	1809	2166
Moesziomyces	0	0	0	0	0	0	0	0	0	0	0	0
Moniliella	0	0	43	0	81	44	0	0	70	318	0	0
Mrakia	0	0	0	0	0	0	0	0	0	0	0	0
Mucor	0	0	0	0	0	0	0	0	0	0	0	0
Mycoarthris	0	0	0	0	0	0	0	0	0	0	0	0
Mycosphaerellaceae_gen_Incertae_sedis	0	0	0	0	0	40	43	0	0	0	54	34
Naganishia	0	0	0	0	0	0	0	65	0	0	462	685
Nakaseomyces	0	0	0	0	0	0	0	0	0	0	0	0
Nannizziopsis	0	0	0	51	0	0	0	127	0	0	0	0
Nectriopsis	0	0	0	0	0	0	0	0	0	0	0	0
Nemania	0	0	0	0	0	0	0	0	0	0	0	0
Neoascochyta	0	0	0	0	0	0	0	0	0	0	0	0
Neoconiothyrium	0	0	0	0	0	0	0	0	0	0	0	0
Neocucurbitaria	0	0	0	0	0	0	0	0	0	0	0	0
Neodevriesia	0	0	0	0	0	0	0	0	0	0	0	0
Neodevriesiaceae_gen_Incertae_sedis	0	0	0	0	0	0	0	0	0	0	0	0
Neoerysiphe	0	0	0	0	0	0	0	0	0	0	0	42
Neofusicoccum	0	0	0	0	0	0	0	0	0	0	0	69
Neosetophoma	0	0	0	0	0	0	903	0	0	0	64	0
Nigrograna	0	46	0	0	0	0	0	0	63	367	0	0
Nigrospora	0	0	0	0	0	0	0	0	0	0	0	0
Nothophoma	0	0	0	0	66	3267	93	0	392	704	0	0
Novakomyces	0	0	0	0	0	0	0	0	0	0	0	0
Ochroconis	0	0	0	0	0	0	0	0	0	0	0	0
Ogataea	0	0	0	0	0	0	0	0	0	0	0	0
Oliveonia	0	0	0	0	0	0	0	0	0	0	0	0
Ophiosphaerella	0	0	0	0	0	68	0	0	0	0	0	0
Ophiostoma	71	86	96	0	2244	42	0	0	60	0	3228	104
Papiliotrema	0	0	0	0	0	0	0	0	0	0	0	0
Paracremonium	0	0	0	0	0	0	0	0	0	0	0	0
Parafuscosporella	72	7663	154	47	1076	5818	127	8440	1109	8914	6013	8947
Paraleptosphaeria	0	0	0	0	0	0	0	0	0	0	0	0

Pararamichloridium	0	0	0	0	0	0	0	0	0	0	0	0
Parasarocladium	0	0	0	0	0	0	0	0	0	0	0	0
Parastagonosporella	125	74	0	0	85	87	0	79	76	726	0	0
Parasympodiella	0	0	0	0	0	0	0	0	0	0	0	0
Parathyridaria	0	0	0	0	0	0	0	0	0	0	0	0
Paratritirachium	0	0	0	0	0	0	0	0	0	0	0	0
Penicillifer	0	0	0	0	0	0	0	0	0	0	0	0
Penicillium	0	0	0	0	0	578	91	0	126	370	56	0
Peniophora	0	42	0	0	72	78	0	120	0	372	0	0
Peniophorella	200	0	0	127	82	159	71	232	76	350	0	0
Periconia	591	3014	108	47	643	755	0	70	111	1086	1797	0
Pestalotiopsis	0	0	0	0	0	0	0	0	0	0	0	0
Peyronellaea	0	0	0	0	0	0	0	0	0	0	0	0
Peziza	0	0	0	0	0	0	0	0	0	0	0	0
Phacidium	0	0	0	0	0	0	0	0	0	0	0	0
Phaeoacremonium	0	0	0	0	0	0	0	0	0	0	0	0
Phaeophleospora	0	0	0	0	0	0	0	0	0	0	0	0
Phaeosphaeria	841	34	287	0	0	0	0	0	0	0	0	0
Phialocephala	0	0	0	0	0	0	0	0	0	0	0	0
Phialosimplex	0	0	0	0	0	0	0	0	0	0	0	0
Phlebiopsis	0	0	0	0	0	0	0	0	0	0	0	0
Phyllachora	0	0	0	0	0	0	0	133	60	0	0	42
Phyllosticta	59	0	0	0	13024	48	0	0	0	341	0	0
Physisporinus	0	0	0	0	0	0	0	0	0	0	0	0
Pichia	0	0	238	0	0	0	46	0	0	381	0	100
Plectosphaerella	0	0	356	0	0	0	0	0	0	0	0	0
Plenodomus	0	0	0	0	0	0	0	0	0	0	0	0
Pleurotus	67	0	103	215	150	94	0	337	136	358	17249	26
Podosphaera	0	0	0	0	0	0	0	0	0	0	0	0
Powellomyces	0	0	0	0	0	0	0	0	0	0	0	0
Preussia	0	0	0	0	0	0	0	0	0	0	0	0
Protomyces	0	0	0	0	0	0	0	0	0	0	0	0
Psathyrella	0	0	0	0	0	0	0	0	0	0	0	0
Pseudocercospora	0	0	0	0	0	0	0	0	0	0	0	45
Pseudogymnoascus	0	0	0	0	0	0	0	0	0	0	0	0
Pseudomicrostroma	0	0	0	0	0	0	0	0	0	0	0	0
Pseudopithomyces	0	0	0	0	0	0	0	0	0	0	0	0
Pseudoramichloridium	0	0	0	0	0	0	0	0	0	0	68	0
Pseudorobillarda	0	0	0	0	0	0	0	0	0	0	0	0
Pseudoveronaea	0	0	0	0	0	0	0	0	70	0	0	0
Puccinia	0	0	0	0	0	0	0	0	0	0	0	0
Punctularia	0	0	0	0	0	0	0	0	0	0	0	0
Purpureocillium	0	0	0	0	0	0	0	0	0	0	0	36
Pyrenochaeta	0	0	0	0	0	0	0	0	0	0	0	0
Pyrenochaetopsis	0	0	0	0	0	0	0	0	0	0	0	34
Pyricularia	0	0	0	0	0	0	0	0	0	0	0	0
Rachicladosporium	0	0	0	0	0	0	0	0	0	0	0	0
Radulomyces	0	0	0	46	0	0	0	76	68	0	0	0
Raffaelea	1240	1239	572	923	939	3271	506805	822	749	4654	1810	647
Ramophialophora	0	0	0	0	0	0	0	0	0	0	0	0
Ramularia	0	0	0	0	0	0	0	0	62	0	0	0
Recurvomyces	0	0	0	0	0	0	0	0	0	0	62	0
Rhexothecium	242	0	0	0	60	0	0	0	0	0	478	0
Rhizopogon	0	0	0	0	0	0	0	0	0	0	0	0
Rhizopus	0	0	0	0	0	0	0	0	0	0	0	0
Rhodosporidiobolus	0	0	0	0	0	0	0	0	0	0	0	0
Rhodotorula	0	0	0	0	0	0	0	0	0	0	2165	0
Rhopalophora	0	0	0	0	0	0	0	0	0	0	0	0

Robbauera	0	0	0	0	0	609	0	0	0	0	0	0
Roussoella	0	0	0	0	0	0	0	0	0	0	0	0
Rozellomycota_gen_Incertae_sedis	0	0	0	0	0	0	0	0	0	337	0	0
Ruhlandiella	0	0	0	0	0	0	0	0	0	0	0	0
Russula	169020	382	859	95282	613	915	423	644	431	3123	17339	192
Russulales_gen_Incertae_sedis	0	0	0	0	0	0	0	0	0	0	0	0
Saccharomyces	0	0	143	53	0	0	153	0	0	2079	0	43
Saccharomycopsis	0	0	0	0	0	0	0	0	0	0	0	0
Saprochaete	0	0	0	0	0	0	0	0	0	0	0	0
Sarocladium	0	0	0	0	0	38	0	0	0	345	0	0
Schizophyllum	0	0	0	0	0	0	0	0	0	0	1088	1584
Scopulariopsis	0	0	0	0	0	0	0	0	0	0	0	0
Scytinostroma	0	0	0	0	0	0	0	0	0	0	0	0
Septoria	0	0	0	0	0	0	0	0	0	0	0	0
Setophaeosphaeria	0	0	0	0	0	0	0	0	0	0	0	0
Sidera	0	0	0	0	0	875	0	0	0	0	0	0
Simplicillium	2405	56016	912	1862	8464	20963	438	3198	39903	5940	13965	787
Sistotrema	0	0	0	0	0	0	0	0	0	0	0	0
Sistotremastrum	0	0	0	0	0	0	0	0	0	0	0	0
Skeletocutis	0	0	0	0	12541	0	50	0	0	0	0	0
Sordaria	0	0	0	0	0	0	0	0	0	0	0	0
Spiromastix	0	0	238	150	0	186	43	0	129	0	0	0
Sporobolomyces	77	0	0	0	0	0	0	0	0	0	7316	0
Sporothrix	0	0	0	0	0	0	0	0	0	0	0	0
Stachybotrys	0	0	0	0	0	0	0	0	0	0	0	0
Stagonosporopsis	90	95	0	39112	154	87	0	195	133	367	0	0
Starmerella	69	41	0	0	0	38	45	0	0	0	0	0
Steccherinum	0	0	0	0	5878	0	0	0	63	0	0	0
Stemphylium	0	0	0	0	0	0	0	132	0	0	13563	37
Sterigmatomyces	0	0	0	0	0	1435	0	0	0	0	0	0
Strelitziana	0	0	0	0	0	0	0	0	0	0	0	0
Strobilurus	0	0	0	0	0	0	0	0	0	0	0	0
Subulicystidium	0	0	0	0	0	0	0	0	0	0	0	0
Symmetrospora	0	0	0	0	0	0	0	0	0	0	0	0
Sympodiomycopsis	0	0	0	0	0	0	0	0	0	0	0	0
Talaromyces	441	84	267	328	331	293	128	407	47509	684	38749	599
Tausonia	0	0	0	49	0	2769	0	0	0	0	0	35
Tetracladium	0	0	0	0	0	0	0	0	0	0	0	0
Thanatephorus	67	0	0	0	0	0	0	0	0	0	3995	113
Thecaphora	0	0	0	0	0	0	0	0	0	0	20567	44
Thelebolus	0	0	0	0	0	0	0	0	0	0	180	190
Thermomyces	0	0	0	0	0	0	0	0	0	0	0	0
Thielaviopsis	0	0	0	0	0	0	0	0	0	0	0	0
Tilletia	66	0	0	40	6351	775	44	74	0	350	68	67
Tilletiopsis	0	0	0	0	0	46	0	0	0	0	67	114
Togniniella	0	0	0	0	0	0	37	0	0	0	0	0
Toxicocladosporium	0	0	0	0	0	0	0	0	0	0	0	0
Trametes	0	0	0	0	0	0	0	0	0	0	11104	615
Trechispora	0	0	0	0	0	0	0	0	0	0	0	0
Tremateia	0	0	0	0	0	0	159	0	0	0	0	0
Trichoderma	0	0	0	0	0	0	0	0	0	0	0	0
Trichomeriaceae_gen_Incertae_sedis	0	0	0	0	0	0	0	0	0	0	0	0
Trichomerium	0	0	0	0	0	0	0	0	0	0	5267	0
Trichomonascus	0	0	0	0	0	0	0	0	0	0	0	0
Trichophaea	0	0	0	0	0	82	0	0	0	0	0	39
Trichosporon	0	0	0	0	0	0	0	0	0	0	0	0
Trigonopsis	0	0	0	41	0	0	0	0	0	0	0	0
Tritirachium	0	0	0	0	0	0	0	0	0	0	0	0

Tuber	0	0	202	0	0	0	0	0	0	0	0	0
Tubeufia	126	0	0	0	0	76	39	0	133	0	0	0
Tulostoma	0	0	86	125	0	0	40	67	0	0	0	0
Typhula	64	0	80	130	147	0	41	74	0	1047	0	211
Udeniomyces	0	0	0	0	0	0	0	67	0	0	475	1239
Umbilicaria	0	0	0	0	0	0	0	0	0	0	0	0
Umthunziomyces	0	0	0	0	0	0	0	0	0	0	0	0
Ustilaginoidea	0	0	0	0	0	0	0	80	0	0	0	0
Uwebraunia	0	0	0	0	0	0	0	0	0	0	0	0
Valsaria	0	0	0	39	0	45	0	0	3263	0	0	0
Vandijckella	0	0	0	0	0	0	0	0	0	0	0	0
Vanrija	0	0	0	0	0	0	0	0	0	0	0	0
Vararia	0	0	0	0	0	0	0	0	0	0	0	0
Venturiaceae_gen_Incertae_sedis	0	0	0	0	0	0	0	0	0	0	0	0
Verrucaria	0	0	50	0	0	0	0	0	0	0	0	0
Vishniacozyma	0	0	0	44	0	0	0	0	0	0	0	0
Wallemia	0	0	48	126	7608	8868	0	400	0	0	1853	0
Wickerhamiella	0	0	0	0	0	2649	0	0	0	0	0	0
Wickerhamomyces	0	0	0	0	0	188	0	0	0	0	0	0
Xenodevriesia	0	0	0	0	0	0	0	0	0	0	0	0
Xeromyces	0	0	0	0	0	0	0	0	0	0	0	0
Xylocoremium	0	0	0	0	0	0	0	0	0	0	0	0
Xylodon	0	0	0	0	0	0	0	0	0	0	0	0
Yamadazyma	0	0	0	0	0	0	0	0	0	0	0	0
Yarrowia	158	84	63	148	18880	2367	0	128	128	0	0	0
Zanclospora	10539	28290	9354	41137	478715	318372	5202	12488	199428	314886	179046	7736
Zasmidium	0	0	0	0	0	0	0	0	0	0	0	0
Zeloasperisporium	0	0	0	0	0	0	0	0	0	0	0	0
Zoophthora	601	0	0	41	78	902	0	0	0	0	3356	31
Zygosaccharomyces	0	0	0	0	0	142	0	0	0	0	0	0
Zygosporium	0	0	0	0	0	0	0	0	0	0	0	0
Zygotorulaspora	2861	0	50	1564	0	0	39	0	0	0	0	41
Zymoseptoria	0	0	0	0	0	0	0	0	0	0	0	0

Table S3 .The abundance and composition at the species level of each sample

species	Control001	Control003	Control004	Control005	Control006	Control007	Control008	Control009	Control010	Control012	Control013	Control017
Acremonium_acutatum	0	0	0	0	0	0	0	0	0	0	0	0
Acremonium_alternatum	0	0	0	0	0	0	0	0	0	0	0	0
Acremonium_cavaraeanum	0	0	0	0	0	0	0	0	0	0	0	0
Acremonium_charticola	0	0	0	0	0	0	0	0	0	0	0	0
Acremonium_egyptiacum	0	0	0	0	0	0	0	80	0	0	0	0
Acremonium_vitellinum	0	0	0	0	0	0	0	0	0	0	0	0
Acrocalymma_fici	0	46	0	0	0	43	49	0	54	0	65	0
Acrodictys_fluminicola	744214	812817	11466	559057	205691	480837	6191	275297	183709	533166	458328	205881
Acrodontium_crateriforme	0	0	0	0	0	0	0	0	0	0	0	0
Actinomucor_elegans	0	0	0	0	0	0	0	0	0	0	0	0
Agaricales_spe_Incertae_sedis	0	0	0	0	0	0	0	0	0	0	0	0
Agaricus_subfloccosus	0	0	0	0	0	0	0	0	0	0	0	0
Ahmadea_dalanensis	0	0	0	0	0	0	0	0	0	0	0	202
Allocryptovalsa_polyspora	0	0	0	0	0	0	0	0	0	0	0	0
Alternaria_alternata	205	6116	260	164	77	164	93	264	31354	1076	6280	671
Amphobotrys_ricini	0	0	0	0	0	0	0	122	0	0	3346	0
Amylostereum_orientale	0	0	0	0	0	0	0	0	0	0	0	0
Anastomitrabeculia_didymospora	0	0	0	0	0	0	0	0	0	0	0	0
Angustimassarina_camporesii	621	3940	0	84	144	2877	0	634	848	10696	3441	36
Anisomeridium_guangdongensis	0	43	0	0	70	167	0	76	0	0	125	0
Antennariella_placitae	0	0	0	0	0	0	0	0	0	0	13314	35
Anthracina_ramosa	0	2359	0	0	0	0	0	0	0	0	0	0
Apiotrichum_brassicae	0	0	0	0	0	0	0	0	0	0	0	0

Apiotrichum_domesticum	0	0	0	0	0	1355	0	80	77	0	72	144
Aquapteridospora_fusiformis	0	0	0	0	0	0	0	0	0	0	0	0
Archaeorhizomyces_borealis	0	0	0	0	0	0	0	0	0	0	0	0
Arthrographis_grakistii	2138	1560	1863	13627	2563	3298	1103	672077	2421	9399	1064	747216
Ascochyta_rabiei	0	0	54	0	0	0	2263	0	0	0	0	0
Ascomycota_spe_Incertae_sedis	0	0	0	0	0	337	0	0	63	0	0	0
Ascosphaera_duoformis	0	0	0	0	0	0	0	0	0	0	0	0
Ascotricha_erinacea	0	0	0	0	0	0	0	0	0	0	51	90
Ascotricha_guamensis	0	0	0	0	0	0	0	0	0	0	0	0
Aspergillus_amstelodami	146	84	352	381	161	11195	530	0	68734	1056	346	128
Aspergillus_baarnensis	0	0	0	0	0	0	0	0	0	0	0	0
Aspergillus_caesiellus	0	0	0	0	0	0	0	0	0	0	0	0
Aspergillus_canadensis	0	0	0	0	0	74	0	0	370	0	0	0
Aspergillus_clavatophorus	0	0	0	0	0	0	0	0	131	0	0	0
Aspergillus_coremiiformis	0	0	0	0	0	0	0	0	0	0	0	0
Aspergillus_flavus	0	0	98	0	0	0	0	57	0	0	0	0
Aspergillus_foetidus	0	0	108	123	10697	169	0	0	74	1050	0	0
Aspergillus_fumisynnematus	0	0	0	0	0	0	0	0	0	0	0	0
Aspergillus_insolitus	0	0	0	0	0	0	0	0	0	0	0	0
Aspergillus_neoglaber	0	0	0	0	0	0	0	0	0	0	0	0
Aspergillus_penicillioides	75	64	0	0	0	430	85	74	12800	0	68	190
Aspergillus_pragensis	0	0	0	0	0	0	0	0	0	0	0	0
Aspergillus_restrictus	0	0	0	0	0	0	52	0	0	0	908	0
Aspergillus_subflavus	1936	872	1322	3242	1240	3106	933	1095	1396	6991	3783	1823
Aspergillus_tennesseensis	0	0	0	0	0	0	0	0	136	0	0	0
Aspergillus_thermomutatus	0	0	0	0	0	984	0	0	0	0	62	0
Aspergillus_versicolor	75	0	38	42	86	64	0	143	12459	343	63	113
Aspergillus_vitricola	0	43	43	38	85	1795	46	0	26833	0	0	0
Aspergillus_westerdijkiae	0	0	0	0	0	0	0	0	0	0	0	0
Aspergillus_whitfieldii	0	0	0	0	0	0	0	0	0	0	0	0
Atractiella_rhizophila	0	0	0	0	0	0	0	0	0	0	0	0
Aureobasidium_pullulans	0	0	0	0	0	0	0	0	0	0	0	205
Aureobasidium_thailandense	0	0	0	0	0	0	0	0	0	0	0	0
Auricularia_auricula-judae	0	0	0	0	0	0	0	64	0	0	179	185
Auricularia_delicata	0	0	0	0	0	0	0	0	0	0	0	0
Austropleospora_keteleeriae	0	0	0	0	0	0	0	0	0	0	0	0
Avachytrium_platense	0	0	0	0	0	0	0	0	0	0	0	0
Bandonia_marina	0	0	0	0	0	0	0	0	0	0	0	0
Banksiophoma_australiensis	0	0	0	0	0	0	0	0	0	0	0	0
Basidiomycota_spe_Incertae_sedis	0	0	0	0	0	0	0	0	0	0	0	0
Beauveria_bassiana	0	0	0	0	0	0	0	160	0	0	0	0
Beauveria_lii	0	0	0	0	0	0	0	0	0	0	0	0
Bergerella_atrofusca	0	0	0	0	0	0	0	0	0	0	175	0
Biatriosporaceae_spe_Incertae_sedis	0	0	0	0	0	0	0	0	0	0	0	0
Bjerkandera_adusta	0	0	0	0	0	1491	0	0	0	334	0	37
Bjerkandera_mikrofumosa	151	0	290	256	322	215	105	138	183	1790	0	0
Blastobotrys_proliferans	0	0	0	0	0	0	0	0	0	0	0	0
Blumeria_graminis	0	0	0	0	0	0	0	0	0	0	0	0
Blumeria_spe_Incertae_sedis	1609	501	1109	1622	1319	2447	873	1447	1855	6484	290	190
Botryosphaeria_corticis	0	0	0	0	0	0	0	0	0	0	0	0
Botryosphaeria_spe_Incertae_sedis	0	0	0	0	0	0	0	0	0	0	0	0
Botryosphaeria_wangensis	0	0	0	48	0	78	100	86	143	0	0	0
Botrytis_cinerea	0	34	0	0	0	0	0	0	0	0	69	0
Bovista_aestivalis	0	0	0	0	0	0	0	0	0	0	0	0
Buckleyzyma_armeniaca	0	0	0	0	0	0	0	0	0	0	0	0
Buckleyzyma_aurantiaca	0	0	0	0	0	0	0	0	0	0	0	0
Bulleromyces_albus	0	0	0	0	0	0	0	0	0	0	0	0
Byssochlamys_spectabilis	0	0	0	0	0	0	0	0	0	0	0	0

Calosphaeria_pulchella	0	424	38	0	0	1830	0	131	253	722	304	34
Calycina_spe_Incertae_sedis	0	0	0	0	0	0	0	0	0	0	0	0
Calyptella_capula	0	0	0	0	0	0	0	0	0	0	0	32
Candida_albicans	61	0	552	158	0	202	0	546	0	0	136	115
Candida_apicola	0	0	0	0	0	0	0	0	0	0	0	0
Candida_ethanolica	0	0	0	0	0	0	0	0	0	0	0	0
Candida_inconspicua	0	0	0	0	0	0	0	0	0	0	0	0
Candida_maltosa	0	0	0	0	0	1636	0	0	0	0	0	0
Candida_nivariensis	0	0	0	0	0	0	0	0	0	0	0	0
Candida_parapsilosis	72	0	0	0	0	0	0	0	0	0	65	259
Candida_saitoana	0	0	0	0	0	0	0	0	0	0	0	0
Candida_saraburiensis	0	0	0	0	0	0	0	0	0	0	0	0
Candida_solani	0	0	0	0	0	0	0	0	0	0	0	0
Candida_spe_Incertae_sedis	505	0	46	56	0	3973	1176	88	0	0	369	272
Candida_tropicalis	0	0	0	0	0	43	0	0	0	0	0	0
Candida_vespimorsuum	0	0	0	0	0	237	0	0	0	0	0	0
Candida_zeylanoides	161	0	0	0	0	0	0	0	0	0	60	30
Capnodiales_spe_Incertae_sedis	641	0	0	0	0	0	0	0	0	0	0	0
Carlosrosaea_hohenbergiae	0	0	0	0	0	0	0	0	0	0	0	0
Ceraceosorus_guamensis	0	0	0	0	0	0	0	0	0	0	0	0
Ceratocystis_manginecans	0	0	0	0	0	0	0	0	0	0	0	0
Ceratocystis_pirilliformis	0	0	0	0	0	0	0	0	0	0	0	0
Cercospora_canescens	0	79	49	0	0	45	0	70	74	0	0	0
Ceriporia_lacerata	0	0	41	41	0	49	0	52	127	0	0	0
Ceriporia_xylostromatoides	0	0	0	0	0	0	0	0	0	0	0	0
Ceriporiopsis_fimbriata	0	0	0	0	0	0	0	0	0	0	0	0
Cerrena_aurantiopora	0	0	0	0	0	0	0	69	0	0	0	0
Chaenothecopsis_resinophila	0	0	0	0	0	0	0	0	0	0	0	0
Chaetomium_globosum	0	0	0	0	0	0	0	0	0	0	0	0
Chalara_hughesii	0	0	0	0	0	0	0	0	0	0	0	0
Choanephora_cucurbitarum	0	0	0	0	0	0	0	0	0	0	0	0
Chrysosphaeria_jan-nelii	132	46	46	42	296	9099	0	0	139	367	0	127
Chytridiomycota_spe_Incertae_sedis	0	0	0	0	0	0	0	0	0	0	0	0
Ciliophora_spe_Incertae_sedis	0	0	0	10477	69	38	48	0	0	0	0	0
Cladophialophora_spe_Incertae_sedis	0	0	0	0	0	0	0	0	0	0	0	0
Cladosporium_cladosporioides	726	206	161	6476	6626	497	126	186	57	687	1852	529
Cladosporium_coloradense	0	0	0	0	63	0	0	0	0	0	0	0
Cladosporium_halotolerans	0	66	43	402	17256	254	1259	415	130	2847	901	962
Cladosporium_kenpeggii	73	0	60	687	1595	3603	627	337	0	0	33347	1546
Cladosporium_spe_Incertae_sedis	272	143	190	26921	255	797	42	780	266	1439	842	353
Cladosporium_sphaerospermum	0	33	0	0	23185	0	0	0	0	0	0	0
Clavispora_lusitaniae	0	0	0	0	0	0	0	0	0	0	0	0
Clonostachys_eriocamporesii	0	0	0	0	0	0	0	0	0	0	0	0
Clonostachys_rosea	0	0	0	0	0	0	0	0	0	0	0	0
Colletotrichum_cliviae	0	0	50	0	0	937	0	0	0	0	0	0
Collybiopsis_filamentipes	0	0	0	0	0	0	0	0	0	0	0	0
Coniochaeta_mutabilis	0	0	0	0	0	0	0	0	0	0	0	0
Coniosporium_apollinis	0	0	0	0	0	0	0	0	0	0	0	0
Coniosporium_spe_Incertae_sedis	0	0	0	0	0	0	0	0	0	0	0	0
Coniothyrium_spe_Incertae_sedis	0	0	0	0	0	0	0	0	0	0	0	0
Cookeina_korfii	0	0	0	0	0	0	0	0	0	0	0	0
Coprinellus_ovatus	77	69	58	0	165	174	0	130	52421	1058	130	123
Coprinellus_pseudodisseminatus	0	0	0	0	0	0	0	0	0	0	68	63
Coprinopsis_strossmayeri	0	0	0	0	0	0	0	0	0	0	0	0
Coprinopsis_urticicola	78	47	44	170	0	0	94	77	136	346	0	0
Cordyceps_lepidopterorum	0	0	0	0	0	0	0	0	0	0	0	0
Cordyceps_succavus	0	0	0	0	0	0	0	0	0	0	0	0
Coronicium_gemmiferum	0	0	0	0	0	0	0	0	0	0	0	0

Crustoderma_spe_Incertae_sedis	0	0	0	0	0	0	0	0	0	0	0	0
Cryptococcus_longus	1739	23993	1422	42617	1143	1569	971	1610	22608	7408	1781	3881
Cryptococcus_neoformans	0	0	0	0	0	0	0	0	0	0	0	0
Cryptococcus_uniguttulatus	0	0	0	0	0	0	0	0	0	0	0	0
Cryptomarasmius_aucubae	0	0	0	0	0	0	0	0	0	0	0	0
Curvularia_soli	0	0	0	0	0	0	0	0	0	0	0	0
Cutaneotrichosporon_arboriforme	0	0	0	0	0	0	0	0	0	0	0	0
Cutaneotrichosporon_cutaneum	0	0	0	0	0	0	0	0	0	717	0	0
Cutaneotrichosporon_jirovecii	141	37	141	96	0	47	0	0	32393	682	0	0
Cyphellaceae_spe_Incertae_sedis	0	0	0	0	0	0	0	0	0	0	0	0
Cyphellophora_olivacea	0	0	0	0	0	0	0	0	0	0	0	0
Cyphellophora_sessilis	0	0	0	0	0	0	0	0	0	0	0	0
Cystofilobasidium_capitatum	0	0	0	0	0	0	0	0	0	0	0	0
Cystofilobasidium_infirmominiatum	0	0	0	0	0	0	0	0	0	0	0	0
Cystofilobasidium_macerans	0	0	0	0	0	0	0	0	63	354	118	382
Cytospora_myrtagena	0	0	0	0	0	0	0	0	0	0	0	0
Cytospora_predappioensis	0	0	0	0	0	0	0	0	0	0	11909	0
Dactylella_spe_Incertae_sedis	0	0	0	48	8548	0	0	0	0	0	0	0
Dactylodendron_pinicola	0	0	0	0	0	0	0	0	0	0	0	0
Debaryomyces_prosopidis	0	0	0	0	0	0	0	0	0	0	119	97
Deconica_spe_Incertae_sedis	0	0	0	0	0	0	0	0	0	0	67	35
Delastria_faustiniana	0	0	0	0	0	0	0	0	0	0	0	0
Dendryphiella_paravinosa	0	0	0	0	0	0	0	0	0	0	0	0
Derxomyces_mrakii	0	0	0	0	0	0	0	0	0	0	0	0
Diaporthe_bohemiae	0	0	0	0	0	0	0	0	0	0	0	0
Diaporthe_caulivora	0	0	0	0	0	0	0	0	0	0	63	204
Diaporthe_celeris	0	0	0	0	0	0	0	0	0	0	0	0
Diaporthe_ganjae	0	0	0	0	0	0	0	0	0	0	0	0
Diaporthe_kochmanii	0	0	0	0	0	0	0	0	0	0	55	265
Diaporthe_passifloricola	0	0	0	0	0	260	0	0	0	0	0	0
Diaporthe_pseudooculi	0	0	0	0	0	0	0	0	0	0	0	0
Diaporthe_searlei	0	0	2356	0	0	0	0	0	0	0	0	0
Didymella_dimorpha	66	68	137	0	0	0	0	56	27160	711	0	0
Didymella_spe_Incertae_sedis	0	0	93	0	0	0	0	0	0	0	68	0
Didymocyrtis_brachylaenae	0	0	0	0	0	0	0	0	0	0	0	0
Dimorphoma_saxea	0	0	0	0	0	0	0	0	0	0	0	0
Dioszegia_catarinonii	0	0	0	0	0	0	0	0	0	0	0	0
Dioszegia_zsoltii_var._yunnanensis	0	0	0	0	0	0	0	0	0	0	61	196
Dipodascus_geotrichum	0	0	0	0	0	0	0	0	0	0	0	0
Diutina_catenulata	0	0	0	0	0	0	0	0	0	0	0	0
Diutina_rugosa	0	0	0	0	0	0	0	979	0	0	0	0
Diutina_sipiczkii	0	0	0	0	0	0	0	0	0	0	0	0
Diversispora_spurca	40067	820	905000	38344	3813	16178	1324	1270	1637	11034	171	299
Dothidea_eucalypti	0	0	0	0	11718	0	0	0	0	0	0	0
Dothidea_sambuci	0	0	0	0	0	0	0	0	0	0	0	0
Dothiora_spartii	0	0	0	0	0	0	0	0	0	0	0	0
Echinoporia_hydnophora	0	0	0	0	0	0	0	135	0	0	0	0
Edenia_gomezpompae	0	0	0	0	0	0	0	0	0	0	0	0
Effuseotrichosporon_vanderwaltii	0	0	0	0	0	0	0	0	0	0	0	0
Elaphomyces_anthracinus	0	0	0	0	0	0	0	0	0	0	2466	0
Elsinoe_leucopogonis	0	0	0	0	0	0	0	0	0	0	0	0
Engyodontium_album	0	42	0	0	0	0	0	0	0	0	0	65
Entoloma_graphitipes_f._cystidiatum	0	0	0	0	0	0	0	0	0	0	0	0
Entorrhiza_citriformis	0	0	0	0	0	0	0	147	0	0	50	0
Entorrhiza_parvula	0	0	0	0	0	0	0	0	0	0	0	0
Entyloma_calendulae	0	0	0	0	0	0	0	0	0	0	0	0
Entyloma_cosmi	0	0	0	0	0	0	0	0	0	0	0	0
Entyloma_linariae	0	0	0	0	0	0	0	0	0	0	0	0

Epicoccum_dendrobii	69	44	117	84	94	115	518	62	28339	692	0	0
Eremothecium_ashbyi	0	0	0	0	0	0	0	0	0	0	0	0
Erythrobasidium_hasegawianum	0	0	0	0	0	0	0	0	0	0	53	47
Eupenidiella_venezuelensis	0	0	0	0	0	0	0	0	0	0	0	0
Eutypella_spe_Incertae_sedis	0	0	0	0	0	0	0	0	0	0	68	37
Extremus_spe_Incertae_sedis	0	0	0	0	0	0	0	0	0	0	0	0
Fantasmomyces_hyalinus	0	2433	43	37	159	2685	80	1275	830	727	753	310
Filobasidium_oeirense	0	39	0	0	0	0	0	0	0	0	0	124
Filobasidium_wieringae	0	0	0	0	0	0	0	0	0	0	119	219
Flagelloscypha_spe_Incertae_sedis	0	0	0	0	0	0	0	0	0	0	0	0
Flammulina_filiformis	0	0	0	0	0	0	0	0	0	0	68	95
Flammulina_velutipes	0	0	0	0	0	0	0	0	0	0	0	0
Flavodon_spe_Incertae_sedis	0	0	0	0	0	0	0	0	0	0	0	0
Fomitopsis_eucalypticola	0	0	0	0	0	0	0	0	0	0	0	0
Fungi_spe_Incertae_sedis	11596	30935	47086	33272	16099	50209	461225	4378	30663	35241	31794	2572
Fusarium_fujikuroi	0	34	0	0	0	688	0	0	0	0	0	0
Fusarium_lacertarum	231	0	0	45	0	1938	243	127	0	362	0	33
Fusarium_neocosmosporiellum	71	0	0	177	76	66	0	76	180	354	0	0
Fusarium_oxysporum	0	0	0	0	0	0	0	0	0	0	0	0
Fusarium_poae	0	0	0	0	0	0	0	140	0	0	0	0
Fusarium_ramigenum	0	0	0	0	0	0	0	0	0	0	0	0
Fuscosporella_aquatica	161	39	0	0	0	0	0	0	0	0	119	0
Fusicolla_acetilerea	0	0	0	0	0	79	0	0	0	0	0	0
Galactomyces_pseudocandidus	0	0	0	0	0	0	0	0	0	0	0	0
Ganoderma_australe	0	0	0	0	0	0	0	0	0	0	0	0
Genea_brunneocarpa	0	0	8482	0	0	125	0	0	72	0	0	0
Gibberella_baccata	0	0	0	0	0	0	0	0	0	0	59	0
Gibberella_fujikuroi	0	0	46	0	0	0	0	0	0	0	60	0
Gibellula_shennongjiaensis	0	265	0	0	62	556	753	0	0	0	1785	0
Gjaerumia_minor	0	0	0	0	0	0	0	0	0	0	0	0
Glomus_macrocarpum	0	0	0	0	0	0	0	0	0	0	43	37
Golovinomyces_latisporus	0	0	0	0	0	0	0	0	0	0	0	0
Golovinomyces_sonchicola	0	0	0	0	0	0	0	0	0	0	0	0
Golubevia_pallescens	0	0	0	0	0	0	0	0	0	0	0	0
Gonatophragmium_spe_Incertae_sedis	0	0	0	0	0	0	0	0	0	0	0	0
Gymnopus_variicolor	0	0	0	0	0	0	0	0	0	0	0	0
Halosphaeriaceae_spe_Incertae_sedis	0	0	0	0	0	0	0	0	0	0	0	0
Hannaella_kunmingensis	0	0	0	0	0	0	0	0	0	0	0	0
Hannaella_oryzae	0	0	0	0	0	0	0	0	0	0	0	0
Hannaella_pagnoccae	0	0	0	0	0	0	0	0	0	0	0	0
Hannaella_sinensis	0	0	0	0	0	0	0	0	0	0	0	77
Hannaella_surugaensis	0	0	0	0	0	0	0	0	0	0	0	0
Hanseniaspora_nectarophila	0	0	0	0	0	0	0	0	0	0	0	0
Hazslinszkyomyces_aloes	0	0	0	0	0	0	0	0	0	0	0	0
Heitmania_litseae	0	249	0	0	694	2995	1692	2621	255	328	268	62
Helicoma_brunneisporum	0	0	0	0	0	0	0	0	0	0	0	0
Heliocybe_villosa	0	0	0	43	0	0	41	0	0	0	0	0
Henningsia_resupinata	0	0	0	0	0	0	0	0	0	0	0	0
Heydenia_spe_Incertae_sedis	0	0	0	0	0	0	0	0	0	0	0	0
Holtermanniella_takashimae	0	0	0	0	0	0	0	0	0	0	0	0
Hormodochis_melanochlora	0	0	0	0	0	0	0	0	0	0	0	0
Hortaea_werneckii	0	0	0	0	0	0	0	0	0	0	0	0
Houjia_yanglingensis	0	0	0	0	0	0	0	0	0	0	0	0
Huntiella_tyalla	0	0	0	0	0	0	0	0	0	0	0	0
Hydnobolites_roseus	64	67	96	45	77	137	0	221	0	681	49	0
Hyphopichia_burtonii	0	0	0	0	0	0	0	0	0	0	0	0
Hypoxylon_perforatum	0	44	135	0	73	84	45	157	69	725	0	0
Irpex_lacteus	0	0	0	0	0	0	0	0	0	0	0	0

Issatchenkia_orientalis	0	0	0	0	0	0	0	0	0	0	0	0
Itersonilia_perplexans	0	0	0	0	0	0	0	0	0	0	0	0
Jaminaea_angkorensis	0	0	0	0	0	0	0	0	0	0	0	0
Kazachstania_exigua	0	0	0	0	0	0	0	0	0	0	0	0
Kazachstania_heterogenica	131	155	91	88	154	129	182	362	57	1410	0	609
Kazachstania_humilis	0	0	0	0	0	0	0	0	0	0	0	0
Keissleriella_quadriseptata	74	47	93	85	80	41	43	214	63	364	0	0
Keratinophyton_linfenense	0	0	0	0	0	0	0	0	0	0	0	0
Kneiffiella_efibulata	0	0	0	0	0	0	0	0	0	0	0	0
Knufia_cryptophialidica	0	0	0	0	0	720	0	0	0	0	0	0
Knufia_epidermidis	0	0	0	0	0	0	0	0	0	0	0	0
Knufia_hypolithi	0	0	0	0	0	0	0	0	0	0	68	108
Knufia_mediterranea	0	0	0	0	0	0	0	0	0	0	0	0
Knufia_tsunedae	0	0	0	0	0	0	0	0	0	0	0	0
Kodamaea_ohmeri	0	0	0	0	0	0	0	0	0	0	0	0
Kurtzmaniella_quercitrusa	0	0	0	0	0	0	0	0	0	0	0	0
Laburnicola_dactylidis	0	0	0	0	0	0	43	57	0	0	0	0
Laetisaria_lichenicola	0	0	0	0	0	28	0	0	0	0	0	0
Lecanicillium_coprophilum	0	0	0	0	0	0	0	0	0	0	0	0
Lentinula_edodes	0	4764	0	0	0	0	0	0	0	0	61	26
Lentinus_arcularius	0	0	0	0	0	0	0	0	0	0	73	123
Lentinus_squarrosulus	0	0	0	0	0	0	0	0	0	0	0	0
Lentithecium_aquaticum	0	0	0	0	0	0	0	0	0	0	0	0
Leptospora_rubella	0	0	0	0	0	0	0	0	0	0	0	0
Leptospora_spe_Incertae_sedis	0	0	0	0	0	0	0	0	0	0	0	0
Leucosporidium_golubevii	0	0	0	0	0	44	0	0	0	0	0	0
Lipomyces_doorenjongii	0	0	0	0	0	0	0	0	0	0	0	0
Lithohypha_aloicola	0	0	0	0	0	0	0	0	0	0	0	0
Lophiostoma_cynaroidis	0	0	0	0	0	0	0	0	0	0	64	0
Lyomyces_spe_Incertae_sedis	0	0	0	0	0	0	0	0	0	0	0	0
Macrohyporia_dictyopora	0	36	50	49	73	0	0	0	146	341	0	0
Malassezia_arunalokei	506	260	524	535	4540	945	236	458	458	3951	69	67
Malassezia_cuniculi	0	0	0	0	0	0	0	0	0	0	0	0
Malassezia_dermatis	0	0	0	0	245	0	0	0	0	0	0	0
Malassezia_furfur	0	0	0	0	0	0	0	0	0	0	0	40
Malassezia_globosa	51	708	0	382	6780	0	85	0	0	353	1846	155
Malassezia_japonica	0	0	0	6398	0	0	42	0	0	0	0	0
Malassezia_restricta	809	5924	1153	71549	40179	7465	1471	2350	107515	5268	61332	1486
Malassezia_sympodialis	50	71	143	161	28783	111	102	0	15776	347	59	64
Marantokordyana_oberwinkleriana	0	0	0	0	0	0	0	0	0	0	0	0
Meira_argovae	0	0	0	0	0	0	0	0	0	0	0	0
Meira_nashicola	0	0	0	0	0	0	0	0	0	0	0	0
Meyerozyma_guilliermondii	0	0	0	0	0	0	0	0	0	0	0	0
Microascus_paisii	0	0	0	0	0	0	0	0	0	0	0	0
Microascus_trigonosporus	0	0	0	0	0	0	0	0	0	0	0	0
Milospium_graphideorum	114	0	102	104	45889	175	49	75	39675	0	1809	2166
Moesziomyces_antarcticus	0	0	0	0	0	0	0	0	0	0	0	0
Moniliella_suaveolens	0	0	43	0	81	44	0	0	70	318	0	0
Mrakia_aquatica	0	0	0	0	0	0	0	0	0	0	0	0
Mrakia_frigida	0	0	0	0	0	0	0	0	0	0	0	0
Mucor_circinelloides	0	0	0	0	0	0	0	0	0	0	0	0
Mycoarthris_spe_Incertae_sedis	0	0	0	0	0	0	0	0	0	0	0	0
Mycosphaerellaceae_spe_Incertae_sedis	0	0	0	0	0	40	43	0	0	0	54	34
Naganishia_albida	0	0	0	0	0	0	0	65	0	0	462	638
Naganishia_albidosimilis	0	0	0	0	0	0	0	0	0	0	0	0
Naganishia_globosa	0	0	0	0	0	0	0	0	0	0	0	47
Nannizziopsis_guarroi	0	0	0	51	0	0	0	127	0	0	0	0
Nectriopsis_exigua	0	0	0	0	0	0	0	0	0	0	0	0

Nemania_viridis	0	0	0	0	0	0	0	0	0	0	0	0
Neoascochyta_paspali	0	0	0	0	0	0	0	0	0	0	0	0
Neoconiothyrium_viticola	0	0	0	0	0	0	0	0	0	0	0	0
Neocucurbitaria_irregularis	0	0	0	0	0	0	0	0	0	0	0	0
Neodevriesia_lagerstroemiae	0	0	0	0	0	0	0	0	0	0	0	0
Neodevriesia_sexualis	0	0	0	0	0	0	0	0	0	0	0	0
Neodevriesiaceae_spe_Incertae_sedis	0	0	0	0	0	0	0	0	0	0	0	0
Neoerysiphe_galeopsidis	0	0	0	0	0	0	0	0	0	0	0	42
Neofusicoccum_buxi	0	0	0	0	0	0	0	0	0	0	0	69
Neosetophoma_poaceicola	0	0	0	0	0	0	0	0	0	0	64	0
Neosetophoma_samararum	0	0	0	0	0	0	903	0	0	0	0	0
Nigrograna_spe_Incertae_sedis	0	46	0	0	0	0	0	0	63	367	0	0
Nigrospora_oryzae	0	0	0	0	0	0	0	0	0	0	0	0
Nigrospora_spe_Incertae_sedis	0	0	0	0	0	0	0	0	0	0	0	0
Nothophoma_macrospora	0	0	0	0	66	3267	93	0	392	704	0	0
Novakomyces_olei	0	0	0	0	0	0	0	0	0	0	0	0
Ochroconis_mirabilis	0	0	0	0	0	0	0	0	0	0	0	0
Oliveonia_spe_Incertae_sedis	0	0	0	0	0	0	0	0	0	0	0	0
Ophiosphaerella_agrostidis	0	0	0	0	0	0	0	0	0	0	0	0
Ophiosphaerella_aquatica	0	0	0	0	0	68	0	0	0	0	0	0
Ophiostoma_acarorum	71	86	96	0	2244	0	0	0	60	0	3228	104
Ophiostoma_pusillum	0	0	0	0	0	42	0	0	0	0	0	0
Papiliotrema_aurea	0	0	0	0	0	0	0	0	0	0	0	0
Papiliotrema_flavescens	0	0	0	0	0	0	0	0	0	0	0	0
Papiliotrema_terrestris	0	0	0	0	0	0	0	0	0	0	0	0
Paracremonium_binnewijzendii	0	0	0	0	0	0	0	0	0	0	0	0
Parafuscosporella_mucosa	72	7663	154	47	1076	5818	127	8440	1109	8914	6013	8947
Paraleptosphaeria_rumicis	0	0	0	0	0	0	0	0	0	0	0	0
Pararamichloridium_caricicola	0	0	0	0	0	0	0	0	0	0	0	0
Parasarocladium_aestuarinum	0	0	0	0	0	0	0	0	0	0	0	0
Parastagonosporella_fallopiae	125	74	0	0	85	87	0	79	76	726	0	0
Parasympodiella_longispora	0	0	0	0	0	0	0	0	0	0	0	0
Parathyridaria_rosae	0	0	0	0	0	0	0	0	0	0	0	0
Paratritirachium_cylindroconium	0	0	0	0	0	0	0	0	0	0	0	0
Penicillifer_martinii	0	0	0	0	0	0	0	0	0	0	0	0
Penicillium_adametzioides	0	0	0	0	0	0	0	0	0	0	0	0
Penicillium_aurantiogriseum	0	0	0	0	0	43	52	0	126	0	0	0
Penicillium_bialowiezense	0	0	0	0	0	0	0	0	0	0	0	0
Penicillium_brevicompactum	0	0	0	0	0	0	0	0	0	0	0	0
Penicillium_cinnamopurpureum	0	0	0	0	0	0	0	0	0	0	0	0
Penicillium_citrinum	0	0	0	0	0	0	0	0	0	0	0	0
Penicillium_fructuariae-cellae	0	0	0	0	0	0	0	0	0	0	0	0
Penicillium_hirsutum	0	0	0	0	0	535	39	0	0	370	56	0
Penicillium_jiangxiense	0	0	0	0	0	0	0	0	0	0	0	0
Penicillium_lanosocoeruleum	0	0	0	0	0	0	0	0	0	0	0	0
Penicillium_ludwigii	0	0	0	0	0	0	0	0	0	0	0	0
Penicillium_melanoconidium	0	0	0	0	0	0	0	0	0	0	0	0
Penicillium_multicolor	0	0	0	0	0	0	0	0	0	0	0	0
Penicillium_oxalicum	0	0	0	0	0	0	0	0	0	0	0	0
Penicillium_pancosmium	0	0	0	0	0	0	0	0	0	0	0	0
Penicillium_pimiteouiense	0	0	0	0	0	0	0	0	0	0	0	0
Penicillium_steckii	0	0	0	0	0	0	0	0	0	0	0	0
Peniophora_cinerea	0	42	0	0	72	78	0	120	0	372	0	0
Peniophora_crassitunicata	0	0	0	0	0	0	0	0	0	0	0	0
Peniophora_pseudoversicolor	0	0	0	0	0	0	0	0	0	0	0	0
Peniophorella_spe_Incertae_sedis	200	0	0	127	82	159	71	232	76	350	0	0
Periconia_byssoides	591	0	0	0	0	0	0	0	0	0	0	0
Periconia_cortaderiae	0	2328	108	47	643	755	0	70	111	1086	1797	0

Periconia_epilithographicola	0	0	0	0	0	0	0	0	0	0	0	0
Periconia_homothallica	0	0	0	0	0	0	0	0	0	0	0	0
Periconia_macrospinosa	0	686	0	0	0	0	0	0	0	0	0	0
Pestalotiopsis_dracontomelon	0	0	0	0	0	0	0	0	0	0	0	0
Pestalotiopsis_vismiae	0	0	0	0	0	0	0	0	0	0	0	0
Peyronellaea_spe_Incertae_sedis	0	0	0	0	0	0	0	0	0	0	0	0
Peziza_fruticosa	0	0	0	0	0	0	0	0	0	0	0	0
Phacidium_lacerum	0	0	0	0	0	0	0	0	0	0	0	0
Phaeoacremonium_griseo-olivaceum	0	0	0	0	0	0	0	0	0	0	0	0
Phaeophleospora_hymenocallidicola	0	0	0	0	0	0	0	0	0	0	0	0
Phaeosphaeria_acaciae	0	0	0	0	0	0	0	0	0	0	0	0
Phaeosphaeria_fuckelii	93	34	0	0	0	0	0	0	0	0	0	0
Phaeosphaeria_oryzae	748	0	287	0	0	0	0	0	0	0	0	0
Phialocephala_trigonospora	0	0	0	0	0	0	0	0	0	0	0	0
Phialosimplex_salinarum	0	0	0	0	0	0	0	0	0	0	0	0
Phlebiopsis_flavidoalba	0	0	0	0	0	0	0	0	0	0	0	0
Phlebiopsis_spe_Incertae_sedis	0	0	0	0	0	0	0	0	0	0	0	0
Phyllachora_cynodonticola	0	0	0	0	0	0	0	133	60	0	0	42
Phyllosticta_capitalensis	59	0	0	0	13024	48	0	0	0	341	0	0
Physisporinus_tibeticus	0	0	0	0	0	0	0	0	0	0	0	0
Pichia_cactophila	0	0	0	0	0	0	0	0	0	0	0	0
Pichia_fermentans	0	0	238	0	0	0	0	0	0	0	0	0
Pichia_kluyveri	0	0	0	0	0	0	0	0	0	381	0	0
Pichia_mandshurica	0	0	0	0	0	0	0	0	0	0	0	100
Plectosphaerella_cucumerina	0	0	356	0	0	0	0	0	0	0	0	0
Plectosphaerella_niemeijerarum	0	0	0	0	0	0	0	0	0	0	0	0
Plenodomus_enteroleucus	0	0	0	0	0	0	0	0	0	0	0	0
Pleurotus_eryngii	0	0	0	39	0	0	0	0	0	0	0	0
Pleurotus_ostreatus	67	0	103	176	150	94	0	337	136	358	17249	26
Podosphaera_aphanis	0	0	0	0	0	0	0	0	0	0	0	0
Podosphaera_cunningtonii	0	0	0	0	0	0	0	0	0	0	0	0
Powellomyces_spe_Incertae_sedis	0	0	0	0	0	0	0	0	0	0	0	0
Preussia_procaviae	0	0	0	0	0	0	0	0	0	0	0	0
Protomyces_inouyei	0	0	0	0	0	0	0	0	0	0	0	0
Psathyrella_candolleana	0	0	0	0	0	0	0	0	0	0	0	0
Pseudocercospora_snelliana	0	0	0	0	0	0	0	0	0	0	0	45
Pseudogymnoascus_spe_Incertae_sedis	0	0	0	0	0	0	0	0	0	0	0	0
Pseudomicrostroma_phylloplanum	0	0	0	0	0	0	0	0	0	0	0	0
Pseudopithomyces_karoo	0	0	0	0	0	0	0	0	0	0	0	0
Pseudoramichloridium_henryi	0	0	0	0	0	0	0	0	0	0	68	0
Pseudorobillarda_sojae	0	0	0	0	0	0	0	0	0	0	0	0
Pseudoveronaea_ellipsoidea	0	0	0	0	0	0	0	0	70	0	0	0
Puccinia_striiformis	0	0	0	0	0	0	0	0	0	0	0	0
Punctularia_atropurpurascens	0	0	0	0	0	0	0	0	0	0	0	0
Purpureocillium_lilacinum	0	0	0	0	0	0	0	0	0	0	0	36
Pyrenochaeta_spe_Incertae_sedis	0	0	0	0	0	0	0	0	0	0	0	0
Pyrenochaetopsis_leptospora	0	0	0	0	0	0	0	0	0	0	0	0
Pyrenochaetopsis_microspora	0	0	0	0	0	0	0	0	0	0	0	34
Pyricularia_oryzae	0	0	0	0	0	0	0	0	0	0	0	0
Rachicladosporium_spe_Incertae_sedis	0	0	0	0	0	0	0	0	0	0	0	0
Radulomyces_paumanokensis	0	0	0	46	0	0	0	76	68	0	0	0
Raffaelea_albimanens	0	0	0	0	0	0	0	0	0	0	0	0
Raffaelea_lauricola	1240	1239	572	923	939	3271	506805	822	749	4654	1810	647
Ramophialophora_spe_Incertae_sedis	0	0	0	0	0	0	0	0	0	0	0	0
Ramularia_coryli	0	0	0	0	0	0	0	0	62	0	0	0
Recurvomyces_spe_Incertae_sedis	0	0	0	0	0	0	0	0	0	0	62	0
Rhexothecium_globosum	242	0	0	0	60	0	0	0	0	0	478	0
Rhizopogon_laricinus	0	0	0	0	0	0	0	0	0	0	0	0

Rhizopus_arrhizus	0	0	0	0	0	0	0	0	0	0	0	0
Rhodosporidiobolus_colostri	0	0	0	0	0	0	0	0	0	0	0	0
Rhodotorula_diobovata	0	0	0	0	0	0	0	0	0	0	2165	0
Rhodotorula_glutinis	0	0	0	0	0	0	0	0	0	0	0	0
Rhodotorula_ingeniosa	0	0	0	0	0	0	0	0	0	0	0	0
Rhodotorula_mucilaginosa	0	0	0	0	0	0	0	0	0	0	0	0
Rhopalophora_clavispora	0	0	0	0	0	0	0	0	0	0	0	0
Robbauera_albescens	0	0	0	0	0	609	0	0	0	0	0	0
Roussoella_neopustulans	0	0	0	0	0	0	0	0	0	0	0	0
Roussoella_solani	0	0	0	0	0	0	0	0	0	0	0	0
Rozellomycota_spe_Incertae_sedis	0	0	0	0	0	0	0	0	0	337	0	0
Ruhlandiella_patagonica	0	0	0	0	0	0	0	0	0	0	0	0
Russula_xerophila	169020	382	859	95282	613	915	423	644	431	3123	17339	192
Russulales_spe_Incertae_sedis	0	0	0	0	0	0	0	0	0	0	0	0
Saccharomyces_cerevisiae	0	0	143	53	0	0	153	0	0	2079	0	43
Saccharomycopsis_fibuligera	0	0	0	0	0	0	0	0	0	0	0	0
Saprochaete_gigas	0	0	0	0	0	0	0	0	0	0	0	0
Sarocladium_kiliense	0	0	0	0	0	38	0	0	0	345	0	0
Sarocladium_oryzae	0	0	0	0	0	0	0	0	0	0	0	0
Sarocladium_strictum	0	0	0	0	0	0	0	0	0	0	0	0
Sarocladium_zeae	0	0	0	0	0	0	0	0	0	0	0	0
Schizophyllum_commune	0	0	0	0	0	0	0	0	0	0	1088	1584
Scopulariopsis_cordiae	0	0	0	0	0	0	0	0	0	0	0	0
Scytinostroma_caudisporum	0	0	0	0	0	0	0	0	0	0	0	0
Septoria_spe_Incertae_sedis	0	0	0	0	0	0	0	0	0	0	0	0
Setophaeosphaeria_badalingensis	0	0	0	0	0	0	0	0	0	0	0	0
Sidera_vesiculosa	0	0	0	0	0	875	0	0	0	0	0	0
Simplicillium_aogashimaense	0	0	0	0	0	0	0	0	0	0	0	0
Simplicillium_lanosoniveum	0	0	0	0	0	0	0	0	0	0	0	0
Simplicillium_sympodiophorum	2405	56016	912	1862	8464	20963	438	3198	39903	5940	13965	787
Sistotrema_brinkmannii	0	0	0	0	0	0	0	0	0	0	0	0
Sistotremastrum_guttuliferum	0	0	0	0	0	0	0	0	0	0	0	0
Skeletocutis_nivea	0	0	0	0	12541	0	50	0	0	0	0	0
Sordaria_equicola	0	0	0	0	0	0	0	0	0	0	0	0
Sordaria_spe_Incertae_sedis	0	0	0	0	0	0	0	0	0	0	0	0
Spiromastix_princeps	0	0	238	150	0	186	43	0	129	0	0	0
Sporobolomyces_beijingensis	77	0	0	0	0	0	0	0	0	0	7134	0
Sporobolomyces_koalae	0	0	0	0	0	0	0	0	0	0	60	0
Sporobolomyces_patagonicus	0	0	0	0	0	0	0	0	0	0	122	0
Sporobolomyces_phaffii	0	0	0	0	0	0	0	0	0	0	0	0
Sporobolomyces_roseus	0	0	0	0	0	0	0	0	0	0	0	0
Sporothrix_nigrograna	0	0	0	0	0	0	0	0	0	0	0	0
Stachybotrys_aloeticola	0	0	0	0	0	0	0	0	0	0	0	0
Stagonosporopsis_citrulli	0	0	0	0	0	0	0	0	0	0	0	0
Stagonosporopsis_lupini	90	95	0	39112	154	87	0	195	133	367	0	0
Stagonosporopsis_nemophilae	0	0	0	0	0	0	0	0	0	0	0	0
Starmerella_etchellsii	69	41	0	0	0	38	45	0	0	0	0	0
Steccherinum_ochraceum	0	0	0	0	5878	0	0	0	63	0	0	0
Stemphylium_lycopersici	0	0	0	0	0	0	0	132	0	0	13563	37
Stemphylium_spe_Incertae_sedis	0	0	0	0	0	0	0	0	0	0	0	0
Sterigmatomyces_halophilus	0	0	0	0	0	1435	0	0	0	0	0	0
Strelitziana_eucalypti	0	0	0	0	0	0	0	0	0	0	0	0
Strobilurus_spe_Incertae_sedis	0	0	0	0	0	0	0	0	0	0	0	0
Subulicystidium_brachysporum	0	0	0	0	0	0	0	0	0	0	0	0
Symmetrospora_oryzicola	0	0	0	0	0	0	0	0	0	0	0	0
Symmetrospora_spe_Incertae_sedis	0	0	0	0	0	0	0	0	0	0	0	0
Sympodiomycopsis_spe_Incertae_sedis	0	0	0	0	0	0	0	0	0	0	0	0
Talaromyces_calidicanius	0	0	0	0	0	0	0	0	0	0	0	0

Talaromyces_funiculosus	84	0	49	0	0	0	88	144	0	360	132	86
Talaromyces_islandicus	0	37	0	161	80	42	40	0	30940	0	0	0
Talaromyces_rogersiae	140	0	0	80	0	46	0	66	135	324	0	0
Talaromyces_scorteus	217	47	218	87	251	205	0	197	16434	0	38560	513
Talaromyces_stollii	0	0	0	0	0	0	0	0	0	0	0	0
Talaromyces_wortmannii	0	0	0	0	0	0	0	0	0	0	57	0
Tausonia_pullulans	0	0	0	49	0	2769	0	0	0	0	0	35
Tetracladium_spe_Incertae_sedis	0	0	0	0	0	0	0	0	0	0	0	0
Thanatephorus_cucumeris	67	0	0	0	0	0	0	0	0	0	3995	113
Thecaphora_australiensis	0	0	0	0	0	0	0	0	0	0	20567	44
Thelebolus_balaustiformis	0	0	0	0	0	0	0	0	0	0	180	190
Thermomyces_lanuginosus	0	0	0	0	0	0	0	0	0	0	0	0
Thielaviopsis_euricoi	0	0	0	0	0	0	0	0	0	0	0	0
Tilletia_barclayana	0	0	0	0	0	0	0	0	0	0	0	0
Tilletia_geeringii	66	0	0	40	6351	775	44	74	0	350	68	32
Tilletia_maclaganii	0	0	0	0	0	0	0	0	0	0	0	35
Tilletiopsis_washingtonensis	0	0	0	0	0	46	0	0	0	0	67	114
Togniniella_acerosa	0	0	0	0	0	0	37	0	0	0	0	0
Toxicocladosporium_spe_Incertae_sedis	0	0	0	0	0	0	0	0	0	0	0	0
Toxicocladosporium_strelitziae	0	0	0	0	0	0	0	0	0	0	0	0
Trametes_cubensis	0	0	0	0	0	0	0	0	0	0	0	0
Trametes_trogii	0	0	0	0	0	0	0	0	0	0	0	390
Trametes_versicolor	0	0	0	0	0	0	0	0	0	0	11104	225
Trechispora_spe_Incertae_sedis	0	0	0	0	0	0	0	0	0	0	0	0
Tremateia_chromolaenae	0	0	0	0	0	0	0	0	0	0	0	0
Tremateia_murispora	0	0	0	0	0	0	159	0	0	0	0	0
Trichoderma_asperellum	0	0	0	0	0	0	0	0	0	0	0	0
Trichoderma_deliquescens	0	0	0	0	0	0	0	0	0	0	0	0
Trichoderma_effusum	0	0	0	0	0	0	0	0	0	0	0	0
Trichoderma_harzianum	0	0	0	0	0	0	0	0	0	0	0	0
Trichoderma_longibrachiatum	0	0	0	0	0	0	0	0	0	0	0	0
Trichoderma_pleuroticola	0	0	0	0	0	0	0	0	0	0	0	0
Trichomeriaceae_spe_Incertae_sedis	0	0	0	0	0	0	0	0	0	0	0	0
Trichomerium_dioscoreae	0	0	0	0	0	0	0	0	0	0	0	0
Trichomerium_flexuosum	0	0	0	0	0	0	0	0	0	0	5267	0
Trichomonascus_ciferrii	0	0	0	0	0	0	0	0	0	0	0	0
Trichophaea_saccata	0	0	0	0	0	82	0	0	0	0	0	39
Trichosporon_asahii	0	0	0	0	0	0	0	0	0	0	0	0
Trigonopsis_californica	0	0	0	41	0	0	0	0	0	0	0	0
Trigonopsis_vinaria	0	0	0	0	0	0	0	0	0	0	0	0
Tritirachium_cinnamomeum	0	0	0	0	0	0	0	0	0	0	0	0
Tritirachium_oryzae	0	0	0	0	0	0	0	0	0	0	0	0
Tuber_alcaracense	0	0	202	0	0	0	0	0	0	0	0	0
Tubeufia_tratensis	126	0	0	0	0	76	39	0	133	0	0	0
Tulostoma_calcareum	0	0	86	125	0	0	40	67	0	0	0	0
Typhula_micans	64	0	80	130	147	0	41	74	0	1047	0	211
Udeniomyces_megalosporus	0	0	0	0	0	0	0	67	0	0	475	1239
Umbilicaria_maculata	0	0	0	0	0	0	0	0	0	0	0	0
Umthunziomyces_hagahagensis	0	0	0	0	0	0	0	0	0	0	0	0
Ustilaginoidea_virens	0	0	0	0	0	0	0	80	0	0	0	0
Uwebraunia_musae	0	0	0	0	0	0	0	0	0	0	0	0
Valsaria_ceratoniae	0	0	0	39	0	45	0	0	3263	0	0	0
Vandijckella_johannae	0	0	0	0	0	0	0	0	0	0	0	0
Vanrija_musci	0	0	0	0	0	0	0	0	0	0	0	0
Vararia_gallica	0	0	0	0	0	0	0	0	0	0	0	0
Venturiaceae_spe_Incertae_sedis	0	0	0	0	0	0	0	0	0	0	0	0
Verrucaria_oulankaensis	0	0	50	0	0	0	0	0	0	0	0	0
Verrucaria_vitikainenii	0	0	0	0	0	0	0	0	0	0	0	0

Vishniacozyma_heimaeyensis	0	0	0	44	0	0	0	0	0	0	0	0
Vishniacozyma_taibaiensis	0	0	0	0	0	0	0	0	0	0	0	0
Vishniacozyma_tephrensis	0	0	0	0	0	0	0	0	0	0	0	0
Vishniacozyma_victoriae	0	0	0	0	0	0	0	0	0	0	0	0
Wallemia_canadensis	0	0	0	0	0	0	0	133	0	0	0	0
Wallemia_mellicola	0	0	0	0	7608	44	0	198	0	0	1853	0
Wallemia_muriae	0	0	0	0	0	0	0	0	0	0	0	0
Wallemia_sebi	0	0	48	126	0	8824	0	69	0	0	0	0
Wickerhamiella_versatilis	0	0	0	0	0	2649	0	0	0	0	0	0
Wickerhamomyces_onychis	0	0	0	0	0	188	0	0	0	0	0	0
Xenodevriesia_strelitziicola	0	0	0	0	0	0	0	0	0	0	0	0
Xeromyces_bisporus	0	0	0	0	0	0	0	0	0	0	0	0
Xylocoremium_spe_Incertae_sedis	0	0	0	0	0	0	0	0	0	0	0	0
Xylodon_filicinus	0	0	0	0	0	0	0	0	0	0	0	0
Yamadazyma_terventina	0	0	0	0	0	0	0	0	0	0	0	0
Yamadazyma_triangularis	0	0	0	0	0	0	0	0	0	0	0	0
Yarrowia_alimentaria	0	0	0	0	0	0	0	0	0	0	0	0
Yarrowia_bubula	85	84	63	77	72	79	0	128	128	0	0	0
Yarrowia_lipolytica	73	0	0	71	18808	2288	0	0	0	0	0	0
Zanclospora_aurea	75	37	46	0	0	175	0	0	65	0	56	0
Zanclospora_jonesii	10464	28253	9308	41137	478715	318197	5202	12488	199363	314886	178990	7736
Zasmidium_spe_Incertae_sedis	0	0	0	0	0	0	0	0	0	0	0	0
Zeloasperisporium_eucalyptorum	0	0	0	0	0	0	0	0	0	0	0	0
Zoophthora_radicans	601	0	0	41	78	902	0	0	0	0	3356	31
Zygosaccharomyces_rouxii	0	0	0	0	0	142	0	0	0	0	0	0
Zygosporium_pseudogibbum	0	0	0	0	0	0	0	0	0	0	0	0
Zygotorulaspora_danielsina	2861	0	50	1564	0	0	39	0	0	0	0	41
Zymoseptoria_spe_Incertae_sedis	0	0	0	0	0	0	0	0	0	0	0	0
[Candida]_aaseri	0	0	0	0	0	0	0	0	0	0	0	0
[Candida]_boidinii	0	0	0	0	0	0	0	0	0	0	0	0
[Candida]_glabrata	0	0	0	0	0	0	0	0	0	0	0	0
[Candida]_incommunis	0	0	0	0	0	0	0	0	0	0	0	0
[Candida]_pseudolambica	0	0	0	0	0	0	46	0	0	0	0	0
[Cryptococcus]_ater	0	2144	0	0	474	1002	0	0	450	0	846	0

Control020	Control026	Control027	Control028	Control030	Control031	Control033	Control034	Control035	Control036	Control037	Control038	Control039	Control040	Control041	Control042
932004	424256	679023	968561	802055	666714	975672	813445	898341	858595	970017	751249	872681	305170	922863	929052
20581	482767	272341	12017	176815	282157	3299	110092	57343	126667	25943	167261	72020	26024	69914	61823
0	0	0	0	0	0	0	0	0	0	0	0	0	0	0	0
47102	19197	13912	16682	3625	15419	19942	28225	35593	13614	3268	18337	35413	32432	7057	7120
313	73780	34724	2685	17505	35542	1087	48158	8723	1124	772	62708	19886	636374	166	2005
0	0	0	55	0	0	0	0	0	0	0	0	0	0	0	0
0	0	0	0	0	168	0	80	0	0	0	445	0	0	0	0

Control020	Control026	Control027	Control028	Control030	Control031	Control033	Control034	Control035	Control036	Control037	Control038	Control039	Control040	Control041	Control042
0	0	0	0	0	221	231	119	7167	245	106	148	169	107	0	584
82	0	0	0	0	0	0	0	0	0	0	0	0	0	0	0
7535	1281	87407	157461	236828	425571	13646	196494	678338	67504	59169	457539	732134	232080	159746	10828
0	0	0	0	0	0	0	0	0	0	0	0	0	0	0	0
0	0	0	0	0	0	0	0	0	0	0	0	0	0	0	0
0	0	0	0	0	0	0	0	0	0	0	0	0	0	0	0
0	0	0	0	0	0	0	0	0	0	0	0	0	0	0	0
144	0	0	0	0	0	0	0	0	0	0	0	0	0	0	0
0	0	0	0	0	0	0	0	0	0	0	0	0	0	0	0
3755	97	5203	357	635	799	6221	286859	775	1295	20930	14460	7324	1210	774	9305
0	0	0	0	0	0	0	0	0	0	0	0	0	0	0	0
0	0	0	0	0	0	0	0	0	0	0	0	0	0	0	0
0	0	0	0	0	0	0	0	0	0	0	0	0	0	0	0
0	15	3486	1026	287	316	47	0	5633	196	0	0	124	130	0	91
0	0	0	0	0	0	0	0	872	0	0	0	72	0	0	0
0	0	0	0	0	0	0	0	0	0	0	0	0	0	0	0
0	0	0	0	0	0	0	0	0	0	0	0	0	0	0	0
90	14	0	0	0	0	0	0	0	0	0	0	0	0	0	180
0	0	0	0	0	0	0	0	0	0	0	0	28	0	0	0
0	0	0	0	0	0	0	0	0	0	0	0	0	0	0	0
708	0	3467	0	275	0	0	0	0	116	35	31	0	0	0	0
0	0	0	0	0	0	0	0	0	0	0	0	0	0	0	0
44	30	2606	6695	168	2370	10495	30	0	0	0	0	51	247	114	0
0	0	0	0	0	0	0	0	0	0	0	0	0	0	0	0
0	19	2599	0	0	3987	0	13	0	0	0	0	0	0	0	0
2894	46272	112685	178989	261454	53574	316316	307547	16301	108474	1335	67619	1090	6391	120155	19006
0	0	0	0	0	0	0	0	0	0	42	0	0	0	0	0
0	0	0	0	94	0	0	0	0	36	0	39	0	0	44	100
252	0	0	0	0	0	0	0	0	0	0	0	0	0	65	0
0	0	0	0	0	0	0	0	0	0	0	0	0	0	0	0
0	0	0	0	0	0	0	0	0	0	0	0	0	0	0	0
0	0	0	0	0	0	0	0	0	0	0	0	0	0	0	581
0	0	0	0	0	53	0	0	0	0	0	0	0	0	0	0
0	0	0	0	0	0	0	0	0	0	0	332	119	977	0	0
0	0	0	0	0	0	0	0	0	0	0	0	0	0	0	0
53	0	0	0	0	0	0	0	0	0	0	0	0	0	0	0
0	0	0	0	0	0	0	0	0	0	0	0	0	0	0	6501
0	0	0	0	0	0	0	0	2091	0	0	39	33	71	0	0
0	0	0	0	0	0	0	0	0	0	0	0	0	0	0	0
803	1160	91968	514611	9947	29886	562475	794	10974	888	890	4882	498	5278	161764	4906
0	0	0	0	0	0	0	0	0	0	0	0	0	0	59	88
0	225	11363	400	669	120	139	31	96	131	1854	17515	25	69	114	88
0	0	0	0	0	0	0	0	0	0	0	0	0	0	0	0
0	0	0	0	0	0	0	0	0	0	0	0	0	0	0	0
0	0	0	0	0	0	0	0	0	0	0	0	0	0	0	0
0	0	0	0	0	0	0	0	0	0	0	0	0	0	0	0
59	28	1770	281	0	64	0	0	347	0	0	40	299	0	0	0

0	0	0	0	0	111	0	0	0	0	0	0	0	0	0	0
62	0	0	0	0	0	0	0	0	0	0	0	0	0	0	0
427	17	1728	2528	83	6133	574	461	446	167091	248	512	182	523	4247	7890
0	0	0	0	0	163	0	0	0	0	0	0	0	0	0	0
0	0	0	0	0	0	0	0	0	0	0	0	0	0	0	0
0	0	0	0	0	0	0	0	0	0	0	0	0	0	0	0
0	0	0	0	0	0	0	0	0	0	0	0	0	0	0	0
0	70	9543	118	462	0	0	0	0	0	0	0	0	0	0	4899
0	54	1743	65	87	0	0	33	0	0	0	0	8209	55	0	0
0	0	0	0	0	0	0	0	0	0	0	0	0	0	0	8093
0	0	0	0	0	0	0	0	0	0	633	0	0	0	0	0
0	0	0	0	0	0	0	0	0	0	0	0	0	0	0	0
0	0	0	0	0	0	0	0	0	0	0	0	0	0	67	0
0	0	0	0	0	45	0	0	0	0	0	0	0	0	0	0
0	0	0	0	0	0	0	0	0	0	0	0	0	360	0	0
48	66	877	393	0	279	97	139	224	131975	125	1900	99	346	1080	9544
0	0	0	0	0	0	0	0	0	0	0	0	0	0	0	0
0	0	0	0	0	0	0	0	0	0	212	0	0	489	55	0
0	0	0	0	0	0	0	0	0	0	0	0	0	0	0	199
6157	122214	58827	3190	2543	19982	1148	563	12459	1023	4604	108339	295	5526	13882	57638
0	0	0	0	0	0	0	0	0	0	0	0	0	0	168	0
0	0	0	0	0	0	0	0	0	0	477	0	0	0	0	0
684	0	0	0	0	0	0	0	0	0	0	0	0	0	0	0
0	0	0	0	0	0	0	0	1443	0	0	0	0	0	0	0
0	0	0	0	0	0	40	130	0	30	0	75	0	63	0	0
0	0	0	0	0	0	0	0	0	0	0	0	0	0	0	0
0	0	0	0	0	0	0	0	0	0	0	0	0	0	0	0
0	0	0	0	0	0	0	0	0	0	0	0	0	0	0	0
0	0	0	0	0	0	0	0	0	0	0	0	0	0	48	0
0	0	868	56	0	0	0	0	0	0	0	0	0	0	0	0
0	0	886	114	74	0	0	0	0	0	0	0	0	0	0	0
0	0	0	0	0	0	0	0	0	0	0	0	0	0	0	0
0	0	0	0	0	0	0	4819	0	0	0	0	0	0	0	0
2047	245466	187400	5833	8219	9757	490	17443	9060	1282	6439	28286	17297	1669	23168	3048
0	34	0	121	0	0	0	0	3528	0	0	0	0	55	0	0
0	0	0	0	0	0	0	0	0	0	0	0	0	0	0	0
0	0	0	63	0	0	0	0	0	0	0	0	0	252	0	5428
0	0	0	0	0	0	39	31	0	22	0	0	5088	0	0	0
0	0	0	0	0	56	0	0	0	0	0	0	0	0	0	0
1234	0	0	132	0	46	0	30	0	0	1091	0	0	0	0	0
0	0	0	0	0	0	53	0	2010	0	0	0	0	56	0	0
0	0	0	0	0	0	0	0	0	0	0	0	0	0	0	0
0	0	0	0	0	0	0	0	0	0	0	0	0	0	0	0
0	0	0	0	0	112	135	83	98	37	29534	0	24	259	0	0
102	0	0	0	0	0	0	0	0	0	0	0	0	0	0	0
0	0	0	71	0	0	43	0	0	0	0	0	0	0	0	0
0	0	0	0	0	0	0	0	0	0	0	0	0	0	0	0
0	0	0	0	0	125	0	0	0	0	0	0	0	0	0	0
1316	0	0	0	0	10240	193	126	276	250	0	39	46	59	299610	325
4961	40	881	0	0	0	0	0	0	0	46	0	0	0	0	0
0	0	0	0	0	0	0	0	0	0	0	0	0	0	0	0
0	0	0	0	0	0	0	0	0	0	0	0	0	1121	0	0
100	0	0	0	0	0	0	0	0	0	0	0	0	0	0	0
0	0	0	0	0	0	0	0	0	0	0	0	0	0	0	101
0	0	0	0	0	0	0	0	0	0	0	0	0	0	0	0
206	73780	34724	2685	17505	35542	1041	48115	8723	1124	772	62666	19849	635940	166	2005
0	0	0	0	0	0	0	0	0	0	0	0	0	0	0	100
0	0	0	0	0	0	0	0	0	0	0	0	0	0	0	717

0	0	0	0	0	0	0	0	0	0	0	0	0	0	0	0
0	0	0	0	0	0	0	0	0	0	0	0	0	0	0	0
0	0	0	0	0	0	0	0	0	0	0	0	0	0	0	0
0	0	0	0	0	0	0	0	0	0	0	0	0	0	0	0
0	0	0	0	0	0	0	0	0	0	0	0	0	0	0	0
0	0	0	0	0	0	0	0	0	0	0	0	0	0	0	668
0	0	0	0	0	0	0	0	0	0	0	0	0	0	0	0
0	0	0	872	0	0	1549	56	2014	0	0	0	6228	0	0	0
0	0	0	0	0	0	0	0	0	0	0	0	0	0	0	0
0	0	0	143	0	0	332	0	0	0	854	0	0	0	0	0
0	0	0	0	0	0	0	0	0	0	0	0	0	0	0	0
0	0	0	0	0	0	0	0	0	0	0	0	0	0	0	0
0	12	1715	134	98	0	0	0	0	0	0	0	0	0	0	0
0	0	0	0	0	0	0	0	0	0	0	0	0	0	0	0
0	0	0	0	0	0	0	0	0	0	0	0	0	0	0	0
0	20	0	1900	89	87	1050	0	5316	33	45	0	77	60	60	90
0	0	0	173	0	0	49	0	2072	0	0	0	1834	0	0	92
150	0	863	0	0	56	0	35	1250	0	0	0	0	53	50	2986
0	0	0	0	0	0	0	0	0	0	0	0	0	0	0	1237
58	0	0	0	0	0	0	0	0	0	0	39	0	0	0	0
0	0	0	0	0	0	0	0	0	0	307	0	0	0	0	2409
0	0	0	0	0	5298	0	0	0	0	0	0	0	57	0	0
47102	19197	13912	15810	3625	15419	18393	28169	33579	13614	3268	18337	29185	32432	7057	7120
0	177	7749	123	355	51	33	0	2828	52	0	0	0	4819	23381	71
0	19	0	15750	180	614	24938	0	35	0	46	38	49	59	12326	1161
0	0	0	0	0	0	0	0	0	0	0	0	0	0	0	0
0	0	0	0	0	107	0	0	0	0	0	0	0	0	0	0
0	0	0	0	0	0	0	0	0	0	0	0	0	0	0	0
0	812	0	51	0	56	0	215	231	0	0	0	162	7896	0	0
0	0	0	0	0	54	586	0	0	0	0	0	35	0	59	0
0	22	860	223	0	0	0	0	394	0	0	0	529	0	0	0
0	0	0	0	0	0	0	0	0	0	0	0	0	0	0	0
107	0	0	0	0	0	0	0	0	0	0	0	0	0	0	0
0	0	0	0	0	0	0	0	0	0	0	0	0	0	64	6028
0	0	0	0	0	0	43	0	53	60	0	7916	0	74	94	0
0	0	873	0	0	0	0	0	0	0	0	0	0	0	0	0
0	0	877	0	0	0	0	0	0	0	0	0	0	0	0	0
0	0	0	0	0	0	0	0	44	31	0	33	0	143	9209	0
0	0	0	0	0	854	0	0	0	0	0	0	0	0	0	0
0	16	0	56	0	0	0	0	0	0	0	0	0	0	0	0
0	0	0	0	0	0	0	0	0	0	0	0	0	1141	0	0
0	0	873	116	165	48	0	0	256	0	0	0	228	0	0	0
0	0	0	0	0	301	0	0	0	0	0	0	0	0	0	0
0	0	0	0	0	0	0	0	0	0	0	0	0	0	0	0
0	0	0	0	0	0	0	0	0	0	0	0	0	0	0	0
0	0	0	0	0	0	0	0	0	0	0	0	0	0	0	2584
0	0	0	354	0	0	0	0	0	0	961	0	0	0	0	0
0	0	0	0	0	0	0	0	0	0	0	0	0	0	0	0
0	0	0	0	0	0	0	0	0	0	0	0	0	0	0	0
0	0	0	0	0	0	0	0	0	0	0	0	0	0	0	0
0	0	0	0	0	0	0	0	0	0	0	0	0	0	0	0
0	0	1755	0	92	113	44	134	0	139	0	45	30	385	0	3197
0	0	0	0	0	0	0	0	0	0	0	0	0	0	0	0
0	0	0	0	0	0	0	0	0	0	0	0	0	0	0	0
0	97	1724	65	284	0	0	0	0	0	0	0	0	3227	0	0
0	0	0	0	0	0	0	0	0	0	0	0	0	0	0	0
0	0	0	0	0	0	0	0	43	0	0	0	0	134	0	0
0	0	0	0	0	0	0	0	0	0	0	0	0	0	0	0

0	0	0	252	0	0	0	0	44	0	0	0	0	66	0	2855
0	0	0	0	0	0	0	0	0	0	0	0	0	0	0	0
0	0	0	0	0	0	0	0	0	0	0	0	0	0	0	0
0	0	0	0	0	0	0	0	0	0	0	0	0	0	0	0
110	0	0	0	0	0	0	0	0	0	0	0	0	0	0	4219
0	0	0	0	0	0	0	0	0	0	0	0	0	0	0	0
0	0	0	0	0	0	0	0	0	0	0	0	0	0	0	0
0	0	0	0	0	0	0	0	0	0	0	0	0	0	0	0
0	0	0	0	0	0	0	0	0	0	0	0	0	0	45	0
54	0	0	0	0	0	0	0	0	0	0	0	0	0	0	0
0	0	0	0	0	0	0	0	0	0	0	0	0	0	0	0
39	0	0	0	0	0	0	0	0	0	0	0	0	0	0	0
0	0	0	0	0	0	0	0	0	0	0	0	0	0	0	0
0	64	8704	378	571	0	52	0	3601	0	0	0	0	0	106	0
0	36	0	0	0	0	0	0	0	0	0	0	0	0	0	0
0	0	862	57	0	0	0	0	0	0	0	0	0	0	0	0
0	0	0	0	0	0	0	0	0	0	0	0	0	0	0	0
0	0	0	0	0	0	0	0	0	0	0	0	0	0	0	0
0	0	0	0	0	0	0	0	0	0	0	0	0	0	6753	0
0	0	0	0	0	0	0	0	0	0	0	0	0	0	0	0
12707	135383	38999	971	2279	152404	1535	1642	8714	54013	507	16488	7577	4760	17475	18875
0	0	0	0	0	0	0	0	0	0	0	0	0	0	0	0
0	0	0	0	0	0	0	0	0	0	0	0	0	0	0	0
0	0	0	0	0	0	0	0	0	0	0	0	0	0	0	0
0	0	0	0	0	0	0	0	0	0	0	0	0	0	0	0
864764	59	3473	480	807	0	48	0	216	30	0	0	25	0	467	197
0	0	0	0	0	0	0	0	0	0	0	0	0	0	0	0
0	0	0	58	0	0	0	0	140	0	0	0	29	0	0	0
0	0	0	0	0	0	0	0	0	0	0	0	0	0	0	0
0	0	0	0	0	0	0	0	0	0	0	0	0	0	0	0
0	0	0	0	0	0	0	0	0	0	0	0	0	0	0	0
34	0	0	0	0	2789	0	28	0	0	0	0	27	0	0	0
503	0	0	68	0	0	0	65	0	60	0	0	9054	112	48	0
0	0	0	0	0	0	0	0	0	0	0	0	0	0	0	0
0	20	0	7633	92	60	9622	28	0	0	0	0	0	0	712	0
0	0	0	0	0	0	0	7179	48	34	0	0	0	0	0	0
0	0	0	0	0	0	0	0	0	0	0	0	0	0	0	0
0	0	0	0	0	0	0	0	0	0	0	0	0	0	53	12233
0	21	0	141	85	0	0	0	0	0	0	0	0	0	0	0
0	0	0	0	0	0	0	0	0	0	0	0	0	0	0	0
0	0	0	0	0	0	0	0	0	0	0	0	0	0	0	0
0	0	0	0	0	191	0	0	0	0	0	0	0	0	0	0
0	0	0	0	0	0	0	0	0	0	0	0	0	0	0	0
46	0	0	0	0	0	0	0	0	0	0	0	0	0	0	0
57	0	0	0	0	0	0	0	0	0	0	0	0	0	0	12183
0	0	0	0	0	0	0	0	0	0	0	0	0	0	0	0
0	42481	4314	241	92	123	0	0	0	0	0	0	44	66	0	98
0	36	2591	114	181	0	0	0	0	0	453	0	0	0	0	0
0	0	0	0	0	0	0	0	0	0	0	0	0	0	0	275
0	0	0	0	0	0	0	0	0	0	0	0	0	0	0	0
0	0	0	0	0	0	0	0	0	0	0	0	0	0	0	0
0	0	0	0	0	1155	0	0	0	0	0	0	0	0	0	0
0	0	0	0	0	0	0	0	0	0	0	0	0	0	0	0
97	0	0	60	0	58	0	0	45	0	0	0	209	0	262	291398
0	0	0	0	0	0	143	35	19638	67	0	0	0	63	0	0
0	0	0	0	0	0	0	0	0	0	0	0	0	0	0	0
104	167	861	2434	177	110	190	64	7317	2190	37	126	4879	142	58	0
0	0	0	0	0	0	0	0	0	0	0	0	0	0	0	0

0	0	0	0	0	0	0	0	659	0	0	0	0	0	0	0
0	0	0	0	0	121	0	0	0	0	0	0	0	0	0	0
0	0	0	0	0	0	0	0	0	0	0	0	0	0	0	0
0	0	885	4846	81	157	6040	0	0	0	0	0	0	0	663	0
0	0	0	0	0	0	0	0	0	0	0	0	0	0	0	0
0	0	0	0	0	0	0	0	0	0	0	0	0	0	0	0
0	0	0	0	0	0	0	0	0	0	0	0	0	0	0	0
90	0	0	0	0	117	96	0	0	0	0	0	0	0	359	880
0	53	6903	53	116	16251	36	33	57	27	0	37	6429	145	0	0
0	0	0	0	0	0	0	0	0	0	0	0	0	0	0	0
47	15	0	1138	90	0	187	0	5840	65	82	22199	916	263	0	106
0	0	0	0	0	0	0	72	0	70	42	45	0	76	0	0
0	0	0	0	0	0	0	0	0	0	0	0	0	0	0	0
0	0	0	0	0	0	0	0	0	0	0	0	0	0	0	0
0	0	0	0	0	0	0	0	0	0	0	0	0	0	0	0
0	0	0	0	0	0	0	0	0	0	0	0	0	0	0	0
0	0	0	0	0	0	0	0	0	0	0	0	0	0	0	179
0	157580	19050	918	1312	442	530	574	371	253429	289	342	310	1290	0	0
0	0	0	0	0	0	0	0	0	0	0	0	0	0	0	0
0	0	0	0	0	0	0	0	411	0	0	0	0	0	0	465
0	0	0	0	0	8692	50	38	0	37	38	74	10131	0	0	0
0	0	0	673	0	44	0	0	59	87	174	0	11718	0	0	0
0	0	0	0	0	0	0	0	0	0	0	0	0	0	0	0
0	0	0	0	0	0	0	0	0	0	0	0	0	0	0	0
0	0	0	0	3139	0	0	0	0	0	0	0	0	0	0	0
0	0	0	0	0	107	130	74	40	59727	23	110	0	70	0	0
0	0	0	0	0	0	0	0	0	0	0	0	0	0	0	0
0	0	0	0	0	66	0	0	0	0	0	0	0	0	68	6104
0	0	0	0	0	0	0	0	0	0	0	0	0	0	9624	0
0	0	0	0	0	0	0	0	0	0	0	0	0	0	0	0
0	0	0	0	0	56	88	0	54	0	47	14514	30	0	0	0
0	0	0	0	0	0	0	0	0	0	0	0	0	0	0	0
0	0	0	0	0	0	0	0	5021	0	0	47	0	0	0	0
0	0	0	0	0	0	0	0	0	0	0	0	0	0	52	0
0	0	0	0	0	0	0	25	0	0	0	0	0	127	0	0
0	0	0	0	0	51	0	0	0	0	0	0	0	0	0	0
0	0	0	0	0	0	0	0	0	0	0	0	0	0	0	0
0	0	0	0	0	302	0	0	0	0	0	0	0	0	0	0
0	0	0	0	0	0	0	0	0	0	0	0	0	0	0	0
0	0	0	0	0	0	0	0	0	0	457	0	0	0	0	0
0	0	0	0	0	0	0	0	0	0	0	0	0	0	0	0
0	0	0	0	0	0	0	0	0	0	0	0	0	0	0	0
0	0	0	0	0	151	97	53	129	84	98	259	46	75	59	102
0	0	0	0	0	0	0	0	0	0	0	0	0	0	0	0
0	0	0	0	0	105	0	0	0	0	0	0	0	0	54	188
0	0	0	0	0	0	0	0	0	0	0	0	0	0	0	0
110	0	0	0	0	0	0	0	0	0	0	0	0	0	0	0
0	0	0	0	0	0	0	0	0	0	0	0	0	0	0	0
189	62	6940	39528	0	129	2424	35	12637	70	0	115	3394	69	107	2706
0	0	0	0	0	0	0	0	0	0	0	0	0	0	0	757
0	0	0	0	0	0	0	0	0	0	0	0	0	0	0	0
0	0	0	0	0	887	0	0	0	0	0	0	0	0	0	0
0	0	0	3913	296	1896	2157	0	187	21	0	33	0	0	37566	6746
0	0	0	0	0	0	0	0	0	0	0	0	0	0	0	579
0	0	0	0	0	0	46	43	0	0	0	42	37	74	0	0
0	0	0	0	0	0	0	0	0	0	0	0	0	0	0	0
0	0	0	68	0	0	0	0	0	0	0	0	0	0	12707	0
0	0	0	0	0	0	0	42	43	0	44	43	0	0	0	0

0	0	0	0	0	0	0	0	0	0	0	0	0	0	0	0
0	0	868	113	104	0	0	0	0	0	0	0	0	0	0	2814
0	0	0	55	0	0	0	0	0	0	0	0	0	0	0	0
0	0	0	0	0	0	0	0	0	0	0	0	0	0	0	5067
0	66	4366	240	164740	78416	401	50056	302	438	312	88031	90	2665	210	1112
0	18	850	0	0	0	0	0	0	0	0	0	0	0	0	0
0	0	883	132	519	336	0	70	0	22	75	0	2127	3676	56	0
0	0	0	0	0	0	420	0	0	0	0	0	0	0	0	0
161	0	0	137	0	0	0	0	0	0	0	0	0	0	0	0
0	0	0	0	0	0	0	0	0	0	0	0	0	0	0	0
870	0	878	0	114	0	0	0	0	0	0	0	0	0	0	0
0	0	0	0	0	0	0	0	0	0	0	0	0	0	0	0
0	0	0	0	0	0	0	0	0	0	0	0	0	0	0	0
0	61	3464	0	0	0	0	0	0	0	0	0	0	0	0	0
0	49	5223	376	484	0	0	0	0	0	0	0	0	0	0	0
0	0	0	0	0	0	0	0	0	0	0	0	0	0	0	0
686	132	5228	9554	748	1574	1648	675	61404	349	84	1946	74764	341	538	872
0	0	0	0	0	0	0	0	0	0	0	0	0	261	0	0
0	0	0	0	0	0	0	0	0	0	0	0	0	0	0	0
0	0	0	0	0	0	0	0	0	0	0	0	0	0	0	0
1127	0	0	0	0	0	0	0	0	0	0	0	0	0	0	0
0	0	0	0	0	0	0	0	0	0	0	0	0	0	0	0
0	48200	10440	289	176	8754	0	35	0	0	44	0	0	69	0	0
0	0	0	0	0	39	0	0	0	906	0	0	0	0	0	0
0	0	0	0	0	0	0	0	0	0	0	0	0	0	0	0
0	56	5205	49	179	0	54	0	3681	0	42	0	0	51	0	0
0	0	0	0	0	0	0	0	0	34	0	0	0	0	0	0
0	0	0	0	0	0	0	0	0	0	0	0	0	0	0	0
52	0	0	0	0	5580	0	0	0	0	0	0	0	0	0	0
0	0	0	0	0	0	96	112	0	90	0	25660	0	63	0	93
0	0	0	0	0	0	0	0	0	0	0	0	0	0	0	0
0	0	0	0	0	0	0	0	0	0	0	0	0	0	0	1319
0	0	0	0	0	0	0	0	0	0	0	0	0	0	0	0
0	0	0	0	0	52	50	34	0	70277	121	73	56	187	0	0
0	0	0	0	0	0	0	0	0	0	0	0	0	0	0	0
11763	32	3446	50	82	292	163	41	174	202	1286	79	145	59	103	112119
1297	0	0	2901	0	0	0	83	67	0	15301	47	25	66	0	0
0	0	0	0	0	0	0	0	0	0	0	0	0	0	0	0
0	0	0	0	0	0	0	0	0	0	0	0	0	0	0	0
0	0	0	0	0	0	0	0	0	0	0	0	0	0	0	0
0	0	0	0	0	0	0	0	0	0	0	0	0	0	0	0
0	0	0	0	0	0	0	0	39	28	0	48	0	67	0	0
0	0	0	0	0	0	0	0	0	0	0	0	0	0	0	0
172	0	0	0	0	0	0	0	0	0	0	0	0	0	0	0
109	0	0	0	0	0	41	0	0	39	50	0	5798	117	0	0
0	0	0	2028	91	0	5540	0	0	34	0	0	0	0	256	0
0	0	0	0	0	0	0	0	0	0	0	0	0	0	0	5425
252	62	9497	107	254	0	0	0	2445	38	0	0	0	0	0	2231
0	0	0	0	0	0	0	0	0	0	0	36	0	479	8885	0
0	0	0	0	0	0	0	0	0	0	0	0	0	0	0	0
0	0	0	0	0	0	0	0	0	0	0	0	0	0	4935	0
0	0	0	0	0	0	0	0	0	0	0	0	0	0	0	0
0	0	0	0	0	0	0	0	0	0	0	0	0	0	0	308
0	0	0	0	0	0	0	0	0	0	0	0	0	0	56	398
0	0	0	0	0	0	0	0	0	0	0	0	0	0	0	98
0	0	0	0	0	0	0	0	0	0	0	0	0	0	0	2984
0	0	0	0	0	1275	1670	840	908	1783	882	1658	556	1814	0	0
0	0	0	0	0	0	0	0	0	0	0	0	0	0	0	0

0	0	0	75	0	0	88	0	0	0	0	0	0	0	0	0
0	0	0	0	0	0	0	0	0	0	0	0	0	0	0	0
0	0	0	0	0	0	0	0	0	0	0	0	0	0	0	0
44	0	0	0	0	0	0	0	0	0	0	0	0	0	0	0
440	0	0	0	0	0	50	11851	0	67	49	35	0	10157	0	0
0	0	0	0	0	0	0	0	0	0	0	0	0	0	0	0
0	0	0	0	0	0	0	0	0	0	0	0	0	0	0	0
0	0	0	0	0	0	0	0	0	0	0	0	0	0	0	0
0	0	0	0	0	0	0	0	0	0	0	0	0	0	0	0
0	0	0	0	0	0	0	0	0	0	0	0	0	0	0	0
0	0	0	0	0	0	0	0	0	0	0	0	0	0	0	0
0	0	0	0	0	0	0	0	0	0	0	0	0	0	0	0
0	0	0	0	0	0	0	0	0	0	0	0	0	0	0	0
0	0	0	0	0	0	0	0	0	0	0	0	0	0	0	0
0	172	13035	711	446	0	35	0	0	0	0	0	0	0	0	1641
0	0	0	0	0	60	224	23717	0	150	48	39	23	0	298	197
0	53284	6060	284	381	72	52	0	1163	0	0	82	0	256	0	4275
0	0	0	505	0	0	0	0	0	0	0	0	0	0	0	0
0	0	0	0	0	0	0	0	0	0	0	0	0	0	0	0
0	0	0	0	0	0	0	0	0	0	0	0	0	0	0	0
0	0	0	0	0	0	0	0	0	0	0	0	0	0	0	0
0	0	0	0	0	0	0	0	0	0	0	0	0	0	0	0
0	0	0	0	0	0	0	0	0	0	0	0	0	0	0	0
0	0	0	0	0	0	0	0	0	0	0	0	0	0	0	0
0	0	0	0	506	0	0	26	18944	41	0	0	0	0	0	0
22896	50637	185743	7411	274312	93709	5517	5490	36626	59843	845392	34000	30149	28331	59799	318014
0	0	0	0	0	0	0	0	0	0	0	0	0	0	0	0
0	0	0	0	0	0	0	0	0	0	0	0	0	0	0	0
0	0	0	0	0	168	0	80	0	0	0	445	0	0	0	0
0	0	0	0	0	0	0	0	0	0	0	0	0	0	0	0
0	0	0	0	0	0	0	0	0	0	0	0	0	0	0	0
0	0	0	0	3328	599	40	4391	0	0	0	2478	0	130	0	0
0	0	0	0	0	0	0	0	0	0	0	0	0	0	0	0

Control020	Control026	Control027	Control028	Control030	Control031	Control033	Control034	Control035	Control036	Control037	Control038	Control039	Control040	Control041	Control042
0	0	0	0	0	0	0	0	0	0	0	0	0	0	0	0
0	0	0	0	0	156	183	97	173	245	77	148	98	50	0	0
0	0	0	0	0	0	0	0	0	0	0	0	0	0	0	0
0	0	0	0	0	0	0	0	0	0	0	0	0	0	0	0
0	0	0	0	0	65	48	22	6994	0	29	0	71	57	0	0
0	0	0	0	0	0	0	0	0	0	0	0	0	0	0	584
82	0	0	0	0	0	0	0	0	0	0	0	0	0	0	0
7535	1281	87407	157461	236828	425571	13646	196494	678338	67504	59169	457539	732134	232080	159746	10828
0	0	0	0	0	0	0	0	0	0	0	0	0	0	0	0
0	0	0	0	0	0	0	0	0	0	0	0	0	0	0	0
0	0	0	0	0	0	0	0	0	0	0	0	0	0	0	0
0	0	0	0	0	0	0	0	0	0	0	0	0	0	0	0
144	0	0	0	0	0	0	0	0	0	0	0	0	0	0	0
0	0	0	0	0	0	0	0	0	0	0	0	0	0	0	0
3755	97	5203	357	635	799	6221	286859	775	1295	20930	14460	7324	1210	774	9305
0	0	0	0	0	0	0	0	0	0	0	0	0	0	0	0
0	0	0	0	0	0	0	0	0	0	0	0	0	0	0	0
0	0	0	0	0	0	0	0	0	0	0	0	0	0	0	0
0	15	3486	1026	287	316	47	0	5633	196	0	0	124	130	0	91
0	0	0	0	0	0	0	0	872	0	0	0	72	0	0	0
0	0	0	0	0	0	0	0	0	0	0	0	0	0	0	0
0	0	0	0	0	0	0	0	0	0	0	0	0	0	0	0
0	0	0	0	0	0	0	0	0	0	0	0	0	0	0	0

90	14	0	0	0	0	0	0	0	0	0	0	0	0	0	180
0	0	0	0	0	0	0	0	0	0	0	0	28	0	0	0
0	0	0	0	0	0	0	0	0	0	0	0	0	0	0	0
708	0	3467	0	275	0	0	0	0	116	35	31	0	0	0	0
0	0	0	0	0	0	0	0	0	0	0	0	0	0	0	0
44	30	2606	6695	168	2370	10495	30	0	0	0	0	51	247	114	0
0	0	0	0	0	0	0	0	0	0	0	0	0	0	0	0
0	19	2599	0	0	3987	0	13	0	0	0	0	0	0	0	0
0	0	0	0	0	0	0	0	0	0	0	0	0	0	0	0
165	62	4347	119	256	0	1027	0	0	0	0	0	0	332	373	4530
156	0	0	0	0	0	0	0	0	0	0	0	0	0	0	0
0	0	0	0	0	0	0	0	0	0	0	0	0	0	0	0
0	0	0	0	0	0	0	0	0	0	0	0	0	0	0	0
0	0	0	0	0	0	0	0	0	0	0	0	0	0	0	0
0	0	0	0	0	0	0	0	0	0	0	0	0	0	5363	120
0	0	0	0	0	0	663	0	0	0	0	0	0	0	0	0
0	0	0	0	0	0	0	0	0	0	0	0	0	0	0	2072
0	0	0	0	0	0	0	0	0	0	0	0	0	0	0	0
0	0	0	0	0	0	0	0	0	0	0	0	0	0	0	0
0	0	1728	0	0	0	0	0	0	0	0	0	0	0	0	0
802	33881	5193	162	489	3209	179	533	1103	107353	297	163	46	477	101	6997
0	0	0	0	0	0	0	0	495	0	0	0	0	0	0	0
814	11023	855	0	90	819	0	0	0	0	0	0	0	0	0	1726
957	1266	99691	178708	260619	49410	314447	307014	13549	1121	919	67456	1044	5524	114259	3356
0	0	0	0	0	0	0	0	0	0	0	0	0	0	0	205
0	0	0	0	0	0	0	0	0	0	0	0	0	0	0	0
0	12	0	0	0	136	0	0	0	0	119	0	0	0	0	0
0	28	871	0	0	0	0	0	1154	0	0	0	0	0	59	0
0	0	0	0	0	0	0	0	0	0	0	0	0	58	0	0
0	0	0	0	0	0	0	0	0	0	0	0	0	0	0	0
0	0	0	0	0	0	0	0	0	0	42	0	0	0	0	0
0	0	0	0	94	0	0	0	0	36	0	39	0	0	0	0
0	0	0	0	0	0	0	0	0	0	0	0	0	0	44	100
252	0	0	0	0	0	0	0	0	0	0	0	0	0	0	0
0	0	0	0	0	0	0	0	0	0	0	0	0	0	65	0
0	0	0	0	0	0	0	0	0	0	0	0	0	0	0	0
0	0	0	0	0	0	0	0	0	0	0	0	0	0	0	0
0	0	0	0	0	0	0	0	0	0	0	0	0	0	0	581
0	0	0	0	0	53	0	0	0	0	0	0	0	0	0	0
0	0	0	0	0	0	0	0	0	0	0	332	119	977	0	0
0	0	0	0	0	0	0	0	0	0	0	0	0	0	0	0
0	0	0	0	0	0	0	0	0	0	0	0	0	0	0	0
53	0	0	0	0	0	0	0	0	0	0	0	0	0	0	0
0	0	0	0	0	0	0	0	0	0	0	0	0	0	0	6501
0	0	0	0	0	0	0	0	2091	0	0	39	33	71	0	0
0	0	0	0	0	0	0	0	0	0	0	0	0	0	0	0
0	0	0	0	0	0	0	0	0	0	0	0	0	0	0	0
0	0	0	0	0	0	42	0	0	0	0	0	0	0	0	0
803	1160	91968	514611	9947	29886	562433	794	10974	888	890	4882	498	5278	161764	4906
0	0	0	0	0	0	0	0	0	0	0	0	0	0	59	88
0	0	0	0	0	0	0	0	0	0	0	0	0	0	0	0
0	0	0	0	0	0	0	0	0	0	0	0	0	0	0	0
0	225	11363	400	669	120	139	31	96	131	1854	17515	25	69	114	88
0	0	0	0	0	0	0	0	0	0	0	0	0	0	0	0
0	0	0	0	0	0	0	0	0	0	0	0	0	0	0	0
0	0	0	0	0	0	0	0	0	0	0	0	0	0	0	0
0	0	0	0	0	0	0	0	0	0	0	0	0	0	0	0
0	0	0	0	0	0	0	0	0	0	0	0	0	0	0	0

59	28	1770	281	0	64	0	0	347	0	0	40	299	0	0	0
0	0	0	0	0	111	0	0	0	0	0	0	0	0	0	0
62	0	0	0	0	0	0	0	0	0	0	0	0	0	0	0
49	0	864	2528	0	0	0	36	0	0	0	280	0	0	173	0
0	0	0	0	0	0	0	0	0	0	0	0	0	0	0	0
0	0	0	0	0	0	0	0	0	0	0	0	0	0	0	0
0	0	0	0	0	0	0	0	0	0	0	0	0	0	0	0
0	0	0	0	0	0	0	0	0	0	0	0	0	0	0	0
0	0	0	0	0	0	0	0	0	0	0	0	0	0	0	0
87	0	0	0	0	299	40	123	0	90	86	39	0	137	118	321
0	0	0	0	0	503	0	0	0	0	0	0	0	0	0	0
0	0	0	0	0	0	0	0	0	0	0	0	0	0	0	0
0	0	0	0	0	0	0	0	0	0	0	0	0	0	0	0
291	17	864	0	0	4749	0	38	0	0	0	0	0	70	0	7093
0	0	0	0	0	224	534	264	446	166974	162	193	182	316	0	0
0	0	0	0	0	263	0	0	0	0	0	0	0	0	0	389
0	0	0	0	83	95	0	0	0	27	0	0	0	0	0	0
0	0	0	0	0	163	0	0	0	0	0	0	0	0	0	0
0	0	0	0	0	0	0	0	0	0	0	0	0	0	0	0
0	0	0	0	0	0	0	0	0	0	0	0	0	0	0	0
0	0	0	0	0	0	0	0	0	0	0	0	0	0	0	0
0	0	0	0	0	0	0	0	0	0	0	0	0	0	0	0
0	70	9543	118	462	0	0	0	0	0	0	0	0	0	0	4899
0	54	1743	65	87	0	0	33	0	0	0	0	8209	55	0	0
0	0	0	0	0	0	0	0	0	0	0	0	0	0	0	0
0	0	0	0	0	0	0	0	0	0	0	0	0	0	0	8093
0	0	0	0	0	0	0	0	0	0	633	0	0	0	0	0
0	0	0	0	0	0	0	0	0	0	0	0	0	0	0	0
0	0	0	0	0	0	0	0	0	0	0	0	0	0	67	0
0	0	0	0	0	45	0	0	0	0	0	0	0	0	0	0
0	0	0	0	0	0	0	0	0	0	0	0	0	360	0	0
48	66	877	393	0	279	97	139	224	131975	125	1900	99	346	1080	9544
0	0	0	0	0	0	0	0	0	0	0	0	0	0	0	0
0	0	0	0	0	0	0	0	0	0	212	0	0	489	55	0
0	0	0	0	0	0	0	0	0	0	0	0	0	0	0	199
849	9157	9530	662	453	12711	474	203	1503	450	319	44768	87	802	57	5495
0	36	3493	65	0	0	0	0	0	0	0	200	0	0	0	0
2392	0	0	0	0	64	153	85	5217	205	1577	19805	103	61	0	12331
459	111817	28460	1029	1309	5909	85	59	303	193	2635	8122	53	3152	219	36184
2457	1204	17344	1434	781	275	400	70	5436	35	34	1164	25	1511	50	3538
0	0	0	0	0	1023	36	146	0	140	39	34280	27	0	13556	90
0	0	0	0	0	0	0	0	0	0	0	0	0	0	168	0
0	0	0	0	0	0	0	0	0	0	477	0	0	0	0	0
0	0	0	0	0	0	0	0	0	0	0	0	0	0	0	0
684	0	0	0	0	0	0	0	0	0	0	0	0	0	0	0
0	0	0	0	0	0	0	0	1443	0	0	0	0	0	0	0
0	0	0	0	0	0	40	130	0	30	0	75	0	63	0	0
0	0	0	0	0	0	0	0	0	0	0	0	0	0	0	0
0	0	0	0	0	0	0	0	0	0	0	0	0	0	0	0
0	0	0	0	0	0	0	0	0	0	0	0	0	0	0	0
0	0	0	0	0	0	0	0	0	0	0	0	0	0	0	0
0	0	0	0	0	0	0	0	0	0	0	0	0	0	48	0
0	0	0	0	0	0	0	0	0	0	0	0	0	0	0	0
0	0	0	0	0	0	0	0	0	0	0	0	0	0	0	0
0	0	868	56	0	0	0	0	0	0	0	0	0	0	0	0
0	0	0	0	0	0	0	0	0	0	0	0	0	0	0	0
0	0	886	114	74	0	0	0	0	0	0	0	0	0	0	0
0	0	0	0	0	0	0	0	0	0	0	0	0	0	0	0

0	0	0	0	0	0	0	4819	0	0	0	0	0	0	0	0
2047	245466	187400	5833	8219	9757	490	17443	9060	1282	6439	28286	17297	1669	23168	3048
0	0	0	0	0	0	0	0	0	0	0	0	0	0	0	0
0	0	0	0	0	0	0	0	0	0	0	0	0	0	0	0
0	34	0	121	0	0	0	0	3528	0	0	0	0	55	0	0
0	0	0	0	0	0	0	0	0	0	0	0	0	0	0	0
0	0	0	0	0	0	0	0	0	0	0	0	0	0	0	0
0	0	0	0	0	0	0	0	0	0	0	0	0	63	0	0
0	0	0	63	0	0	0	0	0	0	0	0	0	189	0	5428
0	0	0	0	0	0	39	31	0	22	0	0	5088	0	0	0
0	0	0	0	0	0	0	0	0	0	0	0	0	0	0	0
0	0	0	0	0	56	0	0	0	0	0	0	0	0	0	0
0	0	0	0	0	0	0	0	0	0	0	0	0	0	0	0
1041	0	0	0	0	46	0	30	0	0	1091	0	0	0	0	0
193	0	0	132	0	0	0	0	0	0	0	0	0	0	0	0
0	0	0	0	0	0	53	0	2010	0	0	0	0	56	0	0
0	0	0	0	0	0	0	0	0	0	0	0	0	0	0	0
0	0	0	0	0	0	0	0	0	0	0	0	0	0	0	0
0	0	0	0	0	0	0	0	0	0	0	0	0	0	0	0
0	0	0	0	0	112	135	83	98	37	29534	0	24	259	0	0
102	0	0	0	0	0	0	0	0	0	0	0	0	0	0	0
0	0	0	71	0	0	43	0	0	0	0	0	0	0	0	0
0	0	0	0	0	0	0	0	0	0	0	0	0	0	0	0
0	0	0	0	0	125	0	0	0	0	0	0	0	0	0	0
0	0	0	0	0	10240	193	126	276	250	0	39	46	59	0	0
0	0	0	0	0	0	0	0	0	0	0	0	0	0	0	0
0	0	0	0	0	0	0	0	0	0	0	0	0	0	0	0
1124	0	0	0	0	0	0	0	0	0	0	0	0	0	20402	0
192	0	0	0	0	0	0	0	0	0	0	0	0	0	279208	325
0	0	0	0	0	0	0	0	0	0	0	0	0	0	0	0
0	0	0	0	0	0	0	0	0	0	0	0	0	0	0	0
0	0	0	0	0	0	0	0	0	0	0	0	0	0	0	0
42	0	0	0	0	0	0	0	0	0	46	0	0	0	0	0
4919	40	881	0	0	0	0	0	0	0	0	0	0	0	0	0
0	0	0	0	0	0	0	0	0	0	0	0	0	0	0	0
0	0	0	0	0	0	0	0	0	0	0	0	0	1121	0	0
0	0	0	0	0	0	0	0	0	0	0	0	0	0	0	0
100	0	0	0	0	0	0	0	0	0	0	0	0	0	0	0
0	0	0	0	0	0	0	0	0	0	0	0	0	0	0	101
0	0	0	0	0	0	0	0	0	0	0	0	0	0	0	0
0	0	0	0	0	0	0	0	0	0	0	0	0	0	0	0
0	0	0	0	0	0	0	0	0	0	0	0	0	0	0	0
206	73780	34724	2685	17505	35542	1041	48115	8723	1124	772	62666	19849	635940	166	2005
0	0	0	0	0	0	0	0	0	0	0	0	0	0	0	0
0	0	0	0	0	0	0	0	0	0	0	0	0	0	0	100
0	0	0	0	0	0	0	0	0	0	0	0	0	0	0	717
0	0	0	0	0	0	0	0	0	0	0	0	0	0	0	0
0	0	0	0	0	0	0	0	0	0	0	0	0	0	0	0
0	0	0	0	0	0	0	0	0	0	0	0	0	0	0	0
0	0	0	0	0	0	0	0	0	0	0	0	0	0	0	0
0	0	0	0	0	0	0	0	0	0	0	0	0	0	0	0
0	0	0	0	0	0	0	0	0	0	0	0	0	0	0	668
0	0	0	0	0	0	0	0	0	0	0	0	0	0	0	0
0	0	0	812	0	0	1549	0	0	0	0	0	65	0	0	0
0	0	0	60	0	0	0	56	2014	0	0	0	6163	0	0	0
0	0	0	0	0	0	0	0	0	0	0	0	0	0	0	0
0	0	0	0	0	0	0	0	0	0	0	0	0	0	0	0
0	0	0	0	0	0	0	0	0	0	0	0	0	0	0	0

0	0	0	143	0	0	332	0	0	0	854	0	0	0	0	0
0	0	0	0	0	0	0	0	0	0	0	0	0	0	0	0
0	0	0	0	0	0	0	0	0	0	0	0	0	0	0	0
0	12	1715	134	98	0	0	0	0	0	0	0	0	0	0	0
0	0	0	0	0	0	0	0	0	0	0	0	0	0	0	0
0	0	0	0	0	0	0	0	0	0	0	0	0	0	0	0
0	20	0	1900	89	87	1050	0	5316	33	45	0	77	60	60	90
47	0	863	0	0	56	0	35	1250	0	0	0	0	53	0	2802
103	0	0	0	0	0	0	0	0	0	0	0	0	0	50	184
0	0	0	0	0	0	0	0	0	0	0	0	0	0	0	1237
58	0	0	0	0	0	0	0	0	0	0	39	0	0	0	0
0	0	0	0	0	0	0	0	0	0	0	0	0	0	0	0
0	0	0	0	0	0	0	0	0	0	307	0	0	0	0	2409
0	0	0	0	0	5298	0	0	0	0	0	0	0	57	0	0
47102	19197	13912	15810	3625	15419	18393	28169	33579	13614	3268	18337	29185	32432	7057	7120
0	0	0	0	0	0	0	0	1197	0	0	0	0	0	6280	0
0	0	0	0	0	51	33	0	1631	52	0	0	0	4819	17101	71
0	0	0	0	0	0	0	0	0	0	0	0	0	0	0	0
0	0	0	0	0	0	0	0	0	0	0	0	0	0	0	0
0	177	7749	123	355	0	0	0	0	0	0	0	0	0	0	0
0	0	0	0	0	0	0	0	0	0	0	0	0	0	0	0
0	19	0	15750	180	614	24938	0	35	0	46	38	49	59	12326	1161
0	0	0	0	0	0	0	0	0	0	0	0	0	0	0	0
0	0	0	0	0	107	0	0	0	0	0	0	0	0	0	0
0	0	0	0	0	0	0	0	0	0	0	0	0	0	0	0
0	812	0	51	0	56	0	215	231	0	0	0	162	7896	0	0
0	0	0	0	0	0	0	0	0	0	0	0	0	0	0	0
0	0	0	0	0	54	586	0	0	0	0	0	35	0	59	0
0	22	860	223	0	0	0	0	394	0	0	0	529	0	0	0
0	0	0	0	0	0	0	0	0	0	0	0	0	0	0	0
107	0	0	0	0	0	0	0	0	0	0	0	0	0	0	0
0	0	0	0	0	0	0	0	0	0	0	0	0	0	64	6028
0	0	0	0	0	0	0	0	0	0	0	0	0	0	0	0
0	0	0	0	0	0	43	0	53	60	0	7916	0	74	94	0
0	0	873	0	0	0	0	0	0	0	0	0	0	0	0	0
0	0	877	0	0	0	0	0	0	0	0	0	0	0	0	0
0	0	0	0	0	0	0	0	44	31	0	33	0	143	9209	0
0	0	0	0	0	0	0	0	0	0	0	0	0	0	0	0
0	0	0	0	0	0	0	0	0	0	0	0	0	0	0	0
0	0	0	0	0	854	0	0	0	0	0	0	0	0	0	0
0	0	0	0	0	0	0	0	0	0	0	0	0	0	0	0
0	0	0	0	0	0	0	0	0	0	0	0	0	0	0	0
0	16	0	56	0	0	0	0	0	0	0	0	0	0	0	0
0	0	0	0	0	0	0	0	0	0	0	0	0	1141	0	0
0	0	873	116	165	48	0	0	256	0	0	0	228	0	0	0
0	0	0	0	0	301	0	0	0	0	0	0	0	0	0	0
0	0	0	0	0	0	0	0	0	0	0	0	0	0	0	0
0	0	0	0	0	0	0	0	0	0	0	0	0	0	0	0
0	0	0	0	0	0	0	0	0	0	0	0	0	0	0	2584
0	0	0	354	0	0	0	0	0	0	961	0	0	0	0	0
0	0	0	0	0	0	0	0	0	0	0	0	0	0	0	0
0	0	0	0	0	0	0	0	0	0	0	0	0	0	0	0
0	0	0	0	0	0	0	0	0	0	0	0	0	0	0	0
0	0	0	0	0	0	0	0	0	0	0	0	0	0	0	0
0	0	1755	0	92	113	44	134	0	139	0	45	30	385	0	3197
0	0	0	0	0	0	0	0	0	0	0	0	0	0	0	0
0	0	0	0	0	0	0	0	0	0	0	0	0	0	0	0
0	97	1724	65	284	0	0	0	0	0	0	0	0	3227	0	0

0	0	0	0	0	0	0	0	0	0	0	0	0	0	0	0
0	0	0	0	0	0	0	0	43	0	0	0	0	134	0	0
0	0	0	0	0	0	0	0	0	0	0	0	0	0	0	0
0	0	0	0	0	0	0	0	0	0	0	0	0	0	0	0
0	0	0	252	0	0	0	0	44	0	0	0	0	66	0	2855
0	0	0	0	0	0	0	0	0	0	0	0	0	0	0	0
0	0	0	0	0	0	0	0	0	0	0	0	0	0	0	0
0	0	0	0	0	0	0	0	0	0	0	0	0	0	0	0
0	0	0	0	0	0	0	0	0	0	0	0	0	0	0	0
0	0	0	0	0	0	0	0	0	0	0	0	0	0	0	0
0	0	0	0	0	0	0	0	0	0	0	0	0	0	0	860
110	0	0	0	0	0	0	0	0	0	0	0	0	0	0	1702
0	0	0	0	0	0	0	0	0	0	0	0	0	0	0	0
0	0	0	0	0	0	0	0	0	0	0	0	0	0	0	1657
0	0	0	0	0	0	0	0	0	0	0	0	0	0	0	0
0	0	0	0	0	0	0	0	0	0	0	0	0	0	0	0
0	0	0	0	0	0	0	0	0	0	0	0	0	0	0	0
0	0	0	0	0	0	0	0	0	0	0	0	0	0	45	0
54	0	0	0	0	0	0	0	0	0	0	0	0	0	0	0
0	0	0	0	0	0	0	0	0	0	0	0	0	0	0	0
39	0	0	0	0	0	0	0	0	0	0	0	0	0	0	0
0	0	0	0	0	0	0	0	0	0	0	0	0	0	0	0
0	0	0	0	0	0	0	0	0	0	0	0	0	0	0	0
0	47	6957	268	571	0	0	0	0	0	0	0	0	0	106	0
0	17	1747	110	0	0	52	0	3601	0	0	0	0	0	0	0
0	36	0	0	0	0	0	0	0	0	0	0	0	0	0	0
0	0	862	57	0	0	0	0	0	0	0	0	0	0	0	0
0	0	0	0	0	0	0	0	0	0	0	0	0	0	0	0
0	0	0	0	0	0	0	0	0	0	0	0	0	0	0	0
0	0	0	0	0	0	0	0	0	0	0	0	0	0	6753	0
0	0	0	0	0	0	0	0	0	0	0	0	0	0	0	0
7606	10976	2602	58	109	23651	373	99	148	280	167	173	28	586	0	641
0	0	0	0	0	0	0	0	0	0	0	0	0	0	0	0
0	0	0	0	0	0	0	0	0	0	0	0	0	0	0	0
48	0	0	0	0	0	0	0	0	0	0	0	0	0	0	545
196	6616	7781	167	110	4302	39	987	573	3960	39	79	337	116	2555	195
0	9	0	0	0	0	0	0	0	0	0	0	0	0	0	0
1694	117683	27758	688	1582	124451	1029	556	4123	49743	301	10577	7212	3884	14920	17293
3163	99	858	58	478	0	94	0	3870	30	0	5659	0	174	0	201
0	0	0	0	0	0	0	0	0	0	0	0	0	0	0	0
0	0	0	0	0	0	0	0	0	0	0	0	0	0	0	0
0	0	0	0	0	0	0	0	0	0	0	0	0	0	0	0
0	0	0	0	0	0	0	0	0	0	0	0	0	0	0	0
0	0	0	0	0	0	0	0	0	0	0	0	0	0	0	0
0	0	0	0	0	0	0	0	0	0	0	0	0	0	0	0
864764	59	3473	480	807	0	48	0	216	30	0	0	25	0	467	197
0	0	0	0	0	0	0	0	0	0	0	0	0	0	0	0
0	0	0	58	0	0	0	0	140	0	0	0	29	0	0	0
0	0	0	0	0	0	0	0	0	0	0	0	0	0	0	0
0	0	0	0	0	0	0	0	0	0	0	0	0	0	0	0
0	0	0	0	0	0	0	0	0	0	0	0	0	0	0	0
0	0	0	0	0	0	0	0	0	0	0	0	0	0	0	0
34	0	0	0	0	2789	0	28	0	0	0	0	27	0	0	0
503	0	0	0	0	0	0	0	0	0	0	0	0	112	48	0
0	0	0	68	0	0	0	0	0	0	0	0	0	0	0	0
0	0	0	0	0	0	0	65	0	60	0	0	9054	0	0	0
0	20	0	7633	92	60	9622	28	0	0	0	0	0	0	712	0
0	0	0	0	0	0	0	7179	48	34	0	0	0	0	0	0

0	0	0	0	0	0	0	0	0	0	0	0	0	0	0	0
0	0	0	0	0	0	0	0	0	0	0	0	0	0	53	12233
0	21	0	141	85	0	0	0	0	0	0	0	0	0	0	0
0	0	0	0	0	0	0	0	0	0	0	0	0	0	0	0
0	0	0	0	0	0	0	0	0	0	0	0	0	0	0	0
0	0	0	0	0	0	0	0	0	0	0	0	0	0	0	0
0	0	0	0	0	191	0	0	0	0	0	0	0	0	0	0
0	0	0	0	0	0	0	0	0	0	0	0	0	0	0	0
46	0	0	0	0	0	0	0	0	0	0	0	0	0	0	0
57	0	0	0	0	0	0	0	0	0	0	0	0	0	0	0
0	0	0	0	0	0	0	0	0	0	0	0	0	0	0	12183
0	0	0	0	0	0	0	0	0	0	0	0	0	0	0	0
0	42481	4314	241	92	123	0	0	0	0	0	0	44	66	0	0
0	0	0	0	0	0	0	0	0	0	0	0	0	0	0	98
0	36	2591	114	181	0	0	0	0	0	453	0	0	0	0	0
0	0	0	0	0	0	0	0	0	0	0	0	0	0	0	275
0	0	0	0	0	0	0	0	0	0	0	0	0	0	0	0
0	0	0	0	0	1155	0	0	0	0	0	0	0	0	0	0
0	0	0	0	0	0	0	0	0	0	0	0	0	0	0	0
0	0	0	0	0	0	0	0	0	0	0	0	0	0	0	0
97	0	0	60	0	58	0	0	0	0	0	0	177	0	262	291398
0	0	0	0	0	0	0	0	45	0	0	0	32	0	0	0
0	0	0	0	0	0	0	0	3806	0	0	0	0	0	0	0
0	0	0	0	0	0	143	35	15832	67	0	0	0	63	0	0
0	0	0	0	0	0	0	0	0	0	0	0	0	0	0	0
0	0	0	0	0	0	0	0	0	0	0	0	0	0	0	0
104	167	861	2434	177	110	190	64	7317	2190	37	126	4879	142	58	0
0	0	0	0	0	0	0	0	0	0	0	0	0	0	0	0
0	0	0	0	0	0	0	0	659	0	0	0	0	0	0	0
0	0	0	0	0	121	0	0	0	0	0	0	0	0	0	0
0	0	0	0	0	0	0	0	0	0	0	0	0	0	0	0
0	0	885	4846	81	157	6040	0	0	0	0	0	0	0	663	0
0	0	0	0	0	0	0	0	0	0	0	0	0	0	0	0
0	0	0	0	0	0	0	0	0	0	0	0	0	0	0	0
0	0	0	0	0	0	0	0	0	0	0	0	0	0	0	0
0	0	0	0	0	0	0	0	0	0	0	0	0	0	0	0
0	0	0	0	0	0	96	0	0	0	0	0	0	0	0	0
0	0	0	0	0	0	0	0	0	0	0	0	0	0	0	0
0	0	0	0	0	117	0	0	0	0	0	0	0	0	0	0
0	0	0	0	0	0	0	0	0	0	0	0	0	0	0	0
0	0	0	0	0	0	0	0	0	0	0	0	0	0	0	0
0	0	0	0	0	0	0	0	0	0	0	0	0	0	0	0
0	0	0	0	0	0	0	0	0	0	0	0	0	0	0	0
90	0	0	0	0	0	0	0	0	0	0	0	0	0	0	0
0	0	0	0	0	0	0	0	0	0	0	0	0	0	0	0
0	0	0	0	0	0	0	0	0	0	0	0	0	0	0	0
0	0	0	0	0	0	0	0	0	0	0	0	0	0	359	880
0	0	0	0	0	0	0	0	0	0	0	0	0	0	0	0
0	0	0	0	0	0	0	0	0	0	0	0	0	0	0	0
0	0	0	0	0	0	0	0	0	0	0	0	0	0	0	0
0	0	0	0	0	0	0	0	0	0	0	0	0	0	0	0
0	0	0	0	0	0	0	0	0	0	0	0	0	0	0	0
0	0	0	0	0	0	0	0	0	0	0	0	0	0	0	0
0	53	6903	53	116	0	36	33	57	27	0	0	6404	145	0	0
0	0	0	0	0	16251	0	0	0	0	0	37	25	0	0	0
0	0	0	0	0	0	0	0	0	0	0	0	0	0	0	0
0	0	0	0	0	0	0	0	0	38	82	22157	0	0	0	0
47	15	0	1138	90	0	187	0	5840	27	0	42	916	263	0	0

0	0	0	0	0	0	0	0	0	0	0	0	0	0	0	0
0	0	0	0	0	0	0	0	0	0	0	0	0	0	0	106
0	0	0	0	0	0	0	0	0	0	0	0	0	0	0	0
0	0	0	0	0	0	0	72	0	70	0	45	0	0	0	0
0	0	0	0	0	0	0	0	0	0	42	0	0	76	0	0
0	0	0	0	0	0	0	0	0	0	0	0	0	0	0	0
0	0	0	0	0	0	0	0	0	0	0	0	0	0	0	0
0	0	0	0	0	0	0	0	0	0	0	0	0	0	0	0
0	0	0	0	0	0	0	0	0	0	0	0	0	0	0	0
0	0	0	0	0	0	0	0	0	0	0	0	0	0	0	179
0	0	0	0	0	223	0	0	0	0	0	22	0	0	0	0
0	0	0	0	0	0	0	0	0	0	0	0	0	0	0	0
0	157580	19050	918	1312	219	530	574	371	253429	289	320	310	1290	0	0
0	0	0	0	0	0	0	0	0	0	0	0	0	0	0	0
0	0	0	0	0	0	0	0	411	0	0	0	0	0	0	465
0	0	0	0	0	0	0	38	0	37	38	74	10101	0	0	0
0	0	0	0	0	8692	50	0	0	0	0	0	30	0	0	0
0	0	0	673	0	44	0	0	59	87	174	0	11718	0	0	0
0	0	0	0	0	0	0	0	0	0	0	0	0	0	0	0
0	0	0	0	0	0	0	0	0	0	0	0	0	0	0	0
0	0	0	0	0	0	0	0	0	0	0	0	0	0	0	0
0	0	0	0	3139	0	0	0	0	0	0	0	0	0	0	0
0	0	0	0	0	0	0	0	0	0	0	0	0	0	0	0
0	0	0	0	0	0	0	0	0	0	0	0	0	0	0	0
0	0	0	0	0	107	130	74	40	59727	23	110	0	70	0	0
0	0	0	0	0	0	0	0	0	0	0	0	0	0	0	0
0	0	0	0	0	0	0	0	0	0	0	0	0	0	0	0
0	0	0	0	0	0	0	0	0	0	0	0	0	0	0	0
0	0	0	0	0	66	0	0	0	0	0	0	0	0	68	6104
0	0	0	0	0	0	0	0	0	0	0	0	0	0	0	0
0	0	0	0	0	0	0	0	0	0	0	0	0	0	9624	0
0	0	0	0	0	0	0	0	0	0	0	0	0	0	0	0
0	0	0	0	0	56	88	0	54	0	47	14514	30	0	0	0
0	0	0	0	0	0	0	0	0	0	0	0	0	0	0	0
0	0	0	0	0	0	0	0	5021	0	0	47	0	0	0	0
0	0	0	0	0	0	0	0	0	0	0	0	0	0	52	0
0	0	0	0	0	0	0	25	0	0	0	0	0	127	0	0
0	0	0	0	0	51	0	0	0	0	0	0	0	0	0	0
0	0	0	0	0	0	0	0	0	0	0	0	0	0	0	0
0	0	0	0	0	302	0	0	0	0	0	0	0	0	0	0
0	0	0	0	0	0	0	0	0	0	0	0	0	0	0	0
0	0	0	0	0	0	0	0	0	0	457	0	0	0	0	0
0	0	0	0	0	0	0	0	0	0	0	0	0	0	0	0
0	0	0	0	0	0	0	0	0	0	0	0	0	0	0	0
0	0	0	0	0	151	97	53	129	84	98	259	46	75	59	102
0	0	0	0	0	0	0	0	0	0	0	0	0	0	0	0
0	0	0	0	0	105	0	0	0	0	0	0	0	0	0	0
0	0	0	0	0	0	0	0	0	0	0	0	0	0	54	188
0	0	0	0	0	0	0	0	0	0	0	0	0	0	0	0
110	0	0	0	0	0	0	0	0	0	0	0	0	0	0	0
0	0	0	0	0	0	0	0	0	0	0	0	0	0	0	0
0	0	0	187	0	0	204	0	0	0	0	0	0	0	0	0
189	62	6940	39341	0	129	2220	35	12637	70	0	115	3394	69	107	2706
0	0	0	0	0	0	0	0	0	0	0	0	0	0	0	757
0	0	0	0	0	0	0	0	0	0	0	0	0	0	0	0
0	0	0	0	0	887	0	0	0	0	0	0	0	0	0	0
0	0	0	3913	296	1896	2157	0	187	21	0	33	0	0	37566	6746
0	0	0	0	0	0	0	0	0	0	0	0	0	0	0	579

0	0	0	0	0	0	46	43	0	0	0	42	37	74	0	0
0	0	0	0	0	0	0	0	0	0	0	0	0	0	0	0
0	0	0	0	0	0	0	0	0	0	0	0	0	0	0	0
0	0	0	0	0	0	0	0	0	0	0	0	0	0	12707	0
0	0	0	0	0	0	0	0	0	0	0	0	0	0	0	0
0	0	0	68	0	0	0	0	0	0	0	0	0	0	0	0
0	0	0	0	0	0	0	42	43	0	44	43	0	0	0	0
0	0	0	0	0	0	0	0	0	0	0	0	0	0	0	0
0	0	868	113	104	0	0	0	0	0	0	0	0	0	0	0
0	0	0	0	0	0	0	0	0	0	0	0	0	0	0	2814
0	0	0	55	0	0	0	0	0	0	0	0	0	0	0	0
0	0	0	0	0	0	0	0	0	0	0	0	0	0	0	5067
0	66	4366	240	164740	78416	401	50056	302	438	312	88031	90	2665	210	1112
0	18	850	0	0	0	0	0	0	0	0	0	0	0	0	0
0	0	883	132	519	336	0	70	0	22	75	0	2127	3676	56	0
0	0	0	0	0	0	420	0	0	0	0	0	0	0	0	0
161	0	0	137	0	0	0	0	0	0	0	0	0	0	0	0
0	0	0	0	0	0	0	0	0	0	0	0	0	0	0	0
0	0	0	0	0	0	0	0	0	0	0	0	0	0	0	0
0	0	0	0	0	0	0	0	0	0	0	0	0	0	0	0
0	0	0	0	0	0	0	0	0	0	0	0	0	0	0	0
870	0	878	0	114	0	0	0	0	0	0	0	0	0	0	0
0	0	0	0	0	0	0	0	0	0	0	0	0	0	0	0
0	0	0	0	0	0	0	0	0	0	0	0	0	0	0	0
0	61	3464	0	0	0	0	0	0	0	0	0	0	0	0	0
0	49	5223	376	484	0	0	0	0	0	0	0	0	0	0	0
0	0	0	0	0	0	0	0	0	0	0	0	0	0	0	0
87	0	0	0	0	0	0	0	0	0	0	0	0	0	0	0
0	0	0	0	0	0	0	0	0	0	0	0	0	0	0	0
599	132	5228	9554	748	1574	1648	675	61404	349	84	1946	74764	341	538	872
0	0	0	0	0	0	0	0	0	0	0	0	0	261	0	0
0	0	0	0	0	0	0	0	0	0	0	0	0	0	0	0
0	0	0	0	0	0	0	0	0	0	0	0	0	0	0	0
0	0	0	0	0	0	0	0	0	0	0	0	0	0	0	0
1127	0	0	0	0	0	0	0	0	0	0	0	0	0	0	0
0	0	0	0	0	0	0	0	0	0	0	0	0	0	0	0
0	0	0	0	0	0	0	0	0	0	0	0	0	0	0	0
0	0	0	0	0	0	0	0	0	0	0	0	0	0	0	0
0	0	0	0	0	8754	0	35	0	0	44	0	0	69	0	0
0	0	0	0	0	0	0	0	0	0	0	0	0	0	0	0
0	48200	10440	289	176	0	0	0	0	0	0	0	0	0	0	0
0	0	0	0	0	39	0	0	0	906	0	0	0	0	0	0
0	0	0	0	0	0	0	0	0	0	0	0	0	0	0	0
0	56	5205	49	179	0	0	0	0	0	0	0	0	0	0	0
0	0	0	0	0	0	0	0	0	0	0	0	0	0	0	0
0	0	0	0	0	0	54	0	3681	0	42	0	0	51	0	0
0	0	0	0	0	0	0	0	0	34	0	0	0	0	0	0
0	0	0	0	0	0	0	0	0	0	0	0	0	0	0	0
52	0	0	0	0	5580	0	0	0	0	0	0	0	0	0	0
0	0	0	0	0	0	0	0	0	0	0	0	0	0	0	0
0	0	0	0	0	0	96	112	0	90	0	25660	0	63	0	93
0	0	0	0	0	0	0	0	0	0	0	0	0	0	0	0
0	0	0	0	0	0	0	0	0	0	0	0	0	0	0	1319
0	0	0	0	0	0	0	0	0	0	0	0	0	0	0	0
0	0	0	0	0	52	50	34	0	70277	121	73	56	187	0	0
0	0	0	0	0	0	0	0	0	0	0	0	0	0	0	0
0	0	0	0	0	0	0	0	0	0	0	0	0	0	0	0
0	0	0	0	0	0	0	0	0	0	0	0	0	0	46	0

0	16	0	0	0	149	163	41	83	177	1286	37	42	59	57	0
0	0	0	0	0	0	0	0	0	0	0	0	0	0	0	0
0	0	0	0	0	0	0	0	0	0	0	0	0	0	0	0
11698	16	877	0	82	143	0	0	91	25	0	42	103	0	0	112119
0	0	0	0	0	0	0	0	0	0	0	0	0	0	0	0
65	0	2569	50	0	0	0	0	0	0	0	0	0	0	0	0
1297	0	0	2901	0	0	0	83	67	0	15301	47	25	66	0	0
0	0	0	0	0	0	0	0	0	0	0	0	0	0	0	0
0	0	0	0	0	0	0	0	0	0	0	0	0	0	0	0
0	0	0	0	0	0	0	0	0	0	0	0	0	0	0	0
0	0	0	0	0	0	0	0	0	0	0	0	0	0	0	0
0	0	0	0	0	0	0	0	39	28	0	48	0	67	0	0
0	0	0	0	0	0	0	0	0	0	0	0	0	0	0	0
0	0	0	0	0	0	0	0	0	0	0	0	0	0	0	0
53	0	0	0	0	0	0	0	0	0	0	0	0	0	0	0
119	0	0	0	0	0	0	0	0	0	0	0	0	0	0	0
109	0	0	0	0	0	41	0	0	39	50	0	5798	117	0	0
0	0	0	2028	91	0	5540	0	0	34	0	0	0	0	256	0
0	0	0	0	0	0	0	0	0	0	0	0	0	0	0	5425
0	0	0	0	0	0	0	0	0	0	0	0	0	0	0	0
0	62	9497	107	254	0	0	0	0	0	0	0	0	0	0	0
111	0	0	0	0	0	0	0	0	0	0	0	0	0	0	0
141	0	0	0	0	0	0	0	2445	38	0	0	0	0	0	2231
0	0	0	0	0	0	0	0	0	0	0	36	0	479	8885	0
0	0	0	0	0	0	0	0	0	0	0	0	0	0	0	0
0	0	0	0	0	0	0	0	0	0	0	0	0	0	0	0
0	0	0	0	0	0	0	0	0	0	0	0	0	0	4883	0
0	0	0	0	0	0	0	0	0	0	0	0	0	0	0	0
0	0	0	0	0	0	0	0	0	0	0	0	0	0	0	0
0	0	0	0	0	0	0	0	0	0	0	0	0	0	0	0
0	0	0	0	0	0	0	0	0	0	0	0	0	0	52	0
0	0	0	0	0	0	0	0	0	0	0	0	0	0	0	0
0	0	0	0	0	0	0	0	0	0	0	0	0	0	0	0
0	0	0	0	0	0	0	0	0	0	0	0	0	0	0	308
0	0	0	0	0	0	0	0	0	0	0	0	0	0	0	0
0	0	0	0	0	0	0	0	0	0	0	0	0	0	56	398
0	0	0	0	0	0	0	0	0	0	0	0	0	0	0	98
0	0	0	0	0	0	0	0	0	0	0	0	0	0	0	2984
0	0	0	0	0	1275	1670	840	908	1783	882	1658	556	1814	0	0
0	0	0	0	0	0	0	0	0	0	0	0	0	0	0	0
0	0	0	0	0	0	0	0	0	0	0	0	0	0	0	0
0	0	0	0	0	0	0	0	0	0	0	0	0	0	0	0
0	0	0	75	0	0	88	0	0	0	0	0	0	0	0	0
0	0	0	0	0	0	0	0	0	0	0	0	0	0	0	0
0	0	0	0	0	0	0	0	0	0	0	0	0	0	0	0
44	0	0	0	0	0	0	0	0	0	0	0	0	0	0	0
440	0	0	0	0	0	50	11851	0	67	49	35	0	10157	0	0
0	0	0	0	0	0	0	0	0	0	0	0	0	0	0	0
0	0	0	0	0	0	0	0	0	0	0	0	0	0	0	0
0	0	0	0	0	0	0	0	0	0	0	0	0	0	0	0
0	0	0	0	0	0	0	0	0	0	0	0	0	0	0	0
0	0	0	0	0	0	0	0	0	0	0	0	0	0	0	0
0	0	0	0	0	0	0	0	0	0	0	0	0	0	0	0
0	0	0	0	0	0	0	0	0	0	0	0	0	0	0	0
0	0	0	0	0	0	0	0	0	0	0	0	0	0	0	0
0	0	0	0	0	0	0	0	0	0	0	0	0	0	0	0
0	83	7828	373	341	0	35	0	0	0	0	0	0	0	0	1641
0	89	5207	338	105	0	0	0	0	0	0	0	0	0	0	0

0	0	0	0	0	60	127	23717	0	110	48	39	23	0	0	0
0	0	0	0	0	0	97	0	0	0	0	0	0	0	0	0
0	0	0	0	0	0	0	0	0	0	0	0	0	0	0	0
0	0	0	0	0	0	0	0	0	40	0	0	0	0	298	197
0	0	0	0	0	0	0	0	0	0	0	0	0	0	0	4275
0	53284	6060	284	381	72	0	0	0	0	0	82	0	256	0	0
0	0	0	0	0	0	0	0	0	0	0	0	0	0	0	0
0	0	0	0	0	0	52	0	1163	0	0	0	0	0	0	0
0	0	0	505	0	0	0	0	0	0	0	0	0	0	0	0
0	0	0	0	0	0	0	0	0	0	0	0	0	0	0	0
0	0	0	0	0	0	0	0	0	0	0	0	0	0	0	0
0	0	0	0	0	0	0	0	0	0	0	0	0	0	0	0
0	0	0	0	0	0	0	0	0	0	0	0	0	0	0	0
0	0	0	0	0	0	0	0	0	0	0	0	0	0	0	0
0	0	0	0	0	0	0	0	0	0	0	0	0	0	0	0
0	0	0	0	0	0	0	0	0	0	0	0	0	0	0	0
0	0	0	0	506	0	0	0	0	0	0	0	0	0	0	0
0	0	0	0	0	0	0	0	0	0	0	0	0	0	0	0
0	0	0	0	0	0	0	26	18944	41	0	0	0	0	0	0
0	0	0	0	0	110	0	0	0	0	0	0	0	0	0	0
22896	50637	185743	7411	274312	93599	5517	5490	36626	59843	845392	34000	30149	28331	59799	318014
0	0	0	0	0	0	0	0	0	0	0	0	0	0	0	0
0	0	0	0	0	0	0	0	0	0	0	0	0	0	0	0
0	0	0	0	0	168	0	80	0	0	0	445	0	0	0	0
0	0	0	0	0	0	0	0	0	0	0	0	0	0	0	0
0	0	0	0	0	0	0	0	0	0	0	0	0	0	0	0
0	0	0	0	3328	599	40	4391	0	0	0	2478	0	130	0	0
0	0	0	0	0	0	0	0	0	0	0	0	0	0	0	0
0	0	0	0	0	0	0	0	0	0	0	0	0	0	0	0
0	0	0	0	0	0	0	0	0	0	0	0	0	0	0	0
0	0	0	0	0	0	0	0	0	0	0	0	0	0	0	0
0	0	0	0	0	0	0	0	0	0	0	0	0	0	3956	87
0	0	0	0	0	0	0	0	0	0	0	0	0	0	0	0
0	0	0	173	0	0	49	0	2072	0	0	0	1834	0	0	92

Control043	Control044	Control045	Control046	Control047	Control048	Control049	Control050	Control051	Control052	Control053	Control054	Control056	Control058	Control059	Control060
952283	938843	882089	718844	864862	882507	974198	954868	945107	712039	208579	932798	618513	908836	847445	973163
41904	53198	86478	263255	84401	7863	3748	6667	6258	8570	84465	28391	237114	37311	139317	3570
0	0	0	0	684	0	0	0	0	0	0	0	0	0	0	0
808	7785	22013	17901	43877	31393	19737	36961	44166	273620	19951	27499	11276	52168	11950	15632
5005	174	6332	0	6176	75286	2246	1283	4439	5288	686744	11246	50015	1587	1288	7546
0	0	0	0	0	90	71	221	30	483	261	66	83082	98	0	0
0	0	3088	0	0	2861	0	0	0	0	0	0	0	0	0	89

Control043	Control044	Control045	Control046	Control047	Control048	Control049	Control050	Control051	Control052	Control053	Control054	Control056	Control058	Control059	Control060
0	0	0	0	0	0	0	165	80	258	37	0	36	77	0	0
0	0	0	0	0	0	0	0	0	0	0	0	0	0	0	0
30724	9944	393220	4373	4555	15373	10189	856360	680712	37482	20683	9137	9468	25255	8329	15967
0	0	0	0	0	0	0	0	0	0	0	0	0	0	0	0
0	0	0	0	0	76	62	0	24	120	35	38	33	35	0	0
0	0	0	0	0	0	0	0	0	0	0	0	0	0	0	0
0	0	0	0	0	0	0	0	0	0	0	0	0	0	0	0
0	0	0	0	0	0	0	0	0	0	0	0	0	0	0	0
0	0	0	0	0	0	0	0	0	0	0	0	0	0	0	0
1051	15200	820	185710	577	85	2224	764	220	133	9041	49	130	37	503	1277
0	0	0	0	0	0	0	0	0	0	0	22	0	0	0	0
0	0	0	0	0	0	0	0	0	0	0	0	0	0	0	0
0	0	0	0	0	0	0	0	0	0	0	0	0	0	0	0
43	0	325	0	0	103229	144	5116	191	514	142	32	36	875	203	0
0	65	119	0	0	0	0	518	0	0	0	0	0	0	0	0
0	0	0	0	0	0	0	0	0	0	0	0	0	0	0	0
0	0	0	0	0	0	0	0	0	0	0	0	0	0	0	0
46	120	68	286	0	271	145	473	149	0	129	69	41744	140	412	157
0	0	0	0	0	0	0	0	0	0	0	0	0	0	0	0
0	0	0	0	0	0	0	0	0	0	0	0	0	0	0	0
0	0	77	55	60	0	0	0	0	0	180	55	0	35	280	281
0	0	0	0	0	0	0	27	0	0	0	0	0	0	0	0
58	10376	25	63	0	1434	17715	32	0	237	1376	180	0	25	97	86
0	1676	41	0	0	0	0	0	0	0	0	0	0	0	0	0
0	0	0	0	0	0	0	0	0	0	0	0	0	0	0	0
4385	209428	50993	47920	69453	17552	385408	9710	3180	618893	35177	10407	68758	17773	15593	4842
0	0	0	0	0	0	0	0	0	0	38	0	0	0	0	0
897	0	109	64	0	0	82	71	86	0	43	35	0	0	0	0
0	59	112	0	20661	0	0	0	0	0	0	0	0	0	0	0
0	0	0	0	0	167	143	135	278	643	345	25	61242	121	0	0
0	0	0	0	684	0	0	0	0	0	0	0	0	0	0	0
0	0	0	0	0	0	81	0	1744	0	0	0	0	0	0	0
0	0	0	0	0	0	0	0	0	0	0	0	0	0	9329	0
0	0	0	0	0	1803	0	0	0	0	13520	146	39	0	0	0
0	0	0	0	0	0	0	0	0	0	0	0	0	0	0	0
0	0	0	0	0	0	0	0	0	0	0	0	0	0	0	0
0	49	0	0	0	0	0	0	0	0	0	0	0	0	0	0
0	0	0	0	0	0	0	0	0	0	0	0	0	0	0	0
0	0	0	0	0	0	0	0	0	0	0	0	0	0	0	85
5407	334876	149006	1964	581844	7540	495960	774	843	2145	26093	11253	55679	1473	1519	4971
93	0	31	0	0	0	0	0	0	0	0	0	0	35	0	0
93	62482	136	50	0	0	0	0	0	0	0	0	0	0	0	0
0	0	0	0	0	0	0	0	0	0	0	0	0	0	84	0
0	0	0	0	0	0	0	0	0	0	0	0	0	0	0	0
0	0	41	0	0	0	0	0	0	0	0	33	0	0	0	0
0	0	0	0	0	0	0	0	121	0	0	0	0	0	0	0
0	0	0	0	0	0	0	1159	0	0	0	0	0	0	0	0

0	0	0	0	0	0	0	0	0	0	0	0	0	0	14312	0
0	0	0	0	0	0	0	0	0	0	0	0	0	0	0	0
933	101	169	90601	1525	55075	2963	1332	1764	4670	2732	35349	1256	1009	61265	1394
851	0	0	71	0	0	0	0	0	0	0	0	0	0	21322	85
0	0	0	0	0	75	65	99	34	0	131	80	27317	0	0	0
0	0	0	0	0	0	0	0	0	0	0	0	0	0	0	0
0	0	0	0	0	4310	0	0	30	0	44	0	0	36	0	0
0	0	0	0	0	0	0	0	0	0	0	0	0	0	0	0
0	0	36	0	0	0	0	0	0	0	0	0	0	0	0	0
0	55	40	0	0	0	0	0	0	0	0	0	0	0	0	0
0	0	0	0	0	0	0	0	0	0	0	0	0	0	0	0
0	0	0	0	0	0	0	0	0	0	0	0	0	0	0	0
41	0	95	0	23121	0	0	0	0	0	0	0	0	0	0	0
0	0	0	0	0	0	0	0	0	0	0	0	0	0	13006	0
0	0	0	0	0	0	0	0	0	0	0	0	0	0	0	0
209	115033	182	232	484	355	587	0	92	1374	140	7265	718	76	93	9271
0	0	0	0	0	0	0	0	0	0	0	0	0	0	0	0
0	0	0	59	0	0	0	0	0	0	0	0	0	0	0	0
40	0	0	12101	0	0	0	0	0	0	0	0	0	0	0	0
7871	12673	29616	103794	731	2065	1009	1006	4375	4677	31671	16998	159376	256454	310908	2855
0	0	40	63675	0	0	0	0	0	0	0	0	0	0	0	0
0	0	0	0	0	0	0	0	0	0	0	0	0	0	0	0
0	0	0	0	0	0	0	0	0	0	0	0	0	0	0	0
0	0	0	0	0	0	0	0	0	0	0	0	0	0	0	0
701	0	0	0	0	0	0	0	0	0	0	0	0	0	0	0
0	0	0	0	0	0	0	0	0	0	0	0	0	0	0	0
0	0	0	0	0	0	0	0	0	0	0	0	0	0	0	0
0	0	0	0	0	0	0	0	0	0	0	0	0	0	0	0
54	0	0	115	0	0	0	0	0	0	0	0	0	0	0	90
0	0	0	0	0	98	0	0	0	0	0	0	0	0	5194	0
0	0	0	0	0	0	0	0	0	0	0	0	0	0	0	0
0	0	0	0	0	0	0	0	0	0	0	0	0	0	0	0
0	0	0	0	0	0	0	0	0	0	0	0	0	0	0	0
3851	23607	51681	110868	44498	557	532	885	1655	833	23873	26697	59680	30915	11215	1282
0	0	0	0	0	0	0	0	0	0	0	0	0	0	0	0
0	0	0	0	0	0	0	0	0	0	0	0	0	0	0	0
0	0	63	0	64	0	0	0	0	0	0	0	0	0	0	0
0	0	0	0	0	0	0	0	0	0	0	0	0	0	0	0
326	0	0	0	0	0	0	0	0	0	0	0	0	0	1699	0
0	0	0	0	0	0	0	0	0	0	0	0	0	0	0	0
0	0	0	0	0	0	0	0	0	0	0	0	0	0	0	0
0	0	0	0	0	0	0	0	0	0	0	0	0	0	0	0
0	0	0	0	0	0	0	0	0	0	0	0	0	0	0	0
0	0	0	0	0	85	431	210	375	876	457	121	101	95	108	164
0	0	0	0	0	0	0	0	0	0	0	0	0	0	0	0
0	0	0	0	0	0	213	0	0	0	0	0	0	0	0	0
0	0	0	0	0	0	0	0	0	0	0	0	0	0	0	0
0	0	0	0	0	0	0	0	0	0	0	0	0	0	14036	93
748	607	439	103	11193	0	145	126	38	0	176	0	35	31	0	90
0	0	0	0	0	0	0	0	0	0	0	0	30	0	0	0
0	0	0	0	0	0	0	0	0	0	0	0	0	0	0	0
0	0	0	0	0	0	0	0	0	0	0	0	0	0	88	0
0	0	0	0	0	0	0	0	0	0	0	0	0	0	7725	0
58	0	38	0	0	0	70	113	124	514	196	85	28	0	582	839
0	0	0	0	0	0	0	106	0	125	0	30	0	0	0	0
5005	174	6299	0	186	74617	1421	1151	4135	3980	685983	10836	49772	1255	917	7461
35409	51	72	60	0	0	0	0	0	0	0	0	0	0	0	0
0	0	0	0	0	0	0	0	0	0	0	0	0	0	0	0

0	0	0	0	0	0	0	0	0	0	0	0	0	0	0	0
0	0	0	0	0	0	0	0	58	0	0	0	0	0	0	0
0	0	0	0	0	0	0	0	0	0	0	0	0	0	0	0
0	0	0	0	0	0	0	0	0	0	0	0	0	0	0	0
0	0	0	0	0	0	0	0	0	0	0	0	0	0	9427	0
0	0	0	0	0	0	0	0	0	0	0	0	0	0	0	0
0	0	0	0	0	0	0	0	0	0	0	0	0	0	0	0
0	0	75	0	50	103	747	712	0	0	0	0	0	0	0	0
0	0	0	0	0	272	136	244	67	287	277	71	69	126	0	0
0	5554	38	0	0	0	0	0	0	0	0	0	0	0	0	0
0	0	0	0	0	0	0	0	0	0	0	0	0	0	0	0
0	0	0	0	0	0	0	0	0	0	0	0	0	0	0	0
0	0	0	0	0	0	0	0	0	0	0	0	0	0	0	0
0	0	0	0	0	0	0	0	0	0	0	0	0	0	0	0
0	0	0	0	0	0	0	0	0	0	0	0	0	0	0	0
356	377	21143	70	112	94	146	3633	31	0	193	26	0	0	107	0
0	0	526	0	0	0	0	2145	0	0	0	0	0	0	0	81
26612	8266	39	120	56	0	0	0	40	0	0	0	0	0	0	0
0	0	0	0	0	0	0	0	0	0	0	0	0	0	0	0
0	0	0	0	0	0	0	0	0	0	0	0	0	0	0	0
0	60	0	0	0	0	0	0	0	0	0	0	0	0	113	165
0	0	0	0	0	0	0	0	0	0	0	0	0	0	0	0
808	7785	21938	17901	43827	31290	18990	36249	44166	273620	19951	27499	11276	52168	11950	15632
0	3767	0	0	95	21517	835	205	24	232	0	0	80	0	0	0
0	17882	307	115	1313	338	16265	0	34	0	329	0	164	0	86	0
0	0	0	0	0	0	0	0	0	0	0	0	0	0	0	0
0	0	28	0	0	548	473	249	168	1156	388	308	170	243	14531	899
0	4865	38	0	0	0	0	0	0	0	0	0	0	0	0	0
56	0	0	0	57	0	0	0	33	0	0	0	498	39	0	88
0	0	0	0	0	0	143	0	0	0	40	0	0	0	0	104
0	0	105	0	0	0	0	578	0	0	0	0	0	0	0	0
0	0	0	0	0	0	0	0	0	0	0	0	0	0	0	0
0	0	0	0	0	0	0	0	0	0	0	0	0	0	0	0
0	0	0	0	0	0	0	0	0	0	0	0	0	0	0	0
274	247	105	214	218	0	0	0	0	0	0	0	0	0	3765	0
0	0	0	0	0	0	0	0	0	0	0	0	0	0	0	0
0	0	0	0	0	0	0	0	0	0	0	0	0	0	0	0
0	0	0	60	0	0	0	0	0	0	0	0	0	0	0	0
0	0	0	0	0	0	0	0	349	0	0	0	0	0	85810	160
0	0	0	0	0	0	0	0	0	0	0	0	0	0	0	0
0	0	0	0	0	0	0	0	0	0	0	0	0	0	0	0
256	831	264	0	0	444	0	1057	0	0	0	0	0	0	0	0
0	0	0	0	0	0	0	0	0	0	0	0	0	0	25208	0
0	0	0	0	0	0	0	0	0	0	0	0	0	0	0	0
0	0	0	0	0	0	0	0	0	0	32	0	0	0	0	0
0	0	0	0	0	0	0	0	0	0	0	0	0	0	0	0
0	0	37	0	0	0	0	0	0	0	0	0	0	0	0	0
0	0	10502	0	0	0	0	0	0	0	0	0	0	0	0	0
0	0	0	0	0	0	0	0	0	0	0	0	0	0	0	0
0	0	0	0	0	0	0	0	0	0	0	0	0	0	0	0
0	0	0	0	0	0	0	0	0	0	5724	0	0	0	0	0
0	0	128	0	0	84	71	0	269	0	70	0	4527	33	101	84
0	0	0	0	0	0	0	0	0	0	0	1335	0	0	0	0
0	0	0	0	0	0	0	0	0	0	0	0	0	0	0	0
0	0	0	0	0	0	0	0	0	0	0	0	0	0	0	0
0	0	0	0	0	0	0	35	0	0	69	0	0	0	0	0
0	0	0	0	0	0	0	0	58	0	0	0	0	0	0	0
0	0	0	0	0	0	0	0	0	0	11111	0	0	28	0	0

0	0	0	0	0	0	0	434	0	0	0	33	0	0	0	0
0	0	0	0	0	0	0	0	0	0	0	0	0	0	0	0
0	0	0	0	0	0	0	0	0	0	0	0	0	0	0	0
0	0	0	0	0	0	0	0	0	0	5180	0	43	0	0	0
333	59	26	60	159	0	0	0	0	0	0	0	0	0	208	426
0	0	0	0	0	0	0	0	0	0	0	0	0	0	0	0
0	0	0	0	0	0	0	0	0	0	0	0	0	0	0	0
0	0	0	0	0	0	0	0	0	0	0	0	0	0	0	0
0	72	20419	63	0	0	0	0	0	0	0	0	0	0	0	0
0	0	32	0	0	0	0	0	0	0	0	0	0	0	0	0
0	0	0	0	0	0	0	0	0	0	0	0	0	0	0	0
0	0	0	0	0	0	0	0	0	0	29	0	0	0	0	0
0	0	0	0	0	0	0	0	0	0	0	0	0	0	0	0
49	62	33538	0	183	288	79	233	34	391	33	145	47908	221	0	0
0	0	0	0	0	0	0	0	0	0	0	0	0	0	0	0
0	0	0	0	0	0	0	0	0	0	0	0	0	0	0	0
338	0	0	0	0	0	0	0	25	0	0	0	0	0	0	0
0	0	0	0	0	0	0	0	0	0	0	0	0	0	2923	0
0	0	65	0	0	0	0	0	0	0	0	0	0	0	0	0
0	0	0	0	0	0	0	0	0	0	0	0	0	0	0	0
9296	12787	2926	132342	17377	928	306	204	162	526	13766	127	46465	90	7175	360
0	0	0	0	0	0	0	0	0	0	0	0	0	0	0	0
0	0	0	0	0	0	0	0	0	0	0	0	0	0	0	0
0	0	0	0	0	0	0	0	0	0	0	0	0	0	0	0
0	0	0	0	0	0	0	0	0	0	0	0	0	0	301	0
729	452	797	500	78744	0	0	0	38	0	0	0	121	0	0	0
0	0	0	0	0	0	0	0	0	0	0	0	0	0	0	0
0	0	839	0	0	0	0	34	0	0	0	0	0	0	0	0
0	0	0	0	0	0	0	0	0	0	0	0	0	0	0	0
0	0	0	0	0	593	763	132	280	1188	726	372	210	297	371	85
0	0	0	0	0	0	0	0	0	0	0	0	0	0	0	0
0	0	0	0	0	0	0	0	0	0	0	0	0	0	0	0
0	0	0	0	0	0	0	0	0	0	0	0	0	0	0	0
0	0	0	0	0	0	0	0	60	0	0	0	0	0	0	0
88	2804	456	0	2707	106	5786	0	0	0	0	31	45	0	0	90
0	0	0	0	0	0	0	0	0	0	0	0	0	0	0	0
0	0	0	0	0	0	0	0	0	0	0	0	0	0	0	0
0	0	0	0	0	0	0	0	0	0	0	0	0	0	0	0
0	0	0	0	0	0	0	0	0	0	0	0	0	0	0	0
0	0	0	0	0	0	0	0	0	0	0	0	0	0	0	0
0	0	0	0	0	0	0	0	22	0	0	0	0	0	0	0
0	0	0	0	0	0	0	0	0	0	0	0	0	0	19727	0
0	0	0	0	0	0	0	0	0	0	0	0	0	0	0	0
0	0	0	0	0	0	0	0	0	0	0	0	0	0	0	0
0	0	0	100	55	0	0	0	0	0	35	0	0	0	0	0
0	0	0	0	0	0	0	0	0	0	0	0	0	0	0	0
0	134	75	107	124	0	0	0	0	0	0	0	0	0	95	0
60	2741	0	0	0	85	0	0	0	0	0	0	0	0	0	0
0	0	0	13385	0	0	0	0	0	0	0	0	0	0	0	0
0	0	0	0	0	0	0	0	0	0	0	0	0	0	0	0
0	0	0	0	0	0	0	0	0	0	0	0	0	0	0	0
0	0	0	0	0	0	0	0	0	0	0	0	0	0	0	0
0	0	0	0	0	0	0	0	0	0	0	0	0	0	0	0
167	4768	412	121	346	73	199	62	0	0	0	250	0	0	0	0
0	0	0	0	0	0	0	40	0	150	0	0	0	0	0	0
262	0	0	0	0	0	0	0	0	0	0	0	0	0	0	0
61	109	2287	60	0	253	228	9425	6296	257	112	2946	43	76	276	2837
0	0	0	0	0	0	0	0	0	0	0	0	0	0	0	0

0	0	0	0	0	0	0	0	0	0	0	0	0	0	0	0
0	0	0	0	0	0	0	0	0	0	0	0	0	0	9075	0
0	0	0	0	0	0	0	0	0	0	0	0	0	0	0	0
0	2428	123	0	152	82	4563	32	488	0	67	31	0	0	0	0
0	0	0	0	0	0	0	0	0	0	0	0	0	0	0	0
0	0	0	0	0	0	0	0	0	0	0	0	0	0	0	0
0	0	0	0	0	0	0	0	0	0	0	0	0	0	0	0
549	686	561	2470	586	0	150	63	239	391	77	27	43253	39	18662	85
0	0	0	0	0	0	0	39	0	0	0	0	0	0	0	0
0	0	0	0	0	0	0	0	0	0	0	0	0	0	0	0
429	7755	429	58	0	87	68	5878	0	0	0	25	0	0	0	0
0	0	0	0	0	0	0	0	0	0	0	0	0	0	0	0
0	0	0	0	0	0	0	0	0	0	0	0	0	0	0	0
0	0	0	0	0	0	0	0	0	0	0	0	0	0	0	0
0	0	0	0	0	0	0	0	0	0	0	0	0	0	0	0
0	0	0	0	0	0	0	0	0	0	0	0	0	0	0	0
0	65	72	0	0	0	0	0	0	0	0	0	0	0	0	0
1469	0	0	0	0	89	0	0	0	0	0	37	0	0	30761	0
0	0	0	0	0	0	0	0	0	0	0	0	0	0	0	0
0	0	0	0	0	0	0	0	0	0	0	0	0	0	0	0
0	0	0	0	0	90	0	0	0	0	0	0	0	0	0	0
0	0	0	0	0	0	0	889	0	0	0	0	0	0	0	0
0	0	0	0	0	0	0	0	0	0	0	0	0	0	0	0
0	0	0	0	0	0	0	0	0	0	0	0	0	0	0	0
0	0	0	0	0	0	65	39	75	0	103	0	0	26	111	108
0	0	0	0	0	0	0	0	0	0	0	0	0	0	0	0
0	0	0	0	0	0	0	0	0	0	0	0	0	0	0	0
358	54	393	146	219	0	0	0	405	0	0	0	0	40	858	770
44	0	0	0	0	0	0	0	0	0	0	0	0	0	0	0
0	0	0	0	0	0	0	0	0	0	0	0	0	0	0	0
0	0	0	62	0	0	0	0	0	0	0	0	0	0	0	0
0	0	0	0	0	0	0	0	0	0	0	0	0	0	0	0
0	0	0	0	0	0	0	0	0	0	0	0	0	0	0	84
0	0	0	0	0	0	0	0	0	0	0	0	0	0	0	0
0	0	0	0	0	0	0	0	0	0	0	0	0	0	0	0
0	0	0	0	0	0	0	0	0	0	35	0	0	0	2183	0
0	0	0	0	0	0	0	0	0	0	0	0	0	0	0	0
0	0	0	0	0	0	0	0	0	0	0	0	0	0	41522	192
0	0	0	0	0	0	0	301	0	0	0	0	0	0	0	0
0	0	0	0	0	0	0	0	0	0	0	0	0	0	0	0
0	0	1518	0	0	0	0	0	0	0	0	0	0	0	0	0
0	0	0	0	0	0	0	0	0	0	0	0	0	0	0	0
40	53	173	176	105	0	0	0	0	0	0	0	0	0	0	0
0	0	0	0	0	0	0	35	0	0	39	0	0	14260	0	0
91	69	185	90684	52	92	0	0	0	0	0	0	0	0	18233	0
0	0	0	0	0	0	0	0	0	0	0	0	0	0	0	0
0	0	0	0	0	0	0	0	0	0	0	0	0	0	0	0
0	0	0	0	0	0	0	0	0	0	0	0	0	0	0	0
0	697	2780	0	65	1883	1642	2639	1350	3019	1457	707480	1171	467	1132	783272
0	131	89	29845	0	0	0	0	0	0	0	0	0	0	0	0
0	0	0	0	0	0	0	0	0	0	0	0	0	0	0	0
0	0	0	0	0	0	0	0	0	0	0	0	0	0	59357	0
505	92398	1062	238	1288	830	675	0	98	126	335	37	87	0	480	0
0	0	0	0	0	0	0	0	0	0	0	0	0	0	0	0
0	0	33	0	5990	0	0	0	0	0	0	0	0	0	0	0
0	0	0	0	0	0	0	0	0	0	0	0	0	0	0	0
0	0	0	0	0	1597	1725	1066	1071	4600	2236	687	978	1008	0	0
0	0	0	0	0	0	0	0	0	0	0	0	0	0	0	0

0	0	0	0	0	0	0	0	0	0	0	0	0	0	0	0
59	0	0	44	0	0	0	0	0	0	0	0	0	0	0	0
0	0	0	0	0	90	71	221	30	483	261	66	83082	98	0	0
0	0	0	0	0	0	0	0	0	0	0	0	0	0	0	0
213	71	6939	115	0	75	64	60	212	142	2714	0	1551	4685	477	328
0	0	0	0	0	0	0	0	0	0	0	0	0	0	0	0
0	0	0	0	0	259	3424	225	164	548	12950	310	40163	3940	459	1043
0	0	0	0	0	0	0	0	0	0	0	0	0	0	0	0
0	0	40	0	0	0	0	0	0	0	0	0	0	0	1855	1836
0	0	0	0	0	0	0	0	0	0	0	0	0	0	0	0
0	0	0	0	0	0	0	0	0	0	0	0	0	0	0	0
0	0	0	0	0	0	0	0	0	0	0	0	0	0	0	0
0	0	0	0	0	0	0	0	0	0	0	0	0	0	0	0
0	0	0	0	0	0	0	0	0	0	0	0	0	0	0	0
0	0	0	0	0	0	0	0	0	0	0	0	0	0	0	0
0	0	0	0	0	0	0	0	0	0	0	0	0	0	0	0
497	320	82074	61	569	350	425	41465	154	516	622	120	202	107	569	609
0	0	0	0	0	0	0	0	0	0	0	0	0	0	0	0
0	0	0	0	0	0	0	0	0	0	0	0	0	0	0	0
0	0	0	0	0	0	0	0	0	0	0	0	0	0	0	0
0	0	0	0	0	0	0	0	33	122	12953	0	83	0	0	0
0	0	0	0	0	0	0	0	0	0	0	0	0	0	0	0
0	0	0	0	0	0	0	0	31	124	0	0	0	0	86	0
0	0	0	0	0	0	0	0	0	0	0	0	0	0	0	0
0	0	0	0	0	0	0	0	0	0	0	0	0	0	0	0
0	0	0	0	0	0	0	0	0	0	0	0	0	0	0	0
0	0	0	0	0	0	0	0	0	0	0	0	0	0	0	0
0	0	0	0	0	0	0	0	0	0	0	0	0	0	0	0
0	3887	50	52	0	0	0	0	0	0	0	0	0	0	0	0
0	0	96	18737	0	0	0	0	0	0	0	0	0	0	0	0
0	0	0	0	0	0	0	0	0	0	0	0	0	0	0	0
0	0	0	0	0	0	0	0	0	0	0	0	0	0	0	0
0	0	0	0	0	0	0	0	0	0	0	0	0	0	0	0
0	0	0	0	0	0	0	0	0	0	0	0	0	0	0	0
0	0	0	0	0	0	0	101	0	240	0	0	16629	77	0	0
210	1229	390	162	18209	79	79	101	24164	355	8967	15	206	108	3234	249
0	0	0	0	0	0	0	0	0	0	35	38	43	0	0	0
781	0	0	0	0	0	0	0	0	0	0	0	0	0	0	0
0	0	0	0	0	0	0	0	0	0	0	0	0	0	0	0
0	0	0	0	0	0	0	0	0	0	0	0	0	0	0	0
0	0	0	0	0	0	0	0	0	0	0	0	0	0	0	0
0	0	0	0	0	0	0	0	0	0	0	0	0	0	0	0
0	0	27	0	0	371	0	32	34	0	1322	26	26467	17039	180	0
0	0	0	0	0	0	0	0	0	0	0	0	0	0	0	0
0	0	0	0	0	0	0	0	0	0	44	0	43	0	0	0
0	1357	31	0	115	98	5664	38	232	252	362	99902	117	72	108	115372
0	0	0	0	0	91	58	56	21	0	24436	38	92	31	0	0
0	0	0	0	0	0	0	0	0	0	0	0	0	0	0	0
0	139	0	0	0	0	0	0	0	0	0	0	0	0	0	0
644	0	0	0	0	0	0	0	0	0	0	0	0	0	0	0
0	53	0	0	0	100	0	0	58	119	100	23	22036	32	0	0
0	0	0	0	0	0	0	0	0	0	0	0	0	0	0	0
211	73	0	0	0	0	0	0	0	0	0	0	0	0	0	0
159	462	218	258	210	0	0	0	0	0	0	0	0	0	0	0
0	906	0	0	0	0	0	0	0	0	0	0	0	0	0	0
0	0	0	0	0	0	79	0	0	147	29	33	0	34	0	0
0	0	0	0	0	0	0	0	0	0	0	0	0	0	0	0
0	0	0	0	1076	0	0	0	0	0	0	0	0	0	0	0

0	55	0	0	0	0	0	0	0	0	0	0	0	0	0	0
0	0	0	0	0	0	0	0	0	0	0	0	0	0	0	0
0	0	0	0	0	0	0	0	0	0	0	0	0	0	0	0
0	0	0	0	0	0	0	0	0	0	0	0	0	0	0	0
47	1507	0	0	0	103	0	0	0	125	10540	41	41	0	0	0
0	0	0	0	0	0	0	0	0	0	0	0	0	0	0	0
0	0	0	0	0	0	0	0	0	0	0	0	0	0	0	0
0	0	0	0	0	0	0	0	287	0	0	0	0	0	105	0
0	0	0	0	0	0	0	0	0	0	0	0	0	0	0	0
0	0	0	0	0	0	0	0	0	0	0	0	0	0	0	0
0	0	0	0	0	0	0	0	0	0	0	0	0	0	0	0
0	0	0	0	0	0	0	0	0	0	0	0	0	0	86	0
221	0	0	0	0	0	0	0	0	0	0	0	0	0	0	0
0	0	0	0	0	0	0	0	0	0	0	0	0	0	0	0
0	0	0	0	0	0	0	0	0	0	0	0	0	0	0	0
676	458	233	249	232	0	0	0	27	0	0	0	0	0	98	0
0	0	0	0	0	1550	615	220	254	1396	746	369	42472	134	0	0
0	0	0	0	0	0	0	0	0	0	0	0	0	0	0	0
0	0	0	0	0	0	0	0	0	0	0	0	0	0	0	0
816	0	33	0	0	0	0	0	0	0	0	0	0	0	0	0
0	939	0	0	0	0	0	0	0	0	0	0	0	0	0	0
0	0	0	0	0	0	63	35	35	106	66	33	17602	41	0	0
0	0	0	0	0	0	0	0	0	0	0	0	0	34	0	0
0	0	0	0	0	0	0	0	0	0	0	0	0	0	0	0
0	0	0	0	0	0	0	0	0	0	0	0	0	0	0	0
852984	13970	98096	56376	66073	647347	15751	10235	217980	14858	8887	28577	41583	568543	129376	23662
0	40	31	12885	0	0	0	0	0	0	0	0	0	0	0	0
0	0	0	0	0	0	0	0	0	0	0	0	0	0	0	0
0	0	3088	0	0	2861	0	0	0	0	0	0	0	0	0	89
0	0	0	0	0	0	63	0	61	0	112	0	14929	0	0	0
0	0	0	0	0	0	0	86	0	0	0	0	0	0	0	0
0	0	198	0	0	93	0	141	33	17050	198	0	73	112	0	0
358	0	0	0	0	0	0	0	0	0	0	0	0	0	0	0

Control043	Control044	Control045	Control046	Control047	Control048	Control049	Control050	Control051	Control052	Control053	Control054	Control056	Control058	Control059	Control060
0	0	0	0	0	0	0	0	0	0	0	0	0	0	0	0
0	0	0	0	0	0	0	0	0	0	0	0	0	0	0	0
0	0	0	0	0	0	0	103	80	258	37	0	36	77	0	0
0	0	0	0	0	0	0	0	0	0	0	0	0	0	0	0
0	0	0	0	0	0	0	62	0	0	0	0	0	0	0	0
0	0	0	0	0	0	0	0	0	0	0	0	0	0	0	0
0	0	0	0	0	0	0	0	0	0	0	0	0	0	0	0
30724	9944	393220	4373	4555	15373	10189	856360	680712	37482	20683	9137	9468	25255	8329	15967
0	0	0	0	0	0	0	0	0	0	0	0	0	0	0	0
0	0	0	0	0	76	62	0	24	120	35	38	33	35	0	0
0	0	0	0	0	0	0	0	0	0	0	0	0	0	0	0
0	0	0	0	0	0	0	0	0	0	0	0	0	0	0	0
0	0	0	0	0	0	0	0	0	0	0	0	0	0	0	0
0	0	0	0	0	0	0	0	0	0	0	0	0	0	0	0
1051	15200	820	185710	577	85	2224	764	220	133	9041	49	130	37	503	1277
0	0	0	0	0	0	0	0	0	0	0	22	0	0	0	0
0	0	0	0	0	0	0	0	0	0	0	0	0	0	0	0
0	0	0	0	0	0	0	0	0	0	0	0	0	0	0	0
43	0	325	0	0	103229	144	5116	191	514	142	32	36	875	203	0
0	65	119	0	0	0	0	518	0	0	0	0	0	0	0	0
0	0	0	0	0	0	0	0	0	0	0	0	0	0	0	0
0	0	0	0	0	0	0	0	0	0	0	0	0	0	0	0
0	0	0	0	0	0	0	0	0	0	0	0	0	0	0	0

46	120	68	286	0	271	145	473	149	0	129	69	41744	140	412	157
0	0	0	0	0	0	0	0	0	0	0	0	0	0	0	0
0	0	0	0	0	0	0	0	0	0	0	0	0	0	0	0
0	0	77	55	60	0	0	0	0	0	180	55	0	35	280	281
0	0	0	0	0	0	0	27	0	0	0	0	0	0	0	0
58	10376	25	63	0	1434	17715	32	0	237	1376	180	0	25	97	86
0	1676	41	0	0	0	0	0	0	0	0	0	0	0	0	0
0	0	0	0	0	0	0	0	0	0	0	0	0	0	0	0
0	0	0	0	0	0	0	0	0	0	0	0	0	0	0	0
652	126	423	527	329	6578	77	212	29	245	117	88	43808	33	0	0
0	0	0	0	0	0	0	0	0	0	0	0	0	0	0	0
0	0	0	0	0	0	0	0	0	0	0	0	0	0	0	0
0	0	0	0	0	0	0	0	0	0	0	0	0	0	0	0
0	0	0	0	0	0	0	0	0	0	0	0	0	0	0	0
49	67	36	0	0	0	0	0	0	0	0	0	0	0	0	0
0	0	0	0	0	0	0	0	0	0	0	0	0	0	0	98
0	0	0	0	0	0	0	0	0	0	0	0	0	0	0	0
0	0	0	0	0	0	0	0	0	0	0	0	0	0	0	0
0	0	0	0	0	0	0	0	0	0	0	0	0	0	0	0
0	0	0	0	0	0	0	0	0	0	0	0	0	0	0	0
241	1152	6184	233	58	0	0	0	68	0	2451	0	0	0	5071	0
0	1734	0	0	0	0	0	0	0	0	0	0	0	0	85	0
43	0	38	14494	0	0	0	0	0	0	0	0	0	0	0	0
3400	205529	38496	1570	69009	10974	385331	9498	3019	618532	32483	10319	24915	17740	8222	4477
0	0	0	16076	0	0	0	0	0	0	0	0	0	0	0	0
0	0	5816	0	0	0	0	0	0	0	0	0	0	0	0	169
0	0	0	2582	0	0	0	0	64	116	86	0	35	0	2132	98
0	820	0	12438	57	0	0	0	0	0	40	0	0	0	0	0
0	0	0	0	0	0	0	0	0	0	0	0	0	0	0	0
0	0	0	0	0	0	0	0	0	0	0	0	0	0	83	0
0	0	0	0	0	0	0	0	0	0	38	0	0	0	0	0
849	0	0	0	0	0	82	71	86	0	43	35	0	0	0	0
48	0	109	64	0	0	0	0	0	0	0	0	0	0	0	0
0	0	0	0	0	0	0	0	0	0	0	0	0	0	0	0
0	59	112	0	20661	0	0	0	0	0	0	0	0	0	0	0
0	0	0	0	0	167	143	135	278	643	345	25	61242	121	0	0
0	0	0	0	684	0	0	0	0	0	0	0	0	0	0	0
0	0	0	0	0	0	81	0	1744	0	0	0	0	0	0	0
0	0	0	0	0	0	0	0	0	0	0	0	0	0	9329	0
0	0	0	0	0	1803	0	0	0	0	13520	146	39	0	0	0
0	0	0	0	0	0	0	0	0	0	0	0	0	0	0	0
0	0	0	0	0	0	0	0	0	0	0	0	0	0	0	0
0	0	0	0	0	0	0	0	0	0	0	0	0	0	0	0
0	49	0	0	0	0	0	0	0	0	0	0	0	0	0	0
0	0	0	0	0	0	0	0	0	0	0	0	0	0	0	0
0	0	0	0	0	0	0	0	0	0	0	0	0	0	0	0
0	0	0	0	0	0	0	0	0	0	0	0	0	0	0	85
0	0	0	0	0	0	0	0	0	0	35	0	11523	0	0	0
5407	334876	149006	1964	581844	7540	495960	774	843	2145	26058	11253	44156	1473	1519	4971
93	0	31	0	0	0	0	0	0	0	0	0	0	35	0	0
0	0	0	0	0	0	0	0	0	0	0	0	0	0	0	0
0	0	0	0	0	0	0	0	0	0	0	0	0	0	0	0
93	62482	136	50	0	0	0	0	0	0	0	0	0	0	0	0
0	0	0	0	0	0	0	0	0	0	0	0	0	0	84	0
0	0	0	0	0	0	0	0	0	0	0	0	0	0	0	0
0	0	0	0	0	0	0	0	0	0	0	0	0	0	0	0
0	0	41	0	0	0	0	0	0	0	0	33	0	0	0	0
0	0	0	0	0	0	0	0	121	0	0	0	0	0	0	0

0	0	0	0	0	0	0	1159	0	0	0	0	0	0	0	0
0	0	0	0	0	0	0	0	0	0	0	0	0	0	14312	0
0	0	0	0	0	0	0	0	0	0	0	0	0	0	0	0
0	0	34	55	0	0	550	139	349	0	188	34	39	37	0	263
0	0	0	0	0	0	0	0	0	0	0	0	0	0	0	0
0	0	0	0	0	0	0	0	0	0	0	0	0	0	0	0
0	0	0	0	0	184	504	129	164	598	364	0	107	165	103	249
0	0	0	0	0	0	0	0	0	0	0	0	0	0	0	0
0	0	0	0	0	0	0	0	0	0	0	0	0	0	0	0
145	48	135	90546	1525	53077	75	67	189	405	304	34561	29	36	0	0
0	0	0	0	0	0	0	0	0	0	0	0	0	0	41729	152
0	0	0	0	0	0	0	0	0	0	0	0	0	0	0	0
0	0	0	0	0	1543	1072	708	800	2531	1500	604	715	654	309	457
735	53	0	0	0	0	426	180	98	612	65	49	221	78	192	179
0	0	0	0	0	0	145	0	44	0	36	0	0	0	0	94
53	0	0	0	0	0	0	0	0	0	44	0	0	0	0	0
0	0	0	0	0	271	191	109	120	524	231	101	145	39	18932	0
851	0	0	71	0	0	0	0	0	0	0	0	0	0	21322	85
0	0	0	0	0	75	65	99	34	0	131	80	27317	0	0	0
0	0	0	0	0	0	0	0	0	0	0	0	0	0	0	0
0	0	0	0	0	4310	0	0	30	0	44	0	0	36	0	0
0	0	0	0	0	0	0	0	0	0	0	0	0	0	0	0
0	0	0	0	0	0	0	0	0	0	0	0	0	0	0	0
0	0	36	0	0	0	0	0	0	0	0	0	0	0	0	0
0	0	0	0	0	0	0	0	0	0	0	0	0	0	0	0
0	55	40	0	0	0	0	0	0	0	0	0	0	0	0	0
0	0	0	0	0	0	0	0	0	0	0	0	0	0	0	0
0	0	0	0	0	0	0	0	0	0	0	0	0	0	0	0
41	0	95	0	23121	0	0	0	0	0	0	0	0	0	0	0
0	0	0	0	0	0	0	0	0	0	0	0	0	0	13006	0
0	0	0	0	0	0	0	0	0	0	0	0	0	0	0	0
209	115033	182	232	484	355	587	0	92	1374	140	7265	718	76	93	9271
0	0	0	0	0	0	0	0	0	0	0	0	0	0	0	0
0	0	0	59	0	0	0	0	0	0	0	0	0	0	0	0
40	0	0	12101	0	0	0	0	0	0	0	0	0	0	0	0
1283	2502	3058	3517	225	208	84	101	2357	775	4630	131	3029	48029	46026	366
0	0	0	0	0	98	214	34	196	764	265	50	69542	84	0	0
112	8147	71	55610	117	107	152	24	82	485	126	27	39688	111	81	0
3592	585	452	44115	325	458	57	353	1066	906	12338	30	45740	21142	241518	2489
2884	1439	26035	552	64	1036	423	397	640	1630	14174	20	551	186980	6480	0
0	0	0	0	0	158	79	97	34	117	138	16740	826	108	16803	0
0	0	40	63675	0	0	0	0	0	0	0	0	0	0	0	0
0	0	0	0	0	0	0	0	0	0	0	0	0	0	0	0
0	0	0	0	0	0	0	0	0	0	0	0	0	0	0	0
0	0	0	0	0	0	0	0	0	0	0	0	0	0	0	0
0	0	0	0	0	0	0	0	0	0	0	0	0	0	0	0
701	0	0	0	0	0	0	0	0	0	0	0	0	0	0	0
0	0	0	0	0	0	0	0	0	0	0	0	0	0	0	0
0	0	0	0	0	0	0	0	0	0	0	0	0	0	0	0
0	0	0	0	0	0	0	0	0	0	0	0	0	0	0	0
0	0	0	0	0	0	0	0	0	0	0	0	0	0	0	0
54	0	0	115	0	0	0	0	0	0	0	0	0	0	0	90
0	0	0	0	0	0	0	0	0	0	0	0	0	0	0	0
0	0	0	0	0	98	0	0	0	0	0	0	0	0	5194	0
0	0	0	0	0	0	0	0	0	0	0	0	0	0	0	0
0	0	0	0	0	0	0	0	0	0	0	0	0	0	0	0
0	0	0	0	0	0	0	0	0	0	0	0	0	0	0	0
0	0	0	0	0	0	0	0	0	0	0	0	0	0	0	0

0	0	0	0	0	0	0	0	0	0	0	0	0	0	0	0
3851	23607	51681	110868	44498	557	532	885	1655	833	23873	26697	59680	30915	11215	1282
0	0	0	0	0	0	0	0	0	0	0	0	0	0	0	0
0	0	0	0	0	0	0	0	0	0	0	0	0	0	0	0
0	0	0	0	0	0	0	0	0	0	0	0	0	0	0	0
0	0	0	0	0	0	0	0	0	0	0	0	0	0	0	0
0	0	0	0	0	0	0	0	0	0	0	0	0	0	0	0
0	0	0	0	64	0	0	0	0	0	0	0	0	0	0	0
0	0	63	0	0	0	0	0	0	0	0	0	0	0	0	0
0	0	0	0	0	0	0	0	0	0	0	0	0	0	0	0
326	0	0	0	0	0	0	0	0	0	0	0	0	0	0	0
0	0	0	0	0	0	0	0	0	0	0	0	0	0	1699	0
0	0	0	0	0	0	0	0	0	0	0	0	0	0	0	0
0	0	0	0	0	0	0	0	0	0	0	0	0	0	0	0
0	0	0	0	0	0	0	0	0	0	0	0	0	0	0	0
0	0	0	0	0	0	0	0	0	0	0	0	0	0	0	0
0	0	0	0	0	0	0	0	0	0	0	0	0	0	0	0
0	0	0	0	0	0	0	0	0	0	0	0	0	0	0	0
0	0	0	0	0	0	0	0	0	0	0	0	0	0	0	0
0	0	0	0	0	85	431	210	375	876	457	121	101	95	108	164
0	0	0	0	0	0	0	0	0	0	0	0	0	0	0	0
0	0	0	0	0	0	213	0	0	0	0	0	0	0	0	0
0	0	0	0	0	0	0	0	0	0	0	0	0	0	0	0
0	0	0	0	0	0	0	0	0	0	0	0	0	0	14036	93
0	0	0	0	0	0	0	0	0	0	0	0	0	0	0	0
0	0	0	0	10882	0	0	126	38	0	176	0	35	31	0	0
0	0	0	0	0	0	0	0	0	0	0	0	0	0	0	0
0	0	0	0	0	0	0	0	0	0	0	0	0	0	0	0
748	607	439	103	311	0	145	0	0	0	0	0	0	0	0	90
0	0	0	0	0	0	0	0	0	0	0	0	0	0	0	0
0	0	0	0	0	0	0	0	0	0	0	0	0	0	0	0
0	0	0	0	0	0	0	0	0	0	0	0	0	0	0	0
0	0	0	0	0	0	0	0	0	0	0	0	0	0	0	0
0	0	0	0	0	0	0	0	0	0	0	0	30	0	0	0
0	0	0	0	0	0	0	0	0	0	0	0	0	0	0	0
0	0	0	0	0	0	0	0	0	0	0	0	0	0	88	0
0	0	0	0	0	0	0	0	0	0	0	0	0	0	0	0
0	0	0	0	0	0	0	0	0	0	0	0	0	0	7725	0
58	0	38	0	0	0	70	113	124	514	196	85	28	0	582	839
0	0	0	0	0	0	0	43	0	125	0	30	0	0	0	0
0	0	0	0	0	0	0	0	0	0	0	0	0	0	0	0
0	0	0	0	0	0	0	63	0	0	0	0	0	0	0	0
5005	174	6299	0	186	74617	1421	1151	4135	3980	685983	10836	49772	1255	917	7461
0	0	0	0	0	0	0	0	0	0	0	0	0	0	0	0
35409	51	72	60	0	0	0	0	0	0	0	0	0	0	0	0
0	0	0	0	0	0	0	0	0	0	0	0	0	0	0	0
0	0	0	0	0	0	0	0	0	0	0	0	0	0	0	0
0	0	0	0	0	0	0	0	58	0	0	0	0	0	0	0
0	0	0	0	0	0	0	0	0	0	0	0	0	0	0	0
0	0	0	0	0	0	0	0	0	0	0	0	0	0	0	0
0	0	0	0	0	0	0	0	0	0	0	0	0	0	9427	0
0	0	0	0	0	0	0	0	0	0	0	0	0	0	0	0
0	0	0	0	0	0	0	0	0	0	0	0	0	0	0	0
0	0	75	0	50	103	747	712	0	0	0	0	0	0	0	0
0	0	0	0	0	0	0	0	0	0	0	0	0	0	0	0
0	0	0	0	0	272	136	217	67	287	248	71	69	126	0	0
0	0	0	0	0	0	0	0	0	0	0	0	0	0	0	0
0	0	0	0	0	0	0	27	0	0	29	0	0	0	0	0

0	5554	38	0	0	0	0	0	0	0	0	0	0	0	0	0
0	0	0	0	0	0	0	0	0	0	0	0	0	0	0	0
0	0	0	0	0	0	0	0	0	0	0	0	0	0	0	0
0	0	0	0	0	0	0	0	0	0	0	0	0	0	0	0
0	0	0	0	0	0	0	0	0	0	0	0	0	0	0	0
0	0	0	0	0	0	0	0	0	0	0	0	0	0	0	0
356	377	21143	70	112	94	146	3633	31	0	193	26	0	0	107	0
0	8266	0	62	0	0	0	0	40	0	0	0	0	0	0	0
26612	0	39	58	56	0	0	0	0	0	0	0	0	0	0	0
0	0	0	0	0	0	0	0	0	0	0	0	0	0	0	0
0	0	0	0	0	0	0	0	0	0	0	0	0	0	0	0
0	0	0	0	0	0	0	0	0	0	0	0	0	0	0	0
0	60	0	0	0	0	0	0	0	0	0	0	0	0	113	165
0	0	0	0	0	0	0	0	0	0	0	0	0	0	0	0
808	7785	21938	17901	43827	31290	18990	36249	44166	273620	19951	27499	11276	52168	11950	15632
0	59	0	0	0	3881	0	43	0	0	0	0	0	0	0	0
0	0	0	0	95	17553	0	162	24	232	0	0	80	0	0	0
0	0	0	0	0	0	0	0	0	0	0	0	0	0	0	0
0	0	0	0	0	0	0	0	0	0	0	0	0	0	0	0
0	3708	0	0	0	83	835	0	0	0	0	0	0	0	0	0
0	0	0	0	0	0	0	0	0	0	0	0	0	0	0	0
0	17882	307	115	1313	338	16265	0	34	0	329	0	164	0	86	0
0	0	0	0	0	0	0	0	0	0	0	0	0	0	0	0
0	0	28	0	0	548	473	249	168	1156	388	308	170	243	14531	899
0	4865	38	0	0	0	0	0	0	0	0	0	0	0	0	0
56	0	0	0	57	0	0	0	33	0	0	0	498	39	0	88
0	0	0	0	0	0	0	0	0	0	0	0	0	0	0	0
0	0	0	0	0	0	143	0	0	0	40	0	0	0	0	104
0	0	105	0	0	0	0	578	0	0	0	0	0	0	0	0
0	0	0	0	0	0	0	0	0	0	0	0	0	0	0	0
0	0	0	0	0	0	0	0	0	0	0	0	0	0	0	0
0	0	0	0	0	0	0	0	0	0	0	0	0	0	0	0
0	0	0	0	0	0	0	0	0	0	0	0	0	0	0	0
274	247	105	214	218	0	0	0	0	0	0	0	0	0	3765	0
0	0	0	0	0	0	0	0	0	0	0	0	0	0	0	0
0	0	0	0	0	0	0	0	0	0	0	0	0	0	0	0
0	0	0	60	0	0	0	0	0	0	0	0	0	0	0	0
0	0	0	0	0	0	0	0	0	0	0	0	0	0	0	0
0	0	0	0	0	0	0	0	0	0	0	0	0	0	0	0
0	0	0	0	0	0	0	0	0	0	0	0	0	0	85810	160
0	0	0	0	0	0	0	0	349	0	0	0	0	0	0	0
0	0	0	0	0	0	0	0	0	0	0	0	0	0	0	0
0	0	0	0	0	0	0	0	0	0	0	0	0	0	0	0
0	0	0	0	0	0	0	0	0	0	0	0	0	0	0	0
256	831	264	0	0	444	0	1057	0	0	0	0	0	0	0	0
0	0	0	0	0	0	0	0	0	0	0	0	0	0	25208	0
0	0	0	0	0	0	0	0	0	0	0	0	0	0	0	0
0	0	0	0	0	0	0	0	0	0	32	0	0	0	0	0
0	0	0	0	0	0	0	0	0	0	0	0	0	0	0	0
0	0	37	0	0	0	0	0	0	0	0	0	0	0	0	0
0	0	10502	0	0	0	0	0	0	0	0	0	0	0	0	0
0	0	0	0	0	0	0	0	0	0	0	0	0	0	0	0
0	0	0	0	0	0	0	0	0	0	0	0	0	0	0	0
0	0	0	0	0	0	0	0	0	0	5724	0	0	0	0	0
0	0	128	0	0	84	71	0	269	0	70	0	4527	33	101	84
0	0	0	0	0	0	0	0	0	0	0	1335	0	0	0	0
0	0	0	0	0	0	0	0	0	0	0	0	0	0	0	0
0	0	0	0	0	0	0	0	0	0	0	0	0	0	0	0

0	0	0	0	0	0	0	35	0	0	69	0	0	0	0	0
0	0	0	0	0	0	0	0	58	0	0	0	0	0	0	0
0	0	0	0	0	0	0	0	0	0	11111	0	0	28	0	0
0	0	0	0	0	0	0	0	0	0	0	0	0	0	0	0
0	0	0	0	0	0	0	434	0	0	0	33	0	0	0	0
0	0	0	0	0	0	0	0	0	0	0	0	0	0	0	0
0	0	0	0	0	0	0	0	0	0	0	0	0	0	0	0
0	0	0	0	0	0	0	0	0	0	0	0	0	0	0	0
0	0	0	0	0	0	0	0	0	0	5180	0	43	0	0	0
0	0	0	0	0	0	0	0	0	0	0	0	0	0	0	0
333	59	0	0	56	0	0	0	0	0	0	0	0	0	0	0
0	0	0	0	0	0	0	0	0	0	0	0	0	0	0	0
0	0	26	60	50	0	0	0	0	0	0	0	0	0	111	182
0	0	0	0	53	0	0	0	0	0	0	0	0	0	97	244
0	0	0	0	0	0	0	0	0	0	0	0	0	0	0	0
0	0	0	0	0	0	0	0	0	0	0	0	0	0	0	0
0	0	0	0	0	0	0	0	0	0	0	0	0	0	0	0
0	72	20419	63	0	0	0	0	0	0	0	0	0	0	0	0
0	0	32	0	0	0	0	0	0	0	0	0	0	0	0	0
0	0	0	0	0	0	0	0	0	0	0	0	0	0	0	0
0	0	0	0	0	0	0	0	0	0	29	0	0	0	0	0
0	0	0	0	0	0	0	0	0	0	0	0	0	0	0	0
0	0	0	0	0	0	0	0	0	0	0	0	0	0	0	0
49	62	33538	0	183	288	79	233	34	391	33	110	47908	221	0	0
0	0	0	0	0	0	0	0	0	0	0	35	0	0	0	0
0	0	0	0	0	0	0	0	0	0	0	0	0	0	0	0
0	0	0	0	0	0	0	0	0	0	0	0	0	0	0	0
338	0	0	0	0	0	0	0	25	0	0	0	0	0	0	0
0	0	0	0	0	0	0	0	0	0	0	0	0	0	2923	0
0	0	65	0	0	0	0	0	0	0	0	0	0	0	0	0
0	0	0	0	0	0	0	0	0	0	0	0	0	0	0	0
0	2433	34	55	0	0	154	0	0	0	0	34	0	0	0	0
0	0	0	0	0	0	0	0	0	0	0	0	0	0	0	0
0	0	0	0	0	0	0	0	0	0	0	0	0	0	0	0
0	0	0	0	0	0	0	0	0	0	0	0	0	0	0	0
151	57	927	11539	2476	838	0	37	22	255	1768	29	14511	0	1528	0
0	0	0	0	0	0	0	0	0	0	0	0	0	0	0	0
8953	9592	1900	107695	14901	0	77	132	140	128	11998	32	26005	90	5647	360
192	705	65	13053	0	90	75	35	0	143	0	32	5949	0	0	0
0	0	0	0	0	0	0	0	0	0	0	0	0	0	0	0
0	0	0	0	0	0	0	0	0	0	0	0	0	0	0	0
0	0	0	0	0	0	0	0	0	0	0	0	0	0	0	0
0	0	0	0	0	0	0	0	0	0	0	0	0	0	0	0
0	0	0	0	0	0	0	0	0	0	0	0	0	0	0	0
0	0	0	0	0	0	0	0	0	0	0	0	0	0	301	0
729	452	797	500	78744	0	0	0	38	0	0	0	121	0	0	0
0	0	0	0	0	0	0	0	0	0	0	0	0	0	0	0
0	0	839	0	0	0	0	34	0	0	0	0	0	0	0	0
0	0	0	0	0	0	0	0	0	0	0	0	0	0	0	0
0	0	0	0	0	0	0	0	0	0	0	0	0	0	0	0
0	0	0	0	0	593	763	132	280	1188	726	372	210	297	371	85
0	0	0	0	0	0	0	0	0	0	0	0	0	0	0	0
0	0	0	0	0	0	0	0	0	0	0	0	0	0	0	0
0	0	0	0	0	0	0	0	0	0	0	0	0	0	0	0
0	0	0	0	0	0	0	0	0	0	0	0	0	0	0	0
0	0	0	0	0	0	0	0	0	0	0	0	0	0	0	0
88	2804	456	0	2707	106	5786	0	0	0	0	31	45	0	0	90
0	0	0	0	0	0	0	0	0	0	0	0	0	0	0	0

0	0	0	0	0	0	0	0	0	0	0	0	0	0	0	0
0	0	0	0	0	0	0	0	0	0	0	0	0	0	0	0
0	0	0	0	0	0	0	0	0	0	0	0	0	0	0	0
0	0	0	0	0	0	0	0	0	0	0	0	0	0	0	0
0	0	0	0	0	0	0	0	22	0	0	0	0	0	0	0
0	0	0	0	0	0	0	0	0	0	0	0	0	0	0	0
0	0	0	0	0	0	0	0	0	0	0	0	0	0	19727	0
0	0	0	0	0	0	0	0	0	0	0	0	0	0	0	0
0	0	0	0	0	0	0	0	0	0	0	0	0	0	0	0
0	0	0	0	0	0	0	0	0	0	35	0	0	0	0	0
0	0	0	100	55	0	0	0	0	0	0	0	0	0	0	0
0	0	0	0	0	0	0	0	0	0	0	0	0	0	0	0
0	0	0	0	0	0	0	0	0	0	0	0	0	0	95	0
0	134	75	107	124	0	0	0	0	0	0	0	0	0	0	0
60	2741	0	0	0	85	0	0	0	0	0	0	0	0	0	0
0	0	0	13385	0	0	0	0	0	0	0	0	0	0	0	0
0	0	0	0	0	0	0	0	0	0	0	0	0	0	0	0
0	0	0	0	0	0	0	0	0	0	0	0	0	0	0	0
0	0	0	0	0	0	0	0	0	0	0	0	0	0	0	0
0	0	0	0	0	0	0	0	0	0	0	0	0	0	0	0
167	4768	369	121	346	73	199	0	0	0	0	250	0	0	0	0
0	0	43	0	0	0	0	62	0	0	0	0	0	0	0	0
0	0	0	0	0	0	0	0	0	0	0	0	0	0	0	0
0	0	0	0	0	0	0	0	0	150	0	0	0	0	0	0
0	0	0	0	0	0	0	40	0	0	0	0	0	0	0	0
262	0	0	0	0	0	0	0	0	0	0	0	0	0	0	0
61	109	2287	60	0	253	228	9425	6296	257	112	2946	43	76	276	2837
0	0	0	0	0	0	0	0	0	0	0	0	0	0	0	0
0	0	0	0	0	0	0	0	0	0	0	0	0	0	0	0
0	0	0	0	0	0	0	0	0	0	0	0	0	0	9075	0
0	0	0	0	0	0	0	0	0	0	0	0	0	0	0	0
0	2428	123	0	152	82	4563	32	488	0	67	31	0	0	0	0
0	0	0	0	0	0	0	0	0	0	0	0	0	0	0	0
0	0	0	0	0	0	0	0	0	0	0	0	0	0	0	0
0	0	0	0	0	0	0	0	0	0	0	0	0	0	0	0
0	0	0	0	0	0	0	0	0	0	0	0	0	0	0	0
0	0	0	0	0	0	0	0	0	0	0	0	0	0	0	0
0	0	0	0	0	0	0	0	0	0	0	0	0	0	0	0
0	0	0	0	0	0	0	0	0	0	0	0	0	39	14642	0
0	0	0	0	0	0	0	0	0	0	0	0	0	0	2276	0
0	0	0	0	0	0	72	0	33	0	0	27	0	0	0	0
0	0	0	0	0	0	0	0	0	0	0	0	0	0	0	0
0	0	0	0	0	0	0	0	0	0	0	0	0	0	0	85
0	0	0	0	0	0	0	0	0	0	0	0	0	0	0	0
0	0	0	0	0	0	0	0	0	0	0	0	0	0	0	0
0	111	0	1802	0	0	0	0	0	135	0	0	3581	0	109	0
549	575	561	668	586	0	0	0	96	0	0	0	0	0	0	0
0	0	0	0	0	0	0	0	0	0	0	0	0	0	0	0
0	0	0	0	0	0	0	0	0	0	0	0	0	0	0	0
0	0	0	0	0	0	0	0	0	0	0	0	0	0	1635	0
0	0	0	0	0	0	78	63	110	115	77	0	32038	0	0	0
0	0	0	0	0	0	0	0	0	141	0	0	7634	0	0	0
0	0	0	0	0	0	0	39	0	0	0	0	0	0	0	0
0	0	0	0	0	0	0	0	0	0	0	0	0	0	0	0
0	0	0	0	0	0	0	0	0	0	0	0	0	0	0	0
0	0	0	0	0	0	0	0	0	0	0	0	0	0	0	0
0	0	0	0	0	0	0	0	0	0	0	0	0	0	0	0
0	0	382	58	0	87	68	5878	0	0	0	25	0	0	0	0

429	0	0	0	0	0	0	0	0	0	0	0	0	0	0	0
0	7755	47	0	0	0	0	0	0	0	0	0	0	0	0	0
0	0	0	0	0	0	0	0	0	0	0	0	0	0	0	0
0	0	0	0	0	0	0	0	0	0	0	0	0	0	0	0
0	0	0	0	0	0	0	0	0	0	0	0	0	0	0	0
0	0	0	0	0	0	0	0	0	0	0	0	0	0	0	0
0	0	0	0	0	0	0	0	0	0	0	0	0	0	0	0
0	0	0	0	0	0	0	0	0	0	0	0	0	0	0	0
0	0	0	0	0	0	0	0	0	0	0	0	0	0	0	0
0	65	72	0	0	0	0	0	0	0	0	0	0	0	0	0
0	0	0	0	0	89	0	0	0	0	0	0	0	0	30761	0
0	0	0	0	0	0	0	0	0	0	0	0	0	0	0	0
1469	0	0	0	0	0	0	0	0	0	0	37	0	0	0	0
0	0	0	0	0	0	0	0	0	0	0	0	0	0	0	0
0	0	0	0	0	0	0	0	0	0	0	0	0	0	0	0
0	0	0	0	0	0	0	0	0	0	0	0	0	0	0	0
0	0	0	0	0	90	0	0	0	0	0	0	0	0	0	0
0	0	0	0	0	0	0	889	0	0	0	0	0	0	0	0
0	0	0	0	0	0	0	0	0	0	0	0	0	0	0	0
0	0	0	0	0	0	0	0	0	0	0	0	0	0	0	0
0	0	0	0	0	0	65	39	75	0	103	0	0	26	111	108
0	0	0	0	0	0	0	0	0	0	0	0	0	0	0	0
0	0	0	0	0	0	0	0	0	0	0	0	0	0	0	0
0	0	0	0	0	0	0	0	0	0	0	0	0	0	0	0
0	0	0	0	0	0	0	0	0	0	0	0	0	0	0	0
0	0	0	0	0	0	0	0	0	0	0	0	0	0	0	0
0	0	0	0	0	0	0	0	0	0	0	0	0	0	0	0
0	0	131	0	170	0	0	0	0	0	0	0	0	0	106	78
358	54	262	146	49	0	0	0	405	0	0	0	0	40	752	692
0	0	0	0	0	0	0	0	0	0	0	0	0	0	0	0
44	0	0	0	0	0	0	0	0	0	0	0	0	0	0	0
0	0	0	0	0	0	0	0	0	0	0	0	0	0	0	0
0	0	0	62	0	0	0	0	0	0	0	0	0	0	0	0
0	0	0	0	0	0	0	0	0	0	0	0	0	0	0	0
0	0	0	0	0	0	0	0	0	0	0	0	0	0	0	84
0	0	0	0	0	0	0	0	0	0	0	0	0	0	0	0
0	0	0	0	0	0	0	0	0	0	0	0	0	0	0	0
0	0	0	0	0	0	0	0	0	0	35	0	0	0	2183	0
0	0	0	0	0	0	0	0	0	0	0	0	0	0	0	0
0	0	0	0	0	0	0	0	0	0	0	0	0	0	41522	192
0	0	0	0	0	0	0	301	0	0	0	0	0	0	0	0
0	0	0	0	0	0	0	0	0	0	0	0	0	0	0	0
0	0	1518	0	0	0	0	0	0	0	0	0	0	0	0	0
0	0	0	0	0	0	0	0	0	0	0	0	0	0	0	0
40	53	173	176	105	0	0	0	0	0	0	0	0	0	0	0
0	0	0	0	0	0	0	35	0	0	39	0	0	14260	0	0
0	0	0	0	0	92	0	0	0	0	0	0	0	0	18233	0
91	69	185	90684	52	0	0	0	0	0	0	0	0	0	0	0
0	0	0	0	0	0	0	0	0	0	0	0	0	0	0	0
0	0	0	0	0	0	0	0	0	0	0	0	0	0	0	0
0	0	0	0	0	0	0	0	0	0	0	0	0	0	0	0
0	0	0	0	0	0	69	0	0	0	0	0	0	0	0	0
0	697	2780	0	65	1883	1573	2639	1350	3019	1457	707480	1171	467	1132	783272
0	131	89	29845	0	0	0	0	0	0	0	0	0	0	0	0
0	0	0	0	0	0	0	0	0	0	0	0	0	0	0	0
0	0	0	0	0	0	0	0	0	0	0	0	0	0	59357	0
505	92398	1062	238	1288	830	675	0	98	126	335	37	87	0	480	0
0	0	0	0	0	0	0	0	0	0	0	0	0	0	0	0

0	0	33	0	5990	0	0	0	0	0	0	0	0	0	0	0
0	0	0	0	0	0	0	0	0	0	0	0	0	0	0	0
0	0	0	0	0	0	0	0	0	0	0	0	0	0	0	0
0	0	0	0	0	0	0	0	0	0	0	0	0	0	0	0
0	0	0	0	0	0	0	0	0	0	0	0	0	0	0	0
0	0	0	0	0	1597	1725	1066	1071	4600	2236	687	978	1008	0	0
0	0	0	0	0	0	0	0	0	0	0	0	0	0	0	0
0	0	0	0	0	0	0	0	0	0	0	0	0	0	0	0
0	0	0	0	0	0	0	0	0	0	0	0	0	0	0	0
59	0	0	44	0	0	0	0	0	0	0	0	0	0	0	0
0	0	0	0	0	90	71	221	30	483	261	66	83082	98	0	0
0	0	0	0	0	0	0	0	0	0	0	0	0	0	0	0
213	71	6939	115	0	75	64	60	212	142	2714	0	1551	4685	477	328
0	0	0	0	0	0	0	0	0	0	0	0	0	0	0	0
0	0	0	0	0	259	3424	225	164	548	12950	310	40163	3940	459	1043
0	0	0	0	0	0	0	0	0	0	0	0	0	0	0	0
0	0	40	0	0	0	0	0	0	0	0	0	0	0	1855	1836
0	0	0	0	0	0	0	0	0	0	0	0	0	0	0	0
0	0	0	0	0	0	0	0	0	0	0	0	0	0	0	0
0	0	0	0	0	0	0	0	0	0	0	0	0	0	0	0
0	0	0	0	0	0	0	0	0	0	0	0	0	0	0	0
0	0	0	0	0	0	0	0	0	0	0	0	0	0	0	0
0	0	0	0	0	0	0	0	0	0	0	0	0	0	0	0
0	0	0	0	0	0	0	0	0	0	0	0	0	0	0	0
0	0	0	0	0	0	0	0	0	0	0	0	0	0	0	0
0	0	0	0	0	0	0	0	0	0	0	0	0	0	0	0
0	0	0	0	0	0	0	0	0	0	0	0	0	0	0	0
0	0	0	0	0	0	0	0	0	0	0	0	0	0	0	0
0	0	0	0	0	0	0	0	0	0	0	0	0	0	0	0
497	320	82074	61	569	350	425	41465	154	516	622	120	202	107	569	609
0	0	0	0	0	0	0	0	0	0	0	0	0	0	0	0
0	0	0	0	0	0	0	0	0	0	0	0	0	0	0	0
0	0	0	0	0	0	0	0	0	0	0	0	0	0	0	0
0	0	0	0	0	0	0	0	33	122	12904	0	34	0	0	0
0	0	0	0	0	0	0	0	0	0	49	0	49	0	0	0
0	0	0	0	0	0	0	0	0	0	0	0	0	0	0	0
0	0	0	0	0	0	0	0	0	0	0	0	0	0	0	0
0	0	0	0	0	0	0	0	0	0	0	0	0	0	0	0
0	0	0	0	0	0	0	0	31	124	0	0	0	0	86	0
0	0	0	0	0	0	0	0	0	0	0	0	0	0	0	0
0	0	0	0	0	0	0	0	0	0	0	0	0	0	0	0
0	0	0	0	0	0	0	0	0	0	0	0	0	0	0	0
0	0	0	0	0	0	0	0	0	0	0	0	0	0	0	0
0	0	0	0	0	0	0	0	0	0	0	0	0	0	0	0
0	0	0	0	0	0	0	0	0	0	0	0	0	0	0	0
0	0	0	0	0	0	0	0	0	0	0	0	0	0	0	0
0	0	0	0	0	0	0	0	0	0	0	0	0	0	0	0
0	0	0	0	0	0	0	0	0	0	0	0	0	0	0	0
0	0	0	0	0	0	0	0	0	0	0	0	0	0	0	0
0	3887	50	52	0	0	0	0	0	0	0	0	0	0	0	0
0	0	96	18737	0	0	0	0	0	0	0	0	0	0	0	0
0	0	0	0	0	0	0	0	0	0	0	0	0	0	0	0
0	0	0	0	0	0	0	0	0	0	0	0	0	0	0	0
0	0	0	0	0	0	0	0	0	0	0	0	0	0	0	0
0	0	0	0	0	0	0	0	0	0	0	0	0	0	0	0
0	0	0	0	0	0	0	0	0	0	0	0	0	0	0	0
0	0	0	0	0	0	0	101	0	240	0	0	16629	77	0	0
153	1176	112	162	0	0	0	0	0	0	0	0	0	0	0	0

0	0	34	0	18107	0	0	0	32	257	6645	0	79	0	0	0
0	0	0	0	0	0	0	0	0	0	0	0	40	0	0	0
0	0	0	0	0	0	0	0	0	0	0	0	0	0	0	0
57	53	244	0	102	79	79	101	24132	98	227	15	87	108	3234	249
0	0	0	0	0	0	0	0	0	0	0	0	0	0	0	0
0	0	0	0	0	0	0	0	0	0	2095	0	0	0	0	0
0	0	0	0	0	0	0	0	0	0	35	38	43	0	0	0
781	0	0	0	0	0	0	0	0	0	0	0	0	0	0	0
0	0	0	0	0	0	0	0	0	0	0	0	0	0	0	0
0	0	0	0	0	0	0	0	0	0	0	0	0	0	0	0
0	0	0	0	0	0	0	0	0	0	0	0	0	0	0	0
0	0	0	0	0	0	0	0	0	0	0	0	0	0	0	0
0	0	27	0	0	371	0	32	34	0	1322	26	26467	17039	180	0
0	0	0	0	0	0	0	0	0	0	0	0	0	0	0	0
0	0	0	0	0	0	0	0	0	0	0	0	0	0	0	0
0	0	0	0	0	0	0	0	0	0	0	0	0	0	0	0
0	0	0	0	0	0	0	0	0	0	44	0	43	0	0	0
0	1357	31	0	115	98	5664	38	232	252	362	99902	117	72	108	115372
0	0	0	0	0	0	0	0	0	0	0	0	0	0	0	0
0	0	0	0	0	91	58	56	21	0	24436	38	92	31	0	0
0	0	0	0	0	0	0	0	0	0	0	0	0	0	0	0
0	0	0	0	0	0	0	0	0	0	0	0	0	0	0	0
0	0	0	0	0	0	0	0	0	0	0	0	0	0	0	0
0	139	0	0	0	0	0	0	0	0	0	0	0	0	0	0
644	0	0	0	0	0	0	0	0	0	0	0	0	0	0	0
0	0	0	0	0	0	0	0	0	0	0	0	0	0	0	0
0	0	0	0	0	0	0	0	0	0	0	0	0	0	0	0
0	0	0	0	0	100	0	0	58	119	100	23	22036	32	0	0
0	0	0	0	0	0	0	0	0	0	0	0	0	0	0	0
0	0	0	0	0	0	0	0	0	0	0	0	0	0	0	0
0	53	0	0	0	0	0	0	0	0	0	0	0	0	0	0
0	0	0	0	0	0	0	0	0	0	0	0	0	0	0	0
0	0	0	0	0	0	0	0	0	0	0	0	0	0	0	0
0	73	0	0	0	0	0	0	0	0	0	0	0	0	0	0
211	0	0	0	0	0	0	0	0	0	0	0	0	0	0	0
159	462	218	258	210	0	0	0	0	0	0	0	0	0	0	0
0	906	0	0	0	0	0	0	0	0	0	0	0	0	0	0
0	0	0	0	0	0	79	0	0	147	29	33	0	34	0	0
0	0	0	0	0	0	0	0	0	0	0	0	0	0	0	0
0	0	0	0	0	0	0	0	0	0	0	0	0	0	0	0
0	0	0	0	1076	0	0	0	0	0	0	0	0	0	0	0
0	0	0	0	0	0	0	0	0	0	0	0	0	0	0	0
0	55	0	0	0	0	0	0	0	0	0	0	0	0	0	0
0	0	0	0	0	0	0	0	0	0	0	0	0	0	0	0
0	0	0	0	0	0	0	0	0	0	0	0	0	0	0	0
0	0	0	0	0	0	0	0	0	0	0	0	0	0	0	0
47	1507	0	0	0	103	0	0	0	125	10540	41	41	0	0	0
0	0	0	0	0	0	0	0	0	0	0	0	0	0	0	0
0	0	0	0	0	0	0	0	0	0	0	0	0	0	0	0
0	0	0	0	0	0	0	0	287	0	0	0	0	0	105	0
0	0	0	0	0	0	0	0	0	0	0	0	0	0	0	0
0	0	0	0	0	0	0	0	0	0	0	0	0	0	0	0
0	0	0	0	0	0	0	0	0	0	0	0	0	0	0	0
0	0	0	0	0	0	0	0	0	0	0	0	0	0	86	0
221	0	0	0	0	0	0	0	0	0	0	0	0	0	0	0
0	0	0	0	0	0	0	0	0	0	0	0	0	0	0	0
0	0	0	0	0	0	0	0	0	0	0	0	0	0	0	0
0	0	0	0	0	0	0	0	0	0	0	0	0	0	0	0

0	0	0	0	0	0	0	0	27	0	0	0	0	0	0	0
0	0	0	0	0	0	0	0	0	0	0	0	0	0	98	0
0	0	0	0	0	0	0	0	0	0	0	0	0	0	0	0
676	458	233	249	232	0	0	0	0	0	0	0	0	0	0	0
0	0	0	0	0	77	0	31	30	381	77	65	51	0	0	0
0	0	0	0	0	452	476	122	193	1015	591	210	16161	75	0	0
0	0	0	0	0	0	68	67	31	0	0	71	0	0	0	0
0	0	0	0	0	1021	71	0	0	0	78	23	26260	59	0	0
0	0	0	0	0	0	0	0	0	0	0	0	0	0	0	0
0	0	0	0	0	0	0	0	0	0	0	0	0	0	0	0
816	0	33	0	0	0	0	0	0	0	0	0	0	0	0	0
0	939	0	0	0	0	0	0	0	0	0	0	0	0	0	0
0	0	0	0	0	0	63	35	35	106	66	33	17602	41	0	0
0	0	0	0	0	0	0	0	0	0	0	0	0	34	0	0
0	0	0	0	0	0	0	0	0	0	0	0	0	0	0	0
0	0	0	0	0	0	0	0	0	0	0	0	0	0	0	0
0	0	0	0	0	0	0	0	0	0	0	0	0	0	0	0
0	0	0	0	0	0	0	0	0	0	0	0	0	0	0	0
0	0	0	0	0	0	0	0	0	0	0	0	0	0	0	0
0	0	2365	0	0	78	0	37	0	0	0	0	0	0	0	0
852984	13970	95731	56376	66073	647269	15751	10198	217980	14858	8887	28577	41583	568543	129376	23662
0	40	31	12885	0	0	0	0	0	0	0	0	0	0	0	0
0	0	0	0	0	0	0	0	0	0	0	0	0	0	0	0
0	0	3088	0	0	2861	0	0	0	0	0	0	0	0	0	89
0	0	0	0	0	0	63	0	61	0	112	0	14929	0	0	0
0	0	0	0	0	0	0	86	0	0	0	0	0	0	0	0
0	0	198	0	0	93	0	141	33	17050	198	0	73	112	0	0
358	0	0	0	0	0	0	0	0	0	0	0	0	0	0	0
0	0	0	0	0	0	0	0	0	0	0	0	0	0	0	0
0	0	0	0	0	0	0	0	0	0	0	0	0	0	0	0
0	0	0	0	0	0	0	0	60	0	0	0	0	0	0	0
0	0	0	0	0	0	0	0	0	0	0	0	0	0	0	0
0	0	0	0	0	0	0	0	0	0	0	0	0	0	0	0
0	0	526	0	0	0	0	2145	0	0	0	0	0	0	0	81

Control061	Control062	Control064	Control065	Control067	Control068	Control069	Control071	Control074	Control076	Control077	Control079	Control081	Control082	Control084	Control085
843348	921593	729007	853189	822083	917230	649580	854474	796699	798800	727860	782462	752805	924585	994748	924941
94323	36213	238576	100705	147581	71215	238791	69837	100150	136468	195153	139555	147683	30469	2890	40498
0	0	0	0	0	0	0	0	0	0	0	338	0	0	0	0
56817	25465	32329	34991	28862	11283	107129	67850	103098	64670	53921	75644	98682	42972	1577	21647
396	15889	88	10732	1422	235	4221	7839	53	62	23010	2001	830	1974	785	10138
4210	0	0	0	0	37	0	0	0	0	0	0	0	0	0	0
906	840	0	383	52	0	279	0	0	0	56	0	0	0	0	2776

Control061	Control062	Control064	Control065	Control067	Control068	Control069	Control071	Control074	Control076	Control077	Control079	Control081	Control082	Control084	Control085
44	0	0	0	8427	0	0	161	0	42	0	0	0	0	0	56
0	0	0	0	0	0	0	0	0	0	17644	0	0	0	0	0
403938	142656	77624	144290	12335	14716	111998	312430	16521	571	98034	494243	11210	536011	7760	303926
0	0	0	0	0	0	0	0	0	0	0	0	0	0	0	0
207	231	88	262	123	43	0	0	0	0	0	0	199	0	0	0
0	0	0	0	0	0	0	0	0	0	0	0	0	0	0	0
0	0	0	0	0	0	0	0	0	0	0	0	0	0	0	0
0	0	0	0	0	0	0	0	0	0	0	0	0	0	0	0
0	0	0	0	0	0	0	0	0	0	0	0	0	0	0	0
82212	5238	358	14070	287	89	371	0	0	0	0	46	109	0	46	2089
0	0	0	0	0	0	0	0	0	0	0	0	0	0	0	0
0	0	0	0	0	0	0	0	0	0	0	0	0	0	0	0
0	0	0	0	0	0	0	0	0	0	0	0	0	0	0	0
2446	303	21260	977	76	47	0	1367	4030	0	7293	107	0	8643	0	837
257	0	48	0	0	0	86	0	0	0	0	0	0	79	0	37
0	0	0	0	0	0	0	0	0	0	0	0	0	0	0	0
0	0	0	0	0	0	0	0	0	0	0	0	0	0	0	0
0	204	27769	2871	131	11591	94	0	0	0	0	0	0	0	0	2951
0	0	0	0	0	0	0	0	0	0	0	0	0	0	0	0
0	0	0	0	0	0	0	0	0	0	0	0	0	0	0	0
83	206	141	268	269	45	741	424	259	96	1609	602	674	1091	974627	1340
0	0	0	0	0	0	0	0	0	0	0	0	94	0	0	0
38	3072	41	0	76	0	0	3781	0	0	0	0	96	0	156	6779
0	0	0	0	0	0	0	0	0	0	0	0	0	0	0	0
0	0	0	0	0	0	0	0	0	0	0	0	0	0	0	0
7884	43132	1393	238044	9667	4114	59542	35642	12254	89	288201	8935	599	7012	2239	181864
0	0	0	6954	0	0	81	0	0	0	0	0	0	0	0	0
0	0	0	0	0	0	0	0	0	0	0	0	0	0	0	0
0	0	0	0	0	0	0	0	0	0	0	0	0	0	0	0
0	0	0	0	0	0	0	0	0	0	0	0	0	0	0	0
0	0	0	0	0	0	0	0	0	0	0	0	0	0	0	0
0	0	0	0	0	0	0	0	0	0	0	326	0	1964	0	0
0	0	0	46	36	0	84	0	0	0	0	0	0	0	0	0
0	0	0	0	0	0	0	0	0	0	0	0	0	0	0	163
0	0	0	0	0	0	0	0	0	0	0	0	0	0	0	0
0	0	0	0	0	0	0	0	0	0	0	0	0	0	0	0
0	0	0	0	0	0	0	0	0	0	0	0	0	0	0	0
0	0	0	0	0	0	0	0	0	0	49	43	152	57	42	118
0	0	0	0	0	0	0	0	0	0	0	0	0	0	0	0
282	40073	671	536	826	1513	881	10471	1618	0	51213	426	394	564	615	277103
0	0	0	0	0	0	0	0	0	0	0	0	0	0	63	0
0	0	0	0	0	0	0	0	0	0	0	0	0	0	0	1682
50	0	0	69	78	51	75	0	0	0	0	0	0	0	0	0
0	0	0	0	0	0	0	0	0	0	0	0	0	0	168	0
0	0	0	0	0	0	0	0	0	0	0	0	0	0	0	0
179	0	44	0	0	0	0	0	0	0	0	0	0	0	0	0
227	0	0	0	0	0	76	307	0	0	0	0	0	3151	0	2998

44	71	51	49	0	47	0	0	0	0	0	0	0	0	0	0
0	0	0	0	0	0	0	0	0	0	0	0	0	0	0	0
577	901	506	1544	625	274	2115	165	219	182	0	109	153	318	0	115
0	153	0	0	6784	0	0	0	0	0	0	0	0	0	0	0
0	0	0	0	0	0	0	0	0	0	0	0	0	0	0	0
0	0	0	0	0	0	0	0	0	0	0	0	0	0	0	0
0	0	0	0	0	0	0	0	0	0	0	0	0	0	0	0
6293	0	0	129	0	0	0	0	0	0	114	0	0	0	0	0
0	0	0	0	0	0	0	0	0	0	58	26385	0	57	61	0
36	0	46	0	0	0	79	0	0	0	0	0	0	0	0	0
0	0	0	0	0	0	0	0	0	0	0	0	0	0	0	0
0	0	0	0	0	0	0	0	0	0	0	0	0	0	0	0
0	0	0	0	0	0	0	0	0	0	0	0	0	0	0	0
0	0	0	0	33	0	86	0	0	0	0	0	0	0	0	0
0	0	0	0	0	0	0	0	0	0	0	0	0	0	0	0
317	2476	710	170	131	1108	0	639	272	362	4258	0	0	228	50	583
0	0	0	0	0	0	0	0	0	0	0	338	0	0	0	0
483	0	0	0	0	0	0	0	0	0	0	101	0	2383	0	32
0	0	0	0	0	0	0	0	0	0	0	0	0	0	0	0
16542	15593	31299	1505	100616	19718	979	71989	50	40	25371	13139	36698	21746	945	1333
0	0	0	0	0	0	0	0	0	0	0	0	0	0	0	0
0	0	0	0	0	0	0	0	0	0	0	0	0	0	0	0
0	0	0	0	0	0	0	0	0	0	0	0	0	0	0	0
0	0	0	0	0	0	0	0	0	0	0	0	0	0	0	0
0	0	0	0	0	0	0	0	0	0	0	0	0	0	0	0
0	68	0	0	0	0	0	0	0	0	0	0	0	0	0	0
0	0	0	0	0	0	0	0	0	0	0	0	0	0	0	0
0	0	0	0	0	0	0	0	0	0	0	0	0	0	0	0
0	0	0	104	0	0	0	0	59764	161	62	110	72	62	0	0
0	0	0	0	0	0	0	0	0	0	54	0	33914	0	0	29
0	0	0	0	0	0	0	0	0	0	0	0	0	0	0	0
0	0	0	0	0	0	0	0	0	0	0	0	0	0	0	0
0	0	0	0	0	0	0	0	0	0	0	0	0	0	0	0
5500	4650	153904	68840	55228	24729	1359	55547	39648	135741	35854	356	25906	4656	796	15248
0	0	0	0	0	0	0	0	0	0	0	0	0	0	0	0
0	0	0	0	0	0	0	0	0	0	0	0	0	0	0	0
0	0	0	0	48	0	0	0	0	0	0	0	0	0	0	78
0	0	0	0	0	0	0	0	0	0	0	0	0	0	0	0
0	0	0	0	0	0	0	0	0	0	0	0	0	0	0	0
0	0	0	0	0	0	0	0	0	0	0	0	0	3852	0	0
0	0	0	0	0	0	0	0	0	0	0	0	0	0	0	0
0	0	0	0	0	0	0	0	0	0	0	0	0	0	0	0
0	0	0	0	0	0	0	0	0	0	0	0	0	0	0	0
95	326	63	119	116	85	177	0	0	0	0	0	0	0	0	0
0	0	0	0	35	0	0	0	0	0	0	0	0	0	0	0
0	0	0	0	0	0	0	0	0	0	0	0	0	0	0	48
0	0	0	0	0	0	0	0	0	0	0	0	0	0	0	0
0	0	44	52	38	36	91	0	0	0	0	0	0	0	0	0
0	0	0	0	0	0	0	152	104	133	0	0	0	0	0	0
0	0	0	0	0	0	0	0	0	0	347	0	36	0	0	76
0	0	0	0	0	0	0	0	0	0	0	0	0	0	0	0
5442	0	0	0	0	0	0	0	0	0	0	0	0	0	0	0
0	56	0	47	0	0	0	0	0	0	0	0	0	0	0	0
554	589	449	520	292	314	705	0	0	0	0	0	0	60	0	0
0	0	0	0	0	0	0	0	0	0	0	0	0	0	0	0
0	15510	0	10324	1196	38	3889	7839	53	62	22885	2001	631	1974	785	10138
0	0	0	0	0	0	0	0	0	0	59	0	0	0	0	0
0	0	0	0	0	0	0	0	0	0	0	0	0	0	0	0

0	0	0	0	0	0	0	0	0	0	0	0	0	0	0	0
0	0	0	0	0	0	0	0	0	0	0	0	0	0	0	0
0	0	0	0	0	0	0	0	0	0	0	0	0	0	0	0
0	0	0	0	0	0	0	0	0	0	0	0	0	0	0	0
0	0	0	0	0	0	0	0	0	0	0	0	0	0	0	0
0	0	0	0	0	0	0	0	0	0	3925	0	0	0	0	0
0	0	0	0	0	0	0	0	0	0	0	0	0	0	0	0
0	0	0	0	0	0	0	0	0	0	0	0	0	0	0	345
0	0	0	0	0	0	0	0	0	0	0	0	0	0	0	0
0	0	0	0	0	0	0	0	0	0	12343	49	0	0	58	50
0	0	0	0	0	0	0	0	0	0	0	0	0	0	0	0
0	0	0	0	0	0	0	0	0	0	0	0	0	0	0	0
0	0	0	0	0	0	0	0	0	0	0	0	0	0	0	0
0	0	0	0	0	0	0	0	0	0	0	0	0	0	0	0
0	0	0	0	0	0	0	0	0	0	0	0	0	0	0	0
5373	140	45	224	102	131	72393	0	0	0	0	58	38	2847	0	9088
972	0	183	125	0	39	178	449	0	0	58	95	52	1380	0	681
940	0	0	0	0	0	0	0	0	0	0	0	0	0	0	0
0	0	0	0	0	0	0	0	0	0	0	0	0	0	0	0
0	0	0	0	0	0	0	0	0	0	0	2637	0	0	0	0
0	162	48839	161	65	26	191	0	0	0	0	0	0	0	0	0
0	0	0	0	0	0	0	0	0	0	0	0	0	0	0	0
56817	25465	32329	34991	28862	11283	107129	67850	103098	64670	53921	75644	98682	42972	1577	21302
0	0	0	0	0	0	0	133	35061	30	177	0	31	0	99	3334
0	232	0	0	0	0	0	0	0	0	0	60	28	0	59	4820
0	0	0	0	0	0	0	0	0	0	0	0	0	0	0	0
191	5983	83	210	210	156	616	0	0	0	0	45	0	0	0	0
0	0	0	0	0	0	0	0	0	0	0	0	0	0	0	0
0	0	0	0	36	0	273	0	0	0	106	45	0	0	0	44
0	154	0	0	0	0	0	0	0	0	0	0	0	0	0	0
1152	0	0	0	0	0	0	0	0	0	0	0	0	114	0	154
0	0	0	0	0	0	0	0	0	0	0	0	0	0	0	904
0	0	0	0	0	0	0	0	0	0	0	0	0	0	0	0
0	0	0	0	0	0	0	0	0	0	0	0	0	0	0	0
52	58	96	40	41	21838	271	0	0	0	0	0	0	0	0	0
0	0	0	0	0	0	0	0	0	0	0	0	0	0	0	0
0	0	0	0	0	0	0	0	0	0	0	0	0	0	0	0
0	0	0	0	0	0	0	0	0	0	0	0	0	0	0	0
89	0	61	78	86	75	0	143	0	0	0	0	0	0	0	0
0	0	0	0	0	0	0	0	0	0	0	0	0	0	0	0
0	0	0	0	0	0	0	0	0	0	0	0	0	0	0	0
3829	211	473	95	39	0	353	1201	0	0	0	481	42	3484	0	1219
0	64	79	0	42	0	84	0	0	0	0	0	0	0	0	0
0	0	0	0	0	0	0	0	0	0	5893	35	0	0	0	0
0	0	0	0	0	0	0	0	0	0	0	0	0	0	0	0
0	0	0	0	0	0	0	0	0	0	0	0	0	0	0	0
0	0	0	41	0	0	0	0	0	0	0	0	0	0	0	0
0	0	0	0	0	0	0	0	0	0	0	0	0	0	0	0
0	0	0	0	0	0	0	0	0	0	0	0	0	0	0	0
0	0	0	0	0	0	0	0	0	0	0	0	0	0	0	0
0	0	0	0	0	0	0	0	0	0	0	0	0	0	0	0
0	0	0	0	0	0	0	0	615	201	257	57	1708	74	0	85
0	0	0	0	0	0	0	0	0	0	0	0	0	0	0	0
0	0	0	0	0	0	0	0	0	0	0	0	0	65	0	0
0	0	0	0	0	0	0	0	0	0	0	0	0	0	0	0
60	0	0	0	0	0	0	0	0	0	0	0	0	0	0	0
0	0	0	0	0	0	0	0	0	0	0	0	0	0	0	0
0	0	0	0	0	0	0	0	0	0	0	0	0	0	0	0

0	0	0	0	0	0	0	0	0	0	0	0	121	1331	0	2657
0	0	0	0	0	0	0	0	0	0	59	0	0	0	0	40
0	0	0	0	0	0	0	0	0	0	0	0	0	0	0	0
0	0	0	0	0	0	0	0	0	0	0	0	0	0	0	0
6523	7091	0	36	87	48	0	0	35469	80	0	0	0	0	0	0
0	0	0	0	0	0	0	0	0	0	0	0	0	0	0	0
0	0	0	0	0	0	0	0	0	0	0	0	0	0	0	0
0	0	0	0	0	0	0	0	0	0	54	0	0	0	0	5841
0	0	0	0	0	0	0	0	0	0	0	0	0	0	0	0
0	0	0	0	0	0	0	0	0	0	0	0	0	0	0	0
0	0	0	0	0	0	0	0	0	0	0	0	0	0	0	0
0	0	0	0	0	0	0	4056	0	0	0	0	0	0	0	0
0	0	0	0	0	0	0	0	0	0	0	0	0	0	0	0
0	0	0	0	0	0	0	0	0	0	0	0	0	0	0	0
0	0	0	39	0	0	0	0	0	0	0	0	0	0	0	0
0	0	0	0	0	0	0	0	0	0	0	0	0	0	0	0
0	0	0	0	0	0	0	0	0	0	0	0	0	0	0	0
0	0	0	0	42	0	0	0	0	0	0	0	0	0	0	0
0	0	0	0	0	0	0	0	0	0	0	0	0	0	0	0
0	0	0	0	0	0	0	0	0	0	0	0	27455	68	47	44
5392	3890	6400	12647	77292	9006	77216	8287	738	497	136551	71760	42619	13092	920	7156
0	0	0	0	0	0	0	0	0	0	0	0	0	0	0	0
0	0	0	0	0	0	0	0	0	0	0	0	0	0	0	0
0	0	0	31	0	0	0	0	0	0	0	0	0	3222	0	0
0	0	0	0	0	0	0	0	0	0	0	0	0	0	0	541
0	0	0	0	0	39	0	139	44	32	58	9406	44	53	185	0
0	0	0	0	0	0	0	0	0	0	0	0	0	0	0	256
0	0	0	0	0	0	0	0	0	0	0	0	0	0	0	230
60	0	0	0	0	0	0	0	0	0	0	0	0	0	0	0
189	148	0	146	103	154	332	0	0	0	125	0	0	0	0	0
0	0	0	0	0	0	0	0	0	0	0	0	0	0	0	0
0	0	0	0	0	0	0	0	0	0	0	0	0	1467	0	0
5180	0	0	0	0	0	0	0	0	0	0	0	0	0	0	0
0	0	0	0	0	0	0	0	0	0	0	0	0	0	0	0
0	0	0	0	0	0	80	0	0	0	0	0	0	0	0	2670
0	0	0	0	0	0	0	0	0	0	0	0	0	0	0	0
0	0	0	0	0	0	0	0	0	0	0	0	0	0	0	0
0	0	0	0	0	0	0	0	0	0	0	0	0	0	0	0
0	0	0	0	0	0	0	0	0	0	0	0	0	0	0	0
0	0	0	0	0	0	0	0	0	0	0	0	0	0	0	0
0	0	0	0	0	0	0	0	0	0	0	0	0	0	0	0
49	0	0	0	0	0	0	0	0	0	0	0	0	0	0	0
0	0	0	0	0	0	0	0	0	0	0	0	0	0	0	0
0	0	0	0	49	0	0	0	0	0	0	0	0	0	0	0
0	0	0	0	0	0	0	0	0	0	0	0	0	0	0	0
0	0	0	0	0	0	0	0	0	0	0	14008	0	0	0	4470
0	0	0	0	0	0	0	0	0	0	0	0	2865	0	0	43
0	0	0	0	0	0	0	0	0	0	2553	0	0	0	0	0
0	0	0	0	0	0	0	0	0	0	0	0	0	0	0	0
0	0	0	0	0	0	0	0	0	0	0	0	0	0	0	0
0	0	0	0	0	0	0	0	0	0	0	0	0	0	0	0
0	0	0	0	0	0	0	0	0	0	0	0	0	0	0	0
0	0	0	0	0	0	0	0	0	66	0	0	0	0	0	0
0	0	0	0	0	0	0	160	0	0	583	0	0	55	0	335
0	0	0	0	0	0	0	0	0	0	0	0	0	0	0	0
0	0	0	0	0	0	0	0	0	0	0	0	0	0	0	0
5166	1433	3215	1438	87	215	1243	6867	2166	2797	2091	1725	2241	4346	1054	3206
0	0	0	0	0	0	0	0	0	0	0	0	0	0	0	0

0	0	0	0	0	0	0	0	0	0	0	0	0	0	0	0
0	0	0	0	0	0	0	0	0	0	0	0	0	0	0	0
0	0	0	0	0	0	0	0	0	0	0	0	0	0	55	64
0	0	0	0	0	0	0	0	0	0	0	0	0	0	0	1046
0	56	0	32	0	0	0	0	0	0	0	0	0	0	0	0
0	0	0	0	0	0	0	0	0	0	0	0	0	0	0	0
0	0	0	0	0	0	0	0	0	0	0	0	0	0	0	0
5333	144	42	36	0	0	0	0	0	0	0	0	39	458	0	66
0	0	0	0	0	0	0	0	0	0	0	11698	0	0	35	0
0	0	0	0	0	0	0	0	0	0	46	0	46	0	0	78
2506	84	0	86	37	0	102	0	0	0	0	1618	37	1978	0	318
0	0	0	0	0	0	0	0	0	0	0	0	0	0	0	0
0	0	0	0	0	0	0	0	0	0	0	0	0	0	0	0
0	0	0	0	0	0	0	0	0	0	0	0	0	0	0	0
0	0	0	0	0	0	0	0	0	0	0	0	0	0	0	0
0	0	0	0	0	0	0	0	0	0	0	0	0	0	0	0
0	0	0	0	0	0	0	0	0	0	0	0	0	0	0	0
54	0	0	173	38	0	0	0	0	0	0	0	45	4697	0	80
0	0	0	0	0	0	0	0	0	0	0	0	0	0	0	0
0	0	0	0	0	0	0	0	0	0	0	0	0	0	0	0
0	0	0	0	0	0	0	0	0	0	0	0	0	0	0	0
0	0	0	0	0	0	0	0	0	0	0	0	0	0	0	0
0	0	0	0	0	0	0	0	0	0	0	0	136	0	0	41
0	0	92	43	0	0	0	0	0	0	0	0	0	0	0	0
0	72	0	124	35	0	0	0	0	0	0	0	0	0	0	0
0	0	0	0	0	37	0	0	0	0	0	0	0	0	180	0
0	0	0	0	0	0	0	0	0	0	0	0	0	0	0	0
64841	542	583	606	430	508	158448	154	0	69	62	25170	0	0	154	79
0	0	0	0	0	0	0	0	0	0	0	0	0	0	0	0
0	0	0	0	0	0	0	0	0	0	0	0	0	0	0	0
0	0	0	0	0	0	0	0	0	0	0	0	0	0	0	0
0	0	0	0	0	0	0	0	0	0	0	0	0	0	0	0
38	0	0	0	12044	0	0	0	0	0	0	0	0	0	0	0
0	0	0	0	0	0	0	0	0	0	0	0	0	0	0	0
0	0	0	0	0	0	0	0	0	0	0	0	0	0	0	0
0	0	0	50	0	0	0	0	0	0	0	0	0	0	0	0
0	0	0	0	0	0	0	0	0	0	0	0	0	0	0	0
0	69	0	0	44	45	0	0	0	0	0	0	0	0	0	0
0	0	0	0	0	0	0	0	0	0	0	0	0	0	0	0
0	0	0	0	0	0	0	0	0	0	0	0	0	0	0	0
0	0	0	0	0	0	0	0	0	0	0	0	0	0	0	0
0	0	0	0	0	0	0	0	0	0	0	0	0	0	0	0
0	0	0	0	0	0	0	0	0	0	0	0	0	0	0	0
0	0	0	0	0	0	0	0	0	0	0	0	0	0	0	0
0	0	0	0	49	0	0	0	0	0	0	0	0	0	57	0
0	0	0	0	0	0	0	0	0	0	0	0	0	0	0	0
0	0	0	0	0	0	0	0	0	0	0	0	0	0	0	0
0	0	0	0	0	0	0	0	0	0	55	0	13790	0	156	0
368	243949	45699	1201	8532	419	771	1345	26432	0	756	286	314	2269	313	16788
0	0	0	0	0	0	0	0	0	0	0	0	0	0	0	0
0	0	0	0	0	0	0	0	0	0	0	0	0	0	0	0
0	140	47	97	60	0	93	0	0	0	0	0	0	0	0	0
0	480	46	0	0	0	0	445	0	0	679	0	0	0	0	3796
0	0	0	0	0	0	0	0	0	0	0	0	0	0	0	0
0	0	0	0	0	0	0	0	0	0	0	0	0	0	0	0
0	0	0	0	0	0	0	0	0	0	0	0	0	0	0	0
0	0	0	0	0	0	0	0	0	0	0	0	0	0	0	0
0	0	0	0	0	0	0	0	0	0	0	0	0	0	0	0

0	0	0	0	0	0	0	0	0	0	0	0	0	0	0	0
0	0	0	0	0	0	0	0	0	0	0	0	0	0	0	0
4210	0	0	0	0	37	0	0	0	0	0	0	0	0	0	0
0	0	0	0	0	0	0	0	0	0	0	0	0	0	0	0
935	26357	48	7696	1766	3185	96	0	0	0	9986	363	128	114	304	194
0	0	0	0	0	0	0	0	0	0	0	0	0	0	0	0
315	30204	257	533	20102	254	571	2763	105	216	2881	0	0	0	0	0
0	0	0	0	0	0	0	0	0	0	625	0	0	0	0	0
1440	2135	1175	2674	1187	778323	3549	0	0	0	0	0	0	0	0	0
0	0	0	0	0	0	0	0	0	0	0	0	0	1690	0	0
0	0	0	0	0	0	0	0	0	0	0	0	0	0	0	0
0	0	0	0	0	0	0	0	0	0	0	0	0	0	0	0
0	0	0	0	0	0	0	0	0	0	0	0	0	0	0	0
0	0	0	0	0	0	0	0	0	0	0	0	0	0	0	0
0	0	0	0	0	0	0	0	0	0	0	0	0	0	0	0
0	0	0	0	0	0	0	0	0	0	0	0	0	0	0	0
32068	467	5531	836	156	271	5397	8385	0	0	499	9896	521	80992	319	20641
0	0	0	0	0	0	0	0	0	0	0	0	0	0	0	0
0	0	0	0	0	0	0	0	0	0	0	0	0	0	0	0
0	0	0	0	0	0	0	0	0	0	0	59	0	0	0	0
0	0	0	0	0	0	0	0	0	0	0	0	0	0	0	0
0	0	0	0	0	0	0	0	0	0	180	0	0	47	0	34
0	0	0	0	0	0	0	0	0	0	0	0	0	0	0	0
0	0	0	0	0	0	0	0	0	0	0	0	0	0	0	0
0	0	0	0	0	0	0	0	0	0	0	0	0	0	0	0
0	0	0	0	0	0	0	0	0	0	0	52	0	67	91	46
0	0	0	52	0	0	0	0	0	0	0	0	0	0	0	41
0	0	0	0	0	0	0	0	0	0	0	0	0	0	0	0
0	0	0	0	0	0	0	0	0	0	0	0	0	0	0	0
0	0	0	47	0	37	154	0	0	0	0	0	51	0	0	0
0	0	0	0	0	0	0	0	0	0	0	0	0	0	0	0
0	0	0	0	0	0	0	0	0	0	0	0	0	0	0	0
0	0	0	46	0	0	0	0	0	0	0	0	0	0	0	799
0	0	0	0	0	0	0	0	0	0	0	0	0	0	0	0
0	0	0	0	0	0	0	0	0	0	0	0	0	0	0	0
271	68	96	98836	103	74	92	0	0	0	126	179	253	76447	152	48
0	0	0	54	42	44	105	0	0	0	0	0	0	0	0	0
0	0	0	0	0	0	0	0	0	0	0	0	0	0	0	0
0	0	0	0	0	0	0	0	0	0	0	0	0	0	0	0
0	0	0	0	0	0	0	0	0	0	0	0	0	0	0	0
0	0	0	0	0	0	0	0	0	0	0	0	0	0	0	0
0	0	0	0	0	0	0	0	0	0	0	0	0	0	0	0
1660	0	50	0	0	38	0	0	0	0	0	0	0	0	0	0
0	0	0	0	0	0	0	0	0	0	183	0	0	59	104	2925
0	0	0	0	0	50	0	0	0	0	0	0	0	0	0	0
45	26621	15799	90	99	0	217	0	1315	0	0	0	0	0	0	760
0	0	0	0	0	0	0	0	0	0	0	0	0	0	0	0
0	0	0	0	0	0	0	0	0	0	0	0	0	0	0	2309
0	0	0	0	0	0	0	0	0	0	0	0	0	0	0	0
0	0	0	0	0	0	0	0	0	0	0	0	0	0	0	0
0	2853	0	36	0	0	0	0	0	0	0	0	0	0	0	0
0	0	0	0	0	0	0	0	0	0	0	0	0	0	0	0
0	71	0	0	0	0	0	0	0	0	0	0	0	2879	0	0
0	0	0	0	0	0	0	0	0	0	0	0	0	0	0	0
0	0	0	0	0	0	0	0	0	0	0	0	0	0	0	0
0	0	0	0	0	0	0	0	0	0	0	0	0	0	0	0
0	0	0	0	0	0	0	0	0	0	0	0	0	0	0	0
0	0	0	0	0	0	0	0	0	0	0	0	0	0	0	0

0	0	0	0	0	0	0	0	0	0	0	0	0	0	0	48
0	0	0	0	0	0	0	0	0	0	0	14784	0	0	0	0
0	0	0	0	0	0	0	0	0	0	54	0	53	0	0	0
0	0	0	0	0	0	0	0	0	0	110	37	76	0	53	5037
0	0	0	0	0	0	0	0	0	0	0	0	0	0	0	0
0	0	0	0	0	0	0	0	0	0	0	0	0	0	0	0
0	0	0	0	0	0	0	0	0	0	0	0	0	0	0	0
44	63	184	76	0	0	68	0	0	0	0	0	0	0	0	0
0	0	0	0	0	0	0	0	0	0	0	0	0	0	0	0
0	0	0	0	0	0	0	0	0	0	0	0	0	55	0	0
0	0	0	0	0	0	0	0	0	0	0	0	0	0	0	0
47	0	0	0	0	0	0	0	0	0	0	0	0	0	50	0
0	0	0	0	0	0	0	0	0	0	0	0	0	0	0	0
0	0	0	0	0	0	0	0	0	0	0	0	0	0	0	0
0	0	0	0	0	0	0	0	0	0	0	0	0	0	0	0
6362	0	0	0	38	0	0	0	0	0	0	0	0	0	0	0
0	83	38	0	180	0	0	0	0	0	6078	0	3327	1624	0	0
0	0	0	0	0	0	0	0	0	0	0	0	0	0	0	0
0	0	0	0	0	0	0	0	0	0	0	0	0	0	0	0
0	0	0	0	0	0	0	0	0	0	0	0	0	0	0	0
0	0	0	0	0	0	0	0	0	0	0	0	0	0	0	0
0	0	0	0	0	0	0	0	0	0	0	0	0	0	0	0
0	0	0	0	0	0	0	0	0	0	0	0	0	0	0	0
0	0	0	0	0	0	0	0	0	0	0	0	0	0	0	0
0	0	0	0	0	0	0	0	0	0	23183	0	145	61	103	81
252705	343649	522001	340842	650390	94933	386190	396709	660112	793863	179001	212486	694176	158085	5522	62767
0	0	0	0	0	0	0	0	0	0	0	0	0	0	0	0
0	0	0	0	0	0	0	0	0	0	0	0	0	0	0	0
906	840	0	383	52	0	279	0	0	0	56	0	0	0	0	2776
0	0	0	0	0	0	0	0	0	0	0	0	0	0	0	0
0	0	0	0	0	0	0	0	0	0	0	0	0	0	0	0
88	514	0	3259	0	177	0	0	53	0	3581	0	0	0	0	0
0	0	0	0	0	0	0	0	0	0	0	0	0	0	0	0

Control061	Control062	Control064	Control065	Control067	Control068	Control069	Control071	Control074	Control076	Control077	Control079	Control081	Control082	Control084	Control085
44	0	0	0	8427	0	0	0	0	0	0	0	0	0	0	0
0	0	0	0	0	0	0	0	0	0	0	0	0	0	0	56
0	0	0	0	0	0	0	0	0	0	0	0	0	0	0	0
0	0	0	0	0	0	0	161	0	42	0	0	0	0	0	0
0	0	0	0	0	0	0	0	0	0	0	0	0	0	0	0
0	0	0	0	0	0	0	0	0	0	0	0	0	0	0	0
0	0	0	0	0	0	0	0	0	0	17644	0	0	0	0	0
403938	142656	77624	144290	12335	14716	111998	312430	16521	571	98034	494243	11210	536011	7760	303926
0	0	0	0	0	0	0	0	0	0	0	0	0	0	0	0
207	231	88	262	123	43	0	0	0	0	0	0	199	0	0	0
0	0	0	0	0	0	0	0	0	0	0	0	0	0	0	0
0	0	0	0	0	0	0	0	0	0	0	0	0	0	0	0
0	0	0	0	0	0	0	0	0	0	0	0	0	0	0	0
0	0	0	0	0	0	0	0	0	0	0	0	0	0	0	0
82212	5238	358	14070	287	89	371	0	0	0	0	46	109	0	46	2089
0	0	0	0	0	0	0	0	0	0	0	0	0	0	0	0
0	0	0	0	0	0	0	0	0	0	0	0	0	0	0	0
0	0	0	0	0	0	0	0	0	0	0	0	0	0	0	0
2446	303	21260	977	76	47	0	1367	4030	0	7293	107	0	8643	0	837
257	0	48	0	0	0	86	0	0	0	0	0	0	79	0	37
0	0	0	0	0	0	0	0	0	0	0	0	0	0	0	0
0	0	0	0	0	0	0	0	0	0	0	0	0	0	0	0
0	0	0	0	0	0	0	0	0	0	0	0	0	0	0	0

0	204	27769	2871	131	11591	94	0	0	0	0	0	0	0	0	2951
0	0	0	0	0	0	0	0	0	0	0	0	0	0	0	0
0	0	0	0	0	0	0	0	0	0	0	0	0	0	0	0
83	206	141	268	269	45	741	424	259	96	1609	602	674	1091	974627	1340
0	0	0	0	0	0	0	0	0	0	0	0	94	0	0	0
38	3072	41	0	76	0	0	3781	0	0	0	0	96	0	156	6779
0	0	0	0	0	0	0	0	0	0	0	0	0	0	0	0
0	0	0	0	0	0	0	0	0	0	0	0	0	0	0	0
0	0	0	0	0	0	0	0	0	0	0	0	0	0	0	0
1330	2938	0	0	0	43	0	0	0	0	212	49	95	52	61	109
0	0	0	0	0	0	0	0	0	0	0	0	0	0	0	0
0	0	0	0	0	0	0	0	0	0	0	0	0	0	0	0
0	0	0	0	0	0	0	0	0	0	0	0	0	0	0	0
0	0	0	0	0	0	0	0	0	0	0	0	0	0	0	0
0	0	0	0	0	0	0	0	0	0	0	0	0	0	0	0
0	0	0	0	0	0	0	0	0	0	0	0	41	0	0	43
0	0	0	0	0	0	0	0	0	0	0	100	0	0	0	0
0	0	0	0	0	0	0	0	0	0	0	0	0	0	0	0
0	0	0	0	0	0	0	0	0	0	0	0	0	0	0	0
0	0	0	0	0	0	0	0	0	0	0	0	0	0	0	0
0	321	0	0	0	0	0	0	0	0	5877	0	0	2676	0	40
0	0	0	0	0	0	0	0	0	0	0	0	0	0	0	0
0	0	0	0	0	0	0	0	0	0	1077	0	0	0	0	0
2805	39592	1208	237758	1682	3828	3272	17818	2062	62	275495	8786	375	2748	2178	181199
53	0	0	0	309	0	0	7475	425	27	65	0	0	0	0	0
33	70	0	153	45	243	0	0	0	0	0	0	0	0	0	0
3568	211	98	0	7585	0	0	5938	9767	0	3929	0	88	56	0	0
0	0	0	0	0	0	0	4411	0	0	1546	0	0	1480	0	473
0	0	0	0	0	0	0	0	0	0	0	0	0	0	0	0
95	0	87	133	46	0	56270	0	0	0	0	0	0	0	0	0
0	0	0	6954	0	0	81	0	0	0	0	0	0	0	0	0
0	0	0	0	0	0	0	0	0	0	0	0	0	0	0	0
0	0	0	0	0	0	0	0	0	0	0	0	0	0	0	0
0	0	0	0	0	0	0	0	0	0	0	0	0	0	0	0
0	0	0	0	0	0	0	0	0	0	0	0	0	0	0	0
0	0	0	0	0	0	0	0	0	0	0	0	0	0	0	0
0	0	0	0	0	0	0	0	0	0	0	0	0	0	0	0
0	0	0	0	0	0	0	0	0	0	0	326	0	1964	0	0
0	0	0	46	36	0	84	0	0	0	0	0	0	0	0	0
0	0	0	0	0	0	0	0	0	0	0	0	0	0	0	163
0	0	0	0	0	0	0	0	0	0	0	0	0	0	0	0
0	0	0	0	0	0	0	0	0	0	0	0	0	0	0	0
0	0	0	0	0	0	0	0	0	0	0	0	0	0	0	0
0	0	0	0	0	0	0	0	0	0	0	0	0	0	0	0
0	0	0	0	0	0	0	0	0	0	0	0	0	0	0	0
0	0	0	0	0	0	0	0	0	0	49	43	152	57	42	118
0	0	0	0	0	0	0	0	0	0	0	0	0	0	0	0
0	0	0	0	0	0	0	0	0	0	0	0	0	0	0	0
282	40073	671	536	826	1513	881	10471	1618	0	51213	426	394	564	615	277103
0	0	0	0	0	0	0	0	0	0	0	0	0	0	0	0
0	0	0	0	0	0	0	0	0	0	0	0	0	0	0	0
0	0	0	0	0	0	0	0	0	0	0	0	0	0	63	0
0	0	0	0	0	0	0	0	0	0	0	0	0	0	0	1682
50	0	0	69	78	51	75	0	0	0	0	0	0	0	0	0
0	0	0	0	0	0	0	0	0	0	0	0	0	0	168	0
0	0	0	0	0	0	0	0	0	0	0	0	0	0	0	0
0	0	0	0	0	0	0	0	0	0	0	0	0	0	0	0
179	0	44	0	0	0	0	0	0	0	0	0	0	0	0	0

227	0	0	0	0	0	76	307	0	0	0	0	0	3151	0	2998
44	71	51	49	0	47	0	0	0	0	0	0	0	0	0	0
0	0	0	0	0	0	0	0	0	0	0	0	0	0	0	0
0	0	0	121	263	0	1031	0	48	41	0	0	0	0	0	0
0	0	0	0	0	0	0	0	0	0	0	0	0	0	0	0
0	0	0	0	0	0	0	0	0	0	0	0	0	0	0	0
222	263	171	519	203	78	450	0	0	0	0	109	0	0	0	47
0	0	0	0	0	0	0	0	0	0	0	0	0	0	0	0
0	0	0	0	0	0	0	0	0	0	0	0	0	0	0	0
0	0	0	0	0	0	111	165	171	141	0	0	0	0	0	0
0	138	41	102	0	36	0	0	0	0	0	0	0	0	0	0
0	0	0	0	0	0	0	0	0	0	0	0	0	0	0	0
231	284	260	692	80	112	248	0	0	0	0	0	153	318	0	68
82	68	0	78	40	0	170	0	0	0	0	0	0	0	0	0
0	0	0	0	39	0	0	0	0	0	0	0	0	0	0	0
0	0	0	0	0	0	0	0	0	0	0	0	0	0	0	0
42	148	34	32	0	48	105	0	0	0	0	0	0	0	0	0
0	153	0	0	6784	0	0	0	0	0	0	0	0	0	0	0
0	0	0	0	0	0	0	0	0	0	0	0	0	0	0	0
0	0	0	0	0	0	0	0	0	0	0	0	0	0	0	0
0	0	0	0	0	0	0	0	0	0	0	0	0	0	0	0
0	0	0	0	0	0	0	0	0	0	0	0	0	0	0	0
6293	0	0	129	0	0	0	0	0	0	114	0	0	0	0	0
0	0	0	0	0	0	0	0	0	0	58	26385	0	57	61	0
0	0	0	0	0	0	0	0	0	0	0	0	0	0	0	0
36	0	46	0	0	0	79	0	0	0	0	0	0	0	0	0
0	0	0	0	0	0	0	0	0	0	0	0	0	0	0	0
0	0	0	0	0	0	0	0	0	0	0	0	0	0	0	0
0	0	0	0	0	0	0	0	0	0	0	0	0	0	0	0
0	0	0	0	33	0	86	0	0	0	0	0	0	0	0	0
0	0	0	0	0	0	0	0	0	0	0	0	0	0	0	0
317	2476	710	170	131	1108	0	639	272	362	4258	0	0	228	50	583
0	0	0	0	0	0	0	0	0	0	0	338	0	0	0	0
483	0	0	0	0	0	0	0	0	0	0	101	0	2383	0	32
0	0	0	0	0	0	0	0	0	0	0	0	0	0	0	0
880	3248	2361	253	5284	0	0	2795	0	0	14229	2765	48	573	48	122
0	0	0	0	0	0	0	150	0	0	0	0	0	0	0	0
88	2968	37	48	1168	19375	285	0	0	0	8422	105	36508	7892	162	1023
13760	8996	28673	1128	93134	298	523	33035	50	0	2056	93	47	13162	52	86
1736	381	228	38	987	45	171	302	0	0	612	10176	47	119	523	102
78	0	0	38	43	0	0	35707	0	40	52	0	48	0	160	0
0	0	0	0	0	0	0	0	0	0	0	0	0	0	0	0
0	0	0	0	0	0	0	0	0	0	0	0	0	0	0	0
0	0	0	0	0	0	0	0	0	0	0	0	0	0	0	0
0	0	0	0	0	0	0	0	0	0	0	0	0	0	0	0
0	0	0	0	0	0	0	0	0	0	0	0	0	0	0	0
0	0	0	0	0	0	0	0	0	0	0	0	0	0	0	0
0	0	0	0	0	0	0	0	0	0	0	0	0	0	0	0
0	68	0	0	0	0	0	0	0	0	0	0	0	0	0	0
0	0	0	0	0	0	0	0	0	0	0	0	0	0	0	0
0	0	0	0	0	0	0	0	0	0	0	0	0	0	0	0
0	0	0	104	0	0	0	0	59764	161	62	110	72	62	0	0
0	0	0	0	0	0	0	0	0	0	0	0	0	0	0	0
0	0	0	0	0	0	0	0	0	0	0	0	0	0	0	0
0	0	0	0	0	0	0	0	0	0	54	0	33914	0	0	29
0	0	0	0	0	0	0	0	0	0	0	0	0	0	0	0
0	0	0	0	0	0	0	0	0	0	0	0	0	0	0	0
0	0	0	0	0	0	0	0	0	0	0	0	0	0	0	0

0	0	0	0	0	0	0	0	0	0	0	0	0	0	0	0
5500	4650	153904	68840	55228	24729	1359	55547	39648	135741	35854	356	25906	4656	796	15248
0	0	0	0	0	0	0	0	0	0	0	0	0	0	0	0
0	0	0	0	0	0	0	0	0	0	0	0	0	0	0	0
0	0	0	0	0	0	0	0	0	0	0	0	0	0	0	0
0	0	0	0	0	0	0	0	0	0	0	0	0	0	0	0
0	0	0	0	0	0	0	0	0	0	0	0	0	0	0	0
0	0	0	0	0	0	0	0	0	0	0	0	0	0	0	0
0	0	0	0	48	0	0	0	0	0	0	0	0	0	0	78
0	0	0	0	0	0	0	0	0	0	0	0	0	0	0	0
0	0	0	0	0	0	0	0	0	0	0	0	0	0	0	0
0	0	0	0	0	0	0	0	0	0	0	0	0	0	0	0
0	0	0	0	0	0	0	0	0	0	0	0	0	0	0	0
0	0	0	0	0	0	0	0	0	0	0	0	0	0	0	0
0	0	0	0	0	0	0	0	0	0	0	0	0	3852	0	0
0	0	0	0	0	0	0	0	0	0	0	0	0	0	0	0
0	0	0	0	0	0	0	0	0	0	0	0	0	0	0	0
0	0	0	0	0	0	0	0	0	0	0	0	0	0	0	0
0	0	0	0	0	0	0	0	0	0	0	0	0	0	0	0
95	326	63	119	116	85	177	0	0	0	0	0	0	0	0	0
0	0	0	0	35	0	0	0	0	0	0	0	0	0	0	0
0	0	0	0	0	0	0	0	0	0	0	0	0	0	0	48
0	0	0	0	0	0	0	0	0	0	0	0	0	0	0	0
0	0	44	52	38	36	91	0	0	0	0	0	0	0	0	0
0	0	0	0	0	0	0	0	0	0	0	0	0	0	0	0
0	0	0	0	0	0	0	0	0	0	0	0	0	0	0	0
0	0	0	0	0	0	0	0	0	0	0	0	0	0	0	0
0	0	0	0	0	0	0	0	0	0	0	0	0	0	0	0
0	0	0	0	0	0	0	152	104	133	0	0	0	0	0	0
0	0	0	0	0	0	0	0	0	0	0	0	0	0	0	0
0	0	0	0	0	0	0	0	0	0	0	0	0	0	0	0
0	0	0	0	0	0	0	0	0	0	0	0	0	0	0	0
0	0	0	0	0	0	0	0	0	0	347	0	36	0	0	76
0	0	0	0	0	0	0	0	0	0	0	0	0	0	0	0
0	0	0	0	0	0	0	0	0	0	0	0	0	0	0	0
5442	0	0	0	0	0	0	0	0	0	0	0	0	0	0	0
0	0	0	0	0	0	0	0	0	0	0	0	0	0	0	0
0	56	0	47	0	0	0	0	0	0	0	0	0	0	0	0
554	589	449	520	292	314	705	0	0	0	0	0	0	60	0	0
0	0	0	0	0	0	0	0	0	0	0	0	0	0	0	0
0	0	0	0	0	0	0	0	0	0	0	0	0	0	0	0
0	0	0	0	0	0	0	0	0	0	0	0	0	0	0	0
0	15510	0	10324	1196	38	3889	7839	53	62	22885	2001	631	1974	785	10138
0	0	0	0	0	0	0	0	0	0	59	0	0	0	0	0
0	0	0	0	0	0	0	0	0	0	0	0	0	0	0	0
0	0	0	0	0	0	0	0	0	0	0	0	0	0	0	0
0	0	0	0	0	0	0	0	0	0	0	0	0	0	0	0
0	0	0	0	0	0	0	0	0	0	0	0	0	0	0	0
0	0	0	0	0	0	0	0	0	0	0	0	0	0	0	0
0	0	0	0	0	0	0	0	0	0	0	0	0	0	0	0
0	0	0	0	0	0	0	0	0	0	0	0	0	0	0	0
0	0	0	0	0	0	0	0	0	0	3925	0	0	0	0	0
0	0	0	0	0	0	0	0	0	0	0	0	0	0	0	0
0	0	0	0	0	0	0	0	0	0	0	0	0	0	0	345
0	0	0	0	0	0	0	0	0	0	0	0	0	0	0	0
0	0	0	0	0	0	0	0	0	0	0	0	0	0	0	0
0	0	0	0	0	0	0	0	0	0	0	0	0	0	0	0
0	0	0	0	0	0	0	0	0	0	0	0	0	0	0	0

0	0	0	0	0	0	0	0	0	0	12343	49	0	0	58	50
0	0	0	0	0	0	0	0	0	0	0	0	0	0	0	0
0	0	0	0	0	0	0	0	0	0	0	0	0	0	0	0
0	0	0	0	0	0	0	0	0	0	0	0	0	0	0	0
0	0	0	0	0	0	0	0	0	0	0	0	0	0	0	0
0	0	0	0	0	0	0	0	0	0	0	0	0	0	0	0
5373	140	45	224	102	131	72393	0	0	0	0	58	38	2847	0	9088
940	0	0	0	0	0	0	0	0	0	0	0	0	0	0	0
0	0	0	0	0	0	0	0	0	0	0	0	0	0	0	0
0	0	0	0	0	0	0	0	0	0	0	0	0	0	0	0
0	0	0	0	0	0	0	0	0	0	0	0	0	0	0	0
0	0	0	0	0	0	0	0	0	0	0	2637	0	0	0	0
0	162	48839	161	65	26	191	0	0	0	0	0	0	0	0	0
0	0	0	0	0	0	0	0	0	0	0	0	0	0	0	0
56817	25465	32329	34991	28862	11283	107129	67850	103098	64670	53921	75644	98682	42972	1577	21302
0	0	0	0	0	0	0	133	35061	30	0	0	0	0	0	0
0	0	0	0	0	0	0	0	0	0	130	0	0	0	41	0
0	0	0	0	0	0	0	0	0	0	47	0	31	0	58	63
0	0	0	0	0	0	0	0	0	0	0	0	0	0	0	0
0	0	0	0	0	0	0	0	0	0	0	0	0	0	0	3271
0	0	0	0	0	0	0	0	0	0	0	0	0	0	0	0
0	232	0	0	0	0	0	0	0	0	0	60	28	0	59	4820
0	0	0	0	0	0	0	0	0	0	0	0	0	0	0	0
191	5983	83	210	210	156	616	0	0	0	0	45	0	0	0	0
0	0	0	0	0	0	0	0	0	0	0	0	0	0	0	0
0	0	0	0	36	0	273	0	0	0	106	45	0	0	0	44
0	0	0	0	0	0	0	0	0	0	0	0	0	0	0	0
0	154	0	0	0	0	0	0	0	0	0	0	0	0	0	0
1152	0	0	0	0	0	0	0	0	0	0	0	0	114	0	154
0	0	0	0	0	0	0	0	0	0	0	0	0	0	0	904
0	0	0	0	0	0	0	0	0	0	0	0	0	0	0	0
0	0	0	0	0	0	0	0	0	0	0	0	0	0	0	0
0	0	0	0	0	0	0	0	0	0	0	0	0	0	0	0
52	58	96	40	41	21838	271	0	0	0	0	0	0	0	0	0
0	0	0	0	0	0	0	0	0	0	0	0	0	0	0	0
0	0	0	0	0	0	0	0	0	0	0	0	0	0	0	0
0	0	0	0	0	0	0	0	0	0	0	0	0	0	0	0
0	0	0	0	0	0	0	0	0	0	0	0	0	0	0	0
0	0	0	0	0	0	0	0	0	0	0	0	0	0	0	0
89	0	61	78	86	75	0	143	0	0	0	0	0	0	0	0
0	0	0	0	0	0	0	0	0	0	0	0	0	0	0	0
0	0	0	0	0	0	0	0	0	0	0	0	0	0	0	0
0	0	0	0	0	0	0	0	0	0	0	0	0	0	0	0
0	0	0	0	0	0	0	0	0	0	0	0	0	0	0	0
3829	211	473	95	39	0	353	1201	0	0	0	481	42	3484	0	1219
0	64	79	0	42	0	84	0	0	0	0	0	0	0	0	0
0	0	0	0	0	0	0	0	0	0	5893	35	0	0	0	0
0	0	0	0	0	0	0	0	0	0	0	0	0	0	0	0
0	0	0	0	0	0	0	0	0	0	0	0	0	0	0	0
0	0	0	41	0	0	0	0	0	0	0	0	0	0	0	0
0	0	0	0	0	0	0	0	0	0	0	0	0	0	0	0
0	0	0	0	0	0	0	0	0	0	0	0	0	0	0	0
0	0	0	0	0	0	0	0	0	0	0	0	0	0	0	0
0	0	0	0	0	0	0	0	0	0	0	0	0	0	0	0
0	0	0	0	0	0	0	0	615	201	257	57	1708	74	0	85
0	0	0	0	0	0	0	0	0	0	0	0	0	0	0	0
0	0	0	0	0	0	0	0	0	0	0	0	0	65	0	0
0	0	0	0	0	0	0	0	0	0	0	0	0	0	0	0

60	0	0	0	0	0	0	0	0	0	0	0	0	0	0	0
0	0	0	0	0	0	0	0	0	0	0	0	0	0	0	0
0	0	0	0	0	0	0	0	0	0	0	0	0	0	0	0
0	0	0	0	0	0	0	0	0	0	0	0	0	0	0	0
0	0	0	0	0	0	0	0	0	0	0	0	121	1331	0	2657
0	0	0	0	0	0	0	0	0	0	0	0	0	0	0	0
0	0	0	0	0	0	0	0	0	0	59	0	0	0	0	40
0	0	0	0	0	0	0	0	0	0	0	0	0	0	0	0
0	0	0	0	0	0	0	0	0	0	0	0	0	0	0	0
0	0	0	0	0	0	0	0	0	0	0	0	0	0	0	0
0	0	0	0	0	0	0	0	35469	80	0	0	0	0	0	0
0	0	0	0	0	0	0	0	0	0	0	0	0	0	0	0
0	5445	0	0	0	0	0	0	0	0	0	0	0	0	0	0
6523	1646	0	36	87	48	0	0	0	0	0	0	0	0	0	0
0	0	0	0	0	0	0	0	0	0	0	0	0	0	0	0
0	0	0	0	0	0	0	0	0	0	0	0	0	0	0	0
0	0	0	0	0	0	0	0	0	0	54	0	0	0	0	5841
0	0	0	0	0	0	0	0	0	0	0	0	0	0	0	0
0	0	0	0	0	0	0	0	0	0	0	0	0	0	0	0
0	0	0	0	0	0	0	0	0	0	0	0	0	0	0	0
0	0	0	0	0	0	0	0	0	0	0	0	0	0	0	0
0	0	0	0	0	0	0	4056	0	0	0	0	0	0	0	0
0	0	0	0	0	0	0	0	0	0	0	0	0	0	0	0
0	0	0	0	0	0	0	0	0	0	0	0	0	0	0	0
0	0	0	0	0	0	0	0	0	0	0	0	0	0	0	0
0	0	0	39	0	0	0	0	0	0	0	0	0	0	0	0
0	0	0	0	0	0	0	0	0	0	0	0	0	0	0	0
0	0	0	0	0	0	0	0	0	0	0	0	0	0	0	0
0	0	0	0	42	0	0	0	0	0	0	0	0	0	0	0
0	0	0	0	0	0	0	0	0	0	0	0	0	0	0	0
0	0	0	0	0	0	0	0	0	0	0	0	27455	68	47	44
0	0	0	0	0	0	0	0	0	0	352	5478	416	1442	143	2380
0	0	0	0	0	0	0	0	0	0	0	0	0	0	0	0
0	0	0	0	0	38	0	0	0	0	0	0	0	0	0	0
0	0	0	0	0	0	0	0	0	0	0	0	0	0	0	0
0	578	1035	874	8307	0	0	3189	0	0	1011	1961	0	542	48	120
0	0	0	0	0	0	0	0	0	0	0	0	0	0	0	0
5392	2102	5324	11773	68985	8968	77216	5098	738	497	135141	64278	33144	11108	677	4560
0	1210	41	0	0	0	0	0	0	0	47	43	9059	0	52	96
0	0	0	0	0	0	0	0	0	0	0	0	0	0	0	0
0	0	0	0	0	0	0	0	0	0	0	0	0	0	0	0
0	0	0	0	0	0	0	0	0	0	0	0	0	0	0	0
0	0	0	31	0	0	0	0	0	0	0	0	0	3222	0	0
0	0	0	0	0	0	0	0	0	0	0	0	0	0	0	541
0	0	0	0	0	0	0	0	0	0	0	0	0	0	0	0
0	0	0	0	0	39	0	139	44	32	58	9406	44	53	185	0
0	0	0	0	0	0	0	0	0	0	0	0	0	0	0	256
0	0	0	0	0	0	0	0	0	0	0	0	0	0	0	230
0	0	0	0	0	0	0	0	0	0	0	0	0	0	0	0
60	0	0	0	0	0	0	0	0	0	0	0	0	0	0	0
189	148	0	146	103	154	332	0	0	0	125	0	0	0	0	0
0	0	0	0	0	0	0	0	0	0	0	0	0	0	0	0
0	0	0	0	0	0	0	0	0	0	0	0	0	1467	0	0
0	0	0	0	0	0	0	0	0	0	0	0	0	0	0	0
5180	0	0	0	0	0	0	0	0	0	0	0	0	0	0	0
0	0	0	0	0	0	0	0	0	0	0	0	0	0	0	0
0	0	0	0	0	0	80	0	0	0	0	0	0	0	0	2670
0	0	0	0	0	0	0	0	0	0	0	0	0	0	0	0

0	0	0	0	0	0	0	0	0	0	0	0	0	0	0	0
0	0	0	0	0	0	0	0	0	0	0	0	0	0	0	0
0	0	0	0	0	0	0	0	0	0	0	0	0	0	0	0
0	0	0	0	0	0	0	0	0	0	0	0	0	0	0	0
0	0	0	0	0	0	0	0	0	0	0	0	0	0	0	0
0	0	0	0	0	0	0	0	0	0	0	0	0	0	0	0
49	0	0	0	0	0	0	0	0	0	0	0	0	0	0	0
0	0	0	0	0	0	0	0	0	0	0	0	0	0	0	0
0	0	0	0	49	0	0	0	0	0	0	0	0	0	0	0
0	0	0	0	0	0	0	0	0	0	0	0	0	0	0	0
0	0	0	0	0	0	0	0	0	0	0	0	0	0	0	0
0	0	0	0	0	0	0	0	0	0	0	14008	0	0	0	4470
0	0	0	0	0	0	0	0	0	0	0	0	2865	0	0	43
0	0	0	0	0	0	0	0	0	0	0	0	0	0	0	0
0	0	0	0	0	0	0	0	0	0	2553	0	0	0	0	0
0	0	0	0	0	0	0	0	0	0	0	0	0	0	0	0
0	0	0	0	0	0	0	0	0	0	0	0	0	0	0	0
0	0	0	0	0	0	0	0	0	0	0	0	0	0	0	0
0	0	0	0	0	0	0	0	0	0	0	0	0	0	0	0
0	0	0	0	0	0	0	0	0	66	0	0	0	0	0	0
0	0	0	0	0	0	0	0	0	0	583	0	0	0	0	299
0	0	0	0	0	0	0	160	0	0	0	0	0	55	0	36
0	0	0	0	0	0	0	0	0	0	0	0	0	0	0	0
0	0	0	0	0	0	0	0	0	0	0	0	0	0	0	0
0	0	0	0	0	0	0	0	0	0	0	0	0	0	0	0
0	0	0	0	0	0	0	0	0	0	0	0	0	0	0	0
5166	1433	3215	1438	87	215	1243	6867	2166	2797	2091	1725	2241	4346	1054	3206
0	0	0	0	0	0	0	0	0	0	0	0	0	0	0	0
0	0	0	0	0	0	0	0	0	0	0	0	0	0	0	0
0	0	0	0	0	0	0	0	0	0	0	0	0	0	0	0
0	0	0	0	0	0	0	0	0	0	0	0	0	0	55	64
0	0	0	0	0	0	0	0	0	0	0	0	0	0	0	1046
0	56	0	32	0	0	0	0	0	0	0	0	0	0	0	0
0	0	0	0	0	0	0	0	0	0	0	0	0	0	0	0
0	0	0	0	0	0	0	0	0	0	0	0	0	0	0	0
0	0	0	0	0	0	0	0	0	0	0	0	0	0	0	0
0	0	0	0	0	0	0	0	0	0	0	0	0	69	0	0
0	0	0	0	0	0	0	0	0	0	0	0	0	0	0	0
50	0	42	0	0	0	0	0	0	0	0	0	0	0	0	0
0	0	0	0	0	0	0	0	0	0	0	0	0	0	0	0
0	0	0	0	0	0	0	0	0	0	0	0	0	0	0	0
0	0	0	0	0	0	0	0	0	0	0	0	0	0	0	0
0	0	0	0	0	0	0	0	0	0	0	0	0	0	0	0
0	0	0	0	0	0	0	0	0	0	0	0	0	0	0	26
0	0	0	0	0	0	0	0	0	0	0	0	39	0	0	40
716	68	0	0	0	0	0	0	0	0	0	0	0	0	0	0
0	0	0	0	0	0	0	0	0	0	0	0	0	389	0	0
0	0	0	0	0	0	0	0	0	0	0	0	0	0	0	0
0	0	0	0	0	0	0	0	0	0	0	0	0	0	0	0
0	0	0	0	0	0	0	0	0	0	0	0	0	0	0	0
4567	76	0	36	0	0	0	0	0	0	0	0	0	0	0	0
0	0	0	0	0	0	0	0	0	0	0	0	0	0	0	0
0	0	0	0	0	0	0	0	0	0	0	11698	0	0	35	0
0	0	0	0	0	0	0	0	0	0	0	0	0	0	0	0
0	0	0	0	0	0	0	0	0	0	0	0	0	0	0	0
0	0	0	0	0	0	0	0	0	0	46	0	46	0	0	78
0	0	0	0	0	0	0	0	0	0	0	0	0	0	0	0
2506	84	0	86	37	0	102	0	0	0	0	1618	37	1978	0	318

0	0	0	0	0	0	0	0	0	0	0	0	0	0	0	0
0	0	0	0	0	0	0	0	0	0	0	0	0	0	0	0
0	0	0	0	0	0	0	0	0	0	0	0	0	0	0	0
0	0	0	0	0	0	0	0	0	0	0	0	0	0	0	0
0	0	0	0	0	0	0	0	0	0	0	0	0	0	0	0
0	0	0	0	0	0	0	0	0	0	0	0	0	0	0	0
0	0	0	0	0	0	0	0	0	0	0	0	0	0	0	0
0	0	0	0	0	0	0	0	0	0	0	0	0	0	0	0
0	0	0	0	0	0	0	0	0	0	0	0	0	0	0	0
0	0	0	0	0	0	0	0	0	0	0	0	0	0	0	0
54	0	0	138	38	0	0	0	0	0	0	0	0	0	0	0
0	0	0	0	0	0	0	0	0	0	0	0	45	4697	0	0
0	0	0	35	0	0	0	0	0	0	0	0	0	0	0	80
0	0	0	0	0	0	0	0	0	0	0	0	0	0	0	0
0	0	0	0	0	0	0	0	0	0	0	0	0	0	0	0
0	0	0	0	0	0	0	0	0	0	0	0	0	0	0	0
0	0	0	0	0	0	0	0	0	0	0	0	0	0	0	0
0	0	0	0	0	0	0	0	0	0	0	0	0	0	0	0
0	0	0	0	0	0	0	0	0	0	0	0	136	0	0	41
0	0	92	43	0	0	0	0	0	0	0	0	0	0	0	0
0	72	0	124	35	0	0	0	0	0	0	0	0	0	0	0
0	0	0	0	0	0	0	0	0	0	0	0	0	0	0	0
0	0	0	0	0	0	0	0	0	0	0	0	0	0	0	0
0	0	0	0	0	0	0	0	0	0	0	0	0	0	0	0
0	0	0	0	0	37	0	0	0	0	0	0	0	0	180	0
0	0	0	0	0	0	0	0	0	0	0	0	0	0	0	0
0	0	0	0	0	0	0	0	0	0	0	0	0	0	0	0
153	121	87	40	146	159	1300	0	0	69	0	0	0	0	0	0
64688	421	496	566	284	349	157148	154	0	0	62	25170	0	0	154	79
0	0	0	0	0	0	0	0	0	0	0	0	0	0	0	0
0	0	0	0	0	0	0	0	0	0	0	0	0	0	0	0
0	0	0	0	0	0	0	0	0	0	0	0	0	0	0	0
0	0	0	0	0	0	0	0	0	0	0	0	0	0	0	0
0	0	0	0	0	0	0	0	0	0	0	0	0	0	0	0
38	0	0	0	12044	0	0	0	0	0	0	0	0	0	0	0
0	0	0	0	0	0	0	0	0	0	0	0	0	0	0	0
0	0	0	0	0	0	0	0	0	0	0	0	0	0	0	0
0	0	0	50	0	0	0	0	0	0	0	0	0	0	0	0
0	0	0	0	0	0	0	0	0	0	0	0	0	0	0	0
0	69	0	0	44	45	0	0	0	0	0	0	0	0	0	0
0	0	0	0	0	0	0	0	0	0	0	0	0	0	0	0
0	0	0	0	0	0	0	0	0	0	0	0	0	0	0	0
0	0	0	0	0	0	0	0	0	0	0	0	0	0	0	0
0	0	0	0	0	0	0	0	0	0	0	0	0	0	0	0
0	0	0	0	0	0	0	0	0	0	0	0	0	0	0	0
0	0	0	0	0	0	0	0	0	0	0	0	0	0	0	0
0	0	0	0	49	0	0	0	0	0	0	0	0	0	57	0
0	0	0	0	0	0	0	0	0	0	0	0	0	0	0	0
0	0	0	0	0	0	0	0	0	0	0	0	0	0	0	0
0	0	0	0	0	0	0	0	0	0	0	0	0	0	0	0
0	0	0	0	0	0	0	0	0	0	55	0	13790	0	156	0
0	0	0	0	0	0	0	0	0	0	0	0	0	0	0	33
368	243949	45699	1201	8532	419	771	1345	26432	0	756	286	314	2269	313	16755
0	0	0	0	0	0	0	0	0	0	0	0	0	0	0	0
0	0	0	0	0	0	0	0	0	0	0	0	0	0	0	0
0	140	47	97	60	0	93	0	0	0	0	0	0	0	0	0
0	480	46	0	0	0	0	445	0	0	679	0	0	0	0	3796
0	0	0	0	0	0	0	0	0	0	0	0	0	0	0	0

0	0	0	0	0	0	0	0	0	0	0	0	0	0	0	0
0	0	0	0	0	0	0	0	0	0	0	0	0	0	0	0
0	0	0	0	0	0	0	0	0	0	0	0	0	0	0	0
0	0	0	0	0	0	0	0	0	0	0	0	0	0	0	0
0	0	0	0	0	0	0	0	0	0	0	0	0	0	0	0
0	0	0	0	0	0	0	0	0	0	0	0	0	0	0	0
0	0	0	0	0	0	0	0	0	0	0	0	0	0	0	0
0	0	0	0	0	0	0	0	0	0	0	0	0	0	0	0
0	0	0	0	0	0	0	0	0	0	0	0	0	0	0	0
0	0	0	0	0	0	0	0	0	0	0	0	0	0	0	0
4210	0	0	0	0	37	0	0	0	0	0	0	0	0	0	0
0	0	0	0	0	0	0	0	0	0	0	0	0	0	0	0
935	26357	48	7696	1766	3185	96	0	0	0	9986	363	128	114	304	194
0	0	0	0	0	0	0	0	0	0	0	0	0	0	0	0
315	30204	257	533	20102	254	571	2763	105	216	2881	0	0	0	0	0
0	0	0	0	0	0	0	0	0	0	625	0	0	0	0	0
1440	2135	1175	2674	1187	778323	3549	0	0	0	0	0	0	0	0	0
0	0	0	0	0	0	0	0	0	0	0	0	0	360	0	0
0	0	0	0	0	0	0	0	0	0	0	0	0	1330	0	0
0	0	0	0	0	0	0	0	0	0	0	0	0	0	0	0
0	0	0	0	0	0	0	0	0	0	0	0	0	0	0	0
0	0	0	0	0	0	0	0	0	0	0	0	0	0	0	0
0	0	0	0	0	0	0	0	0	0	0	0	0	0	0	0
0	0	0	0	0	0	0	0	0	0	0	0	0	0	0	0
0	0	0	0	0	0	0	0	0	0	0	0	0	0	0	0
0	0	0	0	0	0	0	0	0	0	0	0	0	0	0	0
0	0	0	0	0	0	0	0	0	0	0	0	0	0	0	0
0	0	0	0	0	0	0	0	0	0	0	0	0	0	0	0
0	0	0	0	0	0	0	0	0	0	0	0	0	0	0	0
32068	467	5531	836	156	271	5397	8385	0	0	499	9896	521	80992	319	20641
0	0	0	0	0	0	0	0	0	0	0	0	0	0	0	0
0	0	0	0	0	0	0	0	0	0	0	0	0	0	0	0
0	0	0	0	0	0	0	0	0	0	0	59	0	0	0	0
0	0	0	0	0	0	0	0	0	0	0	0	0	0	0	0
0	0	0	0	0	0	0	0	0	0	0	0	0	0	0	0
0	0	0	0	0	0	0	0	0	0	180	0	0	47	0	34
0	0	0	0	0	0	0	0	0	0	0	0	0	0	0	0
0	0	0	0	0	0	0	0	0	0	0	0	0	0	0	0
0	0	0	0	0	0	0	0	0	0	0	0	0	0	0	0
0	0	0	0	0	0	0	0	0	0	0	0	0	0	0	0
0	0	0	0	0	0	0	0	0	0	0	0	0	0	0	0
0	0	0	0	0	0	0	0	0	0	0	0	0	0	0	0
0	0	0	0	0	0	0	0	0	0	0	0	0	0	0	0
0	0	0	0	0	0	0	0	0	0	0	0	0	0	0	0
0	0	0	0	0	0	0	0	0	0	0	52	0	67	91	46
0	0	0	0	0	0	0	0	0	0	0	0	0	0	0	0
0	0	0	52	0	0	0	0	0	0	0	0	0	0	0	41
0	0	0	0	0	0	0	0	0	0	0	0	0	0	0	0
0	0	0	0	0	0	0	0	0	0	0	0	0	0	0	0
0	0	0	0	0	0	0	0	0	0	0	0	0	0	0	0
0	0	0	47	0	37	154	0	0	0	0	0	51	0	0	0
0	0	0	0	0	0	0	0	0	0	0	0	0	0	0	0
0	0	0	0	0	0	0	0	0	0	0	0	0	0	0	0
0	0	0	46	0	0	0	0	0	0	0	0	0	0	0	799
0	0	0	0	0	0	0	0	0	0	0	0	0	0	0	0
0	0	0	0	0	0	0	0	0	0	0	0	0	0	0	0
0	0	0	0	0	0	0	0	0	0	0	0	0	0	0	0
0	0	0	0	0	0	0	0	0	0	0	0	0	0	0	0

37	0	0	33	31	0	0	0	0	0	0	0	0	0	46	0
0	0	0	0	0	0	0	0	0	0	0	0	0	60	0	0
0	0	0	0	0	0	0	0	0	0	0	135	72	0	0	0
234	68	96	98803	72	74	92	0	0	0	126	44	181	76387	47	48
0	0	0	0	0	0	0	0	0	0	0	0	0	0	0	0
0	0	0	0	0	0	0	0	0	0	0	0	0	0	59	0
0	0	0	54	42	44	105	0	0	0	0	0	0	0	0	0
0	0	0	0	0	0	0	0	0	0	0	0	0	0	0	0
0	0	0	0	0	0	0	0	0	0	0	0	0	0	0	0
0	0	0	0	0	0	0	0	0	0	0	0	0	0	0	0
0	0	0	0	0	0	0	0	0	0	0	0	0	0	0	0
0	0	0	0	0	0	0	0	0	0	0	0	0	0	0	0
1660	0	50	0	0	38	0	0	0	0	0	0	0	0	0	0
0	0	0	0	0	0	0	0	0	0	0	0	0	0	0	2925
0	0	0	0	0	0	0	0	0	0	183	0	0	59	104	0
0	0	0	0	0	0	0	0	0	0	0	0	0	0	0	0
0	0	0	0	0	50	0	0	0	0	0	0	0	0	0	0
45	26621	15799	90	99	0	217	0	1315	0	0	0	0	0	0	760
0	0	0	0	0	0	0	0	0	0	0	0	0	0	0	0
0	0	0	0	0	0	0	0	0	0	0	0	0	0	0	0
0	0	0	0	0	0	0	0	0	0	0	0	0	0	0	0
0	0	0	0	0	0	0	0	0	0	0	0	0	0	0	0
0	0	0	0	0	0	0	0	0	0	0	0	0	0	0	2309
0	0	0	0	0	0	0	0	0	0	0	0	0	0	0	0
0	0	0	0	0	0	0	0	0	0	0	0	0	0	0	0
0	0	0	0	0	0	0	0	0	0	0	0	0	0	0	0
0	0	0	0	0	0	0	0	0	0	0	0	0	0	0	0
0	0	0	0	0	0	0	0	0	0	0	0	0	0	0	0
0	0	0	0	0	0	0	0	0	0	0	0	0	0	0	0
0	0	0	0	0	0	0	0	0	0	0	0	0	0	0	0
0	0	0	0	0	0	0	0	0	0	0	0	0	0	0	0
0	2853	0	36	0	0	0	0	0	0	0	0	0	0	0	0
0	0	0	0	0	0	0	0	0	0	0	0	0	0	0	0
0	0	0	0	0	0	0	0	0	0	0	0	0	0	0	0
0	71	0	0	0	0	0	0	0	0	0	0	0	2879	0	0
0	0	0	0	0	0	0	0	0	0	0	0	0	0	0	0
0	0	0	0	0	0	0	0	0	0	0	0	0	0	0	0
0	0	0	0	0	0	0	0	0	0	0	0	0	0	0	0
0	0	0	0	0	0	0	0	0	0	0	0	0	0	0	0
0	0	0	0	0	0	0	0	0	0	0	0	0	0	0	0
0	0	0	0	0	0	0	0	0	0	0	0	0	0	0	0
0	0	0	0	0	0	0	0	0	0	0	0	0	0	0	0
0	0	0	0	0	0	0	0	0	0	0	0	0	0	0	48
0	0	0	0	0	0	0	0	0	0	0	14784	0	0	0	0
0	0	0	0	0	0	0	0	0	0	54	0	53	0	0	0
0	0	0	0	0	0	0	0	0	0	110	37	76	0	53	5037
0	0	0	0	0	0	0	0	0	0	0	0	0	0	0	0
0	0	0	0	0	0	0	0	0	0	0	0	0	0	0	0
0	0	0	0	0	0	0	0	0	0	0	0	0	0	0	0
44	63	184	76	0	0	68	0	0	0	0	0	0	0	0	0
0	0	0	0	0	0	0	0	0	0	0	0	0	0	0	0
0	0	0	0	0	0	0	0	0	0	0	0	0	55	0	0
0	0	0	0	0	0	0	0	0	0	0	0	0	0	0	0
47	0	0	0	0	0	0	0	0	0	0	0	0	0	50	0
0	0	0	0	0	0	0	0	0	0	0	0	0	0	0	0
0	0	0	0	0	0	0	0	0	0	0	0	0	0	0	0
0	0	0	0	0	0	0	0	0	0	0	0	0	0	0	0
0	0	0	0	0	0	0	0	0	0	0	0	0	0	0	0

0	0	0	0	0	0	0	0	0	0	0	0	0	0	0	0
6362	0	0	0	38	0	0	0	0	0	0	0	0	0	0	0
0	0	0	0	0	0	0	0	0	0	0	0	0	0	0	0
0	0	0	0	0	0	0	0	0	0	0	0	0	0	0	0
0	0	0	0	44	0	0	0	0	0	0	0	0	0	0	0
0	0	0	0	91	0	0	0	0	0	6014	0	3327	0	0	0
0	0	0	0	0	0	0	0	0	0	0	0	0	0	0	0
0	83	38	0	45	0	0	0	0	0	64	0	0	1624	0	0
0	0	0	0	0	0	0	0	0	0	0	0	0	0	0	0
0	0	0	0	0	0	0	0	0	0	0	0	0	0	0	0
0	0	0	0	0	0	0	0	0	0	0	0	0	0	0	0
0	0	0	0	0	0	0	0	0	0	0	0	0	0	0	0
0	0	0	0	0	0	0	0	0	0	0	0	0	0	0	0
0	0	0	0	0	0	0	0	0	0	0	0	0	0	0	0
0	0	0	0	0	0	0	0	0	0	0	0	0	0	0	0
0	0	0	0	0	0	0	0	0	0	0	0	0	0	0	0
0	0	0	0	0	0	0	0	0	0	0	0	0	0	0	0
0	0	0	0	0	0	0	0	0	0	23183	0	104	61	103	81
0	0	0	0	0	0	0	0	0	0	0	0	41	0	0	0
0	71	0	371	35	0	0	0	0	0	0	0	0	120	0	0
252705	343578	522001	340471	650355	94933	386190	396709	660112	793863	179001	212486	694176	157965	5522	62767
0	0	0	0	0	0	0	0	0	0	0	0	0	0	0	0
0	0	0	0	0	0	0	0	0	0	0	0	0	0	0	0
906	840	0	383	52	0	279	0	0	0	56	0	0	0	0	2776
0	0	0	0	0	0	0	0	0	0	0	0	0	0	0	0
0	0	0	0	0	0	0	0	0	0	0	0	0	0	0	0
88	514	0	3259	0	177	0	0	53	0	3581	0	0	0	0	0
0	0	0	0	0	0	0	0	0	0	0	0	0	0	0	0
0	0	0	0	0	0	0	0	0	0	0	0	0	0	0	0
0	0	0	0	0	0	0	0	0	0	0	0	0	0	0	0
0	0	0	0	0	0	0	0	0	0	0	0	0	0	0	0
0	0	0	0	0	0	0	0	0	0	0	0	0	0	0	0
0	0	0	0	0	0	0	0	0	0	0	0	0	0	0	0
972	0	183	125	0	39	178	449	0	0	58	95	52	1380	0	681

Control086	Control087	Control088	Control090	Control091	Control092	Control093	Control095	Control096	Control097	Control098	Control101	Control103	Control104	Control106	Control108
573324	620376	867677	833963	686271	430852	18041	422314	686933	985421	924004	372969	912332	953037	941558	823474
355983	341164	2205	68770	212187	550654	981100	548121	290551	8742	48714	591853	58722	14899	51487	12356
0	0	0	0	0	0	0	0	0	0	0	0	0	0	0	0
69693	37317	129605	47919	90666	18206	546	19027	15278	5514	24472	27586	12651	30658	6752	22680
961	1089	513	36752	10876	288	313	10448	7196	250	2084	6823	16295	529	203	141271
0	0	0	0	0	0	0	0	0	0	0	769	0	0	0	0
39	54	0	12596	0	0	0	90	42	73	726	0	0	877	0	219

Control086	Control087	Control088	Control090	Control091	Control092	Control093	Control095	Control096	Control097	Control098	Control101	Control103	Control104	Control106	Control108
0	0	0	0	0	0	0	0	0	0	0	0	0	257	0	0
0	0	51	0	0	0	0	0	0	260	0	0	0	0	0	0
19769	20586	4554	431413	495507	161403	5600	78789	37938	14269	625674	3280	14857	696413	1352	241482
0	0	0	0	0	0	0	0	0	0	0	0	0	0	0	0
0	0	0	0	0	0	39	0	0	0	0	0	0	0	0	0
0	0	0	0	0	0	0	0	0	0	0	0	0	0	0	0
0	0	0	0	0	0	0	0	0	0	0	0	0	0	0	0
0	0	0	0	62708	0	0	42	192	202	114	0	0	401	0	0
0	0	0	0	0	0	0	0	0	0	0	0	0	0	0	0
154	0	53	185	271	459	514	253	292	434	12718	330	57	260	53	1786
0	0	0	0	0	0	0	0	0	0	0	0	0	57	0	0
0	0	0	0	0	0	0	0	0	0	0	0	0	0	0	0
0	0	0	0	0	0	0	0	0	0	0	0	0	0	0	0
383	37	39	3458	836	64	0	292	534	0	6343	0	0	6227	0	2037
0	29	0	0	121	0	0	40	55	0	472	0	0	176	0	123
0	0	0	0	67	0	0	33	0	71	0	0	0	0	0	0
0	0	0	0	0	0	0	0	0	0	0	0	0	0	0	0
0	0	0	0	287	232	0	42360	0	243	34	0	0	0	0	0
0	0	0	0	0	0	0	0	0	0	0	0	0	0	0	0
0	0	0	0	0	0	0	0	0	0	0	0	0	0	0	0
994	552	1139	242	363	875	861	423	935	992	715	0	0	0	0	263
0	0	0	0	0	0	0	0	0	0	0	0	0	0	0	0
51	0	0	0	0	0	125	0	13872	63	0	140	200	0	9754	9293
0	0	0	0	0	0	0	0	0	0	0	0	0	0	0	0
0	0	0	0	0	0	48	0	0	42	81	58	5174	100	0	70
727	10453	63667	292625	14132	1636	1324	1193	134846	1598	1402	3816	24764	4473	302387	214459
0	0	0	0	0	0	0	0	0	0	0	0	0	0	0	0
0	0	0	0	0	0	0	0	0	0	0	0	11640	0	0	69
0	0	0	180	65	0	67	40	0	137	0	0	0	0	0	0
0	0	0	0	0	0	0	0	0	0	0	0	0	0	0	0
0	0	0	0	0	0	0	0	0	0	0	0	0	0	0	0
0	0	0	0	0	0	0	0	0	0	0	0	0	0	0	0
0	0	0	0	0	0	0	0	0	0	0	0	0	0	0	0
0	0	0	0	0	0	0	0	0	0	0	335	0	0	0	0
0	0	0	0	0	0	0	0	0	0	0	0	0	358	0	0
0	0	0	0	0	76	0	35	0	0	0	0	0	0	0	0
0	0	0	0	0	0	0	0	0	0	0	0	0	0	0	0
110	64	40	0	0	0	0	0	10177	0	0	0	0	0	0	0
0	0	0	0	0	0	0	0	0	0	0	0	0	0	0	0
606	470	562	314	141	541	282	254	263465	786	296	6501	1557	2006	595196	266694
42	37	0	0	0	0	0	0	0	0	0	0	0	0	0	0
0	0	0	0	76	0	0	34	0	48	48	166	151	406	157	0
0	0	0	0	0	0	0	0	0	0	0	0	0	0	0	0
0	0	0	0	0	0	0	0	0	0	0	0	0	0	0	0
0	0	0	0	0	0	0	0	0	0	0	0	4575	0	0	0
0	0	0	0	0	0	0	0	0	0	0	0	0	0	0	0
97	0	0	337	62	0	0	0	0	0	2326	78	0	1383	35	122

0	0	0	0	0	0	0	0	0	0	0	0	0	0	0	0
0	0	0	0	0	0	70	0	0	68	0	0	0	0	0	0
0	0	112	1212	262	540	458	35670	237	664	320	82	8973	115	1879	262
0	0	0	0	0	0	0	0	0	0	0	0	3188	49	0	0
0	0	0	0	0	0	0	0	0	0	0	0	0	0	0	0
0	0	0	0	0	0	0	0	0	0	0	0	0	0	0	0
0	0	0	0	0	0	0	0	0	0	0	0	0	0	0	0
72	32	42	0	0	0	0	0	0	0	433	308	56	191	34	255
0	0	0	0	66	0	0	0	0	0	0	157	0	61	0	113
0	0	0	0	0	0	0	0	0	0	0	0	0	0	0	0
0	0	0	0	0	0	0	0	0	0	0	0	0	0	0	0
0	0	0	0	0	0	0	0	0	0	0	0	0	0	0	0
0	0	0	0	0	0	0	0	0	0	0	0	0	0	0	0
0	0	0	0	0	0	0	0	0	0	0	0	0	0	0	0
0	0	0	0	0	0	0	0	0	0	0	0	0	0	0	0
0	0	0	0	0	176	0	0	28685	79	0	78	0	0	515	51
0	0	0	0	0	0	0	0	0	0	0	0	0	0	0	0
91	0	0	0	0	0	0	0	0	0	780	0	0	0	157	0
0	0	0	0	0	0	0	0	0	0	0	0	0	0	0	0
554	110982	530	4822	1382	181127	1575	185028	33938	3094	197426	214289	37478	1378	584	1431
0	0	0	0	0	0	0	0	0	0	0	0	0	0	0	0
0	0	0	0	0	0	0	0	0	0	0	0	0	0	0	0
0	0	0	0	0	0	0	0	0	0	0	0	0	0	0	0
0	0	0	0	0	0	0	0	0	0	0	0	0	0	0	0
0	0	0	0	0	0	0	0	0	0	0	0	0	0	0	241
0	0	0	0	0	0	0	0	0	0	0	0	0	0	2292	0
0	0	0	0	0	0	0	0	0	0	0	0	0	0	0	0
0	0	0	0	0	0	0	0	0	0	0	0	0	0	0	0
38	27	45	155	186	146	63	29	67	0	75	0	0	0	0	0
0	30	0	0	0	0	0	0	0	0	0	0	0	114	0	0
0	0	0	0	0	0	0	0	0	0	0	0	0	49	0	0
0	0	0	1304	0	0	56	0	0	0	0	0	0	0	0	0
0	0	0	0	0	0	0	0	0	0	0	0	0	0	0	0
1326	172711	537	5119	154444	229332	7314	75001	130736	2677	15336	50489	38404	2984	43891	8026
0	0	0	0	0	0	0	0	0	0	0	0	41	0	0	0
0	0	0	0	0	0	0	0	0	0	0	0	0	0	0	0
128	0	82	0	0	0	0	0	0	0	0	0	0	0	0	0
0	0	0	0	0	0	0	0	0	0	0	0	0	0	0	0
0	0	0	0	0	0	0	0	0	0	0	0	0	0	0	0
0	0	0	98	0	154	59	80	55	0	83	0	0	368	0	72
0	0	0	0	0	77	58	0	0	0	0	0	0	0	0	0
0	0	0	0	0	0	0	0	0	0	0	0	0	0	0	0
0	0	0	0	0	0	0	0	5990	0	43	0	0	0	0	0
0	0	0	79	68	91	116	0	61	71	33	0	0	0	0	0
0	0	0	0	0	0	0	0	59	0	0	0	0	0	0	0
0	0	0	0	0	0	0	0	0	0	0	0	0	0	0	0
0	0	0	0	0	0	0	0	0	0	0	0	0	0	0	0
0	0	0	0	0	0	0	0	0	0	0	0	0	0	0	0
0	0	0	240	135	307	232	198	15162	406	169	0	0	0	0	670
37	33	57	0	0	0	0	0	0	0	0	0	11909	0	0	0
0	0	0	0	0	0	0	0	0	0	0	0	0	0	0	0
0	0	0	0	0	0	0	0	0	0	0	0	0	0	0	0
0	0	0	228	80	112498	0	132	54	71	152	0	0	0	0	0
0	0	49	0	0	0	0	0	0	0	0	171	0	0	39	0
0	0	0	0	0	0	0	0	0	0	0	0	0	0	0	0
961	1089	513	36752	10728	288	221	10448	7138	126	1987	6823	16295	529	203	141271
0	0	0	0	0	0	0	0	0	0	0	0	0	0	0	0
0	0	0	0	0	0	0	0	0	0	0	0	0	0	0	0

0	0	0	0	0	0	0	0	0	0	0	0	0	0	0	0
0	0	0	0	0	0	0	0	0	0	0	0	0	0	0	0
0	0	0	0	0	0	0	0	0	0	0	0	0	0	0	0
0	0	0	0	139	0	0	0	0	0	0	0	0	247	0	0
0	0	0	0	0	0	0	0	0	0	0	0	0	0	0	0
0	0	0	90	56	0	0	31865	117	182	41	0	0	0	0	0
0	0	0	0	0	0	0	0	0	0	0	0	0	0	0	0
0	0	0	165	0	0	0	0	63	0	0	0	0	1102	272	116
0	0	0	0	0	0	0	0	0	0	0	0	0	0	0	889
47	0	0	0	0	0	0	0	0	0	0	0	0	0	0	182
0	0	0	0	0	0	0	0	0	0	0	0	0	0	0	0
0	0	0	0	0	0	0	0	0	0	44	0	0	0	0	0
0	0	40	0	0	0	0	0	0	0	0	75	0	0	0	0
0	0	0	0	0	0	0	0	0	0	0	0	0	0	0	0
0	0	0	0	0	0	0	0	0	0	0	0	0	0	0	0
79	27	69	620	54073	284	0	4992	124	0	4496	83	57	27963	212	2943
43	68	0	61	1179	0	62	0	56	0	4379	0	0	1507	0	488
0	0	0	166	62	161	137	105	0	287	95	0	0	46	0	0
0	0	0	0	0	0	0	0	0	0	0	0	0	0	0	0
0	0	0	0	0	0	201	42	58	58	44	0	0	0	0	0
0	0	0	0	0	0	0	0	0	0	0	0	0	0	0	0
0	0	0	0	0	0	0	0	0	0	0	0	0	0	0	0
69693	37317	129605	47754	90666	18206	546	19027	15215	5514	24472	27586	12651	29556	6480	22564
0	26	0	1226	0	81	0	0	0	0	4485	575	63	123	171	1212
0	0	0	0	0	0	0	0	1523	0	0	87	0	0	5432	4886
0	0	0	0	0	0	0	0	0	0	0	0	0	0	0	0
0	0	0	0	0	87	0	0	0	0	0	0	0	55	0	0
0	0	0	0	0	0	0	0	0	0	0	0	0	0	0	0
0	28	0	0	0	0	0	0	0	0	0	0	225	0	0	2109
0	0	0	0	0	0	0	0	0	0	0	0	0	0	0	0
0	36	0	0	234	0	0	0	0	0	2266	0	0	129	0	65
0	0	0	0	0	0	0	0	0	0	0	0	0	0	0	0
0	0	0	0	0	0	0	0	58	124	97	0	0	0	0	0
0	0	0	0	0	0	0	0	0	0	0	0	0	0	0	0
0	0	0	0	0	0	0	0	0	0	0	0	0	0	0	0
0	0	0	0	0	0	0	0	0	0	0	0	0	0	0	0
0	0	0	0	0	0	0	0	0	0	0	13947	0	0	0	0
0	0	0	0	0	0	0	0	0	0	0	0	0	0	0	0
0	0	0	0	0	0	120	0	64	0	0	0	0	0	0	0
0	0	0	0	0	0	0	0	0	0	0	0	0	71	0	0
0	0	0	0	0	0	0	0	0	0	0	0	0	0	0	0
33	31	0	330	0	0	49	0	0	60	458	0	91	5259	0	181
0	0	0	0	0	0	0	0	0	0	0	0	0	0	0	0
0	0	0	0	0	0	0	0	0	0	0	0	0	0	0	0
0	0	0	0	0	0	0	0	0	0	0	0	0	0	0	0
0	0	0	0	0	0	0	0	0	0	0	0	0	0	0	0
0	0	0	0	0	0	0	0	0	0	0	0	0	251	0	0
0	0	0	0	0	0	0	0	0	0	0	0	0	0	0	0
0	0	0	0	0	0	0	0	0	0	0	0	0	0	0	0
0	0	0	0	0	0	0	0	0	0	0	0	0	0	0	0
0	0	0	0	0	0	0	0	0	0	0	0	0	0	0	0
51792	535	0	0	0	0	0	0	0	56	38	155	0	131	0	124
0	0	0	0	0	0	0	0	0	0	0	0	0	0	0	0
0	22572	0	0	0	0	0	0	0	0	0	0	0	0	0	0
0	0	0	0	0	0	57	0	0	0	0	76	43	72	0	65
0	0	0	0	0	0	0	0	0	0	0	0	0	0	0	0
0	0	0	0	0	0	0	0	0	0	0	0	0	0	0	0
0	0	0	0	0	0	0	0	0	0	0	0	0	0	0	0

0	4740	0	329	58	0	0	0	6778	65	997	0	0	564	0	0
0	0	0	0	0	0	0	0	0	0	0	0	0	0	0	0
0	0	0	0	0	0	0	0	0	0	0	0	0	0	0	0
0	0	0	0	0	0	0	0	0	0	0	0	0	0	0	0
0	0	0	86	0	0	63	26	0	0	75	0	0	0	0	0
0	0	0	0	0	0	0	0	0	0	0	0	0	0	0	0
0	0	0	0	0	0	0	0	0	0	0	0	0	0	0	0
0	0	0	0	0	0	0	0	0	67	0	0	0	0	0	0
0	0	0	0	0	0	0	0	0	0	0	0	0	0	0	0
0	0	0	0	0	229	165	0	50	0	84	0	0	0	0	0
0	0	0	0	0	74	0	0	0	0	0	0	0	0	0	0
0	0	0	0	53083	75	243	68	100	0	73	0	0	0	0	0
0	0	0	0	0	0	0	0	0	0	0	0	0	0	0	0
0	0	0	0	0	0	0	0	0	0	0	239	0	56	122	0
0	0	0	0	0	0	0	0	0	0	0	0	46	0	0	269
0	0	0	0	0	0	0	0	0	0	0	0	4817	0	0	72
0	0	0	0	0	0	0	0	0	0	0	0	0	0	0	0
0	0	0	0	0	0	0	0	0	0	0	0	0	0	0	0
0	0	0	0	0	0	0	0	0	0	0	0	0	0	0	0
38	0	46	0	0	0	0	0	0	0	0	0	0	0	0	0
353687	103926	814	4140	677	51228	441	138094	147088	1438	3297	378957	14909	1061	4596	846
0	0	0	1221	0	0	0	0	0	0	0	0	0	0	0	0
0	0	0	952	0	0	0	0	0	0	613	0	0	0	0	0
0	0	0	0	0	0	0	0	0	0	0	0	0	0	0	0
0	0	0	0	0	0	0	0	1451	0	0	0	0	0	0	0
52	60	0	1172	1067	1931	1189	748	5004	1221	1035	593	103	67	113	321
0	0	0	0	0	0	0	0	0	0	0	0	0	0	0	0
0	0	0	0	913	73	0	0	0	0	43	0	0	671	0	60
0	0	0	0	0	0	0	0	0	0	0	0	0	68	0	0
0	0	0	0	148	0	53	0	0	0	0	0	0	0	0	0
0	0	0	0	0	0	0	0	0	0	0	0	0	0	0	0
0	0	0	0	0	0	0	0	0	0	0	0	1504	0	0	0
0	0	45	1392	509	735	697	245982	458	1071	515	0	0	0	0	0
0	0	0	0	0	0	0	0	0	0	0	0	0	0	0	0
0	0	0	0	0	0	0	0	3035	0	0	0	0	0	7565	1326
0	0	0	0	0	0	0	0	0	0	0	0	0	0	0	0
0	0	0	0	0	0	0	0	0	0	0	0	0	0	0	0
0	0	0	0	0	0	0	0	0	0	0	0	0	0	0	0
0	0	0	0	0	0	0	0	0	0	0	0	0	0	104	0
0	0	0	0	0	0	0	0	0	0	0	0	0	0	0	0
0	0	0	0	0	0	0	0	0	0	0	0	0	0	0	0
0	0	0	0	0	0	0	0	0	0	0	0	0	0	0	0
0	0	0	1059	0	0	0	0	0	0	0	0	0	0	0	0
0	0	0	73	0	0	0	0	0	0	0	0	0	0	0	0
0	0	49	0	63	79	0	95	129	128	4488	0	0	0	0	0
34	27	0	0	54	0	0	0	0	0	0	0	0	0	0	0
0	0	0	0	0	0	0	0	0	0	0	151	0	52	0	68
40	0	0	0	0	0	0	0	0	0	0	78	0	0	0	377
0	0	0	0	0	0	0	0	0	0	0	0	0	220	0	0
0	0	0	0	0	0	0	0	0	0	0	0	0	0	0	0
0	0	0	0	0	0	0	0	0	0	0	0	0	0	0	0
0	0	0	0	0	0	0	0	0	0	0	0	0	0	0	0
0	0	0	0	0	0	0	0	0	0	0	0	0	0	0	0
41	0	28	0	129	76	0	0	54	0	35	152	0	669	0	663
0	0	0	0	0	0	0	0	0	0	0	0	0	0	0	0
0	0	0	0	0	0	0	0	0	0	0	0	0	0	0	0
1731	1505	3545	2165	3792	35918	0	30	786	142	6942	0	59	8025	0	1206
0	0	0	0	0	0	0	0	0	0	0	0	0	0	0	0

0	0	0	0	0	0	0	0	0	0	0	0	0	0	0	0
0	0	0	0	0	0	0	0	0	0	0	0	0	0	0	0
38	24319	0	0	0	0	0	0	0	0	0	0	0	0	0	0
0	0	0	0	0	0	0	0	610	0	0	0	0	0	1580	966
0	0	0	0	0	0	0	0	0	0	0	0	0	0	0	0
0	0	0	0	0	0	0	0	0	0	0	0	0	0	0	0
0	0	0	0	0	0	0	0	0	0	0	0	0	0	0	0
42	11311	0	0	0	35145	0	13378	0	0	0	0	1882	0	63	223
53	68	0	0	0	0	0	0	0	0	0	85	0	129	2755	51
47	31836	100	0	0	0	0	0	0	0	0	0	0	0	0	0
0	58	0	315	337	0	0	0	0	133	1336	0	0	3626	0	899
0	0	0	0	0	0	0	0	0	0	0	0	0	0	0	0
0	0	0	0	0	0	0	0	0	0	0	0	0	0	0	0
0	0	0	0	0	0	0	0	0	0	0	0	0	0	0	0
0	0	0	0	0	0	0	0	0	0	0	0	0	0	0	0
0	0	0	0	0	0	0	0	0	0	0	0	0	0	0	0
0	0	0	0	0	0	0	0	0	0	0	0	0	0	0	0
0	0	0	0	0	0	0	0	0	0	0	151	278	913	46	415
0	0	0	0	0	0	0	0	0	0	0	0	0	0	0	0
0	0	0	87	0	0	0	0	0	0	39	0	0	0	0	0
0	0	0	0	0	0	0	0	0	0	0	0	0	0	0	0
0	0	0	0	72	54	0	0	0	71	0	0	0	415	0	0
0	0	0	0	0	0	0	0	0	0	0	0	0	0	0	0
0	0	0	0	0	0	0	0	0	0	0	0	0	0	0	0
0	0	0	0	0	0	79	0	0	0	0	69	0	0	0	0
0	0	0	0	0	0	0	0	0	0	0	0	0	0	0	0
0	0	0	0	0	0	0	0	0	0	0	0	0	0	0	0
36	0	74	499	0	0	62	0	0	0	45	0	0	0	0	0
0	0	0	0	0	0	0	0	0	0	0	0	0	0	0	0
0	0	0	0	0	0	0	0	0	0	0	0	0	0	0	0
0	0	0	0	0	0	0	0	0	0	0	0	0	0	0	0
0	0	0	0	0	0	0	0	0	0	0	0	0	0	0	0
0	0	0	0	0	0	0	0	0	0	0	0	0	0	0	0
0	0	0	0	0	76	57	0	124	0	40	0	0	0	0	0
0	0	0	82	0	0	0	0	0	0	0	0	0	0	0	0
0	0	0	0	0	0	0	0	0	0	0	0	0	0	0	0
0	0	0	0	0	0	0	0	0	0	0	0	0	0	0	0
0	0	0	0	0	0	0	0	0	0	0	0	0	0	0	0
0	0	0	0	0	0	0	0	0	0	0	0	0	0	0	0
0	0	0	0	0	0	0	0	0	0	0	0	0	0	0	0
0	0	0	0	0	0	0	0	0	0	0	0	0	0	0	0
0	0	0	0	0	0	0	0	0	0	0	0	0	0	0	0
0	0	0	0	85	0	0	0	0	0	0	0	0	0	0	0
0	0	0	0	0	0	0	0	0	0	0	0	0	0	0	0
0	0	0	1065	0	0	0	0	0	0	0	0	0	0	0	0
0	0	0	0	0	0	0	0	0	0	0	0	1100	0	0	0
0	0	0	0	0	82	0	46	46	0	0	0	0	0	0	0
0	0	0	0	0	0	0	0	0	0	0	0	0	0	0	0
577	349	564	634	601	297	69	623	2189	390	1173	0	45	11579	57	19115
0	0	0	0	0	0	0	0	0	0	0	0	0	0	0	0
0	0	0	0	0	0	0	0	0	0	0	0	0	0	0	0
0	0	0	0	0	0	0	0	0	0	0	0	0	0	0	0
0	0	0	0	0	0	0	0	407	0	0	0	0	0	598	1756
0	0	0	0	0	0	0	0	0	0	0	0	0	0	0	0
0	0	0	0	0	0	0	0	0	0	0	0	0	0	0	0
0	0	0	0	0	0	0	0	0	0	0	0	0	0	0	0
44	0	0	0	0	0	0	0	0	0	0	0	0	0	0	0
0	0	0	0	0	0	0	0	0	0	0	0	0	0	0	0

0	0	0	0	0	0	0	0	0	0	0	0	0	0	0	0
0	0	0	0	0	0	0	0	0	76	0	82	52	99	0	67
0	0	0	0	0	0	0	0	0	0	0	769	0	0	0	0
0	0	0	0	0	0	0	0	0	0	0	0	0	0	0	0
324	126	272	44026	73	89	171	1508	290	0	39	323	0	116	0	334
0	0	0	0	0	0	0	0	0	0	0	64415	0	0	0	0
403	0	50	0	0	0	0	11053	0	69	95	0	0	0	1440	604
0	0	0	0	0	0	0	0	0	0	0	0	0	0	0	0
0	0	0	0	0	0	0	0	0	0	0	0	0	0	0	258
0	0	0	0	0	0	0	0	0	0	0	0	0	0	0	0
0	0	0	1039	438	793	970895	20672	421	1154	861	82197	0	62	0	0
0	0	0	0	0	0	0	0	0	0	0	0	0	0	0	0
0	0	0	0	0	0	0	0	0	0	0	0	0	0	0	0
0	0	0	0	0	0	0	0	0	0	0	0	0	0	0	0
0	0	0	0	0	0	0	0	0	0	0	240	95	122	97	0
0	0	0	0	0	0	0	0	0	0	0	0	0	0	0	0
535	358	95	13700	33460	667	1104	441	2378	612	27206	254	1158	55459	150	30980
0	0	0	0	0	0	0	0	0	0	0	0	0	0	0	0
0	0	0	0	0	0	0	0	0	0	0	0	0	0	0	0
0	0	0	0	0	0	0	0	0	0	0	0	0	0	0	0
0	0	0	0	0	0	0	0	0	66	0	0	0	0	0	0
0	27	0	0	0	0	0	0	0	0	0	0	0	0	0	0
0	0	0	0	0	0	57	0	0	123	0	312	174	57	34	135
0	0	0	0	0	0	0	0	0	0	0	0	0	0	0	0
0	0	0	0	0	0	0	0	0	0	0	0	0	0	0	0
38	0	82	0	0	0	0	0	0	0	0	237	65	163	0	50
0	0	0	0	0	0	0	0	0	0	0	0	0	0	0	0
0	0	0	0	0	0	0	0	0	0	0	0	0	0	0	0
36	0	0	0	0	0	49	35	0	0	2163	0	0	0	0	0
0	0	0	0	0	0	0	0	0	0	0	0	0	0	0	0
0	0	0	0	0	0	0	0	0	0	0	0	0	0	0	0
0	0	0	0	0	0	0	0	0	0	0	0	0	0	0	0
0	0	0	0	0	0	0	0	0	0	0	0	0	0	0	0
0	0	0	817	0	0	0	0	0	0	0	0	0	0	0	0
0	0	0	0	0	0	0	0	0	0	0	0	0	0	0	0
83	39	188	314	270	303	326	16435	4461	96658	0	18666	0	1035	0	122
0	0	0	0	0	0	0	69	0	0	0	0	0	1311	0	0
0	0	0	0	0	0	0	0	0	0	0	0	0	0	0	0
0	0	0	0	0	0	0	0	0	0	0	0	0	0	0	0
0	0	0	0	0	0	0	0	0	0	0	0	0	0	0	0
0	0	0	0	0	0	255	49	238	107	113	0	0	0	0	0
0	0	0	0	0	0	0	0	0	0	0	0	0	0	0	0
0	0	0	0	0	0	0	0	0	0	0	0	0	0	0	0
0	12111	0	179	0	155	0	32	48	79	0	0	0	0	0	0
0	0	0	81	0	0	0	23515	112	52	0	0	0	0	0	306
0	0	0	0	0	0	0	0	510	0	0	0	0	0	1949	2152
0	0	0	0	0	0	0	0	0	0	0	0	0	0	0	378
0	25	0	72	61	154571	235	178	123	611	149	394	122	112	57	268
0	0	0	0	0	0	0	0	0	0	0	0	0	0	0	0
0	0	0	0	0	0	0	0	0	0	0	0	0	0	0	0
0	0	0	0	0	0	0	0	0	0	0	0	0	0	0	0
0	0	0	0	0	0	0	0	0	0	0	0	0	0	0	0
0	0	0	0	0	0	0	40	0	0	0	0	0	0	0	0
0	0	0	0	0	0	0	0	0	0	0	0	0	0	0	0
0	0	0	0	0	0	0	0	0	0	0	0	0	0	0	0
0	0	0	0	0	0	0	0	0	0	0	0	0	0	0	0
0	0	235	0	0	0	0	0	0	0	0	0	0	0	0	0
0	0	0	0	0	0	0	0	0	0	0	0	160	0	0	0

0	0	0	0	130	0	0	0	0	0	0	0	0	255	0	215
0	0	0	0	0	0	0	0	0	0	0	0	0	0	0	0
0	20086	52	0	0	0	0	0	0	0	0	0	0	0	0	0
36	0	98	82	0	0	0	0	0	69	21590	0	0	0	0	0
0	0	0	6241	64	262	44	132	585	544	445	0	0	0	41	53
0	0	0	0	0	0	0	0	0	0	0	0	0	0	0	0
0	0	0	0	0	0	0	0	0	0	0	0	4280	0	0	57
0	0	0	0	0	0	0	0	0	0	0	0	0	0	0	0
0	0	0	0	0	0	0	0	0	0	0	0	0	0	0	0
0	0	0	0	0	0	0	0	0	0	0	0	0	0	0	0
0	0	0	0	0	0	0	0	0	0	0	0	0	0	0	0
0	0	0	0	0	0	0	0	0	0	0	0	0	0	0	0
0	0	0	0	0	0	0	0	0	0	0	0	0	0	0	0
0	0	0	0	0	0	0	0	0	0	0	0	0	0	0	0
0	0	0	0	0	0	0	0	0	0	0	610	186	117	58	241
0	22	0	0	0	0	0	0	0	0	0	0	0	287	0	0
40	33	0	388	0	0	0	47	0	0	344	166	157	195	113	200
0	0	0	0	0	0	0	0	0	0	0	0	0	0	0	0
0	0	0	0	0	0	0	0	0	0	0	0	0	0	0	0
0	0	0	0	0	0	0	0	0	0	0	0	0	0	0	0
0	0	0	0	0	0	0	0	0	0	0	0	0	0	0	0
0	0	0	0	0	0	0	0	0	0	0	0	0	0	0	0
0	0	0	0	0	0	0	0	0	0	0	0	0	168	0	0
0	0	0	0	0	0	0	0	0	0	0	84	6815	0	0	65
42	32	57	1864	0	0	0	0	0	0	0	0	0	0	0	1833
494137	411116	791820	69614	15520	8247	3492	40127	120717	862299	17464	121091	769544	126583	7367	8216
0	0	0	0	0	0	0	0	0	0	0	0	0	0	0	0
0	0	0	0	0	0	0	0	0	0	0	0	0	0	0	0
39	54	0	12596	0	0	0	90	42	73	726	0	0	877	0	219
0	0	0	0	0	0	0	0	0	0	0	0	0	0	0	0
0	0	0	0	0	0	0	0	0	0	0	0	0	0	0	0
0	0	0	4541	0	0	0	82	0	0	0	0	0	0	0	0
0	0	0	0	0	0	0	0	0	0	0	0	0	0	0	0

Control086	Control087	Control088	Control090	Control091	Control092	Control093	Control095	Control096	Control097	Control098	Control101	Control103	Control104	Control106	Control108
0	0	0	0	0	0	0	0	0	0	0	0	0	0	0	0
0	0	0	0	0	0	0	0	0	0	0	0	0	0	0	0
0	0	0	0	0	0	0	0	0	0	0	0	0	0	0	0
0	0	0	0	0	0	0	0	0	0	0	0	0	0	0	0
0	0	0	0	0	0	0	0	0	0	0	0	0	257	0	0
0	0	0	0	0	0	0	0	0	0	0	0	0	0	0	0
0	0	51	0	0	0	0	0	0	260	0	0	0	0	0	0
19769	20586	4554	431413	495507	161403	5600	78789	37938	14269	625674	3280	14857	696413	1352	241482
0	0	0	0	0	0	0	0	0	0	0	0	0	0	0	0
0	0	0	0	0	0	39	0	0	0	0	0	0	0	0	0
0	0	0	0	0	0	0	0	0	0	0	0	0	0	0	0
0	0	0	0	0	0	0	0	0	0	0	0	0	0	0	0
0	0	0	0	62708	0	0	42	192	202	114	0	0	401	0	0
0	0	0	0	0	0	0	0	0	0	0	0	0	0	0	0
154	0	53	185	271	459	514	253	292	434	12718	330	57	260	53	1786
0	0	0	0	0	0	0	0	0	0	0	0	0	57	0	0
0	0	0	0	0	0	0	0	0	0	0	0	0	0	0	0
0	0	0	0	0	0	0	0	0	0	0	0	0	0	0	0
383	37	39	3458	836	64	0	292	534	0	6343	0	0	6227	0	2037
0	29	0	0	121	0	0	40	55	0	472	0	0	176	0	123
0	0	0	0	67	0	0	33	0	71	0	0	0	0	0	0
0	0	0	0	0	0	0	0	0	0	0	0	0	0	0	0
0	0	0	0	0	0	0	0	0	0	0	0	0	0	0	0

0	0	0	0	287	232	0	42360	0	243	34	0	0	0	0	0
0	0	0	0	0	0	0	0	0	0	0	0	0	0	0	0
0	0	0	0	0	0	0	0	0	0	0	0	0	0	0	0
994	552	1139	242	363	875	861	423	935	992	715	0	0	0	0	263
0	0	0	0	0	0	0	0	0	0	0	0	0	0	0	0
51	0	0	0	0	0	125	0	13872	63	0	140	200	0	9754	9293
0	0	0	0	0	0	0	0	0	0	0	0	0	0	0	0
0	0	0	0	0	0	48	0	0	42	81	58	5174	100	0	70
0	0	0	0	0	0	0	0	0	0	0	0	0	0	0	0
37	73	59	163	55	0	74	68	0	185	0	91	62	0	0	0
0	0	0	0	0	0	0	0	0	0	0	0	0	0	0	0
0	0	0	0	0	0	0	0	0	0	0	0	0	0	0	0
0	0	0	0	0	0	0	0	0	0	0	0	0	0	0	0
0	0	0	0	0	0	0	0	0	0	0	0	0	0	0	0
0	0	0	0	0	0	0	0	0	0	0	0	0	0	0	0
39	0	0	0	0	0	0	0	0	0	0	0	0	0	0	0
30	0	0	0	0	0	0	0	0	0	0	0	0	0	0	0
0	0	0	0	0	0	0	0	0	0	0	0	0	0	0	0
0	0	0	0	0	0	0	0	0	0	0	0	0	0	0	0
0	0	0	0	0	0	0	0	0	0	0	0	2442	0	0	0
0	10146	0	1729	12569	0	0	0	1821	0	0	72	2011	127	52	121
0	0	0	0	0	0	0	0	0	0	0	0	0	0	0	0
0	0	0	0	0	0	0	0	0	0	0	0	0	46	56	40
533	149	63553	290733	1508	1488	1144	1091	129945	1275	1402	3653	20249	4300	302279	214002
0	0	0	0	0	0	0	0	52	0	0	0	0	0	0	0
0	0	0	0	0	76	0	0	0	138	0	0	0	0	0	0
0	0	55	0	0	0	52	34	2977	0	0	0	0	0	0	112
88	85	0	0	0	72	54	0	51	0	0	0	0	0	0	184
0	0	0	0	0	0	0	0	0	0	0	0	0	0	0	0
0	0	0	0	0	0	0	0	0	0	0	0	0	0	0	0
0	0	0	0	0	0	0	0	0	0	0	0	0	0	0	0
0	0	0	0	0	0	0	0	0	0	0	0	11640	0	0	69
0	0	0	0	0	0	0	0	0	0	0	0	0	0	0	0
0	0	0	180	65	0	67	40	0	137	0	0	0	0	0	0
0	0	0	0	0	0	0	0	0	0	0	0	0	0	0	0
0	0	0	0	0	0	0	0	0	0	0	0	0	0	0	0
0	0	0	0	0	0	0	0	0	0	0	0	0	0	0	0
0	0	0	0	0	0	0	0	0	0	0	0	0	0	0	0
0	0	0	0	0	0	0	0	0	0	0	0	0	0	0	0
0	0	0	0	0	0	0	0	0	0	0	335	0	0	0	0
0	0	0	0	0	0	0	0	0	0	0	0	0	0	0	0
0	0	0	0	0	0	0	0	0	0	0	0	0	358	0	0
0	0	0	0	0	76	0	35	0	0	0	0	0	0	0	0
0	0	0	0	0	0	0	0	0	0	0	0	0	0	0	0
0	0	0	0	0	0	0	0	10177	0	0	0	0	0	0	0
110	64	40	0	0	0	0	0	0	0	0	0	0	0	0	0
0	0	0	0	0	0	0	0	0	0	0	0	0	0	0	0
0	0	0	0	0	0	0	0	0	0	0	0	0	0	0	0
606	470	562	314	141	541	282	254	263465	786	296	6501	1557	2006	595196	266694
0	0	0	0	0	0	0	0	0	0	0	0	0	0	0	0
0	0	0	0	0	0	0	0	0	0	0	0	0	0	0	0
42	37	0	0	0	0	0	0	0	0	0	0	0	0	0	0
0	0	0	0	76	0	0	34	0	48	48	166	151	406	157	0
0	0	0	0	0	0	0	0	0	0	0	0	0	0	0	0
0	0	0	0	0	0	0	0	0	0	0	0	0	0	0	0
0	0	0	0	0	0	0	0	0	0	0	0	0	0	0	0
0	0	0	0	0	0	0	0	0	0	0	0	4575	0	0	0
0	0	0	0	0	0	0	0	0	0	0	0	0	0	0	0

97	0	0	337	62	0	0	0	0	0	2326	78	0	1383	35	122
0	0	0	0	0	0	0	0	0	0	0	0	0	0	0	0
0	0	0	0	0	0	70	0	0	68	0	0	0	0	0	0
0	0	0	0	0	0	64	0	0	0	0	82	0	63	0	0
0	0	0	0	0	0	0	0	0	0	0	0	0	0	0	0
0	0	0	0	0	0	0	0	0	0	0	0	0	0	0	0
0	0	57	0	0	0	0	0	0	59	0	0	0	0	0	0
0	0	0	0	0	0	0	0	0	0	0	0	0	0	0	0
0	0	0	0	0	0	0	0	0	0	0	0	0	0	1622	0
0	0	0	235	73	160	117	184	0	65	114	0	0	0	0	0
0	0	0	0	0	0	0	0	0	0	0	0	0	0	0	0
0	0	0	0	0	0	0	0	0	0	0	0	0	0	0	0
0	0	55	0	0	0	105	0	0	255	0	0	57	0	257	0
0	0	0	893	189	380	172	35444	237	216	169	0	4050	0	0	0
0	0	0	0	0	0	0	0	0	0	0	0	0	0	0	0
0	0	0	0	0	0	0	0	0	69	0	0	0	0	0	0
0	0	0	84	0	0	0	42	0	0	37	0	4866	52	0	262
0	0	0	0	0	0	0	0	0	0	0	0	3188	49	0	0
0	0	0	0	0	0	0	0	0	0	0	0	0	0	0	0
0	0	0	0	0	0	0	0	0	0	0	0	0	0	0	0
0	0	0	0	0	0	0	0	0	0	0	0	0	0	0	0
0	0	0	0	0	0	0	0	0	0	0	0	0	0	0	0
72	32	42	0	0	0	0	0	0	0	433	308	56	191	34	255
0	0	0	0	66	0	0	0	0	0	0	157	0	61	0	113
0	0	0	0	0	0	0	0	0	0	0	0	0	0	0	0
0	0	0	0	0	0	0	0	0	0	0	0	0	0	0	0
0	0	0	0	0	0	0	0	0	0	0	0	0	0	0	0
0	0	0	0	0	0	0	0	0	0	0	0	0	0	0	0
0	0	0	0	0	0	0	0	0	0	0	0	0	0	0	0
0	0	0	0	0	0	0	0	0	0	0	0	0	0	0	0
0	0	0	0	0	0	0	0	0	0	0	0	0	0	0	0
0	0	0	0	0	176	0	0	28685	79	0	78	0	0	515	51
0	0	0	0	0	0	0	0	0	0	0	0	0	0	0	0
91	0	0	0	0	0	0	0	0	0	780	0	0	0	157	0
0	0	0	0	0	0	0	0	0	0	0	0	0	0	0	0
65	6720	87	841	150	146195	119	34697	5544	329	258	15810	7710	137	46	124
0	32	0	0	0	61	0	0	0	0	0	78	12357	58	47	59
282	43702	211	1000	484	1330	648	32343	580	963	196136	0	0	0	0	0
82	669	148	571	538	27402	684	44663	1227	1118	841	125530	2518	877	255	824
80	59859	47	2410	210	6139	124	73325	26587	684	191	72871	14792	306	236	424
45	0	37	0	0	0	0	0	0	0	0	0	101	0	0	0
0	0	0	0	0	0	0	0	0	0	0	0	0	0	0	0
0	0	0	0	0	0	0	0	0	0	0	0	0	0	0	0
0	0	0	0	0	0	0	0	0	0	0	0	0	0	0	0
0	0	0	0	0	0	0	0	0	0	0	0	0	0	0	0
0	0	0	0	0	0	0	0	0	0	0	0	0	0	0	0
0	0	0	0	0	0	0	0	0	0	0	0	0	0	0	241
0	0	0	0	0	0	0	0	0	0	0	0	0	0	2292	0
0	0	0	0	0	0	0	0	0	0	0	0	0	0	0	0
0	0	0	0	0	0	0	0	0	0	0	0	0	0	0	0
0	0	0	0	0	0	0	0	0	0	0	0	0	0	0	0
38	27	45	155	186	146	0	29	67	0	0	0	0	0	0	0
0	0	0	0	0	0	63	0	0	0	75	0	0	0	0	0
0	0	0	0	0	0	0	0	0	0	0	0	0	0	0	0
0	30	0	0	0	0	0	0	0	0	0	0	0	114	0	0
0	0	0	0	0	0	0	0	0	0	0	0	0	0	0	0
0	0	0	0	0	0	0	0	0	0	0	0	0	49	0	0
0	0	0	1304	0	0	56	0	0	0	0	0	0	0	0	0

0	0	0	0	0	0	0	0	0	0	0	0	0	0	0	0
1326	172711	537	5119	154444	229332	7314	75001	130736	2677	15336	50489	38404	2917	43891	8026
0	0	0	0	0	0	0	0	0	0	0	0	0	67	0	0
0	0	0	0	0	0	0	0	0	0	0	0	0	0	0	0
0	0	0	0	0	0	0	0	0	0	0	0	41	0	0	0
0	0	0	0	0	0	0	0	0	0	0	0	0	0	0	0
0	0	0	0	0	0	0	0	0	0	0	0	0	0	0	0
0	0	0	0	0	0	0	0	0	0	0	0	0	0	0	0
128	0	82	0	0	0	0	0	0	0	0	0	0	0	0	0
0	0	0	0	0	0	0	0	0	0	0	0	0	0	0	0
0	0	0	0	0	0	0	0	0	0	0	0	0	0	0	0
0	0	0	0	0	0	0	0	0	0	0	0	0	0	0	0
0	0	0	0	0	0	0	0	0	0	0	0	0	0	0	0
0	0	0	0	0	0	0	0	0	0	0	0	0	368	0	0
0	0	0	98	0	154	59	80	55	0	83	0	0	0	0	72
0	0	0	0	0	0	0	0	0	0	0	0	0	0	0	0
0	0	0	0	0	77	58	0	0	0	0	0	0	0	0	0
0	0	0	0	0	0	0	0	0	0	0	0	0	0	0	0
0	0	0	0	0	0	0	0	5990	0	43	0	0	0	0	0
0	0	0	79	68	91	116	0	61	71	33	0	0	0	0	0
0	0	0	0	0	0	0	0	59	0	0	0	0	0	0	0
0	0	0	0	0	0	0	0	0	0	0	0	0	0	0	0
0	0	0	0	0	0	0	0	0	0	0	0	0	0	0	0
0	0	0	0	0	0	0	0	0	0	0	0	0	0	0	0
0	0	0	0	0	0	0	0	0	0	0	0	0	0	0	0
0	0	0	0	0	68	50	42	14985	58	44	0	0	0	0	0
0	0	0	0	0	0	0	0	0	0	0	0	0	0	0	0
0	0	0	0	0	0	0	0	0	0	0	0	0	0	0	0
0	0	0	240	135	239	182	156	177	348	125	0	0	0	0	670
0	0	0	0	0	0	0	0	0	0	0	0	0	0	0	0
0	0	0	0	0	0	0	0	0	0	0	0	0	0	0	0
0	0	0	0	0	0	0	0	0	0	0	0	0	0	0	0
37	33	57	0	0	0	0	0	0	0	0	0	0	0	0	0
0	0	0	0	0	0	0	0	0	0	0	0	11909	0	0	0
0	0	0	0	0	0	0	0	0	0	0	0	0	0	0	0
0	0	0	0	0	0	0	0	0	0	0	0	0	0	0	0
0	0	0	0	0	0	0	0	0	0	0	0	0	0	0	0
0	0	0	228	80	112498	0	132	54	71	152	0	0	0	0	0
0	0	49	0	0	0	0	0	0	0	0	171	0	0	39	0
0	0	0	0	0	0	0	0	0	0	0	0	0	0	0	0
0	0	0	0	0	0	0	0	0	0	0	0	0	0	0	0
0	0	0	0	0	0	0	0	0	0	0	0	0	0	0	0
961	1089	513	36752	10728	288	221	10448	7138	126	1987	6823	16295	529	203	141271
0	0	0	0	0	0	0	0	0	0	0	0	0	0	0	0
0	0	0	0	0	0	0	0	0	0	0	0	0	0	0	0
0	0	0	0	0	0	0	0	0	0	0	0	0	0	0	0
0	0	0	0	0	0	0	0	0	0	0	0	0	0	0	0
0	0	0	0	0	0	0	0	0	0	0	0	0	0	0	0
0	0	0	0	0	0	0	0	0	0	0	0	0	0	0	0
0	0	0	0	139	0	0	0	0	0	0	0	0	247	0	0
0	0	0	0	0	0	0	0	0	0	0	0	0	0	0	0
0	0	0	90	56	0	0	31865	117	182	41	0	0	0	0	0
0	0	0	0	0	0	0	0	0	0	0	0	0	0	0	0
0	0	0	165	0	0	0	0	63	0	0	0	0	679	272	116
0	0	0	0	0	0	0	0	0	0	0	0	0	423	0	0
0	0	0	0	0	0	0	0	0	0	0	0	0	0	0	0
0	0	0	0	0	0	0	0	0	0	0	0	0	0	0	889
0	0	0	0	0	0	0	0	0	0	0	0	0	0	0	0

47	0	0	0	0	0	0	0	0	0	0	0	0	0	0	182
0	0	0	0	0	0	0	0	0	0	0	0	0	0	0	0
0	0	0	0	0	0	0	0	0	0	44	0	0	0	0	0
0	0	40	0	0	0	0	0	0	0	0	75	0	0	0	0
0	0	0	0	0	0	0	0	0	0	0	0	0	0	0	0
0	0	0	0	0	0	0	0	0	0	0	0	0	0	0	0
79	27	69	620	54073	284	0	4992	124	0	4496	83	57	27963	212	2943
0	0	0	82	0	0	95	43	0	133	0	0	0	46	0	0
0	0	0	84	62	161	42	62	0	154	95	0	0	0	0	0
0	0	0	0	0	0	0	0	0	0	0	0	0	0	0	0
0	0	0	0	0	0	201	42	58	58	44	0	0	0	0	0
0	0	0	0	0	0	0	0	0	0	0	0	0	0	0	0
0	0	0	0	0	0	0	0	0	0	0	0	0	0	0	0
0	0	0	0	0	0	0	0	0	0	0	0	0	0	0	0
69693	37317	129605	47754	90666	18206	546	19027	15215	5514	24472	27586	12651	29556	6480	22564
0	0	0	0	0	0	0	0	0	0	49	0	0	0	0	0
0	0	0	1226	0	81	0	0	0	0	4436	0	0	0	0	810
0	26	0	0	0	0	0	0	0	0	0	0	0	0	0	0
0	0	0	0	0	0	0	0	0	0	0	0	0	0	0	0
0	0	0	0	0	0	0	0	0	0	0	575	63	123	171	402
0	0	0	0	0	0	0	0	0	0	0	0	0	0	0	0
0	0	0	0	0	0	0	0	1523	0	0	87	0	0	5432	4886
0	0	0	0	0	0	0	0	0	0	0	0	0	0	0	0
0	0	0	0	0	87	0	0	0	0	0	0	0	55	0	0
0	0	0	0	0	0	0	0	0	0	0	0	0	0	0	0
0	28	0	0	0	0	0	0	0	0	0	0	225	0	0	2109
0	0	0	0	0	0	0	0	0	0	0	0	0	0	0	0
0	0	0	0	0	0	0	0	0	0	0	0	0	0	0	0
0	36	0	0	234	0	0	0	0	0	2266	0	0	129	0	65
0	0	0	0	0	0	0	0	0	0	0	0	0	0	0	0
0	0	0	0	0	0	0	0	58	124	97	0	0	0	0	0
0	0	0	0	0	0	0	0	0	0	0	0	0	0	0	0
0	0	0	0	0	0	0	0	0	0	0	0	0	0	0	0
0	0	0	0	0	0	0	0	0	0	0	0	0	0	0	0
0	0	0	0	0	0	0	0	0	0	0	0	0	0	0	0
0	0	0	0	0	0	0	0	0	0	0	13947	0	0	0	0
0	0	0	0	0	0	0	0	0	0	0	0	0	0	0	0
0	0	0	0	0	0	0	0	0	0	0	0	0	0	0	0
0	0	0	0	0	0	0	0	0	0	0	0	0	0	0	0
0	0	0	0	0	0	0	0	0	0	0	0	0	0	0	0
0	0	0	0	0	0	120	0	64	0	0	0	0	0	0	0
0	0	0	0	0	0	0	0	0	0	0	0	0	0	0	0
0	0	0	0	0	0	0	0	0	0	0	0	0	71	0	0
0	0	0	0	0	0	0	0	0	0	0	0	0	0	0	0
33	31	0	330	0	0	49	0	0	60	458	0	91	5259	0	181
0	0	0	0	0	0	0	0	0	0	0	0	0	0	0	0
0	0	0	0	0	0	0	0	0	0	0	0	0	0	0	0
0	0	0	0	0	0	0	0	0	0	0	0	0	0	0	0
0	0	0	0	0	0	0	0	0	0	0	0	0	0	0	0
0	0	0	0	0	0	0	0	0	0	0	0	0	251	0	0
0	0	0	0	0	0	0	0	0	0	0	0	0	0	0	0
0	0	0	0	0	0	0	0	0	0	0	0	0	0	0	0
0	0	0	0	0	0	0	0	0	0	0	0	0	0	0	0
0	0	0	0	0	0	0	0	0	0	0	0	0	0	0	0
51792	535	0	0	0	0	0	0	0	56	38	155	0	131	0	124
0	0	0	0	0	0	0	0	0	0	0	0	0	0	0	0
0	22572	0	0	0	0	0	0	0	0	0	0	0	0	0	0
0	0	0	0	0	0	57	0	0	0	0	76	43	72	0	65

0	0	0	0	0	0	0	0	0	0	0	0	0	0	0	0
0	0	0	0	0	0	0	0	0	0	0	0	0	0	0	0
0	0	0	0	0	0	0	0	0	0	0	0	0	0	0	0
0	0	0	329	58	0	0	0	6778	65	0	0	0	0	0	0
0	4740	0	0	0	0	0	0	0	0	997	0	0	564	0	0
0	0	0	0	0	0	0	0	0	0	0	0	0	0	0	0
0	0	0	0	0	0	0	0	0	0	0	0	0	0	0	0
0	0	0	0	0	0	0	0	0	0	0	0	0	0	0	0
0	0	0	0	0	0	0	0	0	0	0	0	0	0	0	0
0	0	0	0	0	0	0	0	0	0	0	0	0	0	0	0
0	0	0	0	0	0	0	0	0	0	0	0	0	0	0	0
0	0	0	86	0	0	63	26	0	0	75	0	0	0	0	0
0	0	0	0	0	0	0	0	0	0	0	0	0	0	0	0
0	0	0	0	0	0	0	0	0	0	0	0	0	0	0	0
0	0	0	0	0	0	0	0	0	0	0	0	0	0	0	0
0	0	0	0	0	0	0	0	0	0	0	0	0	0	0	0
0	0	0	0	0	0	0	0	0	67	0	0	0	0	0	0
0	0	0	0	0	0	0	0	0	0	0	0	0	0	0	0
0	0	0	0	0	229	165	0	50	0	84	0	0	0	0	0
0	0	0	0	0	74	0	0	0	0	0	0	0	0	0	0
0	0	0	0	53083	75	243	68	100	0	73	0	0	0	0	0
0	0	0	0	0	0	0	0	0	0	0	0	0	0	0	0
0	0	0	0	0	0	0	0	0	0	0	0	0	0	0	0
0	0	0	0	0	0	0	0	0	0	0	239	0	56	122	0
0	0	0	0	0	0	0	0	0	0	0	0	0	0	0	0
0	0	0	0	0	0	0	0	0	0	0	0	46	0	0	269
0	0	0	0	0	0	0	0	0	0	0	0	4817	0	0	72
0	0	0	0	0	0	0	0	0	0	0	0	0	0	0	0
0	0	0	0	0	0	0	0	0	0	0	0	0	0	0	0
0	0	0	0	0	0	0	0	0	0	0	0	0	0	0	0
38	0	46	0	0	0	0	0	0	0	0	0	0	0	0	0
316377	35096	159	73	68	84	0	29	5811	141	0	21603	0	66	1399	0
0	0	0	0	0	0	0	98	0	0	0	0	0	0	0	0
0	0	0	0	0	0	0	0	0	0	0	0	0	0	0	0
0	0	0	0	0	0	0	0	0	0	0	0	0	0	0	0
0	6205	0	1981	0	81	72	16358	179	0	601	4768	3345	117	314	173
41	0	0	0	0	0	0	0	0	0	0	0	0	0	0	0
37269	62597	560	1417	609	50990	246	121530	137504	1167	2618	334389	7119	878	2883	603
0	28	95	669	0	73	123	79	3594	130	78	18197	4445	0	0	70
0	0	0	1221	0	0	0	0	0	0	0	0	0	0	0	0
0	0	0	952	0	0	0	0	0	0	0	0	0	0	0	0
0	0	0	0	0	0	0	0	0	0	613	0	0	0	0	0
0	0	0	0	0	0	0	0	0	0	0	0	0	0	0	0
0	0	0	0	0	0	0	0	1451	0	0	0	0	0	0	0
0	0	0	0	0	0	0	0	0	0	0	0	0	0	0	0
52	60	0	1172	1067	1931	1189	748	5004	1221	1035	593	103	67	113	321
0	0	0	0	0	0	0	0	0	0	0	0	0	0	0	0
0	0	0	0	913	73	0	0	0	0	43	0	0	671	0	60
0	0	0	0	0	0	0	0	0	0	0	0	0	0	0	0
0	0	0	0	0	0	0	0	0	0	0	0	0	68	0	0
0	0	0	0	148	0	53	0	0	0	0	0	0	0	0	0
0	0	0	0	0	0	0	0	0	0	0	0	0	0	0	0
0	0	0	0	0	0	0	0	0	0	0	0	1504	0	0	0
0	0	45	446	509	587	640	245876	404	948	487	0	0	0	0	0
0	0	0	946	0	0	0	106	0	0	0	0	0	0	0	0
0	0	0	0	0	148	57	0	54	123	28	0	0	0	0	0
0	0	0	0	0	0	0	0	3035	0	0	0	0	0	7565	1326
0	0	0	0	0	0	0	0	0	0	0	0	0	0	0	0

0	0	0	0	0	0	0	0	0	0	0	0	0	0	0	0
0	0	0	0	0	0	0	0	0	0	0	0	0	0	0	0
0	0	0	0	0	0	0	0	0	0	0	0	0	0	104	0
0	0	0	0	0	0	0	0	0	0	0	0	0	0	0	0
0	0	0	0	0	0	0	0	0	0	0	0	0	0	0	0
0	0	0	0	0	0	0	0	0	0	0	0	0	0	0	0
0	0	0	0	0	0	0	0	0	0	0	0	0	0	0	0
0	0	0	1059	0	0	0	0	0	0	0	0	0	0	0	0
0	0	0	73	0	0	0	0	0	0	0	0	0	0	0	0
0	0	0	0	0	79	0	95	129	128	77	0	0	0	0	0
0	0	49	0	63	0	0	0	0	0	4411	0	0	0	0	0
34	27	0	0	54	0	0	0	0	0	0	0	0	0	0	0
0	0	0	0	0	0	0	0	0	0	0	151	0	52	0	68
0	0	0	0	0	0	0	0	0	0	0	0	0	0	0	0
40	0	0	0	0	0	0	0	0	0	0	78	0	0	0	377
0	0	0	0	0	0	0	0	0	0	0	0	0	220	0	0
0	0	0	0	0	0	0	0	0	0	0	0	0	0	0	0
0	0	0	0	0	0	0	0	0	0	0	0	0	0	0	0
0	0	0	0	0	0	0	0	0	0	0	0	0	0	0	0
0	0	0	0	0	0	0	0	0	0	0	0	0	0	0	0
0	0	28	0	66	76	0	0	54	0	0	72	0	0	0	663
41	0	0	0	63	0	0	0	0	0	35	80	0	669	0	0
0	0	0	0	0	0	0	0	0	0	0	0	0	0	0	0
0	0	0	0	0	0	0	0	0	0	0	0	0	0	0	0
0	0	0	0	0	0	0	0	0	0	0	0	0	0	0	0
0	0	0	0	0	0	0	0	0	0	0	0	0	0	0	0
1731	1505	3545	2165	3792	35918	0	30	786	142	6942	0	59	8025	0	1206
0	0	0	0	0	0	0	0	0	0	0	0	0	0	0	0
0	0	0	0	0	0	0	0	0	0	0	0	0	0	0	0
0	0	0	0	0	0	0	0	0	0	0	0	0	0	0	0
38	24319	0	0	0	0	0	0	0	0	0	0	0	0	0	0
0	0	0	0	0	0	0	0	610	0	0	0	0	0	1580	966
0	0	0	0	0	0	0	0	0	0	0	0	0	0	0	0
0	0	0	0	0	0	0	0	0	0	0	0	0	0	0	0
0	0	0	0	0	0	0	0	0	0	0	0	0	0	0	0
0	0	0	0	0	0	0	0	0	0	0	0	0	0	0	0
42	11227	0	0	0	458	0	0	0	0	0	0	103	0	0	0
0	0	0	0	0	0	0	0	0	0	0	0	0	0	0	0
0	0	0	0	0	0	0	0	0	0	0	0	0	0	0	0
0	0	0	0	0	0	0	0	0	0	0	0	0	0	0	0
0	0	0	0	0	0	0	0	0	0	0	0	0	0	0	0
0	0	0	0	0	0	0	0	0	0	0	0	0	0	0	0
0	29	0	0	0	21015	0	0	0	0	0	0	1466	0	0	223
0	0	0	0	0	0	0	13342	0	0	0	0	0	0	0	0
0	55	0	0	0	13672	0	36	0	0	0	0	313	0	0	0
0	0	0	0	0	0	0	0	0	0	0	0	0	0	0	0
0	0	0	0	0	0	0	0	0	0	0	0	0	0	63	0
0	0	0	0	0	0	0	0	0	0	0	0	0	0	0	0
0	0	0	0	0	0	0	0	0	0	0	0	0	0	0	0
0	0	0	0	0	0	0	0	0	0	0	0	0	0	0	0
0	0	0	0	0	0	0	0	0	0	0	0	0	0	0	0
0	0	0	0	0	0	0	0	0	0	0	0	0	0	0	0
53	68	0	0	0	0	0	0	0	0	0	0	0	0	0	0
0	0	0	0	0	0	0	0	0	0	0	85	0	129	2755	51
0	0	0	0	0	0	0	0	0	0	0	0	0	0	0	0
47	31836	100	0	0	0	0	0	0	0	0	0	0	0	0	0
0	0	0	0	0	0	0	0	0	0	0	0	0	0	0	0
0	58	0	315	337	0	0	0	0	133	1336	0	0	3626	0	899

0	0	0	0	0	0	0	0	0	0	0	0	0	0	0	0
0	0	0	0	0	0	0	0	0	0	0	0	0	0	0	0
0	0	0	0	0	0	0	0	0	0	0	0	0	0	0	0
0	0	0	0	0	0	0	0	0	0	0	0	0	0	0	0
0	0	0	0	0	0	0	0	0	0	0	0	0	0	0	0
0	0	0	0	0	0	0	0	0	0	0	0	0	0	0	0
0	0	0	0	0	0	0	0	0	0	0	0	0	0	0	0
0	0	0	0	0	0	0	0	0	0	0	0	0	0	0	0
0	0	0	0	0	0	0	0	0	0	0	0	0	0	0	0
0	0	0	0	0	0	0	0	0	0	0	0	0	0	0	0
0	0	0	0	0	0	0	0	0	0	0	0	0	0	0	0
0	0	0	0	0	0	0	0	0	0	0	0	0	0	0	0
0	0	0	0	0	0	0	0	0	0	0	151	278	913	46	415
0	0	0	0	0	0	0	0	0	0	0	0	0	0	0	0
0	0	0	87	0	0	0	0	0	0	39	0	0	0	0	0
0	0	0	0	0	0	0	0	0	0	0	0	0	0	0	0
0	0	0	0	0	0	0	0	0	0	0	0	0	0	0	0
0	0	0	0	72	54	0	0	0	71	0	0	0	415	0	0
0	0	0	0	0	0	0	0	0	0	0	0	0	0	0	0
0	0	0	0	0	0	0	0	0	0	0	0	0	0	0	0
0	0	0	0	0	0	79	0	0	0	0	69	0	0	0	0
0	0	0	0	0	0	0	0	0	0	0	0	0	0	0	0
0	0	0	0	0	0	0	0	0	0	0	0	0	0	0	0
0	0	0	0	0	0	0	0	0	0	0	0	0	0	0	0
0	0	0	0	0	0	0	0	0	0	0	0	0	0	0	0
0	0	0	0	0	0	0	0	0	0	0	0	0	0	0	0
0	0	0	0	0	0	0	0	0	0	0	0	0	0	0	0
0	0	0	0	0	0	0	0	0	0	0	0	0	0	0	0
36	0	74	499	0	0	62	0	0	0	45	0	0	0	0	0
0	0	0	0	0	0	0	0	0	0	0	0	0	0	0	0
0	0	0	0	0	0	0	0	0	0	0	0	0	0	0	0
0	0	0	0	0	0	0	0	0	0	0	0	0	0	0	0
0	0	0	0	0	0	0	0	0	0	0	0	0	0	0	0
0	0	0	0	0	0	0	0	0	0	0	0	0	0	0	0
0	0	0	0	0	0	0	0	0	0	0	0	0	0	0	0
0	0	0	0	0	76	57	0	124	0	40	0	0	0	0	0
0	0	0	82	0	0	0	0	0	0	0	0	0	0	0	0
0	0	0	0	0	0	0	0	0	0	0	0	0	0	0	0
0	0	0	0	0	0	0	0	0	0	0	0	0	0	0	0
0	0	0	0	0	0	0	0	0	0	0	0	0	0	0	0
0	0	0	0	0	0	0	0	0	0	0	0	0	0	0	0
0	0	0	0	0	0	0	0	0	0	0	0	0	0	0	0
0	0	0	0	0	0	0	0	0	0	0	0	0	0	0	0
0	0	0	0	0	0	0	0	0	0	0	0	0	0	0	0
0	0	0	0	85	0	0	0	0	0	0	0	0	0	0	0
0	0	0	0	0	0	0	0	0	0	0	0	0	0	0	0
0	0	0	0	0	0	0	0	0	0	0	0	0	0	0	0
0	0	0	1065	0	0	0	0	0	0	0	0	0	0	0	0
0	0	0	0	0	0	0	0	0	0	0	0	1100	0	0	0
0	0	0	0	0	82	0	46	46	0	0	0	0	0	0	0
0	0	0	0	0	0	0	0	0	0	0	0	0	0	0	0
0	0	0	0	0	0	0	0	0	0	0	0	0	0	0	0
577	349	564	634	601	297	69	623	2189	390	1173	0	45	11579	57	19115
0	0	0	0	0	0	0	0	0	0	0	0	0	0	0	0
0	0	0	0	0	0	0	0	0	0	0	0	0	0	0	0
0	0	0	0	0	0	0	0	0	0	0	0	0	0	0	0
0	0	0	0	0	0	0	0	407	0	0	0	0	0	598	1756
0	0	0	0	0	0	0	0	0	0	0	0	0	0	0	0

0	0	0	0	0	0	0	0	0	0	0	0	0	0	0	0
0	0	0	0	0	0	0	0	0	0	0	0	0	0	0	0
0	0	0	0	0	0	0	0	0	0	0	0	0	0	0	0
0	0	0	0	0	0	0	0	0	0	0	0	0	0	0	0
0	0	0	0	0	0	0	0	0	0	0	0	0	0	0	0
44	0	0	0	0	0	0	0	0	0	0	0	0	0	0	0
0	0	0	0	0	0	0	0	0	0	0	0	0	0	0	0
0	0	0	0	0	0	0	0	0	0	0	0	0	0	0	0
0	0	0	0	0	0	0	0	0	76	0	82	52	99	0	67
0	0	0	0	0	0	0	0	0	0	0	0	0	0	0	0
0	0	0	0	0	0	0	0	0	0	0	769	0	0	0	0
0	0	0	0	0	0	0	0	0	0	0	0	0	0	0	0
324	126	272	44026	73	89	171	1508	290	0	39	323	0	116	0	334
0	0	0	0	0	0	0	0	0	0	0	64415	0	0	0	0
403	0	50	0	0	0	0	11053	0	69	95	0	0	0	1440	604
0	0	0	0	0	0	0	0	0	0	0	0	0	0	0	0
0	0	0	0	0	0	0	0	0	0	0	0	0	0	0	258
0	0	0	0	0	0	0	0	0	0	0	0	0	0	0	0
0	0	0	0	0	0	0	0	0	0	0	0	0	0	0	0
0	0	0	0	0	0	0	0	0	0	0	0	0	0	0	0
0	0	0	0	0	0	0	0	0	0	0	0	0	0	0	0
0	0	0	1039	438	793	970895	20672	421	1154	861	82197	0	62	0	0
0	0	0	0	0	0	0	0	0	0	0	0	0	0	0	0
0	0	0	0	0	0	0	0	0	0	0	0	0	0	0	0
0	0	0	0	0	0	0	0	0	0	0	0	0	0	0	0
0	0	0	0	0	0	0	0	0	0	0	240	95	122	97	0
0	0	0	0	0	0	0	0	0	0	0	0	0	0	0	0
0	0	0	0	0	70	0	0	0	0	0	0	0	0	0	0
0	0	0	1369	0	0	0	0	0	64	0	0	0	0	0	0
535	358	95	12331	33460	597	1104	441	2378	548	27206	254	1158	55459	150	30980
0	0	0	0	0	0	0	0	0	0	0	0	0	0	0	0
0	0	0	0	0	0	0	0	0	0	0	0	0	0	0	0
0	0	0	0	0	0	0	0	0	0	0	0	0	0	0	0
0	0	0	0	0	0	0	0	0	0	0	0	0	0	0	0
0	0	0	0	0	0	0	0	0	66	0	0	0	0	0	0
0	27	0	0	0	0	0	0	0	0	0	0	0	0	0	0
0	0	0	0	0	0	0	0	0	0	0	0	0	0	0	0
0	0	0	0	0	0	57	0	0	123	0	0	0	0	0	0
0	0	0	0	0	0	0	0	0	0	0	0	0	0	0	0
0	0	0	0	0	0	0	0	0	0	0	0	0	0	0	0
0	0	0	0	0	0	0	0	0	0	0	312	174	57	34	135
0	0	0	0	0	0	0	0	0	0	0	0	0	0	0	0
0	0	0	0	0	0	0	0	0	0	0	0	0	0	0	0
0	0	0	0	0	0	0	0	0	0	0	237	65	163	0	50
38	0	82	0	0	0	0	0	0	0	0	0	0	0	0	0
0	0	0	0	0	0	0	0	0	0	0	0	0	0	0	0
0	0	0	0	0	0	0	0	0	0	0	0	0	0	0	0
0	0	0	0	0	0	0	0	0	0	0	0	0	0	0	0
36	0	0	0	0	0	49	35	0	0	2163	0	0	0	0	0
0	0	0	0	0	0	0	0	0	0	0	0	0	0	0	0
0	0	0	0	0	0	0	0	0	0	0	0	0	0	0	0
0	0	0	0	0	0	0	0	0	0	0	0	0	0	0	0
0	0	0	0	0	0	0	0	0	0	0	0	0	0	0	0
0	0	0	0	0	0	0	0	0	0	0	0	0	0	0	0
0	0	0	0	0	0	0	0	0	0	0	0	0	0	0	0
0	0	0	817	0	0	0	0	0	0	0	0	0	0	0	0
0	0	0	0	0	0	0	0	0	0	0	0	0	0	0	0
0	0	0	0	0	0	0	0	0	0	0	0	0	0	0	0

0	0	0	0	0	0	70	16250	0	78	0	0	0	0	0	0
42	0	42	0	0	0	0	0	0	0	0	0	0	0	0	0
41	0	48	0	0	0	0	0	0	0	0	0	0	0	0	0
0	39	98	234	209	228	185	185	176	96580	0	0	0	1035	0	59
0	0	0	0	0	0	0	0	0	0	0	0	0	0	0	0
0	0	0	80	61	75	71	0	4285	0	0	18666	0	0	0	63
0	0	0	0	0	0	0	69	0	0	0	0	0	1311	0	0
0	0	0	0	0	0	0	0	0	0	0	0	0	0	0	0
0	0	0	0	0	0	0	0	0	0	0	0	0	0	0	0
0	0	0	0	0	0	0	0	0	0	0	0	0	0	0	0
0	0	0	0	0	0	255	49	238	107	113	0	0	0	0	0
0	0	0	0	0	0	0	0	0	0	0	0	0	0	0	0
0	0	0	0	0	0	0	0	0	0	0	0	0	0	0	0
0	0	0	0	0	0	0	0	0	0	0	0	0	0	0	0
0	12111	0	82	0	71	0	0	0	79	0	0	0	0	0	0
0	0	0	97	0	84	0	32	48	0	0	0	0	0	0	0
0	0	0	81	0	0	0	23515	112	52	0	0	0	0	0	306
0	0	0	0	0	0	0	0	510	0	0	0	0	0	1949	2152
0	0	0	0	0	0	0	0	0	0	0	0	0	0	0	378
0	0	0	0	0	0	0	0	0	0	0	0	0	0	0	0
0	25	0	0	0	0	0	0	0	0	0	394	122	112	57	268
0	0	0	72	61	154571	178	39	123	433	37	0	0	0	0	0
0	0	0	0	0	0	57	139	0	178	112	0	0	0	0	0
0	0	0	0	0	0	0	0	0	0	0	0	0	0	0	0
0	0	0	0	0	0	0	0	0	0	0	0	0	0	0	0
0	0	0	0	0	0	0	0	0	0	0	0	0	0	0	0
0	0	0	0	0	0	0	0	0	0	0	0	0	0	0	0
0	0	0	0	0	0	0	0	0	0	0	0	0	0	0	0
0	0	0	0	0	0	0	0	0	0	0	0	0	0	0	0
0	0	0	0	0	0	0	0	0	0	0	0	0	0	0	0
0	0	0	0	0	0	0	0	0	0	0	0	0	0	0	0
0	0	0	0	0	0	0	0	0	0	0	0	0	0	0	0
0	0	0	0	0	0	0	0	0	0	0	0	0	0	0	0
0	0	0	0	0	0	0	0	0	0	0	0	0	0	0	0
0	0	0	0	0	0	0	40	0	0	0	0	0	0	0	0
0	0	0	0	0	0	0	0	0	0	0	0	0	0	0	0
0	0	0	0	0	0	0	0	0	0	0	0	0	0	0	0
0	0	0	0	0	0	0	0	0	0	0	0	0	0	0	0
0	0	235	0	0	0	0	0	0	0	0	0	0	0	0	0
0	0	0	0	0	0	0	0	0	0	0	0	0	0	0	0
0	0	0	0	0	0	0	0	0	0	0	0	0	0	0	0
0	0	0	0	0	0	0	0	0	0	0	0	160	0	0	0
0	0	0	0	130	0	0	0	0	0	0	0	0	255	0	215
0	0	0	0	0	0	0	0	0	0	0	0	0	0	0	0
0	20086	52	0	0	0	0	0	0	0	0	0	0	0	0	0
36	0	98	82	0	0	0	0	0	69	21590	0	0	0	0	0
0	0	0	6241	64	262	44	132	585	544	445	0	0	0	41	53
0	0	0	0	0	0	0	0	0	0	0	0	0	0	0	0
0	0	0	0	0	0	0	0	0	0	0	0	4280	0	0	57
0	0	0	0	0	0	0	0	0	0	0	0	0	0	0	0
0	0	0	0	0	0	0	0	0	0	0	0	0	0	0	0
0	0	0	0	0	0	0	0	0	0	0	0	0	0	0	0
0	0	0	0	0	0	0	0	0	0	0	0	0	0	0	0
0	0	0	0	0	0	0	0	0	0	0	0	0	0	0	0
0	0	0	0	0	0	0	0	0	0	0	0	0	0	0	0
0	0	0	0	0	0	0	0	0	0	0	0	0	0	0	0
0	0	0	0	0	0	0	0	0	0	0	463	84	56	58	123
0	0	0	0	0	0	0	0	0	0	0	147	102	61	0	118

0	0	0	0	0	0	0	0	0	0	0	0	0	0	0	0
0	0	0	0	0	0	0	0	0	0	0	0	0	0	0	0
0	0	0	0	0	0	0	0	0	0	0	0	0	0	0	0
0	22	0	0	0	0	0	0	0	0	0	0	0	287	0	0
0	0	0	0	0	0	0	0	0	0	0	0	0	0	0	0
40	33	0	0	0	0	0	47	0	0	0	166	157	195	113	200
0	0	0	0	0	0	0	0	0	0	0	0	0	0	0	0
0	0	0	388	0	0	0	0	0	0	344	0	0	0	0	0
0	0	0	0	0	0	0	0	0	0	0	0	0	0	0	0
0	0	0	0	0	0	0	0	0	0	0	0	0	0	0	0
0	0	0	0	0	0	0	0	0	0	0	0	0	0	0	0
0	0	0	0	0	0	0	0	0	0	0	0	0	0	0	0
0	0	0	0	0	0	0	0	0	0	0	0	0	0	0	0
0	0	0	0	0	0	0	0	0	0	0	0	0	168	0	0
0	0	0	0	0	0	0	0	0	0	0	0	0	0	0	0
0	0	0	0	0	0	0	0	0	0	0	0	0	0	0	0
0	0	0	0	0	0	0	0	0	0	0	0	0	0	0	1833
0	32	0	0	0	0	0	0	0	0	0	0	0	0	0	0
42	0	57	1864	0	0	0	0	0	0	0	0	0	0	0	0
0	0	0	2357	0	0	0	0	0	0	0	0	0	125	0	0
494137	411116	791820	67257	15520	8247	3492	40127	120717	862299	17464	121091	769544	126458	7367	8216
0	0	0	0	0	0	0	0	0	0	0	0	0	0	0	0
0	0	0	0	0	0	0	0	0	0	0	0	0	0	0	0
39	54	0	12596	0	0	0	90	42	73	726	0	0	877	0	219
0	0	0	0	0	0	0	0	0	0	0	0	0	0	0	0
0	0	0	0	0	0	0	0	0	0	0	0	0	0	0	0
0	0	0	4541	0	0	0	82	0	0	0	0	0	0	0	0
0	0	0	0	0	0	0	0	0	0	0	0	0	0	0	0
0	0	0	0	0	0	0	0	0	0	0	84	6815	0	0	65
0	0	0	0	0	0	0	0	0	0	0	0	0	0	0	0
0	0	0	0	0	0	0	0	0	0	0	0	0	0	0	0
0	0	0	0	0	0	0	0	0	0	0	0	0	0	0	0
0	0	0	0	0	0	0	0	0	0	0	0	0	0	0	0
43	68	0	61	1179	0	62	0	56	0	4379	0	0	1507	0	488

Control110	Control111	Control112	Control115	Control118	Control119	Control121	Control123	Control124	Control126	Control130	Control131	COVID-19-002	COVID-19-003	COVID-19-005	COVID-19-006
952387	694279	820118	776432	408545	954791	975429	975821	482484	875624	816878	135312	941809	966333	893188	961992
32661	282630	177047	100858	365775	16414	2129	13670	474486	85474	104625	821122	24824	6045	16914	5702
0	0	0	0	0	0	0	0	0	0	0	0	0	0	0	0
14553	7085	2117	122060	224111	26687	21471	10509	21053	38595	44716	43566	32785	27304	42859	30462
399	16006	718	650	1569	1909	971	0	21364	307	33781	0	516	163	5148	1746
0	0	0	0	0	0	0	0	0	0	0	0	0	0	0	0
0	0	0	0	0	199	0	0	613	0	0	0	66	155	41891	98

Control110	Control111	Control112	Control115	Control118	Control119	Control121	Control123	Control124	Control126	Control130	Control131	CC002	CC003	CC005	CC006
0	0	0	0	310387	125	35	0	0	0	0	0	0	140	173	283
0	0	0	0	0	0	0	0	0	0	0	0	0	0	0	0
304099	2255	1725	39664	14950	5355	11787	17105	348840	508599	754477	2018	58294	650010	740693	782560
0	0	0	0	0	0	0	0	0	0	0	0	0	0	0	0
0	0	45	0	0	0	0	0	0	0	0	0	134	0	0	0
0	0	0	0	0	0	0	0	0	0	0	0	0	0	0	0
0	0	0	0	0	0	0	0	0	0	0	0	0	0	0	0
0	0	0	0	0	0	0	0	0	0	0	0	0	0	0	0
0	0	0	0	0	0	0	0	0	0	0	0	0	0	0	0
138	262	145	383	1305	433	520	1335	409	11274	218	42689	423	151	0	260
0	0	0	0	0	0	0	0	0	0	0	0	0	0	0	0
0	0	0	0	0	0	0	0	0	0	0	0	0	0	0	0
0	0	0	0	0	0	0	0	0	0	0	0	0	0	0	0
1121	59	51	0	122	33142	25	0	200	4239	708	0	0	11670	3275	1625
0	0	0	0	0	0	0	0	0	123	98	0	64	793	84	302
0	0	0	0	0	0	0	0	0	0	0	0	0	0	0	0
0	0	0	0	0	0	0	0	0	0	0	0	0	0	0	0
0	0	0	0	0	5904	0	0	0	38	93	35390	132	0	0	0
0	0	0	0	0	1375	0	0	0	0	0	0	0	0	0	0
0	0	0	0	0	1174	0	0	0	0	0	0	0	0	0	0
27	0	0	0	0	116	0	0	0	0	0	0	130	0	92	110
0	0	0	0	0	0	0	0	0	0	0	0	0	0	0	0
13456	66	13570	0	0	3569	0	0	0	0	0	0	0	0	95	53
0	0	0	0	0	0	0	0	0	0	0	0	0	0	0	0
0	0	0	0	0	0	0	0	0	0	0	0	0	0	0	0
232784	81577	239632	650	7471	13121	869	149902	18343	6821	39443	61789	171204	4422	17529	1559
0	0	0	0	0	0	0	0	0	0	0	0	0	0	0	0
0	0	0	0	0	0	35	425	0	0	0	63	0	748	0	446
0	0	0	0	0	0	0	435	46	0	0	59	0	0	0	0
0	0	0	0	0	0	0	0	0	0	0	0	0	0	0	0
0	0	0	0	0	0	0	0	0	0	0	0	0	0	0	0
0	0	0	0	0	0	0	0	0	0	186	0	0	0	0	153
0	0	0	0	0	0	0	0	0	0	0	0	0	0	0	0
0	394	0	0	0	0	0	0	0	0	331	0	0	0	0	0
0	0	0	0	0	0	0	0	0	0	0	0	0	632	0	0
0	0	0	0	0	0	0	0	0	0	0	0	0	0	0	0
0	0	0	0	0	0	0	0	0	43	0	0	0	0	0	0
0	0	0	0	0	0	0	0	0	0	0	0	0	0	0	0
0	0	0	0	0	0	0	0	0	0	0	0	0	0	0	0
183825	75475	220937	618	57065	14281	634	6359	375	1051	1219	1097	805	929	1876	932
0	0	0	0	0	0	0	0	8440	0	0	0	0	0	0	0
164	418	195	0	112	0	529	0	0	127	36	58	0	0	0	52
0	0	0	0	0	0	0	0	0	0	0	0	59	0	0	0
0	0	0	0	0	0	0	0	0	0	0	0	0	0	0	0
0	0	0	0	0	0	0	0	0	0	0	0	0	0	0	0
0	0	0	0	0	0	0	0	0	0	0	0	0	0	0	0
174	0	0	0	0	0	0	0	0	479	88	0	0	499	742	861

0	0	0	0	0	0	0	0	0	0	0	0	0	0	0	0
0	0	0	0	0	0	0	0	0	0	0	0	0	0	0	0
0	79	104	113	1147	100	101	472	39	93	74	63	630	26654	2346	2958
0	0	0	0	0	0	0	0	0	0	0	0	61	0	0	0
0	0	0	0	0	0	0	0	0	0	0	0	0	0	0	0
0	0	0	0	0	0	0	0	0	0	0	0	0	0	0	0
0	0	0	0	0	0	0	0	0	0	0	0	0	0	0	53
128	183478	278	71	0	0	290	0	0	0	0	0	0	0	0	0
103	191	0	0	0	0	0	0	0	0	0	0	0	0	0	0
0	0	0	0	0	0	0	0	0	0	0	0	0	0	0	0
0	0	0	0	0	0	0	0	0	0	0	0	0	0	0	0
0	0	0	0	0	0	0	0	0	0	0	0	0	0	0	0
0	0	0	0	0	0	0	0	0	122	40	0	0	0	0	0
0	0	0	0	0	0	0	0	0	0	0	0	0	0	0	0
0	0	0	0	0	0	0	0	0	0	0	0	0	0	0	0
0	0	19601	0	428	73	0	473	102	1386	0	458	0	323	0	0
0	0	0	0	0	0	0	0	0	0	0	0	0	0	0	0
0	0	0	0	0	0	0	0	10592	0	0	60	0	0	0	0
0	0	0	0	0	0	0	0	0	0	38	0	0	0	0	0
1127	214227	1471	340	966	35131	1683	1860	13193	239	681	578	382	1953	309	690
0	0	0	0	0	0	0	0	0	0	42	54	0	0	0	0
0	0	0	0	0	0	0	0	0	0	0	0	0	0	0	0
0	0	0	0	0	0	0	0	0	0	0	0	0	0	0	0
0	0	0	0	0	0	0	0	0	0	0	0	0	0	0	0
0	0	0	0	109	37	0	0	0	0	0	0	0	0	0	0
0	0	0	0	0	0	0	0	0	0	0	0	0	0	0	0
0	0	0	0	0	0	0	0	0	0	0	0	0	0	0	0
0	0	0	0	0	0	0	0	0	0	0	0	0	0	0	0
0	0	0	0	0	0	0	0	19151	0	0	0	0	0	0	0
0	0	0	0	0	0	0	0	0	0	0	0	77	0	0	0
31	0	0	0	0	0	0	0	0	0	0	0	0	0	0	0
0	0	0	0	0	0	0	0	0	0	0	0	0	0	0	0
0	0	0	0	0	0	0	0	0	0	0	0	0	0	0	0
13338	74043	118437	497	358343	7296	626	6850	396730	21704	1595	326130	851	0	0	0
0	0	0	0	0	0	93	0	0	0	0	0	0	0	0	0
0	0	0	0	0	0	0	0	0	0	0	0	0	0	0	0
0	0	0	0	0	0	204	0	0	0	0	0	0	290	82	50
0	0	0	0	0	0	0	0	0	0	0	0	0	0	0	0
0	0	0	0	0	0	0	0	0	0	0	0	0	0	0	0
0	0	0	0	0	0	0	0	0	0	0	0	0	0	0	0
0	0	0	0	0	0	0	0	0	0	0	0	0	0	0	0
0	0	0	0	0	0	0	0	0	0	0	0	0	0	0	0
0	0	0	0	0	0	0	0	0	0	0	0	0	0	0	0
0	0	0	68	109	38	0	0	0	0	0	0	0	0	0	0
0	0	0	0	0	0	0	0	0	0	0	0	0	0	0	0
0	0	0	0	0	0	0	0	0	0	0	0	0	0	0	0
0	0	0	0	0	0	0	0	0	0	0	0	0	0	0	0
0	0	0	0	0	0	0	0	0	0	0	0	0	0	0	0
0	0	0	0	306	119	1833	2313	248	270	256	402	0	0	0	0
0	0	0	0	0	0	0	0	0	0	0	0	0	157	0	0
0	0	101	0	0	0	0	0	0	0	0	0	0	0	0	0
0	0	0	0	0	0	0	0	0	0	0	0	0	0	0	0
0	0	0	0	0	0	0	0	0	0	0	0	67	0	0	0
0	0	0	0	0	0	0	0	0	0	0	0	383	0	0	0
0	0	0	0	0	0	0	0	0	0	0	0	0	0	0	183
399	16006	673	650	1569	1876	971	0	21364	263	33781	0	124	0	5148	1746
0	0	0	0	0	0	0	0	0	82	31	68	0	0	0	0
0	0	0	0	0	0	0	0	0	0	0	0	0	0	0	0

0	0	0	0	0	0	0	0	0	0	0	0	0	0	0	0
0	0	0	0	0	0	0	0	0	0	0	0	0	0	0	0
0	0	0	0	0	0	0	0	0	0	0	0	0	0	0	0
0	0	0	0	0	0	0	0	0	0	0	0	0	310	90	43
0	0	0	0	0	0	0	0	0	0	0	0	0	0	0	0
0	0	0	0	0	0	0	0	0	0	0	0	0	0	0	0
0	0	0	0	0	0	0	0	0	0	0	0	0	0	0	0
0	0	0	0	0	0	41	0	0	0	0	0	0	648	553	343
0	73	0	0	0	0	0	0	0	0	0	0	0	0	0	52
0	0	0	0	0	0	0	0	0	0	0	0	0	1578	660	1091
0	0	0	0	0	0	0	0	0	0	0	0	0	0	0	0
0	0	0	0	0	0	0	0	0	0	0	0	0	0	0	0
0	65	62	0	0	0	175	0	0	0	0	0	0	0	0	0
0	0	0	0	0	0	0	0	0	0	0	0	0	0	0	0
0	0	0	0	0	0	0	0	0	0	0	0	0	0	0	0
260	0	50	60	0	47	0	446	49	11730	0	46	115	17618	2913	6748
684	0	0	0	0	0	0	0	161	1914	486	0	66	1361	1978	1800
0	0	0	0	0	0	0	470	46	97	0	63	0	138	82	145
0	0	0	0	0	0	0	0	0	0	0	0	0	0	0	0
0	0	0	0	0	0	0	0	0	2547	0	0	0	0	0	0
0	0	0	0	0	0	0	0	0	0	0	0	238	0	0	0
0	0	0	0	0	0	0	0	0	0	0	0	0	0	0	0
14553	7085	2117	122060	224111	26687	21430	10509	21053	38595	44716	43566	32785	26656	42306	30119
115	133	204	0	0	0	112	446	0	0	35	65	0	951	0	0
1796	69	1581	0	0	272	0	463	0	0	0	0	0	2004	0	0
0	0	0	0	0	0	0	0	0	0	0	0	0	0	0	0
0	0	0	0	0	0	0	0	0	0	0	0	115	0	0	0
0	0	0	0	0	0	0	0	0	0	0	0	0	0	0	0
0	61	0	0	0	34	0	0	52	0	423	0	0	0	0	140
0	0	0	0	0	0	0	0	0	0	0	0	0	149	84	0
3972	0	0	0	0	0	0	0	284	129	0	0	267	595	0	266
0	0	0	0	0	0	0	0	0	0	0	0	0	0	0	0
0	0	0	0	0	0	0	0	0	0	0	0	0	0	0	0
0	0	0	0	0	0	0	0	0	0	0	0	0	0	0	0
0	0	0	0	207	47	0	451	58	85	0	299	0	0	0	0
0	64	0	0	0	0	0	0	0	0	0	0	0	0	0	0
0	0	0	0	0	0	0	0	0	0	0	0	0	0	0	0
0	0	0	0	0	0	39	463	47	0	0	0	0	0	0	0
0	0	0	0	0	0	0	0	1260	0	0	0	65	0	0	0
0	64	0	0	0	0	0	0	0	0	0	0	0	0	0	0
0	0	0	0	0	0	0	0	0	0	0	0	0	0	0	0
920	0	0	0	0	1167	0	0	0	40	91	0	266	979	498	933
0	0	0	0	0	0	0	0	0	0	0	0	0	0	0	0
0	0	0	0	0	0	0	0	0	0	0	0	0	0	0	0
0	0	0	0	0	0	0	0	0	0	0	0	0	0	0	0
0	0	0	0	0	0	0	0	0	0	0	0	0	0	0	0
0	0	0	0	0	0	0	0	0	0	0	0	0	0	0	56
0	0	0	0	0	0	0	0	44	0	0	0	0	0	0	0
0	0	0	0	0	0	0	0	0	0	0	0	0	0	0	0
0	0	0	0	0	0	0	0	0	0	0	0	0	0	0	0
0	0	0	0	0	0	0	0	0	0	0	0	0	0	0	0
65	60	49	0	102	2785	42	0	0	0	0	0	0	0	75	167
0	0	0	0	0	0	0	0	0	0	0	0	0	326	0	0
0	0	0	0	0	0	39	0	0	0	0	0	0	0	0	0
101	145	93	0	0	0	0	0	0	0	0	0	0	0	0	0
0	0	0	0	0	0	0	0	0	0	0	0	0	0	0	0
0	0	0	0	0	0	0	0	0	0	0	0	0	0	80	103
0	0	0	0	0	0	0	0	0	0	0	0	0	0	0	0

0	0	0	0	0	0	852	0	0	0	0	0	0	480	570	376
0	0	0	0	0	0	0	0	0	0	0	0	0	0	0	0
0	0	0	0	0	0	0	0	0	0	0	0	0	0	0	0
0	0	0	0	0	0	0	0	0	0	0	0	0	0	0	0
0	0	0	0	0	0	35	0	3669	87	0	0	0	0	0	0
0	0	0	0	0	0	0	0	0	0	0	0	0	0	0	0
0	0	0	0	0	0	0	0	0	0	0	0	0	0	0	0
0	0	0	0	0	0	0	0	0	0	0	0	0	0	0	0
0	0	0	0	0	0	0	0	0	0	0	0	0	0	0	0
0	0	0	0	0	0	0	0	0	0	0	10115	0	0	0	0
0	0	0	0	0	0	0	0	0	0	0	0	0	0	0	0
0	0	0	0	0	0	0	0	0	0	0	0	0	0	0	0
0	0	0	0	0	0	0	0	0	0	0	0	0	0	0	0
142	170	162	0	93	0	0	0	0	0	0	99	0	0	0	0
4532	82	0	0	0	0	0	0	0	0	0	0	0	0	0	0
0	0	0	0	0	0	0	0	0	0	0	0	0	0	0	0
0	0	0	0	0	0	0	0	0	0	0	0	0	0	0	0
0	0	0	0	0	0	0	0	0	0	0	0	0	0	0	0
0	0	0	0	0	0	0	440	35	0	0	0	0	0	0	0
0	0	0	0	0	0	0	0	0	0	0	0	0	0	0	0
5566	1058	58083	92983	5861	1236	803	3183	4254	16206	101319	224303	14487	329	332	465
0	0	0	0	0	0	0	0	0	0	0	0	0	0	0	0
0	0	0	0	0	0	0	0	0	0	0	0	0	0	0	0
0	0	0	0	0	0	0	0	0	0	0	0	0	0	0	0
0	0	0	0	0	0	0	0	0	0	0	0	0	0	0	0
66	339	0	0	119	0	10558	464	78	221020	233	46	0	0	0	54
0	0	0	0	0	0	0	0	0	0	0	0	0	0	0	0
0	0	0	0	0	0	0	0	0	0	0	0	0	809	0	238
0	0	0	0	0	0	0	0	0	0	0	0	78	0	0	0
0	0	0	0	0	0	0	0	0	44	0	0	258	0	0	0
0	0	0	0	0	0	0	0	0	0	0	0	0	0	0	0
0	0	0	0	0	0	0	0	0	0	0	0	0	0	0	0
0	0	0	0	109	41	45	0	0	0	41	0	0	0	0	45
0	0	0	0	0	0	0	0	0	0	0	0	0	0	0	0
250	0	149	0	0	35	0	0	0	0	0	0	0	162	0	0
0	0	0	0	94	0	0	0	0	0	0	0	0	0	0	0
0	0	0	0	0	0	0	0	0	0	0	0	0	0	0	0
0	0	0	0	0	0	0	0	0	0	0	0	0	0	0	0
0	0	0	0	0	0	0	0	0	0	0	0	0	0	0	0
0	0	0	0	0	0	0	0	0	0	0	0	0	0	0	0
0	0	0	0	0	0	0	0	0	0	0	0	0	0	0	0
0	0	0	0	0	0	0	0	0	0	0	0	63	0	0	0
0	0	0	0	0	0	0	0	0	0	0	0	0	0	0	0
0	0	0	0	0	0	0	0	0	0	0	0	0	0	0	0
0	0	0	0	0	0	0	0	0	8232	27	0	0	148	0	0
0	0	0	0	0	0	0	0	0	0	0	0	0	0	0	0
99	341	115	0	0	0	0	0	32543	0	116	57	0	323	74	0
28	127	0	0	0	0	0	0	0	0	0	59	0	1207	514	550
0	0	0	0	0	0	0	0	0	0	0	0	0	0	0	0
0	0	0	0	0	0	0	0	0	0	0	0	0	0	0	0
0	0	0	0	0	0	0	0	0	0	0	0	0	0	0	0
0	0	0	0	0	0	0	0	0	0	0	0	0	0	0	0
0	0	0	0	0	0	0	0	0	0	0	0	0	0	0	0
0	0	0	4566	0	2789	0	0	236	67	157	1136	60	481	552	40
0	0	0	0	109	40	0	0	0	0	0	0	0	337	163	198
0	0	0	0	0	0	0	0	0	0	0	0	0	0	0	0
2518	0	0	3512	212	0	34	0	326	6660	2351	0	558	4641	7323	3657
0	0	0	0	0	0	234	0	0	0	0	0	0	0	0	0

0	0	0	0	0	0	0	0	0	0	0	0	0	0	0	0
0	0	0	0	0	0	0	0	0	0	0	0	0	0	0	0
0	0	0	0	0	0	0	0	0	0	0	0	0	0	0	0
130	0	125	0	106	0	0	0	0	0	0	0	0	0	0	122
0	0	0	0	0	0	0	0	0	0	0	0	0	0	0	0
0	0	0	0	0	0	0	0	0	0	0	0	0	0	0	0
0	0	0	0	0	0	0	0	0	0	0	0	0	0	0	169
0	0	0	0	0	0	0	2744	2216	406	166	172	69	0	0	0
33	62	179	68	0	70	39	0	0	0	0	0	0	0	0	0
0	0	0	0	0	0	0	0	0	0	0	0	0	0	0	0
186	0	0	0	0	0	149	477	0	1689	1733	0	73	4952	2297	5427
0	0	0	0	0	5788	0	0	0	0	0	0	0	0	0	0
0	0	0	0	0	0	0	0	0	0	0	0	0	0	0	0
0	0	0	0	0	0	0	0	0	0	0	0	0	0	0	0
0	0	0	0	0	0	0	0	0	0	0	0	0	0	0	0
0	0	0	0	0	0	0	0	0	0	0	0	0	0	0	0
0	0	0	0	0	0	0	438	13892	0	0	76	0	0	0	0
331	262	361	188	1281	312	153	0	50	0	0	0	64	0	0	0
0	0	0	0	0	0	0	0	0	0	0	0	0	0	0	0
0	0	0	0	0	0	0	0	0	0	0	0	0	0	0	0
0	0	0	0	94	84	46	0	0	0	0	0	0	0	0	0
0	0	0	56	0	43	0	0	0	0	0	0	0	276	79	445
0	0	0	0	0	0	0	0	0	0	0	0	0	0	0	0
0	0	0	0	0	0	0	0	0	0	0	0	0	0	0	0
0	0	0	0	0	0	0	0	0	0	0	0	60	0	0	96
0	0	0	46	103	154	176	0	0	0	0	0	0	0	0	0
0	0	0	0	0	0	0	0	0	0	0	0	0	0	0	0
0	0	0	0	0	0	0	0	201	39134	333	105516	255	763	1194	677
0	0	0	0	0	0	0	0	0	0	0	0	0	0	0	0
0	0	0	0	0	0	0	0	0	0	0	0	0	0	0	0
0	0	0	0	0	40	0	0	0	0	0	0	0	0	0	0
0	0	0	0	0	0	0	0	0	0	0	0	0	0	0	0
0	0	0	0	0	0	0	0	0	0	0	0	62	0	0	0
0	0	0	0	0	0	0	0	6780	0	0	0	0	0	0	0
0	0	0	0	0	0	0	0	0	0	0	0	0	0	0	0
0	0	0	0	0	0	0	0	0	0	0	0	0	0	0	0
0	0	0	0	0	0	0	0	0	0	0	0	0	0	0	0
0	0	0	0	0	0	0	0	0	0	0	0	0	0	0	0
0	0	0	0	0	0	0	0	0	0	0	0	0	0	0	0
0	0	0	0	0	0	0	0	0	0	0	0	0	0	0	0
0	0	0	0	0	0	0	0	0	0	0	0	0	0	0	0
0	0	0	0	0	0	0	0	0	0	0	0	0	0	0	0
0	0	0	55	299	123	0	1313	98	0	60	0	0	0	0	0
0	0	0	0	0	0	0	0	0	0	0	0	0	0	0	0
0	0	0	0	0	0	0	460	93	42	38	63	0	0	89	0
0	0	0	0	0	0	0	0	0	0	0	0	0	0	0	0
0	0	0	0	0	0	0	0	0	0	0	0	0	0	0	0
0	0	0	0	0	0	0	0	0	0	0	0	0	0	0	0
5058	197	0	0	0	64	2899	0	2034	98	54	0	847	60795	292	3894
0	0	0	0	0	0	148	0	0	0	43	0	0	0	0	0
0	0	0	0	0	0	0	0	0	0	0	0	0	0	0	0
0	0	0	0	0	0	0	0	0	0	0	0	0	0	0	0
1508	0	5418	0	0	174	35	884	0	128	79	140	0	0	0	0
0	0	0	0	0	0	0	0	0	0	0	0	0	0	0	0
0	0	0	0	0	33	0	0	0	0	0	0	0	163	0	0
0	0	0	0	0	0	0	0	0	0	0	0	0	146	0	198
0	0	0	0	0	0	0	0	0	0	0	0	0	137	168	299
0	0	0	0	0	9227	0	0	0	0	0	0	0	0	0	0

0	0	0	0	0	0	0	0	0	0	0	0	0	0	0	0
0	0	0	0	0	0	0	0	0	0	0	0	0	0	0	0
0	0	0	0	0	0	0	0	0	0	0	0	0	0	0	0
0	0	0	0	0	0	0	0	0	0	0	0	0	0	0	0
153	252	61	7081	304	169	165	463	52408	0	34	177	58	0	12337	61
31	0	0	0	0	0	0	0	0	0	0	0	0	0	0	0
64	0	0	0	0	6993	39	0	0	0	84	66	539	0	0	0
0	0	0	0	0	0	0	0	0	0	0	0	0	0	0	0
0	0	0	0	0	0	0	0	0	0	0	0	1329	0	0	0
0	0	0	0	0	0	0	0	0	0	0	0	0	0	0	0
28	71	0	0	0	0	0	0	0	47	0	0	0	0	0	0
0	0	0	0	0	0	0	0	0	0	0	0	0	0	0	0
0	0	0	0	0	0	0	0	0	0	0	0	0	0	0	0
52	0	0	0	0	0	0	0	0	0	0	0	0	0	0	0
181	153	222	0	0	0	0	0	0	0	0	0	0	0	0	0
0	0	0	0	0	0	0	0	0	0	0	0	0	0	0	0
24011	409	50	121	1178	389	152	1359	10155	81368	3115	184	756	96000	93533	131920
0	0	0	0	0	0	0	0	0	0	0	0	0	0	0	0
0	0	0	0	0	0	0	0	0	0	0	0	0	0	0	0
0	0	0	0	0	0	0	0	0	0	0	0	0	0	0	0
0	0	0	0	0	0	0	0	0	0	0	0	0	0	0	0
0	0	0	0	0	0	0	0	0	0	0	0	0	0	0	0
126	183	40	0	0	0	0	0	0	0	0	0	0	0	0	0
0	0	0	0	0	0	0	0	0	0	0	0	0	0	78	0
0	0	0	0	0	0	0	0	0	0	0	0	0	306	101	55
61	109536	0	0	0	0	0	0	0	0	0	0	0	0	0	0
0	0	0	0	0	0	0	0	0	0	0	0	0	0	0	0
0	0	0	0	0	0	0	0	0	0	0	0	0	0	0	0
0	0	0	0	0	0	0	0	0	0	0	0	0	0	0	0
0	0	0	58	0	78	46	0	0	0	35	0	0	0	0	0
0	0	0	0	0	0	337	0	0	0	0	0	0	0	0	0
0	0	0	0	0	0	0	0	0	0	0	0	0	0	0	0
0	0	0	0	0	0	0	0	0	0	0	0	7943	0	0	0
0	0	0	116	565	201	22	0	0	0	0	0	0	0	0	0
0	0	0	0	0	0	0	0	0	0	0	0	0	0	0	0
26	0	0	0	760	75	41	444	0	91	158	7723	167	1086	173	159
0	0	0	0	0	48	0	0	0	0	0	0	0	157	0	229
0	0	0	0	0	0	0	0	0	0	0	0	0	0	0	0
0	0	0	0	0	0	0	0	0	0	0	0	0	0	0	0
0	0	0	0	0	0	0	0	0	0	0	0	0	0	0	0
0	0	0	0	0	0	0	0	0	0	0	0	0	0	0	964
0	0	0	0	0	38	35	0	0	0	0	0	0	0	0	0
0	0	0	0	0	2604	0	0	0	574	0	57	0	0	0	0
0	0	0	0	0	0	0	0	0	0	0	0	0	0	0	0
0	0	0	0	0	0	0	0	0	0	0	0	0	0	0	0
35	0	163	0	0	0	41	0	0	0	0	0	284	0	0	0
0	0	0	0	0	0	0	0	0	0	0	0	0	0	0	0
32	205939	110	0	0	0	0	0	46	0	0	0	0	157	0	0
0	0	0	0	0	0	0	0	0	0	0	0	0	0	0	0
0	0	0	0	0	0	0	0	0	0	0	0	0	0	0	0
0	0	0	0	0	0	0	0	0	2336	0	0	0	0	0	0
0	0	0	0	0	0	0	0	0	0	0	0	0	0	0	0
0	0	0	0	0	0	0	0	0	0	0	0	0	0	0	0
0	0	0	0	0	0	0	1852	88	87	256	0	0	0	0	0
0	0	0	0	0	0	0	0	0	0	0	0	0	0	0	0
0	0	0	0	0	0	0	0	0	0	0	0	0	0	0	0
0	0	50	952	2022	532	938070	0	0	0	0	0	0	0	0	0
30	0	0	0	0	0	0	0	0	0	0	0	0	0	0	0

0	0	0	0	0	0	0	0	0	0	0	0	0	0	0	57
0	0	0	0	0	0	0	0	0	0	0	0	0	0	0	0
0	0	0	0	0	0	0	0	0	0	0	0	0	0	0	0
0	0	0	0	0	0	0	0	0	0	0	0	0	0	0	0
6926	0	0	0	0	0	40	0	0	0	0	0	0	0	0	0
0	0	0	0	0	0	0	0	0	0	0	0	0	0	0	0
0	0	0	0	0	0	0	0	0	0	0	0	0	0	0	0
0	0	0	0	0	0	0	0	0	0	0	0	0	0	0	0
0	0	0	0	0	0	0	0	0	0	0	0	0	0	0	0
0	0	0	0	0	0	0	0	0	0	0	0	0	0	0	0
0	0	0	0	0	0	71	0	0	0	0	0	0	0	0	0
0	0	0	0	0	0	0	0	0	0	0	0	62	0	0	0
0	0	0	0	0	0	0	0	0	0	0	0	0	0	0	0
0	0	0	0	0	0	0	0	0	0	0	0	0	0	0	0
226	183	387	0	0	0	0	0	0	0	0	0	0	0	0	0
0	0	0	55	183	33	0	1378	90	255	81	129185	0	0	0	0
58	137	44	0	0	0	0	0	0	3407	0	0	58	442	0	0
0	0	0	0	0	0	0	0	0	0	0	0	0	0	0	0
0	0	0	0	0	0	0	0	0	0	0	0	0	0	0	0
0	0	0	0	0	0	0	0	0	0	0	0	0	0	0	0
0	0	0	0	0	0	0	0	0	0	0	0	0	0	0	0
0	0	0	0	0	0	0	0	0	0	0	0	0	0	0	0
0	0	0	0	0	0	0	0	0	0	0	0	0	0	0	0
0	58	0	0	0	0	0	0	0	0	0	0	0	0	0	0
0	0	0	0	0	0	0	0	0	0	0	0	0	0	0	0
174133	24092	313160	724860	7699	814084	2622	782372	8147	5840	10268	5811	701547	72715	16248	12564
0	0	0	0	0	0	0	0	0	92	0	0	0	0	0	0
0	0	0	0	0	0	0	0	0	0	0	0	0	149	0	61
0	0	0	0	0	199	0	0	613	0	0	0	66	155	41891	98
0	0	0	0	0	0	0	0	0	0	0	0	0	0	0	0
0	0	0	0	0	0	0	0	0	0	0	0	0	0	0	0
0	0	0	109	0	0	0	952	822	0	33	0	2520	0	229	0
0	0	0	0	0	0	0	0	0	0	0	0	0	0	0	0

Control110	Control111	Control112	Control115	Control118	Control119	Control121	Control123	Control124	Control126	Control130	Control131	COVID-19-002	COVID-19-003	COVID-19-005	COVID-19-006
0	0	0	0	0	0	0	0	0	0	0	0	0	0	0	0
0	0	0	0	310289	81	35	0	0	0	0	0	0	0	0	0
0	0	0	0	0	0	0	0	0	0	0	0	0	0	0	0
0	0	0	0	0	0	0	0	0	0	0	0	0	0	0	0
0	0	0	0	98	44	0	0	0	0	0	0	0	140	173	283
0	0	0	0	0	0	0	0	0	0	0	0	0	0	0	0
0	0	0	0	0	0	0	0	0	0	0	0	0	0	0	0
304099	2255	1725	39664	14950	5355	11787	17105	348840	508599	754477	2018	58294	650010	740693	782560
0	0	0	0	0	0	0	0	0	0	0	0	0	0	0	0
0	0	45	0	0	0	0	0	0	0	0	0	134	0	0	0
0	0	0	0	0	0	0	0	0	0	0	0	0	0	0	0
0	0	0	0	0	0	0	0	0	0	0	0	0	0	0	0
0	0	0	0	0	0	0	0	0	0	0	0	0	0	0	0
0	0	0	0	0	0	0	0	0	0	0	0	0	0	0	0
138	262	145	383	1305	433	520	1335	409	11274	218	42689	423	151	0	260
0	0	0	0	0	0	0	0	0	0	0	0	0	0	0	0
0	0	0	0	0	0	0	0	0	0	0	0	0	0	0	0
0	0	0	0	0	0	0	0	0	0	0	0	0	0	0	0
1121	59	51	0	122	33142	25	0	200	4239	708	0	0	11670	3275	1625
0	0	0	0	0	0	0	0	0	123	98	0	64	793	84	302
0	0	0	0	0	0	0	0	0	0	0	0	0	0	0	0
0	0	0	0	0	0	0	0	0	0	0	0	0	0	0	0
0	0	0	0	0	0	0	0	0	0	0	0	0	0	0	0

0	0	0	0	0	5904	0	0	0	38	93	35390	132	0	0	0
0	0	0	0	0	1375	0	0	0	0	0	0	0	0	0	0
0	0	0	0	0	1174	0	0	0	0	0	0	0	0	0	0
27	0	0	0	0	116	0	0	0	0	0	0	130	0	92	110
0	0	0	0	0	0	0	0	0	0	0	0	0	0	0	0
13456	66	13570	0	0	3569	0	0	0	0	0	0	0	0	95	53
0	0	0	0	0	0	0	0	0	0	0	0	0	0	0	0
0	0	0	0	0	0	0	0	0	0	0	0	0	0	0	0
0	0	0	0	0	0	0	0	0	0	0	0	0	0	0	0
0	0	60	0	0	0	0	1349	5163	141	173	61105	0	0	0	0
0	0	0	0	0	0	0	0	0	2339	0	0	0	0	0	0
0	0	0	0	0	0	0	0	0	0	0	0	0	0	0	0
0	0	0	0	0	0	0	0	0	0	0	0	0	0	0	0
0	0	0	0	0	0	0	0	0	0	0	0	0	0	0	0
0	0	0	0	0	0	0	0	0	0	0	0	0	0	0	0
0	0	0	0	0	0	0	0	0	0	0	0	0	0	0	0
0	0	0	0	0	0	0	0	0	0	0	0	0	0	0	0
0	0	0	0	0	1645	0	0	0	0	0	0	0	0	0	0
0	0	0	0	0	0	0	0	0	0	0	0	0	0	0	0
0	0	0	0	0	0	0	0	0	0	0	0	0	0	0	0
40	55437	90	0	317	779	82	480	4165	3706	27426	49	0	0	0	55
0	0	0	0	0	0	0	0	0	0	0	0	0	0	0	0
32	0	0	0	0	0	0	447	1682	0	0	0	0	328	0	0
232712	26140	210177	650	7154	10697	787	147626	7333	635	11803	635	1296	4094	17529	1447
0	0	1643	0	0	0	0	0	0	0	0	0	1541	0	0	0
0	0	0	0	0	0	0	0	0	0	0	0	168188	0	0	0
0	0	27662	0	0	0	0	0	0	0	0	0	179	0	0	57
0	0	0	0	0	0	0	0	0	0	41	0	0	0	0	0
0	0	0	0	0	0	0	0	0	0	0	0	0	0	0	0
0	0	0	0	0	0	0	0	0	0	0	0	0	0	0	0
0	0	0	0	0	0	0	0	0	0	0	0	0	0	0	0
0	0	0	0	0	0	35	0	0	0	0	0	0	748	0	446
0	0	0	0	0	0	0	425	0	0	0	63	0	0	0	0
0	0	0	0	0	0	0	0	0	0	0	0	0	0	0	0
0	0	0	0	0	0	0	435	46	0	0	59	0	0	0	0
0	0	0	0	0	0	0	0	0	0	0	0	0	0	0	0
0	0	0	0	0	0	0	0	0	0	0	0	0	0	0	0
0	0	0	0	0	0	0	0	0	0	186	0	0	0	0	153
0	0	0	0	0	0	0	0	0	0	0	0	0	0	0	0
0	394	0	0	0	0	0	0	0	0	331	0	0	0	0	0
0	0	0	0	0	0	0	0	0	0	0	0	0	0	0	0
0	0	0	0	0	0	0	0	0	0	0	0	0	632	0	0
0	0	0	0	0	0	0	0	0	0	0	0	0	0	0	0
0	0	0	0	0	0	0	0	0	43	0	0	0	0	0	0
0	0	0	0	0	0	0	0	0	0	0	0	0	0	0	0
0	0	0	0	0	0	0	0	0	0	0	0	0	0	0	0
0	0	0	0	0	0	0	0	0	0	0	0	0	0	0	0
0	0	0	0	0	0	0	0	0	0	0	0	0	0	0	0
183825	75475	220937	618	57065	14281	634	6359	375	1051	1219	1097	805	929	1876	932
0	0	0	0	0	0	0	0	8440	0	0	0	0	0	0	0
0	0	0	0	0	0	0	0	0	0	0	0	0	0	0	0
0	0	0	0	0	0	0	0	0	0	0	0	0	0	0	0
164	418	195	0	112	0	529	0	0	127	36	58	0	0	0	52
0	0	0	0	0	0	0	0	0	0	0	0	59	0	0	0
0	0	0	0	0	0	0	0	0	0	0	0	0	0	0	0
0	0	0	0	0	0	0	0	0	0	0	0	0	0	0	0
0	0	0	0	0	0	0	0	0	0	0	0	0	0	0	0
0	0	0	0	0	0	0	0	0	0	0	0	0	0	0	0

174	0	0	0	0	0	0	0	0	479	88	0	0	499	742	861
0	0	0	0	0	0	0	0	0	0	0	0	0	0	0	0
0	0	0	0	0	0	0	0	0	0	0	0	0	0	0	0
0	79	0	0	0	39	0	0	0	0	41	0	0	24821	2016	2097
0	0	0	0	0	0	0	0	0	0	0	0	0	156	83	0
0	0	0	0	0	0	0	0	0	0	0	0	0	0	0	0
0	0	0	0	0	0	0	0	0	0	0	0	135	0	0	0
0	0	0	0	0	0	0	0	0	0	0	0	0	0	0	0
0	0	0	0	0	0	0	0	0	0	0	0	0	0	0	0
0	0	0	0	412	0	0	472	39	42	33	63	0	453	0	0
0	0	0	0	0	0	0	0	0	0	0	0	0	0	0	0
0	0	0	0	0	0	0	0	0	0	0	0	0	0	0	0
0	0	56	59	199	0	0	0	0	51	0	0	303	0	0	0
0	0	48	0	0	0	0	0	0	0	0	0	0	756	165	601
0	0	0	54	536	61	101	0	0	0	0	0	73	0	82	210
0	0	0	0	0	0	0	0	0	0	0	0	0	149	0	50
0	0	0	0	0	0	0	0	0	0	0	0	119	319	0	0
0	0	0	0	0	0	0	0	0	0	0	0	61	0	0	0
0	0	0	0	0	0	0	0	0	0	0	0	0	0	0	0
0	0	0	0	0	0	0	0	0	0	0	0	0	0	0	0
0	0	0	0	0	0	0	0	0	0	0	0	0	0	0	0
0	0	0	0	0	0	0	0	0	0	0	0	0	0	0	53
128	183478	278	71	0	0	290	0	0	0	0	0	0	0	0	0
103	191	0	0	0	0	0	0	0	0	0	0	0	0	0	0
0	0	0	0	0	0	0	0	0	0	0	0	0	0	0	0
0	0	0	0	0	0	0	0	0	0	0	0	0	0	0	0
0	0	0	0	0	0	0	0	0	0	0	0	0	0	0	0
0	0	0	0	0	0	0	0	0	0	0	0	0	0	0	0
0	0	0	0	0	0	0	0	0	122	40	0	0	0	0	0
0	0	0	0	0	0	0	0	0	0	0	0	0	0	0	0
0	0	0	0	0	0	0	0	0	0	0	0	0	0	0	0
0	0	19601	0	428	73	0	473	102	1386	0	458	0	323	0	0
0	0	0	0	0	0	0	0	0	0	0	0	0	0	0	0
0	0	0	0	0	0	0	0	10592	0	0	60	0	0	0	0
0	0	0	0	0	0	0	0	0	0	38	0	0	0	0	0
139	13820	193	0	214	7553	185	947	862	68	94	64	71	952	87	33
38	0	0	0	0	0	0	0	0	0	0	0	0	0	0	0
33	0	0	118	0	35	120	0	61	46	259	173	0	838	222	500
537	197755	1049	67	300	27234	1170	913	12270	125	256	243	311	163	0	56
380	2652	229	0	216	282	178	0	0	0	72	98	0	0	0	101
0	0	0	155	236	27	30	0	0	0	0	0	0	0	0	0
0	0	0	0	0	0	0	0	0	0	42	54	0	0	0	0
0	0	0	0	0	0	0	0	0	0	0	0	0	0	0	0
0	0	0	0	0	0	0	0	0	0	0	0	0	0	0	0
0	0	0	0	0	0	0	0	0	0	0	0	0	0	0	0
0	0	0	0	0	0	0	0	0	0	0	0	0	0	0	0
0	0	0	0	109	37	0	0	0	0	0	0	0	0	0	0
0	0	0	0	0	0	0	0	0	0	0	0	0	0	0	0
0	0	0	0	0	0	0	0	0	0	0	0	0	0	0	0
0	0	0	0	0	0	0	0	0	0	0	0	0	0	0	0
0	0	0	0	0	0	0	0	0	0	0	0	0	0	0	0
0	0	0	0	0	0	0	0	19151	0	0	0	0	0	0	0
0	0	0	0	0	0	0	0	0	0	0	0	0	0	0	0
0	0	0	0	0	0	0	0	0	0	0	0	77	0	0	0
0	0	0	0	0	0	0	0	0	0	0	0	0	0	0	0
0	0	0	0	0	0	0	0	0	0	0	0	0	0	0	0
31	0	0	0	0	0	0	0	0	0	0	0	0	0	0	0
0	0	0	0	0	0	0	0	0	0	0	0	0	0	0	0

0	0	0	0	0	0	0	0	0	0	0	0	0	0	0	0
13338	74043	118437	497	358343	7296	626	6850	396730	21704	1595	326130	851	0	0	0
0	0	0	0	0	0	0	0	0	0	0	0	0	0	0	0
0	0	0	0	0	0	0	0	0	0	0	0	0	0	0	0
0	0	0	0	0	0	93	0	0	0	0	0	0	0	0	0
0	0	0	0	0	0	0	0	0	0	0	0	0	0	0	0
0	0	0	0	0	0	0	0	0	0	0	0	0	0	0	0
0	0	0	0	0	0	0	0	0	0	0	0	0	290	82	50
0	0	0	0	0	0	204	0	0	0	0	0	0	0	0	0
0	0	0	0	0	0	0	0	0	0	0	0	0	0	0	0
0	0	0	0	0	0	0	0	0	0	0	0	0	0	0	0
0	0	0	0	0	0	0	0	0	0	0	0	0	0	0	0
0	0	0	0	0	0	0	0	0	0	0	0	0	0	0	0
0	0	0	0	0	0	0	0	0	0	0	0	0	0	0	0
0	0	0	0	0	0	0	0	0	0	0	0	0	0	0	0
0	0	0	0	0	0	0	0	0	0	0	0	0	0	0	0
0	0	0	0	0	0	0	0	0	0	0	0	0	0	0	0
0	0	0	0	0	0	0	0	0	0	0	0	0	0	0	0
0	0	0	0	0	0	0	0	0	0	0	0	0	0	0	0
0	0	0	68	109	38	0	0	0	0	0	0	0	0	0	0
0	0	0	0	0	0	0	0	0	0	0	0	0	0	0	0
0	0	0	0	0	0	0	0	0	0	0	0	0	0	0	0
0	0	0	0	0	0	0	0	0	0	0	0	0	0	0	0
0	0	0	0	0	0	0	0	0	0	0	0	0	0	0	0
0	0	0	0	306	119	112	0	0	0	0	0	0	0	0	0
0	0	0	0	0	0	0	0	0	0	44	64	0	0	0	0
0	0	0	0	0	0	1545	0	0	0	0	0	0	0	0	0
0	0	0	0	0	0	0	0	0	0	76	52	0	0	0	0
0	0	0	0	0	0	0	2313	248	270	136	286	0	0	0	0
0	0	0	0	0	0	0	0	0	0	0	0	0	0	0	0
0	0	0	0	0	0	176	0	0	0	0	0	0	0	0	0
0	0	0	0	0	0	0	0	0	0	0	0	0	0	0	0
0	0	0	0	0	0	0	0	0	0	0	0	0	157	0	0
0	0	0	0	0	0	0	0	0	0	0	0	0	0	0	0
0	0	101	0	0	0	0	0	0	0	0	0	0	0	0	0
0	0	0	0	0	0	0	0	0	0	0	0	0	0	0	0
0	0	0	0	0	0	0	0	0	0	0	0	0	0	0	0
0	0	0	0	0	0	0	0	0	0	0	0	67	0	0	0
0	0	0	0	0	0	0	0	0	0	0	0	383	0	0	0
0	0	0	0	0	0	0	0	0	0	0	0	0	0	0	0
0	0	0	0	0	0	0	0	0	0	0	0	0	0	0	44
0	0	0	0	0	0	0	0	0	0	0	0	0	0	0	139
399	16006	673	650	1569	1876	971	0	21364	263	33781	0	124	0	5148	1746
0	0	0	0	0	0	0	0	0	0	0	0	0	0	0	0
0	0	0	0	0	0	0	0	0	82	31	68	0	0	0	0
0	0	0	0	0	0	0	0	0	0	0	0	0	0	0	0
0	0	0	0	0	0	0	0	0	0	0	0	0	0	0	0
0	0	0	0	0	0	0	0	0	0	0	0	0	0	0	0
0	0	0	0	0	0	0	0	0	0	0	0	0	0	0	0
0	0	0	0	0	0	0	0	0	0	0	0	0	310	90	43
0	0	0	0	0	0	0	0	0	0	0	0	0	0	0	0
0	0	0	0	0	0	0	0	0	0	0	0	0	0	0	0
0	0	0	0	0	0	0	0	0	0	0	0	0	0	0	0
0	0	0	0	0	0	0	0	0	0	0	0	0	338	327	192
0	0	0	0	0	0	41	0	0	0	0	0	0	310	226	151
0	73	0	0	0	0	0	0	0	0	0	0	0	0	0	0
0	0	0	0	0	0	0	0	0	0	0	0	0	0	0	52
0	0	0	0	0	0	0	0	0	0	0	0	0	0	0	0

0	0	0	0	0	0	0	0	0	0	0	0	0	1578	660	1091
0	0	0	0	0	0	0	0	0	0	0	0	0	0	0	0
0	0	0	0	0	0	0	0	0	0	0	0	0	0	0	0
0	65	62	0	0	0	175	0	0	0	0	0	0	0	0	0
0	0	0	0	0	0	0	0	0	0	0	0	0	0	0	0
0	0	0	0	0	0	0	0	0	0	0	0	0	0	0	0
260	0	50	60	0	47	0	446	49	11730	0	46	115	17618	2913	6748
0	0	0	0	0	0	0	470	0	46	0	0	0	138	0	145
0	0	0	0	0	0	0	0	46	51	0	63	0	0	82	0
0	0	0	0	0	0	0	0	0	0	0	0	0	0	0	0
0	0	0	0	0	0	0	0	0	2547	0	0	0	0	0	0
0	0	0	0	0	0	0	0	0	0	0	0	0	0	0	0
0	0	0	0	0	0	0	0	0	0	0	0	238	0	0	0
0	0	0	0	0	0	0	0	0	0	0	0	0	0	0	0
14553	7085	2117	122060	224111	26687	21430	10509	21053	38595	44716	43566	32785	26656	42306	30119
0	0	0	0	0	0	0	0	0	0	0	0	0	0	0	0
0	0	0	0	0	0	112	446	0	0	35	65	0	328	0	0
0	0	0	0	0	0	0	0	0	0	0	0	0	0	0	0
0	0	0	0	0	0	0	0	0	0	0	0	0	0	0	0
115	133	204	0	0	0	0	0	0	0	0	0	0	623	0	0
0	0	0	0	0	0	0	0	0	0	0	0	0	0	0	0
1796	69	1581	0	0	272	0	463	0	0	0	0	0	2004	0	0
0	0	0	0	0	0	0	0	0	0	0	0	0	0	0	0
0	0	0	0	0	0	0	0	0	0	0	0	115	0	0	0
0	0	0	0	0	0	0	0	0	0	0	0	0	0	0	0
0	61	0	0	0	34	0	0	52	0	423	0	0	0	0	140
0	0	0	0	0	0	0	0	0	0	0	0	0	0	0	0
0	0	0	0	0	0	0	0	0	0	0	0	0	149	84	0
3972	0	0	0	0	0	0	0	284	129	0	0	267	595	0	266
0	0	0	0	0	0	0	0	0	0	0	0	0	0	0	0
0	0	0	0	0	0	0	0	0	0	0	0	0	0	0	0
0	0	0	0	0	0	0	0	0	0	0	0	0	0	0	0
0	0	0	0	0	0	0	0	0	0	0	0	0	0	0	0
0	0	0	0	207	47	0	451	58	85	0	299	0	0	0	0
0	64	0	0	0	0	0	0	0	0	0	0	0	0	0	0
0	0	0	0	0	0	0	0	0	0	0	0	0	0	0	0
0	0	0	0	0	0	39	463	47	0	0	0	0	0	0	0
0	0	0	0	0	0	0	0	0	0	0	0	0	0	0	0
0	0	0	0	0	0	0	0	0	0	0	0	0	0	0	0
0	0	0	0	0	0	0	0	0	0	0	0	65	0	0	0
0	0	0	0	0	0	0	0	0	0	0	0	0	0	0	0
0	0	0	0	0	0	0	0	1260	0	0	0	0	0	0	0
0	64	0	0	0	0	0	0	0	0	0	0	0	0	0	0
0	0	0	0	0	0	0	0	0	0	0	0	0	0	0	0
920	0	0	0	0	1167	0	0	0	40	91	0	266	979	498	933
0	0	0	0	0	0	0	0	0	0	0	0	0	0	0	0
0	0	0	0	0	0	0	0	0	0	0	0	0	0	0	0
0	0	0	0	0	0	0	0	0	0	0	0	0	0	0	0
0	0	0	0	0	0	0	0	0	0	0	0	0	0	0	0
0	0	0	0	0	0	0	0	0	0	0	0	0	0	0	56
0	0	0	0	0	0	0	0	44	0	0	0	0	0	0	0
0	0	0	0	0	0	0	0	0	0	0	0	0	0	0	0
0	0	0	0	0	0	0	0	0	0	0	0	0	0	0	0
0	0	0	0	0	0	0	0	0	0	0	0	0	0	0	0
65	60	49	0	102	2785	42	0	0	0	0	0	0	0	75	167
0	0	0	0	0	0	0	0	0	0	0	0	0	326	0	0
0	0	0	0	0	0	39	0	0	0	0	0	0	0	0	0
101	145	93	0	0	0	0	0	0	0	0	0	0	0	0	0

0	0	0	0	0	0	0	0	0	0	0	0	0	0	0	0
0	0	0	0	0	0	0	0	0	0	0	0	0	0	80	103
0	0	0	0	0	0	0	0	0	0	0	0	0	0	0	0
0	0	0	0	0	0	0	0	0	0	0	0	0	0	0	0
0	0	0	0	0	0	852	0	0	0	0	0	0	480	570	376
0	0	0	0	0	0	0	0	0	0	0	0	0	0	0	0
0	0	0	0	0	0	0	0	0	0	0	0	0	0	0	0
0	0	0	0	0	0	0	0	0	0	0	0	0	0	0	0
0	0	0	0	0	0	0	0	0	0	0	0	0	0	0	0
0	0	0	0	0	0	0	0	0	0	0	0	0	0	0	0
0	0	0	0	0	0	0	0	3669	0	0	0	0	0	0	0
0	0	0	0	0	0	0	0	0	0	0	0	0	0	0	0
0	0	0	0	0	0	35	0	0	87	0	0	0	0	0	0
0	0	0	0	0	0	0	0	0	0	0	0	0	0	0	0
0	0	0	0	0	0	0	0	0	0	0	0	0	0	0	0
0	0	0	0	0	0	0	0	0	0	0	0	0	0	0	0
0	0	0	0	0	0	0	0	0	0	0	0	0	0	0	0
0	0	0	0	0	0	0	0	0	0	0	0	0	0	0	0
0	0	0	0	0	0	0	0	0	0	0	10115	0	0	0	0
0	0	0	0	0	0	0	0	0	0	0	0	0	0	0	0
0	0	0	0	0	0	0	0	0	0	0	0	0	0	0	0
0	0	0	0	0	0	0	0	0	0	0	0	0	0	0	0
0	0	0	0	0	0	0	0	0	0	0	0	0	0	0	0
142	111	94	0	0	0	0	0	0	0	0	99	0	0	0	0
0	59	68	0	93	0	0	0	0	0	0	0	0	0	0	0
4532	82	0	0	0	0	0	0	0	0	0	0	0	0	0	0
0	0	0	0	0	0	0	0	0	0	0	0	0	0	0	0
0	0	0	0	0	0	0	0	0	0	0	0	0	0	0	0
0	0	0	0	0	0	0	0	0	0	0	0	0	0	0	0
0	0	0	0	0	0	0	440	35	0	0	0	0	0	0	0
0	0	0	0	0	0	0	0	0	0	0	0	0	0	0	0
0	0	0	0	314	103	209	0	2874	0	31	67	0	0	0	0
0	0	0	0	0	0	0	0	0	0	0	0	0	0	0	0
0	0	0	0	0	0	0	0	0	0	0	0	6353	0	0	0
0	0	0	0	0	0	0	0	0	0	0	0	0	0	0	0
37	69	45	75	4809	896	72	0	1149	13557	0	15342	0	0	0	0
33	0	43	0	0	0	0	0	0	0	0	0	0	0	0	0
343	989	57941	92908	738	237	522	3183	141	2649	101246	184749	8134	329	332	465
5153	0	54	0	0	0	0	0	90	0	42	24145	0	0	0	0
0	0	0	0	0	0	0	0	0	0	0	0	0	0	0	0
0	0	0	0	0	0	0	0	0	0	0	0	0	0	0	0
0	0	0	0	0	0	0	0	0	0	0	0	0	0	0	0
0	0	0	0	0	0	0	0	0	0	0	0	0	0	0	0
0	0	0	0	0	0	0	0	0	0	0	0	0	0	0	0
0	0	0	0	0	0	0	0	0	0	0	0	0	0	0	0
66	339	0	0	119	0	10558	464	78	221020	233	46	0	0	0	54
0	0	0	0	0	0	0	0	0	0	0	0	0	0	0	0
0	0	0	0	0	0	0	0	0	0	0	0	0	809	0	238
0	0	0	0	0	0	0	0	0	0	0	0	0	0	0	0
0	0	0	0	0	0	0	0	0	0	0	0	78	0	0	0
0	0	0	0	0	0	0	0	0	44	0	0	258	0	0	0
0	0	0	0	0	0	0	0	0	0	0	0	0	0	0	0
0	0	0	0	0	0	0	0	0	0	0	0	0	0	0	0
0	0	0	0	0	41	0	0	0	0	0	0	0	0	0	45
0	0	0	0	0	0	0	0	0	0	0	0	0	0	0	0
0	0	0	0	109	0	45	0	0	0	41	0	0	0	0	0
250	0	149	0	0	35	0	0	0	0	0	0	0	162	0	0
0	0	0	0	94	0	0	0	0	0	0	0	0	0	0	0

0	0	0	0	0	0	0	0	0	0	0	0	0	0	0	0
0	0	0	0	0	0	0	0	0	0	0	0	0	0	0	0
0	0	0	0	0	0	0	0	0	0	0	0	0	0	0	0
0	0	0	0	0	0	0	0	0	0	0	0	0	0	0	0
0	0	0	0	0	0	0	0	0	0	0	0	0	0	0	0
0	0	0	0	0	0	0	0	0	0	0	0	0	0	0	0
0	0	0	0	0	0	0	0	0	0	0	0	63	0	0	0
0	0	0	0	0	0	0	0	0	0	0	0	0	0	0	0
0	0	0	0	0	0	0	0	0	0	0	0	0	0	0	0
0	0	0	0	0	0	0	0	0	0	0	0	0	0	0	0
0	0	0	0	0	0	0	0	0	8232	27	0	0	148	0	0
0	0	0	0	0	0	0	0	0	0	0	0	0	0	0	0
99	341	115	0	0	0	0	0	0	0	0	0	0	323	74	0
0	0	0	0	0	0	0	0	32543	0	116	57	0	0	0	0
28	127	0	0	0	0	0	0	0	0	0	59	0	1207	514	550
0	0	0	0	0	0	0	0	0	0	0	0	0	0	0	0
0	0	0	0	0	0	0	0	0	0	0	0	0	0	0	0
0	0	0	0	0	0	0	0	0	0	0	0	0	0	0	0
0	0	0	0	0	0	0	0	0	0	0	0	0	0	0	0
0	0	0	0	0	0	0	0	0	0	0	0	0	0	0	0
0	0	0	4566	0	2789	0	0	236	39	112	1136	0	0	0	0
0	0	0	0	0	0	0	0	0	28	45	0	60	481	552	40
0	0	0	0	0	0	0	0	0	0	0	0	0	0	0	0
0	0	0	0	109	40	0	0	0	0	0	0	0	0	0	0
0	0	0	0	0	0	0	0	0	0	0	0	0	337	163	198
0	0	0	0	0	0	0	0	0	0	0	0	0	0	0	0
2518	0	0	3512	212	0	34	0	326	6660	2351	0	558	4641	7323	3657
0	0	0	0	0	0	234	0	0	0	0	0	0	0	0	0
0	0	0	0	0	0	0	0	0	0	0	0	0	0	0	0
0	0	0	0	0	0	0	0	0	0	0	0	0	0	0	0
0	0	0	0	0	0	0	0	0	0	0	0	0	0	0	0
130	0	125	0	106	0	0	0	0	0	0	0	0	0	0	122
0	0	0	0	0	0	0	0	0	0	0	0	0	0	0	0
0	0	0	0	0	0	0	0	0	0	0	0	0	0	0	0
0	0	0	0	0	0	0	0	0	0	0	0	0	0	0	169
0	0	0	0	0	0	0	0	0	0	0	0	0	0	0	0
0	0	0	0	0	0	0	0	0	0	0	0	0	0	0	0
0	0	0	0	0	0	0	0	0	0	0	0	0	0	0	0
0	0	0	0	0	0	0	0	0	0	0	0	0	0	0	0
0	0	0	0	0	0	0	0	0	0	0	0	0	0	0	0
0	0	0	0	0	0	0	0	0	0	0	0	0	0	0	0
0	0	0	0	0	0	0	0	0	0	0	0	0	0	0	0
0	0	0	0	0	0	0	0	0	0	0	0	0	0	0	0
0	0	0	0	0	0	0	0	0	0	0	0	0	0	0	0
0	0	0	0	0	0	0	0	0	0	0	0	0	0	0	0
0	0	0	0	0	0	0	466	1989	0	0	0	69	0	0	0
0	0	0	0	0	0	0	2278	227	136	166	172	0	0	0	0
0	0	0	0	0	0	0	0	0	270	0	0	0	0	0	0
0	0	0	0	0	0	0	0	0	0	0	0	0	0	0	0
0	0	0	0	0	0	0	0	0	0	0	0	0	0	0	0
0	0	0	0	0	0	0	0	0	0	0	0	0	0	0	0
0	0	0	0	0	0	0	0	0	0	0	0	0	0	0	0
0	0	0	0	0	0	0	0	0	0	0	0	0	0	0	0
33	62	179	0	0	0	39	0	0	0	0	0	0	0	0	0
0	0	0	68	0	70	0	0	0	0	0	0	0	0	0	0
0	0	0	0	0	0	0	0	0	0	0	0	0	0	0	0
0	0	0	0	0	0	85	0	0	0	0	0	0	0	0	0
186	0	0	0	0	0	64	0	0	1689	1733	0	73	4952	2297	5427

0	0	0	0	0	0	0	0	0	0	0	0	0	0	0	0
0	0	0	0	0	0	0	477	0	0	0	0	0	0	0	0
0	0	0	0	0	0	0	0	0	0	0	0	0	0	0	0
0	0	0	0	0	0	0	0	0	0	0	0	0	0	0	0
0	0	0	0	0	5788	0	0	0	0	0	0	0	0	0	0
0	0	0	0	0	0	0	0	0	0	0	0	0	0	0	0
0	0	0	0	0	0	0	0	0	0	0	0	0	0	0	0
0	0	0	0	0	0	0	0	0	0	0	0	0	0	0	0
0	0	0	0	0	0	0	0	0	0	0	0	0	0	0	0
0	0	0	0	0	0	0	438	13892	0	0	76	0	0	0	0
0	0	0	0	0	0	0	0	0	0	0	0	64	0	0	0
0	0	0	0	0	0	0	0	0	0	0	0	0	0	0	0
331	262	361	188	1281	312	153	0	50	0	0	0	0	0	0	0
0	0	0	0	0	0	0	0	0	0	0	0	0	0	0	0
0	0	0	0	0	0	0	0	0	0	0	0	0	0	0	0
0	0	0	0	0	84	46	0	0	0	0	0	0	0	0	0
0	0	0	0	94	0	0	0	0	0	0	0	0	0	0	0
0	0	0	56	0	43	0	0	0	0	0	0	0	276	79	445
0	0	0	0	0	0	0	0	0	0	0	0	0	0	0	0
0	0	0	0	0	0	0	0	0	0	0	0	0	0	0	0
0	0	0	0	0	0	0	0	0	0	0	0	60	0	0	0
0	0	0	0	0	0	0	0	0	0	0	0	0	0	0	0
0	0	0	0	0	0	0	0	0	0	0	0	0	0	0	96
0	0	0	0	0	0	0	0	0	0	0	0	0	0	0	0
0	0	0	46	103	154	27	0	0	0	0	0	0	0	0	0
0	0	0	0	0	0	149	0	0	0	0	0	0	0	0	0
0	0	0	0	0	0	0	0	0	0	0	0	0	0	0	0
0	0	0	0	0	0	0	0	60	41	47	42548	64	0	0	0
0	0	0	0	0	0	0	0	141	39093	286	62968	191	763	1194	677
0	0	0	0	0	0	0	0	0	0	0	0	0	0	0	0
0	0	0	0	0	0	0	0	0	0	0	0	0	0	0	0
0	0	0	0	0	0	0	0	0	0	0	0	0	0	0	0
0	0	0	0	0	40	0	0	0	0	0	0	0	0	0	0
0	0	0	0	0	0	0	0	0	0	0	0	0	0	0	0
0	0	0	0	0	0	0	0	0	0	0	0	62	0	0	0
0	0	0	0	0	0	0	0	6780	0	0	0	0	0	0	0
0	0	0	0	0	0	0	0	0	0	0	0	0	0	0	0
0	0	0	0	0	0	0	0	0	0	0	0	0	0	0	0
0	0	0	0	0	0	0	0	0	0	0	0	0	0	0	0
0	0	0	0	0	0	0	0	0	0	0	0	0	0	0	0
0	0	0	0	0	0	0	0	0	0	0	0	0	0	0	0
0	0	0	0	0	0	0	0	0	0	0	0	0	0	0	0
0	0	0	0	0	0	0	0	0	0	0	0	0	0	0	0
0	0	0	0	0	0	0	0	0	0	0	0	0	0	0	0
0	0	0	55	299	123	0	1313	98	0	60	0	0	0	0	0
0	0	0	0	0	0	0	0	0	0	0	0	0	0	0	0
0	0	0	0	0	0	0	0	0	0	0	0	0	0	0	0
0	0	0	0	0	0	0	460	93	42	38	63	0	0	89	0
0	0	0	0	0	0	0	0	0	0	0	0	0	0	0	0
0	0	0	0	0	0	0	0	0	0	0	0	0	0	0	0
0	0	0	0	0	0	0	0	0	0	0	0	0	0	0	0
0	0	0	0	0	0	0	0	0	0	0	0	0	0	0	0
5058	197	0	0	0	64	2899	0	2034	98	54	0	847	60795	292	3894
0	0	0	0	0	0	148	0	0	0	43	0	0	0	0	0
0	0	0	0	0	0	0	0	0	0	0	0	0	0	0	0
0	0	0	0	0	0	0	0	0	0	0	0	0	0	0	0
1508	0	5418	0	0	174	35	884	0	128	79	140	0	0	0	0
0	0	0	0	0	0	0	0	0	0	0	0	0	0	0	0

0	0	0	0	0	33	0	0	0	0	0	0	0	163	0	0
0	0	0	0	0	0	0	0	0	0	0	0	0	146	0	198
0	0	0	0	0	0	0	0	0	0	0	0	0	0	0	0
0	0	0	0	0	0	0	0	0	0	0	0	0	0	168	113
0	0	0	0	0	0	0	0	0	0	0	0	0	0	0	0
0	0	0	0	0	0	0	0	0	0	0	0	0	137	0	186
0	0	0	0	0	9227	0	0	0	0	0	0	0	0	0	0
0	0	0	0	0	0	0	0	0	0	0	0	0	0	0	0
0	0	0	0	0	0	0	0	0	0	0	0	0	0	0	0
0	0	0	0	0	0	0	0	0	0	0	0	0	0	0	0
0	0	0	0	0	0	0	0	0	0	0	0	0	0	0	0
0	0	0	0	0	0	0	0	0	0	0	0	0	0	0	0
153	252	61	7081	304	169	165	463	52408	0	34	177	58	0	12337	61
31	0	0	0	0	0	0	0	0	0	0	0	0	0	0	0
64	0	0	0	0	6993	39	0	0	0	84	66	539	0	0	0
0	0	0	0	0	0	0	0	0	0	0	0	0	0	0	0
0	0	0	0	0	0	0	0	0	0	0	0	1329	0	0	0
0	0	0	0	0	0	0	0	0	0	0	0	0	0	0	0
0	0	0	0	0	0	0	0	0	0	0	0	0	0	0	0
0	0	0	0	0	0	0	0	0	0	0	0	0	0	0	0
0	0	0	0	0	0	0	0	0	0	0	0	0	0	0	0
28	71	0	0	0	0	0	0	0	47	0	0	0	0	0	0
0	0	0	0	0	0	0	0	0	0	0	0	0	0	0	0
0	0	0	0	0	0	0	0	0	0	0	0	0	0	0	0
52	0	0	0	0	0	0	0	0	0	0	0	0	0	0	0
181	153	222	0	0	0	0	0	0	0	0	0	0	0	0	0
0	0	0	0	0	0	0	0	0	0	0	0	0	0	0	0
0	0	0	0	0	0	0	0	0	0	0	0	0	0	0	0
0	0	0	0	0	0	0	0	0	0	0	0	0	0	0	0
24011	409	50	121	1178	389	152	1359	10155	81368	3115	184	756	96000	93533	131920
0	0	0	0	0	0	0	0	0	0	0	0	0	0	0	0
0	0	0	0	0	0	0	0	0	0	0	0	0	0	0	0
0	0	0	0	0	0	0	0	0	0	0	0	0	0	0	0
0	0	0	0	0	0	0	0	0	0	0	0	0	0	0	0
0	0	0	0	0	0	0	0	0	0	0	0	0	0	0	0
0	0	0	0	0	0	0	0	0	0	0	0	0	0	0	0
0	0	0	0	0	0	0	0	0	0	0	0	0	0	0	0
0	0	0	0	0	0	0	0	0	0	0	0	0	0	0	0
0	0	0	0	0	0	0	0	0	0	0	0	0	0	0	0
0	0	0	0	0	0	0	0	0	0	0	0	0	0	0	0
126	183	40	0	0	0	0	0	0	0	0	0	0	0	0	0
0	0	0	0	0	0	0	0	0	0	0	0	0	0	78	0
0	0	0	0	0	0	0	0	0	0	0	0	0	306	101	55
61	109536	0	0	0	0	0	0	0	0	0	0	0	0	0	0
0	0	0	0	0	0	0	0	0	0	0	0	0	0	0	0
0	0	0	0	0	0	0	0	0	0	0	0	0	0	0	0
0	0	0	0	0	0	0	0	0	0	0	0	0	0	0	0
0	0	0	0	0	0	0	0	0	0	0	0	0	0	0	0
0	0	0	0	0	0	0	0	0	0	0	0	0	0	0	0
0	0	0	0	0	0	0	0	0	0	0	0	0	0	0	0
0	0	0	58	0	78	46	0	0	0	35	0	0	0	0	0
0	0	0	0	0	0	337	0	0	0	0	0	0	0	0	0
0	0	0	0	0	0	0	0	0	0	0	0	0	0	0	0
0	0	0	0	0	0	0	0	0	0	0	0	7943	0	0	0
0	0	0	116	565	201	22	0	0	0	0	0	0	0	0	0
0	0	0	0	0	0	0	0	0	0	0	0	0	0	0	0
0	0	0	0	0	0	0	0	0	0	0	0	0	0	0	0
0	0	0	0	0	0	0	444	0	0	42	110	0	0	0	0

0	0	0	0	669	46	41	0	0	51	35	54	0	0	0	0
0	0	0	0	0	0	0	0	0	0	0	0	0	0	0	0
0	0	0	0	0	0	0	0	0	0	0	0	0	0	0	0
0	0	0	0	91	29	0	0	0	40	81	7559	167	1086	173	159
0	0	0	0	0	0	0	0	0	0	0	0	0	0	0	0
26	0	0	0	0	0	0	0	0	0	0	0	0	0	0	0
0	0	0	0	0	48	0	0	0	0	0	0	0	157	0	229
0	0	0	0	0	0	0	0	0	0	0	0	0	0	0	0
0	0	0	0	0	0	0	0	0	0	0	0	0	0	0	0
0	0	0	0	0	0	0	0	0	0	0	0	0	0	0	0
0	0	0	0	0	0	0	0	0	0	0	0	0	0	0	964
0	0	0	0	0	38	35	0	0	0	0	0	0	0	0	0
0	0	0	0	0	2604	0	0	0	574	0	57	0	0	0	0
0	0	0	0	0	0	0	0	0	0	0	0	0	0	0	0
0	0	0	0	0	0	0	0	0	0	0	0	0	0	0	0
0	0	0	0	0	0	0	0	0	0	0	0	0	0	0	0
0	0	0	0	0	0	0	0	0	0	0	0	0	0	0	0
35	0	163	0	0	0	41	0	0	0	0	0	284	0	0	0
0	0	0	0	0	0	0	0	0	0	0	0	0	0	0	0
0	0	0	0	0	0	0	0	0	0	0	0	0	0	0	0
32	205939	110	0	0	0	0	0	0	0	0	0	0	0	0	0
0	0	0	0	0	0	0	0	0	0	0	0	0	0	0	0
0	0	0	0	0	0	0	0	46	0	0	0	0	157	0	0
0	0	0	0	0	0	0	0	0	0	0	0	0	0	0	0
0	0	0	0	0	0	0	0	0	0	0	0	0	0	0	0
0	0	0	0	0	0	0	0	0	0	0	0	0	0	0	0
0	0	0	0	0	0	0	0	0	0	0	0	0	0	0	0
0	0	0	0	0	0	0	0	0	0	0	0	0	0	0	0
0	0	0	0	0	0	0	0	0	0	0	0	0	0	0	0
0	0	0	0	0	0	0	0	0	0	0	0	0	0	0	0
0	0	0	0	0	0	0	0	0	2336	0	0	0	0	0	0
0	0	0	0	0	0	0	0	0	0	0	0	0	0	0	0
0	0	0	0	0	0	0	0	0	0	0	0	0	0	0	0
0	0	0	0	0	0	0	0	0	0	0	0	0	0	0	0
0	0	0	0	0	0	0	0	0	0	0	0	0	0	0	0
0	0	0	0	0	0	0	1852	88	87	256	0	0	0	0	0
0	0	0	0	0	0	0	0	0	0	0	0	0	0	0	0
0	0	0	0	0	0	0	0	0	0	0	0	0	0	0	0
0	0	50	952	2022	532	938070	0	0	0	0	0	0	0	0	0
0	0	0	0	0	0	0	0	0	0	0	0	0	0	0	0
0	0	0	0	0	0	0	0	0	0	0	0	0	0	0	0
30	0	0	0	0	0	0	0	0	0	0	0	0	0	0	0
0	0	0	0	0	0	0	0	0	0	0	0	0	0	0	57
0	0	0	0	0	0	0	0	0	0	0	0	0	0	0	0
0	0	0	0	0	0	0	0	0	0	0	0	0	0	0	0
0	0	0	0	0	0	0	0	0	0	0	0	0	0	0	0
6926	0	0	0	0	0	40	0	0	0	0	0	0	0	0	0
0	0	0	0	0	0	0	0	0	0	0	0	0	0	0	0
0	0	0	0	0	0	0	0	0	0	0	0	0	0	0	0
0	0	0	0	0	0	0	0	0	0	0	0	0	0	0	0
0	0	0	0	0	0	0	0	0	0	0	0	0	0	0	0
0	0	0	0	0	0	0	0	0	0	0	0	0	0	0	0
0	0	0	0	0	0	71	0	0	0	0	0	0	0	0	0
0	0	0	0	0	0	0	0	0	0	0	0	62	0	0	0
0	0	0	0	0	0	0	0	0	0	0	0	0	0	0	0
0	0	0	0	0	0	0	0	0	0	0	0	0	0	0	0
179	62	215	0	0	0	0	0	0	0	0	0	0	0	0	0
47	121	172	0	0	0	0	0	0	0	0	0	0	0	0	0

0	0	0	55	183	33	0	0	0	0	0	0	0	0	0	0
0	0	0	0	0	0	0	0	0	0	0	0	0	0	0	0
0	0	0	0	0	0	0	0	0	0	0	0	0	0	0	0
0	0	0	0	0	0	0	1378	90	255	81	129185	0	0	0	0
0	0	0	0	0	0	0	0	0	0	0	0	0	0	0	0
58	137	44	0	0	0	0	0	0	3407	0	0	0	0	0	0
0	0	0	0	0	0	0	0	0	0	0	0	0	0	0	0
0	0	0	0	0	0	0	0	0	0	0	0	58	442	0	0
0	0	0	0	0	0	0	0	0	0	0	0	0	0	0	0
0	0	0	0	0	0	0	0	0	0	0	0	0	0	0	0
0	0	0	0	0	0	0	0	0	0	0	0	0	0	0	0
0	0	0	0	0	0	0	0	0	0	0	0	0	0	0	0
0	0	0	0	0	0	0	0	0	0	0	0	0	0	0	0
0	0	0	0	0	0	0	0	0	0	0	0	0	0	0	0
0	0	0	0	0	0	0	0	0	0	0	0	0	0	0	0
0	0	0	0	0	0	0	0	0	0	0	0	0	0	0	0
0	0	0	0	0	0	0	0	0	0	0	0	0	0	0	0
0	0	0	0	0	0	0	0	0	0	0	0	0	0	0	0
0	0	0	0	0	0	0	0	0	0	0	0	0	0	0	0
0	0	0	0	0	0	0	0	219	0	0	0	0	0	10672	100
174133	24092	313160	724860	7699	814084	2622	782372	7928	5840	10268	5811	701547	72715	5576	12464
0	0	0	0	0	0	0	0	0	92	0	0	0	0	0	0
0	0	0	0	0	0	0	0	0	0	0	0	0	149	0	61
0	0	0	0	0	199	0	0	613	0	0	0	66	155	41891	98
0	0	0	0	0	0	0	0	0	0	0	0	0	0	0	0
0	0	0	0	0	0	0	0	0	0	0	0	0	0	0	0
0	0	0	109	0	0	0	952	822	0	33	0	2520	0	229	0
0	0	0	0	0	0	0	0	0	0	0	0	0	0	0	0
0	58	0	0	0	0	0	0	0	0	0	0	0	0	0	0
0	0	0	0	0	0	0	0	0	0	0	0	0	0	0	0
0	0	0	0	0	0	0	0	0	0	0	0	0	0	0	0
0	0	0	0	0	0	0	0	0	0	0	0	0	0	0	0
0	0	0	0	0	0	0	0	0	0	0	0	0	0	0	0
684	0	0	0	0	0	0	0	161	1914	486	0	66	1361	1978	1800

COVID-19-008	COVID-19-010	COVID-19-011	COVID-19-013	COVID-19-014	COVID-19-015	COVID-19-016	COVID-19-017	COVID-19-018	COVID-19-019	COVID-19-023	COVID-19-024	COVID-19-025	COVID-19-026	COVID-19-027	COVID-19-028
940799	933906	937607	917513	916893	822817	913432	794731	929186	914919	980079	792391	898988	986222	949154	990722
17993	10972	21568	19039	11480	132100	10359	91365	9117	27210	9265	201949	94909	4227	28206	7879
0	0	0	0	0	0	0	0	215	0	0	0	0	0	0	0
39265	37888	32195	39425	43337	19273	35003	30383	42908	32770	9843	5274	5509	9437	20224	1016
663	610	5146	3885	618	12351	40466	47321	1563	3845	605	280	379	114	1168	299
0	0	0	0	0	0	0	0	0	0	0	0	0	0	0	0
1280	16624	3484	20138	27672	13459	740	36200	17011	21256	208	106	215	0	1248	84

CC008	CC010	CC011	CC013	CC014	CC015	CC016	CC017	CC018	CC019	CC023	CC024	CC025	CC026	CC027	CC028
100	118	0	646	0	185	278	104	199	615	0	52	0	148	0	87
0	0	0	0	0	0	0	0	0	0	0	0	0	0	0	0
592263	796690	678440	773001	764351	559416	726586	708194	790645	633865	76569	43682	156764	37076	368136	11149
0	0	0	0	0	0	0	0	0	0	0	0	0	0	0	0
0	0	0	0	0	0	0	0	0	0	0	0	0	0	0	0
0	0	0	0	0	0	0	0	0	0	0	0	0	0	0	0
0	0	0	0	0	0	46	58	0	0	0	0	0	0	0	0
0	0	0	0	0	0	0	0	0	0	0	0	0	0	0	0
0	0	0	0	0	0	0	0	0	0	0	0	0	0	0	0
92	0	12232	75	0	103	88	349	0	230	3228	2345	8381	116	0	3420
195	0	0	0	0	0	0	0	0	0	0	0	0	0	0	0
0	0	0	0	0	0	0	0	0	0	0	0	0	0	0	0
0	0	0	0	0	0	0	0	0	0	0	0	4205	0	0	0
1230	2447	2775	3336	2335	2672	2612	2753	3359	2794	0	179	1176	384	1622	206
464	357	305	298	590	0	193	46	185	584	206	0	203	0	414	0
0	0	0	0	0	0	0	0	0	0	0	0	0	0	0	0
0	0	0	0	0	0	0	0	0	0	0	0	0	0	0	0
0	0	0	0	0	0	0	0	0	0	0	0	3499	346	1550	0
0	0	0	0	0	0	0	0	0	0	0	0	0	0	0	0
0	0	0	0	0	0	0	0	0	0	0	0	0	0	0	0
0	0	0	0	0	3609	163	94	286	0	0	0	0	0	0	0
0	0	0	0	0	0	0	0	0	0	0	0	0	0	0	0
0	118	536	0	0	0	0	0	0	70	2894	107	0	989	1720	983
0	0	0	0	0	0	0	0	0	0	0	0	0	0	0	0
0	0	0	0	0	2535	0	0	0	0	0	0	0	0	0	0
7436	9514	11133	8105	18091	119367	3145	23728	12479	8038	64585	21561	8309	5562	34141	20853
0	0	0	0	0	0	0	0	0	0	0	0	0	0	0	0
0	0	0	0	0	60	6538	33	139	0	0	46	0	1817	0	0
0	0	0	0	0	0	0	0	0	0	0	0	0	0	2491	0
0	0	0	0	0	0	0	0	0	65	3683	0	0	0	0	0
0	0	0	0	0	0	0	0	0	0	0	0	0	0	0	0
625	0	212	139	0	51	2317	0	130	0	0	0	0	0	0	0
0	0	0	0	0	0	0	0	0	0	0	0	0	0	0	0
0	0	0	0	0	0	0	0	0	0	0	0	0	0	0	0
0	0	0	0	0	0	0	0	0	0	0	0	200	0	0	0
0	0	0	0	0	0	0	0	0	0	0	0	0	0	0	0
0	0	0	0	0	0	0	0	0	0	0	0	0	0	0	0
0	0	0	237	0	0	0	0	0	0	0	1860	0	0	0	941
0	0	0	0	0	0	0	0	0	0	0	0	0	0	0	0
937	573	4244	520	2893	0	95	587	407	934	91186	996	2057	2955	22976	19151
0	0	0	0	0	0	0	0	0	0	0	0	0	0	0	0
0	0	0	0	0	0	0	0	0	0	610	0	210	0	0	0
0	0	0	0	0	0	0	0	0	0	0	0	0	0	0	0
0	0	0	0	0	0	0	0	54	0	0	0	0	0	0	0
0	0	0	0	0	53	0	0	0	0	0	0	0	0	0	0
0	0	0	0	0	0	0	0	0	0	0	0	0	0	0	0
451	877	118	1083	807	3058	2837	356	1456	680	0	0	0	0	320	0

0	0	0	0	0	0	0	0	0	0	0	0	0	0	0	0
0	0	0	0	0	0	0	0	0	0	0	0	0	0	0	0
1752	3042	7370	1598	6480	1808	135	139	79	2553	1447	30061	258414	5773	16465	415
0	0	0	0	0	0	0	0	0	0	0	0	0	0	0	0
0	0	0	0	0	0	0	0	0	0	0	0	0	0	0	0
0	0	0	0	0	0	0	0	0	0	0	0	0	0	0	0
0	0	0	0	0	0	0	0	0	0	0	0	596	0	0	0
0	0	0	0	0	0	0	0	0	0	0	0	0	0	0	0
0	0	0	0	0	0	0	0	0	0	0	0	0	0	2209	0
0	0	0	0	0	0	0	0	0	0	0	0	0	0	0	0
0	0	0	0	0	0	0	0	0	0	0	0	0	0	0	0
0	0	0	0	0	0	1329	0	0	0	0	0	0	0	0	0
0	0	0	0	0	0	0	0	0	0	592	0	0	0	0	0
0	0	0	0	0	0	0	0	0	0	0	0	0	0	0	0
0	0	0	0	0	0	0	0	0	0	0	0	0	0	0	0
94	0	207	67	0	0	0	0	0	0	521801	320690	13269	859830	223585	44494
0	0	0	0	0	0	0	0	0	0	0	0	0	0	0	0
0	0	0	0	0	0	0	0	0	0	0	0	0	0	0	0
0	0	0	0	0	0	0	0	0	0	0	0	0	0	0	0
797	130	3803	1522	1172	10632	149	239	345	297	5478	27555	81815	1611	15643	7483
0	0	0	0	0	0	0	0	0	0	0	0	0	0	0	0
0	0	0	0	0	0	0	0	0	0	0	1136	0	0	0	449
0	0	0	0	0	0	0	0	0	0	0	0	0	0	0	0
0	0	0	0	0	0	0	0	0	0	0	0	0	0	0	0
0	0	1602	0	0	0	0	0	0	0	0	0	0	0	0	0
0	0	0	69	0	0	0	0	0	0	0	0	0	0	0	0
0	0	0	0	0	0	0	0	0	0	0	0	0	0	0	0
0	0	0	0	0	182	0	0	0	0	0	0	0	0	0	0
0	0	0	0	0	0	0	0	0	0	0	0	0	0	88	0
0	0	0	0	0	0	0	0	0	0	0	0	0	0	0	0
0	0	0	0	0	0	0	0	0	0	0	0	0	0	0	0
0	0	0	0	0	0	0	0	0	0	0	0	0	0	0	0
0	0	0	0	0	0	0	0	0	0	0	0	0	0	0	0
0	0	0	0	0	0	0	0	0	52	0	0	216	0	0	0
0	0	0	0	0	0	0	0	0	0	0	0	0	0	0	0
0	0	0	0	0	0	0	0	0	0	0	0	0	0	0	0
193	0	0	0	296	0	0	0	0	417	377	0	77288	1302	0	0
0	0	0	0	0	0	0	0	0	0	0	0	0	0	0	0
0	0	0	0	0	0	0	0	0	0	0	0	0	0	0	0
0	0	0	0	0	0	0	0	0	0	201	0	0	0	0	0
0	0	0	0	0	0	0	0	0	0	0	0	0	0	0	0
0	0	0	0	0	0	0	0	0	0	0	0	0	0	0	0
0	0	0	0	0	0	0	0	0	0	0	0	0	0	0	0
0	0	18586	0	307	0	0	0	0	62	0	0	3838	0	0	0
0	0	0	0	0	0	0	0	0	0	0	0	0	0	0	0
0	0	0	0	0	0	0	0	0	0	0	0	0	0	0	0
0	0	0	0	0	0	0	0	0	0	219	0	0	0	0	93
0	0	0	0	0	0	0	0	0	0	0	0	0	0	0	0
0	0	0	0	0	0	0	0	0	0	0	0	0	0	0	0
0	0	0	0	0	0	0	0	0	0	0	0	0	0	0	0
0	0	0	0	0	0	0	0	0	0	0	99	0	0	0	0
0	0	1874	0	0	0	0	0	0	0	0	0	0	0	0	0
0	130	0	0	0	0	0	0	0	0	0	0	0	0	0	0
0	0	0	0	0	0	0	0	0	0	0	0	0	0	0	0
166	221	0	0	0	0	0	0	55	0	4099	57	21541	0	0	0
663	610	5146	3885	618	12351	40466	47321	1563	3845	605	280	379	0	1168	299
0	0	0	0	0	0	0	0	0	0	0	0	0	0	0	0
0	0	0	0	0	0	0	0	0	0	0	0	0	0	0	0

0	0	0	0	0	0	0	0	0	0	0	0	0	0	0	0
0	0	0	0	0	0	0	0	0	0	0	0	0	0	0	0
0	0	0	0	0	0	0	0	0	0	0	0	812	0	0	0
0	0	409	0	0	0	0	0	115	138	234	0	0	0	0	0
0	0	0	0	0	0	0	0	0	0	0	0	0	0	0	0
0	0	0	74	0	0	0	45	0	0	0	385	0	135	76	0
0	0	0	0	0	243	0	0	0	0	0	0	0	0	0	0
174	124	110	1219	0	0	34	146	398	305	0	0	0	0	219	182
0	0	0	0	0	0	0	0	0	0	0	0	0	0	0	0
839	0	0	582	2665	0	0	0	0	0	0	0	3288	218	0	369
0	0	0	0	0	0	0	0	0	0	0	0	0	0	0	0
0	0	0	0	0	0	0	0	0	0	0	0	0	0	0	0
0	0	0	0	0	0	0	0	0	0	0	0	0	0	0	0
0	0	0	0	0	0	0	0	0	0	0	0	0	0	0	0
0	0	0	0	0	0	1115	0	0	0	0	0	0	0	0	0
3547	3804	4658	5788	3888	1437	28965	783	4676	4370	1854	510	589	274	4787	434
1347	2623	1543	1817	882	354	2287	485	1783	862	0	69	206	0	839	0
89	0	0	0	0	0	135	0	0	0	0	22706	187	257	5514	264
0	0	0	0	0	0	0	0	0	0	0	0	0	0	0	0
0	0	0	0	0	0	0	0	0	0	0	0	0	0	444	0
0	0	0	0	0	0	0	0	0	0	0	0	0	0	0	0
0	0	0	0	0	0	0	0	0	0	0	0	0	0	0	0
39091	37764	32085	38206	43337	19273	34969	30237	42510	32465	9843	5274	5509	9437	20005	834
0	0	1554	231	0	0	0	0	0	63	4804	42163	1395	10662	0	40713
0	0	881	193	0	238	0	42	59	54	1483	0	200	0	145	790
0	0	0	0	0	0	0	0	0	0	0	0	0	0	0	0
0	206	0	0	0	0	154	175	210	0	0	0	0	0	3601	0
0	0	0	0	0	0	0	0	0	55	0	0	0	0	0	0
0	0	0	0	0	0	509	0	0	0	0	0	0	0	0	0
0	0	334	68	0	60	0	0	0	0	19206	174284	6109	33178	30796	203536
0	0	201	74	0	0	221	0	59	251	199	103	227	256	1132	77
0	0	0	0	0	0	0	0	0	0	0	0	0	0	0	0
0	0	0	0	0	0	0	0	0	0	0	0	0	114	0	0
0	0	0	0	0	0	0	0	0	0	0	0	0	131	0	0
0	0	0	0	0	0	0	0	0	0	0	0	0	0	0	0
0	0	0	0	0	0	0	0	0	0	0	0	0	0	0	0
0	0	0	0	0	0	0	0	0	0	0	0	0	0	0	0
0	0	0	0	0	0	0	0	0	0	0	0	0	0	0	0
0	0	0	70	0	0	0	0	0	0	1061	248	2079	359	7629	191
0	0	0	0	0	0	0	0	0	0	0	0	0	262	0	0
0	0	0	0	0	0	0	0	0	0	0	0	0	0	0	0
13013	2683	1448	1553	3874	130	1479	434	990	4119	205	0	2658	0	659	76
0	0	0	0	0	0	0	0	0	0	0	0	0	0	0	0
0	0	0	0	0	0	0	0	0	0	0	0	0	0	0	0
0	0	0	0	0	0	0	0	0	0	818	45	0	112	0	255
0	0	0	0	0	0	0	0	0	0	0	0	0	0	0	0
0	0	0	0	0	0	0	0	0	0	0	0	0	0	0	0
0	0	0	0	0	0	0	0	0	0	0	0	0	0	0	0
0	0	0	0	0	0	91	123	0	0	0	0	2039	0	0	0
0	0	0	0	0	0	0	0	0	0	0	0	0	0	0	0
0	0	0	0	0	0	0	0	0	0	0	0	0	0	0	0
196	117	0	132	302	110	2645	186	127	320	633	63593	1421	1500	21811	4159
0	0	0	0	0	0	0	0	0	0	0	0	0	0	0	0
0	0	0	0	0	0	0	0	0	0	0	0	0	0	0	0
0	0	0	0	285	0	0	0	0	0	0	0	0	0	0	0
0	0	0	0	0	0	0	0	0	0	216	12384	0	123	0	79
87	0	0	0	0	0	0	0	0	0	0	0	0	0	0	0
0	0	0	0	0	0	0	0	0	0	0	0	0	0	0	0

480	364	0	557	582	0	95	234	336	0	0	0	0	0	0	0
0	0	0	0	0	0	0	0	0	0	0	0	0	0	0	0
0	0	0	0	0	0	0	0	0	0	0	0	0	0	0	0
0	0	0	0	0	0	0	0	0	0	0	0	0	0	0	0
0	0	0	0	0	71	0	0	0	0	0	0	0	0	0	0
0	0	0	0	0	0	0	0	0	0	0	0	0	0	0	0
0	0	0	0	0	0	0	0	0	0	1357	0	0	0	0	0
0	0	0	0	0	0	0	0	0	0	0	0	0	0	0	0
0	0	0	0	0	0	0	0	0	0	0	0	0	0	0	0
0	0	0	0	0	0	0	0	0	0	0	0	0	0	0	0
0	0	0	0	0	45	0	0	0	0	0	0	0	0	0	0
0	0	0	0	0	0	0	0	0	0	0	0	0	0	0	0
0	0	0	0	0	0	0	0	0	0	0	0	0	0	0	0
0	0	0	0	0	0	0	0	0	0	0	4255	2058	127	69	3410
0	0	0	0	0	0	0	0	0	0	0	0	0	0	221	0
0	0	0	0	0	0	0	0	0	0	0	0	0	0	0	0
0	0	0	0	0	0	0	0	0	0	0	0	0	0	0	0
0	0	0	0	0	0	0	0	0	0	0	0	0	0	0	0
0	0	0	0	0	0	0	0	0	0	0	0	0	0	0	0
0	0	0	78	0	0	0	0	0	0	0	0	0	0	0	0
628	0	5698	74	906	373	972	1940	629	104	3120	151859	183	1139	4921	534
0	0	0	0	0	0	0	0	0	0	0	0	0	0	0	0
0	0	0	0	0	0	0	0	0	0	0	0	0	0	0	0
96	0	0	0	0	0	0	0	0	0	0	0	428	364	0	0
0	0	0	0	0	0	0	0	0	0	0	0	0	0	0	0
0	0	116	0	0	0	0	0	0	64	0	64	7317	718	0	99
0	0	0	0	0	0	0	0	0	0	0	0	0	0	0	0
187	378	110	143	0	0	808	0	132	208	0	182	0	128	73	0
0	0	0	0	0	0	0	0	0	0	0	0	0	0	0	0
0	0	0	0	0	0	0	0	0	0	0	0	0	0	0	0
0	0	0	0	0	0	0	0	0	0	1047	0	0	0	0	0
0	0	0	0	0	0	0	0	0	0	389	0	618	575	4002	3364
0	0	663	0	0	0	0	0	0	0	0	0	2031	0	0	0
0	0	0	0	0	0	68	91	133	0	0	0	0	0	0	270
0	0	0	0	0	0	0	0	0	0	594	0	220	0	81	529
0	0	0	0	0	0	0	0	0	0	0	0	0	0	0	0
0	0	0	0	0	0	0	0	0	0	0	0	0	0	0	0
0	0	0	0	0	0	0	0	0	0	0	0	0	0	0	0
0	0	0	0	0	0	0	0	0	0	0	0	0	0	0	0
0	0	0	0	0	0	0	0	0	0	0	0	0	0	3329	0
0	0	0	0	0	0	0	0	0	0	0	0	0	0	0	0
0	0	0	0	0	0	0	0	0	0	0	0	0	0	0	0
0	0	0	0	0	0	0	0	0	0	0	0	0	0	0	0
0	0	0	0	0	0	0	0	0	0	0	0	0	0	0	0
0	0	0	0	0	0	0	0	0	0	0	0	1268	0	0	0
0	0	0	0	0	0	0	0	0	0	0	0	0	0	0	0
88	0	0	456	577	59	153	97	357	0	0	13217	608	0	0	92
0	0	0	184	574	66	0	0	0	0	0	0	1400	0	0	86
0	0	0	0	0	0	0	0	0	0	0	0	0	0	0	0
0	0	0	0	0	0	0	0	0	0	0	0	0	0	0	0
0	0	2075	0	0	0	0	0	0	0	0	0	0	0	0	0
0	0	0	0	0	0	0	0	0	0	0	0	0	0	0	0
0	0	0	0	0	0	0	0	0	0	1664	4393	0	0	0	79
80	356	0	572	288	0	97	347	362	59	0	96	0	0	0	0
79	0	0	575	287	0	0	0	0	0	0	0	218	0	0	0
0	0	0	0	0	0	0	0	0	0	0	0	0	0	0	0
5595	7216	5961	7539	4421	2028	6888	1967	8722	5301	841	231	1237	501	3986	89
0	0	0	0	0	0	0	0	0	0	0	0	0	0	0	0

0	0	0	0	0	0	0	0	0	0	0	0	0	0	0	0
0	0	0	0	0	0	0	0	0	0	0	0	0	0	0	0
0	0	0	0	0	0	0	0	0	0	0	0	0	0	0	0
0	0	112	0	0	0	0	0	0	0	604	0	0	0	92	83
0	0	0	0	0	0	0	0	0	0	0	0	0	0	0	0
0	0	0	0	0	0	0	0	0	0	0	0	0	0	0	0
0	0	0	0	0	0	0	0	0	0	0	0	0	0	0	0
0	0	656	0	0	181	0	0	0	74	200	3727	400	131	0	431
0	0	0	0	0	0	0	0	0	0	0	0	0	0	0	5359
0	0	0	0	0	0	0	0	0	0	0	0	0	0	0	0
5872	2610	1392	1605	2322	604	4301	531	2923	2210	0	104	805	0	2869	79
0	0	0	0	0	0	0	0	0	0	0	0	0	0	0	0
0	0	320	0	0	0	0	0	0	0	0	0	401	0	0	1702
0	0	0	0	0	0	0	0	0	0	0	0	0	0	0	0
0	0	0	0	0	0	0	0	0	0	0	0	0	0	0	0
0	0	0	0	0	0	0	0	0	0	0	0	0	0	0	0
0	0	0	0	0	0	0	0	0	0	0	0	0	0	0	0
0	0	0	0	0	0	27	0	0	0	0	0	0	0	72	0
0	0	0	0	0	0	0	0	62	259	0	0	0	0	0	0
0	0	0	0	0	0	0	0	0	0	0	0	0	0	0	0
0	0	0	0	0	0	0	0	0	0	0	0	0	0	0	0
90	0	0	639	0	0	0	0	132	0	0	0	0	0	0	0
0	0	0	0	0	0	0	0	0	0	0	0	0	0	0	0
0	0	0	0	0	0	0	0	0	0	0	0	0	0	0	0
117	0	285	0	0	0	0	0	0	0	5132	2952	23248	223	9984	362
0	0	0	0	0	0	806	0	0	0	0	0	0	0	0	0
0	0	0	0	0	0	2590	48	0	0	0	0	0	0	0	0
637	0	0	219	890	0	0	0	0	0	3085	2049	1892	0	0	0
0	0	0	0	0	0	0	0	0	0	0	0	0	0	79	0
0	0	0	0	0	0	0	0	215	0	0	0	0	0	0	0
0	0	0	0	0	0	0	0	0	0	0	0	0	0	0	0
0	0	0	0	0	0	2282	0	0	0	0	0	0	0	0	0
0	0	0	0	0	0	0	0	0	0	0	0	0	0	0	0
0	0	0	0	0	0	0	0	0	0	0	0	0	0	0	0
0	0	0	0	0	0	0	0	0	0	0	0	0	0	0	0
0	0	0	0	0	0	0	0	0	0	213	56	0	0	0	0
0	0	0	0	0	0	0	0	0	0	0	0	0	0	0	0
0	0	0	0	0	0	0	0	0	0	0	0	0	0	0	0
0	0	0	0	0	0	0	0	0	0	0	0	0	0	0	0
0	0	0	0	0	0	0	0	0	0	0	0	0	0	0	0
0	0	0	0	0	0	0	0	0	0	0	0	0	0	0	0
0	0	0	0	0	0	0	0	0	0	0	0	0	349	0	0
0	0	0	0	0	0	0	0	0	0	0	0	0	0	0	0
0	0	0	0	0	0	0	0	0	0	0	0	0	0	0	0
0	0	0	0	0	0	0	0	0	0	0	0	0	0	0	0
0	0	0	0	0	0	0	0	0	0	0	0	0	0	0	0
0	0	0	0	0	0	0	0	0	0	0	0	0	0	0	0
0	0	0	0	0	0	0	0	0	0	0	0	0	0	0	0
1225	1720	20919	7706	2358	3865	399	1686	2161	2787	9075	1629	1180	3121	18208	1066
0	0	0	0	0	0	0	0	0	0	0	0	0	0	0	0
0	0	0	0	0	0	497	256	249	0	0	0	0	0	0	0
0	0	0	0	0	0	0	0	0	0	0	0	0	0	0	0
0	0	0	80	0	0	0	0	0	0	847	0	0	0	427	455
0	0	0	0	0	0	0	0	0	0	0	0	0	0	0	0
0	0	0	0	0	0	0	0	0	0	0	0	0	0	0	0
0	0	0	0	296	0	0	0	0	0	0	0	199	0	0	0
0	0	636	0	0	0	0	0	0	55	0	0	824	0	630	0
0	0	0	0	0	0	0	0	0	0	0	0	0	0	0	0

0	0	0	0	0	0	0	0	0	0	0	0	0	0	0	0
0	0	0	0	0	0	0	0	0	0	0	0	0	0	0	0
0	0	0	0	0	0	0	0	0	0	0	0	0	0	0	0
0	0	0	0	0	0	0	0	0	0	0	0	0	0	0	0
937	4656	8394	14066	3466	130739	1125	88402	5284	19908	0	251	398	123	802	259
0	0	0	0	0	0	0	0	0	0	0	0	0	0	0	0
85	0	0	0	579	255	366	537	529	0	116404	1231	8166	3600	44377	599862
0	0	0	0	0	0	0	0	0	0	0	0	0	0	0	0
0	0	0	0	0	0	0	0	0	0	0	0	0	0	0	0
0	0	0	0	0	1344	0	0	0	0	0	450	0	0	0	641
0	0	0	0	0	112	1044	0	0	0	185	0	0	0	0	0
0	0	0	0	0	0	0	0	0	0	0	0	0	0	0	0
0	0	0	0	0	0	0	0	0	0	0	0	0	0	0	0
0	0	0	0	0	0	0	0	0	0	201	0	0	0	0	0
0	0	0	0	0	0	0	0	0	0	0	0	0	0	0	0
0	0	0	0	0	0	0	0	0	0	0	0	0	0	0	0
31898	88074	60309	71561	84743	21289	103943	24718	86413	52299	4976	1502	7771	1212	35863	962
0	0	1914	0	0	0	30	0	61	0	0	0	0	0	0	0
0	0	0	0	0	0	0	0	0	242	0	0	0	0	0	0
0	0	0	0	0	0	0	0	0	0	0	0	0	0	0	0
0	0	0	0	0	0	0	0	0	0	0	0	0	0	0	0
0	0	0	0	0	0	0	0	0	0	0	0	0	0	0	0
0	0	0	0	0	0	0	0	0	0	0	0	0	0	0	0
0	0	0	0	0	0	0	0	0	0	10772	6935	2260	7095	6952	2472
188	0	0	0	584	0	0	0	0	0	0	0	199	0	0	0
0	0	0	0	0	0	0	0	0	0	0	0	0	0	0	0
0	0	0	0	0	0	0	0	0	119	172	52	0	117	76	0
0	0	0	0	0	0	0	0	0	0	0	0	0	0	0	0
0	0	0	0	0	0	0	0	0	0	0	0	0	0	0	0
0	118	120	0	0	0	0	0	0	63	0	0	0	0	0	0
0	0	0	0	0	0	0	0	0	0	0	0	0	0	0	0
0	0	0	0	0	0	0	0	0	0	0	0	0	0	0	0
0	0	0	0	0	0	0	0	0	0	0	0	0	0	0	0
0	0	0	0	0	0	66	0	54	0	0	18440	0	112	0	0
0	0	0	0	0	0	0	0	0	0	0	0	0	0	0	0
209	263	0	298	0	1115	1225	30	399	257	1812	2526	383	1157	3541	3729
80	384	830	68	0	0	0	0	0	1125	0	0	0	0	0	0
0	0	0	0	0	0	0	0	0	0	0	0	0	0	0	0
0	0	0	0	0	0	0	0	0	0	0	0	0	0	0	0
0	0	0	0	0	0	0	0	0	0	0	0	0	0	0	0
0	0	0	0	0	0	0	0	0	0	0	0	0	0	0	0
0	0	0	0	0	0	0	0	0	0	0	0	2486	0	2448	0
0	0	0	0	0	0	0	0	0	0	0	0	0	0	0	0
0	0	0	0	0	0	0	0	0	0	0	0	0	0	0	0
0	0	0	0	0	0	0	0	0	0	0	0	0	0	136	0
0	0	0	0	0	0	0	0	0	0	397	0	0	0	382	340
0	0	0	0	0	0	0	0	0	0	0	0	0	0	0	0
0	0	0	0	0	0	23	46	0	0	0	4184	0	0	0	0
0	0	0	0	0	0	0	0	0	0	0	0	0	0	0	0
0	0	0	0	0	0	0	0	0	0	0	0	0	0	0	0
0	0	241	0	0	0	0	0	0	0	2333	1101	0	619	0	4833
0	0	0	0	0	0	0	51	79	0	0	0	0	0	0	0
0	0	0	0	0	0	0	0	0	0	0	0	0	0	0	0
0	0	0	0	0	0	0	0	0	0	0	0	0	230	0	0
0	0	0	0	0	0	166	121	72	0	0	0	0	0	0	0
0	0	0	0	0	0	0	0	0	0	0	0	0	0	0	0
0	0	0	0	0	0	0	0	0	0	198	0	0	0	0	0
0	0	0	0	0	0	0	0	0	0	0	0	0	0	0	0

0	0	0	0	0	0	137	53	0	64	0	0	0	0	0	0
0	0	0	0	0	0	0	0	0	0	0	0	0	0	0	0
0	0	0	0	0	0	0	0	0	0	0	0	0	0	0	0
0	0	0	0	0	0	0	0	0	0	0	0	0	0	0	0
0	0	0	0	0	0	0	0	0	0	0	0	0	0	0	0
0	0	0	0	0	0	0	0	0	0	0	0	0	0	0	0
0	0	0	0	0	0	0	0	0	0	0	0	0	0	0	0
0	219	217	0	0	0	0	0	0	364	423	1404	208	0	87	0
0	0	0	0	0	0	0	0	0	0	0	0	0	0	2175	0
0	0	0	0	0	0	0	0	0	0	0	0	0	0	0	0
0	0	0	0	0	0	0	0	0	0	0	0	0	0	0	0
0	0	0	0	0	0	0	0	0	0	0	0	0	0	0	0
0	0	0	0	0	0	0	0	0	0	0	0	0	0	0	0
0	0	0	0	0	0	74	0	0	0	0	0	0	0	0	0
0	0	0	0	0	0	0	0	0	0	0	0	0	0	0	0
0	0	0	0	0	0	0	0	0	0	0	0	2219	0	0	0
91	0	0	0	298	0	0	0	0	0	0	0	0	0	0	0
0	0	1548	0	0	0	0	190	0	0	0	0	11567	0	0	0
0	0	0	0	0	0	0	0	0	0	0	0	0	0	0	0
0	0	0	0	0	0	0	0	0	0	0	0	0	0	0	0
0	0	0	0	0	0	0	0	0	0	0	0	0	0	0	0
0	0	0	0	0	0	0	0	0	0	0	0	0	0	0	0
0	0	0	0	0	0	27	0	0	0	0	0	0	0	0	0
0	0	0	558	0	0	0	0	0	0	0	0	0	0	0	0
0	0	7533	0	0	0	0	0	0	0	0	0	5871	0	0	0
284217	14870	84545	28151	15388	84518	11428	24345	11263	194964	15415	4534	241979	3432	62685	6747
0	0	0	0	0	0	0	0	0	0	0	0	0	0	0	0
0	0	0	72	596	0	0	0	0	0	0	0	225	0	0	0
1280	16624	3484	20138	27672	13459	740	36200	17011	21256	208	106	215	0	1248	84
0	0	0	0	0	0	0	0	0	0	0	0	0	0	0	0
0	0	0	0	0	0	0	0	0	0	0	0	0	0	0	0
0	0	116	73	0	1950	42	1453	114	115	0	0	439	0	0	0
0	0	0	0	0	0	0	0	0	0	0	0	0	0	0	0

COVID-19-008	COVID-19-010	COVID-19-011	COVID-19-013	COVID-19-014	COVID-19-015	COVID-19-016	COVID-19-017	COVID-19-018	COVID-19-019	COVID-19-023	COVID-19-024	COVID-19-025	COVID-19-026	COVID-19-027	COVID-19-028
0	0	0	0	0	0	0	0	0	0	0	52	0	148	0	0
0	0	0	0	0	0	0	0	0	0	0	0	0	0	0	0
0	0	0	0	0	0	0	0	0	0	0	0	0	0	0	0
0	0	0	0	0	0	0	49	0	0	0	0	0	0	0	0
100	118	0	646	0	185	278	55	199	615	0	0	0	0	0	87
0	0	0	0	0	0	0	0	0	0	0	0	0	0	0	0
0	0	0	0	0	0	0	0	0	0	0	0	0	0	0	0
592263	796690	678440	773001	764351	559416	726586	708194	790645	633865	76569	43682	156764	37076	368136	11149
0	0	0	0	0	0	0	0	0	0	0	0	0	0	0	0
0	0	0	0	0	0	0	0	0	0	0	0	0	0	0	0
0	0	0	0	0	0	0	0	0	0	0	0	0	0	0	0
0	0	0	0	0	0	46	58	0	0	0	0	0	0	0	0
0	0	0	0	0	0	0	0	0	0	0	0	0	0	0	0
0	0	0	0	0	0	0	0	0	0	0	0	0	0	0	0
92	0	12232	75	0	103	88	349	0	230	3228	2345	8381	116	0	3420
195	0	0	0	0	0	0	0	0	0	0	0	0	0	0	0
0	0	0	0	0	0	0	0	0	0	0	0	0	0	0	0
0	0	0	0	0	0	0	0	0	0	0	0	4205	0	0	0
1230	2447	2775	3336	2335	2672	2612	2753	3359	2794	0	179	1176	384	1622	206
464	357	305	298	590	0	193	46	185	584	206	0	203	0	414	0
0	0	0	0	0	0	0	0	0	0	0	0	0	0	0	0
0	0	0	0	0	0	0	0	0	0	0	0	0	0	0	0
0	0	0	0	0	0	0	0	0	0	0	0	406	0	1550	0

0	0	0	0	0	0	0	0	0	0	0	0	3093	346	0	0
0	0	0	0	0	0	0	0	0	0	0	0	0	0	0	0
0	0	0	0	0	0	0	0	0	0	0	0	0	0	0	0
0	0	0	0	0	3609	163	94	286	0	0	0	0	0	0	0
0	0	0	0	0	0	0	0	0	0	0	0	0	0	0	0
0	118	536	0	0	0	0	0	0	70	2894	107	0	989	1720	983
0	0	0	0	0	0	0	0	0	0	0	0	0	0	0	0
0	0	0	0	0	2535	0	0	0	0	0	0	0	0	0	0
0	0	0	0	0	0	0	0	0	0	0	0	0	0	0	0
0	0	1576	0	0	0	37	89	71	70	0	10721	0	1469	0	1588
0	0	0	0	0	0	0	0	0	0	0	0	0	0	0	0
0	0	0	0	0	0	0	0	0	0	0	0	0	0	0	0
0	0	0	0	0	0	0	0	0	0	0	259	0	0	0	0
0	0	0	0	0	0	0	0	0	0	0	0	0	0	0	0
0	0	0	0	0	0	0	0	0	0	0	0	0	0	0	0
0	0	0	0	0	0	0	0	0	0	405	4808	0	481	0	867
0	0	0	0	0	0	0	0	0	0	0	0	0	0	0	0
0	0	0	0	0	0	0	0	0	0	0	0	0	0	0	0
0	0	0	0	0	0	0	0	0	0	0	0	0	134	0	0
0	0	0	0	0	0	0	0	0	0	0	0	0	0	0	0
0	0	111	81	276	0	56	147	0	0	573	2794	655	118	1211	624
0	0	0	0	0	0	0	0	0	0	0	0	0	0	0	0
0	116	205	0	0	1186	160	0	0	128	425	62	1224	0	236	94
7349	9398	9122	8024	17815	118181	2802	23361	12341	7779	60870	1285	5618	3360	21368	17498
0	0	0	0	0	0	0	0	0	0	590	0	0	0	569	95
0	0	119	0	0	0	0	0	0	0	0	0	0	0	0	0
87	0	0	0	0	0	40	41	0	61	1722	0	0	0	10757	87
0	0	0	0	0	0	50	90	67	0	0	1632	812	0	0	0
0	0	0	0	0	0	0	0	0	0	0	0	0	0	0	0
0	0	0	0	0	0	0	0	0	0	0	0	0	0	0	0
0	0	0	0	0	0	0	0	0	0	0	0	0	0	0	0
0	0	0	0	0	60	6538	33	139	0	0	46	0	1817	0	0
0	0	0	0	0	0	0	0	0	0	0	0	0	0	0	0
0	0	0	0	0	0	0	0	0	0	0	0	0	0	2491	0
0	0	0	0	0	0	0	0	0	0	0	0	0	0	0	0
0	0	0	0	0	0	0	0	0	65	3683	0	0	0	0	0
0	0	0	0	0	0	0	0	0	0	0	0	0	0	0	0
625	0	212	139	0	51	2317	0	130	0	0	0	0	0	0	0
0	0	0	0	0	0	0	0	0	0	0	0	0	0	0	0
0	0	0	0	0	0	0	0	0	0	0	0	0	0	0	0
0	0	0	0	0	0	0	0	0	0	0	0	0	0	0	0
0	0	0	0	0	0	0	0	0	0	0	0	200	0	0	0
0	0	0	0	0	0	0	0	0	0	0	0	0	0	0	0
0	0	0	0	0	0	0	0	0	0	0	0	0	0	0	0
0	0	0	0	0	0	0	0	0	0	0	1860	0	0	0	941
0	0	0	237	0	0	0	0	0	0	0	0	0	0	0	0
0	0	0	0	0	0	0	0	0	0	0	0	0	0	0	0
0	0	0	0	0	0	0	0	0	0	0	0	0	0	0	0
937	573	4244	520	2893	0	95	587	407	934	91186	996	2057	2955	22976	19151
0	0	0	0	0	0	0	0	0	0	0	0	0	0	0	0
0	0	0	0	0	0	0	0	0	0	0	0	0	0	0	0
0	0	0	0	0	0	0	0	0	0	0	0	0	0	0	0
0	0	0	0	0	0	0	0	0	0	610	0	210	0	0	0
0	0	0	0	0	0	0	0	0	0	0	0	0	0	0	0
0	0	0	0	0	0	0	0	0	0	0	0	0	0	0	0
0	0	0	0	0	0	0	0	54	0	0	0	0	0	0	0
0	0	0	0	0	53	0	0	0	0	0	0	0	0	0	0
0	0	0	0	0	0	0	0	0	0	0	0	0	0	0	0

451	877	118	1083	807	3058	2837	356	1456	680	0	0	0	0	320	0
0	0	0	0	0	0	0	0	0	0	0	0	0	0	0	0
0	0	0	0	0	0	0	0	0	0	0	0	0	0	0	0
1480	568	0	843	4728	1754	66	0	0	0	394	0	3902	119	1787	164
91	0	0	71	0	0	0	0	0	0	0	65	35369	3015	0	0
0	0	0	0	0	0	0	0	0	0	0	0	0	0	0	0
0	0	0	0	0	0	0	0	0	0	0	0	0	0	0	0
0	0	0	0	0	0	0	0	0	0	0	0	0	0	0	0
0	0	0	0	0	0	0	0	0	0	0	0	0	0	0	0
0	0	0	78	0	0	69	0	79	0	0	0	8504	0	14524	0
0	0	0	0	0	0	0	0	0	0	0	0	0	0	0	0
0	0	0	0	0	0	0	0	0	0	0	0	0	0	0	0
0	0	0	0	0	0	0	0	0	0	207	0	0	0	0	0
181	2369	5653	537	861	54	0	139	0	2213	648	26339	200485	116	0	251
0	0	0	0	598	0	0	0	0	0	198	0	380	2389	154	0
0	105	842	0	0	0	0	0	0	340	0	1524	7749	134	0	0
0	0	875	69	293	0	0	0	0	0	0	2133	2025	0	0	0
0	0	0	0	0	0	0	0	0	0	0	0	0	0	0	0
0	0	0	0	0	0	0	0	0	0	0	0	0	0	0	0
0	0	0	0	0	0	0	0	0	0	0	0	0	0	0	0
0	0	0	0	0	0	0	0	0	0	0	0	0	0	0	0
0	0	0	0	0	0	0	0	0	0	0	0	596	0	0	0
0	0	0	0	0	0	0	0	0	0	0	0	0	0	0	0
0	0	0	0	0	0	0	0	0	0	0	0	0	0	0	0
0	0	0	0	0	0	0	0	0	0	0	0	0	0	2209	0
0	0	0	0	0	0	0	0	0	0	0	0	0	0	0	0
0	0	0	0	0	0	0	0	0	0	0	0	0	0	0	0
0	0	0	0	0	0	1329	0	0	0	0	0	0	0	0	0
0	0	0	0	0	0	0	0	0	0	592	0	0	0	0	0
0	0	0	0	0	0	0	0	0	0	0	0	0	0	0	0
0	0	0	0	0	0	0	0	0	0	0	0	0	0	0	0
94	0	207	67	0	0	0	0	0	0	521801	320690	13269	859830	223585	44494
0	0	0	0	0	0	0	0	0	0	0	0	0	0	0	0
0	0	0	0	0	0	0	0	0	0	0	0	0	0	0	0
0	0	0	0	0	0	0	0	0	0	0	0	0	0	0	0
0	0	1940	338	0	3328	38	51	0	0	1252	1331	19486	121	5920	377
0	0	0	0	0	0	0	0	0	0	0	0	0	0	0	0
619	130	0	820	898	6670	0	0	0	297	1588	6726	4099	124	6502	2675
0	0	210	297	274	389	0	156	129	0	1395	19152	40319	0	2905	3535
94	0	917	67	0	182	111	32	216	0	1026	346	15689	1366	316	0
84	0	736	0	0	63	0	0	0	0	217	0	2222	0	0	896
0	0	0	0	0	0	0	0	0	0	0	0	0	0	0	0
0	0	0	0	0	0	0	0	0	0	0	1136	0	0	0	90
0	0	0	0	0	0	0	0	0	0	0	0	0	0	0	359
0	0	0	0	0	0	0	0	0	0	0	0	0	0	0	0
0	0	0	0	0	0	0	0	0	0	0	0	0	0	0	0
0	0	1602	0	0	0	0	0	0	0	0	0	0	0	0	0
0	0	0	0	0	0	0	0	0	0	0	0	0	0	0	0
0	0	0	69	0	0	0	0	0	0	0	0	0	0	0	0
0	0	0	0	0	0	0	0	0	0	0	0	0	0	0	0
0	0	0	0	0	182	0	0	0	0	0	0	0	0	0	0
0	0	0	0	0	0	0	0	0	0	0	0	0	0	88	0
0	0	0	0	0	0	0	0	0	0	0	0	0	0	0	0
0	0	0	0	0	0	0	0	0	0	0	0	0	0	0	0
0	0	0	0	0	0	0	0	0	0	0	0	0	0	0	0
0	0	0	0	0	0	0	0	0	0	0	0	0	0	0	0
0	0	0	0	0	0	0	0	0	0	0	0	0	0	0	0
0	0	0	0	0	0	0	0	0	0	0	0	0	0	0	0

0	0	0	0	0	0	0	0	0	0	0	0	0	0	0	0
0	0	0	0	0	0	0	0	0	52	0	0	0	0	0	0
0	0	0	0	0	0	0	0	0	0	0	0	0	0	0	0
0	0	0	0	0	0	0	0	0	0	0	0	216	0	0	0
0	0	0	0	0	0	0	0	0	0	0	0	0	0	0	0
0	0	0	0	0	0	0	0	0	0	0	0	0	0	0	0
0	0	0	0	0	0	0	0	0	0	0	0	1054	0	0	0
193	0	0	0	296	0	0	0	0	0	377	0	76234	1302	0	0
0	0	0	0	0	0	0	0	0	417	0	0	0	0	0	0
0	0	0	0	0	0	0	0	0	0	0	0	0	0	0	0
0	0	0	0	0	0	0	0	0	0	0	0	0	0	0	0
0	0	0	0	0	0	0	0	0	0	0	0	0	0	0	0
0	0	0	0	0	0	0	0	0	0	0	0	0	0	0	0
0	0	0	0	0	0	0	0	0	0	201	0	0	0	0	0
0	0	0	0	0	0	0	0	0	0	0	0	0	0	0	0
0	0	0	0	0	0	0	0	0	0	0	0	0	0	0	0
0	0	0	0	0	0	0	0	0	0	0	0	0	0	0	0
0	0	0	0	0	0	0	0	0	0	0	0	0	0	0	0
0	0	0	0	0	0	0	0	0	0	0	0	0	0	0	0
0	0	18586	0	307	0	0	0	0	62	0	0	3838	0	0	0
0	0	0	0	0	0	0	0	0	0	0	0	0	0	0	0
0	0	0	0	0	0	0	0	0	0	0	0	0	0	0	0
0	0	0	0	0	0	0	0	0	0	219	0	0	0	0	93
0	0	0	0	0	0	0	0	0	0	0	0	0	0	0	0
0	0	0	0	0	0	0	0	0	0	0	0	0	0	0	0
0	0	0	0	0	0	0	0	0	0	0	0	0	0	0	0
0	0	0	0	0	0	0	0	0	0	0	0	0	0	0	0
0	0	0	0	0	0	0	0	0	0	0	0	0	0	0	0
0	0	0	0	0	0	0	0	0	0	0	0	0	0	0	0
0	0	0	0	0	0	0	0	0	0	0	0	0	0	0	0
0	0	0	0	0	0	0	0	0	0	0	0	0	0	0	0
0	0	0	0	0	0	0	0	0	0	0	0	0	0	0	0
0	0	0	0	0	0	0	0	0	0	0	0	0	0	0	0
0	0	0	0	0	0	0	0	0	0	0	0	0	0	0	0
0	0	0	0	0	0	0	0	0	0	0	99	0	0	0	0
0	0	1874	0	0	0	0	0	0	0	0	0	0	0	0	0
0	0	0	0	0	0	0	0	0	0	0	0	0	0	0	0
0	130	0	0	0	0	0	0	0	0	0	0	0	0	0	0
0	0	0	0	0	0	0	0	0	0	0	0	0	0	0	0
0	0	0	0	0	0	0	0	0	0	0	57	12624	0	0	0
0	0	0	0	0	0	0	0	0	0	4099	0	8917	0	0	0
166	221	0	0	0	0	0	0	55	0	0	0	0	0	0	0
663	610	5146	3885	618	12351	40466	47321	1563	3845	605	280	379	0	1168	299
0	0	0	0	0	0	0	0	0	0	0	0	0	0	0	0
0	0	0	0	0	0	0	0	0	0	0	0	0	0	0	0
0	0	0	0	0	0	0	0	0	0	0	0	0	0	0	0
0	0	0	0	0	0	0	0	0	0	0	0	0	0	0	0
0	0	0	0	0	0	0	0	0	0	0	0	0	0	0	0
0	0	0	0	0	0	0	0	0	0	0	0	812	0	0	0
0	0	409	0	0	0	0	0	115	138	234	0	0	0	0	0
0	0	0	0	0	0	0	0	0	0	0	0	0	0	0	0
0	0	0	74	0	0	0	45	0	0	0	385	0	135	76	0
0	0	0	0	0	243	0	0	0	0	0	0	0	0	0	0
89	124	110	290	0	0	34	146	266	64	0	0	0	0	219	182
85	0	0	929	0	0	0	0	132	241	0	0	0	0	0	0
0	0	0	0	0	0	0	0	0	0	0	0	0	0	0	0
0	0	0	0	0	0	0	0	0	0	0	0	0	0	0	0
0	0	0	0	0	0	0	0	0	0	0	0	0	0	0	0

839	0	0	582	2665	0	0	0	0	0	0	0	3288	218	0	369
0	0	0	0	0	0	0	0	0	0	0	0	0	0	0	0
0	0	0	0	0	0	0	0	0	0	0	0	0	0	0	0
0	0	0	0	0	0	0	0	0	0	0	0	0	0	0	0
0	0	0	0	0	0	0	0	0	0	0	0	0	0	0	0
0	0	0	0	0	0	1115	0	0	0	0	0	0	0	0	0
3547	3804	4658	5788	3888	1437	28965	783	4676	4370	1854	510	589	274	4787	434
89	0	0	0	0	0	135	0	0	0	0	22706	187	257	5514	264
0	0	0	0	0	0	0	0	0	0	0	0	0	0	0	0
0	0	0	0	0	0	0	0	0	0	0	0	0	0	0	0
0	0	0	0	0	0	0	0	0	0	0	0	0	0	444	0
0	0	0	0	0	0	0	0	0	0	0	0	0	0	0	0
0	0	0	0	0	0	0	0	0	0	0	0	0	0	0	0
0	0	0	0	0	0	0	0	0	0	0	0	0	0	0	0
39091	37764	32085	38206	43337	19273	34969	30237	42510	32465	9843	5274	5509	9437	20005	834
0	0	0	0	0	0	0	0	0	0	0	5207	0	355	0	689
0	0	119	0	0	0	0	0	0	63	2279	27581	388	6200	0	14233
0	0	0	0	0	0	0	0	0	0	0	0	0	0	0	0
0	0	0	0	0	0	0	0	0	0	0	0	800	0	0	0
0	0	1435	231	0	0	0	0	0	0	2525	4580	207	3607	0	19632
0	0	0	0	0	0	0	0	0	0	0	4795	0	500	0	6159
0	0	881	193	0	238	0	42	59	54	1483	0	200	0	145	790
0	0	0	0	0	0	0	0	0	0	0	0	0	0	0	0
0	206	0	0	0	0	154	175	210	0	0	0	0	0	3601	0
0	0	0	0	0	0	0	0	0	55	0	0	0	0	0	0
0	0	0	0	0	0	509	0	0	0	0	0	0	0	0	0
0	0	0	0	0	0	0	0	0	0	0	51	0	0	0	90
0	0	334	68	0	60	0	0	0	0	19206	174233	6109	33178	30796	203446
0	0	201	74	0	0	221	0	59	251	199	103	227	256	1132	77
0	0	0	0	0	0	0	0	0	0	0	0	0	0	0	0
0	0	0	0	0	0	0	0	0	0	0	0	0	114	0	0
0	0	0	0	0	0	0	0	0	0	0	0	0	0	0	0
0	0	0	0	0	0	0	0	0	0	0	0	0	131	0	0
0	0	0	0	0	0	0	0	0	0	0	0	0	0	0	0
0	0	0	0	0	0	0	0	0	0	0	0	0	0	0	0
0	0	0	0	0	0	0	0	0	0	0	0	0	0	0	0
0	0	0	0	0	0	0	0	0	0	0	0	0	0	0	0
0	0	0	0	0	0	0	0	0	0	0	0	0	0	0	0
0	0	0	0	0	0	0	0	0	0	0	0	2079	0	0	0
0	0	0	70	0	0	0	0	0	0	0	0	0	0	0	0
0	0	0	0	0	0	0	0	0	0	823	248	0	359	0	191
0	0	0	0	0	0	0	0	0	0	238	0	0	0	7629	0
0	0	0	0	0	0	0	0	0	0	0	0	0	262	0	0
0	0	0	0	0	0	0	0	0	0	0	0	0	0	0	0
13013	2683	1448	1553	3874	130	1479	434	990	4119	205	0	2658	0	659	76
0	0	0	0	0	0	0	0	0	0	0	0	0	0	0	0
0	0	0	0	0	0	0	0	0	0	0	0	0	0	0	0
0	0	0	0	0	0	0	0	0	0	818	45	0	112	0	255
0	0	0	0	0	0	0	0	0	0	0	0	0	0	0	0
0	0	0	0	0	0	0	0	0	0	0	0	0	0	0	0
0	0	0	0	0	0	0	0	0	0	0	0	0	0	0	0
0	0	0	0	0	0	91	123	0	0	0	0	2039	0	0	0
0	0	0	0	0	0	0	0	0	0	0	0	0	0	0	0
0	0	0	0	0	0	0	0	0	0	0	0	0	0	0	0
196	117	0	132	302	110	2645	186	127	320	633	63593	1421	1500	21811	4159
0	0	0	0	0	0	0	0	0	0	0	0	0	0	0	0
0	0	0	0	0	0	0	0	0	0	0	0	0	0	0	0
0	0	0	0	285	0	0	0	0	0	0	0	0	0	0	0

0	0	0	0	0	0	0	0	0	0	216	12384	0	123	0	79
87	0	0	0	0	0	0	0	0	0	0	0	0	0	0	0
0	0	0	0	0	0	0	0	0	0	0	0	0	0	0	0
0	0	0	0	0	0	0	0	0	0	0	0	0	0	0	0
480	364	0	557	582	0	95	234	336	0	0	0	0	0	0	0
0	0	0	0	0	0	0	0	0	0	0	0	0	0	0	0
0	0	0	0	0	0	0	0	0	0	0	0	0	0	0	0
0	0	0	0	0	0	0	0	0	0	0	0	0	0	0	0
0	0	0	0	0	0	0	0	0	0	0	0	0	0	0	0
0	0	0	0	0	0	0	0	0	0	0	0	0	0	0	0
0	0	0	0	0	0	0	0	0	0	0	0	0	0	0	0
0	0	0	0	0	0	0	0	0	0	0	0	0	0	0	0
0	0	0	0	0	71	0	0	0	0	0	0	0	0	0	0
0	0	0	0	0	0	0	0	0	0	0	0	0	0	0	0
0	0	0	0	0	0	0	0	0	0	0	0	0	0	0	0
0	0	0	0	0	0	0	0	0	0	1357	0	0	0	0	0
0	0	0	0	0	0	0	0	0	0	0	0	0	0	0	0
0	0	0	0	0	0	0	0	0	0	0	0	0	0	0	0
0	0	0	0	0	0	0	0	0	0	0	0	0	0	0	0
0	0	0	0	0	45	0	0	0	0	0	0	0	0	0	0
0	0	0	0	0	0	0	0	0	0	0	0	0	0	0	0
0	0	0	0	0	0	0	0	0	0	0	0	0	0	0	0
0	0	0	0	0	0	0	0	0	0	0	0	0	0	0	0
0	0	0	0	0	0	0	0	0	0	0	4255	2058	127	69	0
0	0	0	0	0	0	0	0	0	0	0	0	0	0	0	3410
0	0	0	0	0	0	0	0	0	0	0	0	0	0	221	0
0	0	0	0	0	0	0	0	0	0	0	0	0	0	0	0
0	0	0	0	0	0	0	0	0	0	0	0	0	0	0	0
0	0	0	0	0	0	0	0	0	0	0	0	0	0	0	0
0	0	0	0	0	0	0	0	0	0	0	0	0	0	0	0
0	0	0	78	0	0	0	0	0	0	0	0	0	0	0	0
90	0	0	0	0	53	547	0	209	0	0	0	0	0	0	0
0	0	0	0	0	0	0	0	0	0	0	0	0	0	0	0
0	0	0	0	0	0	0	0	0	0	0	0	0	0	0	0
0	0	0	0	0	0	0	0	0	0	0	0	0	0	0	0
0	0	107	0	0	74	0	0	0	0	210	1007	0	131	0	86
0	0	0	0	0	0	0	0	0	0	0	0	0	0	0	0
538	0	5591	74	906	126	425	1940	420	104	2910	146743	183	749	4517	448
0	0	0	0	0	120	0	0	0	0	0	4109	0	259	404	0
0	0	0	0	0	0	0	0	0	0	0	0	0	0	0	0
0	0	0	0	0	0	0	0	0	0	0	0	0	0	0	0
0	0	0	0	0	0	0	0	0	0	0	0	0	0	0	0
96	0	0	0	0	0	0	0	0	0	0	0	428	364	0	0
0	0	0	0	0	0	0	0	0	0	0	0	0	0	0	0
0	0	0	0	0	0	0	0	0	0	0	0	0	0	0	0
0	0	116	0	0	0	0	0	0	64	0	64	7317	718	0	99
0	0	0	0	0	0	0	0	0	0	0	0	0	0	0	0
187	378	110	143	0	0	808	0	132	208	0	182	0	128	73	0
0	0	0	0	0	0	0	0	0	0	0	0	0	0	0	0
0	0	0	0	0	0	0	0	0	0	0	0	0	0	0	0
0	0	0	0	0	0	0	0	0	0	0	0	0	0	0	0
0	0	0	0	0	0	0	0	0	0	1047	0	0	0	0	0
0	0	0	0	0	0	0	0	0	0	389	0	618	575	4002	3364
0	0	0	0	0	0	0	0	0	0	0	0	2031	0	0	0
0	0	0	0	0	0	0	0	0	0	0	0	0	0	0	0
0	0	663	0	0	0	0	0	0	0	0	0	0	0	0	0
0	0	0	0	0	0	0	0	0	0	594	0	220	0	81	529
0	0	0	0	0	0	0	0	0	0	0	0	0	0	0	0

0	0	0	0	0	0	0	0	0	0	0	0	0	0	0	0
0	0	0	0	0	0	0	0	0	0	0	0	0	0	0	0
0	0	0	0	0	0	0	0	0	0	0	0	0	0	0	0
0	0	0	0	0	0	0	0	0	0	0	0	0	0	3329	0
0	0	0	0	0	0	0	0	0	0	0	0	0	0	0	0
0	0	0	0	0	0	0	0	0	0	0	0	0	0	0	0
0	0	0	0	0	0	0	0	0	0	0	0	0	0	0	0
0	0	0	0	0	0	0	0	0	0	0	0	0	0	0	0
0	0	0	0	0	0	0	0	0	0	0	0	0	0	0	0
0	0	0	0	0	0	0	0	0	0	0	0	0	0	0	0
0	0	0	0	0	0	0	0	0	0	0	0	1268	0	0	0
0	0	0	0	0	0	0	0	0	0	0	0	0	0	0	0
88	0	0	456	577	59	153	97	357	0	0	13217	608	0	0	92
0	0	0	0	0	0	0	0	0	0	0	0	0	0	0	0
0	0	0	184	574	66	0	0	0	0	0	0	1400	0	0	86
0	0	0	0	0	0	0	0	0	0	0	0	0	0	0	0
0	0	0	0	0	0	0	0	0	0	0	0	0	0	0	0
0	0	0	0	0	0	0	0	0	0	0	0	0	0	0	0
0	0	0	0	0	0	0	0	0	0	1664	0	0	0	0	0
0	0	0	0	0	0	0	0	0	0	0	4393	0	0	0	79
0	0	0	0	0	0	0	0	0	0	0	96	0	0	0	0
80	356	0	572	288	0	97	347	362	59	0	0	0	0	0	0
0	0	0	499	0	0	0	0	0	0	0	0	0	0	0	0
0	0	0	0	0	0	0	0	0	0	0	0	0	0	0	0
79	0	0	76	287	0	0	0	0	0	0	0	218	0	0	0
0	0	0	0	0	0	0	0	0	0	0	0	0	0	0	0
5595	7216	5961	7539	4421	2028	6888	1967	8722	5301	841	231	1237	501	3986	89
0	0	0	0	0	0	0	0	0	0	0	0	0	0	0	0
0	0	0	0	0	0	0	0	0	0	0	0	0	0	0	0
0	0	0	0	0	0	0	0	0	0	0	0	0	0	0	0
0	0	0	0	0	0	0	0	0	0	0	0	0	0	0	0
0	0	112	0	0	0	0	0	0	0	604	0	0	0	92	83
0	0	0	0	0	0	0	0	0	0	0	0	0	0	0	0
0	0	0	0	0	0	0	0	0	0	0	0	0	0	0	0
0	0	0	0	0	0	0	0	0	0	0	0	0	0	0	0
0	0	0	0	0	0	0	0	0	0	0	0	0	0	0	0
0	0	0	0	0	0	0	0	0	0	0	0	0	0	0	0
0	0	0	0	0	0	0	0	0	0	200	52	0	0	0	0
0	0	0	0	0	0	0	0	0	0	0	0	400	131	0	0
0	0	0	0	0	0	0	0	0	0	0	0	0	0	0	264
0	0	0	0	0	125	0	0	0	0	0	0	0	0	0	0
0	0	0	0	0	0	0	0	0	0	0	2123	0	0	0	0
0	0	330	0	0	56	0	0	0	0	0	1015	0	0	0	0
0	0	0	0	0	0	0	0	0	0	0	0	0	0	0	0
0	0	0	0	0	0	0	0	0	0	0	0	0	0	0	0
0	0	0	0	0	0	0	0	0	0	0	0	0	0	0	0
0	0	326	0	0	0	0	0	0	0	0	0	0	0	0	0
0	0	0	0	0	0	0	0	0	0	0	0	0	0	0	0
0	0	0	0	0	0	0	0	0	74	0	537	0	0	0	167
0	0	0	0	0	0	0	0	0	0	0	0	0	0	0	0
0	0	0	0	0	0	0	0	0	0	0	0	0	0	0	0
0	0	0	0	0	0	0	0	0	0	0	0	0	0	0	0
0	0	0	0	0	0	0	0	0	0	0	0	0	0	0	5359
0	0	0	0	0	0	0	0	0	0	0	0	0	0	0	0
0	0	0	0	0	0	0	0	0	0	0	0	0	0	0	0
0	0	0	0	0	0	0	0	0	0	0	0	0	0	0	0
0	0	0	0	0	0	0	0	0	0	0	0	0	0	0	0
5872	2610	1392	1605	2322	604	4301	531	2923	2210	0	104	805	0	2869	79

0	0	0	0	0	0	0	0	0	0	0	0	0	0	0	0
0	0	0	0	0	0	0	0	0	0	0	0	0	0	0	0
0	0	0	0	0	0	0	0	0	0	0	0	0	0	0	0
0	0	0	0	0	0	0	0	0	0	0	0	0	0	0	0
0	0	0	0	0	0	0	0	0	0	0	0	0	0	0	0
0	0	320	0	0	0	0	0	0	0	0	0	401	0	0	1702
0	0	0	0	0	0	0	0	0	0	0	0	0	0	0	0
0	0	0	0	0	0	0	0	0	0	0	0	0	0	0	0
0	0	0	0	0	0	0	0	0	0	0	0	0	0	0	0
0	0	0	0	0	0	0	0	0	0	0	0	0	0	0	0
0	0	0	0	0	0	0	0	0	0	0	0	0	0	72	0
0	0	0	0	0	0	0	0	0	0	0	0	0	0	0	0
0	0	0	0	0	0	27	0	0	0	0	0	0	0	0	0
0	0	0	0	0	0	0	0	62	259	0	0	0	0	0	0
0	0	0	0	0	0	0	0	0	0	0	0	0	0	0	0
0	0	0	0	0	0	0	0	0	0	0	0	0	0	0	0
0	0	0	0	0	0	0	0	0	0	0	0	0	0	0	0
90	0	0	639	0	0	0	0	132	0	0	0	0	0	0	0
0	0	0	0	0	0	0	0	0	0	0	0	0	0	0	0
0	0	0	0	0	0	0	0	0	0	0	0	0	0	0	0
0	0	0	0	0	0	0	0	0	0	0	0	0	0	0	0
0	0	0	0	0	0	0	0	0	0	0	0	0	0	0	0
117	0	285	0	0	0	0	0	0	0	4917	118	23248	223	6268	86
0	0	0	0	0	0	0	0	0	0	0	0	0	0	0	0
0	0	0	0	0	0	806	0	0	0	0	0	0	0	0	0
0	0	0	0	0	0	0	0	0	0	0	0	0	0	0	0
0	0	0	0	0	0	2590	48	0	0	0	0	0	0	0	0
0	0	0	0	0	0	0	0	0	0	0	0	0	0	0	0
637	0	0	219	890	0	0	0	0	0	3085	2049	1892	0	0	0
0	0	0	0	0	0	0	0	0	0	0	0	0	0	79	0
0	0	0	0	0	0	0	0	0	0	0	0	0	0	0	0
0	0	0	0	0	0	0	0	215	0	0	0	0	0	0	0
0	0	0	0	0	0	0	0	0	0	0	0	0	0	0	0
0	0	0	0	0	0	2282	0	0	0	0	0	0	0	0	0
0	0	0	0	0	0	0	0	0	0	0	0	0	0	0	0
0	0	0	0	0	0	0	0	0	0	0	0	0	0	0	0
0	0	0	0	0	0	0	0	0	0	0	0	0	0	0	0
0	0	0	0	0	0	0	0	0	0	213	56	0	0	0	0
0	0	0	0	0	0	0	0	0	0	0	0	0	0	0	0
0	0	0	0	0	0	0	0	0	0	0	0	0	0	0	0
0	0	0	0	0	0	0	0	0	0	0	0	0	0	0	0
0	0	0	0	0	0	0	0	0	0	0	0	0	0	0	0
0	0	0	0	0	0	0	0	0	0	0	0	0	0	0	0
0	0	0	0	0	0	0	0	0	0	0	0	0	349	0	0
0	0	0	0	0	0	0	0	0	0	0	0	0	0	0	0
0	0	0	0	0	0	0	0	0	0	0	0	0	0	0	0
0	0	0	0	0	0	0	0	0	0	0	0	0	0	0	0
0	0	0	0	0	0	0	0	0	0	0	0	0	0	0	0
0	0	0	0	0	0	0	0	0	0	0	0	0	0	0	0
0	0	0	0	0	0	0	0	0	0	0	0	0	0	0	0
0	0	0	0	0	0	0	0	0	0	0	0	0	0	0	0
0	0	0	0	0	0	0	0	0	0	0	0	0	0	0	0
1225	1720	20919	7706	2358	3865	399	1686	2161	2787	9075	1629	1180	3121	18208	1066
0	0	0	0	0	0	0	0	0	0	0	0	0	0	0	0
0	0	0	0	0	0	497	256	249	0	0	0	0	0	0	0
0	0	0	0	0	0	0	0	0	0	0	0	0	0	0	0
0	0	0	80	0	0	0	0	0	0	847	0	0	0	427	455
0	0	0	0	0	0	0	0	0	0	0	0	0	0	0	0

0	0	0	0	0	0	0	0	0	0	0	0	0	0	0	0
0	0	0	0	296	0	0	0	0	0	0	0	199	0	0	0
0	0	0	0	0	0	0	0	0	0	0	0	0	0	0	0
0	0	0	0	0	0	0	0	0	0	0	0	423	0	0	0
0	0	204	0	0	0	0	0	0	0	0	0	0	0	0	0
0	0	432	0	0	0	0	0	0	55	0	0	401	0	630	0
0	0	0	0	0	0	0	0	0	0	0	0	0	0	0	0
0	0	0	0	0	0	0	0	0	0	0	0	0	0	0	0
0	0	0	0	0	0	0	0	0	0	0	0	0	0	0	0
0	0	0	0	0	0	0	0	0	0	0	0	0	0	0	0
0	0	0	0	0	0	0	0	0	0	0	0	0	0	0	0
0	0	0	0	0	0	0	0	0	0	0	0	0	0	0	0
937	4656	8394	14066	3466	130739	1125	88402	5284	19908	0	251	398	123	802	259
0	0	0	0	0	0	0	0	0	0	0	0	0	0	0	0
85	0	0	0	579	255	366	537	529	0	116404	1231	8166	3600	44377	599862
0	0	0	0	0	0	0	0	0	0	0	0	0	0	0	0
0	0	0	0	0	0	0	0	0	0	0	0	0	0	0	0
0	0	0	0	0	0	0	0	0	0	0	0	0	0	0	0
0	0	0	0	0	0	0	0	0	0	0	0	0	0	0	0
0	0	0	0	0	1344	0	0	0	0	0	0	0	0	0	0
0	0	0	0	0	0	0	0	0	0	0	450	0	0	0	641
0	0	0	0	0	112	1044	0	0	0	185	0	0	0	0	0
0	0	0	0	0	0	0	0	0	0	0	0	0	0	0	0
0	0	0	0	0	0	0	0	0	0	0	0	0	0	0	0
0	0	0	0	0	0	0	0	0	0	201	0	0	0	0	0
0	0	0	0	0	0	0	0	0	0	0	0	0	0	0	0
0	0	0	0	0	0	0	0	0	0	0	0	0	0	0	0
0	0	0	0	0	0	0	0	0	0	0	0	0	0	0	0
0	0	0	0	0	0	0	0	0	0	0	0	0	0	0	0
31898	88074	60309	71561	84743	21289	103943	24718	86413	52299	4976	1502	7771	1212	35863	962
0	0	1914	0	0	0	30	0	61	0	0	0	0	0	0	0
0	0	0	0	0	0	0	0	0	242	0	0	0	0	0	0
0	0	0	0	0	0	0	0	0	0	0	0	0	0	0	0
0	0	0	0	0	0	0	0	0	0	0	0	0	0	0	0
0	0	0	0	0	0	0	0	0	0	0	0	0	0	0	0
0	0	0	0	0	0	0	0	0	0	0	0	0	0	0	0
0	0	0	0	0	0	0	0	0	0	0	0	0	0	0	0
0	0	0	0	0	0	0	0	0	0	0	0	0	0	0	0
0	0	0	0	0	0	0	0	0	0	0	0	0	0	0	0
0	0	0	0	0	0	0	0	0	0	0	0	0	0	0	0
0	0	0	0	0	0	0	0	0	0	0	0	0	0	0	0
0	0	0	0	0	0	0	0	0	0	10772	6935	2260	7095	6952	2472
188	0	0	0	584	0	0	0	0	0	0	0	199	0	0	0
0	0	0	0	0	0	0	0	0	0	0	0	0	0	0	0
0	0	0	0	0	0	0	0	0	0	0	0	0	0	0	0
0	0	0	0	0	0	0	0	0	0	0	0	0	0	0	0
0	0	0	0	0	0	0	0	0	119	172	52	0	117	76	0
0	0	0	0	0	0	0	0	0	0	0	0	0	0	0	0
0	0	0	0	0	0	0	0	0	0	0	0	0	0	0	0
0	0	0	0	0	0	0	0	0	0	0	0	0	0	0	0
0	118	120	0	0	0	0	0	0	63	0	0	0	0	0	0
0	0	0	0	0	0	0	0	0	0	0	0	0	0	0	0
0	0	0	0	0	0	0	0	0	0	0	0	0	0	0	0
0	0	0	0	0	0	0	0	0	0	0	0	0	0	0	0
0	0	0	0	0	0	0	0	0	0	0	18440	0	112	0	0
0	0	0	0	0	0	66	0	54	0	0	0	0	0	0	0
0	0	0	0	0	0	0	0	0	0	0	0	0	0	0	0
0	0	0	0	0	0	0	0	0	0	0	0	0	0	0	0

0	0	0	0	0	0	0	30	0	0	1410	0	0	1037	77	3498
0	0	0	0	0	0	0	0	0	0	0	0	0	0	0	0
0	0	0	0	0	0	0	0	0	0	0	0	0	0	0	0
209	263	0	298	0	1115	1225	0	399	257	0	245	383	0	2375	0
0	0	0	0	0	0	0	0	0	0	0	0	0	0	1089	0
0	0	0	0	0	0	0	0	0	0	402	2281	0	120	0	231
80	384	830	68	0	0	0	0	0	1125	0	0	0	0	0	0
0	0	0	0	0	0	0	0	0	0	0	0	0	0	0	0
0	0	0	0	0	0	0	0	0	0	0	0	0	0	0	0
0	0	0	0	0	0	0	0	0	0	0	0	0	0	0	0
0	0	0	0	0	0	0	0	0	0	0	0	0	0	0	0
0	0	0	0	0	0	0	0	0	0	0	0	2486	0	2448	0
0	0	0	0	0	0	0	0	0	0	0	0	0	0	0	0
0	0	0	0	0	0	0	0	0	0	0	0	0	0	0	0
0	0	0	0	0	0	0	0	0	0	0	0	0	0	0	0
0	0	0	0	0	0	0	0	0	0	0	0	0	0	0	0
0	0	0	0	0	0	0	0	0	0	0	0	0	0	136	0
0	0	0	0	0	0	0	0	0	0	397	0	0	0	382	340
0	0	0	0	0	0	0	0	0	0	0	0	0	0	0	0
0	0	0	0	0	0	0	0	0	0	0	0	0	0	0	0
0	0	0	0	0	0	0	0	0	0	0	0	0	0	0	0
0	0	0	0	0	0	0	0	0	0	0	0	0	0	0	0
0	0	0	0	0	0	23	46	0	0	0	4184	0	0	0	0
0	0	0	0	0	0	0	0	0	0	0	0	0	0	0	0
0	0	0	0	0	0	0	0	0	0	0	0	0	0	0	0
0	0	0	0	0	0	0	0	0	0	0	0	0	0	0	0
0	0	0	0	0	0	0	0	0	0	0	0	0	0	0	451
0	0	0	0	0	0	0	0	0	0	0	0	0	0	0	0
0	0	0	0	0	0	0	0	0	0	0	0	0	0	0	0
0	0	241	0	0	0	0	0	0	0	2333	1101	0	619	0	4382
0	0	0	0	0	0	0	0	0	0	0	0	0	0	0	0
0	0	0	0	0	0	0	0	0	0	0	0	0	0	0	0
0	0	0	0	0	0	0	51	79	0	0	0	0	0	0	0
0	0	0	0	0	0	0	0	0	0	0	0	0	0	0	0
0	0	0	0	0	0	0	0	0	0	0	0	0	0	0	0
0	0	0	0	0	0	0	0	0	0	0	0	0	230	0	0
0	0	0	0	0	0	166	121	72	0	0	0	0	0	0	0
0	0	0	0	0	0	0	0	0	0	0	0	0	0	0	0
0	0	0	0	0	0	0	0	0	0	0	0	0	0	0	0
0	0	0	0	0	0	0	0	0	0	198	0	0	0	0	0
0	0	0	0	0	0	0	0	0	0	0	0	0	0	0	0
0	0	0	0	0	0	0	0	0	0	0	0	0	0	0	0
0	0	0	0	0	0	137	53	0	64	0	0	0	0	0	0
0	0	0	0	0	0	0	0	0	0	0	0	0	0	0	0
0	0	0	0	0	0	0	0	0	0	0	0	0	0	0	0
0	0	0	0	0	0	0	0	0	0	0	0	0	0	0	0
0	0	0	0	0	0	0	0	0	0	0	0	0	0	0	0
0	0	0	0	0	0	0	0	0	0	0	0	0	0	0	0
0	0	0	0	0	0	0	0	0	0	0	0	0	0	0	0
0	219	217	0	0	0	0	0	0	364	423	1404	208	0	87	0
0	0	0	0	0	0	0	0	0	0	0	0	0	0	2175	0
0	0	0	0	0	0	0	0	0	0	0	0	0	0	0	0
0	0	0	0	0	0	0	0	0	0	0	0	0	0	0	0
0	0	0	0	0	0	0	0	0	0	0	0	0	0	0	0
0	0	0	0	0	0	0	0	0	0	0	0	0	0	0	0
0	0	0	0	0	0	74	0	0	0	0	0	0	0	0	0
0	0	0	0	0	0	0	0	0	0	0	0	0	0	0	0
0	0	0	0	0	0	0	0	0	0	0	0	0	0	0	0

0	0	0	0	0	0	0	0	0	0	0	0	0	0	0	0
0	0	0	0	0	0	0	0	0	0	0	0	0	0	0	0
0	0	0	0	0	0	0	0	0	0	0	0	2031	0	0	0
0	0	0	0	0	0	0	0	0	0	0	0	188	0	0	0
0	0	0	0	0	0	0	0	0	0	0	0	0	0	0	0
0	0	0	0	0	0	0	0	0	0	0	0	0	0	0	0
0	0	0	0	0	0	0	0	0	0	0	0	0	0	0	0
91	0	0	0	298	0	0	0	0	0	0	0	0	0	0	0
0	0	1548	0	0	0	0	190	0	0	0	0	11567	0	0	0
0	0	0	0	0	0	0	0	0	0	0	0	0	0	0	0
0	0	0	0	0	0	0	0	0	0	0	0	0	0	0	0
0	0	0	0	0	0	0	0	0	0	0	0	0	0	0	0
0	0	0	0	0	0	0	0	0	0	0	0	0	0	0	0
0	0	0	0	0	0	27	0	0	0	0	0	0	0	0	0
0	0	0	0	0	0	0	0	0	0	0	0	0	0	0	0
0	0	0	558	0	0	0	0	0	0	0	0	0	0	0	0
0	0	7533	0	0	0	0	0	0	0	0	0	5871	0	0	0
0	0	0	0	0	0	0	0	0	0	0	0	0	0	0	0
0	0	0	0	0	0	0	0	0	0	0	0	0	0	0	0
322	3232	334	4021	5576	1604	341	7278	2659	3308	0	114	0	0	143	0
283895	11638	84211	24130	9812	82914	11087	17067	8604	191656	15415	4420	241979	3432	62542	6747
0	0	0	0	0	0	0	0	0	0	0	0	0	0	0	0
0	0	0	72	596	0	0	0	0	0	0	0	225	0	0	0
1280	16624	3484	20138	27672	13459	740	36200	17011	21256	208	106	215	0	1248	84
0	0	0	0	0	0	0	0	0	0	0	0	0	0	0	0
0	0	0	0	0	0	0	0	0	0	0	0	0	0	0	0
0	0	116	73	0	1950	42	1453	114	115	0	0	439	0	0	0
0	0	0	0	0	0	0	0	0	0	0	0	0	0	0	0
0	0	0	0	0	0	0	0	0	0	0	0	0	0	0	0
0	0	2075	0	0	0	0	0	0	0	0	0	0	0	0	0
0	0	0	0	0	0	68	91	133	0	0	0	0	0	0	270
0	0	0	0	0	0	0	0	0	0	0	0	0	0	0	0
0	0	0	0	0	0	0	0	0	0	215	2834	0	0	3716	276
1347	2623	1543	1817	882	354	2287	485	1783	862	0	69	206	0	839	0

COVID-19-029	COVID-19-030	COVID-19-031	COVID-19-032	COVID-19-035	COVID-19-037	COVID-19-038	COVID-19-040	COVID-19-041	COVID-19-043	COVID-19-045	COVID-19-046	COVID-19-047	COVID-19-048	COVID-19-049	COVID-19-050
856377	822704	737663	744726	967355	870219	962609	995318	537718	668958	729910	988393	978166	956638	966850	771396
133817	155166	256095	178412	24281	103471	5745	2626	455535	291429	4477	2406	6385	9447	1685	158978
0	0	0	0	0	0	0	0	0	0	0	0	0	0	0	0
9641	21944	5116	42999	8290	5948	30904	1684	6747	28917	263037	8911	13925	31471	31254	7398
165	186	1030	33863	74	20222	742	0	0	9853	2576	226	815	2267	211	61029
0	0	0	0	0	0	0	0	0	0	0	0	0	0	0	0
0	0	96	0	0	140	0	372	0	843	0	64	709	177	0	1199

CC029	CC030	CC031	CC032	CC035	CC037	CC038	CC040	CC041	CC043	CC045	CC046	CC047	CC048	CC049	CC050
0	17751	0	0	0	0	362	0	0	0	0	58	0	169	0	0
0	0	0	0	0	0	0	0	0	0	0	0	0	0	0	0
14778	29022	46321	182507	118347	129740	799978	18582	49187	149881	33058	247821	2448	750002	4204	539138
0	0	0	0	3952	0	0	0	0	0	0	0	0	0	0	0
0	0	0	0	0	0	0	0	0	0	0	0	0	0	0	0
0	0	83	8483	0	0	0	0	0	0	0	0	0	0	0	0
0	0	0	0	0	0	0	0	0	0	0	0	0	0	0	0
0	0	0	0	0	0	48	0	0	0	0	1371	914	165	0	0
0	0	0	0	0	0	0	0	0	2716	0	0	0	0	0	0
76	96	17505	13231	93	3512	1399	256	0	22549	313	222	0	542	0	5093
0	0	0	0	0	0	0	0	0	0	0	0	0	0	0	0
0	0	0	0	0	0	0	0	0	0	0	0	247	0	0	0
0	0	0	0	0	0	0	0	0	0	0	0	0	0	0	0
178	48	88	266	174	795	1986	41470	441	3919	1305	1158	130346	4703	466	810
0	87	0	80	0	0	580	0	0	276	303	287	0	973	0	0
0	0	0	0	0	0	0	0	0	0	0	0	0	0	0	0
0	0	0	0	0	0	0	0	0	0	0	0	0	0	0	0
319	0	0	0	0	0	0	0	0	150	0	0	0	0	0	0
0	0	0	0	0	0	0	0	0	0	0	0	321	0	0	0
0	0	0	0	0	0	0	0	0	0	0	0	0	0	0	0
0	219	96	0	0	0	65	0	0	283	0	0	63	0	0	0
0	0	0	0	0	0	0	0	0	0	0	0	0	101	0	0
3759	575	1245	260	2104	3019	54	0	0	0	0	0	0	92	113	202
0	0	0	0	0	0	0	0	0	0	0	0	0	0	0	0
0	0	0	0	0	0	0	0	33322	0	331	0	0	0	0	0
57722	73729	59865	26568	62698	102146	2781	1812	22770	10004	631298	1509	93045	3367	592	55083
0	0	0	0	0	0	0	0	0	0	0	0	0	0	0	0
66	0	4847	0	0	0	0	0	90870	0	0	55	0	0	0	0
0	0	0	0	0	0	0	0	0	0	0	0	0	0	0	0
0	0	0	0	0	0	0	0	0	0	0	0	0	0	0	0
0	0	0	0	0	0	0	0	0	0	0	0	0	0	0	0
0	0	0	0	0	0	63	0	0	0	0	0	0	91	0	0
0	0	0	0	0	0	0	0	0	0	0	0	0	0	0	0
0	0	0	0	0	122	0	0	0	0	0	0	0	0	0	0
0	0	0	0	0	0	0	0	0	0	0	273	0	0	0	0
0	0	0	0	0	0	0	0	0	0	0	0	0	0	0	0
0	0	0	0	0	0	0	0	0	0	0	0	0	0	0	0
0	9498	0	0	0	0	0	0	0	0	0	0	0	0	120	282
0	0	0	0	0	0	0	0	0	0	0	0	0	0	0	74
57085	1879	259705	3149	55740	251794	436	135	457	209	3257	105	120	361	344	41784
0	0	0	0	0	0	0	0	0	5200	0	0	0	85	0	0
78	0	1700	0	101	15994	0	0	0	0	0	0	0	0	0	0
0	0	0	0	0	0	0	0	0	0	0	0	0	0	0	0
0	0	0	0	0	0	0	0	0	0	0	0	0	0	0	0
0	0	0	0	0	0	0	0	470	19851	0	0	0	80	0	0
0	0	621	0	0	0	0	0	0	0	0	0	0	0	0	0
0	0	0	0	657	0	1234	0	0	0	0	704	0	1121	0	0

0	0	0	0	0	0	0	0	0	0	0	0	0	0	0	0
0	0	0	0	0	0	0	0	0	0	0	0	0	0	0	0
31084	615	30103	14536	363	210359	2142	642743	6336	6958	10978	695646	1698	1953	11841	37195
0	0	0	0	0	0	0	0	0	0	0	0	0	0	0	0
0	0	0	0	0	0	0	0	0	0	0	0	0	0	0	0
0	0	0	0	0	0	0	0	0	1436	0	0	0	0	0	0
0	0	0	0	0	0	0	0	0	0	0	0	0	0	0	0
0	0	0	0	0	0	0	0	0	0	0	0	0	0	0	0
0	0	0	0	0	0	53	0	0	0	0	0	0	0	0	0
0	0	0	0	0	0	0	0	0	0	0	0	0	0	0	0
0	0	0	0	0	0	0	0	0	0	0	0	0	0	0	0
0	0	0	0	0	0	210	0	0	0	0	55	0	0	0	0
0	0	0	0	0	0	0	0	0	0	0	0	0	0	0	0
0	0	0	0	0	0	0	0	0	0	0	0	0	0	0	0
0	0	0	0	0	0	0	0	0	0	0	0	0	0	0	0
578689	560712	49121	306221	241711	7491	439	1058	2896	567	2163	320	0	465	0	280
0	0	0	0	0	0	0	0	0	0	0	0	0	0	0	0
0	0	0	0	0	0	0	0	0	0	0	0	0	0	0	0
0	0	0	0	0	0	0	0	0	0	0	0	0	0	0	0
3866	321	56772	16796	45896	67110	1115	1105	306807	143370	2859	418	24134	1173	583	1711
0	0	0	0	0	0	0	0	0	0	0	0	0	0	116	0
0	0	0	0	0	0	0	0	0	0	0	0	0	0	0	133
0	0	0	0	0	0	0	0	0	0	0	0	0	0	0	0
0	0	0	0	0	0	0	0	0	0	0	0	0	0	0	0
0	0	0	0	0	0	0	0	0	0	0	0	0	0	0	0
0	0	0	0	0	0	0	0	0	0	0	0	678	0	0	0
0	0	6464	0	0	0	0	0	0	0	0	0	0	80	0	0
0	0	0	0	0	0	0	0	0	0	0	0	0	0	0	0
0	0	0	0	0	0	50	121	85658	0	0	65	0	0	0	0
0	0	0	0	0	0	0	0	0	0	0	0	0	0	0	0
0	0	0	0	0	0	0	0	0	0	0	0	0	0	0	0
0	0	0	0	0	0	0	0	0	0	0	0	0	0	0	0
0	0	0	0	0	0	0	0	0	0	0	0	0	0	0	0
0	73	0	0	87	0	161	139	0	0	0	0	0	258	0	57
0	0	0	0	0	0	0	0	0	0	0	0	0	0	0	0
0	0	0	0	0	0	0	0	0	0	0	0	0	0	0	284
0	0	0	69	4960	0	0	0	0	0	0	0	0	0	0	0
0	0	0	0	0	0	0	0	0	0	0	0	0	0	0	0
0	0	0	0	0	0	0	0	0	0	0	0	0	0	0	0
0	0	91	0	0	0	0	0	0	3967	0	64	0	0	0	69
0	0	0	0	0	0	0	0	0	0	0	0	0	0	0	0
0	0	0	0	0	0	0	0	0	0	0	0	0	0	0	0
0	0	0	280	0	650	0	0	0	0	0	0	0	0	0	0
0	0	0	0	0	0	0	0	0	0	0	0	0	0	0	136
0	0	0	79	0	0	0	0	0	0	0	0	0	0	0	0
0	0	0	0	0	0	0	0	0	0	0	0	0	0	0	0
0	305	0	72	38184	118	0	0	0	0	0	0	0	0	0	0
0	0	0	0	0	0	0	0	0	0	0	0	0	0	0	0
0	0	0	0	0	0	0	0	0	0	0	0	0	0	0	0
0	0	554	0	0	0	0	0	0	0	0	0	0	0	0	0
0	49	120	61	14722	0	0	0	0	0	0	0	0	0	0	0
0	0	0	0	0	0	0	0	0	0	0	0	0	0	0	0
0	0	0	0	0	0	0	0	224	5873	611	0	0	0	0	0
0	0	0	0	0	0	0	0	0	0	0	0	0	0	0	0
84	19737	103	61	0	0	0	0	0	0	0	0	0	0	0	0
82	143	914	0	0	19827	742	0	0	9853	2576	226	815	2267	211	60891
0	0	0	0	0	0	0	0	0	0	0	0	0	0	0	0
0	0	0	0	0	0	0	0	0	0	0	0	0	0	0	0

0	0	0	0	0	0	0	0	0	0	0	0	0	0	0	0
0	0	0	0	0	0	0	0	0	0	0	0	0	0	0	0
0	0	0	0	0	0	0	0	0	0	0	0	0	0	0	0
0	0	0	0	0	0	222	0	0	0	0	0	0	78	0	0
0	0	0	0	0	0	0	0	0	0	0	0	0	0	0	0
0	643	0	0	0	0	0	0	0	0	0	0	0	0	0	0
0	0	0	0	0	0	0	0	0	0	0	0	0	0	0	0
0	0	0	0	0	0	153	0	0	0	0	175	0	397	0	0
0	0	0	0	0	0	0	0	0	0	0	0	0	0	0	0
0	45	15003	0	0	0	0	0	0	0	0	0	0	0	0	61
0	0	0	0	0	0	0	0	0	0	0	0	0	0	0	0
0	0	0	0	0	0	0	0	0	0	0	0	0	0	0	0
0	0	0	0	0	0	0	0	0	0	0	0	0	0	0	0
0	0	0	0	0	0	0	0	0	0	0	0	0	0	0	0
0	0	0	0	0	0	0	0	0	0	0	0	0	0	0	0
144	0	400	6100	9925	122	17961	788	0	9709	965	4442	0	24124	0	261
0	41	0	580	223	0	1601	0	224	102	0	442	0	2481	0	0
102448	126	1031	11636	145	23923	55	135	0	13466	310	0	0	0	0	0
0	0	0	0	0	0	0	0	0	0	0	0	0	0	0	0
0	0	0	0	0	0	0	0	0	0	0	55	65	0	0	0
0	0	0	0	0	0	0	0	0	0	0	0	0	0	0	0
0	0	0	0	0	0	0	0	0	0	0	0	0	0	0	0
9641	21944	5116	42999	8290	5948	30751	1684	6747	28917	263037	8736	13925	31074	31254	7398
161	310	11005	8125	2827	397	0	0	0	47	0	223	0	90	0	2092
489	0	1252	0	675	2666	0	0	0	0	0	0	0	0	129	551
0	0	0	0	0	0	0	0	0	0	0	0	0	0	0	0
0	0	1856	0	0	0	0	0	0	0	0	0	0	0	477	0
0	0	0	0	0	0	0	0	0	0	0	0	0	0	0	0
0	0	0	0	0	0	0	0	0	263	0	0	0	0	0	0
15409	53659	4169	488	132916	12248	99	110	213	2576	1302	149	1282	257	0	0
0	0	206	142	227	0	341	0	0	1376	0	52	0	653	0	0
0	0	0	0	0	0	0	0	0	0	0	0	0	0	0	0
83	43	116	33863	74	395	0	0	0	0	0	0	0	0	0	138
85	47	0	17870	0	127	0	0	0	0	0	0	0	0	0	0
0	0	0	0	0	241	0	0	0	0	0	0	0	0	0	0
0	0	0	0	0	0	0	0	0	0	0	0	0	0	0	0
0	0	0	0	0	0	0	0	0	0	0	0	0	0	0	0
0	0	0	0	0	0	0	0	0	0	0	0	0	0	0	0
315	384	372	128784	249	4774	55	131	223	7093	0	0	0	99	0	0
0	2739	505	0	0	0	0	0	0	0	0	0	0	0	0	0
0	0	0	0	0	0	0	0	0	0	0	0	0	0	0	256
0	0	117	0	0	0	1626	525	209	1376	0	624	2297	1897	114	0
0	0	0	0	0	0	0	0	0	0	0	0	0	0	0	0
0	0	0	0	0	0	0	0	0	0	0	0	0	0	0	0
171	76093	201	130	218	129	0	0	0	0	0	0	0	0	0	0
0	0	0	0	0	0	0	0	0	0	0	0	0	0	0	0
0	0	0	0	0	0	0	130	199	7847	0	57	0	0	0	0
0	0	0	0	0	0	0	0	0	0	0	0	0	0	0	0
0	0	0	0	0	0	0	0	0	0	0	0	0	0	0	0
0	0	0	0	0	0	0	0	0	0	0	0	0	0	0	0
0	0	0	0	0	0	0	0	0	0	0	0	0	0	0	0
67	180	226	0	142	250	517	649	0	1147	0	0	3087	262	0	84
0	0	0	0	0	0	0	0	0	0	0	0	0	0	0	0
0	0	0	0	0	0	0	0	0	0	0	0	0	0	0	0
0	0	0	0	0	0	107	0	117809	163	0	0	0	0	0	0
0	47	0	71	0	137	0	0	0	642	0	0	0	0	0	0
0	0	0	0	0	0	0	0	0	0	0	0	0	0	0	0
0	0	0	0	0	0	0	0	0	0	0	0	58	0	0	0

78	0	14284	0	79	141	263	0	0	0	0	59	0	199	0	60
0	0	0	0	0	0	0	0	0	0	0	0	0	0	0	0
0	0	0	0	0	0	0	0	0	368	0	0	0	0	0	0
0	0	0	0	0	0	0	0	0	0	0	0	0	0	0	0
0	0	0	0	0	1997	0	0	0	0	0	0	0	0	0	0
0	0	0	0	0	3089	0	0	0	0	0	0	0	0	122	0
0	0	0	0	0	0	0	0	0	0	0	0	0	0	0	0
0	0	0	0	0	0	0	0	0	0	0	0	0	0	0	0
0	0	0	0	0	0	0	0	0	0	0	0	0	0	0	0
0	0	0	0	0	0	0	0	0	0	0	0	0	0	0	0
0	0	0	0	0	0	0	0	0	0	0	0	0	0	0	0
0	0	0	0	0	0	0	0	0	0	0	0	0	0	0	0
0	0	1775	0	0	0	0	0	0	0	0	0	0	0	0	0
0	96	0	0	0	0	0	249	0	93	0	0	0	0	0	0
0	0	0	0	0	0	614	651	646	111405	973	240	0	363	0	0
0	0	0	0	0	0	0	0	0	0	0	0	0	0	0	0
0	0	0	0	0	0	0	0	0	0	0	0	0	0	0	0
0	0	0	0	0	147	0	0	0	0	0	0	0	0	0	0
0	0	0	0	0	0	0	0	0	0	0	0	0	0	0	0
0	0	0	0	0	0	0	0	0	0	0	0	0	0	0	0
10460	44152	249923	17680	688	43135	46	0	72398	2309	326	52	2035	240	1093	1244
0	0	0	0	0	0	0	0	0	0	0	0	0	0	0	0
0	0	0	0	0	0	0	0	0	0	0	0	0	0	0	0
75	0	0	0	0	0	0	0	0	0	0	0	0	0	0	0
0	0	0	0	0	0	0	0	0	0	0	0	0	0	0	0
0	49	0	0	1239	388	0	0	0	0	0	0	0	84	0	0
0	2098	0	0	0	0	0	119	0	395	0	0	0	0	0	0
0	0	0	0	0	0	462	0	0	91	0	283	0	843	0	0
0	0	0	0	0	0	0	0	0	604	0	0	0	0	0	0
0	0	0	0	0	0	0	0	0	0	0	0	0	0	0	0
0	0	0	0	0	0	0	0	0	0	0	0	0	0	0	0
0	0	522	0	0	0	0	0	229	1278	0	0	371	0	0	375
0	0	0	0	0	0	0	0	0	0	0	0	0	0	0	0
0	0	0	0	0	0	0	0	0	0	0	0	0	0	0	0
169	53	5386	0	0	2618	0	0	0	0	0	0	0	0	0	769
0	0	0	0	0	0	0	0	0	0	0	0	0	0	0	0
0	0	0	0	0	0	0	0	0	0	0	0	0	0	0	0
0	0	0	0	0	0	0	0	0	0	0	0	0	0	0	0
0	0	0	0	0	0	0	0	0	0	0	0	0	0	0	0
0	0	0	0	0	0	0	0	0	0	0	65	0	0	0	0
9630	0	0	0	0	113	0	0	0	0	0	0	0	0	0	0
0	0	0	0	0	0	0	0	0	0	0	0	0	0	0	0
0	0	0	0	0	0	0	0	0	0	0	0	0	0	0	0
0	0	0	0	0	0	54	0	0	0	0	0	0	0	0	0
0	0	0	0	0	0	0	0	0	0	0	0	0	0	0	113
0	0	0	0	0	0	0	0	0	0	0	0	0	0	0	0
0	0	0	0	77	25467	57	0	0	2530	0	0	0	0	0	198
0	0	6645	0	0	138	0	0	0	0	0	0	0	0	0	79
0	0	0	0	0	0	61	0	0	0	0	0	0	0	0	0
0	0	0	0	0	0	0	0	0	0	0	0	0	0	0	0
0	0	0	0	0	0	0	0	0	0	0	0	0	0	0	0
0	0	0	0	0	0	0	0	0	0	0	0	0	0	0	0
73	0	0	0	0	0	0	0	0	0	0	0	0	0	0	950
0	0	0	0	81	0	557	0	0	0	0	205	0	270	0	0
0	0	0	0	0	0	0	0	0	0	0	0	0	0	0	152
0	0	0	0	0	0	0	0	0	0	0	0	0	0	0	0
158	192	0	1619	888	121	5413	253	437	920	0	2149	0	5263	0	0
0	0	0	0	0	0	0	0	0	0	0	0	0	0	0	0

0	0	0	0	0	0	0	0	0	0	0	0	0	0	0	0
0	0	0	0	0	0	0	0	0	0	0	0	0	0	0	0
0	0	0	0	0	0	0	0	0	0	0	0	0	0	0	0
0	0	594	0	0	786	0	0	0	0	0	0	0	0	0	112
0	0	0	0	0	0	0	0	0	0	0	0	0	0	0	0
0	0	0	0	0	0	0	0	0	0	0	0	0	0	0	0
0	0	0	0	0	0	43	0	0	0	0	0	0	0	0	0
86	146	0	47077	137	279	0	0	0	1977	0	0	64	0	0	70
0	0	0	0	0	0	54	0	87876	74	320	0	0	0	0	0
0	0	0	0	0	0	0	0	0	0	0	0	0	0	0	0
0	0	0	71	304	0	3991	241	241	589	324	1904	0	4175	0	0
0	0	0	0	0	0	0	0	0	0	0	0	0	0	0	0
0	0	1291	0	0	0	0	0	0	0	314	0	0	0	0	0
0	0	0	0	0	0	0	0	0	0	0	0	0	0	0	0
0	0	0	0	0	0	0	0	0	0	0	0	0	0	0	0
0	0	0	0	0	0	0	0	0	0	0	0	0	0	0	0
0	0	0	0	0	0	0	0	0	0	0	0	0	0	0	0
0	41	0	0	0	0	0	109	0	4519	0	0	0	202	0	1340
0	0	0	0	0	0	0	0	0	0	0	0	0	0	0	0
0	0	0	0	0	0	0	0	0	0	0	0	0	0	0	0
0	0	0	0	0	0	0	0	0	0	0	0	0	0	0	0
0	0	0	0	0	0	206	0	0	1224	0	0	0	338	0	0
0	0	0	0	0	0	0	0	0	0	0	0	0	0	0	0
0	0	0	0	0	0	0	0	0	0	0	0	0	0	0	0
233	54	76675	77	0	134	159	257	232	8624	0	0	0	100	84	0
0	0	89	0	2202	0	49	0	0	0	0	0	0	0	0	0
0	0	0	0	0	0	0	0	0	0	0	0	0	0	0	0
78	48	1747	73	10830	1314	0	128	88289	14672	319	0	0	171	103	78
0	0	0	0	0	0	0	0	0	3570	0	0	0	66	0	0
0	0	0	0	0	0	0	0	0	0	0	0	0	0	0	0
0	0	0	0	0	0	0	0	0	0	0	0	0	0	0	0
0	0	0	0	0	0	0	0	0	0	0	0	0	0	0	0
0	0	0	0	0	0	0	0	0	0	0	0	0	0	0	0
0	0	0	0	0	0	0	0	0	0	0	0	0	0	0	0
0	0	0	0	0	0	0	0	0	0	0	0	0	0	0	0
0	22247	0	0	92	0	0	0	0	0	0	0	0	0	0	0
0	62	86	0	0	4213	0	0	0	0	0	0	0	0	0	0
0	0	0	0	0	0	0	0	0	0	0	0	0	0	0	0
0	0	0	0	0	0	0	0	0	0	0	0	0	0	0	0
0	11305	199	0	15264	0	0	0	0	42	0	46	0	0	0	0
0	0	0	0	0	0	0	0	0	0	0	0	0	0	0	0
0	0	0	0	0	0	0	0	0	0	0	0	0	0	0	0
0	0	0	0	0	0	0	0	0	0	0	0	0	0	0	0
0	0	0	0	0	0	0	0	0	0	0	0	0	0	0	0
0	0	0	0	0	0	0	0	0	0	0	0	0	0	0	0
0	0	0	0	0	0	0	0	0	0	0	0	0	0	0	0
0	0	0	0	0	0	0	0	0	0	0	0	0	0	0	0
0	0	0	0	0	0	0	0	0	0	0	0	0	0	0	0
634	2215	5327	3918	6344	283	5443	134	0	601	1296	984	1507	20864	0	45
0	0	0	0	0	0	0	0	0	0	0	0	0	0	0	0
0	0	0	0	0	0	0	0	0	0	0	0	0	0	0	0
0	0	0	0	0	0	0	0	0	0	0	0	0	0	0	0
73	45	1393	0	224	2792	0	0	226	0	0	0	0	0	243	683
0	0	0	0	0	0	0	0	0	0	0	0	0	0	0	0
0	0	0	0	0	0	0	0	0	0	0	0	0	0	0	0
0	0	0	0	0	0	0	0	0	0	0	0	0	0	0	0
79	0	0	0	0	0	0	132	428	0	0	0	0	0	0	0
0	0	0	0	0	0	0	0	0	0	0	0	0	0	0	0

0	0	0	0	0	0	0	0	0	0	0	0	0	0	0	0
0	0	0	0	0	0	0	0	0	0	0	0	0	0	0	0
0	0	0	0	0	0	0	0	0	0	0	0	0	0	0	0
0	0	0	0	0	0	0	0	0	0	0	0	0	0	0	0
2779	43	0	211	75	26123	0	0	0	1137	967	59	190	0	131	153961
0	0	0	0	0	0	0	0	0	0	0	0	0	0	0	0
67360	20112	36084	9198	167600	1473	222	672	0	512	2578	397	180	602	0	0
0	0	0	0	0	0	0	0	0	0	0	0	0	0	0	0
0	0	0	0	79	0	0	0	0	0	0	0	0	0	0	0
0	0	221	0	0	0	0	0	0	334	0	0	353	0	0	0
0	0	296	0	0	0	0	0	0	0	0	0	0	0	0	903
0	0	0	0	0	0	0	0	0	170	0	0	0	0	0	0
0	0	0	0	0	0	0	0	0	0	0	0	1008	0	0	0
948	0	0	0	0	0	0	0	0	0	0	0	0	0	0	0
0	0	0	0	0	0	0	0	0	0	0	0	0	0	0	0
0	0	0	0	0	0	0	0	0	0	0	0	0	0	0	0
84	882	4942	4206	8861	786	95225	1757	2692	23220	2940	21173	72	106234	221	2431
0	0	0	0	0	0	0	0	0	0	0	0	0	0	0	0
0	0	0	0	0	0	0	0	0	0	0	0	0	0	0	0
0	0	0	0	0	0	0	0	0	0	0	0	0	0	0	0
0	0	0	0	0	0	0	0	0	0	0	0	0	0	0	0
0	0	0	0	0	0	0	0	0	0	0	0	0	0	0	0
0	0	0	0	0	0	0	0	230	1600	0	0	0	0	0	0
3099	9735	892	4802	4951	133	0	0	0	49	0	0	0	83	0	0
0	0	0	0	0	0	0	0	0	0	0	0	0	0	0	0
0	0	0	0	0	0	0	0	0	0	0	0	0	0	0	0
148	57	197	31532	0	0	42	130	238	37483	335	173	0	87	0	46
0	0	0	0	0	0	0	0	0	0	0	0	0	0	0	0
0	0	0	0	0	0	0	0	0	0	0	0	0	0	0	0
0	0	0	0	0	1067	0	0	0	0	0	0	263	0	0	0
0	0	0	0	0	0	0	0	0	0	0	0	0	0	0	0
0	0	0	0	0	0	0	0	0	0	0	0	0	0	0	0
0	0	0	0	0	0	0	0	0	0	0	0	0	0	0	0
17008	146	93	0	170	0	0	0	0	0	0	0	0	0	0	0
0	0	0	0	0	0	0	0	0	0	0	0	0	0	0	0
0	0	1998	19971	72	929	1079	268	896	1700	331	503	50	1395	0	0
76	106	0	10543	0	2518	0	0	0	0	0	0	0	0	0	1787
0	0	0	0	0	0	0	0	0	0	0	0	0	0	0	0
0	0	0	0	0	0	0	0	0	0	0	0	0	0	0	0
0	0	0	0	0	125	0	0	0	0	0	0	0	0	0	0
0	0	0	0	0	0	0	0	0	0	0	0	0	0	0	0
0	0	0	0	0	0	0	0	0	0	0	0	0	0	0	0
0	0	0	0	0	0	0	0	0	0	0	0	0	0	0	0
0	0	0	0	0	0	0	0	0	0	0	0	0	0	0	0
0	53	1727	0	0	0	500	415	209	86175	651	229	0	2320	0	0
0	0	586	0	231	669	0	0	231	0	0	0	132	0	0	285
0	0	0	0	0	0	0	0	0	0	0	0	0	0	0	0
0	58	0	0	0	0	0	0	0	0	0	59	0	0	124	0
0	0	0	0	0	0	0	0	0	0	0	0	0	0	0	0
0	0	0	0	0	0	0	0	0	0	0	0	0	0	0	0
528	47	0	0	0	0	0	0	0	0	0	0	0	0	0	0
0	0	0	0	0	0	0	0	0	0	0	0	0	0	0	0
0	8930	0	0	80	136	0	0	0	0	0	0	0	0	0	0
0	0	0	0	0	0	0	0	0	0	0	0	0	0	0	0
0	0	0	0	0	0	0	0	0	0	0	0	0	0	0	144
0	0	0	0	0	0	0	0	0	0	0	0	0	0	0	0
0	0	0	2028	0	0	0	0	0	0	0	0	0	0	0	0
0	0	0	0	0	0	0	0	0	0	0	0	0	0	0	0

0	0	0	0	0	0	133	0	0	0	0	42	0	245	0	0
0	0	0	0	0	0	0	0	0	0	0	0	0	0	0	0
0	0	0	0	0	0	0	0	0	0	0	0	0	0	0	0
0	0	0	0	0	0	0	0	0	0	0	0	0	0	0	0
0	0	414	0	0	0	0	0	218	0	0	0	0	0	0	445
1911	0	0	0	0	0	0	0	0	0	0	0	0	0	0	0
0	0	0	0	0	0	0	0	0	0	0	0	0	0	0	0
78	47	0	5351	12562	1053	0	0	237	1747	0	57	0	79	110	0
0	0	0	0	0	0	0	0	0	0	0	0	0	0	0	0
0	0	0	0	0	0	0	0	10888	0	0	0	0	0	0	0
0	0	0	0	0	0	0	0	0	0	0	0	0	0	0	0
0	0	0	0	0	0	0	0	0	0	0	0	0	0	0	0
0	0	0	0	0	0	0	0	0	0	0	0	0	0	0	0
0	0	0	0	0	0	0	0	0	0	0	0	0	0	0	0
0	0	0	0	0	0	0	0	0	0	318	0	0	0	0	0
0	0	0	0	0	0	45	0	225	10499	0	59	0	363	0	0
84	0	0	144	6544	0	156	0	0	1101	0	0	222	0	0	0
0	0	0	0	0	0	0	0	0	3024	0	0	0	0	0	0
0	0	0	0	0	0	0	0	0	0	0	0	0	0	0	0
0	0	0	0	0	0	0	0	0	0	0	0	0	0	0	0
0	0	0	0	0	0	0	0	0	5881	0	0	0	0	0	0
0	0	0	0	0	0	0	0	0	0	0	0	0	0	0	0
0	0	0	0	0	0	97	0	0	43	0	118	0	241	0	0
0	0	0	0	0	0	0	0	0	0	0	0	0	0	0	0
0	0	0	0	0	0	0	0	0	0	0	0	0	0	0	0
7192	5821	8625	17992	14626	13164	17643	282540	7617	204086	10590	5768	716231	25536	947205	76301
0	0	0	0	0	0	0	0	0	0	0	0	0	0	0	0
0	0	0	0	0	0	0	0	255	2580	0	0	0	0	0	0
0	0	96	0	0	140	0	372	0	843	0	64	709	177	0	1199
0	0	0	0	0	0	0	0	0	0	0	0	0	0	0	0
0	0	0	0	0	0	0	0	0	0	0	0	0	0	0	0
0	0	0	0	0	495	0	0	0	145	22752	0	1070	0	0	2167
0	0	0	0	0	0	0	0	0	0	0	0	0	0	0	0

COVID-19-029	COVID-19-030	COVID-19-031	COVID-19-032	COVID-19-035	COVID-19-037	COVID-19-038	COVID-19-040	COVID-19-041	COVID-19-043	COVID-19-045	COVID-19-046	COVID-19-047	COVID-19-048	COVID-19-049	COVID-19-050
0	17751	0	0	0	0	0	0	0	0	0	0	0	0	0	0
0	0	0	0	0	0	0	0	0	0	0	0	0	0	0	0
0	0	0	0	0	0	0	0	0	0	0	0	0	0	0	0
0	0	0	0	0	0	0	0	0	0	0	0	0	0	0	0
0	0	0	0	0	0	362	0	0	0	0	58	0	169	0	0
0	0	0	0	0	0	0	0	0	0	0	0	0	0	0	0
0	0	0	0	0	0	0	0	0	0	0	0	0	0	0	0
14778	29022	46321	182507	118347	129740	799978	18582	49187	149881	33058	247821	2448	750002	4204	539138
0	0	0	0	3952	0	0	0	0	0	0	0	0	0	0	0
0	0	0	0	0	0	0	0	0	0	0	0	0	0	0	0
0	0	83	8483	0	0	0	0	0	0	0	0	0	0	0	0
0	0	0	0	0	0	0	0	0	0	0	0	0	0	0	0
0	0	0	0	0	0	48	0	0	0	0	1371	914	165	0	0
0	0	0	0	0	0	0	0	0	2716	0	0	0	0	0	0
76	96	17505	13231	93	3512	1399	256	0	22549	313	222	0	542	0	5093
0	0	0	0	0	0	0	0	0	0	0	0	0	0	0	0
0	0	0	0	0	0	0	0	0	0	0	0	247	0	0	0
0	0	0	0	0	0	0	0	0	0	0	0	0	0	0	0
178	48	88	266	174	795	1986	41470	441	3919	1305	1158	130346	4703	466	810
0	87	0	80	0	0	580	0	0	276	303	287	0	973	0	0
0	0	0	0	0	0	0	0	0	0	0	0	0	0	0	0
0	0	0	0	0	0	0	0	0	0	0	0	0	0	0	0
0	0	0	0	0	0	0	0	0	58	0	0	0	0	0	0

319	0	0	0	0	0	0	0	0	92	0	0	0	0	0	0
0	0	0	0	0	0	0	0	0	0	0	0	321	0	0	0
0	0	0	0	0	0	0	0	0	0	0	0	0	0	0	0
0	219	96	0	0	0	65	0	0	283	0	0	63	0	0	0
0	0	0	0	0	0	0	0	0	0	0	0	0	101	0	0
3759	575	1245	260	2104	3019	54	0	0	0	0	0	0	92	113	202
0	0	0	0	0	0	0	0	0	0	0	0	0	0	0	0
0	0	0	0	0	0	0	0	0	0	0	0	0	0	0	0
0	0	0	0	0	0	0	0	33322	0	331	0	0	0	0	0
97	50	0	73	69	4333	0	0	0	761	0	0	0	0	0	566
71	0	112	0	0	273	0	0	0	0	0	0	0	0	0	0
0	0	0	0	0	0	0	0	0	381	0	0	0	0	0	0
0	0	0	0	0	0	0	0	0	0	0	0	0	0	0	0
0	95	0	0	0	0	0	0	0	0	0	0	62	0	0	0
0	0	0	0	0	0	0	0	0	0	0	0	0	0	0	0
0	0	678	73	0	0	0	0	0	0	0	0	0	0	0	781
0	3331	0	0	0	0	0	0	0	0	0	0	0	0	0	71
0	0	0	0	0	0	0	0	0	0	0	0	0	0	0	79
0	9716	0	0	0	0	0	0	0	0	0	0	0	0	0	0
0	0	0	0	0	0	0	0	0	0	0	0	0	0	0	0
0	10652	0	1945	2444	765	0	0	18948	3281	0	0	736	0	0	0
0	0	0	0	0	0	0	0	0	0	0	0	0	0	0	54
405	0	0	13812	1282	0	0	0	0	276	0	0	0	0	0	0
30088	28064	58581	2469	53949	96511	2686	1673	3616	4778	630959	1509	91413	3367	592	51040
23387	556	88	0	309	139	43	0	0	0	0	0	0	0	0	68
0	39	0	8196	97	0	0	0	0	0	0	0	0	0	0	0
3674	21226	406	0	4548	125	52	139	206	51	339	0	834	0	0	439
0	0	0	0	0	0	0	0	0	476	0	0	0	0	0	1985
0	0	0	0	0	0	0	0	0	0	0	0	0	0	0	0
0	0	0	0	0	0	0	0	0	0	0	0	0	0	0	0
0	0	0	0	0	0	0	0	0	0	0	0	0	0	0	0
66	0	4847	0	0	0	0	0	90870	0	0	55	0	0	0	0
0	0	0	0	0	0	0	0	0	0	0	0	0	0	0	0
0	0	0	0	0	0	0	0	0	0	0	0	0	0	0	0
0	0	0	0	0	0	0	0	0	0	0	0	0	0	0	0
0	0	0	0	0	0	0	0	0	0	0	0	0	0	0	0
0	0	0	0	0	0	0	0	0	0	0	0	0	0	0	0
0	0	0	0	0	0	63	0	0	0	0	0	0	91	0	0
0	0	0	0	0	0	0	0	0	0	0	0	0	0	0	0
0	0	0	0	0	122	0	0	0	0	0	0	0	0	0	0
0	0	0	0	0	0	0	0	0	0	0	0	0	0	0	0
0	0	0	0	0	0	0	0	0	0	0	273	0	0	0	0
0	0	0	0	0	0	0	0	0	0	0	0	0	0	0	0
0	0	0	0	0	0	0	0	0	0	0	0	0	0	0	0
0	9498	0	0	0	0	0	0	0	0	0	0	0	0	120	282
0	0	0	0	0	0	0	0	0	0	0	0	0	0	0	0
0	0	0	0	0	0	0	0	0	0	0	0	0	0	0	74
0	0	0	0	0	0	0	0	0	0	0	0	0	0	0	0
57085	1879	259705	3149	55740	251794	436	135	457	209	3257	105	120	361	344	41784
0	0	0	0	0	0	0	0	0	0	0	0	0	0	0	0
0	0	0	0	0	0	0	0	0	5200	0	0	0	85	0	0
0	0	0	0	0	0	0	0	0	0	0	0	0	0	0	0
78	0	1700	0	101	15994	0	0	0	0	0	0	0	0	0	0
0	0	0	0	0	0	0	0	0	0	0	0	0	0	0	0
0	0	0	0	0	0	0	0	0	0	0	0	0	0	0	0
0	0	0	0	0	0	0	0	0	0	0	0	0	0	0	0
0	0	0	0	0	0	0	0	470	19851	0	0	0	80	0	0
0	0	621	0	0	0	0	0	0	0	0	0	0	0	0	0

0	0	0	0	657	0	1234	0	0	0	0	704	0	1121	0	0
0	0	0	0	0	0	0	0	0	0	0	0	0	0	0	0
0	0	0	0	0	0	0	0	0	0	0	0	0	0	0	0
11280	254	0	333	0	0	2142	642605	6336	2617	10978	695540	1624	1953	11722	7373
0	0	0	0	0	0	0	0	0	0	0	0	0	0	0	0
0	82	29811	0	154	0	0	0	0	0	0	0	0	0	0	0
0	0	0	0	0	0	0	0	0	0	0	0	0	0	0	0
0	0	0	0	0	0	0	0	0	0	0	0	0	0	0	0
0	0	0	0	0	0	0	0	0	0	0	0	0	0	0	0
0	0	0	0	0	0	0	0	0	47	0	53	0	0	0	24111
0	0	0	0	0	0	0	0	0	0	0	0	0	0	119	0
0	0	0	0	0	0	0	0	0	0	0	0	0	0	0	0
19476	0	0	0	73	0	0	0	0	0	0	0	0	0	0	0
328	279	292	214	136	198580	0	138	0	3998	0	53	74	0	0	5587
0	0	0	0	0	1491	0	0	0	47	0	0	0	0	0	0
0	0	0	0	0	10015	0	0	0	249	0	0	0	0	0	124
0	0	0	13989	0	273	0	0	0	0	0	0	0	0	0	0
0	0	0	0	0	0	0	0	0	0	0	0	0	0	0	0
0	0	0	0	0	0	0	0	0	0	0	0	0	0	0	0
0	0	0	0	0	0	0	0	0	1436	0	0	0	0	0	0
0	0	0	0	0	0	0	0	0	0	0	0	0	0	0	0
0	0	0	0	0	0	0	0	0	0	0	0	0	0	0	0
0	0	0	0	0	0	0	0	0	0	0	0	0	0	0	0
0	0	0	0	0	0	0	0	0	0	0	0	0	0	0	0
0	0	0	0	0	0	53	0	0	0	0	0	0	0	0	0
0	0	0	0	0	0	0	0	0	0	0	0	0	0	0	0
0	0	0	0	0	0	0	0	0	0	0	0	0	0	0	0
0	0	0	0	0	0	210	0	0	0	0	55	0	0	0	0
0	0	0	0	0	0	0	0	0	0	0	0	0	0	0	0
0	0	0	0	0	0	0	0	0	0	0	0	0	0	0	0
0	0	0	0	0	0	0	0	0	0	0	0	0	0	0	0
578689	560712	49121	306221	241711	7491	439	1058	2896	567	2163	320	0	465	0	280
0	0	0	0	0	0	0	0	0	0	0	0	0	0	0	0
0	0	0	0	0	0	0	0	0	0	0	0	0	0	0	0
0	0	0	0	0	0	0	0	0	0	0	0	0	0	0	0
79	0	13513	0	1433	14539	539	624	26815	48753	339	50	816	452	217	195
0	0	0	0	0	0	0	0	0	0	0	0	0	0	0	0
3698	152	5293	0	35869	20282	96	261	222	193	317	97	3940	185	366	1099
0	96	23634	74	5795	2085	241	220	62311	88595	1288	163	19194	168	0	417
0	38	11936	0	2799	30204	239	0	217459	5829	915	108	184	368	0	0
89	35	2396	16722	0	0	0	0	0	0	0	0	0	0	0	0
0	0	0	0	0	0	0	0	0	0	0	0	0	0	116	0
0	0	0	0	0	0	0	0	0	0	0	0	0	0	0	133
0	0	0	0	0	0	0	0	0	0	0	0	0	0	0	0
0	0	0	0	0	0	0	0	0	0	0	0	0	0	0	0
0	0	0	0	0	0	0	0	0	0	0	0	0	0	0	0
0	0	0	0	0	0	0	0	0	0	0	0	0	0	0	0
0	0	0	0	0	0	0	0	0	0	0	0	0	0	0	0
0	0	0	0	0	0	0	0	0	0	0	0	678	0	0	0
0	0	6464	0	0	0	0	0	0	0	0	0	0	80	0	0
0	0	0	0	0	0	0	0	0	0	0	0	0	0	0	0
0	0	0	0	0	0	50	121	85658	0	0	65	0	0	0	0
0	0	0	0	0	0	0	0	0	0	0	0	0	0	0	0
0	0	0	0	0	0	0	0	0	0	0	0	0	0	0	0
0	0	0	0	0	0	0	0	0	0	0	0	0	0	0	0
0	0	0	0	0	0	0	0	0	0	0	0	0	0	0	0
0	0	0	0	0	0	0	0	0	0	0	0	0	0	0	0
0	0	0	0	0	0	0	0	0	0	0	0	0	0	0	0

0	0	0	0	0	0	0	0	0	0	0	0	0	0	0	0
0	73	0	0	87	0	0	139	0	0	0	0	0	0	0	57
0	0	0	0	0	0	161	0	0	0	0	0	0	258	0	0
0	0	0	0	0	0	0	0	0	0	0	0	0	0	0	0
0	0	0	0	0	0	0	0	0	0	0	0	0	0	0	0
0	0	0	0	0	0	0	0	0	0	0	0	0	0	0	284
0	0	0	0	0	0	0	0	0	0	0	0	0	0	0	0
0	0	0	69	4960	0	0	0	0	0	0	0	0	0	0	0
0	0	0	0	0	0	0	0	0	0	0	0	0	0	0	0
0	0	0	0	0	0	0	0	0	0	0	0	0	0	0	0
0	0	0	0	0	0	0	0	0	0	0	0	0	0	0	0
0	0	0	0	0	0	0	0	0	0	0	0	0	0	0	0
0	0	0	0	0	0	0	0	0	0	0	0	0	0	0	0
0	0	0	0	0	0	0	0	0	0	0	0	0	0	0	0
0	0	91	0	0	0	0	0	0	3967	0	64	0	0	0	69
0	0	0	0	0	0	0	0	0	0	0	0	0	0	0	0
0	0	0	0	0	0	0	0	0	0	0	0	0	0	0	0
0	0	0	0	0	0	0	0	0	0	0	0	0	0	0	0
0	0	0	280	0	650	0	0	0	0	0	0	0	0	0	0
0	0	0	0	0	0	0	0	0	0	0	0	0	0	0	136
0	0	0	79	0	0	0	0	0	0	0	0	0	0	0	0
0	0	0	0	0	0	0	0	0	0	0	0	0	0	0	0
0	305	0	72	38184	118	0	0	0	0	0	0	0	0	0	0
0	0	0	0	0	0	0	0	0	0	0	0	0	0	0	0
0	0	0	0	0	0	0	0	0	0	0	0	0	0	0	0
0	0	0	0	0	0	0	0	0	0	0	0	0	0	0	0
0	0	0	0	0	0	0	0	0	0	0	0	0	0	0	0
0	0	0	0	0	0	0	0	0	0	0	0	0	0	0	0
0	0	0	0	0	0	0	0	0	0	0	0	0	0	0	0
0	0	0	0	0	0	0	0	0	0	0	0	0	0	0	0
0	0	0	0	0	0	0	0	0	0	0	0	0	0	0	0
0	0	0	0	0	0	0	0	0	0	0	0	0	0	0	0
0	0	554	0	0	0	0	0	0	0	0	0	0	0	0	0
0	0	0	0	0	0	0	0	0	0	0	0	0	0	0	0
0	49	120	61	14722	0	0	0	0	0	0	0	0	0	0	0
0	0	0	0	0	0	0	0	0	0	0	0	0	0	0	0
0	0	0	0	0	0	0	0	224	5873	611	0	0	0	0	0
0	0	0	0	0	0	0	0	0	0	0	0	0	0	0	0
0	0	0	0	0	0	0	0	0	0	0	0	0	0	0	0
84	19737	103	61	0	0	0	0	0	0	0	0	0	0	0	0
0	0	0	0	0	0	0	0	0	0	0	0	0	0	0	0
0	0	0	0	0	0	0	0	0	0	0	0	0	0	0	0
82	143	914	0	0	19827	742	0	0	9853	2576	226	815	2267	211	60891
0	0	0	0	0	0	0	0	0	0	0	0	0	0	0	0
0	0	0	0	0	0	0	0	0	0	0	0	0	0	0	0
0	0	0	0	0	0	0	0	0	0	0	0	0	0	0	0
0	0	0	0	0	0	0	0	0	0	0	0	0	0	0	0
0	0	0	0	0	0	0	0	0	0	0	0	0	0	0	0
0	0	0	0	0	0	0	0	0	0	0	0	0	0	0	0
0	0	0	0	0	0	222	0	0	0	0	0	0	78	0	0
0	0	0	0	0	0	0	0	0	0	0	0	0	0	0	0
0	643	0	0	0	0	0	0	0	0	0	0	0	0	0	0
0	0	0	0	0	0	0	0	0	0	0	0	0	0	0	0
0	0	0	0	0	0	108	0	0	0	0	175	0	246	0	0
0	0	0	0	0	0	45	0	0	0	0	0	0	151	0	0
0	0	0	0	0	0	0	0	0	0	0	0	0	0	0	0
0	0	0	0	0	0	0	0	0	0	0	0	0	0	0	0
0	0	0	0	0	0	0	0	0	0	0	0	0	0	0	0

0	45	15003	0	0	0	0	0	0	0	0	0	0	0	0	61
0	0	0	0	0	0	0	0	0	0	0	0	0	0	0	0
0	0	0	0	0	0	0	0	0	0	0	0	0	0	0	0
0	0	0	0	0	0	0	0	0	0	0	0	0	0	0	0
0	0	0	0	0	0	0	0	0	0	0	0	0	0	0	0
0	0	0	0	0	0	0	0	0	0	0	0	0	0	0	0
144	0	400	6100	9925	122	17961	788	0	9709	965	4442	0	24124	0	261
102448	126	1031	11636	145	23923	0	0	0	13466	310	0	0	0	0	0
0	0	0	0	0	0	55	135	0	0	0	0	0	0	0	0
0	0	0	0	0	0	0	0	0	0	0	0	0	0	0	0
0	0	0	0	0	0	0	0	0	0	0	55	65	0	0	0
0	0	0	0	0	0	0	0	0	0	0	0	0	0	0	0
0	0	0	0	0	0	0	0	0	0	0	0	0	0	0	0
0	0	0	0	0	0	0	0	0	0	0	0	0	0	0	0
9641	21944	5116	42999	8290	5948	30751	1684	6747	28917	263037	8736	13925	31074	31254	7398
0	45	0	7988	2435	121	0	0	0	0	0	0	0	0	0	0
80	98	3566	63	172	0	0	0	0	0	0	0	0	0	0	1603
0	0	0	0	0	0	0	0	0	0	0	0	0	0	0	0
0	0	0	0	0	0	0	0	0	0	0	0	0	0	0	0
81	110	7439	74	0	276	0	0	0	47	0	223	0	90	0	489
0	57	0	0	220	0	0	0	0	0	0	0	0	0	0	0
489	0	1252	0	675	2666	0	0	0	0	0	0	0	0	129	551
0	0	0	0	0	0	0	0	0	0	0	0	0	0	0	0
0	0	1856	0	0	0	0	0	0	0	0	0	0	0	477	0
0	0	0	0	0	0	0	0	0	0	0	0	0	0	0	0
0	0	0	0	0	0	0	0	0	263	0	0	0	0	0	0
0	0	101	0	0	11562	0	0	0	0	0	0	0	0	0	0
15409	53659	4068	488	132916	686	99	110	213	2576	1302	149	1282	257	0	0
0	0	206	142	227	0	341	0	0	1376	0	52	0	653	0	0
0	0	0	0	0	0	0	0	0	0	0	0	0	0	0	0
83	43	116	33863	74	395	0	0	0	0	0	0	0	0	0	138
0	0	0	0	0	0	0	0	0	0	0	0	0	0	0	0
85	47	0	17870	0	127	0	0	0	0	0	0	0	0	0	0
0	0	0	0	0	241	0	0	0	0	0	0	0	0	0	0
0	0	0	0	0	0	0	0	0	0	0	0	0	0	0	0
0	0	0	0	0	0	0	0	0	0	0	0	0	0	0	0
0	0	0	0	0	0	0	0	0	0	0	0	0	0	0	0
0	0	0	0	0	4642	0	0	0	0	0	0	0	0	0	0
0	0	0	0	0	0	0	0	0	7093	0	0	0	0	0	0
0	0	0	0	0	0	0	0	0	0	0	0	0	0	0	0
315	384	372	128784	249	132	0	0	0	0	0	0	0	0	0	0
0	0	0	0	0	0	55	131	223	0	0	0	0	99	0	0
0	2739	505	0	0	0	0	0	0	0	0	0	0	0	0	0
0	0	0	0	0	0	0	0	0	0	0	0	0	0	0	256
0	0	117	0	0	0	1626	525	209	1376	0	624	2297	1897	114	0
0	0	0	0	0	0	0	0	0	0	0	0	0	0	0	0
0	0	0	0	0	0	0	0	0	0	0	0	0	0	0	0
171	76093	201	130	218	129	0	0	0	0	0	0	0	0	0	0
0	0	0	0	0	0	0	0	0	0	0	0	0	0	0	0
0	0	0	0	0	0	0	130	199	7847	0	57	0	0	0	0
0	0	0	0	0	0	0	0	0	0	0	0	0	0	0	0
0	0	0	0	0	0	0	0	0	0	0	0	0	0	0	0
0	0	0	0	0	0	0	0	0	0	0	0	0	0	0	0
0	0	0	0	0	0	0	0	0	0	0	0	0	0	0	0
67	180	226	0	142	250	517	649	0	1147	0	0	3087	262	0	84
0	0	0	0	0	0	0	0	0	0	0	0	0	0	0	0
0	0	0	0	0	0	0	0	0	0	0	0	0	0	0	0
0	0	0	0	0	0	107	0	117809	163	0	0	0	0	0	0

0	47	0	71	0	137	0	0	0	642	0	0	0	0	0	0
0	0	0	0	0	0	0	0	0	0	0	0	0	0	0	0
0	0	0	0	0	0	0	0	0	0	0	0	58	0	0	0
0	0	0	0	0	0	0	0	0	0	0	0	0	0	0	0
0	0	0	0	0	0	263	0	0	0	0	59	0	199	0	60
78	0	14284	0	79	141	0	0	0	0	0	0	0	0	0	0
0	0	0	0	0	0	0	0	0	0	0	0	0	0	0	0
0	0	0	0	0	0	0	0	0	368	0	0	0	0	0	0
0	0	0	0	0	0	0	0	0	0	0	0	0	0	0	0
0	0	0	0	0	0	0	0	0	0	0	0	0	0	0	0
0	0	0	0	0	0	0	0	0	0	0	0	0	0	0	0
0	0	0	0	0	1997	0	0	0	0	0	0	0	0	0	0
0	0	0	0	0	0	0	0	0	0	0	0	0	0	0	0
0	0	0	0	0	0	0	0	0	0	0	0	0	0	0	0
0	0	0	0	0	3089	0	0	0	0	0	0	0	0	122	0
0	0	0	0	0	0	0	0	0	0	0	0	0	0	0	0
0	0	0	0	0	0	0	0	0	0	0	0	0	0	0	0
0	0	0	0	0	0	0	0	0	0	0	0	0	0	0	0
0	0	0	0	0	0	0	0	0	0	0	0	0	0	0	0
0	0	0	0	0	0	0	0	0	0	0	0	0	0	0	0
0	0	0	0	0	0	0	0	0	0	0	0	0	0	0	0
0	0	0	0	0	0	0	0	0	0	0	0	0	0	0	0
0	0	1775	0	0	0	0	0	0	0	0	0	0	0	0	0
0	37	0	0	0	0	0	249	0	93	0	0	0	0	0	0
0	59	0	0	0	0	0	0	0	0	0	0	0	0	0	0
0	0	0	0	0	0	614	651	646	111405	973	240	0	363	0	0
0	0	0	0	0	0	0	0	0	0	0	0	0	0	0	0
0	0	0	0	0	0	0	0	0	0	0	0	0	0	0	0
0	0	0	0	0	147	0	0	0	0	0	0	0	0	0	0
0	0	0	0	0	0	0	0	0	0	0	0	0	0	0	0
0	0	0	0	0	0	0	0	0	0	0	0	0	0	0	0
69	0	25860	0	81	0	0	0	23247	0	0	0	0	84	211	0
0	0	0	0	0	0	0	0	0	0	0	0	136	0	0	0
0	0	0	0	0	0	0	0	0	103	0	0	0	0	0	0
0	0	0	0	0	0	0	0	0	0	0	0	0	0	0	0
239	886	164974	2216	401	103	0	0	0	332	0	0	0	80	102	0
0	0	0	0	0	0	0	0	0	0	0	0	0	0	0	0
10152	43266	59089	15464	206	42899	46	0	49151	1874	326	52	1899	76	780	1244
0	0	0	0	0	133	0	0	0	0	0	0	0	0	0	0
0	0	0	0	0	0	0	0	0	0	0	0	0	0	0	0
0	0	0	0	0	0	0	0	0	0	0	0	0	0	0	0
0	0	0	0	0	0	0	0	0	0	0	0	0	0	0	0
75	0	0	0	0	0	0	0	0	0	0	0	0	0	0	0
0	0	0	0	0	0	0	0	0	0	0	0	0	0	0	0
0	0	0	0	0	0	0	0	0	0	0	0	0	0	0	0
0	49	0	0	1239	388	0	0	0	0	0	0	0	84	0	0
0	2098	0	0	0	0	0	119	0	395	0	0	0	0	0	0
0	0	0	0	0	0	462	0	0	91	0	283	0	843	0	0
0	0	0	0	0	0	0	0	0	604	0	0	0	0	0	0
0	0	0	0	0	0	0	0	0	0	0	0	0	0	0	0
0	0	0	0	0	0	0	0	0	0	0	0	0	0	0	0
0	0	0	0	0	0	0	0	0	0	0	0	0	0	0	0
0	0	522	0	0	0	0	0	229	1278	0	0	371	0	0	375
0	0	0	0	0	0	0	0	0	0	0	0	0	0	0	0
0	0	0	0	0	0	0	0	0	0	0	0	0	0	0	0
0	0	0	0	0	0	0	0	0	0	0	0	0	0	0	0
169	53	5386	0	0	2618	0	0	0	0	0	0	0	0	0	769
0	0	0	0	0	0	0	0	0	0	0	0	0	0	0	0

0	0	0	0	0	0	0	0	0	0	0	0	0	0	0	0
0	0	0	0	0	0	0	0	0	0	0	0	0	0	0	0
0	0	0	0	0	0	0	0	0	0	0	0	0	0	0	0
0	0	0	0	0	0	0	0	0	0	0	65	0	0	0	0
9630	0	0	0	0	113	0	0	0	0	0	0	0	0	0	0
0	0	0	0	0	0	0	0	0	0	0	0	0	0	0	0
0	0	0	0	0	0	0	0	0	0	0	0	0	0	0	0
0	0	0	0	0	0	0	0	0	0	0	0	0	0	0	0
0	0	0	0	0	0	54	0	0	0	0	0	0	0	0	0
0	0	0	0	0	0	0	0	0	0	0	0	0	0	0	0
0	0	0	0	0	0	0	0	0	0	0	0	0	0	0	113
0	0	0	0	0	0	0	0	0	0	0	0	0	0	0	0
0	0	0	0	77	25467	57	0	0	2530	0	0	0	0	0	198
0	0	0	0	0	0	0	0	0	0	0	0	0	0	0	0
0	0	6645	0	0	138	0	0	0	0	0	0	0	0	0	79
0	0	0	0	0	0	61	0	0	0	0	0	0	0	0	0
0	0	0	0	0	0	0	0	0	0	0	0	0	0	0	0
0	0	0	0	0	0	0	0	0	0	0	0	0	0	0	0
73	0	0	0	0	0	0	0	0	0	0	0	0	0	0	786
0	0	0	0	0	0	0	0	0	0	0	0	0	0	0	164
0	0	0	0	0	0	0	0	0	0	0	0	0	0	0	0
0	0	0	0	81	0	557	0	0	0	0	205	0	270	0	0
0	0	0	0	0	0	0	0	0	0	0	0	0	0	0	152
0	0	0	0	0	0	0	0	0	0	0	0	0	0	0	0
0	0	0	0	0	0	0	0	0	0	0	0	0	0	0	0
0	0	0	0	0	0	0	0	0	0	0	0	0	0	0	0
158	192	0	1619	888	121	5413	253	437	920	0	2149	0	5263	0	0
0	0	0	0	0	0	0	0	0	0	0	0	0	0	0	0
0	0	0	0	0	0	0	0	0	0	0	0	0	0	0	0
0	0	0	0	0	0	0	0	0	0	0	0	0	0	0	0
0	0	0	0	0	0	0	0	0	0	0	0	0	0	0	0
0	0	594	0	0	786	0	0	0	0	0	0	0	0	0	112
0	0	0	0	0	0	0	0	0	0	0	0	0	0	0	0
0	0	0	0	0	0	0	0	0	0	0	0	0	0	0	0
0	0	0	0	0	0	43	0	0	0	0	0	0	0	0	0
0	0	0	7847	0	0	0	0	0	0	0	0	0	0	0	0
0	0	0	133	0	0	0	0	0	0	0	0	0	0	0	0
0	48	0	27742	75	0	0	0	0	0	0	0	0	0	0	0
0	0	0	0	0	0	0	0	0	1583	0	0	0	0	0	0
0	0	0	0	0	0	0	0	0	0	0	0	0	0	0	0
0	0	0	0	0	0	0	0	0	0	0	0	0	0	0	0
0	0	0	0	0	141	0	0	0	0	0	0	0	0	0	0
86	98	0	11355	0	138	0	0	0	394	0	0	64	0	0	0
0	0	0	0	0	0	0	0	0	0	0	0	0	0	0	0
0	0	0	0	0	0	0	0	0	0	0	0	0	0	0	0
0	0	0	0	0	0	0	0	0	0	0	0	0	0	0	0
0	0	0	0	0	0	0	0	0	0	0	0	0	0	0	0
0	0	0	0	0	0	0	0	0	0	0	0	0	0	0	0
0	0	0	0	0	0	0	0	0	0	0	0	0	0	0	0
0	0	0	0	0	0	0	0	0	0	0	0	0	0	0	0
0	0	0	0	62	0	0	0	0	0	0	0	0	0	0	0
0	0	0	0	0	0	0	0	0	0	0	0	0	0	0	70
0	0	0	0	0	0	0	0	0	0	0	0	0	0	0	0
0	0	0	0	0	0	54	0	87876	74	320	0	0	0	0	0
0	0	0	0	0	0	0	0	0	0	0	0	0	0	0	0
0	0	0	0	0	0	0	0	0	0	0	0	0	0	0	0
0	0	0	0	0	0	0	0	0	0	0	0	0	0	0	0
0	0	0	71	304	0	3991	241	241	589	324	1904	0	4175	0	0

0	0	0	0	0	0	0	0	0	0	0	0	0	0	0	0
0	0	0	0	0	0	0	0	0	0	0	0	0	0	0	0
0	0	0	0	0	0	0	0	0	0	0	0	0	0	0	0
0	0	0	0	0	0	0	0	0	0	0	0	0	0	0	0
0	0	0	0	0	0	0	0	0	0	0	0	0	0	0	0
0	0	1291	0	0	0	0	0	0	0	314	0	0	0	0	0
0	0	0	0	0	0	0	0	0	0	0	0	0	0	0	0
0	0	0	0	0	0	0	0	0	0	0	0	0	0	0	0
0	0	0	0	0	0	0	0	0	0	0	0	0	0	0	0
0	0	0	0	0	0	0	0	0	0	0	0	0	0	0	0
0	41	0	0	0	0	0	0	0	4519	0	0	0	0	0	0
0	0	0	0	0	0	0	0	0	0	0	0	0	0	0	0
0	0	0	0	0	0	0	109	0	0	0	0	0	202	0	1340
0	0	0	0	0	0	0	0	0	0	0	0	0	0	0	0
0	0	0	0	0	0	0	0	0	0	0	0	0	0	0	0
0	0	0	0	0	0	0	0	0	0	0	0	0	0	0	0
0	0	0	0	0	0	0	0	0	0	0	0	0	0	0	0
0	0	0	0	0	0	206	0	0	1224	0	0	0	338	0	0
0	0	0	0	0	0	0	0	0	0	0	0	0	0	0	0
0	0	0	0	0	0	0	0	0	0	0	0	0	0	0	0
0	0	0	0	0	0	0	0	0	0	0	0	0	0	0	0
0	0	0	0	0	0	0	0	0	0	0	0	0	0	84	0
82	0	9417	0	0	0	159	257	232	8589	0	0	0	100	0	0
0	0	0	0	0	0	0	0	0	0	0	0	0	0	0	0
0	0	89	0	2202	0	49	0	0	0	0	0	0	0	0	0
0	0	0	0	0	0	0	0	0	0	0	0	0	0	0	0
0	0	0	0	0	0	0	0	0	0	0	0	0	0	0	0
0	0	0	0	0	0	0	0	0	0	0	0	0	0	0	0
78	48	1747	73	10830	1314	0	128	88289	14672	319	0	0	171	103	78
0	0	0	0	0	0	0	0	0	3570	0	0	0	66	0	0
0	0	0	0	0	0	0	0	0	0	0	0	0	0	0	0
0	0	0	0	0	0	0	0	0	0	0	0	0	0	0	0
0	0	0	0	0	0	0	0	0	0	0	0	0	0	0	0
0	0	0	0	0	0	0	0	0	0	0	0	0	0	0	0
0	0	0	0	0	0	0	0	0	0	0	0	0	0	0	0
0	0	0	0	0	0	0	0	0	0	0	0	0	0	0	0
0	0	0	0	0	0	0	0	0	0	0	0	0	0	0	0
0	22247	0	0	92	0	0	0	0	0	0	0	0	0	0	0
0	62	86	0	0	4213	0	0	0	0	0	0	0	0	0	0
0	0	0	0	0	0	0	0	0	0	0	0	0	0	0	0
0	0	0	0	0	0	0	0	0	0	0	0	0	0	0	0
0	11305	199	0	15264	0	0	0	0	42	0	46	0	0	0	0
0	0	0	0	0	0	0	0	0	0	0	0	0	0	0	0
0	0	0	0	0	0	0	0	0	0	0	0	0	0	0	0
0	0	0	0	0	0	0	0	0	0	0	0	0	0	0	0
0	0	0	0	0	0	0	0	0	0	0	0	0	0	0	0
0	0	0	0	0	0	0	0	0	0	0	0	0	0	0	0
0	0	0	0	0	0	0	0	0	0	0	0	0	0	0	0
0	0	0	0	0	0	0	0	0	0	0	0	0	0	0	0
0	0	0	0	0	0	0	0	0	0	0	0	0	0	0	0
0	0	0	0	0	0	0	0	0	0	0	0	0	0	0	0
0	0	0	0	0	0	0	0	0	0	0	0	0	0	0	0
634	2215	5327	3918	6344	283	5443	134	0	601	1296	984	1507	20864	0	45
0	0	0	0	0	0	0	0	0	0	0	0	0	0	0	0
0	0	0	0	0	0	0	0	0	0	0	0	0	0	0	0
0	0	0	0	0	0	0	0	0	0	0	0	0	0	0	0
73	45	1393	0	224	2792	0	0	226	0	0	0	0	0	243	683
0	0	0	0	0	0	0	0	0	0	0	0	0	0	0	0

0	0	0	0	0	0	0	0	0	0	0	0	0	0	0	0
0	0	0	0	0	0	0	0	0	0	0	0	0	0	0	0
0	0	0	0	0	0	0	0	0	0	0	0	0	0	0	0
0	0	0	0	0	0	0	0	0	0	0	0	0	0	0	0
0	0	0	0	0	0	0	0	0	0	0	0	0	0	0	0
79	0	0	0	0	0	0	132	428	0	0	0	0	0	0	0
0	0	0	0	0	0	0	0	0	0	0	0	0	0	0	0
0	0	0	0	0	0	0	0	0	0	0	0	0	0	0	0
0	0	0	0	0	0	0	0	0	0	0	0	0	0	0	0
0	0	0	0	0	0	0	0	0	0	0	0	0	0	0	0
0	0	0	0	0	0	0	0	0	0	0	0	0	0	0	0
0	0	0	0	0	0	0	0	0	0	0	0	0	0	0	0
2779	43	0	211	75	26123	0	0	0	1137	967	59	190	0	131	153961
0	0	0	0	0	0	0	0	0	0	0	0	0	0	0	0
67360	20112	36084	9198	167600	1473	222	672	0	512	2578	397	180	602	0	0
0	0	0	0	0	0	0	0	0	0	0	0	0	0	0	0
0	0	0	0	79	0	0	0	0	0	0	0	0	0	0	0
0	0	0	0	0	0	0	0	0	0	0	0	0	0	0	0
0	0	0	0	0	0	0	0	0	334	0	0	0	0	0	0
0	0	0	0	0	0	0	0	0	0	0	0	353	0	0	0
0	0	221	0	0	0	0	0	0	0	0	0	0	0	0	0
0	0	296	0	0	0	0	0	0	0	0	0	0	0	0	903
0	0	0	0	0	0	0	0	0	170	0	0	0	0	0	0
0	0	0	0	0	0	0	0	0	0	0	0	1008	0	0	0
948	0	0	0	0	0	0	0	0	0	0	0	0	0	0	0
0	0	0	0	0	0	0	0	0	0	0	0	0	0	0	0
0	0	0	0	0	0	0	0	0	0	0	0	0	0	0	0
0	0	0	0	0	0	0	0	0	0	0	0	0	0	0	0
0	0	0	0	0	0	0	0	0	0	0	0	0	0	0	0
84	882	4942	4206	8861	786	95225	1757	2692	23220	2940	21173	72	106234	221	2431
0	0	0	0	0	0	0	0	0	0	0	0	0	0	0	0
0	0	0	0	0	0	0	0	0	0	0	0	0	0	0	0
0	0	0	0	0	0	0	0	0	0	0	0	0	0	0	0
0	0	0	0	0	0	0	0	0	0	0	0	0	0	0	0
0	0	0	0	0	0	0	0	0	0	0	0	0	0	0	0
0	0	0	0	0	0	0	0	0	0	0	0	0	0	0	0
0	0	0	0	0	0	0	0	0	0	0	0	0	0	0	0
0	0	0	0	0	0	0	0	0	0	0	0	0	0	0	0
0	0	0	0	0	0	0	0	230	1600	0	0	0	0	0	0
0	0	0	0	0	0	0	0	0	0	0	0	0	0	0	0
0	0	0	0	0	0	0	0	0	0	0	0	0	0	0	0
3099	9735	892	4802	4951	133	0	0	0	49	0	0	0	83	0	0
0	0	0	0	0	0	0	0	0	0	0	0	0	0	0	0
0	0	0	0	0	0	0	0	0	0	0	0	0	0	0	0
0	0	0	0	0	0	0	0	0	0	0	0	0	0	0	0
0	0	0	0	0	0	0	0	0	0	0	0	0	0	0	0
148	57	197	31532	0	0	42	130	238	37483	335	173	0	87	0	46
0	0	0	0	0	0	0	0	0	0	0	0	0	0	0	0
0	0	0	0	0	0	0	0	0	0	0	0	0	0	0	0
0	0	0	0	0	0	0	0	0	0	0	0	0	0	0	0
0	0	0	0	0	1067	0	0	0	0	0	0	263	0	0	0
0	0	0	0	0	0	0	0	0	0	0	0	0	0	0	0
0	0	0	0	0	0	0	0	0	0	0	0	0	0	0	0
0	0	0	0	0	0	0	0	0	0	0	0	0	0	0	0
17008	146	93	0	170	0	0	0	0	0	0	0	0	0	0	0
0	0	0	0	0	0	0	0	0	0	0	0	0	0	0	0
0	0	0	0	0	0	0	0	0	0	0	0	0	0	0	0
0	0	0	0	0	0	0	0	0	0	0	0	0	0	0	0

0	0	1998	19971	72	133	0	127	232	42	0	0	0	82	0	0
0	0	0	0	0	0	0	0	0	0	0	0	0	0	0	0
0	0	0	0	0	0	0	0	0	0	0	0	0	0	0	0
0	0	0	0	0	796	1079	141	664	1658	331	503	50	1313	0	0
0	0	0	0	0	0	0	0	0	0	0	0	0	0	0	0
0	0	0	0	0	0	0	0	0	0	0	0	0	0	0	0
76	106	0	10543	0	2518	0	0	0	0	0	0	0	0	0	1787
0	0	0	0	0	0	0	0	0	0	0	0	0	0	0	0
0	0	0	0	0	0	0	0	0	0	0	0	0	0	0	0
0	0	0	0	0	125	0	0	0	0	0	0	0	0	0	0
0	0	0	0	0	0	0	0	0	0	0	0	0	0	0	0
0	0	0	0	0	0	0	0	0	0	0	0	0	0	0	0
0	0	0	0	0	0	0	0	0	0	0	0	0	0	0	0
0	0	0	0	0	0	0	0	0	0	0	0	0	0	0	0
0	0	0	0	0	0	0	0	0	0	0	0	0	0	0	0
0	0	0	0	0	0	0	0	0	0	0	0	0	0	0	0
0	53	1727	0	0	0	500	415	209	86175	651	229	0	2320	0	0
0	0	586	0	231	669	0	0	231	0	0	0	132	0	0	285
0	0	0	0	0	0	0	0	0	0	0	0	0	0	0	0
0	0	0	0	0	0	0	0	0	0	0	0	0	0	0	0
0	0	0	0	0	0	0	0	0	0	0	0	0	0	0	0
0	0	0	0	0	0	0	0	0	0	0	0	0	0	0	0
0	58	0	0	0	0	0	0	0	0	0	59	0	0	124	0
0	0	0	0	0	0	0	0	0	0	0	0	0	0	0	0
0	0	0	0	0	0	0	0	0	0	0	0	0	0	0	0
0	0	0	0	0	0	0	0	0	0	0	0	0	0	0	0
0	0	0	0	0	0	0	0	0	0	0	0	0	0	0	0
0	0	0	0	0	0	0	0	0	0	0	0	0	0	0	0
528	0	0	0	0	0	0	0	0	0	0	0	0	0	0	0
0	47	0	0	0	0	0	0	0	0	0	0	0	0	0	0
0	0	0	0	0	0	0	0	0	0	0	0	0	0	0	0
0	0	0	0	0	0	0	0	0	0	0	0	0	0	0	0
0	0	0	0	0	0	0	0	0	0	0	0	0	0	0	0
0	8930	0	0	80	136	0	0	0	0	0	0	0	0	0	0
0	0	0	0	0	0	0	0	0	0	0	0	0	0	0	0
0	0	0	0	0	0	0	0	0	0	0	0	0	0	0	0
0	0	0	0	0	0	0	0	0	0	0	0	0	0	0	144
0	0	0	0	0	0	0	0	0	0	0	0	0	0	0	0
0	0	0	0	0	0	0	0	0	0	0	0	0	0	0	0
0	0	0	2028	0	0	0	0	0	0	0	0	0	0	0	0
0	0	0	0	0	0	0	0	0	0	0	0	0	0	0	0
0	0	0	0	0	0	0	0	0	0	0	0	0	0	0	0
0	0	0	0	0	0	133	0	0	0	0	42	0	245	0	0
0	0	0	0	0	0	0	0	0	0	0	0	0	0	0	0
0	0	0	0	0	0	0	0	0	0	0	0	0	0	0	0
0	0	0	0	0	0	0	0	0	0	0	0	0	0	0	0
0	0	414	0	0	0	0	0	218	0	0	0	0	0	0	445
1911	0	0	0	0	0	0	0	0	0	0	0	0	0	0	0
0	0	0	0	0	0	0	0	0	0	0	0	0	0	0	0
78	47	0	5351	12562	1053	0	0	237	1747	0	57	0	79	110	0
0	0	0	0	0	0	0	0	0	0	0	0	0	0	0	0
0	0	0	0	0	0	0	0	10888	0	0	0	0	0	0	0
0	0	0	0	0	0	0	0	0	0	0	0	0	0	0	0
0	0	0	0	0	0	0	0	0	0	0	0	0	0	0	0
0	0	0	0	0	0	0	0	0	0	0	0	0	0	0	0
0	0	0	0	0	0	0	0	0	0	0	0	0	0	0	0
0	0	0	0	0	0	0	0	0	0	318	0	0	0	0	0
0	0	0	0	0	0	0	0	0	0	0	0	0	0	0	0

0	0	0	0	0	0	0	0	0	0	0	0	0	0	0	0
0	0	0	0	0	0	0	0	0	0	0	0	0	0	0	0
0	0	0	0	0	0	45	0	0	6650	0	0	0	191	0	0
0	0	0	0	0	0	0	0	225	3849	0	59	0	172	0	0
0	0	0	0	0	0	100	0	0	0	0	0	222	0	0	0
84	0	0	144	6544	0	56	0	0	1101	0	0	0	0	0	0
0	0	0	0	0	0	0	0	0	0	0	0	0	0	0	0
0	0	0	0	0	0	0	0	0	0	0	0	0	0	0	0
0	0	0	0	0	0	0	0	0	3024	0	0	0	0	0	0
0	0	0	0	0	0	0	0	0	0	0	0	0	0	0	0
0	0	0	0	0	0	0	0	0	0	0	0	0	0	0	0
0	0	0	0	0	0	0	0	0	5881	0	0	0	0	0	0
0	0	0	0	0	0	0	0	0	0	0	0	0	0	0	0
0	0	0	0	0	0	97	0	0	43	0	118	0	241	0	0
0	0	0	0	0	0	0	0	0	0	0	0	0	0	0	0
0	0	0	0	0	0	0	0	0	0	0	0	0	0	0	0
0	0	0	0	0	0	0	0	0	0	0	0	0	0	0	0
0	0	0	0	0	0	0	0	0	0	0	0	0	0	0	0
0	0	0	0	0	0	0	0	0	0	0	0	0	0	0	0
0	0	0	0	0	379	49	0	0	53	0	132	0	190	0	69
7192	5821	8625	17992	14626	12785	17594	282540	7617	204033	10590	5636	716231	25346	947205	76232
0	0	0	0	0	0	0	0	0	0	0	0	0	0	0	0
0	0	0	0	0	0	0	0	255	2580	0	0	0	0	0	0
0	0	96	0	0	140	0	372	0	843	0	64	709	177	0	1199
0	0	0	0	0	0	0	0	0	0	0	0	0	0	0	0
0	0	0	0	0	0	0	0	0	0	0	0	0	0	0	0
0	0	0	0	0	495	0	0	0	145	22752	0	1070	0	0	2167
0	0	0	0	0	0	0	0	0	0	0	0	0	0	0	0
0	0	0	0	0	0	0	0	0	0	0	0	0	0	0	0
0	0	0	0	0	0	0	0	0	0	0	0	0	0	0	0
0	0	0	0	0	0	0	0	0	0	0	0	0	0	0	0
0	0	0	0	0	0	0	0	0	0	0	0	0	0	0	0
151	54	67258	77	0	134	0	0	0	35	0	0	0	0	0	0
0	41	0	580	223	0	1601	0	224	102	0	442	0	2481	0	0

COVID-19-051	COVID-19-052	COVID-19-054	COVID-19-055	COVID-19-057	COVID-19-058	COVID-19-061	COVID-19-062	COVID-19-063	COVID-19-064	COVID-19-065	COVID-19-069
934704	960184	970139	869940	979692	998468	453830	745701	911400	935911	776780	879050
40789	6847	1454	97717	1285	365	329146	227611	39185	18176	1597	97034
0	0	0	0	0	0	0	0	0	0	0	0
15422	27746	27215	28358	4469	88	214329	26055	49248	44809	221367	23916
9026	5223	1192	3985	14419	895	2544	222	100	300	82	0
0	0	0	0	0	0	0	0	0	0	0	0
59	0	0	0	135	184	151	411	67	804	174	0

CC051	CC052	CC054	CC055	CC057	CC058	CC061	CC062	CC063	CC064	CC065	CC069
0	0	48	0	63	0	0	0	0	0	0	0
0	0	0	0	0	0	0	0	58	0	0	0
79043	48810	4541	1729	126211	4432	27167	34594	48548	696071	14612	320347
0	0	0	0	0	0	0	0	0	0	0	0
0	0	0	0	0	0	0	0	0	0	0	0
0	0	0	0	0	0	0	0	0	0	0	0
0	0	0	0	71	0	0	35118	0	38	0	0
0	0	0	0	0	0	0	0	0	0	0	0
0	0	0	0	0	0	0	0	0	0	0	0
388	13822	104	126	131	157	36339	24261	862	31	100	0
0	0	0	0	0	0	0	0	0	0	0	0
0	0	0	0	0	0	0	0	0	0	0	0
0	0	0	0	0	0	0	0	0	0	0	0
57	0	743	46	639	0	0	5306	350	3150	181	0
0	0	0	0	0	0	0	0	0	250	0	59
0	0	0	0	0	0	0	0	0	0	0	0
0	0	0	0	0	0	0	0	0	2562	0	0
0	0	0	0	0	0	0	0	0	0	0	0
0	0	0	0	0	0	0	0	0	0	0	0
0	0	0	0	0	0	0	0	0	0	0	0
0	0	167	0	0	0	631	70	118	0	158	0
0	0	0	0	0	0	0	0	0	0	0	0
21306	1625	53	0	0	0	0	7113	0	254	0	0
0	0	0	0	0	0	0	0	0	0	0	0
0	0	0	0	0	0	0	0	0	0	0	0
283769	11090	615	711	290	345	29357	255270	420574	20696	736724	25159
0	0	0	0	0	0	0	0	0	0	0	0
0	0	50	0	0	84	0	0	15442	0	95	67
0	0	0	0	0	0	0	0	0	0	0	59
0	0	0	0	0	0	0	0	0	0	0	0
0	0	0	0	0	0	0	0	0	0	0	0
61	0	0	0	0	0	0	0	0	0	0	0
0	0	0	0	0	0	0	0	0	0	0	0
0	0	0	0	131	0	0	0	0	0	0	0
0	0	0	0	0	0	0	0	0	0	0	0
0	0	0	0	0	0	0	0	0	0	0	0
0	0	0	0	0	0	0	0	0	0	0	0
0	0	53	23709	0	0	0	0	0	0	0	0
118	310	0	0	0	0	0	0	0	0	0	0
483477	13206	744	1959	134	176	366	76121	150	26279	86	6548
0	0	0	0	0	0	0	0	0	0	0	0
0	0	0	0	0	0	0	0	0	0	0	0
0	0	0	0	0	0	0	0	0	0	0	0
0	0	0	0	0	0	0	41	1576	0	0	0
0	0	0	0	0	0	0	0	5827	0	0	0
0	0	0	0	0	0	0	0	0	0	0	0
0	0	0	0	0	0	74	0	0	1664	99	0

0	0	0	0	0	0	0	0	0	0	0	0
0	0	0	0	0	0	0	0	0	0	0	0
5154	7090	8018	4324	3696	487372	937	0	164	672	87	19603
0	0	0	0	0	0	0	0	0	0	0	0
0	0	0	0	0	0	0	0	0	0	0	0
0	0	0	0	0	0	0	0	0	0	0	0
0	0	0	0	0	0	0	0	0	0	0	0
0	0	0	0	0	0	0	0	0	0	0	0
0	0	0	0	0	0	0	0	0	0	0	0
0	0	0	0	0	0	0	0	0	0	0	0
0	0	0	0	0	0	0	0	0	0	0	0
0	0	0	0	0	0	0	0	0	0	0	0
0	0	0	0	0	0	0	0	0	0	0	0
0	0	0	0	0	0	0	0	0	0	0	0
0	0	0	0	0	0	0	0	0	0	0	0
0	0	0	0	0	0	0	0	0	0	0	0
0	0	0	0	0	0	0	0	0	0	0	0
0	0	0	0	0	0	0	0	0	0	0	0
0	0	0	0	0	0	0	0	0	0	0	0
838	11487	98	103	511	584	292	58767	23183	40346	1060	170553
0	0	0	0	0	0	0	0	0	0	0	0
0	0	0	0	0	0	0	0	0	0	0	0
0	0	0	0	0	0	0	0	0	0	0	0
0	0	0	0	0	0	0	0	0	0	0	0
0	0	0	0	0	0	0	0	0	0	0	0
0	0	0	0	0	0	0	0	0	0	0	0
0	0	0	0	0	0	0	0	0	0	0	0
0	0	0	0	0	0	0	0	0	0	0	74
0	0	0	0	0	0	0	0	0	0	0	0
0	0	0	0	0	0	0	0	0	0	0	0
0	0	0	0	0	0	0	0	0	233	0	0
0	0	0	0	0	0	0	0	0	0	0	0
0	0	0	0	0	0	0	0	0	0	0	0
0	0	0	0	0	0	0	0	0	74	0	0
0	0	0	0	0	0	0	0	0	0	0	0
0	0	0	0	0	0	0	0	0	0	0	0
0	0	0	0	0	0	0	0	0	0	0	0
0	0	0	0	0	0	0	0	0	0	0	0
0	0	0	0	0	0	0	0	0	0	0	0
0	978	41	0	0	0	0	0	0	0	0	0
0	0	0	0	0	0	0	0	0	0	0	0
0	0	0	0	0	0	0	0	0	0	0	0
0	0	0	0	0	0	0	6645	0	0	94	0
0	0	0	0	0	0	0	0	0	0	0	0
0	0	0	0	0	0	0	0	0	0	0	0
0	0	0	0	0	0	0	0	0	0	0	0
0	0	0	0	0	0	0	0	0	0	0	0
0	0	0	0	0	0	0	0	0	0	0	0
459	0	0	0	0	0	0	0	0	0	0	0
0	0	0	0	0	0	0	0	0	0	0	649
0	0	0	0	0	0	0	0	0	0	0	0
0	0	0	0	0	0	0	0	0	0	0	0
0	0	0	0	0	0	0	0	0	0	0	0
0	0	0	0	0	0	0	0	0	0	0	50599
0	0	116	0	0	0	0	0	0	724	0	0
9026	5223	1192	3985	14419	895	2544	222	100	300	82	0
0	0	0	0	0	0	0	0	0	0	0	0
0	0	0	0	0	0	0	0	0	0	0	0

0	0	0	0	0	0	0	0	0	0	0	0
0	0	0	0	0	0	0	0	0	0	0	0
0	0	0	0	0	0	0	0	0	0	0	0
0	0	0	0	0	0	0	0	0	0	0	0
0	0	0	0	0	0	0	0	0	0	0	0
0	0	0	0	0	81	3075	0	0	0	0	4473
0	0	0	0	0	0	0	0	0	0	0	0
856	0	0	0	0	0	0	0	56	1548	0	0
0	0	0	0	0	0	0	0	0	0	0	0
0	0	0	0	0	0	0	0	0	0	0	0
0	0	0	0	0	0	0	0	0	0	0	4229
0	0	0	0	0	0	77	0	613	0	0	0
0	0	0	0	0	0	0	0	0	0	0	0
0	0	0	0	0	0	0	0	0	0	0	0
0	0	0	0	0	0	0	0	0	0	0	0
1150	98	0	0	466	0	233	93	58	12729	168	57
0	106	67	0	275	0	0	53	53	911	0	0
0	0	0	0	0	0	0	0	14770	0	0	0
0	0	0	0	0	0	0	0	0	0	0	0
0	0	0	0	0	0	0	0	0	0	0	0
0	0	0	0	0	0	0	0	0	0	0	0
0	0	0	0	0	0	0	0	0	0	0	0
14566	27746	27215	28358	4469	88	214329	26055	49192	43261	221367	23916
0	0	56	0	0	79	0	0	0	0	0	0
14104	833	0	156	0	0	172	715	0	31	0	0
0	0	0	0	0	0	0	0	0	0	0	0
0	0	0	0	0	100	288	226	268475	0	283	0
0	0	0	0	0	0	0	0	0	0	0	0
0	199	0	56	0	0	66	0	0	0	0	0
83	0	0	57	0	0	0	31	0	0	94	64
0	0	0	0	0	0	78	151	0	352	0	0
0	0	0	0	0	0	0	0	0	0	0	0
0	0	0	0	0	0	0	0	0	0	0	0
0	0	0	0	0	0	0	0	0	0	0	0
0	0	0	0	0	0	0	0	0	0	0	0
0	0	0	0	0	0	0	0	0	0	0	0
0	0	0	0	0	0	0	0	0	0	0	0
0	0	0	0	0	0	0	0	0	0	0	0
0	0	0	0	0	0	0	1484	0	0	0	0
0	0	0	0	0	0	0	0	0	0	0	0
0	0	0	0	0	0	0	0	0	0	0	0
0	707	108	0	419	0	0	137	241	392	103	12374
0	0	0	0	0	0	0	0	0	0	0	0
0	0	0	0	0	0	0	0	0	0	0	0
0	0	0	0	0	0	0	0	0	0	0	0
0	0	0	0	0	0	0	0	0	0	0	0
0	0	0	0	0	0	0	0	0	0	0	0
0	0	0	0	0	0	0	0	0	0	0	0
0	0	0	0	0	0	197	37875	56	0	0	0
389	0	0	0	0	0	0	0	0	0	0	0
0	0	0	0	0	0	0	0	0	0	0	0
170	45885	402	16452	833482	481153	0	0	0	31	0	465
0	0	0	0	0	0	0	0	0	0	0	0
0	0	0	0	0	0	0	0	0	0	0	0
0	0	0	0	0	0	0	0	0	719	0	0
0	100	0	0	0	0	0	0	0	0	0	0
0	0	0	0	0	0	0	0	0	0	0	0
0	0	0	0	0	0	0	0	0	0	0	0

0	411	0	0	0	0	137	35	0	0	253	0
0	0	0	0	0	0	0	0	0	0	0	0
0	0	0	0	0	0	0	0	0	0	0	0
0	0	0	0	0	0	0	0	0	0	0	0
0	0	0	0	0	0	0	0	0	0	0	0
0	0	210	0	0	0	0	0	0	0	0	0
0	0	0	0	0	0	0	0	0	0	0	0
0	0	0	0	0	0	0	0	0	0	0	0
0	0	0	0	0	0	0	0	0	0	0	0
0	0	0	0	0	0	0	0	0	0	0	0
0	90	0	0	68	0	0	0	0	0	0	0
0	0	52	0	0	0	0	0	0	0	0	0
0	0	0	0	0	0	0	0	0	0	0	0
0	0	50	0	0	0	0	0	0	0	0	0
0	0	56	0	0	0	0	0	0	0	0	0
0	0	0	0	0	0	0	0	0	0	0	0
0	0	0	0	0	0	0	0	0	0	0	0
0	0	0	0	0	0	0	0	0	0	0	0
0	0	0	0	0	0	0	0	0	0	0	0
0	0	0	0	0	0	0	0	0	0	82	0
20291	3617	595	40734	197	181	216506	180054	15748	7851	681	40253
0	0	0	0	0	0	0	0	0	0	0	0
0	0	0	0	0	0	0	0	0	0	0	0
0	0	0	0	0	0	0	0	0	0	0	0
0	0	0	0	0	0	0	0	0	0	0	0
0	0	57	0	0	0	0	0	0	39	0	0
0	0	0	0	0	0	0	0	0	0	0	0
0	0	0	0	0	0	0	0	45	0	0	0
0	0	0	0	0	0	0	0	0	0	0	0
0	0	0	0	0	0	0	0	0	0	0	0
0	0	0	0	0	0	0	0	0	0	0	0
0	730	48	0	0	0	0	0	0	0	0	0
0	0	0	0	0	0	0	0	0	0	0	0
0	0	0	0	61	0	43739	0	0	90	0	0
6952	0	0	0	0	0	0	168	0	101	0	0
0	0	0	0	0	0	0	0	0	0	0	0
0	0	0	0	0	0	0	4171	0	0	0	0
0	0	0	0	0	0	0	0	0	0	0	0
0	0	0	0	0	0	0	0	0	0	0	0
0	0	0	0	0	0	0	0	0	0	0	0
0	83	0	14388	0	0	0	0	0	0	0	0
0	0	0	0	0	0	0	0	0	0	0	0
0	0	0	0	0	0	0	0	0	0	0	0
0	0	0	0	0	0	0	0	0	0	0	0
0	4647	0	0	0	0	0	0	0	0	0	0
0	0	0	0	0	0	0	0	0	0	0	0
0	110	42	0	0	0	179142	28	59	0	175	0
0	0	0	0	65	0	0	0	0	42	0	30863
0	0	0	0	543	0	0	0	0	0	0	0
0	688	0	0	0	0	0	0	0	0	0	0
0	0	0	0	0	0	0	0	0	0	0	0
0	0	0	0	0	0	0	0	0	0	0	0
0	0	0	0	0	0	0	0	0	0	0	0
0	0	0	0	59	0	0	0	62	38	83	0
0	0	0	0	54	0	0	0	0	0	0	0
0	0	0	0	0	0	0	0	0	0	0	0
67	187	0	49	1849	0	75	475	503	6919	170	162
0	0	0	0	0	0	0	0	0	0	0	0

0	0	0	0	0	0	0	0	0	0	0	0
0	0	0	0	0	0	0	0	0	0	0	0
0	0	0	0	0	0	0	0	0	0	0	0
4144	0	0	0	0	0	0	209	0	119	0	0
0	0	0	0	0	0	0	0	0	0	0	0
0	0	0	0	0	0	0	0	0	2900	0	0
0	0	0	0	0	0	0	0	0	0	0	0
117	0	0	0	85	177	4881	0	6174	76	0	32443
0	0	0	2085	0	95	0	0	0	0	0	0
0	0	0	0	0	0	0	0	0	0	0	0
0	0	0	0	422	0	162	62	107	2166	0	0
0	0	0	0	0	0	0	0	0	0	0	0
0	0	0	0	0	0	0	0	0	0	0	676
0	0	0	0	0	0	0	0	0	0	0	623
0	0	0	0	0	93	0	0	0	0	0	11249
0	0	0	0	0	0	0	0	752	0	0	0
0	0	0	0	0	0	0	0	0	0	0	0
0	0	46	0	0	0	0	0	4950	0	0	0
0	0	0	0	0	0	0	0	0	0	0	0
0	0	0	0	0	0	0	0	0	0	0	0
0	0	0	0	0	0	0	0	0	0	0	0
0	0	0	0	120	0	66	0	0	0	0	0
0	0	0	0	0	0	0	0	0	0	0	0
0	0	0	0	0	0	0	0	0	0	0	0
0	0	0	0	0	0	0	0	0	0	0	23925
0	0	0	0	0	0	0	38	0	0	0	0
0	0	0	0	0	0	0	29	0	34	0	0
70	0	0	30923	0	0	0	0	0	58	0	31458
0	0	0	0	0	0	0	0	0	0	0	0
0	0	0	0	0	0	0	0	0	0	0	0
0	0	0	0	0	0	0	0	0	0	0	0
0	0	0	0	0	0	0	0	0	0	0	0
0	0	0	0	0	0	0	0	0	0	0	0
0	0	0	0	0	0	0	0	0	0	0	0
0	0	0	0	0	0	0	0	0	0	0	0
0	0	0	0	0	0	0	0	0	0	0	12619
0	0	0	0	0	0	0	0	0	0	0	0
0	0	0	0	0	0	0	0	0	0	0	0
0	0	0	0	0	0	0	0	0	0	0	0
0	0	0	57	0	0	0	0	2372	37	98	59
0	0	0	0	0	0	0	0	0	0	0	0
0	0	0	0	0	0	0	0	0	0	0	0
0	0	0	0	0	0	0	0	0	0	0	0
0	0	0	0	0	0	0	0	0	0	0	0
0	0	0	0	0	0	0	0	0	0	0	0
0	0	0	0	0	0	0	0	0	0	0	0
0	0	0	0	0	0	0	0	0	0	0	0
0	0	0	0	0	0	0	0	0	0	0	0
0	7199	0	0	1034	708	6184	439	837	11844	284	6542
0	0	0	0	0	0	0	0	0	0	0	0
0	0	0	0	0	0	286	134818	135	0	598	0
0	0	0	0	0	0	0	0	0	0	0	0
2664	715	0	0	0	81	0	232	0	247	0	0
0	0	0	0	0	0	0	0	0	0	0	0
0	0	0	0	0	0	0	0	0	0	0	0
0	0	0	0	0	0	0	0	0	0	0	0
0	938	0	0	0	0	0	0	0	0	0	0
0	0	0	0	0	0	0	0	0	0	0	0

0	0	0	0	0	0	0	0	0	0	0	0
0	0	0	0	0	0	0	0	0	0	0	0
0	0	0	0	0	0	0	0	0	0	0	0
0	0	0	0	0	0	0	0	0	0	0	0
20046	411	430	266	0	89	369	3294	257	852	654	0
0	0	0	0	0	0	0	0	0	0	0	0
0	0	0	2300	157	154	35787	58893	553	50676	429	34863
0	0	0	0	0	0	0	0	0	0	0	0
0	0	0	0	0	0	0	0	0	0	0	0
0	0	0	0	354	365	0	0	0	482	0	524
0	0	0	0	0	0	0	7267	0	4381	0	0
0	0	0	0	0	0	0	0	0	0	0	0
0	0	0	0	0	0	0	0	0	0	0	0
0	0	0	0	0	0	0	0	0	0	0	0
0	0	0	0	0	0	0	0	0	0	0	0
0	0	0	0	0	0	0	0	0	0	0	0
183	2184	105	62	3709	74	2150	713	1824	32367	1264	353
0	0	0	0	0	0	0	62	0	0	0	0
0	0	0	0	0	0	0	0	0	0	0	0
0	0	0	0	0	0	0	0	0	0	0	0
0	0	0	0	0	0	0	0	0	0	0	0
0	0	0	0	0	0	0	0	0	0	0	0
0	0	0	0	0	0	13533	47	0	0	0	0
0	0	0	0	0	0	0	0	0	0	0	0
0	0	0	0	0	0	0	0	0	0	0	0
0	0	0	0	0	0	0	0	0	0	0	0
300	104	0	0	0	0	0	0	0	0	0	0
0	0	0	0	0	0	0	0	0	0	0	0
0	0	0	0	0	0	0	0	0	0	0	0
135	0	52	0	0	0	0	0	0	0	0	0
0	0	0	0	0	0	0	0	0	0	0	0
130	0	0	0	0	0	0	0	0	0	0	0
0	0	0	0	0	0	0	0	0	0	0	0
0	0	0	0	0	0	65615	22	0	0	0	0
0	0	0	0	0	0	0	0	0	0	0	0
0	0	53	0	0	0	0	0	0	0	0	0
0	0	0	0	0	0	0	0	0	0	0	0
0	0	0	0	0	0	0	0	0	0	0	0
0	0	0	0	0	0	0	0	0	0	0	0
0	0	0	0	0	0	0	0	0	0	0	0
0	0	0	0	0	0	0	0	0	0	0	0
0	0	0	0	0	0	0	0	0	0	0	0
0	0	0	0	0	0	0	0	0	0	0	0
0	0	0	0	0	0	0	0	0	0	0	0
0	0	0	0	0	0	0	0	0	0	0	0
3233	0	0	0	0	87	0	72	0	229	0	0
0	0	0	0	0	0	0	0	0	0	0	0
56	0	0	0	70	0	32952	32	55	0	0	151
0	0	0	0	0	0	0	0	0	0	0	0
0	0	0	0	0	0	0	0	0	0	0	0
0	0	0	0	0	0	0	0	0	0	0	0
0	0	0	0	0	0	69	38	0	0	0	0
0	0	0	0	0	0	0	0	0	0	0	0
0	0	0	0	0	0	0	0	0	0	0	0
0	0	0	0	0	0	275	69	155	0	97	0
0	0	0	0	0	0	0	0	0	0	0	0
0	0	0	0	0	0	0	0	0	0	0	0
0	0	0	0	0	0	0	0	0	0	0	0

0	0	0	0	0	0	0	0	0	0	0	0
0	0	0	0	0	0	0	0	0	0	0	0
0	0	0	0	0	0	0	0	0	0	0	0
0	0	0	0	0	0	0	0	0	0	0	0
0	0	0	0	0	0	0	0	0	0	77	0
0	0	0	0	0	0	0	0	0	0	0	0
0	0	0	0	0	0	0	0	0	0	0	0
0	0	0	0	0	0	0	0	3448	1069	0	22050
0	0	0	0	0	0	0	0	0	0	0	0
0	0	50	0	0	0	0	0	0	0	0	0
0	0	0	0	0	0	0	0	0	5137	0	0
0	0	0	0	0	0	0	0	0	0	0	59
0	0	0	0	0	0	0	0	0	0	0	0
0	0	0	0	0	0	77	15870	0	0	74	0
0	0	0	0	0	0	0	0	0	0	0	0
0	0	0	0	0	0	0	0	0	0	0	0
0	0	0	0	0	0	94	0	0	0	0	0
0	0	0	0	0	84	0	0	0	0	0	0
0	0	0	0	0	0	0	0	0	0	0	0
0	0	0	0	0	0	0	0	0	0	0	0
0	0	0	0	0	0	0	0	0	0	0	0
0	0	0	0	0	0	0	0	0	0	0	0
0	0	0	0	0	0	0	0	0	0	0	61
0	0	0	0	0	0	0	0	0	0	0	28110
0	0	0	0	0	0	0	0	0	0	0	0
26126	788571	953671	806542	5611	22082	81464	19458	105771	18124	758	83662
0	0	0	0	0	0	0	0	0	0	0	0
0	0	0	0	0	0	0	0	0	0	0	0
59	0	0	0	135	184	151	411	67	804	174	0
0	0	0	0	0	0	0	0	0	0	0	0
0	0	0	0	0	0	0	0	0	0	0	0
413	0	52	20823	0	0	64	2646	5660	0	18656	0
0	0	0	0	0	0	0	0	0	0	0	0

COVID-19-051	COVID-19-052	COVID-19-054	COVID-19-055	COVID-19-057	COVID-19-058	COVID-19-061	COVID-19-062	COVID-19-063	COVID-19-064	COVID-19-065	COVID-19-069
0	0	0	0	0	0	0	0	0	0	0	0
0	0	48	0	0	0	0	0	0	0	0	0
0	0	0	0	0	0	0	0	0	0	0	0
0	0	0	0	0	0	0	0	0	0	0	0
0	0	0	0	63	0	0	0	0	0	0	0
0	0	0	0	0	0	0	0	0	0	0	0
0	0	0	0	0	0	0	0	58	0	0	0
79043	48810	4541	1729	126211	4432	27167	34594	48548	696071	14612	320347
0	0	0	0	0	0	0	0	0	0	0	0
0	0	0	0	0	0	0	0	0	0	0	0
0	0	0	0	0	0	0	0	0	0	0	0
0	0	0	0	71	0	0	35118	0	38	0	0
0	0	0	0	0	0	0	0	0	0	0	0
0	0	0	0	0	0	0	0	0	0	0	0
388	13822	104	126	131	157	36339	24261	862	31	100	0
0	0	0	0	0	0	0	0	0	0	0	0
0	0	0	0	0	0	0	0	0	0	0	0
0	0	0	0	0	0	0	0	0	0	0	0
57	0	743	46	639	0	0	5306	350	3150	181	0
0	0	0	0	0	0	0	0	0	250	0	59
0	0	0	0	0	0	0	0	0	0	0	0
0	0	0	0	0	0	0	0	0	2562	0	0
0	0	0	0	0	0	0	0	0	0	0	0

0	0	0	0	0	0	0	0	0	0	0	0
0	0	0	0	0	0	0	0	0	0	0	0
0	0	0	0	0	0	0	0	0	0	0	0
0	0	167	0	0	0	631	70	118	0	158	0
0	0	0	0	0	0	0	0	0	0	0	0
21306	1625	53	0	0	0	0	7113	0	254	0	0
0	0	0	0	0	0	0	0	0	0	0	0
0	0	0	0	0	0	0	0	0	0	0	0
0	0	0	0	0	0	0	0	0	0	0	0
0	0	0	0	0	0	72	16726	0	0	180	168
0	0	0	0	0	0	0	0	0	0	0	0
0	0	0	0	0	0	0	0	0	0	0	0
0	0	0	0	0	0	0	207	0	0	0	0
0	0	0	0	0	0	0	0	0	0	0	0
0	0	0	0	0	0	0	0	0	0	0	0
0	0	0	0	0	0	0	0	0	0	0	0
0	0	0	0	0	0	0	0	0	0	0	0
0	0	0	0	0	0	0	0	0	0	0	0
0	0	0	0	0	0	0	0	0	0	0	0
0	0	0	0	0	0	0	0	0	0	0	0
70	0	0	434	130	0	14140	4344	2094	79	0	342
0	0	0	0	0	0	0	0	58	0	0	0
0	0	0	0	0	99	0	0	641	0	0	0
283512	10281	567	277	160	164	5799	226813	417721	20617	736544	19173
116	0	0	0	0	0	0	0	0	0	0	4841
0	0	0	0	0	0	0	0	0	0	0	0
71	0	0	0	0	0	3927	0	0	0	0	635
0	611	48	0	0	82	5419	7180	60	0	0	0
0	198	0	0	0	0	0	0	0	0	0	0
0	0	0	0	0	0	0	0	0	0	0	0
0	0	0	0	0	0	0	0	0	0	0	0
0	0	50	0	0	84	0	0	15442	0	95	67
0	0	0	0	0	0	0	0	0	0	0	0
0	0	0	0	0	0	0	0	0	0	0	59
0	0	0	0	0	0	0	0	0	0	0	0
0	0	0	0	0	0	0	0	0	0	0	0
0	0	0	0	0	0	0	0	0	0	0	0
61	0	0	0	0	0	0	0	0	0	0	0
0	0	0	0	0	0	0	0	0	0	0	0
0	0	0	0	131	0	0	0	0	0	0	0
0	0	0	0	0	0	0	0	0	0	0	0
0	0	0	0	0	0	0	0	0	0	0	0
0	0	0	0	0	0	0	0	0	0	0	0
0	0	0	0	0	0	0	0	0	0	0	0
0	0	53	23709	0	0	0	0	0	0	0	0
0	0	0	0	0	0	0	0	0	0	0	0
118	310	0	0	0	0	0	0	0	0	0	0
0	0	0	0	0	0	0	0	0	0	0	0
483477	13206	744	1959	134	176	366	76121	150	26279	86	6548
0	0	0	0	0	0	0	0	0	0	0	0
0	0	0	0	0	0	0	0	0	0	0	0
0	0	0	0	0	0	0	0	0	0	0	0
0	0	0	0	0	0	0	0	0	0	0	0
0	0	0	0	0	0	0	0	0	0	0	0
0	0	0	0	0	0	0	0	0	0	0	0
0	0	0	0	0	0	0	41	1576	0	0	0
0	0	0	0	0	0	0	0	5827	0	0	0
0	0	0	0	0	0	0	0	0	0	0	0

0	0	0	0	0	0	74	0	0	1664	99	0
0	0	0	0	0	0	0	0	0	0	0	0
0	0	0	0	0	0	0	0	0	0	0	0
5154	7090	7906	4324	770	485298	798	0	164	672	0	663
0	0	0	0	0	0	0	0	0	0	0	0
0	0	0	0	0	0	0	0	0	0	0	0
0	0	0	0	0	0	0	0	0	0	0	0
0	0	0	0	0	0	0	0	0	0	0	0
0	0	0	0	0	0	0	0	0	0	0	0
0	0	0	0	52	0	0	0	0	0	87	18940
0	0	0	0	0	0	0	0	0	0	0	0
0	0	0	0	1466	1141	0	0	0	0	0	0
0	0	0	0	0	0	0	0	0	0	0	0
0	0	58	0	1336	830	66	0	0	0	0	0
0	0	54	0	0	0	73	0	0	0	0	0
0	0	0	0	72	103	0	0	0	0	0	0
0	0	0	0	0	0	0	0	0	0	0	0
0	0	0	0	0	0	0	0	0	0	0	0
0	0	0	0	0	0	0	0	0	0	0	0
0	0	0	0	0	0	0	0	0	0	0	0
0	0	0	0	0	0	0	0	0	0	0	0
0	0	0	0	0	0	0	0	0	0	0	0
0	0	0	0	0	0	0	0	0	0	0	0
0	0	0	0	0	0	0	0	0	0	0	0
0	0	0	0	0	0	0	0	0	0	0	0
0	0	0	0	0	0	0	0	0	0	0	0
0	0	0	0	0	0	0	0	0	0	0	0
0	0	0	0	0	0	0	0	0	0	0	0
0	0	0	0	0	0	0	0	0	0	0	0
0	0	0	0	0	0	0	0	0	0	0	0
0	0	0	0	0	0	0	0	0	0	0	0
0	0	0	0	0	0	0	0	0	0	0	0
0	0	0	0	0	0	0	0	0	0	0	0
0	0	0	0	0	0	0	0	0	0	0	0
0	0	0	0	0	0	0	0	0	0	0	0
184	2101	0	0	0	68	69	14883	438	4237	0	117
0	0	0	0	0	0	0	0	0	83	0	0
597	6488	0	51	261	341	0	0	19463	8028	87	169997
57	207	48	52	180	0	65	1254	3227	15894	517	76
0	2691	50	0	70	84	158	42630	55	189	166	310
0	0	0	0	0	91	0	0	0	11915	290	53
0	0	0	0	0	0	0	0	0	0	0	0
0	0	0	0	0	0	0	0	0	0	0	0
0	0	0	0	0	0	0	0	0	0	0	0
0	0	0	0	0	0	0	0	0	0	0	0
0	0	0	0	0	0	0	0	0	0	0	0
0	0	0	0	0	0	0	0	0	0	0	0
0	0	0	0	0	0	0	0	0	0	0	0
0	0	0	0	0	0	0	0	0	0	0	0
0	0	0	0	0	0	0	0	0	0	0	0
0	0	0	0	0	0	0	0	0	0	0	74
0	0	0	0	0	0	0	0	0	0	0	0
0	0	0	0	0	0	0	0	0	0	0	0
0	0	0	0	0	0	0	0	0	0	0	0
0	0	0	0	0	0	0	0	0	0	0	0
0	0	0	0	0	0	0	0	0	233	0	0
0	0	0	0	0	0	0	0	0	0	0	0
0	0	0	0	0	0	0	0	0	0	0	0

0	0	0	0	0	0	0	0	0	0	0	0
0	0	0	0	0	0	0	0	0	0	0	0
0	0	0	0	0	0	0	0	0	0	0	0
0	0	0	0	0	0	0	0	0	74	0	0
0	0	0	0	0	0	0	0	0	0	0	0
0	0	0	0	0	0	0	0	0	0	0	0
0	0	0	0	0	0	0	0	0	0	0	0
0	0	0	0	0	0	0	0	0	0	0	0
0	0	0	0	0	0	0	0	0	0	0	0
0	0	0	0	0	0	0	0	0	0	0	0
0	0	0	0	0	0	0	0	0	0	0	0
0	0	0	0	0	0	0	0	0	0	0	0
0	978	41	0	0	0	0	0	0	0	0	0
0	0	0	0	0	0	0	0	0	0	0	0
0	0	0	0	0	0	0	0	0	0	0	0
0	0	0	0	0	0	0	0	0	0	0	0
0	0	0	0	0	0	0	0	0	0	0	0
0	0	0	0	0	0	0	0	0	0	0	0
0	0	0	0	0	0	0	6645	0	0	94	0
0	0	0	0	0	0	0	0	0	0	0	0
0	0	0	0	0	0	0	0	0	0	0	0
0	0	0	0	0	0	0	0	0	0	0	0
0	0	0	0	0	0	0	0	0	0	0	0
0	0	0	0	0	0	0	0	0	0	0	0
0	0	0	0	0	0	0	0	0	0	0	0
0	0	0	0	0	0	0	0	0	0	0	0
459	0	0	0	0	0	0	0	0	0	0	0
0	0	0	0	0	0	0	0	0	0	0	0
0	0	0	0	0	0	0	0	0	0	0	0
0	0	0	0	0	0	0	0	0	0	0	0
0	0	0	0	0	0	0	0	0	0	0	0
0	0	0	0	0	0	0	0	0	0	0	0
0	0	0	0	0	0	0	0	0	0	0	649
0	0	0	0	0	0	0	0	0	0	0	0
0	0	0	0	0	0	0	0	0	0	0	0
0	0	0	0	0	0	0	0	0	0	0	0
0	0	0	0	0	0	0	0	0	0	0	0
0	0	0	0	0	0	0	0	0	0	0	0
0	0	0	0	0	0	0	0	0	0	0	50599
0	0	116	0	0	0	0	0	0	0	0	0
0	0	0	0	0	0	0	0	0	0	0	0
0	0	0	0	0	0	0	0	0	724	0	0
9026	5223	1192	3985	14419	895	2544	222	100	300	82	0
0	0	0	0	0	0	0	0	0	0	0	0
0	0	0	0	0	0	0	0	0	0	0	0
0	0	0	0	0	0	0	0	0	0	0	0
0	0	0	0	0	0	0	0	0	0	0	0
0	0	0	0	0	0	0	0	0	0	0	0
0	0	0	0	0	0	0	0	0	0	0	0
0	0	0	0	0	0	0	0	0	0	0	0
0	0	0	0	0	0	0	0	0	0	0	0
0	0	0	0	0	81	3075	0	0	0	0	4473
0	0	0	0	0	0	0	0	0	0	0	0
856	0	0	0	0	0	0	0	56	1548	0	0
0	0	0	0	0	0	0	0	0	0	0	0
0	0	0	0	0	0	0	0	0	0	0	0
0	0	0	0	0	0	0	0	0	0	0	0
0	0	0	0	0	0	0	0	0	0	0	0

0	0	0	0	0	0	0	0	0	0	0	0
0	0	0	0	0	0	0	0	0	0	0	4229
0	0	0	0	0	0	77	0	613	0	0	0
0	0	0	0	0	0	0	0	0	0	0	0
0	0	0	0	0	0	0	0	0	0	0	0
0	0	0	0	0	0	0	0	0	0	0	0
1150	98	0	0	466	0	233	93	58	12729	168	57
0	0	0	0	0	0	0	0	14770	0	0	0
0	0	0	0	0	0	0	0	0	0	0	0
0	0	0	0	0	0	0	0	0	0	0	0
0	0	0	0	0	0	0	0	0	0	0	0
0	0	0	0	0	0	0	0	0	0	0	0
0	0	0	0	0	0	0	0	0	0	0	0
0	0	0	0	0	0	0	0	0	0	0	0
14566	27746	27215	28358	4469	88	214329	26055	49192	43261	221367	23916
0	0	56	0	0	0	0	0	0	0	0	0
0	0	0	0	0	79	0	0	0	0	0	0
0	0	0	0	0	0	0	0	0	0	0	0
0	0	0	0	0	0	0	0	0	0	0	0
0	0	0	0	0	0	0	0	0	0	0	0
0	0	0	0	0	0	0	0	0	0	0	0
14104	833	0	156	0	0	172	715	0	31	0	0
0	0	0	0	0	0	0	0	0	0	0	0
0	0	0	0	0	100	288	226	268475	0	283	0
0	0	0	0	0	0	0	0	0	0	0	0
0	199	0	56	0	0	66	0	0	0	0	0
0	0	0	0	0	0	0	0	0	0	0	0
83	0	0	57	0	0	0	31	0	0	94	64
0	0	0	0	0	0	78	151	0	352	0	0
0	0	0	0	0	0	0	0	0	0	0	0
0	0	0	0	0	0	0	0	0	0	0	0
0	0	0	0	0	0	0	0	0	0	0	0
0	0	0	0	0	0	0	0	0	0	0	0
0	0	0	0	0	0	0	0	0	0	0	0
0	0	0	0	0	0	0	0	0	0	0	0
0	0	0	0	0	0	0	0	0	0	0	0
0	0	0	0	0	0	0	0	0	0	0	0
0	0	0	0	0	0	0	0	0	0	0	0
0	0	0	0	0	0	0	0	0	0	0	0
0	0	0	0	0	0	0	0	0	0	0	0
0	0	0	0	0	0	0	0	0	0	0	0
0	0	0	0	0	0	0	1484	0	0	0	0
0	0	0	0	0	0	0	0	0	0	0	0
0	0	0	0	0	0	0	0	0	0	0	0
0	707	108	0	419	0	0	137	241	392	103	12374
0	0	0	0	0	0	0	0	0	0	0	0
0	0	0	0	0	0	0	0	0	0	0	0
0	0	0	0	0	0	0	0	0	0	0	0
0	0	0	0	0	0	0	0	0	0	0	0
0	0	0	0	0	0	0	0	0	0	0	0
0	0	0	0	0	0	0	0	0	0	0	0
0	0	0	0	0	0	197	37875	56	0	0	0
389	0	0	0	0	0	0	0	0	0	0	0
0	0	0	0	0	0	0	0	0	0	0	0
170	45885	402	16452	833482	481153	0	0	0	31	0	465
0	0	0	0	0	0	0	0	0	0	0	0
0	0	0	0	0	0	0	0	0	0	0	0
0	0	0	0	0	0	0	0	0	719	0	0

0	100	0	0	0	0	0	0	0	0	0	0
0	0	0	0	0	0	0	0	0	0	0	0
0	0	0	0	0	0	0	0	0	0	0	0
0	411	0	0	0	0	0	0	0	0	0	0
0	0	0	0	0	0	137	35	0	0	253	0
0	0	0	0	0	0	0	0	0	0	0	0
0	0	0	0	0	0	0	0	0	0	0	0
0	0	0	0	0	0	0	0	0	0	0	0
0	0	0	0	0	0	0	0	0	0	0	0
0	0	0	0	0	0	0	0	0	0	0	0
0	0	0	0	0	0	0	0	0	0	0	0
0	0	0	0	0	0	0	0	0	0	0	0
0	0	0	0	0	0	0	0	0	0	0	0
0	0	0	0	0	0	0	0	0	0	0	0
0	0	210	0	0	0	0	0	0	0	0	0
0	0	0	0	0	0	0	0	0	0	0	0
0	0	0	0	0	0	0	0	0	0	0	0
0	0	0	0	0	0	0	0	0	0	0	0
0	0	0	0	0	0	0	0	0	0	0	0
0	90	0	0	68	0	0	0	0	0	0	0
0	0	52	0	0	0	0	0	0	0	0	0
0	0	0	0	0	0	0	0	0	0	0	0
0	0	0	0	0	0	0	0	0	0	0	0
0	0	50	0	0	0	0	0	0	0	0	0
0	0	0	0	0	0	0	0	0	0	0	0
0	0	56	0	0	0	0	0	0	0	0	0
0	0	0	0	0	0	0	0	0	0	0	0
0	0	0	0	0	0	0	0	0	0	0	0
0	0	0	0	0	0	0	0	0	0	0	0
0	0	0	0	0	0	0	0	0	0	0	0
0	0	0	0	0	0	0	0	0	0	82	0
1417	336	0	61	0	0	0	44568	8057	988	159	13624
0	0	0	0	0	0	0	0	0	0	0	1212
0	0	0	0	0	0	0	0	0	0	0	0
0	0	0	0	0	0	0	0	0	0	0	0
0	0	0	4664	0	0	2601	0	0	0	0	5990
0	0	0	0	0	0	0	0	0	0	0	0
18708	3281	595	36009	197	94	207657	135447	7292	6863	522	19427
166	0	0	0	0	87	6248	39	399	0	0	0
0	0	0	0	0	0	0	0	0	0	0	0
0	0	0	0	0	0	0	0	0	0	0	0
0	0	0	0	0	0	0	0	0	0	0	0
0	0	0	0	0	0	0	0	0	0	0	0
0	0	0	0	0	0	0	0	0	0	0	0
0	0	0	0	0	0	0	0	0	0	0	0
0	0	57	0	0	0	0	0	0	39	0	0
0	0	0	0	0	0	0	0	0	0	0	0
0	0	0	0	0	0	0	0	45	0	0	0
0	0	0	0	0	0	0	0	0	0	0	0
0	0	0	0	0	0	0	0	0	0	0	0
0	0	0	0	0	0	0	0	0	0	0	0
0	0	0	0	0	0	0	0	0	0	0	0
0	730	48	0	0	0	0	0	0	0	0	0
0	0	0	0	0	0	0	0	0	0	0	0
0	0	0	0	0	0	0	0	0	0	0	0
0	0	0	0	0	0	0	0	0	0	0	0
6952	0	0	0	0	0	0	168	0	101	0	0
0	0	0	0	0	0	0	0	0	0	0	0

0	0	0	0	0	0	0	4171	0	0	0	0
0	0	0	0	0	0	0	0	0	0	0	0
0	0	0	0	0	0	0	0	0	0	0	0
0	0	0	0	0	0	0	0	0	0	0	0
0	0	0	0	0	0	0	0	0	0	0	0
0	83	0	14388	0	0	0	0	0	0	0	0
0	0	0	0	0	0	0	0	0	0	0	0
0	0	0	0	0	0	0	0	0	0	0	0
0	0	0	0	0	0	0	0	0	0	0	0
0	4325	0	0	0	0	0	0	0	0	0	0
0	322	0	0	0	0	0	0	0	0	0	0
0	0	0	0	0	0	0	0	0	0	0	0
0	110	42	0	0	0	179142	28	59	0	175	0
0	0	0	0	0	0	0	0	0	0	0	0
0	0	0	0	65	0	0	0	0	42	0	30863
0	0	0	0	543	0	0	0	0	0	0	0
0	688	0	0	0	0	0	0	0	0	0	0
0	0	0	0	0	0	0	0	0	0	0	0
0	0	0	0	0	0	0	0	0	0	0	0
0	0	0	0	0	0	0	0	0	0	0	0
0	0	0	0	0	0	0	0	0	0	0	0
0	0	0	0	59	0	0	0	62	38	83	0
0	0	0	0	0	0	0	0	0	0	0	0
0	0	0	0	54	0	0	0	0	0	0	0
0	0	0	0	0	0	0	0	0	0	0	0
0	0	0	0	0	0	0	0	0	0	0	0
67	187	0	49	1849	0	75	475	503	6919	170	162
0	0	0	0	0	0	0	0	0	0	0	0
0	0	0	0	0	0	0	0	0	0	0	0
0	0	0	0	0	0	0	0	0	0	0	0
0	0	0	0	0	0	0	0	0	0	0	0
4144	0	0	0	0	0	0	209	0	119	0	0
0	0	0	0	0	0	0	0	0	0	0	0
0	0	0	0	0	0	0	0	0	2900	0	0
0	0	0	0	0	0	0	0	0	0	0	0
0	0	0	0	0	0	0	0	0	0	0	0
0	0	0	0	0	0	151	0	228	0	0	117
0	0	0	0	0	0	0	0	0	0	0	0
0	0	0	0	0	0	0	0	0	0	0	0
0	0	0	0	0	0	0	0	0	0	0	0
0	0	0	0	0	0	0	0	0	0	0	0
0	0	0	0	0	0	0	0	0	0	0	0
0	0	0	0	85	177	4654	0	5879	76	0	31763
61	0	0	0	0	0	0	0	0	0	0	0
0	0	0	0	0	0	76	0	67	0	0	563
0	0	0	0	0	0	0	0	0	0	0	0
56	0	0	0	0	0	0	0	0	0	0	0
0	0	0	0	0	0	0	0	0	0	0	0
0	0	0	0	0	0	0	0	0	0	0	0
0	0	0	0	0	0	0	0	0	0	0	0
0	0	0	0	0	0	0	0	0	0	0	0
0	0	0	0	0	0	0	0	0	0	0	0
0	0	0	2085	0	95	0	0	0	0	0	0
0	0	0	0	0	0	0	0	0	0	0	0
0	0	0	0	0	0	0	0	0	0	0	0
0	0	0	0	0	0	0	0	0	0	0	0
0	0	0	0	0	0	0	0	0	0	0	0
0	0	0	0	422	0	162	62	107	2166	0	0

0	0	0	0	0	0	0	0	0	0	0	0
0	0	0	0	0	0	0	0	0	0	0	0
0	0	0	0	0	0	0	0	0	0	0	0
0	0	0	0	0	0	0	0	0	0	0	0
0	0	0	0	0	0	0	0	0	0	0	0
0	0	0	0	0	0	0	0	0	0	0	676
0	0	0	0	0	0	0	0	0	0	0	623
0	0	0	0	0	93	0	0	0	0	0	11249
0	0	0	0	0	0	0	0	752	0	0	0
0	0	0	0	0	0	0	0	0	0	0	0
0	0	0	0	0	0	0	0	0	0	0	0
0	0	0	0	0	0	0	0	0	0	0	0
0	0	46	0	0	0	0	0	4950	0	0	0
0	0	0	0	0	0	0	0	0	0	0	0
0	0	0	0	0	0	0	0	0	0	0	0
0	0	0	0	0	0	0	0	0	0	0	0
0	0	0	0	0	0	0	0	0	0	0	0
0	0	0	0	120	0	66	0	0	0	0	0
0	0	0	0	0	0	0	0	0	0	0	0
0	0	0	0	0	0	0	0	0	0	0	0
0	0	0	0	0	0	0	0	0	0	0	0
0	0	0	0	0	0	0	0	0	0	0	0
0	0	0	0	0	0	0	0	0	0	0	23925
0	0	0	0	0	0	0	0	0	0	0	0
0	0	0	0	0	0	0	38	0	0	0	0
0	0	0	0	0	0	0	0	0	0	0	0
0	0	0	0	0	0	0	29	0	34	0	0
0	0	0	0	0	0	0	0	0	0	0	0
70	0	0	30923	0	0	0	0	0	58	0	31458
0	0	0	0	0	0	0	0	0	0	0	0
0	0	0	0	0	0	0	0	0	0	0	0
0	0	0	0	0	0	0	0	0	0	0	0
0	0	0	0	0	0	0	0	0	0	0	0
0	0	0	0	0	0	0	0	0	0	0	0
0	0	0	0	0	0	0	0	0	0	0	0
0	0	0	0	0	0	0	0	0	0	0	0
0	0	0	0	0	0	0	0	0	0	0	0
0	0	0	0	0	0	0	0	0	0	0	12619
0	0	0	0	0	0	0	0	0	0	0	0
0	0	0	0	0	0	0	0	0	0	0	0
0	0	0	0	0	0	0	0	0	0	0	0
0	0	0	57	0	0	0	0	2372	37	98	59
0	0	0	0	0	0	0	0	0	0	0	0
0	0	0	0	0	0	0	0	0	0	0	0
0	0	0	0	0	0	0	0	0	0	0	0
0	0	0	0	0	0	0	0	0	0	0	0
0	0	0	0	0	0	0	0	0	0	0	0
0	0	0	0	0	0	0	0	0	0	0	0
0	0	0	0	0	0	0	0	0	0	0	0
0	0	0	0	0	0	0	0	0	0	0	0
0	0	0	0	0	0	0	0	0	0	0	0
0	0	0	0	0	0	0	0	0	0	0	0
0	7199	0	0	1034	708	6184	439	837	11844	284	6542
0	0	0	0	0	0	0	0	0	0	0	0
0	0	0	0	0	0	286	134818	135	0	598	0
0	0	0	0	0	0	0	0	0	0	0	0
2664	715	0	0	0	81	0	232	0	247	0	0
0	0	0	0	0	0	0	0	0	0	0	0

0	0	0	0	0	0	0	0	0	0	0	0
0	0	0	0	0	0	0	0	0	0	0	0
0	0	0	0	0	0	0	0	0	0	0	0
0	0	0	0	0	0	0	0	0	0	0	0
0	0	0	0	0	0	0	0	0	0	0	0
0	938	0	0	0	0	0	0	0	0	0	0
0	0	0	0	0	0	0	0	0	0	0	0
0	0	0	0	0	0	0	0	0	0	0	0
0	0	0	0	0	0	0	0	0	0	0	0
0	0	0	0	0	0	0	0	0	0	0	0
0	0	0	0	0	0	0	0	0	0	0	0
0	0	0	0	0	0	0	0	0	0	0	0
20046	411	430	266	0	89	369	3294	257	852	654	0
0	0	0	0	0	0	0	0	0	0	0	0
0	0	0	2300	157	154	35787	58893	553	50676	429	34863
0	0	0	0	0	0	0	0	0	0	0	0
0	0	0	0	0	0	0	0	0	0	0	0
0	0	0	0	0	0	0	0	0	0	0	0
0	0	0	0	0	0	0	0	0	0	0	0
0	0	0	0	354	365	0	0	0	482	0	524
0	0	0	0	0	0	0	0	0	0	0	0
0	0	0	0	0	0	0	7267	0	4381	0	0
0	0	0	0	0	0	0	0	0	0	0	0
0	0	0	0	0	0	0	0	0	0	0	0
0	0	0	0	0	0	0	0	0	0	0	0
0	0	0	0	0	0	0	0	0	0	0	0
0	0	0	0	0	0	0	0	0	0	0	0
0	0	0	0	0	0	0	0	0	0	0	0
0	0	0	0	0	0	0	0	0	0	0	0
183	2184	105	62	3709	74	2150	713	1824	32367	1264	353
0	0	0	0	0	0	0	62	0	0	0	0
0	0	0	0	0	0	0	0	0	0	0	0
0	0	0	0	0	0	0	0	0	0	0	0
0	0	0	0	0	0	0	0	0	0	0	0
0	0	0	0	0	0	0	0	0	0	0	0
0	0	0	0	0	0	0	0	0	0	0	0
0	0	0	0	0	0	0	0	0	0	0	0
0	0	0	0	0	0	0	0	0	0	0	0
0	0	0	0	0	0	0	0	0	0	0	0
0	0	0	0	0	0	13533	47	0	0	0	0
0	0	0	0	0	0	0	0	0	0	0	0
0	0	0	0	0	0	0	0	0	0	0	0
0	0	0	0	0	0	0	0	0	0	0	0
0	0	0	0	0	0	0	0	0	0	0	0
0	0	0	0	0	0	0	0	0	0	0	0
0	0	0	0	0	0	0	0	0	0	0	0
300	104	0	0	0	0	0	0	0	0	0	0
0	0	0	0	0	0	0	0	0	0	0	0
0	0	0	0	0	0	0	0	0	0	0	0
0	0	0	0	0	0	0	0	0	0	0	0
135	0	52	0	0	0	0	0	0	0	0	0
0	0	0	0	0	0	0	0	0	0	0	0
130	0	0	0	0	0	0	0	0	0	0	0
0	0	0	0	0	0	0	0	0	0	0	0
0	0	0	0	0	0	0	0	0	0	0	0
0	0	0	0	0	0	65615	22	0	0	0	0
0	0	0	0	0	0	0	0	0	0	0	0
0	0	0	0	0	0	0	0	0	0	0	0

0	0	0	0	0	0	0	0	0	0	0	0
0	0	0	0	0	0	0	0	0	0	0	0
0	0	0	0	0	0	0	0	0	0	0	0
0	0	53	0	0	0	0	0	0	0	0	0
0	0	0	0	0	0	0	0	0	0	0	0
0	0	0	0	0	0	0	0	0	0	0	0
0	0	0	0	0	0	0	0	0	0	0	0
0	0	0	0	0	0	0	0	0	0	0	0
0	0	0	0	0	0	0	0	0	0	0	0
0	0	0	0	0	0	0	0	0	0	0	0
0	0	0	0	0	0	0	0	0	0	0	0
0	0	0	0	0	0	0	0	0	0	0	0
0	0	0	0	0	0	0	0	0	0	0	0
0	0	0	0	0	0	0	0	0	0	0	0
0	0	0	0	0	0	0	0	0	0	0	0
0	0	0	0	0	0	0	0	0	0	0	0
0	0	0	0	0	0	0	0	0	0	0	0
3233	0	0	0	0	87	0	72	0	229	0	0
0	0	0	0	0	0	0	0	0	0	0	0
0	0	0	0	0	0	0	0	0	0	0	0
0	0	0	0	0	0	0	0	0	0	0	0
0	0	0	0	0	0	0	0	0	0	0	0
56	0	0	0	70	0	32952	32	55	0	0	151
0	0	0	0	0	0	0	0	0	0	0	0
0	0	0	0	0	0	0	0	0	0	0	0
0	0	0	0	0	0	0	0	0	0	0	0
0	0	0	0	0	0	0	0	0	0	0	0
0	0	0	0	0	0	0	0	0	0	0	0
0	0	0	0	0	0	0	0	0	0	0	0
0	0	0	0	0	0	0	0	0	0	0	0
0	0	0	0	0	0	0	0	0	0	0	0
0	0	0	0	0	0	0	0	0	0	0	0
0	0	0	0	0	0	69	38	0	0	0	0
0	0	0	0	0	0	0	0	0	0	0	0
0	0	0	0	0	0	0	0	0	0	0	0
0	0	0	0	0	0	0	0	0	0	0	0
0	0	0	0	0	0	275	69	155	0	97	0
0	0	0	0	0	0	0	0	0	0	0	0
0	0	0	0	0	0	0	0	0	0	0	0
0	0	0	0	0	0	0	0	0	0	0	0
0	0	0	0	0	0	0	0	0	0	0	0
0	0	0	0	0	0	0	0	0	0	0	0
0	0	0	0	0	0	0	0	0	0	0	0
0	0	0	0	0	0	0	0	0	0	0	0
0	0	0	0	0	0	0	0	0	0	0	0
0	0	0	0	0	0	0	0	0	0	0	0
0	0	0	0	0	0	0	0	0	0	77	0
0	0	0	0	0	0	0	0	0	0	0	0
0	0	0	0	0	0	0	0	0	0	0	0
0	0	0	0	0	0	0	0	3448	1069	0	22050
0	0	0	0	0	0	0	0	0	0	0	0
0	0	50	0	0	0	0	0	0	0	0	0
0	0	0	0	0	0	0	0	0	5137	0	0
0	0	0	0	0	0	0	0	0	0	0	59
0	0	0	0	0	0	0	0	0	0	0	0
0	0	0	0	0	0	77	15870	0	0	74	0
0	0	0	0	0	0	0	0	0	0	0	0
0	0	0	0	0	0	0	0	0	0	0	0

0	0	0	0	0	0	0	0	0	0	0	0
0	0	0	0	0	0	0	0	0	0	0	0
0	0	0	0	0	0	0	0	0	0	0	0
0	0	0	0	0	0	0	0	0	0	0	0
0	0	0	0	0	0	0	0	0	0	0	0
0	0	0	0	0	0	0	0	0	0	0	0
0	0	0	0	0	0	94	0	0	0	0	0
0	0	0	0	0	0	0	0	0	0	0	0
0	0	0	0	0	84	0	0	0	0	0	0
0	0	0	0	0	0	0	0	0	0	0	0
0	0	0	0	0	0	0	0	0	0	0	0
0	0	0	0	0	0	0	0	0	0	0	0
0	0	0	0	0	0	0	0	0	0	0	0
0	0	0	0	0	0	0	0	0	0	0	61
0	0	0	0	0	0	0	0	0	0	0	27741
0	0	0	0	0	0	0	0	0	0	0	369
0	0	0	0	0	0	0	0	0	0	0	0
0	0	0	0	0	0	0	0	0	0	0	0
0	0	0	0	0	0	0	0	0	0	0	0
0	0	0	0	0	0	146	0	114	0	0	0
26126	788571	953671	806542	5611	22082	81318	19458	105657	18124	758	83662
0	0	0	0	0	0	0	0	0	0	0	0
0	0	0	0	0	0	0	0	0	0	0	0
59	0	0	0	135	184	151	411	67	804	174	0
0	0	0	0	0	0	0	0	0	0	0	0
0	0	0	0	0	0	0	0	0	0	0	0
413	0	52	20823	0	0	64	2646	5660	0	18656	0
0	0	0	0	0	0	0	0	0	0	0	0
0	0	0	0	0	0	0	0	0	0	0	0
0	0	0	0	0	0	0	0	0	0	0	0
0	0	0	0	61	0	43739	0	0	90	0	0
0	0	0	0	0	0	0	0	0	0	0	0
0	0	0	0	0	0	0	0	0	0	0	0
0	106	67	0	275	0	0	53	53	911	0	0

Table S4. The different degree of phylum level (p value) between the controls (n=88) and COVID-19 patients (n=48)

ID	Control.median	Control.mean	Control.se	COVID-19.median	COVID-19.mean	COVID-19.se	p-value	z-score	Sig_mark	q-value	fixp	fixps
Ascomycota	0.8503(0.7115,0.9292)	0.778291	0.023311	0.9233(0.8228,0.9635)	0.88236417	0.016966	0.0013474	-3.2057	**	0.003144	0.0013	** 0.0013
Basidiomycota	0.0782(0.026,0.1816)	0.141961	0.019192	0.0186(0.0063,0.0992)	0.07088092	0.014738	0.0010333	-3.2813	**	0.003144	0.001	** 0.001
Mucoromycota	0.002(0.0007,0.0159)	0.036631	0.014588	0.0009(0.0002,0.0043)	0.0062111	0.001883	0.0102163	-2.5684	*	0.014303	0.0102	* 0.0102
Zoopagomycota	<0.0001(<0.0001,<0.0001)	3.70E-04	1.57E-04	0.0002(<0.0001,0.0009)	0.00432333	0.001421	3.42E-06	-4.644	***	2.39E-05	<0.0001	***<0.0001
Rozellomycota	<0.0001(<0.0001,<0.0001)	0.001021	9.45E-04	<0.0001(<0.0001,<0.0001)	0	0	0.0037686	-2.8969	**	0.006595	0.0038	** 0.0038

Table S4. The different degree of genus level (p value) between the controls (n=88) and COVID-19 patients (n=48)

ID	Control.median	Control.mean	Control.se	COVID-19.median	COVID-19.mean	COVID-19.se	p-value	z-score	Sig_mark	q-value	fixp	fixps
Cryptococcus	0.0105(0.0017,0.044)	0.045024	0.008483	<0.0001(<0.0001,<0.0001)	4.10E-05	1.91E-05	3.65E-22	-9.6804	***	1.35E-19	<0.0001	***<0.0001
Diversispora	0.002(0.0006,0.0156)	0.036457	0.014585	0.0008(0.0002,0.0039)	0.005474	0.001791	0.0086361	-2.6261	**	0.04699	0.0086	** 0.0086
Arthrographis	0.0001(<0.0001,0.0009)	0.027897	0.015719	<0.0001(<0.0001,<0.0001)	1.32E-04	7.58E-05	1.39E-04	-3.81	***	0.003234	0.0001	***0.0001
Raffaelea	0.0007(0.0001,0.0025)	0.0284	0.013353	0.0016(0.0006,0.0054)	0.00478331	0.001399	0.0476792	-1.9802	*	0.143425	0.0477	* 0.0477
Milospium	<0.0001(<0.0001,0.0005)	0.014769	0.010143	<0.0001(<0.0001,<0.0001)	2.14E-04	1.54E-04	2.66E-06	-4.6952	***	1.23E-04	<0.0001	***<0.0001
Malassezia	0.0072(0.0014,0.0477)	0.037437	0.007326	0.0011(0.0003,0.0148)	0.02419129	0.008168	0.001973	-3.0943	**	0.015532	0.002	** 0.002
Cladosporium	0.0047(0.0011,0.0341)	0.035333	0.0069	0.0016(0.0005,0.0184)	0.02383048	0.007903	0.0372201	-2.0833	*	0.125426	0.0372	* 0.0372
Trigonopsis	<0.0001(<0.0001,<0.0001)	0.010833	0.010658	<0.0001(<0.0001,<0.0001)	4.64E-05	4.24E-05	0.0227775	-2.2771	*	0.089656	0.0228	* 0.0228
Phaeosphaeria	<0.0001(<0.0001,0.0003)	0.005452	0.003386	<0.0001(<0.0001,<0.0001)	2.37E-04	1.40E-04	0.0038712	-2.8885	**	0.025578	0.0039	** 0.0039
Diaporthe	<0.0001(<0.0001,0.0003)	0.004012	0.003406	<0.0001(<0.0001,<0.0001)	9.56E-06	9.56E-06	1.08E-08	-5.717	***	1.34E-06	<0.0001	***<0.0001
Naganishia	<0.0001(<0.0001,<0.0001)	0.003045	0.002795	<0.0001(<0.0001,<0.0001)	5.71E-05	4.42E-05	0.0040622	-2.8733	**	0.026369	0.0041	** 0.0041
Cercospora	<0.0001(<0.0001,<0.0001)	0.002359	0.002086	<0.0001(<0.0001,<0.0001)	0	0	2.31E-05	-4.2331	***	7.75E-04	<0.0001	***<0.0001
Apiotrichum	<0.0001(<0.0001,0.0001)	0.002005	8.40E-04	<0.0001(<0.0001,<0.0001)	1.25E-04	7.92E-05	8.05E-04	-3.3512	***	0.008757	0.0008	***0.0008
Stagonosporopsis	<0.0001(<0.0001,<0.0001)	0.001819	0.001317	<0.0001(<0.0001,<0.0001)	0	0	1.51E-05	-4.3278	***	5.57E-04	<0.0001	***<0.0001
Vishniacozyma	<0.0001(<0.0001,<0.0001)	0.001872	0.00149	<0.0001(<0.0001,<0.0001)	2.79E-04	2.22E-04	0.0152317	-2.4268	*	0.071338	0.0152	* 0.0152
Wallemia	<0.0001(<0.0001,0.0003)	0.001696	7.80E-04	<0.0001(<0.0001,<0.0001)	1.92E-04	1.38E-04	0.0013901	-3.1967	**	0.012858	0.0014	** 0.0014
Pyrenochaetopsis	<0.0001(<0.0001,<0.0001)	0.001269	0.001048	<0.0001(<0.0001,<0.0001)	1.85E-06	1.85E-06	0.0023226	-3.0455	**	0.017538	0.0023	** 0.0023
Rozellomycota_gen_Incertae_sedis	<0.0001(<0.0001,<0.0001)	0.001021	9.45E-04	<0.0001(<0.0001,<0.0001)	0	0	0.0037686	-2.8969	**	0.025353	0.0038	** 0.0038
Botrytis	<0.0001(<0.0001,0.0001)	0.001138	7.44E-04	<0.0001(<0.0001,<0.0001)	3.91E-04	3.34E-04	0.0018394	-3.115	**	0.015532	0.0018	** 0.0018
Recurvomyces	<0.0001(<0.0001,<0.0001)	6.90E-04	6.74E-04	<0.0001(<0.0001,<0.0001)	0	0	0.0324831	-2.1384	*	0.113384	0.0325	* 0.0325
Lentinus	<0.0001(<0.0001,<0.0001)	6.59E-04	6.04E-04	<0.0001(<0.0001,<0.0001)	1.08E-06	1.08E-06	0.0390097	-2.0641	*	0.130032	0.039	* 0.039
Knufia	<0.0001(<0.0001,<0.0001)	6.80E-04	4.19E-04	<0.0001(<0.0001,<0.0001)	4.31E-05	4.16E-05	6.14E-04	-3.4255	***	0.008109	0.0006	***0.0006
Flavodon	<0.0001(<0.0001,<0.0001)	5.97E-04	5.55E-04	<0.0001(<0.0001,<0.0001)	4.96E-06	4.96E-06	0.044586	-2.0085	*	0.140605	0.0446	* 0.0446
Thielaviopsis	<0.0001(<0.0001,<0.0001)	5.74E-04	3.57E-04	<0.0001(<0.0001,<0.0001)	0	0	0.0026097	-3.0103	**	0.018933	0.0026	** 0.0026
Udeniomyces	<0.0001(<0.0001,<0.0001)	5.93E-04	2.33E-04	<0.0001(<0.0001,<0.0001)	2.40E-05	1.32E-05	0.001895	-3.1062	**	0.015532	0.0019	** 0.0019
Sporobolomyces	<0.0001(<0.0001,<0.0001)	8.74E-04	5.71E-04	<0.0001(<0.0001,<0.0001)	3.21E-04	2.83E-04	0.0190929	-2.3437	*	0.0812	0.0191	* 0.0191
Dothidea	<0.0001(<0.0001,<0.0001)	5.41E-04	4.22E-04	<0.0001(<0.0001,<0.0001)	0	0	0.0159476	-2.4101	*	0.072691	0.0159	* 0.0159
Yarrowia	<0.0001(<0.0001,<0.0001)	7.83E-04	3.98E-04	<0.0001(<0.0001,<0.0001)	2.79E-04	1.97E-04	0.003703	-2.9024	**	0.025353	0.0037	** 0.0037
Didymella	<0.0001(<0.0001,<0.0001)	5.32E-04	3.39E-04	<0.0001(<0.0001,<0.0001)	2.83E-05	1.78E-05	0.0266866	-2.2161	*	0.100756	0.0267	* 0.0267
Pseudoramichloridium	<0.0001(<0.0001,<0.0001)	4.80E-04	4.72E-04	<0.0001(<0.0001,<0.0001)	0	0	0.0463617	-1.9921	*	0.140605	0.0464	* 0.0464
Coprinopsis	<0.0001(<0.0001,<0.0001)	4.70E-04	3.89E-04	<0.0001(<0.0001,<0.0001)	1.60E-06	1.60E-06	0.0047112	-2.8261	**	0.030054	0.0047	** 0.0047
Golubevia	<0.0001(<0.0001,<0.0001)	4.16E-04	2.65E-04	<0.0001(<0.0001,<0.0001)	5.02E-06	5.02E-06	2.56E-04	-3.6565	***	0.004731	0.0003	***0.0003
Ceriporia	<0.0001(<0.0001,<0.0001)	4.30E-04	3.13E-04	<0.0001(<0.0001,<0.0001)	4.71E-05	4.60E-05	0.0019512	-3.0976	**	0.015532	0.002	** 0.002
Peniophorella	<0.0001(<0.0001,<0.0001)	3.80E-04	3.62E-04	<0.0001(<0.0001,<0.0001)	0	0	0.0037685	-2.8969	**	0.025353	0.0038	** 0.0038
Toxicocladosporium	<0.0001(<0.0001,<0.0001)	3.48E-04	2.84E-04	<0.0001(<0.0001,<0.0001)	0	0	0.0159476	-2.4101	*	0.072691	0.0159	* 0.0159

Typhula	<0.0001(<0.0001,<0.0001)	3.30E-04	2.51E-04	<0.0001(<0.0001,<0.0001)	0	0	5.79E-04	-3.4413	***	0.007934	0.0006	***0.0006
Carlosrosaea	<0.0001(<0.0001,<0.0001)	3.16E-04	3.10E-04	<0.0001(<0.0001,<0.0001)	0	0	0.0463617	-1.9921	*	0.140605	0.0464	* 0.0464
Ciliophora	<0.0001(<0.0001,<0.0001)	2.97E-04	1.70E-04	<0.0001(<0.0001,<0.0001)	0	0	0.0012386	-3.2298	**	0.012386	0.0012	** 0.0012
Parastagonosporella	<0.0001(<0.0001,<0.0001)	2.92E-04	2.76E-04	<0.0001(<0.0001,<0.0001)	0	0	0.0111558	-2.5378	*	0.055779	0.0112	* 0.0112
Stemphylium	<0.0001(<0.0001,<0.0001)	2.91E-04	1.72E-04	<0.0001(<0.0001,<0.0001)	0	0	0.0077887	-2.6611	**	0.043014	0.0078	** 0.0078
Hypoxylon	<0.0001(<0.0001,<0.0001)	2.73E-04	2.56E-04	<0.0001(<0.0001,<0.0001)	0	0	0.0111558	-2.5378	*	0.055779	0.0112	* 0.0112
Tilletia	<0.0001(<0.0001,<0.0001)	2.71E-04	1.58E-04	<0.0001(<0.0001,<0.0001)	0	0	3.93E-04	-3.5445	***	0.005821	0.0004	***0.0004
Tulostoma	<0.0001(<0.0001,<0.0001)	2.34E-04	2.28E-04	<0.0001(<0.0001,<0.0001)	0	0	0.0324831	-2.1384	*	0.113384	0.0325	* 0.0325
Phlebiopsis	<0.0001(<0.0001,<0.0001)	2.20E-04	1.51E-04	<0.0001(<0.0001,<0.0001)	0	0	0.0111554	-2.5378	*	0.055779	0.0112	* 0.0112
Nigrograna	<0.0001(<0.0001,<0.0001)	2.17E-04	1.66E-04	<0.0001(<0.0001,<0.0001)	0	0	0.0324831	-2.1384	*	0.113384	0.0325	* 0.0325
Xylocoremium	<0.0001(<0.0001,<0.0001)	2.04E-04	2.00E-04	<0.0001(<0.0001,<0.0001)	0	0	0.0324819	-2.1384	*	0.113384	0.0325	* 0.0325
Auricularia	<0.0001(<0.0001,<0.0001)	2.57E-04	2.35E-04	<0.0001(<0.0001,<0.0001)	5.31E-05	5.19E-05	0.0232408	-2.2695	*	0.090517	0.0232	* 0.0232
Verrucaria	<0.0001(<0.0001,<0.0001)	2.06E-04	1.49E-04	<0.0001(<0.0001,<0.0001)	6.63E-06	6.63E-06	0.0106017	-2.5556	*	0.055779	0.0106	* 0.0106
Preussia	<0.0001(<0.0001,<0.0001)	1.69E-04	1.65E-04	<0.0001(<0.0001,<0.0001)	0	0	0.0324831	-2.1384	*	0.113384	0.0325	* 0.0325
Calycina	<0.0001(<0.0001,<0.0001)	1.67E-04	1.63E-04	<0.0001(<0.0001,<0.0001)	0	0	0.0463617	-1.9921	*	0.140605	0.0464	* 0.0464
Derxomyces	<0.0001(<0.0001,<0.0001)	1.65E-04	1.59E-04	<0.0001(<0.0001,<0.0001)	0	0	0.0324831	-2.1384	*	0.113384	0.0325	* 0.0325
Lecanicillium	<0.0001(<0.0001,<0.0001)	1.22E-04	1.15E-04	<0.0001(<0.0001,<0.0001)	0	0	0.0463617	-1.9921	*	0.140605	0.0464	* 0.0464
Debaryomyces	<0.0001(<0.0001,<0.0001)	4.00E-04	3.35E-04	<0.0001(<0.0001,<0.0001)	4.78E-04	3.94E-04	3.50E-04	-3.575	***	0.005453	0.0004	***0.0004
Coprinellus	<0.0001(<0.0001,<0.0001)	0.001536	9.20E-04	<0.0001(<0.0001,<0.0001)	0.00179129	0.001784	0.0014972	-3.1752	**	0.013189	0.0015	** 0.0015
Issatchenkia	<0.0001(<0.0001,<0.0001)	1.86E-06	1.10E-06	<0.0001(<0.0001,<0.0001)	2.87E-04	2.58E-04	0.0019304	-3.1007	**	0.015532	0.0019	** 0.0019
Wickerhamiella	<0.0001(<0.0001,<0.0001)	3.58E-05	3.06E-05	<0.0001(<0.0001,<0.0001)	3.42E-04	2.49E-04	0.0413845	-2.0397	*	0.134318	0.0414	* 0.0414
Golovinomyces	<0.0001(<0.0001,<0.0001)	6.92E-05	6.85E-05	<0.0001(<0.0001,<0.0001)	3.80E-04	3.72E-04	0.0413845	-2.0397	*	0.134318	0.0414	* 0.0414
Didymocyrtis	<0.0001(<0.0001,<0.0001)	1.15E-06	1.15E-06	<0.0001(<0.0001,<0.0001)	3.14E-04	3.07E-04	0.0125736	-2.4956	*	0.06083	0.0126	* 0.0126
Venturiaceae_gen_Incertae_sedis	<0.0001(<0.0001,<0.0001)	0	0	<0.0001(<0.0001,<0.0001)	3.35E-04	3.31E-04	0.006324	-2.7305	**	0.036002	0.0063	** 0.0063
Periconia	<0.0001(<0.0001,0.0004)	8.14E-04	2.84E-04	0.0002(<0.0001,0.0022)	0.00116115	2.40E-04	0.0404357	-2.0493	*	0.133582	0.0404	* 0.0404
Neodevriesia	<0.0001(<0.0001,<0.0001)	2.50E-07	2.50E-07	<0.0001(<0.0001,<0.0001)	5.04E-04	3.57E-04	0.0320327	-2.144	*	0.113384	0.032	* 0.032
Pseudoveronaea	<0.0001(<0.0001,<0.0001)	5.99E-06	5.24E-06	<0.0001(<0.0001,<0.0001)	6.14E-04	3.93E-04	3.12E-04	-3.6052	***	0.005435	0.0003	***0.0003
Agaricus	<0.0001(<0.0001,<0.0001)	0	0	<0.0001(<0.0001,<0.0001)	7.36E-04	7.32E-04	0.0021705	-3.0659	**	0.016731	0.0022	** 0.0022
Dendryphiella	<0.0001(<0.0001,<0.0001)	0	0	<0.0001(<0.0001,<0.0001)	8.12E-04	7.95E-04	7.45E-04	-3.3723	***	0.008358	0.0007	***0.0007
Hortaea	<0.0001(<0.0001,<0.0001)	0	0	<0.0001(<0.0001,<0.0001)	8.41E-04	7.89E-04	7.45E-04	-3.3723	***	0.008358	0.0007	***0.0007
Heitmania	<0.0001(<0.0001,0.0003)	3.85E-04	9.57E-05	0.0003(<0.0001,0.0014)	0.00123465	3.77E-04	6.64E-04	-3.4041	***	0.008358	0.0007	***0.0007
Nakaseomyces	<0.0001(<0.0001,<0.0001)	6.82E-07	6.82E-07	<0.0001(<0.0001,<0.0001)	9.26E-04	9.11E-04	0.0013514	-3.2048	**	0.012821	0.0014	** 0.0014
Diutina	<0.0001(<0.0001,<0.0001)	1.41E-05	1.12E-05	<0.0001(<0.0001,<0.0001)	9.82E-04	6.05E-04	1.40E-04	-3.8084	***	0.003234	0.0001	***0.0001
Dipodascus	<0.0001(<0.0001,<0.0001)	7.38E-05	1.90E-05	<0.0001(<0.0001,<0.0001)	0.00106213	0.001054	0.0024996	-3.0234	**	0.018497	0.0025	** 0.0025
Ustilaginoidea	<0.0001(<0.0001,<0.0001)	1.03E-05	4.29E-06	<0.0001(<0.0001,0.0002)	0.00105854	5.32E-04	2.07E-06	-4.7467	***	1.09E-04	<0.0001	***<0.0001
Fantasmomyces	<0.0001(<0.0001,0.0005)	0.002768	0.00109	0.0008(0.0001,0.0047)	0.00391148	9.31E-04	2.11E-04	-3.7053	***	0.004339	0.0002	***0.0002
Sporothrix	<0.0001(<0.0001,<0.0001)	1.07E-05	1.03E-05	<0.0001(<0.0001,<0.0001)	0.00125642	3.94E-04	8.39E-07	-4.926	***	6.21E-05	<0.0001	***<0.0001
Peniophora	<0.0001(<0.0001,<0.0001)	5.25E-04	2.49E-04	<0.0001(<0.0001,<0.0001)	0.00199715	0.001831	0.0207069	-2.3133	*	0.086085	0.0207	* 0.0207
Starmerella	<0.0001(<0.0001,<0.0001)	3.64E-06	1.37E-06	<0.0001(<0.0001,0.0001)	0.00148767	0.001008	1.43E-06	-4.8211	***	8.80E-05	<0.0001	***<0.0001
Henningsia	<0.0001(<0.0001,<0.0001)	3.64E-07	3.64E-07	<0.0001(<0.0001,<0.0001)	0.00162858	0.001584	5.12E-05	-4.0499	***	0.001459	<0.0001	***<0.0001
Hannaella	<0.0001(<0.0001,<0.0001)	0.001014	9.75E-04	<0.0001(<0.0001,0.0002)	0.00324302	0.002681	3.54E-04	-3.5724	***	0.005453	0.0004	***0.0004
Angustimassarina	0.0001(<0.0001,0.001)	0.002873	0.001251	0.001(0.0002,0.0028)	0.00510573	0.002807	0.0062543	-2.7341	**	0.036002	0.0063	** 0.0063
Leucosporidium	<0.0001(<0.0001,<0.0001)	5.74E-05	5.15E-05	<0.0001(<0.0001,<0.0001)	0.00239935	0.002319	0.0444032	-2.0103	*	0.140605	0.0444	* 0.0444

Aureobasidium	<0.0001(<0.0001,<0.0001)	1.61E-04	1.32E-04	<0.0001(<0.0001,<0.0001)	0.00252923	0.001913	0.0493715	-1.9654	*	0.147318	0.0494	* 0.0494
Ramularia	<0.0001(<0.0001,<0.0001)	7.05E-07	7.05E-07	<0.0001(<0.0001,<0.0001)	0.00285081	0.002808	0.0013514	-3.2048	**	0.012821	0.0014	** 0.0014
Pichia	<0.0001(<0.0001,<0.0001)	5.47E-05	3.59E-05	<0.0001(<0.0001,0.0002)	0.00318777	0.001725	1.59E-04	-3.7765	***	0.003461	0.0002	***0.0002
Nigrospora	<0.0001(<0.0001,<0.0001)	9.60E-04	6.06E-04	<0.0001(<0.0001,0.0001)	0.00466638	0.003759	0.0134453	-2.4718	*	0.063779	0.0134	* 0.0134
Zoophthora	<0.0001(<0.0001,<0.0001)	3.70E-04	1.57E-04	0.0002(<0.0001,0.0009)	0.00432333	0.001421	3.42E-06	-4.644	***	1.40E-04	<0.0001	***<0.0001
Gibberella	<0.0001(<0.0001,<0.0001)	1.52E-05	7.26E-06	<0.0001(<0.0001,0.0016)	0.01443771	0.006146	4.19E-11	-6.5973	***	7.75E-09	<0.0001	***<0.0001
Simplicillium	0.0007(0.0003,0.0084)	0.01024	0.002164	0.004(0.0009,0.04)	0.0275095	0.005561	0.0014572	-3.1831	**	0.01315	0.0015	** 0.0015
Saccharomyces	<0.0001(<0.0001,0.0004)	0.001739	6.34E-04	0.0005(<0.0001,0.0084)	0.02640275	0.013063	4.89E-04	-3.4868	***	0.006956	0.0005	***0.0005
Hydnobolites	<0.0001(<0.0001,0.0001)	8.14E-04	5.91E-04	0.0002(<0.0001,0.0008)	0.03087802	0.019833	5.04E-05	-4.0539	***	0.001459	<0.0001	***<0.0001
Candida	0.0005(0.0001,0.0013)	0.005989	0.002359	0.004(0.0013,0.015)	0.05441221	0.022181	1.93E-07	-5.206	***	1.78E-05	<0.0001	***<0.0001
Acrodictys	0.0726(0.0114,0.3599)	0.200955	0.02619	0.128(0.0342,0.6571)	0.30144798	0.045429	0.0318034	-2.1469	*	0.113384	0.0318	* 0.0318

Table S4. The different degree of species level (p value) between the controls (n=88) and COVID-19 patients (n=48)

ID	Control.median	Control.mean	Control.se	COVID-19.median	COVID-19.mean	COVID-19.se	p-value	z-score	Sig_mark	q-value	fixp	fixps
Acrodictys_fluminicola	0.0726(0.0114,0.3599)	0.200955	0.02619	0.128(0.0342,0.6571)	0.30144798	0.045429	0.0318034	-2.1469	*	0.113104	0.0318	* 0.0318
Zanclospora_jonesii	0.0667(0.0146,0.3238)	0.215283	0.028571	0.0188(0.0095,0.0896)	0.14840617	0.038729	0.034467	-2.1146	*	0.117881	0.0345	* 0.0345
Cryptococcus_longus	0.0105(0.0017,0.044)	0.045024	0.008483	<0.0001(<0.0001,<0.0001)	2.62E-05	1.80E-05	2.52E-22	-9.7184	***	1.46E-19	<0.0001	***<0.0001
Diversispora_spurca	0.002(0.0006,0.0156)	0.036457	0.014585	0.0008(0.0002,0.0039)	0.005474	0.001791	0.0086361	-2.6261	**	0.049917	0.0086	** 0.0086
Malassezia_restricta	0.0048(0.0007,0.0342)	0.028644	0.005607	0.0008(0.0003,0.0086)	0.01744896	0.005978	0.0104907	-2.5592	*	0.058619	0.0105	* 0.0105
Raffaelea_lauricola	0.0007(0.0001,0.0024)	0.028395	0.013353	0.0016(0.0006,0.0054)	0.00478331	0.001399	0.0466646	-1.9893	*	0.143469	0.0467	* 0.0467
Arthrographis_grakistii	0.0001(<0.0001,0.0009)	0.027897	0.015719	<0.0001(<0.0001,<0.0001)	1.32E-04	7.58E-05	1.39E-04	-3.81	***	0.004017	0.0001	***0.0001
Simplicillium_sympodiophorum	0.0007(0.0003,0.0084)	0.010222	0.002164	0.004(0.0009,0.04)	0.0275095	0.005561	0.0014572	-3.1831	**	0.015892	0.0015	** 0.0015
Candida_albicans	<0.0001(<0.0001,<0.0001)	1.05E-04	3.37E-05	0.0012(0.0001,0.0048)	0.04067365	0.021616	1.46E-10	-6.4096	***	2.81E-08	<0.0001	***<0.0001
Cladosporium_kenpeggii	0.0008(0.0002,0.0065)	0.014341	0.004126	0.0002(<0.0001,0.0016)	0.00614894	0.002416	0.005339	-2.7858	**	0.037634	0.0053	** 0.0053
Hydnobolites_roseus	<0.0001(<0.0001,0.0001)	8.14E-04	5.91E-04	0.0002(<0.0001,0.0008)	0.03087802	0.019833	5.04E-05	-4.0539	***	0.001816	<0.0001	***<0.0001
Saccharomyces_cerevisiae	<0.0001(<0.0001,0.0004)	0.001739	6.34E-04	0.0005(<0.0001,0.0084)	0.02640275	0.013063	4.89E-04	-3.4868	***	0.008562	0.0005	***0.0005
Milospium_graphideorum	<0.0001(<0.0001,0.0005)	0.014769	0.010143	<0.0001(<0.0001,<0.0001)	2.14E-04	1.54E-04	2.66E-06	-4.6952	***	1.71E-04	<0.0001	***<0.0001
Trigonopsis_californica	<0.0001(<0.0001,<0.0001)	0.010833	0.010658	<0.0001(<0.0001,<0.0001)	0	0	0.0018012	-3.1212	**	0.01833	0.0018	** 0.0018
Cladosporium_spe_Incertae_sedis	0.0004(0.0001,0.0015)	0.006696	0.002514	0.0001(<0.0001,0.0005)	0.00701969	0.004617	0.0049147	-2.8126	**	0.03507	0.0049	** 0.0049
Gibberella_fujikuroi	<0.0001(<0.0001,<0.0001)	1.46E-05	7.18E-06	<0.0001(<0.0001,0.0013)	0.01419179	0.006151	3.14E-11	-6.6397	***	9.08E-09	<0.0001	***<0.0001
Cladosporium_cladosporioides	0.0005(0.0001,0.0031)	0.005654	0.001899	0.0002(<0.0001,0.0014)	0.00345388	0.001263	0.0305779	-2.1625	*	0.113104	0.0306	* 0.0306
Malassezia_arunalokei	<0.0001(<0.0001,0.0005)	0.005199	0.003619	<0.0001(<0.0001,0.0001)	0.00249294	0.001182	0.0149439	-2.4337	*	0.073826	0.0149	* 0.0149
Candida_spe_Incertae_sedis	<0.0001(<0.0001,0.0002)	7.36E-04	4.15E-04	0.0001(<0.0001,0.0007)	0.00944452	0.005809	0.010687	-2.5528	*	0.058619	0.0107	* 0.0107
Angustimassarina_camporesii	0.0001(<0.0001,0.001)	0.002873	0.001251	0.001(0.0002,0.0028)	0.00510573	0.002807	0.0062543	-2.7341	**	0.039736	0.0063	** 0.0063
Phaeosphaeria_oryzae	<0.0001(<0.0001,0.0003)	0.005041	0.003375	<0.0001(<0.0001,<0.0001)	1.39E-04	1.06E-04	0.0024174	-3.0335	**	0.021171	0.0024	** 0.0024
Fantasmomyces_hyalinus	<0.0001(<0.0001,0.0005)	0.002768	0.00109	0.0008(0.0001,0.0047)	0.00391148	9.31E-04	2.11E-04	-3.7053	***	0.005305	0.0002	***0.0002
Malassezia_globosa	0.0002(<0.0001,0.0016)	0.001823	3.85E-04	<0.0001(<0.0001,0.0001)	0.00383756	0.003433	4.35E-05	-4.0881	***	0.001796	<0.0001	***<0.0001
Ophiostoma_acarorum	<0.0001(<0.0001,<0.0001)	0.003579	0.00331	<0.0001(<0.0001,<0.0001)	2.00E-06	2.00E-06	2.65E-06	-4.6964	***	1.71E-04	<0.0001	***<0.0001
Acremonium_alternatum	<0.0001(<0.0001,<0.0001)	0.003542	0.003526	<0.0001(<0.0001,<0.0001)	1.00E-06	1.00E-06	0.0184111	-2.3572	*	0.078431	0.0184	* 0.0184
Diaporthe_kochmanii	<0.0001(<0.0001,0.0001)	0.003277	0.003172	<0.0001(<0.0001,<0.0001)	0	0	9.79E-06	-4.4218	***	5.14E-04	<0.0001	***<0.0001
Nigrospora_oryzae	<0.0001(<0.0001,<0.0001)	5.82E-04	4.85E-04	<0.0001(<0.0001,0.0001)	0.00466638	0.003759	2.39E-04	-3.6738	***	0.005474	0.0002	***0.0002
Aspergillus_amstelodami	<0.0001(<0.0001,0.0003)	0.002515	0.001147	<0.0001(<0.0001,<0.0001)	8.07E-04	4.16E-04	0.0073878	-2.6788	**	0.045915	0.0074	** 0.0074
Naganishia_albida	<0.0001(<0.0001,<0.0001)	0.002861	0.002793	<0.0001(<0.0001,<0.0001)	4.33E-05	4.23E-05	0.0225472	-2.281	*	0.08952	0.0225	* 0.0225
Candida_parapsilosis	<0.0001(<0.0001,0.0001)	0.002108	0.001243	<0.0001(<0.0001,<0.0001)	0.00139577	7.08E-04	0.0096825	-2.587	**	0.05541	0.0097	** 0.0097

Zoophthora_radicans	<0.0001(<0.0001,<0.0001)	3.70E-04	1.57E-04	0.0002(<0.0001,0.0009)	0.00432333	0.001421	3.42E-06	-4.644	***	1.98E-04	<0.0001	***<0.0001
Coprinellus_ovatus	<0.0001(<0.0001,<0.0001)	0.001533	9.20E-04	<0.0001(<0.0001,<0.0001)	0.00179129	0.001784	0.0030157	-2.9661	**	0.024551	0.003	** 0.003
Trametes_cubensis	<0.0001(<0.0001,<0.0001)	0.002466	0.002341	<0.0001(<0.0001,<0.0001)	0	0	0.0054254	-2.7806	**	0.037782	0.0054	** 0.0054
Filobasidium_oeirense	<0.0001(<0.0001,<0.0001)	1.76E-04	1.00E-04	<0.0001(<0.0001,0.0002)	0.00411021	0.002242	0.0147796	-2.4377	*	0.073643	0.0148	* 0.0148
Cercospora_canescens	<0.0001(<0.0001,<0.0001)	0.002359	0.002086	<0.0001(<0.0001,<0.0001)	0	0	2.31E-05	-4.2331	***	0.00111	<0.0001	***<0.0001
Apiotrichum_domesticum	<0.0001(<0.0001,0.0001)	0.002005	8.40E-04	<0.0001(<0.0001,<0.0001)	8.30E-05	6.48E-05	1.92E-04	-3.7296	***	0.005039	0.0002	***0.0002
Malassezia_sympodialis	<0.0001(<0.0001,0.0002)	0.001685	5.30E-04	<0.0001(<0.0001,<0.0001)	2.49E-04	1.54E-04	2.77E-05	-4.192	***	0.00123	<0.0001	***<0.0001
Trametes_trogii	<0.0001(<0.0001,<0.0001)	0.001773	0.001756	<0.0001(<0.0001,<0.0001)	0	0	0.0159476	-2.4101	*	0.074941	0.0159	* 0.0159
Ramularia_coryli	<0.0001(<0.0001,<0.0001)	7.05E-07	7.05E-07	<0.0001(<0.0001,<0.0001)	0.00285081	0.002808	0.0013514	-3.2048	**	0.015022	0.0014	** 0.0014
Aureobasidium_pullulans	<0.0001(<0.0001,<0.0001)	1.51E-04	1.32E-04	<0.0001(<0.0001,<0.0001)	0.00252923	0.001913	0.003382	-2.9307	**	0.02715	0.0034	** 0.0034
Hannaella_sinensis	<0.0001(<0.0001,<0.0001)	6.93E-06	4.31E-06	<0.0001(<0.0001,<0.0001)	0.00274702	0.002682	0.0019648	-3.0955	**	0.018617	0.002	** 0.002
Leucosporidium_golubevii	<0.0001(<0.0001,<0.0001)	5.74E-05	5.15E-05	<0.0001(<0.0001,<0.0001)	0.00239935	0.002319	0.0444032	-2.0103	*	0.1433	0.0444	* 0.0444
Botrytis_cinerea	<0.0001(<0.0001,0.0001)	0.001138	7.44E-04	<0.0001(<0.0001,<0.0001)	3.91E-04	3.34E-04	0.0018394	-3.115	**	0.01833	0.0018	** 0.0018
Stagonosporopsis_citrulli	<0.0001(<0.0001,<0.0001)	0.001314	0.001245	<0.0001(<0.0001,<0.0001)	0	0	0.0159476	-2.4101	*	0.074941	0.0159	* 0.0159
Wallemia_mellicola	<0.0001(<0.0001,0.0001)	0.001176	6.40E-04	<0.0001(<0.0001,<0.0001)	1.65E-04	1.38E-04	4.44E-04	-3.5121	***	0.008029	0.0004	***0.0004
Pyrenochaetopsis_microspora	<0.0001(<0.0001,<0.0001)	0.001058	0.00103	<0.0001(<0.0001,<0.0001)	1.85E-06	1.85E-06	0.0146502	-2.4409	*	0.073633	0.0147	* 0.0147
Heitmania_litseae	<0.0001(<0.0001,0.0003)	3.85E-04	9.57E-05	0.0003(<0.0001,0.0014)	0.00123465	3.77E-04	6.64E-04	-3.4041	***	0.010259	0.0007	***0.0007
Rozellomycota_spe_Incertae_sedis	<0.0001(<0.0001,<0.0001)	0.001021	9.45E-04	<0.0001(<0.0001,<0.0001)	0	0	0.0037686	-2.8969	**	0.028289	0.0038	** 0.0038
Cladosporium_coloradense	<0.0001(<0.0001,<0.0001)	1.00E-03	8.01E-04	<0.0001(<0.0001,<0.0001)	1.73E-06	1.73E-06	5.11E-04	-3.4747	***	0.008695	0.0005	***0.0005
Cutaneotrichosporon_cutaneum	<0.0001(<0.0001,<0.0001)	9.59E-06	8.19E-06	<0.0001(<0.0001,<0.0001)	0.00174694	0.001588	8.56E-04	-3.3341	***	0.011492	0.0009	***0.0009
Penicillium_hirsutum	<0.0001(<0.0001,<0.0001)	2.71E-04	2.39E-04	<0.0001(<0.0001,<0.0001)	0.00117021	7.08E-04	0.0018189	-3.1183	**	0.01833	0.0018	** 0.0018
Pichia_kluyveri	<0.0001(<0.0001,<0.0001)	4.33E-06	4.33E-06	<0.0001(<0.0001,0.0001)	0.00162748	7.33E-04	2.48E-08	-5.5749	***	3.58E-06	<0.0001	***<0.0001
Henningsia_resupinata	<0.0001(<0.0001,<0.0001)	3.64E-07	3.64E-07	<0.0001(<0.0001,<0.0001)	0.00162858	0.001584	5.12E-05	-4.0499	***	0.001816	<0.0001	***<0.0001
[Candida]_pseudolambica	<0.0001(<0.0001,<0.0001)	5.23E-07	5.23E-07	<0.0001(<0.0001,<0.0001)	0.00155729	0.001401	5.34E-05	-4.0402	***	0.001816	<0.0001	***<0.0001
Starmerella_etchellsii	<0.0001(<0.0001,<0.0001)	3.64E-06	1.37E-06	<0.0001(<0.0001,0.0001)	0.00148767	0.001008	1.43E-06	-4.8211	***	1.38E-04	<0.0001	***<0.0001
Symmetrospora_spe_Incertae_sedis	<0.0001(<0.0001,<0.0001)	9.28E-06	9.28E-06	<0.0001(<0.0001,<0.0001)	0.00136994	0.001367	0.0355446	-2.1021	*	0.120145	0.0355	* 0.0355
Sporothrix_nigrograna	<0.0001(<0.0001,<0.0001)	1.07E-05	1.03E-05	<0.0001(<0.0001,<0.0001)	0.00125642	3.94E-04	8.39E-07	-4.926	***	9.70E-05	<0.0001	***<0.0001
Recurvomyces_spe_Incertae_sedis	<0.0001(<0.0001,<0.0001)	6.90E-04	6.74E-04	<0.0001(<0.0001,<0.0001)	0	0	0.0324831	-2.1384	*	0.113104	0.0325	* 0.0325
Sporobolomyces_roseus	<0.0001(<0.0001,<0.0001)	6.84E-04	5.59E-04	<0.0001(<0.0001,<0.0001)	0	0	0.007789	-2.6611	**	0.045939	0.0078	** 0.0078
Fusarium_poae	<0.0001(<0.0001,<0.0001)	2.07E-04	1.04E-04	<0.0001(<0.0001,0.0001)	8.68E-04	4.46E-04	0.0320289	-2.144	*	0.113104	0.032	* 0.032
Aspergillus_tennesseensis	<0.0001(<0.0001,<0.0001)	3.01E-04	2.01E-04	<0.0001(<0.0001,<0.0001)	6.74E-04	4.95E-04	0.030075	-2.1691	*	0.113104	0.0301	* 0.0301
Debaryomyces_prosopidis	<0.0001(<0.0001,<0.0001)	4.00E-04	3.35E-04	<0.0001(<0.0001,<0.0001)	4.78E-04	3.94E-04	3.50E-04	-3.575	***	0.00653	0.0004	***0.0004
Dipodascus_geotrichum	<0.0001(<0.0001,<0.0001)	7.38E-05	1.90E-05	<0.0001(<0.0001,<0.0001)	0.00106213	0.001054	0.0024996	-3.0234	**	0.021524	0.0025	** 0.0025
Udeniomyces_megalosporus	<0.0001(<0.0001,<0.0001)	5.93E-04	2.33E-04	<0.0001(<0.0001,<0.0001)	2.40E-05	1.32E-05	0.001895	-3.1062	**	0.018564	0.0019	** 0.0019
Flavodon_spe_Incertae_sedis	<0.0001(<0.0001,<0.0001)	5.97E-04	5.55E-04	<0.0001(<0.0001,<0.0001)	4.96E-06	4.96E-06	0.044586	-2.0085	*	0.1433	0.0446	* 0.0446
Ustilaginoidea_virens	<0.0001(<0.0001,<0.0001)	1.03E-05	4.29E-06	<0.0001(<0.0001,0.0002)	0.00105854	5.32E-04	2.07E-06	-4.7467	***	1.71E-04	<0.0001	***<0.0001
Thielaviopsis_euricoi	<0.0001(<0.0001,<0.0001)	5.74E-04	3.57E-04	<0.0001(<0.0001,<0.0001)	0	0	0.0026097	-3.0103	**	0.021549	0.0026	** 0.0026
Zanclospora_aurea	<0.0001(<0.0001,<0.0001)	7.21E-05	3.79E-05	<0.0001(<0.0001,0.0002)	8.51E-04	3.06E-04	1.84E-04	-3.7394	***	0.005039	0.0002	***0.0002
Pleurotus_eryngii	<0.0001(<0.0001,<0.0001)	5.15E-04	4.83E-04	<0.0001(<0.0001,<0.0001)	1.33E-06	1.33E-06	0.0045435	-2.8377	**	0.032827	0.0045	** 0.0045
[Candida]_glabrata	<0.0001(<0.0001,<0.0001)	6.82E-07	6.82E-07	<0.0001(<0.0001,<0.0001)	9.26E-04	9.11E-04	0.0013514	-3.2048	**	0.015022	0.0014	** 0.0014
Yarrowia_lipolytica	<0.0001(<0.0001,<0.0001)	4.80E-04	3.03E-04	<0.0001(<0.0001,<0.0001)	0	0	0.0111554	-2.5378	*	0.058619	0.0112	* 0.0112
Pseudoramichloridium_henryi	<0.0001(<0.0001,<0.0001)	4.80E-04	4.72E-04	<0.0001(<0.0001,<0.0001)	0	0	0.0463617	-1.9921	*	0.1433	0.0464	* 0.0464
Stagonosporopsis_lupini	<0.0001(<0.0001,<0.0001)	4.61E-04	4.44E-04	<0.0001(<0.0001,<0.0001)	0	0	0.0037686	-2.8969	**	0.028289	0.0038	** 0.0038

Knufia_epidermidis	<0.0001(<0.0001,<0.0001)	4.61E-04	4.05E-04	<0.0001(<0.0001,<0.0001)	0	0	0.0463617	-1.9921	*	0.1433	0.0464	* 0.0464
Hortaea_werneckii	<0.0001(<0.0001,<0.0001)	0	0	<0.0001(<0.0001,<0.0001)	8.41E-04	7.89E-04	7.45E-04	-3.3723	***	0.010259	0.0007	***0.0007
Cutaneotrichosporon_jirovecii	<0.0001(<0.0001,<0.0001)	4.53E-04	3.72E-04	<0.0001(<0.0001,<0.0001)	8.69E-06	8.69E-06	0.0080193	-2.6513	**	0.04682	0.008	** 0.008
Dendryphiella_paravinosa	<0.0001(<0.0001,<0.0001)	0	0	<0.0001(<0.0001,<0.0001)	8.12E-04	7.95E-04	7.45E-04	-3.3723	***	0.010259	0.0007	***0.0007
Candida_apicola	<0.0001(<0.0001,<0.0001)	0	0	<0.0001(<0.0001,<0.0001)	8.09E-04	7.38E-04	2.55E-04	-3.6571	***	0.005474	0.0003	***0.0003
Ceriporia_lacerata	<0.0001(<0.0001,<0.0001)	4.30E-04	3.13E-04	<0.0001(<0.0001,<0.0001)	0	0	1.20E-04	-3.846	***	0.003653	0.0001	***0.0001
Golubevia_pallescens	<0.0001(<0.0001,<0.0001)	4.16E-04	2.65E-04	<0.0001(<0.0001,<0.0001)	5.02E-06	5.02E-06	2.56E-04	-3.6565	***	0.005474	0.0003	***0.0003
Coprinopsis_urticicola	<0.0001(<0.0001,<0.0001)	4.10E-04	3.85E-04	<0.0001(<0.0001,<0.0001)	0	0	0.0026097	-3.0103	**	0.021549	0.0026	** 0.0026
Dothidea_sambuci	<0.0001(<0.0001,<0.0001)	4.08E-04	4.02E-04	<0.0001(<0.0001,<0.0001)	0	0	0.0324831	-2.1384	*	0.113104	0.0325	* 0.0325
Agaricus_subfloccosus	<0.0001(<0.0001,<0.0001)	0	0	<0.0001(<0.0001,<0.0001)	7.36E-04	7.32E-04	0.0021705	-3.0659	**	0.019301	0.0022	** 0.0022
Candida_solani	<0.0001(<0.0001,0.0001)	1.64E-04	4.22E-05	<0.0001(<0.0001,<0.0001)	4.18E-04	4.06E-04	0.0012334	-3.231	**	0.014611	0.0012	** 0.0012
Peniophorella_spe_Incertae_sedis	<0.0001(<0.0001,<0.0001)	3.80E-04	3.62E-04	<0.0001(<0.0001,<0.0001)	0	0	0.0037685	-2.8969	**	0.028289	0.0038	** 0.0038
Nigrospora_spe_Incertae_sedis	<0.0001(<0.0001,<0.0001)	3.78E-04	3.70E-04	<0.0001(<0.0001,<0.0001)	0	0	0.0324831	-2.1384	*	0.113104	0.0325	* 0.0325
Diutina_catenulata	<0.0001(<0.0001,<0.0001)	2.25E-06	1.53E-06	<0.0001(<0.0001,<0.0001)	6.83E-04	4.83E-04	0.0152996	-2.4252	*	0.074941	0.0153	* 0.0153
Talaromyces_islandicus	<0.0001(<0.0001,<0.0001)	3.58E-04	3.52E-04	<0.0001(<0.0001,<0.0001)	0	0	0.0159445	-2.4102	*	0.074941	0.0159	* 0.0159
Candida_ethanolica	<0.0001(<0.0001,<0.0001)	0	0	<0.0001(<0.0001,<0.0001)	6.26E-04	6.21E-04	0.01859	-2.3537	*	0.078431	0.0186	* 0.0186
Pseudoveronaea_ellipsoidea	<0.0001(<0.0001,<0.0001)	5.99E-06	5.24E-06	<0.0001(<0.0001,<0.0001)	6.14E-04	3.93E-04	3.12E-04	-3.6052	***	0.006217	0.0003	***0.0003
Typhula_micans	<0.0001(<0.0001,<0.0001)	3.30E-04	2.51E-04	<0.0001(<0.0001,<0.0001)	0	0	5.79E-04	-3.4413	***	0.009561	0.0006	***0.0006
Filobasidium_wieringae	<0.0001(<0.0001,<0.0001)	3.21E-04	3.02E-04	<0.0001(<0.0001,<0.0001)	5.67E-06	3.42E-06	0.0217789	-2.2942	*	0.08952	0.0218	* 0.0218
Penicillium_bialowiezense	<0.0001(<0.0001,<0.0001)	0	0	<0.0001(<0.0001,<0.0001)	5.86E-04	5.78E-04	0.0021705	-3.0659	**	0.019301	0.0022	** 0.0022
Carlosrosaea_hohenbergiae	<0.0001(<0.0001,<0.0001)	3.16E-04	3.10E-04	<0.0001(<0.0001,<0.0001)	0	0	0.0463617	-1.9921	*	0.1433	0.0464	* 0.0464
Diaporthe_caulivora	<0.0001(<0.0001,<0.0001)	3.06E-04	2.09E-04	<0.0001(<0.0001,<0.0001)	0	0	0.0018011	-3.1212	**	0.01833	0.0018	** 0.0018
Ciliophora_spe_Incertae_sedis	<0.0001(<0.0001,<0.0001)	2.97E-04	1.70E-04	<0.0001(<0.0001,<0.0001)	0	0	0.0012386	-3.2298	**	0.014611	0.0012	** 0.0012
Parastagonosporella_fallopiae	<0.0001(<0.0001,<0.0001)	2.92E-04	2.76E-04	<0.0001(<0.0001,<0.0001)	0	0	0.0111558	-2.5378	*	0.058619	0.0112	* 0.0112
Toxicocladosporium_strelitziae	<0.0001(<0.0001,<0.0001)	2.82E-04	2.78E-04	<0.0001(<0.0001,<0.0001)	0	0	0.0324831	-2.1384	*	0.113104	0.0325	* 0.0325
Vishniacozyma_heimaeyensis	<0.0001(<0.0001,<0.0001)	2.78E-04	2.69E-04	<0.0001(<0.0001,<0.0001)	0	0	0.007789	-2.6611	**	0.045939	0.0078	** 0.0078
Yarrowia_bubula	<0.0001(<0.0001,<0.0001)	2.76E-04	2.63E-04	<0.0001(<0.0001,<0.0001)	0	0	0.0037685	-2.8969	**	0.028289	0.0038	** 0.0038
Hypoxylon_perforatum	<0.0001(<0.0001,<0.0001)	2.73E-04	2.56E-04	<0.0001(<0.0001,<0.0001)	0	0	0.0111558	-2.5378	*	0.058619	0.0112	* 0.0112
Candida_vespimorsuum	<0.0001(<0.0001,<0.0001)	1.20E-05	5.98E-06	<0.0001(<0.0001,<0.0001)	4.47E-04	2.61E-04	8.75E-04	-3.328	***	0.011492	0.0009	***0.0009
Trichoderma_deliquescens	<0.0001(<0.0001,<0.0001)	2.55E-04	2.50E-04	<0.0001(<0.0001,<0.0001)	0	0	0.0463599	-1.9921	*	0.1433	0.0464	* 0.0464
Stemphylium_lycopersici	<0.0001(<0.0001,<0.0001)	2.46E-04	1.67E-04	<0.0001(<0.0001,<0.0001)	0	0	0.0227671	-2.2773	*	0.08952	0.0228	* 0.0228
Auricularia_delicata	<0.0001(<0.0001,<0.0001)	2.44E-04	2.35E-04	<0.0001(<0.0001,<0.0001)	0	0	0.0463599	-1.9921	*	0.1433	0.0464	* 0.0464
Tulostoma_calcareum	<0.0001(<0.0001,<0.0001)	2.34E-04	2.28E-04	<0.0001(<0.0001,<0.0001)	0	0	0.0324831	-2.1384	*	0.113104	0.0325	* 0.0325
Tilletia_geeringii	<0.0001(<0.0001,<0.0001)	2.33E-04	1.55E-04	<0.0001(<0.0001,<0.0001)	0	0	0.0012386	-3.2298	**	0.014611	0.0012	** 0.0012
Wickerhamiella_versatilis	<0.0001(<0.0001,<0.0001)	3.58E-05	3.06E-05	<0.0001(<0.0001,<0.0001)	3.42E-04	2.49E-04	0.0413845	-2.0397	*	0.138267	0.0414	* 0.0414
Nigrograna_spe_Incertae_sedis	<0.0001(<0.0001,<0.0001)	2.17E-04	1.66E-04	<0.0001(<0.0001,<0.0001)	0	0	0.0324831	-2.1384	*	0.113104	0.0325	* 0.0325
Golovinomyces_sonchicola	<0.0001(<0.0001,<0.0001)	0	0	<0.0001(<0.0001,<0.0001)	3.80E-04	3.72E-04	0.0021705	-3.0659	**	0.019301	0.0022	** 0.0022
Xylocoremium_spe_Incertae_sedis	<0.0001(<0.0001,<0.0001)	2.04E-04	2.00E-04	<0.0001(<0.0001,<0.0001)	0	0	0.0324819	-2.1384	*	0.113104	0.0325	* 0.0325
Didymella_spe_Incertae_sedis	<0.0001(<0.0001,<0.0001)	2.04E-04	1.46E-04	<0.0001(<0.0001,<0.0001)	0	0	0.0463617	-1.9921	*	0.1433	0.0464	* 0.0464
Venturiaceae_spe_Incertae_sedis	<0.0001(<0.0001,<0.0001)	0	0	<0.0001(<0.0001,<0.0001)	3.35E-04	3.31E-04	0.006324	-2.7305	**	0.039736	0.0063	** 0.0063
Didymocyrtis_brachylaenae	<0.0001(<0.0001,<0.0001)	1.15E-06	1.15E-06	<0.0001(<0.0001,<0.0001)	3.14E-04	3.07E-04	0.0125736	-2.4956	*	0.064184	0.0126	* 0.0126
Preussia_procaviae	<0.0001(<0.0001,<0.0001)	1.69E-04	1.65E-04	<0.0001(<0.0001,<0.0001)	0	0	0.0324831	-2.1384	*	0.113104	0.0325	* 0.0325
Calycina_spe_Incertae_sedis	<0.0001(<0.0001,<0.0001)	1.67E-04	1.63E-04	<0.0001(<0.0001,<0.0001)	0	0	0.0463617	-1.9921	*	0.1433	0.0464	* 0.0464

Kazachstania_humilis	<0.0001(<0.0001,<0.0001)	0	0	<0.0001(<0.0001,<0.0001)	3.04E-04	2.97E-04	0.0063247	-2.7305	**	0.039736	0.0063	** 0.0063
Derxomyces_mrakii	<0.0001(<0.0001,<0.0001)	1.65E-04	1.59E-04	<0.0001(<0.0001,<0.0001)	0	0	0.0324831	-2.1384	*	0.113104	0.0325	* 0.0325
Issatchenkia_orientalis	<0.0001(<0.0001,<0.0001)	1.86E-06	1.10E-06	<0.0001(<0.0001,<0.0001)	2.87E-04	2.58E-04	0.0019304	-3.1007	**	0.018596	0.0019	** 0.0019
Gibberella_baccata	<0.0001(<0.0001,<0.0001)	6.70E-07	6.70E-07	<0.0001(<0.0001,<0.0001)	2.46E-04	2.41E-04	0.0331688	-2.13	*	0.114414	0.0332	* 0.0332
Diaporthe_bohemiae	<0.0001(<0.0001,<0.0001)	1.34E-04	1.16E-04	<0.0001(<0.0001,<0.0001)	0	0	0.0111558	-2.5378	*	0.058619	0.0112	* 0.0112
Lecanicillium_coprophilum	<0.0001(<0.0001,<0.0001)	1.22E-04	1.15E-04	<0.0001(<0.0001,<0.0001)	0	0	0.0463617	-1.9921	*	0.1433	0.0464	* 0.0464
Phlebiopsis_flavidoalba	<0.0001(<0.0001,<0.0001)	1.18E-04	1.15E-04	<0.0001(<0.0001,<0.0001)	0	0	0.0463599	-1.9921	*	0.1433	0.0464	* 0.0464

Table S5. The relative abundance and distribution of the key 43 OTUs

	Control001	Control003	Control004	Control005	Control006	Control007	Control008	Control009	Control010	Control012	Control013	Control017	Control020
OTU563 (Purpureocillium_lilacinum)	0	0	0	0	0	0	0	0	0	0	0	0.00955	0
OTU1858 (Fuscosporella_aquatica)	0	0	0.00E+00	0	0	0	0	0	0	0	0	0	0
OTU1210 (Ophiostoma_pusillum)	0	0	0	0	0	0.0069	0	0	0	0	0	0	0
OTU1959 (Acrodictys_fluminicola)	0	0	0	0	0	0	0	0	0	0	0	0	0
OTU2237 (Simplicillium_sympodiophorum)	0	0	0	0	0	0.00561	0.00738	0	0	0	0	0	0
OTU2338 (Acrodictys_fluminicola)	0	0	0	0	0	0	0	0	0	0	0	0	0
OTU2141 (Acrodictys_fluminicola)	0.00692	0.0071	0.0041696	0	0	0.01437	0	0	0.0063	0	0	0	0.00373
OTU2103 (Sporothrix_nigrograna)	0	0	0.00E+00	0	0	0	0	0	0	0	0	0	0
OTU1114 (CryptocoCOVID-19-us_longus)	0.00445	0.02514	0.0028869	0.0255	0	0	0	0.00499	0.01089	0	0.00367	0.00451	0
OTU2318 (Talaromyces_scorteus)	0	0	0.002551	0	0	0.00194	0	0	0	0	0	0	0
OTU1844 (Parafuscosporella_mucosa)	0.00257	0.003	0.0034943	0.00168	0.00296	0	0.00146	0.00221	0	0	0	0	0
OTU1218 (Acrodictys_fluminicola)	0	0.00118	0	0.00238	0.00222	0	0	0	0	0	0	0	0
OTU1602 (SaCOVID-19-haromyces_cerevisia	0	0	0	0	0	0	0	0	0	0	0	0	0
OTU1436 (Sporothrix_nigrograna)	0	0	0	0	0	0	0	0	0	0	0	0	0
OTU142 (Ustilaginoidea_virens)	0	0	0	0	0	0	0	0.00155	0	0	0	0	0
OTU1542 (Candida_albicans)	0	0	0	0	0	0	0	0.00116	0	0	0	0	0
OTU111 (Starmerella_etchellsii)	0.001	0.00059	0	0	0	0.00055	0.00065	0	0	0	0	0	0
OTU133 (Pichia_kluyveri)	0	0	0	0	0	0	0	0	0	0.00489	0	0	0
OTU94 (Henningsia_resupinata)	0	0	0	0	0	0	0	0	0	0	0	0	0
OTU102 (Milospium_graphideorum)	0	0	0	0	0	0	0	0	0	0	0.00157	0.00171	0
OTU1333 (CryptocoCOVID-19-us_longus)	0.00071	0.00567	4.86E-04	0.0114	0	0	0	0.00147	0.00701	0	0	0.00068	0.0005
OTU2121 (Zanclospora_jonesii)	0	0	0.0016101	0	0	0	0	0	0.12373	0.00323	0.0895	0.00163	0.00137
OTU2016 (Simplicillium_sympodiophorum)	0	0	0	0	0	0.00215	0.00026	0	0	0	0.00426	0	0
OTU925 (CryptocoCOVID-19-us_longus)	0	0.00707	0	0.01143	0	0.00029	0.0003	0	0.00547	0.00211	0	0.00064	0
OTU72 (Malassezia_globosa)	0.00029	0.00397	0	0.00214	0.03551	0	0.00024	0	0	0.00198	0.00963	0.00042	0.00085
OTU2166 (Zoophthora_radicans)	0.0026	0	0	0.00018	0.00034	0.0039	0	0	0	0	0.01451	0.00013	0
OTU40 (Diaporthe_kochmanii)	0	0	0	0	0	0	0	0	0	0	0.00019	0.00092	0.00067
OTU1820 (Raffaelea_lauricola)	0.00043	0.0023	0	9.6E-05	0.00049	0.00707	0	0	0.00037	0.00209	0.00464	0	0
OTU50 (Zanclospora_jonesii)	0	8.3E-05	0	0	0	0	0	0	0	0	0	0.00011	0
OTU1794 (Acrodictys_fluminicola)	0.00116	0.00033	0.0001647	0.00044	0.00086	0.00097	0.00027	0.46559	0.00086	0.00574	0.00088	0.51238	0.00065
OTU2353 (Acrodictys_fluminicola)	0.00069	0.00819	0.0004002	0.00025	0.00156	0.00769	0	0.00155	0.00064	0.00267	0.00971	0.0001	0
OTU57 (Chrysosphaeria_jan-nelii)	0	0	0.00E+00	0	0	0	0	0	0.00012	0	0	0	0
OTU35 (Gibberella_fujikuroi)	0	0	6.80E-05	0	0	0	0	0	0	0	8.9E-05	0	0
OTU27 (Zanclospora_jonesii)	0.00046	0.00039	0.0001603	0.00043	0.02468	0.31213	0.00031	0.00088	0.07888	0.0041	0.00101	0.00183	0.00123
OTU22 (Zanclospora_jonesii)	0.00058	0.00838	0.0006859	0.01771	0.00074	0.00098	0.00031	0.00065	0.00028	0.00303	0.00011	3.1E-05	0.00346
OTU21 (Hydnobolites_roseus)	0	2E-05	0	0	0	0	0	0	0	0	3.3E-05	0	0
OTU24 (Candida_albicans)	3.2E-05	0	0.000292	8.4E-05	0	0.00011	0	0.00025	0	0	7.2E-05	6.1E-05	2.6E-05
OTU16 (Zanclospora_jonesii)	0	4.1E-05	0.0000607	0.00017	0.0306	0	1.7E-05	0	0.00059	0	0	0	0
OTU13 (Diversispora_spurca)	0.00053	0.00032	0.4424098	0.00093	0.00174	0.00672	0.00035	0.00053	0.0007	0.00464	0	7.4E-05	2.3E-05
OTU10 (Aspergillus_subflavus)	0.00017	4.4E-05	1.28E-04	0.00013	0.00019	0.00013	0.0001	7.3E-05	8.8E-05	0.0006	0.00046	0.00034	0.00015
OTU11 (Chrysosphaeria_jan-nelii)	1.9E-05	1.5E-05	0	0	5.2E-05	2.2E-05	0	0	2.3E-05	0.00012	0	1.8E-05	0
OTU4 (CryptocoCOVID-19-us_longus)	0.00043	0.00593	0.0003604	0.01064	0.00031	0.00041	0.00025	0.00038	0.00566	0.00192	0.00047	0.00099	0.00054
OTU3 (Zanclospora_jonesii)	0.00088	0.00154	0.0009024	0.00101	0.00137	0.00123	0.00045	0.00124	0.00081	0.0477	0.00027	0.00045	0.00085

Control026	Control027	Control028	Control030	Control031	Control033	Control034	Control035	Control036	Control037	Control038	Control039	Control040	Control041	Control042	Control043	Control044
0	0	0	0	0.04006	0.02574	0.01406	0.03423	0.02229	0.026	0.06872	0.0122	0.0198992	0.01565	0.02706	0.0106129	0.01406
0	0	0	0	0.02375	0	0	0	0	0	0	0.00954	0	0.01343	0	0	0
0	0	0	0	0	0	0	0.0074	0	0	0	0.00526	0	0	0	0	0
0	0	0	0	0	0	0	0	0	0	0	0	0	0	0	0	0
0	0	0	0	0	0	0	0.01723	0	0	0.00533	0.04266	0	0	0	0	0
0	0	0	0	0	0	0	0	0	0	0	0.00596	0	0	0	0	0.00596
0	0	0.00994	0	0	0	0	0.0039	0	0	0	0.00736	0	0	0	0	0
0	0	0	0	0.00284	0	0	0	0	0	0	0	0	0	0	0	0
0.097071	0	0	0	0.01077	0	0.01305	0.01137	0.0012	0.01221	0.02045	0.00902	0.0036086	0.00698	0	0.0031274	0.02171
0	0.04143	0	0.00387	0	0	0	0.00175	0	0	0.00198	0.00383	0.00E+00	0	0	0	0
0.0054553	0	0	0.00332	0	0	0.00082	0	0.00777	0	0.00175	0.00389	0.0023533	0	0	0	0
0	0	0	0	0	0	0	0	0	0	0	0	0	0	0	0	0
0	0	0	0	0	0	0	0	0	0	0	0	0.0036906	0	0	0	0
0	0	0	0	0	0	0	0	0.01907	0	0	0	0	0	0	0	0
0	0	0	0	0	0	0	0	0	0	0	0	0	0	0	0	0
0.00E+00	0	0	0	0	0	0	0	0	0	0.00057	0	0	0	0	0	0
0	0	0	0	0	0	0	0	0.00049	0	0	0	0	0	0	0	0
0	0	0	0	0	0	0	0	0	0	0	0	0	0	0	0	0
0	0	0	0	0	0	0	0	0	0	0	0	0	0	0	0	0
0.000646	0.03802	0.0032	0.00776	0	0	0	0.00191	0	0	0	0	0	0	0	0.0017846	0.00183
0.0561385	0.02719	0.00192	0.00097	0.00362	0	0.00621	0.00296	0.00032	0.00044	0.00488	0.00396	0.0006513	0.00516	0.00099	0	0.00345
0	0	0.00057	0	0.00063	0	0	0	0	0	0	0	0	0	0	0	0
0	0	0	0	0.00039	0	0	0	0	0	0	0	0.0005284	0	0	0	0
0.0639949	0.01027	0.00073	0.00108	0.00241	0	0.0035	0.0014	0.00045	0.00295	0.00748	0.00447	0.0003515	0.00663	0.0011	0.0006434	0.00578
0.0330891	0.02435	0.00094	0.00062	0.0231	0.00022	0.00519	0.00265	0.02089	0	0.00044	0.00157	0.0006508	0.01343	0.00109	0.0008471	0.00032
0	0	0	0	0.00048	0	0.00035	0	0	0	0.00018	0	0	0	0	0	0
0	0	0	0	0	0	0	0	0	0	0	0	0	0.96812	0.00113	0.0025936	0.0021
0.0001867	0.01822	0.10498	0	0.00039	0.00658	0.00011	0.03776	0.00021	0	0.00035	0.01022	0.0002078	0	0.00032	0	0.00159
0.0001623	0.01026	0.00033	0.00055	0	0	0	0	0	0	0	0	0	0	0.00421	0.0002567	0.00075
0	0.00231	0	0	0	0	0	0	0	0	0	0	0	0	0	0.00E+00	0
0	0.00449	0.00157	0	0.00047	0.00021	0.00019	0.01432	0	0	0	0.01776	0.0001411	0	0	0.0004797	0
0	0	0	0	0	0	0	0	8.2E-05	0	0	0	0	0	0	0.00E+00	8.9E-05
0	0	0	0	8E-05	0.00087	0	0	0	0	0	5.2E-05	0	8.7E-05	0	0	0
0.0003058	0.02478	0.00046	0.00134	0.02331	0.00015	0	5.1E-05	5.4E-05	0	0	0	0	0	0	0	0
0.000032	0.00295	8.9E-05	0.00058	0.00045	0	2.6E-05	3.8E-05	0	0.00366	0	0	0.0000935	0.00778	0	0	0
0.00E+00	0.00119	0	6.2E-05	7.7E-05	3E-05	6.9E-05	0	3.9E-05	0	0	2E-05	0.0000475	0	0	0	0
0	0.00046	0.00134	0	0	0	1.9E-05	0	0	0	0.00013	0	0	9.2E-05	0	0	0
2.21E-04	0.01284	0.00032	0.00072	0.00047	0.00028	0.00012	0.00036	0.00023	8.5E-05	0.00036	8.2E-05	0.0002715	0.0006	0.00076	0.4487596	0.00054
0.021783	0.01087	0.00039	0.00079	0.00055	0.0004	0.01297	0.00375	0.00037	0.00024	0.00039	0.00938	0.2970153	2.9E-05	0.0004	0.0015467	3.6E-05
6.19E-06	0.0006	0	0	0.00018	0.00017	0.08436	0.00023	0.00011	0.0002	0.02146	7.8E-05	0.0001348	9.3E-05	0.00025	0.0003192	0.00017
0.000006	0.00028	2E-05	0	8.8E-05	3.1E-05	4.4E-05	4.3E-05	0.04163	3.9E-05	7.4E-05	3.1E-05	0.0001092	0	6.5E-05	0	0
0.0618337	0.04971	0.0015	0.00216	0.0024	0.00013	#######	0.00227	0.00031	0.00155	0.00712	0.00435	0.0004038	0.00582	0.00075	0.0010024	0.00596
0.0062781	0.00448	0.00014	0.00026	0.00137	0.00016	0.00015	0.00015	0.00531	0.00021	0.00238	0.00241	0.0002849	1.1E-05	0.00126	0.0000843	0.00056

Control045	Control046	Control047	Control048	Control049	Control050	Control 051	Control 052	Control 053	Control 054	Control 056	Control 058	Control 059	Control 060	Control 061	Control 062	Control 064	Control 065
0.0459	0.0467	0.02786	0	0	0	0	0	0	0	0	0	0	0	0	0	0	0
0	0	0	0	0	0	0	0	0	0	0	0	0	0	0	0	0	0
0.00707	0	0	0	0	0.01019	0	0	0	0	0	0	0	0	0	0	0	0
0	0	0	0	0	0	0	0	0	0	0	0	0	0	0	0	0	0
0.00602	0	0	0	0	0.00451	0	0	0	0	0	0	0	0	0	0	0	0
0.00923	0	0	0	0	0	0	0	0	0	0	0	0	0	0	0	0	0
0	0	0	0	0	0.04409	0	0	0	0.002	0	0	0	0	0	0	0.0074	0
0	0	0	0	0	0	0	0	0	0	0	0	0	0	0	0	0	0
0.02881	0.04433	0.00361	0	0	0.00174	0.0092	0.0091	0.0191	0.011	0.021	0.0114	0	0	0.0084	0	0.0408	0.0605
0.00283	0	0	0	0	0	0	0	0	0	0	0	0	0	0.0021	0	0	0
0.00221	0	0	0.00631	0	0	0	0	0	0.0055	0	0.0015	0.0066	0	0.0029	0.0057	0.092	0.0455
0	0	0	0	0	0.00104	0.0014	0	0	0.0008	0	0	0.0028	0	0.0021	0	0	0
0	0	0	0	0.00155	0	0	0	0.0063	0	0.0365	0.0064	0	0	0	0.0076	0	0
0	0	0	0	0	0	0	0	0	0	0	0	0	0	0	0	0	0
0	0	0	0	0	0	0.0055	0	0	0	0	0	0.002	0	0.0009	0.0012	0.0036	0.0015
0	0	0	0	0	0	0.0006	0	0	0	0	0	0	0	0	0	0	0
0	0	0	0	0	0	0	0	0	0	0	0	0	0	0	0	0	0.0008
0	0	0	0	0	0	0	0	0	0	0	0	0	0	0	0	0	0
0	0	0	0	0	0	0	0	0.0004	0	0	0	0	0	0	0	0	0
0.00193	0.00174	0.85912	0	0	0	0	0	0	0	0	0	0	0	0	0	0	0
0.01346	0.01715	0.01477	0	0	0.00031	0	0	0.0063	0.0094	0.0133	0.0069	0.002	0	0.0018	0.0021	0.0402	0.0125
0	0	0	0.0025	0.00123	0.00025	0.1552	0.0027	0.0009	0.0007	0.0004	0.0012	0	0	0	0.0014	0	0
0.01929	0	0	0	0	0	0	0	0	0	0	0	0	0	0.0037	0.0005	0	######
0.01336	0.0216	0.01076	0	0	0	0.0002	0	0.0057	0.0042	0.0153	0.0085	0.0039	0.0005	0.0019	0.0008	0.0339	0.0195
0.00495	0.06039	0.01327	0.00408	0	0.00021	0	0.0008	0.0094	0	0.0768	0	0.0026	0	0	0.0032	0.0056	0.0049
0.01335	0	0	0.00038	0	0	0	0	0	0	0	0	0	0.0004	0.0039	0.0036	0	0.0017
0.00152	0.00036	0.00108	0	0.0005	0	0	0	0	0	0	0	0	0.0003	0	0	0	0
0.00837	0	0	0	0.00021	0.0055	0	0	0	0	0	0	0	0	0	0.0004	0.0004	0
0.09097	0	0.00045	0.00026	0	0	0	0	0	0	0	0	0	0.0006	0.0001	0	0	0
8.5E-05	0.00015	0	0	0	0	0	0	0.0002	0	0	0	0	0	0	0	0	1E-04
0.02267	0	0	0.00093	0	0.01407	7E-05	0	6E-05	8E-05	0	0	0.0003	0.0002	0.0056	0.0004	0.0003	0.0005
0	0	0	0.00015	0.00024	0	0	0	0	0	0	0	0	0.0002	0	0	0	0
0	0	0	0	0.00021	0	0	0	6E-05	0	0	0	0	0.0002	0	0.0002	0	0
0	0	0	0	0	0	0	0.0003	7E-05	0	0	0	0	9E-05	4E-05	0	0	9E-05
0	0	0	0.00065	0.00041	0.00053	0.0008	0.0016	0.0009	0.0008	0.0005	0.0375	0.0222	0.0029	0.0058	0.0215	0.0271	0.0239
0	0	0	0	4.8E-05	0	0.0002	0	2E-05	0	3E-05	2E-05	0	0	0	0	0	0
1.8E-05	2.9E-05	0	0	0.00029	7.4E-05	0.0002	0	1E-04	2E-05	2E-05	2E-05	0	0.0001	0	0	0	6E-05
0.00083	0.00066	0.00076	0	0	0	7E-05	0	0	0	0	0	0	0	0	0	######	0
5.6E-05	0	3.4E-05	4.3E-05	0.00012	0.00013	0.0017	0.0007	2E-05	0.0006	0.0247	1E-04	0.0004	0.0038	0	0.0003	0	0
0.00024	0.00014	0.00027	0.00025	0.00024	0.00249	0.0002	0.1178	0.0004	0.0007	0.0001	0.0002	0.0023	0.0004	0.0002	0.0005	0.0002	0.0793
1.1E-05	0	1.7E-05	8.5E-05	4.5E-05	0	2E-05	0.0002	2E-05	0	0.0002	2E-05	3E-05	7E-05	3E-05	0	3E-05	4E-05
0.01294	0.02847	0.01119	0.00015	0.00014	0.00022	0.0004	0.0002	0.006	0.0068	0.0151	0.0078	0.0028	0.0003	0.0013	0.0012	0.039	0.0172
0.00359	9.5E-06	0	0.00334	0.00046	0.00012	0.0002	0.0004	0.0003	0.0021	0.0037	0.002	0.0114	0.0007	0.0051	0.004	0.078	0.0442

Control 067	Control 068	Control 069	Control 071	Control 074	Control 076	Control 077	Control 079	Control 081	Control 082	Control 084	Control 085	Control 086	Control 087	Control 088	Control 090	Control 091	Control 092
0	0	0	0	0	0	0	0	0	0	0	0	0	0	0	0	0.0226	0
0	0	0	0	0	0	0	0.0117	0.0055	0	0	0	0	######	0	0	0	0
0	0	0	0.0263	0	0	0	0	0	0.009	0	0.0059	0.0067	0	0	0	0.0104	0
0	0	0	0	0	0	0	0	######	0	0	0	0	0	0	0	0	0
0	0	0	0	0	0	0	0	0	0.0227	0	0.0611	######	######	0	0	0	0
0	0.0036	0	0	0	0	0	0.0201	0	0	0	0.003	######	0	0	0.0086	0	0
0	0	0	0	0	0	0	0	0	0.0159	0	0.0067	0	0	0	0.0061	0	0
0	0	0	0	0	0	0	0	0	0	0	0	0	0	0	0	0	0
0.0214	0	0	0.0093	0.0167	0.0398	0	0	0.0125	0.0047	0	0.0139	0	######	######	0	0.0172	0.0045
0	0	0	0	0	0	0	0	0	0.2412	0	0	0	0.0018	0	0	0.0068	0
0.0031	0.0077	0.0277	0.0372	0.0734	0.0997	0.0746	0.0161	######	0.0084	0.0018	0.0024	0.0553	0.0474	0.1229	0	0	0.0027
0	0	0	0	######	######	0	0.1014	0	0	0	0	0	0	0	0	0	0
0.0056	0	0	0	0	0	0.0016	0	0	0	0	0	0	0	0	0	0	0
0	0	0	0	0	0	0	0	0	0	0	0	0	######	0	0	0	0
0	0	0.0013	0	0	0	0	0	0	0	0	0	0	0	0	0	0	0
0	0	0	0	0	0	0	0	0	0	0	0	0	0	0	0	0	0
0	0	0	0	0	0	0	0	0	0	0	0.0006	0	0	0	0	0	0
0	0	0	0	0	0	0	0	0	0	0	0	0	0	0	0	0	0
0	0	0	0	0	0	0	0	0	0	0	0	0	0	0	0	0	0
0	0	0	0.0015	0.0005	0.0004	0	0	0	0	0	0	0	0	0	0	0.0008	0
0.0129	0.007	0	0.0109	0.0075	0.0364	0.0063	0.0007	0.0063	0.0006	0	0.004	0.0009	0.0485	0	0.0007	0.0398	0.0689
0	0	0	0	0	0	0	0.0005	0.0005	0.0006	0.0004	0.001	0.0004	0	0	0.0006	0.0006	0.0013
0	0	0	0	0	0	0	0.0009	0.0003	0	0.0005	0	0	0	0	0.0334	0	0
0.0145	0.0094	0	0.0134	0.0081	0.0336	0.008	0	0.0055	0.0007	0	0.0031	0.0005	0.0444	0	0.0013	0.0443	0.0551
0.045	0	0	0.017	0	0	0.0057	0.0103	0	0.003	0.0003	0.0007	0	0.0324	0	0.0097	0	0.0005
0.0002	0	0.0012	0	0	0	0.0002	0	0	0	0	0	0.0002	0.0002	0	0.0544	0	0
0	0	0	0.0005	0.0004	0.0005	0	0	0	0	0	0	0	0	0	0.0008	0.0005	0.0008
0	0.0001	0	0.0032	######	######	0.0004	0.0007	0	0.006	0.0004	0.0477	0.0002	0.0001	0.0001	0.0012	0.0012	0
0	0	0	0	0	0	0	0	######	0	0	0	0	0	######	0	0	0
0.0001	0	0	0	0	0	0.0002	0	0	0.0002	0.0012	0.0002	1E-04	0.0002	0.0005	0.0006	0.0002	0.0008
9E-05	0	0.0011	0.0032	0	0	0	0.0014	0	0.0238	0	0.0029	0	0.0003	0	0.0296	0.0023	0.0002
0	0	0	0	0	0	0	0	0	0	0	0	0	0	0	0	0	0
0	0	0	0	0	0	0	0	0	0	0	0	0	0	0	0	0	0
0	0	9E-05	0	0	0	0.0002	0.0005	0.0003	0.0013	0.0006	0.0003	0.0003	0.0226	0.0003	0.001	0.0006	0.0005
0.0071	0.0158	0.1522	0.0238	0.1225	0.0567	0.0253	0.0515	0.124	0.0134	0.0006	0.0077	0.0976	0.0155	0.0287	0	5E-05	0.0001
0	0	0	0	0	0	0	0	0	0	0	0	0	2E-05	######	0	0	0
0.0001	0	0.0005	0	3E-05	######	0	0	0	0	0	0	0	0	0	0	0	0
0	0	5E-05	0	0	0.0078	3E-05	0	2E-05	0	0	0	0	3E-05	3E-05	9E-05	0	0
0.0006	0	0.002	0.004	0	2E-05	0.0023	0.0009	0.0003	0.0003	0.0003	0.0003	0.0004	0.0005	0.0002	0.0041	0.0055	0.0001
0.0002	0.0001	0.0003	0	0.0006	0	0.0899	4E-05	4E-05	9E-05	4E-05	6E-05	4E-05	2E-05	3E-05	0.0485	0.0003	0.0003
3E-05	1E-05	0	0	0	0	######	0	0	0	0	0	0	0	0	0	0	0
0.0139	0.0061	0.0004	0.0141	0.0101	0.0342	0.0092	8E-05	0.0066	0.0012	0.0002	0.0038	0.0003	0.0433	0.0001	0.0013	0.0388	0.0579
0.0036	0.0106	0.0205	0.0507	0.0797	0.1058	0.0184	0.0205	0.0828	0.0089	0.0006	0.0046	0.0561	0.0556	0.1152	0.0007	0.0001	0.0002

Control 093	Control 095	Control 096	Control 097	Control 098	Control 101	Control 103	Control 104	Control 106	Control 108	Control 110	Control 111	Control 112	Control 115	Control 118	Control 119	Control 121	Control 123
0	0	0	0	0	0	0	0	0	0	0	0	0	0.0146	0.0793	0.0326	0	0.3484
0	0	0.0093	0	0	0	0	0	0	0	0	0	0	0	0	0	0	0
0	0	0	0	0.0058	0.0131	0	0.1099	0	0	0	0	0	0	0	0	0	0
0	0	0	0	0	0	0	0	0	0	0	0	0	0	0	0	0	0
0	0	0	0	0	0	0	0	0	0.0176	0.0037	0	0	0	0	0	0	0
0	0	0	0	0	0	0	0.0337	0	0	0	0	0	0	0	0	0	0
0	0	0	0	0.0309	0	0	0.0739	0	0.0139	0.0036	0	0	0	0	0	0	0
0	0	0	0	0	0	0	0	0	0	0	0	0	0	0	0	0	0
0	0.0077	0.0405	0	0.012	0.0103	0.0273	0	0.012	0	0.0016	0	0.0171	0	0	0	0.0046	0
0	0	0	0	0	0	0	0.0489	0	0.0028	0	0	0	0	0	0	0	0
0	0	0	0	0	0	0	0	0	0	0	0	0	0.1252	0	0	0	0
0	0	0	0	0	0	0	0	0	0	0	0	0	0	0	0	0	0
0	0.0072	0	0	0	0	0	0	0	0	0	0	0	0	0	0.0026	0	0
0	0	0	0	0	0	0	0	0	0	0	0	0	0	0	0	0	0
0	0	0	0	0	0	0	0	0	0	0	0	0	0	0	0	0	0
0	0	0	0	0	0	0	0	0	0	0	0	0	0	0	0	0	0
0	0	0	0	0	0	0	0	0	0	0	0	0	0	0	0	0	0
0	0	0	0	0	0	0	0	0	0	0	0	0	0	0	0	0	0
0	0	0	0	0	0	0	0	0	0	0	0	0	0	0	0	0	0
0.0012	0.0023	0.0446	0.0016	0.0004	0.0065	0.0011	0.0007	0.0012	0.0035	0.0007	0.0037	0	0	0.0013	0	0	0
0.0007	0.0185	0.0324	0.0006	0.0027	0.0144	0.012	0	0.0109	0.0007	0.0028	0.0226	0.0317	0.0007	0.0951	0.0015	0	0
0	0	0.0006	0.6008	0.0004	0	0	0	0	0	0	0	0	0	0	0	0	0
0	0.0003	0	0.001	0.0003	0	0	0.001	0	0	0	0	0	0	0	0	0	0
0.0025	0.0174	0.0361	0	0.0049	0.0108	0.0099	0.0011	0.0134	0.0016	0.003	0.02	0.0327	0.0004	0.1229	0.0016	0.0002	0
0	0.0844	0.0007	0	0.0034	0.0249	0.0185	0.0007	0.0018	0.0004	0.0002	0	0	0.0004	0.0256	0.0046	0.0002	0
0	0.0004	0.0002	0.0003	0.0002	0	0	0.0033	0	0	0	0	0	0	0	0	0	0
0.0006	0.0005	0.0006	0.0012	0.0004	0	0	0	0	0.0023	0	0	0	0	0	0	0	0.008
0	0	0.0055	0	0.0032	0	0	0.0349	0.0002	0.056	0.0151	0.0006	0	0	0	0.0002	0.0002	0
0	0	0	0	0	0.0003	0.0003	0.0002	0.0001	0.0004	0	0	0.259	0	0	0	0	0
0.0003	0.0003	0.0003	0.0005	0.0005	0	0	0	0	0	0	0	0	######	0	0	0	0
0.0003	0	0	0.0004	0.0054	0	0	0.0125	0	0.0054	0.0029	0.0002	0	0	0	0	7E-05	0.0011
0	0	0	0	0	0	0	0	0	0	0	0	0	0	0	0	0	0
0	0	0	0	0	0	0	0	0	0	0	0	0	######	0	0	0	0
0.0008	0.0115	0.0007	0.0013	0.0006	0.0011	0.4734	0.0004	0.0003	0.0002	0.0004	0.0007	0.0002	0	0	0.0002	7E-05	0
0.0001	0	0.0067	0.0002	6E-05	0.0137	0	4E-05	4E-05	6E-05	0.0111	0	0	0	0	0	6E-05	0
0	0	0	4E-05	3E-05	4E-05	0	4E-05	0	8E-05	4E-05	4E-05	3E-05	0	7E-05	0	3E-05	0
3E-05	0	0	0	0	4E-05	0	3E-05	0	0	0	4E-05	0	0	0	2E-05	0	0
0	0	0	0	0	0.0404	0.0001	0.0002	0.0003	0.0004	0.0001	0.0109	6E-05	0.0002	0.0006	0.0002	0.0001	0.4122
8E-05	0.0017	0.0009	3E-05	0.0009	0.0032	0.0044	0.0002	8E-05	0.0703	0.0001	0.0002	0.0002	0.0002	0.0007	0.0006	0.0001	0
0.0001	0.0002	0.0002	0.0002	0.0002	3E-05	0	4E-05	0	3E-05	0	0	0	6E-05	0.0004	8E-05	9E-05	0.0322
0	0	0	2E-05	0	0	0	0	0	0	0	0	0	0	0.0001	2E-05	0	0
0.0019	0.0191	0.0328	0.0007	0.0038	0.0128	0.0095	0.0007	0.011	0.0021	0.0034	0.0186	0.0298	1E-04	0.0892	0.0019	0.0001	0.0019
0.0001	5E-05	0.0001	0.0002	0.0001	0.0002	5E-05	0.0001	0.0004	0.0001	8E-05	0.0001	9E-05	0.1079	0.0004	0.0001	8E-05	0

Control 124	Control 126	Control 130	Control 131	COVID -19-002	COVID -19-003	COVID -19-005	COVID -19-006	COVID -19-008	COVID -19-010	COVID -19-011	COVID -19-013	COVID -19-014	COVID -19-015	COVID -19-016	COVID -19-017	COVID -19-018	COVID -19-019
0.026	0	0.0159	0	0	0	0	0	0	0	0	0	0	0	0	0	0	0
0	0	0	0	0	0.3902	0	0	0	0	0.1499	0.0115	0	0.0463	0	0.0082	0.0115	0.0105
0	0.0046	0.0074	0	0.0099	0.079	0.0907	0.0066	0.0131	0.0585	0	0.094	0.0473	0	0.0159	0.057	0.0595	0.0097
0	0	0	0	0	0	0	0	0	0	0	0	0	0	0	0	0	0
0	0	0	0	0	0.0699	0.0126	0.07	0.0111	0.0826	0.058	0.0498	0.0402	0.0246	0.0355	0.0074	0.0288	0.0185
0	0	0.0915	0	0	0	0.0989	0.0051	0.2438	0.057	0.0095	0.0072	0.0564	0.0151	0.045	0.069	0.0343	0.0197
0	0.0033	0	0	0	0.0687	0.0206	0.0822	0.0144	0.0835	0.0178	0.0458	0.0265	0.0046	0.0129	0.0078	0.0658	0.044
0	0	0	0	0	0	0	0	0	0	0	0	0	0	0	0	0	0
0.0624	0.0051	0	0.0251	0	0	0	0	0	0	0	0	0	0	0	0	0	0
0	0	0	0	0	0.0513	0.0082	0.0075	0.0099	0.0124	0	0.0141	0	0.05	0.0579	0	0.0163	0.0121
0	0	0	0	0	0	0	0	0	0	0	0	0	0	0	0	0	0
0	0.0014	0.0567	0	0	0	0	0.0014	0.6811	0.0067	0.0345	0.0037	0.0151	0.0156	0	0.0086	0	0.005
0	0	0	0	0	0	0	0	0	0	0	0	0	0	0	0.0009	0	0
0	0	0	0	0	0	0.0016	0	0	0	0	0	0	0	0	0	0	0
0	0	0	0	0	0	0	0	0	0.0042	0.0042	0	0	0	0	0	0	0.007
0	0	0	0	0	0	0	0	0.0015	0	0	0	0	0	0	0	0	0
0	0	0	0	0	0	0	0	0	0	0	0	0	0	0	0	0	0.0017
0	0	0	0	0	0	0	0.0012	0.0015	0	0.0037	0	0	0	0	0	0	0
0	0	0	0	0	0	0	0	0	0	0	0	0	0	0	0	0	0
0.0004	0.0009	0	0	0	0	0	0	0	0	0.0013	0	0	0	0	0	0	0
0.1005	0.0047	0.0004	0.1079	0.0007	0	0	0	0	0	0	0	0	0	0	0	0	0
0	0	0	0.0004	0	0	0	0	0	0	0	0	0	0	0	0	0	0
0.0024	0.0003	0	0	0.0009	0.0012	0.2282	0.0004	0.0061	0.1245	0.013	0.0682	0.1689	0.0291	0.0026	0.0913	0.092	0.0813
0.109	0.0042	0	0.0857	0	0	0	0	0	0	0	0	0	0	0	0	0	0
0.0054	0.071	0	0.0771	0	0	0	0	0	0	0	0	0	0.0004	0	0	0	0
0.0026	0	0	0	0.0003	0.0007	0.1811	0.0002	0.0055	0.0719	0.0151	0.087	0.1196	0.0582	0.0032	0.1565	0.0732	0.0919
0.0009	0.0009	0.0005	0.001	0	0	0	0	0	0	0	0	0	0	0	0	0	0
######	0.0003	0.0002	0	0	0.1813	0.0009	0.0115	0.0037	0.0052	0.063	0.0232	0.0053	0.0108	0.0011	0.0051	0.0065	0.0084
0.0001	0.0003	0	0	0	0.0009	0.001	0.0006	0.3033	0.0003	0.0614	0.0062	0.0025	0.0004	0.0001	0.0267	0.0007	0
0	0	0	0	0	0	0.0002	0.0003	0	0	0	0	0	0	0	0	0	0
0.0033	0.0059	0.0004	0	0.0004	0.0099	0.1338	0.0103	0.0239	0.0728	0.0215	0.0588	0.1259	0.0298	0.0143	0.0776	0.0683	0.0586
0	0	0	0	0	0	0	0	0	0	0	0	0	0	0	0	0	0
0	0	0	0	0	0.0002	0.0001	0	0	0	0.0005	0.0001	0	9E-05	0	0	0	0
0	0	0	0	0	0	0	0.0001	0	0	0.001	8E-05	0	0.0003	0	5E-05	7E-05	6E-05
0	0	0	0	0.0005	0	0	9E-05	0	0	0	6E-05	0	0	0	0	0	0
0	0	0	0	0	0	0	0	0	0	0	0	0	0	0	0	0	0
0	0	2E-05	0	0	0.0131	0.0011	0.0011	0.0007	0.0003	0	0.0004	0.0025	0.0009	3E-05	0	0	0
0.0003	0.0005	0.0002	0.0004	0	0	0	0	0	0	0	0	0	0	2E-05	0	4E-05	0
0.0025	3E-05	0.0063	0	3E-05	0	0	0.0008	0	0	5E-05	4E-05	0	0	0.0177	0.0002	3E-05	0
8E-05	5E-05	6E-05	0.0001	0.0001	0	6E-05	2E-05	0	4E-05	7E-05	0	0.0004	0	0.0002	0.0004	0.0007	0
0	1E-05	0	0	0	0.0001	0	0	3E-05	0	4E-05	2E-05	0	0	0	0	0	0
0.0999	0.0056	0.0004	0.0817	0.0002	0	0	0	0	0	0	0	0	0	0	0	0	1E-05
8E-06	0	2E-05	2E-05	0.0003	0	0	1E-05	0	4E-05	5E-05	1E-05	0	0	0	0	0	8E-06

COVID -19-023	COVID -19-024	COVID -19-025	COVID -19-026	COVID -19-027	COVID -19-028	COVID -19-029	COVID -19-030	COVID -19-031	COVID -19-032	COVID -19-035	COVID -19-037	COVID -19-038	COVID -19-040	COVID -19-041	COVID -19-043	COVID -19-045	COVID -19-046
0	0	0	0	0	0	0	0	0	0	0	0	0	0	0	0	0	0
0	0	0.0389	0	0	0	0	0	0	0	0	0	0	0	0	0	0	0
0	0	0	0	0	0	0	0	0	0	0.0133	0	0.0915	0	0	0	0	0.0337
0.1758	0.0963	0.0303	0.087	0.2444	0.0392	0	0.0318	0.0277	0.1506	0.0783	0.0199	0	0.0187	0	0	0	0
0	0	0	0	0.0127	0	0	0	0	0.0096	0.0103	0	0.0751	0	0	0.0068	0	######
0	0	0.0202	0.0123	0.0087	0	0	0	0	0	0	0	0.03	0.0132	0	0.0283	0	0.0119
0	0.0043	0.0537	0.0133	0.0075	0	0	0	0.0089	0	0.0081	0	0.0741	0	0	0	0	0.0124
0.2239	######	0.0909	0.0522	0.1365	0.0204	0.0175	0.2065	0.0171	0.0356	0.1438	0	0	0	0	0	0	0
0	0	0	0	0	0	0	0	0	0	0	0	0	0	0	0	0	0
0	0	0.0181	0	0.1122	0	0	0	0	0	0	0	0.051	0.0067	0.0314	0.0783	0.0156	0.0212
0	0	0	0	0	0	0	0	0	0	0	0	0	0	0	0	0	0
0.0053	0.0073	0.0052	0	0	0	0	0	0	0.0019	0	0	0	0	0	0.0227	0	0.0015
0.0722	0	0.0362	0.0064	0.0283	0.4497	0.0262	0.0181	0.0485	0.0019	0.1217	0	0	0	0	0.0033	0.0082	0
0.1619	0.1307	0.0213	0.1342	0.1068	0.0461	0.0602	0.1452	0.0138	0.0908	0.0626	0.0028	0	0	0	0.001	0	0
0.0082	0.0271	0.004	0	0.0017	0	0.0015	0.0009	0	0.1035	0.2429	0.0204	0	0	0.0046	0.0338	0	0.0011
0	0	0	0	0	0	0	0	0	0	0	0	0.0015	0.6734	0	0	0	0.0219
0.0025	######	0	0.0017	0.0011	0	0.0021	0.0008	0.0029	0.439	0	0	0.0006	0.0019	0.0035	0.5234	0.0049	0.0025
0.0631	0.0015	0.2985	0.0029	0.0805	0.0011	0	0	0.1142	0	0	0	0.002	0.0033	0.003	0.1103	0	0
0.0105	0.0006	0	0.0014	0	0.0033	0.0022	0.973	0.0026	0.0017	0.0028	0.0016	0	0	0	0	0	0
0	0	0	0	0	0	0	0	0	0	0	0.0042	0	0	0	0	0	0
0	0	0	0	0	0	0	0	0	0	0	0	0	0	0	0	0	0
0	0	0	0	0	0	0	0	0	0	0	0	0	0	0	0	0.003	0
0	######	0	0.001	0.0075	0	0	######	0	0	0	0.001	0	0	0	0.0022	0	0
0	0	0	0	0	0	0	0	0	0	0	0	0	0	0	0	0	0
0	0.0041	0	0	0	0.0005	0	######	0.0896	0.0117	0.0022	0	0	0	0	0.0019	0	######
0.0009	0.0005	0.0009	0	0.004	0.0004	0	0	0.0004	0	0	0.0006	0	0	0	0.0036	0	######
0	0	0	0	0	0	0	0	0	0	0	0	0	0	0	0	0	0
0.0243	0.0049	0.0036	0.0094	0.0532	0.0032	0.0019	0.0067	0.0154	0.0114	0.0191	0.0009	0.0162	0	0	0.0001	0.001	0.003
0	0	0.0019	0	0.0004	0	0	0	0	0	0	0	0.0065	0.0008	0	0.2054	0.001	######
0	0	0	0	0	0	0	0	0	0	0	0	0.0001	0	0	0	0	0
0.0019	0.0003	0.0016	0.0009	0.0065	0.0005	0	0.0003	0.0005	0.0021	0.0004	0.0009	0.0135	0.0003	0.0011	0.0021	0.0009	0.0042
0.1481	0.0773	0.0042	0.2368	0.0803	0.012	0.1183	0.1238	0.0169	0.1097	0.0687	0.0014	8E-05	0.0004	0.0004	0.0003	0	0.0002
0.0284	0.2567	0.0087	0.0485	0.0447	0.2996	0.0224	0.0766	0.006	0.0007	0.1945	0.001	0.0001	0.0002	0.0003	0.0038	0.0019	0.0002
0	0	0	0	0	0	0	5E-05	0	0	0	0	0	0	0	6E-05	0	6E-05
0	0	0.0002	0	0	6E-05	0	0	0	0	0	0.0001	0.0001	0.0001	0	0.0236	0	6E-05
0.0004	0.043	0.0003	0.0009	0.0148	0.0028	5E-05	0.0001	0.0002	0	1E-04	0.0002	0	0	0	0.0007	0	0
0.0002	0	0.0021	6E-05	0.0009	9E-05	0.0059	0.0001	0	0.0002	0	0	0.0011	0.3153	0.0034	0.0014	0.0058	0.3665
0	0.0001	0.0004	0	0	0	0.0002	0	0	0	0	0	0	0.0005	0	0	0	######
0.0002	0	0.0001	0	5E-05	0.0001	0	0	0.0002	0	0	0	0.0002	0	0	0.004	0.001	######
0	0	0	8E-05	0.0001	0.0002	0	0	0.0001	0	0	5E-05	9E-05	0.0001	0.0005	4E-05	0.1232	0.0001
0.133	0.0852	0.0034	0.2197	0.0537	0.0116	0.1538	0.1465	0.0121	0.0733	0.0599	0.0016	0.0001	0.0003	0.0008	0.0001	0.0007	7E-05
0	0	0	0	0	0	0	2E-05	0	0	2E-05	0	0	4E-05	0	0	0	0
0	9E-06	0	0	0	3E-05	1E-05	0	0	0	0	9E-05	0	2E-05	3E-05	7E-06	0	0

COVID -19-047	COVID -19-048	COVID -19-049	COVID -19-050	COVID -19-051	COVID -19-052	COVID -19-054	COVID -19-055	COVID -19-057	COVID -19-058	COVID -19-061	COVID -19-062	COVID -19-063	COVID -19-064	COVID -19-065	COVID -19-069
0	0	0	0	0	0	0	0	0	0	0	0	0	0	0	0
0	0	0.0251	0.0423	0	0.14	0	0.0189	0	0	0.0335	0	0	0	0	0
0	0.0444	0	0	0	0	0	0	0.0097	0	0	0	0.0102	0.0062	0.0136	0
0	0	0	0	0	0	0	0	######	0	0	0	0	0	0	0
0	0.1187	0	0	0	0	0	0	0.0093	0	0	0	0	0.0149	0	0
0	0.0231	0	0	0	0	0	0	######	0	0	0	0	0	0.0099	0
0	0.0353	0	0.0059	0	0	0	0	######	0	0	0	0	0	0	0
0	0	0	0	0	0	0	0	0	0	0	0	0	0	0	0
0	0	0	0	0	0	0	0	0	0	0	0	0	0	0	0
0	0.062	0	0	0	0	0	0	0	0	0	0	0	0	0	0
0	0	0	0	0	0	0	0	0	0	0	0	0	0	0	0.0037
0	0.0044	0	0	0	0.003	0	0	0	0.0038	0	0	0	0	0	0
0	0.0046	0	0	0	0	0	0.0013	0	0	0.0017	0.0279	0	0.0353	0	0.0287
0	0.0017	0	0	0	0	0	0	0	0	0	0	0	0	0	0
0	0.0015	0.0021	0	0	0	0	0	0	0	0	0	0.0667	0.0207	0	0.4264
0	0	0.0017	0.0033	0.0028	0.0018	0.0031	0.0016	0	0.2851	0	0	0	0	0	0
0	0.0013	0	0	0.0033	0.0015	0	0	0	0	0	0	0	0	0	0
0	0.0013	0	0	0	0	0	0	0	0	0	0	0	0	0	0.3071
0	0	0	0	0	0	0	0	0	0	0	0	0	0	0	0
0	0	0	0	0	0	0	0	0	0	0	0	0	0	0	0
0	0	0	0	0	0	0	0	0	0	0	0	0	0	0	0
0	0	0	0	0	0	0	0	0	0	0	0	0	0	0	0
0	0	0	0.0014	0	0	0	0	0.0005	0	0.0006	0.0006	0.0004	0.0021	0	0.0008
0	0	0	0	0	0	0	0	0	0	0	0	0	0	0	0
0	0.0004	0.0006	0	0	0	0	0.0255	0	0	0.0126	0	0	0	0	0.0326
0.0009	0	0	0.0052	0.0003	0	0	0	0.0006	0.0008	0.0007	0.0018	0.0003	0.0035	0.0008	0
0	0	0	0	0	0	0	0	0	0	0	0	0	0	0	0
0.0002	0.0626	0	0.0001	0	0.0192	0	0	0.0008	0	0.0023	0.001	0.0002	0.0218	0.0003	0.0002
0.0004	0.003	0	0.0002	0	0	0.0001	0	0.0039	0.0007	0	0	0	0	0	0
0	0	0	0	0	0	0	0	0	0	0	0	0	0	0	0
0.0006	0.0119	0	0.0011	0.0002	0.001	0.0001	0	0.002	0.0005	0.0009	0.0007	0.0002	0.0148	0.0004	0.0003
0	0.0002	0	0	0	0	0	0	0	0	0	0	0	0	0	0
0.0018	0.0004	0	0	0.0001	0	0	8E-05	0	0	0	5E-05	0	0	0.0001	9E-05
0	0	0	0	0	0	6E-05	0	7E-05	9E-05	0	0	0	0	0	6E-05
0	0.0001	0	5E-05	0	0	0	0	0	0	0	0	0.0004	0	0	0
0	0	0	6E-05	0.0001	0.0311	0.0002	0.0111	0.5649	0.3262	0	0	0	2E-05	0	0.0003
0.0009	0.001	0.0061	0.0038	0.0026	0.0037	0.0041	0.0022	0.0004	0.2459	0.0004	0	9E-05	0.0004	0	0.0004
0	0	0	0	0	0	0	0	0	0	8E-05	0.0036	0.0189	6E-05	0	0
4E-05	0.001	6E-05	0	6E-05	0.0018	0.0005	0.0021	0.001	0.0003	0.0012	3E-05	3E-05	2E-05	4E-05	0
0.0234	3E-05	0	0	2E-05	0	0	2E-05	0	0	0.0008	0.0421	0.1258	0	0.1898	0
0	0.0001	0	4E-05	0	0	0	0	0	0	0	0	0	0	0	0
0	0	0	2E-05	0	0	0	0	0	0	0	0	0	0	0	0
0	0	6E-05	3E-05	0	0	0	0	0	0	0	8E-06	0	0.0015	0	0.0092

Supplementary Table S6. The detailed values of oral fungal α−diversity index									
sample	reads	label	ace	chao	shannon	simpson	coverage	observed_otus	Subgroup
Control001	1161	0.97	177.997272	94.428571	1.767396	0.30397	0.972438	59	Control
Control097	1161	0.97	137.666851	96.2	2.580427	0.133773	0.981051	50	Control
Control096	1161	0.97	178.409985	162.12	3.374096	0.06289	0.958656	117	Control
Control095	1161	0.97	206.637282	147.375	2.928213	0.0952	0.969854	73	Control
Control094	1161	0.97	77.946138	72.909091	2.772583	0.115293	0.984496	59	Control
Control093	1161	0.97	137.847812	83	0.271306	0.922439	0.986219	23	Control
Control092	1161	0.97	139.985502	98	2.471787	0.123441	0.979328	52	Control
Control091	1161	0.97	152.307424	101.071429	2.443229	0.188255	0.97416	70	Control
Control090	1161	0.97	257.490748	177.045455	3.162196	0.079683	0.953488	112	Control
Control089	1161	0.97	73.537462	79.666667	1.687042	0.404479	0.982773	48	Control
Control088	1161	0.97	120.286762	67.5	1.389625	0.488166	0.983635	39	Control
Control087	1161	0.97	126.962117	84.1	2.696581	0.145929	0.981051	61	Control
Control086	1161	0.97	103.938776	95	2.012645	0.213327	0.978467	45	Control
Control085	1161	0.97	292.997627	237.896552	3.458815	0.087888	0.940568	157	Control
Control084	1161	0.97	256.064431	92.333333	1.034312	0.497006	0.982773	29	Control
Control083	1161	0.97	250.497894	227.25	3.419134	0.098505	0.951766	131	Control
Control082	1161	0.97	178.079118	167.935484	2.922534	0.19167	0.957795	130	Control
Control081	1161	0.97	97.82309	86	2.205264	0.261154	0.981912	56	Control
Control080	1161	0.97	89.510452	92.25	3.022942	0.078344	0.981912	66	Control
Control079	1161	0.97	226.686252	168.666667	2.591343	0.189894	0.965547	82	Control
Control078	1161	0.97	147.722023	96.625	1.951543	0.349036	0.977606	56	Control
Control077	1161	0.97	141.235226	135.058824	3.158188	0.100824	0.968992	98	Control
Control076	1161	0.97	36.774656	35	1.751404	0.322276	0.993109	28	Control
Control075	1161	0.97	71.701698	56.6	2.510962	0.142266	0.988803	41	Control
Control074	1161	0.97	41.042707	38	2.182768	0.219415	0.993971	35	Control
Control073	1161	0.97	55.89319	54	1.683156	0.311148	0.989664	32	Control
Control072	1161	0.97	63.251698	71.25	2.427898	0.147477	0.98708	45	Control
Control071	1161	0.97	182.490922	149.375	2.552403	0.169763	0.969854	75	Control
Control070	1161	0.97	107.671029	79.666667	2.272615	0.191708	0.981051	54	Control
Control069	1161	0.97	157.723771	152.272727	2.690339	0.103313	0.963824	74	Control
Control098	1161	0.97	211.253769	154.875	2.178837	0.292463	0.96124	93	Control
Control099	1161	0.97	154.673251	140.12	3.219959	0.112961	0.96727	112	Control
Control100	1161	0.97	113.667758	95.083333	3.16343	0.074337	0.980189	74	Control
Control101	1161	0.97	102.438804	76	2.797279	0.103325	0.983635	57	Control
Control132	1161	0.97	176.845816	137	1.084853	0.664035	0.968131	63	Control
Control131	1161	0.97	72.521783	71.5	2.394603	0.156644	0.984496	46	Control
Control130	1161	0.97	199.474053	165.75	2.285088	0.207982	0.97416	57	Control
Control129	1161	0.97	97.55556	72.090909	2.180812	0.198816	0.981912	53	Control
Control128	1161	0.97	114.951615	69.1	1.902713	0.231159	0.981051	46	Control
Control127	1161	0.97	67.283243	65	0.385624	0.896084	0.981912	35	Control
Control126	1161	0.97	165.315906	138.866667	2.48526	0.220617	0.96727	92	Control
Control125	1161	0.97	80.126864	79.6	1.489677	0.480916	0.979328	52	Control
Control124	1161	0.97	206.159832	140.6	2.435497	0.189716	0.968131	74	Control
Control123	1161	0.97	87.682168	115.2	0.984722	0.626667	0.976744	45	Control
Control122	1161	0.97	97.616243	72	2.562433	0.111701	0.982773	53	Control
Control121	1161	0.97	67.387778	72.75	0.359458	0.899413	0.983635	30	Control
Control120	1161	0.97	182.419281	113.75	2.586502	0.14757	0.975022	63	Control
Control119	1161	0.97	138.559826	98	1.665967	0.427619	0.976744	59	Control
Control068	1161	0.97	96.087629	88.333333	1.758392	0.391339	0.985357	43	Control
Control118	1161	0.97	90.577774	87.428571	2.219891	0.220958	0.978467	66	Control
Control116	1161	0.97	75.814498	77.5	2.318262	0.240947	0.984496	52	Control
Control115	1161	0.97	60.032739	54	1.583295	0.42197	0.98708	39	Control
Control114	1161	0.97	610.137824	310.473684	2.025692	0.42142	0.927649	127	Control
Control113	1161	0.97	156.795172	122.142857	1.88887	0.329628	0.97416	60	Control
Control112	1161	0.97	89.015108	84	2.830503	0.086192	0.980189	61	Control
Control111	1161	0.97	123.767447	94.2	2.387957	0.136805	0.981051	48	Control

Control110	1161	0.97	196.456922	139.235294	2.881161	0.114993	0.964686	91	Control
Control109	1161	0.97	332.296971	116.5	1.401353	0.468256	0.983635	31	Control
Control108	1161	0.97	373.92205	272.433333	3.273436	0.091161	0.92851	159	Control
Control107	1161	0.97	106.20442	97.5	2.49073	0.138103	0.982773	50	Control
Control106	1161	0.97	152.323888	182	2.749048	0.129735	0.962102	96	Control
Control105	1161	0.97	111.040825	86.25	3.036538	0.073136	0.981912	60	Control
Control104	1161	0.97	682.365975	398.037037	2.834972	0.234671	0.905254	176	Control
Control103	1161	0.97	103.127162	102.6	1.951251	0.26949	0.980189	52	Control
Control117	1161	0.97	70.174464	62.363636	2.165975	0.238344	0.985357	50	Control
Control067	1161	0.97	147.801943	140.2	1.813566	0.381645	0.975022	59	Control
Control102	1161	0.97	226.802311	132	1.431463	0.46194	0.96124	77	Control
Control065	1161	0.97	241.825829	167.625	2.551312	0.145116	0.966408	75	Control
Control030	1161	0.97	156.4265	221.5	2.599654	0.128893	0.963824	78	Control
Control029	1161	0.97	106.184557	88	2.890661	0.076768	0.981912	53	Control
Control028	1161	0.97	250.715284	204.035714	3.603339	0.063906	0.950043	145	Control
Control027	1161	0.97	461.362636	379.24	4.056134	0.047697	0.916451	193	Control
Control026	1161	0.97	132.573564	89.666667	2.88151	0.089767	0.979328	59	Control
Control025	1161	0.97	133.132313	62.2	1.842743	0.2628	0.985357	35	Control
Control024	1161	0.97	346.557825	136.8125	2.885238	0.123646	0.963824	83	Control
Control023	1161	0.97	199.826896	119	1.541679	0.458064	0.972438	57	Control
Control022	1161	0.97	171.700977	98	1.651188	0.419121	0.975883	56	Control
Control021	1161	0.97	63.543488	62.142857	2.592412	0.154108	0.988803	51	Control
Control019	1161	0.97	114.043134	119	3.158632	0.075746	0.979328	73	Control
Control018	1161	0.97	129.086128	122	2.870741	0.151686	0.971576	89	Control
Control017	1161	0.97	229.172201	98	1.326716	0.397766	0.975883	44	Control
Control016	1161	0.97	185.644686	223.333333	2.447286	0.131725	0.972438	58	Control
Control015	1161	0.97	278.873177	228.666667	3.134304	0.147931	0.944014	142	Control
Control014	1161	0.97	189.114359	144.2	2.815957	0.10165	0.975022	63	Control
Control013	1161	0.97	268.306557	228.3125	3.314455	0.106653	0.950043	125	Control
Control012	1161	0.97	302.056335	222.071429	2.179614	0.260891	0.950043	104	Control
Control011	1161	0.97	196.243984	122.8	2.698382	0.11555	0.971576	70	Control
Control010	1161	0.97	307.407096	183	3.35518	0.059498	0.96124	93	Control
Control009	1161	0.97	200.045282	99.75	1.526234	0.376948	0.975022	49	Control
Control008	1161	0.97	92.614325	67.25	1.099694	0.420936	0.984496	29	Control
Control007	1161	0.97	204.293264	154	2.429498	0.233694	0.959518	107	Control
Control006	1161	0.97	183.199277	132.461538	2.823661	0.139974	0.968992	84	Control
Control005	1161	0.97	154.664984	133.111111	2.727948	0.138568	0.972438	78	Control
Control004	1161	0.97	245.659438	167.2	0.911305	0.729502	0.970715	55	Control
Control003	1161	0.97	200.515224	164.666667	1.836512	0.428431	0.965547	78	Control
Control002	1161	0.97	378.782573	227	2.191876	0.353077	0.939707	122	Control
Control066	1161	0.97	198.236722	180	3.079379	0.126982	0.953488	127	Control
Control031	1161	0.97	153.015447	149.333333	3.335041	0.076395	0.965547	106	Control
Control032	1161	0.97	141.290336	118.909091	3.311081	0.058579	0.975022	82	Control
Control020	1161	0.97	138.905418	91.428571	0.747475	0.7634	0.977606	45	Control
Control034	1161	0.97	109.55903	97.166667	2.380188	0.160608	0.980189	55	Control
Control051	1161	0.97	177.038291	113.5	1.80514	0.420458	0.969854	71	Control
Control052	1161	0.97	168.87007	102.333333	2.074063	0.223672	0.972438	61	Control
Control053	1161	0.97	195.346513	159	1.86382	0.447339	0.965547	81	Control
Control054	1161	0.97	130.592023	101.857143	2.322021	0.196342	0.978467	59	Control
Control055	1161	0.97	149.302086	151.4	2.902043	0.108971	0.963824	94	Control
Control056	1161	0.97	285.706879	195.333333	3.662793	0.03446	0.962963	95	Control
Control050	1161	0.97	200.368186	199.055556	1.723382	0.49828	0.949182	104	Control
Control057	1161	0.97	175.470426	98.625	2.640645	0.133016	0.977606	58	Control
Control059	1161	0.97	165.531759	136	3.267342	0.081162	0.971576	88	Control
Control060	1161	0.97	165.55496	139.428571	2.411592	0.149719	0.971576	64	Control
Control033	1161	0.97	236.142838	209.315789	3.567941	0.069534	0.953488	134	Control
Control062	1161	0.97	195.748629	151.117647	3.027117	0.107805	0.962963	98	Control
Control063	1161	0.97	174.171798	148.583333	3.164894	0.073661	0.96727	90	Control

Control064	1161	0.97	231.205168	114.8	2.178124	0.247257	0.971576	62	Control
Control058	1161	0.97	82.868001	72.857143	2.11927	0.252051	0.984496	51	Control
Control049	1161	0.97	272.137619	219.142857	3.212013	0.108676	0.947459	132	Control
Control061	1161	0.97	225.268676	234.125	2.829556	0.137077	0.959518	99	Control
Control047	1161	0.97	210.930062	141.75	2.774164	0.131168	0.966408	80	Control
Control048	1161	0.97	129.307083	93	2.184379	0.27445	0.976744	66	Control
Control036	1161	0.97	99.407298	69.111111	2.458787	0.121642	0.982773	48	Control
Control037	1161	0.97	91.568196	54.3	1.064402	0.619386	0.984496	39	Control
Control038	1161	0.97	142.686778	105.1	2.829536	0.124422	0.976744	70	Control
Control039	1161	0.97	205.686318	153	2.045832	0.41296	0.965547	88	Control
Control040	1161	0.97	117.461298	112.615385	1.851581	0.369993	0.971576	72	Control
Control035	1161	0.97	291.621806	222	2.609647	0.310248	0.944014	142	Control
Control042	1161	0.97	225.877276	183.142857	3.184155	0.093588	0.955211	120	Control
Control043	1161	0.97	170.892434	89.909091	0.94206	0.704604	0.975022	53	Control
Control044	1161	0.97	168.277257	168.473684	3.268661	0.075702	0.960379	114	Control
Control045	1161	0.97	351.799689	254.066667	3.489481	0.075347	0.946598	128	Control
Control046	1161	0.97	115.734121	109.25	3.014629	0.078958	0.97416	73	Control
Control041	1161	0.97	204.134584	137.333333	2.846813	0.114629	0.970715	75	Control
CC054	1161	0.97	33.936017	30.25	0.31763	0.896694	0.991387	19	COVID-19-N
CC053	1161	0.97	206.513438	174.875	3.017554	0.115036	0.96727	87	COVID-19-N
CC052	1161	0.97	128.50878	99	1.650383	0.469497	0.976744	60	COVID-19-N
CC050	1161	0.97	316.391581	212.545455	2.770248	0.123633	0.955211	92	COVID-19-N
CC049	1161	0.97	848.249242	157	0.331303	0.885793	0.985357	21	COVID-19-N
CC047	1161	0.97	101.777416	85.5	1.508825	0.448113	0.981051	47	COVID-19-N
CC046	1161	0.97	139.489599	118.214286	1.779718	0.335989	0.96727	68	COVID-19-N
CC045	1161	0.97	184.473842	124.333333	2.1401	0.206212	0.970715	62	COVID-19-N
CC044	1161	0.97	613.555391	374.588235	2.374443	0.333259	0.922481	139	COVID-19-N
CC055	1161	0.97	89.077493	59.857143	1.117057	0.614781	0.984496	38	COVID-19-N
CC043	1161	0.97	268.371365	234.55	3.66058	0.050965	0.946598	140	COVID-19-N
CC048	1161	0.97	531.467374	339.125	2.70861	0.264882	0.919035	157	COVID-19-N
CC056	1161	0.97	195.426883	147.571429	2.466214	0.205444	0.964686	89	COVID-19-N
CC064	1161	0.97	277.61828	196.65	2.386726	0.310738	0.950043	114	COVID-19-N
CC059	1161	0.97	333.901698	100	0.516028	0.824471	0.981912	30	COVID-19-N
CC060	1161	0.97	462.156197	266	1.823761	0.319221	0.94832	89	COVID-19-N
CC061	1161	0.97	94.610612	90.714286	2.937261	0.09463	0.979328	71	COVID-19-N
CC062	1161	0.97	106.576254	104.4	3.32563	0.061818	0.979328	86	COVID-19-N
CC063	1161	0.97	151.625555	147.428571	2.394678	0.197268	0.971576	72	COVID-19-N
CC065	1161	0.97	372.916051	104	1.632065	0.333212	0.981912	34	COVID-19-N
CC066	1161	0.97	164.047733	124.75	2.987428	0.127041	0.969854	95	COVID-19-N
CC067	1161	0.97	51.730104	51	0.975967	0.565194	0.986219	27	COVID-19-N
CC070	1161	0.97	43.715735	42.166667	2.042991	0.202291	0.990525	33	COVID-19-N
CC068	1161	0.97	84.514889	71.666667	2.630853	0.119444	0.985357	49	COVID-19-N
CC069	1161	0.97	102.670873	86.75	2.833069	0.129892	0.982773	63	COVID-19-N
CC042	1161	0.97	86.238635	77	1.031194	0.592878	0.982773	39	COVID-19-N
CC057	1161	0.97	301.417131	119.857143	0.928618	0.663082	0.972438	49	COVID-19-N
CC041	1161	0.97	220.26437	114.666667	2.846907	0.08374	0.973299	63	COVID-19-N
CC004	1161	0.97	53.104915	51.5	0.859756	0.706987	0.98708	34	COVID-19-N
CC039	1161	0.97	718.616042	403.157895	2.685407	0.258907	0.91559	153	COVID-19-N
CC002	1161	0.97	217.174298	128.75	1.124585	0.54092	0.976744	41	COVID-19-N
CC003	1161	0.97	376.928122	254.111111	2.5696	0.279783	0.931955	140	COVID-19-N
CC005	1161	0.97	416.045656	264.142857	2.974694	0.193126	0.937123	139	COVID-19-N
CC006	1161	0.97	487.081935	306.105263	1.891302	0.465179	0.92851	127	COVID-19-N
CC007	1161	0.97	451.622772	288.956522	2.312381	0.366585	0.92851	141	COVID-19-N
CC040	1161	0.97	235.77996	103.25	1.556162	0.37698	0.980189	40	COVID-19-N
CC011	1161	0.97	224.422011	216.115385	3.031965	0.197648	0.945736	141	COVID-19-N
CC012	1161	0.97	152.157191	100	1.938388	0.384781	0.976744	61	COVID-19-N
CC013	1161	0.97	419.519902	236.387097	2.602492	0.289792	0.933678	142	COVID-19-N
CC015	1161	0.97	121.960285	124.0625	2.848519	0.103129	0.970715	89	COVID-19-N

CC016	1161	0.97	270.579709	199.103448	2.594603	0.299152	0.947459	136	COVID-19-N
CC017	1161	0.97	218.41396	150.5	2.890446	0.123193	0.96124	101	COVID-19-N
CC010	1161	0.97	427.255455	318.789474	2.524395	0.316374	0.929371	144	COVID-19-N
CC019	1161	0.97	466.168334	235.894737	2.568414	0.221655	0.94143	116	COVID-19-N
CC018	1161	0.97	500.968322	283	2.710498	0.273871	0.925065	154	COVID-19-N
CC038	1161	0.97	387.82118	269	2.398014	0.332885	0.933678	136	COVID-19-N
CC036	1161	0.97	219.751529	153.8125	3.357808	0.065045	0.963824	100	COVID-19-N
CC032	1161	0.97	162.833334	128.090909	3.05133	0.100125	0.972438	83	COVID-19-N
CC030	1161	0.97	176.953146	144.111111	2.456564	0.235057	0.969854	78	COVID-19-N
CC034	1161	0.97	107.938782	134	1.852562	0.378338	0.971576	68	COVID-19-N
CC028	1161	0.97	235.052693	176.5	1.834912	0.36759	0.964686	74	COVID-19-N
CC027	1161	0.97	191.150007	177.357143	3.0825	0.138241	0.955211	130	COVID-19-N
CC024	1161	0.97	130.767842	145.583333	3.022738	0.116359	0.969854	96	COVID-19-N
CC023	1161	0.97	324.443953	214.12	2.604299	0.210568	0.94143	123	COVID-19-N
CC021	1161	0.97	172.474607	154.4	3.199116	0.09717	0.966408	105	COVID-19-N
CC029	1161	0.97	103.245946	102.25	2.419849	0.236817	0.976744	73	COVID-19-N
CC001	1161	0.97	203.569711	162.105263	3.217839	0.091982	0.96124	110	COVID-19-C
CC051	1161	0.97	187.602734	209.5625	3.660507	0.062593	0.956934	133	COVID-19-C
CC037	1161	0.97	272.802393	205.8	3.553794	0.063068	0.94832	135	COVID-19-C
CC035	1161	0.97	241.82044	162	3.080292	0.092937	0.957795	106	COVID-19-C
CC033	1161	0.97	200.048146	222.352941	3.538085	0.068903	0.952627	135	COVID-19-C
CC031	1161	0.97	209.395477	202.545455	3.587085	0.055074	0.950904	130	COVID-19-C
CC008	1161	0.97	386.676842	278.4	2.930965	0.138677	0.940568	122	COVID-19-C
CC025	1161	0.97	210.754853	220.333333	3.04407	0.111144	0.94832	122	COVID-19-C
CC022	1161	0.97	166.126034	168	2.991241	0.111667	0.971576	80	COVID-19-C
CC020	1161	0.97	96.967943	70.666667	2.671143	0.122271	0.985357	48	COVID-19-C
CC014	1161	0.97	451.441662	337.75	2.865736	0.240612	0.925926	155	COVID-19-C
CC009	1161	0.97	494.725222	319.5	2.761421	0.276614	0.921619	162	COVID-19-C
CC058	1161	0.97	33.207015	32	1.222445	0.401215	0.992248	26	COVID-19-C
CC026	1161	0.97	163.484216	118.230769	1.353496	0.520929	0.968131	67	COVID-19-C
CC071	1161	0.97	94.584173	82.5	2.404128	0.179839	0.983635	54	COVID-19-C

Supplementary Table S7. The different degree of phylum level (p value) among control (n=132), COVID-19-N (n=56), and COVID-19-C (n=15) groups															
ID	Control.median	Control.mean	Control.se	COVID-19.median	COVID-19.mean	COVID-19.se	COVID-19-C.median	COVID-19-C.mean	COVID-19-C.se	p-value	z-score	Sig_mark	q-value	fixp	fixps
Ascomycota	0.8321(0.6878,0.9221)	0.761667	0.01934	0.9334(0.8501,0.9633)	0.890036	0.015805	0.9283(0.8887,0.9553)	0.87461	0.04144	6.6E-06	-4.51	***	4E-05	<0.0001	***<0.0001
Basidiomycota	0.1025(0.0325,0.2375)	0.173363	0.01723	0.0388(0.0103,0.1147)	0.078534	0.014131	0.0422(0.0247,0.0932)	0.10749	0.04068	0.00019	-3.73	***	0.0006	0.0002	***0.0002
Mucoromycota	0.002(0.0007,0.0146)	0.029713	0.00983	0.0009(0.0002,0.0032)	0.005506	0.001596	0.0008(0.0001,0.0023)	0.00294	0.00128	0.00366	-2.91	**	0.0073	0.0037	** 0.0037
Rozellomycota	<0.0001(<0.0001,<0.0001)	7.11E-04	6.33E-04	<0.0001(<0.0001,<0.0001)	0.000267	0.000264	<0.0001(<0.0001,<0.0001)	0	0	9.47E-02	-1.67		0.1136	0.0947	0.0947
Chytridiomycota	<0.0001(<0.0001,<0.0001)	0.000122	6.09E-05	<0.0001(<0.0001,<0.0001)	0.001203	0.000629	<0.0001(<0.0001,<0.0001)	0.00023	0.00022	0.00843	-2.63	**	0.0126	0.0084	** 0.0084
Zoopagomycota	<0.0001(<0.0001,<0.0001)	3.25E-05	2.13E-05	<0.0001(<0.0001,<0.0001)	1.18E-05	6.52E-06	<0.0001(<0.0001,<0.0001)	0	0	0.32876	-0.98		0.3288	0.3288	0.3288

Supplementary Table S7. The different degree of genus level (p value) among control (n=132), COVID-19-N (n=56), and COVID-19-C (n=15) groups															
ID	Control.median	Control.mean	Control.se	COVID-19.median	COVID-19.mean	COVID-19.se	COVID-19-C.median	COVID-19-C.mean	COVID-19-C.se	p-value	z-score	Sig_mark	q-value	fixp	fixps
Acrodictys	0.0341(0.0091,0.3007)	0.182386	0.02101	0.1043(0.0181,0.6448)	2.95E-01	4.25E-02	0.0775(0.0294,0.1452)	0.19051	0.07115	6.03E-02	-1.88		0.1649	0.0603	0.0603
Zanclospora	0.0732(0.0146,0.3212)	0.219291	0.02397	0.0179(0.0067,0.0827)	0.147946	0.037686	0.014(0.0087,0.0561)	0.0578	0.02297	0.00121	-3.24	**	0.0098	0.0012	** 0.0012
Aspergillus	0.0177(0.0048,0.089)	0.084935	0.01325	0.0207(0.0062,0.0629)	8.51E-02	2.52E-02	0.0652(0.018,0.1176)	0.10379	0.03261	0.30746	-1.02		0.4634	0.3075	0.3075
Blumeria	0.0016(0.0006,0.0102)	0.045477	0.0103	0.0008(0.0002,0.0035)	0.022175	0.013184	0.0102(0.0016,0.2573)	0.12993	0.04323	0.00151	-3.17	**	0.0108	0.0015	** 0.0015
Cryptococcus	0.0163(0.002,0.0547)	0.062697	0.01085	<0.0001(<0.0001,<0.0001)	3.84E-05	1.63E-05	<0.0001(<0.0001,<0.0001)	4E-05	2.5E-05	4.92E-31	-11.6	***	2E-28	<0.0001	***<0.0001
Cladosporium	0.0046(0.0012,0.0396)	0.040226	0.00606	0.0017(0.0005,0.0172)	0.036696	0.012744	0.0166(0.001,0.0618)	0.05078	0.01927	0.03687	-2.09	*	0.1212	0.0369	* 0.0369
Malassezia	0.0083(0.0014,0.0549)	0.043633	0.00702	0.0011(0.0003,0.0142)	0.027039	0.009708	0.0012(0.0007,0.0312)	0.03976	0.01913	0.0005	-3.48	***	0.0051	0.0005	***0.0005
Chrysosphaeria	0.0001(<0.0001,0.0004)	0.003136	0.00136	<0.0001(<0.0001,0.0005)	6.29E-02	2.23E-02	<0.0001(<0.0001,0.0107)	0.07847	0.0582	0.60559	-0.52		0.7291	0.6056	0.6056
Candida	0.0005(0.0001,0.0015)	0.007518	0.00199	0.0028(0.0011,0.0111)	5.00E-02	2.33E-02	0.005(0.0011,0.0734)	0.07523	0.03645	1.1E-08	-5.72	***	9E-07	<0.0001	***<0.0001
Diversispora	0.0017(0.0006,0.0146)	0.029246	0.00983	0.0009(0.0001,0.003)	4.88E-03	1.51E-03	0.0008(<0.0001,0.0022)	0.00288	0.00127	5.04E-03	-2.8	**	0.0259	0.005	** 0.005
Raffaelea	0.0006(0.0002,0.0024)	0.022379	0.0093	0.0016(0.0005,0.0064)	5.41E-03	1.44E-03	0.0022(0.0011,0.004)	0.00348	0.00093	0.0013	-3.22	**	0.0098	0.0013	** 0.0013
Hydnobolites	<0.0001(<0.0001,<0.0001)	0.001496	0.00072	0.0002(<0.0001,0.0018)	0.042453	0.022936	0.0003(0.0001,0.0009)	0.03189	0.03139	1.33E-05	-4.35	***	0.0003	<0.0001	***<0.0001
Arthrographis	0.0001(<0.0001,0.0008)	0.018769	1.05E-02	<0.0001(<0.0001,0.0001)	1.50E-04	6.68E-05	<0.0001(<0.0001,<0.0001)	8.7E-05	4.1E-05	0.00014	-3.81	***	0.0021	0.0001	***0.0001
Simplicillium	0.0004(0.0002,0.0045)	0.007369	0.00124	0.0026(0.0006,0.0417)	0.022506	0.004189	0.0016(0.0003,0.0083)	0.01324	0.00644	2.15E-04	-3.7	***	0.0028	0.0002	***0.0002
Saccharomyces	<0.0001(<0.0001,0.0003)	0.001336	0.00044	0.0005(<0.0001,0.0026)	2.26E-02	1.12E-02	0.0007(0.0001,0.0216)	0.04922	0.02599	1.4E-06	-4.83	***	5E-05	<0.0001	***<0.0001
Alternaria	0.0005(0.0002,0.0042)	0.011654	3.17E-03	0.0001(<0.0001,0.0007)	2.75E-03	9.26E-04	0.0001(<0.0001,0.0021)	0.00275	0.00134	0.00083	-3.34	***	0.0075	0.0008	***0.0008
Milospium	0.0002(<0.0001,0.001)	0.012121	0.00686	<0.0001(<0.0001,0.0003)	5.19E-04	2.50E-04	<0.0001(<0.0001,0.001)	0.00087	0.00049	0.01694	-2.39	*	0.0671	0.0169	* 0.0169
Filobasidiaceae_gen_Incertae_sedis	0.0004(0.0001,0.0017)	0.006671	1.37E-03	0.0014(0.0003,0.0161)	0.009638	0.001953	0.0002(0.0001,0.0016)	0.00592	0.003	0.00635	-2.73	**	0.0311	0.0063	** 0.0063
Russula	0.0003(<0.0001,0.0023)	0.007204	1.97E-03	0.0004(0.0002,0.0031)	7.62E-03	3.11E-03	0.0014(0.0001,0.0076)	0.00439	0.00148	0.41328	-0.82		0.575	0.4133	0.4133
Penicillium	<0.0001(<0.0001,0.0003)	8.67E-03	7.28E-03	<0.0001(<0.0001,0.0002)	0.004359	0.002444	0.0001(<0.0001,0.0003)	0.00038	0.0002	0.61901	-0.5		0.7421	0.619	0.619
Talaromyces	0.0002(<0.0001,0.0007)	9.27E-03	2.50E-03	<0.0001(<0.0001,0.0002)	1.09E-03	4.66E-04	<0.0001(<0.0001,0.0006)	0.00063	0.00034	0.00012	-3.85	***	0.0018	0.0001	***0.0001
Schizophyllum	<0.0001(<0.0001,<0.0001)	8.26E-03	7.37E-03	<0.0001(<0.0001,<0.0001)	7.00E-04	4.59E-04	<0.0001(<0.0001,<0.0001)	0.00034	0.00032	0.40008	-0.84		0.5642	0.4001	0.4001
Pleurotus	<0.0001(<0.0001,0.0002)	4.67E-03	1.92E-03	<0.0001(<0.0001,0.0002)	3.52E-03	1.77E-03	0.0006(<0.0001,0.0016)	0.02024	0.01889	0.01569	-2.42	*	0.0631	0.0157	* 0.0157
Parafuscosporella	0.0008(0.0001,0.0053)	4.61E-03	1.02E-03	0.0024(0.0004,0.0092)	0.006971	0.002106	0.0003(0.0002,0.0014)	0.00235	0.00104	0.03554	-2.1	*	0.1183	0.0355	* 0.0355
Trigonopsis	<0.0001(<0.0001,<0.0001)	7.45E-03	7.26E-03	<0.0001(<0.0001,<0.0001)	4.15E-05	3.77E-05	<0.0001(<0.0001,<0.0001)	0	0	0.00114	-3.25	**	0.0098	0.0011	** 0.0011
Gibberella	<0.0001(<0.0001,<0.0001)	1.08E-05	4.77E-06	<0.0001(<0.0001,0.0007)	1.17E-02	4.98E-03	<0.0001(<0.0001,0.0051)	0.01269	0.00885	2.3E-14	-7.63	***	5E-12	<0.0001	***<0.0001
Saprochaete	<0.0001(<0.0001,<0.0001)	6.06E-03	5.89E-03	<0.0001(<0.0001,<0.0001)	2.49E-05	2.49E-05	<0.0001(<0.0001,<0.0001)	7.1E-05	6.5E-05	0.07381	-1.79		0.1828	0.0738	0.0738

Neomicrosphaeropsis	<0.0001(<0.0001,<0.0001)	5.38E-04	2.39E-04	<0.0001(<0.0001,<0.0001)	1.12E-02	1.09E-02	0.0001(<0.0001,0.0018)	0.00222	0.00145	0.03013	-2.17	*	0.1036	0.0301	* 0.0301
Angustimassarina	0.0002(<0.0001,0.0026)	2.79E-03	4.37E-04	0.0011(0.0002,0.0105)	5.31E-03	8.76E-04	0.0007(0.0001,0.0022)	0.00293	0.00131	0.00303	-2.96	**	0.0173	0.003	** 0.003
Wallemia	<0.0001(<0.0001,0.0002)	5.04E-03	3.88E-03	<0.0001(<0.0001,<0.0001)	0.000112	5.37E-05	<0.0001(<0.0001,<0.0001)	0.00222	0.00177	0.00526	-2.79	**	0.026	0.0053	** 0.0053
Diaporthe	<0.0001(<0.0001,0.0002)	4.99E-03	2.62E-03	<0.0001(<0.0001,<0.0001)	0.00E+00	0.00E+00	<0.0001(<0.0001,<0.0001)	3.3E-05	3.3E-05	2E-11	-6.7	***	3E-09	<0.0001	***<0.0001
Nigrospora	<0.0001(<0.0001,<0.0001)	7.29E-04	4.13E-04	<0.0001(<0.0001,0.0001)	4.08E-03	3.23E-03	<0.0001(<0.0001,0.0004)	0.02106	0.01921	0.02006	-2.33	*	0.0745	0.0201	* 0.0201
Trametes	<0.0001(<0.0001,0.0001)	3.46E-03	1.96E-03	<0.0001(<0.0001,<0.0001)	2.18E-03	1.39E-03	<0.0001(<0.0001,<0.0001)	4.1E-06	4.1E-06	0.02661	-2.22	*	0.0922	0.0266	* 0.0266
Fantasmomyces	<0.0001(<0.0001,0.0013)	2.10E-03	5.46E-04	0.0008(0.0001,0.0079)	0.004554	0.0009	0.0003(<0.0001,0.0036)	0.00222	0.0009	0.00126	-3.23	**	0.0098	0.0013	** 0.0013
Phaeosphaeria	<0.0001(<0.0001,0.0003)	3.69E-03	2.26E-03	<0.0001(<0.0001,<0.0001)	0.000208	0.00012	<0.0001(<0.0001,<0.0001)	8.2E-06	8.2E-06	0.00031	-3.6	***	0.0036	0.0003	***0.0003
Fusarium	<0.0001(<0.0001,0.0002)	2.39E-03	8.42E-04	<0.0001(<0.0001,0.0002)	0.002021	0.001057	<0.0001(<0.0001,0.0009)	0.00177	0.00096	0.29671	-1.04		0.454	0.2967	0.2967
Naganishia	<0.0001(<0.0001,<0.0001)	3.23E-03	1.99E-03	<0.0001(<0.0001,<0.0001)	1.26E-05	1.16E-05	<0.0001(<0.0001,<0.0001)	0.00013	0.00013	0.00011	-3.87	***	0.0018	0.0001	***0.0001
Filobasidium	<0.0001(<0.0001,<0.0001)	1.43E-03	8.62E-04	<0.0001(<0.0001,0.0001)	0.003527	0.00193	<0.0001(<0.0001,0.0002)	0.00169	0.00154	0.0918	-1.69		0.217	0.0918	0.0918
Acremonium	<0.0001(<0.0001,<0.0001)	2.85E-03	2.40E-03	<0.0001(<0.0001,<0.0001)	0.000374	0.000318	<0.0001(<0.0001,<0.0001)	2.6E-05	1.4E-05	0.47703	-0.71		0.624	0.477	0.477
Ophiostoma	<0.0001(<0.0001,0.0001)	2.66E-03	2.20E-03	<0.0001(<0.0001,<0.0001)	0.000104	2.39E-05	<0.0001(<0.0001,<0.0001)	7.9E-05	4.9E-05	0.14669	-1.45		0.2906	0.1467	0.1467
Trichomonascus	<0.0001(<0.0001,<0.0001)	2.69E-03	2.64E-03	<0.0001(<0.0001,<0.0001)	0	0	<0.0001(<0.0001,<0.0001)	1.5E-05	1.5E-05	0.01719	-2.38	*	0.0675	0.0172	* 0.0172
Botrytis	<0.0001(<0.0001,0.0001)	2.25E-03	1.50E-03	<0.0001(<0.0001,<0.0001)	8.54E-05	7.1E-05	<0.0001(<0.0001,0.0002)	0.00177	0.00116	5.2E-05	-4.05	***	0.001	<0.0001	***<0.0001
Galactomyces	<0.0001(<0.0001,0.0001)	4.50E-04	1.41E-04	<0.0001(<0.0001,<0.0001)	0.004727	0.004644	<0.0001(<0.0001,<0.0001)	0.0001	7E-05	0.21987	-1.23		0.4131	0.2199	0.2199
Aureobasidium	<0.0001(<0.0001,<0.0001)	8.12E-04	4.07E-04	<0.0001(<0.0001,<0.0001)	0.003606	0.002233	<0.0001(<0.0001,<0.0001)	0.00051	0.00038	0.33637	-0.96		0.4859	0.3364	0.3364
Yarrowia	<0.0001(<0.0001,<0.0001)	2.30E-03	1.10E-03	<0.0001(<0.0001,<0.0001)	0.00015	0.000149	<0.0001(<0.0001,<0.0001)	0.00026	0.00026	4.5E-06	-4.59	***	0.0001	<0.0001	***<0.0001
Udeniomyces	<0.0001(<0.0001,<0.0001)	2.26E-03	1.84E-03	<0.0001(<0.0001,<0.0001)	1.53E-05	8.35E-06	<0.0001(<0.0001,<0.0001)	3.1E-05	2.7E-05	0.00186	-3.11	**	0.012	0.0019	** 0.0019
Coprinellus	<0.0001(<0.0001,<0.0001)	1.56E-03	7.24E-04	<0.0001(<0.0001,<0.0001)	1.54E-03	1.53E-03	<0.0001(<0.0001,<0.0001)	0	0	0.00018	-3.75	***	0.0024	0.0002	***0.0002
Zygotorulaspora	<0.0001(<0.0001,<0.0001)	8.48E-04	6.13E-04	<0.0001(<0.0001,0.0001)	3.10E-03	1.85E-03	<0.0001(<0.0001,<0.0001)	4.3E-05	2.8E-05	0.14201	-1.47		0.2885	0.142	0.142
Verrucaria	<0.0001(<0.0001,<0.0001)	2.13E-03	1.40E-03	<0.0001(<0.0001,<0.0001)	5.8E-06	5.8E-06	<0.0001(<0.0001,<0.0001)	0	0	0.0026	-3.01	**	0.0155	0.0026	** 0.0026
Golubevia	<0.0001(<0.0001,<0.0001)	2.12E-03	1.76E-03	<0.0001(<0.0001,<0.0001)	0	0	<0.0001(<0.0001,<0.0001)	1.8E-05	1.8E-05	0.0001	-3.88	***	0.0018	0.0001	***0.0001
Togniniella	<0.0001(<0.0001,<0.0001)	2.03E-03	1.17E-03	<0.0001(<0.0001,<0.0001)	4.96E-05	2.05E-05	<0.0001(<0.0001,0.0001)	0.0002	0.00011	0.05452	-1.92		0.1564	0.0545	0.0545
Hannaella	<0.0001(<0.0001,<0.0001)	7.11E-04	6.51E-04	<0.0001(<0.0001,<0.0001)	0.00266	0.002306	<0.0001(<0.0001,0.0003)	0.00053	0.00032	0.00498	-2.81	**	0.0259	0.005	** 0.005
Bjerkandera	<0.0001(<0.0001,<0.0001)	1.37E-03	1.23E-03	<0.0001(<0.0001,<0.0001)	6.69E-04	4.55E-04	<0.0001(<0.0001,<0.0001)	0.00107	0.00107	0.22043	-1.23		0.4131	0.2204	0.2204
Leptospora	<0.0001(<0.0001,<0.0001)	0.001544	9.01E-04	<0.0001(<0.0001,<0.0001)	0.000456	0.000322	<0.0001(<0.0001,<0.0001)	0.00015	0.00014	0.04394	-2.01	*	0.1306	0.0439	* 0.0439
Cercospora	<0.0001(<0.0001,<0.0001)	1.68E-03	1.40E-03	<0.0001(<0.0001,<0.0001)	0.00E+00	0.00E+00	<0.0001(<0.0001,<0.0001)	0	0	9E-07	-4.91	***	4E-05	<0.0001	***<0.0001
Irpex	<0.0001(<0.0001,<0.0001)	6.70E-04	6.10E-04	<0.0001(<0.0001,<0.0001)	2.11E-03	2.08E-03	<0.0001(<0.0001,<0.0001)	0.00087	0.00085	0.76179	-0.3		0.7869	0.7618	0.7618
Peniophora	<0.0001(<0.0001,<0.0001)	8.83E-04	5.51E-04	<0.0001(<0.0001,<0.0001)	1.72E-03	1.57E-03	<0.0001(<0.0001,<0.0001)	5.6E-06	5.6E-06	0.01902	-2.35	*	0.0719	0.019	* 0.019
Pseudoveronaea	<0.0001(<0.0001,<0.0001)	3.60E-06	3.23E-06	<0.0001(<0.0001,<0.0001)	3.47E-03	2.98E-03	<0.0001(<0.0001,<0.0001)	0.00104	0.00101	9.8E-07	-4.9	***	4E-05	<0.0001	***<0.0001
Purpureocillium	<0.0001(<0.0001,<0.0001)	0.001541	0.001	<0.0001(<0.0001,<0.0001)	0.00E+00	0.00E+00	<0.0001(<0.0001,<0.0001)	4.4E-06	4.4E-06	4.3E-05	-4.09	***	0.0008	<0.0001	***<0.0001

Apiotrichum	<0.0001(<0.0001,0.0001)	1.40E-03	5.66E-04	<0.0001(<0.0001,<0.0001)	0.000142	1.03E-04	<0.0001(<0.0001,<0.0001)	0.00027	0.00023	0.00047	-3.5	***	0.005	0.0005	***0.0005
Rhexotheciu m	<0.0001(<0.0001,0.0001)	1.28E-03	7.55E-04	<0.0001(<0.0001,<0.0001)	1.24E-04	6.57E-05	<0.0001(<0.0001,0.0005)	0.00071	0.00035	0.06611	-1.84		0.1752	0.0661	0.0661
Vishniacozy ma	<0.0001(<0.0001,<0.0001)	1.29E-03	1.01E-03	<0.0001(<0.0001,<0.0001)	2.06E-04	1.92E-04	<0.0001(<0.0001,<0.0001)	0.00015	0.00015	0.01177	-2.52	*	0.0505	0.0118	* 0.0118
Pichia	<0.0001(<0.0001,<0.0001)	4.25E-05	2.5E-05	<0.0001(<0.0001,0.0001)	1.35E-03	6.23E-04	<0.0001(<0.0001,0.0002)	0.00664	0.00515	0.00082	-3.34	***	0.0075	0.0008	***0.0008
Symmetrosp ora	<0.0001(<0.0001,<0.0001)	0.000577	0.00053	<0.0001(<0.0001,<0.0001)	1.81E-03	1.24E-03	<0.0001(<0.0001,<0.0001)	2.6E-05	1.4E-05	0.62408	-0.49		0.746	0.6241	0.6241
Stagonospor opsis	<0.0001(<0.0001,<0.0001)	0.001225	0.00088	<0.0001(<0.0001,<0.0001)	0.00E+00	0.00E+00	<0.0001(<0.0001,<0.0001)	0	0	3E-06	-4.67	***	9E-05	<0.0001	***<0.0001
Cystofilobas idium	<0.0001(<0.0001,<0.0001)	1.07E-03	7.57E-04	<0.0001(<0.0001,<0.0001)	0.000153	8.97E-05	<0.0001(<0.0001,<0.0001)	7E-06	7E-06	0.06907	-1.82		0.1781	0.0691	0.0691
Neosetopho ma	<0.0001(<0.0001,<0.0001)	6.05E-04	4.10E-04	<0.0001(<0.0001,<0.0001)	9.22E-05	8.23E-05	<0.0001(<0.0001,<0.0001)	0.00395	0.00386	0.38772	-0.86		0.5527	0.3877	0.3877
Ramularia	<0.0001(<0.0001,<0.0001)	4.47E-07	4.47E-07	<0.0001(<0.0001,<0.0001)	2.41E-03	2.37E-03	<0.0001(<0.0001,<0.0001)	4.7E-05	4.7E-05	0.00012	-3.85	***	0.0018	0.0001	***0.0001
Kazachstani a	<0.0001(<0.0001,<0.0001)	5.55E-04	3.55E-04	<0.0001(<0.0001,0.0002)	0.000772	0.000629	<0.0001(<0.0001,0.0003)	0.00108	0.00093	0.02102	-2.31	*	0.0774	0.021	* 0.021
Dioszegia	<0.0001(<0.0001,<0.0001)	9.34E-04	8.58E-04	<0.0001(<0.0001,<0.0001)	0.000123	1.05E-04	<0.0001(<0.0001,<0.0001)	0	0	1.23E-01	-1.54		0.2644	0.1227	0.1227
Cutaneotrich osporon	<0.0001(<0.0001,<0.0001)	0.000317	0.00025	<0.0001(<0.0001,<0.0001)	2.97E-05	1.18E-05	<0.0001(<0.0001,0.0002)	0.00561	0.00512	0.03747	-2.08	*	0.1212	0.0375	* 0.0375
Setophaeosp haeria	<0.0001(<0.0001,<0.0001)	9.64E-04	8.91E-04	<0.0001(<0.0001,<0.0001)	0	0.00E+00	<0.0001(<0.0001,<0.0001)	0	0	2.70E-03	-3	**	0.0158	0.0027	** 0.0027
Tilletiopsis	<0.0001(<0.0001,<0.0001)	2.34E-04	1.82E-04	<0.0001(<0.0001,<0.0001)	0.001632	0.001545	<0.0001(<0.0001,<0.0001)	0.00011	0.00011	0.40298	-0.84		0.5663	0.403	0.403
Leucosporid ium	<0.0001(<0.0001,<0.0001)	3.69E-05	3.28E-05	<0.0001(<0.0001,<0.0001)	0.002109	0.002014	<0.0001(<0.0001,<0.0001)	0	0	2.36E-03	-3.04	**	0.0145	0.0024	** 0.0024
Sporobolom yces	<0.0001(<0.0001,<0.0001)	7.86E-04	4.00E-04	<0.0001(<0.0001,<0.0001)	0.00027	0.000239	<0.0001(<0.0001,<0.0001)	7.3E-06	7.3E-06	0.00059	-3.44	***	0.0059	0.0006	***0.0006
Entorrhiza	<0.0001(<0.0001,<0.0001)	0.000258	8.14E-05	<0.0001(<0.0001,0.0005)	0.001154	0.000361	0.0001(<0.0001,0.0006)	0.00122	0.00069	0.00017	-3.76	***	0.0024	0.0002	***0.0002
Pyrenochaet opsis	<0.0001(<0.0001,<0.0001)	0.000855	0.0007	<0.0001(<0.0001,<0.0001)	5.44E-05	5.2E-05	<0.0001(<0.0001,<0.0001)	1.4E-05	1.4E-05	0.04065	-2.05	*	0.1243	0.0406	* 0.0406
Didymella	<0.0001(<0.0001,<0.0001)	6.73E-04	3.37E-04	<0.0001(<0.0001,<0.0001)	0.000432	0.000425	<0.0001(<0.0001,<0.0001)	4.1E-05	4.1E-05	0.00309	-2.96	**	0.0173	0.0031	** 0.0031
Ceriporia	<0.0001(<0.0001,<0.0001)	7.54E-04	5.06E-04	<0.0001(<0.0001,<0.0001)	0.00017	0.000104	<0.0001(<0.0001,<0.0001)	0	0	0.00769	-2.67	**	0.0356	0.0077	** 0.0077
Rhodotorula	<0.0001(<0.0001,<0.0001)	2.59E-04	1.07E-04	<0.0001(<0.0001,<0.0001)	7.32E-05	2.42E-05	<0.0001(<0.0001,<0.0001)	0.00446	0.00437	0.25456	-1.14		0.4209	0.2546	0.2546
Ustilaginoid ea	<0.0001(<0.0001,<0.0001)	1.08E-05	3.68E-06	<0.0001(<0.0001,0.0001)	0.001427	0.000609	<0.0001(<0.0001,0.0002)	0.00145	0.00095	2.9E-07	-5.13	***	2E-05	<0.0001	***<0.0001
Tausonia	<0.0001(<0.0001,<0.0001)	6.30E-04	3.69E-04	<0.0001(<0.0001,<0.0001)	0.000267	0.000186	<0.0001(<0.0001,<0.0001)	0.00018	0.00016	0.99763	-0		0.9976	0.9976	0.9976
Trichophaea	<0.0001(<0.0001,<0.0001)	8.84E-06	7.03E-06	<0.0001(<0.0001,<0.0001)	0.001756	0.001735	<0.0001(<0.0001,<0.0001)	2.6E-05	2.6E-05	4.11E-03	-2.87	**	0.0222	0.0041	** 0.0041
Parasympodi ella	<0.0001(<0.0001,0.0001)	3.93E-04	1.17E-04	<0.0001(<0.0001,0.0003)	0.000382	0.000149	0.0008(<0.0001,0.0017)	0.00177	0.00068	6.33E-04	-3.42	***	0.0061	0.0006	***0.0006
Tilletia	<0.0001(<0.0001,<0.0001)	0.000745	0.00057	<0.0001(<0.0001,<0.0001)	2.62E-05	2.56E-05	<0.0001(<0.0001,<0.0001)	0	0	0.00051	-3.48	***	0.0051	0.0005	***0.0005
Dipodascus	<0.0001(<0.0001,<0.0001)	0.000339	1.78E-04	<0.0001(<0.0001,<0.0001)	0.000927	0.000903	<0.0001(<0.0001,<0.0001)	9.4E-05	6.8E-05	0.01579	-2.41	*	0.0631	0.0158	* 0.0158
Starmerella	<0.0001(<0.0001,<0.0001)	7.76E-05	7.52E-05	<0.0001(<0.0001,<0.0001)	0.0015	0.000895	<0.0001(<0.0001,<0.0001)	5.7E-05	2.7E-05	1.3E-06	-4.85	***	5E-05	<0.0001	***<0.0001
Rozellomyc ota_gen_Inc ertae_sedis	<0.0001(<0.0001,<0.0001)	0.000711	0.00063	<0.0001(<0.0001,<0.0001)	0	0	<0.0001(<0.0001,<0.0001)	0	0	5.14E-03	-2.8	**	0.0259	0.0051	** 0.0051
Auricularia	<0.0001(<0.0001,<0.0001)	0.000485	0.00029	<0.0001(<0.0001,<0.0001)	0.000493	0.000432	<0.0001(<0.0001,<0.0001)	0	0	0.06191	-1.87		0.1673	0.0619	0.0619

Sterigmatomyces	<0.0001(<0.0001,<0.0001)	0.000361	0.00024	<0.0001(<0.0001,<0.0001)	0.000133	0.000122	<0.0001(<0.0001,<0.0001)	0.00227	0.0015	0.07625	-1.77		0.1877	0.0763	0.0763
Tuber	<0.0001(<0.0001,0.0002)	0.000264	5.5E-05	0.0002(<0.0001,0.001)	0.000768	0.000155	0.0001(<0.0001,0.0004)	0.00074	0.00041	1.3E-05	-4.36	***	0.0003	<0.0001	***<0.0001
Blastobotrys	<0.0001(<0.0001,<0.0001)	6.44E-07	6.4E-07	<0.0001(<0.0001,<0.0001)	0.001552	0.001545	<0.0001(<0.0001,<0.0001)	6.7E-06	6.7E-06	0.09692	-1.66		0.2265	0.0969	0.0969
Papiliotrema	<0.0001(<0.0001,<0.0001)	0.000151	0.00015	<0.0001(<0.0001,<0.0001)	2.77E-05	1.26E-05	<0.0001(<0.0001,0.0001)	0.00436	0.0043	0.00769	-2.67	**	0.0356	0.0077	** 0.0077
Phlebia	<0.0001(<0.0001,<0.0001)	0	0	<0.0001(<0.0001,<0.0001)	1.52E-03	1.52E-03	<0.0001(<0.0001,<0.0001)	5.7E-06	5.7E-06	4.15E-02	-2.04	*	0.125	0.0415	* 0.0415
Debaryomyces	<0.0001(<0.0001,<0.0001)	0.000452	0.00026	<0.0001(<0.0001,<0.0001)	0.000336	0.000332	<0.0001(<0.0001,<0.0001)	0.00022	0.00019	1.1E-05	-4.4	***	0.0002	<0.0001	***<0.0001
Ascomycota_gen_Incertae_sedis	<0.0001(<0.0001,<0.0001)	0.000123	3.2E-05	<0.0001(<0.0001,<0.0001)	0.000785	0.000283	0.0002(<0.0001,0.0016)	0.00132	0.00069	0.00361	-2.91	**	0.0197	0.0036	** 0.0036
Henningsia	<0.0001(<0.0001,<0.0001)	2.05E-07	2E-07	<0.0001(<0.0001,<0.0001)	0.001399	0.001369	<0.0001(<0.0001,0.0001)	5.9E-05	2.3E-05	6.5E-07	-4.98	***	4E-05	<0.0001	***<0.0001
Knufia	<0.0001(<0.0001,<0.0001)	0.000583	0.00032	<0.0001(<0.0001,<0.0001)	8.57E-07	8.57E-07	<0.0001(<0.0001,<0.0001)	0.00013	0.00013	0.0007	-3.39	***	0.0066	0.0007	***0.0007
Mycosphaerellaceae_gen_Incertae_sedis	<0.0001(<0.0001,<0.0001)	0.000285	0.00015	<0.0001(<0.0001,<0.0001)	0.000529	0.000232	<0.0001(<0.0001,0.0003)	0.00073	0.0006	0.14424	-1.46		0.2885	0.1442	0.1442
Flammulina	<0.0001(<0.0001,<0.0001)	5.76E-04	5.26E-04	<0.0001(<0.0001,<0.0001)	1.05E-05	8.62E-06	<0.0001(<0.0001,<0.0001)	0	0	9.47E-02	-1.67		0.2225	0.0947	0.0947
Typhula	<0.0001(<0.0001,<0.0001)	0.00057	0.00038	<0.0001(<0.0001,<0.0001)	2.7E-06	1.89E-06	<0.0001(<0.0001,<0.0001)	0	0	0.00131	-3.21	**	0.0098	0.0013	** 0.0013
Coprinopsis	<0.0001(<0.0001,<0.0001)	4.88E-04	3.10E-04	<0.0001(<0.0001,<0.0001)	3.27E-06	1.86E-06	<0.0001(<0.0001,<0.0001)	0.00068	0.00068	0.04488	-2.01	*	0.1306	0.0449	* 0.0449
Glomus	<0.0001(<0.0001,<0.0001)	0.000278	2.73E-04	<0.0001(<0.0001,<0.0001)	0.000612	0.000608	<0.0001(<0.0001,<0.0001)	4.6E-05	2.6E-05	0.03374	-2.12	*	0.1141	0.0337	* 0.0337
Clavispora	<0.0001(<0.0001,<0.0001)	0.000522	0.00048	<0.0001(<0.0001,<0.0001)	7.38E-06	4.91E-06	<0.0001(<0.0001,<0.0001)	0	0	4.26E-01	-0.8		0.581	0.4257	0.4257
Austropleospora	<0.0001(<0.0001,<0.0001)	0.000481	0.00046	<0.0001(<0.0001,<0.0001)	6.65E-05	6.54E-05	<0.0001(<0.0001,<0.0001)	0	0	0.27538	-1.09		0.4275	0.2754	0.2754
Russulales_gen_Incertae_sedis	<0.0001(<0.0001,<0.0001)	0.000501	0.00049	<0.0001(<0.0001,<0.0001)	0	0	<0.0001(<0.0001,<0.0001)	0	0	0.04464	-2.01	*	0.1306	0.0446	* 0.0446
Chytridium	<0.0001(<0.0001,<0.0001)	1.14E-04	6.08E-05	<0.0001(<0.0001,<0.0001)	0.000847	0.000536	<0.0001(<0.0001,<0.0001)	0.00022	0.00022	1.56E-01	-1.42		0.3085	0.1565	0.1565
Ahmadea	<0.0001(<0.0001,<0.0001)	0.00049	0.00048	<0.0001(<0.0001,<0.0001)	1.18E-05	5.17E-06	<0.0001(<0.0001,<0.0001)	6.1E-06	6.1E-06	0.88569	-0.14		0.89	0.8857	0.8857
Eupenidiella	<0.0001(<0.0001,<0.0001)	0.000496	0.00047	<0.0001(<0.0001,<0.0001)	0	0	<0.0001(<0.0001,<0.0001)	0	0	0.018	-2.37	*	0.0693	0.018	* 0.018
Plectosphaerella	<0.0001(<0.0001,<0.0001)	4.68E-04	4.52E-04	<0.0001(<0.0001,<0.0001)	1.61E-05	1.48E-05	<0.0001(<0.0001,<0.0001)	0.00015	0.00014	0.1329	-1.5		0.2809	0.1329	0.1329
Ascotricha	<0.0001(<0.0001,<0.0001)	0.000212	0.00013	<0.0001(<0.0001,<0.0001)	0.000644	0.000593	<0.0001(<0.0001,<0.0001)	0	0	0.12173	-1.55		0.2637	0.1217	0.1217
Recurvomyces	<0.0001(<0.0001,<0.0001)	0.000458	0.00045	<0.0001(<0.0001,<0.0001)	0.00E+00	0.00E+00	<0.0001(<0.0001,<0.0001)	7.6E-06	7.6E-06	0.10973	-1.6		0.239	0.1097	0.1097
Thielaviopsis	<0.0001(<0.0001,<0.0001)	0.000453	0.00024	<0.0001(<0.0001,<0.0001)	1.46E-06	1.46E-06	<0.0001(<0.0001,<0.0001)	0	0	0.01226	-2.5	*	0.0515	0.0123	* 0.0123
Spiromastix	<0.0001(<0.0001,<0.0001)	0.00045	4.40E-04	<0.0001(<0.0001,<0.0001)	0	0	<0.0001(<0.0001,<0.0001)	0	0	0.02444	-2.25	*	0.0854	0.0244	* 0.0244
Lentinus	<0.0001(<0.0001,<0.0001)	0.000443	0.0004	<0.0001(<0.0001,<0.0001)	1.11E-06	1.11E-06	<0.0001(<0.0001,<0.0001)	0	0	2.22E-02	-2.29	*	0.0804	0.0222	* 0.0222
Thecaphora	<0.0001(<0.0001,<0.0001)	0.000425	0.00029	<0.0001(<0.0001,<0.0001)	0.00E+00	0.00E+00	<0.0001(<0.0001,<0.0001)	8.7E-06	8.7E-06	0.05292	-1.94		0.1529	0.0529	0.0529
Heitmania	<0.0001(<0.0001,0.0002)	0.000233	5.7E-05	<0.0001(<0.0001,0.0004)	0.000428	0.000222	<0.0001(<0.0001,<0.0001)	6.9E-05	4.9E-05	8.56E-02	-1.72		0.2036	0.0856	0.0856
Engyodontium	<0.0001(<0.0001,<0.0001)	2.90E-04	2.45E-04	<0.0001(<0.0001,<0.0001)	0.000291	0.000152	<0.0001(<0.0001,<0.0001)	3E-05	1.8E-05	0.48241	-0.7		0.6291	0.4824	0.4824

Calosphaeria	<0.0001(<0.0001,<0.0001)	0.000236	8.7E-05	<0.0001(<0.0001,0.0005)	3.58E-04	8.90E-05	<0.0001(<0.0001,<0.0001)	0.0002	0.00011	0.06771	-1.83		0.1771	0.0677	0.0677
Flavodon	<0.0001(<0.0001,<0.0001)	0.000396	0.00037	<0.0001(<0.0001,<0.0001)	4.91E-06	4.91E-06	<0.0001(<0.0001,<0.0001)	4.3E-06	4.3E-06	0.125	-1.53		0.2681	0.125	0.125
Psathyrella	<0.0001(<0.0001,<0.0001)	0.000155	9.7E-05	<0.0001(<0.0001,<0.0001)	1.23E-06	1.23E-06	<0.0001(<0.0001,<0.0001)	0.00204	0.00204	0.2453	-1.16		0.4209	0.2453	0.2453
Ophiosphaerella	<0.0001(<0.0001,<0.0001)	3.30E-04	3.28E-04	<0.0001(<0.0001,<0.0001)	0.000128	8.43E-05	<0.0001(<0.0001,<0.0001)	0	0	2.04E-01	-1.27		0.3869	0.2037	0.2037
Nakaseomyces	<0.0001(<0.0001,<0.0001)	3.94E-07	3.94E-07	<0.0001(<0.0001,<0.0001)	0.000883	0.000785	<0.0001(<0.0001,<0.0001)	1.9E-06	1.9E-06	1.7E-06	-4.79	***	5E-05	<0.0001	***<0.0001
Dothidea	<0.0001(<0.0001,<0.0001)	0.000371	0.00028	<0.0001(<0.0001,<0.0001)	0	0	<0.0001(<0.0001,<0.0001)	0	0	0.01322	-2.48	*	0.0539	0.0132	* 0.0132
Periconia	<0.0001(<0.0001,<0.0001)	3.03E-04	1.76E-04	<0.0001(<0.0001,<0.0001)	9.74E-05	2.63E-05	<0.0001(<0.0001,<0.0001)	0.00023	0.00021	0.2536	-1.14		0.4209	0.2536	0.2536
Roussoella	<0.0001(<0.0001,<0.0001)	0.000369	0.00033	<0.0001(<0.0001,<0.0001)	0	0	<0.0001(<0.0001,<0.0001)	0	0	0.00514	-2.8	**	0.0259	0.0051	** 0.0051
Thelebolus	<0.0001(<0.0001,<0.0001)	0.000362	3.46E-04	<0.0001(<0.0001,<0.0001)	1.53E-05	1.53E-05	<0.0001(<0.0001,<0.0001)	0	0	0.03474	-2.11	*	0.1166	0.0347	* 0.0347
Acrocalymma	<0.0001(<0.0001,<0.0001)	0.00036	2.51E-04	<0.0001(<0.0001,<0.0001)	1.02E-06	1.02E-06	<0.0001(<0.0001,<0.0001)	0	0	0.01209	-2.51	*	0.0513	0.0121	* 0.0121
Diutina	<0.0001(<0.0001,<0.0001)	1.05E-05	7.9E-06	<0.0001(<0.0001,<0.0001)	0.000431	0.000362	<0.0001(<0.0001,<0.0001)	0.00143	0.00141	0.00516	-2.8	**	0.0259	0.0052	** 0.0052
Vanrija	<0.0001(<0.0001,<0.0001)	1.74E-06	9.15E-07	<0.0001(<0.0001,<0.0001)	3.46E-06	2.03E-06	<0.0001(<0.0001,<0.0001)	0.00305	0.0021	0.1465	-1.45		0.2906	0.1465	0.1465
Coniochaeta	<0.0001(<0.0001,<0.0001)	3.38E-04	3.27E-04	<0.0001(<0.0001,<0.0001)	2.48E-05	2.48E-05	<0.0001(<0.0001,<0.0001)	0	0	0.14082	-1.47		0.2885	0.1408	0.1408
Yamadazyma	<0.0001(<0.0001,<0.0001)	5.54E-05	5.30E-05	<0.0001(<0.0001,<0.0001)	0.000511	0.000502	<0.0001(<0.0001,<0.0001)	0.00065	0.00065	0.8661	-0.17		0.8809	0.8661	0.8661
Pseudocercospora	<0.0001(<0.0001,<0.0001)	3.46E-04	2.69E-04	<0.0001(<0.0001,<0.0001)	0	0	<0.0001(<0.0001,<0.0001)	0	0	0.00706	-2.69	**	0.0336	0.0071	** 0.0071
Lecanicillium	<0.0001(<0.0001,<0.0001)	0.000341	2.63E-04	<0.0001(<0.0001,<0.0001)	0.00E+00	0.00E+00	<0.0001(<0.0001,<0.0001)	0	0	0.00968	-2.59	**	0.0424	0.0097	** 0.0097
Bovista	<0.0001(<0.0001,<0.0001)	5.95E-06	2.4E-06	<0.0001(<0.0001,<0.0001)	0.000004	2.34E-06	<0.0001(<0.0001,<0.0001)	0.0029	0.0029	0.93345	-0.08		0.9357	0.9334	0.9334
Botryosphaeria	<0.0001(<0.0001,<0.0001)	0.000294	2.13E-04	<0.0001(<0.0001,<0.0001)	8.81E-05	8.55E-05	<0.0001(<0.0001,<0.0001)	0	0	0.07677	-1.77		0.1879	0.0768	0.0768
Lentinula	<0.0001(<0.0001,<0.0001)	0.000128	9.6E-05	<0.0001(<0.0001,<0.0001)	4.63E-04	4.57E-04	<0.0001(<0.0001,<0.0001)	0	0	0.42849	-0.79		0.581	0.4285	0.4285
Trichoderma	<0.0001(<0.0001,<0.0001)	2.51E-04	1.73E-04	<0.0001(<0.0001,<0.0001)	0.000162	9.67E-05	<0.0001(<0.0001,<0.0001)	3.9E-05	3.9E-05	0.87793	-0.15		0.8843	0.8779	0.8779
Pseudoramichloridium	<0.0001(<0.0001,<0.0001)	0.000318	3.13E-04	<0.0001(<0.0001,<0.0001)	0	0	<0.0001(<0.0001,<0.0001)	1E-05	7.1E-06	0.06193	-1.87		0.1673	0.0619	0.0619
Zeloasperisporium	<0.0001(<0.0001,<0.0001)	3.08E-06	3.08E-06	<0.0001(<0.0001,<0.0001)	6.00E-05	4.87E-05	<0.0001(<0.0001,<0.0001)	0.0025	0.00244	0.00017	-3.76	***	0.0024	0.0002	***0.0002
Keissleriella	<0.0001(<0.0001,<0.0001)	3.08E-04	2.98E-04	<0.0001(<0.0001,<0.0001)	0	0	<0.0001(<0.0001,<0.0001)	0	0	0.01322	-2.48	*	0.0539	0.0132	* 0.0132
Hortaea	<0.0001(<0.0001,<0.0001)	0.00E+00	#######	<0.0001(<0.0001,<0.0001)	0.000683	6.72E-04	<0.0001(<0.0001,<0.0001)	0.00013	0.00013	0.00028	-3.64	***	0.0033	0.0003	***0.0003
Cordyceps	<0.0001(<0.0001,<0.0001)	2.78E-04	2.55E-04	<0.0001(<0.0001,<0.0001)	4.11E-05	1.11E-05	<0.0001(<0.0001,<0.0001)	2.9E-05	1.6E-05	3.94E-01	-0.85		0.5577	0.3942	0.3942
Capnodiales_gen_Incertae_sedis	<0.0001(<0.0001,<0.0001)	2.72E-04	1.72E-04	<0.0001(<0.0001,<0.0001)	6.03E-05	5.82E-05	<0.0001(<0.0001,<0.0001)	0	0	0.28163	-1.08		0.4355	0.2816	0.2816
Dendryphiella	<0.0001(<0.0001,<0.0001)	0.00E+00	#######	<0.0001(<0.0001,<0.0001)	1.1E-05	6.01E-06	<0.0001(<0.0001,<0.0001)	0.00255	0.00255	0.00144	-3.19	**	0.0105	0.0014	** 0.0014
Bradymyces	<0.0001(<0.0001,<0.0001)	2.92E-04	2.46E-04	<0.0001(<0.0001,<0.0001)	9.46E-07	9.46E-07	<0.0001(<0.0001,<0.0001)	0	0	1.28E-01	-1.52		0.274	0.1284	0.1284
Pseudomicrostroma	<0.0001(<0.0001,<0.0001)	1.74E-05	1.60E-05	<0.0001(<0.0001,<0.0001)	0.000627	0.000453	<0.0001(<0.0001,<0.0001)	5.1E-06	5.1E-06	0.55836	-0.59		0.7105	0.5584	0.5584

Rhodosporidiobolus	<0.0001(<0.0001,<0.0001)	0.00E+00	#######	<0.0001(<0.0001,<0.0001)	1.08E-05	5.37E-06	<0.0001(<0.0001,<0.0001)	0.00243	0.0024	5.57E-05	-4.03	***	0.001	<0.0001	***<0.0001
Agaricus	<0.0001(<0.0001,<0.0001)	0.00E+00	#######	<0.0001(<0.0001,<0.0001)	0.000638	0.000627	<0.0001(<0.0001,<0.0001)	1.3E-05	1.3E-05	5.6E-06	-4.54	***	0.0001	<0.0001	***<0.0001
Trichomerium	<0.0001(<0.0001,<0.0001)	1.66E-04	1.09E-04	<0.0001(<0.0001,<0.0001)	0.00024	0.000179	<0.0001(<0.0001,<0.0001)	1.3E-05	8.9E-06	0.73963	-0.33		0.7869	0.7396	0.7396
Ciliophora	<0.0001(<0.0001,<0.0001)	2.52E-04	1.24E-04	<0.0001(<0.0001,<0.0001)	3.61E-06	2.21E-06	<0.0001(<0.0001,<0.0001)	0.00011	0.0001	0.06666	-1.83		0.1755	0.0667	0.0667
Peniophorella	<0.0001(<0.0001,<0.0001)	2.57E-04	2.43E-04	<0.0001(<0.0001,<0.0001)	0.00E+00	0.00E+00	<0.0001(<0.0001,<0.0001)	0	0	0.00514	-2.8	**	0.0259	0.0051	** 0.0051
Epicoccum	<0.0001(<0.0001,<0.0001)	3.62E-05	2.35E-05	<0.0001(<0.0001,<0.0001)	4.74E-04	4.01E-04	<0.0001(<0.0001,<0.0001)	0.00011	6.1E-05	0.01925	-2.34	*	0.0721	0.0192	* 0.0192
Pestalotiopsis	<0.0001(<0.0001,<0.0001)	2.48E-04	2.06E-04	<0.0001(<0.0001,<0.0001)	0	0	<0.0001(<0.0001,<0.0001)	0	0	0.14379	-1.46		0.2885	0.1438	0.1438
Ramophialophora	<0.0001(<0.0001,<0.0001)	2.45E-04	2.31E-04	<0.0001(<0.0001,<0.0001)	0.00E+00	0.00E+00	<0.0001(<0.0001,<0.0001)	0	0	0.06006	-1.88		0.1649	0.0601	0.0601
Phyllachora	<0.0001(<0.0001,<0.0001)	1.99E-04	1.15E-04	<0.0001(<0.0001,<0.0001)	9.28E-05	3.04E-05	<0.0001(<0.0001,<0.0001)	1.9E-05	1E-05	0.38992	-0.86		0.5536	0.3899	0.3899
Genea	<0.0001(<0.0001,<0.0001)	2.28E-04	9.22E-05	<0.0001(<0.0001,<0.0001)	2.39E-05	1.16E-05	<0.0001(<0.0001,<0.0001)	0	0	1.29E-03	-3.22	**	0.0098	0.0013	** 0.0013
Septoria	<0.0001(<0.0001,<0.0001)	2.27E-04	2.01E-04	<0.0001(<0.0001,<0.0001)	2.03E-05	1.71E-05	<0.0001(<0.0001,<0.0001)	0	0	0.60641	-0.52		0.7291	0.6064	0.6064
Bulleromyces	<0.0001(<0.0001,<0.0001)	3.70E-05	3.47E-05	<0.0001(<0.0001,<0.0001)	4.62E-04	3.55E-04	<0.0001(<0.0001,<0.0001)	0	0	0.06341	-1.86		0.1701	0.0634	0.0634
Toxicocladosporium	<0.0001(<0.0001,<0.0001)	2.32E-04	1.89E-04	<0.0001(<0.0001,<0.0001)	0.00E+00	0.00E+00	<0.0001(<0.0001,<0.0001)	0	0	0.04464	-2.01	*	0.1306	0.0446	* 0.0446
Stachybotrys	<0.0001(<0.0001,<0.0001)	0.00E+00	#######	<0.0001(<0.0001,<0.0001)	8.32E-06	5.13E-06	<0.0001(<0.0001,<0.0001)	0.00194	0.00187	8.9E-07	-4.91	***	4E-05	<0.0001	***<0.0001
Macrohyporia	<0.0001(<0.0001,<0.0001)	2.17E-04	2.10E-04	<0.0001(<0.0001,<0.0001)	0	0	<0.0001(<0.0001,<0.0001)	0	0	0.02444	-2.25	*	0.0854	0.0244	* 0.0244
Carlosrosaea	<0.0001(<0.0001,<0.0001)	2.14E-04	2.10E-04	<0.0001(<0.0001,<0.0001)	0	0	<0.0001(<0.0001,<0.0001)	0	0	0.10779	-1.61		0.236	0.1078	0.1078
Neoconiothyrium	<0.0001(<0.0001,<0.0001)	2.08E-04	2.04E-04	<0.0001(<0.0001,<0.0001)	0	0	<0.0001(<0.0001,<0.0001)	0	0	0.06006	-1.88		0.1649	0.0601	0.0601
Sistotrema	<0.0001(<0.0001,<0.0001)	1.93E-06	1.93E-06	<0.0001(<0.0001,<0.0001)	0.000481	0.000455	<0.0001(<0.0001,<0.0001)	1.2E-05	1.2E-05	0.00168	-3.14	**	0.0118	0.0017	** 0.0017
Stemphylium	<0.0001(<0.0001,<0.0001)	0.000197	0.00012	<0.0001(<0.0001,<0.0001)	0.00E+00	0.00E+00	<0.0001(<0.0001,<0.0001)	0	0	0.00706	-2.69	**	0.0336	0.0071	** 0.0071
Peroneutypa	<0.0001(<0.0001,<0.0001)	2.50E-07	2.50E-07	<0.0001(<0.0001,<0.0001)	4.59E-04	4.58E-04	<0.0001(<0.0001,<0.0001)	0	0	0.30009	-1.04		0.454	0.3001	0.3001
Parastagonosporella	<0.0001(<0.0001,<0.0001)	0.000194	0.00018	<0.0001(<0.0001,<0.0001)	0.00E+00	0.00E+00	<0.0001(<0.0001,<0.0001)	0	0	0.018	-2.37	*	0.0693	0.018	* 0.018
Helicoma	<0.0001(<0.0001,<0.0001)	0.000193	0.00019	<0.0001(<0.0001,<0.0001)	0.00E+00	0.00E+00	<0.0001(<0.0001,<0.0001)	0	0	0.14379	-1.46		0.2885	0.1438	0.1438
Golovinomyces	<0.0001(<0.0001,<0.0001)	4.57E-05	4.52E-05	<0.0001(<0.0001,<0.0001)	0.000323	0.000321	<0.0001(<0.0001,<0.0001)	2.6E-05	1.4E-05	0.00182	-3.12	**	0.012	0.0018	** 0.0018
Hypoxylon	<0.0001(<0.0001,<0.0001)	1.85E-04	1.73E-04	<0.0001(<0.0001,<0.0001)	0.00E+00	0.00E+00	<0.0001(<0.0001,<0.0001)	0	0	0.01322	-2.48	*	0.0539	0.0132	* 0.0132
Chaetomium	<0.0001(<0.0001,<0.0001)	1.80E-04	1.77E-04	<0.0001(<0.0001,<0.0001)	1.04E-05	1.04E-05	<0.0001(<0.0001,<0.0001)	0	0	0.38796	-0.86		0.5527	0.388	0.388
Neodevriesia	<0.0001(<0.0001,<0.0001)	7.80E-07	4.54E-07	<0.0001(<0.0001,<0.0001)	0.00043	0.000305	<0.0001(<0.0001,<0.0001)	8.3E-06	8.3E-06	0.10343	-1.63		0.2351	0.1034	0.1034
Halosphaeriaceae_gen_Incertae_sedis	<0.0001(<0.0001,<0.0001)	0.000181	0.00011	<0.0001(<0.0001,<0.0001)	0.00E+00	0.00E+00	<0.0001(<0.0001,<0.0001)	0	0	0.00968	-2.59	**	0.0424	0.0097	** 0.0097
Valsaria	<0.0001(<0.0001,<0.0001)	2.66E-05	2.54E-05	<0.0001(<0.0001,<0.0001)	3.58E-04	2.51E-04	<0.0001(<0.0001,<0.0001)	0	0	0.52684	-0.63		0.6828	0.5268	0.5268

Erythrobasidium	<0.0001(<0.0001,<0.0001)	4.09E-05	3.88E-05	<0.0001(<0.0001,<0.0001)	0.000317	0.000188	<0.0001(<0.0001,<0.0001)	0	0	0.27315	-1.1		0.4256	0.2731	0.2731
Rachicladosporium	<0.0001(<0.0001,<0.0001)	1.60E-04	1.56E-04	<0.0001(<0.0001,<0.0001)	0	0	<0.0001(<0.0001,<0.0001)	0	0	0.10779	-1.61		0.236	0.1078	0.1078
Basidiomycota_gen_Incertae_sedis	<0.0001(<0.0001,<0.0001)	1.57E-04	1.03E-04	<0.0001(<0.0001,<0.0001)	2.75E-06	2.75E-06	<0.0001(<0.0001,<0.0001)	8.9E-06	8.9E-06	0.03826	-2.07	*	0.1224	0.0383	* 0.0383
Laetisaria	<0.0001(<0.0001,<0.0001)	1.57E-04	1.55E-04	<0.0001(<0.0001,<0.0001)	0	0	<0.0001(<0.0001,<0.0001)	0	0	0.2537	-1.14		0.4209	0.2537	0.2537
Tulostoma	<0.0001(<0.0001,<0.0001)	0.000157	0.00015	<0.0001(<0.0001,<0.0001)	0.00E+00	0.00E+00	<0.0001(<0.0001,<0.0001)	0	0	0.08057	-1.75		0.1938	0.0806	0.0806
Thermomyces	<0.0001(<0.0001,<0.0001)	1.18E-04	1.16E-04	<0.0001(<0.0001,<0.0001)	4.41E-05	4.41E-05	<0.0001(<0.0001,<0.0001)	0.00016	0.00016	0.52297	-0.64		0.6799	0.523	0.523
Monoblepharis	<0.0001(<0.0001,<0.0001)	0.00E+00	#######	<0.0001(<0.0001,<0.0001)	0.000351	0.000345	<0.0001(<0.0001,<0.0001)	8.9E-06	8.9E-06	0.00297	-2.97	**	0.0172	0.003	** 0.003
Wickerhamiella	<0.0001(<0.0001,<0.0001)	2.32E-05	2.00E-05	<0.0001(<0.0001,<0.0001)	8.56E-05	6.08E-05	<0.0001(<0.0001,<0.0001)	0.00079	0.00078	0.03951	-2.06	*	0.1243	0.0395	* 0.0395
Phlebiopsis	<0.0001(<0.0001,<0.0001)	0.000149	0.0001	<0.0001(<0.0001,<0.0001)	0.00E+00	0.00E+00	<0.0001(<0.0001,<0.0001)	0	0	0.02444	-2.25	*	0.0854	0.0244	* 0.0244
Neodevriesiaceae_gen_Incertae_sedis	<0.0001(<0.0001,<0.0001)	1.48E-04	1.46E-04	<0.0001(<0.0001,<0.0001)	2.39E-06	1.72E-06	<0.0001(<0.0001,<0.0001)	0	0	0.71681	-0.36		0.7869	0.7168	0.7168
Nigrograna	<0.0001(<0.0001,<0.0001)	1.45E-04	1.11E-04	<0.0001(<0.0001,<0.0001)	0	0	<0.0001(<0.0001,<0.0001)	0	0	0.04464	-2.01	*	0.1306	0.0446	* 0.0446
Coniosporium	<0.0001(<0.0001,<0.0001)	1.79E-05	1.72E-05	<0.0001(<0.0001,<0.0001)	1.49E-05	1.26E-05	<0.0001(<0.0001,<0.0001)	0.00105	0.00105	0.43169	-0.79		0.581	0.4317	0.4317
Ceriporiopsis	<0.0001(<0.0001,<0.0001)	1.38E-04	9.57E-05	<0.0001(<0.0001,<0.0001)	0	0	<0.0001(<0.0001,<0.0001)	0	0	0.10779	-1.61		0.236	0.1078	0.1078
Cytospora	<0.0001(<0.0001,<0.0001)	1.37E-04	9.50E-05	<0.0001(<0.0001,<0.0001)	0	0	<0.0001(<0.0001,<0.0001)	0	0	0.06006	-1.88		0.1649	0.0601	0.0601
Xylocoremium	<0.0001(<0.0001,<0.0001)	1.34E-04	1.31E-04	<0.0001(<0.0001,<0.0001)	0	0	<0.0001(<0.0001,<0.0001)	0	0	0.08058	-1.75		0.1938	0.0806	0.0806
Novakomyces	<0.0001(<0.0001,<0.0001)	1.26E-04	1.02E-04	<0.0001(<0.0001,<0.0001)	1.48E-05	1.06E-05	<0.0001(<0.0001,<0.0001)	0	0	0.64159	-0.47		0.7648	0.6416	0.6416
Torula	<0.0001(<0.0001,<0.0001)	3.79E-07	3.79E-07	<0.0001(<0.0001,<0.0001)	0.000308	0.000306	<0.0001(<0.0001,<0.0001)	0	0	0.30009	-1.04		0.454	0.3001	0.3001
Sympodiomycopsis	<0.0001(<0.0001,<0.0001)	1.30E-04	1.26E-04	<0.0001(<0.0001,<0.0001)	0.00E+00	0.00E+00	<0.0001(<0.0001,<0.0001)	0	0	0.19126	-1.31		0.365	0.1913	0.1913
Deconica	<0.0001(<0.0001,<0.0001)	1.25E-04	1.22E-04	<0.0001(<0.0001,<0.0001)	1.23E-06	1.23E-06	<0.0001(<0.0001,<0.0001)	0	0	0.29911	-1.04		0.454	0.2991	0.2991
Exobasidium	<0.0001(<0.0001,<0.0001)	0.00E+00	#######	<0.0001(<0.0001,<0.0001)	0.000147	0.000145	<0.0001(<0.0001,<0.0001)	0.00054	0.00054	0.02157	-2.3	*	0.0787	0.0216	* 0.0216
Beauveria	<0.0001(<0.0001,<0.0001)	7.38E-05	2.02E-05	<0.0001(<0.0001,<0.0001)	7.23E-05	2.26E-05	<0.0001(<0.0001,<0.0001)	0.00016	0.00013	0.81393	-0.24		0.834	0.8139	0.8139
Venturiaceae_gen_Incertae_sedis	<0.0001(<0.0001,<0.0001)	0.00E+00	#######	<0.0001(<0.0001,<0.0001)	0.000286	0.000281	<0.0001(<0.0001,<0.0001)	0	0	0.00124	-3.23	**	0.0098	0.0012	** 0.0012
Phaeophleospora	<0.0001(<0.0001,<0.0001)	1.21E-04	1.08E-04	<0.0001(<0.0001,<0.0001)	0	0	<0.0001(<0.0001,<0.0001)	0	0	0.10779	-1.61		0.236	0.1078	0.1078
Bergerella	<0.0001(<0.0001,<0.0001)	1.20E-04	1.16E-04	<0.0001(<0.0001,<0.0001)	0.00E+00	0.00E+00	<0.0001(<0.0001,<0.0001)	0	0	0.06006	-1.88		0.1649	0.0601	0.0601
Tubeufia	<0.0001(<0.0001,<0.0001)	1.18E-04	1.15E-04	<0.0001(<0.0001,<0.0001)	0	0	<0.0001(<0.0001,<0.0001)	0	0	0.14379	-1.46		0.2885	0.1438	0.1438
Didymocyrtis	<0.0001(<0.0001,<0.0001)	6.67E-07	6.7E-07	<0.0001(<0.0001,<0.0001)	8.80E-06	3.71E-06	<0.0001(<0.0001,<0.0001)	0.00098	0.00096	0.00027	-3.64	***	0.0033	0.0003	***0.0003
Zygosaccharomyces	<0.0001(<0.0001,<0.0001)	1.16E-04	1.13E-04	<0.0001(<0.0001,<0.0001)	0	0	<0.0001(<0.0001,<0.0001)	0	0	0.2537	-1.14		0.4209	0.2537	0.2537
Sporothrix	<0.0001(<0.0001,<0.0001)	2.80E-07	2.80E-07	<0.0001(<0.0001,<0.0001)	0.000196	8.24E-05	<0.0001(<0.0001,<0.0001)	0.00027	0.00015	5E-06	-4.56	***	0.0001	<0.0001	***<0.0001

Preussia	<0.0001(<0.0001,<0.0001)	0.000114	0.00011	<0.0001(<0.0001,<0.0001)	0.00E+00	0.00E+00	<0.0001(<0.0001,<0.0001)	0	0	0.02444	-2.25	*	0.0854	0.0244	* 0.0244
GS08_gen_Incertae_sedis	<0.0001(<0.0001,<0.0001)	0.00E+00	#######	<0.0001(<0.0001,<0.0001)	2.67E-04	2.64E-04	<0.0001(<0.0001,<0.0001)	0	0	0.01875	-2.35	*	0.0716	0.0187	* 0.0187
Gymnopus	<0.0001(<0.0001,<0.0001)	1.12E-04	1.05E-04	<0.0001(<0.0001,<0.0001)	0	0	<0.0001(<0.0001,<0.0001)	0	0	0.58243	-0.55		0.7105	0.5824	0.5824
Sordaria	<0.0001(<0.0001,<0.0001)	1.11E-04	9.85E-05	<0.0001(<0.0001,<0.0001)	0	0	<0.0001(<0.0001,<0.0001)	0	0	0.14379	-1.46		0.2885	0.1438	0.1438
Calycina	<0.0001(<0.0001,<0.0001)	0.000111	0.00011	<0.0001(<0.0001,<0.0001)	0.00E+00	0.00E+00	<0.0001(<0.0001,<0.0001)	0	0	0.10779	-1.61		0.236	0.1078	0.1078
Cryptomarasmius	<0.0001(<0.0001,<0.0001)	1.11E-04	7.42E-05	<0.0001(<0.0001,<0.0001)	0	0	<0.0001(<0.0001,<0.0001)	0	0	0.03308	-2.13	*	0.1128	0.0331	* 0.0331
Derxomyces	<0.0001(<0.0001,<0.0001)	1.10E-04	1.07E-04	<0.0001(<0.0001,<0.0001)	0.00E+00	0.00E+00	<0.0001(<0.0001,<0.0001)	0	0	0.10779	-1.61		0.236	0.1078	0.1078
Radulomyces	<0.0001(<0.0001,<0.0001)	1.08E-04	1.05E-04	<0.0001(<0.0001,<0.0001)	0.00E+00	0.00E+00	<0.0001(<0.0001,<0.0001)	0	0	0.14378	-1.46		0.2885	0.1438	0.1438
Pyrenochaeta	<0.0001(<0.0001,<0.0001)	1.07E-04	1.06E-04	<0.0001(<0.0001,<0.0001)	0	0	<0.0001(<0.0001,<0.0001)	0	0	0.3356	-0.96		0.4859	0.3356	0.3356
Dactylodendron	<0.0001(<0.0001,<0.0001)	4.83E-05	4.53E-05	<0.0001(<0.0001,<0.0001)	0.000126	0.00012	<0.0001(<0.0001,<0.0001)	4.5E-05	4.5E-05	0.43936	-0.77		0.581	0.4394	0.4394
Issatchenkia	<0.0001(<0.0001,<0.0001)	1.08E-06	6.57E-07	<0.0001(<0.0001,<0.0001)	0.000244	0.000222	<0.0001(<0.0001,<0.0001)	1.6E-05	1.1E-05	0.00905	-2.61	**	0.0405	0.0091	** 0.0091
Podosphaera	<0.0001(<0.0001,<0.0001)	7.51E-05	7.4E-05	<0.0001(<0.0001,<0.0001)	6.97E-05	6.69E-05	<0.0001(<0.0001,<0.0001)	0	0	0.41116	-0.82		0.574	0.4112	0.4112
Phyllosticta	<0.0001(<0.0001,<0.0001)	0.000104	9.9E-05	<0.0001(<0.0001,<0.0001)	0	0	<0.0001(<0.0001,<0.0001)	0	0	0.14379	-1.46		0.2885	0.1438	0.1438
Sarocladium	<0.0001(<0.0001,<0.0001)	3.45E-05	2.2E-05	<0.0001(<0.0001,<0.0001)	0.000146	4.72E-05	<0.0001(<0.0001,<0.0001)	5.8E-05	3.2E-05	0.00034	-3.59	***	0.0038	0.0003	***0.0003
Gonatophragmium	<0.0001(<0.0001,<0.0001)	0.000103	9.5E-05	<0.0001(<0.0001,<0.0001)	0	0	<0.0001(<0.0001,<0.0001)	0	0	0.2537	-1.14		0.4209	0.2537	0.2537
Zasmidium	<0.0001(<0.0001,<0.0001)	0.000103	9.7E-05	<0.0001(<0.0001,<0.0001)	0	0	<0.0001(<0.0001,<0.0001)	0	0	0.19126	-1.31		0.365	0.1913	0.1913
Antennariella	<0.0001(<0.0001,<0.0001)	0.000103	0.0001	<0.0001(<0.0001,<0.0001)	0	0	<0.0001(<0.0001,<0.0001)	0	0	0.14379	-1.46		0.2885	0.1438	0.1438
Phanerochaete	<0.0001(<0.0001,<0.0001)	4.47E-07	4.5E-07	<0.0001(<0.0001,<0.0001)	0.000241	0.000235	<0.0001(<0.0001,<0.0001)	0	0	0.00791	-2.66	**	0.0362	0.0079	** 0.0079
Meira	<0.0001(<0.0001,<0.0001)	1.12E-05	8.2E-06	<0.0001(<0.0001,<0.0001)	2.18E-06	2.18E-06	<0.0001(<0.0001,<0.0001)	0.0008	0.0008	0.38098	-0.88		0.5465	0.381	0.381
Ochrocladosporium	<0.0001(<0.0001,<0.0001)	1.74E-06	9.1E-07	<0.0001(<0.0001,<0.0001)	0	0	<0.0001(<0.0001,<0.0001)	0.00088	0.00088	0.25398	-1.14		0.4209	0.254	0.254
Rhizopus	<0.0001(<0.0001,<0.0001)	9.98E-05	6.7E-05	<0.0001(<0.0001,<0.0001)	2.93E-06	2.93E-06	<0.0001(<0.0001,<0.0001)	0	0	0.17951	-1.34		0.349	0.1795	0.1795
Pseudogymnoascus	<0.0001(<0.0001,<0.0001)	0.0001	7.2E-05	<0.0001(<0.0001,<0.0001)	1.13E-06	1.13E-06	<0.0001(<0.0001,<0.0001)	0	0	0.37758	-0.88		0.5435	0.3776	0.3776
Neoascochyta	<0.0001(<0.0001,<0.0001)	9.87E-05	9.5E-05	<0.0001(<0.0001,<0.0001)	0	0	<0.0001(<0.0001,<0.0001)	0	0	0.44271	-0.77		0.581	0.4427	0.4427
Chalara	<0.0001(<0.0001,<0.0001)	9.81E-05	9.7E-05	<0.0001(<0.0001,<0.0001)	0	0	<0.0001(<0.0001,<0.0001)	0	0	0.3356	-0.96		0.4859	0.3356	0.3356
Skeletocutis	<0.0001(<0.0001,<0.0001)	9.69E-05	9.6E-05	<0.0001(<0.0001,<0.0001)	0	0	<0.0001(<0.0001,<0.0001)	0	0	0.3356	-0.96		0.4859	0.3356	0.3356
Cladophialophora	<0.0001(<0.0001,<0.0001)	9.29E-05	9E-05	<0.0001(<0.0001,<0.0001)	0	0	<0.0001(<0.0001,<0.0001)	0	0	0.2537	-1.14		0.4209	0.2537	0.2537
Anisomeridium	<0.0001(<0.0001,<0.0001)	0	0	<0.0001(<0.0001,<0.0001)	0.000205	0.000204	<0.0001(<0.0001,<0.0001)	3.6E-05	3.6E-05	0.04061	-2.05	*	0.1243	0.0406	* 0.0406
Calyptella	<0.0001(<0.0001,<0.0001)	0.000089	8.5E-05	<0.0001(<0.0001,<0.0001)	0	0	<0.0001(<0.0001,<0.0001)	0	0	0.08058	-1.75		0.1938	0.0806	0.0806

Atractiella	<0.0001(<0.0001,<0.0001)	8.75E-05	6.2E-05	<0.0001(<0.0001,<0.0001)	0	0	<0.0001(<0.0001,<0.0001)	0	0	0.14379	-1.46		0.2885	0.1438	0.1438
Jaminaea	<0.0001(<0.0001,<0.0001)	8.52E-05	8.5E-05	<0.0001(<0.0001,<0.0001)	1.02E-06	1.02E-06	<0.0001(<0.0001,<0.0001)	0	0	0.87748	-0.15		0.8843	0.8775	0.8775
Phacidium	<0.0001(<0.0001,<0.0001)	0	0	<0.0001(<0.0001,<0.0001)	0.0002	0.000198	<0.0001(<0.0001,<0.0001)	5.1E-06	5.1E-06	0.04145	-2.04	*	0.125	0.0415	* 0.0415
Hormodochis	<0.0001(<0.0001,<0.0001)	8.36E-05	8E-05	<0.0001(<0.0001,<0.0001)	0	0	<0.0001(<0.0001,<0.0001)	0	0	0.44271	-0.77		0.581	0.4427	0.4427
Physisporinus	<0.0001(<0.0001,<0.0001)	8.25E-05	8.1E-05	<0.0001(<0.0001,<0.0001)	0	0	<0.0001(<0.0001,<0.0001)	0	0	0.44271	-0.77		0.581	0.4427	0.4427
Fuscosporella	<0.0001(<0.0001,<0.0001)	5.33E-05	1.3E-05	<0.0001(<0.0001,<0.0001)	2.18E-05	1.01E-05	<0.0001(<0.0001,0.0001)	0.00017	9.5E-05	0.08505	-1.72		0.2033	0.0851	0.0851
Holtermanniella	<0.0001(<0.0001,<0.0001)	1.41E-05	8E-06	<0.0001(<0.0001,<0.0001)	0.000152	0.000142	<0.0001(<0.0001,<0.0001)	0	0	0.16188	-1.4		0.3177	0.1619	0.1619
Itersonilia	<0.0001(<0.0001,<0.0001)	2.15E-06	1.2E-06	<0.0001(<0.0001,<0.0001)	0.000169	0.000165	<0.0001(<0.0001,<0.0001)	1.7E-05	1.2E-05	0.13303	-1.5		0.2809	0.133	0.133
Neofusicoccum	<0.0001(<0.0001,<0.0001)	7.53E-05	7.2E-05	<0.0001(<0.0001,<0.0001)	9.29E-07	9.29E-07	<0.0001(<0.0001,<0.0001)	0	0	0.29618	-1.04		0.454	0.2962	0.2962
Banksiophoma	<0.0001(<0.0001,<0.0001)	7.47E-05	7.3E-05	<0.0001(<0.0001,<0.0001)	0	0	<0.0001(<0.0001,<0.0001)	4.4E-06	4.4E-06	0.2541	-1.14		0.4209	0.2541	0.2541
Rhopalophora	<0.0001(<0.0001,<0.0001)	7.29E-05	7.1E-05	<0.0001(<0.0001,<0.0001)	0	0	<0.0001(<0.0001,<0.0001)	0	0	0.19126	-1.31		0.365	0.1913	0.1913
Trechispora	<0.0001(<0.0001,<0.0001)	7.21E-05	6.7E-05	<0.0001(<0.0001,<0.0001)	0	0	<0.0001(<0.0001,<0.0001)	0	0	0.3356	-0.96		0.4859	0.3356	0.3356
Elsinoe	<0.0001(<0.0001,<0.0001)	7.19E-05	7.2E-05	<0.0001(<0.0001,<0.0001)	0	0	<0.0001(<0.0001,<0.0001)	0	0	0.76419	-0.3		0.7869	0.7642	0.7642
Parasarocladium	<0.0001(<0.0001,<0.0001)	6.95E-05	6.9E-05	<0.0001(<0.0001,<0.0001)	0	0	<0.0001(<0.0001,<0.0001)	0	0	0.58243	-0.55		0.7105	0.5824	0.5824
Mucor	<0.0001(<0.0001,<0.0001)	6.52E-05	1.5E-05	<0.0001(<0.0001,<0.0001)	6.46E-06	5.6E-06	<0.0001(<0.0001,<0.0001)	8.3E-06	8.3E-06	0.00262	-3.01	**	0.0155	0.0026	** 0.0026
Trichomeriaceae_gen_Incertae_sedis	<0.0001(<0.0001,<0.0001)	0	0	<0.0001(<0.0001,<0.0001)	0.000161	0.000158	<0.0001(<0.0001,<0.0001)	0	0	0.00124	-3.23	**	0.0098	0.0012	** 0.0012
Fusicolla	<0.0001(<0.0001,<0.0001)	6.81E-05	6.8E-05	<0.0001(<0.0001,<0.0001)	0	0	<0.0001(<0.0001,<0.0001)	0	0	0.58243	-0.55		0.7105	0.5824	0.5824
Subulicystidium	<0.0001(<0.0001,<0.0001)	7.37E-06	6.1E-06	<0.0001(<0.0001,<0.0001)	0.000139	0.000139	<0.0001(<0.0001,<0.0001)	0	0	0.83326	-0.21		0.8496	0.8333	0.8333
Bandonia	<0.0001(<0.0001,<0.0001)	3.84E-05	2E-05	<0.0001(<0.0001,<0.0001)	5.01E-05	3.65E-05	<0.0001(<0.0001,<0.0001)	4.4E-05	4.1E-05	0.18498	-1.33		0.3579	0.185	0.185
Dimorphoma	<0.0001(<0.0001,<0.0001)	4.93E-05	4E-05	<0.0001(<0.0001,<0.0001)	3.53E-05	3.53E-05	<0.0001(<0.0001,<0.0001)	0	0	0.72286	-0.35		0.7869	0.7229	0.7229
Dactylella	<0.0001(<0.0001,<0.0001)	6.37E-05	6.3E-05	<0.0001(<0.0001,<0.0001)	0	0	<0.0001(<0.0001,<0.0001)	0	0	0.58243	-0.55		0.7105	0.5824	0.5824
Agaricales_gen_Incertae_sedis	<0.0001(<0.0001,<0.0001)	0	0	<0.0001(<0.0001,<0.0001)	0.000148	0.000148	<0.0001(<0.0001,<0.0001)	6.7E-06	6.7E-06	0.03757	-2.08	*	0.1212	0.0376	* 0.0376
Gibellula	<0.0001(<0.0001,<0.0001)	5.08E-05	2.1E-05	<0.0001(<0.0001,<0.0001)	2.44E-05	1.01E-05	<0.0001(<0.0001,<0.0001)	7.1E-06	7.1E-06	0.59625	-0.53		0.7253	0.5962	0.5962
Eutypella	<0.0001(<0.0001,<0.0001)	6.06E-05	6E-05	<0.0001(<0.0001,<0.0001)	0	0	<0.0001(<0.0001,<0.0001)	0	0	0.44271	-0.77		0.581	0.4427	0.4427
Lophiostoma	<0.0001(<0.0001,<0.0001)	5.89E-05	4.1E-05	<0.0001(<0.0001,<0.0001)	0	0	<0.0001(<0.0001,<0.0001)	7.1E-06	7.1E-06	0.23128	-1.2		0.4209	0.2313	0.2313
Paratritirachium	<0.0001(<0.0001,<0.0001)	4.17E-07	4.2E-07	<0.0001(<0.0001,<0.0001)	0.000137	9.97E-05	<0.0001(<0.0001,<0.0001)	0	0	0.30009	-1.04		0.454	0.3001	0.3001
Umthunziomyces	<0.0001(<0.0001,<0.0001)	3.22E-05	3.1E-05	<0.0001(<0.0001,<0.0001)	5.51E-05	5.42E-05	<0.0001(<0.0001,<0.0001)	8.1E-06	8.1E-06	0.60355	-0.52		0.7291	0.6035	0.6035

Nectriopsis	<0.0001(<0.0001,<0.0001)	5.56E-05	5.3E-05	<0.0001(<0.0001,<0.0001)	0	0	<0.0001(<0.0001,<0.0001)	0	0	0.2537	-1.14		0.4209	0.2537	0.2537
Lyomyces	<0.0001(<0.0001,<0.0001)	5.55E-05	5.1E-05	<0.0001(<0.0001,<0.0001)	0	0	<0.0001(<0.0001,<0.0001)	0	0	0.3356	-0.96		0.4859	0.3356	0.3356
Hanseniaspora	<0.0001(<0.0001,<0.0001)	3.02E-05	2.7E-05	<0.0001(<0.0001,<0.0001)	4.78E-05	4.78E-05	<0.0001(<0.0001,<0.0001)	4.4E-05	3.1E-05	0.17926	-1.34		0.349	0.1793	0.1793
Ciboria	<0.0001(<0.0001,<0.0001)	5.3E-05	5.3E-05	<0.0001(<0.0001,<0.0001)	0	0	<0.0001(<0.0001,<0.0001)	0	0	0.44271	-0.77		0.581	0.4427	0.4427
Xeromyces	<0.0001(<0.0001,<0.0001)	6.99E-06	7E-06	<0.0001(<0.0001,<0.0001)	0.000108	0.000104	<0.0001(<0.0001,<0.0001)	0	0	0.101	-1.64		0.2309	0.101	0.101
Coniothyrium	<0.0001(<0.0001,<0.0001)	0	0	<0.0001(<0.0001,<0.0001)	1.34E-06	1.34E-06	<0.0001(<0.0001,<0.0001)	0.00043	0.00043	0.00025	-3.66	***	0.0032	0.0003	***0.0003
Biatriosporaceae_gen_Incertae_sedis	<0.0001(<0.0001,<0.0001)	4.95E-05	4.9E-05	<0.0001(<0.0001,<0.0001)	0	0	<0.0001(<0.0001,<0.0001)	0	0	0.44271	-0.77		0.581	0.4427	0.4427
Vacuiphoma	<0.0001(<0.0001,<0.0001)	2.21E-05	1.6E-05	<0.0001(<0.0001,<0.0001)	3.37E-05	2.55E-05	<0.0001(<0.0001,<0.0001)	8.8E-05	7E-05	0.0975	-1.66		0.2266	0.0975	0.0975
Laburnicola	<0.0001(<0.0001,<0.0001)	4.59E-05	4.4E-05	<0.0001(<0.0001,<0.0001)	0	0	<0.0001(<0.0001,<0.0001)	0	0	0.2537	-1.14		0.4209	0.2537	0.2537
Heliocybe	<0.0001(<0.0001,<0.0001)	4.57E-05	4.5E-05	<0.0001(<0.0001,<0.0001)	0	0	<0.0001(<0.0001,<0.0001)	0	0	0.2537	-1.14		0.4209	0.2537	0.2537
Lipomyces	<0.0001(<0.0001,<0.0001)	4.55E-05	3.8E-05	<0.0001(<0.0001,<0.0001)	0	0	<0.0001(<0.0001,<0.0001)	0	0	0.2537	-1.14		0.4209	0.2537	0.2537
Steccherinum	<0.0001(<0.0001,<0.0001)	4.41E-05	4.4E-05	<0.0001(<0.0001,<0.0001)	0	0	<0.0001(<0.0001,<0.0001)	0	0	0.58243	-0.55		0.7105	0.5824	0.5824
Colletotrichum	<0.0001(<0.0001,<0.0001)	4.37E-05	3.1E-05	<0.0001(<0.0001,<0.0001)	0	0	<0.0001(<0.0001,<0.0001)	0	0	0.3356	-0.96		0.4859	0.3356	0.3356
Striatibotrys	<0.0001(<0.0001,<0.0001)	3.1E-05	1.5E-05	<0.0001(<0.0001,<0.0001)	2.25E-05	6.68E-06	<0.0001(<0.0001,<0.0001)	2.4E-05	1.9E-05	0.33055	-0.97		0.4859	0.3305	0.3305
Huntiella	<0.0001(<0.0001,<0.0001)	4.3E-05	4.3E-05	<0.0001(<0.0001,<0.0001)	0	0	<0.0001(<0.0001,<0.0001)	0	0	0.76419	-0.3		0.7869	0.7642	0.7642
Fomitopsis	<0.0001(<0.0001,<0.0001)	4.18E-05	4.1E-05	<0.0001(<0.0001,<0.0001)	0	0	<0.0001(<0.0001,<0.0001)	0	0	0.44271	-0.77		0.581	0.4427	0.4427
Vandijckella	<0.0001(<0.0001,<0.0001)	5.38E-07	5.4E-07	<0.0001(<0.0001,<0.0001)	9.42E-05	9.42E-05	<0.0001(<0.0001,<0.0001)	0	0	0.74278	-0.33		0.7869	0.7428	0.7428
Kneiffiella	<0.0001(<0.0001,<0.0001)	3.97E-05	3.9E-05	<0.0001(<0.0001,<0.0001)	0	0	<0.0001(<0.0001,<0.0001)	0	0	0.58243	-0.55		0.7105	0.5824	0.5824
Ceratocystis	<0.0001(<0.0001,<0.0001)	3.45E-05	3.4E-05	<0.0001(<0.0001,<0.0001)	1.05E-06	1.05E-06	<0.0001(<0.0001,<0.0001)	3.9E-05	3.9E-05	0.60095	-0.52		0.7289	0.601	0.601
Cyphellaceae_gen_Incertae_sedis	<0.0001(<0.0001,<0.0001)	3.92E-05	3.8E-05	<0.0001(<0.0001,<0.0001)	0	0	<0.0001(<0.0001,<0.0001)	0	0	0.3356	-0.96		0.4859	0.3356	0.3356
Ruhlandiella	<0.0001(<0.0001,<0.0001)	3.92E-05	3.8E-05	<0.0001(<0.0001,<0.0001)	0	0	<0.0001(<0.0001,<0.0001)	0	0	0.58243	-0.55		0.7105	0.5824	0.5824
Moesziomyces	<0.0001(<0.0001,<0.0001)	1.73E-06	1.7E-06	<0.0001(<0.0001,<0.0001)	8.79E-05	5.45E-05	<0.0001(<0.0001,<0.0001)	0	0	0.00813	-2.65	**	0.0368	0.0081	** 0.0081
Peyronellaea	<0.0001(<0.0001,<0.0001)	9.7E-07	9.7E-07	<0.0001(<0.0001,<0.0001)	5.46E-05	3.4E-05	<0.0001(<0.0001,<0.0001)	0.00013	8.8E-05	0.00047	-3.5	***	0.005	0.0005	***0.0005
Anthracina	<0.0001(<0.0001,<0.0001)	1.77E-05	1.7E-05	<0.0001(<0.0001,<0.0001)	4.68E-05	4.57E-05	<0.0001(<0.0001,<0.0001)	0	0	0.55425	-0.59		0.7105	0.5543	0.5543
Zoophthora	<0.0001(<0.0001,<0.0001)	3.25E-05	2.1E-05	<0.0001(<0.0001,<0.0001)	1.18E-05	6.52E-06	<0.0001(<0.0001,<0.0001)	0	0	0.32876	-0.98		0.4859	0.3288	0.3288
Ganoderma	<0.0001(<0.0001,<0.0001)	3.69E-05	3.5E-05	<0.0001(<0.0001,<0.0001)	9.46E-07	9.46E-07	<0.0001(<0.0001,<0.0001)	0	0	0.82925	-0.22		0.8476	0.8293	0.8293
Amphobotrys	<0.0001(<0.0001,<0.0001)	3.5E-05	2.6E-05	<0.0001(<0.0001,<0.0001)	1.52E-06	1.08E-06	<0.0001(<0.0001,<0.0001)	1.3E-05	1.3E-05	0.8128	-0.24		0.834	0.8128	0.8128

Crustoderma	<0.0001(<0.0001,<0.0001)	3.58E-05	3.6E-05	<0.0001(<0.0001,<0.0001)	0	0	<0.0001(<0.0001,<0.0001)	0	0	0.76419	-0.3		0.7869	0.7642	0.7642
Penicillifer	<0.0001(<0.0001,<0.0001)	1.72E-05	1.3E-05	<0.0001(<0.0001,<0.0001)	3.84E-05	1.19E-05	<0.0001(<0.0001,<0.0001)	2.1E-05	1.5E-05	0.00311	-2.96	**	0.0173	0.0031	** 0.0031
Pseudopithomyces	<0.0001(<0.0001,<0.0001)	0	0	<0.0001(<0.0001,<0.0001)	8.39E-07	8.39E-07	<0.0001(<0.0001,<0.0001)	0.0003	0.00028	2E-09	-5.99	***	2E-07	<0.0001	***<0.0001
Akanthomyces	<0.0001(<0.0001,<0.0001)	3.29E-05	2E-05	<0.0001(<0.0001,<0.0001)	0	0	<0.0001(<0.0001,<0.0001)	0	0	0.06006	-1.88		0.1649	0.0601	0.0601
Anastomitrabeculia	<0.0001(<0.0001,<0.0001)	0	0	<0.0001(<0.0001,<0.0001)	0	0	<0.0001(<0.0001,<0.0001)	0.00029	0.00029	0.0019	-3.11	**	0.012	0.0019	** 0.0019
Eremotheciu m	<0.0001(<0.0001,<0.0001)	0	0	<0.0001(<0.0001,<0.0001)	7.72E-05	7.61E-05	<0.0001(<0.0001,<0.0001)	0	0	0.0715	-1.8		0.1781	0.0715	0.0715
Thanatephorus	<0.0001(<0.0001,<0.0001)	3.25E-05	3.1E-05	<0.0001(<0.0001,<0.0001)	0	0	<0.0001(<0.0001,<0.0001)	0	0	0.44271	-0.77		0.581	0.4427	0.4427
Paecilomyces	<0.0001(<0.0001,<0.0001)	0	0	<0.0001(<0.0001,<0.0001)	7.58E-05	7.58E-05	<0.0001(<0.0001,<0.0001)	0	0	0.26915	-1.11		0.4209	0.2691	0.2691
Quambalaria	<0.0001(<0.0001,<0.0001)	3.19E-05	3.2E-05	<0.0001(<0.0001,<0.0001)	0	0	<0.0001(<0.0001,<0.0001)	0	0	0.76419	-0.3		0.7869	0.7642	0.7642
Meyerozyma	<0.0001(<0.0001,<0.0001)	2.51E-05	2.4E-05	<0.0001(<0.0001,<0.0001)	1.39E-06	1.39E-06	<0.0001(<0.0001,<0.0001)	5.4E-05	3.3E-05	0.00111	-3.26	**	0.0098	0.0011	** 0.0011
Nemania	<0.0001(<0.0001,<0.0001)	0	0	<0.0001(<0.0001,<0.0001)	7.46E-05	7.46E-05	<0.0001(<0.0001,<0.0001)	0	0	0.26915	-1.11		0.4209	0.2691	0.2691
Aquapteridospora	<0.0001(<0.0001,<0.0001)	1.63E-05	1.2E-05	<0.0001(<0.0001,<0.0001)	3.32E-05	2.56E-05	<0.0001(<0.0001,<0.0001)	0	0	0.41116	-0.82		0.574	0.4112	0.4112
Acrodontium	<0.0001(<0.0001,<0.0001)	0	0	<0.0001(<0.0001,<0.0001)	0	0	<0.0001(<0.0001,<0.0001)	0.00026	0.00026	0.0019	-3.11	**	0.012	0.0019	** 0.0019
Lembosiniella	<0.0001(<0.0001,<0.0001)	6.21E-07	6.2E-07	<0.0001(<0.0001,<0.0001)	6.33E-05	6.33E-05	<0.0001(<0.0001,<0.0001)	0	0	0.74278	-0.33		0.7869	0.7428	0.7428
Kodamaea	<0.0001(<0.0001,<0.0001)	0	0	<0.0001(<0.0001,<0.0001)	5.64E-06	4.13E-06	<0.0001(<0.0001,<0.0001)	0.00022	0.00022	0.03976	-2.06	*	0.1243	0.0398	* 0.0398
Trichosporon	<0.0001(<0.0001,<0.0001)	2.67E-05	2.2E-05	<0.0001(<0.0001,<0.0001)	0	0	<0.0001(<0.0001,<0.0001)	0	0	0.10779	-1.61		0.236	0.1078	0.1078
Neocucurbitaria	<0.0001(<0.0001,<0.0001)	0	0	<0.0001(<0.0001,<0.0001)	6.07E-05	5.99E-05	<0.0001(<0.0001,<0.0001)	7.4E-06	7.4E-06	0.04061	-2.05	*	0.1243	0.0406	* 0.0406
Elaphomyces	<0.0001(<0.0001,<0.0001)	2.01E-05	1.8E-05	<0.0001(<0.0001,<0.0001)	1.4E-05	7.97E-06	<0.0001(<0.0001,<0.0001)	0	0	0.20483	-1.27		0.3873	0.2048	0.2048
Entyloma	<0.0001(<0.0001,<0.0001)	2.44E-05	9.4E-06	<0.0001(<0.0001,<0.0001)	8.75E-07	8.75E-07	<0.0001(<0.0001,<0.0001)	0	0	0.07038	-1.81		0.1781	0.0704	0.0704
Penidiella	<0.0001(<0.0001,<0.0001)	2.4E-05	2.4E-05	<0.0001(<0.0001,<0.0001)	0	0	<0.0001(<0.0001,<0.0001)	0	0	0.76419	-0.3		0.7869	0.7642	0.7642
Homortomyces	<0.0001(<0.0001,<0.0001)	2.2E-05	2.2E-05	<0.0001(<0.0001,<0.0001)	0	0	<0.0001(<0.0001,<0.0001)	0	0	0.76419	-0.3		0.7869	0.7642	0.7642
Actinomucor	<0.0001(<0.0001,<0.0001)	2.04E-05	4.7E-06	<0.0001(<0.0001,<0.0001)	2.21E-06	2.21E-06	<0.0001(<0.0001,<0.0001)	3.9E-06	3.9E-06	0.00711	-2.69	**	0.0336	0.0071	** 0.0071
Plenodomus	<0.0001(<0.0001,<0.0001)	0	0	<0.0001(<0.0001,<0.0001)	5.11E-05	4.79E-05	<0.0001(<0.0001,<0.0001)	0	0	0.00124	-3.23	**	0.0098	0.0012	** 0.0012
Allocryptovalsa	<0.0001(<0.0001,<0.0001)	0	0	<0.0001(<0.0001,<0.0001)	4.84E-05	4.84E-05	<0.0001(<0.0001,<0.0001)	0	0	0.26915	-1.11		0.4209	0.2691	0.2691
Kluyveromyces	<0.0001(<0.0001,<0.0001)	0	0	<0.0001(<0.0001,<0.0001)	4.66E-05	4.54E-05	<0.0001(<0.0001,<0.0001)	0	0	0.0715	-1.8		0.1781	0.0715	0.0715
Heydenia	<0.0001(<0.0001,<0.0001)	1.97E-05	2E-05	<0.0001(<0.0001,<0.0001)	0	0	<0.0001(<0.0001,<0.0001)	0	0	0.76419	-0.3		0.7869	0.7642	0.7642
Lithohypha	<0.0001(<0.0001,<0.0001)	1.82E-05	1.5E-05	<0.0001(<0.0001,<0.0001)	0	0	<0.0001(<0.0001,<0.0001)	0	0	0.3356	-0.96		0.4859	0.3356	0.3356
Komagataella	<0.0001(<0.0001,<0.0001)	1.78E-05	1.4E-05	<0.0001(<0.0001,<0.0001)	0	0	<0.0001(<0.0001,<0.0001)	0	0	0.58243	-0.55		0.7105	0.5824	0.5824

Protomyces	<0.0001(<0.0001,<0.0001)	0	0	<0.0001(<0.0001,<0.0001)	4.07E-05	4.07E-05	<0.0001(<0.0001,<0.0001)	0	0	0.26915	-1.11		0.4209	0.2691	0.2691
Clonostachys	<0.0001(<0.0001,<0.0001)	3.83E-06	3.8E-06	<0.0001(<0.0001,<0.0001)	3.1E-05	2.22E-05	<0.0001(<0.0001,<0.0001)	0	0	0.101	-1.64		0.2309	0.101	0.101
Microascus	<0.0001(<0.0001,<0.0001)	1.68E-05	1.1E-05	<0.0001(<0.0001,<0.0001)	0	0	<0.0001(<0.0001,<0.0001)	0	0	0.44271	-0.77		0.581	0.4427	0.4427
Pseudohelminthosporium	<0.0001(<0.0001,<0.0001)	1.62E-05	1.6E-05	<0.0001(<0.0001,<0.0001)	0	0	<0.0001(<0.0001,<0.0001)	0	0	0.76419	-0.3		0.7869	0.7642	0.7642
Ogataea	<0.0001(<0.0001,<0.0001)	0	0	<0.0001(<0.0001,<0.0001)	3.68E-05	3.53E-05	<0.0001(<0.0001,<0.0001)	0	0	0.0715	-1.8		0.1781	0.0715	0.0715
Cyphellophora	<0.0001(<0.0001,<0.0001)	1.54E-05	1.3E-05	<0.0001(<0.0001,<0.0001)	0	0	<0.0001(<0.0001,<0.0001)	0	0	0.44271	-0.77		0.581	0.4427	0.4427
Puccinia	<0.0001(<0.0001,<0.0001)	1.16E-05	1.1E-05	<0.0001(<0.0001,<0.0001)	8.07E-06	8.07E-06	<0.0001(<0.0001,<0.0001)	0	0	0.87748	-0.15		0.8843	0.8775	0.8775
Umbilicaria	<0.0001(<0.0001,<0.0001)	0	0	<0.0001(<0.0001,<0.0001)	3.43E-05	3.43E-05	<0.0001(<0.0001,<0.0001)	0	0	0.26915	-1.11		0.4209	0.2691	0.2691
Buckleyzyma	<0.0001(<0.0001,<0.0001)	1.1E-06	1.1E-06	<0.0001(<0.0001,<0.0001)	3.17E-05	2.99E-05	<0.0001(<0.0001,<0.0001)	0	0	0.101	-1.64		0.2309	0.101	0.101
Lentithecium	<0.0001(<0.0001,<0.0001)	0	0	<0.0001(<0.0001,<0.0001)	0	0	<0.0001(<0.0001,<0.0001)	0.00012	0.00012	0.0019	-3.11	**	0.012	0.0019	** 0.0019
Ascosphaera	<0.0001(<0.0001,<0.0001)	1.32E-05	1.3E-05	<0.0001(<0.0001,<0.0001)	0	0	<0.0001(<0.0001,<0.0001)	0	0	0.58243	-0.55		0.7105	0.5824	0.5824
Tetrapisispora	<0.0001(<0.0001,<0.0001)	3.59E-06	2.4E-06	<0.0001(<0.0001,<0.0001)	1.73E-05	6.1E-06	<0.0001(<0.0001,<0.0001)	2E-05	1.4E-05	0.01144	-2.53	*	0.0496	0.0114	* 0.0114
Hyphopichia	<0.0001(<0.0001,<0.0001)	1.04E-05	1E-05	<0.0001(<0.0001,<0.0001)	5.66E-06	5.66E-06	<0.0001(<0.0001,<0.0001)	0	0	0.75049	-0.32		0.7869	0.7505	0.7505
Arthrinium	<0.0001(<0.0001,<0.0001)	9.71E-06	9.2E-06	<0.0001(<0.0001,<0.0001)	5.39E-06	5.39E-06	<0.0001(<0.0001,<0.0001)	0	0	0.87748	-0.15		0.8843	0.8775	0.8775
Kurtzmaniella	<0.0001(<0.0001,<0.0001)	0	0	<0.0001(<0.0001,<0.0001)	2.56E-05	2.56E-05	<0.0001(<0.0001,<0.0001)	9.3E-06	9.3E-06	0.03757	-2.08	*	0.1212	0.0376	* 0.0376
Uwebraunia	<0.0001(<0.0001,<0.0001)	0	0	<0.0001(<0.0001,<0.0001)	2.64E-05	2.64E-05	<0.0001(<0.0001,<0.0001)	0	0	0.26915	-1.11		0.4209	0.2691	0.2691
Curvularia	<0.0001(<0.0001,<0.0001)	9.06E-06	9.1E-06	<0.0001(<0.0001,<0.0001)	4.66E-06	4.66E-06	<0.0001(<0.0001,<0.0001)	0	0	0.75049	-0.32		0.7869	0.7505	0.7505
Collybiopsis	<0.0001(<0.0001,<0.0001)	1.1E-05	1.1E-05	<0.0001(<0.0001,<0.0001)	0	0	<0.0001(<0.0001,<0.0001)	0	0	0.76419	-0.3		0.7869	0.7642	0.7642
Coronicium	<0.0001(<0.0001,<0.0001)	1.1E-05	1E-05	<0.0001(<0.0001,<0.0001)	0	0	<0.0001(<0.0001,<0.0001)	0	0	0.58243	-0.55		0.7105	0.5824	0.5824
Strobilurus	<0.0001(<0.0001,<0.0001)	9.8E-06	9.8E-06	<0.0001(<0.0001,<0.0001)	0	0	<0.0001(<0.0001,<0.0001)	9.1E-06	9.1E-06	0.06381	-1.85		0.1701	0.0638	0.0638
Ceraceosorus	<0.0001(<0.0001,<0.0001)	0	0	<0.0001(<0.0001,<0.0001)	2.5E-05	2.5E-05	<0.0001(<0.0001,<0.0001)	0	0	0.26915	-1.11		0.4209	0.2691	0.2691
Hazslinszkyomyces	<0.0001(<0.0001,<0.0001)	8.36E-06	8.4E-06	<0.0001(<0.0001,<0.0001)	4.73E-06	4.73E-06	<0.0001(<0.0001,<0.0001)	0	0	0.75049	-0.32		0.7869	0.7505	0.7505
Flagelloscypha	<0.0001(<0.0001,<0.0001)	9.7E-06	9.7E-06	<0.0001(<0.0001,<0.0001)	0	0	<0.0001(<0.0001,<0.0001)	0	0	0.76419	-0.3		0.7869	0.7642	0.7642
Tritirachium	<0.0001(<0.0001,<0.0001)	9.51E-06	8E-06	<0.0001(<0.0001,<0.0001)	0	0	<0.0001(<0.0001,<0.0001)	0	0	0.44271	-0.77		0.581	0.4427	0.4427
Marantokordyana	<0.0001(<0.0001,<0.0001)	9.45E-06	9.4E-06	<0.0001(<0.0001,<0.0001)	0	0	<0.0001(<0.0001,<0.0001)	0	0	0.76419	-0.3		0.7869	0.7642	0.7642
Archaeorhizomyces	<0.0001(<0.0001,<0.0001)	9.35E-06	9.3E-06	<0.0001(<0.0001,<0.0001)	0	0	<0.0001(<0.0001,<0.0001)	0	0	0.76419	-0.3		0.7869	0.7642	0.7642
Berkeleyomyces	<0.0001(<0.0001,<0.0001)	0	0	<0.0001(<0.0001,<0.0001)	2.18E-05	1.71E-05	<0.0001(<0.0001,<0.0001)	0	0	0.0715	-1.8		0.1781	0.0715	0.0715
Extremus	<0.0001(<0.0001,<0.0001)	0	0	<0.0001(<0.0001,<0.0001)	2.03E-05	2.03E-05	<0.0001(<0.0001,<0.0001)	0	0	0.26915	-1.11		0.4209	0.2691	0.2691

Oliveonia	<0.0001(<0.0001,<0.0001)	8.56E-06	8.6E-06	<0.0001(<0.0001,<0.0001)	0	0	<0.0001(<0.0001,<0.0001)	0	0	0.76419	-0.3		0.7869	0.7642	0.7642
Saccharomycopsis	<0.0001(<0.0001,<0.0001)	8.09E-06	5.9E-06	<0.0001(<0.0001,<0.0001)	0	0	<0.0001(<0.0001,<0.0001)	0	0	0.58243	-0.55		0.7105	0.5824	0.5824
Neoerysiphe	<0.0001(<0.0001,<0.0001)	7.86E-06	7.5E-06	<0.0001(<0.0001,<0.0001)	0	0	<0.0001(<0.0001,<0.0001)	0	0	0.58243	-0.55		0.7105	0.5824	0.5824
Pyricularia	<0.0001(<0.0001,<0.0001)	7.75E-06	7.5E-06	<0.0001(<0.0001,<0.0001)	0	0	<0.0001(<0.0001,<0.0001)	0	0	0.58243	-0.55		0.7105	0.5824	0.5824
Mycoarthris	<0.0001(<0.0001,<0.0001)	0	0	<0.0001(<0.0001,<0.0001)	1.78E-05	1.78E-05	<0.0001(<0.0001,<0.0001)	0	0	0.26915	-1.11		0.4209	0.2691	0.2691
Byssochlamys	<0.0001(<0.0001,<0.0001)	3.05E-06	2E-06	<0.0001(<0.0001,<0.0001)	0	0	<0.0001(<0.0001,<0.0001)	3.8E-05	3.8E-05	0.22834	-1.2		0.4209	0.2283	0.2283
Ascochyta	<0.0001(<0.0001,<0.0001)	4.86E-06	3.6E-06	<0.0001(<0.0001,<0.0001)	5.39E-06	4.11E-06	<0.0001(<0.0001,<0.0001)	0	0	0.76399	-0.3		0.7869	0.764	0.764
Mrakia	<0.0001(<0.0001,<0.0001)	1.81E-06	1.2E-06	<0.0001(<0.0001,<0.0001)	1.25E-05	1.14E-05	<0.0001(<0.0001,<0.0001)	0	0	0.70358	-0.38		0.7869	0.7036	0.7036
Parathyridaria	<0.0001(<0.0001,<0.0001)	0.000007	6.2E-06	<0.0001(<0.0001,<0.0001)	0	0	<0.0001(<0.0001,<0.0001)	0	0	0.44271	-0.77		0.581	0.4427	0.4427
Xenodevriesia	<0.0001(<0.0001,<0.0001)	6.77E-06	6.4E-06	<0.0001(<0.0001,<0.0001)	0	0	<0.0001(<0.0001,<0.0001)	0	0	0.58243	-0.55		0.7105	0.5824	0.5824
Effuseotrichosporon	<0.0001(<0.0001,<0.0001)	6.29E-07	6.3E-07	<0.0001(<0.0001,<0.0001)	0	0	<0.0001(<0.0001,<0.0001)	5.3E-05	5.3E-05	0.06013	-1.88		0.1649	0.0601	0.0601
Dothiora	<0.0001(<0.0001,<0.0001)	5.14E-06	4.9E-06	<0.0001(<0.0001,<0.0001)	3.54E-06	2.67E-06	<0.0001(<0.0001,<0.0001)	0	0	0.55425	-0.59		0.7105	0.5543	0.5543
Gjaerumia	<0.0001(<0.0001,<0.0001)	6.62E-06	6.6E-06	<0.0001(<0.0001,<0.0001)	0	0	<0.0001(<0.0001,<0.0001)	0	0	0.76419	-0.3		0.7869	0.7642	0.7642
Sidera	<0.0001(<0.0001,<0.0001)	6.61E-06	6.6E-06	<0.0001(<0.0001,<0.0001)	0	0	<0.0001(<0.0001,<0.0001)	0	0	0.76419	-0.3		0.7869	0.7642	0.7642
Neoacladium	<0.0001(<0.0001,<0.0001)	6.43E-06	6.4E-06	<0.0001(<0.0001,<0.0001)	0	0	<0.0001(<0.0001,<0.0001)	0	0	0.76419	-0.3		0.7869	0.7642	0.7642
Strelitziana	<0.0001(<0.0001,<0.0001)	6.14E-06	3.7E-06	<0.0001(<0.0001,<0.0001)	0	0	<0.0001(<0.0001,<0.0001)	0	0	0.44271	-0.77		0.581	0.4427	0.4427
Cerrena	<0.0001(<0.0001,<0.0001)	5.87E-06	4.8E-06	<0.0001(<0.0001,<0.0001)	0	0	<0.0001(<0.0001,<0.0001)	0	0	0.44271	-0.77		0.581	0.4427	0.4427
Phaeoacremonium	<0.0001(<0.0001,<0.0001)	0	0	<0.0001(<0.0001,<0.0001)	1.37E-05	1.37E-05	<0.0001(<0.0001,<0.0001)	0	0	0.26915	-1.11		0.4209	0.2691	0.2691
Tremateia	<0.0001(<0.0001,<0.0001)	5.75E-06	4.8E-06	<0.0001(<0.0001,<0.0001)	0	0	<0.0001(<0.0001,<0.0001)	0	0	0.58243	-0.55		0.7105	0.5824	0.5824
Tetracladium	<0.0001(<0.0001,<0.0001)	5.7E-06	5.7E-06	<0.0001(<0.0001,<0.0001)	0	0	<0.0001(<0.0001,<0.0001)	0	0	0.76419	-0.3		0.7869	0.7642	0.7642
Stephanosporaceae_gen_Incertae_sedis	<0.0001(<0.0001,<0.0001)	5.68E-06	5.7E-06	<0.0001(<0.0001,<0.0001)	0	0	<0.0001(<0.0001,<0.0001)	0	0	0.76419	-0.3		0.7869	0.7642	0.7642
Avachytrium	<0.0001(<0.0001,<0.0001)	5.52E-06	5.5E-06	<0.0001(<0.0001,<0.0001)	0	0	<0.0001(<0.0001,<0.0001)	0	0	0.76419	-0.3		0.7869	0.7642	0.7642
Iodophanus	<0.0001(<0.0001,<0.0001)	5.35E-06	4.3E-06	<0.0001(<0.0001,<0.0001)	0	0	<0.0001(<0.0001,<0.0001)	0	0	0.58243	-0.55		0.7105	0.5824	0.5824
Ochroconis	<0.0001(<0.0001,<0.0001)	0	0	<0.0001(<0.0001,<0.0001)	1.25E-05	1.25E-05	<0.0001(<0.0001,<0.0001)	0	0	0.26915	-1.11		0.4209	0.2691	0.2691
Pararamichloridium	<0.0001(<0.0001,<0.0001)	5.11E-06	5.1E-06	<0.0001(<0.0001,<0.0001)	0	0	<0.0001(<0.0001,<0.0001)	0	0	0.76419	-0.3		0.7869	0.7642	0.7642
Basidiodendron	<0.0001(<0.0001,<0.0001)	5.02E-06	5E-06	<0.0001(<0.0001,<0.0001)	0	0	<0.0001(<0.0001,<0.0001)	0	0	0.76419	-0.3		0.7869	0.7642	0.7642
Coniella	<0.0001(<0.0001,<0.0001)	4.96E-06	5E-06	<0.0001(<0.0001,<0.0001)	0	0	<0.0001(<0.0001,<0.0001)	0	0	0.76419	-0.3		0.7869	0.7642	0.7642

Xylodon	<0.0001(<0.0001,<0.0001)	6.29E-07	4.5E-07	<0.0001(<0.0001,<0.0001)	9.52E-06	3.94E-06	<0.0001(<0.0001,<0.0001)	0	0	0.00237	-3.04	**	0.0145	0.0024	** 0.0024
Iodosphaeria	<0.0001(<0.0001,<0.0001)	4.58E-06	4.6E-06	<0.0001(<0.0001,<0.0001)	0	0	<0.0001(<0.0001,<0.0001)	0	0	0.76419	-0.3		0.7869	0.7642	0.7642
Rhizopogon	<0.0001(<0.0001,<0.0001)	4.55E-06	4.5E-06	<0.0001(<0.0001,<0.0001)	0	0	<0.0001(<0.0001,<0.0001)	0	0	0.76419	-0.3		0.7869	0.7642	0.7642
Robbauera	<0.0001(<0.0001,<0.0001)	4.53E-06	4.5E-06	<0.0001(<0.0001,<0.0001)	0	0	<0.0001(<0.0001,<0.0001)	0	0	0.76419	-0.3		0.7869	0.7642	0.7642
Adisciso	<0.0001(<0.0001,<0.0001)	0	0	<0.0001(<0.0001,<0.0001)	1.05E-05	1.05E-05	<0.0001(<0.0001,<0.0001)	0	0	0.26915	-1.11		0.4209	0.2691	0.2691
Peziza	<0.0001(<0.0001,<0.0001)	0	0	<0.0001(<0.0001,<0.0001)	1.05E-05	1.05E-05	<0.0001(<0.0001,<0.0001)	0	0	0.26915	-1.11		0.4209	0.2691	0.2691
Phialocephala	<0.0001(<0.0001,<0.0001)	0	0	<0.0001(<0.0001,<0.0001)	4.7E-06	3.55E-06	<0.0001(<0.0001,<0.0001)	2E-05	2E-05	0.03976	-2.06	*	0.1243	0.0398	* 0.0398
Wickerhamomyces	<0.0001(<0.0001,<0.0001)	1.71E-06	1.7E-06	<0.0001(<0.0001,<0.0001)	3.55E-06	3.55E-06	<0.0001(<0.0001,<0.0001)	0	0	0.75049	-0.32		0.7869	0.7505	0.7505
Keratinophyton	<0.0001(<0.0001,<0.0001)	0	0	<0.0001(<0.0001,<0.0001)	6.82E-06	6.82E-06	<0.0001(<0.0001,<0.0001)	0	0	0.26915	-1.11		0.4209	0.2691	0.2691
Graphium	<0.0001(<0.0001,<0.0001)	2.86E-06	2.9E-06	<0.0001(<0.0001,<0.0001)	0	0	<0.0001(<0.0001,<0.0001)	0	0	0.76419	-0.3		0.7869	0.7642	0.7642
Groenewaldozyma	<0.0001(<0.0001,<0.0001)	2.84E-06	2.8E-06	<0.0001(<0.0001,<0.0001)	0	0	<0.0001(<0.0001,<0.0001)	0	0	0.76419	-0.3		0.7869	0.7642	0.7642
Choanephora	<0.0001(<0.0001,<0.0001)	2.83E-06	2.8E-06	<0.0001(<0.0001,<0.0001)	0	0	<0.0001(<0.0001,<0.0001)	0	0	0.76419	-0.3		0.7869	0.7642	0.7642
Zymoseptoria	<0.0001(<0.0001,<0.0001)	2.76E-06	2.8E-06	<0.0001(<0.0001,<0.0001)	0	0	<0.0001(<0.0001,<0.0001)	0	0	0.76419	-0.3		0.7869	0.7642	0.7642
Delastria	<0.0001(<0.0001,<0.0001)	2.74E-06	1.8E-06	<0.0001(<0.0001,<0.0001)	0	0	<0.0001(<0.0001,<0.0001)	0	0	0.3356	-0.96		0.4859	0.3356	0.3356
Punctularia	<0.0001(<0.0001,<0.0001)	0	0	<0.0001(<0.0001,<0.0001)	0	0	<0.0001(<0.0001,<0.0001)	2.4E-05	2.4E-05	0.0019	-3.11	**	0.012	0.0019	** 0.0019
Houjia	<0.0001(<0.0001,<0.0001)	0	0	<0.0001(<0.0001,<0.0001)	0	0	<0.0001(<0.0001,<0.0001)	2.4E-05	2.4E-05	0.0019	-3.11	**	0.012	0.0019	** 0.0019
Hydropisphaera	<0.0001(<0.0001,<0.0001)	2.61E-06	2.6E-06	<0.0001(<0.0001,<0.0001)	0	0	<0.0001(<0.0001,<0.0001)	0	0	0.76419	-0.3		0.7869	0.7642	0.7642
Jattaea	<0.0001(<0.0001,<0.0001)	2.61E-06	2.6E-06	<0.0001(<0.0001,<0.0001)	0	0	<0.0001(<0.0001,<0.0001)	0	0	0.76419	-0.3		0.7869	0.7642	0.7642
Fracchiaea	<0.0001(<0.0001,<0.0001)	1.26E-06	1.3E-06	<0.0001(<0.0001,<0.0001)	3.02E-06	3.02E-06	<0.0001(<0.0001,<0.0001)	0	0	0.74278	-0.33		0.7869	0.7428	0.7428
Gibellulopsis	<0.0001(<0.0001,<0.0001)	0	0	<0.0001(<0.0001,<0.0001)	5.91E-06	5.91E-06	<0.0001(<0.0001,<0.0001)	0	0	0.26915	-1.11		0.4209	0.2691	0.2691
Sistotremastrum	<0.0001(<0.0001,<0.0001)	0	0	<0.0001(<0.0001,<0.0001)	5.91E-06	4.73E-06	<0.0001(<0.0001,<0.0001)	0	0	0.0715	-1.8		0.1781	0.0715	0.0715
Paracremonium	<0.0001(<0.0001,<0.0001)	2.45E-06	2E-06	<0.0001(<0.0001,<0.0001)	0	0	<0.0001(<0.0001,<0.0001)	0	0	0.58243	-0.55		0.7105	0.5824	0.5824
Leptosporella	<0.0001(<0.0001,<0.0001)	2.43E-06	2.4E-06	<0.0001(<0.0001,<0.0001)	0	0	<0.0001(<0.0001,<0.0001)	0	0	0.76419	-0.3		0.7869	0.7642	0.7642
Chytridiomycota_gen_Incertae_sedis	<0.0001(<0.0001,<0.0001)	2.42E-06	2.4E-06	<0.0001(<0.0001,<0.0001)	0	0	<0.0001(<0.0001,<0.0001)	0	0	0.76419	-0.3		0.7869	0.7642	0.7642
Pseudorobillarda	<0.0001(<0.0001,<0.0001)	2.36E-06	2.4E-06	<0.0001(<0.0001,<0.0001)	0	0	<0.0001(<0.0001,<0.0001)	0	0	0.76419	-0.3		0.7869	0.7642	0.7642
Cookeina	<0.0001(<0.0001,<0.0001)	0	0	<0.0001(<0.0001,<0.0001)	4.91E-06	3.85E-06	<0.0001(<0.0001,<0.0001)	0	0	0.0715	-1.8		0.1781	0.0715	0.0715
Entoloma	<0.0001(<0.0001,<0.0001)	0	0	<0.0001(<0.0001,<0.0001)	4.64E-06	4.64E-06	<0.0001(<0.0001,<0.0001)	0	0	0.26915	-1.11		0.4209	0.2691	0.2691
Amylostereum	<0.0001(<0.0001,<0.0001)	0	0	<0.0001(<0.0001,<0.0001)	4.27E-06	4.27E-06	<0.0001(<0.0001,<0.0001)	0	0	0.26915	-1.11		0.4209	0.2691	0.2691

Paraleptosphaeria	<0.0001(<0.0001,<0.0001)	1.56E-06	1.6E-06	<0.0001(<0.0001,<0.0001)	0	0	<0.0001(<0.0001,<0.0001)	0	0	0.76419	-0.3		0.7869	0.7642	0.7642
Powellomyces	<0.0001(<0.0001,<0.0001)	0	0	<0.0001(<0.0001,<0.0001)	3.68E-06	3.68E-06	<0.0001(<0.0001,<0.0001)	0	0	0.26915	-1.11		0.4209	0.2691	0.2691
Vararia	<0.0001(<0.0001,<0.0001)	1.48E-06	1.5E-06	<0.0001(<0.0001,<0.0001)	0	0	<0.0001(<0.0001,<0.0001)	0	0	0.76419	-0.3		0.7869	0.7642	0.7642
Scopulariopsis	<0.0001(<0.0001,<0.0001)	0	0	<0.0001(<0.0001,<0.0001)	3.43E-06	3.43E-06	<0.0001(<0.0001,<0.0001)	0	0	0.26915	-1.11		0.4209	0.2691	0.2691
Gregarithecium	<0.0001(<0.0001,<0.0001)	1.3E-06	1.3E-06	<0.0001(<0.0001,<0.0001)	0	0	<0.0001(<0.0001,<0.0001)	0	0	0.76419	-0.3		0.7869	0.7642	0.7642
Scopuloides	<0.0001(<0.0001,<0.0001)	0	0	<0.0001(<0.0001,<0.0001)	2.7E-06	2.7E-06	<0.0001(<0.0001,<0.0001)	0	0	0.26915	-1.11		0.4209	0.2691	0.2691
Chaetosphaeria	<0.0001(<0.0001,<0.0001)	0	0	<0.0001(<0.0001,<0.0001)	2.57E-06	2.57E-06	<0.0001(<0.0001,<0.0001)	0	0	0.26915	-1.11		0.4209	0.2691	0.2691
Echinoporia	<0.0001(<0.0001,<0.0001)	9.02E-07	9E-07	<0.0001(<0.0001,<0.0001)	0	0	<0.0001(<0.0001,<0.0001)	0	0	0.76419	-0.3		0.7869	0.7642	0.7642
Septobasidium	<0.0001(<0.0001,<0.0001)	7.27E-07	7.3E-07	<0.0001(<0.0001,<0.0001)	0	0	<0.0001(<0.0001,<0.0001)	0	0	0.76419	-0.3		0.7869	0.7642	0.7642
Cystobasidium	<0.0001(<0.0001,<0.0001)	0	0	<0.0001(<0.0001,<0.0001)	1.7E-06	1.7E-06	<0.0001(<0.0001,<0.0001)	0	0	0.26915	-1.11		0.4209	0.2691	0.2691
Emericellopsis	<0.0001(<0.0001,<0.0001)	0	0	<0.0001(<0.0001,<0.0001)	1.45E-06	1.45E-06	<0.0001(<0.0001,<0.0001)	0	0	0.26915	-1.11		0.4209	0.2691	0.2691
Chaenothecopsis	<0.0001(<0.0001,<0.0001)	0	0	<0.0001(<0.0001,<0.0001)	1.38E-06	1.38E-06	<0.0001(<0.0001,<0.0001)	0	0	0.26915	-1.11		0.4209	0.2691	0.2691
Zygosporium	<0.0001(<0.0001,<0.0001)	5.83E-07	5.8E-07	<0.0001(<0.0001,<0.0001)	0	0	<0.0001(<0.0001,<0.0001)	0	0	0.76419	-0.3		0.7869	0.7642	0.7642
Sphaerulina	<0.0001(<0.0001,<0.0001)	0	0	<0.0001(<0.0001,<0.0001)	1.34E-06	1.34E-06	<0.0001(<0.0001,<0.0001)	0	0	0.26915	-1.11		0.4209	0.2691	0.2691
Edenia	<0.0001(<0.0001,<0.0001)	5.15E-07	5.2E-07	<0.0001(<0.0001,<0.0001)	0	0	<0.0001(<0.0001,<0.0001)	0	0	0.76419	-0.3		0.7869	0.7642	0.7642
Paludomyces	<0.0001(<0.0001,<0.0001)	0	0	<0.0001(<0.0001,<0.0001)	1.13E-06	1.13E-06	<0.0001(<0.0001,<0.0001)	0	0	0.26915	-1.11		0.4209	0.2691	0.2691
Chloridium	<0.0001(<0.0001,<0.0001)	0	0	<0.0001(<0.0001,<0.0001)	1.09E-06	1.09E-06	<0.0001(<0.0001,<0.0001)	0	0	0.26915	-1.11		0.4209	0.2691	0.2691
Emergomyces	<0.0001(<0.0001,<0.0001)	4.32E-07	4.3E-07	<0.0001(<0.0001,<0.0001)	0	0	<0.0001(<0.0001,<0.0001)	0	0	0.76419	-0.3		0.7869	0.7642	0.7642
Geotrichum	<0.0001(<0.0001,<0.0001)	0	0	<0.0001(<0.0001,<0.0001)	9.11E-07	9.11E-07	<0.0001(<0.0001,<0.0001)	0	0	0.26915	-1.11		0.4209	0.2691	0.2691
Inocyclus	<0.0001(<0.0001,<0.0001)	0	0	<0.0001(<0.0001,<0.0001)	8.75E-07	8.75E-07	<0.0001(<0.0001,<0.0001)	0	0	0.26915	-1.11		0.4209	0.2691	0.2691

Supplementary Table S7. The different degree of species level (p value) among control (n=132), COVID-19-N (n=56), and COVID-19-C (n=15) groups

ID	Control.median	Control.mean	Control.se	COVID-19.median	COVID-19.mean	COVID-19.se	COVID-19-C.median	COVID-19-C.mean	COVID-19-C.se	p-value	z-score	Sig_mark	q-value	fixp	fixps
Acrodictys_fluminicola	0.0341(0.0091,0.3007)	0.182386	0.02101	0.1043(0.0181,0.6448)	0.295128	0.042549	0.0775(0.0294,0.1452)	0.19051	0.07115	0.06026	-1.88		0.1658	0.0603	0.0603
Zanclospora_jonesii	0.0732(0.0146,0.3211)	0.219276	0.02397	0.0175(0.0067,0.0825)	0.147875	0.03769	0.014(0.0082,0.0561)	0.05769	0.02298	0.00111	-3.26	**	0.0103	0.0011	** 0.0011
Aspergillus_subflavus	0.007(0.0018,0.0609)	0.073277	0.01328	0.0104(0.0028,0.0266)	0.074688	0.025349	0.0225(0.0081,0.1115)	0.09332	0.03237	0.17398	-1.36		0.3576	0.174	0.174
Blumeria_spe_Incertae_sedis	0.0016(0.0006,0.0102)	0.04539	0.0103	0.0008(0.0002,0.0035)	0.022175	0.013184	0.0102(0.0016,0.2573)	0.12993	0.04323	0.00151	-3.17	**	0.0125	0.0015	** 0.0015
Cryptococcus_longus	0.0163(0.002,0.0547)	0.062697	0.01085	<0.0001(<0.0001,<0.0001)	2.57E-05	1.56E-05	<0.0001(<0.0001,<0.0001)	2.7E-05	2.2E-05	2.7E-31	-11.6	***	2E-28	<0.0001	***<0.0001
Malassezia_restricta	0.0054(0.0008,0.0336)	0.033444	0.00611	0.0009(0.0003,0.0087)	0.022914	0.008543	0.0009(0.0004,0.0303)	0.02628	0.01173	0.0098	-2.58	**	0.0441	0.0098	** 0.0098
Chrysosphaeria_jan-nelii	0.0001(<0.0001,0.0004)	0.003136	0.00136	<0.0001(<0.0001,0.0005)	0.062906	0.022261	<0.0001(<0.0001,0.0107)	0.07847	0.0582	0.60559	-0.52		0.729	0.6056	0.6056
Diversispora_spurca	0.0017(0.0006,0.0146)	0.029246	0.00983	0.0009(0.0001,0.003)	0.004882	0.001511	0.0008(<0.0001,0.0022)	0.00288	0.00127	0.00504	-2.8	**	0.0281	0.005	** 0.005
Raffaelea_lauricola	0.0005(<0.0001,0.002)	0.022161	0.0093	0.0013(0.0004,0.0064)	0.0053	0.001446	0.0012(0.0004,0.0038)	0.00254	0.00077	0.00523	-2.79	**	0.0283	0.0052	** 0.0052

Hydnobolites_roseus	<0.0001(<0.0001,<0.0001)	0.001496	0.00072	0.0002(<0.0001,0.0018)	0.042453	0.022936	0.0003(0.0001,0.0009)	0.03189	0.03139	1.3E-05	-4.35	***	0.0003	<0.0001	***<0.0001
Candida_albicans	<0.0001(<0.0001,<0.0001)	0.000103	2.5E-05	0.0014(0.0003,0.0048)	0.04441	0.022852	0.0009(<0.0001,0.0041)	0.03379	0.03223	5.7E-17	-8.37	***	2E-14	<0.0001	***<0.0001
Cladosporium_kenpeggii	0.0011(0.0003,0.0089)	0.016085	0.00343	0.0002(<0.0001,0.0017)	0.00606	0.002458	0.0002(<0.0001,0.0105)	0.00766	0.00344	0.00032	-3.6	***	0.0039	0.0003	***0.0003
Cladosporium_cladosporioides	0.0009(0.0003,0.0069)	0.013677	0.00294	0.0002(0.0001,0.0013)	0.010854	0.004854	0.0003(0.0001,0.0066)	0.00724	0.00335	0.00677	-2.71	**	0.035	0.0068	** 0.0068
Arthrographis_grakistii	0.0001(<0.0001,0.0008)	0.018769	0.01051	<0.0001(<0.0001,0.0001)	0.00015	6.68E-05	<0.0001(<0.0001,<0.0001)	8.7E-05	4.1E-05	0.00014	-3.81	***	0.0021	0.0001	***0.0001
Simplicillium_sympodiophorum	0.0004(0.0002,0.0043)	0.007264	0.00124	0.0026(0.0006,0.0417)	0.022493	0.00419	0.0016(0.0003,0.0083)	0.01324	0.00644	0.00016	-3.77	***	0.0023	0.0002	***0.0002
Cladosporium_halotolerans	0.0001(<0.0001,0.0009)	0.006278	0.00191	0.0003(<0.0001,0.0034)	0.018252	0.009082	0.001(0.0005,0.0214)	0.0345	0.01964	0.0016	-3.16	**	0.013	0.0016	** 0.0016
Saccharomyces_cerevisiae	<0.0001(<0.0001,0.0003)	0.001336	0.00044	0.0005(<0.0001,0.0026)	0.022645	0.011213	0.0007(0.0001,0.0216)	0.04922	0.02599	1.4E-06	-4.83	***	5E-05	<0.0001	***<0.0001
Alternaria_alternata	0.0005(0.0002,0.0036)	0.011542	0.00315	0.0001(<0.0001,0.0006)	0.002666	0.000902	0.0001(<0.0001,0.002)	0.00269	0.00131	0.0005	-3.48	***	0.0058	0.0005	***0.0005
Milospium_graphideorum	0.0002(<0.0001,0.001)	0.012121	0.00686	<0.0001(<0.0001,0.0003)	0.000519	0.00025	<0.0001(<0.0001,0.001)	0.00087	0.00049	0.01694	-2.39	*	0.0684	0.0169	* 0.0169
[Cryptococcus]_ater	0.0004(0.0001,0.0017)	0.006671	0.00137	0.0014(0.0003,0.0161)	0.009638	0.001953	0.0002(0.0001,0.0016)	0.00592	0.003	0.00635	-2.73	**	0.0333	0.0063	** 0.0063
Russula_xerophila	0.0003(<0.0001,0.0023)	0.007204	0.00197	0.0004(0.0002,0.0031)	0.007618	0.003107	0.0014(0.0001,0.0076)	0.00439	0.00148	0.41328	-0.82		0.5856	0.4133	0.4133
Schizophyllum_commune	<0.0001(<0.0001,<0.0001)	0.008264	0.00737	<0.0001(<0.0001,<0.0001)	0.0007	0.000459	<0.0001(<0.0001,<0.0001)	0.00034	0.00032	0.40008	-0.84		0.5706	0.4001	0.4001
Parafuscosporella_mucosa	0.0008(0.0001,0.0053)	0.004611	0.00102	0.0024(0.0004,0.0092)	0.006971	0.002106	0.0003(0.0002,0.0014)	0.00235	0.00104	0.03554	-2.1	*	0.1168	0.0355	* 0.0355
Trigonopsis_californica	<0.0001(<0.0001,<0.0001)	0.007453	0.00726	<0.0001(<0.0001,<0.0001)	0	0	<0.0001(<0.0001,<0.0001)	0	0	0.00013	-3.84	***	0.002	0.0001	***0.0001
Penicillium_melanoconidium	<0.0001(<0.0001,<0.0001)	0.007388	0.00727	<0.0001(<0.0001,<0.0001)	5.66E-06	5.66E-06	<0.0001(<0.0001,<0.0001)	7.3E-06	5E-06	0.01648	-2.4	*	0.0673	0.0165	* 0.0165
Pleurotus_ostreatus	<0.0001(<0.0001,0.0002)	0.003574	0.00153	<0.0001(<0.0001,0.0002)	0.00352	0.001771	0.0006(<0.0001,0.0016)	0.02024	0.01889	0.00959	-2.59	**	0.0439	0.0096	** 0.0096
Malassezia_arunalokei	<0.0001(<0.0001,0.0005)	0.004934	0.00246	<0.0001(<0.0001,0.0002)	0.002878	0.001322	<0.0001(<0.0001,0.0002)	0.00226	0.00171	0.01019	-2.57	*	0.0449	0.0102	* 0.0102
Talaromyces_scorteus	<0.0001(<0.0001,0.0002)	0.006259	0.00211	<0.0001(<0.0001,<0.0001)	0.000232	0.000157	<0.0001(<0.0001,<0.0001)	9E-05	5.6E-05	0.00026	-3.66	***	0.0033	0.0003	***0.0003
Gibberella_fujikuroi	<0.0001(<0.0001,<0.0001)	1.04E-05	4.7E-06	<0.0001(<0.0001,0.0007)	0.011717	0.004983	<0.0001(<0.0001,0.0031)	0.01193	0.00889	1.9E-14	-7.66	***	4E-12	<0.0001	***<0.0001
Saprochaete_gigas	<0.0001(<0.0001,<0.0001)	0.00606	0.00589	<0.0001(<0.0001,<0.0001)	2.49E-05	2.49E-05	<0.0001(<0.0001,<0.0001)	7.1E-05	6.5E-05	0.07381	-1.79		0.1898	0.0738	0.0738
Aspergillus_amstelodami	<0.0001(<0.0001,0.0003)	0.004919	0.00187	<0.0001(<0.0001,<0.0001)	0.001865	0.00108	<0.0001(<0.0001,0.0001)	0.00124	0.00086	0.00102	-3.29	**	0.0098	0.001	** 0.001
Neomicrosphaeropsis_alhagi-pseudalhagi	<0.0001(<0.0001,<0.0001)	0.000538	0.00024	<0.0001(<0.0001,<0.0001)	0.011165	0.010884	0.0001(<0.0001,0.0018)	0.00222	0.00145	0.03013	-2.17	*	0.1056	0.0301	* 0.0301
Angustimassarina_camporesii	0.0002(<0.0001,0.0026)	0.002793	0.00044	0.0011(0.0002,0.0105)	0.005309	0.000876	0.0007(0.0001,0.0022)	0.00293	0.00131	0.00303	-2.96	**	0.0189	0.003	** 0.003
Candida_spe_Incertae_sedis	<0.0001(<0.0001,0.0002)	0.001231	0.00054	<0.0001(<0.0001,0.0003)	0.001394	0.000626	0.0003(0.0001,0.0009)	0.02976	0.01703	0.00053	-3.46	***	0.0059	0.0005	***0.0005
Malassezia_globosa	0.0002(<0.0001,0.0019)	0.003037	0.00062	<0.0001(<0.0001,0.0001)	0.000902	0.000524	<0.0001(<0.0001,0.0002)	0.01101	0.01092	1.6E-05	-4.31	***	0.0004	<0.0001	***<0.0001
Nigrospora_oryzae	<0.0001(<0.0001,<0.0001)	0.00047	0.00033	<0.0001(<0.0001,0.0001)	0.004079	0.003234	<0.0001(<0.0001,0.0004)	0.02106	0.01921	0.00014	-3.81	***	0.0021	0.0001	***0.0001
Aspergillus_penicillioides	<0.0001(<0.0001,0.0007)	0.003246	0.00101	<0.0001(<0.0001,0.0009)	0.001848	0.000574	0.0001(<0.0001,0.0016)	0.00374	0.00255	0.87884	-0.15		0.8843	0.8788	0.8788
Fantasmomyces_hyalinus	<0.0001(<0.0001,0.0013)	0.002101	0.00055	0.0008(0.0001,0.0079)	0.004554	0.0009	0.0003(<0.0001,0.0036)	0.00222	0.0009	0.00126	-3.23	**	0.011	0.0013	** 0.0013
Candida_parapsilosis	<0.0001(<0.0001,0.0001)	0.003702	0.0014	<0.0001(<0.0001,<0.0001)	0.001204	0.000608	<0.0001(<0.0001,<0.0001)	0.00057	0.00057	0.00062	-3.42	***	0.0067	0.0006	***0.0006
Diaporthe_kochmanii	<0.0001(<0.0001,0.0001)	0.003735	0.0024	<0.0001(<0.0001,<0.0001)	0	0	<0.0001(<0.0001,<0.0001)	0	0	2.6E-07	-5.15	***	2E-05	<0.0001	***<0.0001

Phaeosphaeria_oryzae	<0.0001(<0.0001,0.0003)	0.003419	0.00225	<0.0001(<0.0001,<0.0001)	0.00012	8.91E-05	<0.0001(<0.0001,<0.0001)	0	0	0.00012	-3.84	***	0.002	0.0001	***0.0001
Wallemia_canadensis	<0.0001(<0.0001,<0.0001)	0.002712	0.00258	<0.0001(<0.0001,<0.0001)	6.1E-05	4.7E-05	<0.0001(<0.0001,<0.0001)	0	0	0.08981	-1.7		0.2191	0.0898	0.0898
Trichomonascus_ciferrii	<0.0001(<0.0001,<0.0001)	0.002686	0.00264	<0.0001(<0.0001,<0.0001)	0	0	<0.0001(<0.0001,<0.0001)	1.5E-05	1.5E-05	0.01719	-2.38	*	0.069	0.0172	* 0.0172
Naganishia_albida	<0.0001(<0.0001,<0.0001)	0.002664	0.00194	<0.0001(<0.0001,<0.0001)	9.29E-07	9.29E-07	<0.0001(<0.0001,<0.0001)	0.00013	0.00013	0.00095	-3.31	***	0.0094	0.0009	***0.0009
Ophiostoma_acarorum	<0.0001(<0.0001,0.0001)	0.002643	0.0022	<0.0001(<0.0001,<0.0001)	1.79E-06	1.79E-06	<0.0001(<0.0001,<0.0001)	0	0	2.2E-09	-5.98	***	4E-07	<0.0001	***<0.0001
Botrytis_cinerea	<0.0001(<0.0001,0.0001)	0.002254	0.0015	<0.0001(<0.0001,<0.0001)	8.54E-05	7.1E-05	<0.0001(<0.0001,0.0002)	0.00177	0.00116	5.2E-05	-4.05	***	0.001	<0.0001	***<0.0001
Galactomyces_pseudocandidus	<0.0001(<0.0001,0.0001)	0.00045	0.00014	<0.0001(<0.0001,<0.0001)	0.004727	0.004644	<0.0001(<0.0001,<0.0001)	0.0001	7E-05	0.21987	-1.23		0.4269	0.2199	0.2199
Acremonium_alternatum	<0.0001(<0.0001,<0.0001)	0.002392	0.00238	<0.0001(<0.0001,<0.0001)	9.11E-07	9.11E-07	<0.0001(<0.0001,<0.0001)	0	0	0.0157	-2.42	*	0.0653	0.0157	* 0.0157
Udeniomyces_megalosporus	<0.0001(<0.0001,<0.0001)	0.002256	0.00184	<0.0001(<0.0001,<0.0001)	1.53E-05	8.35E-06	<0.0001(<0.0001,<0.0001)	3.1E-05	2.7E-05	0.00186	-3.11	**	0.0136	0.0019	** 0.0019
Zygotorulaspora_danielsina	<0.0001(<0.0001,<0.0001)	0.000848	0.00061	<0.0001(<0.0001,0.0001)	0.003098	0.001848	<0.0001(<0.0001,<0.0001)	4.3E-05	2.8E-05	0.14201	-1.47		0.3059	0.142	0.142
Aureobasidium_pullulans	<0.0001(<0.0001,<0.0001)	0.000565	0.00033	<0.0001(<0.0001,<0.0001)	0.003606	0.002233	<0.0001(<0.0001,<0.0001)	0.00051	0.00038	0.04209	-2.03	*	0.1263	0.0421	* 0.0421
Malassezia_sympodialis	<0.0001(<0.0001,0.0002)	0.002014	0.00051	<0.0001(<0.0001,<0.0001)	0.000207	0.000132	<0.0001(<0.0001,0.0001)	0.00021	0.00016	8.4E-07	-4.93	***	5E-05	<0.0001	***<0.0001
Golubevia_pallescens	<0.0001(<0.0001,<0.0001)	0.002124	0.00176	<0.0001(<0.0001,<0.0001)	0	0	<0.0001(<0.0001,<0.0001)	1.8E-05	1.8E-05	0.0001	-3.88	***	0.0019	0.0001	***0.0001
Cladosporium_sphaerospermum	<0.0001(<0.0001,<0.0001)	0.001835	0.00068	<0.0001(<0.0001,<0.0001)	0.000537	0.000347	<0.0001(<0.0001,<0.0001)	0.00033	0.0002	0.28972	-1.06		0.4594	0.2897	0.2897
Filobasidium_oeirense	<0.0001(<0.0001,<0.0001)	0.000411	0.00029	<0.0001(<0.0001,0.0001)	0.003523	0.00193	<0.0001(<0.0001,0.0002)	0.00169	0.00154	0.00364	-2.91	**	0.0216	0.0036	** 0.0036
Coprinellus_ovatus	<0.0001(<0.0001,<0.0001)	0.001423	0.00071	<0.0001(<0.0001,<0.0001)	0.001543	0.001532	<0.0001(<0.0001,<0.0001)	0	0	0.00068	-3.4	***	0.0071	0.0007	***0.0007
Togniniella_acerosa	<0.0001(<0.0001,<0.0001)	0.002027	0.00117	<0.0001(<0.0001,<0.0001)	4.96E-05	2.05E-05	<0.0001(<0.0001,0.0001)	0.0002	0.00011	0.05452	-1.92		0.1585	0.0545	0.0545
Talaromyces_funiculosus	<0.0001(<0.0001,<0.0001)	0.001596	0.00111	<0.0001(<0.0001,<0.0001)	0.000787	0.000445	<0.0001(<0.0001,<0.0001)	0.00021	0.00014	0.1279	-1.52		0.2861	0.1279	0.1279
Cladosporium_spe_Incertae_sedis	<0.0001(<0.0001,0.0005)	0.001298	0.00034	<0.0001(<0.0001,0.0002)	0.000898	0.000495	<0.0001(<0.0001,0.001)	0.00087	0.00041	0.01607	-2.41	*	0.066	0.0161	* 0.0161
Aspergillus_versicolor	<0.0001(<0.0001,<0.0001)	0.000905	0.00028	<0.0001(<0.0001,0.0001)	0.001209	0.000572	<0.0001(<0.0001,0.0003)	0.00299	0.00212	0.18787	-1.32		0.3758	0.1879	0.1879
Penicillium_hirsutum	<0.0001(<0.0001,<0.0001)	0.000257	0.00017	<0.0001(<0.0001,<0.0001)	0.003471	0.002278	<0.0001(<0.0001,<0.0001)	0.00021	0.00018	0.0022	-3.06	**	0.0153	0.0022	** 0.0022
Candida_tropicalis	<0.0001(<0.0001,<0.0001)	0.001311	0.00127	<0.0001(<0.0001,<0.0001)	0.000785	0.000763	<0.0001(<0.0001,0.0003)	0.00034	0.00019	0.32895	-0.98		0.4934	0.329	0.329
Cercospora_canescens	<0.0001(<0.0001,<0.0001)	0.001679	0.0014	<0.0001(<0.0001,<0.0001)	0	0	<0.0001(<0.0001,<0.0001)	0	0	9E-07	-4.91	***	5E-05	<0.0001	***<0.0001
Irpex_lacteus	<0.0001(<0.0001,<0.0001)	0.00067	0.00061	<0.0001(<0.0001,<0.0001)	0.002105	0.002083	<0.0001(<0.0001,<0.0001)	0.00087	0.00085	0.76179	-0.3		0.7866	0.7618	0.7618
Trametes_cubensis	<0.0001(<0.0001,<0.0001)	0.001646	0.00155	<0.0001(<0.0001,<0.0001)	0	0	<0.0001(<0.0001,<0.0001)	0	0	0.00194	-3.1	**	0.0136	0.0019	** 0.0019
Wallemia_mellicola	<0.0001(<0.0001,<0.0001)	0.001572	0.0009	<0.0001(<0.0001,<0.0001)	4.11E-05	2.61E-05	<0.0001(<0.0001,<0.0001)	0.00046	0.00046	0.00015	-3.8	***	0.0021	0.0001	***0.0001
Pseudoveronaea_ellipsoidea	<0.0001(<0.0001,<0.0001)	3.6E-06	3.2E-06	<0.0001(<0.0001,<0.0001)	0.003471	0.002984	<0.0001(<0.0001,<0.0001)	0.00104	0.00101	9.8E-07	-4.9	***	5E-05	<0.0001	***<0.0001
Trametes_versicolor	<0.0001(<0.0001,<0.0001)	0.000627	0.00035	<0.0001(<0.0001,<0.0001)	0.00218	0.00139	<0.0001(<0.0001,<0.0001)	4.1E-06	4.1E-06	0.13077	-1.51		0.2903	0.1308	0.1308
Purpureocillium_lilacinum	<0.0001(<0.0001,<0.0001)	0.001541	0.001	<0.0001(<0.0001,<0.0001)	0	0	<0.0001(<0.0001,<0.0001)	4.4E-06	4.4E-06	4.3E-05	-4.09	***	0.0008	<0.0001	***<0.0001

Fusarium_poae	<0.0001(<0.0001,<0.0001)	0.00117	0.00074	<0.0001(<0.0001,<0.0001)	0.00067	0.000376	<0.0001(<0.0001,0.0001)	0.00075	0.00052	0.23802	-1.18		0.433	0.238	0.238
Leptospora_rubella	<0.0001(<0.0001,<0.0001)	0.001338	0.00077	<0.0001(<0.0001,<0.0001)	0.000396	0.000318	<0.0001(<0.0001,<0.0001)	0.00015	0.00014	0.04141	-2.04	*	0.1249	0.0414	* 0.0414
Aspergillus_thermomutatus	<0.0001(<0.0001,<0.0001)	0.000145	8.3E-05	<0.0001(<0.0001,<0.0001)	0.003149	0.002997	<0.0001(<0.0001,<0.0001)	1.4E-05	9.9E-06	0.6128	-0.51		0.7354	0.6128	0.6128
Apiotrichum_domesticum	<0.0001(<0.0001,0.0001)	0.001397	0.00057	<0.0001(<0.0001,<0.0001)	0.000113	1E-04	<0.0001(<0.0001,<0.0001)	0.00025	0.0002	0.00011	-3.86	***	0.002	0.0001	***0.0001
Rhexothecium_globosum	<0.0001(<0.0001,0.0001)	0.001278	0.00076	<0.0001(<0.0001,<0.0001)	0.000124	6.57E-05	<0.0001(<0.0001,0.0005)	0.00071	0.00035	0.06611	-1.84		0.1783	0.0661	0.0661
Peniophora_crassitunicata	<0.0001(<0.0001,<0.0001)	0.000662	0.00053	<0.0001(<0.0001,<0.0001)	0.001585	0.001573	<0.0001(<0.0001,<0.0001)	0	0	0.08537	-1.72		0.2097	0.0854	0.0854
Yarrowia_bubula	<0.0001(<0.0001,<0.0001)	0.001272	0.00075	<0.0001(<0.0001,<0.0001)	0	0	<0.0001(<0.0001,<0.0001)	0	0	0.00051	-3.48	***	0.0058	0.0005	***0.0005
Bjerkandera_mikrofumosa	<0.0001(<0.0001,<0.0001)	0.001261	0.00122	<0.0001(<0.0001,<0.0001)	3.79E-06	3.79E-06	<0.0001(<0.0001,<0.0001)	0	0	0.0028	-2.99	**	0.0183	0.0028	** 0.0028
Trametes_trogii	<0.0001(<0.0001,<0.0001)	0.001189	0.00117	<0.0001(<0.0001,<0.0001)	0	0	<0.0001(<0.0001,<0.0001)	0	0	0.00373	-2.9	**	0.022	0.0037	** 0.0037
Verrucaria_oulankaensis	<0.0001(<0.0001,<0.0001)	0.001182	0.00109	<0.0001(<0.0001,<0.0001)	5.8E-06	5.8E-06	<0.0001(<0.0001,<0.0001)	0	0	0.0028	-2.99	**	0.0183	0.0028	** 0.0028
Cladosporium_coloradense	<0.0001(<0.0001,<0.0001)	0.001055	0.00061	<0.0001(<0.0001,<0.0001)	9.53E-05	5.35E-05	<0.0001(<0.0001,0.0001)	0.00018	9.9E-05	0.01893	-2.35	*	0.0708	0.0189	* 0.0189
Pleurotus_eryngii	<0.0001(<0.0001,<0.0001)	0.001098	0.00065	<0.0001(<0.0001,<0.0001)	1.13E-06	1.13E-06	<0.0001(<0.0001,<0.0001)	4.1E-06	4.1E-06	0.00612	-2.74	**	0.0326	0.0061	** 0.0061
Vishniacozyma_victoriae	<0.0001(<0.0001,<0.0001)	0.001049	0.001	<0.0001(<0.0001,<0.0001)	7.82E-05	6.92E-05	<0.0001(<0.0001,<0.0001)	1.5E-05	1.5E-05	0.36199	-0.91		0.5287	0.362	0.362
Cystofilobasidium_macerans	<0.0001(<0.0001,<0.0001)	0.001044	0.00076	<0.0001(<0.0001,<0.0001)	7.01E-05	6.77E-05	<0.0001(<0.0001,<0.0001)	7E-06	7E-06	0.00801	-2.65	**	0.0395	0.008	** 0.008
Yarrowia_lipolytica	<0.0001(<0.0001,<0.0001)	0.001013	0.00045	<0.0001(<0.0001,<0.0001)	7.68E-05	7.68E-05	<0.0001(<0.0001,<0.0001)	0	0	0.00014	-3.81	***	0.0021	0.0001	***0.0001
Hannaella_sinensis	<0.0001(<0.0001,<0.0001)	3.72E-05	3.1E-05	<0.0001(<0.0001,<0.0001)	0.002347	0.002304	<0.0001(<0.0001,0.0002)	9.2E-05	3.8E-05	0.00362	-2.91	**	0.0216	0.0036	** 0.0036
Ramularia_coryli	<0.0001(<0.0001,<0.0001)	4.47E-07	4.5E-07	<0.0001(<0.0001,<0.0001)	0.002412	0.002369	<0.0001(<0.0001,<0.0001)	4.7E-05	4.7E-05	0.00012	-3.85	***	0.002	0.0001	***0.0001
Filobasidium_wieringae	<0.0001(<0.0001,<0.0001)	0.001015	0.00081	<0.0001(<0.0001,<0.0001)	4.59E-06	2.84E-06	<0.0001(<0.0001,<0.0001)	0	0	0.00192	-3.1	**	0.0136	0.0019	** 0.0019
Fusarium_lacertarum	<0.0001(<0.0001,<0.0001)	0.00059	0.00024	<0.0001(<0.0001,<0.0001)	0.000816	0.000531	<0.0001(<0.0001,0.0001)	0.00068	0.00044	0.3878	-0.86		0.5577	0.3878	0.3878
Setophaeosphaeria_badalingensis	<0.0001(<0.0001,<0.0001)	0.000964	0.00089	<0.0001(<0.0001,<0.0001)	0	0	<0.0001(<0.0001,<0.0001)	0	0	0.0027	-3	**	0.018	0.0027	** 0.0027
Verrucaria_vitikainenii	<0.0001(<0.0001,<0.0001)	0.000952	0.0009	<0.0001(<0.0001,<0.0001)	0	0	<0.0001(<0.0001,<0.0001)	0	0	0.0027	-3	**	0.018	0.0027	** 0.0027
Tilletiopsis_washingtonensis	<0.0001(<0.0001,<0.0001)	0.000234	0.00018	<0.0001(<0.0001,<0.0001)	0.001632	0.001545	<0.0001(<0.0001,<0.0001)	0.00011	0.00011	0.40298	-0.84		0.5735	0.403	0.403
Dioszegia_zsoltii_var._yunnanensis	<0.0001(<0.0001,<0.0001)	0.000934	0.00086	<0.0001(<0.0001,<0.0001)	3.5E-06	2.55E-06	<0.0001(<0.0001,<0.0001)	0	0	0.01356	-2.47	*	0.0568	0.0136	* 0.0136
Leucosporidium_golubevii	<0.0001(<0.0001,<0.0001)	3.69E-05	3.3E-05	<0.0001(<0.0001,<0.0001)	0.002109	0.002014	<0.0001(<0.0001,<0.0001)	0	0	0.00236	-3.04	**	0.0161	0.0024	** 0.0024
Neosetophoma_poaceicola	<0.0001(<0.0001,<0.0001)	0.000412	0.0004	<0.0001(<0.0001,<0.0001)	8.54E-05	8.23E-05	<0.0001(<0.0001,<0.0001)	0.00383	0.00382	0.31841	-1		0.4909	0.3184	0.3184
Stagonosporopsis_citrulli	<0.0001(<0.0001,<0.0001)	0.000882	0.00083	<0.0001(<0.0001,<0.0001)	0	0	<0.0001(<0.0001,<0.0001)	0	0	0.00514	-2.8	**	0.0281	0.0051	** 0.0051
Kazachstania_heterogenica	<0.0001(<0.0001,<0.0001)	0.000502	0.00035	<0.0001(<0.0001,0.0002)	0.000762	0.000629	<0.0001(<0.0001,<0.0001)	0.00012	6E-05	0.06001	-1.88		0.1658	0.06	0.06
Aspergillus_vitricola	<0.0001(<0.0001,<0.0001)	0.000603	0.00026	<0.0001(<0.0001,<0.0001)	0.000514	0.000229	<0.0001(<0.0001,<0.0001)	5.9E-05	5.3E-05	0.55947	-0.58		0.7066	0.5595	0.5595

Symmetrospora_oryzicola	<0.0001(<0.0001,<0.0001)	0.000547	0.00053	<0.0001(<0.0001,<0.0001)	0.000631	0.00044	<0.0001(<0.0001,<0.0001)	2.6E-05	1.4E-05	0.34083	-0.95		0.5	0.3408	0.3408
Ceriporia_lacerata	<0.0001(<0.0001,<0.0001)	0.000754	0.00051	<0.0001(<0.0001,<0.0001)	0.000129	9.72E-05	<0.0001(<0.0001,<0.0001)	0	0	0.00107	-3.27	**	0.0102	0.0011	** 0.0011
Entorrhiza_citriformis	<0.0001(<0.0001,<0.0001)	0.000185	6.5E-05	<0.0001(<0.0001,0.0004)	0.001109	0.000352	0.0001(<0.0001,0.0005)	0.00119	0.00069	3.2E-05	-4.16	***	0.0007	<0.0001	***<0.0001
Ustilaginoidea_virens	<0.0001(<0.0001,<0.0001)	1.08E-05	3.7E-06	<0.0001(<0.0001,0.0001)	0.001427	0.000609	<0.0001(<0.0001,0.0002)	0.00145	0.00095	2.9E-07	-5.13	***	2E-05	<0.0001	***<0.0001
Pichia_kluyveri	<0.0001(<0.0001,<0.0001)	2.64E-06	2.6E-06	<0.0001(<0.0001,<0.0001)	0.001215	0.000609	<0.0001(<0.0001,0.0001)	0.0022	0.00162	2.7E-09	-5.95	***	4E-07	<0.0001	***<0.0001
Candida_zeylanoides	<0.0001(<0.0001,<0.0001)	0.000383	0.00022	<0.0001(<0.0001,<0.0001)	0.000313	0.000253	<0.0001(<0.0001,0.0002)	0.0022	0.00201	0.00846	-2.63	**	0.041	0.0085	** 0.0085
Tausonia_pullulans	<0.0001(<0.0001,<0.0001)	0.00063	0.00037	<0.0001(<0.0001,<0.0001)	0.000267	0.000186	<0.0001(<0.0001,<0.0001)	0.00018	0.00016	0.99763	-0		0.9976	0.9976	0.9976
Diaporthe_bohemiae	<0.0001(<0.0001,<0.0001)	0.000762	0.00067	<0.0001(<0.0001,<0.0001)	0	0	<0.0001(<0.0001,<0.0001)	0	0	0.00968	-2.59	**	0.0439	0.0097	** 0.0097
Trichophaea_saccata	<0.0001(<0.0001,<0.0001)	8.84E-06	7E-06	<0.0001(<0.0001,<0.0001)	0.001756	0.001735	<0.0001(<0.0001,<0.0001)	2.6E-05	2.6E-05	0.00411	-2.87	**	0.0238	0.0041	** 0.0041
Parasympodiella_longispora	<0.0001(<0.0001,0.0001)	0.000393	0.00012	<0.0001(<0.0001,0.0003)	0.000382	0.000149	0.0008(<0.0001,0.0017)	0.00177	0.00068	0.00063	-3.42	***	0.0067	0.0006	***0.0006
Dipodascus_geotrichum	<0.0001(<0.0001,<0.0001)	0.000339	0.00018	<0.0001(<0.0001,<0.0001)	0.000927	0.000903	<0.0001(<0.0001,<0.0001)	9.4E-05	6.8E-05	0.01579	-2.41	*	0.0653	0.0158	* 0.0158
Starmerella_etchellsii	<0.0001(<0.0001,<0.0001)	7.76E-05	7.5E-05	<0.0001(<0.0001,<0.0001)	0.0015	0.000895	<0.0001(<0.0001,<0.0001)	5.7E-05	2.7E-05	1.3E-06	-4.85	***	5E-05	<0.0001	***<0.0001
Pyrenochaetopsis_microspora	<0.0001(<0.0001,<0.0001)	0.000716	0.00068	<0.0001(<0.0001,<0.0001)	1.46E-06	1.46E-06	<0.0001(<0.0001,<0.0001)	0	0	0.01278	-2.49	*	0.0549	0.0128	* 0.0128
Rozellomycota_spe_Incertae_sedis	<0.0001(<0.0001,<0.0001)	0.000711	0.00063	<0.0001(<0.0001,<0.0001)	0	0	<0.0001(<0.0001,<0.0001)	0	0	0.00514	-2.8	**	0.0281	0.0051	** 0.0051
Didymella_keratinophila	<0.0001(<0.0001,<0.0001)	0.0005	0.0003	<0.0001(<0.0001,<0.0001)	0.000419	0.000412	<0.0001(<0.0001,<0.0001)	4.1E-05	4.1E-05	0.01881	-2.35	*	0.0708	0.0188	* 0.0188
Sterigmatomyces_halophilus	<0.0001(<0.0001,<0.0001)	0.000361	0.00024	<0.0001(<0.0001,<0.0001)	0.000133	0.000122	<0.0001(<0.0001,<0.0001)	0.00227	0.0015	0.07625	-1.77		0.1953	0.0763	0.0763
Tuber_alcaracense	<0.0001(<0.0001,0.0002)	0.000264	5.5E-05	0.0002(<0.0001,0.001)	0.000768	0.000155	0.0001(<0.0001,0.0004)	0.00074	0.00041	1.3E-05	-4.36	***	0.0003	<0.0001	***<0.0001
Hannaella_pagnoccae	<0.0001(<0.0001,<0.0001)	0.000664	0.00065	<0.0001(<0.0001,<0.0001)	2.57E-06	1.83E-06	<0.0001(<0.0001,<0.0001)	0	0	0.20728	-1.26		0.4037	0.2073	0.2073
Blastobotrys_proliferans	<0.0001(<0.0001,<0.0001)	6.44E-07	6.4E-07	<0.0001(<0.0001,<0.0001)	0.001552	0.001545	<0.0001(<0.0001,<0.0001)	6.7E-06	6.7E-06	0.09692	-1.66		0.233	0.0969	0.0969
Phlebia_tuberculata	<0.0001(<0.0001,<0.0001)	0	0	<0.0001(<0.0001,<0.0001)	0.001524	0.001522	<0.0001(<0.0001,<0.0001)	5.7E-06	5.7E-06	0.04145	-2.04	*	0.1249	0.0415	* 0.0415
Cutaneotrichosporon_cutaneum	<0.0001(<0.0001,<0.0001)	8.05E-06	5.7E-06	<0.0001(<0.0001,<0.0001)	2.2E-05	9.33E-06	<0.0001(<0.0001,0.0002)	0.00554	0.00505	1.2E-06	-4.86	***	5E-05	<0.0001	***<0.0001
Aspergillus_restrictus	<0.0001(<0.0001,<0.0001)	0.000262	0.00014	<0.0001(<0.0001,0.0001)	0.000603	0.000351	<0.0001(<0.0001,<0.0001)	0.00097	0.00079	0.105	-1.62		0.245	0.105	0.105
Debaryomyces_prosopidis	<0.0001(<0.0001,<0.0001)	0.000452	0.00026	<0.0001(<0.0001,<0.0001)	0.000336	0.000332	<0.0001(<0.0001,<0.0001)	0.00022	0.00019	1.1E-05	-4.4	***	0.0003	<0.0001	***<0.0001
Ascomycota_spe_Incertae_sedis	<0.0001(<0.0001,<0.0001)	0.000123	3.2E-05	<0.0001(<0.0001,<0.0001)	0.000785	0.000283	0.0002(<0.0001,0.0016)	0.00132	0.00069	0.00361	-2.91	**	0.0216	0.0036	** 0.0036
Candida_vespimorsuum	<0.0001(<0.0001,<0.0001)	9.24E-05	5.3E-05	<0.0001(<0.0001,<0.0001)	0.000162	7.73E-05	<0.0001(<0.0001,0.0001)	0.00388	0.00216	0.01691	-2.39	*	0.0684	0.0169	* 0.0169
Henningsia_resupinata	<0.0001(<0.0001,<0.0001)	2.05E-07	2E-07	<0.0001(<0.0001,<0.0001)	0.001399	0.001369	<0.0001(<0.0001,0.0001)	5.9E-05	2.3E-05	6.5E-07	-4.98	***	4E-05	<0.0001	***<0.0001
Mycosphaerellaceae_spe_Incertae_sedis	<0.0001(<0.0001,<0.0001)	0.000285	0.00015	<0.0001(<0.0001,<0.0001)	0.000529	0.000232	<0.0001(<0.0001,0.0003)	0.00073	0.0006	0.14424	-1.46		0.3059	0.1442	0.1442
Typhula_micans	<0.0001(<0.0001,<0.0001)	0.00057	0.00038	<0.0001(<0.0001,<0.0001)	0	0	<0.0001(<0.0001,<0.0001)	0	0	0.00013	-3.84	***	0.002	0.0001	***0.0001

[Candida]_pseudolambica	<0.0001(<0.0001,<0.0001)	3.41E-07	3.4E-07	<0.0001(<0.0001,<0.0001)	0.000132	8.22E-05	<0.0001(<0.0001,<0.0001)	0.00444	0.00442	3.3E-05	-4.15	***	0.0007	<0.0001	***<0.0001
Flammulina_filiformis	<0.0001(<0.0001,<0.0001)	0.000555	0.00053	<0.0001(<0.0001,<0.0001)	1.05E-05	8.62E-06	<0.0001(<0.0001,<0.0001)	0	0	0.12262	-1.54		0.2768	0.1226	0.1226
Fusarium_fujikuroi	<0.0001(<0.0001,<0.0001)	0.000402	0.00027	<0.0001(<0.0001,<0.0001)	0.000297	0.000174	<0.0001(<0.0001,<0.0001)	0.00019	0.00016	0.48206	-0.7		0.6362	0.4821	0.4821
Candida_intermedia	<0.0001(<0.0001,<0.0001)	0	0	<0.0001(<0.0001,<0.0001)	0.00128	0.001272	<0.0001(<0.0001,<0.0001)	1.3E-05	1.3E-05	0.00028	-3.64	***	0.0034	0.0003	***0.0003
Glomus_macrocarpum	<0.0001(<0.0001,<0.0001)	0.000278	0.00027	<0.0001(<0.0001,<0.0001)	0.000612	0.000608	<0.0001(<0.0001,<0.0001)	4.6E-05	2.6E-05	0.03374	-2.12	*	0.1129	0.0337	* 0.0337
Auricularia_auricula-judae	<0.0001(<0.0001,<0.0001)	0.000322	0.00025	<0.0001(<0.0001,<0.0001)	0.000493	0.000432	<0.0001(<0.0001,<0.0001)	0	0	0.28885	-1.06		0.4591	0.2889	0.2889
Talaromyces_calidicanius	<0.0001(<0.0001,<0.0001)	0.000526	0.0005	<0.0001(<0.0001,<0.0001)	1.29E-06	1.29E-06	<0.0001(<0.0001,<0.0001)	0	0	0.12843	-1.52		0.2861	0.1284	0.1284
Clavispora_lusitaniae	<0.0001(<0.0001,<0.0001)	0.000522	0.00048	<0.0001(<0.0001,<0.0001)	2.88E-06	2.08E-06	<0.0001(<0.0001,<0.0001)	0	0	0.27538	-1.09		0.4398	0.2754	0.2754
Naganishia_globosa	<0.0001(<0.0001,<0.0001)	0.000514	0.00043	<0.0001(<0.0001,<0.0001)	1.16E-05	1.16E-05	<0.0001(<0.0001,<0.0001)	0	0	0.00297	-2.97	**	0.0187	0.003	** 0.003
Bjerkandera_adusta	<0.0001(<0.0001,<0.0001)	0.000111	7.9E-05	<0.0001(<0.0001,<0.0001)	0.000666	0.000455	<0.0001(<0.0001,<0.0001)	0.00107	0.00107	0.52948	-0.63		0.6901	0.5295	0.5295
Wallemia_sebi	<0.0001(<0.0001,<0.0001)	0.000306	0.00021	<0.0001(<0.0001,<0.0001)	9.05E-06	8.13E-06	<0.0001(<0.0001,<0.0001)	0.00177	0.00174	0.03784	-2.08	*	0.1206	0.0378	* 0.0378
Austropleospora_keteleeriae	<0.0001(<0.0001,<0.0001)	0.000481	0.00046	<0.0001(<0.0001,<0.0001)	6.65E-05	6.54E-05	<0.0001(<0.0001,<0.0001)	0	0	0.27538	-1.09		0.4398	0.2754	0.2754
Symmetrospora_spe_Incertae_sedis	<0.0001(<0.0001,<0.0001)	6.82E-06	6.3E-06	<0.0001(<0.0001,<0.0001)	0.00118	0.001174	<0.0001(<0.0001,<0.0001)	0	0	0.00953	-2.59	**	0.0439	0.0095	** 0.0095
Aspergillus_tennesseensis	<0.0001(<0.0001,<0.0001)	0.000206	0.00014	<0.0001(<0.0001,<0.0001)	0.000668	0.000429	<0.0001(<0.0001,0.0001)	0.00011	6.5E-05	0.00849	-2.63	**	0.041	0.0085	** 0.0085
Papiliotrema_terrestris	<0.0001(<0.0001,<0.0001)	2.35E-07	2.3E-07	<0.0001(<0.0001,<0.0001)	1.46E-05	7.19E-06	<0.0001(<0.0001,0.0001)	0.00436	0.0043	2.6E-07	-5.15	***	2E-05	<0.0001	***<0.0001
Russulales_spe_Incertae_sedis	<0.0001(<0.0001,<0.0001)	0.000501	0.00049	<0.0001(<0.0001,<0.0001)	0	0	<0.0001(<0.0001,<0.0001)	0	0	0.04464	-2.01	*	0.131	0.0446	* 0.0446
Chytridium_olla	<0.0001(<0.0001,<0.0001)	0.000114	6.1E-05	<0.0001(<0.0001,<0.0001)	0.000847	0.000536	<0.0001(<0.0001,<0.0001)	0.00022	0.00022	0.15645	-1.42		0.3287	0.1565	0.1565
Ahmadea_dalanensis	<0.0001(<0.0001,<0.0001)	0.00049	0.00048	<0.0001(<0.0001,<0.0001)	1.18E-05	5.17E-06	<0.0001(<0.0001,<0.0001)	6.1E-06	6.1E-06	0.88569	-0.14		0.8897	0.8857	0.8857
Eupenidiella_venezuelensis	<0.0001(<0.0001,<0.0001)	0.000496	0.00047	<0.0001(<0.0001,<0.0001)	0	0	<0.0001(<0.0001,<0.0001)	0	0	0.018	-2.37	*	0.0708	0.018	* 0.018
Plectosphaerella_cucumerina	<0.0001(<0.0001,<0.0001)	0.000467	0.00045	<0.0001(<0.0001,<0.0001)	1.61E-05	1.48E-05	<0.0001(<0.0001,<0.0001)	0.00015	0.00014	0.13309	-1.5		0.2935	0.1331	0.1331
Sporobolomyces_roseus	<0.0001(<0.0001,<0.0001)	0.000464	0.00037	<0.0001(<0.0001,<0.0001)	0	0	<0.0001(<0.0001,<0.0001)	0	0	0.00194	-3.1	**	0.0136	0.0019	** 0.0019
Recurvomyces_spe_Incertae_sedis	<0.0001(<0.0001,<0.0001)	0.000458	0.00045	<0.0001(<0.0001,<0.0001)	0	0	<0.0001(<0.0001,<0.0001)	7.6E-06	7.6E-06	0.10973	-1.6		0.2486	0.1097	0.1097
Thielaviopsis_euricoi	<0.0001(<0.0001,<0.0001)	0.000453	0.00024	<0.0001(<0.0001,<0.0001)	1.46E-06	1.46E-06	<0.0001(<0.0001,<0.0001)	0	0	0.01226	-2.5	*	0.053	0.0123	* 0.0123
Wallemia_muriae	<0.0001(<0.0001,<0.0001)	0.000451	0.00045	<0.0001(<0.0001,<0.0001)	1.21E-06	1.21E-06	<0.0001(<0.0001,<0.0001)	0	0	0.4795	-0.71		0.6349	0.4795	0.4795
Spiromastix_princeps	<0.0001(<0.0001,<0.0001)	0.00045	0.00044	<0.0001(<0.0001,<0.0001)	0	0	<0.0001(<0.0001,<0.0001)	0	0	0.02444	-2.25	*	0.0861	0.0244	* 0.0244
Coprinopsis_urticicola	<0.0001(<0.0001,<0.0001)	0.000449	0.00031	<0.0001(<0.0001,<0.0001)	0	0	<0.0001(<0.0001,<0.0001)	0	0	0.0014	-3.2	**	0.0119	0.0014	** 0.0014
Aspergillus_whitfieldii	<0.0001(<0.0001,<0.0001)	0.000435	0.00043	<0.0001(<0.0001,<0.0001)	0	0	<0.0001(<0.0001,<0.0001)	4.2E-06	4.2E-06	0.13622	-1.49		0.2994	0.1362	0.1362

Talaromyces_wortmannii	<0.0001(<0.0001,<0.0001)	0.000411	0.00019	<0.0001(<0.0001,<0.0001)	5.21E-05	4.08E-05	<0.0001(<0.0001,<0.0001)	7.3E-06	7.3E-06	0.0599	-1.88		0.1658	0.0599	0.0599
Tilletia_geeringii	<0.0001(<0.0001,<0.0001)	0.000427	0.00029	<0.0001(<0.0001,<0.0001)	0	0	<0.0001(<0.0001,<0.0001)	0	0	0.00025	-3.66	***	0.0033	0.0003	***0.0003
Thecaphora_australiensis	<0.0001(<0.0001,<0.0001)	0.000425	0.00029	<0.0001(<0.0001,<0.0001)	0	0	<0.0001(<0.0001,<0.0001)	8.7E-06	8.7E-06	0.05292	-1.94		0.1546	0.0529	0.0529
Heitmania_litseae	<0.0001(<0.0001,0.0002)	0.000233	5.7E-05	<0.0001(<0.0001,0.0004)	0.000428	0.000222	<0.0001(<0.0001,<0.0001)	6.9E-05	4.9E-05	0.08565	-1.72		0.2097	0.0856	0.0856
Engyodontium_album	<0.0001(<0.0001,<0.0001)	0.00029	0.00024	<0.0001(<0.0001,<0.0001)	0.000291	0.000152	<0.0001(<0.0001,<0.0001)	3E-05	1.8E-05	0.48241	-0.7		0.6362	0.4824	0.4824
Lentinus_arcularius	<0.0001(<0.0001,<0.0001)	0.000412	0.0004	<0.0001(<0.0001,<0.0001)	1.11E-06	1.11E-06	<0.0001(<0.0001,<0.0001)	0	0	0.03027	-2.17	*	0.1056	0.0303	* 0.0303
Rhodotorula_mucilaginosa	<0.0001(<0.0001,<0.0001)	0.000145	4.9E-05	<0.0001(<0.0001,<0.0001)	6.14E-05	2.23E-05	<0.0001(<0.0001,<0.0001)	0.00211	0.00206	0.14744	-1.45		0.3107	0.1474	0.1474
Calosphaeria_pulchella	<0.0001(<0.0001,<0.0001)	0.000236	8.7E-05	<0.0001(<0.0001,0.0005)	0.000358	8.9E-05	<0.0001(<0.0001,<0.0001)	0.0002	0.00011	0.06771	-1.83		0.1804	0.0677	0.0677
Flavodon_spe_Incertae_sedis	<0.0001(<0.0001,<0.0001)	0.000396	0.00037	<0.0001(<0.0001,<0.0001)	4.91E-06	4.91E-06	<0.0001(<0.0001,<0.0001)	4.3E-06	4.3E-06	0.125	-1.53		0.2813	0.125	0.125
Aspergillus_foetidus	<0.0001(<0.0001,<0.0001)	0.000362	0.00027	<0.0001(<0.0001,<0.0001)	8.07E-05	6.38E-05	<0.0001(<0.0001,<0.0001)	0	0	0.49469	-0.68		0.6511	0.4947	0.4947
Psathyrella_candolleana	<0.0001(<0.0001,<0.0001)	0.000155	9.7E-05	<0.0001(<0.0001,<0.0001)	1.23E-06	1.23E-06	<0.0001(<0.0001,<0.0001)	0.00204	0.00204	0.2453	-1.16		0.433	0.2453	0.2453
[Candida]_glabrata	<0.0001(<0.0001,<0.0001)	3.94E-07	3.9E-07	<0.0001(<0.0001,<0.0001)	0.000883	0.000785	<0.0001(<0.0001,<0.0001)	1.9E-06	1.9E-06	1.7E-06	-4.79	***	6E-05	<0.0001	***<0.0001
Raffaelea_albimanens	<0.0001(<0.0001,<0.0001)	0.000218	5.4E-05	<0.0001(<0.0001,<0.0001)	0.000107	6.14E-05	0.0004(<0.0001,0.0014)	0.00094	0.00035	0.00323	-2.95	**	0.0197	0.0032	** 0.0032
Thelebolus_balaustiformis	<0.0001(<0.0001,<0.0001)	0.000362	0.00035	<0.0001(<0.0001,<0.0001)	1.53E-05	1.53E-05	<0.0001(<0.0001,<0.0001)	0	0	0.03474	-2.11	*	0.1157	0.0347	* 0.0347
Rhodotorula_glutinis	<0.0001(<0.0001,<0.0001)	9.67E-05	9.6E-05	<0.0001(<0.0001,<0.0001)	8.25E-06	4.83E-06	<0.0001(<0.0001,<0.0001)	0.00236	0.00232	0.0051	-2.8	**	0.0281	0.0051	** 0.0051
Ophiosphaerella_aquatica	<0.0001(<0.0001,<0.0001)	0.00033	0.00033	<0.0001(<0.0001,<0.0001)	8.34E-05	7.82E-05	<0.0001(<0.0001,<0.0001)	0	0	0.53442	-0.62		0.6952	0.5344	0.5344
Acrocalymma_fici	<0.0001(<0.0001,<0.0001)	0.00036	0.00025	<0.0001(<0.0001,<0.0001)	1.02E-06	1.02E-06	<0.0001(<0.0001,<0.0001)	0	0	0.01209	-2.51	*	0.0526	0.0121	* 0.0121
Vanrija_musci	<0.0001(<0.0001,<0.0001)	1.74E-06	9.2E-07	<0.0001(<0.0001,<0.0001)	3.46E-06	2.03E-06	<0.0001(<0.0001,<0.0001)	0.00305	0.0021	0.1465	-1.45		0.3097	0.1465	0.1465
Coniochaeta_mutabilis	<0.0001(<0.0001,<0.0001)	0.000338	0.00033	<0.0001(<0.0001,<0.0001)	2.48E-05	2.48E-05	<0.0001(<0.0001,<0.0001)	0	0	0.14082	-1.47		0.3059	0.1408	0.1408
Roussoella_neopustulans	<0.0001(<0.0001,<0.0001)	0.000347	0.00033	<0.0001(<0.0001,<0.0001)	0	0	<0.0001(<0.0001,<0.0001)	0	0	0.01322	-2.48	*	0.0557	0.0132	* 0.0132
Pseudocercospora_spe_Incertae_sedis	<0.0001(<0.0001,<0.0001)	0.000346	0.00027	<0.0001(<0.0001,<0.0001)	0	0	<0.0001(<0.0001,<0.0001)	0	0	0.00706	-2.69	**	0.0356	0.0071	** 0.0071
Lecanicillium_coprophilum	<0.0001(<0.0001,<0.0001)	0.000341	0.00026	<0.0001(<0.0001,<0.0001)	0	0	<0.0001(<0.0001,<0.0001)	0	0	0.00968	-2.59	**	0.0439	0.0097	** 0.0097
Bovista_aestivalis	<0.0001(<0.0001,<0.0001)	5.5E-06	2.4E-06	<0.0001(<0.0001,<0.0001)	1.09E-06	1.09E-06	<0.0001(<0.0001,<0.0001)	0.0029	0.0029	0.4337	-0.78		0.5909	0.4337	0.4337
Candida_saitoana	<0.0001(<0.0001,<0.0001)	0.000327	0.00032	<0.0001(<0.0001,<0.0001)	2.16E-06	2.16E-06	<0.0001(<0.0001,<0.0001)	3.7E-06	3.7E-06	0.43797	-0.78		0.5909	0.438	0.438
Lentinula_edodes	<0.0001(<0.0001,<0.0001)	0.000128	9.6E-05	<0.0001(<0.0001,<0.0001)	0.000463	0.000457	<0.0001(<0.0001,<0.0001)	0	0	0.42849	-0.79		0.5909	0.4285	0.4285
Candida_solani	<0.0001(<0.0001,<0.0001)	0.000169	4.1E-05	<0.0001(<0.0001,<0.0001)	0.000359	0.000346	<0.0001(<0.0001,<0.0001)	1.3E-05	9.3E-06	0.00156	-3.16	**	0.0128	0.0016	** 0.0016
Acremonium_charticola	<0.0001(<0.0001,<0.0001)	0.000322	0.00032	<0.0001(<0.0001,<0.0001)	7.86E-07	7.86E-07	<0.0001(<0.0001,<0.0001)	0	0	0.82925	-0.22		0.8471	0.8293	0.8293
Pseudoramichloridium_henryi	<0.0001(<0.0001,<0.0001)	0.000318	0.00031	<0.0001(<0.0001,<0.0001)	0	0	<0.0001(<0.0001,<0.0001)	1E-05	7.1E-06	0.06193	-1.87		0.1697	0.0619	0.0619

Diaporthe_caulivora	<0.0001(<0.0001,<0.0001)	0.000317	0.00017	<0.0001(<0.0001,<0.0001)	0	0	<0.0001(<0.0001,<0.0001)	0	0	0.00071	-3.38	***	0.0073	0.0007	***0.0007
Knufia_epidermidis	<0.0001(<0.0001,<0.0001)	0.000312	0.00027	<0.0001(<0.0001,<0.0001)	0	0	<0.0001(<0.0001,<0.0001)	0	0	0.08058	-1.75		0.1995	0.0806	0.0806
Cutaneotrichosporon_jirovecii	<0.0001(<0.0001,<0.0001)	0.000308	0.00025	<0.0001(<0.0001,<0.0001)	7.71E-06	7.71E-06	<0.0001(<0.0001,<0.0001)	0	0	0.00551	-2.78	**	0.0296	0.0055	** 0.0055
Stagonosporopsis_lupini	<0.0001(<0.0001,<0.0001)	0.000312	0.0003	<0.0001(<0.0001,<0.0001)	0	0	<0.0001(<0.0001,<0.0001)	0	0	0.00706	-2.69	**	0.0356	0.0071	** 0.0071
Zeloasperisporium_eucalyptorum	<0.0001(<0.0001,<0.0001)	0	0	<0.0001(<0.0001,<0.0001)	6E-05	4.87E-05	<0.0001(<0.0001,<0.0001)	0.0025	0.00244	2.6E-05	-4.2	***	0.0006	<0.0001	***<0.0001
Keissleriella_quadrisepta	<0.0001(<0.0001,<0.0001)	0.000308	0.0003	<0.0001(<0.0001,<0.0001)	0	0	<0.0001(<0.0001,<0.0001)	0	0	0.01322	-2.48	*	0.0557	0.0132	* 0.0132
Hortaea_werneckii	<0.0001(<0.0001,<0.0001)	0	0	<0.0001(<0.0001,<0.0001)	0.000683	0.000672	<0.0001(<0.0001,<0.0001)	0.00013	0.00013	0.00028	-3.64	***	0.0034	0.0003	***0.0003
Candida_apicola	<0.0001(<0.0001,<0.0001)	5.15E-07	5.2E-07	<0.0001(<0.0001,<0.0001)	1.41E-05	5.1E-06	<0.0001(<0.0001,<0.0001)	0.0026	0.00238	8.4E-06	-4.45	***	0.0002	<0.0001	***<0.0001
Capnodiales_spe_Incertae_sedis	<0.0001(<0.0001,<0.0001)	0.000272	0.00017	<0.0001(<0.0001,<0.0001)	6.03E-05	5.82E-05	<0.0001(<0.0001,<0.0001)	0	0	0.28163	-1.08		0.4487	0.2816	0.2816
Dendryphiella_paravinosa	<0.0001(<0.0001,<0.0001)	0	0	<0.0001(<0.0001,<0.0001)	1.1E-05	6.01E-06	<0.0001(<0.0001,<0.0001)	0.00255	0.00255	0.00144	-3.19	**	0.0121	0.0014	** 0.0014
Bradymyces_alpinus	<0.0001(<0.0001,<0.0001)	0.000292	0.00025	<0.0001(<0.0001,<0.0001)	9.46E-07	9.46E-07	<0.0001(<0.0001,<0.0001)	0	0	0.12843	-1.52		0.2861	0.1284	0.1284
Pseudomicrostroma_phylloplanum	<0.0001(<0.0001,<0.0001)	1.74E-05	1.6E-05	<0.0001(<0.0001,<0.0001)	0.000627	0.000453	<0.0001(<0.0001,<0.0001)	5.1E-06	5.1E-06	0.55836	-0.59		0.7066	0.5584	0.5584
Penicillium_pimiteouiense	<0.0001(<0.0001,<0.0001)	0.000283	0.00024	<0.0001(<0.0001,<0.0001)	0	0	<0.0001(<0.0001,<0.0001)	5.1E-06	5.1E-06	0.06772	-1.83		0.1804	0.0677	0.0677
Dothidea_sambuci	<0.0001(<0.0001,<0.0001)	0.000281	0.00027	<0.0001(<0.0001,<0.0001)	0	0	<0.0001(<0.0001,<0.0001)	0	0	0.03308	-2.13	*	0.1113	0.0331	* 0.0331
Rhodosporidiobolus_colostri	<0.0001(<0.0001,<0.0001)	0	0	<0.0001(<0.0001,<0.0001)	1.08E-05	5.37E-06	<0.0001(<0.0001,<0.0001)	0.00243	0.0024	5.6E-05	-4.03	***	0.001	<0.0001	***<0.0001
Phaeosphaeria_acaciae	<0.0001(<0.0001,<0.0001)	0.000238	0.00023	<0.0001(<0.0001,<0.0001)	8.74E-05	8.28E-05	<0.0001(<0.0001,<0.0001)	8.2E-06	8.2E-06	0.93826	-0.08		0.9397	0.9383	0.9383
Agaricus_subfloccosus	<0.0001(<0.0001,<0.0001)	0	0	<0.0001(<0.0001,<0.0001)	0.000638	0.000627	<0.0001(<0.0001,<0.0001)	1.3E-05	1.3E-05	5.6E-06	-4.54	***	0.0002	<0.0001	***<0.0001
Cordyceps_succavus	<0.0001(<0.0001,<0.0001)	0.000265	0.00025	<0.0001(<0.0001,<0.0001)	5.54E-06	4.68E-06	<0.0001(<0.0001,<0.0001)	0	0	0.34579	-0.94		0.5061	0.3458	0.3458
Ciliophora_spe_Incertae_sedis	<0.0001(<0.0001,<0.0001)	0.000252	0.00012	<0.0001(<0.0001,<0.0001)	3.61E-06	2.21E-06	<0.0001(<0.0001,<0.0001)	0.00011	0.0001	0.06666	-1.83		0.179	0.0667	0.0667
Nigrospora_spe_Incertae_sedis	<0.0001(<0.0001,<0.0001)	0.000259	0.00025	<0.0001(<0.0001,<0.0001)	0	0	<0.0001(<0.0001,<0.0001)	0	0	0.02444	-2.25	*	0.0861	0.0244	* 0.0244
Peniophorella_spe_Incertae_sedis	<0.0001(<0.0001,<0.0001)	0.000257	0.00024	<0.0001(<0.0001,<0.0001)	0	0	<0.0001(<0.0001,<0.0001)	0	0	0.00514	-2.8	**	0.0281	0.0051	** 0.0051
Ascotricha_guamensis	<0.0001(<0.0001,<0.0001)	0	0	<0.0001(<0.0001,<0.0001)	0.0006	0.000592	<0.0001(<0.0001,<0.0001)	0	0	0.01875	-2.35	*	0.0708	0.0187	* 0.0187
Diutina_catenulata	<0.0001(<0.0001,<0.0001)	2.68E-06	1.3E-06	<0.0001(<0.0001,<0.0001)	0.000363	0.000358	<0.0001(<0.0001,<0.0001)	0.00084	0.00083	0.03266	-2.14	*	0.1113	0.0327	* 0.0327
Tilletia_maclaganii	<0.0001(<0.0001,<0.0001)	0.00025	0.00025	<0.0001(<0.0001,<0.0001)	0	0	<0.0001(<0.0001,<0.0001)	0	0	0.06006	-1.88		0.1658	0.0601	0.0601
Aureobasidium_thailandense	<0.0001(<0.0001,<0.0001)	0.000247	0.00024	<0.0001(<0.0001,<0.0001)	0	0	<0.0001(<0.0001,<0.0001)	0	0	0.08058	-1.75		0.1995	0.0806	0.0806
Ramophialophora_spe_Incertae_sedis	<0.0001(<0.0001,<0.0001)	0.000245	0.00023	<0.0001(<0.0001,<0.0001)	0	0	<0.0001(<0.0001,<0.0001)	0	0	0.06006	-1.88		0.1658	0.0601	0.0601
Talaromyces_islandicus	<0.0001(<0.0001,<0.0001)	0.000241	0.00024	<0.0001(<0.0001,<0.0001)	0	0	<0.0001(<0.0001,<0.0001)	0	0	0.03308	-2.13	*	0.1113	0.0331	* 0.0331

Phyllachora_cynodonticola	<0.0001(<0.0001,<0.0001)	0.000199	0.00012	<0.0001(<0.0001,<0.0001)	9.28E-05	3.04E-05	<0.0001(<0.0001,<0.0001)	1.9E-05	1E-05	0.38992	-0.86		0.5585	0.3899	0.3899
Genea_brunneocarpa	<0.0001(<0.0001,<0.0001)	0.000228	9.2E-05	<0.0001(<0.0001,<0.0001)	2.39E-05	1.16E-05	<0.0001(<0.0001,<0.0001)	0	0	0.00129	-3.22	**	0.0111	0.0013	** 0.0013
Talaromyces_rogersiae	<0.0001(<0.0001,<0.0001)	0.000237	0.00023	<0.0001(<0.0001,<0.0001)	0	0	<0.0001(<0.0001,<0.0001)	0	0	0.04464	-2.01	*	0.131	0.0446	* 0.0446
Septoria_spe_Incertae_sedis	<0.0001(<0.0001,<0.0001)	0.000227	0.0002	<0.0001(<0.0001,<0.0001)	2.03E-05	1.71E-05	<0.0001(<0.0001,<0.0001)	0	0	0.60641	-0.52		0.729	0.6064	0.6064
Bulleromyces_albus	<0.0001(<0.0001,<0.0001)	3.7E-05	3.5E-05	<0.0001(<0.0001,<0.0001)	0.000462	0.000355	<0.0001(<0.0001,<0.0001)	0	0	0.06341	-1.86		0.1727	0.0634	0.0634
Leptospora_spe_Incertae_sedis	<0.0001(<0.0001,<0.0001)	0.000205	0.00016	<0.0001(<0.0001,<0.0001)	5.97E-05	5.87E-05	<0.0001(<0.0001,<0.0001)	0	0	0.17567	-1.35		0.3591	0.1757	0.1757
Aspergillus_coremiiformis	<0.0001(<0.0001,<0.0001)	0.000226	0.00015	<0.0001(<0.0001,<0.0001)	3.23E-06	2.32E-06	<0.0001(<0.0001,<0.0001)	2.7E-05	2.7E-05	0.27346	-1.1		0.4389	0.2735	0.2735
Ascotricha_erinacea	<0.0001(<0.0001,<0.0001)	0.000212	0.00013	<0.0001(<0.0001,<0.0001)	0.000044	0.000044	<0.0001(<0.0001,<0.0001)	0	0	0.0101	-2.57	*	0.0448	0.0101	* 0.0101
Candida_ethanolica	<0.0001(<0.0001,<0.0001)	2.8E-07	2.8E-07	<0.0001(<0.0001,<0.0001)	3.34E-06	2.34E-06	<0.0001(<0.0001,<0.0001)	0.00197	0.00196	0.00864	-2.63	**	0.0414	0.0086	** 0.0086
Botryosphaeria_wangensis	<0.0001(<0.0001,<0.0001)	0.000225	0.0002	<0.0001(<0.0001,<0.0001)	0	0	<0.0001(<0.0001,<0.0001)	0	0	0.03308	-2.13	*	0.1113	0.0331	* 0.0331
Stachybotrys_aloeticola	<0.0001(<0.0001,<0.0001)	0	0	<0.0001(<0.0001,<0.0001)	8.32E-06	5.13E-06	<0.0001(<0.0001,<0.0001)	0.00194	0.00187	8.9E-07	-4.91	***	5E-05	<0.0001	***<0.0001
Macrohyporia_dictyopora	<0.0001(<0.0001,<0.0001)	0.000217	0.00021	<0.0001(<0.0001,<0.0001)	0	0	<0.0001(<0.0001,<0.0001)	0	0	0.02444	-2.25	*	0.0861	0.0244	* 0.0244
Carlosrosaea_hohenbergiae	<0.0001(<0.0001,<0.0001)	0.000214	0.00021	<0.0001(<0.0001,<0.0001)	0	0	<0.0001(<0.0001,<0.0001)	0	0	0.10779	-1.61		0.245	0.1078	0.1078
Epicoccum_dendrobii	<0.0001(<0.0001,<0.0001)	3.06E-05	2.3E-05	<0.0001(<0.0001,<0.0001)	0.000399	0.000395	<0.0001(<0.0001,<0.0001)	0.00011	6.1E-05	0.00655	-2.72	**	0.0341	0.0065	** 0.0065
Penicillium_bialowiezense	<0.0001(<0.0001,<0.0001)	0	0	<0.0001(<0.0001,<0.0001)	0.000498	0.000489	<0.0001(<0.0001,<0.0001)	5.3E-06	5.3E-06	0.00295	-2.97	**	0.0187	0.0029	** 0.0029
Yamadazyma_terventina	<0.0001(<0.0001,<0.0001)	0	0	<0.0001(<0.0001,<0.0001)	0.000496	0.000496	<0.0001(<0.0001,<0.0001)	0	0	0.26915	-1.11		0.433	0.2691	0.2691
Neosetophoma_samararum	<0.0001(<0.0001,<0.0001)	0.000193	0.00011	<0.0001(<0.0001,<0.0001)	6.75E-06	4.08E-06	<0.0001(<0.0001,<0.0001)	0.00013	9E-05	0.54243	-0.61		0.7032	0.5424	0.5424
Neoconiothyrium_viticola	<0.0001(<0.0001,<0.0001)	0.000208	0.0002	<0.0001(<0.0001,<0.0001)	0	0	<0.0001(<0.0001,<0.0001)	0	0	0.06006	-1.88		0.1658	0.0601	0.0601
Sistotrema_brinkmannii	<0.0001(<0.0001,<0.0001)	1.93E-06	1.9E-06	<0.0001(<0.0001,<0.0001)	0.000481	0.000455	<0.0001(<0.0001,<0.0001)	1.2E-05	1.2E-05	0.00168	-3.14	**	0.0135	0.0017	** 0.0017
Pestalotiopsis_dracontomelon	<0.0001(<0.0001,<0.0001)	0.000203	0.0002	<0.0001(<0.0001,<0.0001)	0	0	<0.0001(<0.0001,<0.0001)	0	0	0.3356	-0.96		0.4934	0.3356	0.3356
Knufia_hypolithi	<0.0001(<0.0001,<0.0001)	0.000187	0.00017	<0.0001(<0.0001,<0.0001)	0	0	<0.0001(<0.0001,<0.0001)	0.00013	0.00013	0.03178	-2.15	*	0.1102	0.0318	* 0.0318
Acremonium_acutatum	<0.0001(<0.0001,<0.0001)	6.35E-05	6.3E-05	<0.0001(<0.0001,<0.0001)	0.000319	0.000318	<0.0001(<0.0001,<0.0001)	7.2E-06	7.2E-06	0.38857	-0.86		0.5577	0.3886	0.3886
Peroneutypa_scoparia	<0.0001(<0.0001,<0.0001)	2.5E-07	2.5E-07	<0.0001(<0.0001,<0.0001)	0.000459	0.000458	<0.0001(<0.0001,<0.0001)	0	0	0.30009	-1.04		0.467	0.3001	0.3001
Parastagonosporella_fallopiae	<0.0001(<0.0001,<0.0001)	0.000194	0.00018	<0.0001(<0.0001,<0.0001)	0	0	<0.0001(<0.0001,<0.0001)	0	0	0.018	-2.37	*	0.0708	0.018	* 0.018
Helicoma_brunneisporum	<0.0001(<0.0001,<0.0001)	0.000193	0.00019	<0.0001(<0.0001,<0.0001)	0	0	<0.0001(<0.0001,<0.0001)	0	0	0.14379	-1.46		0.3059	0.1438	0.1438
Aspergillus_flavus	<0.0001(<0.0001,<0.0001)	9.52E-05	7.4E-05	<0.0001(<0.0001,<0.0001)	0.000208	0.000108	<0.0001(<0.0001,<0.0001)	7.8E-05	5.5E-05	0.32933	-0.98		0.4934	0.3293	0.3293
Fusarium_neocosmosporiellum	<0.0001(<0.0001,<0.0001)	0.000189	0.00018	<0.0001(<0.0001,<0.0001)	0	0	<0.0001(<0.0001,<0.0001)	0	0	0.00706	-2.69	**	0.0356	0.0071	** 0.0071
Toxicocladosporium_strelitziae	<0.0001(<0.0001,<0.0001)	0.000189	0.00019	<0.0001(<0.0001,<0.0001)	0	0	<0.0001(<0.0001,<0.0001)	0	0	0.08057	-1.75		0.1995	0.0806	0.0806

Vishniacozyma_heimaeyensis	<0.0001(<0.0001,<0.0001)	0.000186	0.00018	<0.0001(<0.0001,<0.0001)	0	0	<0.0001(<0.0001,<0.0001)	0	0	0.02444	-2.25	*	0.0861	0.0244	* 0.0244
Sporobolomyces_patagonicus	<0.0001(<0.0001,<0.0001)	0.00017	0.00012	<0.0001(<0.0001,<0.0001)	3.74E-05	2.8E-05	<0.0001(<0.0001,<0.0001)	0	0	0.31611	-1		0.4885	0.3161	0.3161
Hypoxylon_perforatum	<0.0001(<0.0001,<0.0001)	0.000185	0.00017	<0.0001(<0.0001,<0.0001)	0	0	<0.0001(<0.0001,<0.0001)	0	0	0.01322	-2.48	*	0.0557	0.0132	* 0.0132
Chaetomium_globosum	<0.0001(<0.0001,<0.0001)	0.00018	0.00018	<0.0001(<0.0001,<0.0001)	1.04E-05	1.04E-05	<0.0001(<0.0001,<0.0001)	0	0	0.38796	-0.86		0.5577	0.388	0.388
Aspergillus_baarnensis	<0.0001(<0.0001,<0.0001)	0.000101	7.7E-05	<0.0001(<0.0001,<0.0001)	1.54E-05	1.38E-05	<0.0001(<0.0001,<0.0001)	0.00067	0.00064	0.10026	-1.64		0.2385	0.1003	0.1003
Halosphaeriaceae_spe_Incertae_sedis	<0.0001(<0.0001,<0.0001)	0.000181	0.00011	<0.0001(<0.0001,<0.0001)	0	0	<0.0001(<0.0001,<0.0001)	0	0	0.00968	-2.59	**	0.0439	0.0097	** 0.0097
Valsaria_ceratoniae	<0.0001(<0.0001,<0.0001)	2.66E-05	2.5E-05	<0.0001(<0.0001,<0.0001)	0.000358	0.000251	<0.0001(<0.0001,<0.0001)	0	0	0.52684	-0.63		0.6893	0.5268	0.5268
Periconia_byssoides	<0.0001(<0.0001,<0.0001)	0.000173	0.00017	<0.0001(<0.0001,<0.0001)	1.46E-06	1.46E-06	<0.0001(<0.0001,<0.0001)	0	0	0.37758	-0.88		0.5479	0.3776	0.3776
Malassezia_japonica	<0.0001(<0.0001,<0.0001)	0.000173	0.00013	<0.0001(<0.0001,<0.0001)	0	0	<0.0001(<0.0001,<0.0001)	0	0	0.06006	-1.88		0.1658	0.0601	0.0601
Trichoderma_deliquescens	<0.0001(<0.0001,<0.0001)	0.000172	0.00017	<0.0001(<0.0001,<0.0001)	0	0	<0.0001(<0.0001,<0.0001)	0	0	0.08057	-1.75		0.1995	0.0806	0.0806
Stemphylium_lycopersici	<0.0001(<0.0001,<0.0001)	0.000167	0.00011	<0.0001(<0.0001,<0.0001)	0	0	<0.0001(<0.0001,<0.0001)	0	0	0.02444	-2.25	*	0.0861	0.0244	* 0.0244
Diaporthe_ganjae	<0.0001(<0.0001,<0.0001)	0.000164	0.00015	<0.0001(<0.0001,<0.0001)	0	0	<0.0001(<0.0001,<0.0001)	0	0	0.3356	-0.96		0.4934	0.3356	0.3356
Pyrenochaetopsis_leptospora	<0.0001(<0.0001,<0.0001)	0.000139	0.00014	<0.0001(<0.0001,<0.0001)	5.3E-05	5.2E-05	<0.0001(<0.0001,<0.0001)	1.4E-05	1.4E-05	0.86371	-0.17		0.8782	0.8637	0.8637
Trichomerium_flexuosum	<0.0001(<0.0001,<0.0001)	0.000163	0.00011	<0.0001(<0.0001,<0.0001)	0	0	<0.0001(<0.0001,<0.0001)	0	0	0.10779	-1.61		0.245	0.1078	0.1078
Auricularia_delicata	<0.0001(<0.0001,<0.0001)	0.000163	0.00016	<0.0001(<0.0001,<0.0001)	0	0	<0.0001(<0.0001,<0.0001)	0	0	0.10779	-1.61		0.245	0.1078	0.1078
Rachicladosporium_spe_Incertae_sedis	<0.0001(<0.0001,<0.0001)	0.00016	0.00016	<0.0001(<0.0001,<0.0001)	0	0	<0.0001(<0.0001,<0.0001)	0	0	0.10779	-1.61		0.245	0.1078	0.1078
Basidiomycota_spe_Incertae_sedis	<0.0001(<0.0001,<0.0001)	0.000157	0.0001	<0.0001(<0.0001,<0.0001)	2.75E-06	2.75E-06	<0.0001(<0.0001,<0.0001)	8.9E-06	8.9E-06	0.03826	-2.07	*	0.1213	0.0383	* 0.0383
Laetisaria_lichenicola	<0.0001(<0.0001,<0.0001)	0.000157	0.00016	<0.0001(<0.0001,<0.0001)	0	0	<0.0001(<0.0001,<0.0001)	0	0	0.2537	-1.14		0.433	0.2537	0.2537
Tulostoma_calcareum	<0.0001(<0.0001,<0.0001)	0.000157	0.00015	<0.0001(<0.0001,<0.0001)	0	0	<0.0001(<0.0001,<0.0001)	0	0	0.08057	-1.75		0.1995	0.0806	0.0806
Peniophora_cinerea	<0.0001(<0.0001,<0.0001)	9.73E-05	8.8E-05	<0.0001(<0.0001,<0.0001)	0.000135	0.000103	<0.0001(<0.0001,<0.0001)	5.6E-06	5.6E-06	0.52818	-0.63		0.6897	0.5282	0.5282
Thermomyces_lanuginosus	<0.0001(<0.0001,<0.0001)	0.000118	0.00012	<0.0001(<0.0001,<0.0001)	4.41E-05	4.41E-05	<0.0001(<0.0001,<0.0001)	0.00016	0.00016	0.52297	-0.64		0.687	0.523	0.523
Alternaria_alstroemeriae	<0.0001(<0.0001,<0.0001)	0.000108	2.6E-05	<0.0001(<0.0001,<0.0001)	8.4E-05	4.15E-05	<0.0001(<0.0001,<0.0001)	6.6E-05	3.4E-05	0.88734	-0.14		0.89	0.8873	0.8873
Penicillium_jiangxiense	<0.0001(<0.0001,<0.0001)	0.00015	0.00011	<0.0001(<0.0001,<0.0001)	0	0	<0.0001(<0.0001,<0.0001)	2.7E-06	2.7E-06	0.16761	-1.38		0.3477	0.1676	0.1676
Monoblepharis_macrandra	<0.0001(<0.0001,<0.0001)	0	0	<0.0001(<0.0001,<0.0001)	0.000351	0.000345	<0.0001(<0.0001,<0.0001)	8.9E-06	8.9E-06	0.00297	-2.97	**	0.0187	0.003	** 0.003
Wickerhamiella_versatilis	<0.0001(<0.0001,<0.0001)	2.32E-05	2E-05	<0.0001(<0.0001,<0.0001)	8.56E-05	6.08E-05	<0.0001(<0.0001,<0.0001)	0.00079	0.00078	0.03951	-2.06	*	0.1237	0.0395	* 0.0395
Neodevriesiaceae_spe_Incertae_sedis	<0.0001(<0.0001,<0.0001)	0.000148	0.00015	<0.0001(<0.0001,<0.0001)	2.39E-06	1.72E-06	<0.0001(<0.0001,<0.0001)	0	0	0.71681	-0.36		0.7866	0.7168	0.7168
Nigrograna_spe_Incertae_sedis	<0.0001(<0.0001,<0.0001)	0.000145	0.00011	<0.0001(<0.0001,<0.0001)	0	0	<0.0001(<0.0001,<0.0001)	0	0	0.04464	-2.01	*	0.131	0.0446	* 0.0446

Penicillium_lanosocoeruleum	<0.0001(<0.0001,<0.0001)	0.000121	0.00011	<0.0001(<0.0001,<0.0001)	4.83E-05	3.55E-05	<0.0001(<0.0001,<0.0001)	7.5E-06	7.5E-06	0.75185	-0.32		0.7866	0.7519	0.7519
Coprinellus_pseudodisseminatus	<0.0001(<0.0001,<0.0001)	0.000141	0.00014	<0.0001(<0.0001,<0.0001)	0	0	<0.0001(<0.0001,<0.0001)	0	0	0.14379	-1.46		0.3059	0.1438	0.1438
Golovinomyces_sonchicola	<0.0001(<0.0001,<0.0001)	0	0	<0.0001(<0.0001,<0.0001)	0.000323	0.000321	<0.0001(<0.0001,<0.0001)	2.6E-05	1.4E-05	3.7E-05	-4.12	***	0.0008	<0.0001	***<0.0001
Ceriporiopsis_fimbriata	<0.0001(<0.0001,<0.0001)	0.000138	9.6E-05	<0.0001(<0.0001,<0.0001)	0	0	<0.0001(<0.0001,<0.0001)	0	0	0.10779	-1.61		0.245	0.1078	0.1078
Didymella_spe_Incertae_sedis	<0.0001(<0.0001,<0.0001)	0.000135	9.7E-05	<0.0001(<0.0001,<0.0001)	0	0	<0.0001(<0.0001,<0.0001)	0	0	0.10779	-1.61		0.245	0.1078	0.1078
Xylocoremium_spe_Incertae_sedis	<0.0001(<0.0001,<0.0001)	0.000134	0.00013	<0.0001(<0.0001,<0.0001)	0	0	<0.0001(<0.0001,<0.0001)	0	0	0.08058	-1.75		0.1995	0.0806	0.0806
Novakomyces_olei	<0.0001(<0.0001,<0.0001)	0.000126	0.0001	<0.0001(<0.0001,<0.0001)	1.48E-05	1.06E-05	<0.0001(<0.0001,<0.0001)	0	0	0.64159	-0.47		0.7685	0.6416	0.6416
Torula_fici	<0.0001(<0.0001,<0.0001)	3.79E-07	3.8E-07	<0.0001(<0.0001,<0.0001)	0.000308	0.000306	<0.0001(<0.0001,<0.0001)	0	0	0.30009	-1.04		0.467	0.3001	0.3001
Penicillium_brevicompactum	<0.0001(<0.0001,<0.0001)	0.000114	0.00011	<0.0001(<0.0001,<0.0001)	2.91E-05	2.91E-05	<0.0001(<0.0001,<0.0001)	3.6E-05	2.9E-05	0.16954	-1.37		0.3507	0.1695	0.1695
Sympodiomycopsis_spe_Incertae_sedis	<0.0001(<0.0001,<0.0001)	0.00013	0.00013	<0.0001(<0.0001,<0.0001)	0	0	<0.0001(<0.0001,<0.0001)	0	0	0.19126	-1.31		0.3758	0.1913	0.1913
Coniosporium_spe_Incertae_sedis	<0.0001(<0.0001,<0.0001)	5.23E-07	5.2E-07	<0.0001(<0.0001,<0.0001)	1.49E-05	1.26E-05	<0.0001(<0.0001,<0.0001)	0.00105	0.00105	0.09584	-1.67		0.2321	0.0958	0.0958
Deconica_spe_Incertae_sedis	<0.0001(<0.0001,<0.0001)	0.000125	0.00012	<0.0001(<0.0001,<0.0001)	1.23E-06	1.23E-06	<0.0001(<0.0001,<0.0001)	0	0	0.29911	-1.04		0.467	0.2991	0.2991
Exobasidium_spe_Incertae_sedis	<0.0001(<0.0001,<0.0001)	0	0	<0.0001(<0.0001,<0.0001)	0.000147	0.000145	<0.0001(<0.0001,<0.0001)	0.00054	0.00054	0.02157	-2.3	*	0.0798	0.0216	* 0.0216
Erythrobasidium_hasegawianum	<0.0001(<0.0001,<0.0001)	4.09E-05	3.9E-05	<0.0001(<0.0001,<0.0001)	0.000196	0.000147	<0.0001(<0.0001,<0.0001)	0	0	0.47248	-0.72		0.6281	0.4725	0.4725
Papiliotrema_flavescens	<0.0001(<0.0001,<0.0001)	0.000123	0.00012	<0.0001(<0.0001,<0.0001)	1.48E-06	1.48E-06	<0.0001(<0.0001,<0.0001)	0	0	0.22777	-1.21		0.433	0.2278	0.2278
Peniophora_pseudoversicolor	<0.0001(<0.0001,<0.0001)	0.000124	0.00012	<0.0001(<0.0001,<0.0001)	0	0	<0.0001(<0.0001,<0.0001)	0	0	0.19126	-1.31		0.3758	0.1913	0.1913
Venturiaceae_spe_Incertae_sedis	<0.0001(<0.0001,<0.0001)	0	0	<0.0001(<0.0001,<0.0001)	0.000286	0.000281	<0.0001(<0.0001,<0.0001)	0	0	0.00124	-3.23	**	0.011	0.0012	** 0.0012
Phaeophleospora_hymenocallidicola	<0.0001(<0.0001,<0.0001)	0.000121	0.00011	<0.0001(<0.0001,<0.0001)	0	0	<0.0001(<0.0001,<0.0001)	0	0	0.10779	-1.61		0.245	0.1078	0.1078
Bergerella_atrofusca	<0.0001(<0.0001,<0.0001)	0.00012	0.00012	<0.0001(<0.0001,<0.0001)	0	0	<0.0001(<0.0001,<0.0001)	0	0	0.06006	-1.88		0.1658	0.0601	0.0601
Tubeufia_tratensis	<0.0001(<0.0001,<0.0001)	0.000118	0.00011	<0.0001(<0.0001,<0.0001)	0	0	<0.0001(<0.0001,<0.0001)	0	0	0.14379	-1.46		0.3059	0.1438	0.1438
Didymocyrtis_brachylaenae	<0.0001(<0.0001,<0.0001)	6.67E-07	6.7E-07	<0.0001(<0.0001,<0.0001)	8.8E-06	3.71E-06	<0.0001(<0.0001,<0.0001)	0.00098	0.00096	0.00027	-3.64	***	0.0034	0.0003	***0.0003
Zygosaccharomyces_rouxii	<0.0001(<0.0001,<0.0001)	0.000116	0.00011	<0.0001(<0.0001,<0.0001)	0	0	<0.0001(<0.0001,<0.0001)	0	0	0.2537	-1.14		0.433	0.2537	0.2537
Sporothrix_nigrograna	<0.0001(<0.0001,<0.0001)	2.8E-07	2.8E-07	<0.0001(<0.0001,<0.0001)	0.000196	8.24E-05	<0.0001(<0.0001,<0.0001)	0.00027	0.00015	5E-06	-4.56	***	0.0002	<0.0001	***<0.0001
Periconia_cortaderiae	<0.0001(<0.0001,<0.0001)	4.84E-05	2.4E-05	<0.0001(<0.0001,<0.0001)	9.59E-05	2.6E-05	<0.0001(<0.0001,<0.0001)	0.00023	0.00021	0.02405	-2.26	*	0.0861	0.0241	* 0.0241
Preussia_procaviae	<0.0001(<0.0001,<0.0001)	0.000114	0.00011	<0.0001(<0.0001,<0.0001)	0	0	<0.0001(<0.0001,<0.0001)	0	0	0.02444	-2.25	*	0.0861	0.0244	* 0.0244
GS08_spe_Incertae_sedis	<0.0001(<0.0001,<0.0001)	0	0	<0.0001(<0.0001,<0.0001)	0.000267	0.000264	<0.0001(<0.0001,<0.0001)	0	0	0.01875	-2.35	*	0.0708	0.0187	* 0.0187
Neodevriesia_sexualis	<0.0001(<0.0001,<0.0001)	0	0	<0.0001(<0.0001,<0.0001)	0.000263	0.000259	<0.0001(<0.0001,<0.0001)	0	0	0.01875	-2.35	*	0.0708	0.0187	* 0.0187

Gymnopus_variicolor	<0.0001(<0.0001,<0.0001)	0.000112	0.00011	<0.0001(<0.0001,<0.0001)	0	0	<0.0001(<0.0001,<0.0001)	0	0	0.58243	-0.55		0.7066	0.5824	0.5824
Calycina_spe_Incertae_sedis	<0.0001(<0.0001,<0.0001)	0.000111	0.00011	<0.0001(<0.0001,<0.0001)	0	0	<0.0001(<0.0001,<0.0001)	0	0	0.10779	-1.61		0.245	0.1078	0.1078
Cryptomarasmius_aucubae	<0.0001(<0.0001,<0.0001)	0.000111	7.4E-05	<0.0001(<0.0001,<0.0001)	0	0	<0.0001(<0.0001,<0.0001)	0	0	0.03308	-2.13	*	0.1113	0.0331	* 0.0331
Derxomyces_mrakii	<0.0001(<0.0001,<0.0001)	0.00011	0.00011	<0.0001(<0.0001,<0.0001)	0	0	<0.0001(<0.0001,<0.0001)	0	0	0.10779	-1.61		0.245	0.1078	0.1078
Penicillium_ludwigii	<0.0001(<0.0001,<0.0001)	0.000109	4.4E-05	<0.0001(<0.0001,<0.0001)	2.5E-06	1.76E-06	<0.0001(<0.0001,<0.0001)	0	0	0.20018	-1.28		0.3921	0.2002	0.2002
Kazachstania_humilis	<0.0001(<0.0001,<0.0001)	0	0	<0.0001(<0.0001,<0.0001)	2.98E-06	2.11E-06	<0.0001(<0.0001,<0.0001)	0.00095	0.00094	1E-05	-4.41	***	0.0003	<0.0001	***<0.0001
Radulomyces_paumanokensis	<0.0001(<0.0001,<0.0001)	0.000108	0.00011	<0.0001(<0.0001,<0.0001)	0	0	<0.0001(<0.0001,<0.0001)	0	0	0.14378	-1.46		0.3059	0.1438	0.1438
Pyrenochaeta_spe_Incertae_sedis	<0.0001(<0.0001,<0.0001)	0.000107	0.00011	<0.0001(<0.0001,<0.0001)	0	0	<0.0001(<0.0001,<0.0001)	0	0	0.3356	-0.96		0.4934	0.3356	0.3356
Dactylodendron_pinicola	<0.0001(<0.0001,<0.0001)	4.83E-05	4.5E-05	<0.0001(<0.0001,<0.0001)	0.000126	0.00012	<0.0001(<0.0001,<0.0001)	4.5E-05	4.5E-05	0.43936	-0.77		0.5909	0.4394	0.4394
Beauveria_majiangensis	<0.0001(<0.0001,<0.0001)	7.03E-05	2E-05	<0.0001(<0.0001,<0.0001)	4.73E-05	1.89E-05	<0.0001(<0.0001,<0.0001)	0.00014	0.00013	0.54272	-0.61		0.7032	0.5427	0.5427
Issatchenkia_orientalis	<0.0001(<0.0001,<0.0001)	1.08E-06	6.6E-07	<0.0001(<0.0001,<0.0001)	0.000244	0.000222	<0.0001(<0.0001,<0.0001)	1.6E-05	1.1E-05	0.00905	-2.61	**	0.0428	0.0091	** 0.0091
Trichomerium_dioscoreae	<0.0001(<0.0001,<0.0001)	2.7E-06	2.4E-06	<0.0001(<0.0001,<0.0001)	0.00024	0.000179	<0.0001(<0.0001,<0.0001)	1.3E-05	8.9E-06	0.01757	-2.37	*	0.0701	0.0176	* 0.0176
Penicillium_aurantiogriseum	<0.0001(<0.0001,<0.0001)	9.55E-05	8.6E-05	<0.0001(<0.0001,<0.0001)	2.29E-05	1.27E-05	<0.0001(<0.0001,<0.0001)	0	0	0.47865	-0.71		0.6349	0.4787	0.4787
Phyllosticta_capitalensis	<0.0001(<0.0001,<0.0001)	0.000104	9.9E-05	<0.0001(<0.0001,<0.0001)	0	0	<0.0001(<0.0001,<0.0001)	0	0	0.14379	-1.46		0.3059	0.1438	0.1438
Gonatophragmium_spe_Incertae_sedis	<0.0001(<0.0001,<0.0001)	0.000103	9.5E-05	<0.0001(<0.0001,<0.0001)	0	0	<0.0001(<0.0001,<0.0001)	0	0	0.2537	-1.14		0.433	0.2537	0.2537
Zasmidium_spe_Incertae_sedis	<0.0001(<0.0001,<0.0001)	0.000103	9.7E-05	<0.0001(<0.0001,<0.0001)	0	0	<0.0001(<0.0001,<0.0001)	0	0	0.19126	-1.31		0.3758	0.1913	0.1913
Diutina_rugosa	<0.0001(<0.0001,<0.0001)	7.78E-06	7.8E-06	<0.0001(<0.0001,<0.0001)	6.73E-05	6.63E-05	<0.0001(<0.0001,<0.0001)	0.00059	0.00058	0.00959	-2.59	**	0.0439	0.0096	** 0.0096
Antennariella_placitae	<0.0001(<0.0001,<0.0001)	0.000103	0.0001	<0.0001(<0.0001,<0.0001)	0	0	<0.0001(<0.0001,<0.0001)	0	0	0.14379	-1.46		0.3059	0.1438	0.1438
Phanerochaete_spe_Incertae_sedis	<0.0001(<0.0001,<0.0001)	4.47E-07	4.5E-07	<0.0001(<0.0001,<0.0001)	0.000241	0.000235	<0.0001(<0.0001,<0.0001)	0	0	0.00791	-2.66	**	0.0393	0.0079	** 0.0079
Ochrocladosporium_frigidarii	<0.0001(<0.0001,<0.0001)	1.74E-06	9.1E-07	<0.0001(<0.0001,<0.0001)	0	0	<0.0001(<0.0001,<0.0001)	0.00088	0.00088	0.25398	-1.14		0.433	0.254	0.254
Sporobolomyces_phaffii	<0.0001(<0.0001,<0.0001)	1.61E-06	1.2E-06	<0.0001(<0.0001,<0.0001)	0.000233	0.000232	<0.0001(<0.0001,<0.0001)	7.3E-06	7.3E-06	0.39186	-0.86		0.56	0.3919	0.3919
Rhizopus_arrhizus	<0.0001(<0.0001,<0.0001)	9.98E-05	6.7E-05	<0.0001(<0.0001,<0.0001)	2.93E-06	2.93E-06	<0.0001(<0.0001,<0.0001)	0	0	0.17951	-1.34		0.3634	0.1795	0.1795
Pseudogymnoascus_spe_Incertae_sedis	<0.0001(<0.0001,<0.0001)	0.0001	7.2E-05	<0.0001(<0.0001,<0.0001)	1.13E-06	1.13E-06	<0.0001(<0.0001,<0.0001)	0	0	0.37758	-0.88		0.5479	0.3776	0.3776
Sordaria_equicola	<0.0001(<0.0001,<0.0001)	9.94E-05	9.8E-05	<0.0001(<0.0001,<0.0001)	0	0	<0.0001(<0.0001,<0.0001)	0	0	0.3356	-0.96		0.4934	0.3356	0.3356
Neoascochyta_paspali	<0.0001(<0.0001,<0.0001)	9.87E-05	9.5E-05	<0.0001(<0.0001,<0.0001)	0	0	<0.0001(<0.0001,<0.0001)	0	0	0.44271	-0.77		0.5909	0.4427	0.4427
Chalara_hughesii	<0.0001(<0.0001,<0.0001)	9.81E-05	9.7E-05	<0.0001(<0.0001,<0.0001)	0	0	<0.0001(<0.0001,<0.0001)	0	0	0.3356	-0.96		0.4934	0.3356	0.3356
Candida_haemulonis	<0.0001(<0.0001,<0.0001)	9.71E-05	9.6E-05	<0.0001(<0.0001,<0.0001)	0	0	<0.0001(<0.0001,<0.0001)	0	0	0.44271	-0.77		0.5909	0.4427	0.4427

Skeletocutis_nivea	<0.0001(<0.0001,<0.0001)	9.69E-05	9.6E-05	<0.0001(<0.0001,<0.0001)	0	0	<0.0001(<0.0001,<0.0001)	0	0	0.3356	-0.96		0.4934	0.3356	0.3356
Simplicillium_aogashimaense	<0.0001(<0.0001,<0.0001)	9.57E-05	9.4E-05	<0.0001(<0.0001,<0.0001)	0	0	<0.0001(<0.0001,<0.0001)	0	0	0.2537	-1.14		0.433	0.2537	0.2537
Sporobolomyces_koalae	<0.0001(<0.0001,<0.0001)	9.45E-05	8.9E-05	<0.0001(<0.0001,<0.0001)	0	0	<0.0001(<0.0001,<0.0001)	0	0	0.08058	-1.75		0.1995	0.0806	0.0806
Entorrhiza_parvula	<0.0001(<0.0001,<0.0001)	7.25E-05	5E-05	<0.0001(<0.0001,<0.0001)	4.45E-05	1.83E-05	<0.0001(<0.0001,<0.0001)	2.5E-05	1.5E-05	0.04258	-2.03	*	0.1266	0.0426	* 0.0426
Fusarium_ramigenum	<0.0001(<0.0001,<0.0001)	0	0	<0.0001(<0.0001,<0.0001)	0.000208	0.000146	<0.0001(<0.0001,<0.0001)	4.4E-05	3.5E-05	0.00086	-3.33	***	0.0087	0.0009	***0.0009
Cladophialophora_spe_Incertae_sedis	<0.0001(<0.0001,<0.0001)	9.29E-05	9E-05	<0.0001(<0.0001,<0.0001)	0	0	<0.0001(<0.0001,<0.0001)	0	0	0.2537	-1.14		0.433	0.2537	0.2537
Meira_spe_Incertae_sedis	<0.0001(<0.0001,<0.0001)	0	0	<0.0001(<0.0001,<0.0001)	2.18E-06	2.18E-06	<0.0001(<0.0001,<0.0001)	0.0008	0.0008	0.03578	-2.1	*	0.1168	0.0358	* 0.0358
Cytospora_predappioensis	<0.0001(<0.0001,<0.0001)	9.12E-05	8.9E-05	<0.0001(<0.0001,<0.0001)	0	0	<0.0001(<0.0001,<0.0001)	0	0	0.3356	-0.96		0.4934	0.3356	0.3356
Anisomeridium_guangdongensis	<0.0001(<0.0001,<0.0001)	0	0	<0.0001(<0.0001,<0.0001)	0.000205	0.000204	<0.0001(<0.0001,<0.0001)	3.6E-05	3.6E-05	0.04061	-2.05	*	0.1249	0.0406	* 0.0406
Dothidea_eucalypti	<0.0001(<0.0001,<0.0001)	0.00009	8.9E-05	<0.0001(<0.0001,<0.0001)	0	0	<0.0001(<0.0001,<0.0001)	0	0	0.44271	-0.77		0.5909	0.4427	0.4427
Calyptella_capula	<0.0001(<0.0001,<0.0001)	0.000089	8.5E-05	<0.0001(<0.0001,<0.0001)	0	0	<0.0001(<0.0001,<0.0001)	0	0	0.08058	-1.75		0.1995	0.0806	0.0806
Gibberella_baccata	<0.0001(<0.0001,<0.0001)	4.39E-07	4.4E-07	<0.0001(<0.0001,<0.0001)	2.73E-06	1.97E-06	<0.0001(<0.0001,<0.0001)	0.00076	0.00075	0.00905	-2.61	**	0.0428	0.0091	** 0.0091
Atractiella_rhizophila	<0.0001(<0.0001,<0.0001)	8.75E-05	6.2E-05	<0.0001(<0.0001,<0.0001)	0	0	<0.0001(<0.0001,<0.0001)	0	0	0.14379	-1.46		0.3059	0.1438	0.1438
Blumeria_graminis	<0.0001(<0.0001,<0.0001)	0.000087	8.6E-05	<0.0001(<0.0001,<0.0001)	0	0	<0.0001(<0.0001,<0.0001)	0	0	0.44271	-0.77		0.5909	0.4427	0.4427
Hannaella_surugaensis	<0.0001(<0.0001,<0.0001)	9.77E-06	9.8E-06	<0.0001(<0.0001,<0.0001)	0.00018	0.000143	<0.0001(<0.0001,<0.0001)	0	0	0.00061	-3.43	***	0.0066	0.0006	***0.0006
Jaminaea_angkorensis	<0.0001(<0.0001,<0.0001)	8.52E-05	8.5E-05	<0.0001(<0.0001,<0.0001)	1.02E-06	1.02E-06	<0.0001(<0.0001,<0.0001)	0	0	0.87748	-0.15		0.8843	0.8775	0.8775
Phacidium_lacerum	<0.0001(<0.0001,<0.0001)	0	0	<0.0001(<0.0001,<0.0001)	0.0002	0.000198	<0.0001(<0.0001,<0.0001)	5.1E-06	5.1E-06	0.04145	-2.04	*	0.1249	0.0415	* 0.0415
Acremonium_egyptiacum	<0.0001(<0.0001,<0.0001)	6.06E-05	5.4E-05	<0.0001(<0.0001,<0.0001)	5.33E-05	1.77E-05	<0.0001(<0.0001,<0.0001)	1.8E-05	1.3E-05	0.00629	-2.73	**	0.0333	0.0063	** 0.0063
Hormodochis_melanochlora	<0.0001(<0.0001,<0.0001)	8.36E-05	8E-05	<0.0001(<0.0001,<0.0001)	0	0	<0.0001(<0.0001,<0.0001)	0	0	0.44271	-0.77		0.5909	0.4427	0.4427
Physisporinus_tibeticus	<0.0001(<0.0001,<0.0001)	8.25E-05	8.1E-05	<0.0001(<0.0001,<0.0001)	0	0	<0.0001(<0.0001,<0.0001)	0	0	0.44271	-0.77		0.5909	0.4427	0.4427
Fuscosporella_aquatica	<0.0001(<0.0001,<0.0001)	5.33E-05	1.3E-05	<0.0001(<0.0001,<0.0001)	2.18E-05	1.01E-05	<0.0001(<0.0001,0.0001)	0.00017	9.5E-05	0.08505	-1.72		0.2097	0.0851	0.0851
Phlebiopsis_flavidoalba	<0.0001(<0.0001,<0.0001)	8.15E-05	7.8E-05	<0.0001(<0.0001,<0.0001)	0	0	<0.0001(<0.0001,<0.0001)	0	0	0.08058	-1.75		0.1995	0.0806	0.0806
Yamadazyma_triangularis	<0.0001(<0.0001,<0.0001)	0	0	<0.0001(<0.0001,<0.0001)	1.53E-05	1.11E-05	<0.0001(<0.0001,<0.0001)	0.00065	0.00065	0.03976	-2.06	*	0.1237	0.0398	* 0.0398
Knufia_tsunedae	<0.0001(<0.0001,<0.0001)	7.87E-05	5.2E-05	<0.0001(<0.0001,<0.0001)	0	0	<0.0001(<0.0001,<0.0001)	0	0	0.06006	-1.88		0.1658	0.0601	0.0601
Holtermanniella_takashimae	<0.0001(<0.0001,<0.0001)	1.41E-05	8E-06	<0.0001(<0.0001,<0.0001)	0.000152	0.000142	<0.0001(<0.0001,<0.0001)	0	0	0.16188	-1.4		0.338	0.1619	0.1619
Coprinopsis_clastophylla	<0.0001(<0.0001,<0.0001)	0	0	<0.0001(<0.0001,<0.0001)	1.05E-06	1.05E-06	<0.0001(<0.0001,<0.0001)	0.00068	0.00068	0.03578	-2.1	*	0.1168	0.0358	* 0.0358
Yarrowia_alimentaria	<0.0001(<0.0001,<0.0001)	1.67E-05	1.5E-05	<0.0001(<0.0001,<0.0001)	7.34E-05	7.23E-05	<0.0001(<0.0001,<0.0001)	0.00026	0.00026	0.38365	-0.87		0.5555	0.3837	0.3837

Itersonilia_perplexans	<0.0001(<0.0001,<0.0001)	2.15E-06	1.2E-06	<0.0001(<0.0001,<0.0001)	0.000169	0.000165	<0.0001(<0.0001,<0.0001)	1.7E-05	1.2E-05	0.13303	-1.5		0.2935	0.133	0.133
Neofusicoccum_buxi	<0.0001(<0.0001,<0.0001)	7.53E-05	7.2E-05	<0.0001(<0.0001,<0.0001)	9.29E-07	9.29E-07	<0.0001(<0.0001,<0.0001)	0	0	0.29618	-1.04		0.467	0.2962	0.2962
Banksiophoma_australiensis	<0.0001(<0.0001,<0.0001)	7.47E-05	7.3E-05	<0.0001(<0.0001,<0.0001)	0	0	<0.0001(<0.0001,<0.0001)	4.4E-06	4.4E-06	0.2541	-1.14		0.433	0.2541	0.2541
Podosphaera_cunningtonii	<0.0001(<0.0001,<0.0001)	7.51E-05	7.4E-05	<0.0001(<0.0001,<0.0001)	0	0	<0.0001(<0.0001,<0.0001)	0	0	0.44271	-0.77		0.5909	0.4427	0.4427
Aspergillus_insolitus	<0.0001(<0.0001,<0.0001)	0	0	<0.0001(<0.0001,<0.0001)	0.000172	0.000172	<0.0001(<0.0001,<0.0001)	7.8E-06	7.8E-06	0.03757	-2.08	*	0.1203	0.0376	* 0.0376
Rhopalophora_clavispora	<0.0001(<0.0001,<0.0001)	7.29E-05	7.1E-05	<0.0001(<0.0001,<0.0001)	0	0	<0.0001(<0.0001,<0.0001)	0	0	0.19126	-1.31		0.3758	0.1913	0.1913
Trechispora_spe_Incertae_sedis	<0.0001(<0.0001,<0.0001)	7.21E-05	6.7E-05	<0.0001(<0.0001,<0.0001)	0	0	<0.0001(<0.0001,<0.0001)	0	0	0.3356	-0.96		0.4934	0.3356	0.3356
Elsinoe_leucopogonis	<0.0001(<0.0001,<0.0001)	7.19E-05	7.2E-05	<0.0001(<0.0001,<0.0001)	0	0	<0.0001(<0.0001,<0.0001)	0	0	0.76419	-0.3		0.7866	0.7642	0.7642
Neodevriesia_lagerstroemiae	<0.0001(<0.0001,<0.0001)	2.05E-07	2E-07	<0.0001(<0.0001,<0.0001)	0.000166	0.000165	<0.0001(<0.0001,<0.0001)	8.3E-06	8.3E-06	0.17357	-1.36		0.3576	0.1736	0.1736
Hannaella_oryzae	<0.0001(<0.0001,<0.0001)	0	0	<0.0001(<0.0001,<0.0001)	0.000131	0.000128	<0.0001(<0.0001,<0.0001)	0.00014	0.00014	0.02172	-2.3	*	0.0799	0.0217	* 0.0217
Vishniacozyma_tephrensis	<0.0001(<0.0001,<0.0001)	6.36E-07	6.4E-07	<0.0001(<0.0001,<0.0001)	0.000128	0.000122	<0.0001(<0.0001,<0.0001)	0.00014	0.00014	0.04087	-2.04	*	0.1249	0.0409	* 0.0409
Parasarocladium_aestuarinum	<0.0001(<0.0001,<0.0001)	6.95E-05	6.9E-05	<0.0001(<0.0001,<0.0001)	0	0	<0.0001(<0.0001,<0.0001)	0	0	0.58243	-0.55		0.7066	0.5824	0.5824
Botryosphaeria_corticis	<0.0001(<0.0001,<0.0001)	6.89E-05	6.4E-05	<0.0001(<0.0001,<0.0001)	0	0	<0.0001(<0.0001,<0.0001)	0	0	0.14379	-1.46		0.3059	0.1438	0.1438
Ophiostoma_pusillum	<0.0001(<0.0001,<0.0001)	1.6E-05	6E-06	<0.0001(<0.0001,<0.0001)	0.000102	2.4E-05	<0.0001(<0.0001,<0.0001)	7.9E-05	4.9E-05	0.00053	-3.46	***	0.0059	0.0005	***0.0005
Trichomeriaceae_spe_Incertae_sedis	<0.0001(<0.0001,<0.0001)	0	0	<0.0001(<0.0001,<0.0001)	0.000161	0.000158	<0.0001(<0.0001,<0.0001)	0	0	0.00124	-3.23	**	0.011	0.0012	** 0.0012
Fusicolla_acetilerea	<0.0001(<0.0001,<0.0001)	6.81E-05	6.8E-05	<0.0001(<0.0001,<0.0001)	0	0	<0.0001(<0.0001,<0.0001)	0	0	0.58243	-0.55		0.7066	0.5824	0.5824
Mucor_circinelloides	<0.0001(<0.0001,<0.0001)	6.52E-05	1.5E-05	<0.0001(<0.0001,<0.0001)	4.48E-06	4.48E-06	<0.0001(<0.0001,<0.0001)	8.3E-06	8.3E-06	0.00098	-3.3	***	0.0096	0.001	***0.001
Phlebiopsis_spe_Incertae_sedis	<0.0001(<0.0001,<0.0001)	6.77E-05	6.6E-05	<0.0001(<0.0001,<0.0001)	0	0	<0.0001(<0.0001,<0.0001)	0	0	0.2537	-1.14		0.433	0.2537	0.2537
Subulicystidium_brachysporum	<0.0001(<0.0001,<0.0001)	7.37E-06	6.1E-06	<0.0001(<0.0001,<0.0001)	0.000139	0.000139	<0.0001(<0.0001,<0.0001)	0	0	0.83326	-0.21		0.8485	0.8333	0.8333
Trichoderma_harzianum	<0.0001(<0.0001,<0.0001)	0	0	<0.0001(<0.0001,<0.0001)	0.000145	8.99E-05	<0.0001(<0.0001,<0.0001)	3.9E-05	3.9E-05	0.00295	-2.97	**	0.0187	0.0029	** 0.0029
Penicillium_mallochii	<0.0001(<0.0001,<0.0001)	6.48E-05	6.5E-05	<0.0001(<0.0001,<0.0001)	0	0	<0.0001(<0.0001,<0.0001)	4E-06	4E-06	0.06381	-1.85		0.1727	0.0638	0.0638
Bandonia_marina	<0.0001(<0.0001,<0.0001)	3.84E-05	2E-05	<0.0001(<0.0001,<0.0001)	5.01E-05	3.65E-05	<0.0001(<0.0001,<0.0001)	4.4E-05	4.1E-05	0.18498	-1.33		0.3733	0.185	0.185
Dimorphoma_saxea	<0.0001(<0.0001,<0.0001)	4.93E-05	4E-05	<0.0001(<0.0001,<0.0001)	3.53E-05	3.53E-05	<0.0001(<0.0001,<0.0001)	0	0	0.72286	-0.35		0.7866	0.7229	0.7229
Dactylella_spe_Incertae_sedis	<0.0001(<0.0001,<0.0001)	6.37E-05	6.3E-05	<0.0001(<0.0001,<0.0001)	0	0	<0.0001(<0.0001,<0.0001)	0	0	0.58243	-0.55		0.7066	0.5824	0.5824
Periconia_homothallica	<0.0001(<0.0001,<0.0001)	6.37E-05	5.7E-05	<0.0001(<0.0001,<0.0001)	0	0	<0.0001(<0.0001,<0.0001)	0	0	0.2537	-1.14		0.433	0.2537	0.2537
Agaricales_spe_Incertae_sedis	<0.0001(<0.0001,<0.0001)	0	0	<0.0001(<0.0001,<0.0001)	0.000148	0.000148	<0.0001(<0.0001,<0.0001)	6.7E-06	6.7E-06	0.03757	-2.08	*	0.1203	0.0376	* 0.0376
Gibellula_shennongjiaensis	<0.0001(<0.0001,<0.0001)	5.08E-05	2.1E-05	<0.0001(<0.0001,<0.0001)	2.44E-05	1.01E-05	<0.0001(<0.0001,<0.0001)	7.1E-06	7.1E-06	0.59625	-0.53		0.722	0.5962	0.5962

Eutypella_spe_Incertae_sedis	<0.0001(<0.0001,<0.0001)	6.06E-05	6E-05	<0.0001(<0.0001,<0.0001)	0	0	<0.0001(<0.0001,<0.0001)	0	0	0.44271	-0.77		0.5909	0.4427	0.4427
Penicillium_adametzioides	<0.0001(<0.0001,<0.0001)	0	0	<0.0001(<0.0001,<0.0001)	0.00014	0.00014	<0.0001(<0.0001,<0.0001)	0	0	0.26915	-1.11		0.433	0.2691	0.2691
Paratritirachium_cylindroconium	<0.0001(<0.0001,<0.0001)	4.17E-07	4.2E-07	<0.0001(<0.0001,<0.0001)	0.000137	9.97E-05	<0.0001(<0.0001,<0.0001)	0	0	0.30009	-1.04		0.467	0.3001	0.3001
Penicillium_steckii	<0.0001(<0.0001,<0.0001)	5.69E-05	5.6E-05	<0.0001(<0.0001,<0.0001)	1.55E-06	1.55E-06	<0.0001(<0.0001,<0.0001)	0	0	0.879	-0.15		0.8843	0.879	0.879
Aspergillus_neoglaber	<0.0001(<0.0001,<0.0001)	5.74E-05	3.4E-05	<0.0001(<0.0001,<0.0001)	0	0	<0.0001(<0.0001,<0.0001)	0	0	0.2537	-1.14		0.433	0.2537	0.2537
Zanclospora_aurea	<0.0001(<0.0001,<0.0001)	1.52E-05	8.2E-06	<0.0001(<0.0001,<0.0001)	7.13E-05	2.86E-05	<0.0001(<0.0001,<0.0001)	0.00011	6.8E-05	0.01008	-2.57	*	0.0448	0.0101	* 0.0101
Umthunziomyces_hagahagensis	<0.0001(<0.0001,<0.0001)	3.22E-05	3.1E-05	<0.0001(<0.0001,<0.0001)	5.51E-05	5.42E-05	<0.0001(<0.0001,<0.0001)	8.1E-06	8.1E-06	0.60355	-0.52		0.7282	0.6035	0.6035
Sporobolomyces_beijingensis	<0.0001(<0.0001,<0.0001)	5.6E-05	5.5E-05	<0.0001(<0.0001,<0.0001)	0	0	<0.0001(<0.0001,<0.0001)	0	0	0.44271	-0.77		0.5909	0.4427	0.4427
Fusarium_irregulare	<0.0001(<0.0001,<0.0001)	3.95E-05	1.6E-05	<0.0001(<0.0001,<0.0001)	3.06E-05	2.5E-05	<0.0001(<0.0001,<0.0001)	3E-05	2E-05	0.52444	-0.64		0.6876	0.5244	0.5244
Sarocladium_strictum	<0.0001(<0.0001,<0.0001)	0	0	<0.0001(<0.0001,<0.0001)	0.000119	4.57E-05	<0.0001(<0.0001,<0.0001)	4.5E-05	3.1E-05	5.9E-06	-4.53	***	0.0002	<0.0001	***<0.0001
Nectriopsis_exigua	<0.0001(<0.0001,<0.0001)	5.56E-05	5.3E-05	<0.0001(<0.0001,<0.0001)	0	0	<0.0001(<0.0001,<0.0001)	0	0	0.2537	-1.14		0.433	0.2537	0.2537
Kazachstania_exigua	<0.0001(<0.0001,<0.0001)	5.26E-05	4.9E-05	<0.0001(<0.0001,<0.0001)	0.000007	0.000007	<0.0001(<0.0001,<0.0001)	0	0	0.60138	-0.52		0.7269	0.6014	0.6014
Lyomyces_spe_Incertae_sedis	<0.0001(<0.0001,<0.0001)	5.55E-05	5.1E-05	<0.0001(<0.0001,<0.0001)	0	0	<0.0001(<0.0001,<0.0001)	0	0	0.3356	-0.96		0.4934	0.3356	0.3356
Hanseniaspora_nectarophila	<0.0001(<0.0001,<0.0001)	3.02E-05	2.7E-05	<0.0001(<0.0001,<0.0001)	4.78E-05	4.78E-05	<0.0001(<0.0001,<0.0001)	4.4E-05	3.1E-05	0.17926	-1.34		0.3634	0.1793	0.1793
[Candida]_aaseri	<0.0001(<0.0001,<0.0001)	5.54E-05	5.3E-05	<0.0001(<0.0001,<0.0001)	0	0	<0.0001(<0.0001,<0.0001)	0	0	0.19126	-1.31		0.3758	0.1913	0.1913
Vishniacozyma_taibaiensis	<0.0001(<0.0001,<0.0001)	5.49E-05	4.8E-05	<0.0001(<0.0001,<0.0001)	0	0	<0.0001(<0.0001,<0.0001)	0	0	0.19126	-1.31		0.3758	0.1913	0.1913
Aspergillus_unguis	<0.0001(<0.0001,<0.0001)	6.89E-07	5E-07	<0.0001(<0.0001,<0.0001)	0	0	<0.0001(<0.0001,<0.0001)	0.00047	0.00047	0.15986	-1.41		0.3348	0.1599	0.1599
Ciboria_shiraiana	<0.0001(<0.0001,<0.0001)	5.3E-05	5.3E-05	<0.0001(<0.0001,<0.0001)	0	0	<0.0001(<0.0001,<0.0001)	0	0	0.44271	-0.77		0.5909	0.4427	0.4427
Xeromyces_bisporus	<0.0001(<0.0001,<0.0001)	6.99E-06	7E-06	<0.0001(<0.0001,<0.0001)	0.000108	0.000104	<0.0001(<0.0001,<0.0001)	0	0	0.101	-1.64		0.2385	0.101	0.101
Aspergillus_pragensis	<0.0001(<0.0001,<0.0001)	1.71E-05	1.3E-05	<0.0001(<0.0001,<0.0001)	6.39E-05	6.15E-05	<0.0001(<0.0001,<0.0001)	7.5E-05	7.5E-05	0.44737	-0.76		0.5959	0.4474	0.4474
Erythrobasidium_yunnanense	<0.0001(<0.0001,<0.0001)	0	0	<0.0001(<0.0001,<0.0001)	0.000121	0.000121	<0.0001(<0.0001,<0.0001)	0	0	0.26915	-1.11		0.433	0.2691	0.2691
Aspergillus_conicus	<0.0001(<0.0001,<0.0001)	5.08E-05	5.1E-05	<0.0001(<0.0001,<0.0001)	0	0	<0.0001(<0.0001,<0.0001)	0	0	0.58243	-0.55		0.7066	0.5824	0.5824
Dioszegia_catarinonii	<0.0001(<0.0001,<0.0001)	0	0	<0.0001(<0.0001,<0.0001)	0.00012	0.000105	<0.0001(<0.0001,<0.0001)	0	0	0.01875	-2.35	*	0.0708	0.0187	* 0.0187
Malassezia_dermatis	<0.0001(<0.0001,<0.0001)	2.1E-06	1.8E-06	<0.0001(<0.0001,<0.0001)	0.000114	0.000112	<0.0001(<0.0001,<0.0001)	0	0	0.54847	-0.6		0.7066	0.5485	0.5485
Naganishia_albidosimilis	<0.0001(<0.0001,<0.0001)	5E-05	3.9E-05	<0.0001(<0.0001,<0.0001)	0	0	<0.0001(<0.0001,<0.0001)	0	0	0.19126	-1.31		0.3758	0.1913	0.1913
Coniothyrium_spe_Incertae_sedis	<0.0001(<0.0001,<0.0001)	0	0	<0.0001(<0.0001,<0.0001)	1.34E-06	1.34E-06	<0.0001(<0.0001,<0.0001)	0.00043	0.00043	0.00025	-3.66	***	0.0033	0.0003	***0.0003
Biatriosporaceae_spe_Incertae_sedis	<0.0001(<0.0001,<0.0001)	4.95E-05	4.9E-05	<0.0001(<0.0001,<0.0001)	0	0	<0.0001(<0.0001,<0.0001)	0	0	0.44271	-0.77		0.5909	0.4427	0.4427

Candida_inconspicua	<0.0001(<0.0001,<0.0001)	4.52E-05	9.6E-06	<0.0001(<0.0001,<0.0001)	2.07E-06	2.07E-06	<0.0001(<0.0001,<0.0001)	7E-06	7E-06	0.00171	-3.14	**	0.0136	0.0017	** 0.0017
Tilletia_mactaggartii	<0.0001(<0.0001,<0.0001)	4.67E-05	4.5E-05	<0.0001(<0.0001,<0.0001)	0	0	<0.0001(<0.0001,<0.0001)	0	0	0.44271	-0.77		0.5909	0.4427	0.4427
Vacuiphoma_oculihominis	<0.0001(<0.0001,<0.0001)	2.21E-05	1.6E-05	<0.0001(<0.0001,<0.0001)	3.37E-05	2.55E-05	<0.0001(<0.0001,<0.0001)	8.8E-05	7E-05	0.0975	-1.66		0.2336	0.0975	0.0975
Laburnicola_dactylidis	<0.0001(<0.0001,<0.0001)	4.59E-05	4.4E-05	<0.0001(<0.0001,<0.0001)	0	0	<0.0001(<0.0001,<0.0001)	0	0	0.2537	-1.14		0.433	0.2537	0.2537
Golovinomyces_latisporus	<0.0001(<0.0001,<0.0001)	4.57E-05	4.5E-05	<0.0001(<0.0001,<0.0001)	0	0	<0.0001(<0.0001,<0.0001)	0	0	0.58243	-0.55		0.7066	0.5824	0.5824
Heliocybe_villosa	<0.0001(<0.0001,<0.0001)	4.57E-05	4.5E-05	<0.0001(<0.0001,<0.0001)	0	0	<0.0001(<0.0001,<0.0001)	0	0	0.2537	-1.14		0.433	0.2537	0.2537
Lipomyces_doorenjongii	<0.0001(<0.0001,<0.0001)	4.55E-05	3.8E-05	<0.0001(<0.0001,<0.0001)	0	0	<0.0001(<0.0001,<0.0001)	0	0	0.2537	-1.14		0.433	0.2537	0.2537
Pestalotiopsis_vismiae	<0.0001(<0.0001,<0.0001)	4.47E-05	4.4E-05	<0.0001(<0.0001,<0.0001)	0	0	<0.0001(<0.0001,<0.0001)	0	0	0.44271	-0.77		0.5909	0.4427	0.4427
Steccherinum_ochraceum	<0.0001(<0.0001,<0.0001)	4.41E-05	4.4E-05	<0.0001(<0.0001,<0.0001)	0	0	<0.0001(<0.0001,<0.0001)	0	0	0.58243	-0.55		0.7066	0.5824	0.5824
Colletotrichum_cliviae	<0.0001(<0.0001,<0.0001)	4.37E-05	3.1E-05	<0.0001(<0.0001,<0.0001)	0	0	<0.0001(<0.0001,<0.0001)	0	0	0.3356	-0.96		0.4934	0.3356	0.3356
Didymella_dimorpha	<0.0001(<0.0001,<0.0001)	3.8E-05	2.5E-05	<0.0001(<0.0001,<0.0001)	1.28E-05	1.28E-05	<0.0001(<0.0001,<0.0001)	0	0	0.38449	-0.87		0.5555	0.3845	0.3845
Striatibotrys_yuccae	<0.0001(<0.0001,<0.0001)	3.1E-05	1.5E-05	<0.0001(<0.0001,<0.0001)	2.25E-05	6.68E-06	<0.0001(<0.0001,<0.0001)	2.4E-05	1.9E-05	0.33055	-0.97		0.4934	0.3305	0.3305
Huntiella_tyalla	<0.0001(<0.0001,<0.0001)	4.3E-05	4.3E-05	<0.0001(<0.0001,<0.0001)	0	0	<0.0001(<0.0001,<0.0001)	0	0	0.76419	-0.3		0.7866	0.7642	0.7642
Toxicocladosporium_spe_Incertae_sedis	<0.0001(<0.0001,<0.0001)	4.3E-05	4E-05	<0.0001(<0.0001,<0.0001)	0	0	<0.0001(<0.0001,<0.0001)	0	0	0.58243	-0.55		0.7066	0.5824	0.5824
Fomitopsis_eucalypticola	<0.0001(<0.0001,<0.0001)	4.18E-05	4.1E-05	<0.0001(<0.0001,<0.0001)	0	0	<0.0001(<0.0001,<0.0001)	0	0	0.44271	-0.77		0.5909	0.4427	0.4427
Trichoderma_asperellum	<0.0001(<0.0001,<0.0001)	3.81E-05	3.8E-05	<0.0001(<0.0001,<0.0001)	7.55E-06	7.55E-06	<0.0001(<0.0001,<0.0001)	0	0	0.75049	-0.32		0.7866	0.7505	0.7505
Vandijckella_johannae	<0.0001(<0.0001,<0.0001)	5.38E-07	5.4E-07	<0.0001(<0.0001,<0.0001)	9.42E-05	9.42E-05	<0.0001(<0.0001,<0.0001)	0	0	0.74278	-0.33		0.7866	0.7428	0.7428
Coprinopsis_strossmayeri	<0.0001(<0.0001,<0.0001)	3.93E-05	3.9E-05	<0.0001(<0.0001,<0.0001)	1.21E-06	1.21E-06	<0.0001(<0.0001,<0.0001)	0	0	0.879	-0.15		0.8843	0.879	0.879
Candida_saraburiensis	<0.0001(<0.0001,<0.0001)	3.56E-07	3.6E-07	<0.0001(<0.0001,<0.0001)	7.17E-05	4.27E-05	<0.0001(<0.0001,<0.0001)	7.9E-05	7.9E-05	0.09511	-1.67		0.2312	0.0951	0.0951
Kneiffiella_efibulata	<0.0001(<0.0001,<0.0001)	3.97E-05	3.9E-05	<0.0001(<0.0001,<0.0001)	0	0	<0.0001(<0.0001,<0.0001)	0	0	0.58243	-0.55		0.7066	0.5824	0.5824
Cyphellaceae_spe_Incertae_sedis	<0.0001(<0.0001,<0.0001)	3.92E-05	3.8E-05	<0.0001(<0.0001,<0.0001)	0	0	<0.0001(<0.0001,<0.0001)	0	0	0.3356	-0.96		0.4934	0.3356	0.3356
Ruhlandiella_patagonica	<0.0001(<0.0001,<0.0001)	3.92E-05	3.8E-05	<0.0001(<0.0001,<0.0001)	0	0	<0.0001(<0.0001,<0.0001)	0	0	0.58243	-0.55		0.7066	0.5824	0.5824
Moesziomyces_bullatus	<0.0001(<0.0001,<0.0001)	1.73E-06	1.7E-06	<0.0001(<0.0001,<0.0001)	8.79E-05	5.45E-05	<0.0001(<0.0001,<0.0001)	0	0	0.00813	-2.65	**	0.0398	0.0081	** 0.0081
Peyronellaea_spe_Incertae_sedis	<0.0001(<0.0001,<0.0001)	9.7E-07	9.7E-07	<0.0001(<0.0001,<0.0001)	5.46E-05	3.4E-05	<0.0001(<0.0001,<0.0001)	0.00013	8.8E-05	0.00047	-3.5	***	0.0056	0.0005	***0.0005
Zoophthora_radicans	<0.0001(<0.0001,<0.0001)	3.25E-05	2.1E-05	<0.0001(<0.0001,<0.0001)	1.18E-05	6.52E-06	<0.0001(<0.0001,<0.0001)	0	0	0.32876	-0.98		0.4934	0.3288	0.3288
Botryosphaeria_spe_Incertae_sedis	<0.0001(<0.0001,<0.0001)	0	0	<0.0001(<0.0001,<0.0001)	8.81E-05	8.55E-05	<0.0001(<0.0001,<0.0001)	0	0	0.01875	-2.35	*	0.0708	0.0187	* 0.0187
Ganoderma_australe	<0.0001(<0.0001,<0.0001)	3.69E-05	3.5E-05	<0.0001(<0.0001,<0.0001)	9.46E-07	9.46E-07	<0.0001(<0.0001,<0.0001)	0	0	0.82925	-0.22		0.8471	0.8293	0.8293

Amphobotrys_ricini	<0.0001(<0.0001,<0.0001)	3.5E-05	2.6E-05	<0.0001(<0.0001,<0.0001)	1.52E-06	1.08E-06	<0.0001(<0.0001,<0.0001)	1.3E-05	1.3E-05	0.8128	-0.24		0.8354	0.8128	0.8128
Anthracina_ramosa	<0.0001(<0.0001,<0.0001)	1.77E-05	1.7E-05	<0.0001(<0.0001,<0.0001)	4.58E-05	4.58E-05	<0.0001(<0.0001,<0.0001)	0	0	0.87581	-0.16		0.8843	0.8758	0.8758
Phaeosphaeria_fuckelii	<0.0001(<0.0001,<0.0001)	3.71E-05	3.6E-05	<0.0001(<0.0001,<0.0001)	0	0	<0.0001(<0.0001,<0.0001)	0	0	0.3356	-0.96		0.4934	0.3356	0.3356
Talaromyces_udagawae	<0.0001(<0.0001,<0.0001)	0	0	<0.0001(<0.0001,<0.0001)	0	0	<0.0001(<0.0001,<0.0001)	0.00033	0.00033	0.0019	-3.11	**	0.0136	0.0019	** 0.0019
Crustoderma_spe_Incertae_sedis	<0.0001(<0.0001,<0.0001)	3.58E-05	3.6E-05	<0.0001(<0.0001,<0.0001)	0	0	<0.0001(<0.0001,<0.0001)	0	0	0.76419	-0.3		0.7866	0.7642	0.7642
Penicillifer_martinii	<0.0001(<0.0001,<0.0001)	1.72E-05	1.3E-05	<0.0001(<0.0001,<0.0001)	3.84E-05	1.19E-05	<0.0001(<0.0001,<0.0001)	2.1E-05	1.5E-05	0.00311	-2.96	**	0.0192	0.0031	** 0.0031
Lophiostoma_spe_Incertae_sedis	<0.0001(<0.0001,<0.0001)	3.55E-05	3.4E-05	<0.0001(<0.0001,<0.0001)	0	0	<0.0001(<0.0001,<0.0001)	0	0	0.44271	-0.77		0.5909	0.4427	0.4427
Ceratocystis_mangineca ns	<0.0001(<0.0001,<0.0001)	3.45E-05	3.4E-05	<0.0001(<0.0001,<0.0001)	0	0	<0.0001(<0.0001,<0.0001)	0	0	0.33559	-0.96		0.4934	0.3356	0.3356
Hannaella_kunmingensis	<0.0001(<0.0001,<0.0001)	0	0	<0.0001(<0.0001,<0.0001)	0	0	<0.0001(<0.0001,<0.0001)	0.0003	0.0003	0.0019	-3.11	**	0.0136	0.0019	** 0.0019
Pseudopithomyces_karoo	<0.0001(<0.0001,<0.0001)	0	0	<0.0001(<0.0001,<0.0001)	8.39E-07	8.39E-07	<0.0001(<0.0001,<0.0001)	0.00029	0.00028	0.00025	-3.66	***	0.0033	0.0003	***0.0003
Cystofilobasidium_capitatum	<0.0001(<0.0001,<0.0001)	0	0	<0.0001(<0.0001,<0.0001)	7.91E-05	6.06E-05	<0.0001(<0.0001,<0.0001)	0	0	0.00485	-2.82	**	0.0276	0.0049	** 0.0049
Akanthomyces_thailandicus	<0.0001(<0.0001,<0.0001)	3.29E-05	2E-05	<0.0001(<0.0001,<0.0001)	0	0	<0.0001(<0.0001,<0.0001)	0	0	0.06006	-1.88		0.1658	0.0601	0.0601
Anastomitrabeculia_didymospora	<0.0001(<0.0001,<0.0001)	0	0	<0.0001(<0.0001,<0.0001)	0	0	<0.0001(<0.0001,<0.0001)	0.00029	0.00029	0.0019	-3.11	**	0.0136	0.0019	** 0.0019
Papiliotrema_aurea	<0.0001(<0.0001,<0.0001)	2.79E-05	2.8E-05	<0.0001(<0.0001,<0.0001)	1.16E-05	9.32E-06	<0.0001(<0.0001,<0.0001)	0	0	0.55425	-0.59		0.7066	0.5543	0.5543
Tilletia_barclayana	<0.0001(<0.0001,<0.0001)	2.17E-05	2.2E-05	<0.0001(<0.0001,<0.0001)	2.62E-05	2.56E-05	<0.0001(<0.0001,<0.0001)	0	0	0.31168	-1.01		0.4827	0.3117	0.3117
Eremothecium_ashbyi	<0.0001(<0.0001,<0.0001)	0	0	<0.0001(<0.0001,<0.0001)	7.72E-05	7.61E-05	<0.0001(<0.0001,<0.0001)	0	0	0.0715	-1.8		0.1846	0.0715	0.0715
Thanatephorus_cucumeris	<0.0001(<0.0001,<0.0001)	3.25E-05	3.1E-05	<0.0001(<0.0001,<0.0001)	0	0	<0.0001(<0.0001,<0.0001)	0	0	0.44271	-0.77		0.5909	0.4427	0.4427
Paecilomyces_spe_Incertae_sedis	<0.0001(<0.0001,<0.0001)	0	0	<0.0001(<0.0001,<0.0001)	7.58E-05	7.58E-05	<0.0001(<0.0001,<0.0001)	0	0	0.26915	-1.11		0.433	0.2691	0.2691
Epicoccum_viticis	<0.0001(<0.0001,<0.0001)	0	0	<0.0001(<0.0001,<0.0001)	7.56E-05	7.56E-05	<0.0001(<0.0001,<0.0001)	0	0	0.26915	-1.11		0.433	0.2691	0.2691
Quambalaria_cyanescens	<0.0001(<0.0001,<0.0001)	3.19E-05	3.2E-05	<0.0001(<0.0001,<0.0001)	0	0	<0.0001(<0.0001,<0.0001)	0	0	0.76419	-0.3		0.7866	0.7642	0.7642
Meyerozyma_guilliermondii	<0.0001(<0.0001,<0.0001)	2.51E-05	2.4E-05	<0.0001(<0.0001,<0.0001)	1.39E-06	1.39E-06	<0.0001(<0.0001,<0.0001)	5.4E-05	3.3E-05	0.00111	-3.26	**	0.0103	0.0011	** 0.0011
Nemania_viridis	<0.0001(<0.0001,<0.0001)	0	0	<0.0001(<0.0001,<0.0001)	7.46E-05	7.46E-05	<0.0001(<0.0001,<0.0001)	0	0	0.26915	-1.11		0.433	0.2691	0.2691
[Candida]_incommunis	<0.0001(<0.0001,<0.0001)	3.16E-05	3.1E-05	<0.0001(<0.0001,<0.0001)	0	0	<0.0001(<0.0001,<0.0001)	0	0	0.58243	-0.55		0.7066	0.5824	0.5824
Cordyceps_lepidopterorum	<0.0001(<0.0001,<0.0001)	1.28E-05	3.3E-06	<0.0001(<0.0001,<0.0001)	3.55E-05	9.74E-06	<0.0001(<0.0001,<0.0001)	2.9E-05	1.6E-05	0.05679	-1.9		0.1644	0.0568	0.0568
Stagonosporopsis_nemophilae	<0.0001(<0.0001,<0.0001)	3.1E-05	2.8E-05	<0.0001(<0.0001,<0.0001)	0	0	<0.0001(<0.0001,<0.0001)	0	0	0.2537	-1.14		0.433	0.2537	0.2537
Lentinus_squarrosulus	<0.0001(<0.0001,<0.0001)	3.1E-05	3.1E-05	<0.0001(<0.0001,<0.0001)	0	0	<0.0001(<0.0001,<0.0001)	0	0	0.76419	-0.3		0.7866	0.7642	0.7642
Cytospora_brevispora	<0.0001(<0.0001,<0.0001)	3.05E-05	3E-05	<0.0001(<0.0001,<0.0001)	0	0	<0.0001(<0.0001,<0.0001)	0	0	0.44271	-0.77		0.5909	0.4427	0.4427
Aquapteridospora_fusiformis	<0.0001(<0.0001,<0.0001)	1.63E-05	1.2E-05	<0.0001(<0.0001,<0.0001)	3.32E-05	2.56E-05	<0.0001(<0.0001,<0.0001)	0	0	0.41116	-0.82		0.5839	0.4112	0.4112

Acrodontium_crateriforme	<0.0001(<0.0001,<0.0001)	0	0	<0.0001(<0.0001,<0.0001)	0	0	<0.0001(<0.0001,<0.0001)	0.00026	0.00026	0.0019	-3.11	**	0.0136	0.0019	** 0.0019
Stemphylium_spe_Incertae_sedis	<0.0001(<0.0001,<0.0001)	2.96E-05	2.9E-05	<0.0001(<0.0001,<0.0001)	0	0	<0.0001(<0.0001,<0.0001)	0	0	0.3356	-0.96		0.4934	0.3356	0.3356
Podosphaera_aphanis	<0.0001(<0.0001,<0.0001)	0	0	<0.0001(<0.0001,<0.0001)	6.97E-05	6.69E-05	<0.0001(<0.0001,<0.0001)	0	0	0.01875	-2.35	*	0.0708	0.0187	* 0.0187
Pichia_fermentans	<0.0001(<0.0001,<0.0001)	2.71E-05	2.5E-05	<0.0001(<0.0001,<0.0001)	2.21E-06	2.21E-06	<0.0001(<0.0001,<0.0001)	0	0	0.82925	-0.22		0.8471	0.8293	0.8293
Lembosiniella_eucalyptorum	<0.0001(<0.0001,<0.0001)	6.21E-07	6.2E-07	<0.0001(<0.0001,<0.0001)	6.33E-05	6.33E-05	<0.0001(<0.0001,<0.0001)	0	0	0.74278	-0.33		0.7866	0.7428	0.7428
Malassezia_furfur	<0.0001(<0.0001,<0.0001)	2.71E-05	2.2E-05	<0.0001(<0.0001,<0.0001)	0	0	<0.0001(<0.0001,<0.0001)	0	0	0.2537	-1.14		0.433	0.2537	0.2537
Kodamaea_ohmeri	<0.0001(<0.0001,<0.0001)	0	0	<0.0001(<0.0001,<0.0001)	5.64E-06	4.13E-06	<0.0001(<0.0001,<0.0001)	0.00022	0.00022	0.03976	-2.06	*	0.1237	0.0398	* 0.0398
Trichosporon_asahii	<0.0001(<0.0001,<0.0001)	2.67E-05	2.2E-05	<0.0001(<0.0001,<0.0001)	0	0	<0.0001(<0.0001,<0.0001)	0	0	0.10779	-1.61		0.245	0.1078	0.1078
Neocucurbitaria_irregularis	<0.0001(<0.0001,<0.0001)	0	0	<0.0001(<0.0001,<0.0001)	6.07E-05	5.99E-05	<0.0001(<0.0001,<0.0001)	7.4E-06	7.4E-06	0.04061	-2.05	*	0.1249	0.0406	* 0.0406
Elaphomyces_anthracinus	<0.0001(<0.0001,<0.0001)	2.01E-05	1.8E-05	<0.0001(<0.0001,<0.0001)	1.4E-05	7.97E-06	<0.0001(<0.0001,<0.0001)	0	0	0.20483	-1.27		0.4001	0.2048	0.2048
Lophiostoma_cynaroidis	<0.0001(<0.0001,<0.0001)	2.34E-05	2.3E-05	<0.0001(<0.0001,<0.0001)	0	0	<0.0001(<0.0001,<0.0001)	7.1E-06	7.1E-06	0.23358	-1.19		0.433	0.2336	0.2336
Cystofilobasidium_infirmominiatum	<0.0001(<0.0001,<0.0001)	2.25E-05	1.2E-05	<0.0001(<0.0001,<0.0001)	3.71E-06	3.71E-06	<0.0001(<0.0001,<0.0001)	0	0	0.37758	-0.88		0.5479	0.3776	0.3776
Penidiella_spe_Incertae_sedis	<0.0001(<0.0001,<0.0001)	2.4E-05	2.4E-05	<0.0001(<0.0001,<0.0001)	0	0	<0.0001(<0.0001,<0.0001)	0	0	0.76419	-0.3		0.7866	0.7642	0.7642
Symmetrospora_marina	<0.0001(<0.0001,<0.0001)	2.39E-05	2.4E-05	<0.0001(<0.0001,<0.0001)	0	0	<0.0001(<0.0001,<0.0001)	0	0	0.76419	-0.3		0.7866	0.7642	0.7642
Roussoella_solani	<0.0001(<0.0001,<0.0001)	2.2E-05	2.1E-05	<0.0001(<0.0001,<0.0001)	0	0	<0.0001(<0.0001,<0.0001)	0	0	0.44271	-0.77		0.5909	0.4427	0.4427
Homortomyces_tamaricis	<0.0001(<0.0001,<0.0001)	2.2E-05	2.2E-05	<0.0001(<0.0001,<0.0001)	0	0	<0.0001(<0.0001,<0.0001)	0	0	0.76419	-0.3		0.7866	0.7642	0.7642
Actinomucor_elegans	<0.0001(<0.0001,<0.0001)	2.04E-05	4.7E-06	<0.0001(<0.0001,<0.0001)	2.21E-06	2.21E-06	<0.0001(<0.0001,<0.0001)	3.9E-06	3.9E-06	0.00711	-2.69	**	0.0356	0.0071	** 0.0071
Plenodomus_enteroleucus	<0.0001(<0.0001,<0.0001)	0	0	<0.0001(<0.0001,<0.0001)	5.11E-05	4.79E-05	<0.0001(<0.0001,<0.0001)	0	0	0.00124	-3.23	**	0.011	0.0012	** 0.0012
Trichoderma_pleuroticola	<0.0001(<0.0001,<0.0001)	2.16E-05	2.1E-05	<0.0001(<0.0001,<0.0001)	0	0	<0.0001(<0.0001,<0.0001)	0	0	0.58243	-0.55		0.7066	0.5824	0.5824
Penicillium_glabrum	<0.0001(<0.0001,<0.0001)	3.94E-07	3.9E-07	<0.0001(<0.0001,<0.0001)	2.98E-05	2.98E-05	<0.0001(<0.0001,<0.0001)	7.3E-05	5.7E-05	0.00408	-2.87	**	0.0238	0.0041	** 0.0041
Allocryptovalsa_polyspora	<0.0001(<0.0001,<0.0001)	0	0	<0.0001(<0.0001,<0.0001)	4.84E-05	4.84E-05	<0.0001(<0.0001,<0.0001)	0	0	0.26915	-1.11		0.433	0.2691	0.2691
Flammulina_velutipes	<0.0001(<0.0001,<0.0001)	2.04E-05	2E-05	<0.0001(<0.0001,<0.0001)	0	0	<0.0001(<0.0001,<0.0001)	0	0	0.76419	-0.3		0.7866	0.7642	0.7642
Kluyveromyces_lactis	<0.0001(<0.0001,<0.0001)	0	0	<0.0001(<0.0001,<0.0001)	4.66E-05	4.54E-05	<0.0001(<0.0001,<0.0001)	0	0	0.0715	-1.8		0.1846	0.0715	0.0715
Ophiosphaerella_agrostidis	<0.0001(<0.0001,<0.0001)	7.27E-07	7.3E-07	<0.0001(<0.0001,<0.0001)	4.48E-05	3.18E-05	<0.0001(<0.0001,<0.0001)	0	0	0.09867	-1.65		0.2355	0.0987	0.0987
Heydenia_spe_Incertae_sedis	<0.0001(<0.0001,<0.0001)	1.97E-05	2E-05	<0.0001(<0.0001,<0.0001)	0	0	<0.0001(<0.0001,<0.0001)	0	0	0.76419	-0.3		0.7866	0.7642	0.7642
Penicillium_cinnamopurpureum	<0.0001(<0.0001,<0.0001)	0.000017	1.7E-05	<0.0001(<0.0001,<0.0001)	5.89E-06	4.81E-06	<0.0001(<0.0001,<0.0001)	0	0	0.31168	-1.01		0.4827	0.3117	0.3117
Trichoderma_longibrachiatum	<0.0001(<0.0001,<0.0001)	1.94E-05	1.7E-05	<0.0001(<0.0001,<0.0001)	0	0	<0.0001(<0.0001,<0.0001)	0	0	0.3356	-0.96		0.4934	0.3356	0.3356
Sarocladium_subulatum	<0.0001(<0.0001,<0.0001)	1.87E-05	1.8E-05	<0.0001(<0.0001,<0.0001)	1.77E-06	1.77E-06	<0.0001(<0.0001,<0.0001)	0	0	0.87748	-0.15		0.8843	0.8775	0.8775

Penicillium_fructuariae-cellae	<0.0001(<0.0001,<0.0001)	0	0	<0.0001(<0.0001,<0.0001)	4.34E-05	3.82E-05	<0.0001(<0.0001,<0.0001)	8.7E-06	8.7E-06	0.04145	-2.04	*	0.1249	0.0415	* 0.0415
Lithohypha_aloicola	<0.0001(<0.0001,<0.0001)	1.82E-05	1.5E-05	<0.0001(<0.0001,<0.0001)	0	0	<0.0001(<0.0001,<0.0001)	0	0	0.3356	-0.96		0.4934	0.3356	0.3356
Trigonopsis_vinaria	<0.0001(<0.0001,<0.0001)	3.71E-07	3.7E-07	<0.0001(<0.0001,<0.0001)	4.15E-05	3.77E-05	<0.0001(<0.0001,<0.0001)	0	0	0.30009	-1.04		0.467	0.3001	0.3001
Coniosporium_apollinis	<0.0001(<0.0001,<0.0001)	1.74E-05	1.7E-05	<0.0001(<0.0001,<0.0001)	0	0	<0.0001(<0.0001,<0.0001)	0	0	0.58243	-0.55		0.7066	0.5824	0.5824
Aspergillus_ochraceus	<0.0001(<0.0001,<0.0001)	0	0	<0.0001(<0.0001,<0.0001)	3.38E-05	3.38E-05	<0.0001(<0.0001,<0.0001)	2.7E-05	2.7E-05	0.03757	-2.08	*	0.1203	0.0376	* 0.0376
Protomyces_inouyei	<0.0001(<0.0001,<0.0001)	0	0	<0.0001(<0.0001,<0.0001)	4.07E-05	4.07E-05	<0.0001(<0.0001,<0.0001)	0	0	0.26915	-1.11		0.433	0.2691	0.2691
Ceriporia_xylostromatoides	<0.0001(<0.0001,<0.0001)	0	0	<0.0001(<0.0001,<0.0001)	4.06E-05	3.93E-05	<0.0001(<0.0001,<0.0001)	0	0	0.0715	-1.8		0.1846	0.0715	0.0715
Rhodotorula_diobovata	<0.0001(<0.0001,<0.0001)	1.68E-05	1.7E-05	<0.0001(<0.0001,<0.0001)	0	0	<0.0001(<0.0001,<0.0001)	0	0	0.76419	-0.3		0.7866	0.7642	0.7642
Entyloma_calendulae	<0.0001(<0.0001,<0.0001)	1.66E-05	6.2E-06	<0.0001(<0.0001,<0.0001)	0	0	<0.0001(<0.0001,<0.0001)	0	0	0.03308	-2.13	*	0.1113	0.0331	* 0.0331
Pseudohelminthosporium_clematidis	<0.0001(<0.0001,<0.0001)	1.62E-05	1.6E-05	<0.0001(<0.0001,<0.0001)	0	0	<0.0001(<0.0001,<0.0001)	0	0	0.76419	-0.3		0.7866	0.7642	0.7642
Penicillium_oxalicum	<0.0001(<0.0001,<0.0001)	0	0	<0.0001(<0.0001,<0.0001)	3.73E-05	2.49E-05	<0.0001(<0.0001,<0.0001)	0	0	0.00485	-2.82	**	0.0276	0.0049	** 0.0049
Apiotrichum_brassicae	<0.0001(<0.0001,<0.0001)	4.55E-07	4.5E-07	<0.0001(<0.0001,<0.0001)	2.9E-05	2.82E-05	<0.0001(<0.0001,<0.0001)	2.7E-05	2.7E-05	0.17608	-1.35		0.3591	0.1761	0.1761
Diaporthe_celeris	<0.0001(<0.0001,<0.0001)	1.19E-05	1.2E-05	<0.0001(<0.0001,<0.0001)	0	0	<0.0001(<0.0001,<0.0001)	3.3E-05	3.3E-05	0.16555	-1.39		0.3445	0.1655	0.1655
[Candida]_boidinii	<0.0001(<0.0001,<0.0001)	0	0	<0.0001(<0.0001,<0.0001)	3.68E-05	3.53E-05	<0.0001(<0.0001,<0.0001)	0	0	0.0715	-1.8		0.1846	0.0715	0.0715
Cytospora_myrtagena	<0.0001(<0.0001,<0.0001)	1.55E-05	1.5E-05	<0.0001(<0.0001,<0.0001)	0	0	<0.0001(<0.0001,<0.0001)	0	0	0.44271	-0.77		0.5909	0.4427	0.4427
Microascus_paisii	<0.0001(<0.0001,<0.0001)	1.47E-05	1.1E-05	<0.0001(<0.0001,<0.0001)	0	0	<0.0001(<0.0001,<0.0001)	0	0	0.58243	-0.55		0.7066	0.5824	0.5824
Beauveria_lii	<0.0001(<0.0001,<0.0001)	2.58E-06	2.6E-06	<0.0001(<0.0001,<0.0001)	2.49E-05	1.4E-05	<0.0001(<0.0001,<0.0001)	1.2E-05	1.2E-05	0.04249	-2.03	*	0.1266	0.0425	* 0.0425
Umbilicaria_maculata	<0.0001(<0.0001,<0.0001)	0	0	<0.0001(<0.0001,<0.0001)	3.43E-05	3.43E-05	<0.0001(<0.0001,<0.0001)	0	0	0.26915	-1.11		0.433	0.2691	0.2691
Clonostachys_eriocamporesii	<0.0001(<0.0001,<0.0001)	3.83E-06	3.8E-06	<0.0001(<0.0001,<0.0001)	2.47E-05	2.11E-05	<0.0001(<0.0001,<0.0001)	0	0	0.101	-1.64		0.2385	0.101	0.101
Buckleyzyma_aurantiaca	<0.0001(<0.0001,<0.0001)	0	0	<0.0001(<0.0001,<0.0001)	3.17E-05	2.99E-05	<0.0001(<0.0001,<0.0001)	0	0	0.01875	-2.35	*	0.0708	0.0187	* 0.0187
Lentithecium_aquaticum	<0.0001(<0.0001,<0.0001)	0	0	<0.0001(<0.0001,<0.0001)	0	0	<0.0001(<0.0001,<0.0001)	0.00012	0.00012	0.0019	-3.11	**	0.0136	0.0019	** 0.0019
Komagataella_phaffii	<0.0001(<0.0001,<0.0001)	1.33E-05	1.3E-05	<0.0001(<0.0001,<0.0001)	0	0	<0.0001(<0.0001,<0.0001)	0	0	0.76419	-0.3		0.7866	0.7642	0.7642
Ascosphaera_duoformis	<0.0001(<0.0001,<0.0001)	1.32E-05	1.3E-05	<0.0001(<0.0001,<0.0001)	0	0	<0.0001(<0.0001,<0.0001)	0	0	0.58243	-0.55		0.7066	0.5824	0.5824
Candida_maltosa	<0.0001(<0.0001,<0.0001)	1.32E-05	1.3E-05	<0.0001(<0.0001,<0.0001)	0	0	<0.0001(<0.0001,<0.0001)	0	0	0.58243	-0.55		0.7066	0.5824	0.5824
Tetrapisispora_blattae	<0.0001(<0.0001,<0.0001)	3.59E-06	2.4E-06	<0.0001(<0.0001,<0.0001)	1.73E-05	6.1E-06	<0.0001(<0.0001,<0.0001)	2E-05	1.4E-05	0.01144	-2.53	*	0.0501	0.0114	* 0.0114
Hyphopichia_burtonii	<0.0001(<0.0001,<0.0001)	1.04E-05	1E-05	<0.0001(<0.0001,<0.0001)	5.66E-06	5.66E-06	<0.0001(<0.0001,<0.0001)	0	0	0.75049	-0.32		0.7866	0.7505	0.7505
Cyphellophora_sessilis	<0.0001(<0.0001,<0.0001)	1.27E-05	1.2E-05	<0.0001(<0.0001,<0.0001)	0	0	<0.0001(<0.0001,<0.0001)	0	0	0.58243	-0.55		0.7066	0.5824	0.5824

Aspergillus_fumisynnematus	<0.0001(<0.0001,<0.0001)	1.21E-05	1.2E-05	<0.0001(<0.0001,<0.0001)	1.07E-06	1.07E-06	<0.0001(<0.0001,<0.0001)	0	0	0.75049	-0.32		0.7866	0.7505	0.7505
Penicillium_pancosmium	<0.0001(<0.0001,<0.0001)	1.25E-05	1.3E-05	<0.0001(<0.0001,<0.0001)	0	0	<0.0001(<0.0001,<0.0001)	0	0	0.76419	-0.3		0.7866	0.7642	0.7642
Sarocladium_oryzae	<0.0001(<0.0001,<0.0001)	9.6E-06	9.6E-06	<0.0001(<0.0001,<0.0001)	6.21E-06	6.21E-06	<0.0001(<0.0001,<0.0001)	0	0	0.75049	-0.32		0.7866	0.7505	0.7505
Sordaria_spe_Incertae_sedis	<0.0001(<0.0001,<0.0001)	1.21E-05	9.2E-06	<0.0001(<0.0001,<0.0001)	0	0	<0.0001(<0.0001,<0.0001)	0	0	0.2537	-1.14		0.433	0.2537	0.2537
Candida_nivariensis	<0.0001(<0.0001,<0.0001)	1.21E-05	1.2E-05	<0.0001(<0.0001,<0.0001)	0	0	<0.0001(<0.0001,<0.0001)	0	0	0.76419	-0.3		0.7866	0.7642	0.7642
Arthrinium_rasikravindrae	<0.0001(<0.0001,<0.0001)	9.71E-06	9.2E-06	<0.0001(<0.0001,<0.0001)	5.39E-06	5.39E-06	<0.0001(<0.0001,<0.0001)	0	0	0.87748	-0.15		0.8843	0.8775	0.8775
Pichia_cactophila	<0.0001(<0.0001,<0.0001)	1.15E-05	2.9E-06	<0.0001(<0.0001,<0.0001)	1.09E-06	1.09E-06	<0.0001(<0.0001,<0.0001)	0	0	0.02208	-2.29	*	0.0808	0.0221	* 0.0221
Kurtzmaniella_quercitrusa	<0.0001(<0.0001,<0.0001)	0	0	<0.0001(<0.0001,<0.0001)	2.56E-05	2.56E-05	<0.0001(<0.0001,<0.0001)	9.3E-06	9.3E-06	0.03757	-2.08	*	0.1203	0.0376	* 0.0376
Aspergillus_glabripes	<0.0001(<0.0001,<0.0001)	1.13E-05	7.7E-06	<0.0001(<0.0001,<0.0001)	6.79E-07	6.79E-07	<0.0001(<0.0001,<0.0001)	0	0	0.8271	-0.22		0.8471	0.8271	0.8271
Uwebraunia_musae	<0.0001(<0.0001,<0.0001)	0	0	<0.0001(<0.0001,<0.0001)	2.64E-05	2.64E-05	<0.0001(<0.0001,<0.0001)	0	0	0.26915	-1.11		0.433	0.2691	0.2691
Malassezia_cuniculi	<0.0001(<0.0001,<0.0001)	9.24E-07	9.2E-07	<0.0001(<0.0001,<0.0001)	2.41E-05	2.18E-05	<0.0001(<0.0001,<0.0001)	0	0	0.30009	-1.04		0.467	0.3001	0.3001
Puccinia_striiformis	<0.0001(<0.0001,<0.0001)	1.11E-05	1.1E-05	<0.0001(<0.0001,<0.0001)	0	0	<0.0001(<0.0001,<0.0001)	0	0	0.76419	-0.3		0.7866	0.7642	0.7642
Collybiopsis_filamentipes	<0.0001(<0.0001,<0.0001)	1.1E-05	1.1E-05	<0.0001(<0.0001,<0.0001)	0	0	<0.0001(<0.0001,<0.0001)	0	0	0.76419	-0.3		0.7866	0.7642	0.7642
Coronicium_gemmiferum	<0.0001(<0.0001,<0.0001)	1.1E-05	1E-05	<0.0001(<0.0001,<0.0001)	0	0	<0.0001(<0.0001,<0.0001)	0	0	0.58243	-0.55		0.7066	0.5824	0.5824
Strobilurus_spe_Incertae_sedis	<0.0001(<0.0001,<0.0001)	9.8E-06	9.8E-06	<0.0001(<0.0001,<0.0001)	0	0	<0.0001(<0.0001,<0.0001)	9.1E-06	9.1E-06	0.06381	-1.85		0.1727	0.0638	0.0638
Ceraceosorus_guamensis	<0.0001(<0.0001,<0.0001)	0	0	<0.0001(<0.0001,<0.0001)	2.5E-05	2.5E-05	<0.0001(<0.0001,<0.0001)	0	0	0.26915	-1.11		0.433	0.2691	0.2691
Hazslinszkyomyces_aloes	<0.0001(<0.0001,<0.0001)	8.36E-06	8.4E-06	<0.0001(<0.0001,<0.0001)	4.73E-06	4.73E-06	<0.0001(<0.0001,<0.0001)	0	0	0.75049	-0.32		0.7866	0.7505	0.7505
Sarocladium_zeae	<0.0001(<0.0001,<0.0001)	6.52E-07	6.5E-07	<0.0001(<0.0001,<0.0001)	1.93E-05	1.37E-05	<0.0001(<0.0001,<0.0001)	1.3E-05	1.3E-05	0.1763	-1.35		0.3591	0.1763	0.1763
Simplicillium_lanosoniveum	<0.0001(<0.0001,<0.0001)	9.76E-06	9.3E-06	<0.0001(<0.0001,<0.0001)	0	0	<0.0001(<0.0001,<0.0001)	0	0	0.58243	-0.55		0.7066	0.5824	0.5824
Flagelloscypha_spe_Incertae_sedis	<0.0001(<0.0001,<0.0001)	9.7E-06	9.7E-06	<0.0001(<0.0001,<0.0001)	0	0	<0.0001(<0.0001,<0.0001)	0	0	0.76419	-0.3		0.7866	0.7642	0.7642
Marantokordyana_oberwinkleriana	<0.0001(<0.0001,<0.0001)	9.45E-06	9.4E-06	<0.0001(<0.0001,<0.0001)	0	0	<0.0001(<0.0001,<0.0001)	0	0	0.76419	-0.3		0.7866	0.7642	0.7642
Archaeorhizomyces_borealis	<0.0001(<0.0001,<0.0001)	9.35E-06	9.3E-06	<0.0001(<0.0001,<0.0001)	0	0	<0.0001(<0.0001,<0.0001)	0	0	0.76419	-0.3		0.7866	0.7642	0.7642
Berkeleyomyces_basicola	<0.0001(<0.0001,<0.0001)	0	0	<0.0001(<0.0001,<0.0001)	2.18E-05	1.71E-05	<0.0001(<0.0001,<0.0001)	0	0	0.0715	-1.8		0.1846	0.0715	0.0715
Extremus_spe_Incertae_sedis	<0.0001(<0.0001,<0.0001)	0	0	<0.0001(<0.0001,<0.0001)	2.03E-05	2.03E-05	<0.0001(<0.0001,<0.0001)	0	0	0.26915	-1.11		0.433	0.2691	0.2691
Penicillium_estinogenum	<0.0001(<0.0001,<0.0001)	0	0	<0.0001(<0.0001,<0.0001)	2.03E-05	2.03E-05	<0.0001(<0.0001,<0.0001)	0	0	0.26915	-1.11		0.433	0.2691	0.2691
Oliveonia_spe_Incertae_sedis	<0.0001(<0.0001,<0.0001)	8.56E-06	8.6E-06	<0.0001(<0.0001,<0.0001)	0	0	<0.0001(<0.0001,<0.0001)	0	0	0.76419	-0.3		0.7866	0.7642	0.7642
Fusarium_oxysporum	<0.0001(<0.0001,<0.0001)	0	0	<0.0001(<0.0001,<0.0001)	0	0	<0.0001(<0.0001,<0.0001)	7.4E-05	5.5E-05	5.7E-09	-5.83	***	6E-07	<0.0001	***<0.0001
Cutaneotrichosporon_arboriforme	<0.0001(<0.0001,<0.0001)	0	0	<0.0001(<0.0001,<0.0001)	0	0	<0.0001(<0.0001,<0.0001)	7.2E-05	6.8E-05	3.4E-06	-4.65	***	0.0001	<0.0001	***<0.0001

Periconia_circinata	<0.0001(<0.0001,<0.0001)	8.09E-06	7.8E-06	<0.0001(<0.0001,<0.0001)	0	0	<0.0001(<0.0001,<0.0001)	0	0	0.58243	-0.55		0.7066	0.5824	0.5824
Saccharomycopsis_fibuligera	<0.0001(<0.0001,<0.0001)	8.09E-06	5.9E-06	<0.0001(<0.0001,<0.0001)	0	0	<0.0001(<0.0001,<0.0001)	0	0	0.58243	-0.55		0.7066	0.5824	0.5824
Talaromyces_stollii	<0.0001(<0.0001,<0.0001)	0	0	<0.0001(<0.0001,<0.0001)	1.88E-05	1.88E-05	<0.0001(<0.0001,<0.0001)	0	0	0.26915	-1.11		0.433	0.2691	0.2691
Tritirachium_cinnamomeum	<0.0001(<0.0001,<0.0001)	7.9E-06	7.9E-06	<0.0001(<0.0001,<0.0001)	0	0	<0.0001(<0.0001,<0.0001)	0	0	0.76419	-0.3		0.7866	0.7642	0.7642
Neoerysiphe_galeopsidis	<0.0001(<0.0001,<0.0001)	7.86E-06	7.5E-06	<0.0001(<0.0001,<0.0001)	0	0	<0.0001(<0.0001,<0.0001)	0	0	0.58243	-0.55		0.7066	0.5824	0.5824
Pyricularia_oryzae	<0.0001(<0.0001,<0.0001)	7.75E-06	7.5E-06	<0.0001(<0.0001,<0.0001)	0	0	<0.0001(<0.0001,<0.0001)	0	0	0.58243	-0.55		0.7066	0.5824	0.5824
Mycoarthris_spe_Incertae_sedis	<0.0001(<0.0001,<0.0001)	0	0	<0.0001(<0.0001,<0.0001)	1.78E-05	1.78E-05	<0.0001(<0.0001,<0.0001)	0	0	0.26915	-1.11		0.433	0.2691	0.2691
Byssochlamys_spectabilis	<0.0001(<0.0001,<0.0001)	3.05E-06	2E-06	<0.0001(<0.0001,<0.0001)	0	0	<0.0001(<0.0001,<0.0001)	3.8E-05	3.8E-05	0.22834	-1.2		0.433	0.2283	0.2283
Ascochyta_rabiei	<0.0001(<0.0001,<0.0001)	4.86E-06	3.6E-06	<0.0001(<0.0001,<0.0001)	5.39E-06	4.11E-06	<0.0001(<0.0001,<0.0001)	0	0	0.76399	-0.3		0.7866	0.764	0.764
Meira_argovae	<0.0001(<0.0001,<0.0001)	7.1E-06	7.1E-06	<0.0001(<0.0001,<0.0001)	0	0	<0.0001(<0.0001,<0.0001)	0	0	0.76419	-0.3		0.7866	0.7642	0.7642
Parathyridaria_rosae	<0.0001(<0.0001,<0.0001)	0.000007	6.2E-06	<0.0001(<0.0001,<0.0001)	0	0	<0.0001(<0.0001,<0.0001)	0	0	0.44271	-0.77		0.5909	0.4427	0.4427
Entyloma_cosmi	<0.0001(<0.0001,<0.0001)	6.52E-06	6.5E-06	<0.0001(<0.0001,<0.0001)	8.75E-07	8.75E-07	<0.0001(<0.0001,<0.0001)	0	0	0.75049	-0.32		0.7866	0.7505	0.7505
Xenodevriesia_strelitziicola	<0.0001(<0.0001,<0.0001)	6.77E-06	6.4E-06	<0.0001(<0.0001,<0.0001)	0	0	<0.0001(<0.0001,<0.0001)	0	0	0.58243	-0.55		0.7066	0.5824	0.5824
Effuseotrichosporon_vanderwaltii	<0.0001(<0.0001,<0.0001)	6.29E-07	6.3E-07	<0.0001(<0.0001,<0.0001)	0	0	<0.0001(<0.0001,<0.0001)	5.3E-05	5.3E-05	0.06013	-1.88		0.1658	0.0601	0.0601
Dothiora_spartii	<0.0001(<0.0001,<0.0001)	5.14E-06	4.9E-06	<0.0001(<0.0001,<0.0001)	3.54E-06	2.67E-06	<0.0001(<0.0001,<0.0001)	0	0	0.55425	-0.59		0.7066	0.5543	0.5543
Gjaerumia_minor	<0.0001(<0.0001,<0.0001)	6.62E-06	6.6E-06	<0.0001(<0.0001,<0.0001)	0	0	<0.0001(<0.0001,<0.0001)	0	0	0.76419	-0.3		0.7866	0.7642	0.7642
Sidera_vesiculosa	<0.0001(<0.0001,<0.0001)	6.61E-06	6.6E-06	<0.0001(<0.0001,<0.0001)	0	0	<0.0001(<0.0001,<0.0001)	0	0	0.76419	-0.3		0.7866	0.7642	0.7642
Neoacladium_indicum	<0.0001(<0.0001,<0.0001)	6.43E-06	6.4E-06	<0.0001(<0.0001,<0.0001)	0	0	<0.0001(<0.0001,<0.0001)	0	0	0.76419	-0.3		0.7866	0.7642	0.7642
Strelitziana_eucalypti	<0.0001(<0.0001,<0.0001)	6.14E-06	3.7E-06	<0.0001(<0.0001,<0.0001)	0	0	<0.0001(<0.0001,<0.0001)	0	0	0.44271	-0.77		0.5909	0.4427	0.4427
Curvularia_soli	<0.0001(<0.0001,<0.0001)	3.98E-06	4E-06	<0.0001(<0.0001,<0.0001)	4.66E-06	4.66E-06	<0.0001(<0.0001,<0.0001)	0	0	0.75049	-0.32		0.7866	0.7505	0.7505
Cerrena_aurantiopora	<0.0001(<0.0001,<0.0001)	5.87E-06	4.8E-06	<0.0001(<0.0001,<0.0001)	0	0	<0.0001(<0.0001,<0.0001)	0	0	0.44271	-0.77		0.5909	0.4427	0.4427
Simplicillium_spe_Incertae_sedis	<0.0001(<0.0001,<0.0001)	0	0	<0.0001(<0.0001,<0.0001)	1.38E-05	1.38E-05	<0.0001(<0.0001,<0.0001)	0	0	0.26915	-1.11		0.433	0.2691	0.2691
Phaeoacremonium_griseo-olivaceum	<0.0001(<0.0001,<0.0001)	0	0	<0.0001(<0.0001,<0.0001)	1.37E-05	1.37E-05	<0.0001(<0.0001,<0.0001)	0	0	0.26915	-1.11		0.433	0.2691	0.2691
Tetracladium_spe_Incertae_sedis	<0.0001(<0.0001,<0.0001)	5.7E-06	5.7E-06	<0.0001(<0.0001,<0.0001)	0	0	<0.0001(<0.0001,<0.0001)	0	0	0.76419	-0.3		0.7866	0.7642	0.7642
Stephanosporaceae_spe_Incertae_sedis	<0.0001(<0.0001,<0.0001)	5.68E-06	5.7E-06	<0.0001(<0.0001,<0.0001)	0	0	<0.0001(<0.0001,<0.0001)	0	0	0.76419	-0.3		0.7866	0.7642	0.7642
Sarocladium_kiliense	<0.0001(<0.0001,<0.0001)	5.61E-06	3.8E-06	<0.0001(<0.0001,<0.0001)	0	0	<0.0001(<0.0001,<0.0001)	0	0	0.44271	-0.77		0.5909	0.4427	0.4427
Epicoccum_duchesneae	<0.0001(<0.0001,<0.0001)	5.59E-06	5E-06	<0.0001(<0.0001,<0.0001)	0	0	<0.0001(<0.0001,<0.0001)	0	0	0.58243	-0.55		0.7066	0.5824	0.5824

Periconia_macrospinosa	<0.0001(<0.0001,<0.0001)	5.57E-06	5.6E-06	<0.0001(<0.0001,<0.0001)	0	0	<0.0001(<0.0001,<0.0001)	0	0	0.76419	-0.3		0.7866	0.7642		0.7642
Avachytrium_platense	<0.0001(<0.0001,<0.0001)	5.52E-06	5.5E-06	<0.0001(<0.0001,<0.0001)	0	0	<0.0001(<0.0001,<0.0001)	0	0	0.76419	-0.3		0.7866	0.7642		0.7642
Knufia_cryptophialidica	<0.0001(<0.0001,<0.0001)	5.48E-06	5.5E-06	<0.0001(<0.0001,<0.0001)	0	0	<0.0001(<0.0001,<0.0001)	0	0	0.76419	-0.3		0.7866	0.7642		0.7642
Iodophanus_testaceus	<0.0001(<0.0001,<0.0001)	5.35E-06	4.3E-06	<0.0001(<0.0001,<0.0001)	0	0	<0.0001(<0.0001,<0.0001)	0	0	0.58243	-0.55		0.7066	0.5824		0.5824
Ochroconis_mirabilis	<0.0001(<0.0001,<0.0001)	0	0	<0.0001(<0.0001,<0.0001)	1.25E-05	1.25E-05	<0.0001(<0.0001,<0.0001)	0	0	0.26915	-1.11		0.433	0.2691		0.2691
Pararamichloridium_caricicola	<0.0001(<0.0001,<0.0001)	5.11E-06	5.1E-06	<0.0001(<0.0001,<0.0001)	0	0	<0.0001(<0.0001,<0.0001)	0	0	0.76419	-0.3		0.7866	0.7642		0.7642
Curvularia_chonburiensis	<0.0001(<0.0001,<0.0001)	5.08E-06	5.1E-06	<0.0001(<0.0001,<0.0001)	0	0	<0.0001(<0.0001,<0.0001)	0	0	0.76419	-0.3		0.7866	0.7642		0.7642
Basidiodendron_globisporum	<0.0001(<0.0001,<0.0001)	5.02E-06	5E-06	<0.0001(<0.0001,<0.0001)	0	0	<0.0001(<0.0001,<0.0001)	0	0	0.76419	-0.3		0.7866	0.7642		0.7642
Coniella_quercicola	<0.0001(<0.0001,<0.0001)	4.96E-06	5E-06	<0.0001(<0.0001,<0.0001)	0	0	<0.0001(<0.0001,<0.0001)	0	0	0.76419	-0.3		0.7866	0.7642		0.7642
Ceratocystis_pirilliformis	<0.0001(<0.0001,<0.0001)	0	0	<0.0001(<0.0001,<0.0001)	1.05E-06	1.05E-06	<0.0001(<0.0001,<0.0001)	3.9E-05	3.9E-05	0.03578	-2.1	*	0.1168	0.0358	*	0.0358
Mrakia_aquatica	<0.0001(<0.0001,<0.0001)	0	0	<0.0001(<0.0001,<0.0001)	1.14E-05	1.14E-05	<0.0001(<0.0001,<0.0001)	0	0	0.26915	-1.11		0.433	0.2691		0.2691
Tremateia_chromolaenae	<0.0001(<0.0001,<0.0001)	4.68E-06	4.7E-06	<0.0001(<0.0001,<0.0001)	0	0	<0.0001(<0.0001,<0.0001)	0	0	0.76419	-0.3		0.7866	0.7642		0.7642
Xylodon_filicinus	<0.0001(<0.0001,<0.0001)	6.29E-07	4.5E-07	<0.0001(<0.0001,<0.0001)	9.52E-06	3.94E-06	<0.0001(<0.0001,<0.0001)	0	0	0.00237	-3.04	**	0.0161	0.0024	**	0.0024
Iodosphaeria_phyllophila	<0.0001(<0.0001,<0.0001)	4.58E-06	4.6E-06	<0.0001(<0.0001,<0.0001)	0	0	<0.0001(<0.0001,<0.0001)	0	0	0.76419	-0.3		0.7866	0.7642		0.7642
Rhizopogon_laricinus	<0.0001(<0.0001,<0.0001)	4.55E-06	4.5E-06	<0.0001(<0.0001,<0.0001)	0	0	<0.0001(<0.0001,<0.0001)	0	0	0.76419	-0.3		0.7866	0.7642		0.7642
Robbauera_albescens	<0.0001(<0.0001,<0.0001)	4.53E-06	4.5E-06	<0.0001(<0.0001,<0.0001)	0	0	<0.0001(<0.0001,<0.0001)	0	0	0.76419	-0.3		0.7866	0.7642		0.7642
Acremonium_vitellinum	<0.0001(<0.0001,<0.0001)	4.48E-06	4.5E-06	<0.0001(<0.0001,<0.0001)	0	0	<0.0001(<0.0001,<0.0001)	0	0	0.76419	-0.3		0.7866	0.7642		0.7642
Adisciso_yakushimense	<0.0001(<0.0001,<0.0001)	0	0	<0.0001(<0.0001,<0.0001)	1.05E-05	1.05E-05	<0.0001(<0.0001,<0.0001)	0	0	0.26915	-1.11		0.433	0.2691		0.2691
Peziza_fruticosa	<0.0001(<0.0001,<0.0001)	0	0	<0.0001(<0.0001,<0.0001)	1.05E-05	1.05E-05	<0.0001(<0.0001,<0.0001)	0	0	0.26915	-1.11		0.433	0.2691		0.2691
Komagataella_ulmi	<0.0001(<0.0001,<0.0001)	4.42E-06	4.4E-06	<0.0001(<0.0001,<0.0001)	0	0	<0.0001(<0.0001,<0.0001)	0	0	0.76419	-0.3		0.7866	0.7642		0.7642
Phialocephala_trigonospora	<0.0001(<0.0001,<0.0001)	0	0	<0.0001(<0.0001,<0.0001)	4.7E-06	3.55E-06	<0.0001(<0.0001,<0.0001)	2E-05	2E-05	0.03976	-2.06	*	0.1237	0.0398	*	0.0398
Alternaria_hungarica	<0.0001(<0.0001,<0.0001)	4.14E-06	3.3E-06	<0.0001(<0.0001,<0.0001)	0	0	<0.0001(<0.0001,<0.0001)	0	0	0.44271	-0.77		0.5909	0.4427		0.4427
Meira_nashicola	<0.0001(<0.0001,<0.0001)	4.14E-06	4.1E-06	<0.0001(<0.0001,<0.0001)	0	0	<0.0001(<0.0001,<0.0001)	0	0	0.76419	-0.3		0.7866	0.7642		0.7642
Cryptococcus_neoformans	<0.0001(<0.0001,<0.0001)	3.26E-07	3.3E-07	<0.0001(<0.0001,<0.0001)	8.91E-06	5.52E-06	<0.0001(<0.0001,<0.0001)	0	0	0.09638	-1.66		0.2326	0.0964		0.0964
Acremonium_cavaraeanum	<0.0001(<0.0001,<0.0001)	4.08E-06	2.1E-06	<0.0001(<0.0001,<0.0001)	0	0	<0.0001(<0.0001,<0.0001)	0	0	0.19126	-1.31		0.3758	0.1913		0.1913
Trichoderma_effusum	<0.0001(<0.0001,<0.0001)	0	0	<0.0001(<0.0001,<0.0001)	9.48E-06	9.48E-06	<0.0001(<0.0001,<0.0001)	0	0	0.26915	-1.11		0.433	0.2691		0.2691
Puccinia_recondita	<0.0001(<0.0001,<0.0001)	4.92E-07	4.9E-07	<0.0001(<0.0001,<0.0001)	8.07E-06	8.07E-06	<0.0001(<0.0001,<0.0001)	0	0	0.74278	-0.33		0.7866	0.7428		0.7428
Periconia_epilithographicola	<0.0001(<0.0001,<0.0001)	3.6E-06	3.6E-06	<0.0001(<0.0001,<0.0001)	0	0	<0.0001(<0.0001,<0.0001)	0	0	0.76419	-0.3		0.7866	0.7642		0.7642

Penicillium_sumatraense	<0.0001(<0.0001,<0.0001)	1.63E-06	1.6E-06	<0.0001(<0.0001,<0.0001)	0	0	<0.0001(<0.0001,<0.0001)	1.7E-05	1.7E-05	0.06013	-1.88		0.1658	0.0601	0.0601
Aspergillus_inflatus	<0.0001(<0.0001,<0.0001)	2.26E-06	2E-06	<0.0001(<0.0001,<0.0001)	2.68E-06	1.97E-06	<0.0001(<0.0001,<0.0001)	0	0	0.55425	-0.59		0.7066	0.5543	0.5543
Cryptococcus_uniguttulatus	<0.0001(<0.0001,<0.0001)	0	0	<0.0001(<0.0001,<0.0001)	3.86E-06	2.24E-06	<0.0001(<0.0001,<0.0001)	1.3E-05	1.3E-05	0.02157	-2.3	*	0.0798	0.0216	* 0.0216
Zeloasperisporium_searsiae	<0.0001(<0.0001,<0.0001)	3.08E-06	3.1E-06	<0.0001(<0.0001,<0.0001)	0	0	<0.0001(<0.0001,<0.0001)	0	0	0.76419	-0.3		0.7866	0.7642	0.7642
Keratinophyton_linfenense	<0.0001(<0.0001,<0.0001)	0	0	<0.0001(<0.0001,<0.0001)	6.82E-06	6.82E-06	<0.0001(<0.0001,<0.0001)	0	0	0.26915	-1.11		0.433	0.2691	0.2691
Graphium_penicillioides	<0.0001(<0.0001,<0.0001)	2.86E-06	2.9E-06	<0.0001(<0.0001,<0.0001)	0	0	<0.0001(<0.0001,<0.0001)	0	0	0.76419	-0.3		0.7866	0.7642	0.7642
Groenewaldozyma_tartarivorans	<0.0001(<0.0001,<0.0001)	2.84E-06	2.8E-06	<0.0001(<0.0001,<0.0001)	0	0	<0.0001(<0.0001,<0.0001)	0	0	0.76419	-0.3		0.7866	0.7642	0.7642
Choanephora_cucurbitarum	<0.0001(<0.0001,<0.0001)	2.83E-06	2.8E-06	<0.0001(<0.0001,<0.0001)	0	0	<0.0001(<0.0001,<0.0001)	0	0	0.76419	-0.3		0.7866	0.7642	0.7642
Zymoseptoria_spe_Incertae_sedis	<0.0001(<0.0001,<0.0001)	2.76E-06	2.8E-06	<0.0001(<0.0001,<0.0001)	0	0	<0.0001(<0.0001,<0.0001)	0	0	0.76419	-0.3		0.7866	0.7642	0.7642
Delastria_faustiniana	<0.0001(<0.0001,<0.0001)	2.74E-06	1.8E-06	<0.0001(<0.0001,<0.0001)	0	0	<0.0001(<0.0001,<0.0001)	0	0	0.3356	-0.96		0.4934	0.3356	0.3356
Punctularia_atropurpurascens	<0.0001(<0.0001,<0.0001)	0	0	<0.0001(<0.0001,<0.0001)	0	0	<0.0001(<0.0001,<0.0001)	2.4E-05	2.4E-05	0.0019	-3.11	**	0.0136	0.0019	** 0.0019
Cyphellophora_olivacea	<0.0001(<0.0001,<0.0001)	2.72E-06	2.7E-06	<0.0001(<0.0001,<0.0001)	0	0	<0.0001(<0.0001,<0.0001)	0	0	0.76419	-0.3		0.7866	0.7642	0.7642
Houjia_yanglingensis	<0.0001(<0.0001,<0.0001)	0	0	<0.0001(<0.0001,<0.0001)	0	0	<0.0001(<0.0001,<0.0001)	2.4E-05	2.4E-05	0.0019	-3.11	**	0.0136	0.0019	** 0.0019
Clonostachys_rosea	<0.0001(<0.0001,<0.0001)	0	0	<0.0001(<0.0001,<0.0001)	6.23E-06	6.23E-06	<0.0001(<0.0001,<0.0001)	0	0	0.26915	-1.11		0.433	0.2691	0.2691
Hydropisphaera_spe_Incertae_sedis	<0.0001(<0.0001,<0.0001)	2.61E-06	2.6E-06	<0.0001(<0.0001,<0.0001)	0	0	<0.0001(<0.0001,<0.0001)	0	0	0.76419	-0.3		0.7866	0.7642	0.7642
Jattaea_ribicola	<0.0001(<0.0001,<0.0001)	2.61E-06	2.6E-06	<0.0001(<0.0001,<0.0001)	0	0	<0.0001(<0.0001,<0.0001)	0	0	0.76419	-0.3		0.7866	0.7642	0.7642
Fracchiaea_myricoides	<0.0001(<0.0001,<0.0001)	1.26E-06	1.3E-06	<0.0001(<0.0001,<0.0001)	3.02E-06	3.02E-06	<0.0001(<0.0001,<0.0001)	0	0	0.74278	-0.33		0.7866	0.7428	0.7428
Gibellulopsis_serrae	<0.0001(<0.0001,<0.0001)	0	0	<0.0001(<0.0001,<0.0001)	5.91E-06	5.91E-06	<0.0001(<0.0001,<0.0001)	0	0	0.26915	-1.11		0.433	0.2691	0.2691
Sistotremastrum_guttuliferum	<0.0001(<0.0001,<0.0001)	0	0	<0.0001(<0.0001,<0.0001)	5.91E-06	4.73E-06	<0.0001(<0.0001,<0.0001)	0	0	0.0715	-1.8		0.1846	0.0715	0.0715
Paracremonium_binnewijzendii	<0.0001(<0.0001,<0.0001)	2.45E-06	2E-06	<0.0001(<0.0001,<0.0001)	0	0	<0.0001(<0.0001,<0.0001)	0	0	0.58243	-0.55		0.7066	0.5824	0.5824
Leptosporella_bambusae	<0.0001(<0.0001,<0.0001)	2.43E-06	2.4E-06	<0.0001(<0.0001,<0.0001)	0	0	<0.0001(<0.0001,<0.0001)	0	0	0.76419	-0.3		0.7866	0.7642	0.7642
Chytridiomycota_spe_Incertae_sedis	<0.0001(<0.0001,<0.0001)	2.42E-06	2.4E-06	<0.0001(<0.0001,<0.0001)	0	0	<0.0001(<0.0001,<0.0001)	0	0	0.76419	-0.3		0.7866	0.7642	0.7642
Pseudorobillarda_sojae	<0.0001(<0.0001,<0.0001)	2.36E-06	2.4E-06	<0.0001(<0.0001,<0.0001)	0	0	<0.0001(<0.0001,<0.0001)	0	0	0.76419	-0.3		0.7866	0.7642	0.7642
Mrakia_frigida	<0.0001(<0.0001,<0.0001)	1.81E-06	1.2E-06	<0.0001(<0.0001,<0.0001)	1.13E-06	1.13E-06	<0.0001(<0.0001,<0.0001)	0	0	0.83131	-0.21		0.8479	0.8313	0.8313
Penicillium_citrinum	<0.0001(<0.0001,<0.0001)	8.71E-07	5.5E-07	<0.0001(<0.0001,<0.0001)	3.27E-06	2.32E-06	<0.0001(<0.0001,<0.0001)	0	0	0.69968	-0.39		0.7866	0.6997	0.6997
Microascus_trigonosporus	<0.0001(<0.0001,<0.0001)	2.08E-06	2.1E-06	<0.0001(<0.0001,<0.0001)	0	0	<0.0001(<0.0001,<0.0001)	0	0	0.76419	-0.3		0.7866	0.7642	0.7642
Cookeina_korfii	<0.0001(<0.0001,<0.0001)	0	0	<0.0001(<0.0001,<0.0001)	4.91E-06	3.85E-06	<0.0001(<0.0001,<0.0001)	0	0	0.0715	-1.8		0.1846	0.0715	0.0715
Aspergillus_westerdijkiae	<0.0001(<0.0001,<0.0001)	4.77E-07	4.8E-07	<0.0001(<0.0001,<0.0001)	3.55E-06	3.55E-06	<0.0001(<0.0001,<0.0001)	0	0	0.74278	-0.33		0.7866	0.7428	0.7428

Diaporthe_miriciae	<0.0001(<0.0001,<0.0001)	1.97E-06	2E-06	<0.0001(<0.0001,<0.0001)	0	0	<0.0001(<0.0001,<0.0001)	0	0	0.76419	-0.3		0.7866	0.7642	0.7642
Entoloma_graphitipes_f._cystidiatum	<0.0001(<0.0001,<0.0001)	0	0	<0.0001(<0.0001,<0.0001)	4.64E-06	4.64E-06	<0.0001(<0.0001,<0.0001)	0	0	0.26915	-1.11		0.433	0.2691	0.2691
Penicillium_multicolor	<0.0001(<0.0001,<0.0001)	1.93E-06	1.9E-06	<0.0001(<0.0001,<0.0001)	0	0	<0.0001(<0.0001,<0.0001)	0	0	0.76419	-0.3		0.7866	0.7642	0.7642
[Candida]_pseudointermedia	<0.0001(<0.0001,<0.0001)	0	0	<0.0001(<0.0001,<0.0001)	4.5E-06	4.5E-06	<0.0001(<0.0001,<0.0001)	0	0	0.26915	-1.11		0.433	0.2691	0.2691
Amylostereum_orientale	<0.0001(<0.0001,<0.0001)	0	0	<0.0001(<0.0001,<0.0001)	4.27E-06	4.27E-06	<0.0001(<0.0001,<0.0001)	0	0	0.26915	-1.11		0.433	0.2691	0.2691
Wickerhamomyces_onychis	<0.0001(<0.0001,<0.0001)	1.71E-06	1.7E-06	<0.0001(<0.0001,<0.0001)	0	0	<0.0001(<0.0001,<0.0001)	0	0	0.76419	-0.3		0.7866	0.7642	0.7642
Bovista_psammophila	<0.0001(<0.0001,<0.0001)	4.55E-07	4.5E-07	<0.0001(<0.0001,<0.0001)	2.91E-06	2.09E-06	<0.0001(<0.0001,<0.0001)	0	0	0.30009	-1.04		0.467	0.3001	0.3001
Tritirachium_oryzae	<0.0001(<0.0001,<0.0001)	1.61E-06	1.3E-06	<0.0001(<0.0001,<0.0001)	0	0	<0.0001(<0.0001,<0.0001)	0	0	0.58243	-0.55		0.7066	0.5824	0.5824
Paraleptosphaeria_rumicis	<0.0001(<0.0001,<0.0001)	1.56E-06	1.6E-06	<0.0001(<0.0001,<0.0001)	0	0	<0.0001(<0.0001,<0.0001)	0	0	0.76419	-0.3		0.7866	0.7642	0.7642
Powellomyces_spe_Incertae_sedis	<0.0001(<0.0001,<0.0001)	0	0	<0.0001(<0.0001,<0.0001)	3.68E-06	3.68E-06	<0.0001(<0.0001,<0.0001)	0	0	0.26915	-1.11		0.433	0.2691	0.2691
Wickerhamomyces_anomalus	<0.0001(<0.0001,<0.0001)	0	0	<0.0001(<0.0001,<0.0001)	3.55E-06	3.55E-06	<0.0001(<0.0001,<0.0001)	0	0	0.26915	-1.11		0.433	0.2691	0.2691
Rhodotorula_ingeniosa	<0.0001(<0.0001,<0.0001)	0	0	<0.0001(<0.0001,<0.0001)	3.5E-06	3.5E-06	<0.0001(<0.0001,<0.0001)	0	0	0.26915	-1.11		0.433	0.2691	0.2691
Vararia_gallica	<0.0001(<0.0001,<0.0001)	1.48E-06	1.5E-06	<0.0001(<0.0001,<0.0001)	0	0	<0.0001(<0.0001,<0.0001)	0	0	0.76419	-0.3		0.7866	0.7642	0.7642
Scopulariopsis_cordiae	<0.0001(<0.0001,<0.0001)	0	0	<0.0001(<0.0001,<0.0001)	3.43E-06	3.43E-06	<0.0001(<0.0001,<0.0001)	0	0	0.26915	-1.11		0.433	0.2691	0.2691
Diaporthe_pseudooculi	<0.0001(<0.0001,<0.0001)	1.3E-06	1.3E-06	<0.0001(<0.0001,<0.0001)	0	0	<0.0001(<0.0001,<0.0001)	0	0	0.76419	-0.3		0.7866	0.7642	0.7642
Gregarithecium_curvisporum	<0.0001(<0.0001,<0.0001)	1.3E-06	1.3E-06	<0.0001(<0.0001,<0.0001)	0	0	<0.0001(<0.0001,<0.0001)	0	0	0.76419	-0.3		0.7866	0.7642	0.7642
Entyloma_linariae	<0.0001(<0.0001,<0.0001)	1.23E-06	7.3E-07	<0.0001(<0.0001,<0.0001)	0	0	<0.0001(<0.0001,<0.0001)	0	0	0.44271	-0.77		0.5909	0.4427	0.4427
Plectosphaerella_niemeijerarum	<0.0001(<0.0001,<0.0001)	1.21E-06	1.2E-06	<0.0001(<0.0001,<0.0001)	0	0	<0.0001(<0.0001,<0.0001)	0	0	0.76419	-0.3		0.7866	0.7642	0.7642
Scopuloides_rimosa	<0.0001(<0.0001,<0.0001)	0	0	<0.0001(<0.0001,<0.0001)	2.7E-06	2.7E-06	<0.0001(<0.0001,<0.0001)	0	0	0.26915	-1.11		0.433	0.2691	0.2691
Typhula_setipes	<0.0001(<0.0001,<0.0001)	0	0	<0.0001(<0.0001,<0.0001)	2.7E-06	1.89E-06	<0.0001(<0.0001,<0.0001)	0	0	0.0715	-1.8		0.1846	0.0715	0.0715
Buckleyzyma_armeniaca	<0.0001(<0.0001,<0.0001)	1.1E-06	1.1E-06	<0.0001(<0.0001,<0.0001)	0	0	<0.0001(<0.0001,<0.0001)	0	0	0.76419	-0.3		0.7866	0.7642	0.7642
Chaetosphaeria_guttulata	<0.0001(<0.0001,<0.0001)	0	0	<0.0001(<0.0001,<0.0001)	2.57E-06	2.57E-06	<0.0001(<0.0001,<0.0001)	0	0	0.26915	-1.11		0.433	0.2691	0.2691
Tremateia_murispora	<0.0001(<0.0001,<0.0001)	1.07E-06	1.1E-06	<0.0001(<0.0001,<0.0001)	0	0	<0.0001(<0.0001,<0.0001)	0	0	0.76419	-0.3		0.7866	0.7642	0.7642
Beauveria_bassiana	<0.0001(<0.0001,<0.0001)	1.01E-06	1E-06	<0.0001(<0.0001,<0.0001)	0	0	<0.0001(<0.0001,<0.0001)	0	0	0.76419	-0.3		0.7866	0.7642	0.7642
Alternaria_chlamydospora	<0.0001(<0.0001,<0.0001)	0	0	<0.0001(<0.0001,<0.0001)	2.23E-06	2.23E-06	<0.0001(<0.0001,<0.0001)	0	0	0.26915	-1.11		0.433	0.2691	0.2691
Pichia_mandshurica	<0.0001(<0.0001,<0.0001)	9.32E-07	9.3E-07	<0.0001(<0.0001,<0.0001)	0	0	<0.0001(<0.0001,<0.0001)	0	0	0.76419	-0.3		0.7866	0.7642	0.7642
Echinoporia_hydnophora	<0.0001(<0.0001,<0.0001)	9.02E-07	9E-07	<0.0001(<0.0001,<0.0001)	0	0	<0.0001(<0.0001,<0.0001)	0	0	0.76419	-0.3		0.7866	0.7642	0.7642

Pseudopithomyces_angolensis	<0.0001(<0.0001,<0.0001)	0	0	<0.0001(<0.0001,<0.0001)	0	0	<0.0001(<0.0001,<0.0001)	7.9E-06	6.6E-06	3.4E-06	-4.65	***	0.0001	<0.0001	***<0.0001
Alternaria_nepalensis	<0.0001(<0.0001,<0.0001)	0	0	<0.0001(<0.0001,<0.0001)	1.73E-06	1.73E-06	<0.0001(<0.0001,<0.0001)	0	0	0.26915	-1.11		0.433	0.2691	0.2691
Septobasidium_spe_Incertae_sedis	<0.0001(<0.0001,<0.0001)	7.27E-07	7.3E-07	<0.0001(<0.0001,<0.0001)	0	0	<0.0001(<0.0001,<0.0001)	0	0	0.76419	-0.3		0.7866	0.7642	0.7642
Cystobasidium_slooffiae	<0.0001(<0.0001,<0.0001)	0	0	<0.0001(<0.0001,<0.0001)	1.7E-06	1.7E-06	<0.0001(<0.0001,<0.0001)	0	0	0.26915	-1.11		0.433	0.2691	0.2691
Emericellopsis_robusta	<0.0001(<0.0001,<0.0001)	0	0	<0.0001(<0.0001,<0.0001)	1.45E-06	1.45E-06	<0.0001(<0.0001,<0.0001)	0	0	0.26915	-1.11		0.433	0.2691	0.2691
Chaenothecopsis_resinophila	<0.0001(<0.0001,<0.0001)	0	0	<0.0001(<0.0001,<0.0001)	1.38E-06	1.38E-06	<0.0001(<0.0001,<0.0001)	0	0	0.26915	-1.11		0.433	0.2691	0.2691
Zygosporium_pseudogibbum	<0.0001(<0.0001,<0.0001)	5.83E-07	5.8E-07	<0.0001(<0.0001,<0.0001)	0	0	<0.0001(<0.0001,<0.0001)	0	0	0.76419	-0.3		0.7866	0.7642	0.7642
Neodevriesia_spe_Incertae_sedis	<0.0001(<0.0001,<0.0001)	5.76E-07	4.1E-07	<0.0001(<0.0001,<0.0001)	0	0	<0.0001(<0.0001,<0.0001)	0	0	0.58243	-0.55		0.7066	0.5824	0.5824
Sphaerulina_westendorpii	<0.0001(<0.0001,<0.0001)	0	0	<0.0001(<0.0001,<0.0001)	1.34E-06	1.34E-06	<0.0001(<0.0001,<0.0001)	0	0	0.26915	-1.11		0.433	0.2691	0.2691
Edenia_gomezpompae	<0.0001(<0.0001,<0.0001)	5.15E-07	5.2E-07	<0.0001(<0.0001,<0.0001)	0	0	<0.0001(<0.0001,<0.0001)	0	0	0.76419	-0.3		0.7866	0.7642	0.7642
Paludomyces_mangrovei	<0.0001(<0.0001,<0.0001)	0	0	<0.0001(<0.0001,<0.0001)	1.13E-06	1.13E-06	<0.0001(<0.0001,<0.0001)	0	0	0.26915	-1.11		0.433	0.2691	0.2691
Chloridium_pini	<0.0001(<0.0001,<0.0001)	0	0	<0.0001(<0.0001,<0.0001)	1.09E-06	1.09E-06	<0.0001(<0.0001,<0.0001)	0	0	0.26915	-1.11		0.433	0.2691	0.2691
Mucor_racemosus	<0.0001(<0.0001,<0.0001)	0	0	<0.0001(<0.0001,<0.0001)	1.05E-06	1.05E-06	<0.0001(<0.0001,<0.0001)	0	0	0.26915	-1.11		0.433	0.2691	0.2691
Anthracina_saxicola	<0.0001(<0.0001,<0.0001)	0	0	<0.0001(<0.0001,<0.0001)	1.04E-06	1.04E-06	<0.0001(<0.0001,<0.0001)	0	0	0.26915	-1.11		0.433	0.2691	0.2691
Emergomyces_canadensis	<0.0001(<0.0001,<0.0001)	4.32E-07	4.3E-07	<0.0001(<0.0001,<0.0001)	0	0	<0.0001(<0.0001,<0.0001)	0	0	0.76419	-0.3		0.7866	0.7642	0.7642
Yarrowia_porcina	<0.0001(<0.0001,<0.0001)	4.32E-07	4.3E-07	<0.0001(<0.0001,<0.0001)	0	0	<0.0001(<0.0001,<0.0001)	0	0	0.76419	-0.3		0.7866	0.7642	0.7642
Coprinopsis_atramentaria	<0.0001(<0.0001,<0.0001)	0	0	<0.0001(<0.0001,<0.0001)	0.000001	0.000001	<0.0001(<0.0001,<0.0001)	0	0	0.26915	-1.11		0.433	0.2691	0.2691
Mucor_pseudolusitanicus	<0.0001(<0.0001,<0.0001)	0	0	<0.0001(<0.0001,<0.0001)	9.29E-07	9.29E-07	<0.0001(<0.0001,<0.0001)	0	0	0.26915	-1.11		0.433	0.2691	0.2691
Geotrichum_silvicola	<0.0001(<0.0001,<0.0001)	0	0	<0.0001(<0.0001,<0.0001)	9.11E-07	9.11E-07	<0.0001(<0.0001,<0.0001)	0	0	0.26915	-1.11		0.433	0.2691	0.2691
Inocyclus_angularis	<0.0001(<0.0001,<0.0001)	0	0	<0.0001(<0.0001,<0.0001)	8.75E-07	8.75E-07	<0.0001(<0.0001,<0.0001)	0	0	0.26915	-1.11		0.433	0.2691	0.2691
Knufia_karalitana	<0.0001(<0.0001,<0.0001)	0	0	<0.0001(<0.0001,<0.0001)	8.57E-07	8.57E-07	<0.0001(<0.0001,<0.0001)	0	0	0.26915	-1.11		0.433	0.2691	0.2691

Supplementary Table S8. The relative abundance and distribution of the key 47 OTUs													
	Control001	Control097	Control096	Control095	Control094	Control093	Control092	Control091	Control090	Control089	Control088	Control087	Control086
OTU1653 (Blumeria_spe_Incertae_sedis)	0	0	0.017801	0	0	0	0	0	0	0	0	0	0
OTU1499 (Gibberella_fujikuroi)	0	0	0.00E+00	0	0	0	0	0	0	0	0	0	0
OTU2410 (Candida_albicans)	0	0	0	0	0	0	0	0	0	0	0	0	0
OTU3836 (Acrodictys_fluminicola)	0	0	0	0	0	0	0	0.0013	0.00857	0	0	0	0
OTU2703 (Candida_albicans)	0	0	0	0	0	0	0	0	0	0	0	0	0
OTU434 (Blumeria_spe_Incertae_sedis)	0	0	0.0341579	0	0	0	0	0	0	0	0.00076	0	0
OTU2555 (Blumeria_spe_Incertae_sedis)	0	0	0.037552	0	0	0	0	0	0	0	0	0	0
OTU3193 (Candida_albicans)	0	0	0.00E+00	0	0	0	0	0	0	0	0	0	0
OTU3692 (Blumeria_spe_Incertae_sedis)	0	0	0.0307058	0	0	0	0	0	0.00117	0	0	0	0.00054
OTU179 (Henningsia_resupinata)	0	0	0	0	0	0	0	0	0	0	0	0	0
OTU2746 (Blumeria_spe_Incertae_sedis)	0	0	0.016025	0	0	0	0	0	0	0	0.00059	0	0
OTU286 (Cutaneotrichosporon_cutaneum)	0	0	0	0	0	0	0	0	0	0	0	0	0
OTU152 (Starmerella_etchellsii)	0.00081	0	0	0	0	0	0	0	0	0	0	0	0
OTU217 (Pichia_kluyveri)	0	0	0	0	0	0	0	0	0	0	0	0	0
OTU78 (Ustilaginoidea_virens)	0	0	0	0	0	0	0	0	0	0	0	0	0
OTU3641 (Blumeria_spe_Incertae_sedis)	0.00064	0	0.0369084	0.00032	0	0	0	0	0	0	0	0	0
OTU175 (Blumeria_spe_Incertae_sedis)	0	0	0.0195723	0	0	0	0	0	0	0	0	0	0
OTU88 (Pseudoveronaea_ellipsoidea)	0	0	0	0	0	0	0	0	0	0	0	0	0
OTU1894 (Cryptococcus_longus)	0.00027	0.00038	0.0144756	0.0085	0.01951	0.00031	0.03071	0.01871	0.00041	0.00404	0	0.0217	0.0004
OTU125 (Penicillium_hirsutum)	0	0	0	0	0	0	0.09368	0	0	0.00019	0	0.00013	0
OTU3781 (Zanclospora_jonesii)	0.00152	0	0.00E+00	0.00018	0	0.00023	0	0.00029	0	0.0325	0.05017	0.01878	0.0836
OTU16 (Malassezia_sympodialis)	0.00027	0.00053	0.0129334	0.00026	0	0.00041	0.00025	0	0.00242	0	0.00038	9.2E-05	0
OTU231 (Ophiostoma_acarorum)	0.00021	0	0.0001634	0	0.00019	0	0.0003	0.00021	0	0	0	0	0
OTU2466 (Zanclospora_jonesii)	0.00024	0.00023	0.0002121	0	0	0	0	0.00022	0.00028	0.06202	0.08166	0.03947	0.03696
OTU65 (Milospium_graphideorum)	0	0.00035	0.010866	0.00058	0.13065	0.00034	0	0.00013	0	0.03651	0	0	0
OTU1393 (Cryptococcus_longus)	0	0	0.016621	0.0075	0.01541	0.0011	0.02537	0.02013	0.00066	0.00504	0	0.0203	0.00021
OTU3529 (Raffaelea_lauricola)	0.00035	0	0.0036945	0	0	0	0	0.00121	0.0005	0.0001	0.0001	8.2E-05	0.0002
OTU52 (Diaporthe_kochmanii)	0	0.00064	0.0003407	0.00029	0.00028	0.00034	0.00046	0.00028	0.00047	0.00023	0	0	0
OTU3583 (Blumeria_spe_Incertae_sedis)	0.0001	0	0.0242382	0	9.6E-05	0.00011	0.00023	0	0.00013	0	0.00017	0	0.00012
OTU98 (Chrysosphaeria_jan-nelii)	0	0	0	0	0	0	0	0	0	0	0	0	0
OTU58 (Gibberella_fujikuroi)	0	0	0	0	0	0	0	0	0	0	0	0	0
OTU33 (Aspergillus_subflavus)	0.00014	5.8E-05	3.87E-02	0	0.0001	6.4E-05	0	0	0	0	5.1E-05	3.8E-05	7.5E-05
OTU9 (Candida_albicans)	6.4E-05	0	0.00E+00	0	0	5.7E-05	0	0	0	0	0	0	0
OTU3448 (Zanclospora_jonesii)	0.00037	0.00102	0.0005365	0.00826	0.00039	0.00044	0.00043	0.00044	0.00075	0.00057	0.00018	0.0166	0.00023
OTU26 (Zanclospora_jonesii)	0.00048	0.00012	0.0048965	0	0	6.8E-05	8.9E-05	4.7E-05	0	0.05371	0.0211	0.01136	0.07173
OTU2481 (Candida_albicans)	0	0	0	0	0	0	0	0	0	0	0	0	0
OTU31 (Arthrographis_grakistii)	0.00071	0.00043	0.000355	0.00019	0.00017	0.00041	0.00039	0.00022	0.00014	0.00017	0.00047	0.00027	0.00039
OTU21 (Saccharomyces_cerevisiae)	0	4E-05	0	0.00502	0	0	0	0	0	0	2E-05	0	0.00021
OTU17 (Diversispora_spurca)	0.00041	2.4E-05	0.0006505	0.00132	0	7.5E-05	0.00011	0.00425	0.00318	4.5E-05	0.0002	0.00042	0.00027
OTU3791 (Blumeria_spe_Incertae_sedis)	0.00015	0.00022	3.96E-02	6.5E-05	1.6E-05	3.6E-05	0.00011	2.5E-05	0	0	5.1E-05	6.7E-05	6E-05
OTU22 (Hydnobolites_roseus)	0	2.3E-05	0	0	0	0	0	0	0	2.4E-05	0	1.1E-05	0
OTU10 (Aspergillus_subflavus)	0.00012	2.1E-05	0.0222557	1.2E-05	0.00019	2.4E-05	7.4E-05	4.6E-05	5.5E-05	2.5E-05	9.5E-05	1.3E-05	0.0001
OTU3842 (Blumeria_spe_Incertae_sedis)	0.00029	3.9E-05	0.0282471	1.1E-05	5.1E-05	3.4E-05	0	2.5E-05	2.8E-05	3.3E-05	6.3E-05	7.3E-05	0.0001
OTU19 (Zanclospora_jonesii)	0	0	0	0	0	0	0	0	5.4E-05	5.5E-05	1.1E-05	2.2E-05	0
OTU15 (Aspergillus_subflavus)	0.00011	0.00011	0.0001262	0.00012	0.02858	8.4E-05	0.0002	0.0002	0.03299	9.1E-05	2.5E-05	1.1E-05	2.7E-05
OTU5 (Cryptococcus_longus)	0.00022	0.00033	0.0157291	0.00911	0.01586	0.00091	0.02759	0.01861	0.00061	0.00465	7E-05	0.02089	0.00015
OTU3 (Zanclospora_jonesii)	0.00056	0.00015	7.857E-05	3.1E-05	6.4E-05	8.6E-05	0.0001	7.1E-05	0.00045	0.06846	0.07537	0.03652	0.03512

Control085	Control084	Control083	Control082	Control081	Control080	Control079	Control078	Control077	Control076	Control075	Control074	Control073	Control072	Control071	Control070	Control069
0.0277	0	0	0	0	0	0	0	0	0	0	0	0	0	0	0.0059088	0
0	0	0	0	0	0	0	0	0	0	0	0	0	0	0	0	0
0	0	0	0	0	0	0	0	0	0	0	0	0	0	0	0	0
0.0034	0	0.00189	0.01461	0	0	0	0	0	0	0	0	0	0	0	0	0
0	0	0	0	0	0	0	0	0	0	0	0	0	0	0	0	0.00135
0.0276	0	0	0.00096	0	0	0	0.00885	0.00126	0	0	0	0	0	0.00681	0	0
0.0389	0	0	0	0	0	0	0.01508	0.0008	0	0	0	0	0	0	0	0
0	0	0	0	0	0	0	0	0	0	0	0	0	0	0	0	0.00126
0.0257	0	0	0	0.00065	0	0	0.01142	0.00227	0	0	0	0.0008522	0	0.00194	0	0.00107
0	0	0	0	0	0	0	0	0	0	0	0	0.00E+00	0	0	0	0
0.0583	0.00053	0	0	0	0	0	0.01857	0.0166	0	0	0	0.0009499	0	0.0056	0	0
0	0	0	0	0	0	0	0	0	0	0	0	0	0	0	0.0026835	0
0.0004	0	0.10535	0	0	0	0	0	0	0	0	0	0	0	0	0	0
0	0	0	0	0	0	0	0	0	0	0	0	0	0	0	0	0
0	0	0	0	0	0	0	0	0	0	0	0	0	0	0	0	0.00086
######	0	0	0	0	0	0	0.01243	0.00648	0	0	0	0	0	0.00117	0.00079	0
0.0219	0	0	0	0	0	0	0.00251	0	0	0	0	0	0	0.0008	0	0
0	0	0	0	0	0	0	0	0	0	0	0	0	0	0	0	0
0.0021	0	0.00283	0.00029	0.00275	0.01356	0.00026	0.00417	0.00272	0.01717	0	0.00346	0.0072236	0.02258	0.00472	0.0015374	0
0	0	0	0	0	0.03779	0	0	0	0	0	0	0	0	0	0	0
0.0019	0	0.00306	0.00491	0.0293	0.00974	0.006548	0.18363	0.00412	0.0359	0.02472	0.15153	0.0141685	0.02128	0.01749	0.0325377	0.00799
0.0003	0.00019	0.00892	0	0.03313	0.06339	0.000203	0	0.00021	0	0	0	0	0	0	0	0
0.001	0	0.0065	0	0	0.00013	0	0.01888	0.00221	0	0	0	0	0	0	0	0
0.0045	0	0.00514	0.00743	0.05663	0.01472	0.015056	0.06545	0.01042	0.07148	0.03527	0.0413	0.0230884	0.02615	0.04485	0.0070396	0.01579
0	0	0	0	0	0	0	0	0	0.00011	0.16576	0.00012	0.0002051	0.00039	0.00047	0	0
0.0014	0	0.00327	0.00035	0.00251	0.01485	0	0.00395	0.00375	0.01469	0.00016	0.00364	0.0064693	0.02147	0.00592	0.0023869	0
0.0369	0.00025	0.00024	0.00476	0	9.6E-05	0.000498	0	0.00028	0	0	0	0	0.00089	0.0023	0.0001468	0
0	0	0	0	0	0	0	0	0	0.00027	0.10939	0.00019	0	0	0.00028	0	0
0.0278	0	0	0	0	0	0	0.00892	0.00567	0	0	0	0.00024	0	0.00526	0.0001124	0
0	0	0	0	0	0	0	0	0	0	0	0	0	0	0	0.00E+00	0
0	0	0	0	0	0	0	0	0	0	0	0	7.6E-05	0	0	0	0
0.0437	0.0002	4E-05	0	4.8E-05	4.8E-05	0	0.01677	0.00542	0	0	0	6.368E-05	0	0.00076	0.00E+00	0.00027
0	0	0	0	0	0	0	0	0	3.7E-05	0	5.1E-05	0	0	0	0	0.00061
0.0002	0.00052	0.00025	0.00092	0.00021	0.00022	0.000345	0.00022	0.00015	0	0	0	0	0	0	0	8.2E-05
0.0055	0.00041	0.01763	0.00982	0.09062	0.00011	0.037982	0.00018	0.01844	0.04138	0.04627	0.09009	4.132E-05	0.00835	0.01707	0.0194688	0.11176
######	0	0	0	0	0	0	0	0	0	0	0	0	0	0	0	0.0001
0.0005	0.35392	0.00032	0.0004	0.00028	0.00041	0.000233	0.00033	0.00049	1.7E-05	0	0	0	0	0	0	0
######	0	0	0	0	0	0	0	0.00142	0.00011	7.8E-05	5.5E-05	0.0001271	0.00013	0.0012	0.0005473	0.00027
0.0003	0.00021	0.00014	0.00023	0.00023	0.03781	0.000731	0.00032	0.00176	1.7E-05	0	0	0	0	0.00313	5.766E-05	0.00149
0.0339	9.7E-05	2.3E-05	8.5E-05	5.2E-05	7.9E-05	8.17E-05	0.0131	0.00366	0	0	0	0.0002267	0	0.00085	0.0001208	0.00012
0	0	0	0	0	0	0	0	0	0	0	0	0	0	0	0	0
0.0393	8.9E-05	4.5E-05	6.7E-05	7.2E-05	7.8E-05	1.73E-05	0.0075	0.0003	0	1.7E-05	0	0	0	0.00486	0.0001021	0.00027
0.0256	8E-05	5.4E-05	8.4E-05	5.1E-05	0.00121	4.79E-05	0.00487	0.00979	0	0	0	0	0	0.00034	0.0001803	0.00012
0	0	0.00041	0	1.3E-05	0	0	4.5E-05	2E-05	0.00481	0	0	0	0	0	0	2.5E-05
5E-05	2.5E-05	1.4E-05	5.6E-05	2.7E-05	0.00012	2.04E-05	9E-06	0.06054	0	0	0.0004	5.83E-05	0	0	0.0722004	0.00024
0.0018	0.0001	0.00256	0.00058	0.00316	0.01339	4.23E-05	0.00492	0.0044	0.01633	3.7E-05	0.00486	0.0077914	0.02098	0.00669	0.0017552	0.00018
0.0029	0.00045	0.00529	0.00564	0.0544	0.01791	0.013552	0.06072	0.01098	0.06313	0.03504	0.04911	0.0202882	0.03221	0.03538	0.008111	0.01337

Control098	Control099	Control100	Control101	Control132	Control131	Control 130	Control 129	Control 128	Control 127	Control 126	Control 125	Control 124	Control 123	Control 122	Control 121	Control 120	Control 119
0	0	0	0	0	0	0	0	0	0	0	0	0	0	0	0	0	0.0012
0	0	0	0	0	0	0	0	0	0	0	0	0	0	0	0	0	0
0	0	0	0	0	0	0	0	0	0	0	0	0	0	0	0	0	0
0.00676	0.00266	0	0.00143	0	0	0	0	0	0	0.0016	0	0.0006	0	0	0	0	0
0	0	0	0	0	0	0	0	0	0	0	0	0	0	0	0	0	0
0	0	0	0	0.00721	0	0	0.0025	0	0	0	0.0013	0	0	0	0	0	0.0013
0	0	0	0	0	0	0.0006	0.0023	0	0	0	0	0	0	0.0021	0	0	0.0016
0	0	0	0	0	0	0	0	0	0	0	0	0	0	0	0	0	0
0	0	0	0.00114	0	0	0	0.0019	0.0021	0	0	0	0	0	0	0	0	0.0012
0	0	0	0	0	0	0	0	0	0	0	0	0	0	0	0	0	0
0	0	0	0	0	0	0.0004	0.0018	0	0.0015	0.0006	0.0009	0	0	0	0	0	0.0072
0	0	0	0	0	0	0	0	0	0	0	0	0	0	0	0	0	0
0	0	0	0	0	0	0	0	0	0	0	0	0	0	0	0	0	0
0	0	0	0	0	0	0	0	0	0	0	0	0	0	0	0	0	0
0	0	0	0	0	0	0	0	0	0	0	0	0	0	0.0007	0	0	0
0	0.00035	0	0	0.00401	0	0	0.0022	0	0	0	0.0014	0	0	0.0023	0	0	0.0007
0	0	0	0	0	0	0	0	0	0	0	0	0	0	0	0.0002	0.0007	0.0003
0	0	0	0	0	0	0	0	0	0	0	0	0	0	0	0	0	0
0.00129	0.00278	0.00668	0.00646	0.1153	0.05103	0.0002	0.0524	0.0262	0.0006	0.0021	0.0007	0.0469	0	0.0065	0	0.0064	0.0006
0	0	0	0	0	0	0	0	0	0	0	0.0052	0	0	0	0	0	0
0	0	0	0.00037	0	0	0	0	0	0	0	0.0014	0	0	0	0	0	0.0002
0.00035	0.02412	0.0393	0.06533	0	0.08997	0.0002	0.0013	0	0.0006	0	0	0.0003	0	0.0005	0	0	0
0	0.03671	0	0.00024	0.0017	0.00421	0.0003	0.0005	0.0005	0.001	0.0001	0.0006	0.0006	0	0.0003	0	0	######
0.00014	0.00014	0	0	0	0	0	0	0	0	0	0.0026	0	0	0	0	0	0
0.00011	0.00028	0	0.00174	0	0	0	0.0004	0	0	0.0003	0.0002	0.0001	0	0.0004	0	0	0
0.00229	0.00289	0.00692	0.00494	0.12126	0.03919	0	0.051	0.0377	0.0007	0.0018	0	0.0497	0	0.0063	9E-05	0.0059	0.0007
0.00236	0.00116	0.00012	0.00019	0	0	8E-05	0	0	0	0.0003	0	0	0	0	0.0002	0	0.0002
0.00022	0.00028	0.29706	0	0.00358	0.00062	0.0003	0.0009	0.0006	0.0016	0.0005	0.0005	0.0004	0.0046	0.0013	0	0	0
0	0	0	0.0004	0	7E-05	0.0001	0.0012	0	0	0.0001	0.0003	8E-05	0.0008	0.0005	0.0001	0.0015	0.0021
0	0	0	0	0	0	0	0	0	0	0	0	0	0	0.001	0	0	0
0	0	0	0	0	0	0	0	0	0	0	0	0	0	0	0	0	0
4.8E-05	0	0.00014	0.00039	0	6.9E-05	0	0.0002	0.0001	0	0.0001	0	5E-05	0.0005	0.0001	7E-05	0.0004	0.0016
0	0	0	0	0	0	0	0	0	0	0	0	0	0	0	0	0	0
0.00046	0.00059	0.0003	0.00084	0	0	0	0	0	0	0	0	0	0	0	6E-05	0	0.0001
4.7E-05	5.2E-05	2.6E-05	0.01005	0	0	0	0	0	0	0	0	0	0	4E-05	5E-05	0	0
0	0	0	3.5E-05	0	0	2E-05	0	0	0	0	0	0	0	0	0	0	2E-05
0.00028	0.00011	0.00041	0	0	0	0	0	0	0	0	0	0	0	0	0	0	2E-05
4.4E-05	0	2.3E-05	0	0	2.9E-05	5E-05	0	0	0	0	0.0062	0	0	6E-05	2E-05	######	0.003
0.00068	0.01193	0.00628	0.00247	0.0002	0	0.0049	0	6E-05	0	2E-05	0	0.0019	0	0	0.0001	0.0006	0.0005
6.8E-05	0.00017	4.9E-05	0.0003	0.00108	0.00012	8E-05	0.0026	9E-05	0.0003	8E-05	0.0002	6E-05	0.0006	0.0012	4E-05	7E-05	0.0017
1.3E-05	0.00556	0	2.7E-05	0	0	0	0	0	0	0	0	0	0	0	0	4E-05	0
5.2E-05	0.00112	0.00688	0.00058	0.00075	3.7E-05	3E-05	0.0001	0.0001	5E-05	3E-05	0.0002	3E-05	0.0003	2E-05	0.0001	0.0008	0.0026
2E-05	1.6E-05	0.0044	0.00151	0.00116	0.00019	0.0002	0.0003	0.0005	0.0001	0.0002	0.0002	5E-05	0.0013	0.0003	7E-05	8E-05	0.0018
0	0	0	0.02453	0.00203	0.00022	0.0001	0.0003	0.0006	0.0002	0.0003	0.0005	0.0002	0.2502	0.0005	7E-05	0.0003	0.0001
0.00014	0.01208	0.00014	1.7E-05	0.00072	8.8E-05	5E-05	0.0001	0.0011	0.0008	4E-05	0.1589	6E-05	0.0219	0.0002	6E-05	0.0001	5E-05
0.00185	0.00323	0.00574	0.00615	0.10567	0.03944	0.0002	0.0445	0.032	0.0004	0.0026	0.0004	0.0478	0.0009	0.0061	6E-05	0.0062	0.0009
9E-05	8.3E-05	0.00011	0.00011	0.00021	1.2E-05	1E-05	0	2E-05	0.0002	0	0.0019	5E-06	0	6E-05	6E-05	8E-05	7E-05

Control 068	Control 118	Control 116	Control 115	Control114	Control113	Control 112	Control 111	Control110	Control 109	Control 108	Control 107	Control106	Control105	Control104	Control 103	Control 117
0	0	0	0	0	0	0.0017	0	0.00378	0	0.0462	0	0.04989	0	0	0	0
0	0	0	0	0	0	0	0	0	0	0	0	0	0.00E+00	0	0	0
0	0	0	0	0	0	0	0	0	0	0	0	0	0	0	0	0
0	0	0	0.001	0.038223	0.000897	0	0	5.65E-04	0	0.007	0	0	0	0.026849	0	0.0007
0	0	0	0	0	0	0	0	0	0	0	0	0.00E+00	0.00E+00	0	0	0
0	0.0139	0.0103	0	0	0.004094	0.0247	0.0115	0.01419	0.0005	0.0246	0.0005	7.45E-02	0	0	0	0
0	0	0.0169	0	0	0.00456	0.0267	0.0022	0.01835	0	0.0358	0.0008	0.1154	0	0	0	0
0	0	0	0	0	0	0	0.0011	0	0	0	0	0	0	0	0	0
0	0	0.0112	0	0	0.004866	0.0344	0.0055	0.02515	0	0.03	0	0.08874	7.42E-04	0.00E+00	0	0
0	0	0	0	0	0	0	0	0	0	0	0	0	0	0	0	0
0.0006	0	0.0129	0	0	0.012756	0.0143	0.0035	5.00E-02	0.0005	0.0659	0	0.00468	0	0	0	0
0	0	0	0	0.00E+00	0.00E+00	0	0	0	0	0	0	0	0	0	0	0
0	0	0	0	0	0	0	0	0	0	0	0	0	0	0	0	0
0	0	0	0	0	0	0	0	0	0	0	0	0	0.00E+00	0	0	0
0	0	0	0	0	0	0	0	0	0	0	0	0	0	0	0	0
0	0.0055	0.013	0	0	0.002523	0.035	0.007	0.0212	0	0.031	0	0.09907	0	0.000408	0	0
0.0002	0.0006	0.0011	0.0003	0	0.000348	0.0036	0	0.01992	0	0.0255	0.0002	0.07007	0.000185	0.000337	0	0
0	0	0	0	0	0	0	0	0	0	0	0	0	0	0	0	0
0.0033	0.0437	0.0041	0.0003	0.000203	0.001779	0.0145	0.0105	0.00126	0.0845	0.0003	0.0339	0.00492	0.009154	0	0.0057	0.015
0	0	0	0	0	0	0	0	0	0	0.0011	0	0	0.000169	0	0.0064	0
0.0034	0.0005	0	0.0365	0.000185	0	0	0	0	9E-05	0	0	0.00018	0.000206	0	0	0.0005
0	0	0	0	0	0.01262	0.0002	0	0.01914	0.0001	0.0002	0.0001	0	0.018266	0	0.0167	0.0002
0	0	0	0.0158	0	0	0	0	0	0	0.0002	0	0	0.007998	0	0	0
0.0065	0.0003	0	0.082	0	0	0	0	0	0	0	0	0.00032	0.000129	0	0.0001	0.003
0	0.0003	0	0	0.000118	0	0	0.0006	0.00017	0.0006	0.0006	9E-05	0.00032	0.416306	0.000157	0.0003	0
0.0047	0.0561	0.0045	0.0001	0.000133	0.002338	0.0151	0.0092	0.00141	0.0917	0.0006	0.0308	0.00604	0.009836	0.000555	0.0046	0.0147
0.0001	0	0	0	0.008448	0.000289	0	0.0004	0.01138	0.0001	0.0448	9E-05	9.6E-05	7.78E-05	0.028504	0	0
0	0	0	0	0.00E+00	0.00E+00	0	0	0	0	0.0014	0	0	0	0	0	0
0	0.0213	0.0167	0.0001	0	0.002762	0.0162	0.0122	1.27E-02	0	0.0293	0.0002	0.07104	6.88E-05	0.00E+00	0.0002	7E-05
0	0	0	0	0	0	0	0	0	0	0	0	0	0	0	0	0
0	0	0	0	0	0	0	0	0	0	0	0	0	0	0	0	0
4E-05	1E-04	0.0053	7E-05	4.48E-05	0.003086	0.0415	0.0173	0.02921	0.0002	0.0365	0.0005	0.10306	0.000109	0.000205	5E-05	0
0	0	0	0	5.72E-05	0	0	0	0	0	0	0	0	0	0	0	0
0	0	0	0	4.4E-05	0	0.0001	0.0005	0.00028	0.0003	1E-04	0.0004	8.4E-05	0.000175	0.000263	0.3266	0
0.0114	0	0	0	0	0	0	0	0.00797	1E-05	4E-05	0.0001	2.7E-05	5.92E-05	3.52E-05	0	0
0	0	0	0	0	0	0	0	0	0	0	0	0	0	3.24E-05	0	0
0	0	0	0	0	0.00E+00	0	0	0	0	9E-05	0	0	0	0	0	2E-05
9E-05	0	0	0	0	0	0	0	3.5E-05	0	0.0002	2E-05	0.00059	0	0	0	0
0	0.0005	0.001	0.0001	0.013547	0.039376	0.0001	0.0002	9.5E-05	0.0001	0.0538	0.0542	6.5E-05	0.000399	0.000123	0.0034	0.0057
9E-05	0.0003	0.0123	7E-05	2.96E-05	0.003434	0.0278	0.0035	0.02176	0.0001	0.0342	0.0003	0.08382	0.000171	0.000128	0.0001	7E-05
0	4E-05	4E-05	0	6.46E-05	0	######	2E-05	2.5E-05	0	4E-05	1E-05	0	0.012082	2.16E-05	0	3E-05
0.0009	0.0018	0.0184	6E-05	4.49E-05	0.005277	0.0514	0.0021	0.02325	0.0002	0.0505	0.0032	0.05309	0.000115	0.000255	0.0003	7E-05
0.0003	0	0.0097	4E-05	1.54E-05	0.004016	0.0341	0.0169	0.02664	0.0001	0.0224	0.001	0.05509	0.000182	0.000315	0.0002	6E-05
0	0.0003	9E-05	9E-05	7.54E-05	0.172531	3E-05	0.0065	5.9E-05	0.0057	0.0002	4E-05	0.00018	0.000127	0.000141	9E-05	0.0001
8E-05	0.0002	0.0051	6E-05	0.000108	8.12E-05	0	0	0	0	1E-05	0	0	2.71E-05	2.75E-05	0	6E-05
0.0029	0.043	0.0031	5E-05	0.000103	0.002434	0.0142	0.0089	0.00164	0.0861	0.001	0.0329	0.00515	0.009579	0.000358	0.0046	0.0134
0.007	0.0002	0.0002	0.0706	9.75E-05	8.48E-05	6E-05	8E-05	5E-05	4E-05	8E-05	2E-05	0.00028	5.18E-05	7.73E-05	3E-05	0.0064

Control 067	Control 102	Control 065	Control 030	Control 029	Control 028	Control 027	Control 026	Control 025	Control 024	Control 023	Control 022	Control 021	Control 019	Control 018	Control 017	Control 016	Control 015
0	0.0033	0	0	0	0.0713	0	0	0	0.0022	0	0	0	0	0	0	0	0
0	0	0	0	0	0	0	0	0	0	0	0	0	0	0	0	0	0
0	0	0	0	0	0.0013	0	0	0	0	0	0	0	0	0	0	0	0
0	0.0022	0.0009	0	0.0007	0.0135	0	0	0	0	0	0	0	0.0006	0.0016	0	0	0.0051
0	0	0	0	0	0.0008	0	0	0	0	0	0	0	0.0008	0	0	0	0
0	0	0	0	0	0.0331	0	0.0002	0	0.0141	0	0	0	0	0	0	0	0
0	0	0	0	0.0008	0.0376	0	0.0002	0.0004	0.0208	0	0	0	0	0.0007	0	0	0
0	0	0	0	0	0.0008	0	0	0	0	0	0	0.0004	0	0	0	0	0
0	0.0017	0	0	0	0.0368	0.0116	0.0002	0	0.0162	0.0013	0	0	0	0	0	0	0
0	0	0	0	0	0	0	0	0	0	0	0	0	0	0	0	0	0
0	0.0078	0	0.0071	0.0006	0.0947	0.0107	0	0	0.0059	0	0.0014	0	0	0	0	0	0
0	0	0	0	0	0	0	0	0	0	0	0	0	0	0	0	0	0
0	0	0.0004	0	0	0	0	0	0	0	0	0	0	0	0	0	0	0
0	0	0	0	0	0	0	0	0	0	0	0	0	0	0	0	0	0
0	0	0.0008	0	0	0	0	0	0	0	0	0	0	0	0	0	0	0
0.0007	0.0019	0	0.0008	0.0004	0.0431	0.0293	0.0002	0.0003	0.0128	0	0	0.0004	0	0.0003	0	0	0
0	0.0004	0	0.0009	0	0.079	0	0.0001	0.0003	0.0034	0.001	0	0	0	0	0	0	0
0	0	0	0	0	0	0	0	0	0	0	0	0	0	0	0	0	0
0.0059	0.0004	0.0058	0.0004	0.0006	0.0008	0.0123	0.0254	0.011	0.0415	0.0004	0	0.0003	0	0.0044	0.0003	0.0192	0
0	0	0	0	0	0	0	0	0	0	0	0	0.0001	0	0	0	0.0002	0.0002
0.0007	0	0.0163	0.0003	0.0033	0	0	0.0021	0.0008	0.0001	0	0.0932	0	0	0	0	0	0
0	0	0	0.0016	0.0664	0.0002	0	0.0003	1E-04	0.0005	0	0	0.0303	0.146	0.0003	0.0002	0.0006	0.0003
0	0	0	0	0	0	0	0	0.0001	0.0001	0	0	0.0001	0.0002	0	0.0004	0	0.0041
0.0033	0	0.03	0.0003	0.0054	0	0.0027	0.0045	0.0007	0	0.0003	0.0661	0	0.0003	0	0.0001	0	0
0	0.0018	0	0.0022	0.0005	0.0006	0.0097	0.0001	0.0004	0.0005	0.0016	0.0007	0.0003	0.0002	0.0002	0.0004	0	0.0002
0.0068	0.0004	0.0089	0.0005	0.0003	0.0003	0.0047	0.0295	0.0114	0.0355	0.0003	0	7E-05	0	0.0041	0.0003	0.0148	0.0003
0	0.0151	0	0	0.0001	0.0812	0.0134	0.0001	0.0002	0.0002	0.0002	0.0003	0	0.0006	0.0011	0	0.0001	0.0041
0	0	0	0	0	0	0	0	0	0	0	0	0	0.0003	0.0002	0.0005	0.0003	0.0004
0	0.001	0	0.0011	0.0003	0.0485	0.0072	0.0003	6E-05	0.0103	0.0006	0.0005	0	0	6E-05	0	0	0
0	0	0	0	0	0	0	0	0	0	0	0	0	0	######	0	0	6E-05
0	0	0	0	0	0	0	0	0	7E-05	0	0	0	0	0	0	0	0
4E-05	0.0015	4E-05	0.0013	0.0001	0.0229	0.0137	0.0002	0.0004	0.0243	0.0007	0.0004	2E-05	0.0001	0	3E-05	4E-05	5E-05
4E-05	0	0.0001	0	0.0001	0.0005	0.0008	0	0	0	9E-05	0	0.0001	0	######	9E-05	0	0
0	0.0002	8E-05	0.0008	0.0003	0.0004	0.0168	0.0002	0.0004	0.0002	0.0006	0.0004	0.2866	0.0004	0.0004	0.0014	0.0005	0.0006
0.0053	0	0.0173	0.0004	0	7E-05	0.0021	3E-05	5E-05	7E-05	0.0002	0.0805	3E-05	0.0001	5E-05	2E-05	6E-05	0.0002
0.0001	0	0	0	3E-05	0.0011	0	0	0	0	6E-05	0	0	0.0001	0	0	0	5E-05
0	0	0	6E-05	0	0	0.0014	0	0	0	0	0	0.0002	0.0004	9E-05	0.3198	0.0002	0.0003
0.0093	0	0.0003	0.0002	2E-05	6E-05	0.0004	0	0	0	4E-05	3E-05	2E-05	0	0	1E-05	0	0
0.0005	0.0009	0	0.0006	0.0002	0.0003	0.0084	0.0167	0.0002	0.0001	0.0004	0.0002	0.0003	0.0043	3E-05	6E-05	0.0016	0.0001
0.0001	0.001	7E-05	0.0009	0.0002	0.0444	0.0091	0.0001	0.0003	0.0206	0.0004	0.0003	3E-05	0.0001	6E-05	2E-05	1E-05	0
0	0	0	3E-05	1E-05	0	0.0006	0	2E-05	0	3E-05	0	0	1E-05	0	0	3E-05	1E-05
0.0001	0.0012	0.0001	0.0011	0.0003	0.0325	0.0103	0.0001	0.0002	0.0162	0.0005	0.0002	5E-05	0.0001	0.0017	4E-05	4E-05	0
7E-05	0.0015	3E-05	0.0009	0.0003	0.0427	0.0116	0.0001	0.0003	0.0151	0.0005	0.0004	5E-05	4E-05	5E-05	0	6E-05	0
0	0.2055	0	0.0004	0.0002	0.0002	0.0077	0.0001	0.0002	0.0002	0.0003	0.0002	0	0	0	0	0	2E-05
0.0001	2E-05	0.0537	0	6E-05	0	0.0004	4E-06	2E-05	0	0	0	7E-05	0.0368	0.0001	0.0002	0.0001	0.0002
0.0067	0.0004	0.0083	0.001	0.0005	0.0007	0.0236	0.0299	0.0123	0.04	0.001	0.0006	1E-04	0.0002	0.0047	0.0005	0.018	0.0002
0.0024	8E-05	0.029	0.0002	0.0066	1E-04	0.0031	0.0041	0.0014	4E-05	0.0001	0.0716	9E-05	8E-05	8E-05	0.0003	0.0001	0.0002

Control 014	Control 013	Control 012	Control 011	Control 010	Control 009	Control 008	Control 007	Control 006	Control 005	Control 004	Control 003	Control 002	Control 066	Control 031	Control 032	Control 020	Control 034
0	0	0	0.0059	0	0	0	0	0	0	0	0	0	0	0	0	0	0
0	0	0	0	0	0	0	0	0	0	0	0	0	0	0	0	0	0
0	0	0	0.0014	0	0	0	0.0012	0	0	0	0	0	0	0	0	0	0
0	0.0124	0	0	0	0	0	0.0006	0.0053	0	0.0009	0.0105	0.0185	0.0008	0.0009	0	0	0
0	0	0	0	0	0	0	0.0009	0	0	0	0	0	0	0	0	0	0
0	0	0	0.0498	0	0	0	0	0	0	0	0	0	0	0.001	0.0015	0	0
0	0	0.0051	0.0296	0	0	0	0.0005	0	0	0	0	0	0	0.0046	0	0	0
0	0	0	0.0011	0	0	0	0	0	0	0	0	0	0	0	0	0	0
0.0009	0	0	0.026	0.0009	0	0.0006	0	0	0	0	0	0	0	0.0023	0	0	0
0	0	0	0	0	0	0	0	0	0	0	0	0	0	0	0	0	0
0	0	0	0.0081	0	0	0	0.0015	0	0	0	0.0005	0	0.001	0.0117	0	0	0
0	0	0.0084	0	0	0	0	0	0	0	0	0	0	0	0	0	0	0
0	0	0	0	0	0	0.0004	0.0005	0	0	0	0.0004	0	0	0	0	0	0
0	0	0.0035	0	0	0	0	0	0	0	0	0	0	0	0	0	0	0
0	0	0	0	0	0.0006	0	0	0	0	0	0	0	0	0	0	0	0
0	0	0	0.0224	0.0005	0	0	0	0	0	0	0	0	0	0.0032	0	0.0005	0
0	0	0	0.004	0	0	0.0002	0	0	0.0004	0	0	0	0	0.0019	0	0	0
0	0	0	0	0.0002	0	0	0	0	0	0	0	0	0	0	0	0	0
0.0005	0	0	0.0003	0.0032	0.0006	0	0	0	0.0053	0.0002	0.0026	0.0003	0	0.0016	0.0147	0.0002	0.0029
0	0.0003	0.0017	0.0004	0	0	0.0002	0.0024	0	0	0	0	0.0003	0	0	0	0	0
0	0.0003	0.0102	0.0006	0.0002	0.0017	0.0007	0.0006	0.0009	0.0005	0.0002	0.0006	0.0005	0.0006	0	0.0016	0	0
0.0008	0.0002	0.0014	0.0003	0.0583	0	0.0004	0.0004	0.105	0.0007	0.0005	0.0003	0.0004	0	0	0.001	0.0114	0
0	0.0097	0	0.0117	0.0002	0	0	0	0.0078	0	0.0004	0.0003	0	0	0	0.0003	0.0003	0
0	0.0004	0.0156	0.0002	0.0006	0.0013	0.0003	0.0002	0.001	0	0.0001	0.0014	0.0009	0.0003	0.0003	0.0012	0.0005	0
0	0.0004	0	0	0	0	0	0	0	0	0	0	0	0	0	0	0	0
0.0002	0	0.001	0	0.0027	0	0.0001	0.0001	0	0.0052	0	0.0031	0	0	0.0011	0.0127	0	0.0017
0	0.0036	0.0016	0.0002	0.0004	0	0	0.0053	0.0004	8E-05	0	0.0015	0.0048	0.0006	0.0004	0.001	0	8E-05
0.0012	######	0	0	0	0	0	0	0	0	0	0	0	0	0	0	0.0004	0
0.0001	0.0001	0	0.0244	0.0003	0.0002	0	0.0002	0.0001	6E-05	0.0001	0	0	8E-05	0.0034	0.0002	0.0001	0.0003
0	0	0	0	0.0001	0	0	0	0	0	0	0	0.0002	0	0	0	0	0
0	8E-05	0	0	0	0	0	0	0	0	7E-05	0	0	0	6E-05	0	0	0
0	6E-05	0.001	0.026	0.0003	7E-05	0	0.0001	0.0003	0.0002	5E-05	0.0001	0.0004	0.0006	0.005	0	4E-05	7E-05
0	7E-05	0	0.0005	0	0	0	0.0001	0	0	0.0001	0	0.0004	0	0	0.0001	5E-05	0
0.001	0.0006	0.0031	0.0005	0.0541	0.0006	0.0002	0.2198	0.0171	0.0002	0.0001	0.0003	0.0004	0	0.0162	0	0.0009	0
0.0002	7E-05	0.0022	0.0005	0.0002	0.0005	0.0002	0.0007	0.0005	0.0129	0.0005	0.0061	0.001	0.0037	0.0003	0	0.0026	3E-05
0	4E-05	0	0.0002	0	0.0003	0	0	0	0.0001	0.0002	0	0	0	0	0	0	2E-05
0.001	0.0004	0.0032	0.0007	0.0008	0.3012	0.0004	0.0014	0.001	0.001	0.0008	0.0006	0.0014	0	0	0	0.0003	0
0.0001	0	0.0009	3E-05	0	0	4E-05	0	0	2E-05	8E-05	0	7E-05	0.0002	0.0002	8E-05	0	3E-05
0.0004	0	0.0036	0.0005	0.0005	0.0004	0.0003	0.0052	0.0014	0.0007	0.3432	0.0002	0.0241	0.0005	0.0004	0.0005	2E-05	0.0102
0.0002	7E-05	0.0003	0.0371	0.0002	0.0003	1E-04	0.0002	0.0002	0.0002	0.0001	0.0001	0.0001	0.0002	0.0042	7E-05	8E-05	9E-05
9E-05	2E-05	0	0	0	0	0	0	0	0	0	1E-05	0	0	4E-05	0.0238	0	0
0.0001	8E-05	0.0009	0.0202	0.0002	0.0002	0.0001	0.0001	0.0001	0.0006	0.0001	5E-05	0.0001	0.001	0.0131	0.0002	7E-05	9E-05
0.0002	2E-05	0.0015	0.0731	0.0002	0.0002	0.0001	0.0004	0.0002	0.0002	0.0001	6E-05	0.0002	0.0004	0.0022	3E-05	0.0001	4E-05
0	0	0	7E-05	0.0004	0	1E-05	0	0.0186	9E-05	3E-05	3E-05	3E-05	0	0.0003	0.0018	0	9E-05
0.0005	0.0003	0.0004	7E-05	6E-05	5E-05	7E-05	8E-05	0.0001	8E-05	1E-04	4E-05	5E-05	9E-05	0.0001	9E-05	1E-04	0.0568
0.0004	0.0002	0.0009	0.0001	0.0028	0.0002	0.0001	0.0002	0.0002	0.0051	0.0002	0.0028	0.0003	5E-05	0.0012	0.0124	0.0003	0.0021
0.0002	0.0002	0.0313	0.0007	0.0006	0.0008	0.0003	0.0008	0.0009	0.0007	0.0006	0.001	0.0006	0.0005	0.0009	0.0023	0.0006	0.0001

Control 051	Control 052	Control053	Control 054	Control 055	Control 056	Control 050	Control 057	Control 059	Control 060	Control 033	Control 062	Control 063	Control 064	Control 058	Control 049	Control 061	Control 047
0.0012	0	0.00112	0	0	0	0	0	0.004	0	0.1107	0	0	0	0	0.1261	0	0.0018
0	0	0.00112	0	0	0	0	0	0	0	0	0.0021	0	0	0	0	0	0
0	0	0	0	0	0	0	0	0	0	0	0	0	0	0	0.0013	0	0
0	0	0	0	0	0	0.0198	0.0009	0	0	0	0	0	0	0	0	0.0024	0
0	0	0	0	0	0	0.0006	0	0	0	0	0	0	0	0.0007	0.0012	0	0
0	0	0.00334	0.001	0	0.0069	0	0	0	0	0.048	0.0012	0.0073	0.0008	0	0.0434	0	0.0541
0	0	0.00624	0.0004	0	0.0042	0	0.001	0	0	0.0481	0.0045	0.0087	0	0	0.0607	0	0.0434
0	0	#######	0	0	0	0	0.0009	0	0	0	0	0	0	0	0	0	0
0	0	0.00164	0.0011	0	0.0069	0	0	0	0	0.0449	0.006	0.0039	0	0	0.041	0	0.05
0	0	0.00034	0	0	0	0	0	0	0	0	0	0	0	0	0	0	0
0.0008	0	0.00157	0.0043	0.0016	0.0021	0	0	0	0.0044	0.0153	0.0293	0.0246	0	0.0019	0.0103	0	0.079
0	0	0	0	0	0	0	0	0	0	0	0	0	0	0	0	0	0.0006
0	0	0	0	0	0	0	0	0	0	0	0	0	0	0	0	0	0
0	0	0	0	0	0	0	0	0	0	0	0	0	0	0	0	0	0
0.0027	0	0	0	0.0029	0	0	0	0.001	0	0	0.0008	0.0005	0.0019	0	0	0.0004	0
0	0	0.00426	0.0011	0.0006	0.0087	0	0	0	0	0.0428	0.0029	0.0058	0	0	0.0498	0	0.0589
0.0002	0	9.99E-04	0.0001	0.0004	0.0003	0	0.0003	0.0005	0.0004	0.11	0.0008	0.0017	0	0	0.0867	0	0.0258
0	0	0	0	0	0	0	0	0	0	0	0	0	0	0	0	0	0
0	0	0.00289	0.0043	0.0007	0.0058	0.0002	0	0.0009	0	0	0.0009	0.0046	0.0187	0.0031	0	0.0008	0.0066
0	0	0	0	0	0	0	0	0	0.0003	0	0	0	0	0	0	0	0
0	0	9.5E-05	0.0008	0	0	0	0	0.0063	0	0	0.0006	0.0372	0.0265	0.0002	0.0002	0.0015	0
0	0.0005	0	9E-05	0.0003	0.0213	0.0001	0	0	0	0.0003	0.0045	0	0.0001	0	0.0002	0	0
0	0	#######	0	0	0	0	0	######	0	0	0	0	0	0	0	0	0.0007
0	0	0	0.0013	0	0.0008	0	0.0003	0.0119	0	0.0003	0.0022	0.0068	0.0504	0.0009	0.0003	0.0047	0
0	0	0	0	0	0	0	0	######	0	0	0	0	0	0	0	0	0.2066
6E-05	0	0.00265	0.0019	0.0002	0.0068	0	0	0.0018	0.0002	0	0.0004	0.0033	0.0157	0.0041	0	0.001	0.0048
0	0	0	0	0.0005	0	0.0044	0	0	0	0.0055	0.0003	0	0.0003	0	0.0002	0	0
0	0	0	0	0	0	0	0	0	0.0002	0	0	0	0	0	0.0003	0	0.0007
0.0002	0.0004	0.002	0.0008	0.0007	0.0053	0.0003	9E-05	0.0002	0.0004	0.0421	0.004	0.0068	0	0.0002	0.0459	8E-05	0.056
0	0	0	0	0	0	0	0	0	0.0001	0	0	0	0	0	0.0002	0	0
0	0	0	0	0	0	0	0	0	0.0001	0.0007	8E-05	0	0	0	0.0002	0	0
0.0003	0.0001	0.00353	0.0026	0.0003	0.0019	0.0002	7E-05	0.0004	0.0005	0.0273	0.0034	0.012	5E-05	7E-05	0.0366	8E-05	0.0474
5E-05	0	0.00014	3E-05	0	0	6E-05	0.0004	0	0.0003	0	0	0	0	0	0.0003	0	0
0	0.0002	6.2E-05	0	0	0	0	0.0001	0	0	3E-05	0	0.0066	0	0	0	3E-05	0
0.0006	0.0011	0.00066	0.0005	0.0018	0.0004	0.0004	0.0009	0.016	0.0021	0	0.0154	0.0225	0.0199	0.0273	0.0003	0.0041	0
0.0001	0	1.9E-05	0	0.0004	3E-05	2E-05	7E-05	0	0	0	0	0.0001	0	0	3E-05	0	0
0	0	8.7E-05	0	9E-05	0	0	0	4E-05	0	0	0	0	0	0	0	0	0
7E-05	0.0003	0.00582	0.0001	1E-04	0.0182	0.0001	0.0003	0.0002	0.0005	0	0.0137	0.0003	0.0001	0.0017	0.0014	0.0001	0
0.0014	0.0005	1.7E-05	0.0005	8E-05	0.0191	1E-04	8E-05	0.0003	0.003	0.0003	0.0002	5E-05	0	8E-05	8E-05	0	2E-05
7E-05	0.0002	0.00471	0.0009	0.0005	0.0048	8E-05	0.0001	3E-05	0.0006	0.0484	0.0044	0.0103	6E-05	0.0001	0.0534	4E-05	0.0451
8E-05	0	1.5E-05	0	2E-05	1E-05	0	0	0	0	0	0	0	0	1E-05	2E-05	0	0
0.0002	0.0003	0.00841	0.0011	0.0004	0.007	0.0002	0.0002	3E-05	0.0005	0.0619	0.0101	0.0027	4E-05	0.0013	0.086	4E-05	0.0045
5E-05	0.0002	0.00182	0.0015	0.0003	0.0071	5E-05	0.0001	0.0002	0.0003	0.032	0.0037	0.0102	9E-05	6E-05	0.0317	4E-05	0.1053
4E-05	0	0	0	3E-05	0	0	2E-05	0	0	0.0002	0	0.0002	0	0	0	0	0.0005
0.0001	0.0814	0.00028	0.0005	0.0002	8E-05	0.0017	0.0001	0.0015	0.0003	0.0001	0.0004	0.0003	0.0001	0.0001	0.0002	0.0001	0.0002
0.0002	1E-04	0.00287	0.0032	0.0007	0.0073	1E-04	4E-05	0.0013	0.0002	6E-05	0.0006	0.0047	0.0187	0.0038	6E-05	0.0006	0.0053
0.0001	0.0002	0.00016	0.0014	0.0001	0.0007	7E-05	0.0003	0.0074	0.0004	0.0001	0.0026	0.0088	0.0515	0.0013	0.0003	0.0033	0

Control 048	Control 036	Control 037	Control 038	Control 039	Control 040	Control 035	Control 042	Control 043	Control04 4	Control 045	Control 046	Control 041	COVID -19-N-054	COVID -19-N-053	COVID -19-N-052	COVID -19-N-050	COVID -19-N-049
0	0	0	0	0	0	0	0	0	0.039155	0	0	0.0084	0	0	0	0	0
0	0	0	0	0	0	0	0	0	0	0	0	0	0	0	0	0	0
0	0	0	0	0	0	0	0	0	0	0	0	0	0	0.0042	0	0.0031	0
0	0.0005	0.0005	0	0.014	0	0.0235	0	0	0.00E+00	0.0018	0	0	0	0	0	0	0
######	0	0	0	0	0	0	0	0	0	0	0	0	0.001	0.0017	0	0.001	0.0016
0.0013	0.0009	0	0	0	0	0.0007	0	0	4.17E-02	0.0113	0	0.0218	0	0	0.0032	0.0053	0
0.0013	0	0	0	0	0.001	0.0007	0	0.0006	4.70E-02	0.0137	0	0.0159	0	0	0	0.0042	0
0	0	0	0.0005	0	0	0	0	0	0	0	0	0	0.003	0	0.0014	0.0027	0.0019
0.0024	0	0	0	0	0	0.0005	0	0.0012	0.030857	0.0051	0	0.0561	0	0	0	0.0076	0
0	0	0	0	0	0	0	0	0	0	0	0	0	0	0	0	0	0
0.0053	0	0	0.0011	0	0.0047	0.0036	0.0074	0.0048	0.035035	0.0126	0	0.0151	0	0	0.0036	0.0089	0
0	0	0	0	0	0.0008	0	0	0	0	0	0	0	0	0	0	0	0
0	0.0004	0	0	0	0	0	0	0	0	0	0	0	0	0	0.001	0	0
0	0	0	0	0	0	0	0	0	0	0	0	0	0	0	0	0	0
0	0	0	0	0	0	0	0	0	0	0	0	0	0	0.1757	0	0	0.001
0.0008	0	0.0003	0	0.0004	0.0004	0.0008	0	0.0009	0.033638	0.0059	0	0.0183	0.0005	0	0	0.0042	0
0	0	0.0002	0	0	0.0005	0.0006	0	0.0003	0.035377	0.003	0	0.0128	0	0	0	0.0038	0
0	0	0.002	0	0	0	0	0	0	0	0	0	0	0	0	0	0	0
0	0.0002	0.0002	0.0021	0.0018	0.0003	0.0015	0.0004	0	0.001589	0.0062	0.0084	0.0023	0	0	0	0	0
0	0	0	0	0	0	0	0	0	0	0	0	0	0	0	0	0	0
0.0008	0.0015	0	0.0006	0.0009	0	0	0	0	0.000518	0.0014	0	0	0	0	0	0	0
0.0003	0.0001	0	0.0201	0	0.0007	0.014	0.0007	0.0007	0.002485	0.0003	0.0476	0	0	0	0	0	0
0.0003	0	0	0	0.0007	0	0	0.8401	0.0004	0.014103	0.0009	0.0002	0.0007	0	0	0	0	0
0.0023	0.0048	0.0004	0.0015	0.0019	0.0002	0.0001	0.0016	0.0001	0.000369	0.0018	0	0	0	0	0	0	0
######	0	0	0	0	0	0.0006	0	0.0004	0.000433	0.0005	0.0005	0	0	0	0	0	0
######	0.0002	0.0014	0.0033	0.0019	0.0002	0.0007	0.0006	0.0003	0.002621	0.006	0.0102	0.0028	0	0	0	0	0
0	0.0002	0	0.0003	0.0084	0.0003	0.0292	0.0002	0	0.000999	0.0086	0	0	0	0.015	0.0163	0.0003	0
0	0	0	0	0	0	0	0.0006	0.0014	0.001101	0.001	0.0002	0.5639	0	0	0	0	0
######	0.0001	8E-05	0.0001	8E-05	0.0008	0.0012	0.0003	0.0004	0.034805	0.012	0.0002	0.0182	0	0	0.0007	0.0076	0.0002
0.0002	4E-05	0	0	0	0	0	0	0	8.21E-05	0	0	0	0	0	0	0	0
0	0	0	0	3E-05	0	0	0	0	0	0	0	7E-05	0	0.0001	0	0	0
0.0007	9E-05	4E-05	0.0004	2E-05	7E-05	0.0035	0.0006	0.0005	0.032674	0.0104	0.0004	0.0194	0	0.0001	0.0038	0.0037	0.0002
0	0	0	0	0	0	0	0	0	0	4E-05	0	0.0001	0.0025	0.0036	0.0021	0.0027	0.0071
0	5E-05	0	0	0	0	4E-05	0	0	0	0	0	0	3E-05	0	0	0	0
0.0004	0	0.0027	0	0	8E-05	3E-05	0	0	0	0	0	0.0057	0	0	0	5E-05	0
0	0	0	0.0001	0	0	0	0	0	0	0	3E-05	0	0.0027	0.0028	0.0025	0.0024	0.0019
0	6E-05	0	2E-05	0	0	0	0	0	0	4E-05	3E-05	0	5E-05	0	0	0	0
######	1E-05	4E-05	0	0.001	0.0017	0	0	0	0	0	0	3E-05	0	0	0	0	0
######	0.0003	0.0002	0.0003	0.0072	0.23	0.0029	0.0003	0.0012	2.44E-05	5E-05	0	3E-05	0.0004	0	0.0013	0	5E-05
0.0013	6E-05	0.0002	0.0003	4E-05	0.0004	0.0014	0.0005	0.0004	0.043533	0.0125	0.0002	0.0184	1E-04	0.0002	0.0011	0.0032	8E-05
0	8E-06	0	0	5E-06	3E-05	0	0	0	0	0	0	0	0.0001	4E-05	0.0154	2E-05	0
0.0019	0.0001	4E-05	0.0003	0.0001	0.0009	0.002	0.0006	0.0006	0.047707	0.0042	0.0001	0.0291	0.0002	0.0001	0.002	0.0019	0
0.0006	9E-05	5E-05	0.001	3E-05	0.0003	0.0011	0.0005	0.0005	0.032386	0.029	0.0003	0.0229	5E-05	0.0001	0.002	0.0043	0
0	0.0001	6E-05	0.0002	5E-05	0.0002	0.0002	0.0005	0.2724	0.000333	0.0005	0.0004	0.0004	0	0	0	0	0
0.0002	9E-05	0.0001	0.0144	4E-05	9E-05	0.0001	0.0002	0.0002	0.000106	0.0002	0.0001	7E-05	0	2E-05	0	0	0
7E-05	0.0002	0.0007	0.0034	0.0021	0.0002	0.0011	0.0004	0.0005	0.002842	0.0062	0.0137	0.0027	0	1E-05	0	8E-06	0
0.0022	0.0035	0.0002	0.0016	0.0016	0.0002	1E-04	0.0008	5E-05	0.000365	0.0024	7E-06	7E-06	0	0	0	2E-05	3E-05

COVID-19-N-047	COVID-19-N-046	COVID-19-N-045	COVID-19-N-044	COVID-19-N-055	COVID-19-N-043	COVID-19-N-048	COVID-19-N-056	COVID-19-N-064	COVID-19-N-059	COVID-19-N-060	COVID-19-N-061	COVID-19-N-062	COVID-19-N-063	COVID-19-N-065	COVID-19-N-066	COVID-19-N-067	COVID-19-N-070
0	0	0	0	0	0	0	0	0.0027	0	0	0	0.0012	0	0	0	0	0
0.0014	0	0	0	0	0.0028	0	0.013	0	0	0	0	0	0	0	0.0245	0	0
0	0.2424	0.0068	0	0	0	0	0	0	0	0	0	0	0	0	0	0.0029	0
0	0.0072	0	0.0421	0.001	0.0031	0.0528	0	0.0201	0	0.015	0	0	0	0	0.0027	0.001	0
0	0.3962	0	0	0	0.0007	0.0015	0.0011	0	0	0	0	0	0	0	0	0.0212	0
0	0	0	0	0	0	0	0	0.0022	0	0	0	0.0068	0	0	0	0	0
0	0	0	0	0.0014	0	0	0	0.0022	0	0	0	0.0066	0	0	0	0	0
0.0008	0.3903	0.0043	0	0.0014	0.0014	0	0.001	0.0015	0.0125	0	0	0	0	0	0	0.036	0.0015
0	0	0	0	0.0013	0	0	0.002	0.0032	0	0	0	0.019	0	0	0.0003	0	0
0	0	0	0	0	0	0	0	0	0	0	0	0	0	0	0	0	0
0	0.0005	0	0	0.0032	0.0007	0.0012	0.0008	0.0151	0	0	0	0.0118	0	0	0	0.001	0
0	0	0	0.0012	0	0	0	0	0	0	0	0	0	0	0	0	0	0
0	0.002	0.0037	0	0	0.3966	0.0009	0	0	0	0	0	0	0	0	0	0	0
0	0	0	0.0018	0	0.0869	0.0008	0	0	0	0	0	0	0	0	0	0	0
0	0.0006	0	0.001	0	0.0171	0.0007	0.0504	0.01	0	0	0	0	0.0338	0	0	0	0
0	0.0005	0	0	0	0	0	0.0005	0.0018	0	0	0	0.0033	0	0	0	0	0
0	0	0	0	0	0	0	0.0004	0.0005	0	0	0	0.0011	0	0	0	0	0
0	0.0003	0	0	0.0003	0.0003	0	0.0316	0.0002	0	0.0005	0	0	0.0116	0.0004	0	0	0.7939
0	0	0	0	0	0	0	0	0	0	0	0	0	0	0	0	0	0
0.0003	0	0	0	0	0.002	0	0	0.0004	0.001	0.0024	0.02	0	0.0245	0	0	0.0004	0.5328
0	0	0	0	0	0	0	0	0.0005	0	0	0	0	0	0	0.0001	0	0
0	0	0	0	0	0	0	0	0	0.0004	0	0.0226	0.0001	0.0014	0	0	0	0
0	0	0	0	0	0	0	0	0	0	0	0	0	0	0	0	0	0
0	0	0	0	0	0	0	0	0.0004	0	0	0	0	0	0	0.0003	0	0
0	0	0	0	0	0	0	0	0	0	0	0	0	0	0	0	0	0
0	0	0	0.0003	0	0	0	0	0	0	0	0	0	0	0	0	0	0
0	0.0022	0.0014	0.0092	0	0.0003	0.0531	0.0006	0.0162	0	0.0192	0.0018	0.0007	0.0001	0.0002	0.0001	0	0
0	0	0	0	0	0	0	0	0	0	0	0	0	0	0	0	0	0
1E-04	0	0.0006	0	0.0004	0	0.0002	0.0004	0.0035	0	0.0002	0	0.0055	0	0	0	0	0
0	0.0002	0	0.0001	0	0.0003	0.0001	0	0	0	0	0	0	0	0	0	0	0
0.0015	0.0002	0.0016	0.0005	6E-05	0.0031	0.0003	0.0099	0	0	0	0	5E-05	0	1E-04	0.0128	0	0
0	6E-05	0.0003	0	6E-05	4E-05	0.0002	0.0004	0.0014	0	0.0002	0	0.0032	0	0	0	0	0
0.0003	0.0957	0.0018	0.0002	0.0014	0.0002	0.0002	0.0014	0.0003	0.0001	0.0005	0	0	5E-05	0	0.0001	0.0022	0.0002
0	4E-05	0	0	0	4E-05	0	0	0	0	0	0	0	0	0	0.0009	0	0
0	3E-05	0	7E-05	0	0.0176	0.0001	0	0	0	0	0	0	0.0003	0	0	0	0
0.0007	0.3088	0.0048	0.001	0.0015	0.0014	0.0009	0.0013	6E-05	0.0001	0.0002	0.0003	0	8E-05	0	0	0.0544	0.0001
0	0	0	0	0	0.0001	0	0	0	0	0	4E-05	0	0	0	5E-05	0	0
8E-05	0.0002	0.0011	0.0003	0.0011	0.0002	0.0002	0.0003	0.0234	5E-05	0.0003	0.0155	0.0268	0.0002	0.0002	0.0013	7E-05	3E-05
2E-05	6E-05	0.0008	4E-05	0.0016	0.0031	0.0008	0.0006	2E-05	0.0155	4E-05	0.0009	3E-05	2E-05	4E-05	3E-05	0	0
0	0	0.0006	4E-05	0.0003	3E-05	3E-05	0.0002	0.0024	0	0	3E-05	0.0069	2E-05	3E-05	1E-05	2E-05	0
0	0	0	0	0.0055	0.0004	0	0.0017	8E-06	0.309	0.156	0	0	0	0	0	0.0002	0.0001
0	6E-05	0.0008	6E-05	2E-05	2E-05	6E-05	0.0007	0.0054	0	0	9E-05	0.0087	3E-05	3E-05	3E-05	0	0
2E-05	0	0.0002	3E-05	5E-05	2E-05	5E-05	0.0002	0.0032	4E-05	0	7E-05	0.0128	3E-05	0	2E-05	3E-05	5E-05
0	2E-05	0	0	0	0	0	0	3E-05	0	0.0001	5E-05	0.0022	0.0114	0	0.0006	2E-05	3E-05
0.0158	9E-05	0.0824	0.0001	1E-05	4E-05	2E-05	3E-05	0	0	0	0.0005	0.0281	0.0858	0.13	0.0003	0	0
0	0	0	0	0	0	0	0	0	0	0	0	0	0	0	3E-06	0	0
0	0	0	0	0	5E-06	0	0	0.001	1E-05	1E-05	0	4E-06	0	0	0.0003	8E-06	0

COVID-19-N-068	COVID-19-N-069	COVID-19-N-042	COVID-19-N-057	COVID-19-N-041	COVID-19-N-004	COVID-19-N-039	COVID-19-N-002	COVID-19-N-003	COVID-19-N-005	COVID-19-N-006	COVID-19-N-007	COVID-19-N-040	COVID-19-N-011	COVID-19-N-012	COVID-19-N-013	COVID-19-N-015	COVID-19-N-016
0	0	0	0	0	0	0	0	0	0.0028	0	0	0	0	0	0	0	0
0.1055	0	0	0	0	0	0	0	0	0.0022	0	0	0	0	0	0	0	0
0	0	0	0	0	0.6222	0.0019	0	0.0135	0.0015	0	0	0.0221	0	0.0031	0	0	0
0	0	0	0.008	0	0	0.048	0.0009	0.0363	0.032	0.0303	0.0283	0.0022	0.0744	0	0.0253	0.0257	0.0281
0	0	0	0	0	0.257	0	0	0.0175	0	0.0007	0	0.1984	0	0.0034	0	0	0
0.0055	0	0	0	0.0037	0	0	0	0	0	0	0	0.0021	0.0014	0	0	0	0
0	0	0	0	0	0	0	0	0	0	0	0	0	0.0032	0	0	0	0
0.0031	0	0	0.001	0.003	0.0008	0	0	0.0154	0	0	0	0.3104	0	0	0	0.0009	0
0.0006	0	0	0	0	0	0	0	0	0	0	0	0	0	0	0	0	0
0	0	0	0	0	0	0	0	0	0	0	0	0	0	0	0	0	0
0.0013	0.0022	0	0	0	0	0	0	0.0019	0	0	0	0	0	0	0	0	0
0	0	0	0	0	0	0	0	0.0036	0.0007	0.0008	0	0	0	0.0007	0	0	0
0	0	0.0016	0	0.0022	0	0	0	0	0	0	0.0003	0.0014	0	0	0	0	0
0	0.2378	0	0	0.0022	0	0	0	0	0	0.001	0	0.0024	0.0033	0.0007	0	0	0
0	0.2119	0	0	0.002	0	0.0007	0	0	0	0	0	0	0.0021	0	0	0	0
0.0022	0	0	0	0	0	0	0	0	0	0	0	0	0	0	0	0	0
0	0	0	0	0	0	0	0	0.0009	0.0008	0.0003	0	0	0.0006	0.0004	0	0	0
0	0.0002	0	0	0	0	0.0004	0	0	0	0	0	0	0	0	0	0	0
0	0	0	0	0	0	0	0.0003	0	0	0	0	0	0	0	0	0	0
0.0577	0.137	0.0003	0.0003	0	0	0	0	0	0	0	0	0	0.0009	0	0	0.0002	0
0	0.0032	0	0	0	0	0	0.0002	0	0	0	0	0	0	0	0	0	0
0	0	0	0	0	0.0004	0	0	0	0	0	0.0003	0	0	0	0	0.0004	0
0	0	0	0	0	0	0	0	0	0	0	0	0	0	0	0	0	0
0	0.003	0	0	0	0	0	0.0002	0	0	0.0002	0	0	0	0	0	0	0
0	0	0	0	0	0	0	0	0	0	0	0	0	0.0003	0.0002	0	0	0
0	0	0	0	0	0	0	0	0	0	0	0	0	0	0	0	0	0
0.0001	0.0001	0.0004	0.0006	0.0005	0.0002	0.032	0.0001	0.1607	0.0007	0.011	0.0177	0.0005	0.0515	0.0022	0.0193	0.0098	0.0009
0	0	0	0	0	0	0	0	0	0	0	0	0	0	0	0	0	0
0.0143	0.0006	0	0	0	0.0003	0.0001	9E-05	0	0.0001	0.0002	0	0	0	0.0006	0.0001	0	0
0	0	9E-05	0	0.0003	0	0.0001	0	0	0	0	0	0.0004	0	0	0	0	0
0.0818	6E-05	0.0002	0	0.0003	0	0.0001	0	0.0002	0	0	4E-05	0.0002	0.0004	0	9E-05	7E-05	0
0.0003	6E-05	0	0	0	0.0001	0	0.0001	0.0005	0.0003	6E-05	0.0001	0	9E-05	6E-05	0.0003	0	0
0.0001	0.0005	0.0002	0.0004	0.0017	0.7832	9E-05	0	0.0051	0.0016	0.0019	0	0.0114	0	0.0016	0.0008	0.0009	0
8E-05	4E-05	0	5E-05	0	0	0	0	0	0	9E-05	6E-05	0	0.0007	0	5E-05	0.0002	0
0	0	0	0	0	0	5E-05	0.0004	0	0	5E-05	0	8E-05	0	0	5E-05	0	0
3E-05	0.0001	0.001	0.0002	0.0026	0.001	0.0008	0	0.0091	5E-05	3E-05	0	0.3377	0	0.0001	0	0.0005	4E-05
0	0	0	0	0	0	0	0	0	5E-05	6E-05	0	0	0	0	0	0	0
0.0073	0.0157	3E-05	6E-05	0	3E-05	0.0002	0.0003	0	0	0	0.0002	0.0003	0	0	0	0.0001	0.0002
0	0	3E-05	0.0008	0	0.0029	9E-05	2E-05	0	0	0.0006	1E-05	0	4E-05	0	3E-05	0	0.014
0	0.0003	2E-05	0	7E-05	0.0001	8E-05	0.0002	0.0001	0.0002	6E-05	4E-05	0	0.0004	0.0002	5E-05	0	0
6E-05	0.0001	0	0.2742	0	0	3E-05	0	0	0	0	0	0	0	0	0	0	0
0	0.0016	2E-05	0	7E-05	7E-05	3E-05	9E-05	6E-05	6E-05	9E-05	0.0001	5E-05	0.0002	3E-05	2E-05	0	3E-05
3E-05	0.0015	0	3E-05	0	6E-05	5E-05	4E-05	0.0001	0.0002	6E-05	1E-05	0	0.0006	0.0002	7E-05	0	2E-05
3E-05	0	0	0	0	0	0	0	0	0	0	0	0.0003	0	0	0	0	1E-05
0	0	5E-05	0	0.0004	0	0.0002	9E-05	0	4E-05	1E-05	0.0002	9E-05	5E-05	0	0	0	0.0001
0	0	0	0	0	0	0	0.0001	0	0	0	0	2E-05	0	0	0	0	0
0.0024	0.0062	0	0	3E-05	7E-06	0	0.0002	0	0	0	0	2E-05	4E-05	8E-06	9E-06	0	0

COVID-19-N-017	COVID-19-N-010	COVID-19-N-019	COVID-19-N-018	COVID-19-N-038	COVID-19-N-036	COVID-19-N-032	COVID-19-N-030	COVID-19-N-034	COVID-19-N-028	COVID-19-N-027	COVID-19-N-024	COVID-19-N-023	COVID-19-N-021	COVID-19-N-029	COVID-19-C-001	COVID-19-C-051	COVID-19-C-037
0	0	0	0	0	0.0027	0	0.0022	0	0.0034	0	0	0	0.1509	0	0.0273	0.0933	0.0147
0	0	0	0	0	0.0728	0	0.0996	0.0135	0.1855	0.0487	0.1941	0.0245	0	0.0118	0	0	0
0	0	0	0	0	0	0	0	0.0015	0	0	0	0	0	0.0033	0	0.0009	0
0.0056	0.0288	0.0133	0.0426	0.0427	0	0.0024	0	0	0	0.0012	0	0	0	0	0	0	0
0	0	0	0	0.001	0	0	0	0.0031	0	0	0	0	0	0.0014	0	0.0019	0
0	0	0	0	0	0.0027	0.0012	0	0.0045	0.003	0.0023	0	0.0163	0.0943	0.0049	0.0462	0.0481	0.0295
0	0	0	0	0	0.001	0	0.0007	0.0049	0.0012	0	0	0	0.0628	0.0027	0.0332	0.0532	0.0306
0	0.0016	0	0	0.0015	0	0.0009	0	0	0	0.0012	0	0	0	0.0087	0	0.0026	0
0	0	0	0	0	0.002	0	0	0.0138	0	0	0	0.0054	0.0813	0.01	0.0581	0.04	0.0229
0	0	0	0	0	0	0.0017	0.9682	0.0027	0.0037	0	0.0006	0.0096	0	0.002	0	0	0.0016
0	0	0	0	0	0.0038	0.0008	0	0	0	0.0047	0	0	0.0058	0.0039	0.0156	0.0572	0.0531
0	0	0	0	0	0	0.0007	0	0.001	0	0	0	0.0047	0.001	0	0	0	0
0	0	0.0015	0	0.0006	0.1321	0.333	0.0005	0	0	0.0009	0.0005	0.0021	0	0.0017	0	0.003	0
0	0	0	0	0.0014	0.2249	0	0	0	0.001	0.0613	0.0012	0.0476	0	0	0	0	0
0	0.0025	0.0033	0	0	0.1881	0.0527	0.0004	0.0009	0	0.0007	0.0141	0.0038	0	0.0007	0.078	0	0.0098
0	0	0.0005	0	0	0.0018	0.0005	0	0.0059	0.0007	0.0038	0	0.0036	0.0829	0.009	0.0508	0.0403	0.0213
0	0	0	0	0	0.0004	0	0	0.0032	0.0028	0.0008	0	0.0161	0.1604	0.0004	0.0191	0.0881	0.0251
0	0	0	0	0	0.0289	0	0.055	0.0004	0	0	0	0	0	0	0	0	0
0	0	0	0	0	0	0	0	0	0	0	0	0	0	0	0	0	0
0	0	0	0	0	0	0.0499	0.0004	0	0	0	0.0044	0	0	0.0003	0.0119	0	0.0006
0	0	0	0	0	0.0003	0	0	0	0	0	0	0	0	0	0.0003	0	0
0	0	0	0	0	0	0	0	0	0	0.0014	0.0149	0	0	0	0	0.0006	0.0005
0	0	0	0	0	0	0	0	0	0	0	0.0004	0	0	0	0	0	0
0	0	0	0	0	0	0	0	0	0	0.0003	0	0	0	0	0.0002	0	0
0	0	0	0	0	0	0	0	0	0	0	0	0	0	0	0	0	0.0011
0	0	0	0	0	0	0	0	0	0	0	0	0	0	0	0	0	0
0.0048	0.005	0.0067	0.0046	0.0145	0.0056	0.01	0.006	0.0004	0.0029	0.0441	0.0049	0.0231	0	0.0018	0.0006	0	0.0003
0	0	0	0	0	0	0	0	0	0	0	0	0	0	0	0	0	0
6E-05	0	0	0	0	0.0029	0.0001	0.0003	0.0133	0.0028	0.0026	9E-05	0.0101	0.0907	0.0145	0.0411	0.0473	0.0303
0	0	0	0	7E-05	0.0704	0.0894	0.0964	0.1298	0.0096	0.0646	0.0609	0.1191	0	0.0969	0	0	0.0012
0	0	0	0	0.0001	0.0708	0.0006	0.0629	0.0047	0.2438	0.0367	0.2089	0.0229	0	0.0186	0	6E-05	0.0008
0	0.0001	0	0	5E-05	0.0046	0.0008	0.0002	0.0171	0.0033	0.0044	0.0002	0.0098	0.0415	0.0012	0.0426	0.0354	0.0394
0	0.0003	0	0	0.0003	0.0001	0	0.0002	0.0076	8E-05	0.0006	0	0.0002	0.0009	0.0017	0	0.0014	0
3E-05	0	5E-05	6E-05	0	0	0	3E-05	0	0	0	0	0	0	0	0	0	0
0	0	0	0	9E-05	5E-05	0	0	0	6E-05	0	0	0	0	0	0.0001	0	8E-05
0	7E-05	0	0	0.0009	0.0006	8E-05	0	0	6E-05	0.0007	0	0.0001	0	0.0047	0	0.0019	0
0	0	0	0	3E-05	0	0	0.0001	0	0	0	0	0	0	0	0.0001	0	0
0.0003	0	0	0.0002	1E-04	0.0714	0.0043	0.0093	0.0168	0.2738	0.0206	0.0005	0.0536	4E-05	0.0311	6E-05	0	0.0008
0.0001	0	0	3E-05	0.0001	0	0	0	0	7E-05	3E-05	0	0.0002	0	0	0	5E-05	0
6E-05	4E-05	0.0001	4E-05	8E-05	0.0022	0.0006	0.0001	0.01	0.0017	0.0021	0.0002	0.0084	0.0726	0.0076	0.0407	0.0469	0.0237
0	0	0	0	0	0.0029	0	6E-05	0	0.0013	0.0073	0.0215	0.0002	0	2E-05	0	6E-05	8E-05
5E-05	8E-05	8E-05	0	7E-05	0.0035	0.0004	0.0002	0.0197	0.0038	0.0044	9E-05	0.0138	0.021	0.0089	0.0786	0.057	0.0127
8E-05	3E-05	0.0001	6E-05	3E-05	0.0017	0.0003	0.0003	0.0125	0.0022	0.0036	1E-04	0.0111	0.0648	0.0051	0.0214	0.0325	0.0338
0	0	0	2E-05	0	0	0	0	0.0002	0	0	8E-05	0	0	1E-04	0	0	0
0.0003	3E-05	0	0.0004	8E-05	6E-05	0	0	2E-05	0.0001	8E-05	0	0	0	0	0.0001	1E-05	3E-05
0	0	7E-06	0	0	0	0	1E-05	0	0	0	0	0	0	0	4E-05	0	0
0	1E-05	7E-06	0	0	3E-05	0	0	0	2E-05	0	7E-06	0	0	9E-06	0.0001	0	6E-05

COVID-19-C-035	COVID-19-C-033	COVID-19-C-031	COVID-19-C-008	COVID-19-C-025	COVID-19-C-022	COVID-19-C-020	COVID-19-C-014	COVID-19-C-009	COVID-19-C-058	COVID-19-C-026	COVID-19-C-071
0	0.1434	0.01	0	0	0.0114	0	0	0	0	0.0045	0
0.1666	0	0.003	0	0	0	0	0	0	0	0.0274	0
0	0	0	0	0.0082	0.0072	0.0012	0	0	0.0489	0	0
0	0.0058	0.005	0.0137	0.01	0.003	0	0.0518	0.0336	0	0	0
0	0.0016	0	0	0	0	0	0.0044	0	0.0775	0	0
0.0117	0.0366	0.016	0	0.0034	0.0333	0.0009	0	0	0	0	0
0.0111	0.0248	0.0254	0	0	0.0529	0	0	0	0	0	0
0	0.0013	0	0	0	0	0	0	0	0.1794	0	0.0023
0.0074	0.0273	0.0327	0	0	0.0318	0	0	0	0	0	0
0.0029	0.0026	0.0026	0	0	0	0	0	0	0	0.0015	0
0.0067	0.0581	0.0214	0	0	0.0061	0	0	0	0	0	0
0.0585	0	0	0.0022	0.8923	0	0.0011	0.0034	0	0	0.0156	0
0	0.0023	0.0021	0	0	0	0	0	0	0	0.0012	0
0	0.002	0.0852	0.001	0.2315	0	0	0	0	0	0.0024	0
0.1213	0	0	0	0.0021	0	0	0	0.0011	0	0	0
0.0075	0.0334	0.0222	0	0	0.0395	0	0	0	0	0	0
0.0021	0.082	0.0375	0	0.0012	0.0093	0	0.0016	0	0	0	0
0.0724	0.0004	0.0009	0	0	0	0	0	0	0	0	0
0	0	0	0	0	0	0	0	0	0	0	0
0	0	0	0	0	0	0	0	0	0.0007	0	0.0006
0	0	0	0	0	0	0	0	0	0	0	0.0006
0	0	0	0	0	0	0	0	0	0.0003	0.0009	8E-05
0	0	0	0	0	0	0	0	0	0	0	0
0	0	0	0	0	0	0	0	0.0003	0	0	0.0009
0	0	0	0	0	0	0	0	0	0	0	0
0	0	0	0	0	0	0	0	0	0	0	0
0.0188	0.0191	0.0128	0.0035	0.0028	0.0004	0.0004	0.0038	0.0012	0	0.0095	4E-05
0	0	0	0	0	0	0	0	0	0	0	0
0.0091	0.0423	0.0305	0	0.0004	0.0287	0	0	0	0.0001	0.0004	0
0.0535	0.0013	0.0133	0	0.0033	0	0	0	0	0	0.1865	0
0.1579	0.0027	0.005	0	0.0075	0	0	0	0	0	0.0407	0
0.0109	0.0206	0.0355	0.0003	0.0002	0.0474	0.0134	0	0	7E-05	0.0007	0.0015
0	0.0006	0	0.0013	0.0032	0.0034	0.001	0.0039	0	0.0353	0	0.0003
0	0.0002	0	0	0	0	0	0	0	7E-05	0	0
0	0	0	0	0.0001	0	0	0	0	0	0	0.0105
0	6E-05	0	0	0	0	0	0	0	0.2459	7E-05	4E-05
0	0	0	0	0	0	0	0	0	0	0	0
0.0764	0.0829	0.0158	4E-05	0.0031	0	6E-05	0.0003	0.0003	8E-05	0.0017	0.1599
0	0	0.0002	0	8E-05	0	2E-05	0	3E-05	0.0002	0	0
0.0076	0.0349	0.0273	0.0001	0.0002	0.0306	3E-05	0.0002	3E-05	3E-05	0.0003	3E-05
5E-05	0.0006	6E-05	0	0.0001	0	0	0	3E-05	0.1592	0.0004	9E-05
0.0129	0.0407	0.0045	1E-04	0.0001	0.0174	0.0001	0.0005	6E-05	0	0.0004	0.0008
0.0064	0.0301	0.0341	9E-05	6E-05	0.018	0.003	0.0003	3E-05	0	0.0004	9E-06
0	0	0	0	0.0003	0	0	0	3E-05	0	0	1E-05
0	2E-05	9E-05	0	0	0	1E-05	0.0003	0.0004	0	6E-05	0
1E-05	0	0	0	0	0	0	0	0	0	0	0
0	0	0	0	0	0	0	0	2E-05	0	0	0.0015

Supplementary Table S9. the corresponding output value and POD valueof each optimal fungal marker in the train phase					
rownames(Data)	OTU4 (Cryptococcus_longus)	OTU925 (Cryptococcus_longus)	group	POD(Control)	POD(COVID-19)
Control001	0.001596	0	Control	0.781163435	0.218836565
Control003	0.021841	0.001186	Control	1	0
Control004	0.001327	0	Control	0.752659574	0.247340426
Control005	0.039171	0.001919	Control	1	0
Control006	0.001143	0	Control	0.748502994	0.251497006
Control007	0.001521	4.80E-05	Control	0.997354497	0.002645503
Control008	9.21E-04	5.00E-05	Control	0.991758242	0.008241758
Control009	0.001385	0	Control	0.721311475	0.278688525
Control010	0.020831	9.18E-04	Control	1	0
Control012	0.007054	3.54E-04	Control	1	0
Control013	0.00172	0	Control	0.763297872	0.236702128
Control017	0.003633	1.07E-04	Control	1	0
Control020	0.001999	0	Control	0.772357724	0.227642276
Control026	0.22768	0.010742	Control	1	0
Control027	0.183046	0.001724	Control	1	0
Control028	0.005524	1.23E-04	Control	1	0
Control030	0.007943	1.82E-04	Control	1	0
Control031	0.008824	4.04E-04	Control	1	0
Control033	4.90E-04	0	Control	0.704545455	0.295454545
Control034	0.016037	5.88E-04	Control	1	0
Control035	0.00835	2.35E-04	Control	1	0
Control036	0.001156	7.50E-05	Control	1	0
Control037	0.005697	4.96E-04	Control	1	0
Control038	0.026219	0.001255	Control	1	0
Control039	0.016014	7.50E-04	Control	1	0
Control040	0.001487	5.90E-05	Control	1	0
Control041	0.02144	0.001113	Control	1	0
Control042	0.002767	1.85E-04	Control	1	0
Control043	0.003691	1.08E-04	Control	1	0
Control044	0.021941	9.71E-04	Control	1	0
Control045	0.047657	0.002243	Control	1	0
Control046	0.104847	0.003625	Control	1	0
Control047	0.041203	0.001806	Control	1	0
Control048	5.57E-04	0	Control	0.75070028	0.24929972
Control049	5.32E-04	0	Control	0.723287671	0.276712329
Control050	8.26E-04	0	Control	0.661375661	0.338624339
Control051	0.001467	3.50E-05	Control	0.986449864	0.013550136
Control052	6.82E-04	0	Control	0.552560647	0.447439353
Control053	0.021993	9.49E-04	Control	1	0
Control054	0.024901	7.03E-04	Control	1	0
Control056	0.055479	0.00257	Control	1	0
Control058	0.028637	0.001422	Control	1	0
Control059	0.01036	6.58E-04	Control	1	0
Control060	0.001193	8.90E-05	Control	1	0
Control061	0.004863	3.23E-04	Control	1	0
Control062	0.004306	1.39E-04	Control	1	0
Control064	0.143657	0.005684	Control	1	0
Control065	0.063355	0.003267	Control	1	0
Control067	0.051195	0.002432	Control	1	0
Control068	0.022466	0.001585	Control	1	0
Control069	0.001359	0	Control	0.754985755	0.245014245
Control071	0.052091	0.002243	Control	1	0
Control074	0.037277	0.001365	Control	1	0
Control076	0.125911	0.005644	Control	1	0
Control077	0.033895	0.001345	Control	1	0
Control079	2.91E-04	0	Control	0.437994723	0.562005277

Control081	0.024164	9.28E-04	Control	1	0
Control082	0.004394	1.23E-04	Control	1	0
Control084	7.96E-04	0	Control	0.593830334	0.406169666
Control085	0.014115	5.18E-04	Control	1	0
Control086	0.001158	8.10E-05	Control	1	0
Control087	0.159349	0.007457	Control	1	0
Control088	5.37E-04	0	Control	0.72	0.28
Control090	0.004822	2.25E-04	Control	1	0
Control091	0.142871	0.007437	Control	1	0
Control092	0.213342	0.009251	Control	1	0
Control093	0.006833	4.14E-04	Control	1	0
Control095	0.070168	0.002917	Control	1	0
Control096	0.120873	0.006055	Control	1	0
Control097	0.002623	0	Control	0.752066116	0.247933884
Control098	0.014057	8.16E-04	Control	1	0
Control101	0.047108	0.001821	Control	1	0
Control103	0.035124	0.001666	Control	1	0
Control104	0.00273	1.87E-04	Control	1	0
Control106	0.040396	0.002242	Control	1	0
Control108	0.007686	2.70E-04	Control	1	0
Control110	0.012534	5.03E-04	Control	1	0
Control111	0.06849	0.003363	Control	1	0
Control112	0.109598	0.005485	Control	1	0
Control115	3.57E-04	7.50E-05	Control	0.986149584	0.013850416
Control118	0.328527	0.020622	Control	1	0
Control119	0.006889	2.62E-04	Control	1	0
Control121	5.14E-04	3.60E-05	Control	0.973544974	0.026455026
Control123	0.00685	0	Control	0.779816514	0.220183486
Control124	0.367673	0.018302	Control	1	0
Control126	0.020462	7.01E-04	Control	1	0
Control130	0.001559	0	Control	0.726688103	0.273311897
Control131	0.300897	0.014381	Control	1	0
COVID-19-002	7.84E-04	0	COVID-19	0.739612188	0.260387812
COVID-19-003	0	0	COVID-19	0	1
COVID-19-005	0	0	COVID-19	0	1
COVID-19-006	0	0	COVID-19	0	1
COVID-19-008	0	0	COVID-19	0	1
COVID-19-010	0	0	COVID-19	0	1
COVID-19-011	0	0	COVID-19	0	1
COVID-19-013	0	0	COVID-19	0	1
COVID-19-014	0	0	COVID-19	0	1
COVID-19-015	0	0	COVID-19	0	1
COVID-19-016	0	0	COVID-19	0	1
COVID-19-017	0	0	COVID-19	0	1
COVID-19-018	0	0	COVID-19	0	1
COVID-19-019	5.20E-05	0	COVID-19	0	1
COVID-19-023	0	0	COVID-19	0	1
COVID-19-024	0	0	COVID-19	0	1
COVID-19-025	0	0	COVID-19	0	1
COVID-19-026	0	0	COVID-19	0	1
COVID-19-027	0	0	COVID-19	0	1
COVID-19-028	0	0	COVID-19	0	1
COVID-19-029	0	0	COVID-19	0	1
COVID-19-030	7.30E-05	0	COVID-19	0	1
COVID-19-031	0	0	COVID-19	0	1
COVID-19-032	0	0	COVID-19	0	1
COVID-19-035	8.70E-05	0	COVID-19	0	1
COVID-19-037	0	0	COVID-19	0	1

COVID-19-038	0	0	COVID-19	0	1
COVID-19-040	1.39E-04	0	COVID-19	0	1
COVID-19-041	0	0	COVID-19	0	1
COVID-19-043	0	0	COVID-19	0	1
COVID-19-045	0	0	COVID-19	0	1
COVID-19-046	0	0	COVID-19	0	1
COVID-19-047	0	0	COVID-19	0	1
COVID-19-048	0	0	COVID-19	0	1
COVID-19-049	0	0	COVID-19	0	1
COVID-19-050	5.70E-05	0	COVID-19	0	1
COVID-19-051	0	0	COVID-19	0	1
COVID-19-052	0	0	COVID-19	0	1
COVID-19-054	0	0	COVID-19	0	1
COVID-19-055	0	0	COVID-19	0	1
COVID-19-057	0	0	COVID-19	0	1
COVID-19-058	0	0	COVID-19	0	1
COVID-19-061	0	0	COVID-19	0	1
COVID-19-062	0	0	COVID-19	0	1
COVID-19-063	0	0	COVID-19	0	1
COVID-19-064	0	0	COVID-19	0	1
COVID-19-065	0	0	COVID-19	0	1
COVID-19-069	0	0	COVID-19	0	1

Supplementary Table S10. the corresponding output value and POD valueof each optimal fungal marker in the test phase				
SampleID	OTU4 (Cryptococcus_longus)	OTU925 (Cryptococcus_longus)	POD(Control)	POD(COVID-19)
Control002	0.001953	7.70E-05	1	0
Control011	0.001023	0	0.751	0.249
Control014	0.003433	0	0.755	0.245
Control015	0.001677	0	0.755	0.245
Control016	0.144344	2.58E-04	1	0
Control018	0.038014	1.64E-04	1	0
Control019	0.001433	0	0.755	0.245
Control021	9.17E-04	0	0.738	0.262
Control022	0.004295	0	0.755	0.245
Control023	0.008039	0	0.755	0.245
Control024	0.330219	0.00105	1	0
Control025	0.103649	3.20E-04	1	0
Control029	0.004014	0	0.755	0.245
Control032	0.107035	5.69E-04	1	0
Control055	0.00588	0	0.755	0.245
Control057	3.18E-04	0	0.706	0.294
Control063	0.040175	6.90E-05	1	0
Control066	4.50E-04	0	0.747	0.253
Control070	0.014752	1.77E-04	1	0
Control072	0.179666	9.39E-04	1	0
Control073	0.064218	1.38E-04	1	0
Control075	3.75E-04	0	0.747	0.253
Control078	0.044153	2.55E-04	1	0
Control080	0.111461	2.88E-04	1	0
Control083	0.021916	2.23E-04	1	0
Control089	0.038256	1.64E-04	1	0
Control094	0.132913	4.17E-04	1	0
Control099	0.026437	9.00E-05	1	0
Control100	0.048837	4.90E-05	0.999	0.001
Control102	0.003178	0	0.755	0.245
Control105	0.081342	1.85E-04	1	0
Control107	0.271456	0.001014	1	0
Control109	0.714611	0.002582	1	0
Control113	0.02026	1.24E-04	1	0
Control114	8.77E-04	0	0.738	0.262
Control116	0.026886	2.60E-05	0.99	0.01
Control117	0.11366	2.82E-04	1	0
Control120	0.054056	2.49E-04	1	0
Control122	0.051722	1.30E-04	1	0
Control125	0.002861	0	0.755	0.245
Control127	0.003012	0	0.755	0.245
Control128	0.26702	0.001245	1	0
Control129	0.377366	0.002035	1	0
Control132	0.873676	0.0059	1	0
COVID-19-001	3.50E-04	0	0.747	0.253
COVID-19-004	0	0	0	1
COVID-19-007	0	0	0	1
COVID-19-009	0	0	0	1
COVID-19-012	0	0	0	1
COVID-19-020	0	0	0	1
COVID-19-021	0	0	0	1
COVID-19-022	0	0	0	1
COVID-19-033	0	0	0	1
COVID-19-034	0	0	0	1
COVID-19-036	0	0	0	1

COVID-19-039	0	0	0	1
COVID-19-042	0	0	0	1
COVID-19-044	9.50E-05	0	0	1
COVID-19-053	1.34E-04	0	0	1
COVID-19-056	0	0	0	1
COVID-19-059	0	0	0	1
COVID-19-060	0	0	0	1
COVID-19-066	3.30E-05	0	0	1
COVID-19-067	0	0	0	1
COVID-19-068	0	0	0	1
COVID-19-070	0	0	0	1
COVID-19-071	0	0	0	1

Supplementary Table S11. the corresponding output value and POD valueof each optimal fungal marker in the independent cohort				
SampleID	OTU4 (Cryptococcus_longus)	OTU925 (Cryptococcus_longus)	POD(Control)	POD(COVID-19)
Control002	0.001953	7.70E-05	1	0
Control011	0.001023	0	0.751	0.249
Control014	0.003433	0	0.755	0.245
Control015	0.001677	0	0.755	0.245
Control016	0.144344	2.58E-04	1	0
Control018	0.038014	1.64E-04	1	0
Control019	0.001433	0	0.755	0.245
Control021	9.17E-04	0	0.738	0.262
Control022	0.004295	0	0.755	0.245
Control023	0.008039	0	0.755	0.245
Control024	0.330219	0.00105	1	0
Control025	0.103649	3.20E-04	1	0
Control029	0.004014	0	0.755	0.245
Control032	0.107035	5.69E-04	1	0
Control055	0.00588	0	0.755	0.245
Control057	3.18E-04	0	0.706	0.294
Control063	0.040175	6.90E-05	1	0
Control066	4.50E-04	0	0.747	0.253
Control070	0.014752	1.77E-04	1	0
Control072	0.179666	9.39E-04	1	0
Control073	0.064218	1.38E-04	1	0
Control075	3.75E-04	0	0.747	0.253
Control078	0.044153	2.55E-04	1	0
Control080	0.111461	2.88E-04	1	0
Control083	0.021916	2.23E-04	1	0
Control089	0.038256	1.64E-04	1	0
Control094	0.132913	4.17E-04	1	0
Control099	0.026437	9.00E-05	1	0
Control100	0.048837	4.90E-05	0.999	0.001
Control102	0.003178	0	0.755	0.245
Control105	0.081342	1.85E-04	1	0
Control107	0.271456	0.001014	1	0
Control109	0.714611	0.002582	1	0
Control113	0.02026	1.24E-04	1	0
Control114	8.77E-04	0	0.738	0.262
Control116	0.026886	2.60E-05	0.99	0.01
Control117	0.11366	2.82E-04	1	0
Control120	0.054056	2.49E-04	1	0
Control122	0.051722	1.30E-04	1	0
Control125	0.002861	0	0.755	0.245
Control127	0.003012	0	0.755	0.245
Control128	0.26702	0.001245	1	0
Control129	0.377366	0.002035	1	0
Control132	0.873676	0.0059	1	0
HZ001	0	0	0	1
HZ002	0	0	0	1
HZ003	0	0	0	1
HZ004	0	0	0	1
HZ005	0	0	0	1
HZ006	0	0	0	1
HZ007	0	0	0	1
HZ008	0	0	0	1
HZ009	0	0	0	1
HZ010	0	0	0	1
HZ011	0	0	0	1
HZ012	0	0	0	1
HZ013	0	0	0	1
HZ014	0	0	0	1
HZ015	0	0	0	1
HZ016	0	0	0	1
HZ017	0	0	0	1
HZ018	0	0	0	1
HZ019	0	0	0	1
HZ020	0	0	0	1
HZ021	0	0	0	1
HZ022	0	0	0	1
HZ023	0	0	0	1
HZ024	0	0	0	1
HZ025	0	0	0	1
HZ026	0	0	0	1
HZ027	0	0	0	1
HZ028	0	0	0	1
HZ029	0	0	0	1
HZ030	0	0	0	1
HZ031	0	0	0	1
HZ032	0	0	0	1
HZ033	0	0	0	1
HZ034	0	0	0	1

HZ035	0	0	0	1
HZ036	0	0	0	1
HZ037	0	0	0	1
HZ038	0	0	0	1
HZ039	0	0	0	1
HZ040	0	0	0	1
HZ041	0	0	0	1
HZ042	0	0	0	1
HZ043	0	0	0	1
HZ044	0	0	0	1
HZ045	0	0	0	1
HZ046	0	0	0	1
HZ047	0	0	0	1
HZ048	0	0	0	1
HZ049	0	0	0	1
HZ050	0	0	0	1
HZ051	0	0	0	1
HZ052	0	0	0	1
HZ053	0	0	0	1
HZ054	0	0	0	1
HZ055	0	0	0	1
HZ056	0	0	0	1
HZ057	0	0	0	1
HZ058	0	0	0	1
HZ059	0	0	0	1
HZ060	0	0	0	1
HZ061	0	0	0	1
HZ062	0	0	0	1
HZ063	0	0	0	1
HZ064	0	0	0	1
HZ065	0	0	0	1
HZ066	0	0	0	1
HZ067	0	0	0	1
HZ068	0	0	0	1
HZ069	0	0	0	1
HZ070	0	0	0	1
HZ071	0	0	0	1
HZ072	0	0	0	1
HZ073	0	0	0	1
HZ074	0	0	0	1
HZ075	0	0	0	1

Supplementary Table S12. The levels of Ig-G antibody	
IgG levels	Number
0.78	Control-001
1.56	Control-002
0.74	Control-003
0.37	Control-004
0.38	Control-005
2.31	Control-006
10.13	SCR-001
11.39	SCR-002
18.75	SCR-003
25.48	SCR-004
47.26	SCR-005
23.75	SCR-006
18.86	SCR-007
41.96	SCR-008
54.28	SCR-009
313.62	SCR-010
13.03	SCR-011
17.21	SCR-012
29.31	SCR-013
27.98	SCR-014
13.86	SCR-015
341.49	SCR-016
39.75	SCR-017
23.57	SCR-018
15.64	SCR-019
10.11	SCR-020
15.26	SCR-021
57.27	SCR-022
31.58	SCR-023
19.64	SCR-024
25.16	SCR-025
48.14	SCR-026
11.27	SCR-027
22.74	SCR-028
87.76	SCR-029
308.06	SCR-030
43.96	SCR-031
45.64	SCR-032
37.46	SCR-033
21.57	SCR-034
327.93	SCR-035
13.67	SCR-036
451.37	Post-COVID-19-001
214.03	Post-COVID-19-002
524.97	Post-COVID-19-003
282.51	Post-COVID-19-004
973.87	Post-COVID-19-005
848.58	Post-COVID-19-006
147.04	Post-COVID-19-007
112.88	Post-COVID-19-008
420.79	Post-COVID-19-009
188.59	Post-COVID-19-010
230.16	Post-COVID-19-011
452.60	Post-COVID-19-012
336.60	Post-COVID-19-013
254.92	Post-COVID-19-014
247.17	Post-COVID-19-015
201.13	Post-COVID-19-016
122.54	Post-COVID-19-017
446.07	Post-COVID-19-018
448.21	Post-COVID-19-019
279.65	Post-COVID-19-020
312.12	Post-COVID-19-021
225.38	Post-COVID-19-022

Supplementary Table S13. the corresponding output value and POD valueof each optimal fungal marker in the SC group				
SampleID	OTU4 (Cryptococcus_longus)	OTU925 (Cryptococcus_longus)	POD(Control)	POD(SC)
Control002	0.001953	7.70E-05	1	0
Control011	0.001023	0	0.751	0.249
Control014	0.003433	0	0.755	0.245
Control015	0.001677	0	0.755	0.245
Control016	0.144344	2.58E-04	1	0
Control018	0.038014	1.64E-04	1	0
Control019	0.001433	0	0.755	0.245
Control021	9.17E-04	0	0.738	0.262
Control022	0.004295	0	0.755	0.245
Control023	0.008039	0	0.755	0.245
Control024	0.330219	0.00105	1	0
Control025	0.103649	3.20E-04	1	0
Control029	0.004014	0	0.755	0.245
Control032	0.107035	5.69E-04	1	0
Control055	0.00588	0	0.755	0.245
Control057	3.18E-04	0	0.706	0.294
Control063	0.040175	6.90E-05	1	0
Control066	4.50E-04	0	0.747	0.253
Control070	0.014752	1.77E-04	1	0
Control072	0.179666	9.39E-04	1	0
Control073	0.064218	1.38E-04	1	0
Control075	3.75E-04	0	0.747	0.253
Control078	0.044153	2.55E-04	1	0
Control080	0.111461	2.88E-04	1	0
Control083	0.021916	2.23E-04	1	0
Control089	0.038256	1.64E-04	1	0
Control094	0.132913	4.17E-04	1	0
Control099	0.026437	9.00E-05	1	0
Control100	0.048837	4.90E-05	0.999	0.001
Control102	0.003178	0	0.755	0.245
Control105	0.081342	1.85E-04	1	0
Control107	0.271456	0.001014	1	0
Control109	0.714611	0.002582	1	0
Control113	0.02026	1.24E-04	1	0
Control114	8.77E-04	0	0.738	0.262
Control116	0.026886	2.60E-05	0.99	0.01
Control117	0.11366	2.82E-04	1	0
Control120	0.054056	2.49E-04	1	0
Control122	0.051722	1.30E-04	1	0
Control125	0.002861	0	0.755	0.245
Control127	0.003012	0	0.755	0.245
Control128	0.26702	0.001245	1	0
Control129	0.377366	0.002035	1	0
Control132	0.873676	0.0059	1	0
SC001	0	0	0	1
SC002	0	0	0	1
SC003	9.50E-05	0	0	1
SC004	0	0	0	1
SC005	0	0	0	1
SC006	0	0	0	1
SC007	0	0	0	1
SC008	0	0	0	1
SC009	0	0	0	1
SC010	1.19E-04	0	0	1
SC011	0	0	0	1
SC012	0	0	0	1
SC013	5.70E-05	0	0	1
SC014	0	0	0	1
SC015	0	0	0	1
SC016	0	0	0	1
SC017	6.90E-05	0	0	1
SC018	0	0	0	1
SC019	1.27E-04	0	0	1
SC020	0	0	0	1
SC021	0	0	0	1
SC022	8.11E-04	0	0.727	0.273
SC023	0	0	0	1
SC024	0	0	0	1
SC025	0	0	0	1
SC026	0	0	0	1
SC027	0	0	0	1
SC028	0	0	0	1
SC029	1.12E-04	0	0	1
SC030	0	0	0	1
SC031	0	0	0	1
SC032	7.20E-05	0	0	1
SC033	0	0	0	1
SC034	0	0	0	1
SC035	5.70E-05	0	0	1
SC036	0	0	0	1

Supplementary table S14. The detailed values of oral fungal diversity index and observed OTUs among COVID-19 (n=71), SC (n=36), and control (n=132) groups.								
sample	reads	label	ace	chao	shannon	simpson	coverage	observed_otus
SC001	1161	0.97	23.337782	22.5	0.744831	0.742697	0.996555	21
SC021	1161	0.97	142.858466	131.47619	2.954111	0.12118	0.96727	98
SC022	1161	0.97	164.942887	188.625	2.854811	0.140528	0.966408	96
SC023	1161	0.97	298.763241	210.833333	3.066413	0.121707	0.950043	119
SC024	1161	0.97	189.314137	141	3.470174	0.054746	0.968992	96
SC025	1161	0.97	184.319068	178.130435	3.299303	0.083342	0.957795	127
SC026	1161	0.97	118.048673	118.076923	3.212779	0.080108	0.975883	89
SC027	1161	0.97	125.908879	101.8	2.067735	0.263737	0.975883	64
SC028	1161	0.97	85.520267	79	2.010759	0.265042	0.981051	58
SC029	1161	0.97	370.601756	242.464286	3.264262	0.133987	0.936262	146
SC030	1161	0.97	120.234917	114.0625	2.883208	0.109448	0.973299	85
SC031	1161	0.97	247.745845	168.368421	3.251912	0.078657	0.958656	109
SC032	1161	0.97	159.23226	145.5	2.719755	0.148338	0.968131	90
SC033	1161	0.97	229.813396	165.666667	2.362265	0.229858	0.965547	79
SC034	1161	0.97	226.327903	156.607143	3.227336	0.093207	0.959518	118
SC035	1161	0.97	195.668738	160.363636	3.026762	0.100172	0.966408	93
SC020	1161	0.97	193.058645	206.1	2.757988	0.14261	0.959518	98
SC019	1161	0.97	249.396647	216.555556	3.356164	0.10181	0.951766	131
SC036	1161	0.97	71.576529	65.75	2.650975	0.115676	0.984496	53
SC017	1161	0.97	55.018206	57.6	2.006656	0.252885	0.988803	42
SC002	1161	0.97	96.000942	66.375	2.207476	0.171545	0.983635	45
SC003	1161	0.97	145.480037	138.47619	2.803977	0.182451	0.96727	105
SC004	1161	0.97	32.367347	26	0.521475	0.725283	0.992248	14
SC005	1161	0.97	94.733923	105.857143	2.705951	0.122242	0.978467	63
SC018	1161	0.97	148.6576	148.714286	3.606634	0.065372	0.970715	122
SC007	1161	0.97	79.130041	86.5	2.769719	0.121211	0.983635	58
SC008	1161	0.97	28.878261	25.2	0.398204	0.857295	0.992248	18
SC006	1161	0.97	153.514751	152.157895	3.33475	0.066232	0.964686	109
SC010	1161	0.97	111.04198	124.142857	3.107374	0.082701	0.976744	74
SC011	1161	0.97	320.069029	116.428571	0.943448	0.702842	0.973299	50
SC012	1161	0.97	141.348866	146.352941	3.568843	0.072182	0.973299	119
SC013	1161	0.97	225.720371	166.954545	2.120882	0.387263	0.956072	109
SC014	1161	0.97	235.32522	201.105263	3.695497	0.058658	0.956072	134
SC015	1161	0.97	240.834414	155.714286	2.520885	0.139957	0.96124	85
SC016	1161	0.97	275.114698	212.956522	3.360533	0.105403	0.94832	136
SC009	1161	0.97	80.735766	74.230769	3.325197	0.048032	0.986219	65
COVID-19-052	1161	0.97	128.50878	99	1.650383	0.469497	0.976744	60
COVID-19-051	1161	0.97	187.602734	209.5625	3.660507	0.062593	0.956934	133
COVID-19-049	1161	0.97	848.249242	157	0.331303	0.885793	0.985357	21
COVID-19-048	1161	0.97	531.467374	339.125	2.70861	0.264882	0.919035	157
COVID-19-047	1161	0.97	101.777416	85.5	1.508825	0.448113	0.981051	47
COVID-19-046	1161	0.97	139.489599	118.214286	1.779718	0.335989	0.96727	68
COVID-19-038	1161	0.97	387.82118	269	2.398014	0.332885	0.933678	136
COVID-19-044	1161	0.97	613.555391	374.588235	2.374443	0.333259	0.922481	139
COVID-19-043	1161	0.97	268.371365	234.55	3.66058	0.050965	0.946598	140
COVID-19-042	1161	0.97	86.238635	77	1.031194	0.592878	0.982773	39
COVID-19-041	1161	0.97	220.26437	114.666667	2.846907	0.08374	0.973299	63
COVID-19-040	1161	0.97	235.77996	103.25	1.556162	0.37698	0.980189	40
COVID-19-039	1161	0.97	718.616042	403.157895	2.685407	0.258907	0.91559	153
COVID-19-053	1161	0.97	206.513438	174.875	3.017554	0.115036	0.96727	87
COVID-19-045	1161	0.97	184.473842	124.333333	2.1401	0.206212	0.970715	62
COVID-19-054	1161	0.97	33.936017	30.25	0.31763	0.896694	0.991387	19
COVID-19-063	1161	0.97	151.625555	147.428571	2.394678	0.197268	0.971576	72
COVID-19-056	1161	0.97	195.426883	147.571429	2.466214	0.205444	0.964686	89
COVID-19-037	1161	0.97	272.802393	205.8	3.553794	0.063068	0.94832	135
COVID-19-071	1161	0.97	94.584173	82.5	2.404128	0.179839	0.983635	54
COVID-19-070	1161	0.97	43.715735	42.166667	2.042991	0.202291	0.990525	33
COVID-19-069	1161	0.97	102.670873	86.75	2.833069	0.129892	0.982773	63
COVID-19-068	1161	0.97	84.514889	71.666667	2.630853	0.119444	0.985357	49
COVID-19-067	1161	0.97	51.730104	51	0.975967	0.565194	0.986219	27

COVID-19-066	1161	0.97	164.047733	124.75	2.987428	0.127041	0.969854	95
COVID-19-065	1161	0.97	372.916051	104	1.632065	0.333212	0.981912	34
COVID-19-064	1161	0.97	277.61828	196.65	2.386726	0.310738	0.950043	114
COVID-19-062	1161	0.97	106.576254	104.4	3.32563	0.061818	0.979328	86
COVID-19-061	1161	0.97	94.610612	90.714286	2.937261	0.09463	0.979328	71
COVID-19-060	1161	0.97	462.156197	266	1.823761	0.319221	0.94832	89
COVID-19-059	1161	0.97	333.901698	100	0.516028	0.824471	0.981912	30
COVID-19-058	1161	0.97	33.207015	32	1.222445	0.401215	0.992248	26
COVID-19-057	1161	0.97	301.417131	119.857143	0.928618	0.663082	0.972438	49
COVID-19-055	1161	0.97	89.077493	59.857143	1.117057	0.614781	0.984496	38
COVID-19-036	1161	0.97	219.751529	153.8125	3.357808	0.065045	0.963824	100
COVID-19-050	1161	0.97	316.391581	212.545455	2.770248	0.123633	0.955211	92
COVID-19-034	1161	0.97	107.938782	134	1.852562	0.378338	0.971576	68
COVID-19-015	1161	0.97	121.960285	124.0625	2.848519	0.103129	0.970715	89
COVID-19-014	1161	0.97	451.441662	337.75	2.865736	0.240612	0.925926	155
COVID-19-013	1161	0.97	419.519902	236.387097	2.602492	0.289792	0.933678	142
COVID-19-012	1161	0.97	152.157191	100	1.938388	0.384781	0.976744	61
COVID-19-011	1161	0.97	224.422011	216.115385	3.031965	0.197648	0.945736	141
COVID-19-010	1161	0.97	427.255455	318.789474	2.524395	0.316374	0.929371	144
COVID-19-009	1161	0.97	494.725222	319.5	2.761421	0.276614	0.921619	162
COVID-19-008	1161	0.97	386.676842	278.4	2.930965	0.138677	0.940568	122
COVID-19-007	1161	0.97	451.622772	288.956522	2.312381	0.366585	0.92851	141
COVID-19-006	1161	0.97	487.081935	306.105263	1.891302	0.465179	0.92851	127
COVID-19-005	1161	0.97	416.045656	264.142857	2.974694	0.193126	0.937123	139
COVID-19-004	1161	0.97	53.104915	51.5	0.859756	0.706987	0.98708	34
COVID-19-002	1161	0.97	217.174298	128.75	1.124585	0.54092	0.976744	41
COVID-19-001	1161	0.97	203.569711	162.105263	3.217839	0.091982	0.96124	110
COVID-19-035	1161	0.97	241.82044	162	3.080292	0.092937	0.957795	106
COVID-19-016	1161	0.97	270.579709	199.103448	2.594603	0.299152	0.947459	136
COVID-19-017	1161	0.97	218.41396	150.5	2.890446	0.123193	0.96124	101
COVID-19-003	1161	0.97	376.928122	254.111111	2.5696	0.279783	0.931955	140
COVID-19-019	1161	0.97	466.168334	235.894737	2.568414	0.221655	0.94143	116
COVID-19-018	1161	0.97	500.968322	283	2.710498	0.273871	0.925065	154
COVID-19-032	1161	0.97	162.833334	128.090909	3.05133	0.100125	0.972438	83
COVID-19-030	1161	0.97	176.953146	144.111111	2.456564	0.235057	0.969854	78
COVID-19-029	1161	0.97	103.245946	102.25	2.419849	0.236817	0.976744	73
COVID-19-028	1161	0.97	235.052693	176.5	1.834912	0.36759	0.964686	74
COVID-19-027	1161	0.97	191.150007	177.357143	3.0825	0.138241	0.955211	130
COVID-19-026	1161	0.97	163.484216	118.230769	1.353496	0.520929	0.968131	67
COVID-19-031	1161	0.97	209.395477	202.545455	3.587085	0.055074	0.950904	130
COVID-19-024	1161	0.97	130.767842	145.583333	3.022738	0.116359	0.969854	96
COVID-19-020	1161	0.97	96.967943	70.666667	2.671143	0.122271	0.985357	48
COVID-19-025	1161	0.97	210.754853	220.333333	3.04407	0.111144	0.94832	122
COVID-19-021	1161	0.97	172.474607	154.4	3.199116	0.09717	0.966408	105
COVID-19-033	1161	0.97	200.048146	222.352941	3.538085	0.068903	0.952627	135
COVID-19-022	1161	0.97	166.126034	168	2.991241	0.111667	0.971576	80
COVID-19-023	1161	0.97	324.443953	214.12	2.604299	0.210568	0.94143	123
Control083	1161	0.97	250.497894	227.25	3.419134	0.098505	0.951766	131
Control096	1161	0.97	178.409985	162.12	3.374096	0.06289	0.958656	117
Control094	1161	0.97	77.946138	72.909091	2.772583	0.115293	0.984496	59
Control093	1161	0.97	137.847812	83	0.271306	0.922439	0.986219	23
Control092	1161	0.97	139.985502	98	2.471787	0.123441	0.979328	52
Control090	1161	0.97	257.490748	177.045455	3.162196	0.079683	0.953488	112
Control089	1161	0.97	73.537462	79.666667	1.687042	0.404479	0.982773	48
Control088	1161	0.97	120.286762	67.5	1.389625	0.488166	0.983635	39
Control087	1161	0.97	126.962117	84.1	2.696581	0.145929	0.981051	61
Control086	1161	0.97	103.938776	95	2.012645	0.213327	0.978467	45
Control085	1161	0.97	292.997627	237.896552	3.458815	0.087888	0.940568	157
Control084	1161	0.97	256.064431	92.333333	1.034312	0.497006	0.982773	29
Control095	1161	0.97	206.637282	147.375	2.928213	0.0952	0.969854	73
Control091	1161	0.97	152.307424	101.071429	2.443229	0.188255	0.97416	70
Control076	1161	0.97	36.774656	35	1.751404	0.322276	0.993109	28

Control081	1161	0.97	97.82309	86	2.205264	0.261154	0.981912	56
Control080	1161	0.97	89.510452	92.25	3.022942	0.078344	0.981912	66
Control079	1161	0.97	226.686252	168.666667	2.591343	0.189894	0.965547	82
Control078	1161	0.97	147.722023	96.625	1.951543	0.349036	0.977606	56
Control077	1161	0.97	141.235226	135.058824	3.158188	0.100824	0.968992	98
Control075	1161	0.97	71.701698	56.6	2.510962	0.142266	0.988803	41
Control074	1161	0.97	41.042707	38	2.182768	0.219415	0.993971	35
Control073	1161	0.97	55.89319	54	1.683156	0.311148	0.989664	32
Control072	1161	0.97	63.251698	71.25	2.427898	0.147477	0.98708	45
Control071	1161	0.97	182.490922	149.375	2.552403	0.169763	0.969854	75
Control070	1161	0.97	107.671029	79.666667	2.272615	0.191708	0.981051	54
Control069	1161	0.97	157.723771	152.272727	2.690339	0.103313	0.963824	74
Control097	1161	0.97	137.666851	96.2	2.580427	0.133773	0.981051	50
Control068	1161	0.97	96.087629	88.333333	1.758392	0.391339	0.985357	43
Control082	1161	0.97	178.079118	167.935484	2.922534	0.19167	0.957795	130
Control098	1161	0.97	211.253769	154.875	2.178837	0.292463	0.96124	93
Control116	1161	0.97	75.814498	77.5	2.318262	0.240947	0.984496	52
Control100	1161	0.97	113.667758	95.083333	3.16343	0.074337	0.980189	74
Control067	1161	0.97	147.801943	140.2	1.813566	0.381645	0.975022	59
Control130	1161	0.97	199.474053	165.75	2.285088	0.207982	0.97416	57
Control129	1161	0.97	97.55556	72.090909	2.180812	0.198816	0.981912	53
Control128	1161	0.97	114.951615	69.1	1.902713	0.231159	0.981051	46
Control127	1161	0.97	67.283243	65	0.385624	0.896084	0.981912	35
Control126	1161	0.97	165.315906	138.866667	2.48526	0.220617	0.96727	92
Control125	1161	0.97	80.126864	79.6	1.489677	0.480916	0.979328	52
Control124	1161	0.97	206.159832	140.6	2.435497	0.189716	0.968131	74
Control123	1161	0.97	87.682168	115.2	0.984722	0.626667	0.976744	45
Control122	1161	0.97	97.616243	72	2.562433	0.111701	0.982773	53
Control121	1161	0.97	67.387778	72.75	0.359458	0.899413	0.983635	30
Control120	1161	0.97	182.419281	113.75	2.586502	0.14757	0.975022	63
Control119	1161	0.97	138.559826	98	1.665967	0.427619	0.976744	59
Control118	1161	0.97	90.577774	87.428571	2.219891	0.220958	0.978467	66
Control099	1161	0.97	154.673251	140.12	3.219959	0.112961	0.96727	112
Control117	1161	0.97	70.174464	62.363636	2.165975	0.238344	0.985357	50
Control114	1161	0.97	610.137824	310.473684	2.025692	0.42142	0.927649	127
Control113	1161	0.97	156.795172	122.142857	1.88887	0.329628	0.97416	60
Control112	1161	0.97	89.015108	84	2.830503	0.086192	0.980189	61
Control111	1161	0.97	123.767447	94.2	2.387957	0.136805	0.981051	48
Control110	1161	0.97	196.456922	139.235294	2.881161	0.114993	0.964686	91
Control109	1161	0.97	332.296971	116.5	1.401353	0.468256	0.983635	31
Control108	1161	0.97	373.92205	272.433333	3.273436	0.091161	0.92851	159
Control107	1161	0.97	106.20442	97.5	2.49073	0.138103	0.982773	50
Control106	1161	0.97	152.323888	182	2.749048	0.129735	0.962102	96
Control105	1161	0.97	111.040825	86.25	3.036538	0.073136	0.981912	60
Control104	1161	0.97	682.365975	398.037037	2.834972	0.234671	0.905254	176
Control103	1161	0.97	103.127162	102.6	1.951251	0.26949	0.980189	52
Control102	1161	0.97	226.802311	132	1.431463	0.46194	0.96124	77
Control101	1161	0.97	102.438804	76	2.797279	0.103325	0.983635	57
Control115	1161	0.97	60.032739	54	1.583295	0.42197	0.98708	39
Control066	1161	0.97	198.236722	180	3.079379	0.126982	0.953488	127
Control013	1161	0.97	268.306557	228.3125	3.314455	0.106653	0.950043	125
Control064	1161	0.97	231.205168	114.8	2.178124	0.247257	0.971576	62
Control029	1161	0.97	106.184557	88	2.890661	0.076768	0.981912	53
Control028	1161	0.97	250.715284	204.035714	3.603339	0.063906	0.950043	145
Control027	1161	0.97	461.362636	379.24	4.056134	0.047697	0.916451	193
Control026	1161	0.97	132.573564	89.666667	2.88151	0.089767	0.979328	59
Control025	1161	0.97	133.132313	62.2	1.842743	0.2628	0.985357	35
Control024	1161	0.97	346.557825	136.8125	2.885238	0.123646	0.963824	83
Control023	1161	0.97	199.826896	119	1.541679	0.458064	0.972438	57
Control022	1161	0.97	171.700977	98	1.651188	0.419121	0.975883	56
Control021	1161	0.97	63.543488	62.142857	2.592412	0.154108	0.988803	51
Control020	1161	0.97	138.905418	91.428571	0.747475	0.7634	0.977606	45

Control019	1161	0.97	114.043134	119	3.158632	0.075746	0.979328	73
Control018	1161	0.97	129.086128	122	2.870741	0.151686	0.971576	89
Control017	1161	0.97	229.172201	98	1.326716	0.397766	0.975883	44
Control016	1161	0.97	185.644686	223.333333	2.447286	0.131725	0.972438	58
Control015	1161	0.97	278.873177	228.666667	3.134304	0.147931	0.944014	142
Control014	1161	0.97	189.114359	144.2	2.815957	0.10165	0.975022	63
Control131	1161	0.97	72.521783	71.5	2.394603	0.156644	0.984496	46
Control012	1161	0.97	302.056335	222.071429	2.179614	0.260891	0.950043	104
Control011	1161	0.97	196.243984	122.8	2.698382	0.11555	0.971576	70
Control010	1161	0.97	307.407096	183	3.35518	0.059498	0.96124	93
Control009	1161	0.97	200.045282	99.75	1.526234	0.376948	0.975022	49
Control008	1161	0.97	92.614325	67.25	1.099694	0.420936	0.984496	29
Control007	1161	0.97	204.293264	154	2.429498	0.233694	0.959518	107
Control006	1161	0.97	183.199277	132.461538	2.823661	0.139974	0.968992	84
Control005	1161	0.97	154.664984	133.111111	2.727948	0.138568	0.972438	78
Control004	1161	0.97	245.659438	167.2	0.911305	0.729502	0.970715	55
Control003	1161	0.97	200.515224	164.666667	1.836512	0.428431	0.965547	78
Control002	1161	0.97	378.782573	227	2.191876	0.353077	0.939707	122
Control001	1161	0.97	177.997272	94.428571	1.767396	0.30397	0.972438	59
Control030	1161	0.97	156.4265	221.5	2.599654	0.128893	0.963824	78
Control031	1161	0.97	153.015447	149.333333	3.335041	0.076395	0.965547	106
Control032	1161	0.97	141.290336	118.909091	3.311081	0.058579	0.975022	82
Control033	1161	0.97	236.142838	209.315789	3.567941	0.069534	0.953488	134
Control063	1161	0.97	174.171798	148.583333	3.164894	0.073661	0.96727	90
Control062	1161	0.97	195.748629	151.117647	3.027117	0.107805	0.962963	98
Control061	1161	0.97	225.268676	234.125	2.829556	0.137077	0.959518	99
Control060	1161	0.97	165.55496	139.428571	2.411592	0.149719	0.971576	64
Control059	1161	0.97	165.531759	136	3.267342	0.081162	0.971576	88
Control058	1161	0.97	82.868001	72.857143	2.11927	0.252051	0.984496	51
Control057	1161	0.97	175.470426	98.625	2.640645	0.133016	0.977606	58
Control056	1161	0.97	285.706879	195.333333	3.662793	0.03446	0.962963	95
Control055	1161	0.97	149.302086	151.4	2.902043	0.108971	0.963824	94
Control054	1161	0.97	130.592023	101.857143	2.322021	0.196342	0.978467	59
Control053	1161	0.97	195.346513	159	1.86382	0.447339	0.965547	81
Control052	1161	0.97	168.87007	102.333333	2.074063	0.223672	0.972438	61
Control051	1161	0.97	177.038291	113.5	1.80514	0.420458	0.969854	71
Control050	1161	0.97	200.368186	199.055556	1.723382	0.49828	0.949182	104
Control065	1161	0.97	241.825829	167.625	2.551312	0.145116	0.966408	75
Control049	1161	0.97	272.137619	219.142857	3.212013	0.108676	0.947459	132
Control047	1161	0.97	210.930062	141.75	2.774164	0.131168	0.966408	80
Control046	1161	0.97	115.734121	109.25	3.014629	0.078958	0.97416	73
Control045	1161	0.97	351.799689	254.066667	3.489481	0.075347	0.946598	128
Control044	1161	0.97	168.277257	168.473684	3.268661	0.075702	0.960379	114
Control043	1161	0.97	170.892434	89.909091	0.94206	0.704604	0.975022	53
Control042	1161	0.97	225.877276	183.142857	3.184155	0.093588	0.955211	120
Control041	1161	0.97	204.134584	137.333333	2.846813	0.114629	0.970715	75
Control040	1161	0.97	117.461298	112.615385	1.851581	0.369993	0.971576	72
Control039	1161	0.97	205.686318	153	2.045832	0.41296	0.965547	88
Control038	1161	0.97	142.686778	105.1	2.829536	0.124422	0.976744	70
Control037	1161	0.97	91.568196	54.3	1.064402	0.619386	0.984496	39
Control036	1161	0.97	99.407298	69.111111	2.458787	0.121642	0.982773	48
Control035	1161	0.97	291.621806	222	2.609647	0.310248	0.944014	142
Control034	1161	0.97	109.55903	97.166667	2.380188	0.160608	0.980189	55
Control048	1161	0.97	129.307083	93	2.184379	0.27445	0.976744	66
Control132	1161	0.97	176.845816	137	1.084853	0.664035	0.968131	63

Supplementary table S15. The relative abundance and distribution of the key 47 OTUs among COVID-19 (n=71), SC (n=36), and Control (n=132) groups.									
	Control083	Control096	Control094	Control093	Control092	Control090	Control089	Control088	Control087
OTU3781 (Zanclospora_jonesii)	0.0030437	0	0	0.000225	0	0	0.0323432	0.0499302	0.0186875
OTU106 (Diversispora_spurca)	0	0.0208913	0	0	0	0.0034181	0	0	0
OTU69 (Candida_zeylanoides)	0	0	0.00039	0	0	0.0003212	0.0001415	0	0
OTU2542 (Cladosporium_spe_Incertae_sedis)	0.0175122	0	0.0987435	0	0	0	0.0001503	0	0
OTU231 (Ophiostoma_acarorum)	0.006496	0.0001634	0.0001852	0	0.0002977	0	0	0	0
OTU67 (Dioszegia_zsoltii_var._yunnanensis)	0	0.0001911	0	0	0.3726453	0.000794	4.09E-04	0	0.00E+00
OTU2466 (Zanclospora_jonesii)	0.0050951	0.00021	0	0	0	0.0002769	0.0614311	8.09E-02	0.0390997
OTU76 (Aureobasidium_pullulans)	0	0	0	0	0	0	0	0	0
OTU3895 (Blumeria_spe_Incertae_sedis)	0	0.0025251	0	0	0	0	0	0	0
OTU72 (Didymocyrtis_brachylaenae)	0	0	0	0	0	0	0	0	0
OTU3690 (Aspergillus_subflavus)	0	0.0026439	0	0	0	0	0	0	0
OTU1393 (Cryptococcus_longus)	0.0032741	0.016621	0.0154071	0.0011023	0.0253683	0.0006614	0.0050405	0	0.0203006
OTU80 (Zanclospora_jonesii)	0	0	0	0	0	0	0	0	0
OTU3193 (Candida_albicans)	0	0	0	0	0	0	0	0	0
OTU2845 (Acrodictys_fluminicola)	0	0.0003642	0.0004487	0.0002507	0.0007891	0.0005885	0.0001161	0.0004724	0.0001583
OTU65 (Milospium_graphideorum)	0	0.009563	0.1149836	0.0002967	0	0	0.0321355	0	0
OTU68 (Malassezia_globosa)	0	0.0002988	0	0	0.000191	0.001134	0	0	0.0148957
OTU3529 (Raffaelea_lauricola)	0.0002265	0.0035157	0	0	0	0.0004762	9.948E-05	9.948E-05	7.831E-05
OTU52 (Diaporthe_kochmanii)	0	0.0003407	0.0002819	0.0003428	0.0004584	0.0004746	0.0002251	0	0
OTU94 (Zanclospora_jonesii)	0.0005337	0.0691796	0.0031844	9.282E-05	0.000257	0.0005801	1.13E-02	0	8.14E-04
OTU34 (Acrodictys_fluminicola)	6.004E-05	0	0	0	0	0	0	0	3.695E-05
OTU8 (Acrodictys_fluminicola)	0.0237565	9.166E-05	0	0	0.0001253	0.0262451	6.874E-05	0	9.777E-05
OTU98 (Chrysosphaeria_jan-nelii)	0	0	0	0	0	0	0.00E+00	0.00E+00	0.00E+00
OTU58 (Gibberella_fujikuroi)	0	0	0	0	0	0	0.00E+00	0	0.00E+00
OTU3860 (Simplicillium_sympodiophorum)	0.012918	6.289E-05	0	0.0003358	0.0002516	0.0007927	3.32E-05	0.00E+00	4.272E-05
OTU3583 (Blumeria_spe_Incertae_sedis)	0	0.0153481	6.057E-05	7.119E-05	0.0001456	8.394E-05	0	0.0001105	0
OTU43 (Rhexothecium_globosum)	0	0	0	0	0	0	0	0	0
OTU71 (Aspergillus_subflavus)	7.231E-05	0	0	0.0002726	0.000216	0.0001681	0.0001221	0.0541212	0.00E+00
OTU3448 (Zanclospora_jonesii)	0.0002515	0.0005364	0.0003858	0.0004395	0.0004306	0.0007464	0.0005697	0.0001766	0.0165992
OTU3831 (Acrodictys_fluminicola)	0.0112305	0.0007969	8.38E-05	9.328E-05	0.0001241	0.002812	5.376E-05	4.19E-05	0.0003668
OTU56 (Penicillium_melanoconidium)	0	0	0.0001744	0	0	0	0.00E+00	0	0.00E+00
OTU26 (Zanclospora_jonesii)	0.0176065	0.00489	0	6.836E-05	8.93E-05	0	0.0536349	0.021072	0.0113443
OTU13 (Alternaria_alternata)	0.0121314	0.0001531	9.854E-05	0.000265	0.0002505	9.13E-05	0.0002004	2.45E-05	0
OTU31 (Arthrographis_grakistii)	0.0003246	0.0003548	0.0001701	0.0004145	0.000393	0.0001378	1.70E-04	0.0004689	0.000274
OTU21 (Saccharomyces_cerevisiae)	0	0	0	0	0	0	0	1.986E-05	0
OTU17 (Diversispora_spurca)	0.0001368	0.0006502	0	7.467E-05	0.0001128	0.0031826	4.464E-05	0.0001972	0.0004237
OTU18 (Acrodictys_fluminicola)	5.597E-05	2.496E-05	0	0	3.342E-05	0	1.611E-05	0.0001401	0.0001301
OTU2481 (Candida_albicans)	0	0	0	0	0	0	0.00E+00	0	0.00E+00
OTU9 (Candida_albicans)	0	0	0	2.114E-05	0	0	0	0	0
OTU22 (Hydnobolites_roseus)	0	0	0	0	0	0	2.4E-05	0	1.082E-05
OTU19 (Zanclospora_jonesii)	0.0004141	0	0	0	0	5.385E-05	5.449E-05	1.122E-05	2.18E-05
OTU7 (Cladosporium_halotolerans)	0.000511	0.0001668	0.0005176	0.0001918	0.0003869	0.000323	0.0002102	5.02E-05	0.0125844
OTU2920 (Cladosporium_cladosporioides)	0.001684	0.0078748	8.705E-05	1.907E-05	0.0397324	0.0010236	7.905E-05	5.29E-05	0.014872
OTU15 (Aspergillus_subflavus)	1.445E-05	0.0001261	0.0285602	8.371E-05	0.0002041	0.0329652	9.059E-05	2.454E-05	1.124E-05
OTU5 (Cryptococcus_longus)	2.56E-03	0.0157273	0.0158621	0.000913	0.027585	0.0006113	0.004646	6.974E-05	0.0208885
OTU3 (Zanclospora_jonesii)	5.23E-03	7.779E-05	6.304E-05	8.538E-05	9.957E-05	0.0004454	0.0677756	0.0746231	3.62E-02
OTU3900 (Acrodictys_fluminicola)	0.0101871	0.0012233	9.186E-05	0.0001385	0.0001924	0.0033047	9.729E-05	0.000102	0.0006411

Control086	Control085	Control084	Control095	Control091	Control076	Control081	Control080	Control079	Control078	Control077	Control075	Control074
0.0832103	0.001845	0	0.00018	0.0002905	0.0357264	0.0291604	0.0096956	0.0065169	0.182768	0.0040991	0.0246072	0.1508176
0.0001585	0	0	0	0	0	0	0	0	0	0	0	0
0	0	0	0.0001109	0	0	0	0	0	0	0	0	0
0	0.0001202	0	0.0001352	0.0002742	0	0.1024473	0	0	0	0.0001841	0	0
0	0.0010458	0	0	0.0002106	0	0	0.0001344	0	0.0188781	0.0022077	0	0
0	0	0	0.0005337	0.0001944	0	0	0	0	0	0	0	0
0.0366079	0.0044141	0	0	0.0002164	0.0707971	0.0560941	0.0145789	0.014913	0.06483	0.0103207	0.0349371	0.0409106
0	0	0	0	0	0	0	0	0	0	0	0	0
0	0.0146324	0	0	0	0	0	0	0	0.0004245	0	0	0
0	0	0	0	0	0	0	0	0	0	0	0	0
0	0.0143952	0	0	0	0	0	0	0	0.0005563	0	0	0
0.0002096	0.0013799	0	0.0074981	0.0201264	1.47E-02	0.0025148	0.0148547	0	0.0039518	0.0037477	0.0001606	3.64E-03
0	0	0	0	0	0	0	0	0	0	0	0	0
0	0	0	0	0	0	0	0	0	0	0	0	0
0.0001003	0.0002006	0.0010952	0.0003668	0.0001768	0	0	0.0001214	0	0.0001108	0.0001557	0	0
0	0	0	0.0005142	0.0001187	9.642E-05	0	0	0	0	0	0.1458829	0.0001088
0	0.0003306	0.0001249	0.0370799	0	0	0	0.0225933	0.0046656	0.0001053	0.0025863	0	0
0.0001884	0.0351274	0.0002392	0	0.0011472	0	0	9.101E-05	0.0004741	0	0.0002625	0	0
0	0	0	0.000286	0.0002758	0.0002697	0	0	0	0	0	0.1093856	0.0001927
6.783E-05	0	0.0002088	0.0001499	0.0012049	0	0.004641	0.0069115	0.0070865	0	0	0	0
0	0	8.775E-05	0	0	0	5.234E-05	0.0122175	0	0	0	0	0
0.0001848	0.000191	0.0002994	0.0011717	0.0001696	0	8.708E-05	0.0023862	7.18E-05	5.652E-05	0.0015918	0	0
0	0	0	0	0	0	0	0.00E+00	0	0	0	0	0
0	0	0	0	0	0	0	0	0	0	0	0	0
0.0001614	0.0028468	0	0.0001293	0.0022677	0	1.71E-04	6.764E-05	0.0082603	0.0001104	1.44E-04	0	0
7.863E-05	0.0175933	0	0	0	0	0	0	0	0.0056477	0.0035926	0	0.00E+00
0	0	0	0	0	0	0	0	0	0	0	0	0
0.00E+00	0	0.00E+00	0.0002658	0.0001798	0	0	4.788E-05	0	0	0.0054049	0	0
0.0002303	0.0002149	0.0005185	0.0082621	0.0004379	0	0.0002051	0.0002238	0.0003451	0.0002222	0.0001473	0	0
0.0002245	0.0079584	0	0.0001202	0.0089711	0	3.162E-05	7.352E-05	0.0009945	6.562E-05	3.478E-05	0	0
0	0	0	0	0	0	0.00E+00	0	0.00E+00	0.00E+00	0	0	0
0.071638	0.0055274	4.12E-04	0	4.742E-05	0.0413298	9.05E-02	0.0001109	3.79E-02	0.0001835	0.0184164	0.0462124	0.0899669
9.631E-05	0.0010733	3.118E-05	0.0001258	0.0001548	0	4.565E-05	5.066E-05	2.672E-05	0	0	0	0
0.000386	0.0004888	0.3537419	0.0001857	0.0002164	1.723E-05	0.0002794	0.0004075	0.0002331	0.0003327	0.0004937	0	0
0.0002066	0	0	0.0049106	0	0.0001031	0	0	0	0	0.001392	7.66E-05	5.39E-05
0.0002739	0.0002573	0.0002123	0.0013218	0.0042499	1.705E-05	0.0002256	0.0377933	0.0007309	0.0003247	0.0017585	0	0
0.0002891	9.221E-05	0.0001389	0	8.939E-05	0.00E+00	0.0001929	0.0002275	0.1622133	7.006E-05	0.0002706	0.0051564	0
0.00E+00	0	0	0	0	0	0	0	0	0.00E+00	0	0	0
0	0	0	0	0	1.364E-05	0	0	0	0	0	0	1.875E-05
0	0	0	0	0	0	0	0	0	0	0	0	0
0	0	0	0	0	0.0048048	1.346E-05	0	0	4.455E-05	1.987E-05	0	0
9.104E-05	0.0002828	1.40E-05	0.0092582	1.25E-04	0	0.0020694	2.869E-05	1.372E-05	1.278E-05	0.0001338	1.528E-05	0
5.629E-05	5.783E-05	0.0001627	0.0250103	8.582E-05	0	2.922E-05	0.0101444	0.0035526	3.168E-05	0.0038174	0	0
2.683E-05	4.541E-05	2.454E-05	0.0001195	0.0001952	0	2.729E-05	0.000117	2.041E-05	8.944E-06	0.060495	0	0.0004023
0.0001532	0.0018479	0.0001043	0.0091067	0.0186111	0.016333	0.0031557	0.0133854	4.234E-05	0.004916	0.0043959	3.714E-05	0.0048578
0.0347708	0.0028605	0.000446	3.092E-05	7.02E-05	0.0624976	0.0538584	0.017728	0.013417	0.06011	0.0108721	0.03469	0.0486237
0.0005399	0.0087431	0.0001407	0.0001577	0.0142248	9.922E-06	0.000129	0.0001013	0.0025878	6.979E-05	0.0001301	6.262E-06	1.849E-06

Control073	Control072	Control071	Control070	Control069	Control097	Control068	Control082	Control098	Control116	Control100	Control067	Control130
0.0141016	0.021183	0.017403	0.0323841	0.0079528	0	0.0034119	0.0048846	0	0	0	0.0007282	0
0	0	0	0	0	0	0	0	0	0	0	0	0
0	0	0	0.0003518	0.0003441	0	0.0001453	0	0.0001682	0	0.0002179	0	0
0	0	0	0	0	0	0	0	0.000139	0	0	0	0
0	0	0	0	0	0	0	0	0	0	0	0	0.0002505
0	0	0	0	0	0.0002306	0	0	5.07E-04	0	0.0002207	0	0
0.0228692	0.0259053	0.0444272	0.0069728	0.015645	0.000226	0.0064572	0.007361	0.00014	0	0	0.0033066	0
0	0	0	0	0	0	0	0	0	0	0	0	0
0	0	0	0	0	0	0	0	0	0.0005774	0	0	0
0	0	0	0	0	0	0	0	0	0	0	0	0
0	0	0	0	0	0	0	0	0	0.0010237	0	0	0.0001061
0.0064693	0.0214737	0.005925	0.0023869	0	0	0.0046513	0.0003538	0.0022943	0.0044689	0.0069184	0.0068449	0
0	0	0	0	0	0	0	0	0	0	0	0	0
0	0	0	0	0.0002343	0	0	0	0	0	0	0	0
0	0	0	0	0	0.0005648	0	0.0001715	0.0005357	0	0.0001346	0.0001293	0
0.0001805	0.0003412	0.0004129	0	0	0.0003115	0	0	9.642E-05	0	0	0	0.00E+00
0	0	0.0068306	0	0	0	0	0.0013201	0.0014009	0	0	0.0200609	0
0	0.0008424	0.0021886	0.0001397	0	0	0.0001206	0.0045253	0.0022415	0	0.0001143	0	7.408E-05
0	0	0.0002839	0	0	0.0006429	0	0	0.000217	0	0.2970574	0	0.0002637
0	0.0013602	0	0.0001107	0.0001678	0	0	0.0046731	0.0012084	0.0001499	0.0003356	0.000473	0
0	0	0	0	0	0	0	0	0	0.6983553	0	0	0.0003079
8.249E-05	0	0	0.0001299	0.000139	0	0.0014788	0.0001085	0	0.0040284	0.0010327	0.0011962	0
0	0	0	0	0	0	0	0	0	0	0	0	0.00E+00
7.442E-05	0	0	0	0	0	0	0	0	0.00E+00	0	0	0
0	0	0.0016281	0.000248	0.0009624	6.289E-05	0	0.0121229	0.0017871	5.34E-05	0	0	0
0.000152	0	0.003328	7.119E-05	0	0	0	0	0	0.0105983	0	0	9.351E-05
0	0	0.0001324	0	0	0	0	0	0	0	0	0	9.059E-05
0	0	0	0	0	0.0003215	0	0	0.0001564	0	0.0003195	0	0
0	0	0	0	8.221E-05	0.0010223	0	0.0009173	0.0004615	0	0.0002987	0	0
0	0.0011827	0.0051243	0.0001217	0.0038705	4.664E-05	0.0001059	0.0145975	0.0126456	7.431E-05	0	8.933E-05	0.0013281
0	0	0	0	0	0	0	0.0002833	0	0	0	0	0.000123
4.126E-05	0.0083438	0.0170454	0.019443	0.1116135	0.0001164	0.0114158	0.0098028	4.681E-05	0	2.587E-05	0.0053421	0
0	0	0	8.351E-05	0.0001943	0.000245	5.901E-05	0	0.0062246	0.0226907	0.0001208	0.000167	0.0001208
0	0	0	0	0	0.0004302	0	0.0003957	2.84E-04	0	0.0004092	0	0
0.0001243	0.0001277	0.0011707	0.0005352	0.000261	3.877E-05	8.747E-05	0	4.255E-05	0	2.222E-05	0.0091125	4.444E-05
0	0	0.0031306	5.763E-05	0.0014882	2.435E-05	0	0.0002293	0.0006802	0.0010308	0.0062771	0.0004829	0.0048595
0	0	0	0.0003833	0.0002267	0	0.000145	0.0001631	3.423E-05	0.0001321	0	0.0002674	0.1724677
0	0	0	0	6.774E-05	0	0	0	0	0	0	7.747E-05	1.402E-05
0	0	0	0	0.000227	0	0	0	0	0	0	1.432E-05	0
0	0	0	0	0	2.265E-05	0	0	1.318E-05	4.192E-05	0	0	0
0	0	0	0	2.532E-05	0	0	0	0	9.231E-05	0	0	0.0001292
0	0	0	5.051E-05	4.864E-05	0.0002547	0.0058661	0.0023622	0.0566226	9.977E-06	0.0002273	0.0003551	8.699E-05
0	0	8.766E-05	5.383E-05	2.491E-05	0.0002224	0	3.66E-05	8.09E-05	3.906E-05	0.0089977	6.49E-05	3.906E-05
5.825E-05	0	0	0.0721481	0.0002413	0.0001121	7.752E-05	5.573E-05	0.0001358	0.0050799	0.0001378	0.0001456	4.518E-05
0.0077905	0.0209748	0.0066852	0.0017549	0.0001778	0.0003305	0.0029321	0.0005758	1.85E-03	0.003146	0.0057438	0.0066573	0.0002042
0.020086	0.0318844	0.0350259	0.0080302	0.0132415	0.000144	0.0069339	0.0055822	8.945E-05	0.000171	0.0001086	0.0023738	1.375E-05
5.244E-06	0.0021107	0.0092288	0.0001545	0.002913	0.0001615	9.118E-05	0.0172855	0.0189922	5.553E-05	8.662E-05	0.0001076	0.0024327

Control129	Control128	Control127	Control126	Control125	Control124	Control123	Control122	Control121	Control120	Control119	Control118	Control099
0	0	0	0	0.0013541	0	0	0	0	0	0.0001759	0.0004582	0
0	0	0	0	0	0	0	0	0.0024205	0	0	0	0
0	0	0	0	0	0	0	0	0	0	0	0	0.0002026
0	0	0	0	0	0	0	0	0	0	0	0	0
0.0005338	0.0005229	0.0010167	0.0001126	0.0006318	0.0005556	0	0.0002505	0	0	0.0052106	0	0.0366995
0	0	0	0	0	0	0	0	0	0	0	0	0.0001186
0	0	0	0	0.0025937	0	0	0	0	0	0	0.0003055	0.00014
0	0	0	0	0	0	0	0	0	0	0	0	0
0.0004526	0.0004339	0	0	0	0	0	0	0	0	0.000103	0	0
0	0	0	0	0	0	0	0	0	0	0	0	0
0	0	0	0	0	0	0	0	0	0	0.0002409	0	0
0.0509571	0.0376673	0.0006967	0.0017718	0	0.0497024	0	0.0062543	8.709E-05	0.0058651	0.0007212	0.056139	0.0028904
0.0003178	0	0	0.0001993	0.0004121	0.0001185	0	0.0002101	0	0	0	0	0
0	0	0	0	0	0	0	0	0	0	0	0	0
0	0	0	0	0	0	0	0	0	0	0	0	0.0008129
0.0003239	0	0	0.0002299	0.0001904	0.0001038	0	0.0003832	0	0	0	0.0002225	0.0002472
0.0529136	0.0003331	0	0.0303375	0.0003625	0.0021185	0	0.024871	0.0001004	0	0.001996	0.011195	0.0024369
0	0	0	0.0002857	0	0	0	0	0.0001905	0	0.0001799	0	0.0011028
0.0008822	0.000574	0.0015596	0.0005354	0.0004949	0.0004056	0.0045552	0.0012595	0	0	0	0	0.0002758
0	0	0	0	0.0001535	0.0004605	0	0	7.14E-05	0.0002053	6.783E-05	0.0008229	0.0008497
0.0003556	0.0008652	0.0002063	0.0001293	0.0003664	0	0	0.000525	0.0007051	0.0007113	0.000565	0.0021969	0
0.000113	0.0004048	0	0	0.0001069	2.53E-02	0.0007165	0	5.5E-05	0.0001879	0.0001192	0	5.041E-05
0	0	0	0	0	0	0	0.0010226	0	0	0	0	0
0	0	0	0	0	0	0	0	0	0	0	0	0
8.9E-05	0.0002979	0	0.0053613	0	0.0002575	0	0	4.272E-05	0.0001448	4.628E-05	0.0003857	0.0029346
0.0007343	0	0	9.245E-05	0.0001658	5.313E-05	0.0005069	0.0003315	7.544E-05	0.000934	0.0013612	0.013479	0
0.0001314	0.0001264	0	1.42E-04	0.0001453	0	0.0009159	6.67E-05	4.082E-05	0	3.086E-05	0	0
0	0	0	0	0	0	0	0	0	0	0	0	0
0	0	0	0	0	0	0	0	6.267E-05	0	0.0001009	0	0.0005909
0	0	0	0.0116408	0.0001352	0.0006403	0	5.455E-05	6.878E-05	0.0010325	4.664E-05	0.0002585	0.0115863
0.0003238	0.0025157	0.7473191	8.64E-05	1.98E-04	0.000193	0.0017381	0.0004803	0	0	0	0	0.00E+00
0	0	0	0	0	0	0	4.003E-05	4.865E-05	0	0	0	5.173E-05
0.086501	0.0003669	0.0007371	0.0057386	0.0004448	0.0001921	0.0007822	0.0607756	0.0003051	0.0005138	0.0002405	0.0006497	5.82E-03
0	0	0	0	0	0	0	0	0	0	2.207E-05	0	1.13E-04
0	0	0	0	0.0060236	0	0	5.579E-05	1.513E-05	0.0028922	0.0029806	0	0
0	6.128E-05	0	1.867E-05	0	0.0018758	0	0	0.0001185	0.0005661	0.0004501	0.0005434	0.0119207
0.0001957	0.0003845	0.0004276	0.0001486	0.0003181	0.0001196	0.0009112	0.0002972	0.0001317	0.0231451	0.0002678	0.0005424	3.463E-05
0	0	0	0	0	0	0	0	0	0	1.44E-05	0	0
0	0	0	0	0	0	0	0	0	0	0	0	0
0	0	0	0	0	0	0	0	0	4.259E-05	0	3.989E-05	0.0055608
0.0003148	0.0005661	0.0002205	0.00026	0.000485	2.01E-04	0.2499789	0.0004657	7.18E-05	0.0002548	0.0001196	0.0003212	0
4.646E-05	0	3.617E-05	1.59E-05	0.0002887	1.497E-05	0	0	3.804E-05	0.013966	9.354E-06	0	0.0001559
7.105E-05	0.0001236	0	1.015E-05	0.0002513	4.491E-05	0	0.030385	0.0001157	3.137E-05	0.0015573	9.72E-05	7.51E-05
0.0001115	0.0011086	0.000755	3.968E-05	0.1588008	5.734E-05	0.0219194	0.0001844	6.445E-05	0.0001388	5.206E-05	0.0002481	0.0120719
0.0444705	0.0319753	0.0003521	0.0025783	0.0003507	0.0478029	0.0008879	0.006116	6.442E-05	0.0062002	0.0008917	0.0429697	0.0032281
0	1.65E-05	0.0001539	0	0.001842	5.171E-06	0	5.941E-05	5.6E-05	7.658E-05	7.35E-05	0.0002457	8.208E-05
4.701E-05	0.0001259	5.304E-05	0.0153723	0.0001939	0.0019227	0.0001874	5.142E-05	6.451E-05	0.001042	9.163E-05	0.0002698	0.0117833

Control117	Control114	Control113	Control112	Control111	Control110	Control109	Control108	Control107	Control106	Control105	Control104	Control103
0.0004746	0.0001841	0	0	0	0	8.591E-05	0	0	0.00018	0.0002045	0	0
0	0	0	0	0	0	0	0	0	0	0	0	0
0	0.0004053	0	0	0	0	0	0.0010247	0	0	0	0.0002371	0.0191332
0	0.0003043	0.0001953	0	0	0	0	0	0	0	0	0	0
0	0	0	0	0	0	0	0.0002106	0	0	0.0079957	0	0
0	0	0	0	0	0	0	0	0.00E+00	0	0	0	0
0.0029915	0	0	0	0	0	0	0	0.00E+00	0.0003214	0.0001273	0	0.00014
0	0	0	0	0	0	0	0.0001856	0	0	0	0	0.0065134
0.0001654	0	0.0001904	0.0009645	0.0002403	0.0020444	0.0002091	0.0128939	9.364E-05	0.0211808	0.0003652	0.0001842	0.0003777
0	0	0	0.000264	0	0	0	0	0	0	0	0	0
0.0001462	0	0.0004072	0.0022224	0	0.0015628	0.0001692	0.0140597	0.0001004	0.0202107	0.0001233	0	0
0.0146533	1.33E-04	0.0023379	0.0151404	0.0092154	0.0014098	0.0916754	0.0006314	0.0308442	0.0060447	0.009836	0.0005552	0.0045696
0	0	0	0.2360385	0	0	9.157E-05	0.0003824	0	0.0001266	0.000369	0.0001427	0.0002559
0	0	0	0	0.0002103	0	0	0	0	0	0	0	0
0	0	0	0	0	0	8.445E-05	0	0	0	0	0	0
0	0.0001038	0	0	0.0004871	0.0001459	0.0005266	0.0005538	7.911E-05	0.0002794	0.3663861	1.38E-04	0.0002818
0.0474349	0.0002425	0.0023242	0	0	8.572E-05	0.0719091	0.0001274	0	0.0007543	0	0.0002719	0.0079181
0	0.0080389	0.0002752	0	0.0003662	0.0108265	0.0001206	0.0426287	8.466E-05	9.101E-05	7.408E-05	0.0271244	0
0	0	0	0	0	0	0	0.0014238	0	0	0	0	0
0.0002678	0.0013816	0.0002178	0.0016975	0	0.0010496	0.0001482	0	0	0	0.00E+00	0.0093838	9.639E-05
0.000411	0.0006512	0.0014271	0	0	0	0	0	0	0	0	0	0
0	0.0001314	0.0002796	0.0001344	0	0	3.21E-05	0	0	0	0.002209	8.55E-05	0
0	0	0	0	0	0	0.00E+00	0	0.00E+00	0	0.00E+00	0	0
0	0	0	0	0	0	0	0	0	0	0	0	0
0.000108	0.027394	0.000337	6.289E-05	0.0001578	0.0062834	0.00E+00	0.009034	0.00E+00	0	0	0.0135172	0.000197
4.463E-05	0	0.001749	0.0102519	0.0077017	0.0080109	0	0.0185613	0.0001126	0.0449827	4.357E-05	0	0.0001158
0	0	0.0001424	0.0050741	0	0	0	0.0001822	0	0	0	0	0
0	0	0	0.0002296	0.0006606	0.0003606	0.0001573	0.0003577	0.0001476	0.0001759	0.0002629	0.0002863	0.0001632
0	4.395E-05	0	0.0001278	0.0005209	0.0002792	0.000337	9.767E-05	0.0004021	8.383E-05	0.000175	0.0002629	0.3265249
9.249E-05	0.0259102	8.933E-05	9.012E-05	5.06E-05	0.005039	1.976E-05	0.0083442	3.874E-05	4.348E-05	0	0.0242627	0.0001115
0	0	0	0	0	0	0.00E+00	0	0	4.281E-05	0	0	0
0	0	0	0	0	0.0079583	1.417E-05	3.88E-05	0.0001146	2.71E-05	5.912E-05	3.51E-05	0
0.0262631	0.0001854	0.0054386	7.683E-05	0.0001035	6.013E-05	0.0349067	0.0008551	0.0136897	2.895E-05	0.0150018	0.0001275	3.619E-05
2.315E-05	0	0	0	0	0	0	8.775E-05	0	0	0	0	0
0	0	0	0	0	3.452E-05	0	0.0002388	1.56E-05	0.0005811	0	0	0
0.0056598	1.35E-02	0.0393541	0.0001242	0.0001709	9.537E-05	0.0001144	0.0537817	0.0541254	6.453E-05	0.0003989	0.0001226	0.0033871
1.82E-01	0.0001816	0.0002843	0	3.342E-05	0	0	0	1.892E-05	0	0	2.134E-05	0
0	0	0	0	0	0	0.00E+00	0	0	0	0	2.102E-05	0
0	2.114E-05	0	0	0	0.00E+00	0	0	0	0	0	0	0
3.009E-05	6.457E-05	0	1.352E-05	2.096E-05	2.502E-05	0	3.955E-05	1.251E-05	0	0.0120817	2.163E-05	0
0.0001353	7.533E-05	0.1723932	2.885E-05	0.006478	5.93E-05	0.0056716	0.0002423	3.558E-05	0.0001821	0.0001273	0.0001407	8.783E-05
1.528E-05	0	1.871E-05	0	0	9.67E-06	0	0	0	0	1.434E-05	0	0
0.015616	0.0001624	9.904E-05	8.305E-05	0.0002402	0.0001243	8.828E-05	0.0001464	0.0328653	6.275E-05	3.80E-02	8.735E-05	5.95E-03
6.284E-05	1.08E-04	8.119E-05	0	0	0	0.00E+00	1.261E-05	0	0	2.706E-05	2.752E-05	0
0.0134222	1.03E-04	0.0024339	0.014233	0.0089153	0.0016356	0.0860926	0.0009644	0.0329235	0.0051487	0.009578	0.0003584	0.0046048
0.0063138	9.649E-05	8.395E-05	6.37E-05	7.735E-05	4.962E-05	3.895E-05	7.944E-05	1.628E-05	0.0002784	5.127E-05	7.658E-05	2.487E-05
8.039E-05	0.0235091	9.665E-05	3.648E-05	4.44E-05	0.0097791	2.44E-05	0.0071639	2.916E-05	3.897E-05	4.968E-05	0.0171015	0.000518

Control102	Control101	Control115	Control066	Control013	Control064	Control029	Control028	Control027	Control026	Control025	Control024	Control023
0	0.0003682	0.0362828	0.0006218	0.0003027	0.0264236	0.0032646	0	0	0.0020905	0.0007814	0.0001186	0
0	0	0	0	0	0	0	0	0	0	0	0	0
0	0	0	0.0001988	0.0002332	0.0002447	0	0	0	0	0	0.0001109	0
0	0	0.0002742	0	0.0002592	0	0	0	0	0	0	0	0
0	0.0002433	0.0158062	0	0.0097422	0	0	0	0	0	0.0001198	0.0001271	0
0	0	0	0	0.0002405	0	0	0	0	0	0	0	0
0	0	0.0811815	0.0003373	0.0003851	0.0498947	0.0053975	0	0.0027051	0.0044332	0.0006874	0	0.0002737
0	0	0	0	0	0	0	0	0	0	0	0	0
0	0	0.000181	0	0	0	0	0.064585	0.0025813	0	9.988E-05	0.0027935	0
0	0	0	0	0	0	0	0	0	0	0	0	0
0	0.0002409	0.0001548	0	0	0	0	0.0502743	0.0098989	6.022E-05	0.0002839	0.0018725	0.0005706
0.00043	0.0049425	0.0001388	0	0	0.0156657	0.0002613	0.0002586	0.0046676	0.0294507	0.0114417	0.0354846	0.0002694
0.0002208	0.0001562	0	0	0	0	0.0003259	0.000369	0.0092538	0.0001724	0.0001589	0.0001374	0
0	0	0	0	0	0	0	0.0001544	0	0	0	0	0
0	0	0	0	0.000834	0	0	0	0.0022644	0	0	0	0
0.0015625	0.0015279	0	0	0.000314	0	0.0004623	0.0005068	0.008579	0.0001088	0.0003659	0.0004401	0.0013796
0.0004213	0.0109599	0.0001567	0.0003306	0.0022018	0.0024932	0.0416598	0.0004139	0.0105558	0.0137568	0.000311	0.01565	0.0002572
0.0143824	0.0001841	0	0.0005419	0.0033951	0.0002688	0.0001058	0.0772396	0.0127463	9.736E-05	0.0001947	0.0001863	0.0001884
0	0	0	0	0.0001278	0	0	0	0	0	0	0	0
0.0002588	0	0.0001303	0	0.0020206	0	0.0002267	0	0.0046357	0.0163703	0	0	0.0006265
0	0	0.0005496	0	0	0	0	0	0	0	0	0	0
0.00E+00	0.000246	0.0063871	0.0244929	0.0102154	0.0001222	0	0	0.0013153	5.041E-05	0	0.0027192	0
0	0	0	0	0	0	0	0	0	0	0	0	0
0	0	0	0	8.296E-05	0	0	0	0	0	0	7.08E-05	0
0.0101649	0	0	0.0128764	0.0013599	0.000318	4.272E-05	0.0018856	0	0	3.56E-05	2.61E-05	0.000248
0.0006025	0.000254	6.588E-05	4.888E-05	6.482E-05	0	0.0001615	0.0306951	0.0045893	0.0001679	3.507E-05	0.0065201	0.0004048
7.566E-05	0	0	0	0.0005147	5.177E-05	0	0	0	0	2.987E-05	2.887E-05	0
0.0003	0.0005238	0.00E+00	4.3E-05	0.000341	4.202E-05	0.0330137	0.0004436	0.0261371	0.0002463	0.0002756	0.0002638	0.6587211
0.0001848	0.0008367	0	0	0.000617	0	0.0003142	0.0003891	0.0167603	0.0002067	0.0004053	0.0002051	0.0006487
0.0062074	0	0	0.0073126	0.0105949	0.0005431	0	0.0040081	0.003412	4.743E-05	6.562E-05	2.372E-05	0.0001376
0	0	0	0	0	0	0	0	0	0.00E+00	0	0	0
0	0.0100399	0	0.0037038	7.39E-05	0.0198495	0	6.959E-05	0.0021229	2.648E-05	5.235E-05	7.21E-05	0.0001854
8.128E-05	0.0001308	0.0001971	0.0008245	0.0033036	0.0002199	7.516E-05	0.0001776	0.0028727	6.848E-05	7.293E-05	6.291E-05	0.0001631
0	0	0	0	0.0004388	0.00E+00	0	0	0.0014095	0	0	0.00E+00	0
0	0	0	0.000243	0	0.0001277	1.986E-05	5.816E-05	0.0004066	0	0	0	3.499E-05
0.0009375	0.0024711	0.0001287	0.0005174	0	0	0.0002224	0.0002869	0.0084443	0.0166824	0.0001721	0.0001335	0.0003527
0	0	7.248E-05	0.0001566	0	0.0002118	0	0	0	0	0	1.087E-05	3.906E-05
0	2.258E-05	0	0	2.375E-05	0	1.83E-05	0.0006988	0	0	0	0	3.893E-05
0	0	0	0	2.488E-05	0	5.011E-05	0.0001674	0.0002983	0	0	0	3.273E-05
0	2.738E-05	0	0	1.961E-05	0	1.386E-05	0	0.0005835	0	2.299E-05	0	3.482E-05
0.2053414	0.0245079	9.488E-05	0	0	0	0.0001519	0.0001936	0.0076932	0.0001282	0.0001734	0.0001962	0.0002923
0	0	1.808E-05	4.147E-05	0.0001908	1.372E-05	0	0	0	0	0	0	0
4.645E-05	0.0177165	0	4.306E-05	0.0001366	9.351E-05	0.0001138	0.0004168	0.005284	0.000263	0.0001587	9.474E-05	0.0004097
2.064E-05	1.697E-05	5.848E-05	9.472E-05	0.0003172	0.0001195	5.779E-05	0	0.0003938	3.899E-06	2.133E-05	0.00E+00	0
0.0003894	0.0061529	4.974E-05	5.429E-05	0.0002278	1.87E-02	0.0004745	0.0006903	0.0235746	2.99E-02	0.0123187	0.0399671	0.000951
8.23E-05	0.0001062	0.0698774	0.0005319	0.0001654	0.0509656	0.0065471	9.462E-05	0.0030351	0.0040484	0.0014218	4.137E-05	0.0001433
0.0080723	7.805E-05	6.277E-05	0.0112968	0.0117623	0.0025421	6.391E-05	0.0041716	0.002306	3.041E-05	4.467E-05	4.331E-05	0.0001156

Control022	Control021	Control020	Control019	Control018	Control017	Control016	Control015	Control014	Control131	Control012	Control011	Control010
0.0927954	0	0	0	0	0	0	0	0	0	0.0101374	0.0006177	0.000225
0	0	0	0.0004524	0	0	0	0.0001237	0	0	0	0	0
0	0	0	0.0856329	0.0001071	0.0001376	9.559E-05	0.00E+00	0.0003327	0	0	0	0
0	0.000432	0	0	0.0001803	0.0001164	0	0.0002367	0.000308	0	0.0078207	0.000616	0
0	0.0001089	0.0003304	0.0001852	0	0.000374	0	0.0041213	0	0.0042048	0	0.0116994	0.0002251
0	0.0003459	0.0003657	0.0008434	0.0002372	0.0006292	0.0001153	4.25E-04	0.0010839	0	0	0	0
6.55E-02	0.00E+00	0.0004583	0.0002578	0	1.08E-04	0	0	0	0	0.0154954	0.0002196	0.0005983
0	0	0	0	0	0.0002454	0	0	0	0	0	0	0
0	0	0	0	0	0	0	0	0	0	0	0.0023971	0
0	0	0	0	0	0	0	0	0	0	0	0	0
0.0001635	0	0	0.0001233	0	0	0	0	0	0	0	0.0006452	0
0	6.532E-05	0	0	0.004077	0.0002803	0.0147866	0.0002994	0.0002177	0.039186	0.0010016	0	0.002689
0	0.0014489	0	0	0	8.888E-05	0	0	0	0	0	0	0
0	7.987E-05	0	0	0	0	0	0	0	0	0	0.0001997	0
0	2.90E-04	0.0006149	0.0005146	6.334E-05	0.5070676	0.0005463	0.0006044	0.0010187	0	0.0056214	0.0001821	0.0008656
0.0006131	0.0003016	0	0.0001656	0.0001953	0.0003659	0	0.0001904	0	0	0	0	0
0.0001469	0.0001323	0.0003992	0.0442755	0.0220055	0.0001788	7.837E-05	0.0051775	0.0001984	0.0344251	0.0008107	0	0
0.0003111	0	0	0.000544	0.0010583	0	0.0001333	0.0038861	0	0	0.0015155	0.0001503	0.0003916
0	0	0.0003853	0.0002637	0.0002393	0.0005314	0.0002596	0.00043	0.0011642	0.0006186	0	0	0
0.0003427	1.96E-04	0.0001196	0.0021795	0.0005194	0.0001856	7.50E-05	2.52E-03	0.0005284	0.0012959	0	0.0004141	0.0003606
0	0	0	0	0	0	0	0	0	0.0001016	0	0.0001108	0
0.0007104	2.75E-05	0	6.416E-05	0.0002185	1.18E-04	5.805E-05	0.0047327	0.0001299	0	0.0027177	0.0003834	9.777E-05
0	0	0	0	0	0	0	6.049E-05	0	0	0	0	0.0001023
0.00E+00	0	0	0	0	0	0	0	0	0	0	0	0
6.883E-05	7.357E-05	0.0003572	0.0002575	0.0341769	0.0002029	0.0003869	0.0305624	0.0003513	0	0.0020648	0.0001768	0.0266215
0.000306	0	6.907E-05	0	3.719E-05	0	0	0	8.182E-05	4.463E-05	0	0.0154713	0.0001987
0.0002031	0.00E+00	0	0	0	0	0	0	0	0.0001563	0	0	0
5.44E-04	0.0001622	6.352E-05	0.0003078	0.0001788	0.000172	0.1377577	0.0001524	0.0001534	0	0.000343	0	0.0001124
0.0004371	0.2864798	0.0008807	0.0003516	0.0004053	0.0014227	0.0005413	0.0005527	0.0010028	0	0.0031141	0.0005266	0.0541178
0.0001802	7.115E-05	8.854E-05	0.0015289	0.0053836	0.000147	0.0014325	0.0104787	0.0006285	9.17E-05	0.0041401	0.0001866	0.0027551
0	0.00E+00	0	0	0	0.00E+00	0.00E+00	0	0	0.0001308	0	0	0
0.0803624	3.387E-05	0.0025793	0.0001102	5.36E-05	2.032E-05	6.282E-05	0.0001681	0.0002039	0	0.0021488	0.0004921	0.0002057
0.0002895	0.0351305	0.0021083	0.0083224	0.0429496	0.0003223	2.00E-04	0.0002745	0.0005077	0.0227174	0.0002071	7.961E-05	0.0167204
0	0.000176	0.0003376	0.0004312	8.61E-05	3.20E-01	2.29E-04	0.0003316	0.0010024	0	0.0031925	0.0007133	0.0007618
2.695E-05	1.702E-05	0	0	0	1.371E-05	0	0	0.0001466	2.884E-05	0.0008444	3.073E-05	0
0.0002009	0.0003259	1.867E-05	0.0042597	2.76E-05	5.925E-05	0.0015556	0.0001258	0.0004273	0	0.0036006	0.0004972	0.0005463
0	2.295E-05	0	0	1.49E-05	0	0	4.792E-05	0	0.0002138	0.0019513	0.0003294	0.0003584
0	0	0	9.071E-05	0.00E+00	0	0	2.959E-05	0	0	0	0.000111	0
0	4.295E-05	1.739E-05	0	0	3.41E-05	0	0	0	0	0	0.0001878	0
0	0	0	1.487E-05	0	0	3.009E-05	1.318E-05	8.823E-05	0	0	0	0
0.0001894	0	0	0	0	0	0	1.987E-05	0	0.0002228	0	6.924E-05	0.0003757
0	0.0084188	0.0006604	0.0155911	8.69E-03	2.80E-04	0.0001665	3.30E-02	0.0003386	3.274E-05	0.0001138	0.0002208	3.648E-05
0.0001378	0.0034185	0.0008551	0.0005638	0.0078348	2.12E-04	9.381E-05	0.0022248	0.0002476	1.722E-05	0.0003371	0.0001424	6.182E-05
0	6.88E-05	9.587E-05	0.0367567	0.0001238	0.0002163	0.0001243	0.0001706	0.0005383	8.761E-05	0.0004103	6.789E-05	6.055E-05
0.000564	9.81E-05	0.000261	0.0001865	4.65E-03	4.65E-04	0.018029	0.0002078	0.0004081	0.0394391	0.0009199	0.0001153	0.0027837
0.0708634	8.593E-05	0.0005525	8.351E-05	7.592E-05	0.0003008	0.0001129	0.0001605	0.0001607	1.21E-05	0.0310179	0.0007051	0.0005459
6.538E-05	9.986E-05	0.000194	0.0012158	0.0137342	3.64E-04	0.0058059	0.0135762	0.0003473	3.659E-05	0.0157059	0.0002563	0.0058259

Control009	Control008	Control007	Control006	Control005	Control004	Control003	Control002	Control001	Control030	Control031	Control032	Control033
0.0016691	0.0006873	6.34E-04	0.0009286	0.0005359	0.0002127	5.56E-04	0.0005114	0.0015137	0.0003396	0	0.0015587	0
0	0.0016626	0	0	0	0	0	0	0	0	0	0	0
0	0	0	0	0	0	0	0	0.0005582	0.0003288	0.0003709	0	0
0.0002892	0	0.0003306	0.0002705	0.0001653	0	0	0	0	0	0	0	0
0	0	0	0.0078177	0	0.000374	0.0002614	0	0.0002142	0	0	0.0002941	0
0	0	0	0	0	0	0.00E+00	0	0	0	0	0	0
0.0012889	0.0003087	0.0002323	0.0010375	0	0.0001209	0.0014289	0.0008624	0.0002355	0.0003437	0.0003087	0.0012093	0.000261
0	0	0	0	0	0	0	0	0	0.0002769	0	0	0
0	0	0	0	0	0.00E+00	0	0	0	0.0006149	0.0003496	0	0.1016312
0	0	0	0	0	0	0	0	0	0	0	0	0
0	0	0.00E+00	0	0	0	0	0	0	0.000281	0.000952	0	0.0723517
0	0.0001334	0.0001198	0	0.0052201	0	0.0030918	0	0	0.0005089	0.0011023	0.0127291	0
0	0	0.00E+00	0	0	0	0.0001077	0.0001454	0	0.0004875	0	0	0
0	0	0	0	0	0	0	0	0	0	0	0	0
0.4597875	0.0002454	0.0010055	0.0008867	0.0004698	0.0001504	0.0002666	0.0060172	0.0011322	0	0	0	0
0	0	0	0	0	0	0	0	0	0.001963	0	0	0
0	9.552E-05	0	0.0157014	0.0010237	0.00E+00	0.0017683	0	0.0001812	0.0002278	0.0089394	0.0103648	0.0001151
0	0	0.0050037	0.0003535	7.831E-05	0	0.0014414	0.0045719	0.0003365	0	0.0003662	0.000999	0.0051878
0	0	0	0	0	0	0	0	0	0	0	0	0
0.0002606	6.962E-05	0.0008354	0.0002785	0.0002499	0.0017172	0.0005623	0.0011478	0.0024205	0	0.0364962	0.0522524	0.0002588
0	0	0	0	0.021022	0.0002217	5.234E-05	9.391E-05	0.0002386	0	0.0004218	0.0017997	0.0010392
0.0006508	5.958E-05	0.0007577	8.708E-05	0.0495158	0.0003697	1.10E-04	2.02E-04	0.0964911	0.0399222	0.0632525	0.0001207	0.0002429
0	0	0	0	0	0	0	0.0001973	0	0	0	0	0
0	0	0	0	0	7.076E-05	0	0	0	0	6.1E-05	0	0.0006954
0.0006123	4.865E-05	0.0021182	0.0006337	8.781E-05	0.0002765	0.0049555	0.0035398	8.425E-05	0.0001175	0.0001887	0.0001922	0.000483
0.0001296	0	0.0001275	7.969E-05	3.507E-05	9.245E-05	0	0	6.482E-05	0.0006747	0.0021709	9.67E-05	0.0266317
0	0	0	0	0	0	0	0	0.0002877	0	0.0014096	0	0
0	7.915E-05	9.48E-05	0	0.000127	0.0001016	0.00E+00	0.000342	0	0.0011384	0	0	0
5.55E-04	0.0002181	0.2197105	0.0170883	0.0002303	0.000127	0.0003223	0.00042	0.0003695	0.0008115	0.016219	0	3.093E-05
0.0020135	3.162E-05	0.0081964	0.0039409	0.0002917	9.091E-05	0.0235757	0.0183012	0.0003644	0.0001463	0.0001289	0.0001336	0.0002237
0	0	0	0	0	0	0.00E+00	0	0	0	0	0	0
0.0004681	0.0002346	0.0006639	0.0005099	0.0129093	4.96E-04	0.0061002	0.0009848	0.0004748	0.0003849	0.000303	0	0
0.000157	4.788E-05	9.019E-05	4.12E-05	7.516E-05	0.0001347	0.0031466	0.0005467	0.0001164	0.0003023	0.0004276	0.000407	0.0029918
0.3010677	0.0004172	0.0013928	0.0009594	0.0009621	0.0007984	0.0005814	0.0014444	0.0007117	5.761E-05	0	0	0
0	3.972E-05	0	0	1.844E-05	7.943E-05	0	7.092E-05	0	0.0002132	0.0001603	7.518E-05	0
0.0004046	0.0002605	0.0052118	0.0013575	0.0007305	0.3429919	0.0002431	0.0240999	0.0004083	0.0006153	0.0004338	0.0004915	0.0002804
0.0005734	0.0001462	0.0004103	0.0004284	0.0003169	0.0002565	0.000209	0.0001864	0.0007457	0	0.0002227	0.0004325	0.0002432
0.0002254	0	0	0	7.202E-05	1.62E-04	0	0	0	0	0	0	0
0	0	4.47E-05	0	0	5.42E-05	0	0.000134	2.352E-05	0	0	4.909E-05	0
0.00E+00	0	0.00E+00	0	0	0.00E+00	1.217E-05	0.00E+00	0	3.313E-05	3.887E-05	0.0238111	0
0	1.218E-05	0	0.0185972	9.456E-05	3.205E-05	2.596E-05	2.757E-05	0	0.0004276	0.000292	0.0018165	0.0001628
8.294E-05	0.0003754	4.77E-05	0.0049279	7.608E-05	1.902E-05	1.434E-05	1.871E-05	0	0	1.621E-05	0.011524	2.837E-05
0.0002688	4.122E-05	0.000116	0.0017683	0.0091337	6.92E-05	7.751E-05	0.002807	0.0001978	0.0003156	0.0031106	0.0293786	0.0001753
4.885E-05	7.339E-05	8.05E-05	0.0001491	7.614E-05	9.88E-05	3.922E-05	4.587E-05	0.0001085	0	0.0001303	8.83E-05	0.0001112
0.0001901	0.0001221	0.0001995	0.00015	0.005099	0.0001688	0.0028243	2.56E-04	0.0002213	0.0010323	0.001173	0.0124023	6.195E-05
7.64E-04	0.0002693	0.0008042	0.0008551	0.0006601	0.0006104	0.0009934	0.0005692	0.0005572	0.0001603	0.0009196	0.002254	0.0001129
0.0021812	0.0001369	0.0148999	0.0067247	0.0002197	0.0002326	0.0244992	0.0224049	0.0002641	0.0001179	0.0002475	0.0004099	0.0002719

Control063	Control062	Control061	Control060	Control059	Control058	Control057	Control056	Control055	Control054	Control053	Control052	Control051
0.0370028	0.0005564	0.0015055	0	0.0062428	0.0001595	0	0	0	0.0008059	9.409E-05	0	0
0	0	0	0	0	0	0	0	0	0	0	0	0
0.0004894	0.0005047	0.0001491	0	0.0723689	8.794E-05	0.0004282	0.0007265	0.0005009	0.0003671	0.000975	0.0020227	0.0004894
0	0	0	0	0	0	0	0	0	0	0	0	0
0	0	0	0	0	0	0	0	0	0	0	0	0
0	0.0002636	0	0	0.0255553	0.00E+00	0	0	0	0	0	0	0
0.0067691	0.0022118	0.0046464	0	0.0118324	0.0008847	0.0003151	0.0008052	0	0.0012857	0	0	0
0	0	0	0	0	0	0.0001982	0	0.0413554	0.000107	0	0	0
0.0002403	0.0003683	0	0	0	0	0	0.0003933	0.0004869	0	0.0006648	0	0
0	0	0	0	0	0	0	0	0	0	0	0	0
0	0.0002437	0	0	0	0.000109	0	0.0002466	0	8.889E-05	0.0002351	0.000324	0.0001319
0.0033286	0.0004219	0.0009526	0.0002096	0.0017799	0.0041178	0	0.0068286	0.0001633	0.0018779	0.0026509	0	6.26E-05
0.0182328	0	0.0001293	0.0004228	0	0	0	0	0	0	0	0	0
0	0	0	0	0	0	0.0001651	0	0	0	0	0	0
0	0	0	0	0	0	0	0	0	0	0.0001821	0	0
0	0	0	0	0	0	0	0	0	0	0	0	0
0.0044966	0.0014425	0	0	0.0012099	0	0	0.032669	0.0002033	0	0.0040974	0.0002817	0
0	0.0003069	0	0	0	0	0	0	0.0004932	0	0	0	0
0	0	0	0.0001764	0	0	0	0	0	0	0	0	0.00E+00
0.0001285	0	0.000141	0.0001464	0.000548	0	0.0001017	0	0.0025186	0	0.000116	0.0002178	7.319E-05
0	0	0	0.000137	0	0	0	0	0	0	0	0	0
7.638E-05	0.0164818	0.0007715	0.0003758	0.0003086	0.001572	0	0.0009319	0	5.805E-05	0.001381	0	0.0003269
0	0	0	0.0001195	0	0	0	0	0	0	0	0	0
0	8.052E-05	0	0.0001074	0	0	0	0	0	0	0	0	0
7.951E-05	0	0.0024493	0	0.0001151	0.00E+00	0.0001365	0	0	3.56E-05	3.441E-05	0	0
0.0043035	0.0025035	5.207E-05	0.0002518	0.000119	0.0001307	5.632E-05	0.0033631	0.0004197	0.0005122	0.0012634	0.0002295	0.0001084
0	0	0.00E+00	0	0.0005714	0	0	0	0	0	0	0.0001344	3.584E-05
0.0073994	0	0	0.0001603	0	0.0112954	0	3.518E-05	0	0	3.713E-05	0	3.029E-05
0.0066099	0	3.26E-05	0	0	0	0.0001009	0	0	0	6.186E-05	0.0002002	0
0.0004735	0.0003225	0.0128045	0.0002071	0	2.293E-05	8.696E-05	0.0001051	0.000181	2.846E-05	7.668E-05	0.0002886	7.352E-05
0	0	0	0	0	0	0	0	0	0	0	0	8.329E-05
0.0224238	0.0154134	0.0041183	0.0020521	0.015943	0.0272252	0.0008598	0.0003818	0.0018088	0.0005469	0.0006614	0.0011301	0.0006331
0.0002166	0.0026433	0.0418818	0.0006124	0.000275	1.726E-05	2.561E-05	7.404E-05	0.0018834	2.839E-05	0.0046987	7.905E-05	0.0001013
0	0	0.00E+00	0	4.253E-05	0.00E+00	0	0	9.045E-05	0	8.668E-05	0	0
0.0003059	0.0133796	1.42E-04	0.0004719	0.0002236	0.0016203	0.0003106	0.0178439	9.314E-05	0.0001423	0.0056955	0.0002449	6.667E-05
5.357E-05	0.0001907	0.00E+00	0.0029646	0.0002658	8.441E-05	7.711E-05	0.0190809	8.157E-05	0.0004858	1.664E-05	0.0005183	0.0013644
0.0004578	0.0003294	0.0001397	0.0004929	0.0003048	0.0013203	0.0016352	0.0009877	0.0026	0.0022215	0.0019585	0.0042915	0.2601073
9.46E-05	0	0	0	0	0	4.283E-05	1.752E-05	0.0002772	0	1.246E-05	0	8.915E-05
0	0	0	9.613E-05	0	0	0.0001316	0	0	1.193E-05	5.182E-05	0	1.977E-05
0	0	0	0	0	1.014E-05	0	1.386E-05	2.4E-05	0	1.454E-05	0	7.809E-05
0.0001718	0	0	0	0	0	1.635E-05	0	2.564E-05	0	0	0	3.975E-05
2.12E-05	0.0008259	3.336E-05	0	3.149E-05	3.461E-05	7.577E-05	0.0122389	0.0076593	4.989E-06	5.02E-05	0.0001528	2.151E-05
7.751E-05	0.0007687	0.0005223	8.12E-05	0.0082362	0.0619867	0.0004275	0.0004595	0.0258736	2.861E-05	0.004675	0.0006687	0.0007407
0.0003387	0.0003649	0.0001186	0.00025	0.0014946	0.0001271	0.0001257	7.912E-05	0.0001947	0.0004541	0.0002814	0.0813881	0.0001018
0.0047252	0.0005618	6.41E-04	0.000154	0.0013452	0.0037969	4.052E-05	0.0072736	0.0006814	0.0032416	0.002873	9.507E-05	1.96E-04
0.0086764	0.0026002	0.0033076	0.0004393	0.007318	0.0012859	0.0002624	0.0006895	0.0001313	0.001366	0.0001596	0.0002189	0.0001065
0.0001696	0.0003286	0.0115821	0.0001294	0.0001726	9.854E-05	0.0001643	7.783E-05	0.0003232	7.696E-05	0.000198	0.00038	0.000103

Control050	Control065	Control049	Control047	Control046	Control045	Control044	Control043	Control042	Control041	Control040	Control039	Control038
0	1.62E-02	0.000225	0	0	0.0013991	0.0005155	0	0	0	0	0.0009041	0.0005523
0	0	0	0	0	0	0	0	0	0	0	0	0.0003132
0.0003632	0.0001759	0.000738	0	0	0	0	0	0	0	0	0	0
0	0	0	0	0	0	0	0	0	0	0	0.0001127	0.0507597
0	0	0	0.0007117	0.0001816	0.0008533	0.0140995	0.000374	0.8399014	0.0006972	0	0.000719	0
0	0.0001351	0	0	0	0	0	0	0	0	0	0.00E+00	0
0	0.0297369	0.0002673	0	0	0.0018045	0.000366	0.00014	0.0016294	0.00E+00	0.0002037	0.0018363	0.0014639
0.000107	0	0.0001919	0	0	0	0	0.0001699	0	0	0	0	0
0	0	0.0580803	0.0019976	0	0.0009426	0.01771	0.0001529	0	0.0044291	0.0002154	1.94E-04	0.0001092
0	0	0	0	0	0	0	0	0	0	0	0	0
9.18E-05	0.0001405	0.050908	0.0010209	0	0.0001233	0.0125256	0	0	0.0016058	0	0	0.0001147
0.00E+00	0.0088943	0	0.0047819	0.0101598	0.0059685	0.0026209	0.000264	0.0005661	0.0028441	0.0001687	0.0018752	0.0033013
0	0	0	0.0004174	0	0.0824168	0.000571	0.0002801	0.0038513	0	0	0	0
0	0	0	0	0	0	0	0	0	0	0	0	9.584E-05
0	0.000132	0	0	0.0001452	0.0001161	0	0	0	0	0	0	0
0.00E+00	0	0	0.1818505	0.0004203	0.0004302	0.0003807	0.0003659	0	0	0	0	0
8.082E-05	0.0021014	0	0.0058314	0.0258017	0.0021601	0.0001445	0.0003551	0.0005021	0.0055448	0.0002523	0.0006956	0.0002008
0.0041528	0	0.0001545	0	0	0.0081532	0.0009504	0	0.0002307	0	0.0002984	0.0080283	0.0002921
0	0	0.0003225	0.0007038	0.0002434	0.0009836	0.0011013	0.0014197	0.0006389	0.5638509	0	0	0
6.248E-05	0	0.000614	0	0.0002285	0	0.0003856	0.0001107	0	0	0	0.0005444	0.0028507
0	0	0	9.237E-05	0.0002556	0.0006974	0.0007297	0.0007667	0.0002879	0.2372348	0.0014918	0.0005681	0.0007975
4.888E-05	0.0035564	0.0001024	0.000249	7.027E-05	0.0028155	0	0	0.0011335	0	0.000967	0.000194	0.0435106
0	0	0.0001916	0	0	0	8.21E-05	0	0	0	0	0	0
0	0.00E+00	0.0001854	0	0	0	0	0	0	7.32E-05	0	3.172E-05	0
0.0072256	5.577E-05	0.0001626	0	6.764E-05	0.0083719	7.12E-05	0	0.0001092	0.0001293	7.357E-05	0.0184632	0.0002967
0.0001934	0	0.0290544	0.0354757	0.0001211	0.0075848	0.0220392	0.0002688	0.0001923	0.0115206	0.0004909	5.313E-05	8.607E-05
0	0.00E+00	0	0.000896	0.000226	0.0001901	0.0904878	0.00045	0.0069954	0.038346	0	0	0
0.00E+00	4.984E-05	0.0003342	0	0	0	0.0023775	5.472E-05	0	4.886E-05	0	0	0
0	8.383E-05	0	0	0	0	0	0	0	0	0	0	0
0.0240074	0.0001818	0.0001075	7.668E-05	0	0.007058	9.17E-05	0.0001581	0	9.17E-05	0.0003138	0.0199598	0.0002103
0.00E+00	0	0	0.000481	0.0005316	0.0004297	0.0003814	0.0003837	0.0006935	0.0003176	0	0	0
0.0003627	0.0172918	0.0002993	0	0	0	0	0	0	0.0057381	7.76E-05	0	0
0.0003552	7.19E-03	0.0010188	0.0003006	0.0959857	0.0004548	0.0073893	0.0006018	0.0047009	0.0003953	0.0007042	0.0036626	0.007666
0	0.00E+00	0	0	3.015E-05	4.253E-05	0	0	0	0	0	0	2.046E-05
0.0001087	0.0002506	0.0013816	0	0	0	0	0	0	2.459E-05	0.001618	0.001017	0
9.943E-05	0	7.589E-05	2.354E-05	0	4.951E-05	2.435E-05	0.001166	0.0003263	2.841E-05	0.2298352	0.0072142	0.000321
0.0012853	0.000486	0.0015446	0.0003036	0.0003886	0.0005029	0.0004172	0.0003805	0.0008657	0.0001897	0.0003306	0.0002255	0.0002311
1.168E-05	0.00E+00	2.102E-05	0	1.947E-05	0	0	0	0	0	0	0	8.215E-05
2.25E-05	4.977E-05	0.0001053	0	0	1.466E-05	0	0.00E+00	0	5.318E-05	0	0	0
0	0	2.231E-05	0	0	0	0	0	0	0	2.569E-05	5.07E-06	0
0	0	0	0.0004734	0.0003981	0.0005068	0.0003327	0.2721921	0.0004686	0.0003648	0.0001648	5.449E-05	0.0002148
1.403E-05	1.372E-05	3.929E-05	3.399E-05	0.0163375	2.37E-05	0.0023413	3.274E-05	0.0036019	0	1.93E-05	1.029E-05	0.0014614
0.0001227	3.045E-05	0.0001489	9.597E-05	0.0001744	0.0063694	0.0009692	8.54E-04	0.0014663	1.753E-05	0.0003836	2.584E-05	0.0115492
0.0016554	0.0536776	0.0001592	0.0001901	0.0001108	0.0001628	0.0001064	0.0002291	0.0001816	7.431E-05	8.669E-05	4.426E-05	0.0143649
9.91E-05	0.0083132	5.87E-05	0.0052984	0.0136843	0.0061767	0.0028415	0.0005042	0.0003555	0.0027465	0.0001877	2.11E-03	0.0034251
7.23E-05	0.028757	0.0002499	0	7.152E-06	0.0023487	0.0003613	4.852E-05	0.0008122	7.152E-06	0.0001829	0.0015941	0.0015861
0.0255989	0.0003346	0.0001657	6.062E-05	5.621E-05	0.0091449	0.000179	0.0002273	9.42E-05	7.19E-05	0.0002887	0.0242336	0.0001589

Control037	Control036	Control035	Control034	Control048	Control132	COVID-19-052	COVID-19-051	COVID-19-049	COVID-19-048	COVID-19-047	COVID-19-046	COVID-19-038
0	0.0015218	0	0	0.0008059	0	0	0	0	0	0	0	0.00E+00
0	0.0001431	0	0	0	0	0	0	0	0	0	0	0.0001933
0	0.0001032	0	0	0.001128	0	0	0	0	0	0	0	0.00E+00
0.0004245	0.0002216	0	0.0001127	0	0	0.0003418	0	0	0	0	0	0
0	0	0	0	0.0002869	0.0016957	0	0	0	0	0	0	0
0	0	0	0	0	0	0	0	0	0	0	0	0
0.0004201	0.0047705	0.0001368	0	0.0022564	0	0	0	0	0	0	0	0
0	0	0	0	0	0	0	0	0	0	0	0	0
0.0001373	0.0002872	0.000284	9.988E-05	0	0	0	0.0567163	0	0	0	0	0
0	0	0	0	0	0	0	0	0	0	0	0	0
0	0.0001807	0.0002782	0.0003154	0.0002495	0	0.0002753	0.0576153	0	0	0	0	1.41E-04
0.0014207	0.000166	0.0006559	0.0017473	0	0.1212649	0	0	0	0	0	0	0
0	0	0	0	0.0002343	0	0	0	0	0.0035954	0.000307	0.0015944	0.0063371
0	0	0	0	0	0	0.0002556	0.0004925	0.0003594	0	0.0001517	0.0725621	0.0002822
0	0	0	0	0	0	0	0	0	0	0	0	0.0001188
0	0	0.0004895	0.00E+00	0	0	0	0	0	0	0	0	0
0	0.0087753	0.0008792	0.0022581	0.0017536	0	0	0	0	0.0002253	0	0	0.00E+00
0	0.0002159	0.0277404	7.831E-05	0	0	0.0155529	0	0	0.0505766	0	0.0020743	0.0137622
0	0	0	0	0	0.0035756	0	0	0	0	0	0	0
0.0001178	0.0001017	0.0089179	0.000116	0.0001571	0	0.0469081	0.0006515	0	0.0039681	0	8.033E-05	0.0016351
0.000505	0.0015518	0.0013578	0.0010038	0	0.0006851	0	0	0	0	0	0	0
0.0002093	0.0002612	0.0001589	0.0217309	0	0	0.0003162	0.0155133	0.0001772	0.000139	9.471E-05	0	0
0	4.033E-05	0	0.00E+00	0.0001685	0	0	0	0	0.0001224	0	0.0001772	7.201E-05
0	0	0	0	0	0	0	6.1E-05	0	0.0003026	0.0015177	0.0002147	0.0001305
0	7.832E-05	0.0143111	0.0001305	0	0.0005387	0.0002539	0	0.0002563	0.0568112	0	0.0109078	0.0549434
4.782E-05	7.544E-05	0.0007661	0.0001711	8.182E-05	0	0.0004654	0.0299693	0.0001094	0.000102	6.269E-05	0	0
0	0	0	0	0.0007377	0.000453	0	0	0.000228	0	0	0	0
0	0	0	0.00E+00	0	0	0	0	0	0	0	0	0
0	4.884E-05	3.825E-05	0	0	0	0	0	0	0	0	3.99E-05	0
6.562E-05	0.0001731	0.016543	0.0001099	0.0003605	0.0007352	0.0007344	4.348E-05	0	0.0298954	0	0.0089166	0.0307262
0	0	0	0	0	0.0021218	0	4.515E-05	0	0	0	0	0
0.0026895	0	2.895E-05	2.648E-05	0.0004317	0	0	0	0	0.0001238	0	2.587E-05	8.992E-05
0.0106266	0.0007137	0.0003997	0.14945	4.732E-05	0.0017787	0.0071711	0.0002138	0	0.0002383	0	0.000118	0.0007349
0	6.353E-05	0	0	0	0	0	0	0	0	0	0	2.746E-05
4.019E-05	1.182E-05	0	2.79E-05	0.0001253	0	0	0	0	0.0002076	8.227E-05	0.0002014	9.74E-05
0.0001875	0.0002955	2.93E-03	0.0102032	3.774E-05	0.0001952	0.0013328	5.316E-05	5.235E-05	0.0008161	2.354E-05	6.33E-05	0.0001424
0.0002223	0.0230775	0.0004792	0.0003411	0.0025569	0.0018252	0.0049032	0.0003845	0	0	0	0	1.69E-05
0	0	0	1.402E-05	0	0	0.0016394	0.0012264	0.0012353	0.000577	0.0004816	0.2006281	6.09E-04
0	0	0	0	0	0	0.0007857	0.000513	0.0026058	8.897E-05	0.0001036	0.035359	9.988E-05
0	8.113E-06	0	0	0	0	0.0153495	5.747E-05	0	0	0	0	0
5.641E-05	0.0001308	0.0002228	8.783E-05	0	0.0020255	0	0	0	0	0	1.763E-05	0
0.0004169	4.053E-05	0.0014205	2.34E-05	2.9E-05	0	0.0018377	0.0001812	0.0001172	5.488E-05	0.0011839	2.931E-05	3.96E-05
3.383E-05	0.0001172	1.78E-03	5.167E-05	0.0003749	0	0.0012743	5.26E-05	3.906E-05	0.0001578	3.599E-05	4.829E-05	0.0001276
0.0001479	8.83E-05	0.0001456	0.0567212	0.0001784	0.000722	0	1.17E-05	0	1.812E-05	0.0158223	9.082E-05	8.119E-05
0.0007482	0.0001505	0.0010809	0.0021222	6.883E-05	0.1056594	0	0	0	0	0	0.00E+00	0
0.0001569	0.0034513	9.418E-05	0.0001074	0.0021443	0.0002048	0	0	2.652E-05	0	0	0	0
8.869E-05	0.0001801	2.07E-02	1.48E-04	0.0002366	0.0003603	0.0010214	5.406E-05	7.194E-05	0.0196441	1.958E-05	7.04E-03	0.0222697

COVID-19-044	COVID-19-043	COVID-19-042	COVID-19-041	COVID-19-040	COVID-19-039	COVID-19-053	COVID-19-045	COVID-19-054	COVID-19-063	COVID-19-056	COVID-19-037	COVID-19-071
0	0	0	0	0	0	0	0	0	0	0	0	0.0005727
0	0	0	0	0	0	0	0	0	0	0	0	0
0	0	0	0	0	0	0	0	0	0	0	0.0008832	0
0	0	0	0	0	0	0	0	0	0.0312229	0	0	0.000169
0	0	0	0	0	0	0	0	0	0	0	0	0
0	0	0	0	0	0	0	0	0	0	0	0	0
0	0	0	0	0	0	0	0	0	0	0	0	0.0008593
0	0	0.0001731	0	0	0	0	0	0	0.0098708	0	0	0
0	0	0	0	0	0	0	0	0	0	0	0.0070884	0
0	0	0.000387	0	0	0	0	0	0	0	0	0	0
0	0.0001606	0	0	0	0	0	0	0	0	0	0.0057982	0
0.0002504	0	0	0	0	0	0	0	0	0	0	0	0
0.0049716	0.1898397	0.0004013	0	0.0006544	0.000237	0	0.000843	0.0001347	0	0	0	0
0	0.0002556	0	0.0005617	0.0577068	0	0	0.000796	0.0005591	0	0.0001864	0	0.0004366
0	0	0	0	0	0	0	0	0	0	0	0	0
0	0	0	0	0	0	0	0	0	0	0	0.0009741	0
0	0.0008621	0	0	0	0	0.0010458	0	0	0	0.0044991	0	0.0007078
0.0087543	0.00029	0.0004276	0.0004805	0.0005143	0.0304539	0.0143083	0.0013568	0	0.0001312	0.0006181	0.0002688	4.233E-05
0	0	0	0	0	0	0	0	0	0	0	0	0.00E+00
0.0001678	6.962E-05	0.0001214	0	0	0.002549	0.0110867	0	0.0005855	0.0009032	0	0.0098354	0
0	0	0	0	0	0	0	0	0	0	0	0	0
0	0.0010189	0	0.0003468	0.0002032	0	0.0007104	0	7.027E-05	0.0002475	0.0004064	0.0155607	0
0.0001239	0.0002895	9.074E-05	0.0003428	0.0003586	0.0001484	0	0	0	0	0	0.0011551	0.00E+00
0.0004465	0.0030123	0.0002367	0.0002733	0.0001757	0.0001037	0.0001366	0.00158	0	0	0.0096774	0.0008199	0
0.0545696	0.0089913	0.0006076	0.001551	0.0012341	0.0398919	0.0104592	0.0015082	0	0.0001258	0.000337	0	0
0	0	0	0	0	7.119E-05	0	0.000357	0	0	0.000237	0.0191691	0
0	0	0	0.00022	0	0	0	0	0	0	0	0.0006152	0
0	0	0	0	0	0	0.0002179	0	0	0	0	0	0
0	4.07E-05	0	0	0	0	0	0	3.337E-05	0	0	0	0
0.0341991	0.0042966	0.000336	0.0012151	0.000589	0.035332	0.0116052	0.0012641	4.111E-05	0.0010087	0.0006475	0	0.0001502
0	0	0	0	0	0	0	0	0	0	0	0	0
6.59E-05	0.0175936	0	0	7.575E-05	5.112E-05	0	0	0	0.0003135	0	8.068E-05	1.04E-02
3.619E-05	0.0109746	3.674E-05	0	0.0001676	0.0003235	0	0.0001754	5.4E-05	0.0004053	0	0.0018171	0
0	0.0001384	0	0	0	0	0	0	5.491E-05	0	0	0	0
0.000339	0.0001721	3.262E-05	0	0.0003083	0.000243	0	0.0010511	0	0.0002317	0.0002766	0.0007423	0.1563343
3.531E-05	0.0031294	3.044E-05	0	0	9.213E-05	0	0.0007849	0.0003616	2.273E-05	0.0006023	0	0
0	0.0022154	9.664E-05	0	0.0002307	0	0.0321655	0	0.0002287	0	0.0070248	0.0008238	0.0015426
0.0006716	0.0009017	0.0006221	0.0016865	0.2194381	0.0005116	0.0018298	0.0030982	0.0017862	5.412E-05	0.0008172	0	2.725E-05
7.397E-05	9.034E-05	6.75E-05	0.0006098	0.0042246	3.17E-05	0.0013124	0.0006661	0.000932	1.943E-05	0.0005072	0	1.02E-04
0	0.0003786	0	0	0	2.873E-05	3.786E-05	0	0.0001281	0	0.001748	8.045E-05	9.296E-05
0	0	0	0	0.0002907	0	0	0	0	0.0114079	0	0	1.41E-05
3.149E-05	4.334E-05	0.0004321	7.14E-05	7.545E-05	7.483E-05	0.0082517	9.26E-05	0	0.0031139	0.0067828	0.005926	0.0758411
0.000128	0.012308	5.014E-05	0.0511709	0.0001172	0.0002119	0.0058435	0.0003959	1.63E-05	5.137E-05	0	0.011838	7.69E-06
0.0001383	3.578E-05	4.885E-05	0.0003536	9.311E-05	0.0001887	2.454E-05	0.0823364	0	0.0857807	2.936E-05	3.325E-05	0
0	0	0	0	1.961E-05	0	1.429E-05	0	0	0	0	0	0
0	5.391E-06	0	2.553E-05	1.782E-05	0	0	0	0	0	0	5.523E-05	0.0015062
0.0209085	0.0035554	0.0001924	0.0014102	0.0004802	0.0181279	0.010655	0.0008304	7.975E-05	0.0013558	0.0012871	0.0002097	0.0001739

COVID-19-070	COVID-19-069	COVID-19-068	COVID-19-067	COVID-19-066	COVID-19-065	COVID-19-064	COVID-19-062	COVID-19-061	COVID-19-060	COVID-19-059	COVID-19-058	COVID-19-057
0	0.0032237	0	0	0.0001432	0	0.0005155	0	0	0	0	0	0
0	0	0	0	0	0	0	0	0	0	0	0	0
0	0	0	0	0	0	0	0	0	0	0	0	0
0.0004057	0.0001841	0.0001653	0	0.0001052	0	0	0	0	0	0	0	0
0	0	0	0	0	0	0	0	0	0	0	0	0
0	0	0	0	0	0	0.00E+00	0	0	0	0	0	0
0.00E+00	0.0029597	0	0	0.0002641	0	0.0004074	0	0	0	0	0	0
0	0	0	0	0	0	0	0	0	0	0	0	0
0	0	0	0	0	0	0	0.0008303	0	0	0	0	0.0002372
0	0	0	0	0	0	0	0	0	0	0	0	0
0	0	0	0	0	0	0.0001176	0.0005276	0	0	0	0	0
0	0	0	0	0	0	0	0	0	0	0	0	0
0	0	0	0	5.386E-05	0	0	0	0	0.00129	0.0028494	0.0006706	0.0036143
0.0002875	0	5.75E-04	0.0066849	0	0	0.0002822	0	0	0	0.0023268	0.0333419	0.0001917
0	0	0	0	0	0	0	0	0	0	0	0	0.00E+00
0	0	0.00E+00	0	0	0	0	0	0	0	0	0	0
0.0649168	0.013867	0	0	0.0001567	0	0	0	0.0055767	0	0	0	0
0	9.736E-05	0.0001079	0	0.0001058	0.0001736	0.0153942	0.0006646	0.0017547	0.0182706	0	0	0.0006117
0	0	0	0	0	0	0	0	0	0	0	0	0.00E+00
0	0	0	0.0003499	0.0001642	0.0003106	0.0031862	0	0.0623912	0.002217	0.0001803	0.000432	0.00E+00
0	0	0	0	0	0	0	0	0	0	0	0	0
0	0	0	0	2.31E-04	0.000113	0.0009624	0.0003055	0	0	0	0.0001314	0
0	0	0.00E+00	0	0	0	0	0	0	0	0	0	0
0.00E+00	6.1E-05	0.0801279	0	0.0125177	9.394E-05	0	5.368E-05	0	0	0	0.00E+00	0
6.289E-05	0	0	0	0.0033464	0.0003251	0.0034033	0.0002005	0.0005162	0.00288	0.0001175	0.0001104	0.0007903
0	0.0003602	0.0090395	0	0	0	0.002241	0.0035076	0	0.0001063	0	9.457E-05	0
0	0	0	0	0	0	0	3.385E-05	0	0	0	0	0
0	0	0	0	0	0	0	0	0	0	0	0	0
0	3.988E-05	7.732E-05	0	0.0008717	0	0	0	0	0	0	6.756E-05	5.209E-05
7.985E-05	0.0002719	8.696E-05	0	0.008779	0.000415	0.0220286	0.0004546	0.0008641	0.0114044	0.0002372	0	0.0033717
0	0	0	0	0	0	0	0	0	0	0	0.00E+00	0
0	0	0	0	0	0	0	0	0	0	0	0	0.00E+00
0	0	0	4.565E-05	5.623E-05	4.12E-05	2.505E-05	0.011769	0.0188727	0.0001743	0	9.019E-05	7.571E-05
0	0	0	0	5.438E-05	0	0	0	3.822E-05	0	0	0	0
2.6E-05	0.0154004	0.0071045	6.619E-05	0.0012478	0.0001901	0.0229333	0.0261872	0.0152018	0.0003196	5.154E-05	7.612E-05	5.91E-05
0	0	0	0	3.166E-05	3.977E-05	1.542E-05	3.166E-05	0.0009363	3.937E-05	0.0155115	2.35E-04	7.71E-04
0.0003056	0.0002939	0.0002867	0.0002762	0	0	0.0002339	1.369E-05	0	0.0018933	0.0003946	0.0007155	0.0002525
8.098E-05	6.93E-05	2.024E-05	0.035372	0	0	3.893E-05	0	0.0001803	0.0001137	9.383E-05	0.1597721	0.0001012
8.829E-05	0.0001742	4.74E-05	0.0008277	4.568E-05	0	0.0001197	0	0	0.0001851	3.784E-05	0.0130499	0.0001507
0.0001285	0.0001264	6.254E-05	0.0002045	0	0	7.775E-06	0	0	0.155951	0.3090117	0.1591448	0.2742312
3.237E-05	0	2.981E-05	2.18E-05	0.0005863	0	3.366E-05	0.002187	4.712E-05	0.0001112	0	0	0
0.1182055	0.0500087	0.0825042	8.917E-05	0	2.432E-05	0.0023279	0	0	0.0004063	6.329E-05	0.0001054	8.606E-05
4.952E-05	0.0001227	0.0506735	2.707E-05	2.584E-05	5.383E-05	0.0010602	0.0147148	4.337E-05	6.798E-05	6.398E-05	2.061E-05	1.692E-05
0	0	0	0	2.81E-04	0.1298729	0	0.0280862	0.0005403	0	0	0	0
0	0	0	0	2.727E-06	0	0	0	0	0	0	0	0
0	0.0061249	0.0023815	7.482E-06	0.0002778	0	0.0010223	3.851E-06	0	1.21E-05	1.122E-05	0	0
2.396E-05	0.0003149	1.875E-05	4.855E-05	0.0128186	0.0003479	0.0207468	0.001046	0.000803	0.0127851	8.726E-05	5.84E-05	0.0038754

COVID-19-055	COVID-19-036	COVID-19-050	COVID-19-034	COVID-19-015	COVID-19-014	COVID-19-013	COVID-19-012	COVID-19-011	COVID-19-010	COVID-19-009	COVID-19-008	COVID-19-007
0	0.0003314	0	0	0	0	0	0	0	0	0	0	0.00E+00
0	0	0	0	0	0	0	0	0	0	0	0	0
0	0	0	0	0	0.0010591	0.00026	0	0.0030818	0	0	0	0
0	0.0003193	0.0002479	0	0	0	0	0	0	0	0	0	0
0	0	0	0	0	0	0	0	0	0	0	0	0
0	0	0	0	0	0	0	0	0	0.0004118	0	0	0
0	0	0	0	0	0	0	0	0	0	0.0003214	0	0
0	0	0	0	0	0	0	0	0	0	0	0	0
0	0.0004994	0.0009613	0.0016168	0	0.0009426	0	0	0	0	0	0	0.00E+00
0	0	0	0.000303	0	0	0	0	0	0	0	0	0.000123
0	0.0002065	0.002013	0.0010983	0	0	0	0	0.000238	0.0006997	0	0	0
0	0	0	0	0	0	0	0	0	0	0	0	0
0	0	0.0001912	0	0.000342	0.00223	0.006065	0.0003851	0.0580865	0.000307	0.0010396	0.2804114	0.0012362
0.0002609	0	0.0005032	0	0.000173	0	0	0	0	0.0002928	0	0	0
0	0	0	0	0	0	0	0	0	0	0	0	0
0	0	0	0	0	0	0	0.0001681	0.0002423	0	0	0	0
0.0054885	0.0003478	0	0	0	0	0	0	0	0	0	0	0
0	0.0053233	0.000273	0.0004085	0.0092835	0.0036215	0.0183447	0.0020828	0.048987	0.0047941	0.001126	0.0032935	0.0167975
0	0	0	0	0	0	0	0	0	0	0	0	0.00E+00
0.0031148	0.0001624	0.0275908	0.0001678	0.0053818	0.0004766	0.0002999	0.0064813	0.0012299	0	0.0003052	0.0005355	0.0001178
0	0	0	0	0	0	0	0	0	0	0	0	0
0	0.0002353	0.0760832	0	0.057287	0.0042759	0.0094027	0	0.0052368	0.0021555	0.0058738	0.0022976	0.0023954
0	0.0704233	0	0.1298169	0	0	0	0	0	0	0	0	0
6.344E-05	0.069317	0	0.0045971	6.588E-05	0	8.418E-05	0	0.0003904	0	0	0	4.148E-05
0	0.000655	7.595E-05	0	0.0037202	0.0240856	0.0261112	0.0002706	0.022073	0.0300865	0.0232929	0.0124765	0.0299145
0.0002221	0.0018638	0.0048008	0.008404	0	0	7.757E-05	0.0003762	0	0	0	0	0
0	0.00067	0	0	0	0	6.172E-05	0	0	0	0.0004649	0	0
0	0	0.0003997	0	0.0021792	0	0	0	0	0	0	0	0
0	0	0	0	0.0002369	0	5.291E-05	0	0.0006845	0	0	0	5.697E-05
7.985E-05	0.0010151	4.743E-05	0	0.0066019	0.0284012	0.022797	0.0037883	0.0139808	0.0236888	0.0225551	0.0169043	0.0245789
0	0	0	0	0	0	0	0	0.0002467	0	0	0	0
0	4.557E-05	4.619E-05	0	0	0	4.865E-05	0	0	0	0	0	0
2.951E-05	0.0044771	0.0029517	5.957E-05	7.349E-05	0	4.565E-05	0	0.0061679	0	0	3.953E-05	8.128E-05
0	0	0	0	0	0	0	0	0	0	0	0	0
0.0010586	0.0698551	0	0.0164184	0.0001173	0.0002548	0	0	0	0	0.0003087	3.782E-05	0.0001674
0.0015617	0	0	0	0	0	2.719E-05	0	4.14E-05	0	3.287E-05	0	1.299E-05
4.872E-05	0.0004393	0.0001381	0	0.0006096	0.000118	0.0004127	0.0001599	0.0123877	0.0001023	0.0003966	0.0361256	6.402E-05
0.000976	0.0003632	0.0015604	0	0.0002963	0	0	7.903E-05	0	4.36E-05	0	0	0
0.0005055	5.352E-05	0.0010104	0.002796	0.000316	0.0014573	0.0002884	0.0005959	0	0.0001169	0	0.000481	0
0.0054763	0.0029318	2.197E-05	0	0	0	0	0	0	0	3.347E-05	0	0
0	0	0	0.0002356	0	0	0	0	0	0	3.334E-05	0	0
1.871E-05	0.0131198	0.0002831	2.9E-05	0.0019303	0.0002647	0.0002217	0.0001232	0	3.18E-05	0	0.0001936	0.0001861
0	0.0158166	6.367E-05	2.861E-05	0.0008748	8.305E-05	0.000112	0	0.0007219	0	5.998E-05	3.353E-05	3.353E-05
1.376E-05	5.94E-05	0	1.995E-05	0	0.0002582	0	0	4.977E-05	2.546E-05	0.0004121	0	0.0001601
0	0	8.182E-06	0	0	0	0	0	0	0	0	0	0
0	3.257E-05	1.518E-05	0	0	0	9.242E-06	8.142E-06	3.62E-05	1.32E-05	2.079E-05	0	0
2.595E-05	0.0010826	0.00011	3.09E-05	0.0062049	0.0181714	0.0198695	0.0016506	0.017189	0.0210282	0.0194927	0.0113777	0.0227673

COVID-19-006	COVID-19-005	COVID-19-004	COVID-19-002	COVID-19-001	COVID-19-035	COVID-19-016	COVID-19-017	COVID-19-003	COVID-19-019	COVID-19-018	COVID-19-032	COVID-19-030
0	0	0	0.0002373	0.0002659	0	0	0	0	0	0	0	0
0	0	0	0	0	0	0	0	0	0	0	0	0
0	0	0	0.0004321	0.0002638	0	0	0	0.001258	0	0	0.0537748	0
0	0	0.0133539	0	0	0	0	0	0	0	0	0	0
0	0	0	0	0	0	0	0	0	0	0	0	0
0	0	0	0.0002339	0	0	0	0	0	0	0	0	0
0.0001591	0	0.00E+00	0.0002323	0.0001687	0	0	0	0	0	0	0	0
0.0003084	0	0	0	0	0	0	0	0	0	0.0002108	0	0
0.0001592	0	0	0	0.0110492	0.001339	0	0	0	0.0001904	0	0	0.0002622
0	0	0	0	0	0.0432628	0	0	0	0	0	0.000192	0.00015
0.00E+00	0	0	0	0.017042	0.0006624	0	0	0.0003957	0.0001577	0	0	0
0	0	0	0	0	0	0	0	0	0	0	0	0
0.0005871	0.0008564	0	0	0	0	5.656E-05	0.0250843	0.0008214	0	0.0007406	0	0
0	0	0.0001411	0	0	0	0	0	0.0028566	0	0	0.0001597	0
0.0002243	0.0001795	0.00E+00	0	0	0	0	0	0	0	0	0	0
0.00E+00	0	0	0	0	0	0	0	0	0	0	0	0
0	0	0.0001788	0	0	0.0009111	0	0	0	0	0	0.0050379	0.0014548
0.0104434	0.0006519	0.0001609	0.0001312	0.0006032	0.017896	0.0008149	0.0045507	0.1528981	0.0063689	0.0043623	0.0095248	0.0057508
0	0	0	0	0	0	0	0	0	0	0	0	0
0.0002606	0	0.0002749	0.0003374	0.0408962	0.0002945	0.0002035	0.0002981	0.0013602	9.639E-05	0.0003606	0	0.0002035
0	0	0	9.853E-05	0	0	0	0	0	0	0	0	0
0	0.0076444	0	0	0.0058677	0	9.47E-04	0.0516652	0	0.0105515	0.0044669	0.0001069	5.5E-05
0	0	0.00E+00	0	0	0.0535359	0	0	0	0	0	0.0893546	0.0963761
0	0	0	0	0	0.1546557	0	0	0.0002025	0	0	0.0005954	0.0615746
0.0488903	0.0238613	0.0001566	0.0001483	7.239E-05	0.0008556	0.036504	0.0027744	0.0244321	0.0161884	0.0296581	0.0004794	6.171E-05
0.0001148	7.544E-05	0.0002104	5.525E-05	0.0260388	0.0057614	0	3.825E-05	0	0	0	7.544E-05	0.0002168
0	0	0	0	0	0	0	0	0	0	0.00E+00	0	0
0	0	0	0	0	0	0	0	0.0001524	0	0	0	0
8.628E-05	0	0	0	0	0	0	2.767E-05	0	5.046E-05	6.349E-05	0	3.093E-05
0.0198017	0.0215037	0.0001518	0.0009969	0.0001336	0.0013052	0.0211084	0.0039116	0.0165169	0.0173676	0.0248612	0.0031843	0.0003391
0	0	0	0	0	0	0	0	0	0	0	0	0
5.173E-05	0	0	0.0004028	0.0001127	0	0	0	0	0	0	0	0
0.0001464	0	0	0.0002127	0.0055232	4.565E-05	5.79E-05	0.000211	7.905E-05	0.0001214	0	0.0067853	4.509E-05
6.191E-05	5.276E-05	0	0	0.0001454	0	0	0	0	0	0	0	0.0001131
0	0	3.404E-05	0.0002615	5.437E-05	0.0746948	0.0001674	0.0002809	0	0	0.000209	0.0042265	0.0090742
0.0006108	0	0.0028583	2.232E-05	0	0	0.0139641	0.0001327	0	0	2.76E-05	0	0
5.597E-05	0.0001264	0	0.0001586	0.0008701	0	3.02E-05	0.0010864	0	0.0004743	0.0001144	0	4.308E-05
1.791E-05	2.959E-05	0.0006615	0	0	0	2.92E-05	0	0.0059247	0	0	5.373E-05	0
0.0006872	0.0005805	0.2894149	0	0	0	0	0	0.0018749	0	0	0	8.863E-05
0	0	0	0	0	5.206E-05	0	0	0	0	0	0	6.119E-05
0	0	0.00E+00	0	0	0	1.09E-05	0	0	0	1.955E-05	0	0
0.0001621	7.577E-05	0.0009432	0	0.0021611	0.0102706	0	0	0.0001874	7.67E-05	0	0	4.272E-05
2.65E-05	2.338E-05	0.0002172	0	0.0021718	0.0010249	3.383E-05	2.461E-05	0.0002476	0	6.736E-05	0	0
1.33E-05	3.578E-05	0	8.898E-05	0.0001117	0	0.0001371	0.0002894	0	0	0.0004394	0	0
0	0	0	0.0001029	4.234E-05	9.61E-06	0	0	0	7.403E-06	0	0	1.182E-05
0	0	7.372E-06	0.0001508	0.0001245	0	0	0	0	7.262E-06	0	0	0
0.0261473	0.0162435	0.000111	0.0014845	0.00012	0.0036658	0.0208577	0.0036582	0.0195154	0.0157873	0.0208098	0.0053917	0.0009732

COVID-19-029	COVID-19-028	COVID-19-027	COVID-19-026	COVID-19-031	COVID-19-024	COVID-19-020	COVID-19-025	COVID-19-021	COVID-19-033	COVID-19-022	COVID-19-023	SC001
0	0	0	0	0	0	0	0	0	0	0	0	0
0	0	0	0	0	0	0	0	0	0	0	0	0
0	0	0	0	0	0.0082628	0	0.0078383	0	0.0003594	0.1158698	0	0
0	0.0020472	0	0	0.014785	0	0	0	0	0	0	0	0
0	0	0	0	0	0.0003631	0	0	0	0	0	0	0
0	0	0	0	0	0	0	0	0	0	0	0	0.00E+00
0	0	0.0002482	0	0	0	0	0	0	0	0	0	0.00E+00
0	0	0	0.0057803	0	0.0001668	0	0	0	0	0	0	0
0.0005056	0.0022473	0	0	0.0031712	0	0.0001779	0	0.0692357	0.0647192	0.0009926	0.0018915	0
0	0	0	0	0.000285	0.000324	0	0	0	0.0006929	0	0	0
0.0004674	0.0014453	0.0002122	0	0.0034612	0	0.0001606	0	0.0558144	0.0530673	0.0009635	0.0066671	0
0	0	0	0	0	0	0	0	0	0	0	0	0
0	0	0.0004013	0	0	0	0.0003259	0.0016024	0.0007622	0	0.0004929	0	0.0003932
0.0016107	0	0.0002236	0	0	0	0	0	0	0.0002449	0	0	0.0773116
0	0	0	0	0	0	0	0	0	0	0	0	0
0	0	0	0	0	0	0	0	0	0	0	0	0
0	0.0002376	0	0	0.0382653	0.0017438	0	0	0	0.0005388	0	0	0
0.0016975	0.002741	0.0419408	0.0090528	0.0121409	0.004646	0.0003662	0.0026415	0	0.0181669	0.0003344	0.0219705	0
0	0	0	0	0	0	0	0	0	0	0	0	0
0.0001196	0	0	0	0	0.0007319	0.0027864	0.000407	0	0.0001642	0.1032196	0	3.21E-05
0	0	0.0002586	0	0	0	0	0	0	0	0	0	0
0.0028827	0.000249	0.0008509	0	0.0001619	0.0001726	0	0	0	0	0.0158769	0	0.0001253
0.0969378	0.0096313	0.0645945	0.1864854	0.0133026	0.0608799	0	0.0032738	0	0.0013006	0	0.119114	0
0.0181934	0.2387719	0.0359389	0.0398553	0.0048729	0.2045483	0	0.0073569	0	0.0026304	0	0.0224379	0.00E+00
0	0.0007642	0.0068541	0	0.0009612	0.0001388	0.0001163	0.0012389	0.0002314	0.0002456	0.0006408	0.001513	3.09E-05
0.0091999	0.0017777	0.0016576	0.000272	0.0193413	5.951E-05	0	0.0002285	0.0574118	0.0267667	0.0181724	0.006366	5.951E-05
0	0.0001653	0	0	0	0	0	0	0	0	0	0	0
0	0	0.0003694	0	0	0	0	0	0	0	0	0.0006088	0
0	0	0	0	0	0	0	0	0	0.0001579	0	0	0.00E+00
0	0.0001439	0.0064793	0.0014206	0.0024143	0.0003597	0.000253	0.0051583	7.985E-05	0.0013653	0.0001352	0.001136	5.45E-05
0	0	0	0	0	0	4.048E-05	0	0	0	0	0	0.00E+00
0	6.097E-05	0	0	0	0	0	0.0001293	0	0	0	0	0.0008425
4.621E-05	0.0017988	0	7.738E-05	0.0089748	0.0013311	3.062E-05	0.0041464	0.0003357	0.0003034	0	0.001507	5.122E-05
0	0	0	0	0	0	0	0	0	0	0	0	0
0.0304226	0.2678243	0.020147	0.0016147	0.0154586	0.0005305	5.626E-05	0.0030151	3.924E-05	0.0810825	0	0.0524386	8.038E-06
0	7.346E-05	3.368E-05	0	0.0001636	0	1.786E-05	8.36E-05	0	0	0	0.0001696	8.523E-06
6.362E-05	3.302E-05	0	0	4.55E-05	0.0040572	2.698E-05	0.0008198	7.047E-05	0	0	0.0001526	2.335E-05
0.0030826	3.66E-05	0.0004384	4.516E-05	0	0	0	0	0	3.582E-05	0	7.28E-05	0.3370053
0.0006211	3.034E-05	0.0002052	0	0	0	0.0003535	0.0011989	0.0003283	0.0002127	0.0012742	7.022E-05	0.0064002
2.468E-05	0.0013457	0.0072956	0.0004415	6.119E-05	0.021485	0	0.0001335	0	0.0005987	0	0.0002126	1.048E-05
9.712E-05	0	0	0	0	7.533E-05	0	0.0002619	0	0	0	0	0
0.0010773	0.0006061	0.0017208	3.648E-05	0.0003084	0.0019821	0.0494238	0.0011437	0.000289	3.056E-05	0.0003268	0.0003932	2.027E-05
0	2.307E-05	0.001366	0.000342	0.005666	0.0001569	0	0.0065632	3.014E-05	0.0002405	0	0.0002559	0
0	0.0001211	7.866E-05	5.527E-05	8.898E-05	0	1.468E-05	0	0	2.27E-05	0	0	0
0	0	0	0	0	0	0	0	0	0	0	0	0
8.802E-06	1.881E-05	0	0	0	6.712E-06	0	0	0	0	0	0	0
0.0002919	0.0002437	0.0107181	0.0007557	0.001388	0.0007612	0.000111	0.0034817	0.0001733	0.0005699	0.0002726	0.0017143	2.965E-05

SC021	SC022	SC023	SC024	SC025	SC026	SC027	SC028	SC029	SC030	SC031	SC032	SC033
0	0	0	0	0	0	0.0001186	0	0	0.0003518	0	0	0.0037678
0.3640406	0.0100764	0.0016781	0.0016317	0.5525723	0.0013417	0.0014654	0.0014113	0	0	0.0003557	0	0
0.0003021	0.0015485	0.0008259	0.1635383	0.0030245	0	0.0002294	0	0.0007418	0.0136922	0.0011203	0.0074292	0.0816908
0.0003193	0.001341	0	0.146577	0.0016566	0	0.0005184	0	0	0	0	0	0
0	0	0	0	0	0	0	0	0	0	0	0	0
0	0	0	0	0	0	0	0	0	0.0063485	0.0002965	0.0001944	0.000761
0	0	0	0	0	0	5.03E-04	0	0	0.0002355	0	0	0.0061676
0	0	0.0002423	0.0001196	0	0	0.0099841	0	0	0	0	0	0
0.0086958	0.0032898	0	0.0003215	0.0380637	0.0005056	8.74E-05	0.000977	0	0.0002528	0.0002872	0	0.0001529
0.0019979	0.0165468	0.0022078	0.0030748	0.0008669	0.0031768	0.0011309	0.0018449	0	0	0	0	0
0.0028762	0.0043702	0	0.0001176	0.0349586	0.0004387	0.0001118	0.0005792	0	0	0.0002409	0	0.0001548
0	0	0	0	0	0	0	0	0	0	0	0	0
0	0	0	0	0	0	0	0	0	0.0102045	0	0	0
0.0035301	0.0255496	0.0021058	0.0040679	0.0011953	0.0075022	0.67777	0.0024493	0	0	0	0	0
0	0	0	0.0001346	9.501E-05	0	0	0	0	0	0	0	0
0	0	0	0	0.0011323	0	0	0.0015947	0	0	0	0	0
0	0.0116873	0	0.0186992	6.613E-05	0.0321498	0.0012221	0.0007078	0	0	0	0	0.00277
0	0	0.0056239	0.0019536	0.0021695	0.0003598	6.562E-05	0	0.0038501	0.006225	0.0008593	0.0040449	0.0016425
0	0	0	0	0	0	0	0	0	0	0	0	0
0.053252	0.0012388	0.0019724	0.0046446	0.0192727	0.0010299	0.000116	0.0005641	0	0.0003284	0	0.0002017	8.925E-05
0	0	0	0	0	0	0	0	0	0	0	0	0
0.0025099	0	0.0032218	8.555E-05	0.0002505	0.0018439	0	0	0.0134281	0.0424397	0.0365873	0.0401773	0.0029102
0	0	0	0	0	0	0.00E+00	0	0	0	0	0	0
9.15E-05	0	0	0	0	0	0.00E+00	0	0.0001342	0.0002257	0	8.052E-05	5.734E-05
0.0001032	0	0.0081013	0	0	0.0003596	3.679E-05	0	0.0176385	0.003395	0.0063937	0.0030533	0.0009909
0.0323113	0.0038965	0.000526	0.0003337	0.0491151	0.0006546	0.0002444	0.0007544	0	0	0	6.801E-05	0.0001551
0	0	0	0	0.0003156	0	0	0	0.0002061	0	0	0	0
0	0	0	0	0	0	0	0	0	8.599E-05	0	0	0
0	0	0	3.825E-05	0	0	0	0	0	0.0002059	7.163E-05	0	0
0.0002134	0	0.020287	0.0008	8.775E-05	0	0.0002553	0.0004063	0.0178253	0.0030989	0.0070454	0.0048967	0.0047322
0	0	0	0	0	0	0	0	0	0	0	0	0
0	0.000481	0	0	0	0	0	0	0	0	0	0	0
0	0.0002138	0	6.514E-05	0.0017275	0	0.0010817	0	0	0	4.454E-05	0	3.34E-05
0	0	0	9.529E-05	0	0	0.00E+00	0	0	0	0	0	0
4.444E-05	0	3.83E-05	0	0.0001121	7.896E-05	1.277E-05	0.0001962	0	4.303E-05	0	5.059E-05	0
0.0001376	0	0	0	0	0	0	0.0001161	0	0	0	4.667E-05	0
0	0.0002903	0	2.295E-05	0.0004208	0	0.0007739	0	3.906E-05	3.342E-05	0	2.094E-05	0.000145
0	0.0001472	0	0	1.246E-05	0	0.0003492	0	0	0	0	0	0
0	0	6.75E-05	8.897E-05	1.398E-05	0	3.92E-03	0	0	5.522E-05	0	0	0
0	0	0	0	0	0	0.00E+00	0	0	0	0	0	0
0	0.000117	0	0	0	0	9.937E-06	0	0	2.628E-05	0	0	0.0006138
0.0053663	0.0165168	0.0006934	0.0214946	0.000545	0.0009422	0.1417287	0.0116447	0	0	0	0	0.0017598
0.004575	0.0021436	0.0072799	0.0390475	0.0009104	0.0194405	0.0113274	0.0983321	0	0.0001679	0	1.907E-05	0
6.743E-05	8.83E-05	1.926E-05	2.5E-05	2.706E-05	0.0002484	1.67E-05	0	0	0	0	2.867E-05	0
0	5.039E-05	0	0	0	0	0	0	0	0	0	6.753E-06	0
1.012E-05	4.126E-05	7.042E-06	1.881E-05	0	0	0.0005773	2.74E-05	0.0001064	0.00031	0	3.147E-05	0.0059363
3.833E-05	0.0001795	0.0114599	0.0018211	9.895E-05	5.9E-05	0.0001561	0.0003812	0.014039	0.0029865	0.0055471	0.0047775	0.0066218

SC034	SC035	SC020	SC019	SC036	SC017	SC002	SC003	SC004	SC005	SC018	SC007	SC008
0	0	0	0	0	0	0	0	0	0	0	0	0
0	0	0.0039671	0.0022581	0	0	0	0.0024669	0	0	0.0022349	0.0008429	0
0.0025274	0.0003671	0.0001912	0.0004244	0	0.0005315	0.0025465	0.0003479	0	0	0.0005468	0.3268932	0
0	0	0.0004245	0.0192326	0	0.0015176	0.0020885	0.0006874	0.0010518	0	0.0002968	0.2305918	0
0	0	0	0	0	0	0	0	0	0	0	0.0002578	0
0.0007281	0.0007841	0	0	0.5838281	0	0	0	0	0	0	0	0.00E+00
0.000226	0	0	0	0	0	0	0	0	0	0.0002228	0	0
0	0	0	0	0	0.0023914	0.002533	0.001117	0.0014883	0	0	0.8990422	0
0.0002435	0.000181	0.0213837	0.0382978	0	0.000181	0.001673	0.0350642	0.00E+00	0.0017042	0.0889807	0.0003527	0
0.000651	0	0.91837	0.0025918	0	0	0	0	0	0	0.0014279	0.000129	0
0	0	0.0218079	0.0562073	0	0.0003871	0.0003957	0.0548509	0	0	0.0859096	4.93E-04	0
0	0	0	0	0	0	0	0	0	0	0	0	0
0.0151465	0.0006275	0.0001804	0	0.0006706	0	0	0.0012119	0.00E+00	0	0	1.33E-02	0
0	0	0.0012859	0.002585	0	0	0	0	0	0.0062004	0.0011341	0	0.0005804
0	0.0001478	0	0	0	0	0	0	0	0	0	0	0
0	0	0.0002744	0	0	0	0	0	0	0	0	0.0003659	0
0	0.0014425	0	0.0003257	0	0.0179448	0.0427154	0	0.0001665	0	0	0.0014695	0
0.0017229	0.0026415	0.0008064	0.0005567	0.0002963	0	0	0	0.0001228	0	0.0029252	0	0
0	0	0	0	0	0	0	0	0	0	0	0	0
0	0	0.0304736	0.0314714	0.0004623	0.0004909	0.002192	0.0142051	0.0002178	0.0010228	0.0062493	0.0004123	0.0003873
0	0	0	0	0	0	0	0	0	0	0.000117	0	0
0.0381974	0.0115231	7.944E-05	0.0001864	0.0011946	0	0	0	0	0.0008692	0.0002017	0.0002628	0.0001451
0	0	0	0	0	0	0	0	0.00E+00	0	0.000108	0	0
0	0.0160326	0	0.0003062	0	0	0	0	6.344E-05	0	0.0002696	0.00E+00	0
0.0136323	0.015264	6.052E-05	0	0	0	0	0	0	0	0	7.001E-05	0.00E+00
0	0.0005366	0.0421105	0.0344003	0.0002678	0.0005908	0.000458	0.0344428	0.0001211	0	0.0337011	0.0001573	0
0	0	0	0	0	0.0014156	0.0032006	0.0021135	0.8250014	0	0.000221	0.0009258	0
0	0	0	0.000255	0	0	0.0465928	0	0	0	0	4.495E-05	0
0	0	0	0	0	0	0	0	0	0	0	0	0
0.0094771	0.0081087	5.06E-05	7.985E-05	0.0001921	0	0	0	0	0	0.0001154	0.0003905	4.585E-05
0	0	0	0	0	5.838E-05	0.2381962	0.0001331	0.0001884	0	0	0	0.00E+00
0	0	0	0	0	0	0	0	0	0	0	0	0
4.843E-05	3.841E-05	0	0.0001342	0	0.0001492	8.072E-05	0.0002455	0.000245	0	0.0001152	0.0068131	7.627E-05
0	0.0001346	0	0	0	0	0	0	0	0	0	0	0
3.262E-05	0	0	0.0075059	0.0001768	2.979E-05	7.045E-05	0	0	0	0.0001064	0	0
0	3.937E-05	0	0	0	0	0	0	0	0	0	2.192E-05	0
2.738E-05	2.295E-05	0	0	0.0121804	0	0	0	0	0	0	0	3.825E-05
0	0	0	0	0	0	0	0	0	0.0095189	0	0	0.0013307
0	6.511E-05	0	4.772E-05	0	0.0003794	0.00107	0.0005843	0.0003487	0.005639	0	0.0002332	0.3172457
0	0	0	0	0	0	0	0	0	0	0	0	1.758E-05
0	0	0	0	0	0	0	0	0	0	0	0	0
0	1.871E-05	0.0011193	0.002428	0	8.013E-05	0.0002398	0.0010392	8.761E-05	0.0003611	0.0006941	0.0138952	0.0001855
0	0	0.0002744	0.0007093	0	0.1146875	0.0002919	0.0001873	3.383E-05	0.0215004	0.0002049	0.0155241	1.907E-05
0	0	0	0	9.655E-05	0	6.353E-05	0	0	0	0	0	0
0	7.792E-06	0	1.312E-05	0	8.312E-06	0	9.221E-06	0	0	0	0	0
0.0001078	1.958E-05	0	2.685E-05	2.96E-05	5.391E-06	0	0	0	0	2.465E-05	0	0
0.0087811	0.0074068	4.716E-05	9.337E-05	0.0007255	7.998E-06	0	3.327E-05	9.884E-06	0.000542	8.22E-05	0.0006058	7.36E-05

SC006	SC010	SC011	SC012	SC013	SC014	SC015	SC016	SC009	
0	0.0004827	0.00E+00	0	0	0	0	0	0	
0	0	0.0226467	0	0	0	0	0.0012334	0	
0.0007724	0.0008297	0.0005697	0.0002026	0	0.0003021	0.0016633	0.0006347	0	
0.0003907	0.0008226	0.0003531	0.0006085	0	0	0.0030427	0.0019157	0.2400804	
0	0	0	0	0	0	0	0	0	
0	0	0	0	0.00E+00	0.0003294	0	0	0	
0	0.0021386	0.00E+00	0	0	0	0	0	0	
0.0038892	0.0021365	0.0017401	0.0010887	0	0	0.0055348	0.0013593	0.0014726	
0.0090485	0.0003589	0.0011923	0.0669103	0	0.0948175	0	0.0418092	0.0002497	
0	0	0	0	0	0	0	0	0	
0.0081611	0.0013334	0.0030368	0.0835811	0	0.0960293	0.0012933	0.068274	0.0002093	
0	0	0	0	0	0	0	0	0	
0	0	0.0001374	0.0001266	0	0.0002451	0	0	0	
0	0	0.0004792	0	0.0001464	0	0	0.000205	0	
0	0	0.0001267	0	0.00E+00	0	0	0	0	
0	0	0.00022	0	0	0	0	0	0.1163236	
0	0	0	0	0	0	0	0	0.0040705	
0.0021589	0.0002561	0.0006985	0.0008932	4.51E-03	0.0003641	0	0.0036512	0	
0	0	0	0	0	0	0	0	0	
0.1092993	0.0017761	0.0211648	0.0099032	0.0001946	0	0.0015851	0.0602385	0.0005069	
0	0	0	7.544E-05	0.0054037	0	0	0	0	
0	0	6.26E-05	0	0	0.0005194	0.0012863	0	0	
0	0	0	0	0	0	0	0	0	
0	0	0	0	0	0	0	0.0034686	0	
0	0.0001377	0	4.865E-05	0.0057066	0.0004747	0	0.0001104	0	
0.0294635	0.0029221	0.0046297	0.0308428	0	0.0287665	0.0013707	0.0338828	0	
0.0027167	0.0017939	0.0011608	0.0006979	0	0	0.0054405	0.0010304	0.0017989	
0.0001114	0	0	0	0	0	0.000387	6.254E-05	5.863E-05	
0	0	0	0	0	0	0	0	0	
9.17E-05	0	0	0	0.0331912	0.000355	0	0	0	
0.0001884	0.00026	1.80E-04	0	0	0	0.0009916	5.916E-05	0.0003798	
0	0	0.00E+00	0	0	0	0	0	0	
0.0026416	0.0665566	6.903E-05	0.0002867	0.004545	5.4E-05	0.0002422	0.0007588	0.0172521	
6.46E-05	0	6.891E-05	0	0	0.000133	0	0	0	
0.000661	0.0122089	2.13E-05	6.241E-05	5.579E-05	0	0.0004095	3.924E-05	3.357E-05	
0	0	0	0	0	0	0.000183	0	0	
0	0	0	0	2.658E-05	6.885E-05	0	0	0	
8.954E-05	0	5.68E-04	0	0.0009842	0.0001643	0	0	3.037E-05	
0.000904	0.0007094	0.2882388	0.0001889	0.0005505	0	0.0023382	0.0007639	0.000631	
0	0	0	0	1.555E-05	0	0	0	0	
0	0	0	0	0	3.205E-05	0	0	0	
0.0001419	0.0001868	9.011E-05	0.0002925	0	0	0.0655116	0.0001942	0.027652	
0.000343	0.011005	0.0003583	8.828E-05	0.0006558	0	0.0011654	0.0004648	0.0003466	
0	0	0	4.335E-05	0	0	0	0	0	
0	1.507E-05	0	0	8.701E-06	0	0	0	0	
0	0.0026608	1.045E-05	5.831E-06	0	9.682E-06	0	0	0	
0.0001483	0.0001692	2.15E-05	2.373E-05	0.0233539	0.0002205	1.637E-05	0.000103	4.64E-06	

Supplementary table S16. The average abundance and composition at the genus level among COVID-19 (n=71), SC (n=36), Post-COVD-19 (n=22), and SCR (n=36) groups.				
ID	COVID-19	Post-COVID-19	SC	SCR
Acrodictys	0.273025479	0.012352091	0.153844361	0.225685667
Zanclospora	0.128900817	0.197568591	0.030687861	0.217046111
Aspergillus	0.089071901	0.023247409	0.141242306	0.026150667
Candida	0.055329873	0.062074955	0.131445611	0.012421333
Cladosporium	0.039672338	0.016867909	0.091582	0.071947556
Blumeria	0.044938958	1.71E-05	0.128652361	0.026109417
Actinomucor	2.56E-06	0.335261409	9.61E-06	0.025432889
Malassezia	0.029725394	0.052714273	0.050929111	0.082169917
Chrysosphaeria	0.066193887	0	8.81E-04	1.41E-04
Fungi_gen_Incertae_sedis	0.021221451	0.006595227	0.054046861	0.013336667
Hydnobolites	0.040220437	3.52E-05	4.03E-05	0.001323444
Simplicillium	0.020548577	0.001624727	0.009427917	0.011429806
Saccharomyces	0.02825969	7.42E-04	0.001371111	0.001236139
Alternaria	0.002753169	0.013418864	0.006140667	0.022140778
Udeniomyces	1.86E-05	0.064714591	2.84E-04	5.75E-04
Diversispora	0.004459014	0.001425227	0.010277389	0.020178667
Russula	0.006937141	1.40E-05	0.008792278	0.009571333
Filobasidiaceae_gen_Incertae_sedis	0.008852056	7.69E-05	0.00392125	0.008247833
Galactomyces	0.003749718	0.010632	8.89E-07	0.014582194
Gibberella	0.011924648	0.002501	5.04E-04	4.67E-05
Rhexothecium	2.49E-04	0	0.02432775	2.00E-05
Aureobasidium	0.002952437	0.004585227	0.012679722	0.002306056
Penicillium	0.003517831	0.006055636	0.009427194	0.002230083
Rhodotorula	0.001000746	0.030985727	2.43E-05	7.18E-04
Pleurotus	0.007054535	6.67E-04	0.005522472	0.001426278
Neomicrosphaeropsis	0.009276366	0.001510682	2.47E-05	0.001692333
Debaryomyces	3.11E-04	0.030895	2.17E-05	7.59E-04
Cookeina	3.87E-06	0.001363591	8.75E-06	0.017738
Nigrospora	0.007665718	0.002618682	6.38E-04	0.001205694
Inocyclus	6.90E-07	7.34E-05	0	0.018313972
Raffaelea	0.005000901	0.007370591	0.001732833	0.001612806
Kazachstania	8.36E-04	0.003523955	6.79E-04	0.012403028
Angustimassarina	0.004806141	3.92E-04	0.002974056	0.003392361
Yarrowia	1.74E-04	0	0.00259325	0.0127215
Parafuscosporella	0.005994014	1.71E-04	0.001566889	0.001780028
Novakomyces	1.17E-05	0.001345955	0	0.01395225
Botryosphaeria	6.95E-05	0.011922227	2.50E-06	0.007304167
Filobasidium	0.003139592	0.003336909	0.002895889	0.001647972
Entoloma	3.66E-06	3.82E-06	0	0.011148611
Sterigmatomyces	5.84E-04	0	0.009081639	6.13E-04
Fantasmomyces	0.004061169	3.37E-05	0.0012935	0.00127975
Caloplaca	0	1.43E-04	0	0.010405528
Ustilaginoidea	0.001433056	0.003350318	0.003641083	0.001740056
Chloridium	8.59E-07	4.09E-06	0	0.0101955
Dipodascus	7.51E-04	0.005336273	0	0.005268694
Dioszegia	9.72E-05	0.007831273	0.005002222	0
Fusarium	0.001968254	0.003166864	0.001904639	0.002252667
Mucor	6.85E-06	0.013766409	0	0.00127875
Didymocyrtis	2.15E-04	0	0.008834139	0
Pseudoveronaea	0.002956014	0	7.78E-04	4.04E-04
Others	0.060053704	0.057663727	0.080264667	0.064417444

Table S17. The detailed values of oral fungal diversity index and observed OTUs among COVID-19(n=71), Post-COVID-19(n=22), and controls (n=132)								
sample	reads	label	ace	chao	shannon	simpson	coverage	observed_otus
Post-COVID-19-001	1161	0.97	204.205991	148.142857	2.26548	0.227925	0.970715	68
Post-COVID-19-022	1161	0.97	123.299254	94.083333	3.373911	0.050489	0.980189	73
Post-COVID-19-021	1161	0.97	41.933099	39.6	2.484253	0.123005	0.993109	34
Post-COVID-19-020	1161	0.97	37.540007	39	1.362154	0.485258	0.993109	32
Post-COVID-19-018	1161	0.97	55.29644	56.2	2.169348	0.256307	0.989664	43
Post-COVID-19-017	1161	0.97	22	21	0.830195	0.626092	0.994832	18
Post-COVID-19-016	1161	0.97	116.693813	75.3	1.429335	0.498883	0.980189	50
Post-COVID-19-015	1161	0.97	62.7877	61.666667	2.319243	0.207137	0.988803	53
Post-COVID-19-014	1161	0.97	38.287725	41	1.409094	0.391335	0.991387	32
Post-COVID-19-013	1161	0.97	42.218149	42	1.037398	0.646476	0.992248	36
Post-COVID-19-012	1161	0.97	119.666708	87.333333	2.6952	0.096774	0.985357	42
Post-COVID-19-019	1161	0.97	76.377462	83.857143	2.280684	0.260247	0.984496	62
Post-COVID-19-010	1161	0.97	53.272	60.333333	2.522192	0.144758	0.990525	42
Post-COVID-19-011	1161	0.97	42.862475	50	1.795625	0.272212	0.991387	35
Post-COVID-19-002	1161	0.97	72.24284	75	2.874572	0.088561	0.986219	55
Post-COVID-19-004	1161	0.97	89.753483	91.272727	2.881441	0.126266	0.982773	74
Post-COVID-19-005	1161	0.97	94.046472	86.076923	1.627045	0.467767	0.978467	63
Post-COVID-19-003	1161	0.97	44.994138	41.8	1.268717	0.572065	0.993109	39
Post-COVID-19-007	1161	0.97	67.875149	61	0.336671	0.879557	0.991387	16
Post-COVID-19-008	1161	0.97	44.186526	43.666667	1.797651	0.388678	0.993109	39
Post-COVID-19-009	1161	0.97	44.257568	44.2	2.149065	0.219705	0.993971	40
Post-COVID-19-006	1161	0.97	44.286052	41	0.971574	0.630558	0.989664	30
COVID-19-051	1161	0.97	187.602734	209.5625	3.660507	0.062593	0.956934	133
COVID-19-046	1161	0.97	139.489599	118.214286	1.779718	0.335989	0.96727	68
COVID-19-050	1161	0.97	316.391581	212.545455	2.770248	0.123633	0.955211	92
COVID-19-049	1161	0.97	848.249242	157	0.331303	0.885793	0.985357	21
COVID-19-048	1161	0.97	531.467374	339.125	2.70861	0.264882	0.919035	157
COVID-19-047	1161	0.97	101.777416	85.5	1.508825	0.448113	0.981051	47
COVID-19-045	1161	0.97	184.473842	124.333333	2.1401	0.206212	0.970715	62
COVID-19-040	1161	0.97	235.77996	103.25	1.556162	0.37698	0.980189	40
COVID-19-043	1161	0.97	268.371365	234.55	3.66058	0.050965	0.946598	140
COVID-19-042	1161	0.97	86.238635	77	1.031194	0.592878	0.982773	39
COVID-19-041	1161	0.97	220.26437	114.666667	2.846907	0.08374	0.973299	63
COVID-19-039	1161	0.97	718.616042	403.157895	2.685407	0.258907	0.91559	153
COVID-19-038	1161	0.97	387.82118	269	2.398014	0.332885	0.933678	136
COVID-19-052	1161	0.97	128.50878	99	1.650383	0.469497	0.976744	60
COVID-19-044	1161	0.97	613.555391	374.588235	2.374443	0.333259	0.922481	139
COVID-19-053	1161	0.97	206.513438	174.875	3.017554	0.115036	0.96727	87
COVID-19-065	1161	0.97	372.916051	104	1.632065	0.333212	0.981912	34
COVID-19-055	1161	0.97	89.077493	59.857143	1.117057	0.614781	0.984496	38
COVID-19-071	1161	0.97	94.584173	82.5	2.404128	0.179839	0.983635	54
COVID-19-070	1161	0.97	43.715735	42.166667	2.042991	0.202291	0.990525	33
COVID-19-069	1161	0.97	102.670873	86.75	2.833069	0.129892	0.982773	63
COVID-19-068	1161	0.97	84.514889	71.666667	2.630853	0.119444	0.985357	49
COVID-19-067	1161	0.97	51.730104	51	0.975967	0.565194	0.986219	27
COVID-19-066	1161	0.97	164.047733	124.75	2.987428	0.127041	0.969854	95
COVID-19-037	1161	0.97	272.802393	205.8	3.553794	0.063068	0.94832	135
COVID-19-064	1161	0.97	277.61828	196.65	2.386726	0.310738	0.950043	114
COVID-19-063	1161	0.97	151.625555	147.428571	2.394678	0.197268	0.971576	72
COVID-19-062	1161	0.97	106.576254	104.4	3.32563	0.061818	0.979328	86
COVID-19-061	1161	0.97	94.610612	90.714286	2.937261	0.09463	0.979328	71
COVID-19-060	1161	0.97	462.156197	266	1.823761	0.319221	0.94832	89
COVID-19-059	1161	0.97	333.901698	100	0.516028	0.824471	0.981912	30
COVID-19-058	1161	0.97	33.207015	32	1.222445	0.401215	0.992248	26
COVID-19-056	1161	0.97	195.426883	147.571429	2.466214	0.205444	0.964686	89
COVID-19-054	1161	0.97	33.936017	30.25	0.31763	0.896694	0.991387	19

COVID-19-036	1161	0.97	219.751529	153.8125	3.357808	0.065045	0.963824	100
COVID-19-057	1161	0.97	301.417131	119.857143	0.928618	0.663082	0.972438	49
COVID-19-034	1161	0.97	107.938782	134	1.852562	0.378338	0.971576	68
COVID-19-014	1161	0.97	451.441662	337.75	2.865736	0.240612	0.925926	155
COVID-19-013	1161	0.97	419.519902	236.387097	2.602492	0.289792	0.933678	142
COVID-19-012	1161	0.97	152.157191	100	1.938388	0.384781	0.976744	61
COVID-19-011	1161	0.97	224.422011	216.115385	3.031965	0.197648	0.945736	141
COVID-19-010	1161	0.97	427.255455	318.789474	2.524395	0.316374	0.929371	144
COVID-19-009	1161	0.97	494.725222	319.5	2.761421	0.276614	0.921619	162
COVID-19-015	1161	0.97	121.960285	124.0625	2.848519	0.103129	0.970715	89
COVID-19-008	1161	0.97	386.676842	278.4	2.930965	0.138677	0.940568	122
COVID-19-006	1161	0.97	487.081935	306.105263	1.891302	0.465179	0.92851	127
COVID-19-005	1161	0.97	416.045656	264.142857	2.974694	0.193126	0.937123	139
COVID-19-004	1161	0.97	53.104915	51.5	0.859756	0.706987	0.98708	34
COVID-19-002	1161	0.97	217.174298	128.75	1.124585	0.54092	0.976744	41
COVID-19-001	1161	0.97	203.569711	162.105263	3.217839	0.091982	0.96124	110
COVID-19-035	1161	0.97	241.82044	162	3.080292	0.092937	0.957795	106
COVID-19-007	1161	0.97	451.622772	288.956522	2.312381	0.366585	0.92851	141
COVID-19-016	1161	0.97	270.579709	199.103448	2.594603	0.299152	0.947459	136
COVID-19-003	1161	0.97	376.928122	254.111111	2.5696	0.279783	0.931955	140
COVID-19-018	1161	0.97	500.968322	283	2.710498	0.273871	0.925065	154
COVID-19-017	1161	0.97	218.41396	150.5	2.890446	0.123193	0.96124	101
COVID-19-032	1161	0.97	162.833334	128.090909	3.05133	0.100125	0.972438	83
COVID-19-030	1161	0.97	176.953146	144.111111	2.456564	0.235057	0.969854	78
COVID-19-029	1161	0.97	103.245946	102.25	2.419849	0.236817	0.976744	73
COVID-19-028	1161	0.97	235.052693	176.5	1.834912	0.36759	0.964686	74
COVID-19-027	1161	0.97	191.150007	177.357143	3.0825	0.138241	0.955211	130
COVID-19-026	1161	0.97	163.484216	118.230769	1.353496	0.520929	0.968131	67
COVID-19-031	1161	0.97	209.395477	202.545455	3.587085	0.055074	0.950904	130
COVID-19-024	1161	0.97	130.767842	145.583333	3.022738	0.116359	0.969854	96
COVID-19-019	1161	0.97	466.168334	235.894737	2.568414	0.221655	0.94143	116
COVID-19-025	1161	0.97	210.754853	220.333333	3.04407	0.111144	0.94832	122
COVID-19-020	1161	0.97	96.967943	70.666667	2.671143	0.122271	0.985357	48
COVID-19-033	1161	0.97	200.048146	222.352941	3.538085	0.068903	0.952627	135
COVID-19-022	1161	0.97	166.126034	168	2.991241	0.111667	0.971576	80
COVID-19-023	1161	0.97	324.443953	214.12	2.604299	0.210568	0.94143	123
COVID-19-021	1161	0.97	172.474607	154.4	3.199116	0.09717	0.966408	105
Control095	1161	0.97	206.637282	147.375	2.928213	0.0952	0.969854	73
Control084	1161	0.97	256.064431	92.333333	1.034312	0.497006	0.982773	29
Control085	1161	0.97	292.997627	237.896552	3.458815	0.087888	0.940568	157
Control086	1161	0.97	103.938776	95	2.012645	0.213327	0.978467	45
Control087	1161	0.97	126.962117	84.1	2.696581	0.145929	0.981051	61
Control088	1161	0.97	120.286762	67.5	1.389625	0.488166	0.983635	39
Control089	1161	0.97	73.537462	79.666667	1.687042	0.404479	0.982773	48
Control091	1161	0.97	152.307424	101.071429	2.443229	0.188255	0.97416	70
Control093	1161	0.97	137.847812	83	0.271306	0.922439	0.986219	23
Control094	1161	0.97	77.946138	72.909091	2.772583	0.115293	0.984496	59
Control096	1161	0.97	178.409985	162.12	3.374096	0.06289	0.958656	117
Control083	1161	0.97	250.497894	227.25	3.419134	0.098505	0.951766	131
Control090	1161	0.97	257.490748	177.045455	3.162196	0.079683	0.953488	112
Control092	1161	0.97	139.985502	98	2.471787	0.123441	0.979328	52
Control075	1161	0.97	71.701698	56.6	2.510962	0.142266	0.988803	41
Control081	1161	0.97	97.82309	86	2.205264	0.261154	0.981912	56
Control080	1161	0.97	89.510452	92.25	3.022942	0.078344	0.981912	66
Control079	1161	0.97	226.686252	168.666667	2.591343	0.189894	0.965547	82
Control078	1161	0.97	147.722023	96.625	1.951543	0.349036	0.977606	56
Control077	1161	0.97	141.235226	135.058824	3.158188	0.100824	0.968992	98
Control076	1161	0.97	36.774656	35	1.751404	0.322276	0.993109	28

Control074	1161	0.97	41.042707	38	2.182768	0.219415	0.993971	35
Control073	1161	0.97	55.89319	54	1.683156	0.311148	0.989664	32
Control072	1161	0.97	63.251698	71.25	2.427898	0.147477	0.98708	45
Control071	1161	0.97	182.490922	149.375	2.552403	0.169763	0.969854	75
Control070	1161	0.97	107.671029	79.666667	2.272615	0.191708	0.981051	54
Control069	1161	0.97	157.723771	152.272727	2.690339	0.103313	0.963824	74
Control097	1161	0.97	137.666851	96.2	2.580427	0.133773	0.981051	50
Control068	1161	0.97	96.087629	88.333333	1.758392	0.391339	0.985357	43
Control082	1161	0.97	178.079118	167.935484	2.922534	0.19167	0.957795	130
Control098	1161	0.97	211.253769	154.875	2.178837	0.292463	0.96124	93
Control116	1161	0.97	75.814498	77.5	2.318262	0.240947	0.984496	52
Control100	1161	0.97	113.667758	95.083333	3.16343	0.074337	0.980189	74
Control067	1161	0.97	147.801943	140.2	1.813566	0.381645	0.975022	59
Control130	1161	0.97	199.474053	165.75	2.285088	0.207982	0.97416	57
Control129	1161	0.97	97.55556	72.090909	2.180812	0.198816	0.981912	53
Control128	1161	0.97	114.951615	69.1	1.902713	0.231159	0.981051	46
Control127	1161	0.97	67.283243	65	0.385624	0.896084	0.981912	35
Control126	1161	0.97	165.315906	138.866667	2.48526	0.220617	0.96727	92
Control125	1161	0.97	80.126864	79.6	1.489677	0.480916	0.979328	52
Control124	1161	0.97	206.159832	140.6	2.435497	0.189716	0.968131	74
Control123	1161	0.97	87.682168	115.2	0.984722	0.626667	0.976744	45
Control122	1161	0.97	97.616243	72	2.562433	0.111701	0.982773	53
Control121	1161	0.97	67.387778	72.75	0.359458	0.899413	0.983635	30
Control120	1161	0.97	182.419281	113.75	2.586502	0.14757	0.975022	63
Control119	1161	0.97	138.559826	98	1.665967	0.427619	0.976744	59
Control118	1161	0.97	90.577774	87.428571	2.219891	0.220958	0.978467	66
Control099	1161	0.97	154.673251	140.12	3.219959	0.112961	0.96727	112
Control117	1161	0.97	70.174464	62.363636	2.165975	0.238344	0.985357	50
Control114	1161	0.97	610.137824	310.473684	2.025692	0.42142	0.927649	127
Control113	1161	0.97	156.795172	122.142857	1.88887	0.329628	0.97416	60
Control112	1161	0.97	89.015108	84	2.830503	0.086192	0.980189	61
Control111	1161	0.97	123.767447	94.2	2.387957	0.136805	0.981051	48
Control110	1161	0.97	196.456922	139.235294	2.881161	0.114993	0.964686	91
Control109	1161	0.97	332.296971	116.5	1.401353	0.468256	0.983635	31
Control108	1161	0.97	373.92205	272.433333	3.273436	0.091161	0.92851	159
Control107	1161	0.97	106.20442	97.5	2.49073	0.138103	0.982773	50
Control106	1161	0.97	152.323888	182	2.749048	0.129735	0.962102	96
Control105	1161	0.97	111.040825	86.25	3.036538	0.073136	0.981912	60
Control104	1161	0.97	682.365975	398.037037	2.834972	0.234671	0.905254	176
Control103	1161	0.97	103.127162	102.6	1.951251	0.26949	0.980189	52
Control102	1161	0.97	226.802311	132	1.431463	0.46194	0.96124	77
Control101	1161	0.97	102.438804	76	2.797279	0.103325	0.983635	57
Control115	1161	0.97	60.032739	54	1.583295	0.42197	0.98708	39
Control066	1161	0.97	198.236722	180	3.079379	0.126982	0.953488	127
Control020	1161	0.97	138.905418	91.428571	0.747475	0.7634	0.977606	45
Control064	1161	0.97	231.205168	114.8	2.178124	0.247257	0.971576	62
Control029	1161	0.97	106.184557	88	2.890661	0.076768	0.981912	53
Control028	1161	0.97	250.715284	204.035714	3.603339	0.063906	0.950043	145
Control027	1161	0.97	461.362636	379.24	4.056134	0.047697	0.916451	193
Control026	1161	0.97	132.573564	89.666667	2.88151	0.089767	0.979328	59
Control025	1161	0.97	133.132313	62.2	1.842743	0.2628	0.985357	35
Control024	1161	0.97	346.557825	136.8125	2.885238	0.123646	0.963824	83
Control023	1161	0.97	199.826896	119	1.541679	0.458064	0.972438	57
Control022	1161	0.97	171.700977	98	1.651188	0.419121	0.975883	56
Control021	1161	0.97	63.543488	62.142857	2.592412	0.154108	0.988803	51
Control131	1161	0.97	72.521783	71.5	2.394603	0.156644	0.984496	46
Control019	1161	0.97	114.043134	119	3.158632	0.075746	0.979328	73
Control018	1161	0.97	129.086128	122	2.870741	0.151686	0.971576	89

Control017	1161	0.97	229.172201	98	1.326716	0.397766	0.975883	44
Control016	1161	0.97	185.644686	223.333333	2.447286	0.131725	0.972438	58
Control015	1161	0.97	278.873177	228.666667	3.134304	0.147931	0.944014	142
Control014	1161	0.97	189.114359	144.2	2.815957	0.10165	0.975022	63
Control013	1161	0.97	268.306557	228.3125	3.314455	0.106653	0.950043	125
Control012	1161	0.97	302.056335	222.071429	2.179614	0.260891	0.950043	104
Control011	1161	0.97	196.243984	122.8	2.698382	0.11555	0.971576	70
Control010	1161	0.97	307.407096	183	3.35518	0.059498	0.96124	93
Control009	1161	0.97	200.045282	99.75	1.526234	0.376948	0.975022	49
Control008	1161	0.97	92.614325	67.25	1.099694	0.420936	0.984496	29
Control007	1161	0.97	204.293264	154	2.429498	0.233694	0.959518	107
Control006	1161	0.97	183.199277	132.461538	2.823661	0.139974	0.968992	84
Control005	1161	0.97	154.664984	133.111111	2.727948	0.138568	0.972438	78
Control004	1161	0.97	245.659438	167.2	0.911305	0.729502	0.970715	55
Control003	1161	0.97	200.515224	164.666667	1.836512	0.428431	0.965547	78
Control002	1161	0.97	378.782573	227	2.191876	0.353077	0.939707	122
Control001	1161	0.97	177.997272	94.428571	1.767396	0.30397	0.972438	59
Control030	1161	0.97	156.4265	221.5	2.599654	0.128893	0.963824	78
Control031	1161	0.97	153.015447	149.333333	3.335041	0.076395	0.965547	106
Control032	1161	0.97	141.290336	118.909091	3.311081	0.058579	0.975022	82
Control033	1161	0.97	236.142838	209.315789	3.567941	0.069534	0.953488	134
Control063	1161	0.97	174.171798	148.583333	3.164894	0.073661	0.96727	90
Control062	1161	0.97	195.748629	151.117647	3.027117	0.107805	0.962963	98
Control061	1161	0.97	225.268676	234.125	2.829556	0.137077	0.959518	99
Control060	1161	0.97	165.55496	139.428571	2.411592	0.149719	0.971576	64
Control059	1161	0.97	165.531759	136	3.267342	0.081162	0.971576	88
Control058	1161	0.97	82.868001	72.857143	2.11927	0.252051	0.984496	51
Control057	1161	0.97	175.470426	98.625	2.640645	0.133016	0.977606	58
Control056	1161	0.97	285.706879	195.333333	3.662793	0.03446	0.962963	95
Control055	1161	0.97	149.302086	151.4	2.902043	0.108971	0.963824	94
Control054	1161	0.97	130.592023	101.857143	2.322021	0.196342	0.978467	59
Control053	1161	0.97	195.346513	159	1.86382	0.447339	0.965547	81
Control052	1161	0.97	168.87007	102.333333	2.074063	0.223672	0.972438	61
Control051	1161	0.97	177.038291	113.5	1.80514	0.420458	0.969854	71
Control050	1161	0.97	200.368186	199.055556	1.723382	0.49828	0.949182	104
Control065	1161	0.97	241.825829	167.625	2.551312	0.145116	0.966408	75
Control049	1161	0.97	272.137619	219.142857	3.212013	0.108676	0.947459	132
Control047	1161	0.97	210.930062	141.75	2.774164	0.131168	0.966408	80
Control046	1161	0.97	115.734121	109.25	3.014629	0.078958	0.97416	73
Control045	1161	0.97	351.799689	254.066667	3.489481	0.075347	0.946598	128
Control044	1161	0.97	168.277257	168.473684	3.268661	0.075702	0.960379	114
Control043	1161	0.97	170.892434	89.909091	0.94206	0.704604	0.975022	53
Control042	1161	0.97	225.877276	183.142857	3.184155	0.093588	0.955211	120
Control041	1161	0.97	204.134584	137.333333	2.846813	0.114629	0.970715	75
Control040	1161	0.97	117.461298	112.615385	1.851581	0.369993	0.971576	72
Control039	1161	0.97	205.686318	153	2.045832	0.41296	0.965547	88
Control038	1161	0.97	142.686778	105.1	2.829536	0.124422	0.976744	70
Control037	1161	0.97	91.568196	54.3	1.064402	0.619386	0.984496	39
Control036	1161	0.97	99.407298	69.111111	2.458787	0.121642	0.982773	48
Control035	1161	0.97	291.621806	222	2.609647	0.310248	0.944014	142
Control034	1161	0.97	109.55903	97.166667	2.380188	0.160608	0.980189	55
Control048	1161	0.97	129.307083	93	2.184379	0.27445	0.976744	66
Control132	1161	0.97	176.845816	137	1.084853	0.664035	0.968131	63

Supplementary table S18. The abundance and composition at the phylum level of each sample among COVID-19 (n=71), Post-COVID-19 (n=22), and control (n=132) groups.														
phylum	Post-COVID-19-001	Post-COVID-19-022	Post-COVID-19-021	Post-COVID-19-020	Post-COVID-19-018	Post-COVID-19-017	Post-COVID-19-016	Post-COVID-19-015	Post-COVID-19-014	Post-COVID-19-013	Post-COVID-19-012	Post-COVID-19-019	Post-COVID-19-010	Post-COVID-19-011
Ascomycota	715318	521575	988399	814754	201053	930920	148552	522412	403146	107171	461177	188542	341092	107243
Basidiomycota	34682	416179	1393	106494	283637	2626	37647	39443	6342	32619	321284	286590	335643	472944
Chytridiomycota	0	0	0	0	0	0	0	0	0	0	0	0	0	0
Fungi_phy_Incertae_sedis	20799	3628	9258	424	1963	31835	3005	9508	2378	1500	9373	891	2049	1749
Mucoromycota	228667	58618	950	78328	513347	34465	810450	428469	588134	858710	208166	523514	321216	417814
Rozellomycota	0	0	0	0	0	0	39	0	0	0	0	0	0	250
Zoopagomycota	534	0	0	0	0	154	307	168	0	0	0	463	0	0

Supplementary table S18. The abundance and composition at the genus level of each sample among COVID-19 (n=71), Post-COVID-19 (n=22), and control (n=132) groups.														
genus	Post-COVID-19-001	Post-COVID-19-022	Post-COVID-19-021	Post-COVID-19-020	Post-COVID-19-018	Post-COVID-19-017	Post-COVID-19-016	Post-COVID-19-015	Post-COVID-19-014	Post-COVID-19-013	Post-COVID-19-012	Post-COVID-19-019	Post-COVID-19-010	Post-COVID-19-011
Acremonium	642	0	0	0	0	0	0	0	0	0	0	0	0	0
Acrocalymma	0	0	0	0	0	0	0	0	0	0	0	0	0	0
Acrodictys	7018	133812	1190	3786	1959	192	6535	5407	396	4759	45825	19214	1143	5482
Acrodontium	0	0	0	0	0	0	0	0	0	0	0	0	0	0
Actinomucor	185170	32284	445	78292	504340	33704	747726	421560	580785	853582	182828	523021	309198	414755
Adisciso	0	0	0	0	0	0	0	0	0	0	0	0	0	0
Agaricales_gen_Incertae_sedis	0	0	0	0	0	0	0	0	0	0	0	0	0	0
Agaricus	0	0	0	0	0	0	0	0	0	0	0	0	0	0
Ahmadea	0	0	0	0	0	0	0	0	0	0	0	0	0	0
Akanthomyces	0	0	0	0	0	0	0	0	0	0	0	0	0	0
Allocryptovalsa	0	0	0	0	0	0	0	0	0	0	0	0	0	0
Alternaria	3495	210	743	75431	0	238	2740	9162	0	0	112	42	63003	0
Amphobotrys	0	0	0	0	0	0	0	0	0	0	0	0	0	0
Amylostereum	0	0	0	0	0	0	0	0	0	0	0	0	0	0
Anastomitrabeculia	0	0	0	0	0	0	0	0	0	0	0	0	0	0
Angustimassarina	1400	0	0	0	0	345	222	488	32	0	0	2728	0	0
Anisomeridium	0	0	0	0	0	0	0	0	0	0	0	0	0	0
Antennariella	0	0	0	0	0	0	0	0	0	0	0	0	0	0
Anthracina	0	0	0	0	0	0	0	0	0	0	0	0	0	0
Apiotrichum	0	0	0	0	0	0	0	0	0	0	0	0	0	0
Aquapteridospora	0	0	0	0	0	0	0	0	0	0	0	0	0	0
Arachnomyces	0	0	0	0	0	0	0	0	0	0	0	0	0	0
Archaeorhizomyces	0	0	0	0	0	0	0	0	0	0	0	0	0	0
Arthrinium	0	0	0	0	0	0	0	0	0	0	0	0	0	0
Arthrographis	0	0	0	0	0	0	0	0	0	153	0	471	0	0
Ascochyta	289	0	0	0	0	0	0	0	0	0	0	0	0	143
Ascomycota_gen_Incertae_sedis	0	0	0	0	0	0	0	0	0	0	0	0	0	0
Ascosphaera	0	0	0	0	0	0	0	0	0	0	0	0	0	0
Ascotricha	0	0	0	0	0	0	0	0	0	0	0	0	0	0
Aspergillus	2542	41234	495	5966	54080	223	2482	6902	7001	7110	87437	30156	39639	6131
Atractiella	0	0	0	0	0	0	0	0	0	0	0	0	0	0
Aureobasidium	0	84005	0	0	0	0	0	0	0	0	0	0	0	0
Auricularia	0	0	0	0	0	0	0	0	0	0	0	0	0	0
Austropleospora	0	0	0	0	0	0	0	0	0	0	0	0	0	0
Avachytrium	0	0	0	0	0	0	0	0	0	0	0	0	0	0
Bandonia	0	606	0	0	0	0	0	0	88	0	0	0	0	0
Banksiophoma	0	0	0	0	0	0	0	0	0	0	0	0	0	0
Bannoa	727	0	0	0	0	0	0	0	0	0	0	0	0	0
Basidiodendron	0	0	0	0	0	0	0	0	0	0	0	0	0	0
Basidiomycota_gen_Incertae_sedis	0	0	0	0	0	0	0	0	0	0	0	0	0	0
Beauveria	0	615	0	0	0	0	0	0	0	0	0	205	0	0
Bergerella	0	0	0	0	0	0	0	0	0	0	0	0	0	0
Berkeleyomyces	0	0	0	0	0	0	0	0	0	0	0	0	0	0
Betamyces	0	0	0	0	0	0	0	0	0	0	0	0	0	0
Biatriosporaceae_gen_Incertae_sedis	0	0	0	0	0	0	0	0	0	0	0	0	0	0
Bjerkandera	0	0	0	0	0	0	0	0	0	0	0	8173	0	0
Blastobotrys	0	0	0	0	0	0	0	0	0	0	0	0	0	0
Blumeria	0	0	0	39	0	0	0	0	35	0	0	0	0	0
Botryosphaeria	14217	11476	243	1659	0	49	52	118097	8878	4811	28880	25214	0	0
Botrytis	0	0	0	0	0	0	0	3072	0	0	0	0	0	0
Bovista	0	0	0	0	0	0	0	0	0	0	0	0	0	0
Bradymyces	0	0	0	0	0	0	0	0	0	0	42	0	0	9488
Buckleyzyma	0	0	0	0	0	0	0	0	0	0	0	0	0	0
Bulleromyces	0	0	0	0	0	0	0	0	0	0	0	0	0	0
Byssochlamys	0	0	0	0	0	0	0	0	0	0	0	0	0	0
Cadophora	981	0	0	0	0	0	0	0	0	0	0	0	0	0
Caloplaca	209	0	0	0	0	0	0	0	0	0	0	1322	0	0
Calosphaeria	0	0	0	0	0	0	0	0	0	0	0	0	0	0
Calycina	0	0	0	0	0	0	0	0	0	0	0	0	0	0
Calyptella	0	0	0	0	0	0	0	0	0	0	0	0	0	0
Candida	20268	3340	4446	8158	12740	150	88	7136	312377	7096	58	72	110	297
Capnodiales_gen_Incertae_sedis	0	0	0	0	0	0	0	0	0	0	0	0	0	0
Carlosrosaea	0	0	0	0	0	0	0	0	0	0	0	0	0	0
Ceraceosorus	0	0	0	0	0	0	0	0	0	0	0	0	0	0
Ceratocystis	0	0	0	0	0	0	0	0	0	0	0	0	0	0
Cercospora	2008	0	0	0	0	42	0	0	0	0	0	0	0	0
Ceriporia	0	0	0	0	0	0	0	0	0	0	0	0	0	0
Ceriporiopsis	0	0	0	0	0	0	0	0	0	0	0	0	0	0
Cerrena	0	0	0	0	0	0	0	0	0	0	0	0	0	0

Chaenothecopsis	0	0	0	0	0	0	0	0	0	0	0	0	0	0
Chaetomium	0	0	0	0	0	0	0	0	0	0	0	0	0	0
Chaetosphaeria	0	0	0	0	0	0	0	0	0	0	0	0	0	0
Chalara	0	0	0	0	0	0	0	0	0	0	0	0	0	0
Chloridium	0	0	0	0	38	0	0	0	0	0	0	0	0	0
Choanephora	0	0	0	0	0	0	0	0	0	0	0	0	0	0
Chrysosphaeria	0	0	0	0	0	0	0	0	0	0	0	0	0	0
Chytridiomycota_gen_Incertae_sedis	0	0	0	0	0	0	0	0	0	0	0	0	0	0
Chytridium	0	0	0	0	0	0	0	0	0	0	0	0	0	0
Ciboria	0	0	0	0	0	0	0	0	0	0	0	0	0	0
Ciliophora	0	214	0	44	0	0	0	0	0	0	0	0	0	0
Cladophialophora	0	0	0	0	0	0	0	0	0	0	0	0	0	0
Cladosporium	28753	40601	1162	11202	12100	389	3434	29254	1055	6673	114159	33955	92	44
Clavispora	764	0	0	0	0	346	0	0	0	0	0	0	0	0
Clonostachys	0	0	0	0	0	0	0	3386	0	0	0	0	0	0
Colletotrichum	0	0	0	0	0	0	0	0	0	0	0	0	0	0
Collybiopsis	0	0	0	0	0	0	0	0	0	0	0	0	0	0
Coniella	0	0	0	0	0	0	0	0	0	0	0	0	0	0
Coniochaeta	0	0	0	0	0	0	0	0	0	0	0	0	0	0
Coniosporium	0	0	0	0	0	0	0	0	0	0	0	0	0	0
Coniothyrium	0	0	0	0	0	0	0	0	0	0	0	0	0	0
Cookeina	672	12456	0	4244	0	0	39	0	0	0	0	0	0	0
Coprinellus	0	0	0	0	0	0	0	0	0	0	0	0	0	0
Coprinopsis	0	0	0	0	0	0	0	0	0	0	0	3253	0	0
Cordyceps	0	193	0	0	0	0	0	1998	0	0	0	0	104	148
Coronicium	758	0	0	0	0	0	0	0	0	0	0	0	0	0
Crustoderma	0	0	0	0	0	0	0	0	0	0	0	0	0	0
Cryptococcus	0	0	0	0	0	0	0	0	0	0	0	0	0	0
Cryptomarasmius	0	0	0	4590	0	0	0	0	0	0	0	0	0	0
Curvularia	0	0	0	0	0	0	0	0	0	0	0	0	0	0
Cutaneotrichosporon	0	0	0	0	0	0	0	0	0	0	0	0	0	0
Cyphellaceae_gen_Incertae_sedis	0	0	0	0	0	0	0	0	0	0	0	0	0	0
Cyphellophora	0	0	0	0	0	0	0	0	0	0	0	0	0	0
Cystobasidium	0	9715	0	0	0	0	0	0	625	4319	0	0	0	0
Cystofilobasidium	0	0	0	0	0	0	0	0	0	0	0	0	0	0
Cytospora	0	0	0	0	0	0	0	0	0	0	0	0	0	0
Dactylella	0	0	0	0	0	0	0	0	0	0	0	0	0	0
Dactylodendron	0	0	0	0	0	0	54	26362	0	0	0	0	0	0
Debaryomyces	859	379	0	672623	0	0	0	0	0	0	0	5290	0	31
Deconica	0	0	0	0	0	0	0	0	0	0	0	0	0	0
Delastria	0	0	0	0	0	0	0	0	0	0	0	0	0	0
Dendryphiella	0	0	0	0	0	0	0	0	0	0	0	0	0	0
Derxomyces	0	0	0	0	0	0	0	0	0	0	0	0	0	0
Diaporthe	942	0	0	0	0	0	0	0	0	1213	0	0	0	0
Didymella	0	0	0	0	0	0	0	0	0	0	0	6558	0	0
Didymocyrtis	0	0	0	0	0	0	0	0	0	0	0	0	0	0
Dimorphoma	0	27250	0	0	0	0	0	0	0	0	0	0	0	0
Dioszegia	0	0	0	0	0	42	0	0	0	0	0	15544	0	0
Dipodascus	112	1688	237	0	120	0	88	0	9728	0	0	0	0	2937
Diutina	0	0	0	0	0	0	0	2013	0	0	0	0	0	0
Diversispora	323	0	505	0	0	134	1181	0	5037	0	0	0	0	0
Dothidea	0	0	0	0	0	0	0	0	0	0	0	0	0	0
Dothiora	0	0	0	0	0	0	0	0	0	0	0	0	0	0
Echinoporia	0	0	0	0	0	0	0	0	0	0	0	0	0	0
Edenia	0	0	0	0	0	0	0	0	0	0	0	0	0	0
Effuseotrichosporon	0	0	0	0	0	0	0	0	0	0	0	0	0	0
Elaphomyces	0	0	0	0	0	0	0	0	0	0	0	0	0	0
Elsinoe	0	0	0	0	0	0	0	0	0	0	0	0	0	0
Emergomyces	0	0	0	0	0	0	0	0	0	0	0	0	0	0
Emericellopsis	0	0	0	0	0	0	0	0	815	0	0	0	0	0
Engyodontium	0	6184	0	0	0	0	1702	0	704	0	0	0	0	0
Entoloma	0	0	0	0	0	0	0	0	0	0	84	0	0	0
Entorrhiza	1401	0	0	0	0	84	406	0	0	0	0	0	0	0
Entyloma	0	0	0	0	0	0	0	0	0	0	0	0	0	0
Epicoccum	95	0	0	0	0	0	0	0	0	0	0	0	0	37
Eremothecium	0	0	0	0	0	0	0	0	0	3034	0	0	0	0
Erysiphe	0	0	0	0	0	0	0	0	0	769	0	0	0	0
Erythrobasidium	0	0	0	0	0	0	0	0	0	0	0	0	0	0
Eupenidiella	227	0	0	0	0	0	0	0	0	0	0	0	0	0
Eutypella	0	0	0	0	0	0	38	9057	51	0	0	0	0	0
Exidia	0	0	0	0	0	0	39	0	0	0	0	0	35773	0
Exobasidium	0	0	0	0	0	0	0	0	0	0	0	0	0	0
Exophiala	0	0	0	0	0	0	0	0	0	0	0	0	0	0
Extremus	0	0	0	0	0	0	0	0	0	0	0	0	0	0
Fantasmomyces	0	437	0	0	36	0	0	0	0	0	0	218	0	0
Filobasidiaceae_gen_Incertae_sedis	0	0	0	784	319	0	133	205	104	0	0	105	0	0
Filobasidium	0	0	0	21225	44521	45	40	0	46	0	0	6396	0	0
Flagelloscypha	0	0	0	0	0	0	0	0	0	0	0	0	0	0
Flammocladiella	0	0	0	0	601	0	0	0	0	0	0	0	0	0
Flammulina	0	0	0	0	0	0	0	0	0	0	0	0	0	0
Flavodon	0	0	0	0	0	0	0	0	0	0	0	0	0	0
Fomitopsis	0	0	0	0	0	0	0	0	0	0	0	0	0	0
Fracchiaea	0	0	0	0	0	0	0	0	0	0	0	0	0	0
Fungi_gen_Incertae_sedis	19398	3628	9258	424	1963	31751	2599	9508	2378	1500	9373	891	2049	1749
Fusarium	3208	0	0	5063	0	0	14348	0	49	63	0	8121	0	3463
Fuscosporella	0	0	0	0	0	0	0	0	0	0	0	0	0	0
Fusicolla	0	0	0	0	0	0	0	0	0	0	0	0	0	0

GS08_gen_Incertae_sedis	0	0	0	0	0	0	0	0	0	0	0	0	0	0
Galactomyces	13743	7635	0	0	67851	298	12763	528	1145	3436	0	0	1853	263
Ganoderma	0	0	0	0	0	0	0	0	0	0	0	0	0	0
Genea	0	0	0	0	0	0	0	0	34	0	0	0	0	0
Genolevuria	0	0	0	0	0	0	0	0	0	0	0	0	0	0
Geosmithia	0	0	0	0	0	0	0	0	0	0	0	0	0	0
Geotrichum	0	0	0	0	0	1170	0	0	0	0	0	0	0	0
Gibberella	0	0	0	4723	0	0	0	3665	0	0	0	0	0	12196
Gibellula	0	0	0	0	0	0	0	0	0	0	0	0	0	0
Gibellulopsis	0	0	0	0	0	0	0	0	0	0	0	0	0	0
Gjaerumia	0	0	0	0	0	0	0	0	0	0	0	0	0	0
Glomus	0	0	0	0	0	0	0	0	0	0	0	0	0	0
Golovinomyces	0	0	0	0	0	0	0	0	0	0	0	0	0	0
Golubevia	0	0	0	0	2133	0	0	0	0	0	0	0	0	0
Gonatophragmium	0	0	0	0	0	0	0	0	0	0	0	0	0	0
Graphium	0	0	0	0	0	0	0	0	0	0	0	0	0	0
Gregarithecium	0	0	0	0	0	0	0	0	0	0	0	0	0	0
Groenewaldozyma	0	0	0	0	0	0	0	0	0	0	0	0	0	0
Gymnopus	0	0	0	0	0	0	0	0	0	0	0	0	0	0
Halosphaeriaceae_gen_Incertae_sedis	0	0	0	0	0	0	0	0	0	0	0	0	0	0
Hannaella	0	0	0	0	0	0	0	0	0	0	0	0	0	0
Hanseniaspora	0	0	0	0	0	44	0	0	0	0	0	0	0	0
Hazslinszkyomyces	0	0	0	0	0	0	0	0	0	0	0	0	0	0
Heitmania	0	0	0	0	0	0	0	0	0	0	0	0	0	0
Helicoma	0	0	0	0	0	0	0	0	0	0	0	0	0	0
Heliocybe	0	0	0	0	0	0	0	0	0	0	0	0	0	0
Henningsia	0	0	0	0	0	0	0	0	0	0	0	0	0	0
Heydenia	0	5036	0	0	0	0	0	0	0	0	0	0	0	0
Hohenbuehelia	0	0	0	0	0	0	0	5180	0	0	0	0	0	0
Holtermanniella	0	0	0	0	0	0	0	0	0	0	0	0	0	0
Homortomyces	0	0	0	0	0	0	0	0	0	0	0	0	0	0
Hormodochis	0	0	0	0	0	0	0	0	0	0	0	0	0	0
Hortaea	0	0	0	0	0	0	0	0	0	0	0	0	0	0
Houjia	0	0	0	0	0	0	0	0	0	0	0	0	0	0
Huntiella	0	0	0	0	0	0	0	0	0	0	0	0	0	0
Hydnobolites	0	210	0	91	0	0	0	0	0	0	0	97	52	0
Hydropisphaera	0	0	0	0	0	0	0	0	0	0	0	0	0	0
Hyphopichia	0	0	0	0	0	0	0	0	0	0	0	0	0	0
Hypoxylon	0	0	0	0	0	0	0	0	0	0	0	0	0	0
Hysterobrevium	2752	0	0	0	0	0	0	0	0	0	0	0	0	0
Inocyclus	0	0	0	0	0	0	0	0	0	0	0	0	0	0
Iodophanus	0	0	0	0	0	0	0	0	0	0	0	0	0	0
Iodosphaeria	0	0	0	0	0	0	0	0	0	0	0	0	0	0
Irpex	0	0	0	0	25513	0	0	0	0	0	0	0	0	0
Issatchenkia	0	0	0	0	0	0	0	0	0	0	0	0	0	0
Itersonilia	0	0	0	0	0	47	0	3583	346	0	0	9835	0	0
Jaminaea	0	0	0	0	0	0	0	0	0	0	0	0	0	0
Jattaea	0	0	0	0	0	0	0	0	0	0	0	0	0	0
Kazachstania	1579	21227	0	0	36	55	0	8972	0	0	0	0	25103	5166
Keissleriella	0	0	0	0	0	0	0	0	0	0	0	0	0	0
Keratinophyton	0	0	0	0	0	0	0	0	0	0	0	0	0	0
Kluyveromyces	0	0	0	0	0	0	0	0	0	0	0	0	0	0
Kneiffiella	0	0	0	0	0	0	0	0	0	0	0	0	0	0
Knufia	0	0	0	45	0	0	1106	0	0	84	0	0	0	0
Kodamaea	0	0	0	0	0	0	0	0	0	0	0	0	0	0
Komagataella	0	0	0	0	0	0	0	0	0	0	0	0	0	0
Kondoa	1961	0	0	0	0	0	0	0	0	0	0	0	0	0
Kurtzmaniella	0	0	0	0	0	0	0	0	0	0	0	0	0	0
Laburnicola	0	0	0	0	0	0	0	0	0	0	0	0	0	0
Laetisaria	0	0	0	0	0	0	40	0	0	0	0	0	0	0
Lecanicillium	0	0	0	0	0	0	0	0	0	0	0	0	0	0
Lembosiniella	0	0	0	0	0	0	0	0	0	0	0	0	0	0
Lentinula	0	0	0	0	0	0	0	0	0	0	0	0	0	0
Lentinus	0	0	0	0	0	0	0	0	0	0	0	0	0	0
Lentithecium	0	0	0	0	0	0	0	0	0	0	0	0	0	0
Leptospora	0	0	0	0	0	0	0	0	0	0	0	0	0	4792
Leptosporella	0	0	0	0	33	38	0	85	0	0	44	0	101	0
Leucosporidium	0	0	0	0	0	0	0	0	0	0	0	0	0	0
Lipomyces	0	0	0	0	0	0	0	0	0	0	0	0	0	0
Lithohypha	0	0	0	0	0	0	0	37	865	0	0	0	0	0
Lophiostoma	0	0	0	0	0	0	0	0	0	0	0	0	0	0
Lyomyces	0	0	0	0	0	0	0	0	0	0	0	0	0	0
Macrohyporia	0	0	0	0	0	0	0	0	0	0	0	0	0	0
Malassezia	6176	357292	1136	4072	81743	712	16129	15183	1574	2062	72061	150721	104087	7790
Marantokordyana	0	0	0	0	0	0	0	0	0	0	0	0	0	0
Meira	0	0	0	0	0	0	0	0	0	0	0	0	0	0
Meyerozyma	0	0	0	0	0	0	0	0	0	0	0	0	0	0
Microascus	0	0	0	0	0	0	0	0	0	0	0	0	0	0
Milospium	0	4247	0	0	0	79	0	102	326	0	0	0	0	0
Moesziomyces	310	0	0	0	6350	0	0	0	0	0	0	0	0	0
Monoblepharis	0	0	0	0	0	0	0	0	0	0	0	0	0	0
Montagnula	0	0	233	5078	0	0	0	0	0	0	0	0	0	0
Mrakia	0	0	0	0	0	0	0	0	0	0	0	0	0	0
Mucor	43174	26334	0	36	8922	539	61543	6909	2312	5128	25338	493	12018	3059
Mycoarthris	0	0	0	0	0	0	0	0	0	0	0	0	0	0
lycosphaerellaceae_gen_Incertae_sed	0	0	0	0	0	0	0	0	0	0	0	0	0	0
Naganishia	3122	0	0	0	0	0	0	0	0	0	0	0	0	0

Nakaseomyces	0	0	0	0	0	0	0	0	0	0	0	0	0	0
Nectriopsis	0	0	0	0	0	0	0	0	0	0	0	0	0	0
Nemania	0	0	0	0	0	0	0	0	0	0	0	0	0	0
Neoacladium	0	0	0	0	0	0	0	0	0	0	0	0	0	0
Neoascochyta	0	0	0	0	0	0	0	0	0	0	0	0	0	0
Neocelosporium	0	28331	0	0	0	0	0	0	0	0	0	0	0	0
Neoconiothyrium	0	0	0	0	0	0	0	0	0	0	0	0	0	0
Neocucurbitaria	0	0	0	0	0	0	0	0	0	0	0	0	0	0
Neodevriesia	0	0	0	0	0	0	0	0	0	0	0	0	0	0
Neodevriesiaceae_gen_Incertae_sedis	0	0	0	0	0	0	0	0	0	0	0	0	0	0
Neoerysiphe	0	0	0	0	0	0	0	0	0	0	0	0	0	0
Neofusicoccum	0	0	0	0	0	0	0	0	0	0	0	0	0	0
Neomicrosphaeropsis	12494	0	0	0	0	0	46	0	0	0	0	0	6708	2715
Neosetophoma	0	0	0	0	0	0	0	0	0	0	0	0	0	0
Nigrograna	0	0	0	0	0	0	1503	0	0	0	0	0	0	0
Nigrospora	837	0	0	0	0	0	461	0	2083	2748	0	0	46	1942
Novakomyces	17028	12460	0	0	0	0	0	0	0	63	0	0	0	0
Ochrocladosporium	0	0	0	0	0	0	0	0	0	0	0	0	0	0
Ochroconis	0	0	0	0	0	0	0	0	0	0	0	0	0	0
Ogataea	0	0	0	0	0	0	0	0	0	0	0	0	0	0
Oliveonia	0	0	0	0	0	0	0	0	0	0	0	0	0	0
Ophiosphaerella	0	0	0	0	0	0	0	0	0	0	0	0	0	0
Ophiostoma	0	0	0	0	0	0	0	161	0	0	0	0	0	0
Paecilomyces	0	0	0	0	0	0	0	0	0	0	0	0	0	0
Paludomyces	0	0	0	0	0	0	0	0	0	0	0	0	0	0
Papiliotrema	0	0	0	0	0	0	0	0	0	0	0	0	0	0
Paracremonium	0	0	0	0	0	0	0	0	0	0	0	0	0	0
Parafuscosporella	438	425	663	0	0	879	82	93	0	0	108	128	0	0
Paraleptosphaeria	0	0	0	0	0	0	0	0	0	0	0	0	0	0
Paraloratospora	0	0	0	0	0	0	0	0	2876	70	0	0	0	0
Paraphoma	0	0	0	0	0	0	0	0	0	0	0	0	0	0
Pararamichloridium	0	0	0	0	0	0	0	0	0	0	0	0	0	0
Parasarocladium	0	0	0	0	0	0	0	0	0	0	0	0	0	0
Parastagonosporella	0	0	0	0	0	0	0	0	0	0	0	0	0	0
Parasympodiella	0	0	0	0	0	0	0	0	0	0	0	0	0	0
Parathyridaria	0	0	0	0	0	0	0	0	0	0	0	0	0	0
Paratritirachium	0	0	0	0	0	0	0	0	0	0	0	0	0	0
Penicillifer	0	0	0	0	0	0	0	0	0	0	0	0	0	0
Penicillium	325	204	0	52	0	0	658	0	435	0	16071	54	8210	0
Penidiella	0	0	0	0	0	0	0	0	0	0	0	0	0	0
Peniophora	0	0	0	0	0	0	0	1970	0	78	46546	4166	41	0
Peniophorella	0	0	0	0	0	0	0	0	0	0	0	0	0	0
Periconia	0	0	0	0	0	0	0	0	0	66	0	0	0	8936
Peroneutypa	0	0	0	0	0	0	0	0	0	0	0	0	0	0
Pestalotiopsis	0	0	0	0	0	0	0	0	0	0	0	0	0	0
Peyronellaea	0	0	0	0	0	0	0	0	0	0	0	0	0	0
Peziza	0	0	0	0	0	0	0	0	0	0	0	0	0	0
Phacidium	0	0	0	0	0	0	0	0	0	0	0	0	0	0
Phaeoacremonium	0	0	0	0	0	0	0	0	0	0	0	0	0	0
Phaeophleospora	0	0	0	0	0	0	0	0	0	0	0	0	0	0
Phaeosphaeria	0	0	0	0	0	0	1586	0	0	0	0	0	0	0
Phanerochaete	0	0	0	0	0	0	0	0	0	0	0	0	0	0
Phialemoniopsis	0	0	0	0	0	0	0	0	0	4789	0	0	0	0
Phialocephala	0	0	0	0	0	0	0	0	0	0	0	0	0	0
Phlebia	0	0	0	0	0	0	665	0	0	0	0	0	0	0
Phlebiopsis	0	0	0	0	0	0	0	0	0	0	0	0	0	0
Phomatospora	0	0	0	0	2899	0	0	0	0	0	0	0	0	0
Phyllachora	11341	0	688	0	0	2581	9703	5416	0	72	0	4352	0	0
Phyllosticta	0	0	0	0	0	0	0	0	0	0	0	0	0	0
Physisporinus	0	0	0	0	0	0	0	0	0	0	0	0	3519	0
Pichia	0	0	0	0	0	85	0	0	352	0	0	0	0	0
Plectosphaerella	0	0	0	0	0	0	0	0	0	0	0	0	0	0
Plenodomus	0	0	0	0	0	0	0	0	0	0	0	0	0	0
Pleurotus	0	0	0	0	0	0	0	0	0	1482	0	6417	0	0
Podosphaera	0	0	0	0	0	0	0	0	0	0	0	0	0	0
Powellomyces	0	0	0	0	0	0	0	0	0	0	0	0	0	0
Preussia	0	0	0	0	0	0	0	0	0	0	0	0	0	0
Protomyces	0	0	0	0	0	0	0	0	0	0	0	0	0	0
Psathyrella	0	0	0	0	0	0	0	0	0	0	0	0	0	0
Pseudocercospora	0	0	0	0	0	0	0	0	0	0	0	0	0	0
Pseudogymnoascus	0	0	0	0	0	0	0	0	0	0	0	0	0	0
Pseudohelminthosporium	0	0	0	0	0	0	0	0	0	0	0	0	0	0
Pseudomicrostroma	0	0	0	0	0	0	0	0	0	0	0	0	0	0
Pseudopithomyces	0	0	0	0	0	0	0	0	0	0	0	0	0	0
Pseudoramichloridium	0	0	0	0	0	0	0	0	0	0	0	0	0	0
Pseudorobillarda	0	0	0	0	0	0	0	0	0	0	0	0	0	0
Pseudoveronaea	0	0	0	0	0	0	0	0	0	0	0	0	0	0
Puccinia	0	0	0	0	0	0	0	0	0	0	0	0	0	0
Punctularia	0	0	0	0	0	0	0	0	0	0	0	0	0	0
Purpureocillium	0	0	0	0	0	0	0	0	0	0	0	0	0	0
Pyrenochaeta	0	0	0	0	0	0	0	0	0	0	0	0	0	0
Pyrenochaetopsis	0	0	0	0	0	0	0	0	0	0	0	0	0	0
Pyricularia	0	0	0	0	0	0	0	0	0	0	0	0	0	0
Quambalaria	0	0	0	0	0	0	0	0	0	0	0	0	0	0
Rachicladosporium	0	0	0	0	0	0	0	0	0	0	0	0	0	0
Radulomyces	0	0	0	0	0	0	0	0	0	0	0	0	0	0
Raffaelea	0	396	0	0	40	45	88	0	0	61	180	38	159873	55

Ramophialophora	0	0	0	0	0	0	0	0	0	0	0	0	0	0
Ramularia	0	0	0	0	0	0	0	0	0	0	0	0	4516	0
Recurvomyces	0	0	0	0	0	0	0	0	0	0	0	0	0	0
Resupinatus	112	0	0	0	0	0	0	0	0	0	0	0	0	0
Rhexothecium	0	0	0	0	0	0	0	0	0	0	0	0	0	0
Rhizoctonia	2378	0	0	0	0	0	0	0	0	0	0	0	0	0
Rhizopogon	0	0	0	0	0	0	0	0	0	0	0	0	0	0
Rhizopus	0	0	0	0	85	88	0	0	0	0	0	0	0	0
Rhodosporidiobolus	0	0	0	0	0	0	0	0	0	0	0	0	0	0
Rhodotorula	18620	48566	0	65957	93549	813	2668	126	3431	20399	202113	5951	28764	81
Rhopalophora	0	0	0	0	0	0	0	0	0	0	0	0	0	0
Robbauera	0	0	0	0	0	0	0	0	0	0	0	0	0	0
Rosellinia	0	10030	0	0	0	0	0	0	0	0	0	0	0	35
Roussoella	0	0	0	0	0	0	0	0	0	0	0	0	0	0
Rozellomycota_gen_Incertae_sedis	0	0	0	0	0	0	39	0	0	0	0	0	0	250
Ruhlandiella	807	0	0	0	0	196	47	0	0	0	0	85	0	275
Russula	0	0	257	0	0	0	0	0	0	0	0	0	0	0
Russulales_gen_Incertae_sedis	0	0	0	0	0	0	0	0	0	0	0	0	0	0
Saccharomyces	0	0	0	0	14414	0	47	0	757	0	96	0	61	217
Saccharomycopsis	0	0	0	0	0	0	0	0	0	0	0	0	0	0
Saprochaete	0	0	0	0	0	0	0	0	0	0	0	0	0	0
Sarocladium	106	1922	2384	683	0	0	0	0	39	0	0	0	0	0
Schizophyllum	0	0	0	0	0	0	0	7592	30	1867	0	0	0	0
Schizosaccharomyces	0	0	0	0	0	0	0	0	0	0	0	0	0	0
Scopulariopsis	0	0	0	0	0	0	0	0	0	0	0	0	0	0
Scopuloides	0	0	0	0	0	0	0	0	0	0	0	0	0	0
Septobasidium	0	0	0	0	0	0	0	0	0	0	0	0	0	0
Septoria	0	0	0	0	0	0	0	0	0	0	0	0	0	0
Setophaeosphaeria	0	0	0	0	0	0	0	0	0	0	0	0	0	0
Setophoma	0	0	0	0	0	0	0	7432	0	0	0	0	0	0
Sidera	0	0	0	0	0	0	0	0	0	0	0	0	0	0
Simplicillium	0	403	0	49	0	0	173	0	51	0	34829	0	0	239
Sistotrema	0	0	0	0	0	0	0	0	0	0	0	0	0	0
Sistotremastrum	0	0	0	0	0	0	0	0	0	0	0	4652	0	0
Skeletocutis	0	0	0	0	0	0	0	0	0	0	0	0	0	0
Sordaria	0	0	0	0	0	0	0	0	0	0	0	0	0	0
Sphaerulina	0	0	0	0	0	0	0	0	0	0	0	0	0	0
Spiromastix	0	0	0	0	0	0	0	0	0	0	0	0	0	0
Sporobolomyces	518	0	0	7967	0	0	151	38	0	60	0	53	14961	50707
Sporothrix	0	0	0	0	0	0	0	0	0	0	0	0	0	0
Stachybotrys	0	0	0	0	0	0	0	0	0	0	0	0	0	0
Stagonosporopsis	0	0	0	0	0	0	0	0	0	0	0	0	0	0
Starmerella	0	0	0	0	0	0	0	0	0	0	0	0	0	0
Steccherinum	0	0	0	0	0	0	0	0	0	0	0	0	0	0
Stemphylium	0	0	0	0	0	0	0	0	0	0	0	0	0	0
Stephanosporaceae_gen_Incertae_sedi	0	0	0	0	0	0	0	0	0	0	0	0	0	0
Sterigmatomyces	0	0	0	0	0	0	0	0	0	0	0	0	0	0
Strelitziana	0	0	0	0	0	0	0	20579	0	0	0	0	0	0
Striatibotrys	0	0	0	0	0	0	0	0	0	0	0	0	0	0
Strobilurus	0	0	0	0	0	0	0	0	0	0	0	0	0	0
Subulicystidium	0	0	0	0	0	0	0	0	0	0	0	0	0	0
Symmetrospora	0	0	0	0	0	0	0	0	0	0	0	0	0	0
Sympodiomycopsis	0	0	0	0	0	0	0	0	0	0	0	0	0	0
Talaromyces	0	0	160692	35	0	0	0	0	139	0	0	0	0	0
Taphrina	0	0	0	0	0	0	0	0	0	0	0	189	0	0
Tausonia	0	0	0	0	0	135	0	0	0	0	0	0	0	0
Tetracladium	0	0	0	0	0	0	0	0	0	0	0	0	0	0
Tetrapisispora	0	0	0	0	0	0	0	0	0	0	0	0	0	0
Thanatephorus	0	0	0	0	0	0	0	0	0	0	0	0	0	0
Thecaphora	0	0	0	0	0	0	0	0	0	0	0	0	0	0
Thelebolus	0	0	0	0	0	0	0	0	0	0	0	0	0	0
Thermomyces	0	0	0	0	0	0	0	0	0	0	0	0	0	0
Thielaviopsis	0	0	0	0	0	0	0	0	0	0	0	0	0	0
Tilletia	0	0	0	0	0	0	0	0	0	0	0	0	0	0
Tilletiopsis	0	0	0	0	29348	0	0	41	0	0	0	0	0	0
Togniniella	0	0	0	0	0	0	0	0	0	0	0	0	0	0
Torula	0	0	0	0	0	0	0	0	0	0	0	0	0	0
Toxicocladosporium	0	0	0	0	0	0	0	0	0	1907	0	0	0	0
Trametes	0	0	0	0	0	0	0	5273	0	0	0	0	0	0
Trechispora	0	0	0	0	0	0	0	0	0	0	0	0	0	0
Tremateia	0	0	0	0	0	0	0	0	0	0	0	0	0	0
Trichoderma	0	0	0	5034	0	0	0	0	0	0	0	0	0	0
Trichomeriaceae_gen_Incertae_sedis	0	0	0	0	0	0	0	0	0	0	0	0	0	0
Trichomerium	0	0	0	0	0	0	0	0	0	0	0	0	0	0
Trichomonascus	0	0	0	0	0	0	0	0	0	0	0	0	0	0
Trichophaea	0	0	0	0	0	0	0	0	0	0	0	0	0	0
Trichosporon	0	0	0	0	43	0	17383	0	0	0	0	0	0	0
Trichosporonaceae_gen_Incertae_sedi	0	0	0	0	0	0	0	0	0	0	0	0	0	0
Trigonopsis	0	0	0	0	0	0	0	0	0	0	0	0	0	0
Tritirachium	0	0	0	0	0	0	0	0	0	0	0	0	0	0
Tuber	0	0	0	0	0	0	0	0	0	0	41	41	0	0
Tubeufia	0	0	0	0	0	0	0	0	0	0	0	0	0	0
Tulostoma	0	0	0	0	0	0	0	0	0	0	0	0	0	0
Typhula	0	0	0	0	0	0	0	0	0	0	0	0	0	0
Udeniomyces	0	0	0	0	118	787	359	252	186	380	480	71324	148498	414366
Umbilicaria	0	0	0	0	0	0	0	0	0	0	0	0	0	0
Umthunziomyces	0	0	0	0	0	0	0	0	0	0	0	0	0	0

Ustilaginoidea	942	6583	0	0	0	0	1544	38	0	0	0	20701	17261	5768
Uwebraunia	0	0	0	0	0	0	0	0	0	0	0	0	0	0
Vacuiphoma	96	0	0	0	0	0	0	0	0	0	0	0	662	128
Valsaria	0	0	0	0	0	0	0	0	0	0	0	0	0	0
Vandijckella	0	0	0	0	0	0	0	0	0	0	0	0	0	0
Vanrija	0	0	0	0	0	0	0	0	0	1884	0	0	0	0
Vararia	0	0	0	0	0	0	0	0	0	0	0	0	0	0
Venturiaceae_gen_Incertae_sedis	0	0	0	0	0	0	0	0	0	0	0	0	0	0
Verrucaria	0	0	0	3369	0	0	0	0	0	0	0	0	0	0
Vishniacozyma	0	0	0	0	0	0	0	0	0	0	0	0	0	0
Wallemia	0	0	0	1899	0	45	40	0	0	0	0	0	0	0
Wickerhamiella	0	0	0	0	0	0	0	0	0	0	0	0	0	0
Wickerhamomyces	0	0	0	0	0	0	0	0	0	0	0	0	0	0
Xenodevriesia	0	0	0	0	0	0	0	0	0	0	0	0	0	0
Xeromyces	0	0	0	0	0	0	0	0	0	0	0	0	0	0
Xylariaceae_gen_Incertae_sedis	0	0	0	0	0	0	1402	0	0	0	0	0	0	0
Xylocoremium	0	0	0	0	0	0	0	0	0	0	0	0	0	0
Xylodon	0	0	0	0	0	0	0	0	0	0	0	0	0	0
Yamadazyma	0	0	0	0	0	0	0	0	0	0	0	0	0	0
Yarrowia	0	0	0	0	0	0	0	0	0	0	0	0	0	0
Zanclospora	562963	58372	815223	7380	34106	922995	85521	252970	52923	58194	133295	29291	12555	36315
Zasmidium	0	0	0	0	0	0	0	0	0	0	0	0	0	0
Zeloasperisporium	0	0	0	0	0	0	0	0	0	0	0	0	0	0
Zoophthora	534	0	0	0	0	154	307	168	0	0	0	463	0	0
Zygoascus	1166	0	0	0	0	481	0	0	0	0	0	0	0	0
Zygosaccharomyces	0	0	0	0	0	0	0	0	0	0	0	0	0	0
Zygosporium	0	0	0	0	0	0	0	0	0	0	0	0	0	0
Zygotorulaspora	0	0	0	0	0	0	0	0	0	0	0	0	0	0
Zymoseptoria	0	0	0	0	0	0	0	0	0	0	0	0	0	0

Supplementary table S18. The abundance and composition at the species level of each sample among COVID-19 (n=71), Post-COVID-19 (n=22), and control (n=132) groups.

species	CCR001	CCR022	CCR021	CCR020	CCR018	CCR017	CCR016	CCR015	CCR014	CCR013	CCR012	CCR019	CCR010	CCR011
Acremonium_acutatum	0	0	0	0	0	0	0	0	0	0	0	0	0	0
Acremonium_alternatum	642	0	0	0	0	0	0	0	0	0	0	0	0	0
Acremonium_cavaraeanum	0	0	0	0	0	0	0	0	0	0	0	0	0	0
Acremonium_charticola	0	0	0	0	0	0	0	0	0	0	0	0	0	0
Acremonium_egyptiacum	0	0	0	0	0	0	0	0	0	0	0	0	0	0
Acremonium_vitellinum	0	0	0	0	0	0	0	0	0	0	0	0	0	0
Acrocalymma_fici	0	0	0	0	0	0	0	0	0	0	0	0	0	0
Acrodictys_fluminicola	7018	133812	1190	3786	1959	192	6535	5407	396	4759	45825	19214	1143	5482
Acrodontium_crateriforme	0	0	0	0	0	0	0	0	0	0	0	0	0	0
Actinomucor_elegans	185170	32284	445	78292	504340	33704	747726	421560	580785	853582	182828	523021	309198	414755
Adisciso_yakushimense	0	0	0	0	0	0	0	0	0	0	0	0	0	0
Agaricales_spe_Incertae_sedis	0	0	0	0	0	0	0	0	0	0	0	0	0	0
Agaricus_subfloccosus	0	0	0	0	0	0	0	0	0	0	0	0	0	0
Ahmadea_dalanensis	0	0	0	0	0	0	0	0	0	0	0	0	0	0
Akanthomyces_thailandicus	0	0	0	0	0	0	0	0	0	0	0	0	0	0
Allocryptovalsa_polyspora	0	0	0	0	0	0	0	0	0	0	0	0	0	0
Alternaria_alstroemeriae	0	0	0	292	0	0	0	140	0	0	0	0	0	0
Alternaria_alternata	3495	210	743	75139	0	238	2740	9022	0	0	112	42	63003	0
Alternaria_chlamydospora	0	0	0	0	0	0	0	0	0	0	0	0	0	0
Alternaria_hungarica	0	0	0	0	0	0	0	0	0	0	0	0	0	0
Alternaria_nepalensis	0	0	0	0	0	0	0	0	0	0	0	0	0	0
Amphobotrys_ricini	0	0	0	0	0	0	0	0	0	0	0	0	0	0
Amylostereum_orientale	0	0	0	0	0	0	0	0	0	0	0	0	0	0
Anastomitrabeculia_didymospora	0	0	0	0	0	0	0	0	0	0	0	0	0	0
Angustimassarina_camporesii	1400	0	0	0	0	345	222	488	32	0	0	2728	0	0
Anisomeridium_guangdongensis	0	0	0	0	0	0	0	0	0	0	0	0	0	0
Antennariella_placitae	0	0	0	0	0	0	0	0	0	0	0	0	0	0
Anthracina_ramosa	0	0	0	0	0	0	0	0	0	0	0	0	0	0
Anthracina_saxicola	0	0	0	0	0	0	0	0	0	0	0	0	0	0
Apiotrichum_brassicae	0	0	0	0	0	0	0	0	0	0	0	0	0	0
Apiotrichum_domesticum	0	0	0	0	0	0	0	0	0	0	0	0	0	0
Aquapteridospora_fusiformis	0	0	0	0	0	0	0	0	0	0	0	0	0	0
Arachnomyces_jinanicus	0	0	0	0	0	0	0	0	0	0	0	0	0	0
Archaeorhizomyces_borealis	0	0	0	0	0	0	0	0	0	0	0	0	0	0
Arthrinium_rasikravindrae	0	0	0	0	0	0	0	0	0	0	0	0	0	0
Arthrographis_grakistii	0	0	0	0	0	0	0	0	153	0	471	0	0	0
Ascochyta_rabiei	289	0	0	0	0	0	0	0	0	0	0	0	0	143
Ascomycota_spe_Incertae_sedis	0	0	0	0	0	0	0	0	0	0	0	0	0	0
Ascosphaera_duoformis	0	0	0	0	0	0	0	0	0	0	0	0	0	0
Ascotricha_erinacea	0	0	0	0	0	0	0	0	0	0	0	0	0	0
Ascotricha_guamensis	0	0	0	0	0	0	0	0	0	0	0	0	0	0
Aspergillus_amstelodami	799	0	0	0	0	0	0	0	0	0	0	0	13014	51
Aspergillus_baarnensis	0	14349	0	0	0	0	0	0	0	0	0	1511	0	0
Aspergillus_clavatophorus	0	0	0	0	303	0	0	74	42	68	0	429	0	0
Aspergillus_conicus	0	0	0	0	0	0	0	0	0	0	0	0	0	0
Aspergillus_coremiiformis	0	0	0	0	0	0	0	0	0	0	0	0	0	0
Aspergillus_corrugatus	0	0	0	0	0	0	0	0	0	0	0	0	0	0
Aspergillus_flavus	0	0	0	0	0	0	0	0	0	0	0	0	0	0
Aspergillus_foetidus	0	0	0	0	0	0	0	0	0	0	0	0	0	0
Aspergillus_fumisynnematus	0	0	0	0	0	0	0	0	0	0	0	0	0	0
Aspergillus_glabripes	0	0	0	0	0	0	0	0	0	0	0	0	0	0
Aspergillus_inflatus	0	0	0	0	0	0	0	0	0	0	0	0	0	0
Aspergillus_insolitus	0	0	0	0	0	0	0	0	0	0	0	0	0	0
Aspergillus_kalimae	0	0	0	0	0	0	0	0	0	0	0	0	0	4285
Aspergillus_neoglaber	0	0	0	0	0	0	0	0	0	0	0	0	0	0

Aspergillus_ochraceus	0	0	0	0	0	0	0	0	0	0	0	74	0	0
Aspergillus_penicillioides	1546	18378	236	4606	48600	46	919	6632	668	5542	36377	19696	0	1477
Aspergillus_pragensis	0	0	0	0	0	0	0	196	0	0	0	0	0	0
Aspergillus_restrictus	104	195	0	0	2202	0	0	0	5757	0	0	0	0	0
Aspergillus_subflavus	0	3724	0	0	981	97	141	0	154	67	41319	1966	6172	318
Aspergillus_tennesseensis	0	0	0	86	0	0	160	0	0	1346	0	3634	3685	0
Aspergillus_thermomutatus	0	0	0	0	0	0	0	0	0	0	0	0	0	0
Aspergillus_unguis	0	0	0	0	0	0	0	0	0	0	0	0	0	0
Aspergillus_versicolor	93	0	259	1274	1664	0	1262	0	380	87	9741	213	16768	0
Aspergillus_vitricola	0	4588	0	0	330	80	0	0	0	0	0	2633	0	0
Aspergillus_westerdijkiae	0	0	0	0	0	0	0	0	0	0	0	0	0	0
Aspergillus_whitfieldii	0	0	0	0	0	0	0	0	0	0	0	0	0	0
Atractiella_rhizophila	0	0	0	0	0	0	0	0	0	0	0	0	0	0
Aureobasidium_pullulans	0	84005	0	0	0	0	0	0	0	0	0	0	0	0
Aureobasidium_thailandense	0	0	0	0	0	0	0	0	0	0	0	0	0	0
Auricularia_auricula-judae	0	0	0	0	0	0	0	0	0	0	0	0	0	0
Auricularia_delicata	0	0	0	0	0	0	0	0	0	0	0	0	0	0
Austropleospora_keteleeriae	0	0	0	0	0	0	0	0	0	0	0	0	0	0
Avachytrium_platense	0	0	0	0	0	0	0	0	0	0	0	0	0	0
Bandonia_marina	0	606	0	0	0	0	0	0	0	88	0	0	0	0
Banksiophoma_australiensis	0	0	0	0	0	0	0	0	0	0	0	0	0	0
Bannoa_syzygii	727	0	0	0	0	0	0	0	0	0	0	0	0	0
Basidiodendron_globisporum	0	0	0	0	0	0	0	0	0	0	0	0	0	0
Basidiomycota_spe_Incertae_sedis	0	0	0	0	0	0	0	0	0	0	0	0	0	0
Beauveria_bassiana	0	0	0	0	0	0	0	0	0	0	0	0	0	0
Beauveria_lii	0	0	0	0	0	0	0	0	0	0	0	0	0	0
Beauveria_majiangensis	0	615	0	0	0	0	0	0	0	0	0	205	0	0
Bergerella_atrofusca	0	0	0	0	0	0	0	0	0	0	0	0	0	0
Berkeleyomyces_basicola	0	0	0	0	0	0	0	0	0	0	0	0	0	0
Betamyces_spe_Incertae_sedis	0	0	0	0	0	0	0	0	0	0	0	0	0	0
Biatriosporaceae_spe_Incertae_sedis	0	0	0	0	0	0	0	0	0	0	0	0	0	0
Bjerkandera_adusta	0	0	0	0	0	0	0	0	0	0	0	8173	0	0
Bjerkandera_mikrofumosa	0	0	0	0	0	0	0	0	0	0	0	0	0	0
Blastobotrys_proliferans	0	0	0	0	0	0	0	0	0	0	0	0	0	0
Blumeria_graminis	0	0	0	0	0	0	0	0	0	0	0	0	0	0
Blumeria_spe_Incertae_sedis	0	0	0	39	0	0	0	0	35	0	0	0	0	0
Botryosphaeria_corticis	0	0	0	0	0	0	0	0	0	0	0	0	0	0
Botryosphaeria_spe_Incertae_sedis	14217	11476	243	1659	0	49	52	118097	8878	4811	28880	25214	0	0
Botryosphaeria_wangensis	0	0	0	0	0	0	0	0	0	0	0	0	0	0
Botrytis_cinerea	0	0	0	0	0	0	0	3072	0	0	0	0	0	0
Bovista_aestivalis	0	0	0	0	0	0	0	0	0	0	0	0	0	0
Bovista_psammophila	0	0	0	0	0	0	0	0	0	0	0	0	0	0
Bradymyces_alpinus	0	0	0	0	0	0	0	0	0	0	42	0	0	9488
Buckleyzyma_armeniaca	0	0	0	0	0	0	0	0	0	0	0	0	0	0
Buckleyzyma_aurantiaca	0	0	0	0	0	0	0	0	0	0	0	0	0	0
Bulleromyces_albus	0	0	0	0	0	0	0	0	0	0	0	0	0	0
Byssochlamys_spectabilis	0	0	0	0	0	0	0	0	0	0	0	0	0	0
Cadophora_bubakii	0	0	0	0	0	0	0	0	0	0	0	0	0	0
Cadophora_lacrimiformis	981	0	0	0	0	0	0	0	0	0	0	0	0	0
Caloplaca_micromontana	209	0	0	0	0	0	0	0	0	0	0	1322	0	0
Calosphaeria_pulchella	0	0	0	0	0	0	0	0	0	0	0	0	0	0
Calycina_spe_Incertae_sedis	0	0	0	0	0	0	0	0	0	0	0	0	0	0
Calyptella_capula	0	0	0	0	0	0	0	0	0	0	0	0	0	0
Candida_albicans	317	2916	4446	1007	3350	0	46	5346	312335	141	58	72	110	57
Candida_apicola	0	0	0	0	0	0	0	0	0	0	0	0	0	0
Candida_ethanolica	0	0	0	0	0	0	0	0	0	0	0	0	0	0
Candida_haemulonis	0	0	0	0	0	0	0	0	0	0	0	0	0	0
Candida_inconspicua	19951	0	0	0	9390	150	0	1790	0	1147	0	0	0	0
Candida_intermedia	0	0	0	0	0	0	0	0	0	0	0	0	0	0
Candida_maltosa	0	0	0	0	0	0	0	0	0	0	0	0	0	0
Candida_nivariensis	0	0	0	0	0	0	0	0	0	0	0	0	0	0
Candida_parapsilosis	0	424	0	118	0	0	42	0	0	0	0	0	0	0
Candida_saitoana	0	0	0	0	0	0	0	0	0	0	0	0	0	0
Candida_saraburiensis	0	0	0	0	0	0	0	0	0	0	0	0	0	0
Candida_solani	0	0	0	0	0	0	0	0	42	0	0	0	0	240
Candida_spe_Incertae_sedis	0	0	0	0	0	0	0	0	0	0	0	0	0	0
Candida_tropicalis	0	0	0	0	0	0	0	0	0	0	0	0	0	0
Candida_vespimorsuum	0	0	0	0	0	0	0	0	0	0	0	0	0	0
Candida_zeylanoides	0	0	0	7033	0	0	0	0	0	5808	0	0	0	0
Capnodiales_spe_Incertae_sedis	0	0	0	0	0	0	0	0	0	0	0	0	0	0
Carlosrosaea_hohenbergiae	0	0	0	0	0	0	0	0	0	0	0	0	0	0
Ceraceosorus_guamensis	0	0	0	0	0	0	0	0	0	0	0	0	0	0
Ceratocystis_manginecans	0	0	0	0	0	0	0	0	0	0	0	0	0	0
Ceratocystis_pirilliformis	0	0	0	0	0	0	0	0	0	0	0	0	0	0
Cercospora_canescens	2008	0	0	0	0	42	0	0	0	0	0	0	0	0
Ceriporia_lacerata	0	0	0	0	0	0	0	0	0	0	0	0	0	0
Ceriporia_xylostromatoides	0	0	0	0	0	0	0	0	0	0	0	0	0	0
Ceriporiopsis_fimbriata	0	0	0	0	0	0	0	0	0	0	0	0	0	0
Cerrena_aurantiopora	0	0	0	0	0	0	0	0	0	0	0	0	0	0
Chaenothecopsis_resinophila	0	0	0	0	0	0	0	0	0	0	0	0	0	0
Chaetomium_globosum	0	0	0	0	0	0	0	0	0	0	0	0	0	0
Chaetosphaeria_guttulata	0	0	0	0	0	0	0	0	0	0	0	0	0	0
Chalara_hughesii	0	0	0	0	0	0	0	0	0	0	0	0	0	0
Chloridium_pini	0	0	0	0	38	0	0	0	0	0	0	0	0	0
Choanephora_cucurbitarum	0	0	0	0	0	0	0	0	0	0	0	0	0	0
Chrysosphaeria_jan-nelii	0	0	0	0	0	0	0	0	0	0	0	0	0	0
Chytridiomycota_spe_Incertae_sedis	0	0	0	0	0	0	0	0	0	0	0	0	0	0

Chytridium_olla	0	0	0	0	0	0	0	0	0	0	0	0	0	0
Ciboria_shiraiana	0	0	0	0	0	0	0	0	0	0	0	0	0	0
Ciliophora_spe_Incertae_sedis	0	214	0	44	0	0	0	0	0	0	0	0	0	0
Cladophialophora_spe_Incertae_sedis	0	0	0	0	0	0	0	0	0	0	0	0	0	0
Cladosporium_cladosporioides	22532	0	702	261	4433	87	1334	42	570	3220	76390	28719	92	44
Cladosporium_coloradense	0	1031	0	0	0	0	0	30	0	0	58	36	0	0
Cladosporium_halotolerans	0	30073	0	6030	7137	215	349	29182	35	3370	33700	35	0	0
Cladosporium_kenpeggii	5615	0	460	4836	473	35	138	0	450	83	3265	4460	0	0
Cladosporium_spe_Incertae_sedis	606	0	0	32	57	52	1613	0	0	0	746	705	0	0
Cladosporium_sphaerospermum	0	9497	0	43	0	0	0	0	0	0	0	0	0	0
Clavispora_lusitaniae	764	0	0	0	0	346	0	0	0	0	0	0	0	0
Clonostachys_eriocamporesii	0	0	0	0	0	0	0	3386	0	0	0	0	0	0
Clonostachys_rosea	0	0	0	0	0	0	0	0	0	0	0	0	0	0
Colletotrichum_cliviae	0	0	0	0	0	0	0	0	0	0	0	0	0	0
Collybiopsis_filamentipes	0	0	0	0	0	0	0	0	0	0	0	0	0	0
Coniella_quercicola	0	0	0	0	0	0	0	0	0	0	0	0	0	0
Coniochaeta_mutabilis	0	0	0	0	0	0	0	0	0	0	0	0	0	0
Coniosporium_apollinis	0	0	0	0	0	0	0	0	0	0	0	0	0	0
Coniosporium_spe_Incertae_sedis	0	0	0	0	0	0	0	0	0	0	0	0	0	0
Coniothyrium_spe_Incertae_sedis	0	0	0	0	0	0	0	0	0	0	0	0	0	0
Cookeina_korfii	672	12456	0	4244	0	0	39	0	0	0	0	0	0	0
Coprinellus_ovatus	0	0	0	0	0	0	0	0	0	0	0	0	0	0
Coprinellus_pseudodisseminatus	0	0	0	0	0	0	0	0	0	0	0	0	0	0
Coprinopsis_atramentaria	0	0	0	0	0	0	0	0	0	0	0	3253	0	0
Coprinopsis_clastophylla	0	0	0	0	0	0	0	0	0	0	0	0	0	0
Coprinopsis_strossmayeri	0	0	0	0	0	0	0	0	0	0	0	0	0	0
Coprinopsis_urticicola	0	0	0	0	0	0	0	0	0	0	0	0	0	0
Cordyceps_ghanensis	0	0	0	0	0	0	0	1998	0	0	0	0	0	0
Cordyceps_lepidopterorum	0	0	0	0	0	0	0	0	0	0	0	0	0	0
Cordyceps_succavus	0	193	0	0	0	0	0	0	0	0	0	0	104	148
Coronicium_gemmiferum	758	0	0	0	0	0	0	0	0	0	0	0	0	0
Crustoderma_spe_Incertae_sedis	0	0	0	0	0	0	0	0	0	0	0	0	0	0
Cryptococcus_longus	0	0	0	0	0	0	0	0	0	0	0	0	0	0
Cryptococcus_neoformans	0	0	0	0	0	0	0	0	0	0	0	0	0	0
Cryptococcus_uniguttulatus	0	0	0	0	0	0	0	0	0	0	0	0	0	0
Cryptomarasmius_aucubae	0	0	0	4590	0	0	0	0	0	0	0	0	0	0
Curvularia_chonburiensis	0	0	0	0	0	0	0	0	0	0	0	0	0	0
Curvularia_soli	0	0	0	0	0	0	0	0	0	0	0	0	0	0
Cutaneotrichosporon_arboriforme	0	0	0	0	0	0	0	0	0	0	0	0	0	0
Cutaneotrichosporon_cutaneum	0	0	0	0	0	0	0	0	0	0	0	0	0	0
Cutaneotrichosporon_jirovecii	0	0	0	0	0	0	0	0	0	0	0	0	0	0
Cyphellaceae_spe_Incertae_sedis	0	0	0	0	0	0	0	0	0	0	0	0	0	0
Cyphellophora_olivacea	0	0	0	0	0	0	0	0	0	0	0	0	0	0
Cyphellophora_sessilis	0	0	0	0	0	0	0	0	0	0	0	0	0	0
Cystobasidium_slooffiae	0	9715	0	0	0	0	0	0	625	4319	0	0	0	0
Cystofilobasidium_capitatum	0	0	0	0	0	0	0	0	0	0	0	0	0	0
Cystofilobasidium_infirmominiatum	0	0	0	0	0	0	0	0	0	0	0	0	0	0
Cystofilobasidium_macerans	0	0	0	0	0	0	0	0	0	0	0	0	0	0
Cytospora_brevispora	0	0	0	0	0	0	0	0	0	0	0	0	0	0
Cytospora_myrtagena	0	0	0	0	0	0	0	0	0	0	0	0	0	0
Cytospora_predappioensis	0	0	0	0	0	0	0	0	0	0	0	0	0	0
Dactylella_spe_Incertae_sedis	0	0	0	0	0	0	0	0	0	0	0	0	0	0
Dactylodendron_pinicola	0	0	0	0	0	0	54	26362	0	0	0	0	0	0
Debaryomyces_prosopidis	859	379	0	672623	0	0	0	0	0	0	0	5290	0	31
Deconica_spe_Incertae_sedis	0	0	0	0	0	0	0	0	0	0	0	0	0	0
Delastria_faustiniana	0	0	0	0	0	0	0	0	0	0	0	0	0	0
Dendryphiella_paravinosa	0	0	0	0	0	0	0	0	0	0	0	0	0	0
Derxomyces_mrakii	0	0	0	0	0	0	0	0	0	0	0	0	0	0
Diaporthe_bohemiae	0	0	0	0	0	0	0	0	0	0	0	0	0	0
Diaporthe_caulivora	0	0	0	0	0	0	0	0	0	0	0	0	0	0
Diaporthe_celeris	0	0	0	0	0	0	0	0	0	0	0	0	0	0
Diaporthe_ganjae	0	0	0	0	0	0	0	0	0	0	0	0	0	0
Diaporthe_kochmanii	942	0	0	0	0	0	0	0	0	1213	0	0	0	0
Diaporthe_miriciae	0	0	0	0	0	0	0	0	0	0	0	0	0	0
Diaporthe_pseudooculi	0	0	0	0	0	0	0	0	0	0	0	0	0	0
Didymella_dimorpha	0	0	0	0	0	0	0	0	0	0	0	0	0	0
Didymella_keratinophila	0	0	0	0	0	0	0	0	0	0	0	0	0	0
Didymella_spe_Incertae_sedis	0	0	0	0	0	0	0	0	0	0	0	6558	0	0
Didymocyrtis_brachylaenae	0	0	0	0	0	0	0	0	0	0	0	0	0	0
Dimorphoma_saxea	0	27250	0	0	0	0	0	0	0	0	0	0	0	0
Dioszegia_catarinonii	0	0	0	0	0	0	0	0	0	0	0	0	0	0
Dioszegia_zsoltii_var._yunnanensis	0	0	0	0	0	42	0	0	0	0	0	15544	0	0
Dipodascus_geotrichum	112	1688	237	0	120	0	88	0	9728	0	0	0	0	2937
Diutina_catenulata	0	0	0	0	0	0	0	0	0	0	0	0	0	0
Diutina_rugosa	0	0	0	0	0	0	0	2013	0	0	0	0	0	0
Diversispora_spurca	323	0	505	0	0	134	1181	0	5037	0	0	0	0	0
Dothidea_eucalypti	0	0	0	0	0	0	0	0	0	0	0	0	0	0
Dothidea_sambuci	0	0	0	0	0	0	0	0	0	0	0	0	0	0
Dothiora_spartii	0	0	0	0	0	0	0	0	0	0	0	0	0	0
Echinoporia_hydnophora	0	0	0	0	0	0	0	0	0	0	0	0	0	0
Edenia_gomezpompae	0	0	0	0	0	0	0	0	0	0	0	0	0	0
Effuseotrichosporon_vanderwaltii	0	0	0	0	0	0	0	0	0	0	0	0	0	0
Elaphomyces_anthracinus	0	0	0	0	0	0	0	0	0	0	0	0	0	0
Elsinoe_leucopogonis	0	0	0	0	0	0	0	0	0	0	0	0	0	0
Emergomyces_canadensis	0	0	0	0	0	0	0	0	0	0	0	0	0	0
Emericellopsis_robusta	0	0	0	0	0	0	0	0	815	0	0	0	0	0
Engyodontium_album	0	6184	0	0	0	0	1702	0	704	0	0	0	0	0

Entoloma_graphitipes_f._cystidiatum	0	0	0	0	0	0	0	0	0	0	84	0	0	0
Entorrhiza_citriformis	1298	0	0	0	0	84	320	0	0	0	0	0	0	0
Entorrhiza_parvula	103	0	0	0	0	0	86	0	0	0	0	0	0	0
Entyloma_calendulae	0	0	0	0	0	0	0	0	0	0	0	0	0	0
Entyloma_cosmi	0	0	0	0	0	0	0	0	0	0	0	0	0	0
Entyloma_linariae	0	0	0	0	0	0	0	0	0	0	0	0	0	0
Epicoccum_dendrobii	95	0	0	0	0	0	0	0	0	0	0	0	0	37
Epicoccum_duchesneae	0	0	0	0	0	0	0	0	0	0	0	0	0	0
Epicoccum_viticis	0	0	0	0	0	0	0	0	0	0	0	0	0	0
Eremothecium_ashbyi	0	0	0	0	0	0	0	0	0	0	0	0	0	0
Eremothecium_coryli	0	0	0	0	0	0	0	0	0	3034	0	0	0	0
Erysiphe_azaleae	0	0	0	0	0	0	0	0	0	769	0	0	0	0
Erysiphe_medicaginis	0	0	0	0	0	0	0	0	0	0	0	0	0	0
Erythrobasidium_hasegawianum	0	0	0	0	0	0	0	0	0	0	0	0	0	0
Erythrobasidium_yunnanense	0	0	0	0	0	0	0	0	0	0	0	0	0	0
Eupenidiella_venezuelensis	227	0	0	0	0	0	0	0	0	0	0	0	0	0
Eutypella_spe_Incertae_sedis	0	0	0	0	0	0	0	0	0	0	0	0	0	0
Eutypella_tamaricis	0	0	0	0	0	0	38	9057	51	0	0	0	0	0
Exidia_pithya	0	0	0	0	0	0	39	0	0	0	0	0	35773	0
Exobasidium_spe_Incertae_sedis	0	0	0	0	0	0	0	0	0	0	0	0	0	0
Exophiala_dermatitidis	0	0	0	0	0	0	0	0	0	0	0	0	0	0
Extremus_spe_Incertae_sedis	0	0	0	0	0	0	0	0	0	0	0	0	0	0
Fantasmomyces_hyalinus	0	437	0	0	36	0	0	0	0	0	0	218	0	0
Filobasidium_globisporum	0	0	0	0	0	0	0	0	0	0	0	0	0	0
Filobasidium_oeirense	0	0	0	21225	44521	45	40	0	46	0	0	6396	0	0
Filobasidium_wieringae	0	0	0	0	0	0	0	0	0	0	0	0	0	0
Flagelloscypha_spe_Incertae_sedis	0	0	0	0	0	0	0	0	0	0	0	0	0	0
Flammocladiella_aceris	0	0	0	0	601	0	0	0	0	0	0	0	0	0
Flammulina_filiformis	0	0	0	0	0	0	0	0	0	0	0	0	0	0
Flammulina_velutipes	0	0	0	0	0	0	0	0	0	0	0	0	0	0
Flavodon_spe_Incertae_sedis	0	0	0	0	0	0	0	0	0	0	0	0	0	0
Fomitopsis_eucalypticola	0	0	0	0	0	0	0	0	0	0	0	0	0	0
Fracchiaea_myricoides	0	0	0	0	0	0	0	0	0	0	0	0	0	0
Fungi_spe_Incertae_sedis	19398	3628	9258	424	1963	31751	2599	9508	2378	1500	9373	891	2049	1749
Fusarium_fujikuroi	0	0	0	0	0	0	0	0	49	63	0	8121	0	0
Fusarium_irregulare	0	0	0	185	0	0	467	0	0	0	0	0	0	0
Fusarium_lacertarum	0	0	0	4878	0	0	13881	0	0	0	0	0	0	0
Fusarium_neocosmosporiellum	0	0	0	0	0	0	0	0	0	0	0	0	0	0
Fusarium_oxysporum	0	0	0	0	0	0	0	0	0	0	0	0	0	0
Fusarium_poae	3208	0	0	0	0	0	0	0	0	0	0	0	0	3463
Fusarium_ramigenum	0	0	0	0	0	0	0	0	0	0	0	0	0	0
Fuscosporella_aquatica	0	0	0	0	0	0	0	0	0	0	0	0	0	0
Fusicolla_acetilerea	0	0	0	0	0	0	0	0	0	0	0	0	0	0
GS08_spe_Incertae_sedis	0	0	0	0	0	0	0	0	0	0	0	0	0	0
Galactomyces_pseudocandidus	13743	7635	0	0	67851	298	12763	528	1145	3436	0	0	1853	263
Ganoderma_australe	0	0	0	0	0	0	0	0	0	0	0	0	0	0
Genea_brunneocarpa	0	0	0	0	0	0	0	0	34	0	0	0	0	0
Genolevuria_amylolytica	0	0	0	0	0	0	0	0	0	0	0	0	0	0
Geosmithia_rufescens	0	0	0	0	0	0	0	0	0	0	0	0	0	0
Geotrichum_silvicola	0	0	0	0	0	1170	0	0	0	0	0	0	0	0
Gibberella_baccata	0	0	0	0	0	0	0	0	0	0	0	0	0	0
Gibberella_fujikuroi	0	0	0	4723	0	0	0	3665	0	0	0	0	0	12196
Gibellula_shennongjiaensis	0	0	0	0	0	0	0	0	0	0	0	0	0	0
Gibellulopsis_serrae	0	0	0	0	0	0	0	0	0	0	0	0	0	0
Gjaerumia_minor	0	0	0	0	0	0	0	0	0	0	0	0	0	0
Glomus_macrocarpum	0	0	0	0	0	0	0	0	0	0	0	0	0	0
Golovinomyces_latisporus	0	0	0	0	0	0	0	0	0	0	0	0	0	0
Golovinomyces_sonchicola	0	0	0	0	0	0	0	0	0	0	0	0	0	0
Golubevia_pallescens	0	0	0	0	2133	0	0	0	0	0	0	0	0	0
Gonatophragmium_spe_Incertae_sedis	0	0	0	0	0	0	0	0	0	0	0	0	0	0
Graphium_penicillioides	0	0	0	0	0	0	0	0	0	0	0	0	0	0
Gregarithecium_curvisporum	0	0	0	0	0	0	0	0	0	0	0	0	0	0
Groenewaldozyma_tartarivorans	0	0	0	0	0	0	0	0	0	0	0	0	0	0
Gymnopus_variicolor	0	0	0	0	0	0	0	0	0	0	0	0	0	0
Halosphaeriaceae_spe_Incertae_sedis	0	0	0	0	0	0	0	0	0	0	0	0	0	0
Hannaella_kunmingensis	0	0	0	0	0	0	0	0	0	0	0	0	0	0
Hannaella_oryzae	0	0	0	0	0	0	0	0	0	0	0	0	0	0
Hannaella_pagnoccae	0	0	0	0	0	0	0	0	0	0	0	0	0	0
Hannaella_sinensis	0	0	0	0	0	0	0	0	0	0	0	0	0	0
Hannaella_surugaensis	0	0	0	0	0	0	0	0	0	0	0	0	0	0
Hanseniaspora_nectarophila	0	0	0	0	0	44	0	0	0	0	0	0	0	0
Hazslinszkyomyces_aloes	0	0	0	0	0	0	0	0	0	0	0	0	0	0
Heitmania_litseae	0	0	0	0	0	0	0	0	0	0	0	0	0	0
Helicoma_brunneisporum	0	0	0	0	0	0	0	0	0	0	0	0	0	0
Heliocybe_villosa	0	0	0	0	0	0	0	0	0	0	0	0	0	0
Henningsia_resupinata	0	0	0	0	0	0	0	0	0	0	0	0	0	0
Heydenia_spe_Incertae_sedis	0	5036	0	0	0	0	0	0	0	0	0	0	0	0
Hohenbuehelia_portegna	0	0	0	0	0	0	0	5180	0	0	0	0	0	0
Holtermanniella_takashimae	0	0	0	0	0	0	0	0	0	0	0	0	0	0
Homortomyces_tamaricis	0	0	0	0	0	0	0	0	0	0	0	0	0	0
Hormodochis_melanochlora	0	0	0	0	0	0	0	0	0	0	0	0	0	0
Hortaea_werneckii	0	0	0	0	0	0	0	0	0	0	0	0	0	0
Houjia_yanglingensis	0	0	0	0	0	0	0	0	0	0	0	0	0	0
Huntiella_tyalla	0	0	0	0	0	0	0	0	0	0	0	0	0	0
Hydnobolites_roseus	0	210	0	91	0	0	0	0	0	0	0	97	52	0
Hydropisphaera_spe_Incertae_sedis	0	0	0	0	0	0	0	0	0	0	0	0	0	0
Hyphopichia_burtonii	0	0	0	0	0	0	0	0	0	0	0	0	0	0

Hypoxylon_perforatum	0	0	0	0	0	0	0	0	0	0	0	0	0	0
Hysterobrevium_walvisbayicola	2752	0	0	0	0	0	0	0	0	0	0	0	0	0
Inocyclus_angularis	0	0	0	0	0	0	0	0	0	0	0	0	0	0
Iodophanus_testaceus	0	0	0	0	0	0	0	0	0	0	0	0	0	0
Iodosphaeria_phyllophila	0	0	0	0	0	0	0	0	0	0	0	0	0	0
Irpex_lacteus	0	0	0	0	25513	0	0	0	0	0	0	0	0	0
Issatchenkia_orientalis	0	0	0	0	0	0	0	0	0	0	0	0	0	0
Itersonilia_perplexans	0	0	0	0	0	47	0	3583	346	0	0	9835	0	0
Jaminaea_angkorensis	0	0	0	0	0	0	0	0	0	0	0	0	0	0
Jattaea_ribicola	0	0	0	0	0	0	0	0	0	0	0	0	0	0
Kazachstania_exigua	0	0	0	0	0	0	0	0	0	0	0	0	0	0
Kazachstania_heterogenica	1579	21227	0	0	36	55	0	8972	0	0	0	0	25103	5166
Kazachstania_humilis	0	0	0	0	0	0	0	0	0	0	0	0	0	0
Keissleriella_quadriseptata	0	0	0	0	0	0	0	0	0	0	0	0	0	0
Keratinophyton_linfenense	0	0	0	0	0	0	0	0	0	0	0	0	0	0
Kluyveromyces_lactis	0	0	0	0	0	0	0	0	0	0	0	0	0	0
Kneiffiella_efibulata	0	0	0	0	0	0	0	0	0	0	0	0	0	0
Knufia_cryptophialidica	0	0	0	0	0	0	0	0	0	0	0	0	0	0
Knufia_epidermidis	0	0	0	45	0	0	0	0	0	0	0	0	0	0
Knufia_hypolithi	0	0	0	0	0	0	0	0	0	0	0	0	0	0
Knufia_karalitana	0	0	0	0	0	0	1106	0	0	84	0	0	0	0
Knufia_tsunedae	0	0	0	0	0	0	0	0	0	0	0	0	0	0
Kodamaea_ohmeri	0	0	0	0	0	0	0	0	0	0	0	0	0	0
Komagataella_phaffii	0	0	0	0	0	0	0	0	0	0	0	0	0	0
Komagataella_ulmi	0	0	0	0	0	0	0	0	0	0	0	0	0	0
Kondoa_thailandica	1961	0	0	0	0	0	0	0	0	0	0	0	0	0
Kurtzmaniella_quercitrusa	0	0	0	0	0	0	0	0	0	0	0	0	0	0
Laburnicola_dactylidis	0	0	0	0	0	0	0	0	0	0	0	0	0	0
Laetisaria_lichenicola	0	0	0	0	0	0	40	0	0	0	0	0	0	0
Lecanicillium_coprophilum	0	0	0	0	0	0	0	0	0	0	0	0	0	0
Lembosiniella_eucalyptorum	0	0	0	0	0	0	0	0	0	0	0	0	0	0
Lentinula_edodes	0	0	0	0	0	0	0	0	0	0	0	0	0	0
Lentinus_arcularius	0	0	0	0	0	0	0	0	0	0	0	0	0	0
Lentinus_squarrosulus	0	0	0	0	0	0	0	0	0	0	0	0	0	0
Lentithecium_aquaticum	0	0	0	0	0	0	0	0	0	0	0	0	0	0
Leptospora_rubella	0	0	0	0	0	0	0	0	0	0	0	0	0	4792
Leptospora_spe_Incertae_sedis	0	0	0	0	0	0	0	0	0	0	0	0	0	0
Leptosporella_bambusae	0	0	0	0	33	38	0	85	0	0	44	0	101	0
Leucosporidium_golubevii	0	0	0	0	0	0	0	0	0	0	0	0	0	0
Lipomyces_doorenjongii	0	0	0	0	0	0	0	0	0	0	0	0	0	0
Lithohypha_aloicola	0	0	0	0	0	0	0	37	865	0	0	0	0	0
Lophiostoma_cynaroidis	0	0	0	0	0	0	0	0	0	0	0	0	0	0
Lophiostoma_spe_Incertae_sedis	0	0	0	0	0	0	0	0	0	0	0	0	0	0
Lyomyces_spe_Incertae_sedis	0	0	0	0	0	0	0	0	0	0	0	0	0	0
Macrohyporia_dictyopora	0	0	0	0	0	0	0	0	0	0	0	0	0	0
Malassezia_arunalokei	0	218320	0	0	0	0	998	0	44	0	0	0	58179	0
Malassezia_cuniculi	0	0	0	0	0	0	0	0	0	0	0	0	0	0
Malassezia_dermatis	0	0	0	0	0	0	0	0	0	0	0	0	0	0
Malassezia_furfur	0	0	0	0	0	0	0	0	0	0	0	0	0	0
Malassezia_globosa	0	72297	0	0	0	49	566	0	0	66	0	6785	13407	4181
Malassezia_japonica	0	0	0	0	0	0	0	0	0	0	0	0	0	0
Malassezia_restricta	6176	66675	1136	4072	81743	617	13734	15183	1530	1915	72061	131235	32501	130
Malassezia_sympodialis	0	0	0	0	0	46	831	0	0	81	0	12701	0	3479
Marantokordyana_oberwinkleriana	0	0	0	0	0	0	0	0	0	0	0	0	0	0
Meira_argovae	0	0	0	0	0	0	0	0	0	0	0	0	0	0
Meira_nashicola	0	0	0	0	0	0	0	0	0	0	0	0	0	0
Meira_spe_Incertae_sedis	0	0	0	0	0	0	0	0	0	0	0	0	0	0
Meyerozyma_guilliermondii	0	0	0	0	0	0	0	0	0	0	0	0	0	0
Microascus_paisii	0	0	0	0	0	0	0	0	0	0	0	0	0	0
Microascus_trigonosporus	0	0	0	0	0	0	0	0	0	0	0	0	0	0
Milospium_graphideorum	0	4247	0	0	0	79	0	102	326	0	0	0	0	0
Moesziomyces_bullatus	310	0	0	0	6350	0	0	0	0	0	0	0	0	0
Monoblepharis_macrandra	0	0	0	0	0	0	0	0	0	0	0	0	0	0
Montagnula_opulenta	0	0	233	5078	0	0	0	0	0	0	0	0	0	0
Mrakia_aquatica	0	0	0	0	0	0	0	0	0	0	0	0	0	0
Mrakia_frigida	0	0	0	0	0	0	0	0	0	0	0	0	0	0
Mucor_circinelloides	41260	26334	0	36	8879	186	58854	5370	1386	5128	25186	39	12018	2733
Mucor_pseudolusitanicus	1709	0	0	0	43	353	2643	1268	77	0	152	454	0	326
Mucor_racemosus	205	0	0	0	0	0	46	271	849	0	0	0	0	0
Mycoarthris_spe_Incertae_sedis	0	0	0	0	0	0	0	0	0	0	0	0	0	0
Mycosphaerellaceae_spe_Incertae_sed	0	0	0	0	0	0	0	0	0	0	0	0	0	0
Naganishia_albida	0	0	0	0	0	0	0	0	0	0	0	0	0	0
Naganishia_albidosimilis	3122	0	0	0	0	0	0	0	0	0	0	0	0	0
Naganishia_globosa	0	0	0	0	0	0	0	0	0	0	0	0	0	0
Nectriopsis_exigua	0	0	0	0	0	0	0	0	0	0	0	0	0	0
Nemania_viridis	0	0	0	0	0	0	0	0	0	0	0	0	0	0
Neoacladium_indicum	0	0	0	0	0	0	0	0	0	0	0	0	0	0
Neoascochyta_paspali	0	0	0	0	0	0	0	0	0	0	0	0	0	0
Neocelosporium_corymbiae	0	28331	0	0	0	0	0	0	0	0	0	0	0	0
Neoconiothyrium_viticola	0	0	0	0	0	0	0	0	0	0	0	0	0	0
Neocucurbitaria_irregularis	0	0	0	0	0	0	0	0	0	0	0	0	0	0
Neodevriesia_lagerstroemiae	0	0	0	0	0	0	0	0	0	0	0	0	0	0
Neodevriesia_sexualis	0	0	0	0	0	0	0	0	0	0	0	0	0	0
Neodevriesia_spe_Incertae_sedis	0	0	0	0	0	0	0	0	0	0	0	0	0	0
Neodevriesiaceae_spe_Incertae_sedis	0	0	0	0	0	0	0	0	0	0	0	0	0	0
Neoerysiphe_galeopsidis	0	0	0	0	0	0	0	0	0	0	0	0	0	0
Neofusicoccum_buxi	0	0	0	0	0	0	0	0	0	0	0	0	0	0

eomicrosphaeropsis_alhagi-pseudalha	12494	0	0	0	0	0	46	0	0	0	0	0	6708	2715
Neosetophoma_poaceicola	0	0	0	0	0	0	0	0	0	0	0	0	0	0
Neosetophoma_samararum	0	0	0	0	0	0	0	0	0	0	0	0	0	0
Nigrograna_spe_Incertae_sedis	0	0	0	0	0	0	1503	0	0	0	0	0	0	0
Nigrospora_oryzae	837	0	0	0	0	0	461	0	2083	2748	0	0	46	1942
Nigrospora_spe_Incertae_sedis	0	0	0	0	0	0	0	0	0	0	0	0	0	0
Novakomyces_olei	17028	12460	0	0	0	0	0	0	0	63	0	0	0	0
Ochrocladosporium_frigidarii	0	0	0	0	0	0	0	0	0	0	0	0	0	0
Ochroconis_mirabilis	0	0	0	0	0	0	0	0	0	0	0	0	0	0
Oliveonia_spe_Incertae_sedis	0	0	0	0	0	0	0	0	0	0	0	0	0	0
Ophiosphaerella_agrostidis	0	0	0	0	0	0	0	0	0	0	0	0	0	0
Ophiosphaerella_aquatica	0	0	0	0	0	0	0	0	0	0	0	0	0	0
Ophiostoma_acarorum	0	0	0	0	0	0	0	0	0	0	0	0	0	0
Ophiostoma_pusillum	0	0	0	0	0	0	0	0	0	0	0	0	0	0
Ophiostoma_rachisporum	0	0	0	0	0	0	0	161	0	0	0	0	0	0
Paecilomyces_spe_Incertae_sedis	0	0	0	0	0	0	0	0	0	0	0	0	0	0
Paludomyces_mangrovei	0	0	0	0	0	0	0	0	0	0	0	0	0	0
Papiliotrema_aurea	0	0	0	0	0	0	0	0	0	0	0	0	0	0
Papiliotrema_flavescens	0	0	0	0	0	0	0	0	0	0	0	0	0	0
Papiliotrema_terrestris	0	0	0	0	0	0	0	0	0	0	0	0	0	0
Paracremonium_binnewijzendii	0	0	0	0	0	0	0	0	0	0	0	0	0	0
Parafuscosporella_mucosa	438	425	663	0	0	879	82	93	0	0	108	128	0	0
Paraleptosphaeria_rumicis	0	0	0	0	0	0	0	0	0	0	0	0	0	0
Paraloratospora_gahniae	0	0	0	0	0	0	0	0	2876	70	0	0	0	0
Paraphoma_chlamydocopiosa	0	0	0	0	0	0	0	0	0	0	0	0	0	0
Pararamichloridium_caricicola	0	0	0	0	0	0	0	0	0	0	0	0	0	0
Parasarocladium_aestuarinum	0	0	0	0	0	0	0	0	0	0	0	0	0	0
Parastagonosporella_fallopiae	0	0	0	0	0	0	0	0	0	0	0	0	0	0
Parasympodiella_longispora	0	0	0	0	0	0	0	0	0	0	0	0	0	0
Parathyridaria_rosae	0	0	0	0	0	0	0	0	0	0	0	0	0	0
Paratritirachium_cylindroconium	0	0	0	0	0	0	0	0	0	0	0	0	0	0
Penicillifer_martinii	0	0	0	0	0	0	0	0	0	0	0	0	0	0
Penicillium_adametzioides	0	0	0	0	0	0	0	0	125	0	0	0	0	0
Penicillium_aurantiogriseum	0	0	0	0	0	0	0	0	0	0	0	0	0	0
Penicillium_bialowiezense	0	0	0	0	0	0	0	0	0	0	0	0	0	0
Penicillium_brevicompactum	0	0	0	0	0	0	0	0	0	0	0	0	0	0
Penicillium_cinnamopurpureum	0	0	0	0	0	0	42	0	0	0	0	0	8210	0
Penicillium_citrinum	0	0	0	0	0	0	0	0	0	0	0	0	0	0
Penicillium_estinogenum	0	0	0	0	0	0	0	0	0	0	0	0	0	0
Penicillium_fructuariae-cellae	0	0	0	0	0	0	0	0	0	0	0	0	0	0
Penicillium_glabrum	0	0	0	0	0	0	0	0	0	0	0	0	0	0
Penicillium_hirsutum	325	204	0	52	0	0	616	0	310	0	16071	54	0	0
Penicillium_jiangxiense	0	0	0	0	0	0	0	0	0	0	0	0	0	0
Penicillium_lanosocoeruleum	0	0	0	0	0	0	0	0	0	0	0	0	0	0
Penicillium_ludwigii	0	0	0	0	0	0	0	0	0	0	0	0	0	0
Penicillium_mallochii	0	0	0	0	0	0	0	0	0	0	0	0	0	0
Penicillium_melanoconidium	0	0	0	0	0	0	0	0	0	0	0	0	0	0
Penicillium_multicolor	0	0	0	0	0	0	0	0	0	0	0	0	0	0
Penicillium_oxalicum	0	0	0	0	0	0	0	0	0	0	0	0	0	0
Penicillium_pancosmium	0	0	0	0	0	0	0	0	0	0	0	0	0	0
Penicillium_pimiteouiense	0	0	0	0	0	0	0	0	0	0	0	0	0	0
Penicillium_steckii	0	0	0	0	0	0	0	0	0	0	0	0	0	0
Penicillium_sumatraense	0	0	0	0	0	0	0	0	0	0	0	0	0	0
Penidiella_spe_Incertae_sedis	0	0	0	0	0	0	0	0	0	0	0	0	0	0
Peniophora_cinerea	0	0	0	0	0	0	0	0	0	0	0	0	0	0
Peniophora_crassitunicata	0	0	0	0	0	0	0	0	0	0	0	4166	0	0
Peniophora_pseudoversicolor	0	0	0	0	0	0	0	1970	0	78	46546	0	41	0
Peniophorella_praetermissa	0	0	0	0	0	0	0	0	0	0	0	0	0	0
Peniophorella_spe_Incertae_sedis	0	0	0	0	0	0	0	0	0	0	0	0	0	0
Periconia_byssoides	0	0	0	0	0	0	0	0	0	66	0	0	0	8936
Periconia_circinata	0	0	0	0	0	0	0	0	0	0	0	0	0	0
Periconia_cortaderiae	0	0	0	0	0	0	0	0	0	0	0	0	0	0
Periconia_epilithographicola	0	0	0	0	0	0	0	0	0	0	0	0	0	0
Periconia_homothallica	0	0	0	0	0	0	0	0	0	0	0	0	0	0
Periconia_macrospinosa	0	0	0	0	0	0	0	0	0	0	0	0	0	0
Peroneutypa_scoparia	0	0	0	0	0	0	0	0	0	0	0	0	0	0
Pestalotiopsis_dracontomelon	0	0	0	0	0	0	0	0	0	0	0	0	0	0
Pestalotiopsis_vismiae	0	0	0	0	0	0	0	0	0	0	0	0	0	0
Peyronellaea_spe_Incertae_sedis	0	0	0	0	0	0	0	0	0	0	0	0	0	0
Peziza_fruticosa	0	0	0	0	0	0	0	0	0	0	0	0	0	0
Phacidium_lacerum	0	0	0	0	0	0	0	0	0	0	0	0	0	0
Phaeoacremonium_griseo-olivaceum	0	0	0	0	0	0	0	0	0	0	0	0	0	0
Phaeophleospora_hymenocallidicola	0	0	0	0	0	0	0	0	0	0	0	0	0	0
Phaeosphaeria_acaciae	0	0	0	0	0	0	0	0	0	0	0	0	0	0
Phaeosphaeria_fuckelii	0	0	0	0	0	0	0	0	0	0	0	0	0	0
Phaeosphaeria_oryzae	0	0	0	0	0	0	1586	0	0	0	0	0	0	0
Phanerochaete_chrysosporium	0	0	0	0	0	0	0	0	0	0	0	0	0	0
Phanerochaete_spe_Incertae_sedis	0	0	0	0	0	0	0	0	0	0	0	0	0	0
Phialemoniopsis_spe_Incertae_sedis	0	0	0	0	0	0	0	0	0	4789	0	0	0	0
Phialocephala_trigonospora	0	0	0	0	0	0	0	0	0	0	0	0	0	0
Phlebia_spe_Incertae_sedis	0	0	0	0	0	0	665	0	0	0	0	0	0	0
Phlebia_tuberculata	0	0	0	0	0	0	0	0	0	0	0	0	0	0
Phlebiopsis_flavidoalba	0	0	0	0	0	0	0	0	0	0	0	0	0	0
Phlebiopsis_spe_Incertae_sedis	0	0	0	0	0	0	0	0	0	0	0	0	0	0
Phomatospora_biseriata	0	0	0	0	2899	0	0	0	0	0	0	0	0	0
Phyllachora_cynodonticola	11341	0	688	0	0	2581	9703	5416	0	72	0	4352	0	0
Phyllosticta_capitalensis	0	0	0	0	0	0	0	0	0	0	0	0	0	0

Physisporinus_tibeticus	0	0	0	0	0	0	0	0	0	0	0	0	3519	0
Pichia_cactophila	0	0	0	0	0	0	0	0	352	0	0	0	0	0
Pichia_fermentans	0	0	0	0	0	85	0	0	0	0	0	0	0	0
Pichia_kluyveri	0	0	0	0	0	0	0	0	0	0	0	0	0	0
Pichia_mandshurica	0	0	0	0	0	0	0	0	0	0	0	0	0	0
Plectosphaerella_cucumerina	0	0	0	0	0	0	0	0	0	0	0	0	0	0
Plectosphaerella_niemeijerarum	0	0	0	0	0	0	0	0	0	0	0	0	0	0
Plenodomus_enteroleucus	0	0	0	0	0	0	0	0	0	0	0	0	0	0
Pleurotus_eryngii	0	0	0	0	0	0	0	0	0	0	0	0	0	0
Pleurotus_ostreatus	0	0	0	0	0	0	0	0	0	1482	0	6417	0	0
Podosphaera_aphanis	0	0	0	0	0	0	0	0	0	0	0	0	0	0
Podosphaera_cunningtonii	0	0	0	0	0	0	0	0	0	0	0	0	0	0
Podosphaera_xanthii	0	0	0	0	0	0	0	0	0	0	0	0	0	0
Powellomyces_spe_Incertae_sedis	0	0	0	0	0	0	0	0	0	0	0	0	0	0
Preussia_procaviae	0	0	0	0	0	0	0	0	0	0	0	0	0	0
Protomyces_inouyei	0	0	0	0	0	0	0	0	0	0	0	0	0	0
Psathyrella_candolleana	0	0	0	0	0	0	0	0	0	0	0	0	0	0
Pseudocercospora_spe_Incertae_sedis	0	0	0	0	0	0	0	0	0	0	0	0	0	0
Pseudogymnoascus_spe_Incertae_sedi	0	0	0	0	0	0	0	0	0	0	0	0	0	0
Pseudohelminthosporium_clematidis	0	0	0	0	0	0	0	0	0	0	0	0	0	0
Pseudomicrostroma_phylloplanum	0	0	0	0	0	0	0	0	0	0	0	0	0	0
Pseudopithomyces_angolensis	0	0	0	0	0	0	0	0	0	0	0	0	0	0
Pseudopithomyces_karoo	0	0	0	0	0	0	0	0	0	0	0	0	0	0
Pseudoramichloridium_henryi	0	0	0	0	0	0	0	0	0	0	0	0	0	0
Pseudorobillarda_sojae	0	0	0	0	0	0	0	0	0	0	0	0	0	0
Pseudoveronaea_ellipsoidea	0	0	0	0	0	0	0	0	0	0	0	0	0	0
Puccinia_recondita	0	0	0	0	0	0	0	0	0	0	0	0	0	0
Puccinia_striiformis	0	0	0	0	0	0	0	0	0	0	0	0	0	0
Punctularia_atropurpurascens	0	0	0	0	0	0	0	0	0	0	0	0	0	0
Purpureocillium_lilacinum	0	0	0	0	0	0	0	0	0	0	0	0	0	0
Pyrenochaeta_spe_Incertae_sedis	0	0	0	0	0	0	0	0	0	0	0	0	0	0
Pyrenochaetopsis_leptospora	0	0	0	0	0	0	0	0	0	0	0	0	0	0
Pyrenochaetopsis_microspora	0	0	0	0	0	0	0	0	0	0	0	0	0	0
Pyricularia_oryzae	0	0	0	0	0	0	0	0	0	0	0	0	0	0
Quambalaria_cyanescens	0	0	0	0	0	0	0	0	0	0	0	0	0	0
Rachicladosporium_spe_Incertae_sedi	0	0	0	0	0	0	0	0	0	0	0	0	0	0
Radulomyces_paumanokensis	0	0	0	0	0	0	0	0	0	0	0	0	0	0
Raffaelea_albimanens	0	0	0	0	0	0	0	0	0	0	0	0	0	0
Raffaelea_lauricola	0	396	0	0	40	45	88	0	0	61	180	38	159873	55
Ramophialophora_spe_Incertae_sedis	0	0	0	0	0	0	0	0	0	0	0	0	0	0
Ramularia_coryli	0	0	0	0	0	0	0	0	0	0	0	0	4516	0
Recurvomyces_spe_Incertae_sedis	0	0	0	0	0	0	0	0	0	0	0	0	0	0
Resupinatus_hausknechtii	112	0	0	0	0	0	0	0	0	0	0	0	0	0
Rhexothecium_globosum	0	0	0	0	0	0	0	0	0	0	0	0	0	0
Rhizoctonia_spe_Incertae_sedis	2378	0	0	0	0	0	0	0	0	0	0	0	0	0
Rhizopogon_laricinus	0	0	0	0	0	0	0	0	0	0	0	0	0	0
Rhizopus_arrhizus	0	0	0	0	0	0	0	0	0	0	0	0	0	0
Rhizopus_microsporus	0	0	0	0	85	88	0	0	0	0	0	0	0	0
Rhodosporidiobolus_colostri	0	0	0	0	0	0	0	0	0	0	0	0	0	0
Rhodotorula_diobovata	2261	0	0	0	0	0	0	0	0	0	0	0	0	0
Rhodotorula_glutinis	0	0	0	0	0	0	0	0	0	0	0	0	0	0
Rhodotorula_ingeniosa	0	0	0	0	0	0	0	0	0	0	0	0	0	0
Rhodotorula_mucilaginosa	16359	48566	0	65957	93549	813	2668	126	3431	20399	202113	5951	28764	81
Rhopalophora_clavispora	0	0	0	0	0	0	0	0	0	0	0	0	0	0
Robbauera_albescens	0	0	0	0	0	0	0	0	0	0	0	0	0	0
Rosellinia_australiensis	0	10030	0	0	0	0	0	0	0	0	0	0	0	35
Roussoella_neopustulans	0	0	0	0	0	0	0	0	0	0	0	0	0	0
Roussoella_solani	0	0	0	0	0	0	0	0	0	0	0	0	0	0
Rozellomycota_spe_Incertae_sedis	0	0	0	0	0	0	39	0	0	0	0	0	0	250
Ruhlandiella_patagonica	807	0	0	0	0	196	47	0	0	0	0	85	0	275
Russula_xerophila	0	0	257	0	0	0	0	0	0	0	0	0	0	0
Russulales_spe_Incertae_sedis	0	0	0	0	0	0	0	0	0	0	0	0	0	0
Saccharomyces_cerevisiae	0	0	0	0	14414	0	47	0	757	0	96	0	61	217
Saccharomycopsis_fibuligera	0	0	0	0	0	0	0	0	0	0	0	0	0	0
Saprochaete_gigas	0	0	0	0	0	0	0	0	0	0	0	0	0	0
Sarocladium_kiliense	0	0	0	0	0	0	0	0	0	0	0	0	0	0
Sarocladium_oryzae	0	0	0	0	0	0	0	0	0	0	0	0	0	0
Sarocladium_strictum	106	1922	2384	683	0	0	0	0	39	0	0	0	0	0
Sarocladium_subulatum	0	0	0	0	0	0	0	0	0	0	0	0	0	0
Sarocladium_zeae	0	0	0	0	0	0	0	0	0	0	0	0	0	0
Schizophyllum_commune	0	0	0	0	0	0	0	7592	30	1867	0	0	0	0
Schizosaccharomyces_octosporus	0	0	0	0	0	0	0	0	0	0	0	0	0	0
Scopulariopsis_cordiae	0	0	0	0	0	0	0	0	0	0	0	0	0	0
Scopuloides_rimosa	0	0	0	0	0	0	0	0	0	0	0	0	0	0
Septobasidium_spe_Incertae_sedis	0	0	0	0	0	0	0	0	0	0	0	0	0	0
Septoria_spe_Incertae_sedis	0	0	0	0	0	0	0	0	0	0	0	0	0	0
Setophaeosphaeria_badalingensis	0	0	0	0	0	0	0	0	0	0	0	0	0	0
Setophoma_vernoniae	0	0	0	0	0	0	0	7432	0	0	0	0	0	0
Sidera_vesiculosa	0	0	0	0	0	0	0	0	0	0	0	0	0	0
Simplicillium_aogashimaense	0	0	0	0	0	0	0	0	0	0	0	0	0	0
Simplicillium_lanosoniveum	0	0	0	0	0	0	0	0	51	0	34829	0	0	0
Simplicillium_spe_Incertae_sedis	0	0	0	0	0	0	0	0	0	0	0	0	0	0
Simplicillium_sympodiophorum	0	403	0	49	0	0	173	0	0	0	0	0	0	239
Sistotrema_brinkmannii	0	0	0	0	0	0	0	0	0	0	0	0	0	0
Sistotremastrum_guttuliferum	0	0	0	0	0	0	0	0	0	0	0	4652	0	0
Skeletocutis_nivea	0	0	0	0	0	0	0	0	0	0	0	0	0	0
Sordaria_equicola	0	0	0	0	0	0	0	0	0	0	0	0	0	0

Sordaria_spe_Incertae_sedis	0	0	0	0	0	0	0	0	0	0	0	0	0	0
Sphaerulina_westendorpii	0	0	0	0	0	0	0	0	0	0	0	0	0	0
Spiromastix_princeps	0	0	0	0	0	0	0	0	0	0	0	0	0	0
Sporobolomyces_beijingensis	518	0	0	7967	0	0	151	38	0	60	0	53	14961	50707
Sporobolomyces_koalae	0	0	0	0	0	0	0	0	0	0	0	0	0	0
Sporobolomyces_patagonicus	0	0	0	0	0	0	0	0	0	0	0	0	0	0
Sporobolomyces_phaffii	0	0	0	0	0	0	0	0	0	0	0	0	0	0
Sporobolomyces_roseus	0	0	0	0	0	0	0	0	0	0	0	0	0	0
Sporothrix_nigrograna	0	0	0	0	0	0	0	0	0	0	0	0	0	0
Stachybotrys_aloeticola	0	0	0	0	0	0	0	0	0	0	0	0	0	0
Stagonosporopsis_citrulli	0	0	0	0	0	0	0	0	0	0	0	0	0	0
Stagonosporopsis_lupini	0	0	0	0	0	0	0	0	0	0	0	0	0	0
Stagonosporopsis_nemophilae	0	0	0	0	0	0	0	0	0	0	0	0	0	0
Starmerella_etchellsii	0	0	0	0	0	0	0	0	0	0	0	0	0	0
Steccherinum_ochraceum	0	0	0	0	0	0	0	0	0	0	0	0	0	0
Stemphylium_lycopersici	0	0	0	0	0	0	0	0	0	0	0	0	0	0
Stemphylium_spe_Incertae_sedis	0	0	0	0	0	0	0	0	0	0	0	0	0	0
Stephanosporaceae_spe_Incertae_sedi	0	0	0	0	0	0	0	0	0	0	0	0	0	0
Sterigmatomyces_halophilus	0	0	0	0	0	0	0	0	0	0	0	0	0	0
Strelitziana_eucalypti	0	0	0	0	0	0	0	20579	0	0	0	0	0	0
Striatibotrys_yuccae	0	0	0	0	0	0	0	0	0	0	0	0	0	0
Strobilurus_spe_Incertae_sedis	0	0	0	0	0	0	0	0	0	0	0	0	0	0
Subulicystidium_brachysporum	0	0	0	0	0	0	0	0	0	0	0	0	0	0
Symmetrospora_marina	0	0	0	0	0	0	0	0	0	0	0	0	0	0
Symmetrospora_oryzicola	0	0	0	0	0	0	0	0	0	0	0	0	0	0
Symmetrospora_spe_Incertae_sedis	0	0	0	0	0	0	0	0	0	0	0	0	0	0
Sympodiomycopsis_spe_Incertae_sedi	0	0	0	0	0	0	0	0	0	0	0	0	0	0
Talaromyces_calidicanius	0	0	0	0	0	0	0	0	0	0	0	0	0	0
Talaromyces_funiculosus	0	0	0	0	0	0	0	0	0	0	0	0	0	0
Talaromyces_islandicus	0	0	0	0	0	0	0	0	0	0	0	0	0	0
Talaromyces_rogersiae	0	0	0	0	0	0	0	0	0	0	0	0	0	0
Talaromyces_scorteus	0	0	160692	35	0	0	0	0	139	0	0	0	0	0
Talaromyces_stollii	0	0	0	0	0	0	0	0	0	0	0	0	0	0
Talaromyces_udagawae	0	0	0	0	0	0	0	0	0	0	0	0	0	0
Talaromyces_wortmannii	0	0	0	0	0	0	0	0	0	0	0	0	0	0
Taphrina_padi	0	0	0	0	0	0	0	0	0	0	0	189	0	0
Tausonia_pullulans	0	0	0	0	0	135	0	0	0	0	0	0	0	0
Tetracladium_spe_Incertae_sedis	0	0	0	0	0	0	0	0	0	0	0	0	0	0
Tetrapisispora_blattae	0	0	0	0	0	0	0	0	0	0	0	0	0	0
Thanatephorus_cucumeris	0	0	0	0	0	0	0	0	0	0	0	0	0	0
Thecaphora_australiensis	0	0	0	0	0	0	0	0	0	0	0	0	0	0
Thelebolus_balaustiformis	0	0	0	0	0	0	0	0	0	0	0	0	0	0
Thermomyces_lanuginosus	0	0	0	0	0	0	0	0	0	0	0	0	0	0
Thielaviopsis_euricoi	0	0	0	0	0	0	0	0	0	0	0	0	0	0
Tilletia_barclayana	0	0	0	0	0	0	0	0	0	0	0	0	0	0
Tilletia_geeringii	0	0	0	0	0	0	0	0	0	0	0	0	0	0
Tilletia_maclaganii	0	0	0	0	0	0	0	0	0	0	0	0	0	0
Tilletia_mactaggartii	0	0	0	0	0	0	0	0	0	0	0	0	0	0
Tilletiopsis_washingtonensis	0	0	0	0	29348	0	0	41	0	0	0	0	0	0
Togniniella_acerosa	0	0	0	0	0	0	0	0	0	0	0	0	0	0
Torula_fici	0	0	0	0	0	0	0	0	0	0	0	0	0	0
Toxicocladosporium_spe_Incertae_sed	0	0	0	0	0	0	0	0	0	1907	0	0	0	0
Toxicocladosporium_strelitziae	0	0	0	0	0	0	0	0	0	0	0	0	0	0
Trametes_cubensis	0	0	0	0	0	0	0	0	0	0	0	0	0	0
Trametes_trogii	0	0	0	0	0	0	0	0	0	0	0	0	0	0
Trametes_versicolor	0	0	0	0	0	0	0	5273	0	0	0	0	0	0
Trechispora_spe_Incertae_sedis	0	0	0	0	0	0	0	0	0	0	0	0	0	0
Tremateia_chromolaenae	0	0	0	0	0	0	0	0	0	0	0	0	0	0
Tremateia_murispora	0	0	0	0	0	0	0	0	0	0	0	0	0	0
Trichoderma_asperellum	0	0	0	0	0	0	0	0	0	0	0	0	0	0
Trichoderma_deliquescens	0	0	0	0	0	0	0	0	0	0	0	0	0	0
Trichoderma_effusum	0	0	0	0	0	0	0	0	0	0	0	0	0	0
Trichoderma_harzianum	0	0	0	0	0	0	0	0	0	0	0	0	0	0
Trichoderma_longibrachiatum	0	0	0	5034	0	0	0	0	0	0	0	0	0	0
Trichoderma_pleuroticola	0	0	0	0	0	0	0	0	0	0	0	0	0	0
Trichomeriaceae_spe_Incertae_sedis	0	0	0	0	0	0	0	0	0	0	0	0	0	0
Trichomerium_dioscoreae	0	0	0	0	0	0	0	0	0	0	0	0	0	0
Trichomerium_flexuosum	0	0	0	0	0	0	0	0	0	0	0	0	0	0
Trichomonascus_ciferrii	0	0	0	0	0	0	0	0	0	0	0	0	0	0
Trichophaea_saccata	0	0	0	0	0	0	0	0	0	0	0	0	0	0
Trichosporon_asahii	0	0	0	0	43	0	17383	0	0	0	0	0	0	0
Trichosporonaceae_spe_Incertae_sedi	0	0	0	0	0	0	0	0	0	0	0	0	0	0
Trigonopsis_californica	0	0	0	0	0	0	0	0	0	0	0	0	0	0
Trigonopsis_vinaria	0	0	0	0	0	0	0	0	0	0	0	0	0	0
Tritirachium_cinnamomeum	0	0	0	0	0	0	0	0	0	0	0	0	0	0
Tritirachium_oryzae	0	0	0	0	0	0	0	0	0	0	0	0	0	0
Tuber_alcaracense	0	0	0	0	0	0	0	0	0	0	41	41	0	0
Tubeufia_tratensis	0	0	0	0	0	0	0	0	0	0	0	0	0	0
Tulostoma_calcareum	0	0	0	0	0	0	0	0	0	0	0	0	0	0
Typhula_micans	0	0	0	0	0	0	0	0	0	0	0	0	0	0
Typhula_setipes	0	0	0	0	0	0	0	0	0	0	0	0	0	0
Udeniomyces_megalosporus	0	0	0	0	37	559	125	127	90	143	262	71279	141	328400
Udeniomyces_pyricola	0	0	0	0	81	228	234	125	96	237	218	45	148357	85966
Umbilicaria_maculata	0	0	0	0	0	0	0	0	0	0	0	0	0	0
Umthunziomyces_hagahagensis	0	0	0	0	0	0	0	0	0	0	0	0	0	0
Ustilaginoidea_virens	942	6583	0	0	0	0	1544	38	0	0	0	20701	17261	5768
Uwebraunia_musae	0	0	0	0	0	0	0	0	0	0	0	0	0	0

Vacuiphoma_oculihominis	96	0	0	0	0	0	0	0	0	0	0	0	662	128
Valsaria_ceratoniae	0	0	0	0	0	0	0	0	0	0	0	0	0	0
Vandijckella_johannae	0	0	0	0	0	0	0	0	0	0	0	0	0	0
Vanrija_musci	0	0	0	0	0	0	0	0	0	1884	0	0	0	0
Vararia_gallica	0	0	0	0	0	0	0	0	0	0	0	0	0	0
Venturiaceae_spe_Incertae_sedis	0	0	0	0	0	0	0	0	0	0	0	0	0	0
Verrucaria_oulankaensis	0	0	0	0	0	0	0	0	0	0	0	0	0	0
Verrucaria_rosula	0	0	0	3369	0	0	0	0	0	0	0	0	0	0
Verrucaria_vitikainenii	0	0	0	0	0	0	0	0	0	0	0	0	0	0
Vishniacozyma_heimaeyensis	0	0	0	0	0	0	0	0	0	0	0	0	0	0
Vishniacozyma_taibaiensis	0	0	0	0	0	0	0	0	0	0	0	0	0	0
Vishniacozyma_tephrensis	0	0	0	0	0	0	0	0	0	0	0	0	0	0
Vishniacozyma_victoriae	0	0	0	0	0	0	0	0	0	0	0	0	0	0
Wallemia_canadensis	0	0	0	1899	0	45	0	0	0	0	0	0	0	0
Wallemia_mellicola	0	0	0	0	0	0	0	0	0	0	0	0	0	0
Wallemia_muriae	0	0	0	0	0	0	0	0	0	0	0	0	0	0
Wallemia_sebi	0	0	0	0	0	0	40	0	0	0	0	0	0	0
Wickerhamiella_versatilis	0	0	0	0	0	0	0	0	0	0	0	0	0	0
Wickerhamomyces_anomalus	0	0	0	0	0	0	0	0	0	0	0	0	0	0
Wickerhamomyces_onychis	0	0	0	0	0	0	0	0	0	0	0	0	0	0
Xenodevriesia_strelitziicola	0	0	0	0	0	0	0	0	0	0	0	0	0	0
Xeromyces_bisporus	0	0	0	0	0	0	0	0	0	0	0	0	0	0
Xylariaceae_spe_Incertae_sedis	0	0	0	0	0	0	1402	0	0	0	0	0	0	0
Xylocoremium_spe_Incertae_sedis	0	0	0	0	0	0	0	0	0	0	0	0	0	0
Xylodon_filicinus	0	0	0	0	0	0	0	0	0	0	0	0	0	0
Yamadazyma_terventina	0	0	0	0	0	0	0	0	0	0	0	0	0	0
Yamadazyma_triangularis	0	0	0	0	0	0	0	0	0	0	0	0	0	0
Yarrowia_alimentaria	0	0	0	0	0	0	0	0	0	0	0	0	0	0
Yarrowia_bubula	0	0	0	0	0	0	0	0	0	0	0	0	0	0
Yarrowia_lipolytica	0	0	0	0	0	0	0	0	0	0	0	0	0	0
Yarrowia_porcina	0	0	0	0	0	0	0	0	0	0	0	0	0	0
Zanclospora_aurea	0	0	0	0	0	0	0	0	0	0	0	0	0	0
Zanclospora_jonesii	562963	58372	815223	7380	34106	922995	85521	252970	52923	58194	133295	29291	12555	36315
Zasmidium_spe_Incertae_sedis	0	0	0	0	0	0	0	0	0	0	0	0	0	0
Zeloasperisporium_eucalyptorum	0	0	0	0	0	0	0	0	0	0	0	0	0	0
Zeloasperisporium_searsiae	0	0	0	0	0	0	0	0	0	0	0	0	0	0
Zoophthora_radicans	534	0	0	0	0	154	307	168	0	0	0	463	0	0
Zygoascus_polysorbophila	1166	0	0	0	0	481	0	0	0	0	0	0	0	0
Zygosaccharomyces_rouxii	0	0	0	0	0	0	0	0	0	0	0	0	0	0
Zygosporium_pseudogibbum	0	0	0	0	0	0	0	0	0	0	0	0	0	0
Zygotorulaspora_danielsina	0	0	0	0	0	0	0	0	0	0	0	0	0	0
Zymoseptoria_spe_Incertae_sedis	0	0	0	0	0	0	0	0	0	0	0	0	0	0
[Candida]_aaseri	0	0	0	0	0	0	0	0	0	0	0	0	0	0
[Candida]_boidinii	0	0	0	0	0	0	0	0	0	0	0	0	0	0
[Candida]_glabrata	0	0	0	0	0	0	0	0	0	0	0	0	0	0
[Candida]_incommunis	0	0	0	0	0	0	0	0	0	0	0	0	0	0
[Candida]_pseudointermedia	0	0	0	0	0	0	0	0	0	0	0	0	0	0
[Candida]_pseudolambica	0	0	0	0	0	0	0	0	0	0	0	0	0	0
[Cryptococcus]_ater	0	0	0	784	319	0	133	205	104	0	0	105	0	0

Post-COVID-19-002	Post-COVID-19-004	Post-COVID-19-005	Post-COVID-19-003	Post-COVID-19-007	Post-COVID-19-008	Post-COVID-19-009	Post-COVID-19-006	COVID-19-051	COVID-19-046	COVID-19-050	COVID-19-049	COVID-19-048	COVID-19-047	COVID-19-045	COVID-19-040	COVID-19-043	COVID-19-042
488005	624921	832333	153553	993811	209413	134895	135395	942693	986296	825612	966440	943153	989479	980748	994967	668622	994045
504104	234485	27322	15987	1643	149000	792636	4132	33863	5577	113111	2164	24605	8352	5810	4172	293749	5472
4980	0	0	0	50	0	0	0	134	0	73	0	0	0	0	0	0	0
990	13116	28027	249	408	1080	1799	4112	16017	7167	9201	31141	28418	1519	10886	861	27652	343
1921	125473	110726	830211	4088	640066	70670	856252	7293	960	52003	255	3652	650	2556	0	9977	140
0	0	0	0	0	441	0	0	0	0	0	0	0	0	0	0	0	0
0	2005	1592	0	0	0	0	109	0	0	0	0	172	0	0	0	0	0

Post-COVID-19-002	Post-COVID-19-004	Post-COVID-19-005	Post-COVID-19-003	Post-COVID-19-007	Post-COVID-19-008	Post-COVID-19-009	Post-COVID-19-006	COVID-19-051	COVID-19-046	COVID-19-050	COVID-19-049	COVID-19-048	COVID-19-047	COVID-19-045	COVID-19-040	COVID-19-043	COVID-19-042
0	0	0	0	0	0	0	0	0	69	0	0	178	0	0	0	0	0
0	0	0	0	0	0	0	0	0	0	0	0	0	0	0	0	0	0
3210	4438	2460	2577	357	1782	14991	5213	81273	248825	576922	3634	748633	2430	32996	19194	149984	7016
0	0	0	0	0	0	0	0	0	0	0	0	0	0	0	0	0	0
1921	117887	92813	779220	4039	624216	65492	822473	0	0	0	0	0	0	0	0	0	0
0	0	0	0	0	0	0	0	0	0	0	0	0	0	0	0	0	0
0	0	0	0	0	0	0	0	0	0	0	0	0	0	0	0	0	0
0	0	0	0	0	0	0	0	0	0	0	0	0	0	0	0	0	0
0	0	0	0	0	0	0	0	0	123	0	0	163	0	0	0	0	0
0	0	0	0	0	0	0	0	0	0	0	0	0	0	0	0	0	0
0	0	0	0	0	0	0	0	0	0	0	0	0	0	0	0	2712	0
135141	2645	1665	65	0	0	0	523	384	212	5434	0	518	0	315	301	23156	66
0	0	0	0	0	0	0	0	0	0	0	0	0	0	0	0	0	0
0	0	0	0	0	0	0	0	0	0	0	0	0	239	0	0	0	0
0	0	0	0	0	0	0	0	0	0	0	0	0	0	0	0	0	0
0	2390	908	0	0	0	0	100	73	4805	1112	317	15450	0	965	507	5279	0
0	0	0	0	0	0	0	0	0	0	0	0	0	0	0	0	0	0
0	0	0	0	0	0	0	0	0	0	0	0	0	0	0	0	0	0
0	0	419	0	0	0	0	0	0	0	0	0	0	0	0	0	0	0
0	0	0	0	0	0	0	0	0	0	0	0	0	0	0	0	139	0
0	0	0	0	0	0	0	0	0	0	0	0	0	358	0	110	0	1391
0	75	0	0	0	0	0	0	0	0	0	0	0	0	0	0	0	0
0	0	0	0	0	0	0	0	0	0	0	0	0	0	0	0	0	0
0	0	0	0	0	0	0	0	0	0	0	0	0	0	0	0	0	0
0	0	75	0	0	0	56	0	0	0	0	0	0	66	0	0	312	0
0	1752	945	0	0	0	0	0	0	0	0	0	88	0	0	0	0	0
0	0	0	0	0	0	0	0	1741	0	0	0	93	0	0	0	0	0
0	0	0	0	0	0	0	0	0	0	0	0	0	0	0	0	0	0
0	0	0	0	0	0	0	0	0	0	0	0	0	0	346	0	0	0
125904	8409	2258	58800	223	8793	13678	1980	347412	2482	69731	719	6222	96898	730229	1845	9522	3585
0	0	0	0	0	0	0	0	0	0	0	0	0	0	0	0	0	0
0	0	0	0	0	16870	0	0	0	52	0	0	0	0	0	0	0	188
0	0	0	0	0	0	0	0	0	0	0	0	0	0	0	0	0	0
0	0	0	0	0	0	0	0	0	0	0	0	0	0	0	0	0	0
0	0	0	0	0	0	0	0	0	0	0	0	0	0	0	0	0	0
43	0	0	259	0	0	0	0	42	0	0	0	101	0	0	0	0	0
0	0	0	0	0	0	0	0	0	0	0	0	0	0	0	0	0	0
0	0	0	0	0	0	0	0	0	0	0	0	0	0	0	0	0	0
0	0	0	0	0	0	0	0	0	0	0	0	0	0	0	0	0	0
0	0	0	0	0	0	0	0	0	0	0	0	0	0	0	0	0	0
0	0	0	0	0	0	67	0	0	275	0	0	0	0	0	0	0	0
0	0	0	0	0	0	0	0	0	0	0	0	0	0	0	0	0	0
0	0	0	0	0	0	0	0	0	0	0	0	0	306	0	0	0	915
4980	0	0	0	50	0	0	0	0	0	0	0	0	0	0	0	0	0
0	0	0	0	0	0	0	0	0	0	0	0	0	0	0	0	0	0
0	0	0	0	0	0	0	0	0	0	255	130	0	0	0	0	0	0
0	0	0	0	0	0	0	0	101	0	51	0	0	0	0	0	0	0
0	0	0	0	0	84	0	218	460603	101	42213	342	437	130	3220	131	289	59
0	16525	0	7170	0	19288	5282	448	0	0	0	0	83	0	0	0	4790	0
0	0	0	0	0	43	0	0	0	0	0	0	0	0	0	0	0	0
0	0	0	0	0	0	0	0	0	0	0	0	100	0	0	0	0	0
0	0	0	0	0	0	46	0	0	0	0	0	0	0	0	0	0	0
0	0	0	0	0	0	0	0	0	0	0	0	0	0	0	0	0	0
0	0	0	0	0	0	84	0	0	0	0	0	88	0	0	0	19140	0
0	0	0	0	0	0	0	0	0	0	0	0	0	0	0	0	0	0
0	0	0	8419	0	0	0	0	0	0	0	0	0	0	0	0	0	0
0	1613	0	0	0	0	0	0	0	0	0	0	0	0	0	0	0	0
0	0	0	0	0	0	0	0	0	485	73	0	1908	0	0	119	141	0
0	0	0	0	0	0	0	0	0	0	0	0	0	0	0	0	0	0
0	0	0	0	0	0	0	0	0	0	0	0	0	0	0	0	0	0
246	3820	2189	2379	979254	84	156	1185	4997	695128	37228	11647	1926	1648	10859	642185	7128	2261
0	0	0	0	0	0	0	0	0	0	0	0	0	0	0	0	0	0
0	0	0	0	0	0	0	0	0	0	0	0	0	0	0	0	0	0
0	0	0	0	0	0	0	0	0	0	0	0	0	0	0	0	1402	0
0	0	0	0	0	0	0	0	0	0	0	0	0	0	0	0	0	0
0	0	0	0	0	0	0	0	0	0	0	0	0	0	0	0	0	0
0	0	0	0	0	0	0	0	0	0	0	0	0	0	0	0	0	0
0	0	0	0	0	0	0	0	0	0	0	0	0	0	0	0	0	0
0	0	0	0	0	0	0	0	0	0	0	0	0	0	0	0	0	0

0	0	0	0	0	0	0	0	0	0	0	0	0	0	0	0	0	0
0	0	0	0	0	0	0	0	0	0	0	0	0	0	0	0	0	0
0	0	0	0	0	0	0	0	0	0	0	0	0	0	0	0	0	0
0	0	0	0	0	0	0	0	0	0	0	0	0	0	0	0	0	0
0	0	0	0	0	0	0	52	0	0	0	0	0	0	0	0	0	0
0	0	0	0	0	0	0	0	0	0	0	0	0	0	0	0	0	0
0	0	0	0	0	0	0	0	0	330	492	0	499	0	2202	1035	586	209
0	0	0	0	0	0	0	0	0	0	0	0	0	0	0	0	0	0
0	0	0	0	0	0	0	0	0	0	0	0	0	0	0	0	0	0
0	0	0	0	0	0	0	0	0	0	0	0	0	0	0	0	0	0
0	0	0	0	0	0	0	0	0	0	0	0	0	0	0	0	0	0
0	0	0	0	0	0	0	0	0	0	0	0	0	0	0	0	0	0
27073	16515	2112	30324	215	47	9091	2844	812	425	1847	598	1033	24335	2852	1028	144719	1947
0	0	0	0	54	0	0	0	0	0	60	101	0	0	0	0	0	0
0	0	0	0	0	0	0	0	0	0	132	0	0	0	0	0	0	0
0	0	0	0	0	0	0	0	0	0	0	0	0	0	0	0	0	0
0	0	0	0	0	0	0	0	0	0	0	0	0	0	0	0	0	0
0	0	0	0	0	0	0	0	0	0	0	0	0	0	0	0	0	0
0	0	0	0	0	0	0	0	0	0	0	0	0	0	0	0	0	0
0	0	0	0	0	0	107	0	0	0	0	0	0	699	0	0	0	0
0	0	0	0	0	0	0	0	0	0	0	0	75	0	0	0	0	0
3312	8704	572	0	0	0	0	0	0	0	0	0	0	0	0	0	0	0
0	0	0	0	0	0	0	0	0	57	0	0	0	0	0	125	0	0
0	0	0	0	0	0	0	0	0	0	0	0	0	0	0	0	0	0
0	0	0	0	0	0	0	0	0	65	0	0	73	0	0	0	0	0
0	0	0	0	0	0	0	0	0	0	0	0	0	0	0	0	0	0
0	0	0	0	0	0	0	0	0	0	63	0	256	0	0	151	0	0
0	9203	0	0	0	0	0	0	0	0	0	0	0	0	0	0	0	0
0	0	0	0	0	0	0	0	0	0	261	0	0	0	0	0	0	0
14397	0	0	63	265	0	0	0	0	0	0	0	0	0	0	0	0	0
0	0	0	0	0	0	0	0	0	0	0	0	0	0	0	0	0	0
0	0	0	0	0	0	0	0	0	0	0	0	0	0	0	0	0	0
0	0	0	0	0	0	0	0	0	0	0	0	0	0	0	0	0	0
0	0	0	200	0	0	0	0	0	64	71	0	0	0	0	0	3792	0
0	0	0	0	0	0	0	0	0	0	0	0	0	0	0	0	0	0
0	0	0	0	192	0	0	0	0	0	0	0	0	0	0	0	0	0
0	0	0	0	0	0	0	0	0	0	0	0	0	0	0	0	0	0
0	0	0	0	0	0	0	508	0	0	131	0	0	0	0	0	0	0
0	0	0	0	0	0	0	0	0	0	0	0	0	0	0	0	0	0
0	0	0	0	0	0	0	0	0	0	0	0	0	0	0	0	0	0
0	0	0	0	0	0	0	0	0	0	0	0	0	0	0	0	0	0
0	0	0	0	0	0	0	0	0	0	0	0	0	0	0	0	0	0
0	0	0	0	0	0	0	0	493	0	0	0	0	0	0	0	0	0
0	10243	0	0	0	0	0	0	0	0	0	0	0	0	0	0	0	0
0	0	0	0	0	0	0	0	0	0	0	0	0	0	0	0	0	129
0	0	0	0	0	0	0	0	0	0	0	0	0	0	0	0	0	0
152250	291	174	60	88	0	3779	60	0	0	0	0	0	0	633	0	5861	0
95655	64	0	137	5855	709	0	68	0	0	88	0	0	0	0	240	0	0
0	0	0	0	0	0	0	0	0	0	0	0	0	0	0	0	0	0
0	2100	2385	0	0	0	666	19024	7293	960	51879	255	3652	650	2556	0	9977	140
0	0	0	0	0	0	0	0	0	0	0	0	0	0	0	0	0	0
0	0	0	0	0	0	0	0	0	0	0	0	0	0	0	0	0	0
0	0	0	0	0	0	0	0	0	0	0	0	0	0	0	0	0	0
0	0	0	0	0	0	0	0	0	0	0	0	0	0	0	0	0	0
0	0	0	0	0	0	0	0	0	0	0	0	0	0	0	0	0	0
0	0	0	0	0	0	0	0	0	0	0	0	0	0	0	0	0	0
0	0	0	0	0	0	0	0	0	0	0	0	0	0	0	0	0	0
0	0	0	0	0	0	0	0	0	0	0	0	0	0	0	0	0	0
0	0	0	0	0	0	0	0	0	0	0	0	0	0	0	0	0	0
0	0	0	0	0	0	0	0	0	0	0	0	0	0	0	0	0	0
0	0	0	0	0	0	0	0	0	0	0	0	0	0	0	0	0	0
0	296	859	0	0	0	0	0	463	176	617	0	276	70	0	0	394	0
0	0	0	0	0	0	0	0	0	0	0	0	0	0	0	0	0	0
0	0	0	0	0	0	0	0	0	0	0	0	0	0	0	0	0	0
0	0	0	0	0	0	0	0	0	0	0	0	0	0	0	0	0	0
0	0	989	0	0	0	0	0	0	0	0	0	0	0	0	0	0	0
0	0	0	0	0	0	0	0	0	0	0	0	0	0	0	0	0	0
0	0	0	0	0	0	0	0	0	0	0	0	0	0	0	0	0	0
0	0	0	0	0	0	0	0	0	0	0	0	0	0	0	0	0	0
0	0	0	0	0	0	0	0	0	0	0	0	0	0	0	0	0	0
0	0	0	0	0	0	0	0	0	0	0	0	0	0	0	0	0	0
0	0	0	888	0	0	0	0	0	0	0	0	0	0	0	0	0	0
0	0	0	0	0	0	0	0	0	0	0	0	0	0	0	0	0	0
0	0	0	0	0	0	50	0	0	3269	769	115	11451	0	663	404	6925	136
0	0	0	0	42	0	0	0	121	4205	130	229	19601	2412	1290	993	2094	1462
0	0	1022	70	0	47	0	0	0	0	0	0	0	0	329	134	13620	0
0	0	0	0	0	0	0	0	0	0	0	0	0	0	0	0	0	0
0	0	0	0	0	0	0	0	0	0	0	0	0	0	0	0	0	0
0	0	0	0	0	0	0	0	0	50	0	0	0	62	0	0	0	0
0	0	0	0	0	0	0	0	0	0	0	0	0	0	0	0	0	0
0	0	0	0	0	0	0	0	0	0	0	0	0	0	0	0	0	0
0	0	0	0	0	0	0	0	0	0	0	0	0	0	0	0	0	0
990	12820	27168	249	408	1080	1799	4112	15554	6991	8584	31141	28142	1449	10886	861	27258	343
60	17045	1713	1634	223	2373	0	12308	0	235	2055	0	82	0	0	0	48	0
0	0	0	0	0	0	0	0	869	0	79	0	0	0	0	0	0	0
0	0	0	0	0	0	0	0	0	0	0	0	0	0	0	0	0	0

0	0	0	0	0	0	0	0	0	0	0	0	0	0	0	0	0	0
56602	33972	3792	18154	4512	0	1234	6123	0	0	0	440	0	0	0	0	0	0
0	0	0	0	0	0	0	0	0	0	0	0	0	0	0	0	0	0
0	0	0	0	0	0	0	0	0	0	0	0	0	0	0	0	252	0
0	3079	0	0	0	0	0	0	0	0	0	0	0	0	0	0	0	0
0	0	154	0	0	0	0	0	0	0	0	0	0	0	0	0	0	0
0	0	31348	0	0	0	0	0	0	0	0	0	0	0	0	0	0	0
19882	0	0	3432	0	0	10706	418	50	176	0	0	248	1291	1295	144	2565	194
0	0	0	0	0	0	0	0	0	55	0	0	81	0	0	0	0	0
0	0	0	0	0	0	0	0	0	0	0	0	0	0	0	0	0	0
0	0	0	0	0	0	0	0	0	0	0	0	0	0	0	0	0	0
0	0	0	0	0	0	0	115	0	0	124	0	0	0	0	0	0	0
0	0	0	0	0	0	0	0	0	0	0	0	0	0	0	0	0	0
0	0	0	0	0	0	0	0	0	0	0	0	0	0	0	0	0	0
0	0	0	0	0	0	0	0	0	0	0	0	0	0	0	0	0	0
0	0	0	0	0	0	0	0	0	0	0	0	0	0	0	0	0	0
0	0	0	0	0	0	0	0	0	0	0	0	0	0	0	0	0	0
0	0	0	0	0	0	0	0	0	0	0	0	0	0	0	0	0	0
0	0	0	0	0	0	0	0	0	0	0	0	0	0	0	0	0	0
0	0	0	0	0	0	0	0	0	0	0	0	0	0	0	0	0	0
0	0	0	0	0	0	0	381	0	0	0	0	94	0	0	136	7153	80
0	425	0	0	0	0	0	0	0	0	0	0	0	0	0	0	0	0
0	0	0	0	0	0	0	0	0	0	265	0	0	0	0	0	0	0
0	0	0	0	0	0	0	54	0	374	714	0	444	0	0	0	0	0
0	0	0	0	0	0	0	0	0	0	0	0	0	0	0	0	0	0
0	0	0	0	0	0	0	0	0	0	0	0	0	0	0	0	0	0
0	0	0	0	0	0	0	0	0	0	0	0	0	0	0	0	0	0
0	0	0	0	0	0	0	0	0	0	0	0	0	0	0	0	0	0
0	0	0	0	0	43	0	0	0	0	0	0	0	0	0	0	0	0
0	0	0	0	0	0	0	0	0	53	0	0	0	0	0	139	7941	0
0	0	0	0	0	0	0	0	0	0	0	0	0	0	0	0	0	0
0	0	0	0	0	0	0	0	0	0	0	0	0	0	0	0	0	0
0	0	0	0	0	0	0	0	0	0	0	0	0	0	0	0	0	0
0	0	0	0	0	0	0	0	359	0	0	0	0	0	0	0	0	0
0	0	0	0	0	0	0	0	0	0	0	0	0	0	0	0	0	0
0	0	0	0	0	0	324	0	170	0	65	0	257	3152	0	610	1167	3719
0	0	0	0	0	0	0	0	0	0	0	0	0	0	0	0	0	0
0	0	0	0	0	0	0	0	0	0	0	0	0	0	0	0	0	0
0	0	0	0	0	0	0	0	0	0	0	0	0	0	0	0	0	0
0	0	0	0	0	0	0	0	0	0	0	0	0	0	0	0	0	0
0	0	0	0	0	0	47	1568	0	0	0	0	0	0	0	0	0	0
0	0	0	0	0	0	0	0	0	0	0	0	0	0	0	0	0	0
0	0	0	0	0	0	0	0	0	0	0	0	0	0	0	0	0	0
0	8322	6060	0	0	46	0	0	0	0	0	0	0	0	0	0	164	0
0	0	0	0	0	0	0	0	0	0	0	0	0	0	0	0	688	0
0	6828	1394	0	0	0	0	0	0	0	0	0	0	0	0	0	0	0
0	0	0	0	0	0	0	0	0	0	0	0	0	57	0	0	0	0
0	0	0	0	0	0	0	0	0	0	0	0	0	0	0	0	0	0
6750	0	731	0	0	0	7908	0	0	51	61	0	171	0	0	0	0	0
0	0	0	0	0	0	0	0	0	0	0	0	0	0	0	0	0	0
0	0	0	0	0	0	0	0	0	0	0	0	0	0	0	0	382	0
0	0	0	0	0	0	0	0	0	0	0	0	0	0	0	0	0	0
0	0	0	0	0	0	0	0	0	0	0	0	0	0	0	0	0	0
0	0	0	0	0	0	0	0	0	0	0	0	0	0	0	0	0	0
0	0	0	0	0	0	0	0	0	0	0	111	0	0	0	0	0	0
0	0	0	0	0	0	0	0	0	0	0	0	0	0	0	0	0	0
0	0	0	0	0	0	0	0	0	0	0	0	0	0	0	0	0	0
0	0	0	0	0	0	0	0	0	0	0	0	0	0	0	0	0	0
0	0	0	0	0	0	0	0	0	0	0	0	0	0	0	0	0	0
0	0	0	0	0	0	49701	0	0	0	0	0	0	0	0	0	0	0
0	0	0	0	0	0	0	0	0	0	0	0	0	0	0	0	0	0
0	0	0	0	0	0	0	0	0	0	0	0	0	0	0	0	0	0
0	4827	374	0	0	0	0	0	0	0	0	0	0	0	0	0	0	0
0	0	0	0	0	0	0	0	0	0	0	0	0	0	0	0	0	0
0	0	0	0	0	0	0	0	0	0	0	0	0	0	0	0	0	0
0	0	0	0	0	0	0	430	0	0	0	0	0	0	0	249	104	0
0	0	0	0	0	81783	42	0	0	0	0	0	0	0	0	0	0	0
0	0	0	0	0	0	0	0	0	232	0	0	332	0	972	617	112894	287
0	0	0	0	0	0	0	0	0	0	0	0	0	0	0	0	0	0
0	0	0	0	0	0	0	0	0	0	0	0	0	0	0	0	0	0
0	0	0	0	0	0	0	0	0	0	0	0	0	0	0	0	0	0
0	0	0	0	0	0	0	0	0	0	0	0	0	0	0	0	0	0
0	0	0	0	0	0	0	0	0	0	0	0	0	0	0	0	0	0
53345	83694	7740	13916	1028	125565	52429	1259	19534	56	1217	1190	261	2019	327	0	2414	811
0	0	0	0	0	0	0	0	0	0	0	0	0	0	0	0	0	0
0	0	0	0	0	0	0	0	0	0	0	0	0	0	0	0	0	0
0	0	0	0	0	0	0	0	0	0	0	0	0	0	0	0	0	0
0	0	0	0	0	6103	0	0	0	0	0	0	0	0	0	0	0	0
0	0	0	0	0	11826	0	0	0	171	0	0	933	0	0	0	42	0
0	0	0	0	121	0	0	0	0	0	0	0	0	0	0	132	384	0
0	0	0	0	0	0	0	0	134	0	73	0	0	0	0	0	0	0
0	0	0	0	0	0	0	0	0	0	0	0	0	0	0	0	0	0
0	0	0	0	0	0	0	0	0	0	0	0	0	0	0	0	636	0
0	5486	15528	50991	49	15850	4512	14640	0	0	0	0	0	0	0	0	0	0
0	0	0	0	0	0	0	0	0	0	0	0	0	0	0	0	0	0
0	0	0	0	0	0	0	0	0	0	349	0	0	376	0	0	1210	0
22408	2595	82	0	0	0	0	0	0	0	0	0	0	0	0	0	0	0

0	0	0	0	0	0	0	0	0	0	0	0	0	0	0	0	0	0
0	0	0	0	0	0	0	0	0	0	0	0	0	0	0	0	0	0
0	0	0	0	0	0	0	0	0	0	0	0	0	0	0	0	0	0
0	0	0	0	0	0	0	0	0	0	0	0	0	0	0	0	0	0
0	0	0	0	0	0	0	0	0	0	0	0	0	0	0	0	0	0
0	0	0	0	0	0	0	0	0	0	0	0	0	0	0	0	0	0
0	0	0	0	0	0	0	0	0	0	0	0	0	0	0	0	0	0
0	0	0	0	0	0	0	0	0	43	0	0	0	0	0	0	0	0
0	0	0	0	0	0	0	0	0	0	0	0	0	0	0	0	0	0
0	0	0	0	0	0	0	0	0	0	0	0	0	0	0	0	0	0
0	0	0	0	0	0	0	0	0	0	0	0	0	0	0	0	0	0
0	0	0	0	0	0	0	0	0	0	0	0	0	0	0	0	0	0
0	9040	2232	0	0	0	0	0	0	0	131	0	0	0	0	0	0	0
0	0	0	0	0	0	0	0	0	0	142	0	0	0	0	0	0	0
0	0	0	0	0	0	0	0	0	0	0	0	0	0	0	0	0	0
0	19702	1100	80	203	28409	0	0	0	0	207	0	0	0	0	0	2552	0
60	0	0	0	0	0	0	0	0	0	0	0	0	0	0	0	0	0
0	0	0	0	0	0	0	0	0	0	0	0	0	0	0	0	0	0
0	0	0	0	0	0	0	0	0	0	0	0	0	0	0	0	0	0
0	0	0	0	0	0	0	0	0	0	0	0	0	0	0	0	0	0
0	0	0	0	0	0	0	0	0	0	0	0	0	0	0	0	0	0
0	0	0	0	0	0	0	0	0	0	1010	0	0	0	0	0	0	0
0	0	0	0	0	0	0	0	0	227	0	0	273	0	0	0	0	74
0	0	0	0	0	0	0	0	0	0	0	0	0	0	0	0	0	0
0	0	0	0	0	0	0	0	0	0	0	0	0	0	0	0	0	0
0	0	0	0	0	0	0	0	0	0	142	0	0	0	0	0	0	0
0	0	0	0	0	0	0	0	0	0	0	0	0	0	0	0	0	0
93	0	277	0	0	489	96	0	267	3513	1026	1402	9537	3838	98644	369	1971	152
0	0	0	0	0	0	0	0	0	0	0	0	0	0	0	0	0	0
0	0	0	0	0	0	0	0	0	0	0	0	0	0	0	0	0	0
0	9623	0	0	0	0	0	0	0	0	0	0	0	0	0	0	0	0
0	0	0	0	0	0	0	0	0	0	0	0	0	0	0	0	0	0
0	0	0	0	0	0	0	0	0	0	0	0	0	0	0	0	0	0
0	0	0	0	0	0	0	0	0	0	0	0	0	0	0	0	0	0
0	0	0	0	0	0	0	0	6445	46	131	0	241	0	0	0	489	0
0	0	0	0	0	0	0	0	0	0	0	0	0	0	0	0	0	0
0	0	0	0	0	0	0	0	0	0	0	0	0	0	0	0	0	0
0	0	0	0	0	0	0	0	0	0	0	0	0	0	0	0	0	0
0	97525	7554	2136	0	0	0	0	99	0	87	0	0	65	0	0	2088	78
0	0	0	0	0	0	0	0	0	0	0	0	0	0	0	0	0	0
0	0	0	0	0	0	56	0	0	0	0	0	0	0	302	0	96	67
0	0	0	0	0	0	0	287	0	0	0	0	0	0	0	0	0	0
0	0	0	0	0	0	0	0	0	608	0	0	413	0	0	0	40	0
0	76	56	0	0	0	0	0	0	0	0	0	0	0	0	0	0	0
0	0	0	0	0	0	0	0	0	0	0	0	0	0	0	0	0	0
0	0	0	0	0	0	0	0	0	0	0	0	0	0	309	0	0	0
0	0	0	0	0	0	0	0	0	0	0	0	0	0	0	0	0	0
0	0	0	0	0	0	0	0	0	0	0	0	0	0	0	0	0	0
0	0	0	0	0	0	0	0	0	0	0	0	0	0	0	0	0	0
0	0	0	0	0	0	0	0	0	0	0	0	0	0	0	0	0	0
0	87	0	0	54	0	0	67	0	0	1272	0	186	0	0	134	4640	73
0	5358	0	0	0	0	0	0	0	0	0	0	0	0	0	0	0	0
0	0	0	0	0	0	0	0	0	0	0	0	0	0	0	0	0	0
0	0	0	0	0	0	0	0	0	0	0	0	0	0	0	0	0	0
0	0	0	0	0	0	0	0	0	0	0	0	0	0	0	0	0	0
0	0	0	0	0	0	0	0	0	0	0	0	0	0	0	0	0	0
0	0	0	0	0	0	0	0	0	0	0	0	0	0	0	0	0	0
0	8678	2428	0	0	0	0	3611	0	0	0	0	334	0	0	0	1300	0
0	0	0	0	0	0	0	0	0	0	0	0	0	0	0	0	0	0
0	0	0	0	0	0	0	0	0	0	0	0	0	0	0	0	0	0
0	0	0	0	0	0	0	0	0	0	0	124	78	0	0	244	8805	0
0	355	0	0	0	0	0	0	0	0	0	0	0	0	0	0	0	0
0	0	0	0	0	0	0	0	0	0	0	0	0	0	0	0	0	0
133	0	78	0	46	4627	1829	60	64	0	71	131	145	0	331	132	14967	212
0	0	0	1902	0	0	0	0	0	0	0	0	86	0	0	0	3749	0
0	0	0	0	0	0	0	0	0	0	0	0	0	0	0	0	0	0
0	0	0	0	0	0	0	0	0	0	0	0	0	0	0	0	0	0
0	0	0	0	0	0	0	0	0	0	0	0	0	0	0	0	0	0
0	0	0	0	0	0	0	0	0	0	0	0	0	0	0	0	0	0
0	0	0	0	0	0	0	0	0	0	0	0	0	0	0	0	0	0
0	0	0	0	0	0	0	0	0	0	0	0	0	0	0	0	0	0
0	0	0	0	0	0	0	0	0	0	0	0	0	0	0	0	0	0
0	0	0	0	0	0	0	0	0	0	0	0	0	0	0	0	0	0
0	0	0	0	0	0	0	0	0	0	0	0	0	0	0	0	0	0
0	0	0	0	0	0	0	0	0	0	0	0	0	0	0	0	0	0
0	0	0	0	0	0	0	0	0	0	0	0	0	0	0	0	0	0
0	0	0	0	0	0	0	0	0	57	0	0	0	0	0	0	54	0
0	0	0	0	0	0	0	0	0	0	0	0	0	0	0	0	0	0
0	0	0	0	0	0	0	0	0	0	0	0	0	0	0	0	0	0
0	0	0	0	0	0	0	0	0	0	0	0	0	0	0	0	0	0
0	0	0	0	0	0	0	0	0	0	0	0	0	0	0	0	0	0
0	0	0	0	0	0	0	0	0	0	0	0	0	0	0	0	0	0
0	492	0	0	0	0	0	0	0	0	0	0	0	0	0	0	0	0
0	0	0	0	0	0	0	0	0	0	0	0	0	0	0	0	0	0
0	0	0	0	0	0	0	0	0	0	0	0	0	0	0	0	0	0
0	0	0	0	0	0	0	0	0	0	0	0	0	0	0	0	0	0
0	0	1000	0	85	82	210	0	3764	980	332	0	23895	1355	1559	380	671	202

0	0	0	0	0	0	0	0	0	0	0	0	0	0	0	0	0	0
0	0	0	0	0	0	0	0	0	0	0	0	0	0	0	0	0	0
0	0	0	0	0	0	0	0	0	0	0	0	0	0	0	0	0	0
11900	0	0	0	0	0	0	0	0	0	0	0	0	0	0	0	0	0
0	0	0	0	0	0	0	0	3631	0	133	229	0	0	0	0	0	0
0	0	0	0	0	0	0	0	0	0	0	0	0	0	0	0	0	0
0	0	0	0	0	0	0	0	0	0	0	0	0	0	0	0	0	0
0	0	0	0	0	0	0	0	0	0	0	0	0	0	0	0	0	0
0	0	0	0	0	0	0	0	0	0	0	0	0	0	0	0	0	0
65724	92004	10235	0	0	18131	2905	1649	0	0	0	0	0	0	0	124	0	0
0	0	0	0	0	0	0	0	0	0	0	0	0	0	0	0	0	0
0	0	0	0	0	0	0	0	0	0	0	0	0	0	0	0	0	0
0	0	0	0	0	0	1694	0	0	0	0	0	0	0	0	0	0	0
0	0	0	0	0	0	0	0	0	0	0	0	0	0	0	0	0	0
0	0	0	0	0	441	0	0	0	0	0	0	0	0	0	0	0	0
0	2070	247	0	37	0	0	0	0	0	0	0	0	0	0	0	0	0
0	0	0	0	0	0	51	0	13783	60	107302	351	274	3122	980	1121	1037	2405
0	0	0	0	0	0	0	0	0	0	0	0	0	0	0	0	0	0
0	0	533	0	0	0	194	0	0	426	0	0	612	174	2538	652	545	69
0	0	0	0	0	0	0	0	0	0	0	0	0	0	0	0	0	0
0	0	0	0	0	0	0	0	0	0	0	0	0	0	0	0	0	0
0	0	0	0	0	0	0	0	0	0	0	0	0	356	0	0	348	0
0	11350	0	78	0	0	0	0	0	0	954	0	0	0	0	0	0	0
0	0	77	0	0	0	0	0	0	0	0	0	0	0	0	0	0	0
0	0	0	0	0	0	0	0	0	0	0	0	0	0	0	0	192	0
0	0	0	0	0	0	0	0	0	0	0	0	0	0	0	0	0	0
0	0	0	0	0	0	0	0	0	0	0	0	0	0	0	0	0	0
0	0	0	0	0	0	0	0	0	0	0	0	0	0	0	0	0	0
0	0	0	0	0	0	0	0	0	0	0	0	0	0	0	0	0	0
0	0	0	0	0	0	0	0	0	0	0	0	0	0	0	0	0	0
0	0	0	0	0	0	0	0	0	0	0	0	0	0	0	0	0	0
0	0	0	0	0	0	0	0	72	19558	1750	325	103035	68	2585	1540	24132	1147
0	0	0	0	0	0	0	0	0	0	0	0	0	0	0	0	0	0
0	0	0	0	0	0	0	0	0	0	0	0	0	0	0	0	0	0
0	0	0	0	0	0	0	0	0	0	0	0	0	0	0	0	0	0
0	0	0	0	0	0	0	0	0	0	0	0	0	0	0	0	0	0
0	0	0	0	0	0	0	383	0	0	75	0	0	0	0	0	0	0
0	0	0	0	0	0	0	0	0	0	0	0	0	0	0	0	0	0
0	0	0	0	0	36	68178	0	0	0	0	0	0	0	0	0	1529	0
0	0	0	0	0	0	0	0	0	0	0	0	0	0	0	0	0	0
0	0	0	0	0	0	0	0	0	0	0	0	0	0	0	0	0	0
0	0	0	0	0	0	0	0	0	0	0	0	0	0	0	0	0	0
0	0	0	0	0	0	0	0	335	180	56	0	82	0	337	132	37616	144
0	0	0	0	0	0	0	0	0	0	0	0	0	0	0	0	0	0
0	0	0	0	0	0	0	0	0	0	0	0	0	0	0	0	0	0
0	0	0	0	0	0	0	0	0	0	0	0	0	0	0	0	0	0
0	0	0	0	0	0	0	0	121	0	0	0	0	250	0	0	0	0
0	0	0	0	0	0	0	0	0	0	0	0	0	0	0	0	0	0
0	0	0	0	0	0	0	0	0	0	0	0	0	0	0	0	0	0
0	0	0	0	0	0	0	0	137	0	0	0	0	0	0	0	0	0
0	0	0	0	0	0	0	0	0	0	0	0	0	0	0	0	0	0
0	0	0	0	0	0	0	0	0	0	0	0	0	0	0	0	0	0
0	0	0	0	0	0	0	0	0	0	0	0	0	0	0	0	0	0
52	0	0	0	0	0	0	0	0	54	0	0	635	51	0	130	592	224
0	0	0	0	0	0	0	0	0	0	0	0	0	0	0	0	0	0
0	0	0	1341	0	0	0	0	0	0	1722	0	0	0	0	0	0	0
0	0	0	0	0	0	0	0	0	0	0	0	0	0	0	0	0	0
0	0	0	0	0	0	0	0	0	125	0	0	0	0	0	0	0	0
0	0	0	0	0	0	0	0	0	0	0	0	0	0	0	0	0	0
0	0	0	0	0	0	0	0	0	0	0	0	0	0	0	0	0	0
0	0	0	0	0	0	0	0	0	0	0	0	0	0	0	0	0	0
0	0	0	0	0	0	0	0	0	0	0	0	0	0	0	0	0	0
0	0	0	0	0	0	0	0	0	0	0	0	82	0	0	0	0	0
0	0	0	0	0	0	0	0	0	0	0	0	0	0	0	0	0	0
0	0	0	0	0	57	8281	0	0	223	0	0	2395	0	646	368	86564	148
0	0	0	0	35	0	0	0	823	0	0	0	0	104	0	0	0	0
0	0	0	0	0	0	0	0	0	0	0	0	0	0	0	0	0	0
0	0	0	0	0	0	0	0	0	0	0	0	0	0	0	0	0	0
0	0	0	0	0	0	0	60	61	52	0	133	0	0	0	0	0	0
0	0	0	0	0	0	0	0	0	0	0	0	0	0	0	0	0	0
0	0	0	0	0	0	0	0	0	0	0	0	0	0	0	0	0	0
0	0	0	0	0	0	0	0	0	0	0	0	0	0	0	0	0	0
0	0	0	0	0	0	0	0	0	0	0	0	0	0	0	0	0	0
0	0	0	0	0	0	0	0	0	0	0	0	0	0	0	0	0	0
0	0	0	0	0	0	0	0	0	0	0	0	0	0	0	0	0	0
13004	11863	1488	0	0	6028	0	798	0	0	129	0	0	0	0	0	0	0
0	0	0	0	0	0	0	0	0	0	0	0	0	0	0	0	0	0
0	2852	0	0	0	0	0	0	0	0	0	0	0	0	0	0	0	0
0	0	0	0	0	0	0	0	0	0	0	0	0	0	0	0	0	0
0	0	0	0	0	0	0	0	0	0	0	0	0	0	0	0	0	0
0	0	0	0	0	0	0	0	0	232	61	0	644	186	0	267	360	81
0	0	0	0	0	0	0	0	0	0	0	0	0	0	0	0	0	0
0	0	0	0	0	0	0	0	0	0	0	0	0	0	0	0	0	0
0	0	0	0	0	0	0	0	0	0	72	0	0	0	0	0	0	0
183904	151	163	0	53	448	601930	322	0	0	398	0	0	0	0	0	0	0
0	0	0	0	0	0	0	0	0	0	0	0	0	0	0	0	0	0
0	0	0	0	0	0	0	0	0	0	0	0	0	0	0	0	0	0

0	8536	0	8063	0	0	4143	128	0	57	0	105	76	0	0	0	1799	0
0	2234	0	0	0	0	0	0	0	0	0	0	0	0	0	0	0	0
0	0	0	0	0	0	0	0	0	0	0	0	0	0	0	0	0	0
0	0	0	0	0	0	0	0	0	0	0	0	0	0	0	0	0	0
0	0	0	0	0	0	0	0	0	0	0	0	0	0	0	0	0	0
0	0	0	0	0	0	0	0	0	0	0	0	0	0	0	0	0	0
0	0	0	0	0	0	0	0	0	0	0	0	0	0	0	0	0	0
0	0	0	0	0	0	0	0	0	0	0	0	0	0	0	0	0	0
0	0	0	0	0	0	0	0	0	0	0	0	0	0	325	0	0	0
0	0	0	0	0	0	3413	0	0	47	0	0	354	0	0	0	10731	0
0	3931	0	0	0	0	0	0	0	0	0	0	0	191	0	0	1148	0
0	0	0	0	0	0	0	0	0	0	0	0	0	0	0	0	3041	0
0	0	0	0	0	0	0	0	0	0	0	0	0	0	0	0	0	0
0	0	0	0	0	0	0	0	0	0	0	0	0	0	0	0	0	0
0	0	0	0	0	0	0	0	0	0	0	0	0	0	0	0	5827	144
0	0	0	0	0	0	0	0	0	0	0	0	0	0	0	0	0	0
0	0	0	0	0	0	0	0	0	0	0	0	0	0	0	0	0	0
0	0	0	0	0	0	0	0	0	104	0	0	160	0	0	0	43	0
0	0	0	0	0	0	0	0	0	0	0	0	0	0	0	0	0	0
0	0	0	0	0	0	0	0	0	0	0	0	0	0	0	0	0	0
961	325601	762124	7393	2512	24620	64773	96422	27920	2856	79389	946231	12342	848304	11180	323017	202659	969842
0	0	0	0	0	0	0	0	0	0	0	0	0	0	0	0	0	0
0	0	0	0	0	0	0	0	0	0	0	0	0	0	0	0	2724	0
0	2005	1592	0	0	0	0	109	0	0	0	0	172	0	0	0	0	0
0	404	887	0	0	0	0	0	0	0	0	0	0	0	0	0	0	0
0	0	0	0	0	0	0	0	0	0	0	0	0	0	0	0	0	0
0	0	0	0	0	0	0	0	0	0	0	0	0	0	0	0	0	0
0	0	0	0	0	0	0	0	0	0	263	0	0	3289	77329	0	427	0
0	0	0	0	0	0	0	0	0	0	0	0	0	0	0	0	0	0
CCR002	CCR004	CCR005	CCR003	CCR007	CCR008	CCR009	CCR006	CC051	CC046	CC050	CC049	CC048	CC047	CC045	CC040	CC043	CC042
0	0	0	0	0	0	0	0	0	0	0	0	0	0	0	0	0	0
0	0	0	0	0	0	0	0	0	0	0	0	0	0	0	0	0	0
0	0	0	0	0	0	0	0	0	0	0	0	0	0	0	0	0	0
0	0	0	0	0	0	0	0	0	0	0	0	0	0	0	0	0	0
0	0	0	0	0	0	0	0	0	69	0	0	178	0	0	0	0	0
0	0	0	0	0	0	0	0	0	0	0	0	0	0	0	0	0	0
0	0	0	0	0	0	0	0	0	0	0	0	0	0	0	0	0	0
3210	4438	2460	2577	357	1782	14991	5213	81273	248825	576922	3634	748633	2430	32996	19194	149984	7016
0	0	0	0	0	0	0	0	0	0	0	0	0	0	0	0	0	0
1921	117887	92813	779220	4039	624216	65492	822473	0	0	0	0	0	0	0	0	0	0
0	0	0	0	0	0	0	0	0	0	0	0	0	0	0	0	0	0
0	0	0	0	0	0	0	0	0	0	0	0	0	0	0	0	0	0
0	0	0	0	0	0	0	0	0	0	0	0	0	0	0	0	0	0
0	0	0	0	0	0	0	0	0	123	0	0	163	0	0	0	0	0
0	0	0	0	0	0	0	0	0	0	0	0	0	0	0	0	0	0
0	0	0	0	0	0	0	0	0	0	0	0	0	0	0	0	2712	0
618	0	0	0	0	0	0	0	0	0	65	0	90	0	0	0	2219	0
134523	2645	1665	65	0	0	0	523	384	212	5369	0	428	0	315	301	20937	66
0	0	0	0	0	0	0	0	0	0	0	0	0	0	0	0	0	0
0	0	0	0	0	0	0	0	0	0	0	0	0	0	0	0	0	0
0	0	0	0	0	0	0	0	0	0	0	0	0	0	0	0	0	0
0	0	0	0	0	0	0	0	0	0	0	0	0	239	0	0	0	0
0	0	0	0	0	0	0	0	0	0	0	0	0	0	0	0	0	0
0	2390	908	0	0	0	0	100	73	4805	1112	317	15450	0	965	507	5279	0
0	0	0	0	0	0	0	0	0	0	0	0	0	0	0	0	0	0
0	0	0	0	0	0	0	0	0	0	0	0	0	0	0	0	0	0
0	0	419	0	0	0	0	0	0	0	0	0	0	0	0	0	0	0
0	0	0	0	0	0	0	0	0	0	0	0	0	0	0	0	0	0
0	0	0	0	0	0	0	0	0	0	0	0	0	0	0	0	42	0
0	0	0	0	0	0	0	0	0	0	0	0	0	0	0	0	97	0
0	0	0	0	0	0	0	0	0	0	0	0	0	358	0	110	0	1391
0	75	0	0	0	0	0	0	0	0	0	0	0	0	0	0	0	0
0	0	0	0	0	0	0	0	0	0	0	0	0	0	0	0	0	0
0	0	0	0	0	0	0	0	0	0	0	0	0	0	0	0	0	0
0	0	75	0	0	0	56	0	0	0	0	0	0	66	0	0	312	0
0	1752	945	0	0	0	0	0	0	0	0	0	88	0	0	0	0	0
0	0	0	0	0	0	0	0	1741	0	0	0	93	0	0	0	0	0
0	0	0	0	0	0	0	0	0	0	0	0	0	0	0	0	0	0
0	0	0	0	0	0	0	0	0	0	0	0	0	0	0	0	0	0
0	0	0	0	0	0	0	0	0	0	0	0	0	0	346	0	0	0
0	0	92	0	0	0	0	117	0	0	560	0	0	0	0	0	753	0
0	0	182	0	0	0	0	0	0	0	0	0	0	0	0	0	0	0
52	0	0	69	0	0	0	0	0	0	0	0	0	0	0	0	0	0
0	0	0	0	0	0	0	0	0	0	0	0	0	0	0	0	0	0
0	0	0	0	0	0	0	0	0	0	0	0	0	0	0	0	0	0
0	0	0	1218	0	5732	0	0	0	0	0	0	0	0	0	0	0	0
31478	88	0	1401	0	0	0	0	0	0	718	0	0	0	0	0	0	0
0	0	0	0	43	0	0	0	0	0	69	0	0	0	0	0	0	0
0	0	0	0	0	0	0	0	0	0	60	0	0	0	0	0	0	0
0	0	0	0	0	0	0	0	0	0	0	0	0	0	0	0	0	0
0	0	0	0	0	0	0	0	0	0	0	0	0	0	0	0	0	0
0	0	0	0	0	0	0	0	0	0	0	0	0	0	0	0	0	0
0	0	0	0	0	0	0	0	0	0	0	0	0	0	0	0	0	0
0	0	0	0	0	0	0	0	0	0	0	0	0	0	0	0	0	0

0	0	0	0	0	0	0	0	0	0	0	0	0	0	0	0	0	0
57233	6523	932	7621	180	2415	5411	1624	60	0	0	0	0	834	0	0	3611	0
0	0	0	0	0	0	0	0	0	0	76	0	0	0	0	0	0	0
8728	1403	163	78	0	183	2662	0	0	0	0	0	0	0	0	0	285	0
714	395	658	44603	0	463	5556	187	347166	2482	65688	719	6222	95161	729945	1732	4318	3531
753	0	0	54	0	0	0	0	129	0	78	0	0	0	0	0	0	0
0	0	0	0	0	0	0	0	0	0	0	0	0	0	0	0	0	0
0	0	0	0	0	0	0	0	0	0	0	0	0	0	0	0	0	0
26946	0	231	3756	0	0	49	0	57	0	472	0	0	903	284	113	52	54
0	0	0	0	0	0	0	52	0	0	2010	0	0	0	0	0	503	0
0	0	0	0	0	0	0	0	0	0	0	0	0	0	0	0	0	0
0	0	0	0	0	0	0	0	0	0	0	0	0	0	0	0	0	0
0	0	0	0	0	0	0	0	0	0	0	0	0	0	0	0	0	0
0	0	0	0	0	16870	0	0	0	52	0	0	0	0	0	0	0	188
0	0	0	0	0	0	0	0	0	0	0	0	0	0	0	0	0	0
0	0	0	0	0	0	0	0	0	0	0	0	0	0	0	0	0	0
0	0	0	0	0	0	0	0	0	0	0	0	0	0	0	0	0	0
0	0	0	0	0	0	0	0	0	0	0	0	0	0	0	0	0	0
0	0	0	0	0	0	0	0	0	0	0	0	0	0	0	0	0	0
43	0	0	259	0	0	0	0	42	0	0	0	101	0	0	0	0	0
0	0	0	0	0	0	0	0	0	0	0	0	0	0	0	0	0	0
0	0	0	0	0	0	0	0	0	0	0	0	0	0	0	0	0	0
0	0	0	0	0	0	0	0	0	0	0	0	0	0	0	0	0	0
0	0	0	0	0	0	0	0	0	0	0	0	0	0	0	0	0	0
0	0	0	0	0	0	0	0	0	0	0	0	0	0	0	0	0	0
0	0	0	0	0	0	0	0	0	275	0	0	0	0	0	0	0	0
0	0	0	0	0	0	67	0	0	0	0	0	0	0	0	0	0	0
0	0	0	0	0	0	0	0	0	0	0	0	0	0	0	0	0	0
0	0	0	0	0	0	0	0	0	0	0	0	0	306	0	0	0	915
4980	0	0	0	50	0	0	0	0	0	0	0	0	0	0	0	0	0
0	0	0	0	0	0	0	0	0	0	0	0	0	0	0	0	0	0
0	0	0	0	0	0	0	0	0	0	255	130	0	0	0	0	0	0
0	0	0	0	0	0	0	0	0	0	0	0	0	0	0	0	0	0
0	0	0	0	0	0	0	0	101	0	51	0	0	0	0	0	0	0
0	0	0	0	0	0	0	0	0	0	0	0	0	0	0	0	0	0
0	0	0	0	0	84	0	218	460603	101	42213	342	437	130	3220	131	289	59
0	0	0	0	0	0	0	0	0	0	0	0	0	0	0	0	0	0
0	16525	0	7170	0	19288	5282	448	0	0	0	0	83	0	0	0	4790	0
0	0	0	0	0	0	0	0	0	0	0	0	0	0	0	0	0	0
0	0	0	0	0	43	0	0	0	0	0	0	0	0	0	0	0	0
0	0	0	0	0	0	0	0	0	0	0	0	0	0	0	0	0	0
0	0	0	0	0	0	0	0	0	0	0	0	100	0	0	0	0	0
0	0	0	0	0	0	46	0	0	0	0	0	0	0	0	0	0	0
0	0	0	0	0	0	0	0	0	0	0	0	0	0	0	0	0	0
0	0	0	0	0	0	0	0	0	0	0	0	0	0	0	0	0	0
0	0	0	0	0	0	84	0	0	0	0	0	88	0	0	0	19140	0
0	0	0	0	0	0	0	0	0	0	0	0	0	0	0	0	0	0
0	0	0	8419	0	0	0	0	0	0	0	0	0	0	0	0	0	0
0	0	0	0	0	0	0	0	0	0	0	0	0	0	0	0	0	0
0	1613	0	0	0	0	0	0	0	0	0	0	0	0	0	0	0	0
0	0	0	0	0	0	0	0	0	485	73	0	1908	0	0	119	141	0
0	0	0	0	0	0	0	0	0	0	0	0	0	0	0	0	0	0
0	0	0	0	0	0	0	0	0	0	0	0	0	0	0	0	0	0
246	875	1552	708	978101	84	156	513	4997	695014	7501	11526	1926	1598	10859	642035	2722	1987
0	0	0	0	0	0	0	0	0	0	0	0	0	0	0	0	0	0
0	0	0	0	0	0	0	0	0	0	0	0	0	0	0	0	0	0
0	0	0	0	0	0	0	0	0	0	0	0	0	0	0	0	0	0
0	325	0	0	0	0	0	672	0	0	0	0	0	0	0	0	0	0
0	0	0	0	0	0	0	0	0	0	0	0	0	0	0	0	0	0
0	0	0	0	0	0	0	0	0	0	0	0	0	0	0	0	0	0
0	0	0	0	0	0	0	0	0	0	0	0	0	0	0	0	0	0
0	0	0	0	0	0	0	0	0	60	24028	0	0	0	0	0	45	0
0	0	0	0	0	0	0	0	0	0	0	121	0	0	0	0	0	0
0	0	0	0	0	0	0	0	0	0	0	0	0	0	0	0	0	0
0	0	0	0	0	0	0	0	0	0	0	0	0	0	0	0	0	0
0	2400	567	0	0	0	0	0	0	54	5166	0	0	50	0	150	3869	274
0	0	0	0	959	0	0	0	0	0	0	0	0	0	0	0	39	0
0	220	70	0	0	0	0	0	0	0	533	0	0	0	0	0	453	0
0	0	0	1671	194	0	0	0	0	0	0	0	0	0	0	0	0	0
0	0	0	0	0	0	0	0	0	0	0	0	0	0	0	0	0	0
0	0	0	0	0	0	0	0	0	0	0	0	0	0	0	0	0	0
0	0	0	0	0	0	0	0	0	0	0	0	0	0	0	0	1402	0
0	0	0	0	0	0	0	0	0	0	0	0	0	0	0	0	0	0
0	0	0	0	0	0	0	0	0	0	0	0	0	0	0	0	0	0
0	0	0	0	0	0	0	0	0	0	0	0	0	0	0	0	0	0
0	0	0	0	0	0	0	0	0	0	0	0	0	0	0	0	0	0
0	0	0	0	0	0	0	0	0	0	0	0	0	0	0	0	0	0
0	0	0	0	0	0	0	0	0	0	0	0	0	0	0	0	0	0
0	0	0	0	0	0	0	0	0	0	0	0	0	0	0	0	0	0
0	0	0	0	0	0	0	0	0	0	0	0	0	0	0	0	0	0
0	0	0	0	0	0	0	0	0	0	0	0	0	0	0	0	0	0
0	0	0	0	0	0	0	0	0	0	0	0	0	0	0	0	0	0
0	0	0	0	0	0	0	0	0	0	0	0	0	0	0	0	0	0
0	0	0	0	0	0	0	52	0	0	0	0	0	0	0	0	0	0
0	0	0	0	0	0	0	0	0	0	0	0	0	0	0	0	0	0
0	0	0	0	0	0	0	0	0	330	492	0	499	0	2202	1035	586	209
0	0	0	0	0	0	0	0	0	0	0	0	0	0	0	0	0	0

0	0	0	0	0	0	0	0	0	0	0	0	0	0	0	0	0	0
0	0	0	0	0	0	0	0	0	0	0	0	0	0	0	0	0	0
0	0	0	0	0	0	0	0	0	0	0	0	0	0	0	0	0	0
0	0	0	0	0	0	0	0	0	0	0	0	0	0	0	0	0	0
21965	513	743	17367	0	0	0	0	171	157	207	127	697	1306	1287	518	52059	163
52	0	0	0	0	0	0	0	0	0	0	0	0	0	0	0	44	0
0	81	1088	8782	215	47	158	1967	581	94	1181	376	176	3797	297	242	139	1386
4504	15833	143	3684	0	0	0	121	60	174	393	95	160	19163	1268	268	90914	338
552	88	0	491	0	0	58	756	0	0	66	0	0	69	0	0	1563	0
0	0	138	0	0	0	8875	0	0	0	0	0	0	0	0	0	0	60
0	0	0	0	54	0	0	0	0	0	60	101	0	0	0	0	0	0
0	0	0	0	0	0	0	0	0	0	132	0	0	0	0	0	0	0
0	0	0	0	0	0	0	0	0	0	0	0	0	0	0	0	0	0
0	0	0	0	0	0	0	0	0	0	0	0	0	0	0	0	0	0
0	0	0	0	0	0	0	0	0	0	0	0	0	0	0	0	0	0
0	0	0	0	0	0	0	0	0	0	0	0	0	0	0	0	0	0
0	0	0	0	0	0	0	0	0	0	0	0	0	0	0	0	0	0
0	0	0	0	0	0	107	0	0	0	0	0	0	0	0	0	0	0
0	0	0	0	0	0	0	0	0	0	0	0	0	699	0	0	0	0
0	0	0	0	0	0	0	0	0	0	0	0	75	0	0	0	0	0
3312	8704	572	0	0	0	0	0	0	0	0	0	0	0	0	0	0	0
0	0	0	0	0	0	0	0	0	57	0	0	0	0	0	125	0	0
0	0	0	0	0	0	0	0	0	0	0	0	0	0	0	0	0	0
0	0	0	0	0	0	0	0	0	0	0	0	0	0	0	0	0	0
0	0	0	0	0	0	0	0	0	0	0	0	0	0	0	0	0	0
0	0	0	0	0	0	0	0	0	0	0	0	0	0	0	0	0	0
0	0	0	0	0	0	0	0	0	0	0	0	0	0	0	0	0	0
0	0	0	0	0	0	0	0	0	0	0	0	0	0	0	0	0	0
0	0	0	0	0	0	0	0	0	65	0	0	73	0	0	0	0	0
0	0	0	0	0	0	0	0	0	0	0	0	0	0	0	0	0	0
0	0	0	0	0	0	0	0	0	0	0	0	0	0	0	0	0	0
0	0	0	0	0	0	0	0	0	0	0	0	0	0	0	0	0	0
0	0	0	0	0	0	0	0	0	0	63	0	0	0	0	151	0	0
0	0	0	0	0	0	0	0	0	0	0	0	256	0	0	0	0	0
0	0	0	0	0	0	0	0	0	0	0	0	0	0	0	0	0	0
0	9203	0	0	0	0	0	0	0	0	0	0	0	0	0	0	0	0
0	0	0	0	0	0	0	0	0	0	0	0	0	0	0	0	0	0
0	0	0	0	0	0	0	0	0	0	261	0	0	0	0	0	0	0
0	0	0	0	0	0	0	0	0	0	0	0	0	0	0	0	0	0
0	0	0	0	0	0	0	0	0	0	0	0	0	0	0	0	0	0
14397	0	0	63	265	0	0	0	0	0	0	0	0	0	0	0	0	0
0	0	0	0	0	0	0	0	0	0	0	0	0	0	0	0	0	0
0	0	0	0	0	0	0	0	0	0	0	0	0	0	0	0	0	0
0	0	0	0	0	0	0	0	0	0	0	0	0	0	0	0	0	0
0	0	0	0	0	0	0	0	0	0	0	0	0	0	0	0	0	0
0	0	0	0	0	0	0	0	0	0	0	0	0	0	0	0	0	0
0	0	0	200	0	0	0	0	0	0	0	0	0	0	0	0	0	0
0	0	0	0	0	0	0	0	0	64	71	0	0	0	0	0	3792	0
0	0	0	0	0	0	0	0	0	0	0	0	0	0	0	0	0	0
0	0	0	0	0	0	0	0	0	0	0	0	0	0	0	0	0	0
0	0	0	0	0	0	0	0	0	0	0	0	0	0	0	0	0	0
0	0	0	0	192	0	0	0	0	0	0	0	0	0	0	0	0	0
0	0	0	0	0	0	0	0	0	0	0	0	0	0	0	0	0	0
0	0	0	0	0	0	0	508	0	0	131	0	0	0	0	0	0	0
0	0	0	0	0	0	0	0	0	0	0	0	0	0	0	0	0	0
0	0	0	0	0	0	0	0	0	0	0	0	0	0	0	0	0	0
0	0	0	0	0	0	0	0	0	0	0	0	0	0	0	0	0	0
0	0	0	0	0	0	0	0	0	0	0	0	0	0	0	0	0	0
0	0	0	0	0	0	0	0	0	0	0	0	0	0	0	0	0	0
0	0	0	0	0	0	0	0	0	0	0	0	0	0	0	0	0	0
0	0	0	0	0	0	0	0	493	0	0	0	0	0	0	0	0	0
0	0	0	0	0	0	0	0	0	0	0	0	0	0	0	0	0	0
0	0	0	0	0	0	0	0	0	0	0	0	0	0	0	0	0	0
0	0	0	0	0	0	0	0	0	0	0	0	0	0	0	0	0	0
0	0	0	0	0	0	0	0	0	0	0	0	0	0	0	0	0	0
0	69	0	0	0	0	0	0	0	0	0	0	0	0	0	0	0	0
0	7080	0	0	0	0	0	0	0	0	0	0	0	0	0	0	0	0
0	3094	0	0	0	0	0	0	0	0	0	0	0	0	0	0	0	0
0	0	0	0	0	0	0	0	0	0	0	0	0	0	0	0	0	129
0	0	0	0	0	0	0	0	0	0	0	0	0	0	0	0	0	0
0	0	0	0	0	0	0	0	0	0	0	0	0	0	633	0	5861	0
152250	291	174	60	88	0	3779	60	0	0	0	0	0	0	0	0	0	0
95655	64	0	137	5855	709	0	68	0	0	88	0	0	0	0	240	0	0
0	0	0	0	0	0	0	0	0	0	0	0	0	0	0	0	0	0
0	0	0	0	0	0	0	0	0	0	0	0	0	0	0	0	0	0
0	2100	2385	0	0	0	666	19024	7293	960	51879	255	3652	650	2556	0	9977	140
0	0	0	0	0	0	0	0	0	0	0	0	0	0	0	0	0	0
0	0	0	0	0	0	0	0	0	0	0	0	0	0	0	0	0	0
0	0	0	0	0	0	0	0	0	0	0	0	0	0	0	0	0	0
0	0	0	0	0	0	0	0	0	0	0	0	0	0	0	0	0	0
0	0	0	0	0	0	0	0	0	0	0	0	0	0	0	0	0	0
0	0	0	0	0	0	0	0	0	0	0	0	0	0	0	0	0	0
0	0	0	0	0	0	0	0	0	0	0	0	0	0	0	0	0	0
0	0	0	0	0	0	0	0	0	0	0	0	0	0	0	0	0	0
0	0	0	0	0	0	0	0	0	0	0	0	0	0	0	0	0	0
0	0	0	0	0	0	0	0	0	0	0	0	0	0	0	0	0	0
0	0	0	0	0	0	0	0	0	0	0	0	0	0	0	0	0	0

0	0	0	0	0	0	0	0	0	0	0	0	0	0	0	0	0	0
0	296	0	0	0	0	0	0	463	176	617	0	97	70	0	0	394	0
0	0	859	0	0	0	0	0	0	0	0	0	179	0	0	0	0	0
0	0	0	0	0	0	0	0	0	0	0	0	0	0	0	0	0	0
0	0	0	0	0	0	0	0	0	0	0	0	0	0	0	0	0	0
0	0	0	0	0	0	0	0	0	0	0	0	0	0	0	0	0	0
0	0	0	0	0	0	0	0	0	0	0	0	0	0	0	0	0	0
0	0	0	0	0	0	0	0	0	0	0	0	0	0	0	0	0	0
0	0	0	0	0	0	0	0	0	0	0	0	0	0	0	0	0	0
0	0	0	0	0	0	0	0	0	0	0	0	0	0	0	0	0	0
0	0	0	0	0	0	0	0	0	0	0	0	0	0	0	0	0	0
0	0	0	0	0	0	0	0	0	0	0	0	0	0	0	0	0	0
0	0	989	0	0	0	0	0	0	0	0	0	0	0	0	0	0	0
0	0	0	0	0	0	0	0	0	0	0	0	0	0	0	0	0	0
0	0	0	0	0	0	0	0	0	0	0	0	0	0	0	0	0	0
0	0	0	0	0	0	0	0	0	0	0	0	0	0	0	0	0	0
0	0	0	0	0	0	0	0	0	0	0	0	0	0	0	0	0	0
0	0	0	0	0	0	0	0	0	0	0	0	0	0	0	0	0	0
0	0	0	0	0	0	0	0	0	0	0	0	0	0	0	0	0	0
0	0	0	0	0	0	0	0	0	0	0	0	0	0	0	0	0	0
0	0	0	888	0	0	0	0	0	0	0	0	0	0	0	0	0	0
0	0	0	0	0	0	0	0	0	0	0	0	0	0	0	0	0	0
0	0	0	0	0	0	50	0	0	3269	769	115	11451	0	663	404	6925	136
0	0	1022	0	0	0	0	0	0	0	0	0	0	0	0	0	0	0
0	0	0	70	0	47	0	0	0	0	0	0	0	0	329	0	13620	0
0	0	0	0	0	0	0	0	0	0	0	0	0	0	0	134	0	0
0	0	0	0	0	0	0	0	0	0	0	0	0	0	0	0	0	0
0	0	0	0	0	0	0	0	0	0	0	0	0	0	0	0	0	0
0	0	0	0	0	0	0	0	0	50	0	0	0	62	0	0	0	0
0	0	0	0	0	0	0	0	0	0	0	0	0	0	0	0	0	0
0	0	0	0	0	0	0	0	0	0	0	0	0	0	0	0	0	0
0	0	0	0	0	0	0	0	0	0	0	0	0	0	0	0	0	0
0	0	0	0	0	0	0	0	0	0	0	0	0	0	0	0	0	0
990	12820	27168	249	408	1080	1799	4112	15554	6991	8584	31141	28142	1449	10886	861	27258	343
0	0	0	1634	0	0	0	0	0	0	0	0	0	0	0	0	0	0
0	395	64	0	0	54	0	240	0	0	70	0	0	0	0	0	0	0
60	16650	1649	0	223	2319	0	11830	0	0	1531	0	0	0	0	0	0	0
0	0	0	0	0	0	0	0	0	0	0	0	0	0	0	0	0	0
0	0	0	0	0	0	0	0	0	0	0	0	0	0	0	0	0	0
0	0	0	0	0	0	0	238	0	235	454	0	82	0	0	0	48	0
0	0	0	0	0	0	0	0	0	0	0	0	0	0	0	0	0	0
0	0	0	0	0	0	0	0	869	0	79	0	0	0	0	0	0	0
0	0	0	0	0	0	0	0	0	0	0	0	0	0	0	0	0	0
0	0	0	0	0	0	0	0	0	0	0	0	0	0	0	0	0	0
56602	33972	3792	18154	4512	0	1234	6123	0	0	0	440	0	0	0	0	0	0
0	0	0	0	0	0	0	0	0	0	0	0	0	0	0	0	0	0
0	0	0	0	0	0	0	0	0	0	0	0	0	0	0	0	252	0
0	3079	0	0	0	0	0	0	0	0	0	0	0	0	0	0	0	0
0	0	154	0	0	0	0	0	0	0	0	0	0	0	0	0	0	0
0	0	31348	0	0	0	0	0	0	0	0	0	0	0	0	0	0	0
0	0	0	0	0	0	0	0	0	0	0	0	0	0	0	0	0	0
19882	0	0	3432	0	0	10706	418	50	176	0	0	248	1291	1295	144	2565	194
0	0	0	0	0	0	0	0	0	55	0	0	81	0	0	0	0	0
0	0	0	0	0	0	0	0	0	0	0	0	0	0	0	0	0	0
0	0	0	0	0	0	0	115	0	0	124	0	0	0	0	0	0	0
0	0	0	0	0	0	0	0	0	0	0	0	0	0	0	0	0	0
0	0	0	0	0	0	0	0	0	0	0	0	0	0	0	0	0	0
0	0	0	0	0	0	0	0	0	0	0	0	0	0	0	0	0	0
0	0	0	0	0	0	0	0	0	0	0	0	0	0	0	0	0	0
0	0	0	0	0	0	0	0	0	0	0	0	0	0	0	0	0	0
0	0	0	0	0	0	0	0	0	0	0	0	0	0	0	0	0	0
0	0	0	0	0	0	0	0	0	0	0	0	0	0	0	0	0	0
0	0	0	0	0	0	0	0	0	0	0	0	0	0	0	0	0	0
0	0	0	0	0	0	0	0	0	0	0	0	0	0	0	0	0	0
0	0	0	0	0	0	0	0	0	0	0	0	0	0	0	0	0	0
0	0	0	0	0	0	0	0	0	0	0	0	0	0	0	0	7153	80
0	0	0	0	0	0	0	0	0	0	0	0	0	0	0	0	0	0
0	0	0	0	0	0	0	0	0	0	0	0	0	0	0	0	0	0
0	0	0	0	0	0	0	381	0	0	0	0	94	0	0	136	0	0
0	425	0	0	0	0	0	0	0	0	0	0	0	0	0	0	0	0
0	0	0	0	0	0	0	0	0	0	265	0	0	0	0	0	0	0
0	0	0	0	0	0	0	54	0	374	714	0	444	0	0	0	0	0
0	0	0	0	0	0	0	0	0	0	0	0	0	0	0	0	0	0
0	0	0	0	0	0	0	0	0	0	0	0	0	0	0	0	0	0
0	0	0	0	0	0	0	0	0	0	0	0	0	0	0	0	0	0
0	0	0	0	0	0	0	0	0	0	0	0	0	0	0	0	0	0
0	0	0	0	0	43	0	0	0	0	0	0	0	0	0	0	0	0
0	0	0	0	0	0	0	0	0	53	0	0	0	0	0	139	7941	0
0	0	0	0	0	0	0	0	0	0	0	0	0	0	0	0	0	0
0	0	0	0	0	0	0	0	0	0	0	0	0	0	0	0	0	0
0	0	0	0	0	0	0	0	0	0	0	0	0	0	0	0	0	0
0	0	0	0	0	0	0	0	359	0	0	0	0	0	0	0	0	0
0	0	0	0	0	0	0	0	0	0	0	0	0	0	0	0	0	0
0	0	0	0	0	0	324	0	170	0	65	0	257	3152	0	610	1167	3719
0	0	0	0	0	0	0	0	0	0	0	0	0	0	0	0	0	0
0	0	0	0	0	0	0	0	0	0	0	0	0	0	0	0	0	0

0	0	0	0	0	0	0	0	0	0	0	0	0	0	0	0	0	0
0	0	0	0	0	0	0	0	0	0	0	0	0	0	0	0	0	0
0	0	0	0	0	0	47	1568	0	0	0	0	0	0	0	0	0	0
0	0	0	0	0	0	0	0	0	0	0	0	0	0	0	0	0	0
0	0	0	0	0	0	0	0	0	0	0	0	0	0	0	0	0	0
0	8322	6060	0	0	46	0	0	0	0	0	0	0	0	0	0	164	0
0	0	0	0	0	0	0	0	0	0	0	0	0	0	0	0	688	0
0	6828	1394	0	0	0	0	0	0	0	0	0	0	0	0	0	0	0
0	0	0	0	0	0	0	0	0	0	0	0	0	57	0	0	0	0
0	0	0	0	0	0	0	0	0	0	0	0	0	0	0	0	0	0
0	0	0	0	0	0	0	0	0	0	0	0	0	0	0	0	0	0
6750	0	731	0	0	0	7908	0	0	51	61	0	171	0	0	0	0	0
0	0	0	0	0	0	0	0	0	0	0	0	0	0	0	0	0	0
0	0	0	0	0	0	0	0	0	0	0	0	0	0	0	0	0	0
0	0	0	0	0	0	0	0	0	0	0	0	0	0	0	0	382	0
0	0	0	0	0	0	0	0	0	0	0	0	0	0	0	0	0	0
0	0	0	0	0	0	0	0	0	0	0	0	0	0	0	0	0	0
0	0	0	0	0	0	0	0	0	0	0	0	0	0	0	0	0	0
0	0	0	0	0	0	0	0	0	0	0	0	0	0	0	0	0	0
0	0	0	0	0	0	0	0	0	0	0	0	0	0	0	0	0	0
0	0	0	0	0	0	0	0	0	0	0	0	0	0	0	0	0	0
0	0	0	0	0	0	0	0	0	0	0	0	0	0	0	0	0	0
0	0	0	0	0	0	0	0	0	0	0	111	0	0	0	0	0	0
0	0	0	0	0	0	0	0	0	0	0	0	0	0	0	0	0	0
0	0	0	0	0	0	0	0	0	0	0	0	0	0	0	0	0	0
0	0	0	0	0	0	0	0	0	0	0	0	0	0	0	0	0	0
0	0	0	0	0	0	0	0	0	0	0	0	0	0	0	0	0	0
0	0	0	0	0	0	0	0	0	0	0	0	0	0	0	0	0	0
0	0	0	0	0	0	49701	0	0	0	0	0	0	0	0	0	0	0
0	0	0	0	0	0	0	0	0	0	0	0	0	0	0	0	0	0
0	0	0	0	0	0	0	0	0	0	0	0	0	0	0	0	0	0
0	4827	374	0	0	0	0	0	0	0	0	0	0	0	0	0	0	0
0	0	0	0	0	0	0	0	0	0	0	0	0	0	0	0	0	0
0	0	0	0	0	0	0	0	0	0	0	0	0	0	0	0	0	0
0	0	0	0	0	0	0	0	0	0	0	0	0	0	0	0	0	0
0	0	0	0	0	0	0	0	0	0	0	0	0	0	0	249	104	0
0	0	0	0	0	0	0	430	0	0	0	0	0	0	0	0	0	0
0	0	0	0	0	81783	42	0	0	0	0	0	0	0	0	0	0	0
0	0	0	0	0	0	0	0	0	232	0	0	332	0	972	617	112894	287
0	0	0	0	0	0	0	0	0	0	0	0	0	0	0	0	0	0
0	0	0	0	0	0	0	0	0	0	0	0	0	0	0	0	0	0
0	0	0	0	0	0	0	0	0	0	0	0	0	0	0	0	0	0
0	0	0	0	0	0	0	0	0	0	0	0	0	0	0	0	0	0
0	0	0	0	0	0	0	0	0	0	0	0	0	0	0	0	0	0
0	0	0	0	0	0	0	0	0	0	0	0	0	0	0	0	0	0
6252	0	1817	3528	167	55797	36727	0	1327	0	0	258	76	0	0	0	0	0
0	0	0	0	0	0	0	0	0	0	0	0	0	136	0	0	0	0
0	0	0	0	0	0	0	0	0	0	0	0	0	0	0	0	82	0
0	0	0	0	0	0	0	0	0	0	0	0	0	0	0	0	0	0
850	0	0	1183	0	0	2969	61	0	0	0	125	92	0	0	0	388	0
0	0	0	0	0	0	0	0	0	0	0	0	0	0	0	0	0	0
46243	77148	5923	9205	811	67364	12532	1198	18044	56	1217	807	93	1883	327	0	1944	811
0	6546	0	0	50	2404	201	0	163	0	0	0	0	0	0	0	0	0
0	0	0	0	0	0	0	0	0	0	0	0	0	0	0	0	0	0
0	0	0	0	0	0	0	0	0	0	0	0	0	0	0	0	0	0
0	0	0	0	0	0	0	0	0	0	0	0	0	0	0	0	0	0
0	0	0	0	0	0	0	0	0	0	0	0	0	0	0	0	0	0
0	0	0	0	0	0	0	0	0	0	0	0	0	0	0	0	0	0
0	0	0	0	0	0	0	0	0	0	0	0	0	0	0	0	0	0
0	0	0	0	0	6103	0	0	0	0	0	0	0	0	0	0	0	0
0	0	0	0	0	11826	0	0	0	171	0	0	933	0	0	0	42	0
0	0	0	0	121	0	0	0	0	0	0	0	0	0	0	132	384	0
0	0	0	0	0	0	0	0	134	0	73	0	0	0	0	0	0	0
0	0	0	0	0	0	0	0	0	0	0	0	0	0	0	0	0	0
0	0	0	0	0	0	0	0	0	0	0	0	0	0	0	0	636	0
0	0	0	0	0	0	0	0	0	0	0	0	0	0	0	0	0	0
0	5271	5625	48382	49	9311	4512	12106	0	0	0	0	0	0	0	0	0	0
0	215	9903	56	0	126	0	2534	0	0	0	0	0	0	0	0	0	0
0	0	0	2553	0	6413	0	0	0	0	0	0	0	0	0	0	0	0
0	0	0	0	0	0	0	0	0	0	0	0	0	0	0	0	0	0
0	0	0	0	0	0	0	0	0	0	349	0	0	376	0	0	1210	0
22408	2595	82	0	0	0	0	0	0	0	0	0	0	0	0	0	0	0
0	0	0	0	0	0	0	0	0	0	0	0	0	0	0	0	0	0
0	0	0	0	0	0	0	0	0	0	0	0	0	0	0	0	0	0
0	0	0	0	0	0	0	0	0	0	0	0	0	0	0	0	0	0
0	0	0	0	0	0	0	0	0	0	0	0	0	0	0	0	0	0
0	0	0	0	0	0	0	0	0	0	0	0	0	0	0	0	0	0
0	0	0	0	0	0	0	0	0	0	0	0	0	0	0	0	0	0
0	0	0	0	0	0	0	0	0	0	0	0	0	0	0	0	0	0
0	0	0	0	0	0	0	0	0	0	0	0	0	0	0	0	0	0
0	0	0	0	0	0	0	0	0	43	0	0	0	0	0	0	0	0
0	0	0	0	0	0	0	0	0	0	0	0	0	0	0	0	0	0
0	0	0	0	0	0	0	0	0	0	0	0	0	0	0	0	0	0
0	0	0	0	0	0	0	0	0	0	0	0	0	0	0	0	0	0
0	0	0	0	0	0	0	0	0	0	0	0	0	0	0	0	0	0
0	0	0	0	0	0	0	0	0	0	0	0	0	0	0	0	0	0
0	0	0	0	0	0	0	0	0	0	0	0	0	0	0	0	0	0

0	9040	2232	0	0	0	0	0	0	0	131	0	0	0	0	0	0	0
0	0	0	0	0	0	0	0	0	0	0	0	0	0	0	0	0	0
0	0	0	0	0	0	0	0	0	0	142	0	0	0	0	0	0	0
0	0	0	0	0	0	0	0	0	0	0	0	0	0	0	0	0	0
0	19702	1100	80	203	28409	0	0	0	0	207	0	0	0	0	0	2552	0
0	0	0	0	0	0	0	0	0	0	0	0	0	0	0	0	0	0
60	0	0	0	0	0	0	0	0	0	0	0	0	0	0	0	0	0
0	0	0	0	0	0	0	0	0	0	0	0	0	0	0	0	0	0
0	0	0	0	0	0	0	0	0	0	0	0	0	0	0	0	0	0
0	0	0	0	0	0	0	0	0	0	0	0	0	0	0	0	0	0
0	0	0	0	0	0	0	0	0	0	823	0	0	0	0	0	0	0
0	0	0	0	0	0	0	0	0	0	187	0	0	0	0	0	0	0
0	0	0	0	0	0	0	0	0	0	0	0	0	0	0	0	0	0
0	0	0	0	0	0	0	0	0	227	0	0	273	0	0	0	0	74
0	0	0	0	0	0	0	0	0	0	0	0	0	0	0	0	0	0
0	0	0	0	0	0	0	0	0	0	0	0	0	0	0	0	0	0
0	0	0	0	0	0	0	0	0	0	0	0	0	0	0	0	0	0
0	0	0	0	0	0	0	0	0	0	142	0	0	0	0	0	0	0
0	0	0	0	0	0	0	0	0	0	0	0	0	0	0	0	0	0
0	0	0	0	0	0	0	0	0	0	0	0	0	0	0	0	0	0
0	0	0	0	0	0	0	0	0	0	0	0	0	0	0	0	0	0
93	0	277	0	0	489	96	0	267	3513	1026	1402	9537	3838	98644	369	1971	152
0	0	0	0	0	0	0	0	0	0	0	0	0	0	0	0	0	0
0	0	0	0	0	0	0	0	0	0	0	0	0	0	0	0	0	0
0	9623	0	0	0	0	0	0	0	0	0	0	0	0	0	0	0	0
0	0	0	0	0	0	0	0	0	0	0	0	0	0	0	0	0	0
0	0	0	0	0	0	0	0	0	0	0	0	0	0	0	0	0	0
0	0	0	0	0	0	0	0	0	0	0	0	0	0	0	0	0	0
0	0	0	0	0	0	0	0	6445	46	131	0	241	0	0	0	489	0
0	0	0	0	0	0	0	0	0	0	0	0	0	0	0	0	0	0
0	0	0	0	0	0	0	0	0	0	0	0	0	0	0	0	0	0
0	0	0	0	0	0	0	0	0	0	0	0	0	0	0	0	0	0
0	0	0	0	0	0	0	0	0	0	0	0	0	0	0	0	0	0
0	0	0	0	0	0	0	0	0	0	0	0	0	0	0	0	0	0
0	0	0	0	0	0	0	0	0	0	0	0	0	0	0	0	0	0
0	0	0	0	0	0	0	0	0	0	0	0	0	0	0	0	1630	0
0	0	0	0	0	0	0	0	0	0	0	0	0	0	0	0	0	0
0	0	0	0	0	0	0	0	0	0	0	0	0	0	0	0	0	0
0	97525	7554	0	0	0	0	0	0	0	0	0	0	0	0	0	0	0
0	0	0	0	0	0	0	0	0	0	0	0	0	0	0	0	0	0
0	0	0	0	0	0	0	0	0	0	0	0	0	0	0	0	0	0
0	0	0	0	0	0	0	0	0	0	0	0	0	65	0	0	458	78
0	0	0	0	0	0	0	0	41	0	0	0	0	0	0	0	0	0
0	0	0	0	0	0	0	0	0	0	0	0	0	0	0	0	0	0
0	0	0	0	0	0	0	0	0	0	0	0	0	0	0	0	0	0
0	0	0	0	0	0	0	0	0	0	0	0	0	0	0	0	0	0
0	0	0	0	0	0	0	0	58	0	0	0	0	0	0	0	0	0
0	0	0	0	0	0	0	0	0	0	0	0	0	0	0	0	0	0
0	0	0	1700	0	0	0	0	0	0	0	0	0	0	0	0	0	0
0	0	0	0	0	0	0	0	0	0	0	0	0	0	0	0	0	0
0	0	0	0	0	0	0	0	0	0	0	0	0	0	0	0	0	0
0	0	0	436	0	0	0	0	0	0	87	0	0	0	0	0	0	0
0	0	0	0	0	0	0	0	0	0	0	0	0	0	0	0	0	0
0	0	0	0	0	0	0	0	0	0	0	0	0	0	0	0	0	0
0	0	0	0	0	0	0	0	0	0	0	0	0	0	0	0	0	67
0	0	0	0	0	0	0	0	0	0	0	0	0	0	302	0	96	0
0	0	0	0	0	0	56	0	0	0	0	0	0	0	0	0	0	0
0	0	0	0	0	0	0	287	0	0	0	0	0	0	0	0	0	0
0	0	0	0	0	0	0	0	0	0	0	0	0	0	0	0	0	0
0	0	0	0	0	0	0	0	0	0	0	0	0	0	0	0	0	0
0	0	0	0	0	0	0	0	0	0	0	0	0	0	0	0	0	0
0	0	0	0	0	0	0	0	0	608	0	0	413	0	0	0	40	0
0	0	0	0	0	0	0	0	0	0	0	0	0	0	0	0	0	0
0	0	0	0	0	0	0	0	0	0	0	0	0	0	0	0	0	0
0	0	0	0	0	0	0	0	0	0	0	0	0	0	0	0	0	0
0	76	56	0	0	0	0	0	0	0	0	0	0	0	0	0	0	0
0	0	0	0	0	0	0	0	0	0	0	0	0	0	0	0	0	0
0	0	0	0	0	0	0	0	0	0	0	0	0	0	0	0	0	0
0	0	0	0	0	0	0	0	0	0	0	0	0	0	309	0	0	0
0	0	0	0	0	0	0	0	0	0	0	0	0	0	0	0	0	0
0	0	0	0	0	0	0	0	0	0	0	0	0	0	0	0	0	0
0	0	0	0	0	0	0	0	0	0	0	0	0	0	0	0	0	0
0	0	0	0	0	0	0	0	0	0	0	0	0	0	0	0	0	0
0	0	0	0	0	0	0	0	0	0	0	0	0	0	0	0	4640	73
0	0	0	0	0	0	0	0	0	0	0	0	0	0	0	0	0	0
0	87	0	0	54	0	0	67	0	0	1272	0	186	0	0	134	0	0
0	5284	0	0	0	0	0	0	0	0	0	0	0	0	0	0	0	0
0	74	0	0	0	0	0	0	0	0	0	0	0	0	0	0	0	0
0	0	0	0	0	0	0	0	0	0	0	0	0	0	0	0	0	0
0	0	0	0	0	0	0	0	0	0	0	0	0	0	0	0	0	0
0	0	0	0	0	0	0	0	0	0	0	0	0	0	0	0	0	0
0	0	0	0	0	0	0	0	0	0	0	0	0	0	0	0	0	0
0	0	0	0	0	0	0	0	0	0	0	0	0	0	0	0	0	0
0	0	0	0	0	0	0	0	0	0	0	0	0	0	0	0	0	0
0	0	0	0	0	0	0	0	0	0	0	0	0	0	0	0	0	0
0	8678	2428	0	0	0	0	3611	0	0	0	0	334	0	0	0	1300	0
0	0	0	0	0	0	0	0	0	0	0	0	0	0	0	0	0	0

0	0	0	0	0	0	0	0	0	0	0	0	0	0	0	0	0	0
0	0	0	0	0	0	0	0	0	0	0	0	0	0	0	0	0	0
0	0	0	0	0	0	0	0	0	0	0	124	0	0	0	0	0	0
0	0	0	0	0	0	0	0	0	0	0	0	78	0	0	244	8756	0
0	0	0	0	0	0	0	0	0	0	0	0	0	0	0	0	0	0
0	355	0	0	0	0	0	0	0	0	0	0	0	0	0	0	0	0
0	0	0	0	0	0	0	0	0	0	0	0	0	0	0	0	0	0
0	0	0	0	0	0	0	0	0	0	0	0	0	0	0	0	0	0
0	0	0	0	0	0	0	0	0	0	0	0	0	0	0	0	0	0
133	0	78	0	46	4627	1829	60	64	0	71	131	145	0	331	132	14967	212
0	0	0	0	0	0	0	0	0	0	0	0	86	0	0	0	3749	0
0	0	0	0	0	0	0	0	0	0	0	0	0	0	0	0	0	0
0	0	0	1902	0	0	0	0	0	0	0	0	0	0	0	0	0	0
0	0	0	0	0	0	0	0	0	0	0	0	0	0	0	0	0	0
0	0	0	0	0	0	0	0	0	0	0	0	0	0	0	0	0	0
0	0	0	0	0	0	0	0	0	0	0	0	0	0	0	0	0	0
0	0	0	0	0	0	0	0	0	0	0	0	0	0	0	0	0	0
0	0	0	0	0	0	0	0	0	0	0	0	0	0	0	0	0	0
0	0	0	0	0	0	0	0	0	0	0	0	0	0	0	0	0	0
0	0	0	0	0	0	0	0	0	0	0	0	0	0	0	0	0	0
0	0	0	0	0	0	0	0	0	0	0	0	0	0	0	0	0	0
0	0	0	0	0	0	0	0	0	0	0	0	0	0	0	0	0	0
0	0	0	0	0	0	0	0	0	0	0	0	0	0	0	0	0	0
0	0	0	0	0	0	0	0	0	0	0	0	0	0	0	0	0	0
0	0	0	0	0	0	0	0	0	0	0	0	0	0	0	0	0	0
0	0	0	0	0	0	0	0	0	57	0	0	0	0	0	0	54	0
0	0	0	0	0	0	0	0	0	0	0	0	0	0	0	0	0	0
0	0	0	0	0	0	0	0	0	0	0	0	0	0	0	0	0	0
0	0	0	0	0	0	0	0	0	0	0	0	0	0	0	0	0	0
0	0	0	0	0	0	0	0	0	0	0	0	0	0	0	0	0	0
0	0	0	0	0	0	0	0	0	0	0	0	0	0	0	0	0	0
0	0	0	0	0	0	0	0	0	0	0	0	0	0	0	0	0	0
0	0	0	0	0	0	0	0	0	0	0	0	0	0	0	0	0	0
0	492	0	0	0	0	0	0	0	0	0	0	0	0	0	0	0	0
0	0	0	0	0	0	0	0	0	0	0	0	0	0	0	0	0	0
0	0	0	0	0	0	0	0	0	0	0	0	0	0	0	0	0	0
0	0	0	0	0	0	0	0	0	0	0	0	0	0	0	0	0	0
0	0	91	0	0	0	0	0	3764	0	203	0	0	0	0	0	0	0
0	0	909	0	85	82	210	0	0	980	129	0	23895	1355	1559	380	671	202
0	0	0	0	0	0	0	0	0	0	0	0	0	0	0	0	0	0
0	0	0	0	0	0	0	0	0	0	0	0	0	0	0	0	0	0
0	0	0	0	0	0	0	0	0	0	0	0	0	0	0	0	0	0
11900	0	0	0	0	0	0	0	0	0	0	0	0	0	0	0	0	0
0	0	0	0	0	0	0	0	3631	0	133	229	0	0	0	0	0	0
0	0	0	0	0	0	0	0	0	0	0	0	0	0	0	0	0	0
0	0	0	0	0	0	0	0	0	0	0	0	0	0	0	0	0	0
0	0	0	0	0	0	0	0	0	0	0	0	0	0	0	0	0	0
0	0	0	0	0	0	0	0	0	0	0	0	0	0	0	0	0	0
0	0	0	0	0	0	0	0	0	0	0	0	0	0	0	0	0	0
0	0	0	0	0	0	0	0	0	0	0	0	0	0	0	0	0	0
0	0	0	0	0	0	0	0	0	0	0	0	0	0	0	0	0	0
0	0	0	0	0	0	0	0	0	0	0	0	0	0	0	0	0	0
65724	92004	10235	0	0	18131	2905	1649	0	0	0	0	0	0	0	124	0	0
0	0	0	0	0	0	0	0	0	0	0	0	0	0	0	0	0	0
0	0	0	0	0	0	0	0	0	0	0	0	0	0	0	0	0	0
0	0	0	0	0	0	1694	0	0	0	0	0	0	0	0	0	0	0
0	0	0	0	0	0	0	0	0	0	0	0	0	0	0	0	0	0
0	0	0	0	0	0	0	0	0	0	0	0	0	0	0	0	0	0
0	0	0	0	0	441	0	0	0	0	0	0	0	0	0	0	0	0
0	2070	247	0	37	0	0	0	0	0	0	0	0	0	0	0	0	0
0	0	0	0	0	0	51	0	13783	60	107302	351	274	3122	980	1121	1037	2405
0	0	0	0	0	0	0	0	0	0	0	0	0	0	0	0	0	0
0	0	533	0	0	0	194	0	0	426	0	0	612	174	2538	652	545	69
0	0	0	0	0	0	0	0	0	0	0	0	0	0	0	0	0	0
0	0	0	0	0	0	0	0	0	0	0	0	0	0	0	0	0	0
0	0	0	0	0	0	0	0	0	0	0	0	0	0	0	0	0	0
0	0	0	0	0	0	0	0	0	0	0	0	0	0	0	0	348	0
0	0	0	0	0	0	0	0	0	0	0	0	0	356	0	0	0	0
0	0	0	0	0	0	0	0	0	0	0	0	0	0	0	0	0	0
0	0	0	0	0	0	0	0	0	0	0	0	0	0	0	0	0	0
0	11350	0	78	0	0	0	0	0	0	954	0	0	0	0	0	0	0
0	0	77	0	0	0	0	0	0	0	0	0	0	0	0	0	0	0
0	0	0	0	0	0	0	0	0	0	0	0	0	0	0	0	192	0
0	0	0	0	0	0	0	0	0	0	0	0	0	0	0	0	0	0
0	0	0	0	0	0	0	0	0	0	0	0	0	0	0	0	0	0
0	0	0	0	0	0	0	0	0	0	0	0	0	0	0	0	0	0
0	0	0	0	0	0	0	0	0	0	0	0	0	0	0	0	0	0
0	0	0	0	0	0	0	0	0	0	0	0	0	0	0	0	0	0
0	0	0	0	0	0	0	0	0	0	0	0	0	0	0	0	0	0
0	0	0	0	0	0	0	0	0	0	0	0	0	0	0	0	0	0
0	0	0	0	0	0	0	0	0	0	0	0	0	0	0	0	0	0
0	0	0	0	0	0	0	0	0	0	0	0	0	0	0	0	0	0
0	0	0	0	0	0	0	0	72	19558	1750	325	103035	68	2585	1540	24132	1147
0	0	0	0	0	0	0	0	0	0	0	0	0	0	0	0	0	0
0	0	0	0	0	0	0	0	0	0	0	0	0	0	0	0	0	0
0	0	0	0	0	0	0	0	0	0	0	0	0	0	0	0	0	0
0	0	0	0	0	0	0	0	0	0	0	0	0	0	0	0	0	0

0	0	0	0	0	0	0	0	0	0	0	0	0	0	0	0	0	0
0	0	0	0	0	0	0	383	0	0	75	0	0	0	0	0	0	0
0	0	0	0	0	0	0	0	0	0	0	0	0	0	0	0	0	0
0	0	0	0	0	36	64957	0	0	0	0	0	0	0	0	0	0	0
0	0	0	0	0	0	0	0	0	0	0	0	0	0	0	0	0	0
0	0	0	0	0	0	3170	0	0	0	0	0	0	0	0	0	1529	0
0	0	0	0	0	0	51	0	0	0	0	0	0	0	0	0	0	0
0	0	0	0	0	0	0	0	0	0	0	0	0	0	0	0	0	0
0	0	0	0	0	0	0	0	0	0	0	0	0	0	0	0	0	0
0	0	0	0	0	0	0	0	0	0	0	0	0	0	0	0	0	0
0	0	0	0	0	0	0	0	0	0	0	0	0	0	0	0	0	0
0	0	0	0	0	0	0	0	0	0	0	0	0	0	0	0	0	0
0	0	0	0	0	0	0	0	0	0	0	0	0	0	0	0	0	0
0	0	0	0	0	0	0	0	335	180	56	0	82	0	337	132	37616	144
0	0	0	0	0	0	0	0	0	0	0	0	0	0	0	0	0	0
0	0	0	0	0	0	0	0	0	0	0	0	0	0	0	0	0	0
0	0	0	0	0	0	0	0	0	0	0	0	0	0	0	0	0	0
0	0	0	0	0	0	0	0	0	0	0	0	0	0	0	0	0	0
0	0	0	0	0	0	0	0	121	0	0	0	0	250	0	0	0	0
0	0	0	0	0	0	0	0	0	0	0	0	0	0	0	0	0	0
0	0	0	0	0	0	0	0	0	0	0	0	0	0	0	0	0	0
0	0	0	0	0	0	0	0	137	0	0	0	0	0	0	0	0	0
0	0	0	0	0	0	0	0	0	0	0	0	0	0	0	0	0	0
0	0	0	0	0	0	0	0	0	0	0	0	0	0	0	0	0	0
0	0	0	0	0	0	0	0	0	0	0	0	0	0	0	0	0	0
0	0	0	0	0	0	0	0	0	0	0	0	0	0	0	0	0	0
0	0	0	0	0	0	0	0	0	0	0	0	0	0	0	0	0	0
0	0	0	0	0	0	0	0	0	0	0	0	0	0	0	0	0	72
0	0	0	0	0	0	0	0	0	0	0	0	86	0	0	130	44	0
0	0	0	0	0	0	0	0	0	0	0	0	0	0	0	0	0	0
52	0	0	0	0	0	0	0	0	0	0	0	0	0	0	0	0	0
0	0	0	0	0	0	0	0	0	54	0	0	549	51	0	0	548	152
0	0	0	0	0	0	0	0	0	0	0	0	0	0	0	0	0	0
0	0	0	0	0	0	0	0	0	0	0	0	0	0	0	0	0	0
0	0	0	0	0	0	0	0	0	0	0	0	0	0	0	0	0	0
0	0	0	0	0	0	0	0	0	0	0	0	0	0	0	0	0	0
0	0	0	1341	0	0	0	0	0	0	1722	0	0	0	0	0	0	0
0	0	0	0	0	0	0	0	0	0	0	0	0	0	0	0	0	0
0	0	0	0	0	0	0	0	0	125	0	0	0	0	0	0	0	0
0	0	0	0	0	0	0	0	0	0	0	0	0	0	0	0	0	0
0	0	0	0	0	0	0	0	0	0	0	0	0	0	0	0	0	0
0	0	0	0	0	0	0	0	0	0	0	0	0	0	0	0	0	0
0	0	0	0	0	0	0	0	0	0	0	0	0	0	0	0	0	0
0	0	0	0	0	0	0	0	0	0	0	0	82	0	0	0	0	0
0	0	0	0	0	0	0	0	0	0	0	0	0	0	0	0	0	0
0	0	0	0	0	0	0	0	0	0	0	0	0	0	0	0	0	0
0	0	0	0	0	0	0	0	0	0	0	0	0	0	0	0	0	0
0	0	0	0	0	0	0	0	0	0	0	0	0	0	0	0	0	0
0	0	0	0	0	57	8281	0	0	223	0	0	2395	0	646	368	86564	148
0	0	0	0	35	0	0	0	823	0	0	0	0	104	0	0	0	0
0	0	0	0	0	0	0	0	0	0	0	0	0	0	0	0	0	0
0	0	0	0	0	0	0	0	0	0	0	0	0	0	0	0	0	0
0	0	0	0	0	0	0	0	0	0	0	0	0	0	0	0	0	0
0	0	0	0	0	0	0	0	0	0	0	0	0	0	0	0	0	0
0	0	0	0	0	0	0	0	0	0	0	0	0	0	0	0	0	0
0	0	0	0	0	0	0	60	61	52	0	133	0	0	0	0	0	0
0	0	0	0	0	0	0	0	0	0	0	0	0	0	0	0	0	0
0	0	0	0	0	0	0	0	0	0	0	0	0	0	0	0	0	0
0	0	0	0	0	0	0	0	0	0	0	0	0	0	0	0	0	0
0	0	0	0	0	0	0	0	0	0	0	0	0	0	0	0	0	0
0	0	0	0	0	0	0	0	0	0	0	0	0	0	0	0	0	0
0	0	0	0	0	0	0	0	0	0	0	0	0	0	0	0	0	0
0	0	0	0	0	0	0	0	0	0	0	0	0	0	0	0	0	0
0	0	0	0	0	0	0	0	0	0	0	0	0	0	0	0	0	0
0	0	0	0	0	0	0	0	0	0	0	0	0	0	0	0	0	0
0	0	0	0	0	0	0	0	0	0	0	0	0	0	0	0	0	0
0	0	0	0	0	0	0	0	0	0	0	0	0	0	0	0	0	0
0	0	0	0	0	0	0	0	0	0	0	0	0	0	0	0	0	0
13004	11863	1488	0	0	6028	0	798	0	0	129	0	0	0	0	0	0	0
0	0	0	0	0	0	0	0	0	0	0	0	0	0	0	0	0	0
0	2852	0	0	0	0	0	0	0	0	0	0	0	0	0	0	0	0
0	0	0	0	0	0	0	0	0	0	0	0	0	0	0	0	0	0
0	0	0	0	0	0	0	0	0	0	0	0	0	0	0	0	0	0
0	0	0	0	0	0	0	0	0	0	0	0	0	0	0	0	0	0
0	0	0	0	0	0	0	0	0	0	0	0	0	0	0	0	0	0
0	0	0	0	0	0	0	0	0	232	61	0	644	186	0	267	360	81
0	0	0	0	0	0	0	0	0	0	0	0	0	0	0	0	0	0
0	0	0	0	0	0	0	0	0	0	0	0	0	0	0	0	0	0
0	0	0	0	0	0	0	0	0	0	0	0	0	0	0	0	0	0
0	0	0	0	0	0	0	0	0	0	72	0	0	0	0	0	0	0
183904	151	163	0	53	218	187738	322	0	0	398	0	0	0	0	0	0	0
0	0	0	0	0	230	414192	0	0	0	0	0	0	0	0	0	0	0
0	0	0	0	0	0	0	0	0	0	0	0	0	0	0	0	0	0
0	0	0	0	0	0	0	0	0	0	0	0	0	0	0	0	0	0
0	8536	0	8063	0	0	4143	128	0	57	0	105	76	0	0	0	1799	0
0	2234	0	0	0	0	0	0	0	0	0	0	0	0	0	0	0	0

0	0	0	0	0	0	0	0	0	0	0	0	0	0	0	0	0	0
0	0	0	0	0	0	0	0	0	0	0	0	0	0	0	0	0	0
0	0	0	0	0	0	0	0	0	0	0	0	0	0	0	0	0	0
0	0	0	0	0	0	0	0	0	0	0	0	0	0	0	0	0	0
0	0	0	0	0	0	0	0	0	0	0	0	0	0	0	0	0	0
0	0	0	0	0	0	0	0	0	0	0	0	0	0	0	0	0	0
0	0	0	0	0	0	0	0	0	0	0	0	0	0	325	0	0	0
0	0	0	0	0	0	0	0	0	0	0	0	0	0	0	0	0	0
0	0	0	0	0	0	0	0	0	0	0	0	0	0	0	0	0	0
0	0	0	0	0	0	0	0	0	0	0	0	0	0	0	0	0	0
0	0	0	0	0	0	3413	0	0	0	0	0	0	0	0	0	0	0
0	0	0	0	0	0	0	0	0	0	0	0	168	0	0	0	6859	0
0	0	0	0	0	0	0	0	0	47	0	0	186	0	0	0	3872	0
0	0	0	0	0	0	0	0	0	0	0	0	0	191	0	0	0	0
0	3931	0	0	0	0	0	0	0	0	0	0	0	0	0	0	1148	0
0	0	0	0	0	0	0	0	0	0	0	0	0	0	0	0	0	0
0	0	0	0	0	0	0	0	0	0	0	0	0	0	0	0	0	0
0	0	0	0	0	0	0	0	0	0	0	0	0	0	0	0	3041	0
0	0	0	0	0	0	0	0	0	0	0	0	0	0	0	0	0	0
0	0	0	0	0	0	0	0	0	0	0	0	0	0	0	0	0	0
0	0	0	0	0	0	0	0	0	0	0	0	0	0	0	0	0	0
0	0	0	0	0	0	0	0	0	0	0	0	0	0	0	0	5827	144
0	0	0	0	0	0	0	0	0	0	0	0	0	0	0	0	0	0
0	0	0	0	0	0	0	0	0	0	0	0	0	0	0	0	0	0
0	0	0	0	0	0	0	0	0	104	0	0	160	0	0	0	43	0
0	0	0	0	0	0	0	0	0	0	0	0	0	0	0	0	0	0
0	0	0	0	0	0	0	0	0	0	0	0	0	0	0	0	0	0
0	0	0	0	0	0	0	0	0	0	0	0	0	0	0	0	0	0
0	0	0	0	0	0	0	0	0	0	0	0	0	0	0	0	0	0
0	0	0	0	0	0	0	0	0	0	0	0	0	0	0	0	0	0
0	0	0	0	0	0	0	0	0	0	0	0	0	0	0	0	0	0
0	0	0	0	0	0	0	0	0	0	0	0	0	0	0	0	0	0
961	325601	762124	7393	2512	24620	64773	96422	27920	2856	79389	946231	12342	848304	11180	323017	202659	969842
0	0	0	0	0	0	0	0	0	0	0	0	0	0	0	0	0	0
0	0	0	0	0	0	0	0	0	0	0	0	0	0	0	0	2724	0
0	0	0	0	0	0	0	0	0	0	0	0	0	0	0	0	0	0
0	2005	1592	0	0	0	0	109	0	0	0	0	172	0	0	0	0	0
0	404	887	0	0	0	0	0	0	0	0	0	0	0	0	0	0	0
0	0	0	0	0	0	0	0	0	0	0	0	0	0	0	0	0	0
0	0	0	0	0	0	0	0	0	0	0	0	0	0	0	0	0	0
0	0	0	0	0	0	0	0	0	0	263	0	0	3289	77329	0	427	0
0	0	0	0	0	0	0	0	0	0	0	0	0	0	0	0	0	0
0	0	0	0	0	0	0	0	0	0	0	0	0	0	0	0	0	0
0	0	0	0	0	0	0	0	0	0	0	0	0	0	0	0	0	0
0	0	0	0	0	0	0	0	0	0	0	0	0	0	0	0	0	0
0	0	0	0	0	0	0	0	0	0	0	0	0	0	0	0	0	0
0	0	0	0	0	0	0	0	0	0	0	0	0	0	0	0	0	0
0	0	0	0	0	0	0	0	0	0	0	0	0	0	0	0	49	0
0	0	0	0	42	0	0	0	121	4205	130	229	19601	2412	1290	993	2094	1462

COVID -19-041	COVID -19-039	COVID -19-038	COVID -19-052	COVID -19-044	COVID -19-053	COVID -19-065	COVID -19-055	COVID -19-071	COVID -19-070	COVID -19-069	COVID -19-068	COVID -19-067	COVID -19-066	COVID -19-037	COVID -19-064	COVID -19-063	COVID -19-062
536890	951716	961670	962205	951581	892378	984402	850777	765911	788877	878220	844313	969781	787890	882917	897183	945099	752697
456340	19829	11107	6442	18340	47330	2339	97610	233043	186949	97717	119314	843	158246	93773	83317	41688	229951
0	0	0	512	0	101	0	19422	0	0	52	0	28575	0	0	0	0	0
6770	26658	25136	26095	27073	60067	13161	28278	989	24121	24011	36326	374	53702	6396	19189	12998	17086
0	1797	2087	4746	3006	0	98	3913	57	53	0	47	427	162	16914	311	156	266
0	0	0	0	0	124	0	0	0	0	0	0	0	0	0	0	0	0
0	0	0	0	0	0	0	0	0	0	0	0	0	0	0	0	59	0
COVID -19-041	COVID -19-039	COVID -19-038	COVID -19-052	COVID -19-044	COVID -19-053	COVID -19-065	COVID -19-055	COVID -19-071	COVID -19-070	COVID -19-069	COVID -19-068	COVID -19-067	COVID -19-066	COVID -19-037	COVID -19-064	COVID -19-063	COVID -19-062
0	320	314	0	0	0	0	0	0	0	0	0	0	0	0	0	0	0
0	0	0	0	0	0	0	0	0	0	0	0	0	0	0	0	57	0
48432	725993	803919	49770	785211	444774	14167	2094	10573	2176	319571	106370	3256	411820	137272	669355	47019	32317
0	0	0	0	0	0	0	0	0	0	0	0	0	0	0	0	0	0
0	0	0	0	0	0	0	0	0	0	0	0	0	0	0	0	0	0
0	0	0	0	0	0	0	0	0	0	0	589	0	0	0	0	0	0
0	0	0	0	0	0	0	0	0	0	0	0	0	0	0	0	0	0
0	0	0	0	0	0	0	0	0	58	0	0	0	91	0	37	0	35121
0	87	58	0	190	0	0	0	0	0	0	0	0	0	0	0	0	0
0	0	0	0	0	0	0	0	0	0	0	0	0	0	0	0	0	0
0	0	0	0	0	0	0	0	0	0	0	0	0	0	0	0	0	0
0	659	1409	13587	190	0	74	115	0	0	0	0	82	101	3535	45	801	23731
0	0	0	0	0	0	0	0	0	0	0	50	0	0	0	0	0	0
0	0	0	0	0	0	0	0	0	0	0	0	0	0	0	0	0	0
0	0	0	0	0	0	0	0	0	0	0	0	0	0	0	0	0	0
194	14453	15048	399	13683	4071	244	0	18	0	127	0	0	7762	1304	7745	1105	5482
0	0	0	0	0	0	0	0	547	0	0	0	0	0	0	0	0	0
0	0	0	0	0	0	0	0	0	0	0	0	0	0	0	0	0	0
0	0	0	0	0	0	0	0	0	0	0	0	0	0	0	2562	0	0
0	92	0	0	107	0	0	0	0	0	0	0	0	0	0	0	0	0
0	0	0	0	0	0	0	0	0	0	0	0	0	0	0	0	0	0
0	0	0	0	0	0	0	0	0	0	0	0	0	0	0	0	0	0
0	0	0	0	0	0	0	0	0	0	0	0	0	0	0	0	0	0
0	0	0	0	0	0	0	0	0	0	0	0	0	0	0	0	0	0
0	500	51	0	0	0	165	0	0	0	710	102	0	302	0	37	104	68
0	0	0	0	0	0	0	0	0	0	0	0	0	0	0	0	0	0
0	0	0	0	0	0	0	0	0	0	0	0	0	0	108	40	0	509
0	0	0	0	0	0	0	0	0	0	0	0	0	0	0	0	0	0
33163	0	0	0	0	0	0	0	0	0	0	0	0	0	0	0	0	0
22682	6903	5254	21055	6233	17123	824809	1133	33349	165	24592	81339	850989	24973	113640	20358	446752	268784
0	0	0	0	0	0	0	0	0	0	0	0	0	0	0	0	0	0
90164	0	0	0	95	0	84	0	0	87614	49	0	68	0	0	0	15225	0
0	0	0	0	0	0	0	0	0	0	53	24090	80	0	0	0	0	0
0	0	0	0	0	0	0	0	0	0	0	0	0	0	0	0	0	0
0	0	0	0	0	0	0	0	0	0	0	0	0	0	0	0	0	0
0	0	0	0	0	0	0	0	0	0	0	0	0	0	0	0	0	0
0	0	0	0	0	0	0	0	0	0	0	0	0	0	0	0	0	0
0	0	0	0	0	0	0	0	0	0	0	0	0	0	0	0	0	0
0	0	0	0	0	0	0	0	0	0	0	0	0	0	0	0	0	0
0	0	0	0	0	0	0	0	0	0	0	0	0	0	133	0	0	0
0	90	0	0	403	113	0	0	22	0	0	0	0	710	0	66	0	65
0	0	0	0	0	0	0	0	0	0	0	0	0	0	0	0	0	0
0	0	0	0	0	0	0	0	0	0	0	0	0	0	0	0	0	0
0	0	0	0	0	0	0	0	0	0	0	0	0	0	0	0	0	0
0	0	0	0	0	0	0	0	0	0	0	0	0	0	0	0	0	0
0	0	0	0	0	0	0	23767	16001	0	0	0	0	0	0	0	0	0
0	0	0	311	0	86539	0	0	0	0	0	0	0	0	0	0	0	0
430	466	365	13062	225	1102	99	1927	139	164	6394	11452	226	132	252816	25581	188	75077
0	0	0	0	0	0	0	0	0	0	0	0	0	0	0	0	0	0
0	0	0	0	0	0	0	0	0	0	0	0	55	0	15741	0	0	0
0	0	0	0	0	0	0	0	0	0	0	0	0	0	0	0	0	0
0	0	0	0	0	0	0	0	0	0	0	0	0	0	0	0	0	0
0	0	0	0	0	0	0	0	0	0	0	0	0	0	0	0	1676	32
448	332	0	0	0	0	0	0	0	0	0	0	0	0	0	0	5753	0
0	0	0	0	0	0	0	0	0	0	0	0	0	0	0	0	0	0
0	0	0	0	0	0	0	0	0	0	0	0	0	0	0	0	0	0
0	0	0	0	0	0	0	0	0	0	0	0	0	0	0	0	0	0
0	1142	1696	0	1057	116	0	0	28	0	0	0	0	0	0	29	0	0
0	0	0	0	0	0	0	0	0	0	0	0	0	0	0	0	0	0
0	0	0	0	0	0	0	0	0	0	0	0	0	0	0	0	0	0
6332	1703	2090	6929	2368	9385	87	4241	532	575	19498	449	102437	80057	210569	662	255	31
0	0	55	0	0	0	0	0	0	0	0	0	0	0	0	0	0	0
0	0	0	0	0	0	0	0	0	0	0	0	0	0	0	0	0	0
0	0	0	0	0	0	0	0	0	0	0	0	0	0	0	0	0	0
0	0	0	0	0	0	0	0	0	0	0	0	0	0	0	0	0	0
0	0	0	0	0	0	0	0	0	0	0	0	0	0	0	0	0	0
0	0	73	0	0	0	0	0	0	0	0	0	0	5003	0	0	0	0
0	0	0	0	0	0	0	0	0	0	0	0	0	0	0	0	0	0
0	0	0	0	0	0	0	0	0	0	0	0	0	0	0	0	0	0

0	0	0	0	0	0	0	0	0	0	0	0	0	0	0	0	0	0
0	0	0	0	0	0	0	0	0	0	0	0	0	0	0	0	0	0
0	0	144	0	0	0	0	0	0	0	0	0	0	0	0	0	0	0
0	0	0	0	0	0	0	0	0	0	0	0	0	0	0	0	0	0
0	0	0	0	0	0	0	0	0	0	0	0	0	0	0	0	0	0
0	0	0	0	0	0	0	0	0	0	0	0	0	0	0	0	0	0
2908	358	385	0	919	0	0	0	0	0	0	36	0	26	7822	0	0	0
0	0	0	0	0	0	0	0	0	0	0	0	0	0	0	0	0	0
0	0	0	512	0	0	0	107	0	0	52	0	28575	0	0	0	0	0
0	0	0	0	0	0	0	0	0	0	0	0	0	0	0	0	0	0
0	0	0	0	0	0	0	0	0	54	0	0	0	0	0	43	0	0
0	0	0	0	0	0	0	0	0	0	0	0	0	0	0	0	0	0
307389	1344	1214	11742	1041	53467	1059	122	260494	402504	171593	479991	521	18929	66944	40075	23306	57992
0	0	0	0	0	0	0	0	0	0	0	0	0	252	0	0	0	0
0	0	0	0	0	0	0	0	0	0	0	0	0	0	0	0	0	0
0	0	0	0	0	0	0	0	0	0	0	0	0	0	0	0	0	0
0	0	0	0	0	0	0	0	0	0	0	0	0	0	0	0	0	0
0	0	0	0	0	0	0	0	0	0	0	0	0	0	0	0	0	0
0	0	0	0	0	0	0	0	0	0	0	0	0	0	0	0	0	0
0	0	0	0	0	0	0	0	0	0	0	0	0	0	0	0	0	0
0	0	0	0	0	0	0	0	0	0	0	0	0	0	0	0	0	0
0	0	0	0	0	0	0	0	0	0	70	0	0	0	0	0	0	0
85799	78	62	0	189	0	0	0	0	0	0	0	0	0	0	0	0	0
0	0	0	0	0	0	0	0	0	0	0	0	0	0	0	0	0	0
0	251	85	0	299	0	0	0	0	0	0	0	0	333	0	138	0	0
0	0	0	0	0	0	0	0	0	0	0	0	0	0	0	0	0	0
0	0	0	0	0	0	0	0	0	0	0	0	0	0	0	0	0	0
0	82	161	0	92	110	0	0	0	52	0	91	0	21	0	73	0	0
0	0	0	0	0	0	0	0	0	0	0	0	0	0	0	0	0	0
0	0	0	0	0	0	0	0	0	0	0	0	0	0	0	0	0	0
0	0	0	0	100	0	0	0	0	0	0	0	0	0	0	0	0	0
0	0	0	0	0	0	0	0	0	0	0	0	0	0	0	0	0	0
0	0	0	0	0	0	0	0	0	0	0	0	0	0	0	0	0	0
0	0	0	95	0	0	0	0	0	0	0	0	0	0	0	0	0	0
0	0	0	1029	0	0	0	0	0	0	0	0	0	3253	0	0	0	0
0	0	0	0	0	0	0	0	0	0	0	0	0	0	0	0	0	0
0	0	0	0	0	0	0	0	0	0	0	0	0	0	0	0	0	0
0	0	0	0	0	0	92	0	0	0	0	0	0	0	680	0	0	6698
0	0	0	0	0	0	0	0	0	0	0	0	0	0	0	0	0	0
0	0	0	0	0	0	0	0	0	0	0	0	0	0	0	0	0	0
0	0	0	0	0	0	0	0	0	0	0	0	0	0	0	0	0	0
0	0	0	0	0	0	0	0	0	0	0	0	0	0	121	0	0	0
0	0	0	0	0	0	0	0	0	0	0	0	0	0	0	0	0	0
0	0	0	0	0	0	0	0	0	0	0	0	0	0	0	0	0	0
0	0	0	0	0	0	0	0	0	0	23803	0	0	0	0	38	0	0
0	0	0	0	0	0	0	0	0	0	0	0	0	0	0	0	0	0
0	0	0	0	0	0	0	0	0	0	0	0	0	0	0	0	0	0
213	0	0	0	0	0	0	0	0	0	0	0	0	0	0	0	0	0
0	0	0	0	0	0	0	0	0	0	50608	0	0	0	0	0	0	0
0	0	0	0	0	0	0	0	0	0	0	0	0	0	0	0	0	0
0	1797	2087	4746	3006	0	98	3913	57	53	0	47	427	162	16544	311	104	266
0	0	0	0	0	0	0	0	0	0	0	0	0	0	0	0	0	0
0	0	0	0	0	0	0	0	0	0	0	0	0	0	0	0	0	0
0	0	0	0	0	0	0	0	0	0	0	0	0	0	0	0	0	0
0	0	0	0	0	0	0	0	0	0	0	0	0	0	0	0	0	0
0	0	0	0	0	0	0	0	0	0	0	0	0	0	0	0	0	0
0	0	0	0	0	0	0	0	0	0	0	0	0	0	0	0	0	0
0	0	0	0	0	0	0	0	0	0	0	0	0	0	0	0	0	0
0	0	0	0	0	0	0	0	0	0	0	0	0	0	0	0	0	0
0	0	0	0	0	0	0	0	0	0	0	0	0	0	0	0	0	0
0	0	0	0	0	6905	0	0	230	0	4278	0	0	0	0	0	0	0
0	0	0	0	0	0	0	0	0	0	0	0	0	0	0	0	0	0
0	243	169	0	0	0	0	0	0	0	0	0	0	17	137	1987	118	151
0	0	0	0	0	0	0	0	0	0	0	0	0	0	0	0	0	0
0	0	0	0	0	0	0	0	0	0	0	0	0	0	0	0	0	0
0	0	0	0	0	0	0	0	0	59	4264	0	0	0	0	0	0	0
0	0	0	0	0	0	0	0	0	0	0	0	0	0	0	0	0	0
0	0	0	0	0	0	0	0	0	0	0	0	0	7907	0	0	575	0
0	0	0	0	0	0	0	0	0	0	0	0	0	0	0	0	0	0
0	0	0	0	0	0	0	0	0	0	0	0	0	0	0	0	0	0
0	0	0	0	0	0	0	0	0	0	0	0	0	0	0	0	0	0
0	0	0	0	0	8143	0	0	8156	48	0	0	0	0	0	0	0	0
0	0	0	0	0	0	0	0	0	0	0	0	0	0	0	0	0	0
0	0	0	0	0	0	0	0	0	0	0	0	0	0	0	0	0	0
0	9843	9873	94	12205	34498	228	0	29	0	51	0	0	3183	248	13979	111	180
898	16657	8657	391	15143	1387	854	0	21	0	384	47	76	55955	127	66328	2554	3748
0	0	46	0	0	0	0	0	31	0	0	0	0	0	23180	0	14781	0
0	0	0	0	0	0	0	0	0	0	0	0	0	0	0	0	0	0
0	0	0	0	0	0	0	0	0	0	0	0	0	0	0	0	0	0
0	0	0	0	0	0	0	0	0	0	0	0	0	0	0	0	0	0
0	0	0	0	0	0	0	0	0	0	0	0	0	0	0	0	0	0
0	0	0	0	0	0	0	0	0	0	0	0	0	0	0	0	0	0
0	0	0	0	0	0	0	0	0	0	0	0	0	0	0	0	0	0
6770	26415	24967	26095	27073	60067	13161	28278	989	24121	24011	36326	374	53685	6259	17202	12880	16935
0	0	0	0	109	0	0	0	0	0	0	88	0	27	409	0	0	0
0	0	0	0	0	0	0	0	0	0	0	0	0	0	125	0	0	352
0	0	0	0	0	0	0	0	0	0	0	0	0	0	0	0	0	0

0	0	0	0	0	124	0	0	0	0	0	0	0	0	0	0	0	0
0	0	0	0	0	0	152	0	0	0	124	0	0	214	0	0	260137	87
0	0	0	0	0	0	0	0	0	0	0	0	0	0	0	0	0	0
0	0	0	177	0	0	0	62	0	0	0	0	0	0	0	0	0	0
0	0	0	0	0	0	0	0	0	0	0	0	0	0	0	0	0	0
0	0	0	0	0	0	0	0	0	0	0	0	0	0	0	0	0	0
0	0	0	0	0	0	0	0	0	0	0	0	0	0	0	0	51	0
224	85	107	0	366	112	77	52	0	0	50	69246	0	11088	11952	0	0	44
0	89	0	0	0	0	0	0	0	0	0	0	0	287	0	0	0	0
0	0	0	0	0	0	0	0	0	0	0	0	0	0	0	0	0	0
0	0	0	0	0	0	0	0	0	0	0	0	0	0	0	0	0	0
0	0	0	0	0	0	0	0	0	0	0	0	0	0	370	0	0	0
0	0	0	0	0	0	0	0	0	0	0	0	0	0	147	0	0	0
0	0	0	0	0	0	0	0	0	0	0	0	0	0	272	0	0	0
0	0	0	0	0	0	0	0	0	0	0	0	0	0	0	0	0	0
0	0	0	0	0	0	0	0	0	0	0	0	0	0	0	0	0	0
0	0	0	0	0	0	0	0	0	0	0	0	0	0	0	0	0	0
0	0	0	0	0	0	0	0	0	0	0	0	0	0	0	0	0	0
0	0	0	0	0	0	0	0	0	0	0	0	0	0	0	0	0	0
0	0	0	0	0	0	0	0	0	0	0	0	0	0	0	0	0	0
228	80	52	0	0	0	0	0	0	0	0	0	0	0	4646	0	0	1485
0	0	0	0	0	0	0	0	0	0	0	0	0	0	0	0	0	0
0	0	0	0	0	0	0	0	0	0	0	0	0	0	0	0	0	0
229	726	360	0	496	0	0	0	0	0	12309	0	0	33	0	0	0	69
0	0	0	0	0	0	0	0	0	0	0	0	0	0	0	0	0	0
0	0	0	0	0	0	0	0	0	0	0	0	0	0	0	0	0	0
0	0	0	0	0	0	0	0	0	0	0	0	0	0	124	0	0	0
0	0	0	0	0	0	0	0	0	0	0	0	0	0	0	0	0	0
0	0	0	0	0	0	0	0	0	0	0	0	0	0	0	0	0	0
216	89	0	0	0	0	0	0	0	0	0	0	0	0	0	0	0	0
0	0	0	0	0	0	0	0	0	0	0	0	0	0	0	0	0	0
0	0	0	0	0	0	0	0	0	0	0	0	0	0	0	0	0	0
0	0	0	0	0	0	0	0	0	0	0	0	0	0	0	0	63	37645
0	0	0	0	0	0	0	0	0	0	0	0	0	0	0	0	0	0
0	0	0	0	0	0	0	0	0	0	0	0	0	0	0	0	0	0
0	322	589	45523	102	112	0	16200	275	380	374	185	3457	21	238	23	0	0
0	0	0	0	0	0	0	0	0	0	0	0	0	0	0	0	0	0
0	0	0	0	0	0	0	0	0	0	0	0	0	0	0	0	0	0
0	0	0	0	0	0	0	0	0	0	0	0	0	0	0	0	0	0
0	0	0	0	0	0	0	0	0	0	0	0	0	0	0	0	0	0
0	0	0	0	0	0	0	0	0	0	0	0	0	0	0	0	0	0
0	0	0	0	0	0	0	0	0	0	0	0	0	0	0	0	0	0
0	0	0	0	0	0	0	0	0	0	0	0	0	0	0	0	0	0
116687	90	102	0	0	0	0	0	0	0	0	0	0	0	0	772	0	0
0	0	0	100	0	0	0	0	0	0	0	0	0	0	135	0	0	0
0	0	0	0	0	0	0	0	0	0	0	0	0	0	0	0	0	0
0	0	0	0	0	0	0	0	0	0	0	0	0	0	0	0	0	0
0	0	0	0	0	0	0	0	0	0	0	0	0	0	0	0	0	0
0	592	257	392	423	0	232	1074	0	0	0	0	0	35341	136	0	0	37
0	0	0	0	0	0	0	0	0	0	0	0	0	0	0	0	0	0
0	0	0	0	0	0	0	0	0	0	0	0	0	0	0	0	0	0
0	0	0	0	0	2540	0	67	0	0	0	0	0	0	0	0	0	0
0	0	0	0	0	0	0	0	0	0	0	0	0	0	0	0	0	0
0	0	0	0	0	0	0	0	0	48	0	0	0	0	1977	0	0	0
0	0	0	0	0	0	0	0	0	0	0	0	0	0	3264	0	0	0
0	0	0	0	0	0	0	0	0	0	0	0	0	0	0	0	0	0
0	0	0	0	0	0	0	0	0	0	0	0	0	0	0	0	0	0
0	0	0	0	0	0	0	0	0	0	0	0	0	0	0	0	0	0
0	0	0	0	0	0	0	0	0	0	0	0	0	0	0	0	0	0
0	0	0	0	0	0	0	0	0	0	0	0	0	0	0	0	0	0
0	0	0	0	0	0	0	0	0	0	0	0	0	0	0	0	0	0
0	0	0	0	0	0	0	0	0	0	0	0	0	0	0	0	0	0
0	0	0	98	0	0	0	0	0	25575	0	0	0	0	0	0	0	0
0	0	0	0	0	0	0	0	0	0	0	0	0	0	0	0	0	0
0	0	0	0	0	0	0	0	0	0	0	0	0	0	0	0	0	0
0	0	0	0	0	0	0	0	0	0	0	0	0	0	0	0	0	0
0	0	0	0	0	0	0	0	0	0	0	0	0	0	0	0	0	0
653	581	635	0	581	0	0	0	0	0	0	0	0	0	0	0	0	0
0	0	0	0	0	0	0	0	0	0	0	0	0	0	0	0	0	0
0	0	0	0	0	0	0	0	0	0	0	0	0	0	0	0	0	0
0	0	0	0	0	0	0	0	0	0	0	0	0	0	107	0	0	0
0	0	0	0	0	0	0	0	0	0	0	0	0	0	0	0	0	0
0	0	0	0	0	0	0	0	0	0	0	0	0	0	0	0	0	0
73280	288	47	3506	101	14073	718	40998	140177	75996	40434	298	387	46865	42902	7919	15924	179036
0	0	0	0	0	0	0	0	0	0	0	0	0	0	0	0	0	0
0	0	0	0	0	0	0	0	11927	0	0	0	0	0	0	0	0	0
0	0	0	0	0	0	0	0	0	0	0	0	0	0	0	0	0	0
0	0	0	0	0	0	0	0	0	0	0	0	0	0	0	0	0	0
0	751	943	0	679	0	0	0	0	58	0	0	0	93	394	126	67	0
0	0	0	0	0	2165	0	0	0	0	0	0	68	0	0	0	0	0
0	0	0	0	0	101	0	19315	0	0	0	0	0	0	0	0	0	0
0	0	0	0	0	0	0	0	0	0	0	0	0	0	0	0	0	0
0	0	0	0	0	0	0	0	0	0	0	0	0	0	0	0	0	0
0	0	0	0	0	0	0	0	0	0	0	0	0	0	0	0	52	0
0	0	0	0	0	0	0	0	0	0	0	0	0	0	0	0	0	0
219	80	0	695	0	0	0	0	9106	0	0	0	0	0	0	0	0	0
0	0	0	0	0	0	0	0	0	0	0	0	0	0	0	0	0	0

0	0	0	0	0	0	0	0	28	103	0	0	72	4579	0	75	0	0
0	0	0	0	0	0	0	0	0	0	0	0	0	0	0	0	0	0
0	0	0	0	0	0	0	0	0	0	0	0	0	0	0	0	0	4175
0	0	0	0	0	0	0	0	0	0	0	0	0	0	0	0	0	0
0	0	0	0	0	0	0	0	0	0	0	0	0	0	0	0	0	0
0	0	0	0	0	0	0	0	0	0	0	0	0	0	0	0	0	0
0	0	0	0	0	0	0	0	0	0	0	0	0	0	0	0	0	0
0	0	0	0	0	0	0	0	0	0	0	0	0	0	0	0	0	0
0	0	0	116	0	101	0	14534	0	0	0	0	0	0	124	0	0	0
0	0	0	0	0	0	0	0	0	0	0	0	0	0	0	0	0	0
0	0	0	0	0	0	0	0	0	0	0	0	0	0	0	0	0	0
0	0	52	0	0	0	0	0	0	0	0	0	0	0	0	0	0	0
0	0	0	0	0	0	0	0	0	0	5977	38	0	0	124	0	0	0
0	64	0	4612	0	0	0	0	57989	0	0	0	0	0	0	0	0	0
0	0	0	0	0	0	0	0	0	0	0	0	0	0	0	0	0	0
0	0	59	105	0	30132	205	0	0	0	0	0	0	139	25248	0	51	31
0	0	52	0	0	0	0	0	0	0	0	0	0	0	0	0	0	0
0	0	0	0	0	0	0	0	0	0	0	0	0	0	0	0	0	0
0	0	0	698	0	0	0	0	0	0	0	0	0	0	0	0	0	0
0	0	0	0	0	0	0	0	0	0	0	0	0	0	0	0	0	0
0	0	0	0	0	0	0	0	0	0	0	0	0	0	0	0	0	0
0	0	0	0	0	0	0	0	0	0	0	0	0	0	0	0	0	0
0	527	565	0	484	0	89	0	0	0	0	0	0	0	0	30	56	0
0	0	0	0	0	0	0	0	0	0	0	0	0	4242	0	0	0	0
0	0	0	0	0	0	0	0	0	0	0	0	0	0	0	0	0	0
0	0	0	0	0	0	0	0	0	0	0	0	0	0	0	0	0	0
0	0	0	0	0	0	0	0	0	0	0	0	0	0	0	0	0	0
851	9854	9515	700	10455	4996	69167	340	210	0	291	91	75	6179	399	7448	10730	9132
0	0	0	0	0	0	0	0	0	0	0	0	0	0	0	0	0	0
0	0	0	0	0	0	0	0	0	0	0	0	0	0	0	0	0	0
0	0	0	0	0	0	0	0	0	0	0	0	0	0	0	0	0	0
0	0	0	0	0	0	0	0	0	0	0	0	0	0	0	0	0	0
0	0	0	0	0	0	0	0	0	0	0	0	0	0	0	0	0	0
0	0	0	0	0	0	0	0	0	0	0	0	0	0	0	0	0	0
0	2166	496	528	1430	220	0	0	0	0	0	0	0	67	878	255	0	278
0	0	0	0	0	0	0	0	0	0	0	0	0	0	0	0	0	0
0	0	0	0	0	0	0	0	0	0	0	0	0	0	0	2818	0	0
0	168	400	0	301	0	0	0	0	0	0	0	0	0	0	0	0	0
0	0	0	0	0	0	0	0	131	126128	32759	13313	84	0	264	100	6288	0
0	0	0	0	0	0	0	0	0	0	0	0	0	0	0	0	0	0
88106	0	57	0	0	0	0	2096	0	0	0	0	0	0	0	0	0	0
0	0	0	0	0	0	0	0	0	0	0	0	0	0	0	0	0	0
0	434	367	0	962	0	0	0	0	0	0	0	0	0	0	114	0	0
0	0	0	0	0	0	0	0	0	0	0	25640	0	0	0	45	0	0
0	0	0	0	0	0	0	0	0	0	0	0	0	0	0	0	0	0
0	0	0	0	0	0	0	0	0	0	663	0	0	0	0	0	0	0
0	0	0	0	0	0	0	0	0	0	587	0	0	0	0	0	0	0
0	0	0	0	0	0	0	0	0	0	11106	0	84	0	0	0	0	0
0	0	0	0	0	0	0	0	0	0	0	0	0	0	0	0	767	0
0	0	0	0	0	0	0	0	0	0	0	0	0	0	0	0	0	0
0	0	0	0	0	0	0	0	0	0	0	0	0	0	0	0	4852	0
0	0	0	0	0	0	88	0	0	0	0	0	0	13163	0	0	61	0
0	0	0	0	0	0	0	0	0	0	0	0	0	0	0	0	0	0
0	0	0	0	0	0	0	0	0	0	0	0	0	0	0	0	0	0
0	0	0	0	0	0	0	0	0	85220	0	0	0	0	0	0	0	0
0	0	0	0	0	0	0	0	0	0	0	0	0	0	0	0	0	0
0	0	0	0	0	0	0	0	0	0	0	0	0	0	0	0	0	0
0	437	171	0	415	0	0	0	0	0	0	0	0	0	0	0	0	0
0	0	0	0	0	0	0	0	0	0	0	0	0	0	0	0	0	0
0	0	0	0	0	0	0	0	0	0	0	0	0	0	0	0	0	0
221	0	146	0	186	0	0	0	0	0	23953	0	0	0	125	0	0	0
0	0	38	0	0	0	0	0	0	0	0	0	0	0	0	0	0	36
0	0	0	0	0	0	0	0	0	0	0	54	0	0	0	37	0	29
88257	77	0	0	94	108	0	30446	0	0	31554	0	0	0	1256	44	0	0
0	0	0	0	0	0	0	0	0	0	0	0	0	0	0	0	0	0
0	0	0	0	0	0	0	0	0	0	0	0	0	0	0	0	0	0
0	0	0	0	0	0	0	0	0	0	0	0	0	0	0	0	0	0
0	0	0	0	0	0	0	0	0	0	0	0	0	0	0	0	0	0
0	0	0	0	0	0	0	0	30578	0	0	0	0	0	0	0	0	0
0	0	0	0	0	0	0	0	0	0	0	0	0	0	0	0	0	0
0	0	0	0	0	0	0	0	0	0	0	0	0	0	0	0	0	0
0	0	0	0	0	0	0	0	0	0	0	0	0	0	0	0	0	0
0	0	0	0	0	0	0	0	0	0	12666	0	0	0	0	0	0	0
0	0	0	0	0	0	0	0	20	0	0	0	0	0	4279	0	0	0
0	0	0	0	0	0	0	0	0	0	0	0	0	0	0	0	0	0
0	0	0	0	0	0	0	0	0	0	0	0	0	0	0	0	0	0
0	85	0	0	0	0	94	55	0	166993	52	0	0	0	0	34	2435	0
0	0	0	0	0	0	0	0	0	0	0	0	0	0	0	0	0	0
0	0	0	0	0	0	0	0	0	0	0	0	0	0	0	0	0	0
0	0	0	0	0	0	0	0	0	0	0	0	0	0	0	0	0	0
0	0	0	0	0	0	0	0	0	0	0	0	0	0	0	0	0	0
0	0	0	0	0	0	0	0	0	0	0	0	0	0	0	0	0	0
0	0	0	0	0	0	0	0	0	0	0	0	0	0	0	0	0	0
0	0	0	0	0	0	0	0	0	0	0	0	0	0	0	0	0	0
0	0	0	0	0	0	0	0	0	0	0	0	0	0	0	0	0	0
0	0	0	0	0	0	0	0	0	0	0	0	0	0	0	0	0	0
227	14548	6502	7432	4136	6760	234	0	3438	461	6417	429	731	210	1656	12068	862	471

0	0	0	0	0	0	0	0	0	0	0	0	0	0	0	0	0	0
0	0	0	0	0	0	580	0	0	0	0	0	0	273	0	0	125	132699
0	0	0	0	0	0	0	0	0	0	0	0	0	0	0	0	0	0
0	0	0	0	0	0	0	0	0	0	0	0	0	0	0	0	0	0
221	0	0	122	0	0	0	0	0	0	0	0	0	0	1666	79	0	97
0	0	0	0	0	0	0	0	0	0	0	0	0	0	0	0	0	0
0	0	0	0	0	0	0	0	0	0	0	0	0	0	0	0	0	0
0	0	0	0	0	0	0	0	0	0	0	0	0	0	0	0	0	0
0	0	0	0	0	0	0	0	0	0	0	0	0	0	0	0	0	0
425	0	0	914	0	0	0	0	0	0	0	0	0	0	0	0	0	0
0	0	0	0	0	0	0	0	0	0	0	0	0	0	0	0	0	0
0	0	0	0	0	0	0	0	0	0	0	0	0	0	0	0	0	0
0	0	0	0	0	0	0	0	0	0	0	0	0	0	0	0	0	0
0	0	0	0	0	0	0	0	0	0	0	0	0	0	0	0	0	0
0	0	0	0	0	0	0	0	0	0	0	0	0	0	0	0	0	0
0	0	0	0	0	0	0	0	0	0	0	0	0	0	0	0	0	0
0	392	116	409	1241	414	596	303	0	0	0	0	0	240	17509	1039	246	3176
0	0	0	0	0	0	0	0	0	0	0	0	0	0	0	0	0	0
0	591	206	0	717	0	402	2399	342080	55	34272	15542	140	2897	1570	50708	490	58262
0	0	0	0	0	0	0	0	0	0	0	0	0	0	0	0	0	0
0	0	0	0	0	0	0	0	0	0	0	0	0	0	0	0	0	0
0	0	0	0	0	0	0	0	354	493	497	64	914	0	0	454	0	0
0	0	0	0	0	0	0	0	0	0	66	24607	0	56	0	4287	0	7112
0	0	0	0	0	0	0	0	0	0	0	0	0	0	0	0	0	0
0	0	0	0	0	0	0	0	0	0	0	0	0	0	0	0	0	0
0	0	0	0	0	0	0	0	0	0	0	0	0	0	0	0	0	0
0	0	0	0	0	0	0	0	0	0	0	0	0	0	0	0	0	0
0	0	0	0	0	0	0	0	0	0	0	0	0	0	0	0	0	0
0	0	0	0	0	0	0	0	0	0	0	0	0	0	0	0	0	0
0	0	0	0	0	0	0	0	0	0	0	0	0	0	0	0	0	0
0	0	0	0	0	0	0	0	0	0	0	0	0	0	0	0	0	0
3489	77740	89840	632	97282	15562	1159	50	61	53	164	40	70	40813	1615	20276	1280	390
0	0	0	0	0	0	0	0	0	0	0	0	0	25494	0	0	46	75
0	0	0	0	0	0	0	0	0	0	0	0	76	0	0	0	0	0
0	0	0	0	0	0	0	0	0	0	0	0	0	0	0	0	0	0
0	0	0	0	0	0	0	0	0	0	0	0	0	0	0	0	0	0
0	0	0	0	0	0	0	0	0	0	0	0	0	0	0	0	0	0
0	0	0	0	0	0	0	0	0	0	0	0	0	0	0	0	0	0
241	0	0	0	0	0	0	0	0	0	0	0	0	0	0	0	0	35
0	0	0	0	0	0	0	0	0	0	0	0	0	0	0	0	0	0
0	0	0	0	0	0	0	0	0	0	0	0	0	0	0	0	0	0
0	0	0	0	0	0	0	0	0	0	0	0	0	0	0	0	0	0
201	0	53	95	0	0	0	0	0	0	0	0	0	0	0	0	0	0
0	0	0	0	0	0	0	0	0	0	0	0	0	0	0	0	0	0
0	0	0	0	0	0	0	0	0	0	0	0	0	0	0	0	0	0
0	0	0	0	0	0	0	0	0	0	0	0	0	0	0	0	0	0
0	0	0	0	0	6834	0	0	0	0	0	0	0	0	1009	0	0	0
0	0	0	0	0	0	0	0	0	0	0	0	0	0	0	0	0	0
0	0	0	0	210	0	0	0	0	0	0	0	0	28	0	0	0	0
0	0	0	0	0	0	0	0	0	0	0	0	0	0	0	0	0	0
0	0	0	0	0	0	0	0	0	0	0	0	0	0	0	0	0	0
0	0	0	0	0	0	0	0	0	0	0	0	0	109	0	0	0	22
0	0	0	0	0	0	0	0	0	0	0	0	0	0	0	0	0	0
445	254	457	0	205	8809	0	0	4887	0	0	0	0	0	878	0	0	0
0	0	0	0	0	0	0	0	0	0	0	0	0	0	0	0	0	0
0	0	0	0	0	0	0	0	0	0	0	0	68	0	2485	0	0	0
0	0	0	0	0	0	0	0	0	0	0	0	0	0	0	0	0	0
0	0	53	0	0	0	0	0	0	0	0	0	0	0	0	0	0	0
0	0	0	0	0	0	0	0	0	0	0	0	0	0	0	0	0	0
0	0	0	0	0	0	0	0	0	0	0	0	0	0	130	0	0	0
0	0	0	0	0	0	0	0	0	0	0	0	0	0	0	0	0	0
0	0	0	0	0	0	0	0	0	0	0	0	0	0	0	0	0	0
0	0	0	0	0	0	0	0	0	0	0	0	0	0	0	0	0	0
0	0	0	0	0	0	0	0	0	0	0	0	0	31	0	0	0	0
241	0	477	0	98	0	0	0	0	0	0	0	0	25	0	0	0	0
229	0	0	0	0	0	0	0	48	103	0	50	74	0	111	238	0	0
0	0	0	0	0	0	0	0	0	0	101	17123	0	0	0	0	0	0
0	0	0	0	0	0	0	0	0	0	0	0	0	0	0	0	0	0
0	0	0	0	0	14096	0	0	0	0	143	70181	0	0	0	0	72	40
0	0	0	0	0	0	0	0	0	0	0	0	0	0	0	0	0	0
0	0	0	0	0	0	0	0	0	0	0	0	0	0	0	0	0	0
0	0	0	0	0	0	0	0	0	0	0	0	0	0	0	0	0	0
0	0	0	0	0	0	0	0	0	0	0	0	0	8842	0	0	0	36
0	0	0	0	0	0	0	0	0	0	0	0	0	4030	113	0	0	0
0	0	0	0	0	0	0	0	0	0	0	0	0	0	0	0	0	0
0	0	0	0	0	0	93	0	0	0	0	0	0	97174	0	0	162	58
0	0	0	0	0	0	0	0	0	0	0	0	0	0	0	0	0	0
0	0	0	0	0	0	0	0	0	0	0	0	0	0	0	0	0	0
0	0	0	0	0	0	0	0	0	0	0	0	0	0	0	0	0	0
0	0	0	0	0	0	0	0	0	0	0	0	0	0	0	0	0	0
0	1008	1273	109	1167	327	85	0	18	0	0	46	69	795	130	1716	58	106
0	0	0	0	0	0	0	0	0	0	0	0	0	0	0	0	0	0
0	0	0	0	0	0	0	0	0	0	0	0	0	0	0	0	0	0
0	0	0	0	0	0	0	0	0	0	0	0	0	0	0	0	0	0
224	0	0	0	98	0	83	0	0	0	0	0	0	0	0	0	0	0
0	0	0	0	0	0	0	0	0	0	0	0	0	0	0	0	0	0
0	0	0	0	0	0	0	0	0	0	0	0	0	3036	0	0	0	0

209	71	0	0	98	18058	0	0	0	0	21887	0	0	0	1009	1025	3476	0
0	0	0	0	0	0	0	0	0	0	0	0	0	0	0	0	0	0
0	0	0	0	0	0	0	0	0	0	1371	0	0	0	0	0	0	0
11051	0	0	0	0	8916	0	0	0	0	0	0	0	0	0	0	0	0
0	0	0	0	0	0	0	0	0	0	0	0	0	0	0	5273	0	0
0	0	0	0	0	0	0	0	26152	0	52	0	88	0	0	0	0	0
0	0	0	0	0	0	0	0	0	0	0	0	0	0	0	0	0	0
0	0	0	0	0	0	82	0	0	0	0	0	0	59	0	0	0	15733
0	0	0	0	0	0	0	0	0	0	0	0	0	0	0	0	0	0
195	176	54	0	0	0	0	0	0	0	0	0	0	0	0	0	0	0
0	0	157	0	0	0	0	0	0	0	0	0	0	0	0	0	0	0
0	0	0	0	0	0	0	0	0	0	0	0	0	0	0	0	0	0
0	0	0	199	0	0	0	0	0	0	0	0	0	0	0	0	0	0
0	0	0	0	0	0	0	0	0	0	0	0	0	0	0	0	0	0
0	0	0	0	103	0	0	0	0	0	0	0	0	0	0	0	0	0
0	0	0	0	0	0	0	0	0	0	0	0	0	0	0	0	0	0
0	0	0	0	0	0	0	0	0	0	0	0	0	0	0	0	0	0
0	89	51	0	0	0	0	0	0	0	56	0	0	0	0	0	0	0
0	0	0	0	0	0	0	0	9805	0	28089	0	0	0	0	0	0	0
0	0	0	0	0	0	0	0	0	0	0	0	0	0	0	0	0	0
7385	77738	7569	783021	7521	137640	895	806312	31500	691	83878	21986	6377	18847	12899	16306	104608	19092
0	0	0	0	0	0	0	0	0	0	0	0	0	0	0	0	0	0
224	0	0	0	0	0	0	0	0	0	0	0	0	0	0	0	0	0
0	0	0	0	0	0	0	0	0	0	0	0	0	0	0	0	59	0
0	0	0	0	0	0	0	0	0	0	0	0	0	0	0	0	0	0
0	0	0	0	0	0	0	0	0	0	0	0	0	0	0	0	0	0
0	0	0	0	0	0	0	0	0	0	0	0	0	0	0	0	0	0
0	0	0	0	111	0	69748	0	0	0	0	0	0	29	145	0	13618	2973
0	0	0	0	0	0	0	0	0	0	0	0	0	0	0	0	0	0
CC041	CC039	CC038	CC052	CC044	CC053	CC065	CC055	CC071	CC070	CC069	CC068	CC067	CC066	CC037	CC064	CC063	CC062
0	0	0	0	0	0	0	0	0	0	0	0	0	0	0	0	0	0
0	0	0	0	0	0	0	0	0	0	0	0	0	0	0	0	0	0
0	0	0	0	0	0	0	0	0	0	0	0	0	0	0	0	0	0
0	0	0	0	0	0	0	0	0	0	0	0	0	0	0	0	0	0
0	320	314	0	0	0	0	0	0	0	0	0	0	0	0	0	0	0
0	0	0	0	0	0	0	0	0	0	0	0	0	0	0	0	0	0
0	0	0	0	0	0	0	0	0	0	0	0	0	0	0	0	57	0
48432	725993	803919	49770	785211	444774	14167	2094	10573	2176	319571	106370	3256	411820	137272	669355	47019	32317
0	0	0	0	0	0	0	0	0	0	0	0	0	0	0	0	0	0
0	0	0	0	0	0	0	0	0	0	0	0	0	0	0	0	0	0
0	0	0	0	0	0	0	0	0	0	0	589	0	0	0	0	0	0
0	0	0	0	0	0	0	0	0	0	0	0	0	0	0	0	0	0
0	0	0	0	0	0	0	0	0	58	0	0	0	91	0	37	0	35121
0	87	58	0	190	0	0	0	0	0	0	0	0	0	0	0	0	0
0	0	0	0	0	0	0	0	0	0	0	0	0	0	0	0	0	0
0	0	0	0	0	0	0	0	0	0	0	0	0	0	0	0	0	0
0	78	57	215	0	0	0	0	0	0	0	0	0	0	126	0	0	658
0	581	1352	13372	65	0	74	115	0	0	0	0	82	101	3409	45	801	23073
0	0	0	0	125	0	0	0	0	0	0	0	0	0	0	0	0	0
0	0	0	0	0	0	0	0	0	0	0	0	0	0	0	0	0	0
0	0	0	0	0	0	0	0	0	0	0	0	0	0	0	0	0	0
0	0	0	0	0	0	0	0	0	0	0	50	0	0	0	0	0	0
0	0	0	0	0	0	0	0	0	0	0	0	0	0	0	0	0	0
0	0	0	0	0	0	0	0	0	0	0	0	0	0	0	0	0	0
194	14453	15048	399	13683	4071	244	0	18	0	127	0	0	7762	1304	7745	1105	5482
0	0	0	0	0	0	0	0	547	0	0	0	0	0	0	0	0	0
0	0	0	0	0	0	0	0	0	0	0	0	0	0	0	0	0	0
0	0	0	0	0	0	0	0	0	0	0	0	0	0	0	2562	0	0
0	0	0	0	0	0	0	0	0	0	0	0	0	0	0	0	0	0
0	0	0	0	0	0	0	0	0	0	0	0	0	0	0	0	0	0
0	92	0	0	107	0	0	0	0	0	0	0	0	0	0	0	0	0
0	0	0	0	0	0	0	0	0	0	0	0	0	0	0	0	0	0
0	0	0	0	0	0	0	0	0	0	0	0	0	0	0	0	0	0
0	0	0	0	0	0	0	0	0	0	0	0	0	0	0	0	0	0
0	0	0	0	0	0	0	0	0	0	0	0	0	0	0	0	0	0
0	500	51	0	0	0	165	0	0	0	710	102	0	302	0	37	104	68
0	0	0	0	0	0	0	0	0	0	0	0	0	0	0	0	0	0
0	0	0	0	0	0	0	0	0	0	0	0	0	0	108	40	0	509
0	0	0	0	0	0	0	0	0	0	0	0	0	0	0	0	0	0
0	0	0	0	0	0	0	0	0	0	0	0	0	0	0	0	0	0
33163	0	0	0	0	0	0	0	0	0	0	0	0	0	0	0	0	0
0	0	0	0	0	0	187	0	0	0	152	56777	0	12765	4247	0	0	16520
0	0	0	0	0	0	0	0	0	0	0	0	0	0	265	0	0	0
0	0	0	0	0	0	0	0	0	0	0	0	0	0	0	0	0	0
0	0	0	0	0	0	0	0	0	0	0	0	0	0	0	0	0	0
0	0	0	0	0	0	0	0	0	0	0	0	0	0	400	0	0	0
0	0	0	0	0	0	0	0	0	0	0	0	0	0	0	0	0	0
0	0	0	0	0	0	0	0	0	0	0	0	0	0	0	0	0	0
0	0	0	0	0	0	0	0	0	0	0	0	0	0	0	0	0	0
0	0	0	0	0	0	0	0	0	0	0	0	0	0	0	0	0	0
0	0	0	0	0	0	0	0	0	0	0	0	0	0	0	0	0	0
0	0	0	0	0	0	0	0	0	0	0	0	0	0	0	0	0	0
0	0	0	0	0	0	0	0	0	0	0	0	0	0	0	0	0	0
0	0	0	0	0	0	0	0	0	0	0	0	0	0	0	0	0	0
0	0	0	0	0	0	0	0	0	0	0	0	0	0	0	0	0	0

0	0	0	0	0	0	0	0	0	0	0	0	0	0	0	0	0	0
18937	0	0	0	0	0	0	383	7054	64	336	11442	389	0	762	100	2210	4283
0	0	0	0	0	0	0	0	0	0	0	0	0	0	0	0	56	0
0	0	0	0	0	14316	0	0	11919	0	0	0	0	0	0	0	630	0
3498	6824	5144	20260	6233	2807	824622	750	6084	56	18834	13120	850600	2967	107698	20258	443798	240974
0	79	49	0	0	0	0	0	19	0	4647	0	0	0	143	0	0	0
0	0	0	0	0	0	0	0	0	0	0	0	0	0	0	0	0	0
0	0	0	0	0	0	0	0	0	0	0	0	0	0	0	0	0	0
247	0	61	0	0	0	0	0	8273	45	623	0	0	0	125	0	0	0
0	0	0	596	0	0	0	0	0	0	0	0	0	9241	0	0	58	7007
0	0	0	199	0	0	0	0	0	0	0	0	0	0	0	0	0	0
0	0	0	0	0	0	0	0	0	0	0	0	0	0	0	0	0	0
0	0	0	0	0	0	0	0	0	0	0	0	0	0	0	0	0	0
90164	0	0	0	95	0	84	0	0	87614	49	0	68	0	0	0	15225	0
0	0	0	0	0	0	0	0	0	0	0	0	0	0	0	0	0	0
0	0	0	0	0	0	0	0	0	0	53	24090	80	0	0	0	0	0
0	0	0	0	0	0	0	0	0	0	0	0	0	0	0	0	0	0
0	0	0	0	0	0	0	0	0	0	0	0	0	0	0	0	0	0
0	0	0	0	0	0	0	0	0	0	0	0	0	0	0	0	0	0
0	0	0	0	0	0	0	0	0	0	0	0	0	0	0	0	0	0
0	0	0	0	0	0	0	0	0	0	0	0	0	0	0	0	0	0
0	0	0	0	0	0	0	0	0	0	0	0	0	0	0	0	0	0
0	0	0	0	0	0	0	0	0	0	0	0	0	0	0	0	0	0
0	0	0	0	0	0	0	0	0	0	0	0	0	0	133	0	0	0
0	0	0	0	0	0	0	0	0	0	0	0	0	0	0	0	0	0
0	90	0	0	403	0	0	0	0	0	0	0	0	0	0	0	0	0
0	0	0	0	0	113	0	0	22	0	0	0	0	710	0	66	0	65
0	0	0	0	0	0	0	0	0	0	0	0	0	0	0	0	0	0
0	0	0	0	0	0	0	0	0	0	0	0	0	0	0	0	0	0
0	0	0	0	0	0	0	0	0	0	0	0	0	0	0	0	0	0
0	0	0	0	0	0	0	0	0	0	0	0	0	0	0	0	0	0
0	0	0	0	0	0	0	23767	16001	0	0	0	0	0	0	0	0	0
0	0	0	0	0	0	0	0	0	0	0	0	0	0	0	0	0	0
0	0	0	311	0	86539	0	0	0	0	0	0	0	0	0	0	0	0
0	0	0	0	0	0	0	0	0	0	0	0	0	0	0	0	0	0
430	466	365	13062	225	1102	99	1927	139	164	6394	11452	226	132	252816	25581	188	75077
0	0	0	0	0	0	0	0	0	0	0	0	0	0	0	0	0	0
0	0	0	0	0	0	0	0	0	0	0	0	0	0	0	0	0	0
0	0	0	0	0	0	0	0	0	0	0	0	0	0	0	0	0	0
0	0	0	0	0	0	0	0	0	0	0	0	55	0	15741	0	0	0
0	0	0	0	0	0	0	0	0	0	0	0	0	0	0	0	0	0
0	0	0	0	0	0	0	0	0	0	0	0	0	0	0	0	0	0
0	0	0	0	0	0	0	0	0	0	0	0	0	0	0	0	0	0
0	0	0	0	0	0	0	0	0	0	0	0	0	0	0	0	0	0
0	0	0	0	0	0	0	0	0	0	0	0	0	0	0	0	1676	32
448	332	0	0	0	0	0	0	0	0	0	0	0	0	0	0	5753	0
0	0	0	0	0	0	0	0	0	0	0	0	0	0	0	0	0	0
0	0	0	0	0	0	0	0	0	0	0	0	0	0	0	0	0	0
0	0	0	0	0	0	0	0	0	0	0	0	0	0	0	0	0	0
0	0	0	0	0	0	0	0	0	0	0	0	0	0	0	0	0	0
0	1142	1696	0	1057	116	0	0	28	0	0	0	0	0	0	29	0	0
0	0	0	0	0	0	0	0	0	0	0	0	0	0	0	0	0	0
0	0	0	0	0	0	0	0	0	0	0	0	0	0	0	0	0	0
6332	1604	2090	6929	2048	9062	0	4241	532	575	689	449	102358	134	0	662	196	0
0	0	0	0	0	0	0	0	0	0	0	0	0	0	0	0	0	0
0	0	0	0	0	0	0	0	0	0	0	0	0	0	0	0	0	0
0	0	0	0	0	0	0	0	0	0	0	0	0	0	0	0	0	0
0	0	0	0	0	0	0	0	0	0	0	0	0	0	0	0	0	0
0	0	0	0	0	0	0	0	0	0	0	0	0	71248	0	0	59	31
0	0	0	0	0	0	0	0	0	0	0	0	0	0	0	0	0	0
0	0	0	0	0	0	0	0	0	0	0	0	0	0	0	0	0	0
0	99	0	0	0	112	87	0	0	0	18809	0	0	8675	0	0	0	0
0	0	0	0	0	0	0	0	0	0	0	0	0	0	0	0	0	0
0	0	0	0	0	0	0	0	0	0	0	0	0	0	0	0	0	0
0	0	0	0	108	0	0	0	0	0	0	0	0	0	0	0	0	0
0	0	0	0	212	88	0	0	0	0	0	0	79	0	186588	0	0	0
0	0	0	0	0	123	0	0	0	0	0	0	0	0	1472	0	0	0
0	0	0	0	0	0	0	0	0	0	0	0	0	0	22278	0	0	0
0	0	0	0	0	0	0	0	0	0	0	0	0	0	231	0	0	0
0	0	55	0	0	0	0	0	0	0	0	0	0	0	0	0	0	0
0	0	0	0	0	0	0	0	0	0	0	0	0	0	0	0	0	0
0	0	0	0	0	0	0	0	0	0	0	0	0	0	0	0	0	0
0	0	0	0	0	0	0	0	0	0	0	0	0	0	0	0	0	0
0	0	0	0	0	0	0	0	0	0	0	0	0	0	0	0	0	0
0	0	0	0	0	0	0	0	0	0	0	0	0	0	0	0	0	0
0	0	0	0	0	0	0	0	0	0	0	0	0	5003	0	0	0	0
0	0	73	0	0	0	0	0	0	0	0	0	0	0	0	0	0	0
0	0	0	0	0	0	0	0	0	0	0	0	0	0	0	0	0	0
0	0	0	0	0	0	0	0	0	0	0	0	0	0	0	0	0	0
0	0	0	0	0	0	0	0	0	0	0	0	0	0	0	0	0	0
0	0	0	0	0	0	0	0	0	0	0	0	0	0	0	0	0	0
0	0	144	0	0	0	0	0	0	0	0	0	0	0	0	0	0	0
0	0	0	0	0	0	0	0	0	0	0	0	0	0	0	0	0	0
0	0	0	0	0	0	0	0	0	0	0	0	0	0	0	0	0	0
0	0	0	0	0	0	0	0	0	0	0	0	0	0	0	0	0	0
2908	358	385	0	919	0	0	0	0	0	0	36	0	26	7822	0	0	0
0	0	0	0	0	0	0	0	0	0	0	0	0	0	0	0	0	0

0	0	0	512	0	0	0	107	0	0	52	0	28575	0	0	0	0	0
0	0	0	0	0	0	0	0	0	0	0	0	0	0	0	0	0	0
0	0	0	0	0	0	0	0	0	54	0	0	0	0	0	43	0	0
0	0	0	0	0	0	0	0	0	0	0	0	0	0	0	0	0	0
183980	848	764	4823	515	22161	269	0	42	161	453	183267	157	1075	42295	4111	447	54172
0	0	0	0	0	0	0	0	450	162	125	2457	0	0	495	125	41	0
229	240	127	6438	101	28137	78	60	259957	401956	170856	279313	364	0	20070	7826	11276	0
96820	256	269	100	425	2507	432	62	0	54	53	11217	0	17717	3056	16254	3230	2846
26360	0	54	381	0	662	0	0	45	171	49	3687	0	137	1028	340	8312	974
0	0	0	0	0	0	280	0	0	0	57	50	0	0	0	11419	0	0
0	0	0	0	0	0	0	0	0	0	0	0	0	0	0	0	0	0
0	0	0	0	0	0	0	0	0	0	0	0	0	0	0	0	0	0
0	0	0	0	0	0	0	0	0	0	0	0	0	0	0	0	0	0
0	0	0	0	0	0	0	0	0	0	0	0	0	0	0	0	0	0
0	0	0	0	0	0	0	0	0	0	0	0	0	0	0	0	0	0
0	0	0	0	0	0	0	0	0	0	0	0	0	0	0	0	0	0
0	0	0	0	0	0	0	0	0	0	0	0	0	0	0	0	0	0
0	0	0	0	0	0	0	0	0	0	0	0	0	0	0	0	0	0
0	0	0	0	0	0	0	0	0	0	0	0	0	0	0	0	0	0
0	0	0	0	0	0	0	0	0	0	0	0	0	0	0	0	0	0
0	0	0	0	0	0	0	0	0	0	70	0	0	0	0	0	0	0
85799	78	62	0	189	0	0	0	0	0	0	0	0	0	0	0	0	0
0	0	0	0	0	0	0	0	0	0	0	0	0	0	0	0	0	0
0	0	0	0	0	0	0	0	0	0	0	0	0	0	0	0	0	0
0	0	0	0	0	0	0	0	0	0	0	0	0	0	0	0	0	0
0	0	0	0	0	0	0	0	0	0	0	0	0	0	0	0	0	0
0	0	0	0	0	0	0	0	0	0	0	0	0	0	0	0	0	0
0	0	0	0	0	0	0	0	0	0	0	0	0	0	0	0	0	0
0	251	85	0	299	0	0	0	0	0	0	0	0	75	0	138	0	0
0	0	0	0	0	0	0	0	0	0	0	0	0	258	0	0	0	0
0	0	0	0	0	0	0	0	0	0	0	0	0	0	0	0	0	0
0	0	0	0	0	0	0	0	0	0	0	0	0	0	0	0	0	0
0	0	0	0	92	110	0	0	0	0	0	0	0	21	0	0	0	0
0	82	161	0	0	0	0	0	0	0	0	0	0	0	0	0	0	0
0	0	0	0	0	0	0	0	0	52	0	91	0	0	0	73	0	0
0	0	0	0	0	0	0	0	0	0	0	0	0	0	0	0	0	0
0	0	0	0	0	0	0	0	0	0	0	0	0	0	0	0	0	0
0	0	0	0	0	0	0	0	0	0	0	0	0	0	0	0	0	0
0	0	0	0	0	0	0	0	0	0	0	0	0	0	0	0	0	0
0	0	0	0	100	0	0	0	0	0	0	0	0	0	0	0	0	0
0	0	0	0	0	0	0	0	0	0	0	0	0	0	0	0	0	0
0	0	0	0	0	0	0	0	0	0	0	0	0	0	0	0	0	0
0	0	0	0	0	0	0	0	0	0	0	0	0	0	0	0	0	0
0	0	0	0	0	0	0	0	0	0	0	0	0	0	0	0	0	0
0	0	0	95	0	0	0	0	0	0	0	0	0	0	0	0	0	0
0	0	0	1029	0	0	0	0	0	0	0	0	0	3253	0	0	0	0
0	0	0	0	0	0	0	0	0	0	0	0	0	0	0	0	0	0
0	0	0	0	0	0	0	0	0	0	0	0	0	0	0	0	0	0
0	0	0	0	0	0	0	0	0	0	0	0	0	0	0	0	0	0
0	0	0	0	0	0	0	0	0	0	0	0	0	0	0	0	0	0
0	0	0	0	0	0	0	0	0	0	0	0	0	0	0	0	0	0
0	0	0	0	0	0	0	0	0	0	0	0	0	0	0	0	0	0
0	0	0	0	0	0	92	0	0	0	0	0	0	0	680	0	0	6698
0	0	0	0	0	0	0	0	0	0	0	0	0	0	0	0	0	0
0	0	0	0	0	0	0	0	0	0	0	0	0	0	0	0	0	0
0	0	0	0	0	0	0	0	0	0	0	0	0	0	0	0	0	0
0	0	0	0	0	0	0	0	0	0	0	0	0	0	121	0	0	0
0	0	0	0	0	0	0	0	0	0	0	0	0	0	0	0	0	0
0	0	0	0	0	0	0	0	0	0	0	0	0	0	0	0	0	0
0	0	0	0	0	0	0	0	0	0	0	0	0	0	0	0	0	0
0	0	0	0	0	0	0	0	0	0	0	0	0	0	0	0	0	0
0	0	0	0	0	0	0	0	0	0	0	0	0	0	0	0	0	0
0	0	0	0	0	0	0	0	0	0	0	0	0	0	0	0	0	0
0	0	0	0	0	0	0	0	0	0	0	0	0	0	0	0	0	0
0	0	0	0	0	0	0	0	0	0	0	0	0	0	0	0	0	0
0	0	0	0	0	0	0	0	0	0	718	0	0	0	0	0	0	0
0	0	0	0	0	0	0	0	0	0	23085	0	0	0	0	38	0	0
0	0	0	0	0	0	0	0	0	0	0	0	0	0	0	0	0	0
0	0	0	0	0	0	0	0	0	0	0	0	0	0	0	0	0	0
0	0	0	0	0	0	0	0	0	0	0	0	0	0	0	0	0	0
213	0	0	0	0	0	0	0	0	0	0	0	0	0	0	0	0	0
0	0	0	0	0	0	0	0	0	0	0	0	0	0	0	0	0	0
0	0	0	0	0	0	0	0	0	0	50608	0	0	0	0	0	0	0
0	0	0	0	0	0	0	0	0	0	0	0	0	0	0	0	0	0
0	0	0	0	0	0	0	0	0	0	0	0	0	0	0	0	0	0
0	1797	2087	4746	3006	0	98	3913	57	53	0	47	427	162	16544	311	104	266
0	0	0	0	0	0	0	0	0	0	0	0	0	0	0	0	0	0
0	0	0	0	0	0	0	0	0	0	0	0	0	0	0	0	0	0
0	0	0	0	0	0	0	0	0	0	0	0	0	0	0	0	0	0
0	0	0	0	0	0	0	0	0	0	0	0	0	0	0	0	0	0
0	0	0	0	0	0	0	0	0	0	0	0	0	0	0	0	0	0
0	0	0	0	0	0	0	0	0	0	0	0	0	0	0	0	0	0
0	0	0	0	0	0	0	0	0	0	0	0	0	0	0	0	0	0
0	0	0	0	0	0	0	0	0	0	0	0	0	0	0	0	0	0
0	0	0	0	0	0	0	0	0	0	0	0	0	0	0	0	0	0
0	0	0	0	0	0	0	0	0	0	0	0	0	0	0	0	0	0
0	0	0	0	0	6905	0	0	230	0	4278	0	0	0	0	0	0	0

0	0	0	0	0	0	0	0	0	0	0	0	0	0	0	0	0	0
0	159	119	0	0	0	0	0	0	0	0	0	0	17	137	1987	118	151
0	84	50	0	0	0	0	0	0	0	0	0	0	0	0	0	0	0
0	0	0	0	0	0	0	0	0	0	0	0	0	0	0	0	0	0
0	0	0	0	0	0	0	0	0	0	0	0	0	0	0	0	0	0
0	0	0	0	0	0	0	0	0	0	0	0	0	0	0	0	0	0
0	0	0	0	0	0	0	0	0	0	0	0	0	0	0	0	0	0
0	0	0	0	0	0	0	0	0	0	0	0	0	0	0	0	0	0
0	0	0	0	0	0	0	0	0	0	0	0	0	0	0	0	0	0
0	0	0	0	0	0	0	0	0	59	4264	0	0	0	0	0	0	0
0	0	0	0	0	0	0	0	0	0	0	0	0	0	0	0	0	0
0	0	0	0	0	0	0	0	0	0	0	0	0	0	0	0	0	0
0	0	0	0	0	0	0	0	0	0	0	0	0	0	0	0	0	0
0	0	0	0	0	0	0	0	0	0	0	0	0	7907	0	0	575	0
0	0	0	0	0	0	0	0	0	0	0	0	0	0	0	0	0	0
0	0	0	0	0	0	0	0	0	0	0	0	0	0	0	0	0	0
0	0	0	0	0	0	0	0	0	0	0	0	0	0	0	0	0	0
0	0	0	0	0	0	0	0	0	0	0	0	0	0	0	0	0	0
0	0	0	0	0	0	0	0	0	0	0	0	0	0	0	0	0	0
0	0	0	0	0	8143	0	0	8156	48	0	0	0	0	0	0	0	0
0	0	0	0	0	0	0	0	0	0	0	0	0	0	0	0	0	0
0	0	0	0	0	0	0	0	0	0	0	0	0	0	0	0	0	0
0	9843	9873	94	12205	34498	228	0	29	0	51	0	0	3183	248	13979	111	180
0	0	0	0	0	0	0	0	0	0	0	0	0	0	0	0	0	0
0	0	0	0	0	0	0	0	31	0	0	0	0	0	23180	0	14781	0
0	0	46	0	0	0	0	0	0	0	0	0	0	0	0	0	0	0
0	0	0	0	0	0	0	0	0	0	0	0	0	0	0	0	0	0
0	0	0	0	0	0	0	0	0	0	0	0	0	0	0	0	0	0
0	0	0	0	0	0	0	0	0	0	0	0	0	0	0	0	0	0
0	0	0	0	0	0	0	0	0	0	0	0	0	0	0	0	0	0
0	0	0	0	0	0	0	0	0	0	0	0	0	0	0	0	0	0
0	0	0	0	0	0	0	0	0	0	0	0	0	0	0	0	0	0
0	0	0	0	0	0	0	0	0	0	0	0	0	0	0	0	0	0
6770	26415	24967	26095	27073	60067	13161	28278	989	24121	24011	36326	374	53685	6259	17202	12880	16935
0	0	0	0	0	0	0	0	0	0	0	0	0	0	144	0	0	0
0	0	0	0	0	0	0	0	0	0	0	0	0	0	0	0	0	0
0	0	0	0	0	0	0	0	0	0	0	0	0	0	0	0	0	0
0	0	0	0	0	0	0	0	0	0	0	0	0	0	0	0	0	0
0	0	0	0	0	0	0	0	0	0	0	0	0	0	0	0	0	0
0	0	0	0	109	0	0	0	0	0	0	0	0	0	265	0	0	0
0	0	0	0	0	0	0	0	0	0	0	88	0	27	0	0	0	0
0	0	0	0	0	0	0	0	0	0	0	0	0	0	125	0	0	352
0	0	0	0	0	0	0	0	0	0	0	0	0	0	0	0	0	0
0	0	0	0	0	124	0	0	0	0	0	0	0	0	0	0	0	0
0	0	0	0	0	0	152	0	0	0	124	0	0	214	0	0	260137	87
0	0	0	0	0	0	0	0	0	0	0	0	0	0	0	0	0	0
0	0	0	177	0	0	0	62	0	0	0	0	0	0	0	0	0	0
0	0	0	0	0	0	0	0	0	0	0	0	0	0	0	0	0	0
0	0	0	0	0	0	0	0	0	0	0	0	0	0	0	0	0	0
0	0	0	0	0	0	0	0	0	0	0	0	0	0	0	0	51	0
0	0	0	0	0	0	0	0	0	0	0	0	0	0	11280	0	0	0
224	85	107	0	366	112	77	52	0	0	50	69246	0	11088	672	0	0	44
0	89	0	0	0	0	0	0	0	0	0	0	0	287	0	0	0	0
0	0	0	0	0	0	0	0	0	0	0	0	0	0	0	0	0	0
0	0	0	0	0	0	0	0	0	0	0	0	0	0	0	0	0	0
0	0	0	0	0	0	0	0	0	0	0	0	0	0	370	0	0	0
0	0	0	0	0	0	0	0	0	0	0	0	0	0	0	0	0	0
0	0	0	0	0	0	0	0	0	0	0	0	0	0	147	0	0	0
0	0	0	0	0	0	0	0	0	0	0	0	0	0	272	0	0	0
0	0	0	0	0	0	0	0	0	0	0	0	0	0	0	0	0	0
0	0	0	0	0	0	0	0	0	0	0	0	0	0	0	0	0	0
0	0	0	0	0	0	0	0	0	0	0	0	0	0	0	0	0	0
0	0	0	0	0	0	0	0	0	0	0	0	0	0	0	0	0	0
0	0	0	0	0	0	0	0	0	0	0	0	0	0	0	0	0	0
0	0	0	0	0	0	0	0	0	0	0	0	0	0	4506	0	0	0
0	80	0	0	0	0	0	0	0	0	0	0	0	0	0	0	0	0
0	0	0	0	0	0	0	0	0	0	0	0	0	0	0	0	0	0
0	0	0	0	0	0	0	0	0	0	0	0	0	0	140	0	0	0
228	0	52	0	0	0	0	0	0	0	0	0	0	0	0	0	0	1485
0	0	0	0	0	0	0	0	0	0	0	0	0	0	0	0	0	0
0	0	0	0	0	0	0	0	0	0	0	0	0	0	0	0	0	0
229	726	360	0	496	0	0	0	0	0	12309	0	0	33	0	0	0	69
0	0	0	0	0	0	0	0	0	0	0	0	0	0	0	0	0	0
0	0	0	0	0	0	0	0	0	0	0	0	0	0	0	0	0	0
0	0	0	0	0	0	0	0	0	0	0	0	0	0	124	0	0	0
0	0	0	0	0	0	0	0	0	0	0	0	0	0	0	0	0	0
0	0	0	0	0	0	0	0	0	0	0	0	0	0	0	0	0	0
216	89	0	0	0	0	0	0	0	0	0	0	0	0	0	0	0	0
0	0	0	0	0	0	0	0	0	0	0	0	0	0	0	0	0	0
0	0	0	0	0	0	0	0	0	0	0	0	0	0	0	0	0	0
0	0	0	0	0	0	0	0	0	0	0	0	0	0	0	0	63	37645
0	0	0	0	0	0	0	0	0	0	0	0	0	0	0	0	0	0
0	0	0	0	0	0	0	0	0	0	0	0	0	0	0	0	0	0
0	322	589	45523	102	112	0	16200	275	380	374	185	3457	21	238	23	0	0
0	0	0	0	0	0	0	0	0	0	0	0	0	0	0	0	0	0
0	0	0	0	0	0	0	0	0	0	0	0	0	0	0	0	0	0

0	0	0	0	0	0	0	0	0	0	0	0	0	0	0	0	0	0
0	0	0	0	0	0	0	0	0	0	0	0	0	0	0	0	0	0
0	0	0	0	0	0	0	0	0	0	0	0	0	0	0	0	0	0
0	0	0	0	0	0	0	0	0	0	0	0	0	0	0	0	0	0
0	0	0	0	0	0	0	0	0	0	0	0	0	0	0	0	0	0
116687	90	102	0	0	0	0	0	0	0	0	0	0	0	0	772	0	0
0	0	0	100	0	0	0	0	0	0	0	0	0	0	135	0	0	0
0	0	0	0	0	0	0	0	0	0	0	0	0	0	0	0	0	0
0	0	0	0	0	0	0	0	0	0	0	0	0	0	0	0	0	0
0	0	0	0	0	0	0	0	0	0	0	0	0	0	0	0	0	0
0	0	0	392	0	0	0	0	0	0	0	0	0	0	0	0	0	0
0	592	257	0	423	0	232	1074	0	0	0	0	0	35341	0	0	0	37
0	0	0	0	0	0	0	0	0	0	0	0	0	0	136	0	0	0
0	0	0	0	0	0	0	0	0	0	0	0	0	0	0	0	0	0
0	0	0	0	0	0	0	0	0	0	0	0	0	0	0	0	0	0
0	0	0	0	0	2540	0	67	0	0	0	0	0	0	0	0	0	0
0	0	0	0	0	0	0	0	0	0	0	0	0	0	0	0	0	0
0	0	0	0	0	0	0	0	0	0	0	0	0	0	0	0	0	0
0	0	0	0	0	0	0	0	0	0	0	0	0	0	0	0	0	0
0	0	0	0	0	0	0	0	0	0	0	0	0	0	1977	0	0	0
0	0	0	0	0	0	0	0	0	48	0	0	0	0	0	0	0	0
0	0	0	0	0	0	0	0	0	0	0	0	0	0	0	0	0	0
0	0	0	0	0	0	0	0	0	0	0	0	0	0	3264	0	0	0
0	0	0	0	0	0	0	0	0	0	0	0	0	0	0	0	0	0
0	0	0	0	0	0	0	0	0	0	0	0	0	0	0	0	0	0
0	0	0	0	0	0	0	0	0	0	0	0	0	0	0	0	0	0
0	0	0	0	0	0	0	0	0	0	0	0	0	0	0	0	0	0
0	0	0	0	0	0	0	0	0	0	0	0	0	0	0	0	0	0
0	0	0	0	0	0	0	0	0	0	0	0	0	0	0	0	0	0
0	0	0	0	0	0	0	0	0	0	0	0	0	0	0	0	0	0
0	0	0	0	0	0	0	0	0	0	0	0	0	0	0	0	0	0
0	0	0	98	0	0	0	0	0	25575	0	0	0	0	0	0	0	0
0	0	0	0	0	0	0	0	0	0	0	0	0	0	0	0	0	0
0	0	0	0	0	0	0	0	0	0	0	0	0	0	0	0	0	0
0	0	0	0	0	0	0	0	0	0	0	0	0	0	0	0	0	0
0	0	0	0	0	0	0	0	0	0	0	0	0	0	0	0	0	0
0	0	0	0	0	0	0	0	0	0	0	0	0	0	0	0	0	0
0	0	0	0	0	0	0	0	0	0	0	0	0	0	0	0	0	0
653	581	635	0	581	0	0	0	0	0	0	0	0	0	0	0	0	0
0	0	0	0	0	0	0	0	0	0	0	0	0	0	0	0	0	0
0	0	0	0	0	0	0	0	0	0	0	0	0	0	0	0	0	0
0	0	0	0	0	0	0	0	0	0	0	0	0	0	107	0	0	0
0	0	0	0	0	0	0	0	0	0	0	0	0	0	0	0	0	0
0	0	0	0	0	0	0	0	0	0	0	0	0	0	0	0	0	0
0	0	0	0	0	0	0	0	0	0	0	0	0	0	0	0	0	0
23566	196	0	276	0	108	168	46	6836	0	13691	0	0	5636	0	1051	7991	45681
0	0	0	0	0	0	0	0	0	0	1213	0	0	0	0	0	0	0
0	0	0	0	0	0	0	0	0	0	0	0	0	0	0	0	0	0
0	0	0	0	0	0	0	0	0	0	0	0	0	0	0	0	0	0
0	0	0	0	0	427	0	4754	289	28507	5951	0	0	64	115	0	0	0
0	0	0	0	0	0	0	0	0	0	0	0	0	0	0	0	0	0
49714	92	47	3230	101	13538	550	36198	130552	47489	19579	298	387	41165	42646	6868	7546	133324
0	0	0	0	0	0	0	0	2500	0	0	0	0	0	141	0	387	31
0	0	0	0	0	0	0	0	0	0	0	0	0	0	0	0	0	0
0	0	0	0	0	0	0	0	0	0	0	0	0	0	0	0	0	0
0	0	0	0	0	0	0	0	11927	0	0	0	0	0	0	0	0	0
0	0	0	0	0	0	0	0	0	0	0	0	0	0	0	0	0	0
0	0	0	0	0	0	0	0	0	0	0	0	0	0	0	0	0	0
0	0	0	0	0	0	0	0	0	0	0	0	0	0	0	0	0	0
0	751	943	0	679	0	0	0	0	58	0	0	0	93	394	126	67	0
0	0	0	0	0	2165	0	0	0	0	0	0	68	0	0	0	0	0
0	0	0	0	0	101	0	19315	0	0	0	0	0	0	0	0	0	0
0	0	0	0	0	0	0	0	0	0	0	0	0	0	0	0	0	0
0	0	0	0	0	0	0	0	0	0	0	0	0	0	0	0	0	0
0	0	0	0	0	0	0	0	0	0	0	0	0	0	0	0	0	0
0	0	0	0	0	0	0	0	0	0	0	0	0	0	0	0	0	0
0	0	0	0	0	0	0	0	0	0	0	0	0	0	0	0	52	0
0	0	0	0	0	0	0	0	0	0	0	0	0	0	0	0	0	0
0	0	0	0	0	0	0	0	0	0	0	0	0	0	0	0	0	0
219	80	0	695	0	0	0	0	9106	0	0	0	0	0	0	0	0	0
0	0	0	0	0	0	0	0	0	0	0	0	0	0	0	0	0	0
0	0	0	0	0	0	0	0	0	0	0	0	0	0	0	0	0	0
0	0	0	0	0	0	0	0	0	0	0	0	0	0	0	0	0	0
0	0	0	0	0	0	0	0	0	0	0	0	0	0	0	0	0	0
0	0	0	0	0	0	0	0	0	0	0	0	0	0	0	0	0	4175
0	0	0	0	0	0	0	0	0	0	0	0	0	0	0	0	0	0
0	0	0	0	0	0	0	0	0	0	0	0	0	0	0	0	0	0
0	0	0	0	0	0	0	0	0	0	0	0	0	0	0	0	0	0
0	0	0	0	0	0	0	0	0	0	0	0	0	0	0	0	0	0
0	0	0	0	0	0	0	0	0	0	0	0	0	0	0	0	0	0
0	0	0	0	0	0	0	0	0	0	0	0	0	0	124	0	0	0
0	0	0	116	0	101	0	14534	0	0	0	0	0	0	0	0	0	0
0	0	0	0	0	0	0	0	0	0	0	0	0	0	0	0	0	0
0	0	0	0	0	0	0	0	0	0	0	0	0	0	0	0	0	0
0	0	0	0	0	0	0	0	0	0	0	0	0	0	0	0	0	0
0	0	52	0	0	0	0	0	0	0	0	0	0	0	0	0	0	0

0	0	0	0	0	0	0	0	0	0	5977	38	0	0	124	0	0	0
0	0	0	4612	0	0	0	0	57332	0	0	0	0	0	0	0	0	0
0	64	0	0	0	0	0	0	657	0	0	0	0	0	0	0	0	0
0	0	0	0	0	0	0	0	0	0	0	0	0	0	0	0	0	0
0	0	59	105	0	30132	205	0	0	0	0	0	0	139	25248	0	51	31
0	0	0	0	0	0	0	0	0	0	0	0	0	0	0	0	0	0
0	0	52	0	0	0	0	0	0	0	0	0	0	0	0	0	0	0
0	0	0	0	0	0	0	0	0	0	0	0	0	0	0	0	0	0
0	0	0	698	0	0	0	0	0	0	0	0	0	0	0	0	0	0
0	0	0	0	0	0	0	0	0	0	0	0	0	0	0	0	0	0
0	0	0	0	0	0	0	0	0	0	0	0	0	0	0	0	0	0
0	0	0	0	0	0	0	0	0	0	0	0	0	0	0	0	0	0
0	0	0	0	0	0	0	0	0	0	0	0	0	0	0	0	0	0
0	527	565	0	484	0	89	0	0	0	0	0	0	0	0	30	56	0
0	0	0	0	0	0	0	0	0	0	0	0	0	0	0	0	0	0
0	0	0	0	0	0	0	0	0	0	0	0	0	4242	0	0	0	0
0	0	0	0	0	0	0	0	0	0	0	0	0	0	0	0	0	0
0	0	0	0	0	0	0	0	0	0	0	0	0	0	0	0	0	0
0	0	0	0	0	0	0	0	0	0	0	0	0	0	0	0	0	0
0	0	0	0	0	0	0	0	0	0	0	0	0	0	0	0	0	0
0	0	0	0	0	0	0	0	0	0	0	0	0	0	0	0	0	0
851	9854	9515	700	10455	4996	69167	340	210	0	291	91	75	6179	399	7448	10730	9132
0	0	0	0	0	0	0	0	0	0	0	0	0	0	0	0	0	0
0	0	0	0	0	0	0	0	0	0	0	0	0	0	0	0	0	0
0	0	0	0	0	0	0	0	0	0	0	0	0	0	0	0	0	0
0	0	0	0	0	0	0	0	0	0	0	0	0	0	0	0	0	0
0	0	0	0	0	0	0	0	0	0	0	0	0	0	0	0	0	0
0	0	0	0	0	0	0	0	0	0	0	0	0	0	0	0	0	0
0	2166	496	528	1430	220	0	0	0	0	0	0	0	67	878	255	0	278
0	0	0	0	0	0	0	0	0	0	0	0	0	0	0	0	0	0
0	0	0	0	0	0	0	0	0	0	0	0	0	0	0	2818	0	0
0	168	400	0	301	0	0	0	0	0	0	0	0	0	0	0	0	0
0	0	0	0	0	0	0	0	0	0	0	0	0	0	0	0	0	0
0	0	0	0	0	0	0	0	0	653	126	0	0	0	0	0	209	0
0	0	0	0	0	0	0	0	0	0	0	0	0	0	0	0	0	0
0	0	0	0	0	0	0	0	0	0	0	0	0	0	0	0	0	0
0	0	0	0	0	0	0	0	0	0	0	0	0	0	0	0	0	0
0	0	0	0	0	0	0	0	0	0	0	0	0	0	0	0	0	0
0	0	0	0	0	0	0	0	0	0	0	0	0	0	0	0	0	0
0	0	0	0	0	0	0	0	0	0	0	0	0	0	130	0	0	0
0	0	0	0	0	0	0	0	0	0	0	0	0	0	0	0	0	0
0	0	0	0	0	0	0	0	131	123550	32094	13198	84	0	134	100	6029	0
0	0	0	0	0	0	0	0	0	0	0	0	0	0	0	0	0	0
0	0	0	0	0	0	0	0	0	1925	539	115	0	0	0	0	50	0
0	0	0	0	0	0	0	0	0	0	0	0	0	0	0	0	0	0
0	0	0	0	0	0	0	0	0	0	0	0	0	0	0	0	0	0
0	0	0	0	0	0	0	0	0	0	0	0	0	0	0	0	0	0
0	0	0	0	0	0	0	0	0	0	0	0	0	0	0	0	0	0
0	0	0	0	0	0	0	0	0	0	0	0	0	0	0	0	0	0
0	0	0	0	0	0	0	0	0	0	0	0	0	0	0	0	0	0
0	0	0	0	0	0	0	0	0	0	0	0	0	0	0	0	0	0
0	0	0	0	0	0	0	0	0	0	0	0	0	0	0	0	0	0
0	0	0	0	0	0	0	0	0	0	0	0	0	0	0	0	0	0
0	0	0	0	0	0	0	0	0	0	0	0	0	0	0	0	0	0
0	0	0	0	0	0	0	2096	0	0	0	0	0	0	0	0	0	0
88106	0	57	0	0	0	0	0	0	0	0	0	0	0	0	0	0	0
0	0	0	0	0	0	0	0	0	0	0	0	0	0	0	0	0	0
0	0	0	0	0	0	0	0	0	0	0	0	0	0	0	0	0	0
0	0	0	0	0	0	0	0	0	0	0	0	0	0	0	0	0	0
0	82	0	0	0	0	0	0	0	0	0	0	0	0	0	0	0	0
0	0	0	0	0	0	0	0	0	0	0	0	0	0	0	0	0	0
0	352	367	0	962	0	0	0	0	0	0	0	0	0	0	114	0	0
0	0	0	0	0	0	0	0	0	0	0	0	0	0	0	0	0	0
0	0	0	0	0	0	0	0	0	0	0	0	0	0	0	0	0	0
0	0	0	0	0	0	0	0	0	0	0	0	0	0	0	0	0	0
0	0	0	0	0	0	0	0	0	0	0	25640	0	0	0	45	0	0
0	0	0	0	0	0	0	0	0	0	0	0	0	0	0	0	0	0
0	0	0	0	0	0	0	0	0	0	0	0	0	0	0	0	0	0
0	0	0	0	0	0	0	0	0	0	663	0	0	0	0	0	0	0
0	0	0	0	0	0	0	0	0	0	587	0	0	0	0	0	0	0
0	0	0	0	0	0	0	0	0	0	11106	0	84	0	0	0	0	0
0	0	0	0	0	0	0	0	0	0	0	0	0	0	0	0	767	0
0	0	0	0	0	0	0	0	0	0	0	0	0	0	0	0	0	0
0	0	0	0	0	0	0	0	0	0	0	0	0	0	0	0	0	0
0	0	0	0	0	0	0	0	0	0	0	0	0	0	0	0	0	0
0	0	0	0	0	0	0	0	0	0	0	0	0	0	0	0	4852	0
0	0	0	0	0	0	0	0	0	0	0	0	0	0	0	0	0	0
0	0	0	0	0	0	88	0	0	0	0	0	0	13163	0	0	61	0
0	0	0	0	0	0	0	0	0	0	0	0	0	0	0	0	0	0
0	0	0	0	0	0	0	0	0	0	0	0	0	0	0	0	0	0
0	0	0	0	0	0	0	0	0	0	0	0	0	0	0	0	0	0
0	0	0	0	0	0	0	0	0	85220	0	0	0	0	0	0	0	0
0	0	0	0	0	0	0	0	0	0	0	0	0	0	0	0	0	0
0	0	0	0	0	0	0	0	0	0	0	0	0	0	0	0	0	0
0	0	0	0	0	0	0	0	0	0	0	0	0	0	0	0	0	0
0	437	171	0	415	0	0	0	0	0	0	0	0	0	0	0	0	0
0	0	0	0	0	0	0	0	0	0	0	0	0	0	0	0	0	0

0	0	0	0	0	0	0	0	0	0	0	0	0	0	0	0	0	0
0	0	0	0	0	0	0	0	0	0	0	0	0	0	0	0	0	0
0	0	0	0	0	0	0	0	0	0	0	0	0	0	0	0	0	0
221	0	146	0	186	0	0	0	0	0	23953	0	0	0	0	0	0	0
0	0	0	0	0	0	0	0	0	0	0	0	0	0	0	0	0	0
0	0	38	0	0	0	0	0	0	0	0	0	0	0	0	0	0	36
0	0	0	0	0	0	0	0	0	0	0	0	0	0	0	0	0	0
0	0	0	0	0	0	0	0	0	0	0	54	0	0	0	37	0	29
0	0	0	0	0	0	0	0	0	0	0	0	0	0	0	0	0	0
88257	77	0	0	94	108	0	30446	0	0	31554	0	0	0	1256	44	0	0
0	0	0	0	0	0	0	0	0	0	0	0	0	0	0	0	0	0
0	0	0	0	0	0	0	0	0	0	0	0	0	0	0	0	0	0
0	0	0	0	0	0	0	0	0	0	0	0	0	0	0	0	0	0
0	0	0	0	0	0	0	0	0	0	0	0	0	0	0	0	0	0
0	0	0	0	0	0	0	0	0	0	0	0	0	0	0	0	0	0
0	0	0	0	0	0	0	0	0	0	0	0	0	0	0	0	0	0
0	0	0	0	0	0	0	0	30578	0	0	0	0	0	0	0	0	0
0	0	0	0	0	0	0	0	0	0	0	0	0	0	0	0	0	0
0	0	0	0	0	0	0	0	0	0	0	0	0	0	0	0	0	0
0	0	0	0	0	0	0	0	0	0	0	0	0	0	0	0	0	0
0	0	0	0	0	0	0	0	0	0	12666	0	0	0	0	0	0	0
0	0	0	0	0	0	0	0	20	0	0	0	0	0	0	0	0	0
0	0	0	0	0	0	0	0	0	0	0	0	0	0	4279	0	0	0
0	0	0	0	0	0	0	0	0	0	0	0	0	0	0	0	0	0
0	0	0	0	0	0	0	0	0	0	0	0	0	0	0	0	0	0
0	85	0	0	0	0	94	55	0	166993	52	0	0	0	0	34	2435	0
0	0	0	0	0	0	0	0	0	0	0	0	0	0	0	0	0	0
0	0	0	0	0	0	0	0	0	0	0	0	0	0	0	0	0	0
0	0	0	0	0	0	0	0	0	0	0	0	0	0	0	0	0	0
0	0	0	0	0	0	0	0	0	0	0	0	0	0	0	0	0	0
0	0	0	0	0	0	0	0	0	0	0	0	0	0	0	0	0	0
0	0	0	0	0	0	0	0	0	0	0	0	0	0	0	0	0	0
0	0	0	0	0	0	0	0	0	0	0	0	0	0	0	0	0	0
0	0	0	0	0	0	0	0	0	0	0	0	0	0	0	0	0	0
0	0	0	0	0	0	0	0	0	0	0	0	0	0	0	0	0	0
0	0	0	0	0	0	0	0	0	0	0	0	0	0	0	0	0	0
0	0	0	0	0	0	0	0	0	0	0	0	0	0	0	0	0	0
0	0	0	84	0	0	0	0	110	0	0	48	0	0	1529	154	0	64
227	14548	6502	7348	4136	6760	234	0	3328	461	6417	381	731	210	127	11914	862	407
0	0	0	0	0	0	0	0	0	0	0	0	0	0	0	0	0	0
0	0	0	0	0	0	580	0	0	0	0	0	0	273	0	0	125	132699
0	0	0	0	0	0	0	0	0	0	0	0	0	0	0	0	0	0
0	0	0	0	0	0	0	0	0	0	0	0	0	0	0	0	0	0
221	0	0	122	0	0	0	0	0	0	0	0	0	0	1666	79	0	97
0	0	0	0	0	0	0	0	0	0	0	0	0	0	0	0	0	0
0	0	0	0	0	0	0	0	0	0	0	0	0	0	0	0	0	0
0	0	0	0	0	0	0	0	0	0	0	0	0	0	0	0	0	0
0	0	0	0	0	0	0	0	0	0	0	0	0	0	0	0	0	0
0	0	0	0	0	0	0	0	0	0	0	0	0	0	0	0	0	0
0	0	0	0	0	0	0	0	0	0	0	0	0	0	0	0	0	0
0	0	0	0	0	0	0	0	0	0	0	0	0	0	0	0	0	0
0	0	0	0	0	0	0	0	0	0	0	0	0	0	0	0	0	0
425	0	0	914	0	0	0	0	0	0	0	0	0	0	0	0	0	0
0	0	0	0	0	0	0	0	0	0	0	0	0	0	0	0	0	0
0	0	0	0	0	0	0	0	0	0	0	0	0	0	0	0	0	0
0	0	0	0	0	0	0	0	0	0	0	0	0	0	0	0	0	0
0	0	0	0	0	0	0	0	0	0	0	0	0	0	0	0	0	0
0	0	0	0	0	0	0	0	0	0	0	0	0	0	0	0	0	0
0	0	0	0	0	0	0	0	0	0	0	0	0	0	0	0	0	0
0	0	0	0	0	0	0	0	0	0	0	0	0	0	0	0	0	0
0	392	116	409	1241	414	596	303	0	0	0	0	0	240	17509	1039	246	3176
0	0	0	0	0	0	0	0	0	0	0	0	0	0	0	0	0	0
0	591	206	0	717	0	402	2399	342080	55	34272	15542	140	2897	1570	50708	490	58262
0	0	0	0	0	0	0	0	0	0	0	0	0	0	0	0	0	0
0	0	0	0	0	0	0	0	0	0	0	0	0	0	0	0	0	0
0	0	0	0	0	0	0	0	0	0	0	0	0	0	0	0	0	0
0	0	0	0	0	0	0	0	0	0	0	0	0	0	0	0	0	0
0	0	0	0	0	0	0	0	354	493	497	64	914	0	0	454	0	0
0	0	0	0	0	0	0	0	0	0	0	0	0	0	0	0	0	0
0	0	0	0	0	0	0	0	0	0	0	0	0	0	0	0	0	0
0	0	0	0	0	0	0	0	0	0	66	24607	0	56	0	4287	0	7112
0	0	0	0	0	0	0	0	0	0	0	0	0	0	0	0	0	0
0	0	0	0	0	0	0	0	0	0	0	0	0	0	0	0	0	0
0	0	0	0	0	0	0	0	0	0	0	0	0	0	0	0	0	0
0	0	0	0	0	0	0	0	0	0	0	0	0	0	0	0	0	0
0	0	0	0	0	0	0	0	0	0	0	0	0	0	0	0	0	0
0	0	0	0	0	0	0	0	0	0	0	0	0	0	0	0	0	0
0	0	0	0	0	0	0	0	0	0	0	0	0	0	0	0	0	0
0	0	0	0	0	0	0	0	0	0	0	0	0	0	0	0	0	0
0	0	0	0	0	0	0	0	0	0	0	0	0	0	0	0	0	0
0	0	0	0	0	0	0	0	0	0	0	0	0	0	0	0	0	0
0	0	0	0	0	0	0	0	0	0	0	0	0	0	0	0	0	0
3489	77740	89840	632	97282	15562	1159	50	61	53	164	40	70	40813	1615	20276	1280	390
0	0	0	0	0	0	0	0	0	0	0	0	0	25494	0	0	46	75
0	0	0	0	0	0	0	0	0	0	0	0	76	0	0	0	0	0
0	0	0	0	0	0	0	0	0	0	0	0	0	0	0	0	0	0
0	0	0	0	0	0	0	0	0	0	0	0	0	0	0	0	0	0

0	0	0	0	0	0	0	0	0	0	0	0	0	0	0	0	0	0
0	0	0	0	0	0	0	0	0	0	0	0	0	0	0	0	0	0
0	0	0	0	0	0	0	0	0	0	0	0	0	0	0	0	0	0
0	0	0	0	0	0	0	0	0	0	0	0	0	0	0	0	0	0
0	0	0	0	0	0	0	0	0	0	0	0	0	0	0	0	0	0
241	0	0	0	0	0	0	0	0	0	0	0	0	0	0	0	0	0
0	0	0	0	0	0	0	0	0	0	0	0	0	0	0	0	0	35
0	0	0	0	0	0	0	0	0	0	0	0	0	0	0	0	0	0
0	0	0	0	0	0	0	0	0	0	0	0	0	0	0	0	0	0
0	0	0	0	0	0	0	0	0	0	0	0	0	0	0	0	0	0
0	0	0	0	0	0	0	0	0	0	0	0	0	0	0	0	0	0
0	0	0	0	0	0	0	0	0	0	0	0	0	0	0	0	0	0
0	0	0	0	0	0	0	0	0	0	0	0	0	0	0	0	0	0
201	0	53	95	0	0	0	0	0	0	0	0	0	0	0	0	0	0
0	0	0	0	0	0	0	0	0	0	0	0	0	0	0	0	0	0
0	0	0	0	0	0	0	0	0	0	0	0	0	0	0	0	0	0
0	0	0	0	0	0	0	0	0	0	0	0	0	0	0	0	0	0
0	0	0	0	0	0	0	0	0	0	0	0	0	0	0	0	0	0
0	0	0	0	0	6834	0	0	0	0	0	0	0	0	1009	0	0	0
0	0	0	0	0	0	0	0	0	0	0	0	0	0	0	0	0	0
0	0	0	0	210	0	0	0	0	0	0	0	0	28	0	0	0	0
0	0	0	0	0	0	0	0	0	0	0	0	0	0	0	0	0	0
0	0	0	0	0	0	0	0	0	0	0	0	0	0	0	0	0	0
0	0	0	0	0	0	0	0	0	0	0	0	0	0	0	0	0	0
0	0	0	0	0	0	0	0	0	0	0	0	0	0	0	0	0	0
0	0	0	0	0	0	0	0	0	0	0	0	0	109	0	0	0	22
0	0	0	0	0	0	0	0	0	0	0	0	0	0	0	0	0	0
0	0	0	0	0	0	0	0	0	0	0	0	0	0	0	0	0	0
236	0	0	0	100	0	0	0	0	0	0	0	0	0	112	0	0	0
0	0	0	0	0	0	0	0	0	0	0	0	0	0	0	0	0	0
0	0	0	0	0	0	0	0	0	0	0	0	0	0	0	0	0	0
209	254	457	0	105	8809	0	0	0	0	0	0	0	0	766	0	0	0
0	0	0	0	0	0	0	0	0	0	0	0	0	0	0	0	0	0
0	0	0	0	0	0	0	0	4887	0	0	0	0	0	0	0	0	0
0	0	0	0	0	0	0	0	0	0	0	0	0	0	0	0	0	0
0	0	0	0	0	0	0	0	0	0	0	0	0	0	0	0	0	0
0	0	0	0	0	0	0	0	0	0	0	0	68	0	2485	0	0	0
0	0	0	0	0	0	0	0	0	0	0	0	0	0	0	0	0	0
0	0	53	0	0	0	0	0	0	0	0	0	0	0	0	0	0	0
0	0	0	0	0	0	0	0	0	0	0	0	0	0	0	0	0	0
0	0	0	0	0	0	0	0	0	0	0	0	0	0	130	0	0	0
0	0	0	0	0	0	0	0	0	0	0	0	0	0	0	0	0	0
0	0	0	0	0	0	0	0	0	0	0	0	0	0	0	0	0	0
0	0	0	0	0	0	0	0	0	0	0	0	0	0	0	0	0	0
0	0	0	0	0	0	0	0	0	0	0	0	0	31	0	0	0	0
0	0	0	0	0	0	0	0	0	0	0	0	0	0	0	0	0	0
0	0	0	0	0	0	0	0	0	0	0	0	0	0	0	0	0	0
0	0	0	0	0	0	0	0	0	0	0	0	0	0	0	0	0	0
241	0	477	0	98	0	0	0	0	0	0	0	0	25	0	0	0	0
229	0	0	0	0	0	0	0	48	103	0	50	74	0	111	238	0	0
0	0	0	0	0	0	0	0	0	0	101	17123	0	0	0	0	0	0
0	0	0	0	0	0	0	0	0	0	0	0	0	0	0	0	0	0
0	0	0	0	0	0	0	0	0	0	0	0	0	0	0	0	0	0
0	0	0	0	0	0	0	0	0	0	0	0	0	0	0	0	0	0
0	0	0	0	0	0	0	0	0	0	0	0	0	0	0	0	0	0
0	0	0	0	0	14096	0	0	0	0	143	70181	0	0	0	0	72	40
0	0	0	0	0	0	0	0	0	0	0	0	0	0	0	0	0	0
0	0	0	0	0	0	0	0	0	0	0	0	0	0	0	0	0	0
0	0	0	0	0	0	0	0	0	0	0	0	0	0	0	0	0	0
0	0	0	0	0	0	0	0	0	0	0	0	0	0	0	0	0	0
0	0	0	0	0	0	0	0	0	0	0	0	0	0	0	0	0	0
0	0	0	0	0	0	0	0	0	0	0	0	0	0	0	0	0	0
0	0	0	0	0	0	0	0	0	0	0	0	0	0	0	0	0	0
0	0	0	0	0	0	0	0	0	0	0	0	0	0	0	0	0	0
0	0	0	0	0	0	0	0	0	0	0	0	0	0	0	0	0	0
0	0	0	0	0	0	0	0	0	0	0	0	0	8842	0	0	0	36
0	0	0	0	0	0	0	0	0	0	0	0	0	4030	113	0	0	0
0	0	0	0	0	0	0	0	0	0	0	0	0	0	0	0	0	0
0	0	0	0	0	0	0	0	0	0	0	0	0	0	0	0	0	0
0	0	0	0	0	0	93	0	0	0	0	0	0	97174	0	0	162	58
0	0	0	0	0	0	0	0	0	0	0	0	0	0	0	0	0	0
0	0	0	0	0	0	0	0	0	0	0	0	0	0	0	0	0	0
0	0	0	0	0	0	0	0	0	0	0	0	0	0	0	0	0	0
0	0	0	0	0	0	0	0	0	0	0	0	0	0	0	0	0	0
0	0	0	0	0	0	0	0	0	0	0	0	0	0	0	0	0	0
0	0	0	0	0	0	0	0	0	0	0	0	0	0	0	0	0	0
0	1008	1273	109	1167	327	85	0	18	0	0	46	69	795	130	1716	58	106
0	0	0	0	0	0	0	0	0	0	0	0	0	0	0	0	0	0
0	0	0	0	0	0	0	0	0	0	0	0	0	0	0	0	0	0
0	0	0	0	0	0	0	0	0	0	0	0	0	0	0	0	0	0
0	0	0	0	0	0	0	0	0	0	0	0	0	0	0	0	0	0
224	0	0	0	98	0	83	0	0	0	0	0	0	0	0	0	0	0
0	0	0	0	0	0	0	0	0	0	0	0	0	0	0	0	0	0
0	0	0	0	0	0	0	0	0	0	0	0	0	0	0	0	0	0
0	0	0	0	0	0	0	0	0	0	0	0	0	3036	0	0	0	0
209	71	0	0	98	18058	0	0	0	0	21887	0	0	0	1009	1025	3476	0
0	0	0	0	0	0	0	0	0	0	0	0	0	0	0	0	0	0

0	0	0	0	0	0	0	0	0	0	1371	0	0	0	0	0	0	0
11051	0	0	0	0	8916	0	0	0	0	0	0	0	0	0	0	0	0
0	0	0	0	0	0	0	0	0	0	0	0	0	0	0	5273	0	0
0	0	0	0	0	0	0	0	26152	0	52	0	88	0	0	0	0	0
0	0	0	0	0	0	0	0	0	0	0	0	0	0	0	0	0	0
0	0	0	0	0	0	82	0	0	0	0	0	0	59	0	0	0	15733
0	0	0	0	0	0	0	0	0	0	0	0	0	0	0	0	0	0
0	0	0	0	0	0	0	0	0	0	0	0	0	0	0	0	0	0
0	0	0	0	0	0	0	0	0	0	0	0	0	0	0	0	0	0
0	0	0	0	0	0	0	0	0	0	0	0	0	0	0	0	0	0
0	0	0	0	0	0	0	0	0	0	0	0	0	0	0	0	0	0
0	95	54	0	0	0	0	0	0	0	0	0	0	0	0	0	0	0
195	81	0	0	0	0	0	0	0	0	0	0	0	0	0	0	0	0
0	0	157	0	0	0	0	0	0	0	0	0	0	0	0	0	0	0
0	0	0	0	0	0	0	0	0	0	0	0	0	0	0	0	0	0
0	0	0	0	0	0	0	0	0	0	0	0	0	0	0	0	0	0
0	0	0	0	0	0	0	0	0	0	0	0	0	0	0	0	0	0
0	0	0	0	0	0	0	0	0	0	0	0	0	0	0	0	0	0
0	0	0	199	0	0	0	0	0	0	0	0	0	0	0	0	0	0
0	0	0	0	0	0	0	0	0	0	0	0	0	0	0	0	0	0
0	0	0	0	0	0	0	0	0	0	0	0	0	0	0	0	0	0
0	0	0	0	103	0	0	0	0	0	0	0	0	0	0	0	0	0
0	0	0	0	0	0	0	0	0	0	0	0	0	0	0	0	0	0
0	0	0	0	0	0	0	0	0	0	0	0	0	0	0	0	0	0
0	89	51	0	0	0	0	0	0	0	56	0	0	0	0	0	0	0
0	0	0	0	0	0	0	0	0	0	27772	0	0	0	0	0	0	0
0	0	0	0	0	0	0	0	9805	0	317	0	0	0	0	0	0	0
0	0	0	0	0	0	0	0	0	0	0	0	0	0	0	0	0	0
0	0	0	0	0	0	0	0	0	0	0	0	0	0	0	0	0	0
0	0	0	0	0	0	0	0	0	0	0	0	0	0	0	0	0	0
0	0	0	0	0	0	0	0	0	0	0	0	0	0	0	0	0	0
0	0	0	0	0	0	0	0	0	0	0	0	0	0	0	0	0	0
7385	77738	7569	783021	7521	137640	895	806312	31500	691	83878	21986	6377	18847	12899	16306	104608	19092
0	0	0	0	0	0	0	0	0	0	0	0	0	0	0	0	0	0
224	0	0	0	0	0	0	0	0	0	0	0	0	0	0	0	0	0
0	0	0	0	0	0	0	0	0	0	0	0	0	0	0	0	0	0
0	0	0	0	0	0	0	0	0	0	0	0	0	0	0	0	59	0
0	0	0	0	0	0	0	0	0	0	0	0	0	0	0	0	0	0
0	0	0	0	0	0	0	0	0	0	0	0	0	0	0	0	0	0
0	0	0	0	0	0	0	0	0	0	0	0	0	0	0	0	0	0
0	0	0	0	111	0	69748	0	0	0	0	0	0	29	145	0	13618	2973
0	0	0	0	0	0	0	0	0	0	0	0	0	0	0	0	0	0
0	0	0	0	0	0	0	0	0	0	0	0	0	0	0	0	0	0
0	0	0	0	0	0	0	0	0	0	0	0	0	0	0	0	0	0
0	0	0	0	0	0	0	0	28	103	0	0	72	4579	0	75	0	0
0	0	0	0	0	0	0	0	0	0	0	0	0	0	0	0	0	0
0	0	0	0	0	0	0	0	0	0	0	0	0	252	0	0	0	0
0	0	0	0	0	0	0	0	0	0	0	0	0	0	125	0	0	0
898	16657	8657	391	15143	1387	854	0	21	0	384	47	76	55955	127	66328	2554	3748

COVID-19-061	COVID-19-060	COVID-19-059	COVID-19-058	COVID-19-056	COVID-19-054	COVID-19-036	COVID-19-057	COVID-19-034	COVID-19-014	COVID-19-013	COVID-19-012	COVID-19-011	COVID-19-010	COVID-19-009	COVID-19-015	COVID-19-008	COVID-19-006
453495	955986	937940	988971	515776	970454	923846	956152	990488	931066	926824	857312	917267	931086	928328	834393	938611	939894
329802	33894	14316	6679	457438	1530	19876	20917	4378	38907	43055	122086	46279	42222	42241	138367	23986	10505
0	4252	6639	3352	137	35	0	6697	0	0	0	0	0	0	0	63	0	0
214244	5665	1184	159	10256	26836	54216	1875	5134	29119	27113	20602	32067	25885	27289	16359	37043	47791
2459	203	39921	839	1626	1093	2062	14359	0	908	3008	0	4387	807	2142	10818	360	1763
0	0	0	0	14767	52	0	0	0	0	0	0	0	0	0	0	0	0
0	0	0	0	0	0	0	0	0	0	0	0	0	0	0	0	0	47

COVID-19-061	COVID-19-060	COVID-19-059	COVID-19-058	COVID-19-056	COVID-19-054	COVID-19-036	COVID-19-057	COVID-19-034	COVID-19-014	COVID-19-013	COVID-19-012	COVID-19-011	COVID-19-010	COVID-19-009	COVID-19-015	COVID-19-008	COVID-19-006
0	108	0	0	0	51	0	53	0	0	605	0	0	119	184	0	91	111
0	0	0	0	0	0	0	0	0	0	0	0	0	0	0	0	0	0
27663	414174	5180	4616	63030	4619	38880	123508	6139	766102	775125	102305	654317	787942	776874	569574	585595	807465
0	0	0	0	0	0	0	0	0	0	0	0	0	0	0	0	0	0
0	0	0	0	0	0	0	0	0	0	0	0	0	0	0	0	0	0
0	0	0	0	0	0	0	0	0	0	0	0	0	0	0	0	0	0
0	0	0	0	0	0	0	0	0	0	0	0	0	0	0	0	0	0
0	130	121	0	0	0	0	59	0	0	0	0	0	0	202	0	0	0
0	0	0	0	0	0	0	0	0	0	0	0	0	0	92	0	0	0
0	0	0	0	0	0	0	0	0	0	0	0	0	0	0	0	0	0
0	0	0	0	0	0	0	0	0	0	0	0	0	0	0	0	0	0
36294	313	0	162	0	97	9075	136	107	0	82	0	12118	0	0	229	71	263
0	0	0	0	0	0	0	0	0	0	0	0	0	0	0	0	197	0
0	0	0	0	0	0	0	0	0	0	0	0	0	0	0	0	0	0
0	0	0	0	0	0	0	0	0	0	0	0	0	0	0	0	0	0
370	5293	0	0	536	711	722	1354	0	14260	15226	835	13209	14936	15127	3874	6457	9853
0	0	0	0	0	0	0	0	0	0	62	11404	0	0	0	0	0	0
0	0	0	0	0	0	0	0	0	0	0	0	0	0	0	0	0	0
0	0	0	0	0	0	0	0	0	0	0	0	0	0	0	0	0	0
0	0	0	0	0	0	5601	0	0	0	0	0	0	0	0	0	0	0
0	0	0	0	0	0	0	0	0	0	0	0	0	0	0	0	0	0
0	0	0	0	0	0	0	0	0	0	0	0	0	0	0	0	0	0
0	0	0	0	0	0	0	0	0	0	0	0	0	0	0	0	0	0
0	0	0	0	0	0	0	0	0	0	0	0	0	0	0	0	0	0
650	0	0	0	131	156	0	0	108	0	0	0	0	0	474	3647	0	115
0	0	0	0	0	0	0	0	0	0	0	214	0	0	0	0	0	0
0	0	0	0	0	0	1984	0	8698	0	0	0	114	0	0	0	0	0
0	0	0	0	0	0	0	0	0	0	0	0	0	0	0	0	0	0
0	0	0	0	0	0	70	0	0	0	0	0	0	0	0	2464	0	0
35294	2421	721	565	16964	788	52046	608	92290	22722	14109	21560	11250	16188	22686	123193	3498	3845
0	0	0	0	0	0	0	0	0	0	0	0	0	0	0	0	0	0
0	113	0	84	0	49	0	0	0	0	0	0	0	0	267	53	0	480
0	0	0	0	0	0	0	0	0	0	0	0	0	0	0	0	0	0
0	0	0	0	0	0	0	0	0	0	0	0	0	0	0	0	0	0
0	0	0	0	0	0	0	0	0	0	0	0	0	0	0	0	0	0
0	0	0	0	0	0	0	0	0	0	73	0	211	0	0	73	617	152
0	0	0	0	0	0	0	0	0	0	0	0	0	0	0	0	0	0
0	0	0	0	0	0	0	0	0	0	0	0	0	0	0	0	0	0
0	0	0	0	0	0	0	0	0	0	0	0	0	0	0	0	0	0
0	0	0	0	0	0	0	154	0	0	0	0	0	0	0	0	0	0
0	0	0	0	716	0	0	0	0	0	0	72	0	0	0	0	0	0
0	0	0	0	0	0	0	0	0	0	0	0	0	0	0	0	0	0
0	0	0	0	0	0	0	0	0	0	0	0	0	0	0	0	0	0
0	0	0	0	0	0	0	0	0	0	0	0	0	0	0	0	0	0
0	0	0	0	0	0	0	0	0	0	0	0	0	0	0	0	0	0
0	0	0	0	0	64	0	0	0	0	212	0	0	0	0	0	0	0
0	0	0	0	0	0	0	0	0	0	0	0	0	0	0	0	0	0
359	208	112	186	2549	781	18338	156	90838	2798	485	1564	4244	575	473	0	787	951
0	0	0	0	0	0	0	0	0	0	0	0	0	0	0	62	0	0
0	0	0	0	0	0	63	0	3945	0	0	0	0	0	0	0	0	41
0	0	0	0	0	0	0	0	0	0	0	0	0	0	0	0	0	0
0	0	0	0	0	0	0	0	0	0	0	0	0	0	0	53	0	0
0	0	0	0	0	0	0	0	0	0	0	0	0	0	0	0	0	0
0	0	0	0	0	0	63	0	0	0	0	0	0	0	0	56	0	0
0	0	0	0	0	0	0	0	0	0	0	0	0	0	0	0	0	0
0	0	0	0	0	0	0	0	0	0	0	0	0	0	0	0	0	0
0	0	0	0	0	0	0	0	0	0	0	0	0	0	0	0	0	0
0	352	0	0	0	0	0	64	0	1106	678	64	105	1043	1339	3485	447	1058
0	0	0	0	0	0	0	0	0	0	0	0	0	0	0	0	0	0
0	0	0	0	0	0	0	0	0	0	0	0	0	0	0	0	0	0
930	29139	5426	487095	4090	7906	1090	3803	9041	6229	1593	2817	7310	3149	777	1809	1769	2850
0	0	0	0	0	0	3260	0	0	0	0	0	0	0	0	0	0	0
0	0	0	0	0	0	0	0	0	0	0	0	0	0	0	0	0	0
0	0	0	0	0	0	0	0	0	0	0	0	0	0	0	0	0	0
0	0	0	0	0	0	0	0	0	0	0	0	0	0	0	0	0	59
0	0	0	0	0	0	0	0	0	0	0	0	0	0	0	0	0	0
0	0	0	0	2238	0	0	0	0	0	0	0	0	0	0	0	0	0
0	0	0	0	0	0	0	0	0	0	0	0	0	0	0	0	0	0
0	0	0	0	0	0	0	0	0	0	0	0	0	0	0	0	0	0

0	0	0	0	0	0	0	0	0	0	0	0	0	0	0	0	0	0
0	0	0	0	0	0	0	0	0	0	0	0	0	0	0	0	0	0
0	0	0	0	0	0	0	0	0	0	0	0	0	0	0	0	0	0
0	0	0	0	0	0	0	0	0	0	0	0	0	0	0	0	0	0
0	0	0	0	0	0	0	0	0	0	0	0	0	0	0	61	0	0
0	0	0	0	0	0	0	0	0	0	0	0	0	0	0	0	0	0
0	0	0	0	60	0	222836	0	723276	0	64	77	213	0	0	0	83	0
0	0	0	0	0	0	0	0	0	0	0	0	0	0	0	0	0	0
0	4252	6639	3352	0	0	0	6697	0	0	0	0	0	0	0	0	0	0
0	0	0	0	0	0	0	0	0	0	0	0	0	0	0	0	0	0
0	0	105	0	0	0	0	0	0	0	0	0	0	0	0	0	0	0
0	0	0	0	0	0	0	0	0	0	0	0	0	0	0	0	0	0
252	1942	411	567	31071	110	162323	531	631	1119	1472	459	3962	102	195	10476	827	694
0	0	0	0	0	0	0	0	0	0	0	0	0	0	0	0	0	0
0	0	0	0	0	0	0	0	0	0	0	0	0	0	0	0	0	0
0	0	0	0	0	0	0	0	0	0	0	0	0	0	0	0	0	0
0	0	0	0	0	0	0	0	0	0	0	0	0	0	0	0	0	0
0	0	0	0	0	0	0	0	0	0	0	0	0	0	0	0	0	0
0	0	0	0	0	0	0	0	0	0	0	0	1386	0	0	0	0	0
0	0	0	0	0	0	0	0	0	0	72	0	0	0	0	0	0	0
0	0	0	0	0	0	0	0	0	0	0	0	0	0	0	0	0	0
0	0	0	0	0	0	0	0	0	0	0	0	0	0	0	205	0	0
0	0	0	0	0	0	0	0	0	0	0	0	0	0	0	0	0	0
0	0	0	0	0	0	0	0	0	0	0	0	0	0	0	56	0	59
0	116	0	0	0	0	0	0	0	0	0	0	0	210	177	66	106	94
0	0	0	0	0	0	0	0	0	0	0	0	0	0	0	0	0	0
0	0	0	0	0	0	0	0	0	0	0	0	0	0	0	0	0	0
0	0	0	0	0	0	0	0	0	0	0	0	0	0	0	0	0	0
0	0	0	0	0	0	0	0	0	0	0	0	0	0	0	0	0	0
0	0	0	0	0	0	0	0	0	0	0	0	0	0	0	0	0	0
0	0	0	0	0	0	0	0	89	290	0	60	0	0	0	0	188	68
0	0	0	0	0	0	0	0	0	0	0	0	0	0	0	0	0	0
0	0	0	0	0	0	0	0	0	0	0	0	0	0	0	0	0	0
0	0	0	0	0	0	0	0	0	0	0	0	0	0	0	0	0	0
0	0	0	0	0	47	0	0	0	0	0	0	0	0	0	0	0	0
0	0	0	0	0	0	0	0	0	0	0	0	0	0	0	0	0	0
0	0	0	0	0	0	0	0	0	0	0	0	0	0	0	0	0	0
0	0	0	0	0	0	0	0	0	0	0	0	0	0	0	0	0	0
0	0	0	0	0	0	0	0	0	274	0	0	18616	0	0	0	0	0
0	0	0	0	0	0	0	0	0	0	0	0	0	0	0	0	0	0
0	0	0	0	0	0	0	0	0	0	0	0	0	0	0	0	0	0
0	0	0	0	0	0	0	0	0	0	0	0	0	0	0	0	0	0
0	0	0	0	0	0	0	0	0	0	0	0	0	0	0	0	0	0
0	0	0	0	0	0	0	0	0	0	0	0	0	0	0	0	0	0
0	0	0	0	0	0	0	0	0	0	0	347	0	0	0	0	0	0
0	0	0	0	0	0	0	0	101	0	0	0	0	0	0	0	0	0
0	0	0	0	0	0	0	0	0	0	0	0	1979	0	0	0	0	0
0	0	0	0	0	0	0	0	0	0	0	0	0	125	0	0	0	0
0	123	0	0	0	0	0	0	0	0	0	0	0	0	0	0	0	0
0	0	0	0	0	105	0	0	0	0	0	0	0	0	0	0	0	54
2459	203	39921	839	1626	1093	2062	14359	0	908	3008	0	4387	807	2142	10818	360	1763
0	0	0	0	0	0	0	0	0	0	0	0	0	0	0	0	0	0
0	0	0	0	0	0	0	0	0	0	0	0	0	0	0	0	0	60
0	0	0	0	0	0	0	0	0	0	0	0	0	0	0	0	0	0
0	0	0	0	0	0	0	0	0	0	0	0	0	0	0	0	0	0
0	0	0	0	0	0	0	0	0	0	0	0	0	0	0	0	0	0
0	0	0	0	0	0	0	0	0	0	0	0	412	0	0	0	0	57
0	0	0	0	0	0	0	0	0	0	0	0	0	0	0	0	0	0
0	0	0	0	0	0	0	0	0	0	0	0	0	0	0	0	0	0
0	0	0	0	0	0	0	81	0	0	0	0	0	0	0	0	0	0
3042	0	0	90	0	0	235	0	335	0	66	0	0	0	0	0	0	0
0	0	0	0	0	0	0	0	0	0	0	0	0	0	0	260	0	0
66	0	0	0	0	0	77	124	90	8164	7021	153	926	5233	7421	5799	713	476
0	0	0	0	0	0	0	0	0	0	0	0	0	0	0	0	0	49
0	0	0	0	0	0	4232	0	0	283	0	22122	0	0	0	0	0	0
0	0	0	0	0	0	0	0	0	0	0	0	0	0	0	0	0	0
0	0	0	0	0	0	0	0	0	0	0	0	0	0	0	0	0	0
71	0	0	0	0	0	2428	0	0	0	0	6776	0	0	0	0	0	0
0	0	0	0	0	0	0	0	0	0	0	0	0	0	0	0	0	0
0	0	0	0	0	0	0	0	0	0	0	0	0	0	0	0	0	0
0	0	0	0	0	0	0	0	0	0	0	0	0	0	0	0	0	0
0	0	0	0	0	52	0	0	0	0	0	0	0	0	0	0	0	0
0	0	0	0	0	0	0	0	0	0	0	0	0	0	0	0	0	0
0	0	0	0	0	0	0	0	0	0	0	0	0	0	0	0	0	0
68	2931	0	0	3802	0	529	618	800	5892	8765	25928	9049	9107	8950	2542	5162	9648
782	27676	126	0	206	152	1065	6895	1370	32647	26932	352	27678	37284	31200	15963	19369	7168
0	0	0	0	0	0	151	0	516	0	0	24474	0	0	0	0	81	143
0	0	0	0	0	0	0	0	0	0	0	0	0	0	0	0	0	0
0	0	0	0	0	0	0	0	0	0	0	0	0	0	0	0	0	0
0	0	0	0	0	0	0	0	0	0	0	0	0	0	0	0	0	0
0	0	0	0	0	0	0	0	0	0	0	0	0	0	0	0	0	0
0	0	0	0	0	0	0	0	0	0	0	0	0	0	0	0	0	0
0	0	0	0	0	0	0	0	0	0	0	0	0	0	0	0	0	0
214178	5665	1184	159	10256	26836	54139	1751	5044	20955	20092	20449	31141	20652	19868	10560	36330	47315
0	439	0	65	0	56	9941	0	283	0	228	0	1504	0	0	0	0	0
0	0	0	0	0	0	70	0	357	0	0	0	0	0	0	0	0	0
0	0	0	0	0	0	0	0	0	0	0	0	0	0	0	0	0	0

0	0	0	0	14767	52	0	0	0	0	0	0	0	0	0	0	0	0
242	0	0	182	0	54	0	0	0	0	0	0	0	245	186	0	0	0
0	0	0	0	0	0	0	0	0	0	0	0	0	0	0	0	0	0
83	0	0	0	0	0	0	0	0	0	0	0	0	0	0	0	0	138
0	0	0	0	0	0	0	0	0	0	0	0	0	0	0	0	0	0
0	0	0	0	0	0	0	0	0	0	0	0	0	0	0	0	0	0
0	0	0	0	0	0	0	0	0	0	0	0	0	0	0	0	0	0
0	0	0	0	8371	0	59279	0	4224	0	69	0	320	0	0	54	0	0
0	0	0	0	0	0	0	0	0	0	0	0	0	0	107	0	0	176
0	0	0	0	0	0	0	0	0	0	0	0	331	0	0	0	0	0
0	0	0	0	0	0	0	0	0	0	0	0	0	0	0	0	0	0
0	0	0	0	0	0	0	0	0	0	0	0	0	0	0	0	0	0
0	0	0	0	0	0	0	0	0	0	0	0	0	0	0	0	0	0
0	0	0	0	0	0	0	0	0	0	0	0	0	0	0	0	0	0
0	0	0	0	0	0	0	0	0	0	0	0	0	0	0	0	0	0
0	0	0	0	0	0	0	0	0	0	0	0	0	0	0	0	0	0
0	0	0	0	0	0	0	0	0	0	0	0	0	0	0	0	0	0
0	0	0	0	0	0	0	0	0	0	0	0	0	0	0	0	0	0
0	0	0	0	0	0	0	0	0	0	0	0	0	0	0	0	0	0
0	0	0	0	0	0	0	0	0	0	0	0	0	0	0	0	0	0
0	0	0	0	0	0	0	0	444	0	84	0	0	0	0	0	0	0
0	0	0	0	0	0	0	0	0	0	0	0	0	0	0	0	0	0
0	0	0	0	0	0	0	0	0	0	0	0	0	0	0	0	0	0
0	112	0	0	0	46	0	184	0	0	620	0	420	118	385	109	653	195
0	0	0	0	0	0	0	0	0	0	0	0	0	0	0	0	0	0
0	0	0	0	0	0	0	0	0	0	0	0	0	0	0	0	0	0
0	0	0	0	0	0	0	0	216	0	0	0	0	0	0	0	0	0
0	0	0	0	0	0	0	0	0	0	0	0	0	0	0	0	0	0
0	0	0	0	0	0	0	0	0	0	0	0	0	0	0	0	0	0
0	0	0	0	0	0	0	0	0	0	0	0	0	0	0	0	0	49
0	0	0	0	0	0	0	0	0	0	0	0	0	0	0	0	0	0
0	0	0	0	0	0	0	0	0	0	0	0	0	0	0	0	0	0
195	0	0	0	0	0	0	0	0	0	0	0	0	0	0	0	0	0
0	0	0	0	0	0	0	0	0	0	0	0	0	0	0	0	0	0
0	0	0	0	0	0	0	0	0	0	0	0	0	0	0	0	0	0
0	461882	916892	471317	5313	379	8673	812460	1438	289	129	0	0	123	387	0	98	112
0	0	0	0	0	0	0	0	0	0	0	0	0	0	0	0	0	0
0	0	0	0	0	0	0	0	0	0	0	0	0	0	0	0	0	0
0	0	0	0	0	0	0	0	0	0	0	0	0	0	0	0	0	0
0	0	0	0	0	0	0	0	0	0	0	0	0	0	0	0	0	0
0	0	0	0	0	0	0	0	0	0	0	0	0	0	0	49	0	0
0	0	0	0	0	0	0	0	0	0	0	0	0	0	0	0	0	0
0	0	0	0	0	0	0	0	0	0	0	0	0	0	0	0	0	0
0	0	0	0	0	0	0	0	0	268	0	0	0	0	0	0	0	0
0	0	0	0	0	0	0	0	0	0	0	0	0	0	0	0	0	0
0	0	0	0	0	0	0	0	0	0	0	81	0	0	0	0	79	102
0	0	0	0	0	0	0	0	0	0	0	0	0	0	0	0	0	0
0	0	0	0	0	0	0	0	0	0	0	0	0	0	0	0	0	0
140	540	0	0	142	0	0	0	95	552	535	0	0	362	689	65	398	303
0	0	0	0	0	0	0	0	0	0	0	0	0	0	0	0	0	0
0	0	0	0	0	0	0	0	0	0	0	0	0	0	0	0	0	0
0	0	0	0	0	0	0	0	0	0	0	0	0	0	0	0	0	0
0	0	0	0	0	0	0	0	0	0	0	0	0	0	0	0	0	0
0	0	0	0	0	0	0	0	0	0	0	0	0	0	0	0	0	0
0	0	0	0	0	205	0	0	0	0	0	0	0	0	0	0	0	0
0	0	0	0	0	0	0	0	0	0	0	0	0	0	0	0	0	0
0	0	0	0	0	0	0	0	0	0	0	0	0	0	0	0	0	0
0	0	0	0	0	0	0	0	0	0	0	0	0	0	0	0	0	0
0	0	0	0	0	0	0	0	0	0	0	0	0	0	0	0	0	0
0	0	0	0	0	0	0	0	0	0	0	0	0	0	0	0	0	0
0	0	0	0	0	0	0	0	0	0	0	0	0	0	0	0	0	0
0	0	0	0	0	0	3547	0	0	0	0	0	0	0	0	0	0	0
0	121	0	0	0	0	0	71	0	0	0	0	0	0	0	59	0	0
0	0	0	0	0	62	0	0	0	0	0	0	0	0	0	0	0	0
0	0	0	0	0	0	0	0	0	0	0	0	0	0	0	0	0	0
0	0	0	0	0	57	17354	0	0	0	0	0	0	0	0	0	0	0
0	0	0	0	0	0	0	0	0	0	0	0	0	0	0	0	0	0
0	0	0	0	0	68	0	0	0	0	0	0	0	0	0	0	0	0
0	0	0	0	0	0	0	0	0	0	0	0	0	0	0	0	0	0
0	0	0	0	0	0	0	0	0	0	0	0	0	0	0	0	0	0
0	0	0	0	0	0	0	0	0	0	0	0	0	0	0	0	0	0
0	0	0	0	0	0	0	0	0	0	0	0	0	0	0	0	0	0
0	0	0	0	0	0	0	0	0	0	0	0	0	0	0	0	0	0
216675	695	428	171	451899	614	2782	231	1024	817	75	69937	5800	0	1162	357	637	454
0	0	0	0	0	0	0	0	0	0	0	0	0	0	0	0	0	0
0	122	0	0	0	0	0	0	0	0	0	0	0	0	0	0	0	0
0	0	0	0	0	0	0	0	0	0	0	0	0	0	0	0	80	0
0	0	0	0	0	0	0	0	0	0	0	0	0	0	0	0	0	0
0	962	107	0	0	62	79	126	0	1383	1630	13876	322	811	1658	0	282	797
0	0	0	0	0	0	0	0	0	0	0	0	0	0	0	0	0	0
0	0	0	0	137	35	0	0	0	0	0	0	0	0	0	0	0	0
0	0	0	0	0	0	0	0	0	0	0	0	0	0	0	0	0	0
0	0	0	0	0	0	0	0	0	0	0	0	0	0	0	0	0	0
0	0	0	0	0	0	0	0	0	0	0	0	0	0	0	0	0	0
0	0	0	0	0	0	0	0	0	0	0	0	0	0	0	0	0	0
0	0	0	0	5505	60	10738	0	2483	0	0	0	0	0	0	0	0	0
0	0	0	0	0	0	0	0	0	0	0	0	651	0	0	0	0	52

43822	124	0	0	0	0	0	62	0	0	0	0	0	0	0	0	0	0
0	0	0	0	0	0	0	0	0	0	0	0	0	0	0	0	0	0
0	0	0	0	0	0	0	0	0	0	0	0	0	0	0	0	0	0
0	0	0	0	0	0	0	0	0	0	0	0	0	0	0	0	0	0
0	0	0	0	0	0	0	0	0	0	0	0	0	0	0	0	0	0
0	0	0	0	0	0	0	0	0	0	0	0	0	0	0	0	0	0
0	0	0	0	0	0	0	0	0	0	0	0	0	0	0	0	0	0
0	0	0	0	0	0	0	0	0	0	0	0	0	0	0	0	0	0
0	0	0	0	0	0	0	0	88	0	0	0	0	0	0	0	0	0
0	0	0	0	0	0	82	0	0	0	0	0	0	0	0	0	0	0
0	0	0	0	0	0	0	0	0	0	0	0	0	0	0	0	0	0
0	0	0	0	0	0	0	0	0	0	0	0	0	0	0	0	0	0
0	114	105	0	0	0	0	75	91	2595	792	609723	0	0	0	56	812	1683
0	116	0	0	0	0	0	0	0	0	0	0	0	0	0	0	0	57
0	0	0	0	0	0	0	0	0	0	0	0	0	0	0	0	0	0
179027	0	0	0	0	53	0	0	0	576	433	263	0	0	197	55	91	0
0	0	223	0	0	0	0	555	0	0	0	0	0	0	0	0	0	0
0	0	0	0	0	0	0	0	0	0	0	0	0	0	0	0	0	0
0	0	0	0	0	0	0	0	0	0	0	0	0	0	0	0	0	0
0	0	0	0	0	0	0	0	81	0	0	0	1978	0	0	0	0	0
0	0	0	0	0	0	0	0	0	0	0	0	0	0	0	0	0	0
0	0	0	0	0	0	0	0	0	0	0	0	0	0	0	0	0	0
0	0	0	0	67	0	0	65	0	296	527	0	0	373	705	0	106	47
0	0	0	0	0	0	0	0	0	0	0	0	0	0	0	0	0	0
0	0	0	0	0	0	0	0	0	0	0	0	0	0	0	63	0	0
0	0	0	0	0	0	0	83	0	307	582	0	0	0	0	0	86	207
0	0	0	0	0	0	0	0	0	0	0	0	0	0	0	0	0	0
343	4962	0	0	228	938	400	1732	95	10200	11236	620	10124	11190	11902	3858	7786	7269
0	0	0	0	0	0	0	0	0	0	0	0	0	0	0	0	0	0
0	0	0	0	0	0	0	0	0	0	0	0	0	0	0	0	0	0
0	0	0	0	0	0	0	0	0	0	0	0	0	0	0	0	0	0
0	0	0	0	0	0	0	0	0	0	0	0	0	0	0	0	0	0
0	0	0	0	0	0	0	0	0	0	0	0	0	0	0	0	0	0
0	0	0	0	0	0	0	0	0	0	0	0	0	0	0	0	0	0
0	0	0	0	0	50	290	136	0	1137	503	0	729	583	180	60	5299	489
0	0	0	0	0	0	0	0	0	0	0	0	0	0	0	0	0	0
0	0	0	0	0	0	4872	0	0	0	0	0	0	0	0	0	0	0
0	0	0	0	0	0	0	0	0	0	213	0	0	218	215	0	95	253
4939	544	341	150	0	0	4532	62	182	270	0	78	634	0	0	161	0	0
0	0	0	0	0	0	0	0	0	0	0	0	0	0	0	0	0	0
0	0	0	84	0	0	76	0	0	0	0	0	0	0	0	0	0	0
0	0	0	0	0	0	0	0	0	0	0	0	0	0	0	0	0	0
0	112	0	0	0	0	0	66	0	0	86	0	106	428	279	0	3102	297
0	0	0	0	0	0	0	0	0	0	0	0	0	0	0	0	0	0
0	0	0	0	0	0	0	0	0	0	0	0	0	0	0	0	0	0
0	0	0	0	0	0	0	0	0	0	0	0	330	0	0	0	0	0
0	0	0	0	0	0	0	0	0	0	0	0	0	0	0	0	0	0
0	0	0	77	0	0	0	0	0	0	0	0	0	0	0	0	0	0
0	0	0	0	0	0	0	0	0	0	0	0	0	0	0	0	0	0
0	0	0	0	0	0	0	0	0	0	0	0	0	0	0	0	0	0
0	221	0	0	0	51	0	0	0	0	0	0	0	0	0	0	0	0
0	0	0	0	0	0	0	0	0	0	114	0	0	0	0	0	0	0
0	0	0	0	0	0	0	0	0	0	0	0	0	0	0	0	0	0
0	0	0	0	0	0	0	0	0	0	0	0	0	0	293	0	0	0
0	0	96	86	0	0	0	0	0	0	0	0	0	0	0	0	0	0
0	0	0	0	0	0	0	0	0	0	0	0	0	0	0	0	0	0
0	0	0	0	0	0	0	0	0	0	0	0	0	0	0	0	0	0
74	579	0	88	0	0	0	120	0	0	635	0	0	0	99	0	94	487
0	0	0	0	0	0	0	0	0	0	0	0	0	0	0	0	0	0
0	0	0	0	0	0	0	0	0	0	0	0	0	0	0	0	0	0
0	0	0	0	0	0	22659	0	76	0	0	70	329	0	0	0	98	99
0	0	0	0	0	0	0	0	0	0	0	0	0	0	0	0	0	0
0	0	0	0	0	0	0	0	0	0	0	0	0	0	0	0	0	0
0	110	0	0	67	0	0	0	0	860	211	19999	0	0	81	0	633	634
0	0	0	0	0	0	0	0	0	0	0	0	0	0	0	0	0	0
0	0	0	0	0	0	0	0	0	0	0	0	0	0	0	0	0	0
0	0	0	0	0	0	0	0	0	0	0	0	0	0	0	0	0	0
0	0	0	0	0	0	0	0	0	0	0	0	0	0	0	0	0	0
0	0	0	0	0	0	0	0	0	0	0	0	0	0	0	0	0	0
0	0	0	0	0	0	0	0	0	0	0	0	0	0	0	0	0	0
0	0	0	0	0	0	0	0	0	0	0	0	0	0	0	0	0	0
0	0	0	0	0	0	0	0	0	0	0	0	0	0	0	0	0	0
0	0	0	0	0	0	0	0	0	0	0	0	0	0	0	0	0	0
0	0	0	98	0	0	0	0	0	0	0	0	0	0	0	0	0	0
0	0	0	0	0	0	0	0	0	0	0	0	0	0	94	0	0	0
0	0	0	0	0	0	0	0	0	0	0	0	0	0	0	0	0	0
0	104	0	0	6646	0	6088	0	90	0	0	0	0	0	0	0	0	0
0	0	0	0	0	0	0	0	452	0	0	0	0	0	0	0	0	0
0	0	0	0	0	0	0	0	0	0	0	0	0	0	0	0	0	0
0	0	0	0	0	0	0	0	0	0	0	0	0	0	0	0	0	0
0	0	0	0	0	0	0	0	0	0	0	0	0	0	0	0	0	0
0	0	0	0	0	0	0	0	0	0	0	0	0	0	0	0	0	52
0	0	0	0	0	0	0	0	0	0	0	0	0	0	0	0	0	0
0	0	0	0	0	0	0	0	0	0	0	0	0	0	0	0	0	0
0	0	0	0	0	0	0	0	0	0	0	0	0	0	0	0	0	0
0	0	0	0	0	0	0	0	0	0	0	0	0	0	0	0	0	0
6105	9407	718	671	377	0	10993	1026	581	2272	8729	984	23144	2265	532	4667	1556	4934

0	0	0	0	0	0	0	0	0	0	0	0	0	0	0	0	0	0
277	0	0	0	0	0	0	0	0	0	0	0	0	0	711	0	0	0
0	0	0	0	0	0	0	0	0	0	0	0	0	0	0	0	0	0
0	0	0	0	0	0	0	0	0	0	0	0	0	0	0	0	0	0
0	0	0	0	3515	53	673	0	0	0	62	0	0	0	467	0	0	0
0	0	0	0	0	0	0	0	0	0	0	0	0	0	0	0	0	0
0	0	0	0	0	0	0	0	0	0	0	0	0	0	0	0	0	0
0	0	0	0	0	0	0	0	0	0	0	0	0	0	0	0	0	0
0	0	0	0	0	0	0	0	0	247	0	56	0	0	0	0	0	226
0	0	0	0	0	0	0	0	0	0	0	206	618	0	0	0	77	391
0	0	0	0	0	0	0	0	0	0	0	0	0	0	0	0	0	0
0	0	0	0	0	0	0	0	0	0	0	0	0	0	0	0	0	0
0	0	0	0	0	0	0	0	0	0	0	0	0	0	0	0	0	0
0	0	0	0	0	0	0	0	0	0	0	0	0	0	0	0	0	0
0	0	0	0	0	0	0	0	0	0	0	0	0	0	0	0	0	0
0	0	0	0	0	0	0	0	0	0	0	0	0	0	0	0	0	0
294	4928	13545	6338	1963	377	221	13170	92	3203	14086	66	8730	4216	8920	121319	1379	331
0	0	0	0	0	0	0	0	0	0	0	0	0	0	0	0	0	0
35746	676	109	161	585	0	163522	125	35275	539	0	0	0	0	653	248	80	0
0	0	0	0	0	0	0	0	0	0	0	0	0	0	0	0	0	0
0	0	0	0	0	0	0	0	0	0	0	0	0	0	0	0	0	0
0	1856	341	323	0	0	0	315	0	0	0	0	0	0	0	1375	0	0
0	0	0	0	0	0	0	0	0	0	0	0	0	0	0	115	0	0
0	0	0	0	0	0	0	0	0	0	0	0	0	0	0	0	0	0
0	0	0	0	0	0	0	0	0	0	0	0	0	0	0	0	0	0
0	0	0	0	0	0	0	0	0	0	0	0	0	0	0	0	0	0
0	0	0	0	0	0	0	0	0	0	0	0	0	0	0	0	0	0
0	0	0	0	0	0	0	0	0	0	0	0	0	0	0	0	0	0
0	0	0	0	0	0	0	0	0	0	0	0	0	0	0	0	0	0
0	0	0	0	0	0	0	0	0	0	0	0	0	0	0	0	0	0
0	0	0	0	0	0	0	0	0	0	0	0	0	0	0	0	0	0
1009	10424	402	183	2676	100	1997	2650	397	74004	59348	7703	45684	69171	70146	16299	28069	73484
0	0	0	0	0	0	0	0	0	0	0	0	1222	0	181	0	0	0
0	0	0	0	0	0	0	0	0	0	0	0	0	0	0	0	0	0
0	0	0	0	0	0	0	0	0	0	0	0	0	0	0	0	0	0
0	0	0	0	0	0	0	0	0	0	0	0	0	0	0	0	0	0
0	0	0	0	0	0	0	0	0	0	0	0	0	0	0	0	0	0
0	0	0	0	0	0	0	0	0	0	0	0	0	0	0	0	0	0
13332	0	0	0	0	0	0	0	0	0	0	0	0	0	110	0	0	0
0	0	0	0	0	0	767	0	701	0	0	0	0	0	0	0	0	0
0	0	0	0	0	0	0	0	0	571	0	0	0	0	0	0	194	57
0	0	0	0	0	0	0	0	0	0	0	0	0	0	0	0	0	0
0	0	0	0	0	0	12379	0	0	0	0	0	0	0	0	0	0	0
0	0	0	0	0	0	0	0	0	0	0	0	0	0	0	0	0	0
0	0	0	0	0	0	0	0	0	0	0	0	0	0	0	0	0	0
0	0	0	0	0	0	0	0	0	0	0	0	0	0	0	0	0	0
0	0	0	0	0	48	0	0	0	0	0	0	123	130	0	0	0	0
0	0	0	0	0	0	0	0	0	0	0	0	0	0	0	0	0	0
0	117	0	0	0	0	0	81	0	0	56	0	99	115	275	0	89	0
0	0	0	0	0	0	0	0	0	0	0	0	0	0	0	0	0	0
0	0	0	0	0	0	0	0	0	0	0	0	0	0	0	0	0	0
65756	0	0	0	0	0	0	0	96	0	0	0	0	0	0	0	0	0
0	0	0	0	0	0	0	0	0	0	0	0	0	0	0	0	0	0
0	0	0	0	0	63	14451	0	3874	0	0	0	0	0	100	53	0	0
0	0	0	0	0	0	0	0	0	0	0	0	0	0	0	0	0	0
0	0	0	0	0	0	0	0	0	0	66	0	826	349	0	0	94	225
0	0	0	0	0	0	0	0	0	0	0	0	0	0	0	0	0	0
0	0	0	0	0	0	0	0	0	0	0	0	0	120	113	0	181	127
0	0	0	0	0	0	0	0	0	0	0	0	0	0	0	0	0	0
0	0	0	0	0	0	0	0	0	0	0	0	0	0	0	0	0	0
0	0	0	0	0	0	0	0	0	0	0	0	0	0	0	0	0	857
0	0	0	0	0	0	0	0	0	0	0	0	0	0	0	0	0	0
0	0	0	0	0	0	0	0	0	0	0	0	0	0	0	0	0	0
0	0	0	0	0	0	0	0	0	0	0	0	0	0	0	0	0	0
0	0	0	0	0	0	0	0	0	0	0	0	0	0	0	0	0	0
0	0	0	93	0	0	80	0	0	0	0	0	0	0	0	0	0	0
0	0	0	0	0	0	0	0	0	0	0	0	0	0	0	0	0	0
0	0	0	0	0	0	0	0	0	0	0	0	0	0	0	0	0	0
32824	0	0	0	0	0	0	70	0	0	0	0	0	0	0	0	0	0
0	0	0	0	0	0	0	0	0	0	0	0	0	0	0	0	0	0
0	0	0	0	0	0	0	0	0	0	0	0	0	0	0	0	0	0
0	0	0	0	0	0	0	0	0	0	0	0	222	0	0	0	0	0
62	0	0	0	0	0	0	0	0	0	0	0	0	0	0	0	0	0
0	0	0	0	0	0	0	0	0	0	0	0	0	0	0	0	0	0
0	0	0	0	0	0	0	0	0	0	0	0	0	0	0	0	0	0
295	0	0	0	0	0	0	0	0	0	0	0	0	0	397	0	0	0
0	0	0	0	0	0	0	0	0	0	0	0	0	0	0	0	0	0
0	0	0	0	0	0	0	0	0	0	0	0	0	0	0	0	0	0
0	0	0	0	0	0	0	0	0	0	0	0	0	0	0	0	0	0
0	0	0	0	0	0	0	0	0	0	0	0	0	0	0	0	0	0
233	558	0	0	1853	0	203	266	0	5404	1834	69	1483	3733	3563	2677	754	895
0	0	0	0	0	0	0	0	0	0	0	0	0	0	0	0	0	0
0	0	0	0	0	0	0	0	0	0	0	0	0	0	0	0	0	0
0	0	0	0	0	0	0	0	0	0	0	79	0	0	0	0	0	0
0	0	0	0	55	0	0	0	0	0	0	0	0	0	0	0	0	0
0	0	0	0	0	0	0	0	0	0	0	0	0	0	0	0	0	0
0	0	0	0	0	52	0	0	0	0	0	0	0	0	0	0	0	0

0	0	0	0	5301	0	19397	0	97	0	0	0	218	262	108	0	0	0
0	0	0	0	0	0	0	0	0	0	0	0	0	0	0	0	0	0
0	0	0	0	0	0	0	0	91	285	0	426	0	0	0	0	0	0
0	0	0	0	0	67	0	0	0	0	0	0	0	0	0	0	0	0
0	0	0	0	0	0	0	0	0	0	0	0	0	0	0	0	0	0
0	0	0	0	0	0	0	0	0	0	0	0	0	0	0	0	0	0
0	0	0	0	0	0	0	0	0	0	0	0	0	0	0	0	0	0
74	0	0	0	0	0	0	0	0	0	0	0	0	0	0	0	0	0
0	0	0	0	0	0	0	0	0	0	0	0	0	0	0	0	0	0
0	0	0	0	0	0	0	0	0	0	0	0	0	0	0	0	0	0
68	0	0	0	1010	0	2617	0	79	268	0	0	0	0	0	0	93	0
0	0	0	110	0	0	0	0	0	0	0	0	1585	0	0	0	0	0
0	0	0	0	0	0	0	0	0	0	0	0	0	0	0	0	0	0
0	0	0	0	0	0	0	0	0	0	0	0	0	0	0	0	0	0
0	0	0	0	0	0	0	0	0	0	0	0	0	0	0	0	0	0
0	0	0	0	0	0	0	0	0	0	0	0	0	0	0	0	0	0
0	0	0	0	0	0	0	0	0	0	0	0	0	0	0	0	0	0
0	0	0	0	0	0	0	0	0	0	0	0	0	0	0	0	0	0
0	0	0	0	0	0	0	0	0	0	542	0	0	0	0	0	0	0
0	0	0	0	0	0	0	0	0	0	0	0	8350	0	0	0	0	0
75907	5016	6747	22088	351293	952781	39890	5318	3580	9326	20754	33732	81394	7586	6564	82321	284257	9530
0	0	0	0	0	0	0	0	0	0	0	0	0	0	0	0	0	0
0	0	0	0	0	0	79	0	0	582	79	0	0	0	0	0	0	53
0	0	0	0	0	0	0	0	0	0	0	0	0	0	0	0	0	47
0	0	0	0	0	0	0	0	0	0	0	0	0	0	0	0	0	0
0	0	0	0	0	0	0	0	0	0	0	0	0	0	0	0	0	0
0	0	0	0	0	0	0	0	0	0	0	0	0	0	0	0	0	0
0	0	0	0	955	0	0	0	0	0	0	0	97	130	93	602	0	0
0	0	0	0	0	0	0	0	0	0	0	0	0	0	0	0	0	0
CC061	CC060	CC059	CC058	CC056	CC054	CC036	CC057	CC034	CC014	CC013	CC012	CC011	CC010	CC009	CC015	CC008	CC006
0	0	0	0	0	0	0	0	0	0	0	0	0	0	0	0	0	0
0	0	0	0	0	51	0	0	0	0	0	0	0	0	0	0	0	0
0	0	0	0	0	0	0	0	0	0	0	0	0	0	0	0	0	0
0	0	0	0	0	0	0	0	0	0	0	0	0	0	0	0	0	0
0	108	0	0	0	0	0	53	0	0	605	0	0	119	184	0	91	111
0	0	0	0	0	0	0	0	0	0	0	0	0	0	0	0	0	0
0	0	0	0	0	0	0	0	0	0	0	0	0	0	0	0	0	0
27663	414174	5180	4616	63030	4619	38880	123508	6139	766102	775125	102305	654317	787942	776874	569574	585595	807465
0	0	0	0	0	0	0	0	0	0	0	0	0	0	0	0	0	0
0	0	0	0	0	0	0	0	0	0	0	0	0	0	0	0	0	0
0	0	0	0	0	0	0	0	0	0	0	0	0	0	0	0	0	0
0	0	0	0	0	0	0	0	0	0	0	0	0	0	0	0	0	0
0	130	121	0	0	0	0	59	0	0	0	0	0	0	202	0	0	0
0	0	0	0	0	0	0	0	0	0	0	0	0	0	92	0	0	0
0	0	0	0	0	0	0	0	0	0	0	0	0	0	0	0	0	0
0	0	0	0	0	0	0	0	0	0	0	0	0	0	0	0	0	0
230	0	0	0	0	0	300	0	0	0	0	0	0	0	0	0	0	0
36064	313	0	162	0	97	8775	136	107	0	82	0	12118	0	0	132	71	263
0	0	0	0	0	0	0	0	0	0	0	0	0	0	0	0	0	0
0	0	0	0	0	0	0	0	0	0	0	0	0	0	0	0	0	0
0	0	0	0	0	0	0	0	0	0	0	0	0	0	0	97	0	0
0	0	0	0	0	0	0	0	0	0	0	0	0	0	0	0	197	0
0	0	0	0	0	0	0	0	0	0	0	0	0	0	0	0	0	0
0	0	0	0	0	0	0	0	0	0	0	0	0	0	0	0	0	0
370	5293	0	0	536	711	722	1354	0	14260	15226	835	13209	14936	15127	3874	6457	9853
0	0	0	0	0	0	0	0	0	0	62	11404	0	0	0	0	0	0
0	0	0	0	0	0	0	0	0	0	0	0	0	0	0	0	0	0
0	0	0	0	0	0	0	0	0	0	0	0	0	0	0	0	0	0
0	0	0	0	0	0	0	0	0	0	0	0	0	0	0	0	0	0
0	0	0	0	0	0	0	0	0	0	0	0	0	0	0	0	0	0
0	0	0	0	0	0	5601	0	0	0	0	0	0	0	0	0	0	0
0	0	0	0	0	0	0	0	0	0	0	0	0	0	0	0	0	0
0	0	0	0	0	0	0	0	0	0	0	0	0	0	0	0	0	0
0	0	0	0	0	0	0	0	0	0	0	0	0	0	0	0	0	0
0	0	0	0	0	0	0	0	0	0	0	0	0	0	0	0	0	0
650	0	0	0	131	156	0	0	108	0	0	0	0	0	474	3647	0	115
0	0	0	0	0	0	0	0	0	0	0	214	0	0	0	0	0	0
0	0	0	0	0	0	1984	0	8698	0	0	0	114	0	0	0	0	0
0	0	0	0	0	0	0	0	0	0	0	0	0	0	0	0	0	0
0	0	0	0	0	0	0	0	0	0	0	0	0	0	0	2464	0	0
0	0	0	0	0	0	70	0	0	0	0	0	0	0	0	0	0	0
72	0	0	0	0	0	0	0	2142	0	0	0	1676	0	205	0	0	0
0	0	0	0	0	0	768	0	0	0	0	0	0	0	0	0	0	0
0	0	0	0	0	0	0	0	0	0	0	0	0	0	0	0	0	0
0	0	0	0	0	0	0	0	0	0	0	0	0	0	0	0	0	0
0	0	0	0	0	0	0	0	0	0	70	0	0	0	0	0	0	0
0	0	0	0	0	0	0	0	0	0	0	0	0	0	0	0	0	0
0	0	0	0	3343	0	0	0	1370	0	0	0	0	0	0	0	0	0
0	0	0	0	0	0	1001	0	0	0	0	0	0	0	0	0	0	0
0	0	0	0	0	0	0	0	0	0	0	0	0	0	0	0	0	0
0	0	0	0	0	0	0	0	0	0	0	0	0	0	0	0	0	0
0	0	0	0	0	0	0	0	0	0	0	0	0	99	0	0	0	0
0	0	0	0	0	0	0	0	0	0	0	0	0	0	0	0	0	0
0	0	0	0	0	0	0	0	0	0	0	0	0	0	0	0	0	0
0	0	0	0	0	0	0	0	0	0	0	0	0	0	0	0	0	0

0	0	0	0	0	0	1893	0	0	0	0	0	0	0	0	0	0	0
14123	468	0	0	7995	0	1428	137	1271	284	63	16736	108	0	0	0	0	50
0	0	0	0	0	0	0	0	0	0	0	3446	0	0	0	0	0	0
0	0	0	79	772	0	0	0	631	0	0	0	209	120	0	1213	0	0
11558	1953	514	405	4854	731	16982	471	86790	22438	13976	1378	9155	15969	22481	121980	3408	3760
0	0	0	0	0	0	6270	0	0	0	0	0	0	0	0	0	0	0
0	0	0	0	0	0	0	0	0	0	0	0	102	0	0	0	0	0
0	0	0	0	0	0	0	0	0	0	0	0	0	0	0	0	0	0
4042	0	110	0	0	0	21848	0	86	0	0	0	0	0	0	0	90	35
5499	0	97	81	0	57	1856	0	0	0	0	0	0	0	0	0	0	0
0	0	0	0	0	0	0	0	0	0	0	0	0	0	0	0	0	0
0	0	0	0	0	0	0	0	0	0	0	0	0	0	0	0	0	0
0	0	0	0	0	0	0	0	0	0	0	0	0	0	0	0	0	0
0	113	0	84	0	49	0	0	0	0	0	0	0	0	267	53	0	480
0	0	0	0	0	0	0	0	0	0	0	0	0	0	0	0	0	0
0	0	0	0	0	0	0	0	0	0	0	0	0	0	0	0	0	0
0	0	0	0	0	0	0	0	0	0	0	0	0	0	0	0	0	0
0	0	0	0	0	0	0	0	0	0	0	0	0	0	0	0	0	0
0	0	0	0	0	0	0	0	0	0	0	0	0	0	0	0	0	0
0	0	0	0	0	0	0	0	0	0	73	0	211	0	0	73	617	152
0	0	0	0	0	0	0	0	0	0	0	0	0	0	0	0	0	0
0	0	0	0	0	0	0	0	0	0	0	0	0	0	0	0	0	0
0	0	0	0	0	0	0	0	0	0	0	0	0	0	0	0	0	0
0	0	0	0	0	0	0	154	0	0	0	0	0	0	0	0	0	0
0	0	0	0	0	0	0	0	0	0	0	0	0	0	0	0	0	0
0	0	0	0	0	0	0	0	0	0	0	0	0	0	0	0	0	0
0	0	0	0	716	0	0	0	0	0	0	72	0	0	0	0	0	0
0	0	0	0	0	0	0	0	0	0	0	0	0	0	0	0	0	0
0	0	0	0	0	0	0	0	0	0	0	0	0	0	0	0	0	0
0	0	0	0	0	0	0	0	0	0	0	0	0	0	0	0	0	0
0	0	0	0	0	0	0	0	0	0	0	0	0	0	0	0	0	0
0	0	0	0	0	64	0	0	0	0	0	0	0	0	0	0	0	0
0	0	0	0	0	0	0	0	0	0	212	0	0	0	0	0	0	0
0	0	0	0	0	0	0	0	0	0	0	0	0	0	0	0	0	0
0	0	0	0	0	0	0	0	0	0	0	0	0	0	0	0	0	0
359	208	112	186	2549	781	18338	156	90838	2798	485	1564	4244	575	473	0	787	951
0	0	0	0	0	0	0	0	0	0	0	0	0	0	0	0	0	0
0	0	0	0	0	0	0	0	0	0	0	0	0	0	0	62	0	0
0	0	0	0	0	0	0	0	0	0	0	0	0	0	0	0	0	0
0	0	0	0	0	0	63	0	3945	0	0	0	0	0	0	0	0	41
0	0	0	0	0	0	0	0	0	0	0	0	0	0	0	0	0	0
0	0	0	0	0	0	0	0	0	0	0	0	0	0	0	0	0	0
0	0	0	0	0	0	0	0	0	0	0	0	0	0	0	53	0	0
0	0	0	0	0	0	0	0	0	0	0	0	0	0	0	0	0	0
0	0	0	0	0	0	0	0	0	0	0	0	0	0	0	0	0	0
0	0	0	0	0	0	63	0	0	0	0	0	0	0	0	56	0	0
0	0	0	0	0	0	0	0	0	0	0	0	0	0	0	0	0	0
0	0	0	0	0	0	0	0	0	0	0	0	0	0	0	0	0	0
0	0	0	0	0	0	0	0	0	0	0	0	0	0	0	0	0	0
0	0	0	0	0	0	0	0	0	0	0	0	0	0	0	0	0	0
0	352	0	0	0	0	0	64	0	1106	678	64	105	1043	1339	3485	447	1058
0	0	0	0	0	0	0	0	0	0	0	0	0	0	0	0	0	0
0	0	0	0	0	0	0	0	0	0	0	0	0	0	0	0	0	0
733	835	1226	484991	4007	7798	1090	774	8464	4542	846	2301	0	565	0	1753	1500	2105
0	0	0	0	0	0	0	0	186	0	69	69	0	0	0	0	102	0
0	0	0	0	0	0	0	0	96	0	0	0	0	0	0	0	0	0
0	0	0	0	0	0	0	0	0	0	0	0	0	0	0	0	0	0
0	0	0	0	0	0	0	0	0	0	0	0	0	0	0	0	0	0
67	0	0	0	0	0	0	0	0	0	0	0	0	0	202	0	0	0
0	0	0	0	0	0	0	0	0	0	0	0	0	0	0	0	0	0
0	0	0	0	0	0	0	0	0	0	0	0	0	0	0	0	0	0
0	0	0	0	83	0	0	57	0	0	72	0	0	0	0	0	0	0
0	0	0	0	0	0	0	0	0	0	0	0	0	0	0	0	0	0
0	766	1768	1188	0	0	0	1481	0	0	0	0	0	0	0	0	0	0
0	0	0	0	0	0	0	0	90	0	0	0	0	0	0	0	0	0
69	24707	2331	820	0	57	0	1278	205	882	538	290	5623	2248	464	56	167	532
61	0	0	0	0	51	0	0	0	528	0	157	0	0	0	0	0	158
0	2831	101	96	0	0	0	213	0	0	0	0	881	336	111	0	0	55
0	0	0	0	0	0	0	0	0	277	68	0	806	0	0	0	0	0
0	0	0	0	0	0	3260	0	0	0	0	0	0	0	0	0	0	0
0	0	0	0	0	0	0	0	0	0	0	0	0	0	0	0	0	0
0	0	0	0	0	0	0	0	0	0	0	0	0	0	0	0	0	0
0	0	0	0	0	0	0	0	0	0	0	0	0	0	0	0	0	0
0	0	0	0	0	0	0	0	0	0	0	0	0	0	0	0	0	59
0	0	0	0	0	0	0	0	0	0	0	0	0	0	0	0	0	0
0	0	0	0	2238	0	0	0	0	0	0	0	0	0	0	0	0	0
0	0	0	0	0	0	0	0	0	0	0	0	0	0	0	0	0	0
0	0	0	0	0	0	0	0	0	0	0	0	0	0	0	0	0	0
0	0	0	0	0	0	0	0	0	0	0	0	0	0	0	0	0	0
0	0	0	0	0	0	0	0	0	0	0	0	0	0	0	0	0	0
0	0	0	0	0	0	0	0	0	0	0	0	0	0	0	0	0	0
0	0	0	0	0	0	0	0	0	0	0	0	0	0	0	0	0	0
0	0	0	0	0	0	0	0	0	0	0	0	0	0	0	0	0	0
0	0	0	0	0	0	0	0	0	0	0	0	0	0	0	61	0	0
0	0	0	0	0	0	0	0	0	0	0	0	0	0	0	0	0	0
0	0	0	0	60	0	222836	0	723276	0	64	77	213	0	0	0	83	0
0	0	0	0	0	0	0	0	0	0	0	0	0	0	0	0	0	0

0	4252	6639	3352	0	0	0	6697	0	0	0	0	0	0	0	0	0	0
0	0	0	0	0	0	0	0	0	0	0	0	0	0	0	0	0	0
0	0	105	0	0	0	0	0	0	0	0	0	0	0	0	0	0	0
0	0	0	0	0	0	0	0	0	0	0	0	0	0	0	0	0	0
141	221	208	67	420	53	74007	55	93	270	504	0	3022	0	195	3363	109	130
0	0	0	0	502	0	1715	0	0	0	0	0	0	0	0	0	0	0
0	1393	203	338	24597	0	45858	276	93	849	758	395	0	102	0	6512	621	520
111	107	0	92	5552	57	38856	200	365	0	210	64	220	0	0	540	0	44
0	112	0	0	0	0	1887	0	0	0	0	0	0	0	0	0	0	0
0	109	0	70	0	0	0	0	80	0	0	0	720	0	0	61	97	0
0	0	0	0	0	0	0	0	0	0	0	0	0	0	0	0	0	0
0	0	0	0	0	0	0	0	0	0	0	0	0	0	0	0	0	0
0	0	0	0	0	0	0	0	0	0	0	0	0	0	0	0	0	0
0	0	0	0	0	0	0	0	0	0	0	0	0	0	0	0	0	0
0	0	0	0	0	0	0	0	0	0	0	0	0	0	0	0	0	0
0	0	0	0	0	0	0	0	0	0	0	0	0	0	0	0	0	0
0	0	0	0	0	0	0	0	0	0	0	0	1386	0	0	0	0	0
0	0	0	0	0	0	0	0	0	0	0	0	0	0	0	0	0	0
0	0	0	0	0	0	0	0	0	0	72	0	0	0	0	0	0	0
0	0	0	0	0	0	0	0	0	0	0	0	0	0	0	0	0	0
0	0	0	0	0	0	0	0	0	0	0	0	0	0	0	205	0	0
0	0	0	0	0	0	0	0	0	0	0	0	0	0	0	0	0	0
0	0	0	0	0	0	0	0	0	0	0	0	0	0	0	0	0	0
0	0	0	0	0	0	0	0	0	0	0	0	0	0	0	56	0	0
0	0	0	0	0	0	0	0	0	0	0	0	0	0	0	0	0	59
0	0	0	0	0	0	0	0	0	0	0	0	0	0	0	0	0	0
0	0	0	0	0	0	0	0	0	0	0	0	0	0	0	0	0	0
0	0	0	0	0	0	0	0	0	0	0	0	0	0	0	0	0	0
0	116	0	0	0	0	0	0	0	0	0	0	0	210	177	66	106	42
0	0	0	0	0	0	0	0	0	0	0	0	0	0	0	0	0	52
0	0	0	0	0	0	0	0	0	0	0	0	0	0	0	0	0	0
0	0	0	0	0	0	0	0	0	0	0	0	0	0	0	0	0	0
0	0	0	0	0	0	0	0	0	0	0	0	0	0	0	0	0	0
0	0	0	0	0	0	0	0	0	0	0	0	0	0	0	0	0	0
0	0	0	0	0	0	0	0	0	0	0	0	0	0	0	0	0	0
0	0	0	0	0	0	0	0	0	0	0	0	0	0	0	0	0	0
0	0	0	0	0	0	0	0	0	0	0	0	0	0	0	0	0	0
0	0	0	0	0	0	0	0	0	0	0	0	0	0	0	0	0	0
0	0	0	0	0	0	0	0	0	0	0	0	0	0	0	0	0	0
0	0	0	0	0	0	0	0	89	290	0	60	0	0	0	0	188	68
0	0	0	0	0	0	0	0	0	0	0	0	0	0	0	0	0	0
0	0	0	0	0	0	0	0	0	0	0	0	0	0	0	0	0	0
0	0	0	0	0	0	0	0	0	0	0	0	0	0	0	0	0	0
0	0	0	0	0	0	0	0	0	0	0	0	0	0	0	0	0	0
0	0	0	0	0	0	0	0	0	0	0	0	0	0	0	0	0	0
0	0	0	0	0	47	0	0	0	0	0	0	0	0	0	0	0	0
0	0	0	0	0	0	0	0	0	0	0	0	0	0	0	0	0	0
0	0	0	0	0	0	0	0	0	0	0	0	0	0	0	0	0	0
0	0	0	0	0	0	0	0	0	0	0	0	0	0	0	0	0	0
0	0	0	0	0	0	0	0	0	0	0	0	0	0	0	0	0	0
0	0	0	0	0	0	0	0	0	0	0	0	0	0	0	0	0	0
0	0	0	0	0	0	0	0	0	0	0	0	0	0	0	0	0	0
0	0	0	0	0	0	0	0	0	0	0	0	0	0	0	0	0	0
0	0	0	0	0	0	0	0	0	274	0	0	18616	0	0	0	0	0
0	0	0	0	0	0	0	0	0	0	0	0	0	0	0	0	0	0
0	0	0	0	0	0	0	0	0	0	0	0	0	0	0	0	0	0
0	0	0	0	0	0	0	0	0	0	0	0	0	0	0	0	0	0
0	0	0	0	0	0	0	0	0	0	0	0	0	0	0	0	0	0
0	0	0	0	0	0	0	0	0	0	0	0	0	0	0	0	0	0
0	0	0	0	0	0	0	0	0	0	0	0	0	0	0	0	0	0
0	0	0	0	0	0	0	0	0	0	0	0	0	0	0	0	0	0
0	0	0	0	0	0	0	0	0	0	0	0	0	0	0	0	0	0
0	0	0	0	0	0	0	0	0	0	0	0	0	0	0	0	0	0
0	0	0	0	0	0	0	0	0	0	0	0	0	0	0	0	0	0
0	0	0	0	0	0	0	0	0	0	0	0	0	0	0	0	0	0
0	0	0	0	0	0	0	0	0	0	0	0	0	0	0	0	0	0
0	0	0	0	0	0	0	0	0	0	0	347	0	0	0	0	0	0
0	0	0	0	0	0	0	0	0	0	0	0	0	0	0	0	0	0
0	0	0	0	0	0	0	0	101	0	0	0	0	0	0	0	0	0
0	0	0	0	0	0	0	0	0	0	0	0	1979	0	0	0	0	0
0	0	0	0	0	0	0	0	0	0	0	0	0	0	0	0	0	0
0	0	0	0	0	0	0	0	0	0	0	0	0	125	0	0	0	0
0	123	0	0	0	0	0	0	0	0	0	0	0	0	0	0	0	0
0	0	0	0	0	105	0	0	0	0	0	0	0	0	0	0	0	0
0	0	0	0	0	0	0	0	0	0	0	0	0	0	0	0	0	54
2459	203	39921	839	1626	1093	2062	14359	0	908	3008	0	4387	807	2142	10818	360	1763
0	0	0	0	0	0	0	0	0	0	0	0	0	0	0	0	0	0
0	0	0	0	0	0	0	0	0	0	0	0	0	0	0	0	0	0
0	0	0	0	0	0	0	0	0	0	0	0	0	0	0	0	0	60
0	0	0	0	0	0	0	0	0	0	0	0	0	0	0	0	0	0
0	0	0	0	0	0	0	0	0	0	0	0	0	0	0	0	0	0
0	0	0	0	0	0	0	0	0	0	0	0	0	0	0	0	0	0
0	0	0	0	0	0	0	0	0	0	0	0	412	0	0	0	0	57
0	0	0	0	0	0	0	0	0	0	0	0	0	0	0	0	0	0
0	0	0	0	0	0	0	0	0	0	0	0	0	0	0	0	0	0
0	0	0	0	0	0	0	81	0	0	0	0	0	0	0	0	0	0
3042	0	0	90	0	0	235	0	335	0	66	0	0	0	0	0	0	0

0	0	0	0	0	0	0	0	0	0	0	0	0	0	0	260	0	0
66	0	0	0	0	0	77	124	90	8164	6122	153	926	5233	7339	5799	619	347
0	0	0	0	0	0	0	0	0	0	899	0	0	0	82	0	94	129
0	0	0	0	0	0	0	0	0	0	0	0	0	0	0	0	0	0
0	0	0	0	0	0	0	0	0	0	0	0	0	0	0	0	0	49
0	0	0	0	0	0	0	0	0	0	0	0	0	0	0	0	0	0
0	0	0	0	0	0	0	0	0	283	0	22122	0	0	0	0	0	0
0	0	0	0	0	0	0	0	0	0	0	0	0	0	0	0	0	0
0	0	0	0	0	0	4232	0	0	0	0	0	0	0	0	0	0	0
0	0	0	0	0	0	0	0	0	0	0	0	0	0	0	0	0	0
0	0	0	0	0	0	0	0	0	0	0	0	0	0	0	0	0	0
0	0	0	0	0	0	0	0	0	0	0	0	0	0	0	0	0	0
0	0	0	0	0	0	0	0	0	0	0	0	0	0	0	0	0	0
71	0	0	0	0	0	2428	0	0	0	0	0	0	0	0	0	0	0
0	0	0	0	0	0	0	0	0	0	0	6776	0	0	0	0	0	0
0	0	0	0	0	0	0	0	0	0	0	0	0	0	0	0	0	0
0	0	0	0	0	0	0	0	0	0	0	0	0	0	0	0	0	0
0	0	0	0	0	0	0	0	0	0	0	0	0	0	0	0	0	0
0	0	0	0	0	0	0	0	0	0	0	0	0	0	0	0	0	0
0	0	0	0	0	52	0	0	0	0	0	0	0	0	0	0	0	0
0	0	0	0	0	0	0	0	0	0	0	0	0	0	0	0	0	0
0	0	0	0	0	0	0	0	0	0	0	0	0	0	0	0	0	0
68	2931	0	0	3802	0	529	618	800	5892	8765	25928	9049	9107	8950	2542	5162	9648
0	0	0	0	0	0	0	0	0	0	0	0	0	0	0	0	0	0
0	0	0	0	0	0	151	0	516	0	0	24474	0	0	0	0	81	143
0	0	0	0	0	0	0	0	0	0	0	0	0	0	0	0	0	0
0	0	0	0	0	0	0	0	0	0	0	0	0	0	0	0	0	0
0	0	0	0	0	0	0	0	0	0	0	0	0	0	0	0	0	0
0	0	0	0	0	0	0	0	0	0	0	0	0	0	0	0	0	0
0	0	0	0	0	0	0	0	0	0	0	0	0	0	0	0	0	0
0	0	0	0	0	0	0	0	0	0	0	0	0	0	0	0	0	0
0	0	0	0	0	0	0	0	0	0	0	0	0	0	0	0	0	0
0	0	0	0	0	0	0	0	0	0	0	0	0	0	0	0	0	0
214178	5665	1184	159	10256	26836	54139	1751	5044	20955	20092	20449	31141	20652	19868	10560	36330	47315
0	0	0	0	0	56	2667	0	0	0	0	0	0	0	0	0	0	0
0	0	0	0	0	0	0	0	0	0	0	0	0	0	0	0	0	0
0	439	0	65	0	0	0	0	185	0	0	0	114	0	0	0	0	0
0	0	0	0	0	0	0	0	0	0	0	0	0	0	0	0	0	0
0	0	0	0	0	0	0	0	0	0	0	0	0	0	0	0	0	0
0	0	0	0	0	0	7274	0	98	0	228	0	1390	0	0	0	0	0
0	0	0	0	0	0	0	0	0	0	0	0	0	0	0	0	0	0
0	0	0	0	0	0	70	0	357	0	0	0	0	0	0	0	0	0
0	0	0	0	0	0	0	0	0	0	0	0	0	0	0	0	0	0
0	0	0	0	14767	52	0	0	0	0	0	0	0	0	0	0	0	0
242	0	0	182	0	54	0	0	0	0	0	0	0	245	186	0	0	0
0	0	0	0	0	0	0	0	0	0	0	0	0	0	0	0	0	0
83	0	0	0	0	0	0	0	0	0	0	0	0	0	0	0	0	138
0	0	0	0	0	0	0	0	0	0	0	0	0	0	0	0	0	0
0	0	0	0	0	0	0	0	0	0	0	0	0	0	0	0	0	0
0	0	0	0	0	0	0	0	0	0	0	0	0	0	0	0	0	0
0	0	0	0	0	0	0	0	0	0	0	0	0	0	0	0	0	0
0	0	0	0	8371	0	59279	0	4224	0	69	0	320	0	0	54	0	0
0	0	0	0	0	0	0	0	0	0	0	0	0	0	107	0	0	176
0	0	0	0	0	0	0	0	0	0	0	0	331	0	0	0	0	0
0	0	0	0	0	0	0	0	0	0	0	0	0	0	0	0	0	0
0	0	0	0	0	0	0	0	0	0	0	0	0	0	0	0	0	0
0	0	0	0	0	0	0	0	0	0	0	0	0	0	0	0	0	0
0	0	0	0	0	0	0	0	0	0	0	0	0	0	0	0	0	0
0	0	0	0	0	0	0	0	0	0	0	0	0	0	0	0	0	0
0	0	0	0	0	0	0	0	0	0	0	0	0	0	0	0	0	0
0	0	0	0	0	0	0	0	0	0	0	0	0	0	0	0	0	0
0	0	0	0	0	0	0	0	0	0	0	0	0	0	0	0	0	0
0	0	0	0	0	0	0	0	0	0	0	0	0	0	0	0	0	0
0	0	0	0	0	0	0	0	0	0	0	0	0	0	0	0	0	0
0	0	0	0	0	0	0	0	0	0	0	0	0	0	0	0	0	0
0	0	0	0	0	0	0	0	0	0	0	0	0	0	0	0	0	0
0	0	0	0	0	0	0	0	0	0	0	0	0	0	0	0	0	0
0	0	0	0	0	0	0	0	0	0	84	0	0	0	0	0	0	0
0	0	0	0	0	0	0	0	444	0	0	0	0	0	0	0	0	0
0	0	0	0	0	0	0	0	0	0	0	0	0	0	0	0	0	0
0	0	0	0	0	0	0	0	0	0	0	0	0	0	0	0	0	0
0	0	0	0	0	0	0	0	0	0	0	0	0	0	0	0	0	0
0	112	0	0	0	46	0	184	0	0	620	0	420	118	385	109	653	195
0	0	0	0	0	0	0	0	0	0	0	0	0	0	0	0	0	0
0	0	0	0	0	0	0	0	0	0	0	0	0	0	0	0	0	0
0	0	0	0	0	0	0	0	216	0	0	0	0	0	0	0	0	0
0	0	0	0	0	0	0	0	0	0	0	0	0	0	0	0	0	0
0	0	0	0	0	0	0	0	0	0	0	0	0	0	0	0	0	0
0	0	0	0	0	0	0	0	0	0	0	0	0	0	0	0	0	49
0	0	0	0	0	0	0	0	0	0	0	0	0	0	0	0	0	0
0	0	0	0	0	0	0	0	0	0	0	0	0	0	0	0	0	0
195	0	0	0	0	0	0	0	0	0	0	0	0	0	0	0	0	0
0	0	0	0	0	0	0	0	0	0	0	0	0	0	0	0	0	0
0	0	0	0	0	0	0	0	0	0	0	0	0	0	0	0	0	0
0	461882	916892	471317	5313	379	8673	812460	1438	289	129	0	0	123	387	0	98	112
0	0	0	0	0	0	0	0	0	0	0	0	0	0	0	0	0	0
0	0	0	0	0	0	0	0	0	0	0	0	0	0	0	0	0	0

0	0	0	0	0	0	0	0	0	0	0	0	0	0	0	0	0	0
0	0	0	0	0	0	0	0	0	0	0	0	0	0	0	0	0	0
0	0	0	0	0	0	0	0	0	0	0	0	0	0	0	49	0	0
0	0	0	0	0	0	0	0	0	0	0	0	0	0	0	0	0	0
0	0	0	0	0	0	0	0	0	0	0	0	0	0	0	0	0	0
0	0	0	0	0	0	0	0	0	268	0	0	0	0	0	0	0	0
0	0	0	0	0	0	0	0	0	0	0	0	0	0	0	0	0	0
0	0	0	0	0	0	0	0	0	0	0	81	0	0	0	0	79	102
0	0	0	0	0	0	0	0	0	0	0	0	0	0	0	0	0	0
0	0	0	0	0	0	0	0	0	0	0	0	0	0	0	0	0	0
0	0	0	0	0	0	0	0	0	0	0	0	0	0	0	0	0	0
140	540	0	0	142	0	0	0	0	552	535	0	0	362	689	65	398	303
0	0	0	0	0	0	0	0	95	0	0	0	0	0	0	0	0	0
0	0	0	0	0	0	0	0	0	0	0	0	0	0	0	0	0	0
0	0	0	0	0	0	0	0	0	0	0	0	0	0	0	0	0	0
0	0	0	0	0	0	0	0	0	0	0	0	0	0	0	0	0	0
0	0	0	0	0	0	0	0	0	0	0	0	0	0	0	0	0	0
0	0	0	0	0	0	0	0	0	0	0	0	0	0	0	0	0	0
0	0	0	0	0	0	0	0	0	0	0	0	0	0	0	0	0	0
0	0	0	0	0	0	0	0	0	0	0	0	0	0	0	0	0	0
0	0	0	0	0	0	0	0	0	0	0	0	0	0	0	0	0	0
0	0	0	0	0	0	0	0	0	0	0	0	0	0	0	0	0	0
0	0	0	0	0	205	0	0	0	0	0	0	0	0	0	0	0	0
0	0	0	0	0	0	0	0	0	0	0	0	0	0	0	0	0	0
0	0	0	0	0	0	0	0	0	0	0	0	0	0	0	0	0	0
0	0	0	0	0	0	0	0	0	0	0	0	0	0	0	0	0	0
0	0	0	0	0	0	0	0	0	0	0	0	0	0	0	0	0	0
0	0	0	0	0	0	0	0	0	0	0	0	0	0	0	0	0	0
0	0	0	0	0	0	0	0	0	0	0	0	0	0	0	0	0	0
0	0	0	0	0	0	0	0	0	0	0	0	0	0	0	0	0	0
0	0	0	0	0	0	3547	0	0	0	0	0	0	0	0	0	0	0
0	121	0	0	0	0	0	71	0	0	0	0	0	0	0	59	0	0
0	0	0	0	0	62	0	0	0	0	0	0	0	0	0	0	0	0
0	0	0	0	0	0	0	0	0	0	0	0	0	0	0	0	0	0
0	0	0	0	0	0	0	0	0	0	0	0	0	0	0	0	0	0
0	0	0	0	0	57	17354	0	0	0	0	0	0	0	0	0	0	0
0	0	0	0	0	0	0	0	0	0	0	0	0	0	0	0	0	0
0	0	0	0	0	0	0	0	0	0	0	0	0	0	0	0	0	0
0	0	0	0	0	68	0	0	0	0	0	0	0	0	0	0	0	0
0	0	0	0	0	0	0	0	0	0	0	0	0	0	0	0	0	0
0	0	0	0	0	0	0	0	0	0	0	0	0	0	0	0	0	0
0	0	0	0	0	0	0	0	0	0	0	0	0	0	0	0	0	0
0	0	0	0	0	0	0	0	0	0	0	0	0	0	0	0	0	0
0	0	0	0	0	0	0	0	0	0	0	0	0	0	0	0	0	0
0	0	0	0	0	0	0	0	0	0	0	0	0	0	0	0	0	0
0	0	0	0	53540	0	681	0	0	0	0	7113	0	0	0	54	78	0
0	0	0	0	0	0	0	0	0	0	0	0	0	0	0	0	0	0
0	0	0	0	0	0	0	0	0	0	0	0	0	0	0	0	0	0
0	0	0	0	0	0	0	0	0	0	0	0	0	0	0	0	0	0
2651	0	0	0	2406	0	142	0	84	0	0	0	99	0	0	63	0	0
0	0	0	0	0	0	0	0	0	0	0	0	0	0	0	0	0	0
207836	695	312	85	395953	614	1959	231	940	817	75	62824	5701	0	1162	119	559	454
6188	0	116	86	0	0	0	0	0	0	0	0	0	0	0	121	0	0
0	0	0	0	0	0	0	0	0	0	0	0	0	0	0	0	0	0
0	0	0	0	0	0	0	0	0	0	0	0	0	0	0	0	0	0
0	0	0	0	0	0	0	0	0	0	0	0	0	0	0	0	0	0
0	122	0	0	0	0	0	0	0	0	0	0	0	0	0	0	0	0
0	0	0	0	0	0	0	0	0	0	0	0	0	0	0	0	80	0
0	0	0	0	0	0	0	0	0	0	0	0	0	0	0	0	0	0
0	0	0	0	0	0	0	0	0	0	0	0	0	0	0	0	0	0
0	962	107	0	0	62	79	126	0	1383	1630	13876	322	811	1658	0	282	797
0	0	0	0	0	0	0	0	0	0	0	0	0	0	0	0	0	0
0	0	0	0	137	35	0	0	0	0	0	0	0	0	0	0	0	0
0	0	0	0	0	0	0	0	0	0	0	0	0	0	0	0	0	0
0	0	0	0	0	0	0	0	0	0	0	0	0	0	0	0	0	0
0	0	0	0	0	0	0	0	0	0	0	0	0	0	0	0	0	0
0	0	0	0	0	0	0	0	0	0	0	0	0	0	0	0	0	0
0	0	0	0	0	0	0	0	0	0	0	0	0	0	0	0	0	0
0	0	0	0	0	0	0	0	0	0	0	0	0	0	0	0	0	0
0	0	0	0	0	0	0	0	0	0	0	0	0	0	0	0	0	0
0	0	0	0	5505	60	10738	0	2483	0	0	0	0	0	0	0	0	0
0	0	0	0	0	0	0	0	0	0	0	0	0	0	0	0	0	52
0	0	0	0	0	0	0	0	0	0	0	0	0	0	0	0	0	0
0	0	0	0	0	0	0	0	0	0	0	0	651	0	0	0	0	0
0	0	0	0	0	0	0	0	0	0	0	0	0	0	0	0	0	0
0	0	0	0	0	0	0	0	0	0	0	0	0	0	0	0	0	0
0	0	0	0	0	0	0	0	0	0	0	0	0	0	0	0	0	0
0	0	0	0	0	0	0	0	0	0	0	0	0	0	0	0	0	0
0	0	0	0	0	0	0	0	0	0	0	0	0	0	0	0	0	0
0	0	0	0	0	0	0	0	0	0	0	0	0	0	0	0	0	0
0	0	0	0	0	0	0	0	0	0	0	0	0	0	0	0	0	0
0	0	0	0	0	0	0	0	88	0	0	0	0	0	0	0	0	0
0	0	0	0	0	0	0	0	0	0	0	0	0	0	0	0	0	0
0	0	0	0	0	0	0	0	0	0	0	0	0	0	0	0	0	0
0	0	0	0	0	0	82	0	0	0	0	0	0	0	0	0	0	0
0	0	0	0	0	0	0	0	0	0	0	0	0	0	0	0	0	0
0	0	0	0	0	0	0	0	0	0	0	0	0	0	0	0	0	0

0	114	105	0	0	0	0	75	91	2595	792	609723	0	0	0	56	812	1683
0	116	0	0	0	0	0	0	0	0	0	0	0	0	0	0	0	57
0	0	0	0	0	0	0	0	0	0	0	0	0	0	0	0	0	0
0	0	0	0	0	0	0	0	0	0	0	0	0	0	0	0	0	0
179027	0	0	0	0	53	0	0	0	576	433	263	0	0	197	55	91	0
0	0	0	0	0	0	0	0	0	0	0	0	0	0	0	0	0	0
0	0	223	0	0	0	0	555	0	0	0	0	0	0	0	0	0	0
0	0	0	0	0	0	0	0	0	0	0	0	0	0	0	0	0	0
0	0	0	0	0	0	0	0	0	0	0	0	0	0	0	0	0	0
0	0	0	0	0	0	0	0	0	0	0	0	0	0	0	0	0	0
0	0	0	0	0	0	0	0	0	0	0	0	0	0	0	0	0	0
0	0	0	0	0	0	0	0	0	0	0	0	0	0	0	0	0	0
0	0	0	0	0	0	0	0	0	0	0	0	0	0	0	0	0	0
0	0	0	0	67	0	0	65	0	296	527	0	0	373	705	0	106	47
0	0	0	0	0	0	0	0	0	0	0	0	0	0	0	0	0	0
0	0	0	0	0	0	0	0	0	0	0	0	0	0	0	0	0	0
0	0	0	0	0	0	0	0	0	0	0	0	0	0	0	63	0	0
0	0	0	0	0	0	0	0	0	0	505	0	0	0	0	0	0	0
0	0	0	0	0	0	0	83	0	0	0	0	0	0	0	0	0	0
0	0	0	0	0	0	0	0	0	307	77	0	0	0	0	0	86	207
0	0	0	0	0	0	0	0	0	0	0	0	0	0	0	0	0	0
343	4962	0	0	228	938	400	1732	95	10200	11236	620	10124	11190	11902	3858	7786	7269
0	0	0	0	0	0	0	0	0	0	0	0	0	0	0	0	0	0
0	0	0	0	0	0	0	0	0	0	0	0	0	0	0	0	0	0
0	0	0	0	0	0	0	0	0	0	0	0	0	0	0	0	0	0
0	0	0	0	0	0	0	0	0	0	0	0	0	0	0	0	0	0
0	0	0	0	0	0	0	0	0	0	0	0	0	0	0	0	0	0
0	0	0	0	0	0	0	0	0	0	0	0	0	0	0	0	0	0
0	0	0	0	0	50	290	136	0	1137	503	0	729	583	180	60	5299	489
0	0	0	0	0	0	0	0	0	0	0	0	0	0	0	0	0	0
0	0	0	0	0	0	4872	0	0	0	0	0	0	0	0	0	0	0
0	0	0	0	0	0	0	0	0	0	213	0	0	218	215	0	95	253
0	0	0	0	0	0	0	0	0	0	0	0	0	0	0	0	0	0
143	0	0	0	0	0	0	0	0	0	0	0	0	0	0	0	0	0
0	0	0	0	0	0	0	0	182	0	0	0	0	0	0	0	0	0
0	0	0	0	0	0	0	0	0	0	0	0	0	0	0	0	0	0
0	0	0	0	0	0	68	0	0	0	0	0	0	0	0	0	0	0
0	0	0	0	0	0	0	0	0	0	0	78	0	0	0	105	0	0
0	0	0	0	0	0	1137	0	0	0	0	0	0	0	0	0	0	0
0	0	0	0	0	0	306	0	0	0	0	0	0	0	0	0	0	0
0	0	0	0	0	0	1669	0	0	270	0	0	0	0	0	0	0	0
4721	544	341	150	0	0	0	62	0	0	0	0	317	0	0	56	0	0
0	0	0	0	0	0	0	0	0	0	0	0	0	0	0	0	0	0
75	0	0	0	0	0	0	0	0	0	0	0	0	0	0	0	0	0
0	0	0	0	0	0	63	0	0	0	0	0	0	0	0	0	0	0
0	0	0	0	0	0	0	0	0	0	0	0	0	0	0	0	0	0
0	0	0	0	0	0	0	0	0	0	0	0	317	0	0	0	0	0
0	0	0	0	0	0	0	0	0	0	0	0	0	0	0	0	0	0
0	0	0	0	0	0	1289	0	0	0	0	0	0	0	0	0	0	0
0	0	0	0	0	0	0	0	0	0	0	0	0	0	0	0	0	0
0	0	0	0	0	0	0	0	0	0	0	0	0	0	0	0	0	0
0	0	0	0	0	0	0	0	0	0	0	0	0	0	0	0	0	0
0	0	0	0	0	0	0	0	0	0	0	0	0	0	0	0	0	0
0	0	0	0	0	0	0	0	0	0	0	0	0	0	0	0	0	0
0	0	0	84	0	0	0	0	0	0	0	0	0	0	0	0	0	0
0	0	0	0	0	0	76	0	0	0	0	0	0	0	0	0	0	0
0	0	0	0	0	0	0	0	0	0	0	0	0	0	0	0	0	0
0	0	0	0	0	0	0	0	0	0	0	0	0	0	0	0	0	0
0	0	0	0	0	0	0	0	0	0	0	0	0	0	0	0	0	0
0	0	0	0	0	0	0	0	0	0	0	0	0	0	0	0	0	0
0	0	0	0	0	0	0	0	0	0	0	0	0	0	0	0	0	0
0	112	0	0	0	0	0	66	0	0	86	0	106	428	279	0	3102	297
0	0	0	0	0	0	0	0	0	0	0	0	0	0	0	0	0	0
0	0	0	0	0	0	0	0	0	0	0	0	0	0	0	0	0	0
0	0	0	0	0	0	0	0	0	0	0	0	0	0	0	0	0	0
0	0	0	0	0	0	0	0	0	0	0	0	0	0	0	0	0	0
0	0	0	0	0	0	0	0	0	0	0	0	0	0	0	0	0	0
0	0	0	0	0	0	0	0	0	0	0	0	0	0	0	0	0	0
0	0	0	0	0	0	0	0	0	0	0	0	330	0	0	0	0	0
0	0	0	0	0	0	0	0	0	0	0	0	0	0	0	0	0	0
0	0	0	77	0	0	0	0	0	0	0	0	0	0	0	0	0	0
0	0	0	0	0	0	0	0	0	0	0	0	0	0	0	0	0	0
0	0	0	0	0	0	0	0	0	0	0	0	0	0	0	0	0	0
0	0	0	0	0	0	0	0	0	0	0	0	0	0	0	0	0	0
0	0	0	0	0	0	0	0	0	0	0	0	0	0	0	0	0	0
0	221	0	0	0	51	0	0	0	0	0	0	0	0	0	0	0	0
0	0	0	0	0	0	0	0	0	0	0	0	0	0	0	0	0	0
0	0	0	0	0	0	0	0	0	0	114	0	0	0	0	0	0	0
0	0	0	0	0	0	0	0	0	0	0	0	0	0	0	0	0	0
0	0	0	0	0	0	0	0	0	0	0	0	0	0	293	0	0	0
0	0	0	0	0	0	0	0	0	0	0	0	0	0	0	0	0	0
0	0	96	86	0	0	0	0	0	0	0	0	0	0	0	0	0	0
0	0	0	0	0	0	0	0	0	0	0	0	0	0	0	0	0	0
0	0	0	0	0	0	0	0	0	0	0	0	0	0	0	0	0	0
0	0	0	0	0	0	0	0	0	0	0	0	0	0	0	0	0	0
74	579	0	88	0	0	0	120	0	0	635	0	0	0	99	0	94	487
0	0	0	0	0	0	0	0	0	0	0	0	0	0	0	0	0	0

0	0	0	0	0	0	0	0	0	0	0	0	0	0	0	0	0	0
0	0	0	0	0	0	0	0	0	0	0	0	0	0	0	0	0	0
0	0	0	0	0	0	0	0	0	0	0	0	0	0	0	0	0	0
0	0	0	0	0	0	22659	0	0	0	0	70	329	0	0	0	98	99
0	0	0	0	0	0	0	0	0	0	0	0	0	0	0	0	0	0
0	0	0	0	0	0	0	0	0	0	0	0	0	0	0	0	0	0
0	0	0	0	0	0	0	0	0	0	0	0	0	0	0	0	0	0
0	0	0	0	0	0	0	0	0	0	0	0	0	0	0	0	0	0
0	0	0	0	0	0	0	0	0	0	0	0	0	0	0	0	0	0
0	110	0	0	67	0	0	0	0	860	211	19999	0	0	81	0	633	634
0	0	0	0	0	0	0	0	0	0	0	0	0	0	0	0	0	0
0	0	0	0	0	0	0	0	0	0	0	0	0	0	0	0	0	0
0	0	0	0	0	0	0	0	0	0	0	0	0	0	0	0	0	0
0	0	0	0	0	0	0	0	0	0	0	0	0	0	0	0	0	0
0	0	0	0	0	0	0	0	0	0	0	0	0	0	0	0	0	0
0	0	0	0	0	0	0	0	0	0	0	0	0	0	0	0	0	0
0	0	0	0	0	0	0	0	0	0	0	0	0	0	0	0	0	0
0	0	0	0	0	0	0	0	0	0	0	0	0	0	0	0	0	0
0	0	0	0	0	0	0	0	0	0	0	0	0	0	0	0	0	0
0	0	0	0	0	0	0	0	0	0	0	0	0	0	0	0	0	0
0	0	0	0	0	0	0	0	0	0	0	0	0	0	0	0	0	0
0	0	0	98	0	0	0	0	0	0	0	0	0	0	0	0	0	0
0	0	0	0	0	0	0	0	0	0	0	0	0	0	0	0	0	0
0	0	0	0	0	0	0	0	0	0	0	0	0	0	94	0	0	0
0	0	0	0	0	0	0	0	0	0	0	0	0	0	0	0	0	0
0	104	0	0	6646	0	6088	0	90	0	0	0	0	0	0	0	0	0
0	0	0	0	0	0	0	0	452	0	0	0	0	0	0	0	0	0
0	0	0	0	0	0	0	0	0	0	0	0	0	0	0	0	0	0
0	0	0	0	0	0	0	0	0	0	0	0	0	0	0	0	0	0
0	0	0	0	0	0	0	0	0	0	0	0	0	0	0	0	0	0
0	0	0	0	0	0	0	0	0	0	0	0	0	0	0	0	0	0
0	0	0	0	0	0	0	0	0	0	0	0	0	0	0	0	0	52
0	0	0	0	0	0	0	0	0	0	0	0	0	0	0	0	0	0
0	0	0	0	0	0	0	0	0	0	0	0	0	0	0	0	0	0
0	0	0	0	0	0	0	0	0	0	0	0	0	0	0	0	0	0
0	0	0	0	0	0	0	0	0	0	0	0	0	0	0	0	0	0
0	0	0	0	0	0	0	0	0	0	0	0	0	0	0	0	0	0
0	0	0	0	0	0	79	0	388	0	62	0	0	0	0	0	0	0
6105	9407	718	671	377	0	10914	1026	193	2272	8667	984	23144	2265	532	4667	1556	4934
0	0	0	0	0	0	0	0	0	0	0	0	0	0	0	0	0	0
277	0	0	0	0	0	0	0	0	0	0	0	0	0	711	0	0	0
0	0	0	0	0	0	0	0	0	0	0	0	0	0	0	0	0	0
0	0	0	0	0	0	0	0	0	0	0	0	0	0	0	0	0	0
0	0	0	0	3515	53	673	0	0	0	62	0	0	0	467	0	0	0
0	0	0	0	0	0	0	0	0	0	0	0	0	0	0	0	0	0
0	0	0	0	0	0	0	0	0	0	0	0	0	0	0	0	0	0
0	0	0	0	0	0	0	0	0	0	0	0	0	0	0	0	0	0
0	0	0	0	0	0	0	0	0	0	0	0	0	0	0	0	0	0
0	0	0	0	0	0	0	0	0	247	0	56	0	0	0	0	0	226
0	0	0	0	0	0	0	0	0	0	0	0	0	0	0	0	0	0
0	0	0	0	0	0	0	0	0	0	0	0	0	0	0	0	0	114
0	0	0	0	0	0	0	0	0	0	0	0	196	0	0	0	0	0
0	0	0	0	0	0	0	0	0	0	0	206	422	0	0	0	77	277
0	0	0	0	0	0	0	0	0	0	0	0	0	0	0	0	0	0
0	0	0	0	0	0	0	0	0	0	0	0	0	0	0	0	0	0
0	0	0	0	0	0	0	0	0	0	0	0	0	0	0	0	0	0
0	0	0	0	0	0	0	0	0	0	0	0	0	0	0	0	0	0
0	0	0	0	0	0	0	0	0	0	0	0	0	0	0	0	0	0
0	0	0	0	0	0	0	0	0	0	0	0	0	0	0	0	0	0
0	0	0	0	0	0	0	0	0	0	0	0	0	0	0	0	0	0
294	4928	13545	6338	1963	377	221	13170	92	3203	14086	66	8730	4216	8920	121319	1379	331
0	0	0	0	0	0	0	0	0	0	0	0	0	0	0	0	0	0
35746	676	109	161	585	0	163522	125	35275	539	0	0	0	0	653	248	80	0
0	0	0	0	0	0	0	0	0	0	0	0	0	0	0	0	0	0
0	0	0	0	0	0	0	0	0	0	0	0	0	0	0	0	0	0
0	0	0	0	0	0	0	0	0	0	0	0	0	0	0	0	0	0
0	0	0	0	0	0	0	0	0	0	0	0	0	0	0	0	0	0
0	1856	341	323	0	0	0	315	0	0	0	0	0	0	0	1375	0	0
0	0	0	0	0	0	0	0	0	0	0	0	0	0	0	0	0	0
0	0	0	0	0	0	0	0	0	0	0	0	0	0	0	0	0	0
0	0	0	0	0	0	0	0	0	0	0	0	0	0	0	115	0	0
0	0	0	0	0	0	0	0	0	0	0	0	0	0	0	0	0	0
0	0	0	0	0	0	0	0	0	0	0	0	0	0	0	0	0	0
0	0	0	0	0	0	0	0	0	0	0	0	0	0	0	0	0	0
0	0	0	0	0	0	0	0	0	0	0	0	0	0	0	0	0	0
0	0	0	0	0	0	0	0	0	0	0	0	0	0	0	0	0	0
0	0	0	0	0	0	0	0	0	0	0	0	0	0	0	0	0	0
0	0	0	0	0	0	0	0	0	0	0	0	0	0	0	0	0	0
0	0	0	0	0	0	0	0	0	0	0	0	0	0	0	0	0	0
0	0	0	0	0	0	0	0	0	0	0	0	0	0	0	0	0	0
0	0	0	0	0	0	0	0	0	0	0	0	0	0	0	0	0	0
0	0	0	0	0	0	774	0	0	0	0	0	0	0	0	0	0	0
1009	10424	402	183	2676	100	1223	2650	397	74004	59348	7703	45684	69171	70146	16299	28069	73484
0	0	0	0	0	0	0	0	0	0	0	0	1222	0	181	0	0	0
0	0	0	0	0	0	0	0	0	0	0	0	0	0	0	0	0	0
0	0	0	0	0	0	0	0	0	0	0	0	0	0	0	0	0	0
0	0	0	0	0	0	0	0	0	0	0	0	0	0	0	0	0	0

0	0	0	0	0	0	0	0	0	0	0	0	0	0	0	0	0	0
0	0	0	0	0	0	0	0	0	0	0	0	0	0	0	0	0	0
0	0	0	0	0	0	0	0	0	0	0	0	0	0	0	0	0	0
0	0	0	0	0	0	0	0	0	0	0	0	0	0	0	0	0	0
0	0	0	0	0	0	0	0	0	0	0	0	0	0	0	0	0	0
322	0	0	0	0	0	0	0	0	0	0	0	0	0	0	0	0	0
13010	0	0	0	0	0	0	0	0	0	0	0	0	0	110	0	0	0
0	0	0	0	0	0	0	0	0	0	0	0	0	0	0	0	0	0
0	0	0	0	0	0	767	0	701	0	0	0	0	0	0	0	0	0
0	0	0	0	0	0	0	0	0	571	0	0	0	0	0	0	194	57
0	0	0	0	0	0	0	0	0	0	0	0	0	0	0	0	0	0
0	0	0	0	0	0	0	0	0	0	0	0	0	0	0	0	0	0
0	0	0	0	0	0	0	0	0	0	0	0	0	0	0	0	0	0
0	0	0	0	0	0	12379	0	0	0	0	0	0	0	0	0	0	0
0	0	0	0	0	0	0	0	0	0	0	0	0	0	0	0	0	0
0	0	0	0	0	0	0	0	0	0	0	0	0	0	0	0	0	0
0	0	0	0	0	0	0	0	0	0	0	0	0	0	0	0	0	0
0	0	0	0	0	0	0	0	0	0	0	0	0	0	0	0	0	0
0	0	0	0	0	48	0	0	0	0	0	0	123	130	0	0	0	0
0	0	0	0	0	0	0	0	0	0	0	0	0	0	0	0	0	0
0	117	0	0	0	0	0	81	0	0	56	0	99	115	275	0	89	0
0	0	0	0	0	0	0	0	0	0	0	0	0	0	0	0	0	0
0	0	0	0	0	0	0	0	0	0	0	0	0	0	0	0	0	0
0	0	0	0	0	0	0	0	0	0	0	0	0	0	0	0	0	0
0	0	0	0	0	0	0	0	96	0	0	0	0	0	0	0	0	0
65756	0	0	0	0	0	0	0	0	0	0	0	0	0	0	0	0	0
0	0	0	0	0	0	0	0	0	0	0	0	0	0	0	0	0	0
0	0	0	0	0	0	0	0	0	0	0	0	0	0	0	0	0	0
0	0	0	0	0	0	14451	0	3874	0	0	0	0	0	0	0	0	0
0	0	0	0	0	0	0	0	0	0	0	0	0	0	0	0	0	0
0	0	0	0	0	0	0	0	0	0	0	0	0	0	0	0	0	0
0	0	0	0	0	63	0	0	0	0	0	0	0	0	100	53	0	0
0	0	0	0	0	0	0	0	0	0	0	0	0	0	0	0	0	0
0	0	0	0	0	0	0	0	0	0	0	0	0	0	0	0	0	0
0	0	0	0	0	0	0	0	0	0	0	0	0	0	0	0	0	0
0	0	0	0	0	0	0	0	0	0	0	0	0	0	0	0	0	0
0	0	0	0	0	0	0	0	0	0	66	0	826	349	0	0	94	225
0	0	0	0	0	0	0	0	0	0	0	0	0	0	0	0	0	0
0	0	0	0	0	0	0	0	0	0	0	0	0	120	113	0	181	127
0	0	0	0	0	0	0	0	0	0	0	0	0	0	0	0	0	0
0	0	0	0	0	0	0	0	0	0	0	0	0	0	0	0	0	0
0	0	0	0	0	0	0	0	0	0	0	0	0	0	0	0	0	857
0	0	0	0	0	0	0	0	0	0	0	0	0	0	0	0	0	0
0	0	0	0	0	0	0	0	0	0	0	0	0	0	0	0	0	0
0	0	0	0	0	0	0	0	0	0	0	0	0	0	0	0	0	0
0	0	0	0	0	0	0	0	0	0	0	0	0	0	0	0	0	0
0	0	0	0	0	0	0	0	0	0	0	0	0	0	0	0	0	0
0	0	0	0	0	0	0	0	0	0	0	0	0	0	0	0	0	0
0	0	0	0	0	0	0	0	0	0	0	0	0	0	0	0	0	0
0	0	0	93	0	0	80	0	0	0	0	0	0	0	0	0	0	0
0	0	0	0	0	0	0	0	0	0	0	0	0	0	0	0	0	0
0	0	0	0	0	0	0	0	0	0	0	0	0	0	0	0	0	0
0	0	0	0	0	0	0	0	0	0	0	0	0	0	0	0	0	0
0	0	0	0	0	0	0	0	0	0	0	0	0	0	0	0	0	0
0	0	0	0	0	0	0	0	0	0	0	0	0	0	0	0	0	0
32824	0	0	0	0	0	0	70	0	0	0	0	0	0	0	0	0	0
0	0	0	0	0	0	0	0	0	0	0	0	0	0	0	0	0	0
0	0	0	0	0	0	0	0	0	0	0	0	0	0	0	0	0	0
0	0	0	0	0	0	0	0	0	0	0	0	0	0	0	0	0	0
0	0	0	0	0	0	0	0	0	0	0	0	0	0	0	0	0	0
0	0	0	0	0	0	0	0	0	0	0	0	0	0	0	0	0	0
0	0	0	0	0	0	0	0	0	0	0	0	0	0	0	0	0	0
0	0	0	0	0	0	0	0	0	0	0	0	222	0	0	0	0	0
0	0	0	0	0	0	0	0	0	0	0	0	0	0	0	0	0	0
0	0	0	0	0	0	0	0	0	0	0	0	0	0	0	0	0	0
62	0	0	0	0	0	0	0	0	0	0	0	0	0	0	0	0	0
0	0	0	0	0	0	0	0	0	0	0	0	0	0	0	0	0	0
0	0	0	0	0	0	0	0	0	0	0	0	0	0	0	0	0	0
0	0	0	0	0	0	0	0	0	0	0	0	0	0	0	0	0	0
295	0	0	0	0	0	0	0	0	0	0	0	0	0	397	0	0	0
0	0	0	0	0	0	0	0	0	0	0	0	0	0	0	0	0	0
0	0	0	0	0	0	0	0	0	0	0	0	0	0	0	0	0	0
0	0	0	0	0	0	0	0	0	0	0	0	0	0	0	0	0	0
0	0	0	0	0	0	0	0	0	0	0	0	0	0	0	0	0	0
0	0	0	0	0	0	0	0	0	0	0	0	0	0	0	0	0	0
0	0	0	0	0	0	0	0	0	0	0	0	0	0	0	0	0	0
233	558	0	0	1853	0	203	266	0	5404	1834	69	1483	3733	3563	2677	754	895
0	0	0	0	0	0	0	0	0	0	0	0	0	0	0	0	0	0
0	0	0	0	0	0	0	0	0	0	0	0	0	0	0	0	0	0
0	0	0	0	0	0	0	0	0	0	0	0	0	0	0	0	0	0
0	0	0	0	0	0	0	0	0	0	0	79	0	0	0	0	0	0
0	0	0	0	55	0	0	0	0	0	0	0	0	0	0	0	0	0
0	0	0	0	0	0	0	0	0	0	0	0	0	0	0	0	0	0
0	0	0	0	0	0	0	0	0	0	0	0	0	0	0	0	0	0
0	0	0	0	0	52	0	0	0	0	0	0	0	0	0	0	0	0
0	0	0	0	5301	0	19397	0	97	0	0	0	218	262	108	0	0	0
0	0	0	0	0	0	0	0	0	0	0	0	0	0	0	0	0	0

0	0	0	0	0	0	0	0	91	285	0	426	0	0	0	0	0	0
0	0	0	0	0	67	0	0	0	0	0	0	0	0	0	0	0	0
0	0	0	0	0	0	0	0	0	0	0	0	0	0	0	0	0	0
0	0	0	0	0	0	0	0	0	0	0	0	0	0	0	0	0	0
0	0	0	0	0	0	0	0	0	0	0	0	0	0	0	0	0	0
74	0	0	0	0	0	0	0	0	0	0	0	0	0	0	0	0	0
0	0	0	0	0	0	0	0	0	0	0	0	0	0	0	0	0	0
0	0	0	0	0	0	0	0	0	0	0	0	0	0	0	0	0	0
0	0	0	0	0	0	0	0	0	0	0	0	0	0	0	0	0	0
0	0	0	0	0	0	0	0	0	0	0	0	0	0	0	0	0	0
0	0	0	0	0	0	0	0	0	0	0	0	0	0	0	0	0	0
0	0	0	0	0	0	0	0	0	0	0	0	0	0	0	0	0	0
0	0	0	0	0	0	0	0	0	0	0	0	0	0	0	0	0	0
0	0	0	0	83	0	2617	0	0	0	0	0	0	0	0	0	0	0
0	0	0	0	927	0	0	0	79	0	0	0	0	0	0	0	0	0
68	0	0	0	0	0	0	0	0	0	0	0	0	0	0	0	0	0
0	0	0	0	0	0	0	0	0	268	0	0	0	0	0	0	93	0
0	0	0	110	0	0	0	0	0	0	0	0	1585	0	0	0	0	0
0	0	0	0	0	0	0	0	0	0	0	0	0	0	0	0	0	0
0	0	0	0	0	0	0	0	0	0	0	0	0	0	0	0	0	0
0	0	0	0	0	0	0	0	0	0	0	0	0	0	0	0	0	0
0	0	0	0	0	0	0	0	0	0	0	0	0	0	0	0	0	0
0	0	0	0	0	0	0	0	0	0	0	0	0	0	0	0	0	0
0	0	0	0	0	0	0	0	0	0	0	0	0	0	0	0	0	0
0	0	0	0	0	0	0	0	0	0	0	0	0	0	0	0	0	0
0	0	0	0	0	0	0	0	0	0	0	0	0	0	0	0	0	0
0	0	0	0	0	0	0	0	0	0	542	0	0	0	0	0	0	0
0	0	0	0	0	0	0	0	0	0	0	0	4051	0	0	0	0	0
0	0	0	0	0	0	0	0	0	0	0	0	0	0	0	0	0	0
0	0	0	0	0	0	0	0	0	0	0	0	4299	0	0	0	0	0
0	0	0	0	0	0	0	0	0	0	0	0	0	0	0	0	0	0
61	0	0	0	0	0	0	0	0	861	209	0	0	580	627	293	88	0
75846	5016	6747	22088	351293	952781	39890	5318	3580	8465	20545	33732	81394	7006	5937	82028	284169	9530
0	0	0	0	0	0	0	0	0	0	0	0	0	0	0	0	0	0
0	0	0	0	0	0	79	0	0	582	79	0	0	0	0	0	0	53
0	0	0	0	0	0	0	0	0	0	0	0	0	0	0	0	0	0
0	0	0	0	0	0	0	0	0	0	0	0	0	0	0	0	0	47
0	0	0	0	0	0	0	0	0	0	0	0	0	0	0	0	0	0
0	0	0	0	0	0	0	0	0	0	0	0	0	0	0	0	0	0
0	0	0	0	0	0	0	0	0	0	0	0	0	0	0	0	0	0
0	0	0	0	955	0	0	0	0	0	0	0	97	130	93	602	0	0
0	0	0	0	0	0	0	0	0	0	0	0	0	0	0	0	0	0
0	0	0	0	0	0	0	0	0	0	0	0	0	0	0	0	0	0
0	0	0	0	0	0	0	0	81	0	0	0	1978	0	0	0	0	0
43822	124	0	0	0	0	0	62	0	0	0	0	0	0	0	0	0	0
0	0	0	0	0	0	0	0	0	0	0	0	0	0	0	0	0	0
0	0	0	0	0	0	0	0	0	0	0	0	0	0	0	0	0	0
0	0	0	0	0	0	0	0	76	0	0	0	0	0	0	0	0	0
782	27676	126	0	206	152	1065	6895	1370	32647	26932	352	27678	37284	31200	15963	19369	7168

COVID -19-005	COVID -19-004	COVID -19-002	COVID -19-001	COVID -19-035	COVID -19-007	COVID -19-016	COVID -19-003	COVID -19-018	COVID -19-017	COVID -19-032	COVID -19-030	COVID -19-029	COVID -19-028	COVID -19-027	COVID -19-026	COVID -19-031	COVID -19-024
921313	966407	943346	894399	967932	935781	883725	932782	934013	847997	747513	827712	859083	990331	938360	994237	736860	794945
39887	14621	24657	86800	25504	41275	23511	25291	37627	84955	181031	156340	133339	8160	44878	4378	257568	203060
0	0	0	0	0	0	0	0	206	0	0	0	0	90	83	0	0	233
34535	10142	31447	16336	6472	21885	47573	41427	26368	29807	37421	15756	7419	1146	15463	1265	4580	1547
4265	8830	550	2465	92	1059	45191	500	1716	37241	34035	192	159	273	902	120	992	215
0	0	0	0	0	0	0	0	0	0	0	0	0	0	0	0	0	0
0	0	0	0	0	0	0	0	70	0	0	0	0	0	314	0	0	0
COVID -19-005	COVID -19-004	COVID -19-002	COVID -19-001	COVID -19-035	COVID -19-007	COVID -19-016	COVID -19-003	COVID -19-018	COVID -19-017	COVID -19-032	COVID -19-030	COVID -19-029	COVID -19-028	COVID -19-027	COVID -19-026	COVID -19-031	COVID -19-024
63	0	0	0	0	62	0	151	132	95	0	17824	0	0	0	108	0	56
0	0	0	0	0	0	0	0	0	0	0	0	0	0	0	0	0	0
754390	4672	58777	35077	117291	793879	734431	641574	791166	742299	181224	29002	14953	10576	367229	37473	46205	44489
0	0	0	0	3943	0	0	0	0	0	0	0	0	0	0	0	0	0
0	0	124	58	0	0	0	0	0	0	0	0	0	0	0	0	0	0
0	0	0	0	0	0	0	0	0	0	0	0	0	0	0	0	0	0
0	0	0	0	0	0	0	0	0	0	8308	0	0	0	0	0	100	0
0	0	0	0	0	28	55	0	0	46	0	0	0	0	0	0	0	0
0	0	0	0	0	38	0	0	0	0	0	0	0	0	0	0	0	0
0	0	0	0	0	0	0	0	0	0	0	0	0	0	0	0	0	0
0	0	0	0	0	0	0	0	0	0	0	0	0	0	0	0	0	0
0	46	450	10442	82	146	104	142	0	379	13237	81	83	3413	0	139	17190	2391
0	0	0	0	0	35	0	0	0	0	0	0	0	0	0	0	0	0
0	0	0	0	0	0	0	0	0	0	0	0	0	0	0	0	0	0
0	0	0	0	0	0	0	0	0	0	0	0	0	0	0	0	0	0
18344	252	310	316	1738	16697	12596	22659	18728	14120	3251	811	211	94	7746	742	706	393
0	0	0	0	0	0	0	0	0	0	0	0	0	0	0	0	0	0
0	0	0	0	0	0	0	0	0	0	0	0	0	0	0	0	0	0
0	58	0	0	0	0	0	0	0	0	0	0	0	0	0	0	0	0
0	0	139	54	0	0	0	0	0	0	0	0	302	0	1580	367	0	0
0	0	0	0	0	0	0	0	0	0	0	0	0	0	0	0	0	0
0	0	0	0	0	0	0	0	0	0	0	0	0	0	0	0	0	0
0	0	0	0	0	0	0	0	0	0	0	0	0	0	0	0	0	0
0	0	0	0	0	0	0	0	0	0	0	0	0	0	0	0	0	0
98	0	135	331	0	179	146	0	237	82	0	210	0	86	0	0	196	0
0	0	0	0	0	0	0	0	0	0	0	0	0	0	0	0	0	0
0	0	0	2041	3404	0	0	0	0	0	3678	7030	9128	973	1851	10300	382	3314
0	0	0	0	0	0	0	0	0	0	0	0	0	0	0	0	0	0
0	0	0	0	0	0	0	0	0	0	0	0	0	0	0	0	0	0
32944	859	170952	372747	65163	11922	6121	8367	15845	26433	26643	74216	60680	24394	37445	5576	67891	21409
0	0	0	0	0	0	0	0	0	0	0	0	0	0	0	0	0	0
0	0	0	0	0	0	6643	749	129	53	0	0	71	0	0	1837	5503	53
0	937	0	0	0	0	0	0	0	0	0	0	0	0	2436	0	0	0
0	0	0	0	0	0	0	0	0	0	0	0	0	0	0	0	0	0
0	0	0	0	0	0	0	0	0	0	0	0	0	0	0	0	0	0
0	0	0	0	0	34	2036	0	127	0	0	0	0	0	0	0	0	0
0	0	0	66	0	0	0	0	0	0	0	0	0	0	0	0	0	0
0	0	0	0	0	0	0	0	0	0	0	0	0	0	0	0	0	0
0	0	0	0	0	0	0	0	0	0	0	0	0	0	0	0	0	0
0	0	0	0	0	0	0	0	0	0	0	0	0	0	0	0	0	0
0	0	0	0	0	256	0	629	0	91	221	47	0	0	77	0	1925	217
0	0	0	0	0	0	0	0	0	0	0	0	0	0	0	0	0	0
0	0	0	0	0	0	0	0	0	0	0	0	0	0	0	0	0	0
0	0	0	0	0	0	0	0	0	0	0	0	0	0	0	0	0	0
0	0	0	0	0	0	0	0	0	0	0	0	0	0	0	0	0	0
0	665	0	0	0	0	0	0	0	0	0	9580	0	966	0	0	0	1850
0	0	0	0	0	0	0	0	0	0	0	0	0	0	0	0	0	0
1875	1005	752	269037	57885	152	108	969	359	639	3274	1837	59401	19970	23673	3065	261862	969
0	0	0	0	0	0	0	0	0	0	0	0	0	0	0	0	0	0
0	0	0	0	70	0	0	0	0	0	0	0	78	0	0	0	1677	0
0	0	61	43427	0	0	63	0	0	0	0	0	0	0	0	0	0	0
0	0	0	0	0	0	0	0	0	0	0	0	0	0	0	0	0	0
0	0	0	0	0	0	0	0	67	0	0	0	0	0	0	0	0	0
0	0	0	0	0	0	0	0	0	0	0	0	0	0	0	0	0	0
0	0	0	0	0	0	0	0	0	0	0	0	0	0	0	0	569	0
0	0	0	0	0	0	0	0	0	0	0	0	0	0	0	0	0	0
0	0	0	0	0	0	0	0	0	0	0	0	0	0	0	0	0	0
835	0	0	0	0	1436	1427	603	1062	0	0	0	0	0	0	0	101	0
0	0	0	0	0	0	0	0	0	0	0	0	0	0	0	0	0	0
0	0	0	0	0	0	0	0	0	0	0	0	0	0	0	0	0	0
2220	943791	616	410	380	55	241	25712	132	233	14606	656	30967	466	16639	5953	29728	30143
0	0	61	0	0	0	0	0	0	0	0	0	0	0	0	0	0	0
0	0	0	0	0	0	0	0	0	0	0	0	0	0	0	0	0	0
0	0	0	0	0	0	0	0	0	0	0	0	0	0	0	0	0	0
0	0	0	0	0	0	0	0	0	0	0	0	0	0	0	0	0	0
0	0	0	0	0	0	0	0	0	0	0	0	0	0	0	0	0	0
0	0	0	0	0	0	0	0	0	0	0	0	0	0	2203	0	0	0
0	0	0	0	0	0	0	0	0	0	0	0	0	0	0	0	0	0
0	0	0	0	0	0	0	0	0	0	0	0	0	0	0	0	0	0

0	0	0	0	0	0	77	0	0	0	0	0	0	0	0	0	0	0
0	0	0	0	0	0	0	0	0	0	0	0	0	0	0	0	0	0
0	0	0	0	0	0	0	0	0	0	0	0	0	0	0	0	0	0
0	0	0	0	0	0	0	0	0	0	0	0	0	0	0	0	0	0
0	0	0	0	0	0	0	0	0	0	0	0	0	0	0	0	0	0
0	0	0	0	0	0	0	0	0	0	0	0	0	0	0	0	0	0
83	0	70	0	239340	0	0	310	0	0	308275	563653	574815	45036	226783	862351	48867	324164
0	0	0	0	0	0	0	0	0	0	0	0	0	0	0	0	0	0
0	0	0	0	0	0	0	0	0	0	0	0	0	90	83	0	0	233
0	0	0	0	0	0	0	0	0	0	0	0	0	0	0	0	0	0
0	0	0	0	0	0	0	0	0	0	0	0	0	0	0	0	0	0
0	0	0	0	0	0	0	0	0	0	0	0	0	0	0	0	0	0
319	7819	422	56687	45813	831	161	1882	343	250	16564	330	3769	7288	15195	1596	55393	27614
0	0	0	0	0	0	0	0	0	0	0	0	0	0	0	0	0	0
0	0	0	0	0	0	0	0	0	0	0	0	0	429	0	0	0	1173
0	0	0	0	0	0	0	0	0	0	0	0	0	0	0	0	0	0
0	0	0	0	0	0	0	0	0	0	0	0	0	0	0	0	0	0
0	0	0	0	0	0	0	0	0	0	0	0	0	0	0	0	0	0
0	0	0	0	0	0	0	0	0	0	0	0	0	0	0	0	0	0
0	65	0	0	0	0	0	0	0	0	0	0	0	0	0	0	0	0
0	0	0	0	0	0	0	0	0	0	0	0	0	0	0	0	6384	0
0	0	0	0	0	0	0	0	0	0	0	0	0	0	0	0	0	0
0	0	0	0	0	0	0	0	0	0	0	0	0	0	70	0	0	0
0	0	68	0	0	0	0	0	0	0	0	0	0	0	0	0	0	0
89	0	0	0	0	0	0	291	78	44	68	0	0	0	0	146	0	0
0	0	0	0	0	0	0	0	0	0	0	0	0	0	0	0	0	0
0	0	0	0	0	0	0	0	0	0	0	0	0	0	0	0	0	0
0	0	852	326	74	0	0	0	0	0	0	91	0	0	0	0	0	0
0	0	0	0	0	0	0	0	0	0	0	0	0	0	0	0	0	0
0	0	0	0	0	0	0	0	0	0	0	0	0	0	0	0	0	0
62	0	0	0	4993	0	0	306	0	0	61	0	0	0	0	1327	0	0
0	0	0	0	0	0	0	0	0	0	0	0	0	0	0	0	0	0
0	0	0	0	0	0	0	0	0	0	0	0	0	0	0	0	0	0
0	0	0	0	0	0	0	0	0	0	0	0	0	0	0	0	0	0
0	0	0	0	0	0	0	0	0	0	0	0	0	0	0	0	105	0
0	0	0	0	0	0	0	0	0	0	0	0	0	0	0	0	0	0
0	0	0	0	0	0	0	0	0	0	0	0	0	0	0	0	0	0
0	0	0	0	0	0	0	0	0	0	289	0	0	0	0	0	0	0
0	0	0	118	0	0	0	0	0	0	0	0	0	0	0	0	0	0
0	0	0	0	0	0	0	0	0	0	69	0	0	0	0	0	0	0
0	0	0	0	0	0	0	0	0	0	0	0	0	0	0	0	0	0
0	0	0	0	38188	0	0	0	0	0	66	255	0	94	0	0	0	0
0	0	0	0	0	0	0	0	0	0	0	0	0	0	0	0	0	0
0	0	0	0	0	0	0	0	0	0	0	0	0	0	0	0	0	0
0	0	0	0	0	0	0	0	0	0	0	0	0	0	0	0	0	0
0	0	0	0	14422	41	0	0	0	0	64	50	0	0	0	0	95	108
0	0	0	0	0	0	0	0	0	0	0	0	0	0	0	0	0	0
0	0	71	0	0	0	0	0	0	0	0	0	0	0	0	0	0	0
0	0	870	466	0	0	0	0	0	0	0	0	0	0	0	0	944	0
0	0	0	56	0	0	0	0	0	0	71	20030	85	0	0	0	88	61
4265	8830	116	2283	0	1059	45191	336	1716	37241	0	150	78	273	902	0	889	215
0	0	0	0	0	0	0	0	0	0	0	0	0	0	0	0	0	0
0	0	0	0	0	0	0	0	0	0	138	0	0	0	0	0	0	0
0	0	0	0	0	0	0	0	0	0	0	0	0	0	0	0	0	0
0	0	0	0	0	0	0	0	0	0	0	0	0	0	0	0	0	0
0	0	0	0	0	0	0	0	0	0	0	0	0	0	0	0	0	0
80	0	0	0	0	0	0	0	119	0	0	0	0	0	0	0	0	0
0	0	0	0	0	0	0	0	0	0	0	0	0	0	0	0	0	0
0	0	0	0	0	0	0	0	0	0	0	0	0	0	0	0	0	0
0	0	0	0	0	0	0	0	0	0	0	0	0	0	0	0	0	0
0	190	0	0	0	0	0	0	0	43	0	743	0	0	82	133	0	391
0	0	0	0	0	0	0	0	0	0	0	0	0	0	0	0	0	0
12680	0	0	67	0	2534	240	632	5286	10096	0	0	0	115	520	0	0	51
0	0	0	0	0	0	0	0	0	0	0	0	0	0	0	0	0	0
0	68	0	0	0	0	0	144	0	0	0	0	0	0	0	0	788	0
0	0	0	0	0	0	0	0	0	0	0	0	0	0	0	0	0	0
0	0	0	0	0	0	0	0	0	0	0	0	0	0	0	0	0	0
0	0	0	0	0	0	0	0	0	0	0	0	0	0	0	0	0	0
0	0	0	0	0	0	0	0	0	0	0	0	0	0	0	0	0	0
0	0	0	0	0	0	0	0	0	0	0	0	0	0	0	0	0	0
0	0	0	0	0	0	0	0	0	0	0	0	0	0	0	0	0	0
0	0	0	0	0	0	0	0	0	0	0	0	0	0	0	0	0	0
0	0	0	0	0	0	0	0	0	0	0	0	0	0	0	0	0	0
0	0	0	0	0	0	1139	0	0	0	0	0	0	0	0	0	0	0
6273	59	542	62	9969	7638	14081	13499	9622	1780	6543	53	168	297	3129	373	308	222
26654	60	203	59	974	34847	15789	21000	31606	7173	3215	41	78	276	18113	144	2189	1167
77	0	0	0	162	0	119	160	0	0	12074	143	102377	265	5516	239	1002	22612
0	0	0	0	0	0	0	0	0	0	0	0	0	0	0	0	0	0
0	0	0	0	0	0	0	0	0	0	0	0	0	0	0	0	0	0
0	0	0	0	0	0	0	0	0	0	0	0	0	0	478	0	0	0
0	0	275	64	0	0	0	0	0	0	0	0	0	0	0	0	0	0
0	0	0	0	0	0	0	0	0	0	0	0	0	0	0	0	0	0
0	0	0	0	0	0	0	0	0	0	0	0	0	0	169	0	0	0
21855	10142	31447	16269	6472	19351	47333	40795	21082	19711	37421	15756	7419	1031	14943	1265	4580	1496
0	0	0	0	2709	157	0	952	0	0	8154	294	172	41053	0	10702	10847	42323
0	0	0	1225	225	0	0	0	0	0	0	0	252	0	0	0	0	0
0	0	0	0	0	0	0	0	0	0	0	0	0	0	0	0	0	0

0	0	0	0	0	0	0	0	0	0	0	0	0	0	0	0	0	0
0	0	1645	1045	0	109	73	0	146	1053	0	0	0	0	0	0	0	0
0	0	0	0	0	0	0	0	0	0	0	0	0	0	0	0	0	0
0	64	0	0	0	0	564	0	0	0	0	0	0	0	0	0	0	0
0	0	0	0	0	0	0	0	0	0	0	0	0	0	0	0	0	0
0	0	0	0	0	0	0	0	0	0	0	0	0	0	0	0	0	0
0	0	0	0	0	0	0	0	0	0	0	0	0	0	0	0	0	0
76	0	0	0	132401	34	0	166	0	0	488	53839	15313	202082	31104	33594	4179	174284
0	0	0	0	0	46	0	451	63	0	0	0	0	0	0	0	0	59
0	0	0	0	0	0	0	0	0	0	0	0	0	0	0	0	0	0
0	0	0	0	0	0	0	0	0	0	0	0	0	0	0	0	0	0
0	0	0	0	92	0	0	0	0	0	34035	42	81	0	0	120	103	0
0	0	0	0	0	0	0	0	0	0	17991	42	71	0	0	130	0	0
0	0	0	0	0	0	0	0	0	0	0	0	0	0	0	0	0	0
0	0	0	0	0	0	0	0	0	0	0	0	0	0	0	0	0	0
0	0	0	0	0	0	0	0	0	0	0	0	0	0	0	0	0	0
0	0	0	0	0	0	0	0	0	0	0	0	0	0	0	0	0	0
0	0	0	0	0	0	0	0	0	0	0	0	0	0	0	0	0	0
0	0	0	0	0	0	0	0	0	0	0	0	0	0	0	0	0	0
0	0	0	0	0	0	0	0	0	0	0	0	0	0	0	0	0	0
0	0	60	0	238	0	0	0	0	0	129078	399	295	189	7894	384	391	227
0	0	0	0	0	0	0	0	0	0	0	2674	0	0	0	251	411	0
0	0	0	0	0	0	0	0	0	0	0	0	0	0	0	0	0	0
672	62	0	0	0	374	1860	778	338	1453	0	0	0	0	0	0	0	0
0	0	0	0	0	0	0	0	0	0	0	0	0	0	0	0	0	0
0	0	0	0	0	0	0	0	0	0	0	0	0	0	0	0	0	0
0	0	0	0	230	0	0	0	0	0	136	76715	159	291	0	118	203	51
0	0	0	0	0	0	0	0	0	0	0	0	0	0	0	0	0	0
0	0	0	0	0	0	0	0	0	0	0	0	0	0	0	0	0	0
0	0	0	0	0	0	0	0	0	0	0	0	0	0	0	0	0	0
0	0	0	0	0	0	0	0	0	0	0	0	0	0	0	0	0	0
0	0	0	0	0	0	0	0	0	0	0	0	0	0	0	0	0	0
0	0	0	0	0	34	101	0	0	107	0	0	0	0	0	0	0	0
0	0	0	0	0	0	0	0	0	0	0	0	0	0	0	0	0	0
0	0	0	0	0	0	0	0	0	0	0	0	0	0	0	0	0	0
0	0	0	0	308	184	2731	0	258	184	0	181	73	3981	21656	1409	181	63779
0	0	0	0	0	0	0	0	0	0	0	0	0	0	0	0	0	0
0	0	0	0	0	0	0	317	0	0	0	0	0	0	0	0	0	0
0	0	0	0	0	0	0	0	0	0	0	0	0	0	0	0	0	0
0	0	0	0	0	0	0	0	0	0	0	0	0	0	0	0	0	0
0	0	0	0	0	0	0	0	0	0	0	0	0	0	0	0	0	0
0	0	0	0	0	0	0	0	0	0	0	0	0	0	0	0	0	0
0	0	0	0	0	0	0	0	0	0	0	0	0	0	0	0	0	0
0	71	0	0	0	0	0	0	0	0	0	0	0	0	0	0	0	0
0	0	0	0	0	0	0	0	0	0	76	53	0	101	0	101	0	12436
83	9220	0	0	0	0	0	0	0	0	0	0	0	0	0	0	0	0
0	0	0	0	0	0	0	0	0	0	0	0	0	0	0	0	0	0
0	0	0	0	0	0	0	0	0	0	0	0	0	0	0	0	0	0
567	0	0	0	85	561	107	458	335	205	0	0	72	0	0	0	14087	0
0	0	0	0	0	0	0	0	0	0	0	0	0	0	0	0	0	0
0	0	0	0	0	0	0	0	0	0	0	0	0	0	0	0	0	0
0	0	0	0	0	0	0	0	0	0	0	0	0	0	0	0	0	0
0	0	0	0	0	0	0	0	0	0	0	0	0	0	0	0	0	0
0	0	0	0	0	0	0	0	0	0	0	0	0	0	0	0	0	0
0	0	0	0	0	0	0	0	0	0	0	0	0	0	0	0	0	0
0	0	0	0	0	0	0	0	0	0	0	0	0	0	0	0	0	0
0	0	0	0	0	0	0	0	0	0	0	0	0	0	0	0	0	0
0	0	0	0	0	0	0	0	0	0	0	0	0	0	0	140	0	0
0	0	0	0	0	0	0	0	0	0	0	0	0	0	0	0	0	0
0	0	0	0	0	0	0	0	0	0	0	0	0	0	0	0	0	0
0	0	0	0	0	0	0	0	0	0	0	0	0	0	0	0	0	0
0	0	0	0	0	0	0	0	0	0	0	0	0	0	0	0	0	0
0	0	0	0	0	0	0	0	0	0	0	0	0	0	0	0	0	0
0	0	0	0	0	0	0	0	0	0	0	0	0	0	0	0	0	0
0	0	0	0	0	0	0	0	0	0	0	0	0	0	0	0	1770	0
0	0	0	0	0	0	0	0	0	0	0	104	0	3287	78	120	0	4303
0	0	0	0	0	0	0	0	0	0	0	0	0	0	0	0	0	0
0	0	0	0	0	0	0	0	0	0	0	0	0	0	232	0	0	0
0	0	0	0	0	0	0	0	0	0	0	0	0	0	0	0	0	0
0	0	0	0	0	0	0	0	0	0	0	0	0	0	0	0	0	0
0	0	0	0	0	0	0	0	0	0	0	0	0	0	0	0	0	0
0	0	0	0	0	0	0	0	0	0	0	0	0	0	0	0	0	0
0	0	0	0	0	0	0	0	0	0	0	0	0	0	0	0	0	0
319	1183	14522	110	678	1011	1034	306	597	1992	17427	44533	10277	518	4846	1189	249461	152369
0	0	0	0	0	0	0	0	0	0	0	0	0	0	0	0	0	0
0	0	0	0	0	0	0	0	0	0	0	0	0	0	0	0	0	0
0	0	0	0	0	0	0	0	0	0	0	0	78	0	0	331	0	0
0	0	0	0	0	0	0	0	0	0	0	0	0	0	0	0	0	0
872	0	0	0	1244	1105	247	933	1948	92	66	62	0	91	78	709	0	225
0	0	0	0	0	0	0	0	0	0	0	2171	0	0	0	0	0	0
0	0	0	0	0	0	0	0	0	0	0	0	0	0	0	0	0	0
0	0	0	0	0	0	0	0	0	0	0	0	0	0	0	0	0	0
0	0	63	0	0	0	0	0	0	0	0	0	0	0	0	0	0	0
0	0	310	124	0	0	0	0	0	0	0	0	0	0	0	0	0	0
0	0	0	0	0	0	0	0	0	0	0	0	0	0	0	0	0	0
0	0	0	0	0	0	0	0	0	0	0	0	0	3330	4001	626	532	0
0	0	0	0	0	0	0	0	0	0	0	0	0	0	0	0	0	0

0	0	0	0	0	33	67	0	146	90	0	0	0	259	0	0	0	0
0	0	0	0	0	0	0	0	0	0	0	0	0	0	0	0	0	0
0	0	0	0	0	0	0	0	0	0	0	0	0	0	0	0	0	0
0	0	0	0	0	0	0	0	0	0	0	0	0	0	0	0	0	0
0	0	0	0	0	0	0	0	0	0	0	0	0	0	0	0	0	0
0	0	0	0	0	0	0	0	0	0	0	0	0	0	0	0	0	0
0	0	0	0	0	0	0	0	0	0	0	0	0	0	0	0	0	0
0	0	0	0	0	0	0	0	0	0	0	0	0	0	3354	0	0	0
0	0	0	0	0	0	0	0	0	0	0	0	9227	0	0	0	0	0
0	0	52	0	0	0	0	0	0	0	0	0	0	0	0	0	0	0
0	0	0	0	0	0	0	0	0	0	0	0	0	0	0	0	0	0
0	0	0	0	0	0	0	0	0	0	0	0	0	0	0	0	0	0
1166	883	0	0	0	0	0	2833	0	0	0	98	0	457	0	257	22077	0
0	0	0	0	0	0	0	172	0	0	0	0	0	0	0	0	0	0
0	0	0	0	0	0	0	0	0	0	0	0	0	0	0	0	0	0
88	517	0	0	80	78	171	336	335	75	0	0	0	102	0	0	0	13159
0	0	0	0	0	0	0	0	0	0	0	0	0	0	0	0	0	0
0	0	0	13215	0	0	0	0	0	0	0	0	0	0	0	0	0	0
0	0	0	0	0	0	0	0	0	0	0	0	0	0	0	0	0	0
0	0	0	0	0	0	0	0	0	0	0	0	0	0	0	0	0	0
0	0	0	0	0	0	0	0	0	0	0	0	0	0	0	0	0	0
0	0	0	0	0	0	0	0	0	0	0	0	87	99	0	0	0	4382
523	0	62	0	78	501	80	477	323	300	0	0	0	0	0	0	0	100
0	0	0	0	0	0	0	0	0	0	0	0	0	0	0	0	0	0
0	0	0	0	0	0	0	0	0	0	0	0	0	0	0	0	0	0
135	0	0	0	0	0	0	310	0	0	0	0	0	0	0	0	0	0
0	0	0	0	0	0	0	0	0	0	0	0	0	0	0	0	0	0
10681	113	2699	130	1468	10429	9213	6672	13320	4955	2128	433	79	0	5079	755	104	522
0	0	0	0	0	0	0	0	0	0	0	0	0	0	0	0	0	0
0	0	0	0	0	0	0	0	0	0	0	0	0	0	0	0	0	0
0	0	0	0	0	0	0	0	0	0	0	0	0	0	0	0	0	0
0	0	0	0	0	0	0	0	0	0	0	0	0	0	0	0	0	0
0	0	0	0	0	0	0	0	0	0	0	0	0	0	0	0	0	0
0	0	0	0	0	0	0	0	0	0	0	0	0	0	0	0	0	0
74	0	147	1951	83	474	0	168	216	139	0	0	0	180	581	0	783	0
0	0	0	0	0	0	0	0	0	0	0	0	0	0	0	0	0	0
0	0	0	0	0	0	0	0	0	0	0	0	0	0	0	0	0	0
77	0	0	0	0	208	21	166	62	0	0	0	0	0	0	0	0	0
0	0	77	2903	404	0	0	0	0	0	46865	135	69	451	0	124	0	3775
0	0	0	0	0	0	0	0	0	0	0	0	0	0	0	0	0	0
0	0	0	0	0	0	0	0	0	0	0	0	0	5387	0	0	0	0
0	0	0	0	0	0	0	0	0	0	0	0	0	0	0	0	0	0
0	56	67	0	0	628	83	303	57	0	0	0	0	0	0	0	0	0
0	0	0	0	0	0	0	0	0	0	0	0	0	0	0	0	0	0
0	0	0	0	0	0	0	0	0	0	0	0	0	0	0	0	0	0
0	0	0	0	0	0	0	0	0	0	0	0	0	1758	0	0	1294	0
0	0	0	0	0	0	0	0	0	0	0	0	0	0	0	0	0	0
0	0	0	0	0	0	0	0	0	0	0	0	0	0	0	0	0	0
0	0	0	0	0	0	0	0	0	0	0	0	0	0	0	0	0	0
0	0	0	0	0	0	0	0	0	0	0	0	0	0	0	0	0	0
0	0	57	123	0	0	30	0	0	0	0	46	0	0	77	0	0	0
0	0	0	0	0	0	0	0	0	0	0	53	0	0	0	0	0	0
0	0	0	0	0	0	0	0	0	0	0	0	0	0	0	0	0	0
0	0	0	0	0	79	0	0	0	0	0	0	0	0	0	0	0	0
0	0	0	0	0	0	0	0	0	0	0	0	0	0	0	0	0	0
0	0	0	0	0	0	0	0	0	0	0	0	0	0	0	0	0	0
0	0	0	0	0	0	0	0	0	0	0	0	0	0	0	0	0	0
75	0	0	0	0	122	0	325	124	0	0	0	0	0	0	0	0	0
0	0	0	0	0	0	0	0	0	0	0	0	0	0	0	0	0	0
0	0	0	0	0	0	0	0	0	0	0	0	0	0	0	0	0	0
0	0	61	0	0	0	0	0	0	0	80	57	230	365	9895	237	75455	2924
0	0	0	0	2161	0	828	0	0	0	0	0	0	0	0	0	101	0
0	0	0	0	0	0	2683	0	0	56	0	0	0	0	0	0	0	0
1106	1450	250	347	10941	0	0	764	0	0	67	46	77	0	0	0	1662	2041
0	0	0	0	0	0	0	0	0	0	0	0	0	0	66	0	0	0
0	0	0	0	0	0	0	0	206	0	0	0	0	0	0	0	0	0
0	0	0	0	0	0	0	0	0	0	0	0	0	0	0	0	0	0
0	0	0	0	0	0	2280	0	0	0	0	0	0	0	0	0	0	0
0	0	69	0	0	0	0	0	0	0	0	0	0	0	0	0	0	0
0	0	0	0	0	0	0	0	0	0	0	0	0	0	0	0	0	0
0	63	0	0	0	0	0	0	0	0	0	0	0	0	0	0	0	0
0	0	0	0	0	0	0	0	0	0	0	0	0	0	0	0	0	0
0	0	0	0	76	0	0	0	0	0	0	22189	0	0	0	0	0	61
0	0	0	0	0	0	0	0	0	0	0	47	0	0	0	0	112	0
0	0	0	57	0	0	0	0	0	0	0	0	0	0	0	0	0	0
0	0	0	0	0	0	0	0	0	0	0	0	0	0	0	0	0	0
0	0	0	0	15239	0	0	0	0	0	0	11564	0	0	0	0	197	0
0	0	0	0	0	0	0	0	0	0	0	0	0	0	0	0	0	0
0	0	0	0	0	0	0	0	0	0	0	0	0	0	0	361	0	0
0	0	0	66	0	0	0	0	0	0	0	0	0	0	0	0	0	0
0	0	0	0	0	0	0	0	0	0	0	0	0	0	0	0	0	0
82	2915	0	0	0	0	0	0	0	0	0	0	0	0	0	0	0	0
0	0	0	0	0	0	0	0	0	0	0	0	0	0	0	0	0	0
0	0	0	0	0	0	0	0	0	0	0	0	0	0	0	0	0	0
0	0	0	0	0	0	0	0	0	0	0	0	0	0	0	0	0	0
0	0	0	0	0	0	0	0	0	0	0	0	0	0	0	0	0	0
308	76	844	2217	8845	7936	424	72851	2118	2150	4637	2717	952	1652	20609	4277	7599	2195

0	0	0	0	0	0	0	0	0	0	0	0	0	0	0	0	0	0
0	0	0	0	0	112	499	0	249	237	0	0	0	0	0	0	0	0
0	0	0	114	0	0	0	0	0	0	0	0	0	0	0	0	0	0
0	0	0	0	0	0	0	0	0	0	0	0	0	0	0	0	0	0
0	0	0	502	167	0	0	0	0	0	0	0	0	166	88	0	206	0
0	0	0	0	0	0	0	0	0	0	0	0	0	0	0	0	0	0
0	0	0	0	0	0	0	0	0	0	0	0	0	0	0	0	0	0
0	0	0	0	0	0	0	164	0	0	0	0	0	0	0	0	0	0
0	65	0	0	0	0	0	170	0	0	0	0	0	0	0	0	0	0
217	63	0	0	0	0	0	146	0	0	0	0	76	0	599	0	0	0
0	0	0	0	0	0	0	0	0	0	0	0	0	0	0	0	0	0
0	0	0	0	0	0	0	0	0	0	0	0	0	0	0	0	0	0
0	0	0	0	0	0	0	0	0	0	0	0	0	0	0	0	0	0
0	0	0	0	0	0	0	0	0	0	0	0	0	0	0	0	0	0
0	0	0	0	0	0	0	0	0	0	0	0	0	0	0	0	0	0
0	0	0	0	0	0	0	0	0	0	0	0	0	0	0	0	0	0
10562	59	110	3766	146	3476	1388	439	4750	74249	213	44	2656	268	755	133	0	251
0	0	0	0	0	0	0	0	0	0	0	0	0	0	0	0	0	0
0	72	553	115	167535	385	354	0	509	643	9216	20436	67150	599034	44451	3656	35296	1192
0	0	0	0	0	0	0	0	0	0	0	0	0	0	0	0	0	0
0	0	1396	976	86	0	0	0	0	0	0	0	0	0	0	0	0	0
0	0	0	0	0	0	0	0	0	0	0	0	0	727	0	0	189	453
0	786	0	0	0	0	1018	0	0	0	0	0	0	0	0	0	282	0
0	0	0	0	0	0	0	0	0	0	0	0	0	0	0	0	0	0
0	0	0	0	0	0	0	0	0	0	0	0	0	0	0	0	0	0
0	0	0	0	0	0	0	151	0	0	0	0	0	0	0	0	0	0
0	0	0	0	0	0	0	0	0	0	0	0	0	0	0	0	0	0
0	0	0	0	0	0	0	0	0	0	0	0	943	0	0	0	0	0
0	0	0	0	0	0	0	0	0	0	0	0	0	0	0	0	0	0
0	0	0	0	0	0	0	0	0	0	0	0	0	0	0	0	0	0
0	0	0	0	0	0	0	0	0	0	0	0	0	0	0	0	0	0
79546	357	679	322	8241	60978	82784	59970	65476	29202	1510	391	0	1515	31938	1005	2891	1533
0	0	0	0	0	29	27	0	69	0	0	0	0	0	0	0	0	0
0	0	0	0	0	0	0	0	0	0	0	0	0	0	0	0	0	0
0	0	0	0	0	0	0	0	0	0	0	0	0	0	0	0	0	0
0	0	0	0	0	0	0	0	0	0	0	0	0	0	0	0	0	0
0	0	0	0	0	0	0	0	0	0	0	0	0	0	0	0	0	0
0	0	0	0	0	0	0	0	0	0	0	0	0	0	0	0	0	0
0	0	0	0	0	0	0	0	0	0	0	0	0	0	0	0	0	0
0	0	0	0	2035	0	0	0	0	0	480	2837	232	252	1896	709	166	756
80	62	0	0	0	0	0	267	0	0	0	0	0	0	0	0	0	0
0	0	0	0	0	0	0	0	0	0	0	0	0	0	0	0	0	0
0	0	0	0	0	31	0	0	0	0	32043	46	154	0	79	114	191	42
0	0	0	0	0	0	0	0	0	0	0	0	0	0	0	0	0	0
0	0	0	0	0	0	0	0	0	0	0	0	0	0	0	0	0	0
0	0	0	0	0	0	0	0	0	0	0	0	0	0	0	0	0	0
0	0	0	18957	0	0	0	0	0	0	0	0	0	0	0	0	0	0
0	0	0	0	0	0	0	0	0	0	0	0	0	0	0	0	0	0
159	0	0	0	0	38	61	167	127	0	0	0	0	0	0	0	0	0
0	0	0	0	0	0	0	0	0	0	0	0	0	0	0	0	0	0
0	0	7806	0	0	0	0	0	0	0	0	0	0	0	0	0	0	0
0	0	0	0	166	40	66	0	73	0	0	132	16884	0	0	116	103	18240
0	0	0	0	0	0	0	0	0	0	0	0	0	0	0	0	0	0
0	0	179	61	77	84	20	451	123	37	20135	0	0	3750	1603	1190	1894	2482
0	0	0	0	0	0	0	0	0	0	0	0	0	0	0	0	0	0
0	0	0	0	0	0	0	153	0	0	10243	100	82	0	0	0	0	0
0	0	0	0	0	0	0	0	0	0	0	0	0	0	0	0	0	0
0	0	0	0	0	171	0	181	130	0	0	0	0	0	0	0	0	0
0	0	0	0	0	0	0	0	0	0	0	0	0	0	0	0	0	0
0	0	0	0	0	0	0	0	0	0	0	0	0	0	0	0	0	0
0	0	0	0	0	0	0	0	0	0	0	0	0	0	0	0	0	0
0	0	0	0	0	0	0	0	0	0	0	0	0	0	2467	0	0	0
0	0	0	0	0	0	0	0	0	0	0	0	0	0	0	0	0	0
0	0	0	0	0	1436	0	0	0	0	0	0	0	0	0	0	0	0
0	0	0	0	0	0	0	0	0	0	0	57	0	0	156	0	1666	0
0	0	341	184	0	0	0	0	0	0	0	0	0	66	77	0	277	0
0	0	0	0	0	0	0	0	0	0	0	0	0	0	0	0	0	0
0	0	0	0	0	0	0	0	0	0	0	0	0	0	0	0	0	0
0	0	0	0	0	0	26	155	0	42	0	46	0	0	0	0	0	4191
0	0	0	0	0	0	0	0	0	0	0	0	0	0	0	0	0	0
0	0	0	0	0	0	0	0	0	0	0	0	0	0	0	0	0	0
0	0	0	0	0	0	0	0	0	0	0	54	531	4855	0	583	0	1125
0	0	0	0	0	0	0	0	53	27	0	0	0	0	0	0	0	0
0	0	0	0	81	37	23	0	0	0	0	9270	0	83	0	0	0	0
0	0	0	0	0	0	0	0	0	0	0	0	0	0	0	222	0	0
0	0	0	0	0	81	186	0	66	114	0	0	0	0	0	0	0	0
0	0	0	0	0	0	0	0	0	0	0	0	0	0	0	0	0	0
0	0	0	0	0	0	0	0	0	0	0	0	0	0	0	0	0	0
0	0	0	0	0	0	0	0	0	0	2101	0	0	0	0	0	0	0
0	0	0	0	0	0	0	0	0	0	0	0	0	0	0	0	0	0
4936	68	0	0	234	2149	629	1397	4485	2858	430	0	0	175	737	374	208	62
0	0	0	0	0	0	0	0	0	0	0	0	0	0	0	0	0	0
0	0	0	0	0	0	0	0	0	0	0	0	0	0	0	0	0	0
0	0	0	0	0	0	0	0	0	0	0	0	0	0	0	0	0	0
0	0	0	61	0	0	0	0	0	0	0	0	0	0	0	0	404	0
0	0	0	0	0	0	0	0	0	0	0	0	1921	0	0	0	0	0
0	0	0	0	0	0	0	0	0	0	0	0	0	0	0	0	0	0

0	0	0	8018	12465	0	0	0	0	0	5414	46	76	0	75	0	0	1447
0	0	0	0	0	0	0	0	0	0	0	0	0	0	1481	0	0	0
0	0	0	0	0	0	0	0	0	0	0	0	0	0	0	0	1035	0
0	0	0	0	0	0	0	0	0	0	0	0	0	0	0	0	0	0
0	0	0	0	0	0	0	0	0	0	0	0	0	0	0	0	0	0
0	0	54	19629	0	0	0	0	0	0	0	0	0	0	0	0	0	0
0	0	0	0	0	0	0	0	0	0	0	0	0	0	0	0	0	0
0	0	0	0	0	0	55	0	0	0	0	0	0	0	0	0	0	0
0	0	0	0	0	0	0	0	0	0	0	0	0	0	0	0	0	0
0	0	0	0	0	0	0	0	0	0	0	0	0	0	0	0	0	0
0	0	54	0	6826	0	0	453	0	0	140	0	76	0	0	0	0	0
0	0	0	0	0	0	0	0	0	165	0	0	0	0	0	0	0	0
0	0	0	0	0	0	0	0	0	0	0	0	0	0	0	0	0	0
0	0	0	0	0	0	0	0	0	0	0	0	0	0	0	0	0	0
0	0	0	0	0	0	0	0	0	0	0	0	0	0	0	0	0	0
0	0	0	0	0	0	0	0	0	0	0	0	0	0	0	0	0	0
0	0	0	0	0	0	0	0	0	0	0	0	0	0	0	0	0	0
0	0	0	0	0	0	30	0	0	0	0	0	0	0	0	0	0	0
0	0	0	0	0	0	0	0	0	0	0	0	0	0	0	0	0	0
0	0	0	0	0	0	0	0	0	0	0	0	0	0	0	0	0	0
4057	2219	697876	113319	13993	15519	2062	64944	5205	18336	17487	5458	6992	7254	56872	2829	7976	4220
0	0	0	0	0	0	0	0	0	0	0	0	0	0	0	0	0	0
0	58	0	0	0	0	0	144	0	0	0	0	0	0	0	0	0	0
0	0	0	0	0	0	0	0	70	0	0	0	0	0	314	0	0	0
0	0	0	0	0	0	0	0	0	0	0	0	0	0	0	0	0	0
0	0	0	0	0	0	0	0	0	0	0	0	0	0	0	0	0	0
0	0	0	0	0	0	0	0	0	0	0	0	0	0	0	0	0	0
333	0	2654	0	0	111	74	0	187	431	0	0	0	90	0	0	0	0
0	0	0	0	0	0	0	0	0	0	0	0	0	0	0	0	0	0
CC005	CC004	CC002	CC001	CC035	CC007	CC016	CC003	CC018	CC017	CC032	CC030	CC029	CC028	CC027	CC026	CC031	CC024
0	0	0	0	0	0	0	0	0	0	0	17824	0	0	0	108	0	56
0	0	0	0	0	0	0	0	0	0	0	0	0	0	0	0	0	0
0	0	0	0	0	0	0	0	0	0	0	0	0	0	0	0	0	0
0	0	0	0	0	0	0	0	0	44	0	0	0	0	0	0	0	0
63	0	0	0	0	62	0	151	132	51	0	0	0	0	0	0	0	0
0	0	0	0	0	0	0	0	0	0	0	0	0	0	0	0	0	0
0	0	0	0	0	0	0	0	0	0	0	0	0	0	0	0	0	0
754390	4672	58777	35077	117291	793879	734431	641574	791166	742299	181224	29002	14953	10576	367229	37473	46205	44489
0	0	0	0	3943	0	0	0	0	0	0	0	0	0	0	0	0	0
0	0	124	58	0	0	0	0	0	0	0	0	0	0	0	0	0	0
0	0	0	0	0	0	0	0	0	0	0	0	0	0	0	0	0	0
0	0	0	0	0	0	0	0	0	0	8308	0	0	0	0	0	100	0
0	0	0	0	0	28	55	0	0	46	0	0	0	0	0	0	0	0
0	0	0	0	0	38	0	0	0	0	0	0	0	0	0	0	0	0
0	0	0	0	0	0	0	0	0	0	0	0	0	0	0	0	0	0
0	0	0	0	0	0	0	0	0	0	0	0	0	0	0	0	0	0
0	46	68	71	0	0	0	0	0	0	211	0	0	182	0	0	488	0
0	0	382	10371	82	146	104	142	0	379	13026	81	83	3231	0	139	16702	2391
0	0	0	0	0	0	0	0	0	0	0	0	0	0	0	0	0	0
0	0	0	0	0	0	0	0	0	0	0	0	0	0	0	0	0	0
0	0	0	0	0	0	0	0	0	0	0	0	0	0	0	0	0	0
0	0	0	0	0	35	0	0	0	0	0	0	0	0	0	0	0	0
0	0	0	0	0	0	0	0	0	0	0	0	0	0	0	0	0	0
0	0	0	0	0	0	0	0	0	0	0	0	0	0	0	0	0	0
18344	252	310	316	1738	16697	12596	22659	18728	14120	3251	811	211	94	7746	742	706	393
0	0	0	0	0	0	0	0	0	0	0	0	0	0	0	0	0	0
0	0	0	0	0	0	0	0	0	0	0	0	0	0	0	0	0	0
0	0	0	0	0	0	0	0	0	0	0	0	0	0	0	0	0	0
0	58	0	0	0	0	0	0	0	0	0	0	0	0	0	0	0	0
0	0	0	0	0	0	0	0	0	0	0	0	0	0	1580	0	0	0
0	0	139	54	0	0	0	0	0	0	0	0	302	0	0	367	0	0
0	0	0	0	0	0	0	0	0	0	0	0	0	0	0	0	0	0
0	0	0	0	0	0	0	0	0	0	0	0	0	0	0	0	0	0
0	0	0	0	0	0	0	0	0	0	0	0	0	0	0	0	0	0
0	0	0	0	0	0	0	0	0	0	0	0	0	0	0	0	0	0
98	0	135	331	0	179	146	0	237	82	0	210	0	86	0	0	196	0
0	0	0	0	0	0	0	0	0	0	0	0	0	0	0	0	0	0
0	0	0	2041	3404	0	0	0	0	0	3678	7030	9128	973	1851	10300	382	3314
0	0	0	0	0	0	0	0	0	0	0	0	0	0	0	0	0	0
0	0	0	0	0	0	0	0	0	0	0	0	0	0	0	0	0	0
0	0	0	0	0	0	0	0	0	0	0	0	0	0	0	0	0	0
0	0	0	12544	91	66	32	0	69	75	61	43	71	1626	0	1453	0	10759
0	0	0	9628	0	0	0	0	0	0	0	0	94	0	0	0	106	0
0	0	0	0	0	0	0	0	0	0	0	0	0	0	0	0	0	0
0	0	0	0	0	0	0	0	0	0	0	0	0	0	0	0	0	0
0	0	0	0	0	0	0	0	0	0	0	0	0	111	0	0	0	0
0	0	0	0	0	0	0	0	0	0	0	0	0	0	0	0	0	0
0	0	0	0	0	0	0	0	0	0	50	0	0	880	0	467	708	4848
0	0	0	0	0	0	0	0	0	0	0	3447	0	0	0	0	0	0
0	0	0	0	0	0	0	0	0	0	0	0	0	0	0	0	0	0
0	0	0	0	0	38	0	0	0	0	0	0	0	0	0	0	0	0
0	0	0	0	0	0	0	0	0	51	0	0	0	0	0	0	0	0
0	0	0	0	0	0	0	0	0	0	0	9634	0	0	0	117	0	0
0	0	0	0	0	0	0	0	0	0	0	0	0	0	0	0	0	0
0	0	0	0	0	0	0	0	0	0	0	0	0	0	0	0	0	0

0	0	0	0	0	0	0	0	0	0	0	0	0	0	0	0	0	0
0	138	0	0	2436	0	97	0	0	117	1705	10950	0	641	1243	127	0	2820
0	0	0	1120	0	0	0	0	0	0	0	0	0	0	0	0	0	0
0	0	0	0	1288	0	147	327	0	0	13862	0	389	84	228	0	0	60
32944	584	1433	342269	56449	11818	5748	8040	15709	26044	2581	28177	33376	20842	24464	3412	66563	1256
0	0	1550	0	321	0	0	0	0	0	0	515	22989	99	548	0	90	0
0	0	167775	137	68	0	0	0	0	0	8384	56	0	0	0	0	0	0
0	0	0	6986	0	0	0	0	0	0	0	0	0	0	0	0	0	0
0	137	194	0	4510	0	35	0	0	58	0	21394	3761	111	10962	0	424	0
0	0	0	0	0	0	62	0	67	88	0	0	0	0	0	0	0	1666
0	0	0	0	0	0	0	0	0	0	0	0	0	0	0	0	0	0
0	0	0	63	0	0	0	0	0	0	0	0	0	0	0	0	0	0
0	0	0	0	0	0	0	0	0	0	0	0	0	0	0	0	0	0
0	0	0	0	0	0	6643	749	129	53	0	0	71	0	0	1837	5503	53
0	0	0	0	0	0	0	0	0	0	0	0	0	0	0	0	0	0
0	937	0	0	0	0	0	0	0	0	0	0	0	0	2436	0	0	0
0	0	0	0	0	0	0	0	0	0	0	0	0	0	0	0	0	0
0	0	0	0	0	0	0	0	0	0	0	0	0	0	0	0	0	0
0	0	0	0	0	0	0	0	0	0	0	0	0	0	0	0	0	0
0	0	0	0	0	34	2036	0	127	0	0	0	0	0	0	0	0	0
0	0	0	66	0	0	0	0	0	0	0	0	0	0	0	0	0	0
0	0	0	0	0	0	0	0	0	0	0	0	0	0	0	0	0	0
0	0	0	0	0	0	0	0	0	0	0	0	0	0	0	0	0	0
0	0	0	0	0	0	0	0	0	0	0	0	0	0	0	0	0	0
0	0	0	0	0	0	0	0	0	0	0	0	0	0	0	0	0	0
0	0	0	0	0	0	0	629	0	0	0	0	0	0	0	0	0	0
0	0	0	0	0	256	0	0	0	91	221	47	0	0	77	0	1925	217
0	0	0	0	0	0	0	0	0	0	0	0	0	0	0	0	0	0
0	0	0	0	0	0	0	0	0	0	0	0	0	0	0	0	0	0
0	0	0	0	0	0	0	0	0	0	0	0	0	0	0	0	0	0
0	0	0	0	0	0	0	0	0	0	0	0	0	0	0	0	0	0
0	665	0	0	0	0	0	0	0	0	0	9580	0	966	0	0	0	1850
0	0	0	0	0	0	0	0	0	0	0	0	0	0	0	0	0	0
0	0	0	0	0	0	0	0	0	0	0	0	0	0	0	0	0	0
0	0	0	0	0	0	0	0	0	0	0	0	0	0	0	0	0	0
1875	1005	752	269037	57885	152	108	969	359	639	3274	1837	59401	19970	23673	3065	261862	969
0	0	0	0	0	0	0	0	0	0	0	0	0	0	0	0	0	0
0	0	0	0	0	0	0	0	0	0	0	0	0	0	0	0	0	0
0	0	0	0	0	0	0	0	0	0	0	0	0	0	0	0	0	0
0	0	0	0	70	0	0	0	0	0	0	0	78	0	0	0	1677	0
0	0	61	43427	0	0	0	0	0	0	0	0	0	0	0	0	0	0
0	0	0	0	0	0	63	0	0	0	0	0	0	0	0	0	0	0
0	0	0	0	0	0	0	0	0	0	0	0	0	0	0	0	0	0
0	0	0	0	0	0	0	0	0	0	0	0	0	0	0	0	0	0
0	0	0	0	0	0	0	0	67	0	0	0	0	0	0	0	0	0
0	0	0	0	0	0	0	0	0	0	0	0	0	0	0	0	0	0
0	0	0	0	0	0	0	0	0	0	0	0	0	0	0	0	569	0
0	0	0	0	0	0	0	0	0	0	0	0	0	0	0	0	0	0
0	0	0	0	0	0	0	0	0	0	0	0	0	0	0	0	0	0
0	0	0	0	0	0	0	0	0	0	0	0	0	0	0	0	0	0
835	0	0	0	0	1436	1427	603	1062	0	0	0	0	0	0	0	101	0
0	0	0	0	0	0	0	0	0	0	0	0	0	0	0	0	0	0
0	0	0	0	0	0	0	0	0	0	0	0	0	0	0	0	0	0
1922	900962	0	0	0	0	75	23820	0	0	328	260	11213	183	1812	116	0	0
75	82	0	0	0	0	0	142	0	0	0	0	0	0	0	3114	0	63
0	0	0	0	146	0	0	0	0	0	0	91	0	0	0	0	29426	0
0	0	0	0	0	0	0	0	0	0	0	0	0	0	0	0	0	0
0	0	116	105	0	0	0	0	0	0	0	0	0	0	0	0	0	0
0	0	0	0	0	0	97	0	72	103	0	0	0	0	0	0	0	0
0	0	0	0	0	0	0	0	0	0	0	0	0	0	0	0	0	0
0	0	0	0	0	0	0	0	0	0	0	0	0	0	0	0	0	0
0	0	0	0	0	55	69	477	60	0	0	0	0	0	14626	0	0	0
0	0	0	56	0	0	0	0	0	0	0	0	0	0	0	0	0	0
0	0	0	0	0	0	0	0	0	0	0	0	0	0	0	0	0	0
0	0	323	122	76	0	0	0	0	0	0	0	19410	0	0	0	0	0
153	0	0	58	158	0	0	944	0	92	214	256	344	283	0	116	302	24705
70	42747	64	0	0	0	0	0	0	0	0	0	0	0	201	2511	0	0
0	0	0	0	0	0	0	0	0	38	0	49	0	0	0	96	0	3214
0	0	113	69	0	0	0	329	0	0	14064	0	0	0	0	0	0	2161
0	0	61	0	0	0	0	0	0	0	0	0	0	0	0	0	0	0
0	0	0	0	0	0	0	0	0	0	0	0	0	0	0	0	0	0
0	0	0	0	0	0	0	0	0	0	0	0	0	0	0	0	0	0
0	0	0	0	0	0	0	0	0	0	0	0	0	0	0	0	0	0
0	0	0	0	0	0	0	0	0	0	0	0	0	0	0	0	0	0
0	0	0	0	0	0	0	0	0	0	0	0	0	0	0	0	0	0
0	0	0	0	0	0	0	0	0	0	0	0	0	0	0	0	0	0
0	0	0	0	0	0	0	0	0	0	0	0	0	0	2203	0	0	0
0	0	0	0	0	0	0	0	0	0	0	0	0	0	0	0	0	0
0	0	0	0	0	0	0	0	0	0	0	0	0	0	0	0	0	0
0	0	0	0	0	0	77	0	0	0	0	0	0	0	0	0	0	0
0	0	0	0	0	0	0	0	0	0	0	0	0	0	0	0	0	0
0	0	0	0	0	0	0	0	0	0	0	0	0	0	0	0	0	0
0	0	0	0	0	0	0	0	0	0	0	0	0	0	0	0	0	0
0	0	0	0	0	0	0	0	0	0	0	0	0	0	0	0	0	0
0	0	0	0	0	0	0	0	0	0	0	0	0	0	0	0	0	0
83	0	70	0	239340	0	0	310	0	0	308275	563653	574815	45036	226783	862351	48867	324164
0	0	0	0	0	0	0	0	0	0	0	0	0	0	0	0	0	0

0	0	0	0	0	0	0	0	0	0	0	0	0	90	83	0	0	233
0	0	0	0	0	0	0	0	0	0	0	0	0	0	0	0	0	0
0	0	0	0	0	0	0	0	0	0	0	0	0	0	0	0	0	0
0	0	0	0	0	0	0	0	0	0	0	0	0	0	0	0	0	0
76	706	76	9154	4026	150	161	963	219	80	0	46	83	168	5399	1112	23290	1618
0	0	0	63	93	0	0	0	0	0	0	0	0	0	166	0	118	0
243	3271	0	22776	35777	681	0	755	0	0	0	137	3603	2105	6352	117	989	6895
0	138	346	20159	5846	0	0	164	124	170	60	84	0	3566	2992	228	24093	18933
0	3704	0	4535	71	0	0	0	0	0	549	0	0	545	286	139	4628	168
0	0	0	0	0	0	0	0	0	0	15955	63	83	904	0	0	2275	0
0	0	0	0	0	0	0	0	0	0	0	0	0	0	0	0	0	0
0	0	0	0	0	0	0	0	0	0	0	0	0	80	0	0	0	1173
0	0	0	0	0	0	0	0	0	0	0	0	0	349	0	0	0	0
0	0	0	0	0	0	0	0	0	0	0	0	0	0	0	0	0	0
0	0	0	0	0	0	0	0	0	0	0	0	0	0	0	0	0	0
0	0	0	0	0	0	0	0	0	0	0	0	0	0	0	0	0	0
0	0	0	0	0	0	0	0	0	0	0	0	0	0	0	0	0	0
0	0	0	0	0	0	0	0	0	0	0	0	0	0	0	0	0	0
0	65	0	0	0	0	0	0	0	0	0	0	0	0	0	0	0	0
0	0	0	0	0	0	0	0	0	0	0	0	0	0	0	0	6384	0
0	0	0	0	0	0	0	0	0	0	0	0	0	0	0	0	0	0
0	0	0	0	0	0	0	0	0	0	0	0	0	0	70	0	0	0
0	0	0	0	0	0	0	0	0	0	0	0	0	0	0	0	0	0
0	0	0	0	0	0	0	0	0	0	0	0	0	0	0	0	0	0
0	0	0	0	0	0	0	0	0	0	0	0	0	0	0	0	0	0
0	0	68	0	0	0	0	0	0	0	0	0	0	0	0	0	0	0
0	0	0	0	0	0	0	0	0	0	0	0	0	0	0	0	0	0
0	0	0	0	0	0	0	0	0	0	0	0	0	0	0	0	0	0
89	0	0	0	0	0	0	291	78	44	68	0	0	0	0	146	0	0
0	0	0	0	0	0	0	0	0	0	0	0	0	0	0	0	0	0
0	0	0	0	0	0	0	0	0	0	0	0	0	0	0	0	0	0
0	0	0	0	0	0	0	0	0	0	0	0	0	0	0	0	0	0
0	0	852	326	74	0	0	0	0	0	0	91	0	0	0	0	0	0
0	0	0	0	0	0	0	0	0	0	0	0	0	0	0	0	0	0
0	0	0	0	0	0	0	0	0	0	0	0	0	0	0	0	0	0
0	0	0	0	0	0	0	0	0	0	0	0	0	0	0	0	0	0
0	0	0	0	0	0	0	0	0	0	0	0	0	0	0	0	0	0
0	0	0	0	0	0	0	0	0	0	0	0	0	0	0	0	0	0
0	0	0	0	0	0	0	0	0	0	0	0	0	0	0	0	0	0
62	0	0	0	4993	0	0	306	0	0	61	0	0	0	0	1327	0	0
0	0	0	0	0	0	0	0	0	0	0	0	0	0	0	0	0	0
0	0	0	0	0	0	0	0	0	0	0	0	0	0	0	0	0	0
0	0	0	0	0	0	0	0	0	0	0	0	0	0	0	0	0	0
0	0	0	0	0	0	0	0	0	0	0	0	0	0	0	0	0	0
0	0	0	0	0	0	0	0	0	0	0	0	0	0	0	0	0	0
0	0	0	0	0	0	0	0	0	0	0	0	0	0	0	0	0	0
0	0	0	0	0	0	0	0	0	0	0	0	0	0	0	0	0	0
0	0	0	0	0	0	0	0	0	0	0	0	0	0	0	0	105	0
0	0	0	0	0	0	0	0	0	0	0	0	0	0	0	0	0	0
0	0	0	0	0	0	0	0	0	0	0	0	0	0	0	0	0	0
0	0	0	0	0	0	0	0	0	0	0	0	0	0	0	0	0	0
0	0	0	0	0	0	0	0	0	0	0	0	0	0	0	0	0	0
0	0	0	0	0	0	0	0	0	0	289	0	0	0	0	0	0	0
0	0	0	118	0	0	0	0	0	0	0	0	0	0	0	0	0	0
0	0	0	0	0	0	0	0	0	0	69	0	0	0	0	0	0	0
0	0	0	0	0	0	0	0	0	0	0	0	0	0	0	0	0	0
0	0	0	0	38188	0	0	0	0	0	66	255	0	94	0	0	0	0
0	0	0	0	0	0	0	0	0	0	0	0	0	0	0	0	0	0
0	0	0	0	0	0	0	0	0	0	0	0	0	0	0	0	0	0
0	0	0	0	0	0	0	0	0	0	0	0	0	0	0	0	0	0
0	0	0	0	0	0	0	0	0	0	0	0	0	0	0	0	0	0
0	0	0	0	0	0	0	0	0	0	0	0	0	0	0	0	0	0
0	0	0	0	0	0	0	0	0	0	0	0	0	0	0	0	0	0
0	0	0	0	0	0	0	0	0	0	0	0	0	0	0	0	0	0
0	0	0	0	0	0	0	0	0	0	0	0	0	0	0	0	0	0
0	0	0	0	0	0	0	0	0	0	0	0	0	0	0	0	0	0
0	0	0	0	0	0	0	0	0	0	0	0	0	0	0	0	0	0
0	0	0	0	0	0	0	0	0	0	0	0	0	0	0	0	0	0
0	0	0	0	14422	41	0	0	0	0	64	50	0	0	0	0	95	108
0	0	0	0	0	0	0	0	0	0	0	0	0	0	0	0	0	0
0	0	0	0	0	0	0	0	0	0	0	0	0	0	0	0	0	0
0	0	71	0	0	0	0	0	0	0	0	0	0	0	0	0	0	0
0	0	870	466	0	0	0	0	0	0	0	0	0	0	0	0	944	0
0	0	0	56	0	0	0	0	0	0	71	20030	85	0	0	0	88	61
0	0	0	0	0	0	0	0	0	0	0	0	0	0	0	0	0	0
4265	8830	116	2283	0	1059	45191	336	1716	37241	0	150	78	273	902	0	889	215
0	0	0	0	0	0	0	0	0	0	0	0	0	0	0	0	0	0
0	0	0	0	0	0	0	0	0	0	0	0	0	0	0	0	0	0
0	0	0	0	0	0	0	0	0	0	138	0	0	0	0	0	0	0
0	0	0	0	0	0	0	0	0	0	0	0	0	0	0	0	0	0
0	0	0	0	0	0	0	0	0	0	0	0	0	0	0	0	0	0
0	0	0	0	0	0	0	0	0	0	0	0	0	0	0	0	0	0
80	0	0	0	0	0	0	0	119	0	0	0	0	0	0	0	0	0
0	0	0	0	0	0	0	0	0	0	0	0	0	0	0	0	0	0
0	0	0	0	0	0	0	0	0	0	0	0	0	0	0	0	0	0
0	0	0	0	0	0	0	0	0	0	0	0	0	0	0	0	0	0
0	190	0	0	0	0	0	0	0	43	0	743	0	0	82	133	0	391

0	0	0	0	0	0	0	0	0	0	0	0	0	0	0	0	0	0
12441	0	0	67	0	2322	240	309	5134	10096	0	0	0	115	520	0	0	51
239	0	0	0	0	212	0	323	152	0	0	0	0	0	0	0	0	0
0	0	0	0	0	0	0	0	0	0	0	0	0	0	0	0	0	0
0	0	0	0	0	0	0	0	0	0	0	0	0	0	0	0	0	0
0	0	0	0	0	0	0	0	0	0	0	0	0	0	0	0	0	0
0	68	0	0	0	0	0	144	0	0	0	0	0	0	0	0	788	0
0	0	0	0	0	0	0	0	0	0	0	0	0	0	0	0	0	0
0	0	0	0	0	0	0	0	0	0	0	0	0	0	0	0	0	0
0	0	0	0	0	0	0	0	0	0	0	0	0	0	0	0	0	0
0	0	0	0	0	0	0	0	0	0	0	0	0	0	0	0	0	0
0	0	0	0	0	0	0	0	0	0	0	0	0	0	0	0	0	0
0	0	0	0	0	0	0	0	0	0	0	0	0	0	0	0	0	0
0	0	0	0	0	0	0	0	0	0	0	0	0	0	0	0	0	0
0	0	0	0	0	0	0	0	0	0	0	0	0	0	0	0	0	0
0	0	0	0	0	0	0	0	0	0	0	0	0	0	0	0	0	0
0	0	0	0	0	0	0	0	0	0	0	0	0	0	0	0	0	0
0	0	0	0	0	0	0	0	0	0	0	0	0	0	0	0	0	0
0	0	0	0	0	0	0	0	0	0	0	0	0	0	0	0	0	0
0	0	0	0	0	0	0	0	0	0	0	0	0	0	0	0	0	0
0	0	0	0	0	0	0	0	0	0	0	0	0	0	0	0	0	0
0	0	0	0	0	0	1139	0	0	0	0	0	0	0	0	0	0	0
6273	59	542	62	9969	7638	14081	13499	9622	1780	6543	53	168	297	3129	373	308	222
0	0	0	0	0	0	0	0	0	0	0	0	0	0	0	0	0	0
0	0	0	0	162	0	119	160	0	0	12074	143	102377	265	5516	239	1002	22612
77	0	0	0	0	0	0	0	0	0	0	0	0	0	0	0	0	0
0	0	0	0	0	0	0	0	0	0	0	0	0	0	0	0	0	0
0	0	0	0	0	0	0	0	0	0	0	0	0	0	0	0	0	0
0	0	0	0	0	0	0	0	0	0	0	0	0	0	478	0	0	0
0	0	0	0	0	0	0	0	0	0	0	0	0	0	0	0	0	0
0	0	275	64	0	0	0	0	0	0	0	0	0	0	0	0	0	0
0	0	0	0	0	0	0	0	0	0	0	0	0	0	0	0	0	0
0	0	0	0	0	0	0	0	0	0	0	0	0	0	169	0	0	0
21855	10142	31447	16269	6472	19351	47333	40795	21082	19711	37421	15756	7419	1031	14943	1265	4580	1496
0	0	0	0	2401	0	0	0	0	0	8005	61	0	708	0	372	0	5128
0	0	0	0	0	0	0	0	0	0	0	0	0	261	0	234	214	1381
0	0	0	0	159	73	0	318	0	0	82	93	80	13980	0	6035	3355	26489
0	0	0	0	0	0	0	0	0	0	0	0	0	0	0	0	0	0
0	0	0	0	0	0	0	0	0	0	0	0	0	0	0	0	0	0
0	0	0	0	0	84	0	634	0	0	67	93	92	19438	0	3554	7278	4501
0	0	0	0	149	0	0	0	0	0	0	47	0	6666	0	507	0	4824
0	0	0	1225	225	0	0	0	0	0	0	0	252	0	0	0	0	0
0	0	0	0	0	0	0	0	0	0	0	0	0	0	0	0	0	0
0	0	0	0	0	0	0	0	0	0	0	0	0	0	0	0	0	0
0	0	1645	1045	0	109	73	0	146	1053	0	0	0	0	0	0	0	0
0	0	0	0	0	0	0	0	0	0	0	0	0	0	0	0	0	0
0	64	0	0	0	0	564	0	0	0	0	0	0	0	0	0	0	0
0	0	0	0	0	0	0	0	0	0	0	0	0	0	0	0	0	0
0	0	0	0	0	0	0	0	0	0	0	0	0	0	0	0	0	0
0	0	0	0	0	0	0	0	0	0	0	0	0	0	0	0	0	0
0	0	0	0	0	0	0	0	0	0	0	0	0	95	0	0	85	58
76	0	0	0	132401	34	0	166	0	0	488	53839	15313	201987	31104	33594	4094	174226
0	0	0	0	0	46	0	451	63	0	0	0	0	0	0	0	0	59
0	0	0	0	0	0	0	0	0	0	0	0	0	0	0	0	0	0
0	0	0	0	0	0	0	0	0	0	0	0	0	0	0	0	0	0
0	0	0	0	92	0	0	0	0	0	34035	42	81	0	0	120	103	0
0	0	0	0	0	0	0	0	0	0	0	0	0	0	0	0	0	0
0	0	0	0	0	0	0	0	0	0	17991	42	71	0	0	130	0	0
0	0	0	0	0	0	0	0	0	0	0	0	0	0	0	0	0	0
0	0	0	0	0	0	0	0	0	0	0	0	0	0	0	0	0	0
0	0	0	0	0	0	0	0	0	0	0	0	0	0	0	0	0	0
0	0	0	0	0	0	0	0	0	0	0	0	0	0	0	0	0	0
0	0	0	0	0	0	0	0	0	0	0	0	0	0	0	0	0	0
0	0	0	0	0	0	0	0	0	0	0	0	0	0	0	0	0	0
0	0	0	0	0	0	0	0	0	0	0	0	0	0	0	0	0	0
0	0	0	0	0	0	0	0	0	0	0	0	0	0	0	0	0	0
0	0	0	0	0	0	0	0	0	0	0	0	0	0	0	0	0	0
0	0	60	0	0	0	0	0	0	0	0	0	0	0	0	0	0	0
0	0	0	0	238	0	0	0	0	0	129078	399	295	189	0	384	391	227
0	0	0	0	0	0	0	0	0	0	0	0	0	0	7894	0	0	0
0	0	0	0	0	0	0	0	0	0	0	2674	0	0	0	251	411	0
0	0	0	0	0	0	0	0	0	0	0	0	0	0	0	0	0	0
672	62	0	0	0	374	1860	778	338	1453	0	0	0	0	0	0	0	0
0	0	0	0	0	0	0	0	0	0	0	0	0	0	0	0	0	0
0	0	0	0	0	0	0	0	0	0	0	0	0	0	0	0	0	0
0	0	0	0	230	0	0	0	0	0	136	76715	159	291	0	118	203	51
0	0	0	0	0	0	0	0	0	0	0	0	0	0	0	0	0	0
0	0	0	0	0	0	0	0	0	0	0	0	0	0	0	0	0	0
0	0	0	0	0	0	0	0	0	0	0	0	0	0	0	0	0	0
0	0	0	0	0	0	0	0	0	0	0	0	0	0	0	0	0	0
0	0	0	0	0	0	0	0	0	0	0	0	0	0	0	0	0	0
0	0	0	0	0	34	101	0	0	107	0	0	0	0	0	0	0	0
0	0	0	0	0	0	0	0	0	0	0	0	0	0	0	0	0	0
0	0	0	0	0	0	0	0	0	0	0	0	0	0	0	0	0	0
0	0	0	0	308	184	2731	0	258	184	0	181	73	3981	21656	1409	181	63779
0	0	0	0	0	0	0	0	0	0	0	0	0	0	0	0	0	0
0	0	0	0	0	0	0	317	0	0	0	0	0	0	0	0	0	0

0	0	0	0	0	0	0	0	0	0	0	0	0	0	0	0	0	0
0	0	0	0	0	0	0	0	0	0	0	0	0	0	0	0	0	0
0	0	0	0	0	0	0	0	0	0	0	0	0	0	0	0	0	0
0	0	0	0	0	0	0	0	0	0	0	0	0	0	0	0	0	0
0	0	0	0	0	0	0	0	0	0	0	0	0	0	0	0	0	0
0	71	0	0	0	0	0	0	0	0	0	0	0	0	0	0	0	0
0	0	0	0	0	0	0	0	0	0	76	53	0	101	0	101	0	12436
83	9220	0	0	0	0	0	0	0	0	0	0	0	0	0	0	0	0
0	0	0	0	0	0	0	0	0	0	0	0	0	0	0	0	0	0
0	0	0	0	0	0	0	0	0	0	0	0	0	0	0	0	0	0
0	0	0	0	0	0	0	0	0	0	0	0	0	0	0	0	0	0
567	0	0	0	0	561	107	458	335	205	0	0	0	0	0	0	0	0
0	0	0	0	85	0	0	0	0	0	0	0	72	0	0	0	14087	0
0	0	0	0	0	0	0	0	0	0	0	0	0	0	0	0	0	0
0	0	0	0	0	0	0	0	0	0	0	0	0	0	0	0	0	0
0	0	0	0	0	0	0	0	0	0	0	0	0	0	0	0	0	0
0	0	0	0	0	0	0	0	0	0	0	0	0	0	0	0	0	0
0	0	0	0	0	0	0	0	0	0	0	0	0	0	0	0	0	0
0	0	0	0	0	0	0	0	0	0	0	0	0	0	0	0	0	0
0	0	0	0	0	0	0	0	0	0	0	0	0	0	0	0	0	0
0	0	0	0	0	0	0	0	0	0	0	0	0	0	0	0	0	0
0	0	0	0	0	0	0	0	0	0	0	0	0	0	0	0	0	0
0	0	0	0	0	0	0	0	0	0	0	0	0	0	0	0	0	0
0	0	0	0	0	0	0	0	0	0	0	0	0	0	0	0	0	0
0	0	0	0	0	0	0	0	0	0	0	0	0	0	0	0	0	0
0	0	0	0	0	0	0	0	0	0	0	0	0	0	0	0	0	0
0	0	0	0	0	0	0	0	0	0	0	0	0	0	0	140	0	0
0	0	0	0	0	0	0	0	0	0	0	0	0	0	0	0	0	0
0	0	0	0	0	0	0	0	0	0	0	0	0	0	0	0	0	0
0	0	0	0	0	0	0	0	0	0	0	0	0	0	0	0	0	0
0	0	0	0	0	0	0	0	0	0	0	0	0	0	0	0	0	0
0	0	0	0	0	0	0	0	0	0	0	0	0	0	0	0	0	0
0	0	0	0	0	0	0	0	0	0	0	0	0	0	0	0	0	0
0	0	0	0	0	0	0	0	0	0	0	0	0	0	0	0	0	0
0	0	0	0	0	0	0	0	0	0	0	0	0	0	0	0	1770	0
0	0	0	0	0	0	0	0	0	0	0	50	0	0	78	120	0	4303
0	0	0	0	0	0	0	0	0	0	0	54	0	3287	0	0	0	0
0	0	0	0	0	0	0	0	0	0	0	0	0	0	0	0	0	0
0	0	0	0	0	0	0	0	0	0	0	0	0	0	232	0	0	0
0	0	0	0	0	0	0	0	0	0	0	0	0	0	0	0	0	0
0	0	0	0	0	0	0	0	0	0	0	0	0	0	0	0	0	0
0	0	0	0	0	0	0	0	0	0	0	0	0	0	0	0	0	0
0	0	0	0	0	0	0	0	0	0	0	0	0	0	0	0	0	0
0	0	0	0	0	0	0	0	0	0	0	0	0	0	0	0	0	0
0	0	0	0	0	0	0	0	0	0	0	0	0	0	0	0	0	0
0	0	0	0	87	125	603	0	215	0	0	0	96	0	0	0	25274	0
0	0	0	0	0	0	0	0	0	0	0	0	0	0	0	0	0	0
0	0	6279	0	0	0	0	0	0	0	0	0	0	0	0	0	0	0
0	0	0	0	0	0	0	0	0	0	0	0	0	0	0	0	0	0
0	129	0	0	372	0	0	0	0	0	2202	832	235	97	0	130	163857	978
0	0	0	0	0	0	0	0	0	0	0	0	0	0	0	0	0	0
319	934	8243	110	219	794	431	306	382	1992	15225	43701	9946	421	4467	816	60330	147206
0	120	0	0	0	92	0	0	0	0	0	0	0	0	379	243	0	4185
0	0	0	0	0	0	0	0	0	0	0	0	0	0	0	0	0	0
0	0	0	0	0	0	0	0	0	0	0	0	0	0	0	0	0	0
0	0	0	0	0	0	0	0	0	0	0	0	0	0	0	0	0	0
0	0	0	0	0	0	0	0	0	0	0	0	0	0	0	0	0	0
0	0	0	0	0	0	0	0	0	0	0	0	78	0	0	331	0	0
0	0	0	0	0	0	0	0	0	0	0	0	0	0	0	0	0	0
0	0	0	0	0	0	0	0	0	0	0	0	0	0	0	0	0	0
872	0	0	0	1244	1105	247	933	1948	92	66	62	0	91	78	709	0	225
0	0	0	0	0	0	0	0	0	0	0	2171	0	0	0	0	0	0
0	0	0	0	0	0	0	0	0	0	0	0	0	0	0	0	0	0
0	0	0	0	0	0	0	0	0	0	0	0	0	0	0	0	0	0
0	0	0	0	0	0	0	0	0	0	0	0	0	0	0	0	0	0
0	0	63	0	0	0	0	0	0	0	0	0	0	0	0	0	0	0
0	0	251	124	0	0	0	0	0	0	0	0	0	0	0	0	0	0
0	0	0	0	0	0	0	0	0	0	0	0	0	0	0	0	0	0
0	0	59	0	0	0	0	0	0	0	0	0	0	0	0	0	0	0
0	0	0	0	0	0	0	0	0	0	0	0	0	0	0	0	0	0
0	0	0	0	0	0	0	0	0	0	0	0	0	3330	4001	626	532	0
0	0	0	0	0	0	0	0	0	0	0	0	0	0	0	0	0	0
0	0	0	0	0	0	0	0	0	0	0	0	0	0	0	0	0	0
0	0	0	0	0	0	0	0	0	0	0	0	0	0	0	0	0	0
0	0	0	0	0	0	0	0	0	0	0	0	0	0	0	0	0	0
0	0	0	0	0	0	0	0	0	0	0	0	0	0	0	0	0	0
0	0	0	0	0	0	0	0	0	0	0	0	0	0	0	0	0	0
0	0	0	0	0	0	0	0	0	0	0	0	0	0	0	0	0	0
0	0	0	0	0	0	0	0	0	0	0	0	0	0	0	0	0	0
0	0	0	0	0	0	0	0	0	0	0	0	0	0	0	0	0	0
0	0	0	0	0	0	0	0	0	0	0	0	0	0	3354	0	0	0
0	0	0	0	0	0	0	0	0	0	0	0	9227	0	0	0	0	0
0	0	0	0	0	0	0	0	0	0	0	0	0	0	0	0	0	0
0	0	0	0	0	0	0	0	0	0	0	0	0	0	0	0	0	0
0	0	52	0	0	0	0	0	0	0	0	0	0	0	0	0	0	0
0	0	0	0	0	0	0	0	0	0	0	0	0	0	0	0	0	0
0	0	0	0	0	0	0	0	0	0	0	0	0	0	0	0	0	0

1166	883	0	0	0	0	0	2833	0	0	0	98	0	457	0	257	22077	0
0	0	0	0	0	0	0	0	0	0	0	0	0	0	0	0	0	0
0	0	0	0	0	0	0	172	0	0	0	0	0	0	0	0	0	0
0	0	0	0	0	0	0	0	0	0	0	0	0	0	0	0	0	0
88	517	0	0	80	78	171	336	335	75	0	0	0	102	0	0	0	13159
0	0	0	0	0	0	0	0	0	0	0	0	0	0	0	0	0	0
0	0	0	0	0	0	0	0	0	0	0	0	0	0	0	0	0	0
0	0	0	13215	0	0	0	0	0	0	0	0	0	0	0	0	0	0
0	0	0	0	0	0	0	0	0	0	0	0	0	0	0	0	0	0
0	0	0	0	0	0	0	0	0	0	0	0	0	0	0	0	0	0
0	0	0	0	0	0	0	0	0	0	0	0	87	0	0	0	0	0
0	0	0	0	0	0	0	0	0	0	0	0	0	99	0	0	0	4382
0	0	0	0	0	0	0	0	0	0	0	0	0	0	0	0	0	100
523	0	62	0	78	501	80	477	323	300	0	0	0	0	0	0	0	0
0	0	0	0	0	0	0	0	0	0	0	0	0	0	0	0	0	0
0	0	0	0	0	0	0	0	0	0	0	0	0	0	0	0	0	0
0	0	0	0	0	0	0	0	0	0	0	0	0	0	0	0	0	0
0	0	0	0	0	0	0	0	0	0	0	0	0	0	0	0	0	0
0	0	0	0	0	0	0	0	0	0	0	0	0	0	0	0	0	0
135	0	0	0	0	0	0	310	0	0	0	0	0	0	0	0	0	0
0	0	0	0	0	0	0	0	0	0	0	0	0	0	0	0	0	0
10681	113	2699	130	1468	10429	9213	6672	13320	4955	2128	433	79	0	5079	755	104	522
0	0	0	0	0	0	0	0	0	0	0	0	0	0	0	0	0	0
0	0	0	0	0	0	0	0	0	0	0	0	0	0	0	0	0	0
0	0	0	0	0	0	0	0	0	0	0	0	0	0	0	0	0	0
0	0	0	0	0	0	0	0	0	0	0	0	0	0	0	0	0	0
0	0	0	0	0	0	0	0	0	0	0	0	0	0	0	0	0	0
0	0	0	0	0	0	0	0	0	0	0	0	0	0	0	0	0	0
74	0	147	1951	83	474	0	168	216	139	0	0	0	180	581	0	783	0
0	0	0	0	0	0	0	0	0	0	0	0	0	0	0	0	0	0
0	0	0	0	0	0	0	0	0	0	0	0	0	0	0	0	0	0
77	0	0	0	0	208	21	166	62	0	0	0	0	0	0	0	0	0
0	0	0	0	0	0	0	0	0	0	7857	0	0	0	0	0	0	0
0	0	0	0	0	0	0	0	0	0	152	0	0	0	0	0	0	0
0	0	0	0	80	0	0	0	0	0	27417	43	0	0	0	0	0	52
0	0	0	0	0	0	0	0	0	0	0	0	0	0	0	124	0	0
0	0	0	0	0	0	0	0	0	0	0	0	0	262	0	0	0	0
0	0	0	0	0	0	0	0	0	0	0	0	0	0	0	0	0	0
0	0	0	0	0	0	0	0	0	0	0	0	0	0	0	0	0	0
0	0	0	0	0	0	0	0	0	0	0	0	0	0	0	0	0	2122
0	0	0	0	0	0	0	0	0	0	0	0	0	0	0	0	0	0
0	0	0	2730	0	0	0	0	0	0	11439	92	69	0	0	0	0	1061
0	0	0	0	0	0	0	0	0	0	0	0	0	0	0	0	0	0
0	0	0	113	0	0	0	0	0	0	0	0	0	0	0	0	0	0
0	0	77	0	0	0	0	0	0	0	0	0	0	0	0	0	0	0
0	0	0	60	0	0	0	0	0	0	0	0	0	0	0	0	0	0
0	0	0	0	0	0	0	0	0	0	0	0	0	0	0	0	0	0
0	0	0	0	0	0	0	0	0	0	0	0	0	0	0	0	0	0
0	0	0	0	0	0	0	0	0	0	0	0	0	189	0	0	0	540
0	0	0	0	0	0	0	0	0	0	0	0	0	0	0	0	0	0
0	0	0	0	76	0	0	0	0	0	0	0	0	0	0	0	0	0
0	0	0	0	0	0	0	0	0	0	0	0	0	0	0	0	0	0
0	0	0	0	248	0	0	0	0	0	0	0	0	0	0	0	0	0
0	0	0	0	0	0	0	0	0	0	0	0	0	0	0	0	0	0
0	0	0	0	0	0	0	0	0	0	0	0	0	5387	0	0	0	0
0	0	0	0	0	0	0	0	0	0	0	0	0	0	0	0	0	0
0	0	0	0	0	0	0	0	0	0	0	0	0	0	0	0	0	0
0	0	0	0	0	0	0	0	0	0	0	0	0	0	0	0	0	0
0	0	0	0	0	0	0	0	0	0	0	0	0	0	0	0	0	0
0	0	0	0	0	0	0	0	0	0	0	0	0	0	0	0	0	0
0	0	0	0	0	0	0	0	0	0	0	0	0	0	0	0	0	0
0	56	67	0	0	628	83	303	57	0	0	0	0	0	0	0	0	0
0	0	0	0	0	0	0	0	0	0	0	0	0	0	0	0	0	0
0	0	0	0	0	0	0	0	0	0	0	0	0	0	0	0	0	0
0	0	0	0	0	0	0	0	0	0	0	0	0	0	0	0	0	0
0	0	0	0	0	0	0	0	0	0	0	0	0	0	0	0	0	0
0	0	0	0	0	0	0	0	0	0	0	0	0	0	0	0	0	0
0	0	0	0	0	0	0	0	0	0	0	0	0	0	0	0	0	0
0	0	0	0	0	0	0	0	0	0	0	0	0	1758	0	0	1294	0
0	0	0	0	0	0	0	0	0	0	0	0	0	0	0	0	0	0
0	0	0	0	0	0	0	0	0	0	0	0	0	0	0	0	0	0
0	0	0	0	0	0	0	0	0	0	0	0	0	0	0	0	0	0
0	0	0	0	0	0	0	0	0	0	0	0	0	0	0	0	0	0
0	0	57	123	0	0	0	0	0	0	0	46	0	0	77	0	0	0
0	0	0	0	0	0	0	0	0	0	0	0	0	0	0	0	0	0
0	0	0	0	0	0	30	0	0	0	0	0	0	0	0	0	0	0
0	0	0	0	0	0	0	0	0	0	0	0	0	0	0	0	0	0
0	0	0	0	0	0	0	0	0	0	0	53	0	0	0	0	0	0
0	0	0	0	0	0	0	0	0	0	0	0	0	0	0	0	0	0
0	0	0	0	0	79	0	0	0	0	0	0	0	0	0	0	0	0
0	0	0	0	0	0	0	0	0	0	0	0	0	0	0	0	0	0
0	0	0	0	0	0	0	0	0	0	0	0	0	0	0	0	0	0
0	0	0	0	0	0	0	0	0	0	0	0	0	0	0	0	0	0
0	0	0	0	0	0	0	0	0	0	0	0	0	0	0	0	0	0
0	0	0	0	0	0	0	0	0	0	0	0	0	0	0	0	0	0
75	0	0	0	0	122	0	325	124	0	0	0	0	0	0	0	0	0
0	0	0	0	0	0	0	0	0	0	0	0	0	0	0	0	0	0

0	0	0	0	0	0	0	0	0	0	0	0	0	0	0	0	0	0
0	0	61	0	0	0	0	0	0	0	0	0	0	0	0	0	0	0
0	0	0	0	0	0	0	0	0	0	0	0	0	0	0	0	0	0
0	0	0	0	0	0	0	0	0	0	0	0	88	101	6180	237	9068	123
0	0	0	0	0	0	0	0	0	0	0	0	0	0	0	0	0	0
0	0	0	0	2161	0	828	0	0	0	0	0	0	0	0	0	101	0
0	0	0	0	0	0	0	0	0	0	0	0	0	0	0	0	0	0
0	0	0	0	0	0	2683	0	0	56	0	0	0	0	0	0	0	0
0	0	63	62	0	0	0	0	0	0	0	0	0	0	0	0	0	0
1106	1450	187	285	10941	0	0	764	0	0	67	46	77	0	0	0	1662	2041
0	0	0	0	0	0	0	0	0	0	0	0	0	0	66	0	0	0
0	0	0	0	0	0	0	0	0	0	0	0	0	0	0	0	0	0
0	0	0	0	0	0	0	0	0	0	0	0	0	0	0	0	0	0
0	0	0	0	0	0	0	0	206	0	0	0	0	0	0	0	0	0
0	0	0	0	0	0	0	0	0	0	0	0	0	0	0	0	0	0
0	0	0	0	0	0	2280	0	0	0	0	0	0	0	0	0	0	0
0	0	69	0	0	0	0	0	0	0	0	0	0	0	0	0	0	0
0	0	0	0	0	0	0	0	0	0	0	0	0	0	0	0	0	0
0	63	0	0	0	0	0	0	0	0	0	0	0	0	0	0	0	0
0	0	0	0	0	0	0	0	0	0	0	0	0	0	0	0	0	0
0	0	0	0	76	0	0	0	0	0	0	22189	0	0	0	0	0	61
0	0	0	0	0	0	0	0	0	0	0	0	0	0	0	0	0	0
0	0	0	0	0	0	0	0	0	0	0	47	0	0	0	0	112	0
0	0	0	57	0	0	0	0	0	0	0	0	0	0	0	0	0	0
0	0	0	0	0	0	0	0	0	0	0	0	0	0	0	0	0	0
0	0	0	0	15239	0	0	0	0	0	0	11564	0	0	0	0	197	0
0	0	0	0	0	0	0	0	0	0	0	0	0	0	0	0	0	0
0	0	0	0	0	0	0	0	0	0	0	0	0	0	0	0	0	0
0	0	0	0	0	0	0	0	0	0	0	0	0	0	0	361	0	0
0	0	0	66	0	0	0	0	0	0	0	0	0	0	0	0	0	0
0	0	0	0	0	0	0	0	0	0	0	0	0	0	0	0	0	0
0	2915	0	0	0	0	0	0	0	0	0	0	0	0	0	0	0	0
82	0	0	0	0	0	0	0	0	0	0	0	0	0	0	0	0	0
0	0	0	0	0	0	0	0	0	0	0	0	0	0	0	0	0	0
0	0	0	0	0	0	0	0	0	0	0	0	0	0	0	0	0	0
0	0	0	0	0	0	0	0	0	0	0	0	0	0	0	0	0	0
0	0	0	0	0	0	0	0	0	0	0	0	0	0	0	0	0	0
0	0	0	1357	390	0	0	0	0	0	0	0	150	357	230	0	1670	0
308	76	844	860	8455	7936	424	72851	2118	2150	4637	2717	802	1295	20379	4277	5929	2195
0	0	0	0	0	0	0	0	0	0	0	0	0	0	0	0	0	0
0	0	0	0	0	112	499	0	249	237	0	0	0	0	0	0	0	0
0	0	0	114	0	0	0	0	0	0	0	0	0	0	0	0	0	0
0	0	0	0	0	0	0	0	0	0	0	0	0	0	0	0	0	0
0	0	0	502	167	0	0	0	0	0	0	0	0	166	88	0	206	0
0	0	0	0	0	0	0	0	0	0	0	0	0	0	0	0	0	0
0	0	0	0	0	0	0	0	0	0	0	0	0	0	0	0	0	0
0	0	0	0	0	0	0	164	0	0	0	0	0	0	0	0	0	0
0	0	0	0	0	0	0	0	0	0	0	0	0	0	0	0	0	0
0	65	0	0	0	0	0	170	0	0	0	0	0	0	0	0	0	0
0	0	0	0	0	0	0	0	0	0	0	0	0	0	0	0	0	0
142	0	0	0	0	0	0	0	0	0	0	0	0	0	0	0	0	0
0	0	0	0	0	0	0	0	0	0	0	0	0	0	0	0	0	0
75	63	0	0	0	0	0	146	0	0	0	0	76	0	599	0	0	0
0	0	0	0	0	0	0	0	0	0	0	0	0	0	0	0	0	0
0	0	0	0	0	0	0	0	0	0	0	0	0	0	0	0	0	0
0	0	0	0	0	0	0	0	0	0	0	0	0	0	0	0	0	0
0	0	0	0	0	0	0	0	0	0	0	0	0	0	0	0	0	0
0	0	0	0	0	0	0	0	0	0	0	0	0	0	0	0	0	0
0	0	0	0	0	0	0	0	0	0	0	0	0	0	0	0	0	0
0	0	0	0	0	0	0	0	0	0	0	0	0	0	0	0	0	0
10562	59	110	3766	146	3476	1388	439	4750	74249	213	44	2656	268	755	133	0	251
0	0	0	0	0	0	0	0	0	0	0	0	0	0	0	0	0	0
0	72	553	115	167535	385	354	0	509	643	9216	20436	67150	599034	44451	3656	35296	1192
0	0	0	0	0	0	0	0	0	0	0	0	0	0	0	0	0	0
0	0	1396	976	86	0	0	0	0	0	0	0	0	0	0	0	0	0
0	0	0	0	0	0	0	0	0	0	0	0	0	0	0	0	0	0
0	0	0	0	0	0	0	0	0	0	0	0	0	0	0	0	0	0
0	0	0	0	0	0	0	0	0	0	0	0	0	0	0	0	0	0
0	0	0	0	0	0	0	0	0	0	0	0	0	99	0	0	0	0
0	0	0	0	0	0	0	0	0	0	0	0	0	628	0	0	189	453
0	786	0	0	0	0	1018	0	0	0	0	0	0	0	0	0	282	0
0	0	0	0	0	0	0	0	0	0	0	0	0	0	0	0	0	0
0	0	0	0	0	0	0	0	0	0	0	0	0	0	0	0	0	0
0	0	0	0	0	0	0	151	0	0	0	0	0	0	0	0	0	0
0	0	0	0	0	0	0	0	0	0	0	0	0	0	0	0	0	0
0	0	0	0	0	0	0	0	0	0	0	0	943	0	0	0	0	0
0	0	0	0	0	0	0	0	0	0	0	0	0	0	0	0	0	0
0	0	0	0	0	0	0	0	0	0	0	0	0	0	0	0	0	0
0	0	0	0	0	0	0	0	0	0	0	0	0	0	0	0	0	0
0	0	0	0	0	0	0	0	0	0	0	0	0	0	0	0	0	0
0	0	0	0	0	0	0	0	0	0	0	0	0	0	0	0	0	0
0	0	0	0	0	0	0	0	0	0	0	0	0	0	0	0	0	0
79546	357	679	322	8241	60978	82784	59970	65476	29202	1510	391	0	1515	31938	1005	2891	1533
0	0	0	0	0	29	27	0	69	0	0	0	0	0	0	0	0	0
0	0	0	0	0	0	0	0	0	0	0	0	0	0	0	0	0	0
0	0	0	0	0	0	0	0	0	0	0	0	0	0	0	0	0	0
0	0	0	0	0	0	0	0	0	0	0	0	0	0	0	0	0	0

0	0	0	0	0	0	0	0	0	0	0	0	0	0	0	0	0	0
0	0	0	0	0	0	0	0	0	0	0	0	0	0	0	0	0	0
0	0	0	0	0	0	0	0	0	0	0	0	0	0	0	0	0	0
0	0	0	0	0	0	0	0	0	0	0	0	0	0	0	0	0	0
0	0	0	0	0	0	0	0	0	0	0	0	0	0	0	0	0	0
0	0	0	0	0	0	0	0	0	0	0	0	0	0	0	0	0	0
0	0	0	0	0	0	0	0	0	0	0	0	0	0	0	0	0	0
0	0	0	0	0	0	0	0	0	0	0	0	0	0	0	0	0	0
0	0	0	0	2035	0	0	0	0	0	480	2837	232	252	1896	709	166	756
80	62	0	0	0	0	0	267	0	0	0	0	0	0	0	0	0	0
0	0	0	0	0	0	0	0	0	0	0	0	0	0	0	0	0	0
0	0	0	0	0	0	0	0	0	0	0	0	0	0	0	0	0	0
0	0	0	0	0	0	0	0	0	0	0	0	0	0	0	0	0	0
0	0	0	0	0	31	0	0	0	0	32043	46	154	0	79	114	191	42
0	0	0	0	0	0	0	0	0	0	0	0	0	0	0	0	0	0
0	0	0	0	0	0	0	0	0	0	0	0	0	0	0	0	0	0
0	0	0	0	0	0	0	0	0	0	0	0	0	0	0	0	0	0
0	0	0	0	0	0	0	0	0	0	0	0	0	0	0	0	0	0
0	0	0	18957	0	0	0	0	0	0	0	0	0	0	0	0	0	0
0	0	0	0	0	0	0	0	0	0	0	0	0	0	0	0	0	0
159	0	0	0	0	38	61	167	127	0	0	0	0	0	0	0	0	0
0	0	0	0	0	0	0	0	0	0	0	0	0	0	0	0	0	0
0	0	7806	0	0	0	0	0	0	0	0	0	0	0	0	0	0	0
0	0	0	0	0	0	0	0	0	0	0	0	0	0	0	0	0	0
0	0	0	0	166	0	0	0	0	0	0	132	16884	0	0	116	103	18240
0	0	0	0	0	40	66	0	73	0	0	0	0	0	0	0	0	0
0	0	0	0	0	0	0	0	0	0	0	0	0	0	0	0	0	0
0	0	0	0	0	0	0	0	0	0	0	0	0	0	0	0	0	0
0	0	0	0	77	0	0	0	0	37	20135	0	0	3483	58	1080	1894	0
0	0	0	0	0	0	0	0	0	0	0	0	0	0	0	0	0	0
0	0	0	0	0	0	0	0	0	0	0	0	0	0	0	0	0	0
0	0	179	61	0	84	20	451	123	0	0	0	0	0	492	0	0	236
0	0	0	0	0	0	0	0	0	0	0	0	0	0	1053	0	0	0
0	0	0	0	0	0	0	0	0	0	0	0	0	0	0	0	0	0
0	0	0	0	0	0	0	0	0	0	0	0	0	267	0	110	0	2246
0	0	0	0	0	0	0	0	0	0	0	0	0	0	0	0	0	0
0	0	0	0	0	0	0	153	0	0	10243	100	82	0	0	0	0	0
0	0	0	0	0	0	0	0	0	0	0	0	0	0	0	0	0	0
0	0	0	0	0	171	0	181	130	0	0	0	0	0	0	0	0	0
0	0	0	0	0	0	0	0	0	0	0	0	0	0	0	0	0	0
0	0	0	0	0	0	0	0	0	0	0	0	0	0	0	0	0	0
0	0	0	0	0	0	0	0	0	0	0	0	0	0	0	0	0	0
0	0	0	0	0	0	0	0	0	0	0	0	0	0	2467	0	0	0
0	0	0	0	0	0	0	0	0	0	0	0	0	0	0	0	0	0
0	0	0	0	0	1436	0	0	0	0	0	0	0	0	0	0	0	0
0	0	0	0	0	0	0	0	0	0	0	0	0	0	0	0	0	0
0	0	0	0	0	0	0	0	0	0	0	0	0	0	0	0	0	0
0	0	0	0	0	0	0	0	0	0	0	0	0	0	0	0	0	0
0	0	0	0	0	0	0	0	0	0	0	57	0	0	156	0	1666	0
0	0	341	184	0	0	0	0	0	0	0	0	0	66	77	0	277	0
0	0	0	0	0	0	0	0	0	0	0	0	0	0	0	0	0	0
0	0	0	0	0	0	0	0	0	0	0	0	0	0	0	0	0	0
0	0	0	0	0	0	0	0	0	0	0	0	0	0	0	0	0	0
0	0	0	0	0	0	0	0	0	0	0	0	0	0	0	0	0	0
0	0	0	0	0	0	0	0	0	0	0	0	0	0	0	0	0	0
0	0	0	0	0	0	26	155	0	42	0	46	0	0	0	0	0	4191
0	0	0	0	0	0	0	0	0	0	0	0	0	0	0	0	0	0
0	0	0	0	0	0	0	0	0	0	0	0	0	0	0	0	0	0
0	0	0	0	0	0	0	0	0	0	0	0	0	0	0	0	0	0
0	0	0	0	0	0	0	0	0	0	0	0	0	423	0	0	0	0
0	0	0	0	0	0	0	0	0	0	0	0	0	0	0	0	0	0
0	0	0	0	0	0	0	0	0	0	0	0	531	0	0	0	0	0
0	0	0	0	0	0	0	0	0	0	0	54	0	4432	0	583	0	1125
0	0	0	0	0	0	0	0	0	0	0	0	0	0	0	0	0	0
0	0	0	0	0	0	0	0	0	0	0	0	0	0	0	0	0	0
0	0	0	0	0	0	0	0	53	27	0	0	0	0	0	0	0	0
0	0	0	0	81	37	23	0	0	0	0	9270	0	83	0	0	0	0
0	0	0	0	0	0	0	0	0	0	0	0	0	0	0	0	0	0
0	0	0	0	0	0	0	0	0	0	0	0	0	0	0	222	0	0
0	0	0	0	0	81	186	0	66	114	0	0	0	0	0	0	0	0
0	0	0	0	0	0	0	0	0	0	0	0	0	0	0	0	0	0
0	0	0	0	0	0	0	0	0	0	0	0	0	0	0	0	0	0
0	0	0	0	0	0	0	0	0	0	0	0	0	0	0	0	0	0
0	0	0	0	0	0	0	0	0	0	2101	0	0	0	0	0	0	0
0	0	0	0	0	0	0	0	0	0	0	0	0	0	0	0	0	0
0	0	0	0	0	0	0	0	0	0	0	0	0	0	0	0	0	0
4936	68	0	0	234	2149	629	1397	4485	2858	430	0	0	175	737	374	208	62
0	0	0	0	0	0	0	0	0	0	0	0	0	0	0	0	0	0
0	0	0	0	0	0	0	0	0	0	0	0	0	0	0	0	0	0
0	0	0	0	0	0	0	0	0	0	0	0	0	0	0	0	0	0
0	0	0	0	0	0	0	0	0	0	0	0	0	0	0	0	0	0
0	0	0	61	0	0	0	0	0	0	0	0	0	0	0	0	404	0
0	0	0	0	0	0	0	0	0	0	0	0	0	0	0	0	0	0
0	0	0	0	0	0	0	0	0	0	0	0	1921	0	0	0	0	0
0	0	0	0	0	0	0	0	0	0	0	0	0	0	0	0	0	0
0	0	0	8018	12465	0	0	0	0	0	5414	46	76	0	75	0	0	1447
0	0	0	0	0	0	0	0	0	0	0	0	0	0	1481	0	0	0

0	0	0	0	0	0	0	0	0	0	0	0	0	0	0	0	1035	0
0	0	0	0	0	0	0	0	0	0	0	0	0	0	0	0	0	0
0	0	0	0	0	0	0	0	0	0	0	0	0	0	0	0	0	0
0	0	54	19629	0	0	0	0	0	0	0	0	0	0	0	0	0	0
0	0	0	0	0	0	0	0	0	0	0	0	0	0	0	0	0	0
0	0	0	0	0	0	55	0	0	0	0	0	0	0	0	0	0	0
0	0	0	0	0	0	0	0	0	0	0	0	0	0	0	0	0	0
0	0	0	0	0	0	0	0	0	0	0	0	0	0	0	0	0	0
0	0	0	0	0	0	0	0	0	0	0	0	0	0	0	0	0	0
0	0	0	0	0	0	0	0	0	0	0	0	0	0	0	0	0	0
0	0	0	0	0	0	0	0	0	0	0	0	0	0	0	0	0	0
0	0	0	0	0	0	0	0	0	0	0	0	0	0	0	0	0	0
0	0	0	0	0	0	0	0	0	0	0	0	0	0	0	0	0	0
0	0	0	0	0	0	0	0	0	0	66	0	0	0	0	0	0	0
0	0	0	0	6826	0	0	0	0	0	74	0	76	0	0	0	0	0
0	0	0	0	0	0	0	0	0	0	0	0	0	0	0	0	0	0
0	0	54	0	0	0	0	453	0	0	0	0	0	0	0	0	0	0
0	0	0	0	0	0	0	0	0	165	0	0	0	0	0	0	0	0
0	0	0	0	0	0	0	0	0	0	0	0	0	0	0	0	0	0
0	0	0	0	0	0	0	0	0	0	0	0	0	0	0	0	0	0
0	0	0	0	0	0	0	0	0	0	0	0	0	0	0	0	0	0
0	0	0	0	0	0	0	0	0	0	0	0	0	0	0	0	0	0
0	0	0	0	0	0	0	0	0	0	0	0	0	0	0	0	0	0
0	0	0	0	0	0	0	0	0	0	0	0	0	0	0	0	0	0
0	0	0	0	0	0	30	0	0	0	0	0	0	0	0	0	0	0
0	0	0	0	0	0	0	0	0	0	0	0	0	0	0	0	0	0
0	0	0	0	0	0	0	0	0	0	0	0	0	0	0	0	0	0
0	0	0	0	0	0	0	0	0	0	0	0	0	0	0	0	0	0
0	0	0	0	0	0	0	0	0	0	0	0	0	0	0	0	0	0
0	0	0	0	0	0	0	0	0	0	0	0	0	0	0	0	0	0
0	0	0	0	0	0	0	0	0	0	0	0	0	0	0	0	0	0
1200	0	0	0	0	200	34	0	220	813	0	0	0	0	0	0	0	0
2857	2219	697876	113319	13993	15319	2028	64944	4985	17523	17487	5458	6992	7254	56872	2829	7976	4220
0	0	0	0	0	0	0	0	0	0	0	0	0	0	0	0	0	0
0	58	0	0	0	0	0	144	0	0	0	0	0	0	0	0	0	0
0	0	0	0	0	0	0	0	0	0	0	0	0	0	0	0	0	0
0	0	0	0	0	0	0	0	70	0	0	0	0	0	314	0	0	0
0	0	0	0	0	0	0	0	0	0	0	0	0	0	0	0	0	0
0	0	0	0	0	0	0	0	0	0	0	0	0	0	0	0	0	0
0	0	0	0	0	0	0	0	0	0	0	0	0	0	0	0	0	0
333	0	2654	0	0	111	74	0	187	431	0	0	0	90	0	0	0	0
0	0	0	0	0	0	0	0	0	0	0	0	0	0	0	0	0	0
0	0	0	0	0	0	0	0	0	0	0	0	0	0	0	0	0	0
0	0	0	0	0	0	0	0	0	0	0	0	0	0	0	0	0	0
0	0	0	0	0	33	67	0	146	90	0	0	0	259	0	0	0	0
0	0	0	0	0	0	0	0	0	0	0	0	0	0	0	0	0	0
0	0	0	0	0	0	0	0	0	0	0	0	0	0	0	0	0	0
0	0	0	0	0	0	0	0	0	0	80	57	142	264	3715	0	66387	2801
26654	60	203	59	974	34847	15789	21000	31606	7173	3215	41	78	276	18113	144	2189	1167

COVID-19-019	COVID-19-025	COVID-19-020	COVID-19-033	COVID-19-022	COVID-19-023	COVID-19-021	Control 095	Control 084	Control 085	Control 086	Control 087	Control 088	Control 089	Control 091	Control 093	Control 094	Control 096
928720	902109	358665	975862	910653	985829	988193	427347	994577	923043	575979	638896	869044	815686	672183	18057	688810	684726
41848	92640	605280	12165	55577	9508	2876	546597	3121	47008	355465	342134	6867	57273	218121	980746	237912	291654
0	0	0	0	0	193	0	0	0	0	0	0	0	77	0	0	0	121
26699	4419	36011	11973	22778	3854	8837	18473	1475	17083	67672	17857	123554	121015	98958	885	73278	16442
2733	832	44	0	10992	616	94	7583	827	10175	884	1113	535	5949	10738	312	0	7057
0	0	0	0	0	0	0	0	0	0	0	0	0	0	0	0	0	0
0	0	0	0	0	0	0	0	0	2691	0	0	0	0	0	0	0	0
COVID-19-019	COVID-19-025	COVID-19-020	COVID-19-033	COVID-19-022	COVID-19-023	COVID-19-021	Control 095	Control 084	Control 085	Control 086	Control 087	Control 088	Control 089	Control 091	Control 093	Control 094	Control 096
651	0	0	0	0	0	0	0	0	37	0	0	0	0	0	0	0	0
0	0	0	0	0	0	0	0	0	0	0	0	43	89	0	0	0	0
640546	153216	4967	23637	77463	75283	6886	82229	7752	294114	18575	20713	4637	3697	508212	5504	9416	37060
0	0	0	0	0	0	0	0	0	0	0	0	0	0	0	0	0	0
0	0	0	0	0	0	0	0	0	0	0	0	0	0	0	72	0	0
0	0	0	0	0	0	0	0	0	0	0	0	0	0	0	0	0	0
0	0	0	0	0	0	0	0	0	0	0	0	0	0	0	0	0	0
0	0	0	0	0	0	0	0	0	0	0	0	0	0	0	0	0	0
0	0	0	0	0	0	0	38	0	0	0	0	0	74	63010	0	0	166
0	0	0	0	0	0	0	0	0	0	0	70	0	0	0	0	0	0
0	0	0	0	0	0	0	0	0	0	0	0	0	0	0	0	0	0
218	8475	55	739	0	3103	898	265	56	2036	173	0	44	394	278	536	177	275
0	0	0	0	0	0	0	0	0	0	0	0	0	0	0	0	0	0
0	0	0	0	0	0	0	0	0	0	0	0	0	0	0	0	0	0
0	4334	0	0	0	0	0	0	0	0	0	0	0	0	0	0	0	0
13154	2657	56	309	170	2270	0	263	137	5272	301	255	49	48	6581	255	0	1102
0	0	0	0	0	0	0	0	0	0	0	0	0	0	0	0	0	0
0	0	0	0	0	0	0	39	0	0	0	0	0	0	70	0	0	0
0	0	0	0	0	0	0	0	0	0	0	0	0	0	0	0	0	0
0	3477	0	218	0	0	0	42343	0	2983	0	0	0	0	283	0	54	0
0	0	0	0	0	0	0	0	0	0	0	0	0	0	0	0	0	0
0	0	0	0	0	0	0	0	0	0	0	0	0	0	0	0	0	0
0	0	0	0	0	0	0	0	0	0	0	0	0	0	0	0	0	0
0	0	0	0	0	0	302	0	0	0	0	0	0	0	0	0	0	0
0	0	0	300	0	0	0	405	973659	1282	970	624	1121	401	402	814	316	950
0	0	0	0	0	0	0	0	0	0	0	0	0	0	0	0	0	0
0	201	0	1408	190	6251	281	0	41	587	0	0	0	0	0	0	0	1071
0	0	0	0	0	0	0	0	0	0	0	0	0	0	0	0	0	0
0	0	0	0	0	0	0	0	0	0	0	0	0	34	0	65	0	0
11911	13244	85712	281163	121515	74000	227427	1864	2632	208304	901	10721	57606	8547	13998	1522	138941	149339
0	0	0	0	0	0	0	0	0	0	0	0	0	0	0	0	0	0
0	0	0	0	0	0	0	0	0	0	0	0	0	0	0	0	0	0
0	0	0	0	0	0	0	35	0	0	0	0	0	149	72	60	0	0
59	0	0	0	0	3663	0	0	0	0	0	0	0	0	0	0	0	0
0	0	0	0	0	0	0	0	0	0	0	0	0	0	0	0	0	0
0	0	0	0	0	0	0	0	0	0	0	0	0	0	0	0	0	0
0	0	0	0	0	0	0	0	0	0	0	0	0	0	0	0	0	0
0	0	0	0	0	0	0	0	0	0	0	0	0	0	0	0	0	0
0	0	0	0	0	0	0	0	0	0	0	0	0	0	0	0	0	0
0	0	0	0	0	0	0	0	0	181	0	0	0	70	0	0	0	0
0	404	0	0	0	0	0	0	0	156	42	33	0	0	0	0	0	167
0	0	0	0	0	0	0	44	0	0	0	0	0	15250	0	0	0	0
0	0	0	0	0	0	0	0	0	0	0	0	0	0	0	0	0	0
0	0	0	0	0	0	0	0	0	0	0	0	0	0	0	0	0	0
0	0	0	0	0	0	0	0	0	0	0	0	0	0	0	0	0	0
0	0	0	0	0	0	0	0	48	114	113	45	62	0	0	0	0	10069
0	0	0	0	0	0	0	0	0	0	0	0	0	0	0	0	0	0
1144	2415	10239	433238	193348	90510	730166	262	615	271612	637	492	543	146	150	276	260	266569
0	0	0	0	0	0	0	0	54	0	43	35	0	0	0	0	0	0
0	214	0	104	8754	601	0	38	0	1672	0	0	0	66	78	0	0	0
0	0	0	0	0	0	0	0	0	0	0	0	0	0	0	0	0	0
0	0	0	0	0	0	0	0	0	0	0	0	0	0	0	0	0	0
0	0	0	0	0	0	0	0	145	0	0	0	0	0	0	0	0	0
0	0	0	0	0	0	0	0	0	0	0	0	0	0	0	0	0	0
0	0	0	0	0	0	0	0	0	0	0	0	0	0	0	0	0	0
0	0	0	0	0	0	0	0	0	0	0	0	0	0	0	0	0	0
0	0	0	0	0	0	0	0	0	0	0	0	0	0	0	0	0	0
1047	0	0	0	0	0	0	0	0	3638	87	0	0	0	184	0	0	0
0	0	0	0	0	0	0	0	0	0	0	0	0	0	0	0	0	0
0	0	0	0	0	0	0	0	0	0	0	0	0	78	0	53	0	0
2717	260053	1429	1493	116990	1410	1351	35884	0	136	0	0	101	450	291	490	34859	232
0	0	0	0	0	0	0	0	0	0	0	0	0	0	0	0	0	0
0	0	0	0	0	0	0	0	0	0	0	0	0	0	0	0	0	0
0	0	0	0	0	0	0	0	0	0	0	0	0	0	0	0	0	0
0	578	0	0	0	0	0	0	0	0	0	0	0	0	0	0	0	0
0	0	0	0	0	0	0	0	0	0	78	36	62	0	0	0	0	0
0	0	0	0	0	0	0	0	46	0	0	0	0	0	74	0	0	0
0	0	0	0	0	0	0	0	0	0	0	0	0	0	0	0	0	0
0	0	0	0	0	0	0	0	0	0	0	0	0	0	0	0	0	0

0	0	0	0	0	0	0	0	0	0	0	0	0	0	0	0	0	0
0	0	0	0	0	582	0	0	0	0	0	0	0	0	0	0	0	0
0	0	0	0	0	0	0	0	0	0	0	0	0	0	0	0	0	0
0	0	0	0	0	0	0	0	0	0	0	0	0	0	0	0	0	0
0	0	0	0	0	0	0	0	0	0	0	0	0	0	0	0	0	0
0	0	0	0	0	0	0	0	0	0	0	0	0	0	0	0	0	0
0	13638	51	4853	0	522871	190	35	44	528	0	0	0	37	74	0	10836	35765
0	0	0	0	0	0	0	0	0	0	0	0	0	0	0	0	0	0
0	0	0	0	0	193	0	0	0	0	0	0	0	77	0	0	0	121
0	0	0	0	0	0	0	0	0	0	0	0	0	0	0	0	0	0
0	0	0	1556	154	0	0	0	0	41	94	0	0	0	0	0	0	0
0	0	0	0	0	0	0	0	0	0	0	0	0	0	0	0	0	0
304	81306	170488	2892	16624	5354	1025	186049	974	1296	608	111401	531	1319	1364	1591	62941	34179
0	0	0	0	0	0	0	0	0	0	0	0	0	0	0	0	0	0
0	0	0	0	0	0	0	0	0	0	0	0	0	0	0	0	0	0
0	0	0	0	0	0	0	0	0	0	0	0	0	0	0	0	0	0
0	0	0	0	0	0	0	0	0	0	0	0	0	0	0	0	0	0
0	0	0	0	0	0	0	0	0	0	0	0	0	0	0	0	0	0
0	0	0	0	0	0	0	0	0	0	0	0	0	0	0	0	0	0
0	0	15779	0	0	0	0	0	0	0	0	0	0	0	0	0	0	0
0	0	0	101	0	0	0	0	0	0	0	0	0	0	0	0	0	0
0	0	0	0	0	0	0	0	0	0	0	0	0	0	0	0	0	0
0	0	0	0	0	0	0	24	0	0	35	27	46	29	222	47	0	54
0	0	0	0	10188	0	0	0	0	38	0	34	0	0	0	0	0	0
0	0	0	0	0	0	0	0	0	165	0	0	0	0	0	0	0	0
0	0	0	0	0	0	0	0	0	0	0	0	0	0	0	69	0	0
0	0	0	0	0	0	0	0	0	0	0	0	0	0	0	0	0	0
57	196	0	0	0	0	0	74673	803	15176	1342	172886	537	38481	154654	7500	131922	130266
0	0	0	0	0	0	0	0	0	0	0	0	0	0	0	0	0	0
0	0	0	0	0	0	0	0	0	0	0	0	0	0	0	0	0	0
432	77168	159	0	0	401	83	0	0	84	133	0	101	0	0	0	0	0
0	0	0	0	0	0	0	0	0	0	0	0	0	0	0	0	0	0
0	0	0	0	0	0	0	0	0	0	0	0	0	0	0	0	0	0
0	0	0	0	0	0	0	0	0	0	0	0	0	0	0	0	0	0
0	0	0	0	0	208	99	82	0	0	0	0	0	90	0	62	0	47
0	0	0	0	0	0	0	0	0	0	0	0	0	0	0	66	0	0
0	0	0	0	0	0	0	0	0	0	0	0	0	0	0	0	0	0
0	0	0	0	0	0	0	0	0	0	0	0	0	0	0	0	0	5975
75	2867	0	0	0	0	0	0	0	0	0	0	0	0	66	124	109	50
0	0	0	0	0	0	0	0	0	0	0	0	0	0	0	0	0	47
0	0	0	0	0	0	0	0	0	45	0	0	0	0	0	0	0	0
0	0	0	0	0	199	0	0	0	0	0	0	0	0	0	0	0	0
0	0	0	0	0	0	0	0	0	0	0	0	0	0	0	0	0	0
0	0	0	0	0	0	0	188	0	0	0	0	0	111	136	215	139	14944
0	611	0	0	0	0	0	0	0	92	76	35	49	0	0	0	0	0
0	0	0	231	0	0	0	0	0	0	0	0	0	0	0	0	0	0
0	0	0	0	0	0	0	0	0	0	0	0	0	0	0	0	0	0
0	0	0	0	0	0	0	162	0	0	0	0	0	124	59	0	0	58
0	0	0	0	0	0	0	0	0	41	0	0	50	0	0	61	0	0
0	21209	0	102	0	3713	0	0	0	0	0	0	0	0	0	0	0	0
2733	832	44	0	10992	616	94	7583	827	10175	884	1113	535	5876	10629	184	0	7005
0	0	0	0	0	0	0	0	0	0	0	0	0	0	0	0	0	0
0	0	0	0	0	0	0	0	0	0	0	0	0	0	0	0	0	0
0	0	0	0	0	0	0	0	0	0	0	0	0	0	0	0	0	0
0	0	0	0	0	0	0	0	0	0	0	0	0	0	0	0	0	0
0	795	0	0	0	0	0	0	0	0	0	0	0	0	0	0	0	0
115	0	0	0	0	0	0	0	0	0	0	0	0	0	0	0	0	0
0	0	0	0	0	0	0	0	0	0	0	0	0	0	0	0	0	0
0	0	0	0	0	0	0	0	0	0	0	0	0	0	0	0	0	0
0	0	0	0	0	0	0	0	0	0	0	0	0	0	0	0	0	0
0	0	0	0	0	0	0	32085	0	0	0	0	0	0	62	0	0	132
0	0	0	0	0	0	0	0	0	0	0	0	0	0	0	0	0	0
7788	177	64	913	176	0	489	0	0	117	39	73	0	0	0	0	0	66
0	0	0	0	0	0	0	0	0	0	0	0	0	0	0	0	0	0
0	0	41	0	499	0	0	0	0	0	0	0	0	0	0	0	0	0
0	0	0	0	0	0	0	0	0	0	0	0	0	0	0	0	0	0
0	0	0	0	0	0	0	0	0	0	0	0	0	0	0	0	0	0
0	0	0	0	0	0	0	0	0	0	0	0	0	0	0	0	0	0
0	0	0	0	0	0	0	0	0	0	0	0	45	0	0	0	0	0
0	0	0	0	0	0	0	0	0	0	0	0	0	0	0	0	0	0
0	0	0	0	0	0	0	0	0	0	0	0	0	0	0	0	0	0
0	0	0	0	0	0	0	0	0	0	0	0	0	0	0	0	0	0
0	0	0	0	0	0	0	0	0	0	0	0	0	0	0	0	0	0
0	0	0	0	0	0	0	0	0	0	0	0	0	0	0	0	0	0
7634	2033	0	284	0	1633	96	5020	0	9440	81	157	105	0	11826	0	0	272
19516	986	184	592	183	627	191	182	207	8839	1958	176	138	161	7785	297	273	2490
0	197	0	318	164	0	0	109	0	0	0	0	0	129	63	178	37667	0
0	0	0	0	0	0	0	0	0	0	0	0	0	0	0	0	0	0
0	0	0	0	0	0	0	0	0	0	0	0	0	0	0	0	0	0
0	0	0	0	0	0	0	48	0	0	0	0	0	116	0	193	239	47
0	0	0	0	0	0	0	0	0	0	0	0	0	0	0	0	0	0
0	0	0	0	0	0	0	0	0	0	0	0	0	0	0	0	0	0
0	0	0	0	0	0	0	0	0	0	0	0	0	0	0	0	0	0
18911	4242	35947	11060	22602	3854	8348	18473	1475	16966	67633	17784	123554	121015	98958	885	73278	16376
64	1439	0	225	177	4715	194	0	81	3325	0	44	0	0	0	0	0	0
0	0	0	102	0	0	109	0	0	271	0	0	0	0	0	0	0	623
0	0	0	0	0	0	0	0	0	0	0	0	0	0	0	0	0	0

0	0	0	0	0	0	0	0	0	0	0	0	0	0	0	0	0	0
0	0	0	96	0	0	0	0	0	82	0	0	42	35	198	349	0	0
53	0	0	0	0	0	0	0	0	0	0	0	0	0	0	0	0	0
0	0	0	0	0	0	0	0	0	35	0	35	0	0	0	0	0	0
0	0	0	0	0	0	0	0	0	0	0	0	0	0	0	0	0	0
0	0	0	0	0	0	0	0	0	0	0	0	0	0	0	0	0	0
0	0	0	0	0	0	0	0	0	0	0	0	0	0	0	0	0	0
0	6030	0	2156	0	19221	0	0	0	0	0	0	0	0	0	0	0	0
59	0	0	0	0	0	0	33	0	0	0	0	0	0	0	0	748	0
0	0	0	0	0	0	0	0	0	0	0	0	0	0	0	0	0	0
0	0	0	0	0	0	0	0	0	874	0	0	0	0	0	0	0	0
0	0	0	0	0	0	0	0	0	0	0	0	0	30	0	0	0	52
0	0	0	109	0	0	0	0	0	0	0	0	0	0	0	0	0	0
0	0	0	0	0	0	0	0	0	0	0	0	0	0	0	0	0	0
0	0	0	0	0	0	0	0	0	0	0	0	0	0	0	0	0	0
0	0	0	0	0	0	0	0	0	0	0	0	0	0	0	0	0	0
0	0	0	0	0	0	0	0	0	0	0	0	0	0	0	0	0	0
0	0	0	0	0	0	0	0	0	0	0	0	0	0	0	0	0	0
0	0	0	0	0	0	0	0	0	0	0	0	0	0	0	0	0	0
0	0	0	0	0	0	0	0	0	0	0	0	0	0	0	0	0	0
0	2033	0	220	0	992	0	0	0	0	0	0	0	88	0	126	0	53
0	0	0	0	0	0	0	0	0	0	0	0	0	0	0	0	0	0
0	0	0	0	0	0	0	0	0	0	0	0	0	0	0	0	0	0
853	0	0	0	0	0	0	39	0	0	48	85	314	178	0	0	0	76
0	0	0	0	0	0	0	0	0	0	0	0	0	0	0	0	0	0
0	0	0	0	0	0	0	0	0	0	0	0	0	0	0	0	0	0
0	0	0	206	0	759	0	0	0	0	0	0	0	0	0	0	0	0
0	0	0	0	0	0	0	0	0	0	0	0	0	0	0	0	0	0
0	0	0	0	0	0	0	0	0	0	0	0	0	0	0	0	0	0
0	0	0	0	0	0	0	0	0	0	0	0	0	0	0	0	0	0
0	0	0	0	0	0	0	0	0	0	0	0	0	0	0	0	0	0
0	0	0	0	0	0	0	0	0	0	0	0	0	0	0	0	0	0
0	1983	0	0	0	0	87	0	0	0	0	0	0	0	0	0	0	0
0	0	0	0	0	0	0	0	0	0	0	0	0	0	0	0	0	0
0	0	0	0	0	0	0	0	0	0	0	0	0	0	0	0	0	0
54	1839	0	1771	0	629	0	0	0	81	51814	625	0	301	0	0	0	0
0	0	0	0	0	0	0	0	0	0	0	0	0	0	0	0	0	0
0	0	0	0	0	0	0	0	0	0	0	0	0	0	0	0	0	0
0	0	0	0	0	0	0	0	0	0	0	22836	0	0	0	0	0	0
0	0	0	0	0	0	0	0	0	0	0	0	0	0	0	0	0	0
0	0	0	0	0	0	0	0	0	0	0	0	0	0	0	0	0	0
0	0	0	0	0	0	0	0	0	0	0	0	0	0	0	0	0	0
0	0	0	0	0	0	0	0	0	0	0	0	0	0	0	0	0	0
0	0	0	0	12827	0	0	0	0	0	0	0	0	0	0	51	0	0
0	0	0	0	0	191	0	0	0	0	0	0	0	0	0	0	0	0
0	0	0	0	171	0	0	0	0	0	0	0	0	0	0	0	0	0
0	0	0	0	0	0	0	0	0	0	0	0	0	0	0	0	0	0
0	0	0	0	0	0	0	0	0	0	0	0	0	0	0	0	0	0
106	0	0	198	0	0	0	0	0	2737	0	4685	0	0	62	0	0	6421
0	0	0	0	0	0	0	0	0	36	0	0	0	0	0	0	0	0
0	0	0	0	0	0	0	0	0	0	0	0	0	0	0	0	0	0
0	0	0	0	0	0	0	0	0	0	0	0	0	0	0	0	0	0
0	0	0	0	0	0	0	0	0	0	0	0	0	0	0	0	0	0
0	0	0	0	0	0	0	42	0	0	0	0	0	0	0	58	0	0
0	0	0	0	0	0	0	0	0	0	0	0	0	0	0	0	0	0
0	0	0	0	0	0	0	0	0	0	0	0	0	0	0	0	0	0
0	0	0	0	0	0	0	0	0	0	0	0	0	0	0	0	0	0
0	0	0	0	0	1431	0	0	0	0	0	0	0	0	0	0	0	0
0	0	0	0	0	0	0	0	0	5843	0	0	0	0	0	0	0	0
0	0	0	0	0	0	0	0	0	0	0	0	0	0	0	0	0	0
0	0	0	0	0	0	0	0	0	0	0	0	0	44	0	160	0	62
0	0	0	0	0	0	0	0	0	0	0	0	0	0	0	0	0	0
0	0	0	0	0	0	0	0	0	0	0	0	0	43	0	0	0	0
0	0	0	0	0	0	0	78	0	0	0	0	0	0	53080	242	0	111
0	0	0	0	0	0	0	0	0	0	0	0	0	0	0	0	0	0
0	2061	0	0	0	0	0	0	0	0	0	0	0	0	0	0	0	0
0	0	0	0	0	0	0	0	0	0	0	0	0	0	0	0	0	0
0	0	0	0	0	0	0	0	0	0	0	0	0	0	0	0	0	0
0	0	0	0	0	0	0	0	0	0	0	0	0	0	0	0	0	0
0	0	0	0	0	0	0	0	0	0	0	0	0	0	0	0	0	0
0	0	0	0	0	0	0	0	0	0	0	0	0	0	0	0	0	0
0	0	0	0	0	0	0	0	0	0	0	0	0	0	0	0	0	0
0	0	0	0	0	0	0	0	48	49	53	0	50	0	0	0	0	0
126	409	127732	10500	855	2935	1038	137767	949	7091	351299	104270	828	722	692	380	25833	146257
0	0	0	0	0	0	0	0	0	0	0	0	0	0	0	0	0	0
0	0	0	0	0	0	0	0	0	0	0	0	0	0	0	0	0	0
0	400	0	0	0	0	0	0	0	0	0	0	0	0	0	0	0	0
0	0	0	0	0	0	0	0	0	572	0	0	0	0	0	0	0	1374
414	7331	0	0	0	0	286	750	223	187	41	62	0	13880	1108	1165	47298	4780
0	0	0	0	0	0	0	0	0	228	0	0	0	0	0	0	0	0
0	0	0	0	0	0	0	0	0	0	0	0	0	0	0	0	0	0
0	0	0	0	0	0	0	0	0	0	0	0	0	0	0	0	0	0
0	0	0	0	0	0	0	0	0	0	0	0	0	0	0	0	0	0
0	0	0	0	0	0	0	0	0	0	0	0	0	43	109	56	0	0
0	0	0	0	0	996	0	0	0	0	0	0	0	0	0	0	0	0
0	618	0	0	0	398	164	0	0	0	0	0	0	72	0	0	7644	0
0	1968	0	0	0	0	0	245470	0	0	0	0	32	345	545	670	550	488

0	0	0	0	0	0	0	0	0	0	0	0	0	0	0	0	0	0
0	0	0	0	0	0	0	0	0	0	0	0	0	0	0	0	0	0
0	0	0	0	0	0	0	0	0	0	0	0	0	0	0	0	0	0
0	0	0	0	0	0	0	0	0	0	0	0	0	0	0	0	0	0
0	0	0	0	0	0	0	0	0	0	0	0	0	0	0	0	0	0
0	0	0	0	0	0	0	0	0	0	0	0	0	0	0	0	0	0
0	0	0	0	0	0	0	0	0	0	0	0	0	0	0	0	0	0
0	0	0	111	0	0	0	0	0	0	0	0	0	0	0	0	0	0
0	0	0	0	0	0	0	0	0	0	0	0	0	0	0	0	0	0
0	0	0	0	0	0	0	0	0	0	0	0	0	0	0	0	0	0
0	0	0	0	0	0	0	0	0	0	0	0	0	0	0	0	0	0
0	0	0	0	0	0	0	0	0	0	0	0	0	0	0	0	0	0
0	3932	1010	0	2567	0	1026	0	51	38	47	0	0	0	0	0	0	0
0	1229	50	0	0	0	0	70	0	0	0	0	49	81	52	0	0	113
0	0	0	0	0	0	0	0	0	4350	44	35	0	0	57	0	0	0
0	618	50	0	288972	0	261	0	0	37	0	0	0	0	0	0	0	0
0	0	0	0	0	0	0	0	0	0	0	0	0	0	0	0	0	0
0	0	0	0	0	0	0	0	0	0	0	0	0	0	0	0	0	0
0	0	0	0	0	0	0	0	0	0	0	0	0	0	0	0	0	0
0	0	0	0	0	0	0	0	0	0	0	0	0	0	0	0	0	0
0	0	0	0	0	0	0	0	0	0	0	0	0	0	0	0	0	0
0	0	0	0	0	1596	0	0	0	0	0	0	0	0	0	0	0	0
60	0	0	0	0	0	0	48	46	321	42	0	51	0	125	0	870	45
0	0	0	0	0	0	0	0	0	0	0	0	0	0	0	0	0	0
0	0	0	0	0	0	0	0	0	0	0	0	0	0	0	0	0	0
0	193	64626	0	180	0	90	0	0	0	0	0	0	0	0	0	0	0
0	0	0	0	0	0	0	0	0	0	0	0	0	0	0	0	0	0
8883	1417	59	199	328	1874	0	30	941	3997	1197	445	3860	112	5236	189	61	704
0	0	0	0	0	0	0	0	0	0	0	0	0	0	0	0	0	0
0	0	0	0	0	0	0	0	0	0	0	0	0	0	0	0	0	0
0	0	0	0	0	0	0	0	0	0	0	0	0	0	0	0	0	0
0	0	0	0	0	0	0	0	0	0	0	0	0	0	0	0	0	0
0	0	0	0	0	0	0	0	0	0	0	0	0	0	0	0	0	0
0	0	0	0	0	0	0	0	42	86	45	23986	0	0	0	0	0	0
1223	1399	61	8148	204	1002	8024	0	0	2264	0	28	0	35	0	0	0	825
0	0	0	0	0	0	0	0	0	0	0	0	0	0	0	0	0	0
0	0	0	0	0	0	0	0	0	0	0	0	0	0	0	0	0	0
65	0	0	0	0	0	0	0	0	0	0	0	0	0	0	0	0	0
68	1254	52	0	0	212	0	13372	0	80	46	11442	0	76	0	0	224	0
0	0	0	0	0	0	0	0	0	0	0	0	0	0	0	0	0	0
0	0	0	0	0	0	117	0	54	0	30	69	0	0	0	0	0	0
0	0	0	0	0	0	0	0	0	76	40	32124	93	0	0	0	0	0
226	0	0	0	0	0	0	0	0	37	0	0	0	0	0	0	0	0
0	0	0	0	0	0	0	0	0	0	0	0	0	0	0	0	0	0
0	0	0	0	0	0	0	0	0	0	0	0	0	0	0	0	0	0
0	396	0	206	0	0	0	0	0	0	0	0	0	0	0	0	0	0
0	0	0	0	0	0	0	0	0	0	0	0	0	0	0	0	0	0
0	0	0	0	0	0	0	0	0	0	0	0	0	0	0	0	0	0
0	0	0	0	0	0	0	0	0	0	0	0	0	0	0	0	0	0
0	0	0	0	0	0	0	0	0	0	0	0	0	0	0	0	0	0
0	0	0	0	0	0	0	0	0	93	0	0	0	0	0	0	0	0
0	0	0	0	0	0	0	0	0	0	0	0	0	0	0	0	0	0
0	0	0	0	0	0	0	0	0	0	0	0	0	0	0	0	0	0
184	0	0	0	0	0	0	0	0	0	0	0	0	0	0	0	0	0
0	0	0	0	0	0	0	0	0	0	0	0	0	0	0	0	0	0
0	0	0	0	0	0	0	0	0	0	0	0	0	0	0	0	0	0
0	0	0	0	0	0	0	0	0	0	0	0	0	0	0	0	0	0
0	0	0	0	0	0	0	0	0	0	0	0	0	0	58	0	0	0
0	0	0	0	0	0	0	0	0	52	0	0	0	0	0	0	0	0
0	0	0	0	0	0	0	0	0	0	0	0	0	0	0	0	0	0
0	23324	0	306	0	4986	0	0	0	0	0	0	0	0	0	69	0	0
0	0	0	0	0	0	0	0	149	0	0	0	0	0	0	0	0	0
0	0	0	0	0	0	0	0	0	0	0	0	0	0	0	0	0	0
0	1769	284553	0	1500	3153	662	0	152	84	28	0	79	0	0	70	53	0
0	0	0	0	0	0	0	0	0	0	0	0	0	0	0	0	0	0
0	0	0	0	0	0	0	0	0	0	0	0	0	0	0	0	0	0
0	0	0	0	0	0	0	0	0	0	0	0	0	0	0	0	0	0
0	0	0	0	0	0	0	0	0	0	0	0	0	0	0	0	0	0
0	0	0	0	0	0	0	0	0	0	0	0	0	0	0	0	0	0
0	0	0	0	0	0	0	0	0	0	0	0	0	38	0	59	0	120
0	0	0	0	0	0	0	0	0	0	0	0	0	0	0	0	0	0
0	0	0	0	0	0	0	0	0	0	0	0	0	0	0	0	0	0
0	0	0	0	0	209	0	0	0	0	0	0	0	0	0	0	0	0
0	0	0	0	0	0	0	0	0	0	0	0	0	0	0	0	0	0
0	0	0	0	0	0	0	0	0	0	0	0	0	0	0	0	0	0
0	0	0	0	0	0	0	0	0	0	0	0	0	0	0	0	0	0
0	0	0	90	0	0	0	0	0	0	0	0	0	0	0	0	0	0
0	0	0	0	0	0	0	0	0	0	0	0	0	0	0	0	0	0
0	0	0	0	0	0	0	0	0	0	0	0	0	0	0	0	0	0
0	0	0	0	0	0	0	0	0	0	0	0	0	0	66	0	0	0
0	0	0	0	0	0	0	0	0	0	0	0	0	0	0	0	0	0
0	217	0	0	0	0	0	0	49	0	0	0	0	0	0	0	0	0
0	0	0	0	0	0	0	0	0	0	0	0	0	0	0	0	0	0
0	0	0	0	0	0	0	0	0	0	0	0	0	0	0	0	0	0
0	0	0	0	0	0	0	40	0	0	0	0	0	0	0	0	0	49
0	0	0	0	0	0	0	0	161	0	0	0	0	0	0	0	0	0
3009	1248	880	12774	529	12201	3335	575	259	19876	578	374	607	212	748	69	346502	2979

0	0	0	0	0	0	0	0	0	0	0	0	0	0	0	0	0	0
0	0	0	0	0	0	0	0	0	0	0	0	0	0	0	0	0	0
0	0	0	0	0	0	0	0	0	0	0	0	0	0	0	0	0	0
0	0	0	0	0	0	0	0	0	0	0	0	0	0	0	0	0	0
0	0	0	4059	0	657	865	0	0	1346	0	0	0	0	0	0	0	155
0	0	0	0	0	0	0	0	0	0	0	0	0	0	0	0	0	0
0	0	0	0	0	0	0	0	0	0	0	0	0	0	0	0	0	0
0	0	0	0	0	0	0	0	0	0	0	0	0	0	0	0	0	0
0	207	36067	0	0	0	90	0	0	0	0	0	0	0	0	0	0	0
113	800	65706	0	372	0	206	0	0	0	33	0	0	45	0	0	0	0
0	0	0	0	0	0	0	0	0	0	0	0	0	0	0	0	0	0
0	0	0	0	0	0	0	0	0	0	0	0	0	0	0	0	0	0
0	0	0	0	0	0	0	0	0	0	0	0	0	0	0	0	0	0
0	0	0	0	0	0	0	0	0	0	0	0	0	0	0	0	0	0
0	0	0	0	0	0	0	0	0	0	0	0	0	0	0	0	0	0
0	0	0	0	0	0	0	0	0	0	0	0	0	0	0	0	0	0
19253	187	70	0	10468	0	0	1210	309	254	279	60	4426	37	75	110	0	309
0	0	0	0	0	0	0	0	0	0	0	0	0	0	0	0	0	0
0	7972	119	178397	171	116033	83	11047	0	0	437	0	42	0	0	0	0	0
0	0	0	0	0	0	0	0	0	0	0	0	0	0	0	0	0	0
0	0	0	0	0	0	0	0	0	0	0	0	0	0	0	0	0	0
0	0	0	0	0	0	0	0	0	0	0	0	0	0	0	0	0	0
0	0	0	0	4801	224	0	20513	0	0	0	0	0	532	384	970335	566	436
0	0	0	0	0	0	0	0	0	0	0	0	0	0	0	0	0	0
0	0	0	0	0	0	0	0	0	0	0	0	0	0	0	0	0	0
0	0	0	0	0	0	0	0	0	0	0	0	0	0	0	0	0	0
0	0	0	0	0	0	0	0	0	0	0	0	0	0	0	0	0	0
0	0	0	0	0	191	0	0	0	0	0	0	0	0	0	0	0	0
0	0	0	0	0	0	0	0	0	0	0	0	0	0	0	0	0	0
0	0	0	0	0	0	0	0	0	0	0	0	0	0	0	0	0	0
0	0	0	0	0	0	0	0	0	0	0	0	0	0	0	0	0	0
44405	8338	226	2032	1386	5054	696	441	163	16492	699	229	182	228	45995	534	119	1035
0	0	0	0	0	0	0	0	0	0	0	0	0	0	0	0	0	0
255	0	0	0	0	0	0	0	0	0	0	0	0	0	0	0	0	0
0	0	0	0	0	0	0	0	0	0	0	0	0	0	0	0	0	0
0	0	0	0	0	0	0	0	0	0	0	0	0	0	0	0	0	0
0	0	0	0	0	0	0	0	0	0	0	0	0	0	0	0	0	0
0	0	0	0	0	0	0	0	0	47	0	32	0	0	0	0	0	0
0	0	0	0	0	0	0	0	0	0	0	0	0	120	0	37	13174	0
0	1202	0	0	0	3073	0	0	0	0	0	0	0	0	0	0	0	0
0	203	28142	0	0	0	0	0	0	0	0	0	0	0	0	0	0	0
0	0	0	0	0	0	0	0	95	40	46	0	93	0	0	0	0	0
133	0	0	211	0	194	0	0	0	41	0	0	0	0	0	0	0	0
0	0	0	0	0	0	0	0	0	0	0	0	0	0	0	0	0	0
0	0	0	0	0	0	0	40	0	0	47	0	0	0	0	72	0	0
0	0	0	0	0	0	0	0	0	0	0	0	0	0	0	0	0	0
62	0	66	0	13868	0	0	0	0	0	0	0	0	0	0	0	0	0
0	0	0	0	0	0	0	0	0	0	0	0	0	0	0	0	0	0
0	0	0	0	0	0	0	0	0	0	0	0	0	0	0	0	0	0
0	0	0	0	0	0	0	0	0	0	0	0	0	0	0	0	0	0
0	0	0	0	0	0	0	0	0	790	0	0	0	0	0	0	0	0
0	0	0	0	0	0	0	0	0	0	0	0	0	0	0	0	0	0
0	0	0	0	0	0	0	0	0	0	0	0	0	0	0	0	0	0
115	421	0	0	0	1830	0	16281	144	47	88	36	188	34	146	286	189	4333
0	0	0	0	0	0	0	0	0	0	0	0	0	0	0	0	0	0
1128	0	0	111	0	0	0	69	0	0	0	0	0	0	0	0	0	0
0	0	0	0	0	0	0	0	0	0	0	0	0	0	0	0	0	0
60	0	0	0	0	0	0	0	0	0	0	0	0	0	0	0	0	0
0	0	0	0	0	0	0	0	0	0	0	0	0	0	0	0	0	0
0	0	0	0	0	0	0	0	0	0	0	0	0	0	0	0	0	0
0	0	0	0	0	0	0	34	0	0	0	0	0	103	0	247	69	244
0	2376	0	0	0	0	0	0	0	0	0	0	0	0	0	0	0	0
0	0	0	0	0	0	0	0	0	0	0	0	0	0	0	0	0	0
0	0	0	0	0	0	0	32	107	2860	0	12137	0	33	0	0	68	52
0	0	0	0	0	0	0	23384	0	0	0	0	0	37	0	0	62	105
0	0	48	1458	0	424	989	0	0	198	0	0	0	0	0	0	1582	114
0	0	0	0	0	0	0	0	0	0	0	0	0	0	0	0	0	0
0	0	0	0	0	0	0	0	0	0	0	0	0	0	0	0	0	0
0	0	0	0	0	0	0	185	0	2312	0	34	0	204	70	207	27032	103
0	0	0	0	0	0	0	0	0	0	0	0	0	0	0	0	0	0
0	0	0	0	0	0	0	0	0	0	0	0	0	0	0	0	0	0
0	0	0	0	0	2262	0	0	0	0	0	0	0	0	0	0	0	0
0	0	0	0	0	0	0	0	0	0	0	0	0	0	0	0	0	0
0	0	0	0	0	0	0	37	0	0	0	0	0	0	0	0	0	0
0	0	0	0	0	0	0	0	0	0	0	0	0	0	0	0	0	0
0	0	0	0	0	0	0	0	0	0	0	0	0	0	0	0	0	0
0	0	0	0	0	0	0	0	0	0	0	0	0	0	0	0	0	0
0	0	0	0	0	0	0	0	0	0	0	0	0	0	0	0	0	0
0	0	0	0	0	222	0	0	0	0	0	0	233	0	0	0	0	0
0	0	0	0	0	0	0	0	0	0	0	0	0	0	0	0	0	0
2561	420	0	0	0	0	101	30	0	360	87	0	0	0	405	0	0	0
0	0	0	0	0	0	0	0	0	0	0	0	0	0	0	0	0	0
0	0	0	0	0	0	0	0	0	0	0	20119	61	0	0	0	0	0
0	0	0	0	0	0	0	0	39	4975	35	0	100	0	0	0	0	0
0	0	0	0	0	0	0	103	0	0	0	0	0	212	63	59	419	586
0	0	0	0	0	0	0	0	0	0	0	0	0	0	0	0	0	0
0	0	122	0	0	0	0	0	0	0	0	0	0	0	0	0	0	0

341	218	0	0	0	394	0	0	0	0	0	0	0	0	0	0	0	0
0	0	0	0	0	0	0	0	0	0	0	0	0	0	0	0	0	0
0	0	0	0	0	0	0	0	0	0	0	0	0	0	0	0	0	0
0	0	0	0	0	0	0	0	0	0	0	0	0	0	0	0	0	0
0	0	0	0	0	0	0	0	0	0	0	0	0	0	0	0	0	0
0	0	0	0	0	0	0	0	53	0	0	0	0	0	0	0	0	0
0	0	0	0	0	0	0	0	0	0	0	0	0	0	0	0	0	0
0	0	0	0	0	0	0	0	0	0	0	0	0	0	0	0	0	0
0	0	0	0	0	0	0	0	0	0	0	0	0	0	0	0	0	0
0	2255	0	0	0	0	0	0	0	0	0	33	0	0	0	0	0	0
0	0	26117	0	0	0	300	45	0	0	39	35	0	0	0	0	0	0
0	11732	0	0	0	0	0	0	0	0	0	0	0	0	0	0	0	0
0	0	0	0	0	0	0	0	0	0	0	0	0	0	0	0	0	0
0	0	0	0	0	0	0	0	0	0	0	0	0	0	0	0	0	0
0	0	0	0	0	0	0	0	0	0	0	0	0	0	0	0	0	0
0	0	0	0	0	0	0	0	0	0	0	0	0	0	0	0	0	0
0	0	0	0	0	0	0	0	0	0	0	0	0	0	0	0	0	0
0	0	0	0	0	0	0	0	0	0	0	0	0	0	0	0	0	0
0	0	0	0	0	0	0	0	0	0	0	0	0	0	0	0	0	0
60	3913	0	0	0	0	0	32	269	78	227	57	53	0	0	100	0	0
187001	240513	2370	10804	80612	14600	3351	39781	6101	59500	497828	429373	798658	785022	11145	3181	25084	112456
0	0	0	0	0	0	0	0	0	0	0	0	0	0	0	0	0	0
0	200	36659	0	0	0	0	0	0	0	0	0	0	0	0	0	0	0
0	0	0	0	0	0	0	0	0	2691	0	0	0	0	0	0	0	0
0	0	0	0	0	0	0	0	0	0	0	0	0	0	0	0	0	0
0	0	0	0	0	0	0	0	0	0	0	0	0	0	0	0	0	0
0	0	0	0	0	0	0	0	0	0	0	0	0	0	0	0	0	0
54	411	0	0	0	0	0	0	0	0	0	0	0	0	0	0	426	46
0	0	0	0	0	0	0	0	0	0	0	0	0	0	0	0	0	0
CC019	CC025	CC020	CC033	CC022	CC023	CC021	Control09	Control08	Control08	Control08	Control08	Control08	Control08	Control09	Control09	Control09	Control09
0	0	0	0	0	0	0	0	0	0	0	0	0	0	0	0	0	0
0	0	0	0	0	0	0	0	0	37	0	0	0	0	0	0	0	0
0	0	0	0	0	0	0	0	0	0	0	0	0	0	0	0	0	0
0	0	0	0	0	0	0	0	0	0	0	0	0	0	0	0	0	0
651	0	0	0	0	0	0	0	0	0	0	0	0	0	0	0	0	0
0	0	0	0	0	0	0	0	0	0	0	0	0	0	0	0	0	0
0	0	0	0	0	0	0	0	0	0	0	0	43	89	0	0	0	0
640546	153216	4967	23637	77463	75283	6886	82229	7752	294114	18575	20713	4637	3697	508212	5504	9416	37060
0	0	0	0	0	0	0	0	0	0	0	0	0	0	0	0	0	0
0	0	0	0	0	0	0	0	0	0	0	0	0	0	0	72	0	0
0	0	0	0	0	0	0	0	0	0	0	0	0	0	0	0	0	0
0	0	0	0	0	0	0	0	0	0	0	0	0	0	0	0	0	0
0	0	0	0	0	0	0	0	0	0	0	0	0	0	0	0	0	0
0	0	0	0	0	0	0	38	0	0	0	0	0	74	63010	0	0	166
0	0	0	0	0	0	0	0	0	0	0	70	0	0	0	0	0	0
0	0	0	0	0	0	0	0	0	0	0	0	0	0	0	0	0	0
0	203	0	101	0	189	98	39	0	0	0	0	0	34	0	0	0	0
218	8272	55	638	0	2914	800	226	56	2036	173	0	44	360	278	536	177	275
0	0	0	0	0	0	0	0	0	0	0	0	0	0	0	0	0	0
0	0	0	0	0	0	0	0	0	0	0	0	0	0	0	0	0	0
0	0	0	0	0	0	0	0	0	0	0	0	0	0	0	0	0	0
0	0	0	0	0	0	0	0	0	0	0	0	0	0	0	0	0	0
0	0	0	0	0	0	0	0	0	0	0	0	0	0	0	0	0	0
0	4334	0	0	0	0	0	0	0	0	0	0	0	0	0	0	0	0
13154	2657	56	309	170	2270	0	263	137	5272	301	255	49	48	6581	255	0	1102
0	0	0	0	0	0	0	0	0	0	0	0	0	0	0	0	0	0
0	0	0	0	0	0	0	39	0	0	0	0	0	0	70	0	0	0
0	0	0	0	0	0	0	0	0	0	0	0	0	0	0	0	0	0
0	0	0	0	0	0	0	0	0	0	0	0	0	0	0	0	0	0
0	402	0	0	0	0	0	0	0	0	0	0	0	0	0	0	0	0
0	3075	0	218	0	0	0	42343	0	2983	0	0	0	0	283	0	54	0
0	0	0	0	0	0	0	0	0	0	0	0	0	0	0	0	0	0
0	0	0	0	0	0	0	0	0	0	0	0	0	0	0	0	0	0
0	0	0	0	0	0	0	0	0	0	0	0	0	0	0	0	0	0
0	0	0	0	0	0	302	0	0	0	0	0	0	0	0	0	0	0
0	0	0	300	0	0	0	405	973659	1282	970	624	1121	401	402	814	316	950
0	0	0	0	0	0	0	0	0	0	0	0	0	0	0	0	0	0
0	201	0	1408	190	6251	281	0	41	587	0	0	0	0	0	0	0	1071
0	0	0	0	0	0	0	0	0	0	0	0	0	0	0	0	0	0
0	0	0	0	0	0	0	0	0	0	0	0	0	34	0	65	0	0
0	0	0	0	0	0	0	0	0	0	0	0	0	0	0	0	0	0
58	0	0	0	0	0	0	75	44	112	31	78	51	80	62	64	0	0
0	0	0	0	0	0	0	0	0	0	0	0	0	38	0	0	0	0
0	0	0	0	0	0	0	0	0	0	0	0	0	0	0	0	0	0
0	0	0	0	0	0	0	0	0	0	0	0	0	0	0	0	0	0
0	0	0	0	0	0	0	0	0	0	0	0	0	0	0	0	0	0
0	0	0	0	0	0	0	0	0	0	0	0	0	0	0	0	0	0
0	0	0	0	0	415	0	0	0	33	36	0	0	0	0	0	0	0
0	0	0	0	0	0	0	0	0	0	44	0	0	0	0	0	0	0
0	0	0	0	0	0	0	0	0	0	0	0	0	0	0	0	0	0
0	0	0	0	0	0	0	0	0	743	0	0	0	0	0	0	0	0
0	0	0	0	0	0	0	0	0	0	0	0	0	0	0	0	0	0
0	0	0	0	0	0	0	0	0	0	0	0	0	0	0	0	0	0
0	0	0	0	0	0	0	0	0	0	0	0	0	0	0	0	0	0
0	0	0	0	0	0	0	0	0	0	0	0	0	0	0	0	0	0

0	399	0	0	0	0	0	0	0	0	0	0	0	0	0	0	0	0
0	622	38487	0	6239	610	188	0	0	32	0	10316	0	0	12329	0	0	1835
0	0	0	0	0	0	0	0	0	0	0	0	0	0	0	0	0	0
114	1277	0	0	0	385	0	0	0	0	0	0	0	0	0	0	0	0
11691	10149	14880	281163	115276	70280	226945	1759	2588	207384	707	235	57509	8385	1607	1345	130061	144431
0	0	967	0	0	594	0	0	0	0	0	0	0	0	0	0	347	58
0	0	0	0	0	0	0	0	0	0	0	0	0	44	0	0	0	0
0	0	0	0	0	0	0	0	0	0	0	0	0	0	0	0	0	0
48	0	31378	0	0	1716	294	30	0	0	0	0	46	0	0	58	8533	2955
0	797	0	0	0	0	0	0	0	0	83	92	0	0	0	55	0	60
0	0	0	0	0	0	0	0	0	0	0	0	0	0	0	0	0	0
0	0	0	0	0	0	0	0	0	0	0	0	0	0	0	0	0	0
0	0	0	0	0	0	0	0	0	0	0	0	0	0	0	0	0	0
0	0	0	0	0	0	0	0	0	0	0	0	0	0	0	0	0	0
0	0	0	0	0	0	0	0	0	0	0	0	0	0	0	0	0	0
0	0	0	0	0	0	0	35	0	0	0	0	0	149	72	60	0	0
0	0	0	0	0	0	0	0	0	0	0	0	0	0	0	0	0	0
59	0	0	0	0	3663	0	0	0	0	0	0	0	0	0	0	0	0
0	0	0	0	0	0	0	0	0	0	0	0	0	0	0	0	0	0
0	0	0	0	0	0	0	0	0	0	0	0	0	0	0	0	0	0
0	0	0	0	0	0	0	0	0	0	0	0	0	0	0	0	0	0
0	0	0	0	0	0	0	0	0	0	0	0	0	0	0	0	0	0
0	0	0	0	0	0	0	0	0	0	0	0	0	0	0	0	0	0
0	0	0	0	0	0	0	0	0	181	0	0	0	70	0	0	0	0
0	0	0	0	0	0	0	0	0	0	0	0	0	0	0	0	0	0
0	186	0	0	0	0	0	0	0	0	0	0	0	0	0	0	0	0
0	218	0	0	0	0	0	0	0	156	42	33	0	0	0	0	0	167
0	0	0	0	0	0	0	44	0	0	0	0	0	15250	0	0	0	0
0	0	0	0	0	0	0	0	0	0	0	0	0	0	0	0	0	0
0	0	0	0	0	0	0	0	0	0	0	0	0	0	0	0	0	0
0	0	0	0	0	0	0	0	0	0	0	0	0	0	0	0	0	0
0	0	0	0	0	0	0	0	0	0	0	0	0	0	0	0	0	10069
0	0	0	0	0	0	0	0	48	114	113	45	62	0	0	0	0	0
0	0	0	0	0	0	0	0	0	0	0	0	0	0	0	0	0	0
0	0	0	0	0	0	0	0	0	0	0	0	0	0	0	0	0	0
1144	2415	10239	433238	193348	90510	730166	262	615	271612	637	492	543	146	150	276	260	266569
0	0	0	0	0	0	0	0	0	0	0	0	0	0	0	0	0	0
0	0	0	0	0	0	0	0	0	0	0	0	0	0	0	0	0	0
0	0	0	0	0	0	0	0	54	0	43	35	0	0	0	0	0	0
0	214	0	104	8754	601	0	38	0	1672	0	0	0	66	78	0	0	0
0	0	0	0	0	0	0	0	0	0	0	0	0	0	0	0	0	0
0	0	0	0	0	0	0	0	0	0	0	0	0	0	0	0	0	0
0	0	0	0	0	0	0	0	0	0	0	0	0	0	0	0	0	0
0	0	0	0	0	0	0	0	145	0	0	0	0	0	0	0	0	0
0	0	0	0	0	0	0	0	0	0	0	0	0	0	0	0	0	0
0	0	0	0	0	0	0	0	0	0	0	0	0	0	0	0	0	0
0	0	0	0	0	0	0	0	0	0	0	0	0	0	0	0	0	0
0	0	0	0	0	0	0	0	0	0	0	0	0	0	0	0	0	0
0	0	0	0	0	0	0	0	0	0	0	0	0	0	0	0	0	0
0	0	0	0	0	0	0	0	0	0	0	0	0	0	0	0	0	0
1047	0	0	0	0	0	0	0	0	3638	87	0	0	0	184	0	0	0
0	0	0	0	0	0	0	0	0	0	0	0	0	0	0	0	0	0
0	0	0	0	0	0	0	0	0	0	0	0	0	78	0	53	0	0
0	3907	1096	904	4234	393	963	0	0	0	0	0	0	0	0	62	0	0
0	35766	58	0	0	0	103	0	0	0	0	0	0	0	0	0	0	0
0	0	0	0	0	0	0	0	0	0	0	0	0	0	0	0	0	0
0	0	0	0	0	0	0	0	0	0	0	0	0	0	0	0	0	0
0	0	0	0	0	0	0	0	0	50	0	0	46	0	0	0	0	0
0	0	0	0	0	0	0	0	0	0	0	0	0	0	0	0	0	0
0	0	0	0	0	0	0	0	0	0	0	0	0	0	0	0	0	0
0	0	0	0	0	0	0	0	0	0	0	0	0	0	0	0	0	0
0	8500	0	0	0	0	0	205	0	0	0	0	0	156	72	124	34295	0
0	0	0	0	0	0	0	0	0	0	0	0	0	0	0	0	0	0
0	0	0	0	0	0	0	0	0	0	0	0	0	0	0	0	0	0
0	0	0	0	0	192	0	0	0	86	0	0	55	39	0	121	221	0
2458	185520	275	495	70528	631	92	35623	0	0	0	0	0	218	219	183	175	232
0	391	0	0	167	194	99	0	0	0	0	0	0	0	0	0	0	0
259	23919	0	0	11757	0	94	27	0	0	0	0	0	0	0	0	66	0
0	2050	0	94	30304	0	0	29	0	0	0	0	0	37	0	0	102	0
0	0	0	0	0	0	0	0	0	0	0	0	0	0	0	0	0	0
0	0	0	0	0	0	0	0	0	0	0	0	0	0	0	0	0	0
0	0	0	0	0	0	0	0	0	0	0	0	0	0	0	0	0	0
0	0	0	0	0	0	0	0	0	0	0	0	0	0	0	0	0	0
0	578	0	0	0	0	0	0	0	0	0	0	0	0	0	0	0	0
0	0	0	0	0	0	0	0	0	0	78	36	62	0	0	0	0	0
0	0	0	0	0	0	0	0	46	0	0	0	0	0	74	0	0	0
0	0	0	0	0	0	0	0	0	0	0	0	0	0	0	0	0	0
0	0	0	0	0	0	0	0	0	0	0	0	0	0	0	0	0	0
0	0	0	0	0	0	0	0	0	0	0	0	0	0	0	0	0	0
0	0	0	0	0	0	0	0	0	0	0	0	0	0	0	0	0	0
0	0	0	0	0	582	0	0	0	0	0	0	0	0	0	0	0	0
0	0	0	0	0	0	0	0	0	0	0	0	0	0	0	0	0	0
0	0	0	0	0	0	0	0	0	0	0	0	0	0	0	0	0	0
0	0	0	0	0	0	0	0	0	0	0	0	0	0	0	0	0	0
0	0	0	0	0	0	0	0	0	0	0	0	0	0	0	0	0	0
0	13638	51	4853	0	522871	190	35	44	528	0	0	0	37	74	0	10836	35765
0	0	0	0	0	0	0	0	0	0	0	0	0	0	0	0	0	0

0	0	0	0	0	193	0	0	0	0	0	0	0	77	0	0	0	121
0	0	0	0	0	0	0	0	0	0	0	0	0	0	0	0	0	0
0	0	0	1556	154	0	0	0	0	41	94	0	0	0	0	0	0	0
0	0	0	0	0	0	0	0	0	0	0	0	0	0	0	0	0	0
0	26714	0	1011	161	2034	98	92667	621	229	215	53371	172	327	342	113	2724	28850
0	1437	118	0	0	0	0	196	0	0	0	469	0	0	0	0	0	55
304	3668	170370	302	1048	1461	927	31918	143	956	292	43868	161	674	402	672	3034	535
0	45893	0	418	15073	1435	0	54299	81	36	48	8934	144	278	547	699	30738	2736
0	1373	0	1065	184	220	0	6969	0	75	0	4759	0	40	73	107	26445	2003
0	2221	0	96	158	204	0	0	129	0	53	0	54	0	0	0	0	0
0	0	0	0	0	0	0	0	0	0	0	0	0	0	0	0	0	0
0	0	0	0	0	0	0	0	0	0	0	0	0	0	0	0	0	0
0	0	0	0	0	0	0	0	0	0	0	0	0	0	0	0	0	0
0	0	0	0	0	0	0	0	0	0	0	0	0	0	0	0	0	0
0	0	0	0	0	0	0	0	0	0	0	0	0	0	0	0	0	0
0	0	0	0	0	0	0	0	0	0	0	0	0	0	0	0	0	0
0	0	0	0	0	0	0	0	0	0	0	0	0	0	0	0	0	0
0	0	0	0	0	0	0	0	0	0	0	0	0	0	0	0	0	0
0	0	15779	0	0	0	0	0	0	0	0	0	0	0	0	0	0	0
0	0	0	101	0	0	0	0	0	0	0	0	0	0	0	0	0	0
0	0	0	0	0	0	0	0	0	0	0	0	0	0	0	0	0	0
0	0	0	0	0	0	0	24	0	0	35	27	46	29	222	0	0	54
0	0	0	0	0	0	0	0	0	0	0	0	0	0	0	47	0	0
0	0	0	0	0	0	0	0	0	0	0	0	0	0	0	0	0	0
0	0	0	0	10188	0	0	0	0	0	0	0	0	0	0	0	0	0
0	0	0	0	0	0	0	0	0	0	0	0	0	0	0	0	0	0
0	0	0	0	0	0	0	0	0	38	0	34	0	0	0	0	0	0
0	0	0	0	0	0	0	0	0	0	0	0	0	0	0	0	0	0
0	0	0	0	0	0	0	0	0	165	0	0	0	0	0	0	0	0
0	0	0	0	0	0	0	0	0	0	0	0	0	0	0	0	0	0
0	0	0	0	0	0	0	0	0	0	0	0	0	0	0	69	0	0
0	0	0	0	0	0	0	0	0	0	0	0	0	0	0	0	0	0
57	0	0	0	0	0	0	74673	803	15176	1342	172886	537	38481	154654	7500	131922	130266
0	0	0	0	0	0	0	0	0	0	0	0	0	0	0	0	0	0
0	196	0	0	0	0	0	0	0	0	0	0	0	0	0	0	0	0
0	0	0	0	0	0	0	0	0	0	0	0	0	0	0	0	0	0
0	0	0	0	0	0	0	0	0	0	0	0	0	0	0	0	0	0
0	1020	63	0	0	0	0	0	0	0	0	0	0	0	0	0	0	0
0	76148	96	0	0	401	83	0	0	0	0	0	0	0	0	0	0	0
432	0	0	0	0	0	0	0	0	84	133	0	101	0	0	0	0	0
0	0	0	0	0	0	0	0	0	0	0	0	0	0	0	0	0	0
0	0	0	0	0	0	0	0	0	0	0	0	0	0	0	0	0	0
0	0	0	0	0	0	0	0	0	0	0	0	0	0	0	0	0	0
0	0	0	0	0	0	0	0	0	0	0	0	0	0	0	0	0	0
0	0	0	0	0	0	99	0	0	0	0	0	0	0	0	0	0	0
0	0	0	0	0	208	0	0	0	0	0	0	0	0	0	0	0	0
0	0	0	0	0	0	0	82	0	0	0	0	0	90	0	62	0	47
0	0	0	0	0	0	0	0	0	0	0	0	0	0	0	0	0	0
0	0	0	0	0	0	0	0	0	0	0	0	0	0	0	0	0	0
0	0	0	0	0	0	0	0	0	0	0	0	0	0	0	66	0	0
0	0	0	0	0	0	0	0	0	0	0	0	0	0	0	0	0	0
0	0	0	0	0	0	0	0	0	0	0	0	0	0	0	0	0	5975
75	2867	0	0	0	0	0	0	0	0	0	0	0	0	66	124	109	50
0	0	0	0	0	0	0	0	0	0	0	0	0	0	0	0	0	47
0	0	0	0	0	0	0	0	0	45	0	0	0	0	0	0	0	0
0	0	0	0	0	199	0	0	0	0	0	0	0	0	0	0	0	0
0	0	0	0	0	0	0	0	0	0	0	0	0	0	0	0	0	0
0	0	0	0	0	0	0	0	0	0	0	0	0	0	0	0	0	0
0	0	0	0	0	0	0	47	0	0	0	0	0	0	0	46	0	14776
0	0	0	0	0	0	0	0	0	0	0	0	0	0	0	0	0	0
0	0	0	0	0	0	0	0	0	0	0	0	0	0	0	0	0	0
0	0	0	0	0	0	0	141	0	0	0	0	0	111	136	169	139	168
0	0	0	0	0	0	0	0	0	0	0	0	0	0	0	0	0	0
0	0	0	0	0	0	0	0	0	0	0	0	0	0	0	0	0	0
0	0	0	0	0	0	0	0	0	0	0	0	0	0	0	0	0	0
0	611	0	0	0	0	0	0	0	92	76	35	49	0	0	0	0	0
0	0	0	0	0	0	0	0	0	0	0	0	0	0	0	0	0	0
0	0	0	231	0	0	0	0	0	0	0	0	0	0	0	0	0	0
0	0	0	0	0	0	0	0	0	0	0	0	0	0	0	0	0	0
0	0	0	0	0	0	0	0	0	0	0	0	0	0	0	0	0	0
0	0	0	0	0	0	0	162	0	0	0	0	0	124	59	0	0	58
0	0	0	0	0	0	0	0	0	41	0	0	50	0	0	61	0	0
0	12523	0	0	0	0	0	0	0	0	0	0	0	0	0	0	0	0
0	8686	0	102	0	3713	0	0	0	0	0	0	0	0	0	0	0	0
2733	832	44	0	10992	616	94	7583	827	10175	884	1113	535	5876	10629	184	0	7005
0	0	0	0	0	0	0	0	0	0	0	0	0	0	0	0	0	0
0	0	0	0	0	0	0	0	0	0	0	0	0	0	0	0	0	0
0	0	0	0	0	0	0	0	0	0	0	0	0	0	0	0	0	0
0	0	0	0	0	0	0	0	0	0	0	0	0	0	0	0	0	0
0	0	0	0	0	0	0	0	0	0	0	0	0	0	0	0	0	0
0	795	0	0	0	0	0	0	0	0	0	0	0	0	0	0	0	0
115	0	0	0	0	0	0	0	0	0	0	0	0	0	0	0	0	0
0	0	0	0	0	0	0	0	0	0	0	0	0	0	0	0	0	0
0	0	0	0	0	0	0	0	0	0	0	0	0	0	0	0	0	0
0	0	0	0	0	0	0	0	0	0	0	0	0	0	0	0	0	0
0	0	0	0	0	0	0	32085	0	0	0	0	0	0	62	0	0	132

0	0	0	0	0	0	0	0	0	0	0	0	0	0	0	0	0	0
7564	177	64	718	176	0	489	0	0	117	39	73	0	0	0	0	0	66
224	0	0	195	0	0	0	0	0	0	0	0	0	0	0	0	0	0
0	0	0	0	0	0	0	0	0	0	0	0	0	0	0	0	0	0
0	0	0	0	0	0	0	0	0	0	0	0	0	0	0	0	0	0
0	0	0	0	0	0	0	0	0	0	0	0	0	0	0	0	0	0
0	0	41	0	499	0	0	0	0	0	0	0	0	0	0	0	0	0
0	0	0	0	0	0	0	0	0	0	0	0	0	0	0	0	0	0
0	0	0	0	0	0	0	0	0	0	0	0	0	0	0	0	0	0
0	0	0	0	0	0	0	0	0	0	0	0	0	0	0	0	0	0
0	0	0	0	0	0	0	0	0	0	0	0	0	0	0	0	0	0
0	0	0	0	0	0	0	0	0	0	0	0	0	0	0	0	0	0
0	0	0	0	0	0	0	0	0	0	0	0	0	0	0	0	0	0
0	0	0	0	0	0	0	0	0	0	0	0	0	0	0	0	0	0
0	0	0	0	0	0	0	0	0	0	0	0	0	0	0	0	0	0
0	0	0	0	0	0	0	0	0	0	0	0	45	0	0	0	0	0
0	0	0	0	0	0	0	0	0	0	0	0	0	0	0	0	0	0
0	0	0	0	0	0	0	0	0	0	0	0	0	0	0	0	0	0
0	0	0	0	0	0	0	0	0	0	0	0	0	0	0	0	0	0
0	0	0	0	0	0	0	0	0	0	0	0	0	0	0	0	0	0
0	0	0	0	0	0	0	0	0	0	0	0	0	0	0	0	0	0
0	0	0	0	0	0	0	0	0	0	0	0	0	0	0	0	0	0
7634	2033	0	284	0	1633	96	5020	0	9440	81	157	105	0	11826	0	0	272
0	0	0	0	0	0	0	0	0	0	0	0	0	0	0	0	0	0
0	197	0	318	164	0	0	38	0	0	0	0	0	0	0	110	37617	0
0	0	0	0	0	0	0	71	0	0	0	0	0	129	63	68	50	0
0	0	0	0	0	0	0	0	0	0	0	0	0	0	0	0	0	0
0	0	0	0	0	0	0	0	0	0	0	0	0	0	0	0	0	0
0	0	0	0	0	0	0	48	0	0	0	0	0	116	0	193	239	47
0	0	0	0	0	0	0	0	0	0	0	0	0	0	0	0	0	0
0	0	0	0	0	0	0	0	0	0	0	0	0	0	0	0	0	0
0	0	0	0	0	0	0	0	0	0	0	0	0	0	0	0	0	0
0	0	0	0	0	0	0	0	0	0	0	0	0	0	0	0	0	0
18911	4242	35947	11060	22602	3854	8348	18473	1475	16966	67633	17784	123554	121015	98958	885	73278	16376
0	0	0	0	0	0	0	0	0	0	0	0	0	0	0	0	0	0
0	0	0	0	0	0	0	0	0	0	0	0	0	0	0	0	0	0
64	409	0	116	0	2234	0	0	44	0	0	0	0	0	0	0	0	0
0	0	0	0	0	0	0	0	37	91	0	44	0	0	0	0	0	0
0	818	0	109	177	0	0	0	0	0	0	0	0	0	0	0	0	0
0	212	0	0	0	2481	194	0	0	3234	0	0	0	0	0	0	0	0
0	0	0	0	0	0	0	0	0	0	0	0	0	0	0	0	0	0
0	0	0	102	0	0	109	0	0	271	0	0	0	0	0	0	0	623
0	0	0	0	0	0	0	0	0	0	0	0	0	0	0	0	0	0
0	0	0	0	0	0	0	0	0	0	0	0	0	0	0	0	0	0
0	0	0	96	0	0	0	0	0	82	0	0	42	35	198	349	0	0
53	0	0	0	0	0	0	0	0	0	0	0	0	0	0	0	0	0
0	0	0	0	0	0	0	0	0	35	0	35	0	0	0	0	0	0
0	0	0	0	0	0	0	0	0	0	0	0	0	0	0	0	0	0
0	0	0	0	0	0	0	0	0	0	0	0	0	0	0	0	0	0
0	0	0	0	0	0	0	0	0	0	0	0	0	0	0	0	0	0
0	0	0	0	0	0	0	0	0	0	0	0	0	0	0	0	0	0
0	6030	0	2156	0	19221	0	0	0	0	0	0	0	0	0	0	0	0
59	0	0	0	0	0	0	33	0	0	0	0	0	0	0	0	748	0
0	0	0	0	0	0	0	0	0	0	0	0	0	0	0	0	0	0
0	0	0	0	0	0	0	0	0	874	0	0	0	0	0	0	0	0
0	0	0	0	0	0	0	0	0	0	0	0	0	30	0	0	0	52
0	0	0	0	0	0	0	0	0	0	0	0	0	0	0	0	0	0
0	0	0	109	0	0	0	0	0	0	0	0	0	0	0	0	0	0
0	0	0	0	0	0	0	0	0	0	0	0	0	0	0	0	0	0
0	0	0	0	0	0	0	0	0	0	0	0	0	0	0	0	0	0
0	0	0	0	0	0	0	0	0	0	0	0	0	0	0	0	0	0
0	0	0	0	0	0	0	0	0	0	0	0	0	0	0	0	0	0
0	0	0	0	0	0	0	0	0	0	0	0	0	0	0	0	0	0
0	0	0	0	0	0	0	0	0	0	0	0	0	0	0	0	0	0
0	0	0	0	0	0	0	0	0	0	0	0	0	0	0	0	0	0
0	0	0	0	0	0	0	0	0	0	0	0	0	0	0	0	0	0
0	2033	0	0	0	0	0	0	0	0	0	0	0	0	0	0	0	0
0	0	0	0	0	0	0	0	0	0	0	0	0	0	0	0	0	0
0	0	0	220	0	795	0	0	0	0	0	0	0	88	0	126	0	53
0	0	0	0	0	197	0	0	0	0	0	0	0	0	0	0	0	0
0	0	0	0	0	0	0	0	0	0	0	0	0	0	0	0	0	0
0	0	0	0	0	0	0	0	0	0	0	0	0	0	0	0	0	0
853	0	0	0	0	0	0	39	0	0	48	85	314	178	0	0	0	76
0	0	0	0	0	0	0	0	0	0	0	0	0	0	0	0	0	0
0	0	0	0	0	0	0	0	0	0	0	0	0	0	0	0	0	0
0	0	0	206	0	759	0	0	0	0	0	0	0	0	0	0	0	0
0	0	0	0	0	0	0	0	0	0	0	0	0	0	0	0	0	0
0	0	0	0	0	0	0	0	0	0	0	0	0	0	0	0	0	0
0	0	0	0	0	0	0	0	0	0	0	0	0	0	0	0	0	0
0	0	0	0	0	0	0	0	0	0	0	0	0	0	0	0	0	0
0	0	0	0	0	0	0	0	0	0	0	0	0	0	0	0	0	0
0	1983	0	0	0	0	87	0	0	0	0	0	0	0	0	0	0	0
0	0	0	0	0	0	0	0	0	0	0	0	0	0	0	0	0	0
0	0	0	0	0	0	0	0	0	0	0	0	0	0	0	0	0	0
54	1839	0	1771	0	629	0	0	0	81	51814	625	0	301	0	0	0	0
0	0	0	0	0	0	0	0	0	0	0	0	0	0	0	0	0	0
0	0	0	0	0	0	0	0	0	0	0	0	0	0	0	0	0	0

0	0	0	0	0	0	0	0	0	0	0	22836	0	0	0	0	0	0
0	0	0	0	0	0	0	0	0	0	0	0	0	0	0	0	0	0
0	0	0	0	0	0	0	0	0	0	0	0	0	0	0	0	0	0
0	0	0	0	0	0	0	0	0	0	0	0	0	0	0	0	0	0
0	0	0	0	0	0	0	0	0	0	0	0	0	0	0	0	0	0
0	0	0	0	12827	0	0	0	0	0	0	0	0	0	0	51	0	0
0	0	0	0	0	191	0	0	0	0	0	0	0	0	0	0	0	0
0	0	0	0	171	0	0	0	0	0	0	0	0	0	0	0	0	0
0	0	0	0	0	0	0	0	0	0	0	0	0	0	0	0	0	0
0	0	0	0	0	0	0	0	0	0	0	0	0	0	0	0	0	0
0	0	0	0	0	0	0	0	0	0	0	0	0	0	62	0	0	6421
106	0	0	198	0	0	0	0	0	2737	0	4685	0	0	0	0	0	0
0	0	0	0	0	0	0	0	0	0	0	0	0	0	0	0	0	0
0	0	0	0	0	0	0	0	0	36	0	0	0	0	0	0	0	0
0	0	0	0	0	0	0	0	0	0	0	0	0	0	0	0	0	0
0	0	0	0	0	0	0	0	0	0	0	0	0	0	0	0	0	0
0	0	0	0	0	0	0	0	0	0	0	0	0	0	0	0	0	0
0	0	0	0	0	0	0	0	0	0	0	0	0	0	0	0	0	0
0	0	0	0	0	0	0	0	0	0	0	0	0	0	0	0	0	0
0	0	0	0	0	0	0	42	0	0	0	0	0	0	0	58	0	0
0	0	0	0	0	0	0	0	0	0	0	0	0	0	0	0	0	0
0	0	0	0	0	0	0	0	0	0	0	0	0	0	0	0	0	0
0	0	0	0	0	0	0	0	0	0	0	0	0	0	0	0	0	0
0	0	0	0	0	0	0	0	0	0	0	0	0	0	0	0	0	0
0	0	0	0	0	0	0	0	0	0	0	0	0	0	0	0	0	0
0	0	0	0	0	0	0	0	0	0	0	0	0	0	0	0	0	0
0	0	0	0	0	1431	0	0	0	0	0	0	0	0	0	0	0	0
0	0	0	0	0	0	0	0	0	5843	0	0	0	0	0	0	0	0
0	0	0	0	0	0	0	0	0	0	0	0	0	0	0	0	0	0
0	0	0	0	0	0	0	0	0	0	0	0	0	44	0	160	0	62
0	0	0	0	0	0	0	0	0	0	0	0	0	0	0	0	0	0
0	0	0	0	0	0	0	0	0	0	0	0	0	43	0	0	0	0
0	0	0	0	0	0	0	78	0	0	0	0	0	0	53080	242	0	111
0	0	0	0	0	0	0	0	0	0	0	0	0	0	0	0	0	0
0	0	0	0	0	0	0	0	0	0	0	0	0	0	0	0	0	0
0	2061	0	0	0	0	0	0	0	0	0	0	0	0	0	0	0	0
0	0	0	0	0	0	0	0	0	0	0	0	0	0	0	0	0	0
0	0	0	0	0	0	0	0	0	0	0	0	0	0	0	0	0	0
0	0	0	0	0	0	0	0	0	0	0	0	0	0	0	0	0	0
0	0	0	0	0	0	0	0	0	0	0	0	0	0	0	0	0	0
0	0	0	0	0	0	0	0	0	0	0	0	0	0	0	0	0	0
0	0	0	0	0	0	0	0	0	0	0	0	0	0	0	0	0	0
0	0	0	0	0	0	0	0	0	0	0	0	0	0	0	0	0	0
0	0	0	0	0	0	0	0	0	0	0	0	0	0	0	0	0	0
0	0	0	0	0	0	0	0	48	49	53	0	50	0	0	0	0	0
0	216	0	90	0	0	0	45	201	2350	313105	35039	152	89	73	0	62	6029
0	0	0	0	0	0	0	122	0	0	0	0	0	0	0	0	0	0
0	0	0	0	0	0	0	0	0	0	0	0	0	0	0	0	0	0
0	0	0	0	0	0	0	0	0	0	0	0	0	0	0	0	0	0
0	0	0	326	0	192	82	16484	51	135	0	6556	0	0	0	54	0	168
0	0	0	0	0	0	0	0	0	0	38	0	0	0	0	0	0	0
126	193	127732	10084	855	2743	956	121045	645	4529	38156	62650	572	633	619	215	25771	136558
0	0	0	0	0	0	0	71	52	77	0	25	104	0	0	111	0	3502
0	0	0	0	0	0	0	0	0	0	0	0	0	0	0	0	0	0
0	0	0	0	0	0	0	0	0	0	0	0	0	0	0	0	0	0
0	0	0	0	0	0	0	0	0	0	0	0	0	0	0	0	0	0
0	0	0	0	0	0	0	0	0	0	0	0	0	0	0	0	0	0
0	400	0	0	0	0	0	0	0	0	0	0	0	0	0	0	0	0
0	0	0	0	0	0	0	0	0	572	0	0	0	0	0	0	0	1374
0	0	0	0	0	0	0	0	0	0	0	0	0	0	0	0	0	0
414	7331	0	0	0	0	286	750	223	187	41	62	0	13880	1108	1165	47298	4780
0	0	0	0	0	0	0	0	0	228	0	0	0	0	0	0	0	0
0	0	0	0	0	0	0	0	0	0	0	0	0	0	0	0	0	0
0	0	0	0	0	0	0	0	0	0	0	0	0	0	0	0	0	0
0	0	0	0	0	0	0	0	0	0	0	0	0	0	0	0	0	0
0	0	0	0	0	0	0	0	0	0	0	0	0	0	0	0	0	0
0	0	0	0	0	0	0	0	0	0	0	0	0	43	109	56	0	0
0	0	0	0	0	0	0	0	0	0	0	0	0	0	0	0	0	0
0	0	0	0	0	0	0	0	0	0	0	0	0	0	0	0	0	0
0	0	0	0	0	996	0	0	0	0	0	0	0	0	0	0	0	0
0	618	0	0	0	398	164	0	0	0	0	0	0	72	0	0	7644	0
0	1968	0	0	0	0	0	245361	0	0	0	0	32	308	545	603	418	451
0	0	0	0	0	0	0	109	0	0	0	0	0	0	0	0	0	0
0	0	0	0	0	0	0	0	0	0	0	0	0	37	0	67	132	37
0	0	0	0	0	0	0	0	0	0	0	0	0	0	0	0	0	0
0	0	0	0	0	0	0	0	0	0	0	0	0	0	0	0	0	0
0	0	0	0	0	0	0	0	0	0	0	0	0	0	0	0	0	0
0	0	0	0	0	0	0	0	0	0	0	0	0	0	0	0	0	0
0	0	0	0	0	0	0	0	0	0	0	0	0	0	0	0	0	0
0	0	0	0	0	0	0	0	0	0	0	0	0	0	0	0	0	0
0	0	0	111	0	0	0	0	0	0	0	0	0	0	0	0	0	0
0	0	0	0	0	0	0	0	0	0	0	0	0	0	0	0	0	0
0	0	0	0	0	0	0	0	0	0	0	0	0	0	0	0	0	0
0	0	0	0	0	0	0	0	0	0	0	0	0	0	0	0	0	0
0	0	0	0	0	0	0	0	0	0	0	0	0	0	0	0	0	0
0	0	0	0	0	0	0	0	0	0	0	0	0	0	0	0	0	0
0	0	0	0	0	0	0	0	0	0	0	0	0	0	0	0	0	0

0	3932	1010	0	2567	0	1026	0	51	38	47	0	0	0	0	0	0	0
0	0	50	0	0	0	0	70	0	0	0	0	0	81	0	0	0	113
0	1229	0	0	0	0	0	0	0	0	0	0	49	0	52	0	0	0
0	0	0	0	0	0	0	0	0	4350	44	35	0	0	57	0	0	0
0	618	50	0	288972	0	261	0	0	37	0	0	0	0	0	0	0	0
0	0	0	0	0	0	0	0	0	0	0	0	0	0	0	0	0	0
0	0	0	0	0	0	0	0	0	0	0	0	0	0	0	0	0	0
0	0	0	0	0	0	0	0	0	0	0	0	0	0	0	0	0	0
0	0	0	0	0	0	0	0	0	0	0	0	0	0	0	0	0	0
0	0	0	0	0	0	0	0	0	0	0	0	0	0	0	0	0	0
0	0	0	0	0	1596	0	0	0	0	0	0	0	0	0	0	0	0
0	0	0	0	0	0	0	0	0	0	0	0	0	0	0	0	0	0
0	0	0	0	0	0	0	48	46	288	0	0	51	0	58	0	870	45
60	0	0	0	0	0	0	0	0	33	42	0	0	0	67	0	0	0
0	0	0	0	0	0	0	0	0	0	0	0	0	0	0	0	0	0
0	0	0	0	0	0	0	0	0	0	0	0	0	0	0	0	0	0
0	0	0	0	0	0	0	0	0	0	0	0	0	0	0	0	0	0
0	0	0	0	0	0	0	0	0	0	0	0	0	0	0	0	0	0
0	0	0	0	0	0	0	0	0	0	0	0	0	0	0	0	0	0
0	193	64626	0	180	0	90	0	0	0	0	0	0	0	0	0	0	0
0	0	0	0	0	0	0	0	0	0	0	0	0	0	0	0	0	0
8883	1417	59	199	328	1874	0	30	941	3997	1197	445	3860	112	5236	189	61	704
0	0	0	0	0	0	0	0	0	0	0	0	0	0	0	0	0	0
0	0	0	0	0	0	0	0	0	0	0	0	0	0	0	0	0	0
0	0	0	0	0	0	0	0	0	0	0	0	0	0	0	0	0	0
0	0	0	0	0	0	0	0	0	0	0	0	0	0	0	0	0	0
0	0	0	0	0	0	0	0	0	0	0	0	0	0	0	0	0	0
0	0	0	0	0	0	0	0	42	86	45	23986	0	0	0	0	0	0
1223	1399	61	8148	204	1002	8024	0	0	2264	0	28	0	35	0	0	0	825
0	0	0	0	0	0	0	0	0	0	0	0	0	0	0	0	0	0
0	0	0	0	0	0	0	0	0	0	0	0	0	0	0	0	0	0
65	0	0	0	0	0	0	0	0	0	0	0	0	0	0	0	0	0
0	0	0	0	0	0	0	0	0	0	0	0	0	0	0	0	0	0
0	0	0	0	0	0	0	0	0	0	46	11353	0	0	0	0	0	0
0	0	0	0	0	212	0	0	0	0	0	0	0	0	0	0	0	0
0	423	0	0	0	0	0	0	0	0	0	0	0	0	0	0	0	0
0	0	0	0	0	0	0	0	0	0	0	0	0	0	0	0	0	0
0	0	0	0	0	0	0	0	0	0	0	0	0	0	0	0	0	0
0	0	0	0	0	0	0	0	0	0	0	0	0	0	0	0	0	0
0	0	0	0	0	0	0	0	0	0	0	0	0	0	0	0	0	0
0	831	0	0	0	0	0	0	0	0	0	0	0	0	0	0	0	0
0	0	0	0	0	0	0	0	0	0	0	29	0	42	0	0	0	0
0	0	0	0	0	0	0	13333	0	34	0	0	0	0	0	0	0	0
0	0	0	0	0	0	0	39	0	46	0	60	0	34	0	0	0	0
0	0	0	0	0	0	0	0	0	0	0	0	0	0	0	0	0	0
0	0	0	0	0	0	0	0	0	0	0	0	0	0	0	0	0	0
0	0	52	0	0	0	0	0	0	0	0	0	0	0	0	0	224	0
0	0	0	0	0	0	0	0	0	0	0	0	0	0	0	0	0	0
68	0	0	0	0	0	0	0	0	0	0	0	0	0	0	0	0	0
0	0	0	0	0	0	0	0	0	0	0	0	0	0	0	0	0	0
0	0	0	0	0	0	0	0	0	0	0	0	0	0	0	0	0	0
0	0	0	0	0	0	0	0	0	0	0	0	0	0	0	0	0	0
0	0	0	0	0	0	0	0	0	0	0	0	0	0	0	0	0	0
0	0	0	0	0	0	0	0	0	0	0	0	0	0	0	0	0	0
0	0	0	0	0	0	0	0	54	0	30	69	0	0	0	0	0	0
0	0	0	0	0	0	117	0	0	0	0	0	0	0	0	0	0	0
0	0	0	0	0	0	0	0	0	0	0	0	0	0	0	0	0	0
0	0	0	0	0	0	0	0	0	0	0	0	0	0	0	0	0	0
0	0	0	0	0	0	0	0	0	76	40	32124	93	0	0	0	0	0
0	0	0	0	0	0	0	0	0	0	0	0	0	0	0	0	0	0
0	0	0	0	0	0	0	0	0	0	0	0	0	0	0	0	0	0
226	0	0	0	0	0	0	0	0	37	0	0	0	0	0	0	0	0
0	0	0	0	0	0	0	0	0	0	0	0	0	0	0	0	0	0
0	0	0	0	0	0	0	0	0	0	0	0	0	0	0	0	0	0
0	0	0	0	0	0	0	0	0	0	0	0	0	0	0	0	0	0
0	0	0	0	0	0	0	0	0	0	0	0	0	0	0	0	0	0
0	0	0	0	0	0	0	0	0	0	0	0	0	0	0	0	0	0
0	0	0	0	0	0	0	0	0	0	0	0	0	0	0	0	0	0
0	396	0	206	0	0	0	0	0	0	0	0	0	0	0	0	0	0
0	0	0	0	0	0	0	0	0	0	0	0	0	0	0	0	0	0
0	0	0	0	0	0	0	0	0	0	0	0	0	0	0	0	0	0
0	0	0	0	0	0	0	0	0	0	0	0	0	0	0	0	0	0
0	0	0	0	0	0	0	0	0	0	0	0	0	0	0	0	0	0
0	0	0	0	0	0	0	0	0	0	0	0	0	0	0	0	0	0
0	0	0	0	0	0	0	0	0	0	0	0	0	0	0	0	0	0
0	0	0	0	0	0	0	0	0	93	0	0	0	0	0	0	0	0
0	0	0	0	0	0	0	0	0	0	0	0	0	0	0	0	0	0
0	0	0	0	0	0	0	0	0	0	0	0	0	0	0	0	0	0
0	0	0	0	0	0	0	0	0	0	0	0	0	0	0	0	0	0
184	0	0	0	0	0	0	0	0	0	0	0	0	0	0	0	0	0
0	0	0	0	0	0	0	0	0	0	0	0	0	0	0	0	0	0
0	0	0	0	0	0	0	0	0	0	0	0	0	0	0	0	0	0
0	0	0	0	0	0	0	0	0	0	0	0	0	0	0	0	0	0
0	0	0	0	0	0	0	0	0	0	0	0	0	0	0	0	0	0
0	0	0	0	0	0	0	0	0	0	0	0	0	0	0	0	0	0
0	0	0	0	0	0	0	0	0	0	0	0	0	0	58	0	0	0
0	0	0	0	0	0	0	0	0	52	0	0	0	0	0	0	0	0

0	0	0	0	0	0	0	0	0	0	0	0	0	0	0	0	0	0
0	0	0	0	0	0	0	0	0	0	0	0	0	0	0	69	0	0
0	0	0	0	0	0	0	0	0	0	0	0	0	0	0	0	0	0
0	23324	0	200	0	4798	0	0	0	0	0	0	0	0	0	0	0	0
0	0	0	0	0	0	0	0	0	0	0	0	0	0	0	0	0	0
0	0	0	0	0	0	0	0	149	0	0	0	0	0	0	0	0	0
0	0	0	0	0	0	0	0	0	0	0	0	0	0	0	0	0	0
0	0	0	0	0	0	0	0	0	0	0	0	0	0	0	0	0	0
0	0	0	0	0	0	0	0	0	0	0	0	0	0	0	0	53	0
0	1769	284553	0	1500	3153	662	0	152	84	28	0	79	0	0	70	0	0
0	0	0	0	0	0	0	0	0	0	0	0	0	0	0	0	0	0
0	0	0	0	0	0	0	0	0	0	0	0	0	0	0	0	0	0
0	0	0	0	0	0	0	0	0	0	0	0	0	0	0	0	0	0
0	0	0	0	0	0	0	0	0	0	0	0	0	0	0	0	0	0
0	0	0	0	0	0	0	0	0	0	0	0	0	0	0	0	0	0
0	0	0	0	0	0	0	0	0	0	0	0	0	0	0	0	0	0
0	0	0	0	0	0	0	0	0	0	0	0	0	0	0	0	0	0
0	0	0	0	0	0	0	0	0	0	0	0	0	38	0	59	0	120
0	0	0	0	0	0	0	0	0	0	0	0	0	0	0	0	0	0
0	0	0	0	0	0	0	0	0	0	0	0	0	0	0	0	0	0
0	0	0	0	0	209	0	0	0	0	0	0	0	0	0	0	0	0
0	0	0	0	0	0	0	0	0	0	0	0	0	0	0	0	0	0
0	0	0	0	0	0	0	0	0	0	0	0	0	0	0	0	0	0
0	0	0	0	0	0	0	0	0	0	0	0	0	0	0	0	0	0
0	0	0	0	0	0	0	0	0	0	0	0	0	0	0	0	0	0
0	0	0	90	0	0	0	0	0	0	0	0	0	0	0	0	0	0
0	0	0	0	0	0	0	0	0	0	0	0	0	0	0	0	0	0
0	0	0	0	0	0	0	0	0	0	0	0	0	0	0	0	0	0
0	0	0	0	0	0	0	0	0	0	0	0	0	0	0	0	0	0
0	0	0	0	0	0	0	0	0	0	0	0	0	0	66	0	0	0
0	0	0	0	0	0	0	0	0	0	0	0	0	0	0	0	0	0
0	217	0	0	0	0	0	0	49	0	0	0	0	0	0	0	0	0
0	0	0	0	0	0	0	0	0	0	0	0	0	0	0	0	0	0
0	0	0	0	0	0	0	0	0	0	0	0	0	0	0	0	0	0
0	0	0	0	0	0	0	0	0	0	0	0	0	0	0	0	0	0
0	0	0	0	0	0	0	40	0	0	0	0	0	0	0	0	0	49
0	0	0	0	0	0	0	0	161	0	0	0	0	0	0	0	0	0
0	0	707	4191	371	830	3335	0	0	2389	0	33	0	0	0	0	0	1017
3009	1248	173	8583	158	11371	0	575	259	17487	578	341	607	212	748	69	346502	1962
0	0	0	0	0	0	0	0	0	0	0	0	0	0	0	0	0	0
0	0	0	0	0	0	0	0	0	0	0	0	0	0	0	0	0	0
0	0	0	0	0	0	0	0	0	0	0	0	0	0	0	0	0	0
0	0	0	0	0	0	0	0	0	0	0	0	0	0	0	0	0	0
0	0	0	4059	0	657	865	0	0	1346	0	0	0	0	0	0	0	155
0	0	0	0	0	0	0	0	0	0	0	0	0	0	0	0	0	0
0	0	0	0	0	0	0	0	0	0	0	0	0	0	0	0	0	0
0	0	0	0	0	0	0	0	0	0	0	0	0	0	0	0	0	0
0	0	0	0	0	0	0	0	0	0	0	0	0	0	0	0	0	0
0	207	36067	0	0	0	90	0	0	0	0	0	0	0	0	0	0	0
0	0	0	0	0	0	0	0	0	0	0	0	0	0	0	0	0	0
0	401	34765	0	180	0	206	0	0	0	0	0	0	45	0	0	0	0
0	0	0	0	0	0	0	0	0	0	0	0	0	0	0	0	0	0
113	399	30941	0	192	0	0	0	0	0	33	0	0	0	0	0	0	0
0	0	0	0	0	0	0	0	0	0	0	0	0	0	0	0	0	0
0	0	0	0	0	0	0	0	0	0	0	0	0	0	0	0	0	0
0	0	0	0	0	0	0	0	0	0	0	0	0	0	0	0	0	0
0	0	0	0	0	0	0	0	0	0	0	0	0	0	0	0	0	0
0	0	0	0	0	0	0	0	0	0	0	0	0	0	0	0	0	0
0	0	0	0	0	0	0	0	0	0	0	0	0	0	0	0	0	0
0	0	0	0	0	0	0	0	0	0	0	0	0	0	0	0	0	0
19253	187	70	0	10468	0	0	1210	309	254	279	60	4426	37	75	110	0	309
0	0	0	0	0	0	0	0	0	0	0	0	0	0	0	0	0	0
0	7972	119	178397	171	116033	83	11047	0	0	437	0	42	0	0	0	0	0
0	0	0	0	0	0	0	0	0	0	0	0	0	0	0	0	0	0
0	0	0	0	0	0	0	0	0	0	0	0	0	0	0	0	0	0
0	0	0	0	0	0	0	0	0	0	0	0	0	0	0	0	0	0
0	0	0	0	0	0	0	0	0	0	0	0	0	0	0	0	0	0
0	0	0	0	0	0	0	0	0	0	0	0	0	0	0	0	0	0
0	0	0	0	0	0	0	0	0	0	0	0	0	0	0	0	0	0
0	0	0	0	0	0	0	0	0	0	0	0	0	0	0	0	0	0
0	0	0	0	4801	224	0	20513	0	0	0	0	0	532	384	970335	566	436
0	0	0	0	0	0	0	0	0	0	0	0	0	0	0	0	0	0
0	0	0	0	0	0	0	0	0	0	0	0	0	0	0	0	0	0
0	0	0	0	0	0	0	0	0	0	0	0	0	0	0	0	0	0
0	0	0	0	0	0	0	0	0	0	0	0	0	0	0	0	0	0
0	0	0	0	0	191	0	0	0	0	0	0	0	0	0	0	0	0
0	0	0	0	0	0	0	0	0	0	0	0	0	0	0	0	0	0
0	0	0	0	0	0	0	0	0	0	0	0	0	0	0	0	0	0
0	0	0	0	0	0	0	0	0	0	0	0	0	0	0	0	0	0
0	0	0	0	0	0	0	0	0	0	0	0	0	0	0	0	0	0
0	0	0	0	0	0	0	0	0	0	0	0	0	0	0	0	0	0
0	0	0	0	0	0	0	0	0	0	0	0	0	0	0	0	0	0
44405	8338	226	2032	1386	5054	696	441	163	16492	699	229	182	228	45995	534	119	1035
0	0	0	0	0	0	0	0	0	0	0	0	0	0	0	0	0	0
255	0	0	0	0	0	0	0	0	0	0	0	0	0	0	0	0	0
0	0	0	0	0	0	0	0	0	0	0	0	0	0	0	0	0	0
0	0	0	0	0	0	0	0	0	0	0	0	0	0	0	0	0	0

0	0	0	0	0	0	0	0	0	0	0	0	0	0	0	0	0	0
0	0	0	0	0	0	0	0	0	0	0	0	0	0	0	0	0	0
0	0	0	0	0	0	0	0	0	47	0	32	0	0	0	0	0	0
0	0	0	0	0	0	0	0	0	0	0	0	0	0	0	0	0	0
0	0	0	0	0	0	0	0	0	0	0	0	0	120	0	37	0	0
0	0	0	0	0	0	0	0	0	0	0	0	0	0	0	0	13110	0
0	0	0	0	0	0	0	0	0	0	0	0	0	0	0	0	64	0
0	0	0	0	0	0	0	0	0	0	0	0	0	0	0	0	0	0
0	1202	0	0	0	3073	0	0	0	0	0	0	0	0	0	0	0	0
0	203	28142	0	0	0	0	0	0	0	0	0	0	0	0	0	0	0
0	0	0	0	0	0	0	0	0	0	0	0	0	0	0	0	0	0
0	0	0	0	0	0	0	0	95	40	46	0	93	0	0	0	0	0
0	0	0	0	0	0	0	0	0	0	0	0	0	0	0	0	0	0
133	0	0	211	0	194	0	0	0	41	0	0	0	0	0	0	0	0
0	0	0	0	0	0	0	0	0	0	0	0	0	0	0	0	0	0
0	0	0	0	0	0	0	40	0	0	47	0	0	0	0	72	0	0
0	0	0	0	0	0	0	0	0	0	0	0	0	0	0	0	0	0
0	0	0	0	0	0	0	0	0	0	0	0	0	0	0	0	0	0
62	0	66	0	13868	0	0	0	0	0	0	0	0	0	0	0	0	0
0	0	0	0	0	0	0	0	0	0	0	0	0	0	0	0	0	0
0	0	0	0	0	0	0	0	0	0	0	0	0	0	0	0	0	0
0	0	0	0	0	0	0	0	0	0	0	0	0	0	0	0	0	0
0	0	0	0	0	0	0	0	0	790	0	0	0	0	0	0	0	0
0	0	0	0	0	0	0	0	0	0	0	0	0	0	0	0	0	0
0	0	0	0	0	0	0	0	0	0	0	0	0	0	0	0	0	0
0	0	0	0	0	0	0	0	0	0	0	0	0	0	0	0	0	0
0	0	0	0	0	0	0	0	0	0	0	0	0	0	0	0	0	0
0	0	0	0	0	0	0	0	0	0	0	0	0	0	0	0	0	0
0	0	0	0	0	1424	0	16171	47	0	0	0	0	0	0	53	114	0
0	0	0	0	0	0	0	0	0	0	50	0	37	0	0	0	0	0
0	0	0	0	0	0	0	0	0	0	38	0	48	0	0	0	0	0
115	421	0	0	0	0	0	110	52	47	0	36	103	34	66	185	75	165
0	0	0	0	0	0	0	0	0	0	0	0	0	0	0	0	0	0
0	0	0	0	0	0	0	0	0	0	0	0	0	0	0	0	0	0
0	0	0	0	0	406	0	0	45	0	0	0	0	0	80	48	0	4168
0	0	0	0	0	0	0	0	0	0	0	0	0	0	0	0	0	0
1128	0	0	111	0	0	0	69	0	0	0	0	0	0	0	0	0	0
0	0	0	0	0	0	0	0	0	0	0	0	0	0	0	0	0	0
60	0	0	0	0	0	0	0	0	0	0	0	0	0	0	0	0	0
0	0	0	0	0	0	0	0	0	0	0	0	0	0	0	0	0	0
0	0	0	0	0	0	0	0	0	0	0	0	0	0	0	0	0	0
0	0	0	0	0	0	0	34	0	0	0	0	0	103	0	247	69	244
0	2376	0	0	0	0	0	0	0	0	0	0	0	0	0	0	0	0
0	0	0	0	0	0	0	0	0	0	0	0	0	0	0	0	0	0
0	0	0	0	0	0	0	0	0	2860	0	0	0	0	0	0	0	0
0	0	0	0	0	0	0	0	107	0	0	11962	0	33	0	0	68	0
0	0	0	0	0	0	0	32	0	0	0	0	0	0	0	0	0	52
0	0	0	0	0	0	0	0	0	0	0	175	0	0	0	0	0	0
0	0	0	0	0	0	0	23384	0	0	0	0	0	37	0	0	62	105
0	0	48	1458	0	424	989	0	0	198	0	0	0	0	0	0	1582	114
0	0	0	0	0	0	0	0	0	0	0	0	0	0	0	0	0	0
0	0	0	0	0	0	0	0	0	0	0	0	0	0	0	0	0	0
0	0	0	0	0	0	0	0	0	0	0	0	0	0	0	0	0	0
0	0	0	0	0	0	0	0	0	0	0	34	0	0	0	0	0	0
0	0	0	0	0	0	0	32	0	0	0	0	0	162	70	147	52	103
0	0	0	0	0	0	0	153	0	2312	0	0	0	42	0	60	26980	0
0	0	0	0	0	0	0	0	0	0	0	0	0	0	0	0	0	0
0	0	0	0	0	0	0	0	0	0	0	0	0	0	0	0	0	0
0	0	0	0	0	0	0	0	0	0	0	0	0	0	0	0	0	0
0	0	0	0	0	0	0	0	0	0	0	0	0	0	0	0	0	0
0	0	0	0	0	0	0	0	0	0	0	0	0	0	0	0	0	0
0	0	0	0	0	0	0	0	0	0	0	0	0	0	0	0	0	0
0	0	0	0	0	2262	0	0	0	0	0	0	0	0	0	0	0	0
0	0	0	0	0	0	0	0	0	0	0	0	0	0	0	0	0	0
0	0	0	0	0	0	0	0	0	0	0	0	0	0	0	0	0	0
0	0	0	0	0	0	0	0	0	0	0	0	0	0	0	0	0	0
0	0	0	0	0	0	0	0	0	0	0	0	0	0	0	0	0	0
0	0	0	0	0	0	0	37	0	0	0	0	0	0	0	0	0	0
0	0	0	0	0	0	0	0	0	0	0	0	0	0	0	0	0	0
0	0	0	0	0	0	0	0	0	0	0	0	0	0	0	0	0	0
0	0	0	0	0	0	0	0	0	0	0	0	0	0	0	0	0	0
0	0	0	0	0	0	0	0	0	0	0	0	0	0	0	0	0	0
0	0	0	0	0	0	0	0	0	0	0	0	233	0	0	0	0	0
0	0	0	0	0	222	0	0	0	0	0	0	0	0	0	0	0	0
0	0	0	0	0	0	0	0	0	0	0	0	0	0	0	0	0	0
0	0	0	0	0	0	0	0	0	0	0	0	0	0	0	0	0	0
2561	420	0	0	0	0	101	30	0	360	87	0	0	0	405	0	0	0
0	0	0	0	0	0	0	0	0	0	0	0	0	0	0	0	0	0
0	0	0	0	0	0	0	0	0	0	0	20119	61	0	0	0	0	0
0	0	0	0	0	0	0	0	39	4975	35	0	100	0	0	0	0	0
0	0	0	0	0	0	0	0	0	0	0	0	0	0	0	0	0	0
0	0	0	0	0	0	0	103	0	0	0	0	0	212	63	59	419	586
0	0	0	0	0	0	0	0	0	0	0	0	0	0	0	0	0	0
0	0	0	0	0	0	0	0	0	0	0	0	0	0	0	0	0	0
0	0	122	0	0	0	0	0	0	0	0	0	0	0	0	0	0	0
341	218	0	0	0	394	0	0	0	0	0	0	0	0	0	0	0	0
0	0	0	0	0	0	0	0	0	0	0	0	0	0	0	0	0	0

0	0	0	0	0	0	0	0	0	0	0	0	0	0	0	0	0	0
0	0	0	0	0	0	0	0	0	0	0	0	0	0	0	0	0	0
0	0	0	0	0	0	0	0	0	0	0	0	0	0	0	0	0	0
0	0	0	0	0	0	0	0	53	0	0	0	0	0	0	0	0	0
0	0	0	0	0	0	0	0	0	0	0	0	0	0	0	0	0	0
0	0	0	0	0	0	0	0	0	0	0	0	0	0	0	0	0	0
0	0	0	0	0	0	0	0	0	0	0	0	0	0	0	0	0	0
0	0	0	0	0	0	0	0	0	0	0	0	0	0	0	0	0	0
0	0	0	0	0	0	0	0	0	0	0	0	0	0	0	0	0	0
0	0	0	0	0	0	0	0	0	0	0	0	0	0	0	0	0	0
0	0	0	0	0	0	0	0	0	0	0	0	0	0	0	0	0	0
0	2033	0	0	0	0	0	0	0	0	0	0	0	0	0	0	0	0
0	222	0	0	0	0	0	0	0	0	0	33	0	0	0	0	0	0
0	0	0	0	0	0	300	0	0	0	0	0	0	0	0	0	0	0
0	0	0	0	0	0	0	45	0	0	39	35	0	0	0	0	0	0
0	0	0	0	0	0	0	0	0	0	0	0	0	0	0	0	0	0
0	0	26117	0	0	0	0	0	0	0	0	0	0	0	0	0	0	0
0	11732	0	0	0	0	0	0	0	0	0	0	0	0	0	0	0	0
0	0	0	0	0	0	0	0	0	0	0	0	0	0	0	0	0	0
0	0	0	0	0	0	0	0	0	0	0	0	0	0	0	0	0	0
0	0	0	0	0	0	0	0	0	0	0	0	0	0	0	0	0	0
0	0	0	0	0	0	0	0	0	0	0	0	0	0	0	0	0	0
0	0	0	0	0	0	0	0	0	0	0	0	0	0	0	0	0	0
0	0	0	0	0	0	0	0	0	0	0	0	0	0	0	0	0	0
0	0	0	0	0	0	0	0	0	0	0	0	0	0	0	0	0	0
0	0	0	0	0	0	0	0	0	0	0	0	0	0	0	0	0	0
0	0	0	0	0	0	0	0	0	0	0	0	0	0	0	0	0	0
60	3913	0	0	0	0	0	0	0	0	0	0	0	0	0	0	0	0
0	0	0	0	0	0	0	0	269	78	184	57	0	0	0	0	0	0
0	0	0	0	0	0	0	32	0	0	43	0	53	0	0	100	0	0
0	0	0	0	0	0	0	0	0	0	0	0	0	0	0	0	0	0
385	0	0	0	0	0	0	0	0	0	0	0	0	0	0	0	0	0
186616	240513	2370	10804	80612	14600	3351	39781	6101	59500	497828	429373	798658	785022	11145	3181	25084	112456
0	0	0	0	0	0	0	0	0	0	0	0	0	0	0	0	0	0
0	200	36659	0	0	0	0	0	0	0	0	0	0	0	0	0	0	0
0	0	0	0	0	0	0	0	0	0	0	0	0	0	0	0	0	0
0	0	0	0	0	0	0	0	0	2691	0	0	0	0	0	0	0	0
0	0	0	0	0	0	0	0	0	0	0	0	0	0	0	0	0	0
0	0	0	0	0	0	0	0	0	0	0	0	0	0	0	0	0	0
0	0	0	0	0	0	0	0	0	0	0	0	0	0	0	0	0	0
54	411	0	0	0	0	0	0	0	0	0	0	0	0	0	0	426	46
0	0	0	0	0	0	0	0	0	0	0	0	0	0	0	0	0	0
0	0	0	0	0	0	0	0	0	0	0	0	0	0	0	0	0	0
0	0	0	0	0	0	0	0	0	0	0	0	0	0	0	0	0	0
0	0	0	0	0	0	0	0	0	0	0	0	0	0	0	0	0	0
0	0	0	0	0	0	0	0	0	0	0	0	0	0	0	0	0	0
0	0	0	0	0	0	0	0	0	0	0	0	0	0	0	0	0	0
0	0	0	106	0	188	0	0	0	0	0	0	0	0	0	0	0	0
19516	986	184	592	183	627	191	182	207	8839	1958	176	138	161	7785	297	273	2490

Control 083	Control 090	Control 092	Control 075	Control 081	Control 080	Control 079	Control 078	Control 077	Control 076	Control 074	Control 073	Control 072	Control 071	Control 070	Control 069	Control 097	Control 068
877581	865010	430759	915061	796017	575416	809669	902888	730075	799427	840961	286531	725708	860955	923880	645809	977501	917938
86024	82430	550300	32591	148499	310517	140264	41577	187424	135732	100161	619814	236310	101221	27703	241805	9193	71440
0	0	0	0	0	0	319	0	0	0	0	0	0	0	0	0	0	0
30192	19744	18663	52291	54709	19868	47669	54702	60434	64762	58829	93413	37982	29961	47675	108259	13050	10349
6203	32816	278	57	775	94199	2079	833	22067	79	49	242	0	7863	742	4127	256	234
0	0	0	0	0	0	0	0	0	0	0	0	0	0	0	0	0	39
0	0	0	0	0	0	0	0	0	0	0	0	0	0	0	0	0	0
Control 083	Control 090	Control 092	Control 075	Control 081	Control 080	Control 079	Control 078	Control 077	Control 076	Control 074	Control 073	Control 072	Control 071	Control 070	Control 069	Control 097	Control 068
0	0	0	0	0	0	0	0	0	28	0	0	42372	146	0	0	0	0
34	0	0	0	0	45	0	0	17457	0	0	0	0	0	0	0	253	0
581296	425996	160985	154353	11977	28266	516301	4134	99822	800	16660	412	62442	298532	9005	117343	7194	15442
0	0	0	0	0	0	0	0	0	0	0	0	0	0	0	0	0	0
0	0	0	0	176	0	0	0	0	0	0	0	0	0	242	0	0	50
0	0	0	0	0	0	0	0	0	0	0	0	0	0	0	0	0	0
0	0	0	0	0	0	0	0	0	0	0	0	0	0	0	0	0	0
0	0	0	0	0	0	0	0	0	0	0	0	0	0	0	0	0	0
0	0	0	0	0	0	0	0	0	0	0	0	0	0	0	0	178	0
0	92	0	0	0	0	0	0	0	0	0	0	0	0	0	0	0	0
0	0	0	0	0	0	0	0	0	0	0	0	0	0	0	0	0	0
23960	164	450	0	82	91	48	0	0	0	0	0	0	0	150	349	440	106
0	0	0	0	0	0	0	0	0	0	0	0	0	0	0	0	0	0
0	0	0	0	0	0	0	0	0	0	0	0	0	0	0	0	0	0
0	0	0	0	0	0	0	0	0	0	0	0	0	0	0	0	0	0
16312	7113	135	0	205	0	1617	0	7126	0	0	0	989	6534	3980	630	0	118
0	0	0	0	0	0	0	0	0	0	0	0	0	0	0	0	0	0
0	0	0	0	0	0	0	0	0	0	0	0	0	0	0	0	54	0
0	0	0	0	0	0	0	0	0	0	0	0	0	0	0	0	0	0
0	0	234	0	0	0	0	52	0	0	0	0	0	0	65	100	250	11753
0	0	0	0	0	0	0	0	0	0	0	0	0	0	0	0	0	0
0	0	0	0	0	0	0	0	0	0	0	0	0	0	0	0	0	0
0	0	0	0	0	0	0	0	0	0	0	0	0	0	0	0	0	0
0	0	0	0	0	0	0	0	0	0	0	0	0	0	0	0	0	0
631	256	888	632	654	1003	595	914	1630	93	294	130	403	460	391	777	1015	41
0	0	0	0	48	0	0	0	0	0	0	0	0	0	0	0	0	0
0	0	0	0	0	0	0	48	0	0	0	0	0	299	0	0	0	0
0	0	0	0	0	0	0	0	0	0	0	0	0	0	0	0	0	0
0	0	0	0	0	0	0	0	0	0	0	0	0	0	0	0	72	0
25055	306248	1614	109	799	23414	8923	44569	296695	29	12373	649	0	38682	683660	60255	1796	4031
0	0	0	0	0	0	0	0	0	0	0	0	0	0	0	81	0	0
0	0	0	0	0	0	0	0	0	0	0	0	0	0	0	0	0	0
0	169	0	0	0	0	0	0	0	0	0	0	0	0	0	0	132	0
0	0	0	0	0	0	0	0	0	0	0	0	0	0	0	0	0	0
0	0	0	0	0	0	0	0	0	0	0	0	0	0	0	0	0	0
0	0	0	0	0	0	307	0	0	0	0	0	0	0	0	0	0	0
0	0	0	0	0	0	0	0	0	0	0	0	0	0	0	90	0	0
0	0	0	0	0	0	0	0	0	0	0	0	0	0	0	0	0	0
0	0	0	0	0	0	0	0	0	0	0	0	0	0	0	0	0	0
0	0	0	0	0	0	0	0	0	0	0	0	0	0	0	0	0	0
317	83	0	0	0	0	0	0	0	0	0	0	0	158	0	98	0	0
0	0	75	0	0	0	0	0	0	0	0	0	0	0	0	0	0	0
0	0	0	0	0	0	0	0	0	0	0	0	0	0	0	0	0	0
0	0	0	0	0	0	0	0	0	0	0	0	0	0	0	0	0	0
0	0	0	0	0	0	0	0	0	0	0	0	0	0	0	0	0	0
58	0	0	0	143	38	55	52	70	0	0	0	0	0	0	0	0	0
0	0	0	0	0	0	0	0	0	0	0	0	0	0	0	0	0	0
264	345	539	0	509	4157	386	69072	51713	0	1630	1090	0	10066	1447	895	763	1456
0	0	0	0	0	26857	0	0	0	0	0	0	0	0	0	0	0	0
0	0	0	0	0	0	0	0	0	0	0	0	0	0	0	0	73	0
0	0	0	0	0	0	0	0	0	0	0	0	0	0	0	102	0	47
0	0	0	0	0	0	0	0	0	0	0	0	0	0	0	0	0	0
0	0	0	0	0	0	0	0	0	0	0	0	0	0	0	0	0	0
0	0	0	0	0	0	0	0	0	0	0	0	0	0	0	0	0	0
0	0	0	0	0	0	0	0	0	0	0	0	0	0	0	0	0	0
0	0	0	0	0	0	0	0	0	0	0	0	0	0	0	0	0	0
0	0	0	0	0	0	0	0	0	0	0	0	0	0	0	0	0	0
1717	0	0	0	0	0	0	0	0	0	0	0	0	0	0	0	0	0
0	0	0	0	0	0	0	0	0	0	0	0	0	0	59	0	0	41
0	0	0	0	0	0	0	0	0	0	0	0	0	0	0	0	81	0
12717	1269	511	77728	134	53	102	85	0	162	204	125	146	139	5481	2151	639	308
0	0	0	0	0	0	0	0	0	0	0	0	0	0	69	0	0	0
0	0	0	0	0	0	0	0	0	0	0	0	0	0	0	0	0	0
0	0	0	0	0	0	0	0	0	0	0	0	0	0	0	0	0	0
0	0	0	0	0	0	0	0	0	0	0	0	0	0	0	0	0	0
10667	0	0	0	0	0	0	0	123	0	0	0	0	0	0	0	0	0
0	0	0	0	0	40	26499	0	52	0	0	0	0	0	0	0	0	0
0	0	0	0	0	0	0	0	0	0	0	0	0	0	93	88	0	0
0	0	0	0	0	0	0	0	0	0	0	0	0	0	0	0	0	0

0	0	0	0	0	0	0	0	0	0	0	0	0	0	0	0	0	0
0	0	0	0	0	0	0	0	0	0	0	0	0	0	0	0	0	0
0	0	0	0	0	0	0	0	0	0	0	0	0	0	0	0	0	0
0	0	0	0	0	0	0	0	0	0	0	0	0	0	0	95	0	0
0	0	0	0	0	0	0	0	0	0	0	0	0	0	0	0	0	0
0	0	0	0	0	0	0	0	0	0	0	0	0	0	0	0	0	0
37	0	153	249	0	0	0	0	4147	325	449	138	512	585	75	0	69	1085
0	0	0	0	0	0	319	0	0	0	0	0	0	0	0	0	0	0
0	0	0	0	0	0	0	0	0	0	0	0	0	0	0	0	0	0
0	0	0	0	0	0	0	0	0	0	0	0	0	0	0	0	0	0
0	0	0	0	0	0	107	0	0	0	0	0	0	0	70	0	0	0
0	0	0	0	0	0	0	0	0	0	0	0	0	0	0	0	0	0
14114	5015	181244	188	36726	69598	13142	144	25649	32	54	0	43677	70944	1157	922	3095	19602
0	0	0	0	0	0	0	0	0	0	0	0	0	0	0	0	0	0
0	0	0	0	0	0	0	0	0	0	0	0	0	0	0	0	0	0
0	0	0	0	0	0	0	0	0	0	0	0	0	0	0	0	0	0
0	0	0	0	0	0	0	0	0	0	0	0	0	0	0	0	0	0
0	0	0	0	0	0	0	0	0	0	0	0	0	0	0	0	0	0
0	0	0	0	0	0	0	0	0	0	0	0	0	0	0	0	0	0
0	0	0	0	0	0	0	0	0	0	0	0	0	0	0	0	0	0
0	0	0	0	0	0	0	0	0	0	0	0	0	0	0	0	0	0
0	0	0	0	0	0	0	0	0	0	0	0	0	0	0	0	0	0
74	138	149	0	93	0	95	0	64	167	59748	72	272	0	0	0	0	0
0	0	0	0	33987	91	0	48	60	0	0	0	0	0	0	0	0	0
0	78	0	0	0	0	0	0	0	0	0	0	0	0	0	0	0	0
0	1377	0	0	0	0	0	0	0	0	0	0	0	0	0	0	0	0
0	0	0	0	0	0	0	0	0	0	0	0	0	0	0	0	0	0
21527	5036	228216	345	25804	111391	381	40186	35799	134789	39475	63890	174168	54651	14715	1369	2625	24988
0	0	0	0	0	0	0	0	0	0	0	0	0	0	0	0	0	0
0	0	0	0	0	0	0	0	0	0	0	0	0	0	0	0	0	0
41	0	0	0	0	0	0	59	0	0	0	0	0	0	299	0	0	0
0	0	0	0	0	0	0	0	0	0	0	0	0	0	0	0	0	0
0	0	0	0	0	0	0	0	0	0	0	0	0	0	0	0	0	0
0	0	0	0	0	0	0	0	0	0	0	0	0	0	0	0	0	0
25	72	150	0	0	14574	0	0	69	0	0	0	0	0	0	0	70	0
0	0	83	0	0	0	0	0	0	0	0	0	0	0	0	0	0	0
0	0	0	0	0	0	0	0	0	0	0	0	0	0	0	0	0	0
0	0	0	0	0	0	0	0	0	0	0	0	0	0	0	0	0	0
0	70	84	0	0	0	0	0	0	0	0	0	0	0	0	188	77	78
0	0	0	0	0	0	0	0	0	0	0	0	0	0	0	0	0	0
0	0	0	0	0	0	0	0	0	0	0	0	0	0	0	0	0	0
0	0	0	0	0	0	0	0	0	0	0	0	0	0	0	0	0	0
0	0	0	0	0	0	0	0	0	0	0	0	0	0	0	82	0	49
0	234	308	53934	0	0	0	0	0	133	95	0	0	140	0	0	373	0
0	0	0	0	47	38	0	0	0	0	0	0	0	0	0	0	0	0
0	0	0	0	0	0	0	0	0	0	0	0	0	0	0	0	0	0
0	0	0	0	0	0	0	0	0	0	0	0	0	0	0	0	0	0
0	241	113112	0	0	0	0	0	0	0	0	0	0	0	0	0	70	0
0	0	255	0	36	111	0	0	0	0	0	0	0	0	1082	882	0	552
0	0	0	0	0	0	0	0	0	0	0	0	0	0	0	0	0	0
6203	32816	278	57	599	94064	2079	833	21959	79	49	242	0	7863	290	3771	127	0
0	0	0	0	0	48	0	0	66	0	0	0	0	0	0	0	0	0
0	0	0	0	0	0	0	0	0	0	0	0	0	0	0	0	0	0
0	0	0	0	0	0	0	0	0	0	0	0	0	0	0	0	0	0
0	0	0	0	0	0	0	0	0	0	0	0	0	0	0	0	0	0
0	0	0	0	0	0	0	0	0	0	0	0	0	0	0	0	0	0
0	0	0	0	0	0	0	0	0	0	0	0	0	0	0	0	0	0
0	0	0	0	0	0	0	0	0	0	0	0	0	0	0	0	0	0
0	0	0	0	0	0	0	0	0	0	0	0	0	0	0	0	0	0
0	0	0	0	0	0	0	0	0	0	0	0	0	0	0	0	0	0
0	86	0	0	0	0	0	0	4109	0	0	0	0	0	0	0	199	0
0	0	0	0	0	0	0	0	0	0	0	0	0	0	0	0	0	0
5565	2669	0	0	0	53	0	40	69	0	0	0	0	0	0	91	0	0
0	0	0	0	0	0	0	0	0	0	0	0	0	0	0	0	0	0
0	0	0	0	0	0	0	0	597	0	0	0	0	0	0	0	0	0
0	0	0	0	0	0	0	0	0	0	0	0	0	0	0	0	0	0
0	0	0	0	0	0	0	0	0	0	0	0	0	0	0	0	0	0
0	0	0	0	0	0	0	0	0	0	0	0	0	0	0	0	0	0
0	0	0	0	0	0	0	0	0	0	0	0	0	0	0	0	0	0
0	0	0	0	0	0	0	0	0	0	0	0	0	0	0	0	0	0
0	0	0	0	0	0	0	0	0	0	0	0	0	0	0	0	0	0
0	0	0	0	0	0	0	0	0	0	0	0	0	0	0	0	0	0
0	0	0	0	0	0	0	0	0	0	0	0	0	0	0	0	0	0
0	0	0	0	0	0	0	0	0	0	0	0	0	0	0	0	0	0
3359	1548	68	0	318	0	774	0	52	0	0	0	233	1740	167	63401	67	84
10473	18284	284	0	118	185	598	108	127	37	59	0	3327	33911	404	881	522	125
0	169	135	0	0	0	0	0	0	0	0	0	0	0	0	0	256	0
0	0	0	0	0	0	0	0	0	0	0	0	0	0	0	0	0	0
0	0	0	0	0	0	0	0	0	0	0	0	0	0	0	0	0	0
0	0	0	0	0	0	2687	0	0	0	0	0	0	0	0	0	51	0
0	0	0	0	0	0	0	0	0	0	0	0	0	0	0	178	0	33
0	0	0	0	0	0	0	0	0	0	0	0	0	0	0	0	0	0
0	0	0	0	0	0	0	0	0	0	0	0	0	0	0	0	0	0
24627	17075	18663	52291	54709	19815	47669	54662	60365	64762	58829	93413	37982	29961	47675	108168	13050	10349
103	1254	83	0	45	19707	0	23392	191	34	35174	0	0	145	0	0	0	0
0	0	0	0	0	0	0	89	0	0	0	0	0	0	0	0	0	0
0	0	0	0	0	8922	0	0	0	0	0	0	0	0	0	0	0	0

0	0	0	0	0	0	0	0	0	0	0	0	0	0	0	0	0	0
0	0	0	0	524	0	257	0	0	0	0	0	0	0	1751	2022	215	879
0	0	0	0	0	0	0	0	0	0	0	0	0	0	0	0	0	0
0	0	0	0	0	1076	60	0	113	0	0	0	0	0	0	283	0	0
0	0	0	0	0	0	0	0	0	0	0	0	0	0	0	0	0	0
0	0	0	0	0	0	0	0	0	0	0	0	0	0	0	0	0	0
0	0	0	0	0	0	0	0	0	0	0	0	0	0	0	0	0	0
0	0	0	0	0	0	0	0	0	0	0	61	0	0	0	0	0	0
0	0	0	0	0	0	0	0	0	0	55	0	0	0	0	0	0	0
0	0	0	0	0	0	0	0	0	0	0	0	0	0	0	0	0	0
0	0	0	0	0	0	0	0	0	0	0	0	0	0	0	0	0	0
0	0	0	0	0	0	0	0	0	0	0	0	0	0	0	0	129	0
0	0	0	0	0	0	0	0	0	0	0	0	0	0	0	0	0	0
0	0	0	0	0	0	0	0	0	0	0	0	0	0	167	295	0	21907
0	0	0	0	0	0	0	0	0	0	0	0	0	0	0	0	0	0
0	0	0	0	0	0	0	0	0	0	0	0	0	0	0	0	0	0
0	0	0	0	0	0	0	0	0	0	0	0	0	0	0	0	0	0
0	0	0	0	0	0	0	0	0	0	0	0	0	0	0	0	0	0
0	0	0	0	0	0	0	0	0	0	0	0	0	0	0	0	0	0
0	0	0	0	0	49	0	0	0	0	0	0	0	0	0	0	0	0
0	0	0	0	0	0	0	0	0	0	0	0	0	129	0	0	0	92
0	0	0	0	0	0	0	0	0	0	0	0	0	0	0	0	0	0
0	0	0	0	0	0	0	0	0	0	0	0	0	0	0	0	0	0
243	504	0	324	288	678	0	78	0	156	114	329	0	169	176	175	0	42
0	0	0	0	0	0	0	0	0	0	0	0	0	0	0	97	0	0
31	0	0	0	0	0	41	0	5881	0	0	0	0	0	0	0	0	0
0	0	0	0	0	0	0	0	0	0	0	0	0	0	0	0	0	0
0	0	0	0	0	0	0	0	0	0	0	0	0	0	0	0	0	0
0	0	0	0	0	0	0	0	0	0	0	0	0	0	0	0	0	0
0	0	0	0	0	0	0	0	0	0	0	0	0	0	0	0	0	0
0	0	0	0	0	0	0	0	0	0	0	0	0	0	0	0	0	0
0	0	0	0	0	0	0	0	0	0	0	0	0	0	0	0	0	0
0	0	0	0	0	0	0	0	0	0	0	0	0	0	0	0	0	0
0	0	0	0	0	0	0	0	0	0	0	0	0	0	0	0	0	0
0	0	0	0	0	0	0	0	0	0	0	0	0	0	0	0	0	0
37	0	0	64	1814	38	0	44	225	214	679	0	0	0	0	0	67	0
0	0	0	0	0	0	0	0	0	0	0	0	0	0	0	0	0	0
0	0	0	0	0	0	0	0	0	0	0	0	0	0	0	0	0	0
0	0	0	0	0	56	0	56	0	0	0	0	0	0	0	0	0	0
0	0	0	0	0	0	0	0	0	0	0	0	0	0	0	0	0	0
0	0	0	0	0	0	0	0	0	0	0	0	0	0	0	0	0	0
0	0	0	0	0	0	0	0	0	0	0	0	0	0	0	0	0	0
0	0	0	0	0	0	0	0	0	0	0	0	0	0	0	0	0	0
0	0	0	0	0	0	0	0	0	0	0	0	0	0	0	0	0	0
0	0	0	0	0	0	0	0	0	0	0	0	0	0	0	0	0	0
0	0	0	0	0	0	0	0	0	0	0	0	0	0	0	0	0	0
0	0	0	0	0	0	0	0	0	0	0	0	0	0	0	0	0	0
0	0	0	0	0	0	0	0	0	0	0	0	0	0	0	0	0	0
87	343	0	0	143	46137	0	0	0	0	0	0	0	0	0	0	73	0
0	0	0	0	0	39279	0	37	63	0	0	0	0	0	0	0	0	0
0	0	0	0	0	0	0	0	0	0	0	0	0	0	0	0	0	0
0	0	0	0	0	0	0	0	0	0	0	0	0	0	0	0	0	0
0	0	0	0	0	0	0	0	0	0	0	0	0	0	0	0	0	0
0	80	0	0	0	0	0	0	0	81	35818	0	0	0	0	0	0	43
0	0	0	0	0	0	0	0	0	0	0	0	0	0	0	0	0	0
1761	0	0	0	0	0	0	0	0	0	0	0	0	0	0	0	0	0
0	0	0	0	0	0	0	0	0	0	0	0	0	0	0	0	0	0
0	0	0	0	0	0	0	0	0	0	0	0	0	0	0	0	0	0
0	0	0	0	0	0	0	0	65	0	0	0	0	0	0	0	66	0
0	0	0	0	0	0	0	0	0	0	0	0	0	0	0	0	0	0
0	0	239	0	0	0	0	0	0	0	0	0	0	0	0	0	0	0
0	0	0	0	0	0	0	0	0	0	0	0	0	0	0	0	0	0
0	0	85	0	0	0	0	0	0	0	0	0	0	0	0	0	0	0
0	0	64	0	0	0	0	0	0	0	0	0	0	4093	0	0	0	0
0	0	0	0	0	0	0	0	0	0	0	0	0	0	0	0	0	0
0	0	0	0	0	0	0	0	0	0	0	0	0	0	0	0	0	0
0	0	0	0	0	0	0	0	0	0	0	0	0	0	0	0	0	0
0	0	0	0	0	0	0	0	0	0	0	0	0	0	0	0	0	0
0	0	0	0	0	0	0	0	0	0	0	0	0	0	0	0	0	0
36	0	0	0	0	0	0	0	0	0	0	0	0	0	0	0	0	0
0	0	0	0	0	0	0	0	0	0	0	0	0	0	0	0	0	0
0	0	0	0	0	0	0	0	0	0	0	0	0	0	0	0	0	0
0	0	0	0	27701	0	0	0	0	0	0	0	0	0	0	0	0	0
21772	4233	51422	31876	42689	133882	72227	646	135734	503	765	555458	28021	8118	0	77859	1397	8915
0	1247	0	0	0	0	0	0	0	0	0	0	0	0	0	0	0	0
0	937	0	0	0	0	0	0	0	0	0	0	0	0	0	0	0	0
0	0	0	0	0	0	0	0	0	0	0	0	0	0	0	0	0	0
0	0	0	0	0	0	0	0	0	0	0	0	0	0	0	0	0	0
86	1136	1932	59006	89	0	9510	0	55	39	44	73	284	167	0	0	1163	42
0	0	0	0	0	0	0	0	0	0	0	0	0	0	0	0	0	0
0	0	0	0	0	0	0	0	0	0	0	0	0	0	0	0	0	0
0	0	0	0	0	0	0	0	0	0	0	0	0	0	0	0	0	0
0	0	0	0	0	0	0	0	0	0	0	0	0	0	0	0	0	0
0	0	0	0	0	135	0	0	108	0	0	0	0	0	210	356	0	184
0	0	0	0	0	0	0	0	0	0	0	0	0	0	0	0	0	0
0	0	0	0	0	0	0	0	0	0	0	0	0	0	0	0	0	0
0	1417	744	0	0	0	0	0	0	0	0	65	0	0	0	0	1034	0

0	0	0	0	0	0	0	0	0	0	0	0	0	0	0	0	0	0
0	0	0	0	0	0	0	0	0	0	0	0	0	0	0	0	0	0
0	0	0	0	0	0	0	0	0	0	0	0	0	0	0	0	0	0
0	0	0	0	0	0	0	0	0	0	0	0	0	0	0	0	0	0
0	0	0	0	0	0	0	0	0	0	0	0	0	0	0	0	0	0
0	0	0	0	0	0	0	0	0	0	0	0	0	0	0	0	0	0
0	0	0	0	0	0	0	0	0	0	0	0	0	0	0	0	0	0
0	0	0	0	0	0	0	0	0	0	0	0	0	0	0	0	0	0
0	0	0	0	0	0	0	0	0	0	0	0	0	0	0	0	0	0
0	0	0	0	0	0	0	0	0	0	0	0	0	0	0	0	0	0
0	996	0	0	0	0	0	0	0	0	0	0	0	0	0	0	0	0
0	95	0	0	0	0	0	0	0	0	0	0	0	0	0	0	0	0
36	0	0	0	39	0	49	0	15076	0	0	0	0	0	0	0	0	0
0	0	65	0	0	0	0	0	0	0	0	0	0	0	0	0	117	0
0	0	0	0	0	35	14029	0	0	0	0	0	0	0	0	0	0	0
33	0	0	0	2767	0	0	0	0	0	0	0	0	0	0	0	0	0
0	0	0	0	0	0	0	0	0	0	0	0	0	0	0	0	0	0
0	0	0	0	0	0	0	0	0	0	0	0	0	0	81	0	0	0
0	0	0	0	0	0	0	0	0	0	0	0	0	0	0	0	0	0
0	0	0	0	0	0	0	0	0	0	0	0	0	0	0	0	0	0
0	0	0	0	0	0	0	0	0	0	0	0	0	0	0	0	0	0
0	0	0	0	0	0	0	0	0	55	0	73	43284	0	0	0	0	0
3658	0	82	0	0	37	0	8143	608	0	0	0	0	152	70	0	0	0
0	0	0	0	0	0	0	0	0	0	0	0	0	0	0	0	0	0
0	0	0	0	0	0	0	0	0	0	0	0	0	0	0	0	0	0
0	0	0	0	0	0	0	0	0	0	0	0	0	0	0	0	0	0
0	0	0	0	0	0	0	0	0	0	0	0	0	0	0	0	0	0
6388	12111	35814	58182	972	163	6763	89	400	3040	543	218	654	7738	57413	1162	1515	170
0	0	0	0	0	0	0	0	0	0	0	0	0	0	0	0	0	0
0	0	0	0	0	0	0	0	0	0	0	0	0	0	0	0	0	0
0	0	0	0	0	0	0	0	0	0	0	0	0	0	0	0	0	0
0	0	0	0	0	0	0	0	0	0	0	0	0	0	0	0	0	0
0	0	0	0	0	0	0	0	0	0	0	0	0	0	0	0	0	0
64	0	0	0	0	0	0	28	0	0	0	0	0	0	0	0	0	0
462	0	0	0	0	0	221	0	0	0	0	0	112	735	0	95	64	0
0	0	0	0	0	0	0	0	0	0	0	0	0	0	0	0	0	0
0	0	0	0	0	0	0	0	0	0	0	0	0	0	0	0	0	0
0	0	0	0	0	0	0	0	0	0	0	0	0	0	0	0	0	0
2644	0	35381	0	37	10274	0	0	0	0	0	0	0	0	0	0	0	0
3164	0	0	0	0	0	0	0	0	0	0	0	0	0	0	0	0	0
0	0	0	0	0	54	11674	0	0	0	0	0	0	0	0	0	0	0
29	0	0	0	49	40	0	47	47	0	0	0	0	0	0	0	0	0
0	72	0	0	0	0	0	0	0	0	0	0	0	0	0	0	0	0
0	0	0	0	0	0	0	0	0	0	0	0	0	0	0	0	0	0
0	0	0	0	0	0	0	0	0	0	0	0	0	0	0	0	0	0
0	0	0	0	0	0	0	0	0	0	0	0	0	0	0	0	0	0
0	0	0	0	0	0	0	0	0	0	0	0	0	0	0	0	0	0
0	0	0	0	0	0	0	0	0	0	0	0	0	0	0	0	0	0
0	0	0	0	0	0	0	0	0	0	0	0	0	0	0	0	0	0
0	0	0	0	0	0	0	0	0	0	0	0	0	0	0	0	0	0
0	0	0	0	45	0	0	0	0	0	0	0	0	0	147	0	0	0
0	0	0	0	0	0	0	0	0	0	0	0	0	0	0	0	0	0
0	0	0	0	0	0	0	0	0	0	0	0	0	0	0	0	0	0
0	0	0	0	0	0	0	0	0	0	0	0	0	0	0	0	0	0
0	0	0	0	0	0	0	0	0	0	0	0	0	0	0	0	0	0
0	0	0	0	0	0	0	0	0	0	0	0	0	0	0	0	0	0
0	0	0	0	0	0	0	0	0	0	0	0	0	0	0	0	0	0
0	0	76	0	0	0	0	0	0	0	0	0	0	0	0	0	76	0
0	0	0	0	142	54	0	0	0	0	0	0	0	0	0	0	0	0
0	0	0	0	0	0	0	0	0	0	0	0	0	0	10754	0	0	0
0	0	0	0	0	0	0	0	0	0	0	0	0	0	0	0	0	0
0	0	0	0	0	0	0	0	0	0	0	0	0	0	0	0	0	37
0	0	0	0	0	0	0	0	0	0	0	0	0	0	0	0	0	0
38	498	0	0	0	0	25258	0	62	80	0	0	30522	150	696	160223	0	541
0	0	0	0	0	0	0	0	0	0	0	0	0	0	0	0	0	0
0	0	0	0	0	0	0	0	0	0	0	0	0	0	0	0	0	0
0	0	0	0	0	0	0	0	0	0	0	0	0	0	0	0	0	0
0	0	0	0	0	0	0	0	0	0	0	0	0	0	0	0	0	0
0	0	0	0	0	0	0	0	0	0	0	0	0	0	0	0	62	0
0	0	73	0	0	0	0	0	0	0	0	0	0	0	0	0	0	0
0	85	0	0	0	0	0	0	0	0	0	0	0	0	0	0	0	0
0	0	0	0	0	0	0	0	0	0	0	0	0	0	0	0	0	0
0	0	0	0	0	0	0	0	0	0	0	0	0	0	0	0	0	0
0	0	0	0	0	0	0	0	0	0	0	0	0	0	0	0	0	0
0	0	0	0	0	0	0	0	0	0	0	0	0	0	0	0	0	41
0	0	0	0	0	0	0	0	0	0	0	0	0	0	0	0	0	0
0	0	0	0	0	0	0	0	0	0	0	0	0	0	0	0	0	0
0	0	0	0	0	0	0	0	0	0	0	0	0	0	0	0	0	0
0	0	0	0	0	0	0	0	0	0	0	0	0	0	0	0	0	0
0	0	0	0	0	0	0	0	0	0	0	0	0	0	0	0	0	0
0	0	0	0	0	0	0	0	0	0	0	0	0	0	0	0	0	0
0	1031	0	0	0	0	0	0	0	0	0	0	0	0	80	0	0	0
0	0	0	0	0	0	0	0	0	0	0	0	0	0	0	0	0	0
0	0	0	0	0	0	0	0	0	0	0	0	0	0	0	0	0	0
0	0	67	0	0	0	0	0	0	0	0	0	0	0	0	0	0	0
0	0	0	0	13868	0	0	0	49	0	0	0	0	0	0	0	0	0
328	555	317	0	354	218	277	1340	1035	0	26792	128	519	1485	4244	644	349	398

0	0	0	0	0	0	0	0	0	0	0	0	0	0	0	0	0	0
0	0	0	0	0	0	0	0	0	0	0	0	0	0	0	0	0	0
0	0	0	0	0	0	0	0	0	0	0	0	0	0	81	91	0	0
0	0	0	0	0	0	0	0	0	0	0	0	0	0	0	0	0	0
0	0	0	0	0	0	0	0	0	0	0	0	0	133	0	0	0	0
0	0	0	0	0	0	0	0	0	0	0	0	0	0	0	0	0	0
0	0	0	0	0	0	0	0	0	0	0	0	0	0	0	0	0	0
0	0	0	0	0	0	0	0	0	0	0	0	0	0	0	0	0	0
0	0	0	0	0	0	0	0	0	0	0	0	0	0	0	0	0	0
0	0	0	0	0	0	0	0	0	0	0	0	0	0	0	0	0	0
0	0	0	0	0	0	0	0	0	0	0	0	0	0	0	0	0	0
0	0	0	0	0	0	0	0	0	0	0	0	0	0	0	0	0	0
0	0	0	0	0	0	0	0	0	0	0	0	0	0	0	0	0	0
0	0	0	0	0	0	0	0	0	0	0	0	0	0	0	0	73	0
0	0	0	0	0	0	0	0	0	0	0	0	0	0	0	0	0	39
0	0	0	0	0	0	0	0	0	0	0	0	0	0	0	0	0	0
31617	39089	75	46	90	4074	341	125	2656	0	0	0	0	0	168	86	0	2826
0	0	0	0	0	0	0	0	0	0	0	0	0	0	0	0	0	0
0	0	0	162	0	0	0	0	2998	218	114	263	270	2634	1303	552	82	220
0	0	0	0	0	0	0	0	664	0	0	0	0	0	0	0	0	0
0	0	0	0	0	0	0	0	0	0	0	0	0	0	1442	3435	0	777884
0	0	0	0	0	0	0	0	0	0	0	0	0	0	0	0	0	0
0	1039	781	0	0	0	0	0	0	0	0	0	0	0	0	0	1172	0
0	0	0	0	0	0	0	0	0	0	0	0	0	0	0	0	0	0
0	0	0	0	0	0	0	0	0	0	0	0	0	0	0	0	0	0
0	0	0	0	0	0	0	0	0	0	0	0	0	0	0	0	0	0
0	0	0	0	0	0	0	0	0	0	0	0	0	0	0	0	0	0
0	0	0	0	0	0	0	0	0	0	0	0	0	0	0	0	0	0
0	0	0	0	0	0	0	0	0	0	0	0	0	0	0	0	0	0
0	0	0	0	0	0	0	0	0	0	0	0	0	0	0	0	0	0
0	0	0	0	0	0	0	0	0	0	0	0	0	0	0	0	0	0
40779	18914	671	0	484	281	8502	180	7002	0	0	0	399	4310	283	3067	440	187
0	0	0	0	0	0	0	0	0	0	0	0	0	0	0	0	0	0
0	0	0	0	0	0	0	0	0	0	0	0	0	0	0	0	0	0
0	0	0	0	0	0	48	39	0	0	0	0	0	0	0	0	0	0
0	0	0	0	0	0	0	0	0	0	0	0	0	0	0	0	58	0
0	0	0	0	0	0	0	0	0	0	0	0	0	0	0	0	0	0
40	0	0	0	0	47	0	0	193	0	0	0	0	0	0	0	0	0
0	0	0	0	0	0	0	0	0	0	0	0	0	0	0	0	122	0
0	0	0	0	0	0	0	0	0	0	0	0	0	0	0	0	0	0
0	0	0	0	0	0	0	0	0	0	0	0	0	0	0	0	0	0
31	0	0	0	0	0	60	45	0	0	0	0	0	0	0	0	0	0
9927	0	0	0	0	0	0	0	0	0	0	0	0	0	0	0	0	0
0	0	0	0	0	0	0	0	0	0	0	0	0	0	0	0	0	0
0	0	0	0	0	0	0	0	0	0	0	0	0	0	0	0	0	0
0	0	0	0	0	0	0	0	0	0	0	0	0	0	0	0	0	0
0	0	0	0	46	0	0	0	0	0	0	0	0	0	82	189	0	45
0	0	0	0	0	0	0	0	0	0	0	0	0	0	0	0	0	0
0	182	0	0	0	0	0	0	0	0	0	0	0	0	0	0	62	0
0	0	0	0	0	0	0	0	0	0	0	0	0	0	0	0	0	0
0	0	0	0	0	0	0	0	0	0	0	0	0	0	0	0	0	0
0	832	0	0	0	0	0	0	0	0	0	0	0	0	0	0	0	0
0	0	0	0	0	0	0	0	0	0	0	0	0	0	0	0	0	0
12299	337	324	0	274	36	178	0	126	0	0	0	0	0	170	86	126839	87
0	0	0	0	0	0	0	0	0	0	0	0	0	0	0	0	0	0
0	0	0	0	0	0	0	0	0	0	0	0	0	0	0	97	0	40
0	0	0	0	0	0	0	0	0	0	0	0	0	0	0	0	0	0
0	0	0	0	0	0	0	0	0	0	0	0	0	0	0	0	0	0
0	0	0	0	0	0	0	0	0	0	0	0	0	0	0	0	0	0
0	0	0	0	0	0	0	0	0	0	0	0	0	0	0	0	0	0
0	0	0	0	0	0	0	0	0	0	0	0	0	0	0	0	142	0
0	0	0	0	0	0	0	0	0	0	0	0	0	0	0	0	0	0
0	0	0	0	0	0	0	0	0	0	0	0	0	0	0	0	0	49
32	153	148	0	0	0	0	50	185	0	0	0	0	0	0	0	74	0
0	81	0	0	0	0	0	0	0	0	0	0	0	0	0	0	64	37
0	0	0	0	0	0	0	0	0	0	1291	0	0	0	240	283	0	0
0	0	0	0	0	0	0	0	0	0	0	0	0	0	0	0	0	0
0	0	0	0	0	0	0	0	0	0	0	0	0	0	0	0	0	0
0	88	154391	0	0	0	0	0	0	0	0	0	0	0	0	0	623	0
0	0	0	0	0	0	0	0	0	0	0	0	0	0	0	0	0	0
0	0	0	0	0	0	0	0	0	0	0	0	0	0	0	0	0	0
0	0	0	0	0	0	0	0	0	0	0	0	0	0	0	0	0	0
0	0	0	0	0	0	0	0	0	0	0	0	0	0	0	0	0	0
64	0	0	0	0	0	0	0	0	0	0	0	0	0	0	0	0	0
0	0	0	0	0	0	0	0	0	0	0	0	0	0	0	0	0	0
0	0	0	0	0	0	0	0	0	0	0	0	0	0	0	0	0	0
0	0	0	0	0	0	0	0	0	0	0	0	0	0	0	0	0	0
0	0	0	0	0	0	0	0	0	0	0	0	0	0	0	0	0	0
0	0	0	0	0	0	0	0	0	0	0	0	0	0	0	0	0	0
0	0	0	0	0	0	0	0	0	0	0	0	0	0	0	0	0	0
2428	3775	0	0	0	46	114	0	0	0	0	0	126	850	70	79	0	0
38	0	0	0	0	0	15156	0	0	0	0	0	0	0	0	0	0	0
0	0	0	0	55	51	0	0	51	0	0	0	0	0	0	0	0	0
64	77	0	0	93	45419	53	87	117	0	0	0	0	0	0	0	67	0
0	6354	235	0	0	0	0	0	0	0	0	0	0	0	0	0	521	0
0	0	0	0	0	0	0	0	0	0	0	0	0	0	0	0	0	0
0	0	0	0	0	0	0	0	0	0	0	0	0	0	0	0	0	0

0	0	0	0	0	0	0	0	0	0	0	0	0	0	0	88	0	0
0	0	0	0	0	0	0	0	0	0	0	0	0	0	0	0	0	0
0	0	0	0	0	0	0	0	0	0	0	0	0	0	0	0	0	0
0	0	0	0	0	0	0	0	0	0	0	0	0	0	0	0	0	0
0	0	0	0	0	0	0	0	0	0	0	0	0	0	0	0	0	0
0	0	0	0	0	0	0	0	0	0	0	0	0	0	0	0	0	0
0	0	0	0	0	0	0	0	0	0	0	0	0	0	0	0	0	0
0	0	0	0	0	0	0	0	0	0	0	0	0	0	0	0	0	0
0	0	0	0	0	0	0	0	0	0	0	0	0	0	0	0	0	0
0	0	0	0	0	0	0	0	0	0	0	0	0	0	84	0	0	0
0	395	0	0	3475	0	0	0	6401	0	0	0	0	0	0	0	0	0
0	0	0	0	0	0	0	0	0	0	0	0	0	0	0	0	0	0
0	0	0	0	0	0	0	0	0	0	0	0	0	0	0	0	0	0
0	0	0	0	0	0	0	0	0	0	0	0	0	0	0	0	0	0
0	0	0	0	0	0	0	0	0	0	0	0	0	0	0	0	0	0
0	0	0	0	0	0	0	0	0	0	0	0	0	0	0	0	0	0
0	0	0	0	0	0	0	0	0	0	0	0	0	0	0	0	0	0
0	0	0	0	0	0	0	0	0	0	0	0	0	0	0	0	0	0
0	0	0	0	0	0	0	0	0	0	0	0	0	0	0	0	0	0
17130	1843	0	0	144	139	47	134342	23245	0	0	0	33258	0	0	0	0	0
85238	66465	8318	510454	736609	295141	212451	616103	168228	794144	708692	283171	496028	414181	132859	385749	829743	94957
0	0	0	0	0	0	0	0	0	0	0	0	0	0	0	0	0	0
0	0	0	0	0	0	0	0	0	0	0	0	0	0	0	0	0	0
0	0	0	0	0	0	0	0	0	0	0	0	0	0	0	0	0	0
0	0	0	0	0	0	0	0	0	0	0	0	0	0	0	0	0	0
0	0	0	0	0	0	0	0	0	0	0	0	0	0	0	0	0	0
0	0	0	0	0	0	0	0	0	0	0	0	0	0	0	0	0	0
240	7139	0	0	0	0	0	34	602	0	0	0	0	0	16853	0	0	0
0	0	0	0	0	0	0	0	0	0	0	0	0	0	0	0	0	0
Control08	Control09	Control09	Control07	Control08	Control08	Control07	Control07	Control07	Control07	Control07	Control07	Control07	Control07	Control07	Control06	Control09	Control06
0	0	0	0	0	0	0	0	0	0	0	0	0	0	0	0	0	0
0	0	0	0	0	0	0	0	0	0	0	0	0	0	0	0	0	0
0	0	0	0	0	0	0	0	0	0	0	0	0	0	0	0	0	0
0	0	0	0	0	0	0	0	0	28	0	0	42372	146	0	0	0	0
0	0	0	0	0	0	0	0	0	0	0	0	0	0	0	0	0	0
0	0	0	0	0	0	0	0	0	0	0	0	0	0	0	0	0	0
34	0	0	0	0	45	0	0	17457	0	0	0	0	0	0	0	253	0
581296	425996	160985	154353	11977	28266	516301	4134	99822	800	16660	412	62442	298532	9005	117343	7194	15442
0	0	0	0	0	0	0	0	0	0	0	0	0	0	0	0	0	0
0	0	0	0	176	0	0	0	0	0	0	0	0	0	242	0	0	50
0	0	0	0	0	0	0	0	0	0	0	0	0	0	0	0	0	0
0	0	0	0	0	0	0	0	0	0	0	0	0	0	0	0	0	0
0	0	0	0	0	0	0	0	0	0	0	0	0	0	0	0	0	0
0	0	0	0	0	0	0	0	0	0	0	0	0	0	0	0	178	0
0	92	0	0	0	0	0	0	0	0	0	0	0	0	0	0	0	0
0	0	0	0	0	0	0	0	0	0	0	0	0	0	0	0	0	0
504	0	0	0	0	0	0	0	0	0	0	0	0	0	0	0	0	0
23456	164	450	0	82	91	48	0	0	0	0	0	0	0	150	349	440	106
0	0	0	0	0	0	0	0	0	0	0	0	0	0	0	0	0	0
0	0	0	0	0	0	0	0	0	0	0	0	0	0	0	0	0	0
0	0	0	0	0	0	0	0	0	0	0	0	0	0	0	0	0	0
0	0	0	0	0	0	0	0	0	0	0	0	0	0	0	0	0	0
0	0	0	0	0	0	0	0	0	0	0	0	0	0	0	0	0	0
0	0	0	0	0	0	0	0	0	0	0	0	0	0	0	0	0	0
16312	7113	135	0	205	0	1617	0	7126	0	0	0	989	6534	3980	630	0	118
0	0	0	0	0	0	0	0	0	0	0	0	0	0	0	0	0	0
0	0	0	0	0	0	0	0	0	0	0	0	0	0	0	0	54	0
0	0	0	0	0	0	0	0	0	0	0	0	0	0	0	0	0	0
0	0	0	0	0	0	0	0	0	0	0	0	0	0	0	0	0	0
0	0	0	0	0	0	0	0	0	0	0	0	0	0	0	0	0	0
0	0	234	0	0	0	0	52	0	0	0	0	0	0	65	100	250	11753
0	0	0	0	0	0	0	0	0	0	0	0	0	0	0	0	0	0
0	0	0	0	0	0	0	0	0	0	0	0	0	0	0	0	0	0
0	0	0	0	0	0	0	0	0	0	0	0	0	0	0	0	0	0
0	0	0	0	0	0	0	0	0	0	0	0	0	0	0	0	0	0
631	256	888	632	654	1003	595	914	1630	93	294	130	403	460	391	777	1015	41
0	0	0	0	48	0	0	0	0	0	0	0	0	0	0	0	0	0
0	0	0	0	0	0	0	48	0	0	0	0	0	299	0	0	0	0
0	0	0	0	0	0	0	0	0	0	0	0	0	0	0	0	0	0
0	0	0	0	0	0	0	0	0	0	0	0	0	0	0	0	72	0
0	0	0	0	0	0	0	0	0	0	0	0	0	0	0	0	0	0
28	155	0	0	83	126	61	0	254	0	0	0	0	0	0	0	201	41
0	65	0	0	0	0	0	0	0	0	0	0	0	0	0	0	0	0
0	0	0	0	0	0	0	0	0	0	0	0	0	0	0	0	0	0
0	0	0	0	0	0	0	0	0	0	0	0	0	0	0	0	0	0
0	0	0	0	0	0	0	0	0	0	0	0	0	0	0	0	0	0
0	0	0	0	0	0	0	0	0	0	0	0	0	0	0	0	0	0
0	0	0	0	38	9637	0	0	0	0	0	0	0	0	0	0	0	0
0	0	0	0	0	0	99	0	0	0	0	0	0	0	0	0	0	0
0	0	0	0	0	0	0	0	0	0	0	0	0	0	0	0	0	0
0	0	0	0	0	0	0	0	0	0	0	0	0	0	0	0	0	0
260	0	0	0	0	0	0	0	0	0	0	0	0	0	0	0	0	0
0	0	0	0	0	0	0	0	0	0	0	0	0	0	0	0	0	0
0	0	0	0	0	0	0	0	0	0	0	0	0	0	0	0	0	0
0	0	0	0	0	0	0	0	0	0	0	0	0	0	0	0	0	0

0	0	0	0	0	0	0	0	0	0	0	0	0	0	0	0	0	0
69	1701	0	0	0	49	0	48	5762	0	0	0	0	0	0	0	0	0
0	0	0	0	0	0	0	0	0	0	0	0	0	0	0	0	0	0
0	0	0	0	0	0	0	0	1078	0	0	0	0	0	0	0	0	0
18957	304327	1480	51	574	4031	8763	44521	284166	0	2077	579	0	21418	683493	3467	1499	3725
0	0	0	0	0	351	0	0	55	29	443	0	0	7237	0	0	0	0
0	0	68	0	0	2138	0	0	0	0	0	0	0	0	0	0	96	265
0	0	0	0	0	0	0	0	0	0	0	0	0	0	0	0	0	0
5741	0	0	58	104	7041	0	0	3852	0	9853	0	0	5768	79	0	0	0
0	0	66	0	0	41	0	0	1528	0	0	70	0	4259	0	0	0	0
0	0	0	0	0	0	0	0	0	0	0	0	0	0	0	0	0	0
0	0	0	0	0	0	0	0	0	0	0	0	0	0	88	56788	0	0
0	0	0	0	0	0	0	0	0	0	0	0	0	0	0	81	0	0
0	0	0	0	0	0	0	0	0	0	0	0	0	0	0	0	0	0
0	0	0	0	0	0	0	0	0	0	0	0	0	0	0	0	0	0
0	169	0	0	0	0	0	0	0	0	0	0	0	0	0	0	132	0
0	0	0	0	0	0	0	0	0	0	0	0	0	0	0	0	0	0
0	0	0	0	0	0	0	0	0	0	0	0	0	0	0	0	0	0
0	0	0	0	0	0	0	0	0	0	0	0	0	0	0	0	0	0
0	0	0	0	0	0	307	0	0	0	0	0	0	0	0	0	0	0
0	0	0	0	0	0	0	0	0	0	0	0	0	0	0	90	0	0
0	0	0	0	0	0	0	0	0	0	0	0	0	0	0	0	0	0
0	0	0	0	0	0	0	0	0	0	0	0	0	0	0	0	0	0
0	0	0	0	0	0	0	0	0	0	0	0	0	0	0	0	0	0
0	0	0	0	0	0	0	0	0	0	0	0	0	0	0	0	0	0
0	0	0	0	0	0	0	0	0	0	0	0	0	0	0	0	0	0
317	83	0	0	0	0	0	0	0	0	0	0	0	158	0	98	0	0
0	0	75	0	0	0	0	0	0	0	0	0	0	0	0	0	0	0
0	0	0	0	0	0	0	0	0	0	0	0	0	0	0	0	0	0
0	0	0	0	0	0	0	0	0	0	0	0	0	0	0	0	0	0
0	0	0	0	0	0	0	0	0	0	0	0	0	0	0	0	0	0
0	0	0	0	0	0	0	0	0	0	0	0	0	0	0	0	0	0
58	0	0	0	143	38	55	52	70	0	0	0	0	0	0	0	0	0
0	0	0	0	0	0	0	0	0	0	0	0	0	0	0	0	0	0
0	0	0	0	0	0	0	0	0	0	0	0	0	0	0	0	0	0
264	345	539	0	509	4157	386	69072	51713	0	1630	1090	0	10066	1447	895	763	1456
0	0	0	0	0	0	0	0	0	0	0	0	0	0	0	0	0	0
0	0	0	0	0	0	0	0	0	0	0	0	0	0	0	0	0	0
0	0	0	0	0	26857	0	0	0	0	0	0	0	0	0	0	0	0
0	0	0	0	0	0	0	0	0	0	0	0	0	0	0	0	73	0
0	0	0	0	0	0	0	0	0	0	0	0	0	0	0	102	0	47
0	0	0	0	0	0	0	0	0	0	0	0	0	0	0	0	0	0
0	0	0	0	0	0	0	0	0	0	0	0	0	0	0	0	0	0
0	0	0	0	0	0	0	0	0	0	0	0	0	0	0	0	0	0
0	0	0	0	0	0	0	0	0	0	0	0	0	0	0	0	0	0
0	0	0	0	0	0	0	0	0	0	0	0	0	0	0	0	0	0
0	0	0	0	0	0	0	0	0	0	0	0	0	0	0	0	0	0
0	0	0	0	0	0	0	0	0	0	0	0	0	0	0	0	0	0
0	0	0	0	0	0	0	0	0	0	0	0	0	0	0	0	0	0
0	0	0	0	0	0	0	0	0	0	0	0	0	0	0	0	0	0
1717	0	0	0	0	0	0	0	0	0	0	0	0	0	0	0	0	0
0	0	0	0	0	0	0	0	0	0	0	0	0	0	59	0	0	41
0	0	0	0	0	0	0	0	0	0	0	0	0	0	0	0	81	0
0	0	0	0	0	0	0	0	0	40	55	0	0	0	0	1099	0	0
0	0	0	0	0	0	0	0	0	0	0	0	0	0	68	0	0	0
0	0	0	0	0	0	0	0	0	0	0	0	0	0	0	0	0	0
12717	0	0	0	0	0	0	36	0	0	0	0	0	0	0	0	0	0
0	0	0	0	0	0	102	0	0	0	0	0	0	0	77	421	61	76
0	0	0	0	0	0	0	0	0	0	0	0	0	0	0	0	0	0
0	0	0	0	0	0	0	49	0	0	0	0	0	0	0	0	0	0
0	0	0	0	0	0	0	0	0	0	0	0	0	0	0	0	0	0
0	231	141	77728	0	0	0	0	0	122	149	125	146	139	4661	97	68	0
0	0	0	0	0	0	0	0	0	0	0	0	0	0	0	0	0	45
0	0	0	0	0	0	0	0	0	0	0	0	0	0	0	0	0	0
0	0	0	0	134	53	0	0	0	0	0	0	0	0	343	276	265	149
0	885	370	0	0	0	0	0	0	0	0	0	0	0	240	79	185	0
0	0	0	0	0	0	0	0	0	0	0	0	0	0	0	0	0	0
0	69	0	0	0	0	0	0	0	0	0	0	0	0	0	89	60	0
0	84	0	0	0	0	0	0	0	0	0	0	0	0	92	90	0	38
0	0	0	0	0	0	0	0	0	0	0	0	0	0	69	0	0	0
0	0	0	0	0	0	0	0	0	0	0	0	0	0	0	0	0	0
0	0	0	0	0	0	0	0	0	0	0	0	0	0	0	0	0	0
0	0	0	0	0	0	0	0	0	0	0	0	0	0	0	0	0	0
0	0	0	0	0	0	0	0	0	0	0	0	0	0	0	0	0	0
10667	0	0	0	0	0	0	0	123	0	0	0	0	0	0	0	0	0
0	0	0	0	0	40	26499	0	52	0	0	0	0	0	0	0	0	0
0	0	0	0	0	0	0	0	0	0	0	0	0	0	0	0	0	0
0	0	0	0	0	0	0	0	0	0	0	0	0	0	93	88	0	0
0	0	0	0	0	0	0	0	0	0	0	0	0	0	0	0	0	0
0	0	0	0	0	0	0	0	0	0	0	0	0	0	0	0	0	0
0	0	0	0	0	0	0	0	0	0	0	0	0	0	0	0	0	0
0	0	0	0	0	0	0	0	0	0	0	0	0	0	0	0	0	0
0	0	0	0	0	0	0	0	0	0	0	0	0	0	0	95	0	0
0	0	0	0	0	0	0	0	0	0	0	0	0	0	0	0	0	0
0	0	0	0	0	0	0	0	0	0	0	0	0	0	0	0	0	0
37	0	153	249	0	0	0	0	4147	325	449	138	512	585	75	0	69	1085
0	0	0	0	0	0	319	0	0	0	0	0	0	0	0	0	0	0

0	0	0	0	0	0	0	0	0	0	0	0	0	0	0	0	0	0
0	0	0	0	0	0	0	0	0	0	0	0	0	0	0	0	0	0
0	0	0	0	0	0	107	0	0	0	0	0	0	0	70	0	0	0
0	0	0	0	0	0	0	0	0	0	0	0	0	0	0	0	0	0
6188	3502	143464	0	95	37220	12329	103	14692	0	0	0	0	2561	250	81	852	0
0	0	145	0	0	0	62	0	0	0	0	0	0	146	0	0	0	0
2060	1036	1321	49	9258	92	94	41	8491	0	0	0	0	0	236	238	878	19263
750	477	28081	84	51	30426	498	0	2070	0	54	0	0	32804	671	603	1297	339
5116	0	8233	0	27273	1814	159	0	337	0	0	0	0	140	0	0	68	0
0	0	0	55	49	46	0	0	59	32	0	0	43677	35293	0	0	0	0
0	0	0	0	0	0	0	0	0	0	0	0	0	0	0	0	0	0
0	0	0	0	0	0	0	0	0	0	0	0	0	0	0	0	0	0
0	0	0	0	0	0	0	0	0	0	0	0	0	0	0	0	0	0
0	0	0	0	0	0	0	0	0	0	0	0	0	0	0	0	0	0
0	0	0	0	0	0	0	0	0	0	0	0	0	0	0	0	0	0
0	0	0	0	0	0	0	0	0	0	0	0	0	0	0	0	0	0
0	0	0	0	0	0	0	0	0	0	0	0	0	0	0	0	0	0
0	0	0	0	0	0	0	0	0	0	0	0	0	0	0	0	0	0
0	0	0	0	0	0	0	0	0	0	0	0	0	0	0	0	0	0
0	0	0	0	0	0	0	0	0	0	0	0	0	0	0	0	0	0
0	0	0	0	0	0	0	0	0	0	0	0	0	0	0	0	0	0
74	138	149	0	93	0	95	0	64	167	59748	72	272	0	0	0	0	0
0	0	0	0	0	0	0	0	0	0	0	0	0	0	0	0	0	0
0	0	0	0	0	0	0	0	0	0	0	0	0	0	0	0	0	0
0	0	0	0	0	0	0	0	0	0	0	0	0	0	0	0	0	0
0	0	0	0	0	0	0	0	0	0	0	0	0	0	0	0	0	0
0	0	0	0	33987	91	0	48	60	0	0	0	0	0	0	0	0	0
0	0	0	0	0	0	0	0	0	0	0	0	0	0	0	0	0	0
0	78	0	0	0	0	0	0	0	0	0	0	0	0	0	0	0	0
0	0	0	0	0	0	0	0	0	0	0	0	0	0	0	0	0	0
0	1377	0	0	0	0	0	0	0	0	0	0	0	0	0	0	0	0
0	0	0	0	0	0	0	0	0	0	0	0	0	0	0	0	0	0
21527	5036	228216	345	25804	111391	381	40186	35799	134789	39475	63890	174168	54651	14715	1369	2625	24988
0	0	0	0	0	0	0	0	0	0	0	0	0	0	0	0	0	0
0	0	0	0	0	0	0	0	0	0	0	0	0	0	0	0	0	0
0	0	0	0	0	0	0	0	0	0	0	0	0	0	0	0	0	0
0	0	0	0	0	0	0	0	0	0	0	0	0	0	0	0	0	0
0	0	0	0	0	0	0	0	0	0	0	0	0	0	0	0	0	0
0	0	0	0	0	0	0	0	0	0	0	0	0	0	0	0	0	0
0	0	0	0	0	0	0	0	0	0	0	0	0	0	229	0	0	0
41	0	0	0	0	0	0	59	0	0	0	0	0	0	70	0	0	0
0	0	0	0	0	0	0	0	0	0	0	0	0	0	0	0	0	0
0	0	0	0	0	0	0	0	0	0	0	0	0	0	0	0	0	0
0	0	0	0	0	0	0	0	0	0	0	0	0	0	0	0	0	0
0	0	0	0	0	0	0	0	0	0	0	0	0	0	0	0	0	0
0	0	0	0	0	0	0	0	0	0	0	0	0	0	0	0	0	0
0	0	0	0	0	49	0	0	0	0	0	0	0	0	0	0	0	0
25	72	150	0	0	14525	0	0	69	0	0	0	0	0	0	0	70	0
0	0	0	0	0	0	0	0	0	0	0	0	0	0	0	0	0	0
0	0	0	0	0	0	0	0	0	0	0	0	0	0	0	0	0	0
0	0	83	0	0	0	0	0	0	0	0	0	0	0	0	0	0	0
0	0	0	0	0	0	0	0	0	0	0	0	0	0	0	0	0	0
0	0	0	0	0	0	0	0	0	0	0	0	0	0	0	0	0	0
0	70	84	0	0	0	0	0	0	0	0	0	0	0	0	188	77	78
0	0	0	0	0	0	0	0	0	0	0	0	0	0	0	0	0	0
0	0	0	0	0	0	0	0	0	0	0	0	0	0	0	0	0	0
0	0	0	0	0	0	0	0	0	0	0	0	0	0	0	0	0	0
0	0	0	0	0	0	0	0	0	0	0	0	0	0	0	82	0	49
0	0	0	0	0	0	0	0	0	0	0	0	0	0	0	0	0	0
0	0	82	0	0	0	0	0	0	0	0	0	0	0	0	0	56	0
0	0	0	0	0	0	0	0	0	0	0	0	0	0	0	0	0	0
0	0	0	0	0	0	0	0	0	0	0	0	0	0	0	0	0	0
0	234	226	53934	0	0	0	0	0	133	95	0	0	140	0	0	317	0
0	0	0	0	0	0	0	0	0	0	0	0	0	0	0	0	0	0
0	0	0	0	0	0	0	0	0	0	0	0	0	0	0	0	0	0
0	0	0	0	0	0	0	0	0	0	0	0	0	0	0	0	0	0
0	0	0	0	47	38	0	0	0	0	0	0	0	0	0	0	0	0
0	0	0	0	0	0	0	0	0	0	0	0	0	0	0	0	0	0
0	0	0	0	0	0	0	0	0	0	0	0	0	0	0	0	0	0
0	0	0	0	0	0	0	0	0	0	0	0	0	0	0	0	0	0
0	241	113112	0	0	0	0	0	0	0	0	0	0	0	0	0	70	0
0	0	255	0	36	111	0	0	0	0	0	0	0	0	1082	882	0	552
0	0	0	0	0	0	0	0	0	0	0	0	0	0	0	0	0	0
0	0	0	0	0	0	0	0	0	0	0	0	0	0	0	0	0	0
6203	32816	278	57	599	94064	2079	833	21959	79	49	242	0	7863	290	3771	127	0
0	0	0	0	0	48	0	0	66	0	0	0	0	0	0	0	0	0
0	0	0	0	0	0	0	0	0	0	0	0	0	0	0	0	0	0
0	0	0	0	0	0	0	0	0	0	0	0	0	0	0	0	0	0
0	0	0	0	0	0	0	0	0	0	0	0	0	0	0	0	0	0
0	0	0	0	0	0	0	0	0	0	0	0	0	0	0	0	0	0
0	0	0	0	0	0	0	0	0	0	0	0	0	0	0	0	0	0
0	0	0	0	0	0	0	0	0	0	0	0	0	0	0	0	0	0
0	0	0	0	0	0	0	0	0	0	0	0	0	0	0	0	0	0
0	0	0	0	0	0	0	0	0	0	0	0	0	0	0	0	0	0
0	0	0	0	0	0	0	0	0	0	0	0	0	0	0	0	0	0
0	0	0	0	0	0	0	0	0	0	0	0	0	0	0	0	0	0
0	86	0	0	0	0	0	0	4109	0	0	0	0	0	0	0	199	0

0	0	0	0	0	0	0	0	0	0	0	0	0	0	0	0	0	0
5565	2669	0	0	0	53	0	40	69	0	0	0	0	0	0	91	0	0
0	0	0	0	0	0	0	0	0	0	0	0	0	0	0	0	0	0
0	0	0	0	0	0	0	0	0	0	0	0	0	0	0	0	0	0
0	0	0	0	0	0	0	0	0	0	0	0	0	0	0	0	0	0
0	0	0	0	0	0	0	0	0	0	0	0	0	0	0	0	0	0
0	0	0	0	0	0	0	0	597	0	0	0	0	0	0	0	0	0
0	0	0	0	0	0	0	0	0	0	0	0	0	0	0	0	0	0
0	0	0	0	0	0	0	0	0	0	0	0	0	0	0	0	0	0
0	0	0	0	0	0	0	0	0	0	0	0	0	0	0	0	0	0
0	0	0	0	0	0	0	0	0	0	0	0	0	0	0	0	0	0
0	0	0	0	0	0	0	0	0	0	0	0	0	0	0	0	0	0
0	0	0	0	0	0	0	0	0	0	0	0	0	0	0	0	0	0
0	0	0	0	0	0	0	0	0	0	0	0	0	0	0	0	0	0
0	0	0	0	0	0	0	0	0	0	0	0	0	0	0	0	0	0
0	0	0	0	0	0	0	0	0	0	0	0	0	0	0	0	0	0
0	0	0	0	0	0	0	0	0	0	0	0	0	0	0	0	0	0
0	0	0	0	0	0	0	0	0	0	0	0	0	0	0	0	0	0
0	0	0	0	0	0	0	0	0	0	0	0	0	0	0	0	0	0
0	0	0	0	0	0	0	0	0	0	0	0	0	0	0	0	0	0
0	0	0	0	0	0	0	0	0	0	0	0	0	0	0	0	0	0
0	0	0	0	0	0	0	0	0	0	0	0	0	0	0	0	0	0
3359	1548	68	0	318	0	774	0	52	0	0	0	233	1740	167	63401	67	84
0	0	0	0	0	0	0	0	0	0	0	0	0	0	0	0	0	0
0	86	0	0	0	0	0	0	0	0	0	0	0	0	0	0	119	0
0	83	135	0	0	0	0	0	0	0	0	0	0	0	0	0	137	0
0	0	0	0	0	0	0	0	0	0	0	0	0	0	0	0	0	0
0	0	0	0	0	0	0	0	0	0	0	0	0	0	0	0	0	0
0	0	0	0	0	0	0	0	0	0	0	0	0	0	0	0	51	0
0	0	0	0	0	0	2687	0	0	0	0	0	0	0	0	0	0	0
0	0	0	0	0	0	0	0	0	0	0	0	0	0	0	178	0	33
0	0	0	0	0	0	0	0	0	0	0	0	0	0	0	0	0	0
0	0	0	0	0	0	0	0	0	0	0	0	0	0	0	0	0	0
24627	17075	18663	52291	54709	19815	47669	54662	60365	64762	58829	93413	37982	29961	47675	108168	13050	10349
0	0	0	0	0	0	0	0	0	34	35174	0	0	145	0	0	0	0
0	318	0	0	0	1283	0	0	0	0	0	0	0	0	0	0	0	0
72	936	83	0	0	18376	0	0	142	0	0	0	0	0	0	0	0	0
31	0	0	0	45	48	0	23392	49	0	0	0	0	0	0	0	0	0
0	0	0	0	0	0	0	0	0	0	0	0	0	0	0	0	0	0
0	0	0	0	0	0	0	0	0	0	0	0	0	0	0	0	0	0
0	0	0	0	0	0	0	0	0	0	0	0	0	0	0	0	0	0
0	0	0	0	0	0	0	89	0	0	0	0	0	0	0	0	0	0
0	0	0	0	0	8922	0	0	0	0	0	0	0	0	0	0	0	0
0	0	0	0	0	0	0	0	0	0	0	0	0	0	0	0	0	0
0	0	0	0	524	0	257	0	0	0	0	0	0	0	1751	2022	215	879
0	0	0	0	0	0	0	0	0	0	0	0	0	0	0	0	0	0
0	0	0	0	0	1076	60	0	113	0	0	0	0	0	0	283	0	0
0	0	0	0	0	0	0	0	0	0	0	0	0	0	0	0	0	0
0	0	0	0	0	0	0	0	0	0	0	0	0	0	0	0	0	0
0	0	0	0	0	0	0	0	0	0	0	0	0	0	0	0	0	0
0	0	0	0	0	0	0	0	0	0	0	0	0	0	0	0	0	0
0	0	0	0	0	0	0	0	0	0	0	61	0	0	0	0	0	0
0	0	0	0	0	0	0	0	0	0	55	0	0	0	0	0	0	0
0	0	0	0	0	0	0	0	0	0	0	0	0	0	0	0	0	0
0	0	0	0	0	0	0	0	0	0	0	0	0	0	0	0	0	0
0	0	0	0	0	0	0	0	0	0	0	0	0	0	0	0	129	0
0	0	0	0	0	0	0	0	0	0	0	0	0	0	0	0	0	0
0	0	0	0	0	0	0	0	0	0	0	0	0	0	0	0	0	0
0	0	0	0	0	0	0	0	0	0	0	0	0	0	167	295	0	21907
0	0	0	0	0	0	0	0	0	0	0	0	0	0	0	0	0	0
0	0	0	0	0	0	0	0	0	0	0	0	0	0	0	0	0	0
0	0	0	0	0	0	0	0	0	0	0	0	0	0	0	0	0	0
0	0	0	0	0	0	0	0	0	0	0	0	0	0	0	0	0	0
0	0	0	0	0	0	0	0	0	0	0	0	0	0	0	0	0	0
0	0	0	0	0	49	0	0	0	0	0	0	0	0	0	0	0	0
0	0	0	0	0	0	0	0	0	0	0	0	0	0	0	0	0	0
0	0	0	0	0	0	0	0	0	0	0	0	0	0	0	0	0	0
0	0	0	0	0	0	0	0	0	0	0	0	0	129	0	0	0	92
0	0	0	0	0	0	0	0	0	0	0	0	0	0	0	0	0	0
0	0	0	0	0	0	0	0	0	0	0	0	0	0	0	0	0	0
0	0	0	0	0	0	0	0	0	0	0	0	0	0	0	0	0	0
0	0	0	0	0	0	0	0	0	0	0	0	0	0	0	0	0	0
243	504	0	324	288	678	0	78	0	156	114	329	0	169	176	175	0	42
0	0	0	0	0	0	0	0	0	0	0	0	0	0	0	97	0	0
31	0	0	0	0	0	41	0	5881	0	0	0	0	0	0	0	0	0
0	0	0	0	0	0	0	0	0	0	0	0	0	0	0	0	0	0
0	0	0	0	0	0	0	0	0	0	0	0	0	0	0	0	0	0
0	0	0	0	0	0	0	0	0	0	0	0	0	0	0	0	0	0
0	0	0	0	0	0	0	0	0	0	0	0	0	0	0	0	0	0
0	0	0	0	0	0	0	0	0	0	0	0	0	0	0	0	0	0
0	0	0	0	0	0	0	0	0	0	0	0	0	0	0	0	0	0
0	0	0	0	0	0	0	0	0	0	0	0	0	0	0	0	0	0
0	0	0	0	0	0	0	0	0	0	0	0	0	0	0	0	0	0
0	0	0	0	0	0	0	0	0	0	0	0	0	0	0	0	0	0
37	0	0	64	1814	38	0	44	225	214	679	0	0	0	0	0	67	0
0	0	0	0	0	0	0	0	0	0	0	0	0	0	0	0	0	0
0	0	0	0	0	0	0	0	0	0	0	0	0	0	0	0	0	0

0	0	0	0	0	56	0	56	0	0	0	0	0	0	0	0	0	0
0	0	0	0	0	0	0	0	0	0	0	0	0	0	0	0	0	0
0	0	0	0	0	0	0	0	0	0	0	0	0	0	0	0	0	0
0	0	0	0	0	0	0	0	0	0	0	0	0	0	0	0	0	0
0	0	0	0	0	0	0	0	0	0	0	0	0	0	0	0	0	0
0	0	0	0	0	0	0	0	0	0	0	0	0	0	0	0	0	0
0	0	0	0	0	0	0	0	0	0	0	0	0	0	0	0	0	0
0	0	0	0	0	0	0	0	0	0	0	0	0	0	0	0	0	0
0	0	0	0	0	0	0	0	0	0	0	0	0	0	0	0	0	0
0	0	0	0	0	0	0	0	0	0	0	0	0	0	0	0	0	0
0	343	0	0	0	0	0	0	0	0	0	0	0	0	0	0	73	0
87	0	0	0	143	46137	0	0	0	0	0	0	0	0	0	0	0	0
0	0	0	0	0	0	0	0	0	0	0	0	0	0	0	0	0	0
0	0	0	0	0	39279	0	37	63	0	0	0	0	0	0	0	0	0
0	0	0	0	0	0	0	0	0	0	0	0	0	0	0	0	0	0
0	0	0	0	0	0	0	0	0	0	0	0	0	0	0	0	0	0
0	0	0	0	0	0	0	0	0	0	0	0	0	0	0	0	0	0
0	0	0	0	0	0	0	0	0	0	0	0	0	0	0	0	0	0
0	0	0	0	0	0	0	0	0	81	35818	0	0	0	0	0	0	0
0	80	0	0	0	0	0	0	0	0	0	0	0	0	0	0	0	0
0	0	0	0	0	0	0	0	0	0	0	0	0	0	0	0	0	0
0	0	0	0	0	0	0	0	0	0	0	0	0	0	0	0	0	43
0	0	0	0	0	0	0	0	0	0	0	0	0	0	0	0	0	0
1761	0	0	0	0	0	0	0	0	0	0	0	0	0	0	0	0	0
0	0	0	0	0	0	0	0	0	0	0	0	0	0	0	0	0	0
0	0	0	0	0	0	0	0	0	0	0	0	0	0	0	0	0	0
0	0	0	0	0	0	0	0	0	0	0	0	0	0	0	0	0	0
0	0	0	0	0	0	0	0	65	0	0	0	0	0	0	0	66	0
0	0	0	0	0	0	0	0	0	0	0	0	0	0	0	0	0	0
0	0	239	0	0	0	0	0	0	0	0	0	0	0	0	0	0	0
0	0	0	0	0	0	0	0	0	0	0	0	0	0	0	0	0	0
0	0	85	0	0	0	0	0	0	0	0	0	0	0	0	0	0	0
0	0	64	0	0	0	0	0	0	0	0	0	0	0	0	0	0	0
0	0	0	0	0	0	0	0	0	0	0	0	0	4093	0	0	0	0
0	0	0	0	0	0	0	0	0	0	0	0	0	0	0	0	0	0
0	0	0	0	0	0	0	0	0	0	0	0	0	0	0	0	0	0
0	0	0	0	0	0	0	0	0	0	0	0	0	0	0	0	0	0
0	0	0	0	0	0	0	0	0	0	0	0	0	0	0	0	0	0
0	0	0	0	0	0	0	0	0	0	0	0	0	0	0	0	0	0
0	0	0	0	0	0	0	0	0	0	0	0	0	0	0	0	0	0
36	0	0	0	0	0	0	0	0	0	0	0	0	0	0	0	0	0
0	0	0	0	0	0	0	0	0	0	0	0	0	0	0	0	0	0
0	0	0	0	0	0	0	0	0	0	0	0	0	0	0	0	0	0
0	0	0	0	0	0	0	0	0	0	0	0	0	0	0	0	0	0
0	0	0	0	27701	0	0	0	0	0	0	0	0	0	0	0	0	0
2193	82	77	0	421	273	5621	173	352	0	0	0	0	0	0	0	120	0
0	0	0	0	0	0	0	0	0	0	0	0	0	0	0	0	0	0
0	0	0	0	0	0	0	0	0	0	0	0	0	0	0	0	0	49
0	0	0	0	0	0	0	0	0	0	0	0	0	0	0	0	0	0
690	2034	78	0	0	9733	2007	43	1056	0	0	0	0	3111	0	0	0	0
0	0	0	0	0	0	0	0	0	0	0	0	0	0	0	0	0	0
16473	1462	51199	31876	33200	105947	64544	430	134269	503	765	555458	28021	5007	0	77859	1134	8866
2416	655	68	0	9068	17929	55	0	57	0	0	0	0	0	0	0	143	0
0	1247	0	0	0	0	0	0	0	0	0	0	0	0	0	0	0	0
0	937	0	0	0	0	0	0	0	0	0	0	0	0	0	0	0	0
0	0	0	0	0	0	0	0	0	0	0	0	0	0	0	0	0	0
0	0	0	0	0	0	0	0	0	0	0	0	0	0	0	0	0	0
0	0	0	0	0	0	0	0	0	0	0	0	0	0	0	0	0	0
0	0	0	0	0	0	0	0	0	0	0	0	0	0	0	0	0	0
0	0	0	0	0	0	0	0	0	0	0	0	0	0	0	0	0	0
86	1136	1932	59006	89	0	9510	0	55	39	44	73	284	167	0	0	1163	42
0	0	0	0	0	0	0	0	0	0	0	0	0	0	0	0	0	0
0	0	0	0	0	0	0	0	0	0	0	0	0	0	0	0	0	0
0	0	0	0	0	0	0	0	0	0	0	0	0	0	0	0	0	0
0	0	0	0	0	0	0	0	0	0	0	0	0	0	0	0	0	0
0	0	0	0	0	0	0	0	0	0	0	0	0	0	0	0	0	0
0	0	0	0	0	135	0	0	108	0	0	0	0	0	210	356	0	184
0	0	0	0	0	0	0	0	0	0	0	0	0	0	0	0	0	0
0	0	0	0	0	0	0	0	0	0	0	0	0	0	0	0	0	0
0	0	0	0	0	0	0	0	0	0	0	0	0	0	0	0	0	0
0	0	0	0	0	0	0	0	0	0	0	0	0	0	0	0	0	0
0	463	606	0	0	0	0	0	0	0	0	65	0	0	0	0	898	0
0	954	0	0	0	0	0	0	0	0	0	0	0	0	0	0	0	0
0	0	138	0	0	0	0	0	0	0	0	0	0	0	0	0	136	0
0	0	0	0	0	0	0	0	0	0	0	0	0	0	0	0	0	0
0	0	0	0	0	0	0	0	0	0	0	0	0	0	0	0	0	0
0	0	0	0	0	0	0	0	0	0	0	0	0	0	0	0	0	0
0	0	0	0	0	0	0	0	0	0	0	0	0	0	0	0	0	0
0	0	0	0	0	0	0	0	0	0	0	0	0	0	0	0	0	0
0	0	0	0	0	0	0	0	0	0	0	0	0	0	0	0	0	0
0	0	0	0	0	0	0	0	0	0	0	0	0	0	0	0	0	0
0	0	0	0	0	0	0	0	0	0	0	0	0	0	0	0	0	0
0	0	0	0	0	0	0	0	0	0	0	0	0	0	0	0	0	0
0	0	0	0	0	0	0	0	0	0	0	0	0	0	0	0	0	0
0	0	0	0	0	0	0	0	0	0	0	0	0	0	0	0	0	0
0	996	0	0	0	0	0	0	0	0	0	0	0	0	0	0	0	0
0	95	0	0	0	0	0	0	0	0	0	0	0	0	0	0	0	0

36	0	0	0	39	0	49	0	15076	0	0	0	0	0	0	0	0	0
0	0	65	0	0	0	0	0	0	0	0	0	0	0	0	0	117	0
0	0	0	0	0	0	0	0	0	0	0	0	0	0	0	0	0	0
0	0	0	0	0	35	14029	0	0	0	0	0	0	0	0	0	0	0
33	0	0	0	2767	0	0	0	0	0	0	0	0	0	0	0	0	0
0	0	0	0	0	0	0	0	0	0	0	0	0	0	0	0	0	0
0	0	0	0	0	0	0	0	0	0	0	0	0	0	0	0	0	0
0	0	0	0	0	0	0	0	0	0	0	0	0	0	81	0	0	0
0	0	0	0	0	0	0	0	0	0	0	0	0	0	0	0	0	0
0	0	0	0	0	0	0	0	0	0	0	0	0	0	0	0	0	0
0	0	0	0	0	0	0	0	0	0	0	0	0	0	0	0	0	0
0	0	0	0	0	0	0	0	0	55	0	73	43284	0	0	0	0	0
3658	0	82	0	0	37	0	8143	608	0	0	0	0	0	70	0	0	0
0	0	0	0	0	0	0	0	0	0	0	0	0	152	0	0	0	0
0	0	0	0	0	0	0	0	0	0	0	0	0	0	0	0	0	0
0	0	0	0	0	0	0	0	0	0	0	0	0	0	0	0	0	0
0	0	0	0	0	0	0	0	0	0	0	0	0	0	0	0	0	0
0	0	0	0	0	0	0	0	0	0	0	0	0	0	0	0	0	0
0	0	0	0	0	0	0	0	0	0	0	0	0	0	0	0	0	0
0	0	0	0	0	0	0	0	0	0	0	0	0	0	0	0	0	0
0	0	0	0	0	0	0	0	0	0	0	0	0	0	0	0	0	0
6388	12111	35814	58182	972	163	6763	89	400	3040	543	218	654	7738	57413	1162	1515	170
0	0	0	0	0	0	0	0	0	0	0	0	0	0	0	0	0	0
0	0	0	0	0	0	0	0	0	0	0	0	0	0	0	0	0	0
0	0	0	0	0	0	0	0	0	0	0	0	0	0	0	0	0	0
0	0	0	0	0	0	0	0	0	0	0	0	0	0	0	0	0	0
0	0	0	0	0	0	0	0	0	0	0	0	0	0	0	0	0	0
64	0	0	0	0	0	0	28	0	0	0	0	0	0	0	0	0	0
462	0	0	0	0	0	221	0	0	0	0	0	112	735	0	95	64	0
0	0	0	0	0	0	0	0	0	0	0	0	0	0	0	0	0	0
0	0	0	0	0	0	0	0	0	0	0	0	0	0	0	0	0	0
0	0	0	0	0	0	0	0	0	0	0	0	0	0	0	0	0	0
0	0	0	0	0	0	0	0	0	0	0	0	0	0	0	0	0	0
0	0	442	0	0	187	0	0	0	0	0	0	0	0	0	0	0	0
0	0	0	0	0	0	0	0	0	0	0	0	0	0	0	0	0	0
0	0	0	0	0	0	0	0	0	0	0	0	0	0	0	0	0	0
0	0	0	0	0	0	0	0	0	0	0	0	0	0	0	0	0	0
0	0	0	0	0	0	0	0	0	0	0	0	0	0	0	0	0	0
0	0	0	0	0	0	0	0	0	0	0	0	0	0	0	0	0	0
0	0	0	0	0	0	0	0	0	0	0	0	0	0	0	0	0	0
0	0	0	0	0	0	0	0	0	0	0	0	0	0	0	0	0	0
0	0	21032	0	0	8532	0	0	0	0	0	0	0	0	0	0	0	0
2644	0	0	0	0	0	0	0	0	0	0	0	0	0	0	0	0	0
0	0	13907	0	37	1555	0	0	0	0	0	0	0	0	0	0	0	0
0	0	0	0	0	0	0	0	0	0	0	0	0	0	0	0	0	0
0	0	0	0	0	0	0	0	0	0	0	0	0	0	0	0	0	0
0	0	0	0	0	0	0	0	0	0	0	0	0	0	0	0	0	0
0	0	0	0	0	0	0	0	0	0	0	0	0	0	0	0	0	0
0	0	0	0	0	0	0	0	0	0	0	0	0	0	0	0	0	0
0	0	0	0	0	0	0	0	0	0	0	0	0	0	0	0	0	0
0	0	0	0	0	0	0	0	0	0	0	0	0	0	0	0	0	0
0	0	0	0	0	0	0	0	0	0	0	0	0	0	0	0	0	0
0	0	0	0	0	0	0	0	0	0	0	0	0	0	0	0	0	0
3164	0	0	0	0	0	0	0	0	0	0	0	0	0	0	0	0	0
0	0	0	0	0	54	11674	0	0	0	0	0	0	0	0	0	0	0
0	0	0	0	0	0	0	0	0	0	0	0	0	0	0	0	0	0
0	0	0	0	0	0	0	0	0	0	0	0	0	0	0	0	0	0
0	0	0	0	0	0	0	0	0	0	0	0	0	0	0	0	0	0
29	0	0	0	49	40	0	47	47	0	0	0	0	0	0	0	0	0
0	0	0	0	0	0	0	0	0	0	0	0	0	0	0	0	0	0
0	0	0	0	0	0	0	0	0	0	0	0	0	0	0	0	0	0
0	72	0	0	0	0	0	0	0	0	0	0	0	0	0	0	0	0
0	0	0	0	0	0	0	0	0	0	0	0	0	0	0	0	0	0
0	0	0	0	0	0	0	0	0	0	0	0	0	0	0	0	0	0
0	0	0	0	0	0	0	0	0	0	0	0	0	0	0	0	0	0
0	0	0	0	0	0	0	0	0	0	0	0	0	0	0	0	0	0
0	0	0	0	0	0	0	0	0	0	0	0	0	0	0	0	0	0
0	0	0	0	0	0	0	0	0	0	0	0	0	0	0	0	0	0
0	0	0	0	0	0	0	0	0	0	0	0	0	0	0	0	0	0
0	0	0	0	0	0	0	0	0	0	0	0	0	0	0	0	0	0
0	0	0	0	0	0	0	0	0	0	0	0	0	0	0	0	0	0
0	0	0	0	0	0	0	0	0	0	0	0	0	0	0	0	0	0
0	0	0	0	0	0	0	0	0	0	0	0	0	0	0	0	0	0
0	0	0	0	0	0	0	0	0	0	0	0	0	0	147	0	0	0
0	0	0	0	45	0	0	0	0	0	0	0	0	0	0	0	0	0
0	0	0	0	0	0	0	0	0	0	0	0	0	0	0	0	0	0
0	0	0	0	0	0	0	0	0	0	0	0	0	0	0	0	0	0
0	0	0	0	0	0	0	0	0	0	0	0	0	0	0	0	0	0
0	0	0	0	0	0	0	0	0	0	0	0	0	0	0	0	0	0
0	0	0	0	0	0	0	0	0	0	0	0	0	0	0	0	0	0
0	0	0	0	0	0	0	0	0	0	0	0	0	0	0	0	0	0
0	0	0	0	0	0	0	0	0	0	0	0	0	0	0	0	0	0
0	0	0	0	0	0	0	0	0	0	0	0	0	0	0	0	0	0
0	0	0	0	0	0	0	0	0	0	0	0	0	0	0	0	0	0
0	0	0	0	0	0	0	0	0	0	0	0	0	0	0	0	0	0
0	0	76	0	0	0	0	0	0	0	0	0	0	0	0	0	76	0
0	0	0	0	142	54	0	0	0	0	0	0	0	0	0	0	0	0

0	0	0	0	0	0	0	0	0	0	0	0	0	0	10754	0	0	0
0	0	0	0	0	0	0	0	0	0	0	0	0	0	0	0	0	0
0	0	0	0	0	0	0	0	0	0	0	0	0	0	0	0	0	0
0	0	0	0	0	0	0	0	0	0	0	0	0	0	0	0	0	0
0	0	0	0	0	0	0	0	0	0	0	0	0	0	0	0	0	0
0	0	0	0	0	0	0	0	0	0	0	0	0	0	0	0	0	37
0	0	0	0	0	0	0	0	0	0	0	0	0	0	0	0	0	0
0	0	0	0	0	0	0	0	0	0	0	0	0	0	0	0	0	0
0	0	0	0	0	0	0	0	0	80	0	0	29248	0	159	1311	0	198
38	498	0	0	0	0	25258	0	62	0	0	0	1274	150	537	158912	0	343
0	0	0	0	0	0	0	0	0	0	0	0	0	0	0	0	0	0
0	0	0	0	0	0	0	0	0	0	0	0	0	0	0	0	0	0
0	0	0	0	0	0	0	0	0	0	0	0	0	0	0	0	0	0
0	0	0	0	0	0	0	0	0	0	0	0	0	0	0	0	0	0
0	0	0	0	0	0	0	0	0	0	0	0	0	0	0	0	0	0
0	0	0	0	0	0	0	0	0	0	0	0	0	0	0	0	0	0
0	0	0	0	0	0	0	0	0	0	0	0	0	0	0	0	62	0
0	0	73	0	0	0	0	0	0	0	0	0	0	0	0	0	0	0
0	85	0	0	0	0	0	0	0	0	0	0	0	0	0	0	0	0
0	0	0	0	0	0	0	0	0	0	0	0	0	0	0	0	0	0
0	0	0	0	0	0	0	0	0	0	0	0	0	0	0	0	0	0
0	0	0	0	0	0	0	0	0	0	0	0	0	0	0	0	0	0
0	0	0	0	0	0	0	0	0	0	0	0	0	0	0	0	0	0
0	0	0	0	0	0	0	0	0	0	0	0	0	0	0	0	0	41
0	0	0	0	0	0	0	0	0	0	0	0	0	0	0	0	0	0
0	0	0	0	0	0	0	0	0	0	0	0	0	0	0	0	0	0
0	0	0	0	0	0	0	0	0	0	0	0	0	0	0	0	0	0
0	0	0	0	0	0	0	0	0	0	0	0	0	0	0	0	0	0
0	0	0	0	0	0	0	0	0	0	0	0	0	0	0	0	0	0
0	0	0	0	0	0	0	0	0	0	0	0	0	0	0	0	0	0
0	0	0	0	0	0	0	0	0	0	0	0	0	0	0	0	0	0
0	0	0	0	0	0	0	0	0	0	0	0	0	0	80	0	0	0
0	1031	0	0	0	0	0	0	0	0	0	0	0	0	0	0	0	0
0	0	0	0	0	0	0	0	0	0	0	0	0	0	0	0	0	0
0	0	0	0	0	0	0	0	0	0	0	0	0	0	0	0	0	0
0	0	67	0	0	0	0	0	0	0	0	0	0	0	0	0	0	0
0	0	0	0	13868	0	0	0	49	0	0	0	0	0	0	0	0	0
0	83	0	0	0	0	0	1119	350	0	0	0	0	145	81	0	0	0
328	472	317	0	354	218	277	221	685	0	26792	128	519	1340	4163	644	349	398
0	0	0	0	0	0	0	0	0	0	0	0	0	0	0	0	0	0
0	0	0	0	0	0	0	0	0	0	0	0	0	0	0	0	0	0
0	0	0	0	0	0	0	0	0	0	0	0	0	0	81	91	0	0
0	0	0	0	0	0	0	0	0	0	0	0	0	0	0	0	0	0
0	0	0	0	0	0	0	0	0	0	0	0	0	133	0	0	0	0
0	0	0	0	0	0	0	0	0	0	0	0	0	0	0	0	0	0
0	0	0	0	0	0	0	0	0	0	0	0	0	0	0	0	0	0
0	0	0	0	0	0	0	0	0	0	0	0	0	0	0	0	0	0
0	0	0	0	0	0	0	0	0	0	0	0	0	0	0	0	0	0
0	0	0	0	0	0	0	0	0	0	0	0	0	0	0	0	0	0
0	0	0	0	0	0	0	0	0	0	0	0	0	0	0	0	0	0
0	0	0	0	0	0	0	0	0	0	0	0	0	0	0	0	0	0
0	0	0	0	0	0	0	0	0	0	0	0	0	0	0	0	0	0
0	0	0	0	0	0	0	0	0	0	0	0	0	0	0	0	0	0
0	0	0	0	0	0	0	0	0	0	0	0	0	0	0	0	0	0
0	0	0	0	0	0	0	0	0	0	0	0	0	0	0	0	0	0
0	0	0	0	0	0	0	0	0	0	0	0	0	0	0	0	73	0
0	0	0	0	0	0	0	0	0	0	0	0	0	0	0	0	0	0
0	0	0	0	0	0	0	0	0	0	0	0	0	0	0	0	0	39
0	0	0	0	0	0	0	0	0	0	0	0	0	0	0	0	0	0
31617	39089	75	46	90	4074	341	125	2656	0	0	0	0	0	168	86	0	2826
0	0	0	0	0	0	0	0	0	0	0	0	0	0	0	0	0	0
0	0	0	162	0	0	0	0	2998	218	114	263	270	2634	1303	552	82	220
0	0	0	0	0	0	0	0	664	0	0	0	0	0	0	0	0	0
0	0	0	0	0	0	0	0	0	0	0	0	0	0	1442	3435	0	777884
0	0	0	0	0	0	0	0	0	0	0	0	0	0	0	0	0	0
0	0	0	0	0	0	0	0	0	0	0	0	0	0	0	0	0	0
0	0	0	0	0	0	0	0	0	0	0	0	0	0	0	0	0	0
0	0	0	0	0	0	0	0	0	0	0	0	0	0	0	0	0	0
0	0	0	0	0	0	0	0	0	0	0	0	0	0	0	0	0	0
0	1039	781	0	0	0	0	0	0	0	0	0	0	0	0	0	1172	0
0	0	0	0	0	0	0	0	0	0	0	0	0	0	0	0	0	0
0	0	0	0	0	0	0	0	0	0	0	0	0	0	0	0	0	0
0	0	0	0	0	0	0	0	0	0	0	0	0	0	0	0	0	0
0	0	0	0	0	0	0	0	0	0	0	0	0	0	0	0	0	0
0	0	0	0	0	0	0	0	0	0	0	0	0	0	0	0	0	0
0	0	0	0	0	0	0	0	0	0	0	0	0	0	0	0	0	0
0	0	0	0	0	0	0	0	0	0	0	0	0	0	0	0	0	0
0	0	0	0	0	0	0	0	0	0	0	0	0	0	0	0	0	0
0	0	71	0	0	0	0	0	0	0	0	0	0	0	0	0	0	0
0	1225	0	0	0	0	0	0	0	0	0	0	0	0	0	0	63	0
0	0	0	0	0	0	0	0	0	0	0	0	0	0	0	0	0	0
40779	17689	600	0	484	281	8502	180	7002	0	0	0	399	4310	283	3067	377	187
0	0	0	0	0	0	0	0	0	0	0	0	0	0	0	0	0	0
0	0	0	0	0	0	0	0	0	0	0	0	0	0	0	0	0	0
0	0	0	0	0	0	48	39	0	0	0	0	0	0	0	0	0	0
0	0	0	0	0	0	0	0	0	0	0	0	0	0	0	0	0	0

0	0	0	0	0	0	0	0	0	0	0	0	0	0	0	0	58	0
0	0	0	0	0	0	0	0	0	0	0	0	0	0	0	0	0	0
40	0	0	0	0	47	0	0	193	0	0	0	0	0	0	0	0	0
0	0	0	0	0	0	0	0	0	0	0	0	0	0	0	0	0	0
0	0	0	0	0	0	0	0	0	0	0	0	0	0	0	0	122	0
0	0	0	0	0	0	0	0	0	0	0	0	0	0	0	0	0	0
0	0	0	0	0	0	0	0	0	0	0	0	0	0	0	0	0	0
0	0	0	0	0	0	0	0	0	0	0	0	0	0	0	0	0	0
0	0	0	0	0	0	0	0	0	0	0	0	0	0	0	0	0	0
0	0	0	0	0	0	0	0	0	0	0	0	0	0	0	0	0	0
0	0	0	0	0	0	0	0	0	0	0	0	0	0	0	0	0	0
31	0	0	0	0	0	60	45	0	0	0	0	0	0	0	0	0	0
0	0	0	0	0	0	0	0	0	0	0	0	0	0	0	0	0	0
9927	0	0	0	0	0	0	0	0	0	0	0	0	0	0	0	0	0
0	0	0	0	0	0	0	0	0	0	0	0	0	0	0	0	0	0
0	0	0	0	0	0	0	0	0	0	0	0	0	0	0	0	0	0
0	0	0	0	0	0	0	0	0	0	0	0	0	0	0	0	0	0
0	0	0	0	0	0	0	0	0	0	0	0	0	0	0	0	0	0
0	0	0	0	46	0	0	0	0	0	0	0	0	0	82	189	0	45
0	0	0	0	0	0	0	0	0	0	0	0	0	0	0	0	0	0
0	182	0	0	0	0	0	0	0	0	0	0	0	0	0	0	62	0
0	0	0	0	0	0	0	0	0	0	0	0	0	0	0	0	0	0
0	0	0	0	0	0	0	0	0	0	0	0	0	0	0	0	0	0
0	0	0	0	0	0	0	0	0	0	0	0	0	0	0	0	0	0
0	0	0	0	0	0	0	0	0	0	0	0	0	0	0	0	0	0
0	832	0	0	0	0	0	0	0	0	0	0	0	0	0	0	0	0
0	0	0	0	0	0	0	0	0	0	0	0	0	0	0	0	0	0
0	0	0	0	0	0	0	0	0	0	0	0	0	0	0	0	0	0
12000	0	0	0	0	0	0	0	0	0	0	0	0	0	0	0	63	0
0	0	0	0	0	36	0	0	0	0	0	0	0	0	0	0	0	0
0	0	0	0	98	0	116	0	0	0	0	0	0	0	0	0	0	0
299	262	247	0	176	0	62	0	126	0	0	0	0	0	170	86	126776	87
0	0	0	0	0	0	0	0	0	0	0	0	0	0	0	0	0	0
0	0	0	0	0	0	0	0	0	0	0	0	0	0	0	0	0	0
0	75	77	0	0	0	0	0	0	0	0	0	0	0	0	0	0	0
0	0	0	0	0	0	0	0	0	0	0	0	0	0	0	0	0	0
0	0	0	0	0	0	0	0	0	0	0	0	0	0	0	97	0	40
0	0	0	0	0	0	0	0	0	0	0	0	0	0	0	0	0	0
0	0	0	0	0	0	0	0	0	0	0	0	0	0	0	0	0	0
0	0	0	0	0	0	0	0	0	0	0	0	0	0	0	0	0	0
0	0	0	0	0	0	0	0	0	0	0	0	0	0	0	0	0	0
0	0	0	0	0	0	0	0	0	0	0	0	0	0	0	0	142	0
0	0	0	0	0	0	0	0	0	0	0	0	0	0	0	0	0	0
0	0	0	0	0	0	0	0	0	0	0	0	0	0	0	0	0	49
0	0	0	0	0	0	0	0	0	0	0	0	0	0	0	0	0	0
32	79	73	0	0	0	0	50	185	0	0	0	0	0	0	0	74	0
0	74	75	0	0	0	0	0	0	0	0	0	0	0	0	0	0	0
0	0	0	0	0	0	0	0	0	0	0	0	0	0	0	0	0	0
0	81	0	0	0	0	0	0	0	0	0	0	0	0	0	0	64	37
0	0	0	0	0	0	0	0	0	0	1291	0	0	0	240	283	0	0
0	0	0	0	0	0	0	0	0	0	0	0	0	0	0	0	0	0
0	0	0	0	0	0	0	0	0	0	0	0	0	0	0	0	0	0
0	0	0	0	0	0	0	0	0	0	0	0	0	0	0	0	0	0
0	0	0	0	0	0	0	0	0	0	0	0	0	0	0	0	0	0
0	88	154391	0	0	0	0	0	0	0	0	0	0	0	0	0	421	0
0	0	0	0	0	0	0	0	0	0	0	0	0	0	0	0	202	0
0	0	0	0	0	0	0	0	0	0	0	0	0	0	0	0	0	0
0	0	0	0	0	0	0	0	0	0	0	0	0	0	0	0	0	0
0	0	0	0	0	0	0	0	0	0	0	0	0	0	0	0	0	0
0	0	0	0	0	0	0	0	0	0	0	0	0	0	0	0	0	0
0	0	0	0	0	0	0	0	0	0	0	0	0	0	0	0	0	0
0	0	0	0	0	0	0	0	0	0	0	0	0	0	0	0	0	0
0	0	0	0	0	0	0	0	0	0	0	0	0	0	0	0	0	0
0	0	0	0	0	0	0	0	0	0	0	0	0	0	0	0	0	0
0	0	0	0	0	0	0	0	0	0	0	0	0	0	0	0	0	0
0	0	0	0	0	0	0	0	0	0	0	0	0	0	0	0	0	0
0	0	0	0	0	0	0	0	0	0	0	0	0	0	0	0	0	0
64	0	0	0	0	0	0	0	0	0	0	0	0	0	0	0	0	0
0	0	0	0	0	0	0	0	0	0	0	0	0	0	0	0	0	0
0	0	0	0	0	0	0	0	0	0	0	0	0	0	0	0	0	0
0	0	0	0	0	0	0	0	0	0	0	0	0	0	0	0	0	0
0	0	0	0	0	0	0	0	0	0	0	0	0	0	0	0	0	0
0	0	0	0	0	0	0	0	0	0	0	0	0	0	0	0	0	0
0	0	0	0	0	0	0	0	0	0	0	0	0	0	0	0	0	0
0	0	0	0	0	0	0	0	0	0	0	0	0	0	0	0	0	0
0	0	0	0	0	0	0	0	0	0	0	0	0	0	0	0	0	0
2428	3775	0	0	0	46	114	0	0	0	0	0	126	850	70	79	0	0
38	0	0	0	0	0	15156	0	0	0	0	0	0	0	0	0	0	0
0	0	0	0	55	51	0	0	51	0	0	0	0	0	0	0	0	0
64	77	0	0	93	45419	53	87	117	0	0	0	0	0	0	0	67	0
0	0	0	0	0	0	0	0	0	0	0	0	0	0	0	0	0	0
0	6354	235	0	0	0	0	0	0	0	0	0	0	0	0	0	521	0
0	0	0	0	0	0	0	0	0	0	0	0	0	0	0	0	0	0
0	0	0	0	0	0	0	0	0	0	0	0	0	0	0	0	0	0
0	0	0	0	0	0	0	0	0	0	0	0	0	0	0	0	0	0
0	0	0	0	0	0	0	0	0	0	0	0	0	0	0	88	0	0
0	0	0	0	0	0	0	0	0	0	0	0	0	0	0	0	0	0

0	0	0	0	0	0	0	0	0	0	0	0	0	0	0	0	0	0
0	0	0	0	0	0	0	0	0	0	0	0	0	0	0	0	0	0
0	0	0	0	0	0	0	0	0	0	0	0	0	0	0	0	0	0
0	0	0	0	0	0	0	0	0	0	0	0	0	0	0	0	0	0
0	0	0	0	0	0	0	0	0	0	0	0	0	0	0	0	0	0
0	0	0	0	0	0	0	0	0	0	0	0	0	0	0	0	0	0
0	0	0	0	0	0	0	0	0	0	0	0	0	0	0	0	0	0
0	0	0	0	0	0	0	0	0	0	0	0	0	0	0	0	0	0
0	0	0	0	0	0	0	0	0	0	0	0	0	0	0	0	0	0
0	0	0	0	0	0	0	0	0	0	0	0	0	0	0	0	0	0
0	0	0	0	0	0	0	0	0	0	0	0	0	0	0	0	0	0
0	0	0	0	0	0	0	0	0	0	0	0	0	0	84	0	0	0
0	0	0	0	0	0	0	0	0	0	0	0	0	0	0	0	0	0
0	0	0	0	0	0	0	0	60	0	0	0	0	0	0	0	0	0
0	0	0	0	3475	0	0	0	6278	0	0	0	0	0	0	0	0	0
0	0	0	0	0	0	0	0	0	0	0	0	0	0	0	0	0	0
0	395	0	0	0	0	0	0	63	0	0	0	0	0	0	0	0	0
0	0	0	0	0	0	0	0	0	0	0	0	0	0	0	0	0	0
0	0	0	0	0	0	0	0	0	0	0	0	0	0	0	0	0	0
0	0	0	0	0	0	0	0	0	0	0	0	0	0	0	0	0	0
0	0	0	0	0	0	0	0	0	0	0	0	0	0	0	0	0	0
0	0	0	0	0	0	0	0	0	0	0	0	0	0	0	0	0	0
0	0	0	0	0	0	0	0	0	0	0	0	0	0	0	0	0	0
0	0	0	0	0	0	0	0	0	0	0	0	0	0	0	0	0	0
0	0	0	0	0	0	0	0	0	0	0	0	0	0	0	0	0	0
0	0	0	0	0	0	0	0	0	0	0	0	0	0	0	0	0	0
0	0	0	0	0	0	0	0	0	0	0	0	0	0	0	0	0	0
0	0	0	0	0	0	0	0	0	0	0	0	0	0	0	0	0	0
17051	0	0	0	102	42	47	89497	23245	0	0	0	33258	0	0	0	0	0
79	1843	0	0	42	97	0	44845	0	0	0	0	0	0	0	0	0	0
0	0	0	0	0	0	0	0	0	0	0	0	0	0	0	0	0	0
949	156	0	0	0	0	0	0	0	0	0	0	0	0	0	0	0	0
84289	66309	8318	510454	736609	295141	212451	616103	168228	794144	708692	283171	496028	414181	132859	385749	829743	94957
0	0	0	0	0	0	0	0	0	0	0	0	0	0	0	0	0	0
0	0	0	0	0	0	0	0	0	0	0	0	0	0	0	0	0	0
0	0	0	0	0	0	0	0	0	0	0	0	0	0	0	0	0	0
0	0	0	0	0	0	0	0	0	0	0	0	0	0	0	0	0	0
0	0	0	0	0	0	0	0	0	0	0	0	0	0	0	0	0	0
0	0	0	0	0	0	0	0	0	0	0	0	0	0	0	0	0	0
0	0	0	0	0	0	0	0	0	0	0	0	0	0	0	0	0	0
240	7139	0	0	0	0	0	34	602	0	0	0	0	0	16853	0	0	0
0	0	0	0	0	0	0	0	0	0	0	0	0	0	0	0	0	0
0	0	0	0	0	0	0	0	0	0	0	0	0	0	0	0	0	0
0	0	0	0	0	0	0	0	0	0	0	0	0	0	0	0	0	0
0	0	0	0	0	0	0	0	0	0	0	0	0	0	0	0	0	0
0	0	0	0	0	0	0	0	0	0	0	0	0	0	0	0	0	0
0	0	0	0	0	0	0	0	0	0	0	0	0	0	0	0	0	0
0	0	0	0	0	0	0	0	0	0	0	0	0	0	0	0	0	0
10473	18284	284	0	118	185	598	108	127	37	59	0	3327	33911	404	881	522	125

Control 082	Control 098	Control 116	Control 100	Control 067	Control 130	Control 129	Control 128	Control 127	Control 126	Control 125	Control 124	Control 123	Control 122	Control 121	Control 120	Control 119	Control 118
913917	896184	818591	599876	823866	822253	289192	468926	988346	853390	961371	488989	980454	755467	995765	546892	956310	413310
30375	89772	112056	319727	146655	135117	683476	500455	11103	121712	9602	471372	14641	243258	2090	52618	15795	368282
0	0	0	0	41	0	0	0	0	0	4091	0	0	0	0	0	0	0
53745	11345	33920	28832	28007	8732	27263	30056	276	24659	21086	21152	4905	1275	1195	398554	26109	216887
1963	2019	35433	51565	1431	33898	69	563	275	239	3850	18487	0	0	950	1936	1786	1521
0	0	0	0	0	0	0	0	0	0	0	0	0	0	0	0	0	0
0	680	0	0	0	0	0	0	0	0	0	0	0	0	0	0	0	0

Control 082	Control 098	Control 116	Control 100	Control 067	Control 130	Control 129	Control 128	Control 127	Control 126	Control 125	Control 124	Control 123	Control 122	Control 121	Control 120	Control 119	Control 118
0	0	0	0	8339	0	0	0	0	0	0	0	0	0	40	137	105	313837
0	0	0	223	0	0	0	0	0	0	0	0	0	0	0	0	0	0
553827	599868	479760	13786	12863	757252	3125	7940	4329	505179	11851	357547	17783	4043	11897	102118	5374	15494
0	0	0	0	0	0	0	0	0	0	0	0	0	0	0	0	0	0
0	0	0	0	124	0	0	0	146	0	0	0	0	0	0	0	0	0
0	0	0	0	0	0	0	0	0	0	0	0	0	0	0	0	0	0
0	0	0	0	0	0	0	0	0	0	0	0	0	0	0	0	0	0
0	0	0	0	0	0	0	0	0	0	0	0	0	0	0	0	0	0
0	125	0	60	0	0	0	0	0	0	0	0	0	0	0	0	0	0
118	0	0	0	0	0	0	0	0	0	0	0	0	0	0	0	0	0
0	0	0	0	0	0	0	0	0	0	0	0	0	0	0	0	0	0
0	12383	44353	217	300	217	163429	659	1324	11012	1393	385	1405	116983	548	923	432	1263
0	0	0	0	0	0	0	0	0	0	0	0	0	0	0	0	0	0
0	0	0	0	0	0	0	0	0	0	0	0	0	0	0	0	0	0
0	0	0	0	0	0	0	0	0	0	0	0	0	0	0	0	0	0
14539	14185	124	100	87	1314	0	0	0	14383	248	1486	0	0	37	337	77	0
0	0	0	0	0	0	0	0	0	0	0	0	0	0	0	0	0	0
0	0	0	0	0	0	0	0	0	0	0	0	0	0	0	0	0	0
0	0	0	0	0	0	0	0	0	0	0	0	0	0	0	0	0	0
0	40	0	148	122	83	68	127	134	42	240	0	0	161	0	0	5936	0
0	0	0	0	0	0	0	0	0	0	0	0	0	0	0	0	1545	0
0	0	0	0	0	0	0	0	0	0	0	0	0	0	0	0	0	0
0	0	0	0	0	0	0	0	0	0	0	0	0	0	0	0	1234	0
0	0	0	0	0	0	0	0	0	0	0	0	0	0	0	0	0	0
1093	740	0	955	283	0	0	0	0	0	0	0	0	0	0	0	117	0
0	0	0	0	0	0	0	0	0	0	0	0	0	0	0	0	0	0
0	0	136	0	0	0	0	0	0	0	0	0	0	771	0	0	193	0
0	0	0	0	0	0	0	0	0	0	0	0	0	0	0	0	0	0
0	66	0	41	0	0	0	0	0	0	0	0	0	0	0	0	0	0
7009	1882	97710	43466	9755	39147	1675	5887	6925	7343	853173	20309	156730	195394	917	20982	16067	7833
0	0	0	0	0	0	0	0	0	0	0	0	0	0	0	0	0	0
0	0	0	0	0	0	0	0	0	0	1210	0	457	31266	41	0	0	0
0	0	0	0	0	0	64	0	0	0	0	57	440	0	0	0	0	0
0	0	0	0	0	0	0	0	0	0	0	0	0	0	0	0	0	0
0	0	0	0	0	0	0	0	0	0	0	0	0	0	0	0	0	0
1908	0	0	0	0	199	0	0	0	0	0	0	0	0	0	119	0	0
0	0	0	0	41	0	0	0	0	0	0	0	0	0	0	0	0	0
0	0	0	0	0	0	0	0	0	0	0	0	0	0	0	0	0	0
0	0	0	0	0	0	0	0	0	0	0	0	0	0	0	0	0	0
0	0	549	0	0	326	340	0	0	0	0	0	0	0	0	0	0	0
62	166	0	46	0	285	0	0	0	475	0	0	0	0	0	0	0	0
0	0	0	57	0	0	0	0	0	0	0	0	0	66	0	0	0	0
0	0	0	0	0	0	0	0	0	0	0	0	0	0	0	0	0	0
0	0	0	0	0	0	0	0	0	0	0	0	0	0	0	0	0	0
0	0	0	0	0	0	0	0	0	57	0	0	0	0	0	0	0	0
59	0	0	56	0	0	0	0	0	0	0	0	0	0	0	0	0	0
0	0	0	0	0	0	0	0	0	0	0	0	0	0	0	0	0	0
566	303	88303	14248	851	1225	10669	2143	1391	1006	1778	402	6448	5415	600	2026	14399	57758
0	0	0	0	0	0	0	0	2078	0	87	8411	0	0	0	0	0	0
0	43	40	0	0	52	287	121	162	118	230	0	0	0	514	0	0	99
0	0	0	0	62	0	0	0	0	0	0	0	0	0	0	0	0	0
0	0	0	0	0	0	0	0	0	90	90	0	0	32011	66	0	0	0
0	0	0	0	0	0	0	0	0	0	0	0	0	0	0	0	0	0
0	0	0	45	0	0	0	0	0	0	0	0	0	0	0	0	0	0
0	0	0	0	0	0	0	0	0	0	0	0	0	0	0	0	0	0
0	0	0	0	0	0	0	0	0	0	0	0	0	0	0	0	0	0
0	0	0	0	0	0	0	0	0	0	0	0	0	0	0	0	0	0
421	2112	0	0	0	38	0	0	0	33	0	0	0	0	0	0	0	0
0	0	0	0	0	0	0	0	0	0	0	0	0	0	0	0	0	0
0	0	0	0	0	0	0	0	0	0	0	0	0	0	0	0	0	0
333	306	199	65616	668	68	0	543	293	97	14819	43	450	158	113	110532	108	1166
0	0	0	0	6905	0	0	0	0	0	0	0	0	0	0	0	0	0
0	0	0	0	0	0	0	0	0	0	0	0	0	0	0	0	0	0
0	0	0	0	0	0	0	0	0	0	0	0	0	0	0	0	0	0
0	0	0	0	0	0	0	0	0	0	0	0	0	0	0	0	0	0
0	0	0	0	0	0	0	0	0	0	0	0	0	0	0	0	0	0
0	445	0	0	0	0	62	0	132	0	0	0	0	0	290	0	0	0
52	0	0	0	0	0	0	0	0	0	0	0	0	0	0	0	0	0
0	0	0	0	0	0	0	0	0	0	0	0	0	0	0	0	0	0
0	0	0	0	0	0	0	0	0	0	0	0	0	0	0	0	0	0

0	0	0	0	0	0	0	0	0	0	0	0	0	0	0	0	0	0
0	0	0	0	0	38	50	0	0	107	0	0	0	0	0	0	0	0
0	0	0	0	0	0	0	0	0	0	0	0	0	0	0	0	0	0
0	0	0	0	42	0	0	0	0	0	0	0	0	0	0	0	0	0
0	0	0	0	0	0	0	0	0	0	0	0	0	0	0	0	0	0
0	0	0	0	0	0	0	0	0	0	0	0	0	0	0	0	0	0
2050	0	170	0	107	0	315	423	129	1330	179	94	460	23385	0	225	78	444
0	0	0	0	0	0	0	0	0	0	0	0	0	0	0	0	0	0
0	0	0	0	41	0	0	0	0	0	4091	0	0	0	0	0	0	0
0	0	0	0	0	0	0	0	0	0	0	0	0	0	0	0	0	0
2350	652	0	0	0	0	74	120	0	0	0	10556	0	0	0	0	0	0
0	0	0	0	0	34	0	0	0	0	0	0	0	0	0	0	0	0
21966	195891	201	229820	100523	662	527	1218	4126	258	4230	13337	1862	251123	1726	47292	35581	942
0	0	0	0	0	32	66	274	0	0	4874	0	0	147	0	0	0	0
0	0	0	0	0	0	0	0	0	0	0	0	0	0	0	0	0	0
0	0	0	0	0	0	0	0	0	0	0	0	0	0	0	0	0	0
0	0	0	0	0	0	0	0	0	0	0	0	0	0	0	0	0	0
0	0	0	0	0	0	0	0	0	0	0	0	0	0	0	0	0	0
0	0	0	0	0	0	0	0	0	0	0	0	0	0	0	43148	34	91
0	0	0	0	0	0	0	0	0	0	0	0	0	0	0	0	0	0
0	0	0	0	0	0	0	0	0	0	0	0	0	0	0	0	0	0
0	0	0	0	0	0	0	0	0	0	0	0	0	0	0	0	0	0
51	81	0	48377	0	0	159	124	0	0	0	19348	0	138	0	0	0	0
0	0	0	0	0	0	0	0	0	0	0	0	0	0	0	0	0	0
56	36	0	0	0	0	0	0	0	55	0	0	0	0	0	0	0	0
0	0	0	0	0	0	0	0	0	0	0	0	0	0	0	0	0	0
0	0	0	0	0	0	0	0	0	0	0	0	0	0	0	0	0	0
4625	15391	26733	48182	55022	1618	372230	265592	3096	20953	2854	396257	6837	50761	528	51239	7265	360728
0	0	0	0	0	0	0	0	0	0	0	0	0	0	107	0	0	0
0	0	0	0	0	0	0	0	0	0	0	0	0	0	0	0	0	0
0	0	0	0	38	0	0	0	0	0	93	0	0	0	228	0	0	0
0	0	0	0	0	0	0	0	0	0	0	0	0	0	0	0	0	0
0	0	0	0	0	0	0	0	0	0	0	0	0	0	0	0	0	0
0	0	0	0	0	0	0	0	0	0	0	0	0	0	0	0	0	0
3666	90	0	186	0	0	0	0	0	0	273	0	0	0	0	0	0	0
0	0	0	0	0	0	0	0	0	0	0	0	0	0	0	0	0	0
0	0	0	0	0	0	0	0	0	0	0	0	0	0	0	0	0	0
0	33	0	0	0	0	0	0	0	0	0	0	0	0	0	0	0	0
0	35	85	97	121	0	0	0	0	0	486	0	0	0	0	0	40	114
0	0	0	0	41	0	0	0	0	0	0	0	0	0	0	0	0	0
0	0	0	0	0	0	0	0	0	0	0	0	0	0	0	0	0	0
0	0	0	0	0	0	0	0	0	0	0	0	0	0	0	0	0	0
0	0	0	0	46	0	0	0	0	0	0	0	0	0	0	0	0	0
0	146	87787	146514	0	231	508	393	769	264	244	200	2246	621	1809	347	106	357
0	0	0	0	0	0	0	0	0	0	0	0	0	0	0	0	0	0
0	0	0	0	0	0	0	0	0	0	0	0	0	0	0	0	0	0
0	0	0	0	0	0	0	0	0	0	0	0	0	0	0	0	0	0
0	154	0	67	0	0	0	0	0	0	0	0	0	0	0	0	0	0
0	0	0	0	497	0	0	0	0	0	23193	0	0	0	0	0	0	0
0	0	0	0	0	0	0	0	0	0	87	0	0	0	0	0	0	0
1963	1941	29024	15530	1190	33898	69	563	0	195	3691	18487	0	0	950	1936	1749	1521
0	0	0	0	0	45	76	136	0	89	0	0	0	58	0	0	0	0
0	0	0	0	0	0	0	0	0	0	0	0	0	0	0	0	0	0
0	0	0	0	0	0	0	0	0	0	0	0	0	0	0	0	0	0
0	0	0	0	0	0	0	0	0	0	0	0	0	0	0	0	0	0
0	0	0	0	0	0	0	0	0	0	0	0	0	0	0	0	0	0
0	0	0	0	0	0	0	0	0	0	0	0	0	0	0	0	0	0
0	0	0	0	0	0	0	0	0	0	0	0	0	0	0	0	0	0
0	0	0	0	0	0	0	0	0	0	0	0	0	0	0	0	0	0
0	0	0	0	0	0	0	0	0	0	0	0	0	0	0	0	0	0
0	37	0	57	0	0	0	0	0	0	0	0	0	0	0	0	0	0
0	0	0	0	0	0	0	0	0	0	0	0	0	0	0	0	0	0
0	24	0	0	38	0	0	0	0	0	0	187	0	0	40	0	0	0
0	0	0	0	0	0	0	0	0	0	0	0	0	0	0	0	0	0
0	0	0	0	0	0	0	0	0	0	0	0	0	0	0	0	0	0
0	0	0	0	0	0	0	0	0	0	0	0	0	0	0	0	0	0
0	0	0	0	0	0	0	0	0	0	0	0	0	0	0	0	0	0
0	42	0	0	0	0	0	0	0	0	0	0	0	0	0	0	0	0
0	0	0	0	0	0	0	0	0	0	0	0	0	0	192	0	0	0
0	0	0	7898	0	0	0	0	0	0	0	0	0	0	0	0	0	0
0	0	0	0	0	0	0	0	0	0	0	0	0	0	0	0	0	0
0	0	0	0	0	0	0	0	0	0	0	0	0	0	0	0	0	0
0	0	0	0	0	0	0	0	0	0	0	0	0	0	0	0	0	0
0	0	0	0	0	0	0	0	0	0	0	0	0	0	0	0	0	0
5284	6037	42	211	129	481	152	0	0	13527	74	720	906	97	72	109	82	0
4961	45695	48	99	227	30809	0	281	659	39978	914	89	905	199	84	341	185	115
0	70	0	322	0	0	0	266	0	79	662	51	463	0	0	0	0	0
0	0	0	0	0	0	0	0	0	0	0	0	0	0	0	0	0	0
0	0	0	0	0	0	0	0	0	0	0	0	0	0	0	0	0	0
0	31	0	54	0	0	0	0	0	2508	0	0	0	0	0	0	0	0
0	0	0	0	67	0	0	0	119	0	0	0	0	0	0	0	0	0
0	0	0	0	0	0	0	0	0	0	0	0	0	0	0	0	0	0
0	0	0	0	0	0	0	0	0	0	0	0	0	0	0	0	0	0
53745	11321	33920	28832	27969	8732	27263	30056	276	24659	21086	20965	4905	1275	1155	398554	26109	216887
0	4480	0	56	0	39	0	0	0	0	0	0	438	0	116	120	0	0
0	0	123	0	0	0	0	0	0	0	0	0	0	0	0	0	251	0
0	0	0	0	0	0	0	0	0	0	0	0	0	0	0	0	0	0

0	0	0	0	0	0	0	0	0	0	0	0	0	0	0	0	0	0
132	0	0	49	741	0	219	0	126	128	388	0	0	0	62	0	0	290
0	0	0	0	0	0	0	146	0	0	0	0	0	0	0	0	0	0
0	0	0	178	35	428	0	0	0	0	0	49	0	0	0	0	39	0
0	0	0	0	0	0	0	0	0	0	0	0	0	0	0	0	0	0
0	0	0	0	0	0	0	0	0	0	0	0	0	0	0	0	0	0
0	0	0	0	0	0	0	0	0	0	0	0	0	0	0	0	0	0
0	0	0	0	0	0	0	0	0	0	0	0	0	0	0	0	0	0
0	163	0	0	0	0	0	0	0	0	0	50	0	0	0	0	0	0
0	0	0	0	0	0	0	0	0	0	0	0	0	0	0	0	0	0
0	0	0	0	0	0	0	0	0	0	0	0	0	0	0	0	0	0
0	78	0	36035	0	0	0	0	0	0	0	0	0	0	0	0	0	0
0	0	0	0	0	0	0	0	0	0	0	0	0	0	0	0	0	0
0	0	45	0	33	0	86	230830	416	86	173	49	458	0	0	0	38	218
0	0	0	0	0	0	0	0	0	0	0	0	0	0	0	0	0	0
0	0	0	0	0	0	0	0	0	0	0	0	0	0	0	0	0	0
0	0	0	0	0	0	0	0	0	0	171	0	0	0	0	0	0	0
0	0	0	0	0	0	0	0	0	0	0	0	0	0	0	0	0	0
0	0	0	0	0	0	0	0	0	0	0	0	0	0	0	0	0	0
1133	0	11960	0	0	0	0	0	0	0	0	67	459	0	33	115	0	0
0	0	0	0	75	0	0	0	0	0	0	1289	0	0	0	0	0	0
0	0	0	0	0	0	0	0	0	0	0	0	0	0	0	0	0	0
0	0	0	0	0	0	0	0	0	0	0	0	0	0	0	0	0	0
77	50	0	0	0	0	0	0	0	0	0	269	0	0	0	0	0	0
0	0	0	0	47	0	0	0	0	0	0	0	0	0	0	0	0	0
0	0	0	0	0	0	0	0	0	0	0	0	0	0	0	0	0	0
0	0	0	0	0	0	0	0	0	0	0	0	0	0	0	0	0	0
0	0	0	0	0	0	0	0	0	0	0	0	0	0	0	0	0	0
0	0	0	0	0	0	0	0	0	0	0	0	0	0	0	0	0	0
0	0	0	0	0	0	0	0	0	0	231	0	0	0	0	0	0	0
0	0	0	0	0	0	0	0	0	0	0	0	0	0	0	0	0	0
0	0	0	0	0	0	0	0	0	0	0	49	0	0	0	0	0	0
0	0	0	0	0	0	0	0	0	0	0	0	0	0	0	0	0	0
0	0	0	0	0	0	0	0	0	0	0	0	0	0	0	0	0	0
0	0	0	0	0	0	0	0	0	0	0	0	0	0	0	0	0	0
48	39	124	0	0	0	0	0	0	0	0	0	0	0	0	126	2809	118
0	0	0	0	0	0	0	0	0	0	0	0	0	0	0	0	0	0
0	0	0	0	0	0	0	0	0	0	0	0	0	0	0	0	0	0
63	0	0	0	0	0	0	0	0	0	0	0	0	0	35	0	0	0
0	0	0	0	0	0	0	0	0	0	0	0	0	0	0	0	0	0
0	0	0	0	0	0	0	0	0	0	0	0	0	0	0	0	0	0
0	0	0	0	0	0	0	0	0	0	0	0	0	0	0	0	0	0
0	0	0	0	0	0	0	0	0	0	0	0	0	0	0	0	0	0
0	0	0	0	0	0	0	0	0	0	0	0	0	0	0	0	0	0
0	0	0	0	0	0	0	0	0	0	0	0	0	0	0	0	0	0
0	0	0	0	0	0	0	0	0	0	0	0	0	0	0	0	0	0
0	0	0	0	0	0	0	0	0	0	0	0	0	0	0	0	0	0
0	0	0	0	0	0	0	0	0	0	0	0	0	0	0	0	0	0
1250	987	0	0	0	0	0	0	0	0	0	0	0	0	903	0	0	0
0	0	0	0	0	0	0	0	0	0	0	0	0	0	0	0	0	0
0	0	0	0	0	0	0	0	0	0	0	0	0	0	0	0	0	0
0	0	0	0	0	0	0	0	0	0	0	0	0	0	0	0	0	0
0	0	0	0	0	0	0	0	0	0	0	0	0	0	0	0	0	0
0	82	0	53	85	0	0	0	0	0	0	3753	0	62	0	0	0	0
0	0	0	0	0	0	0	0	0	0	0	0	0	0	0	0	0	0
0	0	0	583	0	0	0	0	0	0	0	0	0	0	0	0	0	0
0	0	0	0	0	0	0	0	0	0	0	0	0	0	0	0	0	0
0	0	0	0	0	0	0	0	0	0	0	0	0	0	0	0	0	0
0	0	0	0	0	0	0	0	0	0	0	0	0	0	0	0	0	0
0	0	0	0	0	0	0	0	0	0	0	0	0	0	0	0	0	0
0	78	0	111	0	0	0	129	0	0	0	0	0	0	0	0	0	0
0	0	0	0	0	0	0	0	0	0	0	0	0	0	0	0	0	0
0	0	0	52	0	0	0	0	0	0	0	0	0	0	0	0	0	0
0	69	0	111	0	0	0	0	0	0	0	0	0	0	0	0	0	0
0	0	0	0	0	0	0	0	0	0	0	0	0	0	0	0	0	0
0	0	0	0	0	0	0	0	0	0	72	0	0	70	0	0	0	103
0	0	0	0	0	0	0	0	0	0	0	0	0	0	0	0	0	0
0	0	0	0	0	0	0	0	0	0	0	0	0	0	0	0	0	0
0	0	0	0	0	0	0	0	0	0	0	0	0	0	0	0	0	0
0	0	0	0	0	0	0	0	0	0	0	0	0	0	0	0	0	0
0	0	0	0	44	0	0	0	0	0	0	0	0	0	0	0	0	0
0	0	0	0	0	0	0	0	0	0	0	50	449	0	0	0	0	0
50	0	0	0	0	0	0	0	0	0	0	0	0	0	0	0	0	0
13016	3323	78976	68357	76995	101568	310180	1847	6679	15830	1317	4172	3232	191575	765	251	1232	5808
0	0	0	0	0	0	0	0	0	0	0	0	0	0	0	0	0	0
0	547	0	0	0	0	0	0	0	0	0	0	0	0	0	0	0	0
3207	0	0	0	0	0	0	0	0	0	75	0	0	0	0	0	0	0
0	0	0	0	0	0	0	0	0	0	0	0	0	0	0	0	0	0
68	4051	0	922	0	269	194	574	131	223436	830	184	460	599	10505	113	0	90
0	0	0	0	0	0	0	0	0	0	0	0	0	0	0	0	0	0
0	0	0	0	0	0	0	0	0	0	0	0	0	0	0	0	0	0
0	0	0	0	0	0	0	0	0	0	0	0	0	0	0	0	0	0
0	0	0	0	0	0	0	0	0	0	0	0	0	0	0	0	0	0
0	0	0	0	117	0	0	0	129	44	159	0	0	0	0	0	0	0
0	0	0	0	0	0	0	0	0	0	0	0	0	0	0	0	0	0
1621	0	0	16444	0	0	0	0	0	0	0	0	0	0	0	0	0	0
0	572	72	75947	0	41	0	0	0	0	1258	0	0	0	37	0	34	124

0	0	0	0	0	0	0	0	0	0	0	0	0	0	0	0	0	0
0	0	0	0	0	0	0	0	0	0	0	0	0	0	0	0	0	118
0	0	0	0	0	0	0	0	0	0	0	0	0	0	0	0	0	0
0	0	0	0	0	0	0	0	0	0	0	0	0	0	0	0	0	0
0	0	0	0	0	0	0	0	0	0	0	0	0	0	0	0	0	0
0	0	0	0	0	0	0	0	0	0	0	0	0	0	0	0	0	0
0	0	0	0	0	0	0	0	0	0	0	0	0	0	0	0	0	0
0	0	0	0	0	0	0	0	0	0	0	0	0	0	0	0	0	0
0	0	0	0	0	0	0	0	0	0	0	0	0	0	0	0	0	0
0	0	0	0	0	0	0	0	0	0	0	0	0	0	0	0	0	0
0	0	0	0	0	0	0	0	0	0	0	0	0	0	0	0	0	0
0	0	0	0	42	0	0	0	0	0	0	0	0	0	0	0	0	0
0	0	0	0	0	0	0	0	401	0	0	0	0	0	0	0	0	0
0	4354	0	265	0	35	0	0	0	8176	0	0	0	0	0	0	0	0
0	0	0	0	0	0	0	0	0	0	0	0	0	0	0	0	0	0
0	0	0	0	0	126	69	140	127	0	89	32966	0	137	0	0	0	0
0	0	0	0	0	0	0	0	0	0	0	0	0	0	0	0	0	0
0	0	0	0	38	0	0	0	0	0	0	0	0	0	0	0	0	0
0	0	0	0	0	0	0	0	0	0	0	0	0	0	0	0	0	0
0	0	0	0	0	0	0	0	0	0	0	0	0	0	0	0	0	0
0	0	0	0	0	0	0	0	0	0	0	0	0	0	0	0	0	0
0	0	0	0	0	0	0	0	0	0	0	0	0	0	0	0	0	0
136	41	0	0	0	147	147	144	280	72	252	256	0	69	0	0	2723	0
0	0	0	0	0	0	0	0	0	0	0	0	0	0	0	0	0	0
0	0	0	0	0	0	0	0	0	0	0	0	0	0	0	0	0	0
0	0	32	0	0	0	0	0	0	0	0	0	0	0	0	0	39	115
0	0	0	0	0	0	0	0	0	0	0	0	0	0	0	0	0	0
7004	9737	206	125	296	5444	0	0	258	8450	0	1208	0	0	112	30469	306	225
0	0	0	0	0	0	0	0	0	0	0	0	0	0	206	0	0	0
0	0	0	0	0	0	0	0	0	0	0	0	0	0	0	0	0	0
0	0	0	0	0	0	0	0	0	0	0	0	0	0	0	0	0	0
0	0	0	0	0	0	0	0	0	0	0	0	0	0	0	0	0	0
0	0	0	0	0	0	0	0	0	0	0	0	0	0	0	0	0	0
0	0	0	0	0	0	0	0	0	0	0	0	0	0	0	0	0	0
2278	114	0	55	37	0	0	0	123	0	0	0	0	0	0	117	0	111
0	0	0	0	0	0	0	0	0	0	0	0	0	0	0	0	0	0
0	0	0	0	0	0	0	0	0	0	0	0	0	0	0	0	0	0
0	0	0	0	0	0	0	0	0	0	0	0	0	0	0	121	0	0
412	0	2318	56	0	158	416	3232	960104	366	1505	2261	2697	3535	0	0	0	0
0	0	0	0	0	0	0	0	0	0	0	0	0	0	0	0	0	0
0	0	0	0	0	0	0	0	0	0	0	0	0	0	27	0	90	0
0	0	0	0	0	0	0	0	0	0	0	0	0	0	0	0	0	0
117	33	0	0	0	678	0	272	0	80	0	0	437	0	89	0	0	0
0	0	0	0	0	0	0	0	0	0	0	0	0	0	0	0	0	0
0	0	0	0	0	0	0	0	0	0	0	0	0	0	0	0	5791	0
0	0	0	0	0	0	0	0	0	0	0	0	0	0	0	0	0	0
0	0	0	0	0	0	0	0	0	0	0	0	0	0	0	0	0	0
0	0	0	0	0	0	0	0	0	0	0	0	0	0	0	0	0	0
0	0	0	0	0	0	0	0	0	0	0	0	0	0	0	0	0	0
0	0	0	0	0	0	0	141	0	0	0	14256	459	0	0	0	0	0
4736	0	291	0	50	0	0	0	0	0	0	40	0	0	153	692	299	1287
0	0	0	0	0	0	0	0	0	0	0	0	0	0	0	0	0	0
0	0	0	0	0	0	0	0	0	0	0	0	0	0	0	0	0	0
0	0	0	0	0	0	0	0	0	0	0	0	0	0	0	0	0	0
0	0	0	0	0	0	0	0	0	0	0	0	0	0	0	0	0	0
0	0	0	0	0	0	0	0	0	0	0	0	0	0	41	100	67	99
0	0	0	0	0	0	0	0	0	0	0	0	0	0	0	0	0	0
0	0	0	0	0	0	0	0	0	0	0	0	0	0	0	0	37	0
0	0	0	0	0	0	0	0	0	0	0	0	0	0	0	0	0	0
0	0	0	0	0	0	0	0	0	0	0	0	0	0	0	0	0	0
0	0	0	0	34	0	0	0	0	0	179	0	0	0	0	0	0	0
0	0	51	57	0	0	0	0	0	0	0	0	0	0	206	0	146	104
0	0	0	0	0	0	0	0	0	0	0	0	0	0	0	0	0	0
0	47	0	0	400	317	282	394	0	38601	367	179	0	222	0	0	0	0
0	0	0	0	0	0	0	117	0	0	0	0	0	0	0	0	0	0
0	0	0	0	0	0	0	0	0	0	0	0	0	0	0	0	0	0
0	0	0	0	0	0	0	0	0	0	0	0	0	0	0	123	41	0
0	0	0	0	0	0	0	0	0	0	0	0	0	0	0	0	0	0
0	0	0	0	11601	0	0	0	0	0	0	0	0	0	0	0	0	0
0	30	0	107	0	0	0	0	0	0	0	6659	0	0	0	0	0	0
0	0	35	0	0	0	0	0	0	0	0	0	0	0	0	0	0	0
0	0	0	0	0	0	0	0	0	0	0	0	0	0	0	0	0	0
0	0	0	0	0	0	0	0	0	0	0	0	0	0	0	0	0	0
0	0	0	0	0	0	0	0	0	0	0	0	0	0	0	0	0	0
0	0	0	0	47	0	0	0	0	0	0	0	0	0	0	0	0	0
0	0	0	0	0	0	0	0	0	0	0	0	0	0	0	0	0	0
0	0	0	0	0	0	0	0	0	0	0	0	0	0	0	0	0	0
0	0	0	0	0	0	0	0	0	0	0	0	0	0	0	0	0	0
0	0	0	0	0	0	0	0	0	0	0	0	0	0	0	0	0	0
0	0	36	13641	0	77	104714	146	0	0	0	91	1371	139	0	110	102	320
0	0	0	0	0	0	0	0	0	0	0	0	0	0	0	0	0	0
0	0	0	0	44	40	79	116	1056	48	78	75	455	284	0	0	0	0
0	0	0	0	0	0	0	0	0	0	0	0	0	0	0	0	0	0
0	0	0	0	0	0	0	0	0	0	0	0	0	0	0	0	0	0
0	0	0	20655	0	0	0	0	0	0	0	0	0	0	0	0	0	0
0	0	0	0	0	0	0	0	0	0	0	0	0	0	0	0	0	0
2463	1144	209	220	8792	35	0	0	0	135	0	1961	0	0	2951	0	172	0

0	0	0	0	0	30	0	0	0	0	62	0	0	76	144	0	0	0
0	0	0	0	0	0	0	0	0	0	0	0	0	0	0	0	0	0
0	0	0	0	101	0	0	0	0	0	0	0	0	0	0	0	0	0
0	0	0	0	0	0	0	0	0	0	0	0	0	0	0	0	0	0
0	0	0	0	0	91	132	127	0	143	146	0	920	67	41	0	64	0
0	0	0	0	0	0	0	0	0	0	0	0	0	0	0	0	0	0
0	0	0	0	0	0	0	0	0	0	0	0	0	0	0	0	0	0
0	0	6409	0	0	0	0	0	0	0	0	0	0	0	0	0	37	0
0	0	0	0	0	0	0	0	0	0	0	0	0	0	0	0	0	0
0	0	0	0	0	0	0	0	0	0	595	0	0	0	0	0	0	0
0	0	0	0	0	0	0	0	0	0	0	0	0	0	0	118	9346	0
0	0	0	0	0	0	0	0	0	0	0	0	0	0	0	0	0	0
0	0	0	0	0	0	0	0	0	0	0	0	0	0	0	0	0	0
0	0	0	0	0	0	0	0	0	0	0	0	0	0	0	0	0	0
0	0	0	0	0	0	0	0	0	0	0	0	0	0	0	0	0	0
0	0	0	0	0	0	0	0	0	0	0	0	0	138	0	0	0	0
197	108	5557	2179	1617	31	0	263	0	47	0	49407	458	0	169	350	501	299
0	0	0	0	0	0	0	0	0	0	0	0	0	0	0	0	0	0
0	90	0	47	20018	94	0	0	0	0	12991	0	0	118	32	6470	6941	0
0	0	0	0	0	0	0	0	0	0	0	0	0	0	0	0	0	0
0	0	0	0	1155	0	0	0	0	0	0	0	0	0	0	0	0	0
1646	0	0	0	0	0	0	0	0	0	0	0	0	0	0	0	0	0
0	871	0	506	0	0	0	0	0	41	0	0	0	0	0	0	0	0
0	0	0	0	0	0	0	0	0	0	0	0	0	0	0	0	0	0
0	0	0	0	0	0	0	0	0	0	0	0	0	0	0	0	0	0
0	0	0	0	0	0	0	0	0	0	0	0	0	0	0	0	0	0
0	0	0	0	0	0	0	0	0	0	0	0	0	0	0	0	0	0
0	0	0	0	0	0	0	0	0	0	0	0	0	0	0	0	0	0
0	0	0	0	0	0	0	0	0	0	0	0	0	0	0	0	0	0
0	0	0	0	0	0	0	0	0	0	0	0	0	0	0	0	0	0
0	0	0	0	0	0	0	0	0	0	0	0	0	0	0	0	0	0
40914	18611	143	292	35	1664	292	251	0	47146	0	3642	0	132	73	711	105	553
0	0	0	0	0	0	0	0	0	0	0	0	0	0	0	0	0	0
0	0	0	0	0	0	0	0	0	0	0	0	0	0	0	0	0	0
0	0	0	0	0	0	0	0	0	0	0	0	0	0	0	0	0	0
0	0	0	0	0	0	0	0	0	0	0	0	0	0	0	0	0	0
0	0	0	0	0	0	0	0	0	0	0	0	0	0	0	0	0	0
60	0	0	0	0	0	0	0	0	0	0	0	0	0	0	0	0	0
0	0	0	167	0	0	0	0	0	0	0	0	0	0	0	0	0	0
0	0	0	0	0	0	0	0	0	0	0	0	0	0	0	0	0	0
0	0	0	0	0	0	0	0	0	0	0	0	0	0	0	0	0	0
48	0	0	0	0	0	0	0	0	0	0	0	0	0	0	0	0	0
0	0	0	0	0	0	0	0	0	0	0	0	0	0	0	0	0	0
0	0	0	0	0	0	0	0	0	0	0	0	0	0	0	0	0	0
0	2133	0	47	0	0	0	0	0	0	0	0	0	0	0	0	0	0
0	0	0	0	0	0	0	0	0	0	0	0	0	0	0	0	0	0
0	0	0	0	0	43	0	0	0	0	0	0	0	0	29	118	81	0
0	0	0	0	0	0	0	0	0	0	0	0	0	0	339	0	0	0
0	31	0	0	0	1795	0	0	0	324	0	0	0	0	0	0	0	0
0	0	0	0	0	0	0	0	0	0	0	0	0	0	0	0	0	0
0	0	0	0	0	0	0	0	0	0	0	0	0	0	0	0	0	0
0	0	44	0	0	0	0	0	0	0	0	0	0	0	36	0	247	574
0	0	0	0	0	0	0	0	0	0	0	0	0	0	0	0	0	0
75908	39	87	52	106	140	203	364	675	76	1096	0	469	66675	34	170227	81	762
0	0	0	0	0	0	0	0	0	0	0	0	0	0	0	0	0	0
0	0	0	0	40	0	0	0	0	0	0	0	0	0	0	100	43	0
0	0	0	0	0	0	0	0	0	0	0	0	0	0	0	0	0	0
0	0	0	0	0	0	0	0	0	0	0	0	0	0	0	0	0	0
0	0	0	0	0	0	0	0	0	0	0	0	0	0	0	0	0	0
0	0	0	0	0	0	0	0	0	0	0	0	0	0	0	0	0	0
0	107	0	144	0	0	0	0	0	0	0	0	0	0	0	0	0	0
0	0	0	0	0	0	0	0	0	0	0	0	0	0	38	0	38	0
0	0	0	0	0	0	84	0	0	580	0	0	0	7769	0	0	2715	0
62	0	0	73862	0	0	0	0	0	0	0	0	0	0	0	0	0	0
0	0	0	0	0	0	0	0	0	0	0	0	0	0	0	0	0	0
0	0	50	0	117	0	0	0	0	0	0	0	0	0	0	0	0	0
0	0	0	0	0	0	0	0	0	0	0	0	0	0	0	0	0	0
0	0	0	0	0	0	0	0	0	0	0	0	0	0	0	0	0	0
0	155	0	206	0	0	0	0	0	0	0	53	0	0	0	0	0	0
0	0	0	0	0	0	0	0	0	0	0	0	0	0	0	0	0	0
0	0	0	0	0	0	0	0	0	0	0	0	0	0	0	0	0	0
0	0	0	0	0	0	0	0	150	2281	0	0	0	0	0	0	0	0
0	0	0	0	0	0	0	0	0	0	0	0	0	0	0	0	0	0
2795	0	0	0	0	0	0	0	0	0	0	0	0	0	0	0	0	0
0	0	0	0	0	282	73	347997	133	78	245	89	1849	272	0	0	0	0
0	0	0	0	0	0	0	0	0	0	0	0	0	0	0	0	0	0
0	0	0	0	0	0	0	0	0	0	267	0	0	0	0	0	0	0
0	0	0	0	0	0	0	0	0	0	0	0	0	0	0	0	0	0
748	0	565	257	0	0	0	0	0	0	0	0	0	0	958073	1598	499	1912
0	0	0	0	0	0	0	0	0	0	0	0	0	0	0	0	0	0
836	928	0	0	40	228	83	0	0	3869	0	90	0	0	0	130	0	0
0	0	0	0	0	0	0	0	0	0	0	0	0	0	0	0	0	0
0	0	0	0	0	0	0	0	0	0	0	0	0	0	0	0	0	0
0	21635	0	110	0	0	0	0	0	0	0	0	0	0	0	0	0	0
0	449	0	585	0	0	0	0	0	0	0	0	0	0	39	0	0	0
0	0	0	0	0	0	0	0	0	0	0	0	0	0	0	0	0	0
0	0	0	0	0	0	0	0	0	0	0	0	0	0	0	0	0	0

0	0	0	0	0	0	0	0	0	0	0	0	0	74	0	0	0	0
0	0	0	0	0	0	0	0	0	0	0	0	0	0	0	0	0	0
0	0	0	0	0	0	0	0	0	0	0	0	0	0	0	0	0	0
63	0	0	0	0	0	0	0	0	0	0	0	0	0	0	0	0	0
0	0	0	0	0	0	0	0	0	0	0	0	0	0	71	0	0	0
0	0	0	0	0	0	0	0	0	0	0	0	0	0	0	0	0	0
0	0	0	0	0	0	0	0	0	0	0	0	0	0	0	0	0	0
0	0	0	0	0	0	0	0	0	0	0	0	0	0	0	0	0	0
0	0	0	0	0	0	0	0	0	0	0	0	0	0	0	0	0	0
0	0	0	0	35	82	67	585	0	251	260	102	1399	136	0	0	37	202
1651	352	0	52	234	0	0	0	0	3296	98	0	0	0	0	0	0	0
0	0	0	0	0	0	0	0	0	0	0	0	0	0	0	0	0	0
0	0	0	0	0	0	0	0	0	0	0	0	0	0	0	0	0	0
0	0	0	0	0	0	0	0	0	0	0	0	0	0	0	0	0	0
0	0	0	0	0	0	0	0	0	0	0	0	0	0	0	0	0	0
0	0	0	0	0	0	0	0	0	0	0	0	0	0	0	0	0	0
0	0	0	0	0	0	0	0	0	0	0	0	0	0	0	0	0	0
0	0	0	0	0	0	0	0	0	0	0	0	0	0	0	0	0	0
0	0	0	0	0	0	0	0	0	0	81	0	0	0	0	0	0	0
53	0	0	0	0	0	0	0	0	0	0	0	0	0	0	0	0	0
156504	13421	3449	22105	650451	9332	1472	95319	3003	2506	22915	7702	781693	13779	2687	7356	848233	7919
0	0	0	0	0	0	0	0	121	73	0	0	0	0	0	0	0	0
0	0	0	0	0	0	0	0	0	0	0	0	0	0	0	0	0	0
0	680	0	0	0	0	0	0	0	0	0	0	0	0	0	0	0	0
0	0	0	0	0	0	0	0	0	0	0	0	0	0	0	0	0	0
0	0	0	0	0	0	0	0	0	0	0	0	0	0	0	0	0	0
0	0	0	0	0	0	0	0	0	0	0	0	0	0	0	0	0	0
0	0	34	0	0	0	0	0	0	0	1950	51	0	0	0	0	0	0
0	0	0	0	0	0	0	0	0	0	0	0	0	0	0	0	0	0
Control08	Control09	Control11	Control10	Control06	Control13	Control12	Control12	Control12	Control12	Control12	Control12	Control12	Control12	Control12	Control12	Control11	Control11
0	0	0	0	8339	0	0	0	0	0	0	0	0	0	0	0	0	0
0	0	0	0	0	0	0	0	0	0	0	0	0	0	40	137	67	313710
0	0	0	0	0	0	0	0	0	0	0	0	0	0	0	0	0	0
0	0	0	0	0	0	0	0	0	0	0	0	0	0	0	0	0	0
0	0	0	0	0	0	0	0	0	0	0	0	0	0	0	0	38	127
0	0	0	0	0	0	0	0	0	0	0	0	0	0	0	0	0	0
0	0	0	223	0	0	0	0	0	0	0	0	0	0	0	0	0	0
553827	599868	479760	13786	12863	757252	3125	7940	4329	505179	11851	357547	17783	4043	11897	102118	5374	15494
0	0	0	0	0	0	0	0	0	0	0	0	0	0	0	0	0	0
0	0	0	0	124	0	0	0	146	0	0	0	0	0	0	0	0	0
0	0	0	0	0	0	0	0	0	0	0	0	0	0	0	0	0	0
0	0	0	0	0	0	0	0	0	0	0	0	0	0	0	0	0	0
0	0	0	0	0	0	0	0	0	0	0	0	0	0	0	0	0	0
0	125	0	60	0	0	0	0	0	0	0	0	0	0	0	0	0	0
118	0	0	0	0	0	0	0	0	0	0	0	0	0	0	0	0	0
0	0	0	0	0	0	0	0	0	0	0	0	0	0	0	0	0	0
0	214	421	0	0	0	58	0	0	0	173	0	0	1109	0	0	0	0
0	12169	43932	217	300	217	163371	659	1324	11012	799	385	1405	115874	548	923	432	1263
0	0	0	0	0	0	0	0	0	0	0	0	0	0	0	0	0	0
0	0	0	0	0	0	0	0	0	0	421	0	0	0	0	0	0	0
0	0	0	0	0	0	0	0	0	0	0	0	0	0	0	0	0	0
0	0	0	0	0	0	0	0	0	0	0	0	0	0	0	0	0	0
0	0	0	0	0	0	0	0	0	0	0	0	0	0	0	0	0	0
0	0	0	0	0	0	0	0	0	0	0	0	0	0	0	0	0	0
14539	14185	124	100	87	1314	0	0	0	14383	248	1486	0	0	37	337	77	0
0	0	0	0	0	0	0	0	0	0	0	0	0	0	0	0	0	0
0	0	0	0	0	0	0	0	0	0	0	0	0	0	0	0	0	0
0	0	0	0	0	0	0	0	0	0	0	0	0	0	0	0	0	0
0	0	0	0	0	0	0	0	0	0	0	0	0	0	0	0	0	0
0	0	0	0	0	0	0	0	0	0	0	0	0	0	0	0	0	0
0	40	0	148	122	83	68	127	134	42	240	0	0	161	0	0	5936	0
0	0	0	0	0	0	0	0	0	0	0	0	0	0	0	0	1545	0
0	0	0	0	0	0	0	0	0	0	0	0	0	0	0	0	0	0
0	0	0	0	0	0	0	0	0	0	0	0	0	0	0	0	1234	0
0	0	0	0	0	0	0	0	0	0	0	0	0	0	0	0	0	0
1093	740	0	955	283	0	0	0	0	0	0	0	0	0	0	0	117	0
0	0	0	0	0	0	0	0	0	0	0	0	0	0	0	0	0	0
0	0	136	0	0	0	0	0	0	0	0	0	0	771	0	0	193	0
0	0	0	0	0	0	0	0	0	0	0	0	0	0	0	0	0	0
0	66	0	41	0	0	0	0	0	0	0	0	0	0	0	0	0	0
0	0	0	0	0	0	0	0	0	0	0	0	0	0	0	0	0	0
63	0	0	146	0	161	370	267	668	119	328	5420	1331	188004	0	0	0	0
0	31	0	0	0	0	0	0	0	2247	0	0	0	0	0	0	0	0
0	0	0	0	0	0	0	0	0	0	0	0	0	0	0	0	0	0
0	0	0	0	0	0	0	0	0	0	0	0	0	0	0	0	0	0
0	0	0	0	0	0	0	0	0	0	82	0	0	63	0	0	0	0
0	0	0	0	0	0	0	0	0	0	0	0	0	0	0	0	0	0
0	0	0	0	0	0	0	0	0	0	0	0	0	0	0	0	0	0
0	0	0	0	0	0	0	0	0	0	0	0	0	0	0	0	0	0
0	0	0	0	0	0	0	0	0	0	0	0	0	0	0	0	1593	0
0	0	0	0	0	0	0	0	0	0	0	0	0	0	0	0	0	0
0	0	0	0	0	0	0	0	0	0	0	0	0	0	0	0	0	0
0	0	0	0	0	0	0	0	0	0	0	0	0	0	0	0	0	0
0	0	0	0	0	0	0	0	0	0	0	0	0	0	0	0	0	0
0	0	0	0	0	0	0	0	0	0	0	0	0	0	0	0	0	0

0	0	0	0	0	0	0	0	0	0	0	0	0	0	0	0	0	0
2706	0	7215	7372	0	27105	0	0	135	3659	151	3965	440	86	74	16672	722	303
0	0	0	0	0	0	0	0	0	0	0	0	0	0	0	0	0	0
0	0	0	0	0	0	0	0	0	0	0	1681	455	0	0	0	0	0
2710	1851	90495	22867	1653	11839	1305	5620	6122	1318	852536	9243	154504	7241	810	4310	13752	7530
0	0	0	0	337	0	0	0	0	0	0	0	0	0	0	0	0	0
0	0	0	0	38	0	0	0	0	0	0	0	0	0	0	0	0	0
0	0	0	0	0	0	0	0	0	0	0	0	0	0	33	0	0	0
54	0	0	13081	7697	0	0	0	0	0	0	0	0	0	0	0	0	0
1476	0	0	0	0	42	0	0	0	0	76	0	0	0	0	0	0	0
0	0	0	0	0	0	0	0	0	0	0	0	0	0	0	0	0	0
0	0	0	0	30	0	0	0	0	0	0	0	0	0	0	0	0	0
0	0	0	0	0	0	0	0	0	0	0	0	0	0	0	0	0	0
0	0	0	0	0	0	0	0	0	0	1210	0	0	0	41	0	0	0
0	0	0	0	0	0	0	0	0	0	0	0	457	31266	0	0	0	0
0	0	0	0	0	0	0	0	0	0	0	0	0	0	0	0	0	0
0	0	0	0	0	0	64	0	0	0	0	57	440	0	0	0	0	0
0	0	0	0	0	0	0	0	0	0	0	0	0	0	0	0	0	0
0	0	0	0	0	0	0	0	0	0	0	0	0	0	0	0	0	0
1908	0	0	0	0	199	0	0	0	0	0	0	0	0	0	119	0	0
0	0	0	0	41	0	0	0	0	0	0	0	0	0	0	0	0	0
0	0	0	0	0	0	0	0	0	0	0	0	0	0	0	0	0	0
0	0	0	0	0	0	0	0	0	0	0	0	0	0	0	0	0	0
0	0	549	0	0	326	340	0	0	0	0	0	0	0	0	0	0	0
0	0	0	0	0	0	0	0	0	0	0	0	0	0	0	0	0	0
0	0	0	0	0	0	0	0	0	0	0	0	0	0	0	0	0	0
62	166	0	46	0	285	0	0	0	475	0	0	0	0	0	0	0	0
0	0	0	57	0	0	0	0	0	0	0	0	0	66	0	0	0	0
0	0	0	0	0	0	0	0	0	0	0	0	0	0	0	0	0	0
0	0	0	0	0	0	0	0	0	0	0	0	0	0	0	0	0	0
0	0	0	0	0	0	0	0	0	57	0	0	0	0	0	0	0	0
0	0	0	56	0	0	0	0	0	0	0	0	0	0	0	0	0	0
59	0	0	0	0	0	0	0	0	0	0	0	0	0	0	0	0	0
0	0	0	0	0	0	0	0	0	0	0	0	0	0	0	0	0	0
0	0	0	0	0	0	0	0	0	0	0	0	0	0	0	0	0	0
566	303	88303	14248	851	1225	10669	2143	1391	1006	1778	402	6448	5415	600	2026	14399	57758
0	0	0	0	0	0	0	0	0	0	0	8411	0	0	0	0	0	0
0	0	0	0	0	0	0	0	0	0	0	0	0	0	0	0	0	0
0	0	0	0	0	0	0	0	2078	0	87	0	0	0	0	0	0	0
0	43	40	0	0	52	287	121	162	118	230	0	0	0	514	0	0	99
0	0	0	0	62	0	0	0	0	0	0	0	0	0	0	0	0	0
0	0	0	0	0	0	0	0	0	0	0	0	0	0	0	0	0	0
0	0	0	0	0	0	0	0	0	90	90	0	0	32011	66	0	0	0
0	0	0	0	0	0	0	0	0	0	0	0	0	0	0	0	0	0
0	0	0	0	0	0	0	0	0	0	0	0	0	0	0	0	0	0
0	0	0	45	0	0	0	0	0	0	0	0	0	0	0	0	0	0
0	0	0	0	0	0	0	0	0	0	0	0	0	0	0	0	0	0
0	0	0	0	0	0	0	0	0	0	0	0	0	0	0	0	0	0
0	0	0	0	0	0	0	0	0	0	0	0	0	0	0	0	0	0
0	0	0	0	0	0	0	0	0	0	0	0	0	0	0	0	0	0
421	2112	0	0	0	38	0	0	0	33	0	0	0	0	0	0	0	0
0	0	0	0	0	0	0	0	0	0	0	0	0	0	0	0	0	0
0	0	0	0	0	0	0	0	0	0	0	0	0	0	0	0	0	0
0	0	0	0	284	36	0	0	0	0	0	0	0	0	0	0	37	0
0	0	0	0	0	0	0	0	0	0	0	0	0	0	0	0	0	0
0	0	0	0	0	0	0	0	0	0	0	0	0	0	0	0	0	0
0	0	0	0	0	0	0	0	0	0	0	0	0	0	0	0	0	0
0	0	0	114	212	0	0	0	0	0	80	0	0	0	0	0	0	0
0	0	0	0	0	0	0	0	0	0	0	0	0	0	0	0	0	0
0	0	0	0	0	0	0	0	0	0	0	0	0	0	0	0	0	0
0	0	0	0	0	0	0	0	0	0	0	0	0	0	0	0	0	0
0	119	35	64924	0	32	0	543	0	45	84	43	450	158	0	110185	0	416
0	0	0	0	0	0	0	0	0	0	0	0	0	0	0	0	0	0
0	0	0	0	0	0	0	0	0	0	0	0	0	0	0	0	0	0
333	0	0	52	83	0	0	0	293	52	3686	0	0	0	0	0	0	205
0	143	0	427	48	0	0	0	0	0	10304	0	0	0	0	0	0	0
0	0	164	0	41	0	0	0	0	0	0	0	0	0	113	347	71	545
0	0	0	42	0	0	0	0	0	0	665	0	0	0	0	0	0	0
0	44	0	57	0	0	0	0	0	0	0	0	0	0	0	0	0	0
0	0	0	0	6905	0	0	0	0	0	0	0	0	0	0	0	0	0
0	0	0	0	0	0	0	0	0	0	0	0	0	0	0	0	0	0
0	0	0	0	0	0	0	0	0	0	0	0	0	0	0	0	0	0
0	0	0	0	0	0	0	0	0	0	0	0	0	0	0	0	0	0
0	0	0	0	0	0	0	0	0	0	0	0	0	0	0	0	0	0
0	445	0	0	0	0	62	0	132	0	0	0	0	0	290	0	0	0
52	0	0	0	0	0	0	0	0	0	0	0	0	0	0	0	0	0
0	0	0	0	0	0	0	0	0	0	0	0	0	0	0	0	0	0
0	0	0	0	0	0	0	0	0	0	0	0	0	0	0	0	0	0
0	0	0	0	0	0	0	0	0	0	0	0	0	0	0	0	0	0
0	0	0	0	0	0	0	0	0	0	0	0	0	0	0	0	0	0
0	0	0	0	0	38	50	0	0	107	0	0	0	0	0	0	0	0
0	0	0	0	0	0	0	0	0	0	0	0	0	0	0	0	0	0
0	0	0	0	42	0	0	0	0	0	0	0	0	0	0	0	0	0
0	0	0	0	0	0	0	0	0	0	0	0	0	0	0	0	0	0
0	0	0	0	0	0	0	0	0	0	0	0	0	0	0	0	0	0
2050	0	170	0	107	0	315	423	129	1330	179	94	460	23385	0	225	78	444
0	0	0	0	0	0	0	0	0	0	0	0	0	0	0	0	0	0

0	0	0	0	41	0	0	0	0	0	4091	0	0	0	0	0	0	0
0	0	0	0	0	0	0	0	0	0	0	0	0	0	0	0	0	0
2350	652	0	0	0	0	74	120	0	0	0	10556	0	0	0	0	0	0
0	0	0	0	0	34	0	0	0	0	0	0	0	0	0	0	0	0
618	352	127	42865	5766	157	312	402	546	33	1417	859	461	116541	419	102	7254	316
0	118	42	0	48	0	0	0	0	0	0	0	0	145	0	0	0	0
8106	194502	32	823	1171	279	149	146	116	51	926	48	0	73	122	46839	30	0
13181	882	0	181684	92721	226	66	670	3464	174	1812	12344	1401	129627	1153	351	28027	417
61	37	0	4448	784	0	0	0	0	0	75	86	0	4737	0	0	235	0
0	0	0	0	33	0	0	0	0	0	0	0	0	0	32	0	35	209
0	0	0	0	0	32	66	274	0	0	4874	0	0	147	0	0	0	0
0	0	0	0	0	0	0	0	0	0	0	0	0	0	0	0	0	0
0	0	0	0	0	0	0	0	0	0	0	0	0	0	0	0	0	0
0	0	0	0	0	0	0	0	0	0	0	0	0	0	0	0	0	0
0	0	0	0	0	0	0	0	0	0	0	0	0	0	0	0	0	0
0	0	0	0	0	0	0	0	0	0	0	0	0	0	0	0	0	0
0	0	0	0	0	0	0	0	0	0	0	0	0	0	0	43148	34	91
0	0	0	0	0	0	0	0	0	0	0	0	0	0	0	0	0	0
0	0	0	0	0	0	0	0	0	0	0	0	0	0	0	0	0	0
0	0	0	0	0	0	0	0	0	0	0	0	0	0	0	0	0	0
0	0	0	0	0	0	0	0	0	0	0	0	0	0	0	0	0	0
51	0	0	48335	0	0	159	124	0	0	0	19348	0	138	0	0	0	0
0	81	0	42	0	0	0	0	0	0	0	0	0	0	0	0	0	0
0	0	0	0	0	0	0	0	0	0	0	0	0	0	0	0	0	0
0	0	0	0	0	0	0	0	0	0	0	0	0	0	0	0	0	0
0	0	0	0	0	0	0	0	0	0	0	0	0	0	0	0	0	0
0	0	0	0	0	0	0	0	0	0	0	0	0	0	0	0	0	0
0	0	0	0	0	0	0	0	0	0	0	0	0	0	0	0	0	0
56	36	0	0	0	0	0	0	0	55	0	0	0	0	0	0	0	0
0	0	0	0	0	0	0	0	0	0	0	0	0	0	0	0	0	0
0	0	0	0	0	0	0	0	0	0	0	0	0	0	0	0	0	0
0	0	0	0	0	0	0	0	0	0	0	0	0	0	0	0	0	0
4625	15391	26733	48182	55022	1618	372230	265592	3096	20953	2854	396257	6837	50761	528	51239	7265	360728
0	0	0	0	0	0	0	0	0	0	0	0	0	0	0	0	0	0
0	0	0	0	0	0	0	0	0	0	0	0	0	0	0	0	0	0
0	0	0	0	0	0	0	0	0	0	0	0	0	0	107	0	0	0
0	0	0	0	0	0	0	0	0	0	0	0	0	0	0	0	0	0
0	0	0	0	0	0	0	0	0	0	0	0	0	0	0	0	0	0
0	0	0	0	0	0	0	0	0	0	0	0	0	0	0	0	0	0
0	0	0	0	0	0	0	0	0	0	0	0	0	0	0	0	0	0
0	0	0	0	38	0	0	0	0	0	93	0	0	0	228	0	0	0
0	0	0	0	0	0	0	0	0	0	0	0	0	0	0	0	0	0
0	0	0	0	0	0	0	0	0	0	0	0	0	0	0	0	0	0
0	0	0	0	0	0	0	0	0	0	0	0	0	0	0	0	0	0
0	0	0	0	0	0	0	0	0	0	0	0	0	0	0	0	0	0
0	0	0	0	0	0	0	0	0	0	0	0	0	0	0	0	0	0
0	0	0	0	0	0	0	0	0	0	273	0	0	0	0	0	0	0
3666	90	0	186	0	0	0	0	0	0	0	0	0	0	0	0	0	0
0	0	0	0	0	0	0	0	0	0	0	0	0	0	0	0	0	0
0	0	0	0	0	0	0	0	0	0	0	0	0	0	0	0	0	0
0	0	0	0	0	0	0	0	0	0	0	0	0	0	0	0	0	0
0	0	0	0	0	0	0	0	0	0	0	0	0	0	0	0	0	0
0	33	0	0	0	0	0	0	0	0	0	0	0	0	0	0	0	0
0	35	85	97	121	0	0	0	0	0	486	0	0	0	0	0	40	114
0	0	0	0	41	0	0	0	0	0	0	0	0	0	0	0	0	0
0	0	0	0	0	0	0	0	0	0	0	0	0	0	0	0	0	0
0	0	0	0	0	0	0	0	0	0	0	0	0	0	0	0	0	0
0	0	0	0	46	0	0	0	0	0	0	0	0	0	0	0	0	0
0	0	87787	0	0	0	0	0	0	0	0	0	0	0	113	347	106	357
0	39	0	0	0	30	73	110	0	0	0	0	0	0	0	0	0	0
0	0	0	46	0	0	0	0	0	0	0	0	0	0	1525	0	0	0
0	0	0	0	0	71	0	0	0	0	0	0	0	0	0	0	0	0
0	107	0	146468	0	130	435	283	769	264	244	200	2246	621	0	0	0	0
0	0	0	0	0	0	0	0	0	0	0	0	0	0	0	0	0	0
0	0	0	0	0	0	0	0	0	0	0	0	0	0	171	0	0	0
0	0	0	0	0	0	0	0	0	0	0	0	0	0	0	0	0	0
0	0	0	0	0	0	0	0	0	0	0	0	0	0	0	0	0	0
0	0	0	0	0	0	0	0	0	0	0	0	0	0	0	0	0	0
0	0	0	0	0	0	0	0	0	0	0	0	0	0	0	0	0	0
0	0	0	0	0	0	0	0	0	0	0	0	0	0	0	0	0	0
0	0	0	0	0	0	0	0	0	0	0	0	0	0	0	0	0	0
0	154	0	67	0	0	0	0	0	0	0	0	0	0	0	0	0	0
0	0	0	0	497	0	0	0	0	0	23193	0	0	0	0	0	0	0
0	0	0	0	0	0	0	0	0	0	87	0	0	0	0	0	0	0
0	0	0	0	0	0	0	0	0	0	0	0	0	0	0	0	0	0
1963	1941	29024	15530	1190	33898	69	563	0	195	3691	18487	0	0	950	1936	1749	1521
0	0	0	0	0	0	0	0	0	0	0	0	0	0	0	0	0	0
0	0	0	0	0	45	76	136	0	89	0	0	0	58	0	0	0	0
0	0	0	0	0	0	0	0	0	0	0	0	0	0	0	0	0	0
0	0	0	0	0	0	0	0	0	0	0	0	0	0	0	0	0	0
0	0	0	0	0	0	0	0	0	0	0	0	0	0	0	0	0	0
0	0	0	0	0	0	0	0	0	0	0	0	0	0	0	0	0	0
0	0	0	0	0	0	0	0	0	0	0	0	0	0	0	0	0	0
0	0	0	0	0	0	0	0	0	0	0	0	0	0	0	0	0	0
0	0	0	0	0	0	0	0	0	0	0	0	0	0	0	0	0	0
0	0	0	0	0	0	0	0	0	0	0	0	0	0	0	0	0	0
0	37	0	57	0	0	0	0	0	0	0	0	0	0	0	0	0	0

0	0	0	0	0	0	0	0	0	0	0	0	0	0	0	0	0	0
0	24	0	0	38	0	0	0	0	0	0	187	0	0	0	0	0	0
0	0	0	0	0	0	0	0	0	0	0	0	0	0	40	0	0	0
0	0	0	0	0	0	0	0	0	0	0	0	0	0	0	0	0	0
0	0	0	0	0	0	0	0	0	0	0	0	0	0	0	0	0	0
0	0	0	0	0	0	0	0	0	0	0	0	0	0	0	0	0	0
0	0	0	0	0	0	0	0	0	0	0	0	0	0	0	0	0	0
0	0	0	0	0	0	0	0	0	0	0	0	0	0	0	0	0	0
0	0	0	0	0	0	0	0	0	0	0	0	0	0	0	0	0	0
0	0	0	0	0	0	0	0	0	0	0	0	0	0	0	0	0	0
0	0	0	0	0	0	0	0	0	0	0	0	0	0	0	0	0	0
0	0	0	0	0	0	0	0	0	0	0	0	0	0	0	0	0	0
0	0	0	0	0	0	0	0	0	0	0	0	0	0	0	0	0	0
0	42	0	0	0	0	0	0	0	0	0	0	0	0	0	0	0	0
0	0	0	0	0	0	0	0	0	0	0	0	0	0	0	0	0	0
0	0	0	0	0	0	0	0	0	0	0	0	0	0	192	0	0	0
0	0	0	7898	0	0	0	0	0	0	0	0	0	0	0	0	0	0
0	0	0	0	0	0	0	0	0	0	0	0	0	0	0	0	0	0
0	0	0	0	0	0	0	0	0	0	0	0	0	0	0	0	0	0
0	0	0	0	0	0	0	0	0	0	0	0	0	0	0	0	0	0
0	0	0	0	0	0	0	0	0	0	0	0	0	0	0	0	0	0
0	0	0	0	0	0	0	0	0	0	0	0	0	0	0	0	0	0
5284	6037	42	211	129	481	152	0	0	13527	74	720	906	97	72	109	82	0
0	0	0	0	0	0	0	0	0	0	0	0	0	0	0	0	0	0
0	0	0	102	0	0	0	134	0	35	662	0	463	0	0	0	0	0
0	70	0	220	0	0	0	132	0	44	0	51	0	0	0	0	0	0
0	0	0	0	0	0	0	0	0	0	0	0	0	0	0	0	0	0
0	0	0	0	0	0	0	0	0	0	0	0	0	0	0	0	0	0
0	31	0	54	0	0	0	0	0	2508	0	0	0	0	0	0	0	0
0	0	0	0	0	0	0	0	0	0	0	0	0	0	0	0	0	0
0	0	0	0	67	0	0	0	119	0	0	0	0	0	0	0	0	0
0	0	0	0	0	0	0	0	0	0	0	0	0	0	0	0	0	0
0	0	0	0	0	0	0	0	0	0	0	0	0	0	0	0	0	0
53745	11321	33920	28832	27969	8732	27263	30056	276	24659	21086	20965	4905	1275	1155	398554	26109	216887
0	32	0	0	0	0	0	0	0	0	0	0	0	0	0	0	0	0
0	245	0	0	0	0	0	0	0	0	0	0	0	0	0	0	0	0
0	4203	0	56	0	39	0	0	0	0	0	0	438	0	116	120	0	0
0	0	0	0	0	0	0	0	0	0	0	0	0	0	0	0	0	0
0	0	0	0	0	0	0	0	0	0	0	0	0	0	0	0	0	0
0	0	0	0	0	0	0	0	0	0	0	0	0	0	0	0	0	0
0	0	0	0	0	0	0	0	0	0	0	0	0	0	0	0	0	0
0	0	123	0	0	0	0	0	0	0	0	0	0	0	0	0	251	0
0	0	0	0	0	0	0	0	0	0	0	0	0	0	0	0	0	0
0	0	0	0	0	0	0	0	0	0	0	0	0	0	0	0	0	0
132	0	0	49	741	0	219	0	126	128	388	0	0	0	62	0	0	290
0	0	0	0	0	0	0	146	0	0	0	0	0	0	0	0	0	0
0	0	0	178	35	428	0	0	0	0	0	49	0	0	0	0	39	0
0	0	0	0	0	0	0	0	0	0	0	0	0	0	0	0	0	0
0	0	0	0	0	0	0	0	0	0	0	0	0	0	0	0	0	0
0	0	0	0	0	0	0	0	0	0	0	0	0	0	0	0	0	0
0	0	0	0	0	0	0	0	0	0	0	0	0	0	0	0	0	0
0	0	0	0	0	0	0	0	0	0	0	0	0	0	0	0	0	0
0	163	0	0	0	0	0	0	0	0	0	50	0	0	0	0	0	0
0	0	0	0	0	0	0	0	0	0	0	0	0	0	0	0	0	0
0	0	0	0	0	0	0	0	0	0	0	0	0	0	0	0	0	0
0	78	0	36035	0	0	0	0	0	0	0	0	0	0	0	0	0	0
0	0	0	0	0	0	0	0	0	0	0	0	0	0	0	0	0	0
0	0	0	0	0	0	0	0	0	0	0	0	0	0	0	0	0	0
0	0	45	0	33	0	86	230830	416	86	173	49	458	0	0	0	38	218
0	0	0	0	0	0	0	0	0	0	0	0	0	0	0	0	0	0
0	0	0	0	0	0	0	0	0	0	0	0	0	0	0	0	0	0
0	0	0	0	0	0	0	0	0	0	171	0	0	0	0	0	0	0
0	0	0	0	0	0	0	0	0	0	0	0	0	0	0	0	0	0
0	0	0	0	0	0	0	0	0	0	0	0	0	0	0	0	0	0
1133	0	11960	0	0	0	0	0	0	0	0	67	459	0	33	115	0	0
0	0	0	0	0	0	0	0	0	0	0	0	0	0	0	0	0	0
0	0	0	0	0	0	0	0	0	0	0	0	0	0	0	0	0	0
0	0	0	0	75	0	0	0	0	0	0	0	0	0	0	0	0	0
0	0	0	0	0	0	0	0	0	0	0	0	0	0	0	0	0	0
0	0	0	0	0	0	0	0	0	0	0	1289	0	0	0	0	0	0
0	0	0	0	0	0	0	0	0	0	0	0	0	0	0	0	0	0
0	0	0	0	0	0	0	0	0	0	0	0	0	0	0	0	0	0
77	50	0	0	0	0	0	0	0	0	0	269	0	0	0	0	0	0
0	0	0	0	47	0	0	0	0	0	0	0	0	0	0	0	0	0
0	0	0	0	0	0	0	0	0	0	0	0	0	0	0	0	0	0
0	0	0	0	0	0	0	0	0	0	0	0	0	0	0	0	0	0
0	0	0	0	0	0	0	0	0	0	0	0	0	0	0	0	0	0
0	0	0	0	0	0	0	0	0	0	0	0	0	0	0	0	0	0
0	0	0	0	0	0	0	0	0	0	231	0	0	0	0	0	0	0
0	0	0	0	0	0	0	0	0	0	0	0	0	0	0	0	0	0
0	0	0	0	0	0	0	0	0	0	0	49	0	0	0	0	0	0
0	0	0	0	0	0	0	0	0	0	0	0	0	0	0	0	0	0
0	0	0	0	0	0	0	0	0	0	0	0	0	0	0	0	0	0
0	0	0	0	0	0	0	0	0	0	0	0	0	0	0	0	0	0
48	39	124	0	0	0	0	0	0	0	0	0	0	0	0	126	2809	118
0	0	0	0	0	0	0	0	0	0	0	0	0	0	0	0	0	0
0	0	0	0	0	0	0	0	0	0	0	0	0	0	0	0	0	0

63	0	0	0	0	0	0	0	0	0	0	0	0	0	35	0	0	0
0	0	0	0	0	0	0	0	0	0	0	0	0	0	0	0	0	0
0	0	0	0	0	0	0	0	0	0	0	0	0	0	0	0	0	0
0	0	0	0	0	0	0	0	0	0	0	0	0	0	0	0	0	0
0	0	0	0	0	0	0	0	0	0	0	0	0	0	0	0	0	0
0	0	0	0	0	0	0	0	0	0	0	0	0	0	0	0	0	0
0	0	0	0	0	0	0	0	0	0	0	0	0	0	0	0	0	0
0	0	0	0	0	0	0	0	0	0	0	0	0	0	0	0	0	0
0	0	0	0	0	0	0	0	0	0	0	0	0	0	0	0	0	0
0	0	0	0	0	0	0	0	0	0	0	0	0	0	0	0	0	0
0	0	0	0	0	0	0	0	0	0	0	0	0	0	0	0	0	0
1250	987	0	0	0	0	0	0	0	0	0	0	0	0	903	0	0	0
0	0	0	0	0	0	0	0	0	0	0	0	0	0	0	0	0	0
0	0	0	0	0	0	0	0	0	0	0	0	0	0	0	0	0	0
0	0	0	0	0	0	0	0	0	0	0	0	0	0	0	0	0	0
0	0	0	0	0	0	0	0	0	0	0	0	0	0	0	0	0	0
0	0	0	0	0	0	0	0	0	0	0	0	0	0	0	0	0	0
0	0	0	0	0	0	0	0	0	0	0	0	0	0	0	0	0	0
0	0	0	0	0	0	0	0	0	0	0	3753	0	62	0	0	0	0
0	82	0	53	0	0	0	0	0	0	0	0	0	0	0	0	0	0
0	0	0	0	0	0	0	0	0	0	0	0	0	0	0	0	0	0
0	0	0	0	85	0	0	0	0	0	0	0	0	0	0	0	0	0
0	0	0	0	0	0	0	0	0	0	0	0	0	0	0	0	0	0
0	0	0	0	0	0	0	0	0	0	0	0	0	0	0	0	0	0
0	0	0	583	0	0	0	0	0	0	0	0	0	0	0	0	0	0
0	0	0	0	0	0	0	0	0	0	0	0	0	0	0	0	0	0
0	0	0	0	0	0	0	0	0	0	0	0	0	0	0	0	0	0
0	0	0	0	0	0	0	0	0	0	0	0	0	0	0	0	0	0
0	0	0	0	0	0	0	0	0	0	0	0	0	0	0	0	0	0
0	78	0	111	0	0	0	129	0	0	0	0	0	0	0	0	0	0
0	0	0	0	0	0	0	0	0	0	0	0	0	0	0	0	0	0
0	0	0	52	0	0	0	0	0	0	0	0	0	0	0	0	0	0
0	69	0	111	0	0	0	0	0	0	0	0	0	0	0	0	0	0
0	0	0	0	0	0	0	0	0	0	0	0	0	0	0	0	0	0
0	0	0	0	0	0	0	0	0	0	0	0	0	0	0	0	0	0
0	0	0	0	0	0	0	0	0	0	72	0	0	70	0	0	0	0
0	0	0	0	0	0	0	0	0	0	0	0	0	0	0	0	0	103
0	0	0	0	0	0	0	0	0	0	0	0	0	0	0	0	0	0
0	0	0	0	0	0	0	0	0	0	0	0	0	0	0	0	0	0
0	0	0	0	0	0	0	0	0	0	0	0	0	0	0	0	0	0
0	0	0	0	0	0	0	0	0	0	0	0	0	0	0	0	0	0
0	0	0	0	44	0	0	0	0	0	0	0	0	0	0	0	0	0
0	0	0	0	0	0	0	0	0	0	0	0	0	0	0	0	0	0
0	0	0	0	0	0	0	0	0	0	0	50	449	0	0	0	0	0
50	0	0	0	0	0	0	0	0	0	0	0	0	0	0	0	0	0
1502	0	44297	15879	0	21	25752	124	0	0	0	2897	0	0	176	125	101	301
0	0	0	0	0	0	0	0	0	0	0	0	0	0	0	0	0	0
0	0	0	0	0	0	0	0	0	0	0	0	0	0	0	0	0	0
0	0	0	0	0	0	0	0	0	0	0	0	0	0	0	0	0	0
539	607	11974	0	8474	0	23377	136	0	13277	148	1053	0	11206	80	0	895	4772
0	0	0	0	0	0	0	0	0	0	0	0	0	0	0	0	0	0
10975	2622	22705	41595	68521	101504	260691	1587	6528	2553	1169	147	3232	180235	509	126	236	735
0	94	0	10883	0	43	360	0	151	0	0	75	0	134	0	0	0	0
0	0	0	0	0	0	0	0	0	0	0	0	0	0	0	0	0	0
0	0	0	0	0	0	0	0	0	0	0	0	0	0	0	0	0	0
0	547	0	0	0	0	0	0	0	0	0	0	0	0	0	0	0	0
0	0	0	0	0	0	0	0	0	0	0	0	0	0	0	0	0	0
3207	0	0	0	0	0	0	0	0	0	75	0	0	0	0	0	0	0
0	0	0	0	0	0	0	0	0	0	0	0	0	0	0	0	0	0
0	0	0	0	0	0	0	0	0	0	0	0	0	0	0	0	0	0
68	4051	0	922	0	269	194	574	131	223436	830	184	460	599	10505	113	0	90
0	0	0	0	0	0	0	0	0	0	0	0	0	0	0	0	0	0
0	0	0	0	0	0	0	0	0	0	0	0	0	0	0	0	0	0
0	0	0	0	0	0	0	0	0	0	0	0	0	0	0	0	0	0
0	0	0	0	0	0	0	0	0	0	0	0	0	0	0	0	0	0
0	0	0	0	0	0	0	0	0	0	0	0	0	0	0	0	0	0
0	0	0	0	117	0	0	0	129	44	159	0	0	0	0	0	0	0
0	0	0	0	0	0	0	0	0	0	0	0	0	0	0	0	0	0
0	0	0	0	0	0	0	0	0	0	0	0	0	0	0	0	0	0
0	0	0	0	0	0	0	0	0	0	0	0	0	0	0	0	0	0
1621	0	0	16444	0	0	0	0	0	0	0	0	0	0	0	0	0	0
0	531	0	75799	0	0	0	0	0	0	510	0	0	0	0	0	34	0
0	0	0	39	0	0	0	0	0	0	340	0	0	0	0	0	0	0
0	41	72	109	0	41	0	0	0	0	408	0	0	0	37	0	0	124
0	0	0	0	0	0	0	0	0	0	0	0	0	0	0	0	0	118
0	0	0	0	0	0	0	0	0	0	0	0	0	0	0	0	0	0
0	0	0	0	0	0	0	0	0	0	0	0	0	0	0	0	0	0
0	0	0	0	0	0	0	0	0	0	0	0	0	0	0	0	0	0
0	0	0	0	0	0	0	0	0	0	0	0	0	0	0	0	0	0
0	0	0	0	0	0	0	0	0	0	0	0	0	0	0	0	0	0
0	0	0	0	0	0	0	0	0	0	0	0	0	0	0	0	0	0
0	0	0	0	0	0	0	0	0	0	0	0	0	0	0	0	0	0
0	0	0	0	0	0	0	0	0	0	0	0	0	0	0	0	0	0
0	0	0	0	0	0	0	0	0	0	0	0	0	0	0	0	0	0
0	0	0	0	0	0	0	0	0	0	0	0	0	0	0	0	0	0
0	0	0	0	0	0	0	0	0	0	0	0	0	0	0	0	0	0
0	0	0	0	42	0	0	0	0	0	0	0	0	0	0	0	0	0

0	0	0	0	0	0	0	0	401	0	0	0	0	0	0	0	0	0
0	77	0	265	0	0	0	0	0	0	0	0	0	0	0	0	0	0
0	4277	0	0	0	35	0	0	0	8176	0	0	0	0	0	0	0	0
0	0	0	0	0	0	0	0	0	0	0	0	0	0	0	0	0	0
0	0	0	0	0	0	0	0	0	0	0	0	0	0	0	0	0	0
0	0	0	0	0	126	69	140	127	0	89	32966	0	137	0	0	0	0
0	0	0	0	0	0	0	0	0	0	0	0	0	0	0	0	0	0
0	0	0	0	38	0	0	0	0	0	0	0	0	0	0	0	0	0
0	0	0	0	0	0	0	0	0	0	0	0	0	0	0	0	0	0
0	0	0	0	0	0	0	0	0	0	0	0	0	0	0	0	0	0
0	0	0	0	0	0	0	0	0	0	0	0	0	0	0	0	0	0
0	0	0	0	0	0	0	0	0	0	0	0	0	0	0	0	0	0
68	0	0	0	0	104	147	144	280	31	252	256	0	69	0	0	2723	0
68	41	0	0	0	43	0	0	0	41	0	0	0	0	0	0	0	0
0	0	0	0	0	0	0	0	0	0	0	0	0	0	0	0	0	0
0	0	0	0	0	0	0	0	0	0	0	0	0	0	0	0	0	0
0	0	0	0	0	0	0	0	0	0	0	0	0	0	0	0	0	0
0	0	32	0	0	0	0	0	0	0	0	0	0	0	0	0	0	0
0	0	0	0	0	0	0	0	0	0	0	0	0	0	0	0	39	115
0	0	0	0	0	0	0	0	0	0	0	0	0	0	0	0	0	0
0	0	0	0	0	0	0	0	0	0	0	0	0	0	0	0	0	0
7004	9737	206	125	296	5444	0	0	258	8450	0	1208	0	0	112	30469	306	225
0	0	0	0	0	0	0	0	0	0	0	0	0	0	206	0	0	0
0	0	0	0	0	0	0	0	0	0	0	0	0	0	0	0	0	0
0	0	0	0	0	0	0	0	0	0	0	0	0	0	0	0	0	0
0	0	0	0	0	0	0	0	0	0	0	0	0	0	0	0	0	0
0	0	0	0	0	0	0	0	0	0	0	0	0	0	0	0	0	0
0	0	0	0	0	0	0	0	0	0	0	0	0	0	0	0	0	0
2278	114	0	55	37	0	0	0	123	0	0	0	0	0	0	117	0	111
0	0	0	0	0	0	0	0	0	0	0	0	0	0	0	0	0	0
0	0	0	0	0	0	0	0	0	0	0	0	0	0	0	0	0	0
0	0	0	0	0	0	0	0	0	0	0	0	0	0	0	121	0	0
0	0	0	0	0	0	0	0	0	0	0	0	0	0	0	0	0	0
48	0	0	0	0	0	0	0	0	0	0	0	0	0	0	0	0	0
0	0	0	0	0	0	0	0	0	0	0	0	0	0	0	0	0	0
0	0	0	0	0	0	0	0	0	0	73	0	0	0	0	0	0	0
0	0	0	0	0	0	0	0	0	0	0	0	0	0	0	0	0	0
0	0	0	0	0	0	0	0	0	0	0	0	0	0	0	0	0	0
0	0	0	0	0	0	0	0	0	0	0	0	0	0	0	0	0	0
0	0	0	0	0	0	0	0	0	0	0	0	0	0	0	0	0	0
0	0	0	0	0	0	0	0	0	0	0	0	0	0	0	0	0	0
0	0	0	0	0	0	0	0	0	0	1177	0	0	0	0	0	0	0
0	0	0	56	0	0	0	0	0	0	0	0	0	0	0	0	0	0
0	0	0	0	0	0	0	0	0	0	0	0	0	0	0	0	0	0
0	0	2318	0	0	0	0	0	0	0	0	2013	464	2918	0	0	0	0
0	0	0	0	0	0	0	0	0	0	0	0	0	0	0	0	0	0
364	0	0	0	0	158	416	3232	960104	111	255	248	2233	617	0	0	0	0
0	0	0	0	0	0	0	0	0	255	0	0	0	0	0	0	0	0
0	0	0	0	0	0	0	0	0	0	0	0	0	0	0	0	0	0
0	0	0	0	0	0	0	0	0	0	0	0	0	0	0	0	0	0
0	0	0	0	0	0	0	0	0	0	0	0	0	0	0	0	0	0
0	0	0	0	0	0	0	0	0	0	0	0	0	0	0	0	0	0
0	0	0	0	0	0	0	0	0	0	0	0	0	0	0	0	0	0
0	0	0	0	0	0	0	0	0	0	0	0	0	0	0	0	0	0
0	0	0	0	0	0	0	0	0	0	0	0	0	0	0	0	0	0
0	0	0	0	0	0	0	0	0	0	0	0	0	0	27	0	0	0
0	0	0	0	0	0	0	0	0	0	0	0	0	0	0	0	90	0
0	0	0	0	0	0	0	0	0	0	0	0	0	0	0	0	0	0
0	0	0	0	0	0	0	0	0	0	0	0	0	0	0	0	0	0
0	0	0	0	0	0	0	0	0	0	0	0	0	0	89	0	0	0
0	0	0	0	0	0	0	0	0	0	0	0	0	0	0	0	0	0
117	33	0	0	0	678	0	0	0	80	0	0	0	0	0	0	0	0
0	0	0	0	0	0	0	0	0	0	0	0	0	0	0	0	0	0
0	0	0	0	0	0	0	272	0	0	0	0	437	0	0	0	0	0
0	0	0	0	0	0	0	0	0	0	0	0	0	0	0	0	0	0
0	0	0	0	0	0	0	0	0	0	0	0	0	0	0	0	0	0
0	0	0	0	0	0	0	0	0	0	0	0	0	0	0	0	0	0
0	0	0	0	0	0	0	0	0	0	0	0	0	0	0	0	5791	0
0	0	0	0	0	0	0	0	0	0	0	0	0	0	0	0	0	0
0	0	0	0	0	0	0	0	0	0	0	0	0	0	0	0	0	0
0	0	0	0	0	0	0	0	0	0	0	0	0	0	0	0	0	0
0	0	0	0	0	0	0	0	0	0	0	0	0	0	0	0	0	0
0	0	0	0	0	0	0	141	0	0	0	14256	459	0	0	0	0	0
0	0	0	0	50	0	0	0	0	0	0	0	0	0	0	0	0	0
4736	0	0	0	0	0	0	0	0	0	0	0	0	0	0	0	0	0
0	0	291	0	0	0	0	0	0	0	0	40	0	0	153	692	299	1287
0	0	0	0	0	0	0	0	0	0	0	0	0	0	0	0	0	0
0	0	0	0	0	0	0	0	0	0	0	0	0	0	0	0	0	0
0	0	0	0	0	0	0	0	0	0	0	0	0	0	0	0	0	0
0	0	0	0	0	0	0	0	0	0	0	0	0	0	0	0	0	0
0	0	0	0	0	0	0	0	0	0	0	0	0	0	0	0	0	0
0	0	0	0	0	0	0	0	0	0	0	0	0	0	0	0	0	0
0	0	0	0	0	0	0	0	0	0	0	0	0	0	41	100	67	0
0	0	0	0	0	0	0	0	0	0	0	0	0	0	0	0	0	99
0	0	0	0	0	0	0	0	0	0	0	0	0	0	0	0	0	0
0	0	0	0	0	0	0	0	0	0	0	0	0	0	0	0	37	0
0	0	0	0	0	0	0	0	0	0	0	0	0	0	0	0	0	0

0	0	0	0	0	0	0	0	0	0	0	0	0	0	0	0	0	0
0	0	0	0	34	0	0	0	0	0	179	0	0	0	0	0	0	0
0	0	0	0	0	0	0	0	0	0	0	0	0	0	0	0	0	0
0	0	0	0	0	0	0	0	0	0	0	0	0	0	0	0	0	0
0	0	0	0	0	0	0	0	0	0	0	0	0	0	0	0	0	0
0	0	51	57	0	0	0	0	0	0	0	0	0	0	46	0	146	104
0	0	0	0	0	0	0	0	0	0	0	0	0	0	160	0	0	0
0	0	0	0	0	0	0	0	0	0	0	0	0	0	0	0	0	0
0	0	0	0	148	39	0	275	0	36	86	42	0	0	0	0	0	0
0	47	0	0	252	278	282	119	0	38565	281	137	0	222	0	0	0	0
0	0	0	0	0	0	0	0	0	0	0	0	0	0	0	0	0	0
0	0	0	0	0	0	0	117	0	0	0	0	0	0	0	0	0	0
0	0	0	0	0	0	0	0	0	0	0	0	0	0	0	0	0	0
0	0	0	0	0	0	0	0	0	0	0	0	0	0	0	0	0	0
0	0	0	0	0	0	0	0	0	0	0	0	0	0	0	123	41	0
0	0	0	0	0	0	0	0	0	0	0	0	0	0	0	0	0	0
0	0	0	0	11601	0	0	0	0	0	0	0	0	0	0	0	0	0
0	30	0	107	0	0	0	0	0	0	0	6659	0	0	0	0	0	0
0	0	35	0	0	0	0	0	0	0	0	0	0	0	0	0	0	0
0	0	0	0	0	0	0	0	0	0	0	0	0	0	0	0	0	0
0	0	0	0	0	0	0	0	0	0	0	0	0	0	0	0	0	0
0	0	0	0	0	0	0	0	0	0	0	0	0	0	0	0	0	0
0	0	0	0	0	0	0	0	0	0	0	0	0	0	0	0	0	0
0	0	0	0	47	0	0	0	0	0	0	0	0	0	0	0	0	0
0	0	0	0	0	0	0	0	0	0	0	0	0	0	0	0	0	0
0	0	0	0	0	0	0	0	0	0	0	0	0	0	0	0	0	0
0	0	0	0	0	0	0	0	0	0	0	0	0	0	0	0	0	0
0	0	0	0	0	0	0	0	0	0	0	0	0	0	0	0	0	0
0	0	0	0	0	0	0	0	0	0	0	0	0	0	0	0	0	0
0	0	36	13641	0	77	104714	146	0	0	0	91	1371	139	0	110	102	320
0	0	0	0	0	0	0	0	0	0	0	0	0	0	0	0	0	0
0	0	0	0	44	0	0	0	0	0	0	0	0	0	0	0	0	0
0	0	0	0	0	40	79	116	1056	48	78	75	455	284	0	0	0	0
0	0	0	0	0	0	0	0	0	0	0	0	0	0	0	0	0	0
0	0	0	0	0	0	0	0	0	0	0	0	0	0	0	0	0	0
0	0	0	20655	0	0	0	0	0	0	0	0	0	0	0	0	0	0
0	0	0	0	0	0	0	0	0	0	0	0	0	0	0	0	0	0
0	0	209	0	0	0	0	0	0	0	0	0	0	0	45	0	87	0
2463	1144	0	220	8792	35	0	0	0	135	0	1961	0	0	2906	0	85	0
0	0	0	0	0	30	0	0	0	0	62	0	0	76	144	0	0	0
0	0	0	0	0	0	0	0	0	0	0	0	0	0	0	0	0	0
0	0	0	0	101	0	0	0	0	0	0	0	0	0	0	0	0	0
0	0	0	0	0	0	0	0	0	0	0	0	0	0	0	0	0	0
0	0	0	0	0	91	132	127	0	143	146	0	920	67	41	0	64	0
0	0	0	0	0	0	0	0	0	0	0	0	0	0	0	0	0	0
0	0	0	0	0	0	0	0	0	0	0	0	0	0	0	0	0	0
0	0	6409	0	0	0	0	0	0	0	0	0	0	0	0	0	37	0
0	0	0	0	0	0	0	0	0	0	0	0	0	0	0	0	0	0
0	0	0	0	0	0	0	0	0	0	0	0	0	0	0	0	0	0
0	0	0	0	0	0	0	0	0	0	0	0	0	0	0	0	0	0
0	0	0	0	0	0	0	0	0	0	88	0	0	0	0	0	0	0
0	0	0	0	0	0	0	0	0	0	0	0	0	0	0	0	0	0
0	0	0	0	0	0	0	0	0	0	507	0	0	0	0	0	0	0
0	0	0	0	0	0	0	0	0	0	0	0	0	0	0	118	9346	0
0	0	0	0	0	0	0	0	0	0	0	0	0	0	0	0	0	0
0	0	0	0	0	0	0	0	0	0	0	0	0	0	0	0	0	0
0	0	0	0	0	0	0	0	0	0	0	0	0	0	0	0	0	0
0	0	0	0	0	0	0	0	0	0	0	0	0	0	0	0	0	0
0	0	0	0	0	0	0	0	0	0	0	0	0	0	0	0	0	0
0	0	0	0	0	0	0	0	0	0	0	0	0	138	0	0	0	0
197	108	5557	2179	1617	31	0	263	0	47	0	49407	458	0	169	350	501	299
0	0	0	0	0	0	0	0	0	0	0	0	0	0	0	0	0	0
0	90	0	47	20018	94	0	0	0	0	12991	0	0	118	32	6470	6941	0
0	0	0	0	0	0	0	0	0	0	0	0	0	0	0	0	0	0
0	0	0	0	1155	0	0	0	0	0	0	0	0	0	0	0	0	0
379	0	0	0	0	0	0	0	0	0	0	0	0	0	0	0	0	0
1267	0	0	0	0	0	0	0	0	0	0	0	0	0	0	0	0	0
0	0	0	0	0	0	0	0	0	0	0	0	0	0	0	0	0	0
0	0	0	0	0	0	0	0	0	0	0	0	0	0	0	0	0	0
0	0	0	0	0	0	0	0	0	0	0	0	0	0	0	0	0	0
0	871	0	506	0	0	0	0	0	41	0	0	0	0	0	0	0	0
0	0	0	0	0	0	0	0	0	0	0	0	0	0	0	0	0	0
0	0	0	0	0	0	0	0	0	0	0	0	0	0	0	0	0	0
0	0	0	0	0	0	0	0	0	0	0	0	0	0	0	0	0	0
0	0	0	0	0	0	0	0	0	0	0	0	0	0	0	0	0	0
0	0	0	0	0	0	0	0	0	0	0	0	0	0	0	0	0	0
0	0	0	0	0	0	0	0	0	0	0	0	0	0	0	0	0	0
0	0	0	0	0	0	0	0	0	0	0	0	0	0	0	0	0	0
0	0	0	0	0	0	0	0	0	0	0	0	0	0	0	0	0	0
0	0	0	0	0	0	0	0	0	0	0	0	0	0	0	0	0	0
0	0	0	0	0	0	0	0	0	0	0	0	0	0	0	0	0	0
0	0	0	0	0	0	0	0	0	0	0	0	0	0	0	0	0	0
40914	18611	143	292	35	1664	292	251	0	47146	0	3642	0	132	73	711	105	553
0	0	0	0	0	0	0	0	0	0	0	0	0	0	0	0	0	0
0	0	0	0	0	0	0	0	0	0	0	0	0	0	0	0	0	0
0	0	0	0	0	0	0	0	0	0	0	0	0	0	0	0	0	0
0	0	0	0	0	0	0	0	0	0	0	0	0	0	0	0	0	0

0	0	0	0	0	0	0	0	0	0	0	0	0	0	0	0	0	0
0	0	0	0	0	0	0	0	0	0	0	0	0	0	0	0	0	0
60	0	0	0	0	0	0	0	0	0	0	0	0	0	0	0	0	0
0	0	0	0	0	0	0	0	0	0	0	0	0	0	0	0	0	0
0	0	0	58	0	0	0	0	0	0	0	0	0	0	0	0	0	0
0	0	0	109	0	0	0	0	0	0	0	0	0	0	0	0	0	0
0	0	0	0	0	0	0	0	0	0	0	0	0	0	0	0	0	0
0	0	0	0	0	0	0	0	0	0	0	0	0	0	0	0	0	0
0	0	0	0	0	0	0	0	0	0	0	0	0	0	0	0	0	0
0	0	0	0	0	0	0	0	0	0	0	0	0	0	0	0	0	0
0	0	0	0	0	0	0	0	0	0	0	0	0	0	0	0	0	0
48	0	0	0	0	0	0	0	0	0	0	0	0	0	0	0	0	0
0	0	0	0	0	0	0	0	0	0	0	0	0	0	0	0	0	0
0	0	0	0	0	0	0	0	0	0	0	0	0	0	0	0	0	0
0	0	0	0	0	0	0	0	0	0	0	0	0	0	0	0	0	0
0	2133	0	47	0	0	0	0	0	0	0	0	0	0	0	0	0	0
0	0	0	0	0	0	0	0	0	0	0	0	0	0	0	0	0	0
0	0	0	0	0	0	0	0	0	0	0	0	0	0	0	0	0	0
0	0	0	0	0	43	0	0	0	0	0	0	0	0	29	118	81	0
0	0	0	0	0	0	0	0	0	0	0	0	0	0	339	0	0	0
0	31	0	0	0	1795	0	0	0	324	0	0	0	0	0	0	0	0
0	0	0	0	0	0	0	0	0	0	0	0	0	0	0	0	0	0
0	0	0	0	0	0	0	0	0	0	0	0	0	0	0	0	0	0
0	0	0	0	0	0	0	0	0	0	0	0	0	0	0	0	0	0
0	0	44	0	0	0	0	0	0	0	0	0	0	0	36	0	247	574
0	0	0	0	0	0	0	0	0	0	0	0	0	0	0	0	0	0
0	0	0	0	0	0	0	0	0	0	0	0	0	0	0	0	0	0
0	0	0	0	0	29	0	0	259	0	295	0	469	66606	0	0	0	0
0	0	45	0	35	36	0	114	0	37	0	0	0	69	34	144278	37	650
61	0	0	0	0	0	0	0	0	0	0	0	0	0	0	0	0	0
0	0	0	0	0	0	0	0	0	0	0	0	0	0	0	0	0	0
75847	39	42	52	71	75	203	250	416	39	801	0	0	0	0	25949	44	112
0	0	0	0	0	0	0	0	0	0	0	0	0	0	0	0	0	0
0	0	0	0	0	0	0	0	0	0	0	0	0	0	0	0	0	0
0	0	0	0	0	0	0	0	0	0	0	0	0	0	0	0	0	0
0	0	0	0	0	0	0	0	0	0	0	0	0	0	0	0	0	0
0	0	0	0	40	0	0	0	0	0	0	0	0	0	0	100	43	0
0	0	0	0	0	0	0	0	0	0	0	0	0	0	0	0	0	0
0	0	0	0	0	0	0	0	0	0	0	0	0	0	0	0	0	0
0	0	0	0	0	0	0	0	0	0	0	0	0	0	0	0	0	0
0	0	0	0	0	0	0	0	0	0	0	0	0	0	0	0	0	0
0	107	0	144	0	0	0	0	0	0	0	0	0	0	0	0	0	0
0	0	0	0	0	0	0	0	0	0	0	0	0	0	38	0	38	0
0	0	0	0	0	0	84	0	0	580	0	0	0	7769	0	0	2715	0
0	0	0	0	0	0	0	0	0	0	0	0	0	0	0	0	0	0
62	0	0	35507	0	0	0	0	0	0	0	0	0	0	0	0	0	0
0	0	0	32397	0	0	0	0	0	0	0	0	0	0	0	0	0	0
0	0	0	5958	0	0	0	0	0	0	0	0	0	0	0	0	0	0
0	0	0	0	0	0	0	0	0	0	0	0	0	0	0	0	0	0
0	0	50	0	117	0	0	0	0	0	0	0	0	0	0	0	0	0
0	0	0	0	0	0	0	0	0	0	0	0	0	0	0	0	0	0
0	0	0	0	0	0	0	0	0	0	0	0	0	0	0	0	0	0
0	0	0	0	0	0	0	0	0	0	0	0	0	0	0	0	0	0
0	0	0	0	0	0	0	0	0	0	0	0	0	0	0	0	0	0
0	43	0	150	0	0	0	0	0	0	0	0	0	0	0	0	0	0
0	112	0	56	0	0	0	0	0	0	0	53	0	0	0	0	0	0
0	0	0	0	0	0	0	0	0	0	0	0	0	0	0	0	0	0
0	0	0	0	0	0	0	0	0	0	0	0	0	0	0	0	0	0
0	0	0	0	0	0	0	0	0	0	0	0	0	0	0	0	0	0
0	0	0	0	0	0	0	0	0	0	0	0	0	0	0	0	0	0
0	0	0	0	0	0	0	0	0	0	0	0	0	0	0	0	0	0
0	0	0	0	0	0	0	0	0	0	0	0	0	0	0	0	0	0
0	0	0	0	0	0	0	0	0	0	0	0	0	0	0	0	0	0
0	0	0	0	0	0	0	0	150	2281	0	0	0	0	0	0	0	0
0	0	0	0	0	0	0	0	0	0	0	0	0	0	0	0	0	0
0	0	0	0	0	0	0	0	0	0	0	0	0	0	0	0	0	0
0	0	0	0	0	0	0	0	0	0	0	0	0	0	0	0	0	0
2795	0	0	0	0	0	0	0	0	0	0	0	0	0	0	0	0	0
0	0	0	0	0	282	73	347997	133	78	245	89	1849	272	0	0	0	0
0	0	0	0	0	0	0	0	0	0	0	0	0	0	0	0	0	0
0	0	0	0	0	0	0	0	0	0	267	0	0	0	0	0	0	0
0	0	0	0	0	0	0	0	0	0	0	0	0	0	0	0	0	0
748	0	565	257	0	0	0	0	0	0	0	0	0	0	958073	1598	499	1912
0	0	0	0	0	0	0	0	0	0	0	0	0	0	0	0	0	0
0	0	0	0	0	0	0	0	0	0	0	0	0	0	0	0	0	0
0	0	0	0	0	0	0	0	0	0	0	0	0	0	0	0	0	0
836	928	0	0	40	228	83	0	0	3869	0	90	0	0	0	130	0	0
0	0	0	0	0	0	0	0	0	0	0	0	0	0	0	0	0	0
0	0	0	0	0	0	0	0	0	0	0	0	0	0	0	0	0	0
0	21635	0	110	0	0	0	0	0	0	0	0	0	0	0	0	0	0
0	0	0	0	0	0	0	0	0	0	0	0	0	0	0	0	0	0
0	449	0	585	0	0	0	0	0	0	0	0	0	0	39	0	0	0
0	0	0	0	0	0	0	0	0	0	0	0	0	0	0	0	0	0
0	0	0	0	0	0	0	0	0	0	0	0	0	0	0	0	0	0
0	0	0	0	0	0	0	0	0	0	0	0	0	0	0	0	0	0
0	0	0	0	0	0	0	0	0	0	0	0	0	74	0	0	0	0
0	0	0	0	0	0	0	0	0	0	0	0	0	0	0	0	0	0

0	0	0	0	0	0	0	0	0	0	0	0	0	0	0	0	0	0
63	0	0	0	0	0	0	0	0	0	0	0	0	0	0	0	0	0
0	0	0	0	0	0	0	0	0	0	0	0	0	0	71	0	0	0
0	0	0	0	0	0	0	0	0	0	0	0	0	0	0	0	0	0
0	0	0	0	0	0	0	0	0	0	0	0	0	0	0	0	0	0
0	0	0	0	0	0	0	0	0	0	0	0	0	0	0	0	0	0
0	0	0	0	0	0	0	0	0	0	0	0	0	0	0	0	0	0
0	0	0	0	0	0	0	0	0	0	0	0	0	0	0	0	0	0
0	0	0	0	0	0	0	0	0	0	0	0	0	0	0	0	0	0
0	0	0	0	0	0	0	0	0	0	0	0	0	0	0	0	37	202
0	0	0	0	35	0	0	0	0	0	0	0	0	0	0	0	0	0
0	0	0	0	0	0	0	0	0	0	0	0	0	0	0	0	0	0
0	0	0	0	0	82	67	585	0	251	260	102	1399	136	0	0	0	0
0	0	0	0	72	0	0	0	0	0	0	0	0	0	0	0	0	0
0	0	0	52	113	0	0	0	0	3296	98	0	0	0	0	0	0	0
0	0	0	0	0	0	0	0	0	0	0	0	0	0	0	0	0	0
1651	352	0	0	49	0	0	0	0	0	0	0	0	0	0	0	0	0
0	0	0	0	0	0	0	0	0	0	0	0	0	0	0	0	0	0
0	0	0	0	0	0	0	0	0	0	0	0	0	0	0	0	0	0
0	0	0	0	0	0	0	0	0	0	0	0	0	0	0	0	0	0
0	0	0	0	0	0	0	0	0	0	0	0	0	0	0	0	0	0
0	0	0	0	0	0	0	0	0	0	0	0	0	0	0	0	0	0
0	0	0	0	0	0	0	0	0	0	0	0	0	0	0	0	0	0
0	0	0	0	0	0	0	0	0	0	0	0	0	0	0	0	0	0
0	0	0	0	0	0	0	0	0	0	0	0	0	0	0	0	0	0
0	0	0	0	0	0	0	0	0	0	0	0	0	0	0	0	0	0
0	0	0	0	0	0	0	0	0	0	0	0	0	0	0	0	0	0
0	0	0	0	0	0	0	0	0	0	0	0	0	0	0	0	0	0
53	0	0	0	0	0	0	0	0	0	0	0	0	0	0	0	0	0
0	0	0	0	0	0	0	0	0	0	0	0	0	0	0	0	0	0
0	0	0	0	0	0	0	0	0	0	0	0	0	0	0	0	0	0
0	0	0	0	0	0	0	0	0	0	0	0	0	0	0	0	0	0
156504	13421	3449	22105	650451	9332	1472	95319	3003	2506	22915	7702	781693	13779	2687	7356	848233	7919
0	0	0	0	0	0	0	0	121	73	0	0	0	0	0	0	0	0
0	0	0	0	0	0	0	0	0	0	0	0	0	0	0	0	0	0
0	0	0	0	0	0	0	0	0	0	0	0	0	0	0	0	0	0
0	680	0	0	0	0	0	0	0	0	0	0	0	0	0	0	0	0
0	0	0	0	0	0	0	0	0	0	0	0	0	0	0	0	0	0
0	0	0	0	0	0	0	0	0	0	0	0	0	0	0	0	0	0
0	0	0	0	0	0	0	0	0	0	0	0	0	0	0	0	0	0
0	0	34	0	0	0	0	0	0	0	1950	51	0	0	0	0	0	0
0	0	0	0	0	0	0	0	0	0	0	0	0	0	0	0	0	0
0	0	0	0	0	0	0	0	0	0	81	0	0	0	0	0	0	0
0	0	0	0	0	0	0	0	0	0	0	0	0	0	0	0	0	0
0	0	0	0	0	0	0	0	0	0	0	0	0	0	0	0	0	0
0	0	0	0	0	0	0	0	0	0	0	0	0	0	0	0	0	0
0	0	0	0	0	0	0	0	0	0	0	0	0	0	0	0	0	0
0	0	0	0	0	0	0	0	0	0	0	0	0	0	0	0	0	0
4961	45695	48	99	227	30809	0	281	659	39978	914	89	905	199	84	341	185	115

Control 099	Control 117	Control 114	Control 113	Control 112	Control 111	Control 110	Control 109	Control 108	Control 107	Control 106	Control 105	Control 104	Control 103	Control 102	Control 101	Control 115	Control 066
769588	771697	915535	846613	814563	694449	952296	166760	820394	538851	939820	738364	922655	912343	978106	373242	828956	897154
174362	207545	31512	31615	176810	281967	32092	827555	15509	287945	50557	195641	50479	58753	7332	592025	100905	52784
131	0	0	0	0	0	1866	0	0	0	0	166	0	0	0	0	0	0
17892	6310	18833	12509	7876	7513	13350	5260	25049	20460	9418	21870	24335	12606	11625	27274	69569	31175
38027	14448	34028	109263	751	16071	396	425	138794	152744	205	40915	2384	16298	2937	6660	570	18887
0	0	0	0	0	0	0	0	0	0	0	3044	0	0	0	799	0	0
0	0	92	0	0	0	0	0	254	0	0	0	147	0	0	0	0	0
Control 099	Control 117	Control 114	Control 113	Control 112	Control 111	Control 110	Control 109	Control 108	Control 107	Control 106	Control 105	Control 104	Control 103	Control 102	Control 101	Control 115	Control 066
0	132	166	241	0	0	0	0	0	0	0	0	251	0	0	0	0	0
89	0	0	0	0	0	0	0	0	0	0	0	0	0	0	0	0	0
415313	483607	790506	173424	1764	2330	307255	1147	235927	1079	1421	77895	684041	15327	262235	3321	40841	695429
0	0	0	0	0	0	0	0	0	0	0	0	0	0	0	0	0	0
0	0	0	0	51	0	0	0	0	0	0	81	0	0	0	0	0	96
0	0	0	0	0	0	0	0	0	0	0	0	0	0	0	0	0	0
0	0	0	0	0	0	0	0	0	0	0	0	0	0	0	0	0	0
0	0	0	0	0	0	0	0	0	0	0	0	0	0	0	0	0	0
0	0	0	0	0	0	0	0	0	0	0	0	430	0	0	0	0	0
0	0	0	0	0	0	0	0	0	0	0	0	0	422	0	0	0	0
0	0	0	0	0	0	0	0	0	0	0	0	0	0	0	0	0	0
11512	50854	333	10352	194	250	108	66169	1707	26438	52	29630	229	65	214	330	354	1523
0	0	62	0	0	0	0	0	0	0	0	0	55	0	0	0	0	0
0	0	0	0	0	0	0	0	0	0	0	0	0	0	0	0	0	0
0	0	0	0	0	0	0	0	0	0	0	0	0	0	0	0	0	0
9424	85	15078	103	49	0	6067	0	3803	0	0	162	14885	63	4330	72	0	17316
0	0	0	0	0	0	0	0	0	0	0	0	0	0	0	0	0	0
0	0	0	0	0	0	0	0	0	0	0	0	0	0	0	0	0	0
0	0	0	0	0	0	0	0	0	0	0	0	0	0	0	0	0	0
128	0	0	124	0	0	0	0	0	0	0	0	0	0	0	0	0	55
0	0	0	0	0	0	0	0	0	0	0	0	0	0	0	0	0	0
0	0	0	0	0	0	0	0	0	0	0	0	0	0	0	0	0	0
0	0	0	0	0	0	0	0	0	0	0	0	0	0	0	0	0	0
0	0	0	0	0	0	0	0	0	0	0	0	0	0	0	0	0	0
335	94	0	0	0	0	26	0	215	0	0	0	0	0	0	0	0	251
0	0	0	0	0	0	0	0	0	0	0	0	0	0	0	0	0	0
0	0	0	70	600	0	1196	0	814	0	842	0	0	0	0	0	0	0
0	0	0	0	0	0	0	0	0	0	0	0	0	0	0	0	0	0
0	0	0	0	0	0	0	0	71	0	0	0	116	5256	0	67	0	0
112090	1049	3933	27530	249888	80362	243455	31115	239175	27277	324616	8894	7866	25022	8954	4081	815	21620
4519	0	0	0	0	0	0	0	0	0	0	0	0	0	0	0	0	0
0	0	47	10741	0	0	0	0	59	81	0	0	0	11381	0	0	0	1722
97	0	0	0	0	0	0	0	0	0	0	0	0	0	0	0	0	0
0	0	0	0	0	0	0	0	0	0	0	0	0	0	0	0	0	0
0	0	0	0	0	0	0	0	0	0	0	0	0	0	0	0	0	0
0	117	0	0	0	0	0	0	0	0	0	0	0	0	0	0	0	0
0	0	0	0	0	0	0	0	0	0	0	0	0	0	0	0	0	0
0	0	0	0	0	0	0	0	0	0	0	0	0	0	0	0	0	0
0	0	0	0	0	0	0	0	0	0	0	0	0	0	0	0	0	0
0	0	0	0	0	381	0	0	0	368	0	713	0	0	0	300	0	0
521	0	144	0	0	0	36	0	0	0	0	0	396	0	64	0	0	610
38	0	0	0	0	0	0	0	0	0	0	0	0	0	0	0	0	0
0	0	0	0	0	0	0	0	0	0	0	0	0	0	0	0	0	0
0	0	0	0	0	0	0	0	0	0	0	0	0	0	0	0	0	0
0	0	0	0	0	0	0	0	0	0	0	0	0	0	0	0	0	0
0	42	0	0	0	0	0	0	0	0	0	0	0	0	0	0	0	0
0	0	0	0	0	0	0	0	0	0	0	0	0	0	0	0	0	0
630	581	322	30202	221800	77059	184800	1123	262440	4718	590572	1489	1947	1646	12474	6319	597	2465
0	0	0	0	0	0	0	0	0	0	0	0	0	0	0	0	0	0
0	107	0	54	216	361	177	53	0	187487	134	242	427	147	83	146	0	0
0	0	0	0	0	0	0	0	0	0	0	0	60	0	0	0	0	40
0	0	0	0	0	0	0	0	0	0	0	0	0	0	0	0	0	0
0	0	0	0	0	0	0	0	0	0	0	0	0	0	0	0	0	0
0	0	0	0	0	0	0	0	0	0	0	0	0	4586	0	0	0	0
0	0	0	0	0	0	0	0	0	0	0	0	0	0	0	0	0	0
0	0	0	0	0	0	0	0	0	0	0	0	0	0	0	0	0	0
0	0	0	0	0	0	0	0	0	0	0	0	0	0	0	0	0	0
137	0	1288	0	0	0	0	19	355	0	42	0	1128	0	759	79	0	1290
0	0	0	0	0	0	0	0	0	0	0	0	0	0	0	0	0	0
0	0	0	0	0	0	0	0	0	0	0	0	0	0	0	0	0	0
211	359	607	462	87	79	0	94	268	0	1847	0	116	9076	0	58	123	523
0	0	0	0	0	0	0	0	0	0	0	0	45	3275	0	0	0	0
0	0	0	0	0	0	0	0	0	0	0	0	0	0	0	0	0	0
0	0	0	0	0	0	0	0	0	0	0	0	0	0	0	0	0	0
0	0	0	0	0	0	0	0	0	0	0	0	0	0	0	0	0	0
0	0	0	0	262	184407	125	153	222	109	56	162	186	52	149	362	77	0
0	0	0	0	0	208	118	61142	122	31	0	0	44	0	0	158	0	0
0	0	0	0	0	0	0	0	0	0	0	0	0	0	0	0	0	0
0	0	0	64	0	0	0	0	0	0	0	0	0	0	0	0	0	0

0	0	0	0	0	0	0	0	0	0	0	0	0	0	0	0	0	0
0	0	0	0	0	0	0	0	0	0	0	0	0	0	0	0	0	0
0	0	0	0	0	0	0	0	0	0	0	0	0	0	0	0	0	0
0	0	0	0	0	0	0	0	0	0	0	0	0	0	0	0	0	0
0	0	0	0	0	0	0	0	0	0	0	0	0	0	0	0	0	0
0	0	0	0	0	0	0	0	0	0	0	0	0	0	0	0	0	0
0	42	214	241	19208	0	0	24	80	32	464	75	0	0	0	79	0	48
0	0	0	0	0	0	0	0	0	0	0	0	0	0	0	0	0	0
131	0	0	0	0	0	1866	0	0	0	0	166	0	0	0	0	0	0
0	0	0	0	0	0	0	0	0	0	0	6939	0	0	0	0	0	0
0	0	0	0	0	0	0	0	0	0	163	0	0	0	0	0	0	0
0	0	0	0	0	0	0	0	0	0	0	0	0	0	0	0	0	0
1277	69411	1340	8640	1514	213722	1083	763	1462	147452	595	213633	1276	38242	1165	213963	383	1603
0	0	0	0	0	0	0	0	0	0	0	0	0	0	0	0	0	0
0	0	0	0	0	0	0	0	0	0	0	0	0	0	0	0	0	0
0	0	0	0	0	0	0	0	0	0	0	0	0	0	0	0	0	0
0	0	0	0	0	0	0	0	0	0	0	0	0	0	0	0	0	0
0	0	0	0	0	0	0	0	0	0	0	0	0	0	0	0	0	0
0	49	0	0	0	0	0	0	258	0	0	0	0	0	0	0	0	0
0	0	0	0	0	0	0	0	0	30	2268	0	0	0	0	0	0	0
0	0	0	0	0	0	0	0	0	0	0	0	0	0	0	0	0	0
0	0	0	0	0	0	0	0	0	0	0	0	0	0	0	0	0	0
53	0	0	0	0	0	0	0	0	0	0	0	0	0	0	0	0	2568
0	0	0	0	0	0	0	23	0	0	0	0	109	0	84	0	0	0
0	0	80	0	0	0	37	33	108	32	0	0	54	51	74	0	0	46
0	0	0	0	0	0	0	0	0	0	0	0	0	0	0	0	0	0
0	0	0	0	0	0	0	0	0	0	0	0	0	0	0	0	0	0
26506	111902	882	19976	118221	74253	13379	714460	7711	272009	42906	79299	3007	38342	3241	50558	497	418
0	0	0	0	0	0	0	0	0	41	0	9108	0	53	72	0	0	0
0	0	0	0	0	0	0	0	0	0	0	0	0	0	0	0	0	0
0	0	49	0	0	0	0	0	0	0	0	0	0	0	0	0	0	0
0	0	0	0	0	0	0	0	0	0	0	0	0	0	0	0	0	0
0	0	0	0	0	0	0	0	0	0	0	0	0	0	0	0	0	0
0	0	0	0	0	0	0	0	0	0	0	0	0	0	0	0	0	0
143	0	39	0	0	0	0	0	47	0	0	0	440	0	0	0	0	0
0	0	0	0	0	0	0	0	0	0	0	0	0	0	0	0	0	0
0	0	0	0	0	0	0	0	0	0	0	0	0	0	0	0	0	0
0	0	0	0	0	0	0	0	0	0	0	0	0	0	0	0	0	0
0	0	0	62	0	0	0	0	0	0	0	0	0	0	0	0	52	91
0	0	0	0	0	0	0	0	0	0	0	0	0	0	0	0	0	0
0	0	0	0	0	0	0	0	0	0	0	0	0	0	0	0	0	0
0	0	0	0	0	0	0	0	0	0	0	0	0	0	0	0	0	0
0	0	0	0	0	0	0	0	0	0	0	0	0	0	0	0	0	0
14077	0	123	230	0	0	0	0	702	0	0	0	0	0	0	0	0	0
0	0	0	0	0	134	43	0	53	0	0	51	0	11815	0	77	0	0
0	0	0	0	88	0	0	0	0	0	0	0	0	0	0	0	0	0
0	0	0	0	0	0	0	0	0	0	0	0	0	0	0	0	0	58
36	0	0	0	0	0	0	0	0	0	0	0	0	0	0	0	0	0
0	0	0	0	0	0	0	0	0	0	0	43	37	0	0	165	0	651
0	0	0	0	0	0	0	0	0	0	0	0	0	0	0	0	0	0
38027	14448	34028	109263	700	16071	396	425	138794	152744	205	40834	2384	16298	2937	6660	570	18602
0	0	0	0	0	0	0	0	0	0	0	0	0	0	0	0	0	0
0	0	0	0	0	0	0	0	0	0	0	0	0	0	0	0	0	0
0	0	0	0	0	0	0	0	0	0	0	0	0	0	0	0	0	0
0	0	0	0	0	0	0	0	0	0	0	0	0	0	0	0	0	0
0	0	0	0	0	0	0	0	0	0	0	0	0	0	0	0	0	0
0	0	54	0	0	0	0	0	0	0	0	0	57	0	0	0	0	0
0	0	0	0	0	0	0	0	0	0	0	0	0	0	0	0	0	0
0	0	57	0	0	0	0	0	0	0	0	0	0	0	0	0	0	0
0	0	0	0	0	0	0	0	0	0	0	0	0	0	0	0	0	0
52	0	0	0	0	0	0	0	0	0	0	298	0	0	0	0	0	0
0	0	0	0	0	0	0	0	0	0	0	0	0	0	0	0	0	0
1690	43	497	0	0	0	0	0	0	0	96	0	973	0	782	0	0	5605
0	0	0	0	0	67	0	0	860	0	0	0	0	0	0	0	0	0
0	0	0	0	0	0	0	0	0	0	0	0	0	0	0	0	0	0
0	0	0	0	0	0	0	0	0	0	0	0	0	0	0	0	0	0
0	0	0	0	0	0	0	0	0	0	0	0	0	0	0	0	0	0
0	0	44	0	0	0	0	0	0	0	0	0	0	0	0	0	0	0
0	0	0	0	56	57	0	31	0	0	0	0	0	0	0	79	0	0
0	0	0	0	0	0	0	0	0	0	0	0	0	0	0	0	0	0
0	0	0	0	0	0	0	0	0	0	0	0	0	0	0	0	0	0
0	0	0	0	0	0	0	0	0	0	0	0	0	0	0	0	0	0
0	0	0	0	0	0	0	0	0	0	0	0	0	0	0	0	0	0
[truncated: 890,949 more chars]
